# A DNA barcode-assisted annotated checklist of the spider (Arachnida, Araneae) communities associated to white oak woodlands in Spanish National Parks

**DOI:** 10.3897/BDJ.6.e29443

**Published:** 2018-11-29

**Authors:** Luís C Crespo, Marc Domènech, Alba Enguídanos, Jagoba Malumbres-Olarte, Pedro Cardoso, Jordi Moya-Laraño, Cristina Frías-López, Nuria Macías-Hernández, Eva De Mas, Paola Mazzuca, Elisa Mora, Vera Opatova, Enric Planas, Carles Ribera, Marcos Roca-Cusachs, Dolores Ruiz, Pedro Sousa, Vanina Tonzo, Miquel A. Arnedo

**Affiliations:** 1 Department of Evolutionary Biology, Ecology and Environmental Sciences & Biodiversity Research Institute (IRBio), Universitat de Barcelona, Av. Diagonal 643, E-08028, Barcelona, Spain Department of Evolutionary Biology, Ecology and Environmental Sciences & Biodiversity Research Institute (IRBio), Universitat de Barcelona, Av. Diagonal 643, E-08028 Barcelona Spain; 2 Laboratory for Integrative Biodiversity Research, Finnish Museum of Natural History, University of Helsinki; PO Box 17, 00014, Helsinki, Finland Laboratory for Integrative Biodiversity Research, Finnish Museum of Natural History, University of Helsinki; PO Box 17, 00014 Helsinki Finland; 3 Laboratory for Integrative Biodiversity Research, Finnish Museum of Natural History, University of Helsinki; PO Box 17, 00014, Helsinki, Finland Laboratory for Integrative Biodiversity Research, Finnish Museum of Natural History, University of Helsinki; PO Box 17, 00014 Helsinki Finland; 4 cE3c - Centre for Ecology, Evolution and Environmental Changes, University of the Azores; Rua Capitão João d´Ávila, Pico da Urze, 9700-042 , Angra do Heroísmo, Terceira, Azores, Portugal cE3c - Centre for Ecology, Evolution and Environmental Changes, University of the Azores; Rua Capitão João d´Ávila, Pico da Urze, 9700-042 Angra do Heroísmo, Terceira, Azores Portugal; 5 Department of Functional and Evolutionary Ecology, Estación Experimenta de Zonas Áridas (EEZA, CSIC); Carretera de Sacramento, s/n. La Cañada de San Urbano 04120, Almeria, Spain Department of Functional and Evolutionary Ecology, Estación Experimenta de Zonas Áridas (EEZA, CSIC); Carretera de Sacramento, s/n. La Cañada de San Urbano 04120 Almeria Spain; 6 Department of Genetics, Microbiology and Statistics, & Biodiversity Research Institute (IRBio), Universitat de Barcelona, Av. Diagonal 643, E-08028, Barcelona, Spain Department of Genetics, Microbiology and Statistics, & Biodiversity Research Institute (IRBio), Universitat de Barcelona, Av. Diagonal 643, E-08028 Barcelona Spain; 7 Island Ecology and Evolution Research Group, Instituto de Productos Naturales y Agrobiologíıa, C/Astrofísico Francisco Sánchez 3, La Laguna, Tenerife, Canary Islands, Spain Island Ecology and Evolution Research Group, Instituto de Productos Naturales y Agrobiologíıa, C/Astrofísico Francisco Sánchez 3 La Laguna, Tenerife, Canary Islands Spain; 8 Department of Functional and Evolutionary Ecology, Estación Experimenta de Zonas Áridas (EEZA, CSIC); Carretera de Sacramento, s/n. La Cañada de San Urbano 04120, Almeria, Spain Department of Functional and Evolutionary Ecology, Estación Experimenta de Zonas Áridas (EEZA, CSIC); Carretera de Sacramento, s/n. La Cañada de San Urbano 04120 Almeria Spain; 9 Department of Entomology and Nematology, University of California, Davis, CA 95616, Davis, United States of America Department of Entomology and Nematology, University of California, Davis, CA 95616 Davis United States of America; 10 Laboratory of Systematic Entomology in the Department of Applied Biology of Chungnam National University, Daejeon, Korea, South Laboratory of Systematic Entomology in the Department of Applied Biology of Chungnam National University Daejeon Korea, South; 11 CIBIO, Centro de Investigação em Biodiversidade e Recursos Genéticos, Universidade do Porto, Vila do Conde, Portugal CIBIO, Centro de Investigação em Biodiversidade e Recursos Genéticos, Universidade do Porto Vila do Conde Portugal

**Keywords:** DNA barcoding, faunistics, COBRA protocol, Mediterranean region, Iberian Peninsula, Dictynidae, Gnaphosidae, Linyphiidae, Philodromidae

## Abstract

**Background:**

A large scale semi-quantitative biodiversity assessment was conducted in white oak woodlands in areas included in the Spanish Network of National Parks, as part of a project aimed at revealing biogeographic patterns and identify biodiversity drivers. The semi-quantitative COBRA sampling protocol was conducted in sixteen 1-ha plots across six national parks using a nested design. All adult specimens were identified to species level based on morphology. Uncertain delimitations and identifications due to either limited information of diagnostic characters or conflicting taxonomy were further investigated using DNA barcode information.

**New information:**

We identified 376 species belonging to 190 genera in 39 families, from the 8,521 adults found amongst the 20,539 collected specimens. Faunistic results include the discovery of 7 new species to the Iberian Peninsula, 3 new species to Spain and 11 putative new species to science. As largely expected by environmental features, the southern parks showed a higher proportion of Iberian and Mediterranean species than the northern parks, where the Palearctic elements were largely dominant. The analysis of approximately 3,200 DNA barcodes generated in the present study, corroborated and provided finer resolution to the morphologically based delimitation and identification of specimens in some taxonomically challenging families. Specifically, molecular data confirmed putative new species with diagnosable morphology, identified overlooked lineages that may constitute new species, confirmed assignment of specimens of unknown sexes to species and identified cases of misidentifications and phenotypic polymorphisms.

## Introduction

The Iberian Peninsula is one of the most diverse regions in the Mediterranean Basin because of its location at the crossroads between Europe and Africa and its complex orography and variable climate, ranging from a central and southern Mediterranean climate to a northern Eurosiberian one. The high level of species richness in the Iberian Peninsula is particularly evident in spiders (Carvalho et al. 2012, Carvalho et al. 2011, Jiménez-Valverde et al. 2010), where approximately 1,400 species have been catalogued to date (Morano et al. 2014). The Iberian biota is also highly endemic and threatened, with most of the south of the peninsula being identified as one of the most important biodiversity hotspots in the Mediterranean region (Medail and Quezel 1999). Amongst the order Araneae, 18% of the species in the Iberian Peninsula are Iberian endemics, a value that rises above 50% in families such as Dysderidae C. L. Koch, 1837, Zodariidae Thorell, 1881 or Nemesiidae Simon, 1889 (Cardoso and Morano 2010, Morano et al. 2014).

Despite the high number of spiders recorded in the Iberian Peninsula, the species-richness is lower than in neighbouring countries of similar size, yet less complex or younger geological history, such as France (1587 species) (Nentwig et al. 2017) or Italy (1632 species) (Pantini and Isaia 2017). The relatively shorter tradition in natural history of Iberian countries leads us to suspect that the Iberian arachnofauna is fewer because it is far from being fully catalogued, which is one of the main impediments for invertebrate conservation in the region (Cardoso et al. 2011). Gradually, new faunistic records are helping to build up our knowledge on both the richness and distribution of Iberian species (Barrientos and Fernández 2015, Barrientos et al. 2014, Barrientos et al. 2015a, Barrientos et al. 2015b, Barrientos et al. 2016, Barriga et al. 2007, Cardenas and Barrientos 2011, Jimenez-Segura et al. 2017, Melic et al. 2016, Pérez 2016, Pérez and Castro 2016, Pérez et al. 2015). Unfortunately, many specimens acquired in local ecological assessments frequently remain unidentified in collections due to either a lack of expertise or informative taxonomic literature. Diverse taxa such as Nemesiidae, Dysderidae, Gnaphosidae or Oonopidae continue to demand revisionary taxonomic work, which, given the current downward trend in funding for basic taxonomic research and the time needed to complete these thorough works, can only be afforded by a decreasing number of taxonomists.

The use of DNA barcoding – standardised, short fragments of DNA, as a species identifier (Hebert et al. 2003) – has become very popular amongst spider taxonomists (Astrin et al. 2016, Barrett and Hebert 2005, Blagoev et al. 2013, Blagoev et al. 2015, Candek and Kuntner 2015, Castalanelli et al. 2014, Robinson et al. 2009). Although the use of DNA barcoding is not yet fully incorporated into standard diversity assessments, when available, this tool provides many advantages to the taxonomists working on a medium- or large-sized collection of spiders. DNA barcodes can facilitate and accelerate taxonomic research by increasing the ability of matching individuals regardless of sex, stage or body parts, identifying specimens with morphological diagnostic characters either subtle, difficult to visualise or absent or reassessing intraspecific polymorphisms.

Here we present the checklist of spider species identified from the adult specimens collected as part of a large-scale biodiversity assessment of the spider communities in white-oak (*Quercus* L.) woodlands across the Spanish Natural Parks Network (hereafter referred to as the IBERCODING project). Specimens were collected using the COBRA protocol (Cardoso 2009), a semi-quantitative sampling protocol initially designed to assess biodiversity patterns in Mediterranean spider communities and then adapted to other habitats (Malumbres-Olarte et al. 2017) and potentially extendable to other taxa. The identification of the collected specimens is the first necessary step towards calculating α- and β-diversity values across broad geographic and climatic ranges and ultimately inferring the drivers responsible for those patterns.

We chose to focus on white-oak forests because they represent common forests in the focal national parks and their high levels of endemicity (Franco 1990), relevance for conservation (García and Mejías 2009, Marañón and Pérez-Ramos 2009) and relatively well-characterised evolutionary history in the Iberian Peninsula (Olalde et al. 2002, Petit et al. 2002).

As part of the IBERCODING project, we generated DNA barcodes for more than 3,200 specimens with the aim of revealing fine scale geographic patterns in genetic diversity, retrieving phylogenetic information for assessing phylogenetic diversity of communities and facilitating sorting and identification of the specimens.

The present publication focuses on the identification of the individuals collected, with comments on their distribution and spatial location, as well as new records to the region and the discovery of putative new species. The availability of DNA barcodes helped identification and delimitation in some taxonomically challenging groups, such as the families Dictynidae, Gnaphosidae, Linyphiidae or Philodromidae We characterised the biogeographic patterns of the different plots and parks based on the species distribution information available in the literature and complemented it with our own data.

## Materials and methods

### Study area

**Figure 1.**
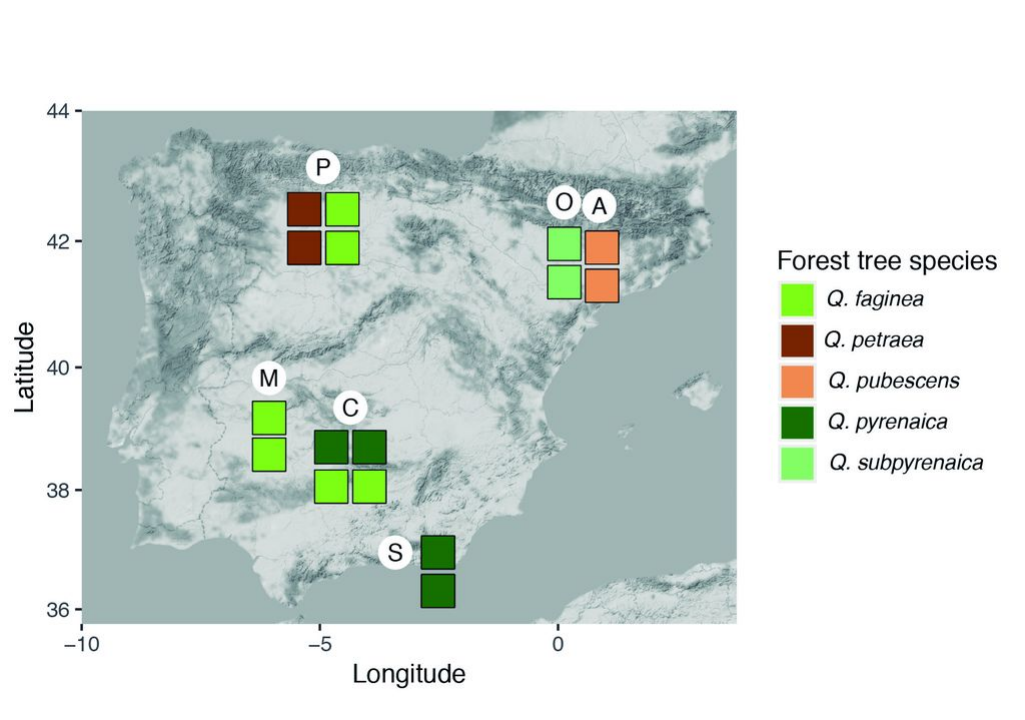
Map of the Iberian Peninsula with the location of the national parks and the plots where the sampling protocol COBRA was applied. For each park, squares denote the number of plots and the oak forest type (colour code labels in the inset). Northern parks are Picos de Europa (P), Ordesa (O), Aigüestortes (A). Southern parks Monfragüe (M), Cabañeros (C), Sierra Nevada (S). See Table 1 for additional information on plots and parks.

Spider communities were sampled in white oak and related oak forests from six Spanish national parks (Fig. 1), namely Picos de Europa (P), Ordesa y Monte Perdido (O), Aigüestortes i Estany de Sant Maurici (A) (hereafter referred to as the northern parks) Fig. 2), Monfragüe (M), Cabañeros (C) and Sierra Nevada (S) (hereafter referred to as the southern parks) (Fig. 3). The chosen parks fulfilled three conditions: (1) they had representative white oak forests, (2) they represented the main biogeographic areas within the Iberian Peninsula (Atlantic, Alpine and Mediterranean) (European Environment Agency 2012) and (3) they covered a broad latitudinal and elevational gradient within the Iberian Peninsula. The selected parks spanned distances ranging from 80 km apart (A to O) to 720 km (S to A) and elevations from 320 m (Monfragüe) to 1786 m (Sierra Nevada). Sampling was conducted between May and June, when the richness and abundance of adult spiders in Mediterranean habitats are at its maximum (Cardoso et al. 2007), in two consecutive years, 2013 for the northern parks and 2014 for the southern parks. Two replicates (plots) were set up in each park, except in Picos de Europa and Cabañeros, where two different types of oak forest were available and hence two replicates were set up per forest type, resulting in a total of 16 plots (northern parks P=4, O=2, A=2; southern parks M=2, C=4 S=2, respectively). Additional details of the sampling plots are available in Table 1.

### Sample collection

In each plot, a COBRA 50 sampling protocol was conducted, which is specifically designed to collect 50% of the spider diversity in the sampling area in an optimised manner (Cardoso 2009). This protocol consists of using different sampling methods to obtain the maximum possible number of species. Direct sampling (methods that require the presence of the collector and her/ his active participation in the specimen capture) were foliage beating, vegetation sweeping and aerial hand collection. For foliage beating, a 1 m^2^ beating tray and a wooden pole were used to beat tree branches as high as possible. For vegetation sweeping, a round sweep net with a diameter of 58 cm was used to sweep tall plants and bushes, below the collector‘s waist. Aerial hand collection was done through visual inspection and hand-capture (aided by forceps, pooter or brush, if needed) on the vegetation above knee-level. The maximum possible number of spiders was caught and transferred to a vial with ethanol. Each sampling consisted of one hour of continuous collecting by one collector. In two plots (P1, A1), we conducted two additional ground hand collecting samples but focused on specimens present below knee-level.

Indirect sampling (techniques that do not involve the presence of the collector), consisted in the use of pitfall traps, i.e. vessels 7.5 cm in diameter buried in the ground with the rim at the ground level and filled with propylene glycol, which preserved spiders for both morphologic and genetic analyses. A few detergent drops were added to the liquid to break the surface tension and to allow spiders to sink to the bottom of the vessel. Pitfalls were covered with labelled plastic caps, held about 1 cm above the ground by four short wires anchored to the ground, in order to prevent the fall of debris into the trap and propylene glycol dilution or overflow caused by rainwater.

Direct sampling in each plot consisted of 2 hours of diurnal and 2 hours of nocturnal foliage beating, 2 hours of diurnal and 2 hours of nocturnal vegetation sweeping and 4 hours of nocturnal aerial hand collecting, which totals 12 hours of sampling, equating to 12 man-hours of sampling. Indirect samples were uniformly distributed within each plot in groups of 4 pitfalls, set in squares with 5 m sides. The traps were left active during two weeks. For subsequent analyses, each group of 4 contiguous pitfall traps were combined and considered as a single sample, which totals 12 indirect samples (Carvalho et al. 2011). All in all, the study included 388 samples (24 samples per plot x 16 plots + 2 extra ground samples x 2 plots, P1 and A1, respectively).

### Identification of specimens

All adults were identified, when possible, to species level. Amongt a wide spectrum of taxonomic literature, the “Araneae: Spiders of Europe” database was used to identify most of the known species found in the samples (Nentwig et al. 2017). Identifications were made mainly with the use of a ZEISS Stemi 2000 stereomicroscope. Images were taken with a Leica DFC 450 camera attached to a Leica MZ 16A stereomicroscope, using the software Leica Application Suite v4.4. After collection, specimens were stored in 95% ethanol and kept at -20ºC in Falcon vials until these were sequentially sorted and identified (materials from the northern parks were collected and sorted before the materials from the southern parks), from which they were moved to smaller vials of 2 ml and returned to -20ºC, for subsequent genetic analyses.

### Annotated checklist

For each species, we provided the number of male and female specimens identified by plot (see abbreviations in Table 1) and collecting technique, namely foliage beating (beating), vegetation sweeping (sweeping), aerial hand collection and pitfall trapping. Unidentified morphs and putative new species were provisionally labelled using the genus name and a sequential numeration (e.g. *Brigittea* sp04). Distributions were based on information available in public databases (Nentwig et al. 2017, World Spider Catalog 2018).

### Molecular procedures

DNA barcodes were obtained from all sampled species – five individuals were analysed per morpho-species and per plot when possible, as many species collected without taxonomic targeting are usually found in singletons or doubletons. Legs were used for DNA extraction and the rest of the individual was kept as a voucher, although for small species, the entire specimen was used. In these cases, the extractions were non-destructive (i.e. specimens were not ground up) and specimens were recovered as vouchers after the lyses of soft internal tissues. Total genomic DNA was extracted using the REDExtract-N-Amp™ Tissue PCR Kit Protocol from Sigma-Aldrich, following the manufacturer’s protocol and performed in 96 well-plates. The primers used for amplification are listed in Table 2. The LCOI1490/HCO2198 was the preferred combination, while Nancy was used as a replacement for HCOI2198 and Ron as replacement for LCOI1490 (in that order). For problematic amplifications, we used the internal primers mlCOIintF/jgHCOI2198. The polymerase chain reaction (PCR) was performed in 96-well plates using 8 µl REDExtract-N-Amp™ PCR ReadyMix from Sigma-Aldrich, primers forward and reverse, 4 µl of diluted DNA and ultrapure, distilled water up to a total reaction volume of 20 µl. PCR conditions were as follows: initial denaturing step at 95°C for 5 min, 35 amplification cycles (94°C for 30 s, 45°C for 35 s, 72°C for 45 s) and a final step at 72°C for 5 min. In some cases a Touchdown protocol was used, consisting of 16 cycles of annealing temperature starting at 62°C and decreasing 1°C each cycle and 25 additional cycles of annealing at 46°C. PCR products were cycle-sequenced in both directions at Macrogen Inc. (Seoul, South Korea).

Raw chromatograms were assembled, edited and further manipulated using the software Geneious v7.1.9 (Kearse et al. 2012).

### DNA barcode analysis

Although DNA barcodes were obtained for all species, we decided to investigate a step further with several families that presented us with cases of incongruence between morphology-based identification and genetic-based identification, namely the Dictynidae, Gnaphosidae, Linyphiidae and Philodromidae. Alignments were obtained by combining all DNA barcodes of focal species (see Results) for each family. We inferred the maximum-likelihood tree for each alignment by finding the best partition scheme first (Kalyaanamoorthy et al. 2017), followed by tree inference using the edge-linked partition model in the IQ-TREE software v1.6.1 (Chernomor et al. 2016, Nguyen et al. 2015). The best tree was then used to delimit putative species using the mPTP algorithm (Kapli et al. 2017). The mPTP method allows identifying species boundaries based on branch lengths obtained from a single-locus, without the need for an ultrametric tree and has been shown to generate more stable outputs than alternative approaches (Blair and Bryson 2017). Genetic distances within and between the clusters identified by mPTP were estimated using the Kimura 2 parameter model (Kimura 1980) in MEGA v.6 (Tamura et al. 2013). One DNA barcode per genetic cluster was further used for automatic identification using BOLD (Ratnasingham and Hebert 2007).

### Biogeographic composition

The delimited and identified species were subsequently grouped in four groups, namely "Cosmopolitan", "Palearctic", "Mediterranean" and "Iberian", based on the distribution information available at the World Spider Catalog 2018, further refined with our own species presence data (see Suppl. material 1. We note that species found only in Iberia were considered Iberian and not Mediterranean and the species recorded only in countries of the Mediterranean basin (including north-African countries) were considered Mediterranean and not Palearctic. Percentage of each of the four groups per plot were estimated and visualised using R (R Development Core Team 2017).

## Checklists

### Checklist of spider (Arachnida, Araneae) communities of white oak woodlands of Spanish National Parks

#### Agelenidae

C. L. Koch, 1837

#### Eratigena feminea

(Simon, 1870)

##### Materials

- **Type status:** Other material. **Occurrence:** individualCount: 1; sex: female; **Location:** locationID: C1; continent: Europe; country: Spain; countryCode: ES; stateProvince: Castilla-La Mancha; county: Ciudad Real; locality: Valle Brezoso; verbatimElevation: 756.56; decimalLatitude: 39.35663; decimalLongitude: -4.35912; geodeticDatum: WGS84; **Event:** eventID: A; samplingProtocol: Pitfall
- **Type status:** Other material. **Occurrence:** individualCount: 1; sex: male; **Location:** locationID: S2; continent: Europe; country: Spain; countryCode: ES; stateProvince: Andalucía; county: Granada; locality: Camarate; verbatimElevation: 1713.96; decimalLatitude: 37.18377; decimalLongitude: -3.26282; geodeticDatum: WGS84; **Event:** eventID: L; samplingProtocol: Pitfall

##### Distribution

Iberian Peninsula, Madeira, Algeria

#### Eratigena fuesslini

(Pavesi, 1873)

##### Materials

- **Type status:** Other material. **Occurrence:** individualCount: 1; sex: male; **Location:** locationID: A1; continent: Europe; country: Spain; countryCode: ES; stateProvince: Catalonia; county: Lleida; locality: Sola de Boi; verbatimElevation: 1759.8; decimalLatitude: 42.54958; decimalLongitude: 0.87254; geodeticDatum: WGS84; **Event:** eventID: B; samplingProtocol: Pitfall
- **Type status:** Other material. **Occurrence:** individualCount: 1; sex: male; **Location:** locationID: A1; continent: Europe; country: Spain; countryCode: ES; stateProvince: Catalonia; county: Lleida; locality: Sola de Boi; verbatimElevation: 1759.8; decimalLatitude: 42.54958; decimalLongitude: 0.87254; geodeticDatum: WGS84; **Event:** eventID: C; samplingProtocol: Pitfall
- **Type status:** Other material. **Occurrence:** individualCount: 4; sex: male; **Location:** locationID: A1; continent: Europe; country: Spain; countryCode: ES; stateProvince: Catalonia; county: Lleida; locality: Sola de Boi; verbatimElevation: 1759.8; decimalLatitude: 42.54958; decimalLongitude: 0.87254; geodeticDatum: WGS84; **Event:** eventID: D; samplingProtocol: Pitfall
- **Type status:** Other material. **Occurrence:** individualCount: 1; sex: female; **Location:** locationID: A1; continent: Europe; country: Spain; countryCode: ES; stateProvince: Catalonia; county: Lleida; locality: Sola de Boi; verbatimElevation: 1759.8; decimalLatitude: 42.54958; decimalLongitude: 0.87254; geodeticDatum: WGS84; **Event:** eventID: E; samplingProtocol: Pitfall
- **Type status:** Other material. **Occurrence:** individualCount: 1; sex: male; **Location:** locationID: A1; continent: Europe; country: Spain; countryCode: ES; stateProvince: Catalonia; county: Lleida; locality: Sola de Boi; verbatimElevation: 1759.8; decimalLatitude: 42.54958; decimalLongitude: 0.87254; geodeticDatum: WGS84; **Event:** eventID: F; samplingProtocol: Pitfall
- **Type status:** Other material. **Occurrence:** individualCount: 1; sex: male; **Location:** locationID: A1; continent: Europe; country: Spain; countryCode: ES; stateProvince: Catalonia; county: Lleida; locality: Sola de Boi; verbatimElevation: 1759.8; decimalLatitude: 42.54958; decimalLongitude: 0.87254; geodeticDatum: WGS84; **Event:** eventID: G; samplingProtocol: Pitfall
- **Type status:** Other material. **Occurrence:** individualCount: 1; sex: male; **Location:** locationID: A1; continent: Europe; country: Spain; countryCode: ES; stateProvince: Catalonia; county: Lleida; locality: Sola de Boi; verbatimElevation: 1759.8; decimalLatitude: 42.54958; decimalLongitude: 0.87254; geodeticDatum: WGS84; **Event:** eventID: H; samplingProtocol: Pitfall
- **Type status:** Other material. **Occurrence:** individualCount: 2; sex: male; **Location:** locationID: A1; continent: Europe; country: Spain; countryCode: ES; stateProvince: Catalonia; county: Lleida; locality: Sola de Boi; verbatimElevation: 1759.8; decimalLatitude: 42.54958; decimalLongitude: 0.87254; geodeticDatum: WGS84; **Event:** eventID: J; samplingProtocol: Pitfall
- **Type status:** Other material. **Occurrence:** individualCount: 1; sex: female; **Location:** locationID: A1; continent: Europe; country: Spain; countryCode: ES; stateProvince: Catalonia; county: Lleida; locality: Sola de Boi; verbatimElevation: 1759.8; decimalLatitude: 42.54958; decimalLongitude: 0.87254; geodeticDatum: WGS84; **Event:** eventID: J; samplingProtocol: Pitfall
- **Type status:** Other material. **Occurrence:** individualCount: 1; sex: male; **Location:** locationID: A1; continent: Europe; country: Spain; countryCode: ES; stateProvince: Catalonia; county: Lleida; locality: Sola de Boi; verbatimElevation: 1759.8; decimalLatitude: 42.54958; decimalLongitude: 0.87254; geodeticDatum: WGS84; **Event:** eventID: L; samplingProtocol: Pitfall
- **Type status:** Other material. **Occurrence:** individualCount: 1; sex: male; **Location:** locationID: A2; continent: Europe; country: Spain; countryCode: ES; stateProvince: Catalonia; county: Lleida; locality: Sola de Boi; verbatimElevation: 1738.7; decimalLatitude: 42.54913; decimalLongitude: 0.87137; geodeticDatum: WGS84; **Event:** eventID: 2; samplingProtocol: Aerial; eventTime: Night
- **Type status:** Other material. **Occurrence:** individualCount: 1; sex: male; **Location:** locationID: A2; continent: Europe; country: Spain; countryCode: ES; stateProvince: Catalonia; county: Lleida; locality: Sola de Boi; verbatimElevation: 1738.7; decimalLatitude: 42.54913; decimalLongitude: 0.87137; geodeticDatum: WGS84; **Event:** eventID: D; samplingProtocol: Pitfall
- **Type status:** Other material. **Occurrence:** individualCount: 3; sex: male; **Location:** locationID: A2; continent: Europe; country: Spain; countryCode: ES; stateProvince: Catalonia; county: Lleida; locality: Sola de Boi; verbatimElevation: 1738.7; decimalLatitude: 42.54913; decimalLongitude: 0.87137; geodeticDatum: WGS84; **Event:** eventID: I; samplingProtocol: Pitfall
- **Type status:** Other material. **Occurrence:** individualCount: 1; sex: female; **Location:** locationID: A2; continent: Europe; country: Spain; countryCode: ES; stateProvince: Catalonia; county: Lleida; locality: Sola de Boi; verbatimElevation: 1738.7; decimalLatitude: 42.54913; decimalLongitude: 0.87137; geodeticDatum: WGS84; **Event:** eventID: J; samplingProtocol: Pitfall
- **Type status:** Other material. **Occurrence:** individualCount: 1; sex: male; **Location:** locationID: A2; continent: Europe; country: Spain; countryCode: ES; stateProvince: Catalonia; county: Lleida; locality: Sola de Boi; verbatimElevation: 1738.7; decimalLatitude: 42.54913; decimalLongitude: 0.87137; geodeticDatum: WGS84; **Event:** eventID: K; samplingProtocol: Pitfall
- **Type status:** Other material. **Occurrence:** individualCount: 1; sex: female; **Location:** locationID: A2; continent: Europe; country: Spain; countryCode: ES; stateProvince: Catalonia; county: Lleida; locality: Sola de Boi; verbatimElevation: 1738.7; decimalLatitude: 42.54913; decimalLongitude: 0.87137; geodeticDatum: WGS84; **Event:** eventID: L; samplingProtocol: Pitfall
- **Type status:** Other material. **Occurrence:** individualCount: 1; sex: female; **Location:** locationID: O2; continent: Europe; country: Spain; countryCode: ES; stateProvince: Aragón; county: Huesca; locality: Rebilla; verbatimElevation: 1158.13; decimalLatitude: 42.59427; decimalLongitude: 0.1529; geodeticDatum: WGS84; **Event:** eventID: E; samplingProtocol: Pitfall
- **Type status:** Other material. **Occurrence:** individualCount: 1; sex: female; **Location:** locationID: O2; continent: Europe; country: Spain; countryCode: ES; stateProvince: Aragón; county: Huesca; locality: Rebilla; verbatimElevation: 1158.13; decimalLatitude: 42.59427; decimalLongitude: 0.1529; geodeticDatum: WGS84; **Event:** eventID: I; samplingProtocol: Pitfall
- **Type status:** Other material. **Occurrence:** individualCount: 1; sex: male; **Location:** locationID: O2; continent: Europe; country: Spain; countryCode: ES; stateProvince: Aragón; county: Huesca; locality: Rebilla; verbatimElevation: 1158.13; decimalLatitude: 42.59427; decimalLongitude: 0.1529; geodeticDatum: WGS84; **Event:** eventID: L; samplingProtocol: Pitfall
- **Type status:** Other material. **Occurrence:** individualCount: 4; sex: male; **Location:** locationID: P2; continent: Europe; country: Spain; countryCode: ES; stateProvince: Castilla y León; county: León; locality: Joyoguelas; verbatimElevation: 763.98; decimalLatitude: 43.17771; decimalLongitude: -4.90579; geodeticDatum: WGS84; **Event:** eventID: A; samplingProtocol: Pitfall
- **Type status:** Other material. **Occurrence:** individualCount: 1; sex: female; **Location:** locationID: P2; continent: Europe; country: Spain; countryCode: ES; stateProvince: Castilla y León; county: León; locality: Joyoguelas; verbatimElevation: 763.98; decimalLatitude: 43.17771; decimalLongitude: -4.90579; geodeticDatum: WGS84; **Event:** eventID: A; samplingProtocol: Pitfall
- **Type status:** Other material. **Occurrence:** individualCount: 5; sex: male; **Location:** locationID: P2; continent: Europe; country: Spain; countryCode: ES; stateProvince: Castilla y León; county: León; locality: Joyoguelas; verbatimElevation: 763.98; decimalLatitude: 43.17771; decimalLongitude: -4.90579; geodeticDatum: WGS84; **Event:** eventID: C; samplingProtocol: Pitfall
- **Type status:** Other material. **Occurrence:** individualCount: 4; sex: male; **Location:** locationID: P2; continent: Europe; country: Spain; countryCode: ES; stateProvince: Castilla y León; county: León; locality: Joyoguelas; verbatimElevation: 763.98; decimalLatitude: 43.17771; decimalLongitude: -4.90579; geodeticDatum: WGS84; **Event:** eventID: D; samplingProtocol: Pitfall
- **Type status:** Other material. **Occurrence:** individualCount: 2; sex: female; **Location:** locationID: P2; continent: Europe; country: Spain; countryCode: ES; stateProvince: Castilla y León; county: León; locality: Joyoguelas; verbatimElevation: 763.98; decimalLatitude: 43.17771; decimalLongitude: -4.90579; geodeticDatum: WGS84; **Event:** eventID: D; samplingProtocol: Pitfall
- **Type status:** Other material. **Occurrence:** individualCount: 7; sex: male; **Location:** locationID: P2; continent: Europe; country: Spain; countryCode: ES; stateProvince: Castilla y León; county: León; locality: Joyoguelas; verbatimElevation: 763.98; decimalLatitude: 43.17771; decimalLongitude: -4.90579; geodeticDatum: WGS84; **Event:** eventID: F; samplingProtocol: Pitfall
- **Type status:** Other material. **Occurrence:** individualCount: 1; sex: female; **Location:** locationID: P2; continent: Europe; country: Spain; countryCode: ES; stateProvince: Castilla y León; county: León; locality: Joyoguelas; verbatimElevation: 763.98; decimalLatitude: 43.17771; decimalLongitude: -4.90579; geodeticDatum: WGS84; **Event:** eventID: F; samplingProtocol: Pitfall
- **Type status:** Other material. **Occurrence:** individualCount: 5; sex: male; **Location:** locationID: P2; continent: Europe; country: Spain; countryCode: ES; stateProvince: Castilla y León; county: León; locality: Joyoguelas; verbatimElevation: 763.98; decimalLatitude: 43.17771; decimalLongitude: -4.90579; geodeticDatum: WGS84; **Event:** eventID: G; samplingProtocol: Pitfall
- **Type status:** Other material. **Occurrence:** individualCount: 1; sex: female; **Location:** locationID: P2; continent: Europe; country: Spain; countryCode: ES; stateProvince: Castilla y León; county: León; locality: Joyoguelas; verbatimElevation: 763.98; decimalLatitude: 43.17771; decimalLongitude: -4.90579; geodeticDatum: WGS84; **Event:** eventID: G; samplingProtocol: Pitfall
- **Type status:** Other material. **Occurrence:** individualCount: 3; sex: male; **Location:** locationID: P2; continent: Europe; country: Spain; countryCode: ES; stateProvince: Castilla y León; county: León; locality: Joyoguelas; verbatimElevation: 763.98; decimalLatitude: 43.17771; decimalLongitude: -4.90579; geodeticDatum: WGS84; **Event:** eventID: H; samplingProtocol: Pitfall
- **Type status:** Other material. **Occurrence:** individualCount: 1; sex: female; **Location:** locationID: P2; continent: Europe; country: Spain; countryCode: ES; stateProvince: Castilla y León; county: León; locality: Joyoguelas; verbatimElevation: 763.98; decimalLatitude: 43.17771; decimalLongitude: -4.90579; geodeticDatum: WGS84; **Event:** eventID: H; samplingProtocol: Pitfall
- **Type status:** Other material. **Occurrence:** individualCount: 2; sex: male; **Location:** locationID: P2; continent: Europe; country: Spain; countryCode: ES; stateProvince: Castilla y León; county: León; locality: Joyoguelas; verbatimElevation: 763.98; decimalLatitude: 43.17771; decimalLongitude: -4.90579; geodeticDatum: WGS84; **Event:** eventID: I; samplingProtocol: Pitfall
- **Type status:** Other material. **Occurrence:** individualCount: 2; sex: male; **Location:** locationID: P2; continent: Europe; country: Spain; countryCode: ES; stateProvince: Castilla y León; county: León; locality: Joyoguelas; verbatimElevation: 763.98; decimalLatitude: 43.17771; decimalLongitude: -4.90579; geodeticDatum: WGS84; **Event:** eventID: J; samplingProtocol: Pitfall
- **Type status:** Other material. **Occurrence:** individualCount: 1; sex: female; **Location:** locationID: P2; continent: Europe; country: Spain; countryCode: ES; stateProvince: Castilla y León; county: León; locality: Joyoguelas; verbatimElevation: 763.98; decimalLatitude: 43.17771; decimalLongitude: -4.90579; geodeticDatum: WGS84; **Event:** eventID: J; samplingProtocol: Pitfall
- **Type status:** Other material. **Occurrence:** individualCount: 2; sex: male; **Location:** locationID: P2; continent: Europe; country: Spain; countryCode: ES; stateProvince: Castilla y León; county: León; locality: Joyoguelas; verbatimElevation: 763.98; decimalLatitude: 43.17771; decimalLongitude: -4.90579; geodeticDatum: WGS84; **Event:** eventID: K; samplingProtocol: Pitfall
- **Type status:** Other material. **Occurrence:** individualCount: 5; sex: male; **Location:** locationID: P2; continent: Europe; country: Spain; countryCode: ES; stateProvince: Castilla y León; county: León; locality: Joyoguelas; verbatimElevation: 763.98; decimalLatitude: 43.17771; decimalLongitude: -4.90579; geodeticDatum: WGS84; **Event:** eventID: L; samplingProtocol: Pitfall
- **Type status:** Other material. **Occurrence:** individualCount: 3; sex: male; **Location:** locationID: P4; continent: Europe; country: Spain; countryCode: ES; stateProvince: Castilla y León; county: León; locality: El Canto; verbatimElevation: 943.48; decimalLatitude: 43.17227; decimalLongitude: -4.90857; geodeticDatum: WGS84; **Event:** eventID: A; samplingProtocol: Pitfall
- **Type status:** Other material. **Occurrence:** individualCount: 2; sex: female; **Location:** locationID: P4; continent: Europe; country: Spain; countryCode: ES; stateProvince: Castilla y León; county: León; locality: El Canto; verbatimElevation: 943.48; decimalLatitude: 43.17227; decimalLongitude: -4.90857; geodeticDatum: WGS84; **Event:** eventID: A; samplingProtocol: Pitfall
- **Type status:** Other material. **Occurrence:** individualCount: 3; sex: male; **Location:** locationID: P4; continent: Europe; country: Spain; countryCode: ES; stateProvince: Castilla y León; county: León; locality: El Canto; verbatimElevation: 943.48; decimalLatitude: 43.17227; decimalLongitude: -4.90857; geodeticDatum: WGS84; **Event:** eventID: B; samplingProtocol: Pitfall
- **Type status:** Other material. **Occurrence:** individualCount: 8; sex: male; **Location:** locationID: P4; continent: Europe; country: Spain; countryCode: ES; stateProvince: Castilla y León; county: León; locality: El Canto; verbatimElevation: 943.48; decimalLatitude: 43.17227; decimalLongitude: -4.90857; geodeticDatum: WGS84; **Event:** eventID: C; samplingProtocol: Pitfall
- **Type status:** Other material. **Occurrence:** individualCount: 1; sex: female; **Location:** locationID: P4; continent: Europe; country: Spain; countryCode: ES; stateProvince: Castilla y León; county: León; locality: El Canto; verbatimElevation: 943.48; decimalLatitude: 43.17227; decimalLongitude: -4.90857; geodeticDatum: WGS84; **Event:** eventID: C; samplingProtocol: Pitfall
- **Type status:** Other material. **Occurrence:** individualCount: 8; sex: male; **Location:** locationID: P4; continent: Europe; country: Spain; countryCode: ES; stateProvince: Castilla y León; county: León; locality: El Canto; verbatimElevation: 943.48; decimalLatitude: 43.17227; decimalLongitude: -4.90857; geodeticDatum: WGS84; **Event:** eventID: D; samplingProtocol: Pitfall
- **Type status:** Other material. **Occurrence:** individualCount: 1; sex: female; **Location:** locationID: P4; continent: Europe; country: Spain; countryCode: ES; stateProvince: Castilla y León; county: León; locality: El Canto; verbatimElevation: 943.48; decimalLatitude: 43.17227; decimalLongitude: -4.90857; geodeticDatum: WGS84; **Event:** eventID: D; samplingProtocol: Pitfall
- **Type status:** Other material. **Occurrence:** individualCount: 4; sex: male; **Location:** locationID: P4; continent: Europe; country: Spain; countryCode: ES; stateProvince: Castilla y León; county: León; locality: El Canto; verbatimElevation: 943.48; decimalLatitude: 43.17227; decimalLongitude: -4.90857; geodeticDatum: WGS84; **Event:** eventID: E; samplingProtocol: Pitfall
- **Type status:** Other material. **Occurrence:** individualCount: 1; sex: female; **Location:** locationID: P4; continent: Europe; country: Spain; countryCode: ES; stateProvince: Castilla y León; county: León; locality: El Canto; verbatimElevation: 943.48; decimalLatitude: 43.17227; decimalLongitude: -4.90857; geodeticDatum: WGS84; **Event:** eventID: E; samplingProtocol: Pitfall
- **Type status:** Other material. **Occurrence:** individualCount: 9; sex: male; **Location:** locationID: P4; continent: Europe; country: Spain; countryCode: ES; stateProvince: Castilla y León; county: León; locality: El Canto; verbatimElevation: 943.48; decimalLatitude: 43.17227; decimalLongitude: -4.90857; geodeticDatum: WGS84; **Event:** eventID: G; samplingProtocol: Pitfall
- **Type status:** Other material. **Occurrence:** individualCount: 10; sex: male; **Location:** locationID: P4; continent: Europe; country: Spain; countryCode: ES; stateProvince: Castilla y León; county: León; locality: El Canto; verbatimElevation: 943.48; decimalLatitude: 43.17227; decimalLongitude: -4.90857; geodeticDatum: WGS84; **Event:** eventID: H; samplingProtocol: Pitfall
- **Type status:** Other material. **Occurrence:** individualCount: 2; sex: female; **Location:** locationID: P4; continent: Europe; country: Spain; countryCode: ES; stateProvince: Castilla y León; county: León; locality: El Canto; verbatimElevation: 943.48; decimalLatitude: 43.17227; decimalLongitude: -4.90857; geodeticDatum: WGS84; **Event:** eventID: H; samplingProtocol: Pitfall
- **Type status:** Other material. **Occurrence:** individualCount: 7; sex: male; **Location:** locationID: P4; continent: Europe; country: Spain; countryCode: ES; stateProvince: Castilla y León; county: León; locality: El Canto; verbatimElevation: 943.48; decimalLatitude: 43.17227; decimalLongitude: -4.90857; geodeticDatum: WGS84; **Event:** eventID: I; samplingProtocol: Pitfall
- **Type status:** Other material. **Occurrence:** individualCount: 2; sex: male; **Location:** locationID: P4; continent: Europe; country: Spain; countryCode: ES; stateProvince: Castilla y León; county: León; locality: El Canto; verbatimElevation: 943.48; decimalLatitude: 43.17227; decimalLongitude: -4.90857; geodeticDatum: WGS84; **Event:** eventID: K; samplingProtocol: Pitfall
- **Type status:** Other material. **Occurrence:** individualCount: 3; sex: male; **Location:** locationID: P4; continent: Europe; country: Spain; countryCode: ES; stateProvince: Castilla y León; county: León; locality: El Canto; verbatimElevation: 943.48; decimalLatitude: 43.17227; decimalLongitude: -4.90857; geodeticDatum: WGS84; **Event:** eventID: L; samplingProtocol: Pitfall

##### Distribution

Europe

#### Eratigena inermis

(Simon, 1870)

##### Materials

- **Type status:** Other material. **Occurrence:** individualCount: 1; sex: female; **Location:** locationID: P1; continent: Europe; country: Spain; countryCode: ES; stateProvince: Castilla y León; county: León; locality: Monte Robledo; verbatimElevation: 1071.58; decimalLatitude: 43.1445; decimalLongitude: -4.92675; geodeticDatum: WGS84; **Event:** eventID: 1; samplingProtocol: Aerial; eventTime: Night
- **Type status:** Other material. **Occurrence:** individualCount: 1; sex: male; **Location:** locationID: P1; continent: Europe; country: Spain; countryCode: ES; stateProvince: Castilla y León; county: León; locality: Monte Robledo; verbatimElevation: 1071.58; decimalLatitude: 43.1445; decimalLongitude: -4.92675; geodeticDatum: WGS84; **Event:** eventID: 1; samplingProtocol: Aerial; eventTime: Night
- **Type status:** Other material. **Occurrence:** individualCount: 3; sex: female; **Location:** locationID: P1; continent: Europe; country: Spain; countryCode: ES; stateProvince: Castilla y León; county: León; locality: Monte Robledo; verbatimElevation: 1071.58; decimalLatitude: 43.1445; decimalLongitude: -4.92675; geodeticDatum: WGS84; **Event:** eventID: 1; samplingProtocol: Ground; eventTime: Day
- **Type status:** Other material. **Occurrence:** individualCount: 1; sex: female; **Location:** locationID: P1; continent: Europe; country: Spain; countryCode: ES; stateProvince: Castilla y León; county: León; locality: Monte Robledo; verbatimElevation: 1071.58; decimalLatitude: 43.1445; decimalLongitude: -4.92675; geodeticDatum: WGS84; **Event:** eventID: 2; samplingProtocol: Ground; eventTime: Day
- **Type status:** Other material. **Occurrence:** individualCount: 1; sex: female; **Location:** locationID: P1; continent: Europe; country: Spain; countryCode: ES; stateProvince: Castilla y León; county: León; locality: Monte Robledo; verbatimElevation: 1071.58; decimalLatitude: 43.1445; decimalLongitude: -4.92675; geodeticDatum: WGS84; **Event:** eventID: J; samplingProtocol: Pitfall
- **Type status:** Other material. **Occurrence:** individualCount: 1; sex: male; **Location:** locationID: P2; continent: Europe; country: Spain; countryCode: ES; stateProvince: Castilla y León; county: León; locality: Joyoguelas; verbatimElevation: 763.98; decimalLatitude: 43.17771; decimalLongitude: -4.90579; geodeticDatum: WGS84; **Event:** eventID: 2; samplingProtocol: Aerial; eventTime: Night
- **Type status:** Other material. **Occurrence:** individualCount: 1; sex: male; **Location:** locationID: P2; continent: Europe; country: Spain; countryCode: ES; stateProvince: Castilla y León; county: León; locality: Joyoguelas; verbatimElevation: 763.98; decimalLatitude: 43.17771; decimalLongitude: -4.90579; geodeticDatum: WGS84; **Event:** eventID: H; samplingProtocol: Pitfall
- **Type status:** Other material. **Occurrence:** individualCount: 1; sex: female; **Location:** locationID: P2; continent: Europe; country: Spain; countryCode: ES; stateProvince: Castilla y León; county: León; locality: Joyoguelas; verbatimElevation: 763.98; decimalLatitude: 43.17771; decimalLongitude: -4.90579; geodeticDatum: WGS84; **Event:** eventID: 1; samplingProtocol: Sweep; eventTime: Night
- **Type status:** Other material. **Occurrence:** individualCount: 1; sex: male; **Location:** locationID: P4; continent: Europe; country: Spain; countryCode: ES; stateProvince: Castilla y León; county: León; locality: El Canto; verbatimElevation: 943.48; decimalLatitude: 43.17227; decimalLongitude: -4.90857; geodeticDatum: WGS84; **Event:** eventID: 2; samplingProtocol: Aerial; eventTime: Night

##### Distribution

Iberian Peninsula, France

#### Eratigena montigena

(Simon, 1937)

##### Materials

- **Type status:** Other material. **Occurrence:** individualCount: 10; sex: male; **Location:** locationID: C1; continent: Europe; country: Spain; countryCode: ES; stateProvince: Castilla-La Mancha; county: Ciudad Real; locality: Valle Brezoso; verbatimElevation: 756.56; decimalLatitude: 39.35663; decimalLongitude: -4.35912; geodeticDatum: WGS84; **Event:** eventID: A; samplingProtocol: Pitfall
- **Type status:** Other material. **Occurrence:** individualCount: 1; sex: female; **Location:** locationID: C1; continent: Europe; country: Spain; countryCode: ES; stateProvince: Castilla-La Mancha; county: Ciudad Real; locality: Valle Brezoso; verbatimElevation: 756.56; decimalLatitude: 39.35663; decimalLongitude: -4.35912; geodeticDatum: WGS84; **Event:** eventID: A; samplingProtocol: Pitfall
- **Type status:** Other material. **Occurrence:** individualCount: 4; sex: male; **Location:** locationID: C1; continent: Europe; country: Spain; countryCode: ES; stateProvince: Castilla-La Mancha; county: Ciudad Real; locality: Valle Brezoso; verbatimElevation: 756.56; decimalLatitude: 39.35663; decimalLongitude: -4.35912; geodeticDatum: WGS84; **Event:** eventID: B; samplingProtocol: Pitfall
- **Type status:** Other material. **Occurrence:** individualCount: 1; sex: female; **Location:** locationID: C1; continent: Europe; country: Spain; countryCode: ES; stateProvince: Castilla-La Mancha; county: Ciudad Real; locality: Valle Brezoso; verbatimElevation: 756.56; decimalLatitude: 39.35663; decimalLongitude: -4.35912; geodeticDatum: WGS84; **Event:** eventID: B; samplingProtocol: Pitfall
- **Type status:** Other material. **Occurrence:** individualCount: 17; sex: male; **Location:** locationID: C1; continent: Europe; country: Spain; countryCode: ES; stateProvince: Castilla-La Mancha; county: Ciudad Real; locality: Valle Brezoso; verbatimElevation: 756.56; decimalLatitude: 39.35663; decimalLongitude: -4.35912; geodeticDatum: WGS84; **Event:** eventID: C; samplingProtocol: Pitfall
- **Type status:** Other material. **Occurrence:** individualCount: 4; sex: female; **Location:** locationID: C1; continent: Europe; country: Spain; countryCode: ES; stateProvince: Castilla-La Mancha; county: Ciudad Real; locality: Valle Brezoso; verbatimElevation: 756.56; decimalLatitude: 39.35663; decimalLongitude: -4.35912; geodeticDatum: WGS84; **Event:** eventID: C; samplingProtocol: Pitfall
- **Type status:** Other material. **Occurrence:** individualCount: 3; sex: male; **Location:** locationID: C1; continent: Europe; country: Spain; countryCode: ES; stateProvince: Castilla-La Mancha; county: Ciudad Real; locality: Valle Brezoso; verbatimElevation: 756.56; decimalLatitude: 39.35663; decimalLongitude: -4.35912; geodeticDatum: WGS84; **Event:** eventID: D; samplingProtocol: Pitfall
- **Type status:** Other material. **Occurrence:** individualCount: 11; sex: male; **Location:** locationID: C1; continent: Europe; country: Spain; countryCode: ES; stateProvince: Castilla-La Mancha; county: Ciudad Real; locality: Valle Brezoso; verbatimElevation: 756.56; decimalLatitude: 39.35663; decimalLongitude: -4.35912; geodeticDatum: WGS84; **Event:** eventID: E; samplingProtocol: Pitfall
- **Type status:** Other material. **Occurrence:** individualCount: 2; sex: female; **Location:** locationID: C1; continent: Europe; country: Spain; countryCode: ES; stateProvince: Castilla-La Mancha; county: Ciudad Real; locality: Valle Brezoso; verbatimElevation: 756.56; decimalLatitude: 39.35663; decimalLongitude: -4.35912; geodeticDatum: WGS84; **Event:** eventID: E; samplingProtocol: Pitfall
- **Type status:** Other material. **Occurrence:** individualCount: 7; sex: male; **Location:** locationID: C1; continent: Europe; country: Spain; countryCode: ES; stateProvince: Castilla-La Mancha; county: Ciudad Real; locality: Valle Brezoso; verbatimElevation: 756.56; decimalLatitude: 39.35663; decimalLongitude: -4.35912; geodeticDatum: WGS84; **Event:** eventID: F; samplingProtocol: Pitfall
- **Type status:** Other material. **Occurrence:** individualCount: 3; sex: male; **Location:** locationID: C1; continent: Europe; country: Spain; countryCode: ES; stateProvince: Castilla-La Mancha; county: Ciudad Real; locality: Valle Brezoso; verbatimElevation: 756.56; decimalLatitude: 39.35663; decimalLongitude: -4.35912; geodeticDatum: WGS84; **Event:** eventID: G; samplingProtocol: Pitfall
- **Type status:** Other material. **Occurrence:** individualCount: 2; sex: female; **Location:** locationID: C1; continent: Europe; country: Spain; countryCode: ES; stateProvince: Castilla-La Mancha; county: Ciudad Real; locality: Valle Brezoso; verbatimElevation: 756.56; decimalLatitude: 39.35663; decimalLongitude: -4.35912; geodeticDatum: WGS84; **Event:** eventID: G; samplingProtocol: Pitfall
- **Type status:** Other material. **Occurrence:** individualCount: 3; sex: male; **Location:** locationID: C1; continent: Europe; country: Spain; countryCode: ES; stateProvince: Castilla-La Mancha; county: Ciudad Real; locality: Valle Brezoso; verbatimElevation: 756.56; decimalLatitude: 39.35663; decimalLongitude: -4.35912; geodeticDatum: WGS84; **Event:** eventID: I; samplingProtocol: Pitfall
- **Type status:** Other material. **Occurrence:** individualCount: 1; sex: female; **Location:** locationID: C1; continent: Europe; country: Spain; countryCode: ES; stateProvince: Castilla-La Mancha; county: Ciudad Real; locality: Valle Brezoso; verbatimElevation: 756.56; decimalLatitude: 39.35663; decimalLongitude: -4.35912; geodeticDatum: WGS84; **Event:** eventID: I; samplingProtocol: Pitfall
- **Type status:** Other material. **Occurrence:** individualCount: 12; sex: male; **Location:** locationID: C1; continent: Europe; country: Spain; countryCode: ES; stateProvince: Castilla-La Mancha; county: Ciudad Real; locality: Valle Brezoso; verbatimElevation: 756.56; decimalLatitude: 39.35663; decimalLongitude: -4.35912; geodeticDatum: WGS84; **Event:** eventID: J; samplingProtocol: Pitfall
- **Type status:** Other material. **Occurrence:** individualCount: 2; sex: female; **Location:** locationID: C1; continent: Europe; country: Spain; countryCode: ES; stateProvince: Castilla-La Mancha; county: Ciudad Real; locality: Valle Brezoso; verbatimElevation: 756.56; decimalLatitude: 39.35663; decimalLongitude: -4.35912; geodeticDatum: WGS84; **Event:** eventID: J; samplingProtocol: Pitfall
- **Type status:** Other material. **Occurrence:** individualCount: 9; sex: male; **Location:** locationID: C1; continent: Europe; country: Spain; countryCode: ES; stateProvince: Castilla-La Mancha; county: Ciudad Real; locality: Valle Brezoso; verbatimElevation: 756.56; decimalLatitude: 39.35663; decimalLongitude: -4.35912; geodeticDatum: WGS84; **Event:** eventID: K; samplingProtocol: Pitfall
- **Type status:** Other material. **Occurrence:** individualCount: 1; sex: female; **Location:** locationID: C1; continent: Europe; country: Spain; countryCode: ES; stateProvince: Castilla-La Mancha; county: Ciudad Real; locality: Valle Brezoso; verbatimElevation: 756.56; decimalLatitude: 39.35663; decimalLongitude: -4.35912; geodeticDatum: WGS84; **Event:** eventID: K; samplingProtocol: Pitfall
- **Type status:** Other material. **Occurrence:** individualCount: 3; sex: male; **Location:** locationID: C1; continent: Europe; country: Spain; countryCode: ES; stateProvince: Castilla-La Mancha; county: Ciudad Real; locality: Valle Brezoso; verbatimElevation: 756.56; decimalLatitude: 39.35663; decimalLongitude: -4.35912; geodeticDatum: WGS84; **Event:** eventID: L; samplingProtocol: Pitfall
- **Type status:** Other material. **Occurrence:** individualCount: 4; sex: male; **Location:** locationID: C2; continent: Europe; country: Spain; countryCode: ES; stateProvince: Castilla-La Mancha; county: Ciudad Real; locality: Valle Brezoso; verbatimElevation: 739.31; decimalLatitude: 39.35159; decimalLongitude: -4.3589; geodeticDatum: WGS84; **Event:** eventID: A; samplingProtocol: Pitfall
- **Type status:** Other material. **Occurrence:** individualCount: 1; sex: male; **Location:** locationID: C2; continent: Europe; country: Spain; countryCode: ES; stateProvince: Castilla-La Mancha; county: Ciudad Real; locality: Valle Brezoso; verbatimElevation: 739.31; decimalLatitude: 39.35159; decimalLongitude: -4.3589; geodeticDatum: WGS84; **Event:** eventID: C; samplingProtocol: Pitfall
- **Type status:** Other material. **Occurrence:** individualCount: 1; sex: female; **Location:** locationID: C2; continent: Europe; country: Spain; countryCode: ES; stateProvince: Castilla-La Mancha; county: Ciudad Real; locality: Valle Brezoso; verbatimElevation: 739.31; decimalLatitude: 39.35159; decimalLongitude: -4.3589; geodeticDatum: WGS84; **Event:** eventID: C; samplingProtocol: Pitfall
- **Type status:** Other material. **Occurrence:** individualCount: 1; sex: male; **Location:** locationID: C2; continent: Europe; country: Spain; countryCode: ES; stateProvince: Castilla-La Mancha; county: Ciudad Real; locality: Valle Brezoso; verbatimElevation: 739.31; decimalLatitude: 39.35159; decimalLongitude: -4.3589; geodeticDatum: WGS84; **Event:** eventID: D; samplingProtocol: Pitfall
- **Type status:** Other material. **Occurrence:** individualCount: 3; sex: male; **Location:** locationID: C2; continent: Europe; country: Spain; countryCode: ES; stateProvince: Castilla-La Mancha; county: Ciudad Real; locality: Valle Brezoso; verbatimElevation: 739.31; decimalLatitude: 39.35159; decimalLongitude: -4.3589; geodeticDatum: WGS84; **Event:** eventID: E; samplingProtocol: Pitfall
- **Type status:** Other material. **Occurrence:** individualCount: 1; sex: male; **Location:** locationID: C2; continent: Europe; country: Spain; countryCode: ES; stateProvince: Castilla-La Mancha; county: Ciudad Real; locality: Valle Brezoso; verbatimElevation: 739.31; decimalLatitude: 39.35159; decimalLongitude: -4.3589; geodeticDatum: WGS84; **Event:** eventID: F; samplingProtocol: Pitfall
- **Type status:** Other material. **Occurrence:** individualCount: 1; sex: female; **Location:** locationID: C2; continent: Europe; country: Spain; countryCode: ES; stateProvince: Castilla-La Mancha; county: Ciudad Real; locality: Valle Brezoso; verbatimElevation: 739.31; decimalLatitude: 39.35159; decimalLongitude: -4.3589; geodeticDatum: WGS84; **Event:** eventID: G; samplingProtocol: Pitfall
- **Type status:** Other material. **Occurrence:** individualCount: 6; sex: male; **Location:** locationID: C2; continent: Europe; country: Spain; countryCode: ES; stateProvince: Castilla-La Mancha; county: Ciudad Real; locality: Valle Brezoso; verbatimElevation: 739.31; decimalLatitude: 39.35159; decimalLongitude: -4.3589; geodeticDatum: WGS84; **Event:** eventID: H; samplingProtocol: Pitfall
- **Type status:** Other material. **Occurrence:** individualCount: 2; sex: male; **Location:** locationID: C2; continent: Europe; country: Spain; countryCode: ES; stateProvince: Castilla-La Mancha; county: Ciudad Real; locality: Valle Brezoso; verbatimElevation: 739.31; decimalLatitude: 39.35159; decimalLongitude: -4.3589; geodeticDatum: WGS84; **Event:** eventID: J; samplingProtocol: Pitfall
- **Type status:** Other material. **Occurrence:** individualCount: 2; sex: male; **Location:** locationID: C2; continent: Europe; country: Spain; countryCode: ES; stateProvince: Castilla-La Mancha; county: Ciudad Real; locality: Valle Brezoso; verbatimElevation: 739.31; decimalLatitude: 39.35159; decimalLongitude: -4.3589; geodeticDatum: WGS84; **Event:** eventID: K; samplingProtocol: Pitfall
- **Type status:** Other material. **Occurrence:** individualCount: 6; sex: male; **Location:** locationID: C2; continent: Europe; country: Spain; countryCode: ES; stateProvince: Castilla-La Mancha; county: Ciudad Real; locality: Valle Brezoso; verbatimElevation: 739.31; decimalLatitude: 39.35159; decimalLongitude: -4.3589; geodeticDatum: WGS84; **Event:** eventID: L; samplingProtocol: Pitfall
- **Type status:** Other material. **Occurrence:** individualCount: 1; sex: female; **Location:** locationID: C3; continent: Europe; country: Spain; countryCode: ES; stateProvince: Castilla-La Mancha; county: Ciudad Real; locality: La Quesera; verbatimElevation: 767.55; decimalLatitude: 39.36177; decimalLongitude: -4.41733; geodeticDatum: WGS84; **Event:** eventID: B; samplingProtocol: Pitfall
- **Type status:** Other material. **Occurrence:** individualCount: 6; sex: male; **Location:** locationID: C3; continent: Europe; country: Spain; countryCode: ES; stateProvince: Castilla-La Mancha; county: Ciudad Real; locality: La Quesera; verbatimElevation: 767.55; decimalLatitude: 39.36177; decimalLongitude: -4.41733; geodeticDatum: WGS84; **Event:** eventID: L; samplingProtocol: Pitfall
- **Type status:** Other material. **Occurrence:** individualCount: 1; sex: male; **Location:** locationID: C4; continent: Europe; country: Spain; countryCode: ES; stateProvince: Castilla-La Mancha; county: Ciudad Real; locality: La Quesera; verbatimElevation: 772.3; decimalLatitude: 39.36337; decimalLongitude: -4.41704; geodeticDatum: WGS84; **Event:** eventID: C; samplingProtocol: Pitfall
- **Type status:** Other material. **Occurrence:** individualCount: 1; sex: male; **Location:** locationID: C4; continent: Europe; country: Spain; countryCode: ES; stateProvince: Castilla-La Mancha; county: Ciudad Real; locality: La Quesera; verbatimElevation: 772.3; decimalLatitude: 39.36337; decimalLongitude: -4.41704; geodeticDatum: WGS84; **Event:** eventID: F; samplingProtocol: Pitfall
- **Type status:** Other material. **Occurrence:** individualCount: 1; sex: male; **Location:** locationID: C4; continent: Europe; country: Spain; countryCode: ES; stateProvince: Castilla-La Mancha; county: Ciudad Real; locality: La Quesera; verbatimElevation: 772.3; decimalLatitude: 39.36337; decimalLongitude: -4.41704; geodeticDatum: WGS84; **Event:** eventID: G; samplingProtocol: Pitfall
- **Type status:** Other material. **Occurrence:** individualCount: 1; sex: female; **Location:** locationID: C4; continent: Europe; country: Spain; countryCode: ES; stateProvince: Castilla-La Mancha; county: Ciudad Real; locality: La Quesera; verbatimElevation: 772.3; decimalLatitude: 39.36337; decimalLongitude: -4.41704; geodeticDatum: WGS84; **Event:** eventID: G; samplingProtocol: Pitfall
- **Type status:** Other material. **Occurrence:** individualCount: 1; sex: female; **Location:** locationID: C4; continent: Europe; country: Spain; countryCode: ES; stateProvince: Castilla-La Mancha; county: Ciudad Real; locality: La Quesera; verbatimElevation: 772.3; decimalLatitude: 39.36337; decimalLongitude: -4.41704; geodeticDatum: WGS84; **Event:** eventID: I; samplingProtocol: Pitfall
- **Type status:** Other material. **Occurrence:** individualCount: 1; sex: male; **Location:** locationID: C4; continent: Europe; country: Spain; countryCode: ES; stateProvince: Castilla-La Mancha; county: Ciudad Real; locality: La Quesera; verbatimElevation: 772.3; decimalLatitude: 39.36337; decimalLongitude: -4.41704; geodeticDatum: WGS84; **Event:** eventID: L; samplingProtocol: Pitfall
- **Type status:** Other material. **Occurrence:** individualCount: 1; sex: female; **Location:** locationID: M1; continent: Europe; country: Spain; countryCode: ES; stateProvince: Extremadura; county: Cáceres; locality: Peña Falcón; verbatimElevation: 320.6; decimalLatitude: 39.83296; decimalLongitude: -6.0641; geodeticDatum: WGS84; **Event:** eventID: A; samplingProtocol: Pitfall
- **Type status:** Other material. **Occurrence:** individualCount: 1; sex: female; **Location:** locationID: M1; continent: Europe; country: Spain; countryCode: ES; stateProvince: Extremadura; county: Cáceres; locality: Peña Falcón; verbatimElevation: 320.6; decimalLatitude: 39.83296; decimalLongitude: -6.0641; geodeticDatum: WGS84; **Event:** eventID: C; samplingProtocol: Pitfall
- **Type status:** Other material. **Occurrence:** individualCount: 1; sex: female; **Location:** locationID: M1; continent: Europe; country: Spain; countryCode: ES; stateProvince: Extremadura; county: Cáceres; locality: Peña Falcón; verbatimElevation: 320.6; decimalLatitude: 39.83296; decimalLongitude: -6.0641; geodeticDatum: WGS84; **Event:** eventID: E; samplingProtocol: Pitfall
- **Type status:** Other material. **Occurrence:** individualCount: 1; sex: female; **Location:** locationID: M1; continent: Europe; country: Spain; countryCode: ES; stateProvince: Extremadura; county: Cáceres; locality: Peña Falcón; verbatimElevation: 320.6; decimalLatitude: 39.83296; decimalLongitude: -6.0641; geodeticDatum: WGS84; **Event:** eventID: G; samplingProtocol: Pitfall
- **Type status:** Other material. **Occurrence:** individualCount: 1; sex: female; **Location:** locationID: M1; continent: Europe; country: Spain; countryCode: ES; stateProvince: Extremadura; county: Cáceres; locality: Peña Falcón; verbatimElevation: 320.6; decimalLatitude: 39.83296; decimalLongitude: -6.0641; geodeticDatum: WGS84; **Event:** eventID: H; samplingProtocol: Pitfall
- **Type status:** Other material. **Occurrence:** individualCount: 1; sex: female; **Location:** locationID: M1; continent: Europe; country: Spain; countryCode: ES; stateProvince: Extremadura; county: Cáceres; locality: Peña Falcón; verbatimElevation: 320.6; decimalLatitude: 39.83296; decimalLongitude: -6.0641; geodeticDatum: WGS84; **Event:** eventID: I; samplingProtocol: Pitfall
- **Type status:** Other material. **Occurrence:** individualCount: 1; sex: female; **Location:** locationID: M1; continent: Europe; country: Spain; countryCode: ES; stateProvince: Extremadura; county: Cáceres; locality: Peña Falcón; verbatimElevation: 320.6; decimalLatitude: 39.83296; decimalLongitude: -6.0641; geodeticDatum: WGS84; **Event:** eventID: J; samplingProtocol: Pitfall
- **Type status:** Other material. **Occurrence:** individualCount: 1; sex: female; **Location:** locationID: M1; continent: Europe; country: Spain; countryCode: ES; stateProvince: Extremadura; county: Cáceres; locality: Peña Falcón; verbatimElevation: 320.6; decimalLatitude: 39.83296; decimalLongitude: -6.0641; geodeticDatum: WGS84; **Event:** eventID: K; samplingProtocol: Pitfall
- **Type status:** Other material. **Occurrence:** individualCount: 1; sex: female; **Location:** locationID: M2; continent: Europe; country: Spain; countryCode: ES; stateProvince: Extremadura; county: Cáceres; locality: Fuente del Frances; verbatimElevation: 320.72; decimalLatitude: 39.828; decimalLongitude: -6.03249; geodeticDatum: WGS84; **Event:** eventID: 4; samplingProtocol: Aerial; eventTime: Night
- **Type status:** Other material. **Occurrence:** individualCount: 8; sex: male; **Location:** locationID: M2; continent: Europe; country: Spain; countryCode: ES; stateProvince: Extremadura; county: Cáceres; locality: Fuente del Frances; verbatimElevation: 320.72; decimalLatitude: 39.828; decimalLongitude: -6.03249; geodeticDatum: WGS84; **Event:** eventID: A; samplingProtocol: Pitfall
- **Type status:** Other material. **Occurrence:** individualCount: 1; sex: female; **Location:** locationID: M2; continent: Europe; country: Spain; countryCode: ES; stateProvince: Extremadura; county: Cáceres; locality: Fuente del Frances; verbatimElevation: 320.72; decimalLatitude: 39.828; decimalLongitude: -6.03249; geodeticDatum: WGS84; **Event:** eventID: A; samplingProtocol: Pitfall
- **Type status:** Other material. **Occurrence:** individualCount: 2; sex: male; **Location:** locationID: M2; continent: Europe; country: Spain; countryCode: ES; stateProvince: Extremadura; county: Cáceres; locality: Fuente del Frances; verbatimElevation: 320.72; decimalLatitude: 39.828; decimalLongitude: -6.03249; geodeticDatum: WGS84; **Event:** eventID: B; samplingProtocol: Pitfall
- **Type status:** Other material. **Occurrence:** individualCount: 4; sex: male; **Location:** locationID: M2; continent: Europe; country: Spain; countryCode: ES; stateProvince: Extremadura; county: Cáceres; locality: Fuente del Frances; verbatimElevation: 320.72; decimalLatitude: 39.828; decimalLongitude: -6.03249; geodeticDatum: WGS84; **Event:** eventID: C; samplingProtocol: Pitfall
- **Type status:** Other material. **Occurrence:** individualCount: 6; sex: male; **Location:** locationID: M2; continent: Europe; country: Spain; countryCode: ES; stateProvince: Extremadura; county: Cáceres; locality: Fuente del Frances; verbatimElevation: 320.72; decimalLatitude: 39.828; decimalLongitude: -6.03249; geodeticDatum: WGS84; **Event:** eventID: D; samplingProtocol: Pitfall
- **Type status:** Other material. **Occurrence:** individualCount: 14; sex: male; **Location:** locationID: M2; continent: Europe; country: Spain; countryCode: ES; stateProvince: Extremadura; county: Cáceres; locality: Fuente del Frances; verbatimElevation: 320.72; decimalLatitude: 39.828; decimalLongitude: -6.03249; geodeticDatum: WGS84; **Event:** eventID: E; samplingProtocol: Pitfall
- **Type status:** Other material. **Occurrence:** individualCount: 8; sex: male; **Location:** locationID: M2; continent: Europe; country: Spain; countryCode: ES; stateProvince: Extremadura; county: Cáceres; locality: Fuente del Frances; verbatimElevation: 320.72; decimalLatitude: 39.828; decimalLongitude: -6.03249; geodeticDatum: WGS84; **Event:** eventID: F; samplingProtocol: Pitfall
- **Type status:** Other material. **Occurrence:** individualCount: 14; sex: male; **Location:** locationID: M2; continent: Europe; country: Spain; countryCode: ES; stateProvince: Extremadura; county: Cáceres; locality: Fuente del Frances; verbatimElevation: 320.72; decimalLatitude: 39.828; decimalLongitude: -6.03249; geodeticDatum: WGS84; **Event:** eventID: G; samplingProtocol: Pitfall
- **Type status:** Other material. **Occurrence:** individualCount: 1; sex: female; **Location:** locationID: M2; continent: Europe; country: Spain; countryCode: ES; stateProvince: Extremadura; county: Cáceres; locality: Fuente del Frances; verbatimElevation: 320.72; decimalLatitude: 39.828; decimalLongitude: -6.03249; geodeticDatum: WGS84; **Event:** eventID: G; samplingProtocol: Pitfall
- **Type status:** Other material. **Occurrence:** individualCount: 9; sex: male; **Location:** locationID: M2; continent: Europe; country: Spain; countryCode: ES; stateProvince: Extremadura; county: Cáceres; locality: Fuente del Frances; verbatimElevation: 320.72; decimalLatitude: 39.828; decimalLongitude: -6.03249; geodeticDatum: WGS84; **Event:** eventID: H; samplingProtocol: Pitfall
- **Type status:** Other material. **Occurrence:** individualCount: 4; sex: male; **Location:** locationID: M2; continent: Europe; country: Spain; countryCode: ES; stateProvince: Extremadura; county: Cáceres; locality: Fuente del Frances; verbatimElevation: 320.72; decimalLatitude: 39.828; decimalLongitude: -6.03249; geodeticDatum: WGS84; **Event:** eventID: J; samplingProtocol: Pitfall
- **Type status:** Other material. **Occurrence:** individualCount: 2; sex: male; **Location:** locationID: M2; continent: Europe; country: Spain; countryCode: ES; stateProvince: Extremadura; county: Cáceres; locality: Fuente del Frances; verbatimElevation: 320.72; decimalLatitude: 39.828; decimalLongitude: -6.03249; geodeticDatum: WGS84; **Event:** eventID: K; samplingProtocol: Pitfall
- **Type status:** Other material. **Occurrence:** individualCount: 1; sex: female; **Location:** locationID: M2; continent: Europe; country: Spain; countryCode: ES; stateProvince: Extremadura; county: Cáceres; locality: Fuente del Frances; verbatimElevation: 320.72; decimalLatitude: 39.828; decimalLongitude: -6.03249; geodeticDatum: WGS84; **Event:** eventID: K; samplingProtocol: Pitfall
- **Type status:** Other material. **Occurrence:** individualCount: 4; sex: male; **Location:** locationID: M2; continent: Europe; country: Spain; countryCode: ES; stateProvince: Extremadura; county: Cáceres; locality: Fuente del Frances; verbatimElevation: 320.72; decimalLatitude: 39.828; decimalLongitude: -6.03249; geodeticDatum: WGS84; **Event:** eventID: L; samplingProtocol: Pitfall
- **Type status:** Other material. **Occurrence:** individualCount: 1; sex: female; **Location:** locationID: M2; continent: Europe; country: Spain; countryCode: ES; stateProvince: Extremadura; county: Cáceres; locality: Fuente del Frances; verbatimElevation: 320.72; decimalLatitude: 39.828; decimalLongitude: -6.03249; geodeticDatum: WGS84; **Event:** eventID: L; samplingProtocol: Pitfall
- **Type status:** Other material. **Occurrence:** individualCount: 1; sex: female; **Location:** locationID: P1; continent: Europe; country: Spain; countryCode: ES; stateProvince: Castilla y León; county: León; locality: Monte Robledo; verbatimElevation: 1071.58; decimalLatitude: 43.1445; decimalLongitude: -4.92675; geodeticDatum: WGS84; **Event:** eventID: 1; samplingProtocol: Ground; eventTime: Day
- **Type status:** Other material. **Occurrence:** individualCount: 3; sex: female; **Location:** locationID: P1; continent: Europe; country: Spain; countryCode: ES; stateProvince: Castilla y León; county: León; locality: Monte Robledo; verbatimElevation: 1071.58; decimalLatitude: 43.1445; decimalLongitude: -4.92675; geodeticDatum: WGS84; **Event:** eventID: 2; samplingProtocol: Ground; eventTime: Day
- **Type status:** Other material. **Occurrence:** individualCount: 1; sex: male; **Location:** locationID: P1; continent: Europe; country: Spain; countryCode: ES; stateProvince: Castilla y León; county: León; locality: Monte Robledo; verbatimElevation: 1071.58; decimalLatitude: 43.1445; decimalLongitude: -4.92675; geodeticDatum: WGS84; **Event:** eventID: A; samplingProtocol: Pitfall
- **Type status:** Other material. **Occurrence:** individualCount: 2; sex: male; **Location:** locationID: P1; continent: Europe; country: Spain; countryCode: ES; stateProvince: Castilla y León; county: León; locality: Monte Robledo; verbatimElevation: 1071.58; decimalLatitude: 43.1445; decimalLongitude: -4.92675; geodeticDatum: WGS84; **Event:** eventID: B; samplingProtocol: Pitfall
- **Type status:** Other material. **Occurrence:** individualCount: 2; sex: female; **Location:** locationID: P1; continent: Europe; country: Spain; countryCode: ES; stateProvince: Castilla y León; county: León; locality: Monte Robledo; verbatimElevation: 1071.58; decimalLatitude: 43.1445; decimalLongitude: -4.92675; geodeticDatum: WGS84; **Event:** eventID: B; samplingProtocol: Pitfall
- **Type status:** Other material. **Occurrence:** individualCount: 2; sex: male; **Location:** locationID: P1; continent: Europe; country: Spain; countryCode: ES; stateProvince: Castilla y León; county: León; locality: Monte Robledo; verbatimElevation: 1071.58; decimalLatitude: 43.1445; decimalLongitude: -4.92675; geodeticDatum: WGS84; **Event:** eventID: C; samplingProtocol: Pitfall
- **Type status:** Other material. **Occurrence:** individualCount: 4; sex: male; **Location:** locationID: P1; continent: Europe; country: Spain; countryCode: ES; stateProvince: Castilla y León; county: León; locality: Monte Robledo; verbatimElevation: 1071.58; decimalLatitude: 43.1445; decimalLongitude: -4.92675; geodeticDatum: WGS84; **Event:** eventID: E; samplingProtocol: Pitfall
- **Type status:** Other material. **Occurrence:** individualCount: 1; sex: male; **Location:** locationID: P1; continent: Europe; country: Spain; countryCode: ES; stateProvince: Castilla y León; county: León; locality: Monte Robledo; verbatimElevation: 1071.58; decimalLatitude: 43.1445; decimalLongitude: -4.92675; geodeticDatum: WGS84; **Event:** eventID: F; samplingProtocol: Pitfall
- **Type status:** Other material. **Occurrence:** individualCount: 2; sex: male; **Location:** locationID: P1; continent: Europe; country: Spain; countryCode: ES; stateProvince: Castilla y León; county: León; locality: Monte Robledo; verbatimElevation: 1071.58; decimalLatitude: 43.1445; decimalLongitude: -4.92675; geodeticDatum: WGS84; **Event:** eventID: G; samplingProtocol: Pitfall
- **Type status:** Other material. **Occurrence:** individualCount: 7; sex: male; **Location:** locationID: P1; continent: Europe; country: Spain; countryCode: ES; stateProvince: Castilla y León; county: León; locality: Monte Robledo; verbatimElevation: 1071.58; decimalLatitude: 43.1445; decimalLongitude: -4.92675; geodeticDatum: WGS84; **Event:** eventID: H; samplingProtocol: Pitfall
- **Type status:** Other material. **Occurrence:** individualCount: 1; sex: female; **Location:** locationID: P1; continent: Europe; country: Spain; countryCode: ES; stateProvince: Castilla y León; county: León; locality: Monte Robledo; verbatimElevation: 1071.58; decimalLatitude: 43.1445; decimalLongitude: -4.92675; geodeticDatum: WGS84; **Event:** eventID: H; samplingProtocol: Pitfall
- **Type status:** Other material. **Occurrence:** individualCount: 6; sex: male; **Location:** locationID: P1; continent: Europe; country: Spain; countryCode: ES; stateProvince: Castilla y León; county: León; locality: Monte Robledo; verbatimElevation: 1071.58; decimalLatitude: 43.1445; decimalLongitude: -4.92675; geodeticDatum: WGS84; **Event:** eventID: J; samplingProtocol: Pitfall
- **Type status:** Other material. **Occurrence:** individualCount: 1; sex: female; **Location:** locationID: P1; continent: Europe; country: Spain; countryCode: ES; stateProvince: Castilla y León; county: León; locality: Monte Robledo; verbatimElevation: 1071.58; decimalLatitude: 43.1445; decimalLongitude: -4.92675; geodeticDatum: WGS84; **Event:** eventID: K; samplingProtocol: Pitfall
- **Type status:** Other material. **Occurrence:** individualCount: 2; sex: male; **Location:** locationID: P1; continent: Europe; country: Spain; countryCode: ES; stateProvince: Castilla y León; county: León; locality: Monte Robledo; verbatimElevation: 1071.58; decimalLatitude: 43.1445; decimalLongitude: -4.92675; geodeticDatum: WGS84; **Event:** eventID: L; samplingProtocol: Pitfall
- **Type status:** Other material. **Occurrence:** individualCount: 1; sex: male; **Location:** locationID: P3; continent: Europe; country: Spain; countryCode: ES; stateProvince: Castilla y León; county: León; locality: Las Arroyas; verbatimElevation: 1097.1; decimalLatitude: 43.14351; decimalLongitude: -4.94878; geodeticDatum: WGS84; **Event:** eventID: A; samplingProtocol: Pitfall
- **Type status:** Other material. **Occurrence:** individualCount: 1; sex: male; **Location:** locationID: P3; continent: Europe; country: Spain; countryCode: ES; stateProvince: Castilla y León; county: León; locality: Las Arroyas; verbatimElevation: 1097.1; decimalLatitude: 43.14351; decimalLongitude: -4.94878; geodeticDatum: WGS84; **Event:** eventID: C; samplingProtocol: Pitfall
- **Type status:** Other material. **Occurrence:** individualCount: 1; sex: female; **Location:** locationID: P3; continent: Europe; country: Spain; countryCode: ES; stateProvince: Castilla y León; county: León; locality: Las Arroyas; verbatimElevation: 1097.1; decimalLatitude: 43.14351; decimalLongitude: -4.94878; geodeticDatum: WGS84; **Event:** eventID: C; samplingProtocol: Pitfall
- **Type status:** Other material. **Occurrence:** individualCount: 4; sex: male; **Location:** locationID: P3; continent: Europe; country: Spain; countryCode: ES; stateProvince: Castilla y León; county: León; locality: Las Arroyas; verbatimElevation: 1097.1; decimalLatitude: 43.14351; decimalLongitude: -4.94878; geodeticDatum: WGS84; **Event:** eventID: D; samplingProtocol: Pitfall
- **Type status:** Other material. **Occurrence:** individualCount: 4; sex: male; **Location:** locationID: P3; continent: Europe; country: Spain; countryCode: ES; stateProvince: Castilla y León; county: León; locality: Las Arroyas; verbatimElevation: 1097.1; decimalLatitude: 43.14351; decimalLongitude: -4.94878; geodeticDatum: WGS84; **Event:** eventID: E; samplingProtocol: Pitfall
- **Type status:** Other material. **Occurrence:** individualCount: 1; sex: male; **Location:** locationID: P3; continent: Europe; country: Spain; countryCode: ES; stateProvince: Castilla y León; county: León; locality: Las Arroyas; verbatimElevation: 1097.1; decimalLatitude: 43.14351; decimalLongitude: -4.94878; geodeticDatum: WGS84; **Event:** eventID: F; samplingProtocol: Pitfall
- **Type status:** Other material. **Occurrence:** individualCount: 1; sex: female; **Location:** locationID: P3; continent: Europe; country: Spain; countryCode: ES; stateProvince: Castilla y León; county: León; locality: Las Arroyas; verbatimElevation: 1097.1; decimalLatitude: 43.14351; decimalLongitude: -4.94878; geodeticDatum: WGS84; **Event:** eventID: F; samplingProtocol: Pitfall
- **Type status:** Other material. **Occurrence:** individualCount: 1; sex: male; **Location:** locationID: P3; continent: Europe; country: Spain; countryCode: ES; stateProvince: Castilla y León; county: León; locality: Las Arroyas; verbatimElevation: 1097.1; decimalLatitude: 43.14351; decimalLongitude: -4.94878; geodeticDatum: WGS84; **Event:** eventID: G; samplingProtocol: Pitfall
- **Type status:** Other material. **Occurrence:** individualCount: 1; sex: female; **Location:** locationID: P3; continent: Europe; country: Spain; countryCode: ES; stateProvince: Castilla y León; county: León; locality: Las Arroyas; verbatimElevation: 1097.1; decimalLatitude: 43.14351; decimalLongitude: -4.94878; geodeticDatum: WGS84; **Event:** eventID: G; samplingProtocol: Pitfall
- **Type status:** Other material. **Occurrence:** individualCount: 4; sex: male; **Location:** locationID: P3; continent: Europe; country: Spain; countryCode: ES; stateProvince: Castilla y León; county: León; locality: Las Arroyas; verbatimElevation: 1097.1; decimalLatitude: 43.14351; decimalLongitude: -4.94878; geodeticDatum: WGS84; **Event:** eventID: H; samplingProtocol: Pitfall
- **Type status:** Other material. **Occurrence:** individualCount: 1; sex: male; **Location:** locationID: P3; continent: Europe; country: Spain; countryCode: ES; stateProvince: Castilla y León; county: León; locality: Las Arroyas; verbatimElevation: 1097.1; decimalLatitude: 43.14351; decimalLongitude: -4.94878; geodeticDatum: WGS84; **Event:** eventID: I; samplingProtocol: Pitfall
- **Type status:** Other material. **Occurrence:** individualCount: 1; sex: male; **Location:** locationID: P3; continent: Europe; country: Spain; countryCode: ES; stateProvince: Castilla y León; county: León; locality: Las Arroyas; verbatimElevation: 1097.1; decimalLatitude: 43.14351; decimalLongitude: -4.94878; geodeticDatum: WGS84; **Event:** eventID: J; samplingProtocol: Pitfall
- **Type status:** Other material. **Occurrence:** individualCount: 2; sex: male; **Location:** locationID: P3; continent: Europe; country: Spain; countryCode: ES; stateProvince: Castilla y León; county: León; locality: Las Arroyas; verbatimElevation: 1097.1; decimalLatitude: 43.14351; decimalLongitude: -4.94878; geodeticDatum: WGS84; **Event:** eventID: L; samplingProtocol: Pitfall
- **Type status:** Other material. **Occurrence:** individualCount: 1; sex: female; **Location:** locationID: P3; continent: Europe; country: Spain; countryCode: ES; stateProvince: Castilla y León; county: León; locality: Las Arroyas; verbatimElevation: 1097.1; decimalLatitude: 43.14351; decimalLongitude: -4.94878; geodeticDatum: WGS84; **Event:** eventID: L; samplingProtocol: Pitfall

##### Distribution

Iberian Peninsula

#### Eratigena picta

(Simon, 1870)

##### Materials

- **Type status:** Other material. **Occurrence:** individualCount: 2; sex: male; **Location:** locationID: A1; continent: Europe; country: Spain; countryCode: ES; stateProvince: Catalonia; county: Lleida; locality: Sola de Boi; verbatimElevation: 1759.8; decimalLatitude: 42.54958; decimalLongitude: 0.87254; geodeticDatum: WGS84; **Event:** eventID: 2; samplingProtocol: Ground; eventTime: Day
- **Type status:** Other material. **Occurrence:** individualCount: 1; sex: female; **Location:** locationID: A1; continent: Europe; country: Spain; countryCode: ES; stateProvince: Catalonia; county: Lleida; locality: Sola de Boi; verbatimElevation: 1759.8; decimalLatitude: 42.54958; decimalLongitude: 0.87254; geodeticDatum: WGS84; **Event:** eventID: 2; samplingProtocol: Ground; eventTime: Day
- **Type status:** Other material. **Occurrence:** individualCount: 12; sex: male; **Location:** locationID: A1; continent: Europe; country: Spain; countryCode: ES; stateProvince: Catalonia; county: Lleida; locality: Sola de Boi; verbatimElevation: 1759.8; decimalLatitude: 42.54958; decimalLongitude: 0.87254; geodeticDatum: WGS84; **Event:** eventID: A; samplingProtocol: Pitfall
- **Type status:** Other material. **Occurrence:** individualCount: 4; sex: male; **Location:** locationID: A1; continent: Europe; country: Spain; countryCode: ES; stateProvince: Catalonia; county: Lleida; locality: Sola de Boi; verbatimElevation: 1759.8; decimalLatitude: 42.54958; decimalLongitude: 0.87254; geodeticDatum: WGS84; **Event:** eventID: B; samplingProtocol: Pitfall
- **Type status:** Other material. **Occurrence:** individualCount: 4; sex: male; **Location:** locationID: A1; continent: Europe; country: Spain; countryCode: ES; stateProvince: Catalonia; county: Lleida; locality: Sola de Boi; verbatimElevation: 1759.8; decimalLatitude: 42.54958; decimalLongitude: 0.87254; geodeticDatum: WGS84; **Event:** eventID: C; samplingProtocol: Pitfall
- **Type status:** Other material. **Occurrence:** individualCount: 15; sex: male; **Location:** locationID: A1; continent: Europe; country: Spain; countryCode: ES; stateProvince: Catalonia; county: Lleida; locality: Sola de Boi; verbatimElevation: 1759.8; decimalLatitude: 42.54958; decimalLongitude: 0.87254; geodeticDatum: WGS84; **Event:** eventID: D; samplingProtocol: Pitfall
- **Type status:** Other material. **Occurrence:** individualCount: 4; sex: male; **Location:** locationID: A1; continent: Europe; country: Spain; countryCode: ES; stateProvince: Catalonia; county: Lleida; locality: Sola de Boi; verbatimElevation: 1759.8; decimalLatitude: 42.54958; decimalLongitude: 0.87254; geodeticDatum: WGS84; **Event:** eventID: E; samplingProtocol: Pitfall
- **Type status:** Other material. **Occurrence:** individualCount: 1; sex: male; **Location:** locationID: A1; continent: Europe; country: Spain; countryCode: ES; stateProvince: Catalonia; county: Lleida; locality: Sola de Boi; verbatimElevation: 1759.8; decimalLatitude: 42.54958; decimalLongitude: 0.87254; geodeticDatum: WGS84; **Event:** eventID: F; samplingProtocol: Pitfall
- **Type status:** Other material. **Occurrence:** individualCount: 2; sex: male; **Location:** locationID: A1; continent: Europe; country: Spain; countryCode: ES; stateProvince: Catalonia; county: Lleida; locality: Sola de Boi; verbatimElevation: 1759.8; decimalLatitude: 42.54958; decimalLongitude: 0.87254; geodeticDatum: WGS84; **Event:** eventID: G; samplingProtocol: Pitfall
- **Type status:** Other material. **Occurrence:** individualCount: 6; sex: male; **Location:** locationID: A1; continent: Europe; country: Spain; countryCode: ES; stateProvince: Catalonia; county: Lleida; locality: Sola de Boi; verbatimElevation: 1759.8; decimalLatitude: 42.54958; decimalLongitude: 0.87254; geodeticDatum: WGS84; **Event:** eventID: H; samplingProtocol: Pitfall
- **Type status:** Other material. **Occurrence:** individualCount: 3; sex: male; **Location:** locationID: A1; continent: Europe; country: Spain; countryCode: ES; stateProvince: Catalonia; county: Lleida; locality: Sola de Boi; verbatimElevation: 1759.8; decimalLatitude: 42.54958; decimalLongitude: 0.87254; geodeticDatum: WGS84; **Event:** eventID: I; samplingProtocol: Pitfall
- **Type status:** Other material. **Occurrence:** individualCount: 2; sex: male; **Location:** locationID: A1; continent: Europe; country: Spain; countryCode: ES; stateProvince: Catalonia; county: Lleida; locality: Sola de Boi; verbatimElevation: 1759.8; decimalLatitude: 42.54958; decimalLongitude: 0.87254; geodeticDatum: WGS84; **Event:** eventID: J; samplingProtocol: Pitfall
- **Type status:** Other material. **Occurrence:** individualCount: 1; sex: male; **Location:** locationID: A2; continent: Europe; country: Spain; countryCode: ES; stateProvince: Catalonia; county: Lleida; locality: Sola de Boi; verbatimElevation: 1738.7; decimalLatitude: 42.54913; decimalLongitude: 0.87137; geodeticDatum: WGS84; **Event:** eventID: 2; samplingProtocol: Aerial; eventTime: Night
- **Type status:** Other material. **Occurrence:** individualCount: 2; sex: male; **Location:** locationID: A2; continent: Europe; country: Spain; countryCode: ES; stateProvince: Catalonia; county: Lleida; locality: Sola de Boi; verbatimElevation: 1738.7; decimalLatitude: 42.54913; decimalLongitude: 0.87137; geodeticDatum: WGS84; **Event:** eventID: A; samplingProtocol: Pitfall
- **Type status:** Other material. **Occurrence:** individualCount: 3; sex: male; **Location:** locationID: A2; continent: Europe; country: Spain; countryCode: ES; stateProvince: Catalonia; county: Lleida; locality: Sola de Boi; verbatimElevation: 1738.7; decimalLatitude: 42.54913; decimalLongitude: 0.87137; geodeticDatum: WGS84; **Event:** eventID: B; samplingProtocol: Pitfall
- **Type status:** Other material. **Occurrence:** individualCount: 4; sex: male; **Location:** locationID: A2; continent: Europe; country: Spain; countryCode: ES; stateProvince: Catalonia; county: Lleida; locality: Sola de Boi; verbatimElevation: 1738.7; decimalLatitude: 42.54913; decimalLongitude: 0.87137; geodeticDatum: WGS84; **Event:** eventID: D; samplingProtocol: Pitfall
- **Type status:** Other material. **Occurrence:** individualCount: 4; sex: male; **Location:** locationID: A2; continent: Europe; country: Spain; countryCode: ES; stateProvince: Catalonia; county: Lleida; locality: Sola de Boi; verbatimElevation: 1738.7; decimalLatitude: 42.54913; decimalLongitude: 0.87137; geodeticDatum: WGS84; **Event:** eventID: F; samplingProtocol: Pitfall
- **Type status:** Other material. **Occurrence:** individualCount: 1; sex: female; **Location:** locationID: A2; continent: Europe; country: Spain; countryCode: ES; stateProvince: Catalonia; county: Lleida; locality: Sola de Boi; verbatimElevation: 1738.7; decimalLatitude: 42.54913; decimalLongitude: 0.87137; geodeticDatum: WGS84; **Event:** eventID: F; samplingProtocol: Pitfall
- **Type status:** Other material. **Occurrence:** individualCount: 1; sex: male; **Location:** locationID: A2; continent: Europe; country: Spain; countryCode: ES; stateProvince: Catalonia; county: Lleida; locality: Sola de Boi; verbatimElevation: 1738.7; decimalLatitude: 42.54913; decimalLongitude: 0.87137; geodeticDatum: WGS84; **Event:** eventID: G; samplingProtocol: Pitfall
- **Type status:** Other material. **Occurrence:** individualCount: 3; sex: male; **Location:** locationID: A2; continent: Europe; country: Spain; countryCode: ES; stateProvince: Catalonia; county: Lleida; locality: Sola de Boi; verbatimElevation: 1738.7; decimalLatitude: 42.54913; decimalLongitude: 0.87137; geodeticDatum: WGS84; **Event:** eventID: H; samplingProtocol: Pitfall
- **Type status:** Other material. **Occurrence:** individualCount: 1; sex: male; **Location:** locationID: A2; continent: Europe; country: Spain; countryCode: ES; stateProvince: Catalonia; county: Lleida; locality: Sola de Boi; verbatimElevation: 1738.7; decimalLatitude: 42.54913; decimalLongitude: 0.87137; geodeticDatum: WGS84; **Event:** eventID: I; samplingProtocol: Pitfall
- **Type status:** Other material. **Occurrence:** individualCount: 1; sex: male; **Location:** locationID: A2; continent: Europe; country: Spain; countryCode: ES; stateProvince: Catalonia; county: Lleida; locality: Sola de Boi; verbatimElevation: 1738.7; decimalLatitude: 42.54913; decimalLongitude: 0.87137; geodeticDatum: WGS84; **Event:** eventID: K; samplingProtocol: Pitfall
- **Type status:** Other material. **Occurrence:** individualCount: 3; sex: male; **Location:** locationID: A2; continent: Europe; country: Spain; countryCode: ES; stateProvince: Catalonia; county: Lleida; locality: Sola de Boi; verbatimElevation: 1738.7; decimalLatitude: 42.54913; decimalLongitude: 0.87137; geodeticDatum: WGS84; **Event:** eventID: L; samplingProtocol: Pitfall
- **Type status:** Other material. **Occurrence:** individualCount: 1; sex: male; **Location:** locationID: C1; continent: Europe; country: Spain; countryCode: ES; stateProvince: Castilla-La Mancha; county: Ciudad Real; locality: Valle Brezoso; verbatimElevation: 756.56; decimalLatitude: 39.35663; decimalLongitude: -4.35912; geodeticDatum: WGS84; **Event:** eventID: F; samplingProtocol: Pitfall
- **Type status:** Other material. **Occurrence:** individualCount: 1; sex: male; **Location:** locationID: C1; continent: Europe; country: Spain; countryCode: ES; stateProvince: Castilla-La Mancha; county: Ciudad Real; locality: Valle Brezoso; verbatimElevation: 756.56; decimalLatitude: 39.35663; decimalLongitude: -4.35912; geodeticDatum: WGS84; **Event:** eventID: G; samplingProtocol: Pitfall
- **Type status:** Other material. **Occurrence:** individualCount: 1; sex: female; **Location:** locationID: C1; continent: Europe; country: Spain; countryCode: ES; stateProvince: Castilla-La Mancha; county: Ciudad Real; locality: Valle Brezoso; verbatimElevation: 756.56; decimalLatitude: 39.35663; decimalLongitude: -4.35912; geodeticDatum: WGS84; **Event:** eventID: G; samplingProtocol: Pitfall
- **Type status:** Other material. **Occurrence:** individualCount: 2; sex: female; **Location:** locationID: C1; continent: Europe; country: Spain; countryCode: ES; stateProvince: Castilla-La Mancha; county: Ciudad Real; locality: Valle Brezoso; verbatimElevation: 756.56; decimalLatitude: 39.35663; decimalLongitude: -4.35912; geodeticDatum: WGS84; **Event:** eventID: I; samplingProtocol: Pitfall
- **Type status:** Other material. **Occurrence:** individualCount: 1; sex: female; **Location:** locationID: C1; continent: Europe; country: Spain; countryCode: ES; stateProvince: Castilla-La Mancha; county: Ciudad Real; locality: Valle Brezoso; verbatimElevation: 756.56; decimalLatitude: 39.35663; decimalLongitude: -4.35912; geodeticDatum: WGS84; **Event:** eventID: J; samplingProtocol: Pitfall
- **Type status:** Other material. **Occurrence:** individualCount: 1; sex: female; **Location:** locationID: C2; continent: Europe; country: Spain; countryCode: ES; stateProvince: Castilla-La Mancha; county: Ciudad Real; locality: Valle Brezoso; verbatimElevation: 739.31; decimalLatitude: 39.35159; decimalLongitude: -4.3589; geodeticDatum: WGS84; **Event:** eventID: E; samplingProtocol: Pitfall
- **Type status:** Other material. **Occurrence:** individualCount: 4; sex: female; **Location:** locationID: M1; continent: Europe; country: Spain; countryCode: ES; stateProvince: Extremadura; county: Cáceres; locality: Peña Falcón; verbatimElevation: 320.6; decimalLatitude: 39.83296; decimalLongitude: -6.0641; geodeticDatum: WGS84; **Event:** eventID: A; samplingProtocol: Pitfall
- **Type status:** Other material. **Occurrence:** individualCount: 2; sex: female; **Location:** locationID: M1; continent: Europe; country: Spain; countryCode: ES; stateProvince: Extremadura; county: Cáceres; locality: Peña Falcón; verbatimElevation: 320.6; decimalLatitude: 39.83296; decimalLongitude: -6.0641; geodeticDatum: WGS84; **Event:** eventID: B; samplingProtocol: Pitfall
- **Type status:** Other material. **Occurrence:** individualCount: 2; sex: female; **Location:** locationID: M1; continent: Europe; country: Spain; countryCode: ES; stateProvince: Extremadura; county: Cáceres; locality: Peña Falcón; verbatimElevation: 320.6; decimalLatitude: 39.83296; decimalLongitude: -6.0641; geodeticDatum: WGS84; **Event:** eventID: F; samplingProtocol: Pitfall
- **Type status:** Other material. **Occurrence:** individualCount: 2; sex: female; **Location:** locationID: M1; continent: Europe; country: Spain; countryCode: ES; stateProvince: Extremadura; county: Cáceres; locality: Peña Falcón; verbatimElevation: 320.6; decimalLatitude: 39.83296; decimalLongitude: -6.0641; geodeticDatum: WGS84; **Event:** eventID: I; samplingProtocol: Pitfall
- **Type status:** Other material. **Occurrence:** individualCount: 1; sex: female; **Location:** locationID: M1; continent: Europe; country: Spain; countryCode: ES; stateProvince: Extremadura; county: Cáceres; locality: Peña Falcón; verbatimElevation: 320.6; decimalLatitude: 39.83296; decimalLongitude: -6.0641; geodeticDatum: WGS84; **Event:** eventID: J; samplingProtocol: Pitfall
- **Type status:** Other material. **Occurrence:** individualCount: 1; sex: female; **Location:** locationID: M1; continent: Europe; country: Spain; countryCode: ES; stateProvince: Extremadura; county: Cáceres; locality: Peña Falcón; verbatimElevation: 320.6; decimalLatitude: 39.83296; decimalLongitude: -6.0641; geodeticDatum: WGS84; **Event:** eventID: K; samplingProtocol: Pitfall
- **Type status:** Other material. **Occurrence:** individualCount: 1; sex: female; **Location:** locationID: M1; continent: Europe; country: Spain; countryCode: ES; stateProvince: Extremadura; county: Cáceres; locality: Peña Falcón; verbatimElevation: 320.6; decimalLatitude: 39.83296; decimalLongitude: -6.0641; geodeticDatum: WGS84; **Event:** eventID: L; samplingProtocol: Pitfall
- **Type status:** Other material. **Occurrence:** individualCount: 1; sex: female; **Location:** locationID: M2; continent: Europe; country: Spain; countryCode: ES; stateProvince: Extremadura; county: Cáceres; locality: Fuente del Frances; verbatimElevation: 320.72; decimalLatitude: 39.828; decimalLongitude: -6.03249; geodeticDatum: WGS84; **Event:** eventID: A; samplingProtocol: Pitfall
- **Type status:** Other material. **Occurrence:** individualCount: 1; sex: female; **Location:** locationID: M2; continent: Europe; country: Spain; countryCode: ES; stateProvince: Extremadura; county: Cáceres; locality: Fuente del Frances; verbatimElevation: 320.72; decimalLatitude: 39.828; decimalLongitude: -6.03249; geodeticDatum: WGS84; **Event:** eventID: B; samplingProtocol: Pitfall
- **Type status:** Other material. **Occurrence:** individualCount: 1; sex: female; **Location:** locationID: M2; continent: Europe; country: Spain; countryCode: ES; stateProvince: Extremadura; county: Cáceres; locality: Fuente del Frances; verbatimElevation: 320.72; decimalLatitude: 39.828; decimalLongitude: -6.03249; geodeticDatum: WGS84; **Event:** eventID: C; samplingProtocol: Pitfall
- **Type status:** Other material. **Occurrence:** individualCount: 1; sex: female; **Location:** locationID: M2; continent: Europe; country: Spain; countryCode: ES; stateProvince: Extremadura; county: Cáceres; locality: Fuente del Frances; verbatimElevation: 320.72; decimalLatitude: 39.828; decimalLongitude: -6.03249; geodeticDatum: WGS84; **Event:** eventID: E; samplingProtocol: Pitfall
- **Type status:** Other material. **Occurrence:** individualCount: 3; sex: female; **Location:** locationID: M2; continent: Europe; country: Spain; countryCode: ES; stateProvince: Extremadura; county: Cáceres; locality: Fuente del Frances; verbatimElevation: 320.72; decimalLatitude: 39.828; decimalLongitude: -6.03249; geodeticDatum: WGS84; **Event:** eventID: F; samplingProtocol: Pitfall
- **Type status:** Other material. **Occurrence:** individualCount: 2; sex: female; **Location:** locationID: M2; continent: Europe; country: Spain; countryCode: ES; stateProvince: Extremadura; county: Cáceres; locality: Fuente del Frances; verbatimElevation: 320.72; decimalLatitude: 39.828; decimalLongitude: -6.03249; geodeticDatum: WGS84; **Event:** eventID: J; samplingProtocol: Pitfall
- **Type status:** Other material. **Occurrence:** individualCount: 1; sex: female; **Location:** locationID: M2; continent: Europe; country: Spain; countryCode: ES; stateProvince: Extremadura; county: Cáceres; locality: Fuente del Frances; verbatimElevation: 320.72; decimalLatitude: 39.828; decimalLongitude: -6.03249; geodeticDatum: WGS84; **Event:** eventID: L; samplingProtocol: Pitfall
- **Type status:** Other material. **Occurrence:** individualCount: 1; sex: male; **Location:** locationID: O2; continent: Europe; country: Spain; countryCode: ES; stateProvince: Aragón; county: Huesca; locality: Rebilla; verbatimElevation: 1158.13; decimalLatitude: 42.59427; decimalLongitude: 0.1529; geodeticDatum: WGS84; **Event:** eventID: 1; samplingProtocol: Sweep; eventTime: Night
- **Type status:** Other material. **Occurrence:** individualCount: 1; sex: female; **Location:** locationID: O2; continent: Europe; country: Spain; countryCode: ES; stateProvince: Aragón; county: Huesca; locality: Rebilla; verbatimElevation: 1158.13; decimalLatitude: 42.59427; decimalLongitude: 0.1529; geodeticDatum: WGS84; **Event:** eventID: A; samplingProtocol: Pitfall
- **Type status:** Other material. **Occurrence:** individualCount: 1; sex: female; **Location:** locationID: P1; continent: Europe; country: Spain; countryCode: ES; stateProvince: Castilla y León; county: León; locality: Monte Robledo; verbatimElevation: 1071.58; decimalLatitude: 43.1445; decimalLongitude: -4.92675; geodeticDatum: WGS84; **Event:** eventID: 1; samplingProtocol: Ground; eventTime: Day
- **Type status:** Other material. **Occurrence:** individualCount: 1; sex: male; **Location:** locationID: P1; continent: Europe; country: Spain; countryCode: ES; stateProvince: Castilla y León; county: León; locality: Monte Robledo; verbatimElevation: 1071.58; decimalLatitude: 43.1445; decimalLongitude: -4.92675; geodeticDatum: WGS84; **Event:** eventID: A; samplingProtocol: Pitfall
- **Type status:** Other material. **Occurrence:** individualCount: 1; sex: male; **Location:** locationID: P1; continent: Europe; country: Spain; countryCode: ES; stateProvince: Castilla y León; county: León; locality: Monte Robledo; verbatimElevation: 1071.58; decimalLatitude: 43.1445; decimalLongitude: -4.92675; geodeticDatum: WGS84; **Event:** eventID: D; samplingProtocol: Pitfall
- **Type status:** Other material. **Occurrence:** individualCount: 1; sex: male; **Location:** locationID: P1; continent: Europe; country: Spain; countryCode: ES; stateProvince: Castilla y León; county: León; locality: Monte Robledo; verbatimElevation: 1071.58; decimalLatitude: 43.1445; decimalLongitude: -4.92675; geodeticDatum: WGS84; **Event:** eventID: F; samplingProtocol: Pitfall
- **Type status:** Other material. **Occurrence:** individualCount: 1; sex: male; **Location:** locationID: P1; continent: Europe; country: Spain; countryCode: ES; stateProvince: Castilla y León; county: León; locality: Monte Robledo; verbatimElevation: 1071.58; decimalLatitude: 43.1445; decimalLongitude: -4.92675; geodeticDatum: WGS84; **Event:** eventID: K; samplingProtocol: Pitfall
- **Type status:** Other material. **Occurrence:** individualCount: 2; sex: male; **Location:** locationID: P1; continent: Europe; country: Spain; countryCode: ES; stateProvince: Castilla y León; county: León; locality: Monte Robledo; verbatimElevation: 1071.58; decimalLatitude: 43.1445; decimalLongitude: -4.92675; geodeticDatum: WGS84; **Event:** eventID: L; samplingProtocol: Pitfall
- **Type status:** Other material. **Occurrence:** individualCount: 2; sex: male; **Location:** locationID: P2; continent: Europe; country: Spain; countryCode: ES; stateProvince: Castilla y León; county: León; locality: Joyoguelas; verbatimElevation: 763.98; decimalLatitude: 43.17771; decimalLongitude: -4.90579; geodeticDatum: WGS84; **Event:** eventID: C; samplingProtocol: Pitfall
- **Type status:** Other material. **Occurrence:** individualCount: 1; sex: male; **Location:** locationID: P2; continent: Europe; country: Spain; countryCode: ES; stateProvince: Castilla y León; county: León; locality: Joyoguelas; verbatimElevation: 763.98; decimalLatitude: 43.17771; decimalLongitude: -4.90579; geodeticDatum: WGS84; **Event:** eventID: E; samplingProtocol: Pitfall
- **Type status:** Other material. **Occurrence:** individualCount: 2; sex: male; **Location:** locationID: P2; continent: Europe; country: Spain; countryCode: ES; stateProvince: Castilla y León; county: León; locality: Joyoguelas; verbatimElevation: 763.98; decimalLatitude: 43.17771; decimalLongitude: -4.90579; geodeticDatum: WGS84; **Event:** eventID: G; samplingProtocol: Pitfall
- **Type status:** Other material. **Occurrence:** individualCount: 1; sex: male; **Location:** locationID: P2; continent: Europe; country: Spain; countryCode: ES; stateProvince: Castilla y León; county: León; locality: Joyoguelas; verbatimElevation: 763.98; decimalLatitude: 43.17771; decimalLongitude: -4.90579; geodeticDatum: WGS84; **Event:** eventID: H; samplingProtocol: Pitfall
- **Type status:** Other material. **Occurrence:** individualCount: 1; sex: male; **Location:** locationID: P2; continent: Europe; country: Spain; countryCode: ES; stateProvince: Castilla y León; county: León; locality: Joyoguelas; verbatimElevation: 763.98; decimalLatitude: 43.17771; decimalLongitude: -4.90579; geodeticDatum: WGS84; **Event:** eventID: J; samplingProtocol: Pitfall
- **Type status:** Other material. **Occurrence:** individualCount: 2; sex: male; **Location:** locationID: P2; continent: Europe; country: Spain; countryCode: ES; stateProvince: Castilla y León; county: León; locality: Joyoguelas; verbatimElevation: 763.98; decimalLatitude: 43.17771; decimalLongitude: -4.90579; geodeticDatum: WGS84; **Event:** eventID: K; samplingProtocol: Pitfall
- **Type status:** Other material. **Occurrence:** individualCount: 2; sex: male; **Location:** locationID: P2; continent: Europe; country: Spain; countryCode: ES; stateProvince: Castilla y León; county: León; locality: Joyoguelas; verbatimElevation: 763.98; decimalLatitude: 43.17771; decimalLongitude: -4.90579; geodeticDatum: WGS84; **Event:** eventID: L; samplingProtocol: Pitfall
- **Type status:** Other material. **Occurrence:** individualCount: 1; sex: male; **Location:** locationID: P3; continent: Europe; country: Spain; countryCode: ES; stateProvince: Castilla y León; county: León; locality: Las Arroyas; verbatimElevation: 1097.1; decimalLatitude: 43.14351; decimalLongitude: -4.94878; geodeticDatum: WGS84; **Event:** eventID: B; samplingProtocol: Pitfall
- **Type status:** Other material. **Occurrence:** individualCount: 1; sex: male; **Location:** locationID: P4; continent: Europe; country: Spain; countryCode: ES; stateProvince: Castilla y León; county: León; locality: El Canto; verbatimElevation: 943.48; decimalLatitude: 43.17227; decimalLongitude: -4.90857; geodeticDatum: WGS84; **Event:** eventID: A; samplingProtocol: Pitfall
- **Type status:** Other material. **Occurrence:** individualCount: 1; sex: male; **Location:** locationID: P4; continent: Europe; country: Spain; countryCode: ES; stateProvince: Castilla y León; county: León; locality: El Canto; verbatimElevation: 943.48; decimalLatitude: 43.17227; decimalLongitude: -4.90857; geodeticDatum: WGS84; **Event:** eventID: D; samplingProtocol: Pitfall
- **Type status:** Other material. **Occurrence:** individualCount: 2; sex: male; **Location:** locationID: P4; continent: Europe; country: Spain; countryCode: ES; stateProvince: Castilla y León; county: León; locality: El Canto; verbatimElevation: 943.48; decimalLatitude: 43.17227; decimalLongitude: -4.90857; geodeticDatum: WGS84; **Event:** eventID: G; samplingProtocol: Pitfall
- **Type status:** Other material. **Occurrence:** individualCount: 3; sex: male; **Location:** locationID: P4; continent: Europe; country: Spain; countryCode: ES; stateProvince: Castilla y León; county: León; locality: El Canto; verbatimElevation: 943.48; decimalLatitude: 43.17227; decimalLongitude: -4.90857; geodeticDatum: WGS84; **Event:** eventID: H; samplingProtocol: Pitfall
- **Type status:** Other material. **Occurrence:** individualCount: 2; sex: male; **Location:** locationID: P4; continent: Europe; country: Spain; countryCode: ES; stateProvince: Castilla y León; county: León; locality: El Canto; verbatimElevation: 943.48; decimalLatitude: 43.17227; decimalLongitude: -4.90857; geodeticDatum: WGS84; **Event:** eventID: L; samplingProtocol: Pitfall
- **Type status:** Other material. **Occurrence:** individualCount: 2; sex: male; **Location:** locationID: S2; continent: Europe; country: Spain; countryCode: ES; stateProvince: Andalucía; county: Granada; locality: Camarate; verbatimElevation: 1713.96; decimalLatitude: 37.18377; decimalLongitude: -3.26282; geodeticDatum: WGS84; **Event:** eventID: B; samplingProtocol: Pitfall
- **Type status:** Other material. **Occurrence:** individualCount: 1; sex: female; **Location:** locationID: S2; continent: Europe; country: Spain; countryCode: ES; stateProvince: Andalucía; county: Granada; locality: Camarate; verbatimElevation: 1713.96; decimalLatitude: 37.18377; decimalLongitude: -3.26282; geodeticDatum: WGS84; **Event:** eventID: B; samplingProtocol: Pitfall
- **Type status:** Other material. **Occurrence:** individualCount: 3; sex: male; **Location:** locationID: S2; continent: Europe; country: Spain; countryCode: ES; stateProvince: Andalucía; county: Granada; locality: Camarate; verbatimElevation: 1713.96; decimalLatitude: 37.18377; decimalLongitude: -3.26282; geodeticDatum: WGS84; **Event:** eventID: C; samplingProtocol: Pitfall
- **Type status:** Other material. **Occurrence:** individualCount: 1; sex: male; **Location:** locationID: S2; continent: Europe; country: Spain; countryCode: ES; stateProvince: Andalucía; county: Granada; locality: Camarate; verbatimElevation: 1713.96; decimalLatitude: 37.18377; decimalLongitude: -3.26282; geodeticDatum: WGS84; **Event:** eventID: E; samplingProtocol: Pitfall
- **Type status:** Other material. **Occurrence:** individualCount: 1; sex: female; **Location:** locationID: S2; continent: Europe; country: Spain; countryCode: ES; stateProvince: Andalucía; county: Granada; locality: Camarate; verbatimElevation: 1713.96; decimalLatitude: 37.18377; decimalLongitude: -3.26282; geodeticDatum: WGS84; **Event:** eventID: E; samplingProtocol: Pitfall
- **Type status:** Other material. **Occurrence:** individualCount: 1; sex: male; **Location:** locationID: S2; continent: Europe; country: Spain; countryCode: ES; stateProvince: Andalucía; county: Granada; locality: Camarate; verbatimElevation: 1713.96; decimalLatitude: 37.18377; decimalLongitude: -3.26282; geodeticDatum: WGS84; **Event:** eventID: G; samplingProtocol: Pitfall
- **Type status:** Other material. **Occurrence:** individualCount: 1; sex: male; **Location:** locationID: S2; continent: Europe; country: Spain; countryCode: ES; stateProvince: Andalucía; county: Granada; locality: Camarate; verbatimElevation: 1713.96; decimalLatitude: 37.18377; decimalLongitude: -3.26282; geodeticDatum: WGS84; **Event:** eventID: H; samplingProtocol: Pitfall
- **Type status:** Other material. **Occurrence:** individualCount: 1; sex: male; **Location:** locationID: S2; continent: Europe; country: Spain; countryCode: ES; stateProvince: Andalucía; county: Granada; locality: Camarate; verbatimElevation: 1713.96; decimalLatitude: 37.18377; decimalLongitude: -3.26282; geodeticDatum: WGS84; **Event:** eventID: I; samplingProtocol: Pitfall
- **Type status:** Other material. **Occurrence:** individualCount: 1; sex: male; **Location:** locationID: S2; continent: Europe; country: Spain; countryCode: ES; stateProvince: Andalucía; county: Granada; locality: Camarate; verbatimElevation: 1713.96; decimalLatitude: 37.18377; decimalLongitude: -3.26282; geodeticDatum: WGS84; **Event:** eventID: J; samplingProtocol: Pitfall
- **Type status:** Other material. **Occurrence:** individualCount: 2; sex: female; **Location:** locationID: S2; continent: Europe; country: Spain; countryCode: ES; stateProvince: Andalucía; county: Granada; locality: Camarate; verbatimElevation: 1713.96; decimalLatitude: 37.18377; decimalLongitude: -3.26282; geodeticDatum: WGS84; **Event:** eventID: K; samplingProtocol: Pitfall
- **Type status:** Other material. **Occurrence:** individualCount: 2; sex: male; **Location:** locationID: S2; continent: Europe; country: Spain; countryCode: ES; stateProvince: Andalucía; county: Granada; locality: Camarate; verbatimElevation: 1713.96; decimalLatitude: 37.18377; decimalLongitude: -3.26282; geodeticDatum: WGS84; **Event:** eventID: L; samplingProtocol: Pitfall
- **Type status:** Other material. **Occurrence:** individualCount: 3; sex: female; **Location:** locationID: S2; continent: Europe; country: Spain; countryCode: ES; stateProvince: Andalucía; county: Granada; locality: Camarate; verbatimElevation: 1713.96; decimalLatitude: 37.18377; decimalLongitude: -3.26282; geodeticDatum: WGS84; **Event:** eventID: L; samplingProtocol: Pitfall

##### Distribution

Europe, Russia, North Africa

#### Malthonica lusitanica

Simon, 1898

##### Materials

- **Type status:** Other material. **Occurrence:** individualCount: 2; sex: female; **Location:** locationID: P1; continent: Europe; country: Spain; countryCode: ES; stateProvince: Castilla y León; county: León; locality: Monte Robledo; verbatimElevation: 1071.58; decimalLatitude: 43.1445; decimalLongitude: -4.92675; geodeticDatum: WGS84; **Event:** eventID: 1; samplingProtocol: Ground; eventTime: Day
- **Type status:** Other material. **Occurrence:** individualCount: 1; sex: male; **Location:** locationID: P1; continent: Europe; country: Spain; countryCode: ES; stateProvince: Castilla y León; county: León; locality: Monte Robledo; verbatimElevation: 1071.58; decimalLatitude: 43.1445; decimalLongitude: -4.92675; geodeticDatum: WGS84; **Event:** eventID: 2; samplingProtocol: Ground; eventTime: Day
- **Type status:** Other material. **Occurrence:** individualCount: 2; sex: male; **Location:** locationID: P1; continent: Europe; country: Spain; countryCode: ES; stateProvince: Castilla y León; county: León; locality: Monte Robledo; verbatimElevation: 1071.58; decimalLatitude: 43.1445; decimalLongitude: -4.92675; geodeticDatum: WGS84; **Event:** eventID: A; samplingProtocol: Pitfall
- **Type status:** Other material. **Occurrence:** individualCount: 1; sex: female; **Location:** locationID: P1; continent: Europe; country: Spain; countryCode: ES; stateProvince: Castilla y León; county: León; locality: Monte Robledo; verbatimElevation: 1071.58; decimalLatitude: 43.1445; decimalLongitude: -4.92675; geodeticDatum: WGS84; **Event:** eventID: A; samplingProtocol: Pitfall
- **Type status:** Other material. **Occurrence:** individualCount: 5; sex: male; **Location:** locationID: P1; continent: Europe; country: Spain; countryCode: ES; stateProvince: Castilla y León; county: León; locality: Monte Robledo; verbatimElevation: 1071.58; decimalLatitude: 43.1445; decimalLongitude: -4.92675; geodeticDatum: WGS84; **Event:** eventID: B; samplingProtocol: Pitfall
- **Type status:** Other material. **Occurrence:** individualCount: 2; sex: female; **Location:** locationID: P1; continent: Europe; country: Spain; countryCode: ES; stateProvince: Castilla y León; county: León; locality: Monte Robledo; verbatimElevation: 1071.58; decimalLatitude: 43.1445; decimalLongitude: -4.92675; geodeticDatum: WGS84; **Event:** eventID: B; samplingProtocol: Pitfall
- **Type status:** Other material. **Occurrence:** individualCount: 3; sex: male; **Location:** locationID: P1; continent: Europe; country: Spain; countryCode: ES; stateProvince: Castilla y León; county: León; locality: Monte Robledo; verbatimElevation: 1071.58; decimalLatitude: 43.1445; decimalLongitude: -4.92675; geodeticDatum: WGS84; **Event:** eventID: C; samplingProtocol: Pitfall
- **Type status:** Other material. **Occurrence:** individualCount: 5; sex: female; **Location:** locationID: P1; continent: Europe; country: Spain; countryCode: ES; stateProvince: Castilla y León; county: León; locality: Monte Robledo; verbatimElevation: 1071.58; decimalLatitude: 43.1445; decimalLongitude: -4.92675; geodeticDatum: WGS84; **Event:** eventID: C; samplingProtocol: Pitfall
- **Type status:** Other material. **Occurrence:** individualCount: 5; sex: male; **Location:** locationID: P1; continent: Europe; country: Spain; countryCode: ES; stateProvince: Castilla y León; county: León; locality: Monte Robledo; verbatimElevation: 1071.58; decimalLatitude: 43.1445; decimalLongitude: -4.92675; geodeticDatum: WGS84; **Event:** eventID: D; samplingProtocol: Pitfall
- **Type status:** Other material. **Occurrence:** individualCount: 1; sex: female; **Location:** locationID: P1; continent: Europe; country: Spain; countryCode: ES; stateProvince: Castilla y León; county: León; locality: Monte Robledo; verbatimElevation: 1071.58; decimalLatitude: 43.1445; decimalLongitude: -4.92675; geodeticDatum: WGS84; **Event:** eventID: D; samplingProtocol: Pitfall
- **Type status:** Other material. **Occurrence:** individualCount: 1; sex: male; **Location:** locationID: P1; continent: Europe; country: Spain; countryCode: ES; stateProvince: Castilla y León; county: León; locality: Monte Robledo; verbatimElevation: 1071.58; decimalLatitude: 43.1445; decimalLongitude: -4.92675; geodeticDatum: WGS84; **Event:** eventID: E; samplingProtocol: Pitfall
- **Type status:** Other material. **Occurrence:** individualCount: 2; sex: female; **Location:** locationID: P1; continent: Europe; country: Spain; countryCode: ES; stateProvince: Castilla y León; county: León; locality: Monte Robledo; verbatimElevation: 1071.58; decimalLatitude: 43.1445; decimalLongitude: -4.92675; geodeticDatum: WGS84; **Event:** eventID: E; samplingProtocol: Pitfall
- **Type status:** Other material. **Occurrence:** individualCount: 7; sex: male; **Location:** locationID: P1; continent: Europe; country: Spain; countryCode: ES; stateProvince: Castilla y León; county: León; locality: Monte Robledo; verbatimElevation: 1071.58; decimalLatitude: 43.1445; decimalLongitude: -4.92675; geodeticDatum: WGS84; **Event:** eventID: F; samplingProtocol: Pitfall
- **Type status:** Other material. **Occurrence:** individualCount: 2; sex: male; **Location:** locationID: P1; continent: Europe; country: Spain; countryCode: ES; stateProvince: Castilla y León; county: León; locality: Monte Robledo; verbatimElevation: 1071.58; decimalLatitude: 43.1445; decimalLongitude: -4.92675; geodeticDatum: WGS84; **Event:** eventID: G; samplingProtocol: Pitfall
- **Type status:** Other material. **Occurrence:** individualCount: 1; sex: female; **Location:** locationID: P1; continent: Europe; country: Spain; countryCode: ES; stateProvince: Castilla y León; county: León; locality: Monte Robledo; verbatimElevation: 1071.58; decimalLatitude: 43.1445; decimalLongitude: -4.92675; geodeticDatum: WGS84; **Event:** eventID: G; samplingProtocol: Pitfall
- **Type status:** Other material. **Occurrence:** individualCount: 2; sex: male; **Location:** locationID: P1; continent: Europe; country: Spain; countryCode: ES; stateProvince: Castilla y León; county: León; locality: Monte Robledo; verbatimElevation: 1071.58; decimalLatitude: 43.1445; decimalLongitude: -4.92675; geodeticDatum: WGS84; **Event:** eventID: H; samplingProtocol: Pitfall
- **Type status:** Other material. **Occurrence:** individualCount: 4; sex: male; **Location:** locationID: P1; continent: Europe; country: Spain; countryCode: ES; stateProvince: Castilla y León; county: León; locality: Monte Robledo; verbatimElevation: 1071.58; decimalLatitude: 43.1445; decimalLongitude: -4.92675; geodeticDatum: WGS84; **Event:** eventID: J; samplingProtocol: Pitfall
- **Type status:** Other material. **Occurrence:** individualCount: 2; sex: female; **Location:** locationID: P1; continent: Europe; country: Spain; countryCode: ES; stateProvince: Castilla y León; county: León; locality: Monte Robledo; verbatimElevation: 1071.58; decimalLatitude: 43.1445; decimalLongitude: -4.92675; geodeticDatum: WGS84; **Event:** eventID: J; samplingProtocol: Pitfall
- **Type status:** Other material. **Occurrence:** individualCount: 2; sex: male; **Location:** locationID: P1; continent: Europe; country: Spain; countryCode: ES; stateProvince: Castilla y León; county: León; locality: Monte Robledo; verbatimElevation: 1071.58; decimalLatitude: 43.1445; decimalLongitude: -4.92675; geodeticDatum: WGS84; **Event:** eventID: K; samplingProtocol: Pitfall
- **Type status:** Other material. **Occurrence:** individualCount: 5; sex: male; **Location:** locationID: P1; continent: Europe; country: Spain; countryCode: ES; stateProvince: Castilla y León; county: León; locality: Monte Robledo; verbatimElevation: 1071.58; decimalLatitude: 43.1445; decimalLongitude: -4.92675; geodeticDatum: WGS84; **Event:** eventID: L; samplingProtocol: Pitfall
- **Type status:** Other material. **Occurrence:** individualCount: 1; sex: male; **Location:** locationID: P2; continent: Europe; country: Spain; countryCode: ES; stateProvince: Castilla y León; county: León; locality: Joyoguelas; verbatimElevation: 763.98; decimalLatitude: 43.17771; decimalLongitude: -4.90579; geodeticDatum: WGS84; **Event:** eventID: C; samplingProtocol: Pitfall
- **Type status:** Other material. **Occurrence:** individualCount: 1; sex: male; **Location:** locationID: P2; continent: Europe; country: Spain; countryCode: ES; stateProvince: Castilla y León; county: León; locality: Joyoguelas; verbatimElevation: 763.98; decimalLatitude: 43.17771; decimalLongitude: -4.90579; geodeticDatum: WGS84; **Event:** eventID: D; samplingProtocol: Pitfall
- **Type status:** Other material. **Occurrence:** individualCount: 1; sex: male; **Location:** locationID: P2; continent: Europe; country: Spain; countryCode: ES; stateProvince: Castilla y León; county: León; locality: Joyoguelas; verbatimElevation: 763.98; decimalLatitude: 43.17771; decimalLongitude: -4.90579; geodeticDatum: WGS84; **Event:** eventID: E; samplingProtocol: Pitfall
- **Type status:** Other material. **Occurrence:** individualCount: 2; sex: male; **Location:** locationID: P2; continent: Europe; country: Spain; countryCode: ES; stateProvince: Castilla y León; county: León; locality: Joyoguelas; verbatimElevation: 763.98; decimalLatitude: 43.17771; decimalLongitude: -4.90579; geodeticDatum: WGS84; **Event:** eventID: G; samplingProtocol: Pitfall
- **Type status:** Other material. **Occurrence:** individualCount: 5; sex: male; **Location:** locationID: P2; continent: Europe; country: Spain; countryCode: ES; stateProvince: Castilla y León; county: León; locality: Joyoguelas; verbatimElevation: 763.98; decimalLatitude: 43.17771; decimalLongitude: -4.90579; geodeticDatum: WGS84; **Event:** eventID: I; samplingProtocol: Pitfall
- **Type status:** Other material. **Occurrence:** individualCount: 1; sex: male; **Location:** locationID: P2; continent: Europe; country: Spain; countryCode: ES; stateProvince: Castilla y León; county: León; locality: Joyoguelas; verbatimElevation: 763.98; decimalLatitude: 43.17771; decimalLongitude: -4.90579; geodeticDatum: WGS84; **Event:** eventID: J; samplingProtocol: Pitfall
- **Type status:** Other material. **Occurrence:** individualCount: 3; sex: male; **Location:** locationID: P2; continent: Europe; country: Spain; countryCode: ES; stateProvince: Castilla y León; county: León; locality: Joyoguelas; verbatimElevation: 763.98; decimalLatitude: 43.17771; decimalLongitude: -4.90579; geodeticDatum: WGS84; **Event:** eventID: K; samplingProtocol: Pitfall
- **Type status:** Other material. **Occurrence:** individualCount: 2; sex: male; **Location:** locationID: P2; continent: Europe; country: Spain; countryCode: ES; stateProvince: Castilla y León; county: León; locality: Joyoguelas; verbatimElevation: 763.98; decimalLatitude: 43.17771; decimalLongitude: -4.90579; geodeticDatum: WGS84; **Event:** eventID: L; samplingProtocol: Pitfall
- **Type status:** Other material. **Occurrence:** individualCount: 1; sex: male; **Location:** locationID: P3; continent: Europe; country: Spain; countryCode: ES; stateProvince: Castilla y León; county: León; locality: Las Arroyas; verbatimElevation: 1097.1; decimalLatitude: 43.14351; decimalLongitude: -4.94878; geodeticDatum: WGS84; **Event:** eventID: A; samplingProtocol: Pitfall
- **Type status:** Other material. **Occurrence:** individualCount: 2; sex: male; **Location:** locationID: P3; continent: Europe; country: Spain; countryCode: ES; stateProvince: Castilla y León; county: León; locality: Las Arroyas; verbatimElevation: 1097.1; decimalLatitude: 43.14351; decimalLongitude: -4.94878; geodeticDatum: WGS84; **Event:** eventID: C; samplingProtocol: Pitfall
- **Type status:** Other material. **Occurrence:** individualCount: 1; sex: male; **Location:** locationID: P3; continent: Europe; country: Spain; countryCode: ES; stateProvince: Castilla y León; county: León; locality: Las Arroyas; verbatimElevation: 1097.1; decimalLatitude: 43.14351; decimalLongitude: -4.94878; geodeticDatum: WGS84; **Event:** eventID: D; samplingProtocol: Pitfall
- **Type status:** Other material. **Occurrence:** individualCount: 1; sex: male; **Location:** locationID: P3; continent: Europe; country: Spain; countryCode: ES; stateProvince: Castilla y León; county: León; locality: Las Arroyas; verbatimElevation: 1097.1; decimalLatitude: 43.14351; decimalLongitude: -4.94878; geodeticDatum: WGS84; **Event:** eventID: E; samplingProtocol: Pitfall
- **Type status:** Other material. **Occurrence:** individualCount: 3; sex: male; **Location:** locationID: P3; continent: Europe; country: Spain; countryCode: ES; stateProvince: Castilla y León; county: León; locality: Las Arroyas; verbatimElevation: 1097.1; decimalLatitude: 43.14351; decimalLongitude: -4.94878; geodeticDatum: WGS84; **Event:** eventID: F; samplingProtocol: Pitfall
- **Type status:** Other material. **Occurrence:** individualCount: 3; sex: male; **Location:** locationID: P3; continent: Europe; country: Spain; countryCode: ES; stateProvince: Castilla y León; county: León; locality: Las Arroyas; verbatimElevation: 1097.1; decimalLatitude: 43.14351; decimalLongitude: -4.94878; geodeticDatum: WGS84; **Event:** eventID: G; samplingProtocol: Pitfall
- **Type status:** Other material. **Occurrence:** individualCount: 4; sex: male; **Location:** locationID: P3; continent: Europe; country: Spain; countryCode: ES; stateProvince: Castilla y León; county: León; locality: Las Arroyas; verbatimElevation: 1097.1; decimalLatitude: 43.14351; decimalLongitude: -4.94878; geodeticDatum: WGS84; **Event:** eventID: H; samplingProtocol: Pitfall
- **Type status:** Other material. **Occurrence:** individualCount: 6; sex: male; **Location:** locationID: P3; continent: Europe; country: Spain; countryCode: ES; stateProvince: Castilla y León; county: León; locality: Las Arroyas; verbatimElevation: 1097.1; decimalLatitude: 43.14351; decimalLongitude: -4.94878; geodeticDatum: WGS84; **Event:** eventID: I; samplingProtocol: Pitfall
- **Type status:** Other material. **Occurrence:** individualCount: 1; sex: male; **Location:** locationID: P3; continent: Europe; country: Spain; countryCode: ES; stateProvince: Castilla y León; county: León; locality: Las Arroyas; verbatimElevation: 1097.1; decimalLatitude: 43.14351; decimalLongitude: -4.94878; geodeticDatum: WGS84; **Event:** eventID: J; samplingProtocol: Pitfall
- **Type status:** Other material. **Occurrence:** individualCount: 7; sex: male; **Location:** locationID: P3; continent: Europe; country: Spain; countryCode: ES; stateProvince: Castilla y León; county: León; locality: Las Arroyas; verbatimElevation: 1097.1; decimalLatitude: 43.14351; decimalLongitude: -4.94878; geodeticDatum: WGS84; **Event:** eventID: K; samplingProtocol: Pitfall
- **Type status:** Other material. **Occurrence:** individualCount: 4; sex: male; **Location:** locationID: P3; continent: Europe; country: Spain; countryCode: ES; stateProvince: Castilla y León; county: León; locality: Las Arroyas; verbatimElevation: 1097.1; decimalLatitude: 43.14351; decimalLongitude: -4.94878; geodeticDatum: WGS84; **Event:** eventID: L; samplingProtocol: Pitfall
- **Type status:** Other material. **Occurrence:** individualCount: 1; sex: female; **Location:** locationID: P3; continent: Europe; country: Spain; countryCode: ES; stateProvince: Castilla y León; county: León; locality: Las Arroyas; verbatimElevation: 1097.1; decimalLatitude: 43.14351; decimalLongitude: -4.94878; geodeticDatum: WGS84; **Event:** eventID: L; samplingProtocol: Pitfall
- **Type status:** Other material. **Occurrence:** individualCount: 3; sex: male; **Location:** locationID: P4; continent: Europe; country: Spain; countryCode: ES; stateProvince: Castilla y León; county: León; locality: El Canto; verbatimElevation: 943.48; decimalLatitude: 43.17227; decimalLongitude: -4.90857; geodeticDatum: WGS84; **Event:** eventID: A; samplingProtocol: Pitfall
- **Type status:** Other material. **Occurrence:** individualCount: 1; sex: male; **Location:** locationID: P4; continent: Europe; country: Spain; countryCode: ES; stateProvince: Castilla y León; county: León; locality: El Canto; verbatimElevation: 943.48; decimalLatitude: 43.17227; decimalLongitude: -4.90857; geodeticDatum: WGS84; **Event:** eventID: B; samplingProtocol: Pitfall
- **Type status:** Other material. **Occurrence:** individualCount: 1; sex: male; **Location:** locationID: P4; continent: Europe; country: Spain; countryCode: ES; stateProvince: Castilla y León; county: León; locality: El Canto; verbatimElevation: 943.48; decimalLatitude: 43.17227; decimalLongitude: -4.90857; geodeticDatum: WGS84; **Event:** eventID: C; samplingProtocol: Pitfall
- **Type status:** Other material. **Occurrence:** individualCount: 1; sex: male; **Location:** locationID: P4; continent: Europe; country: Spain; countryCode: ES; stateProvince: Castilla y León; county: León; locality: El Canto; verbatimElevation: 943.48; decimalLatitude: 43.17227; decimalLongitude: -4.90857; geodeticDatum: WGS84; **Event:** eventID: D; samplingProtocol: Pitfall
- **Type status:** Other material. **Occurrence:** individualCount: 2; sex: male; **Location:** locationID: P4; continent: Europe; country: Spain; countryCode: ES; stateProvince: Castilla y León; county: León; locality: El Canto; verbatimElevation: 943.48; decimalLatitude: 43.17227; decimalLongitude: -4.90857; geodeticDatum: WGS84; **Event:** eventID: E; samplingProtocol: Pitfall
- **Type status:** Other material. **Occurrence:** individualCount: 8; sex: male; **Location:** locationID: P4; continent: Europe; country: Spain; countryCode: ES; stateProvince: Castilla y León; county: León; locality: El Canto; verbatimElevation: 943.48; decimalLatitude: 43.17227; decimalLongitude: -4.90857; geodeticDatum: WGS84; **Event:** eventID: G; samplingProtocol: Pitfall
- **Type status:** Other material. **Occurrence:** individualCount: 7; sex: male; **Location:** locationID: P4; continent: Europe; country: Spain; countryCode: ES; stateProvince: Castilla y León; county: León; locality: El Canto; verbatimElevation: 943.48; decimalLatitude: 43.17227; decimalLongitude: -4.90857; geodeticDatum: WGS84; **Event:** eventID: H; samplingProtocol: Pitfall
- **Type status:** Other material. **Occurrence:** individualCount: 1; sex: female; **Location:** locationID: P4; continent: Europe; country: Spain; countryCode: ES; stateProvince: Castilla y León; county: León; locality: El Canto; verbatimElevation: 943.48; decimalLatitude: 43.17227; decimalLongitude: -4.90857; geodeticDatum: WGS84; **Event:** eventID: H; samplingProtocol: Pitfall
- **Type status:** Other material. **Occurrence:** individualCount: 8; sex: male; **Location:** locationID: P4; continent: Europe; country: Spain; countryCode: ES; stateProvince: Castilla y León; county: León; locality: El Canto; verbatimElevation: 943.48; decimalLatitude: 43.17227; decimalLongitude: -4.90857; geodeticDatum: WGS84; **Event:** eventID: I; samplingProtocol: Pitfall
- **Type status:** Other material. **Occurrence:** individualCount: 1; sex: female; **Location:** locationID: P4; continent: Europe; country: Spain; countryCode: ES; stateProvince: Castilla y León; county: León; locality: El Canto; verbatimElevation: 943.48; decimalLatitude: 43.17227; decimalLongitude: -4.90857; geodeticDatum: WGS84; **Event:** eventID: I; samplingProtocol: Pitfall
- **Type status:** Other material. **Occurrence:** individualCount: 2; sex: male; **Location:** locationID: P4; continent: Europe; country: Spain; countryCode: ES; stateProvince: Castilla y León; county: León; locality: El Canto; verbatimElevation: 943.48; decimalLatitude: 43.17227; decimalLongitude: -4.90857; geodeticDatum: WGS84; **Event:** eventID: J; samplingProtocol: Pitfall
- **Type status:** Other material. **Occurrence:** individualCount: 3; sex: male; **Location:** locationID: P4; continent: Europe; country: Spain; countryCode: ES; stateProvince: Castilla y León; county: León; locality: El Canto; verbatimElevation: 943.48; decimalLatitude: 43.17227; decimalLongitude: -4.90857; geodeticDatum: WGS84; **Event:** eventID: K; samplingProtocol: Pitfall
- **Type status:** Other material. **Occurrence:** individualCount: 8; sex: male; **Location:** locationID: P4; continent: Europe; country: Spain; countryCode: ES; stateProvince: Castilla y León; county: León; locality: El Canto; verbatimElevation: 943.48; decimalLatitude: 43.17227; decimalLongitude: -4.90857; geodeticDatum: WGS84; **Event:** eventID: L; samplingProtocol: Pitfall
- **Type status:** Other material. **Occurrence:** individualCount: 1; sex: female; **Location:** locationID: P4; continent: Europe; country: Spain; countryCode: ES; stateProvince: Castilla y León; county: León; locality: El Canto; verbatimElevation: 943.48; decimalLatitude: 43.17227; decimalLongitude: -4.90857; geodeticDatum: WGS84; **Event:** eventID: L; samplingProtocol: Pitfall

##### Distribution

Portugal to France

#### Textrix caudata

L. Koch, 1872

##### Materials

- **Type status:** Other material. **Occurrence:** individualCount: 1; sex: male; **Location:** locationID: S1; continent: Europe; country: Spain; countryCode: ES; stateProvince: Andalucía; county: Granada; locality: Soportujar; verbatimElevation: 1786.57; decimalLatitude: 36.96151; decimalLongitude: -3.41881; geodeticDatum: WGS84; **Event:** eventID: 3; samplingProtocol: Aerial; eventTime: Night
- **Type status:** Other material. **Occurrence:** individualCount: 2; sex: female; **Location:** locationID: S1; continent: Europe; country: Spain; countryCode: ES; stateProvince: Andalucía; county: Granada; locality: Soportujar; verbatimElevation: 1786.57; decimalLatitude: 36.96151; decimalLongitude: -3.41881; geodeticDatum: WGS84; **Event:** eventID: 4; samplingProtocol: Aerial; eventTime: Night
- **Type status:** Other material. **Occurrence:** individualCount: 1; sex: male; **Location:** locationID: S2; continent: Europe; country: Spain; countryCode: ES; stateProvince: Andalucía; county: Granada; locality: Camarate; verbatimElevation: 1713.96; decimalLatitude: 37.18377; decimalLongitude: -3.26282; geodeticDatum: WGS84; **Event:** eventID: 2; samplingProtocol: Aerial; eventTime: Night
- **Type status:** Other material. **Occurrence:** individualCount: 1; sex: male; **Location:** locationID: S2; continent: Europe; country: Spain; countryCode: ES; stateProvince: Andalucía; county: Granada; locality: Camarate; verbatimElevation: 1713.96; decimalLatitude: 37.18377; decimalLongitude: -3.26282; geodeticDatum: WGS84; **Event:** eventID: 3; samplingProtocol: Aerial; eventTime: Night
- **Type status:** Other material. **Occurrence:** individualCount: 1; sex: male; **Location:** locationID: S2; continent: Europe; country: Spain; countryCode: ES; stateProvince: Andalucía; county: Granada; locality: Camarate; verbatimElevation: 1713.96; decimalLatitude: 37.18377; decimalLongitude: -3.26282; geodeticDatum: WGS84; **Event:** eventID: I; samplingProtocol: Pitfall
- **Type status:** Other material. **Occurrence:** individualCount: 1; sex: male; **Location:** locationID: S2; continent: Europe; country: Spain; countryCode: ES; stateProvince: Andalucía; county: Granada; locality: Camarate; verbatimElevation: 1713.96; decimalLatitude: 37.18377; decimalLongitude: -3.26282; geodeticDatum: WGS84; **Event:** eventID: J; samplingProtocol: Pitfall

##### Distribution

Mediterranean, introduced in Central Europe

#### Textrix denticulata

(Olivier, 1789)

##### Materials

- **Type status:** Other material. **Occurrence:** individualCount: 1; sex: female; **Location:** locationID: A1; continent: Europe; country: Spain; countryCode: ES; stateProvince: Catalonia; county: Lleida; locality: Sola de Boi; verbatimElevation: 1759.8; decimalLatitude: 42.54958; decimalLongitude: 0.87254; geodeticDatum: WGS84; **Event:** eventID: 1; samplingProtocol: Aerial; eventTime: Night
- **Type status:** Other material. **Occurrence:** individualCount: 7; sex: male; **Location:** locationID: A1; continent: Europe; country: Spain; countryCode: ES; stateProvince: Catalonia; county: Lleida; locality: Sola de Boi; verbatimElevation: 1759.8; decimalLatitude: 42.54958; decimalLongitude: 0.87254; geodeticDatum: WGS84; **Event:** eventID: 1; samplingProtocol: Aerial; eventTime: Night
- **Type status:** Other material. **Occurrence:** individualCount: 16; sex: female; **Location:** locationID: A1; continent: Europe; country: Spain; countryCode: ES; stateProvince: Catalonia; county: Lleida; locality: Sola de Boi; verbatimElevation: 1759.8; decimalLatitude: 42.54958; decimalLongitude: 0.87254; geodeticDatum: WGS84; **Event:** eventID: 1; samplingProtocol: Aerial; eventTime: Night
- **Type status:** Other material. **Occurrence:** individualCount: 3; sex: male; **Location:** locationID: A1; continent: Europe; country: Spain; countryCode: ES; stateProvince: Catalonia; county: Lleida; locality: Sola de Boi; verbatimElevation: 1759.8; decimalLatitude: 42.54958; decimalLongitude: 0.87254; geodeticDatum: WGS84; **Event:** eventID: 2; samplingProtocol: Aerial; eventTime: Night
- **Type status:** Other material. **Occurrence:** individualCount: 7; sex: female; **Location:** locationID: A1; continent: Europe; country: Spain; countryCode: ES; stateProvince: Catalonia; county: Lleida; locality: Sola de Boi; verbatimElevation: 1759.8; decimalLatitude: 42.54958; decimalLongitude: 0.87254; geodeticDatum: WGS84; **Event:** eventID: 2; samplingProtocol: Aerial; eventTime: Night
- **Type status:** Other material. **Occurrence:** individualCount: 1; sex: male; **Location:** locationID: A1; continent: Europe; country: Spain; countryCode: ES; stateProvince: Catalonia; county: Lleida; locality: Sola de Boi; verbatimElevation: 1759.8; decimalLatitude: 42.54958; decimalLongitude: 0.87254; geodeticDatum: WGS84; **Event:** eventID: B; samplingProtocol: Pitfall
- **Type status:** Other material. **Occurrence:** individualCount: 1; sex: male; **Location:** locationID: A1; continent: Europe; country: Spain; countryCode: ES; stateProvince: Catalonia; county: Lleida; locality: Sola de Boi; verbatimElevation: 1759.8; decimalLatitude: 42.54958; decimalLongitude: 0.87254; geodeticDatum: WGS84; **Event:** eventID: D; samplingProtocol: Pitfall
- **Type status:** Other material. **Occurrence:** individualCount: 1; sex: male; **Location:** locationID: A2; continent: Europe; country: Spain; countryCode: ES; stateProvince: Catalonia; county: Lleida; locality: Sola de Boi; verbatimElevation: 1738.7; decimalLatitude: 42.54913; decimalLongitude: 0.87137; geodeticDatum: WGS84; **Event:** eventID: 1; samplingProtocol: Aerial; eventTime: Night
- **Type status:** Other material. **Occurrence:** individualCount: 1; sex: female; **Location:** locationID: A2; continent: Europe; country: Spain; countryCode: ES; stateProvince: Catalonia; county: Lleida; locality: Sola de Boi; verbatimElevation: 1738.7; decimalLatitude: 42.54913; decimalLongitude: 0.87137; geodeticDatum: WGS84; **Event:** eventID: 1; samplingProtocol: Aerial; eventTime: Night
- **Type status:** Other material. **Occurrence:** individualCount: 3; sex: female; **Location:** locationID: A2; continent: Europe; country: Spain; countryCode: ES; stateProvince: Catalonia; county: Lleida; locality: Sola de Boi; verbatimElevation: 1738.7; decimalLatitude: 42.54913; decimalLongitude: 0.87137; geodeticDatum: WGS84; **Event:** eventID: 1; samplingProtocol: Aerial; eventTime: Night
- **Type status:** Other material. **Occurrence:** individualCount: 1; sex: female; **Location:** locationID: A2; continent: Europe; country: Spain; countryCode: ES; stateProvince: Catalonia; county: Lleida; locality: Sola de Boi; verbatimElevation: 1738.7; decimalLatitude: 42.54913; decimalLongitude: 0.87137; geodeticDatum: WGS84; **Event:** eventID: 2; samplingProtocol: Aerial; eventTime: Night
- **Type status:** Other material. **Occurrence:** individualCount: 1; sex: female; **Location:** locationID: A2; continent: Europe; country: Spain; countryCode: ES; stateProvince: Catalonia; county: Lleida; locality: Sola de Boi; verbatimElevation: 1738.7; decimalLatitude: 42.54913; decimalLongitude: 0.87137; geodeticDatum: WGS84; **Event:** eventID: 1; samplingProtocol: Beating; eventTime: Day
- **Type status:** Other material. **Occurrence:** individualCount: 3; sex: male; **Location:** locationID: P1; continent: Europe; country: Spain; countryCode: ES; stateProvince: Castilla y León; county: León; locality: Monte Robledo; verbatimElevation: 1071.58; decimalLatitude: 43.1445; decimalLongitude: -4.92675; geodeticDatum: WGS84; **Event:** eventID: 1; samplingProtocol: Aerial; eventTime: Night
- **Type status:** Other material. **Occurrence:** individualCount: 3; sex: female; **Location:** locationID: P1; continent: Europe; country: Spain; countryCode: ES; stateProvince: Castilla y León; county: León; locality: Monte Robledo; verbatimElevation: 1071.58; decimalLatitude: 43.1445; decimalLongitude: -4.92675; geodeticDatum: WGS84; **Event:** eventID: 1; samplingProtocol: Aerial; eventTime: Night
- **Type status:** Other material. **Occurrence:** individualCount: 2; sex: male; **Location:** locationID: P1; continent: Europe; country: Spain; countryCode: ES; stateProvince: Castilla y León; county: León; locality: Monte Robledo; verbatimElevation: 1071.58; decimalLatitude: 43.1445; decimalLongitude: -4.92675; geodeticDatum: WGS84; **Event:** eventID: 1; samplingProtocol: Aerial; eventTime: Night
- **Type status:** Other material. **Occurrence:** individualCount: 3; sex: female; **Location:** locationID: P1; continent: Europe; country: Spain; countryCode: ES; stateProvince: Castilla y León; county: León; locality: Monte Robledo; verbatimElevation: 1071.58; decimalLatitude: 43.1445; decimalLongitude: -4.92675; geodeticDatum: WGS84; **Event:** eventID: 1; samplingProtocol: Aerial; eventTime: Night
- **Type status:** Other material. **Occurrence:** individualCount: 1; sex: female; **Location:** locationID: P2; continent: Europe; country: Spain; countryCode: ES; stateProvince: Castilla y León; county: León; locality: Joyoguelas; verbatimElevation: 763.98; decimalLatitude: 43.17771; decimalLongitude: -4.90579; geodeticDatum: WGS84; **Event:** eventID: 1; samplingProtocol: Aerial; eventTime: Night
- **Type status:** Other material. **Occurrence:** individualCount: 1; sex: female; **Location:** locationID: P3; continent: Europe; country: Spain; countryCode: ES; stateProvince: Castilla y León; county: León; locality: Las Arroyas; verbatimElevation: 1097.1; decimalLatitude: 43.14351; decimalLongitude: -4.94878; geodeticDatum: WGS84; **Event:** eventID: 1; samplingProtocol: Aerial; eventTime: Night
- **Type status:** Other material. **Occurrence:** individualCount: 1; sex: female; **Location:** locationID: P3; continent: Europe; country: Spain; countryCode: ES; stateProvince: Castilla y León; county: León; locality: Las Arroyas; verbatimElevation: 1097.1; decimalLatitude: 43.14351; decimalLongitude: -4.94878; geodeticDatum: WGS84; **Event:** eventID: 1; samplingProtocol: Aerial; eventTime: Night
- **Type status:** Other material. **Occurrence:** individualCount: 1; sex: female; **Location:** locationID: P4; continent: Europe; country: Spain; countryCode: ES; stateProvince: Castilla y León; county: León; locality: El Canto; verbatimElevation: 943.48; decimalLatitude: 43.17227; decimalLongitude: -4.90857; geodeticDatum: WGS84; **Event:** eventID: 1; samplingProtocol: Aerial; eventTime: Night
- **Type status:** Other material. **Occurrence:** individualCount: 1; sex: female; **Location:** locationID: P4; continent: Europe; country: Spain; countryCode: ES; stateProvince: Castilla y León; county: León; locality: El Canto; verbatimElevation: 943.48; decimalLatitude: 43.17227; decimalLongitude: -4.90857; geodeticDatum: WGS84; **Event:** eventID: 2; samplingProtocol: Aerial; eventTime: Night

##### Distribution

Europe

#### Textrix pinicola

Simon, 1875

##### Materials

- **Type status:** Other material. **Occurrence:** individualCount: 2; sex: male; **Location:** locationID: M1; continent: Europe; country: Spain; countryCode: ES; stateProvince: Extremadura; county: Cáceres; locality: Peña Falcón; verbatimElevation: 320.6; decimalLatitude: 39.83296; decimalLongitude: -6.0641; geodeticDatum: WGS84; **Event:** eventID: C; samplingProtocol: Pitfall
- **Type status:** Other material. **Occurrence:** individualCount: 1; sex: female; **Location:** locationID: M2; continent: Europe; country: Spain; countryCode: ES; stateProvince: Extremadura; county: Cáceres; locality: Fuente del Frances; verbatimElevation: 320.72; decimalLatitude: 39.828; decimalLongitude: -6.03249; geodeticDatum: WGS84; **Event:** eventID: 2; samplingProtocol: Aerial; eventTime: Night
- **Type status:** Other material. **Occurrence:** individualCount: 1; sex: male; **Location:** locationID: M2; continent: Europe; country: Spain; countryCode: ES; stateProvince: Extremadura; county: Cáceres; locality: Fuente del Frances; verbatimElevation: 320.72; decimalLatitude: 39.828; decimalLongitude: -6.03249; geodeticDatum: WGS84; **Event:** eventID: 3; samplingProtocol: Aerial; eventTime: Night
- **Type status:** Other material. **Occurrence:** individualCount: 2; sex: male; **Location:** locationID: M2; continent: Europe; country: Spain; countryCode: ES; stateProvince: Extremadura; county: Cáceres; locality: Fuente del Frances; verbatimElevation: 320.72; decimalLatitude: 39.828; decimalLongitude: -6.03249; geodeticDatum: WGS84; **Event:** samplingProtocol: Pitfall
- **Type status:** Other material. **Occurrence:** individualCount: 1; sex: male; **Location:** locationID: M2; continent: Europe; country: Spain; countryCode: ES; stateProvince: Extremadura; county: Cáceres; locality: Fuente del Frances; verbatimElevation: 320.72; decimalLatitude: 39.828; decimalLongitude: -6.03249; geodeticDatum: WGS84; **Event:** samplingProtocol: Pitfall
- **Type status:** Other material. **Occurrence:** individualCount: 1; sex: male; **Location:** locationID: M2; continent: Europe; country: Spain; countryCode: ES; stateProvince: Extremadura; county: Cáceres; locality: Fuente del Frances; verbatimElevation: 320.72; decimalLatitude: 39.828; decimalLongitude: -6.03249; geodeticDatum: WGS84; **Event:** samplingProtocol: Pitfall

##### Distribution

Portugal to Italy

#### Anyphaenidae

Bertkau, 1878

#### Anyphaena accentuata

(Walckenaer, 1802)

##### Materials

- **Type status:** Other material. **Occurrence:** individualCount: 1; sex: male; **Location:** locationID: A2; continent: Europe; country: Spain; countryCode: ES; stateProvince: Catalonia; county: Lleida; locality: Sola de Boi; verbatimElevation: 1738.7; decimalLatitude: 42.54913; decimalLongitude: 0.87137; geodeticDatum: WGS84; **Event:** eventID: 1; samplingProtocol: Aerial; eventTime: Night
- **Type status:** Other material. **Occurrence:** individualCount: 1; sex: male; **Location:** locationID: A2; continent: Europe; country: Spain; countryCode: ES; stateProvince: Catalonia; county: Lleida; locality: Sola de Boi; verbatimElevation: 1738.7; decimalLatitude: 42.54913; decimalLongitude: 0.87137; geodeticDatum: WGS84; **Event:** eventID: 1; samplingProtocol: Aerial; eventTime: Night
- **Type status:** Other material. **Occurrence:** individualCount: 1; sex: male; **Location:** locationID: O1; continent: Europe; country: Spain; countryCode: ES; stateProvince: Aragón; county: Huesca; locality: O Furno; verbatimElevation: 1396.73; decimalLatitude: 42.60677; decimalLongitude: 0.13135; geodeticDatum: WGS84; **Event:** eventID: 1; samplingProtocol: Aerial; eventTime: Night
- **Type status:** Other material. **Occurrence:** individualCount: 1; sex: female; **Location:** locationID: O1; continent: Europe; country: Spain; countryCode: ES; stateProvince: Aragón; county: Huesca; locality: O Furno; verbatimElevation: 1396.73; decimalLatitude: 42.60677; decimalLongitude: 0.13135; geodeticDatum: WGS84; **Event:** eventID: 1; samplingProtocol: Aerial; eventTime: Night
- **Type status:** Other material. **Occurrence:** individualCount: 1; sex: female; **Location:** locationID: O1; continent: Europe; country: Spain; countryCode: ES; stateProvince: Aragón; county: Huesca; locality: O Furno; verbatimElevation: 1396.73; decimalLatitude: 42.60677; decimalLongitude: 0.13135; geodeticDatum: WGS84; **Event:** eventID: 1; samplingProtocol: Aerial; eventTime: Night
- **Type status:** Other material. **Occurrence:** individualCount: 1; sex: female; **Location:** locationID: O1; continent: Europe; country: Spain; countryCode: ES; stateProvince: Aragón; county: Huesca; locality: O Furno; verbatimElevation: 1396.73; decimalLatitude: 42.60677; decimalLongitude: 0.13135; geodeticDatum: WGS84; **Event:** eventID: 2; samplingProtocol: Aerial; eventTime: Night
- **Type status:** Other material. **Occurrence:** individualCount: 2; sex: female; **Location:** locationID: O1; continent: Europe; country: Spain; countryCode: ES; stateProvince: Aragón; county: Huesca; locality: O Furno; verbatimElevation: 1396.73; decimalLatitude: 42.60677; decimalLongitude: 0.13135; geodeticDatum: WGS84; **Event:** eventID: 2; samplingProtocol: Aerial; eventTime: Night
- **Type status:** Other material. **Occurrence:** individualCount: 1; sex: male; **Location:** locationID: O1; continent: Europe; country: Spain; countryCode: ES; stateProvince: Aragón; county: Huesca; locality: O Furno; verbatimElevation: 1396.73; decimalLatitude: 42.60677; decimalLongitude: 0.13135; geodeticDatum: WGS84; **Event:** eventID: 2; samplingProtocol: Aerial; eventTime: Night
- **Type status:** Other material. **Occurrence:** individualCount: 1; sex: male; **Location:** locationID: O1; continent: Europe; country: Spain; countryCode: ES; stateProvince: Aragón; county: Huesca; locality: O Furno; verbatimElevation: 1396.73; decimalLatitude: 42.60677; decimalLongitude: 0.13135; geodeticDatum: WGS84; **Event:** eventID: 1; samplingProtocol: Beating; eventTime: Night
- **Type status:** Other material. **Occurrence:** individualCount: 3; sex: female; **Location:** locationID: O1; continent: Europe; country: Spain; countryCode: ES; stateProvince: Aragón; county: Huesca; locality: O Furno; verbatimElevation: 1396.73; decimalLatitude: 42.60677; decimalLongitude: 0.13135; geodeticDatum: WGS84; **Event:** eventID: 1; samplingProtocol: Beating; eventTime: Night
- **Type status:** Other material. **Occurrence:** individualCount: 1; sex: male; **Location:** locationID: O1; continent: Europe; country: Spain; countryCode: ES; stateProvince: Aragón; county: Huesca; locality: O Furno; verbatimElevation: 1396.73; decimalLatitude: 42.60677; decimalLongitude: 0.13135; geodeticDatum: WGS84; **Event:** eventID: 1; samplingProtocol: Beating; eventTime: Night
- **Type status:** Other material. **Occurrence:** individualCount: 2; sex: female; **Location:** locationID: O1; continent: Europe; country: Spain; countryCode: ES; stateProvince: Aragón; county: Huesca; locality: O Furno; verbatimElevation: 1396.73; decimalLatitude: 42.60677; decimalLongitude: 0.13135; geodeticDatum: WGS84; **Event:** eventID: 1; samplingProtocol: Beating; eventTime: Night
- **Type status:** Other material. **Occurrence:** individualCount: 1; sex: male; **Location:** locationID: O1; continent: Europe; country: Spain; countryCode: ES; stateProvince: Aragón; county: Huesca; locality: O Furno; verbatimElevation: 1396.73; decimalLatitude: 42.60677; decimalLongitude: 0.13135; geodeticDatum: WGS84; **Event:** eventID: 2; samplingProtocol: Beating; eventTime: Day
- **Type status:** Other material. **Occurrence:** individualCount: 2; sex: female; **Location:** locationID: O1; continent: Europe; country: Spain; countryCode: ES; stateProvince: Aragón; county: Huesca; locality: O Furno; verbatimElevation: 1396.73; decimalLatitude: 42.60677; decimalLongitude: 0.13135; geodeticDatum: WGS84; **Event:** eventID: 2; samplingProtocol: Beating; eventTime: Day
- **Type status:** Other material. **Occurrence:** individualCount: 1; sex: female; **Location:** locationID: O1; continent: Europe; country: Spain; countryCode: ES; stateProvince: Aragón; county: Huesca; locality: O Furno; verbatimElevation: 1396.73; decimalLatitude: 42.60677; decimalLongitude: 0.13135; geodeticDatum: WGS84; **Event:** eventID: 1; samplingProtocol: Sweeping; eventTime: Night
- **Type status:** Other material. **Occurrence:** individualCount: 1; sex: female; **Location:** locationID: O1; continent: Europe; country: Spain; countryCode: ES; stateProvince: Aragón; county: Huesca; locality: O Furno; verbatimElevation: 1396.73; decimalLatitude: 42.60677; decimalLongitude: 0.13135; geodeticDatum: WGS84; **Event:** eventID: 1; samplingProtocol: Sweeping; eventTime: Day
- **Type status:** Other material. **Occurrence:** individualCount: 1; sex: female; **Location:** locationID: O2; continent: Europe; country: Spain; countryCode: ES; stateProvince: Aragón; county: Huesca; locality: Rebilla; verbatimElevation: 1158.13; decimalLatitude: 42.59427; decimalLongitude: 0.1529; geodeticDatum: WGS84; **Event:** eventID: 1; samplingProtocol: Beating; eventTime: Night
- **Type status:** Other material. **Occurrence:** individualCount: 1; sex: female; **Location:** locationID: P2; continent: Europe; country: Spain; countryCode: ES; stateProvince: Castilla y León; county: León; locality: Joyoguelas; verbatimElevation: 763.98; decimalLatitude: 43.17771; decimalLongitude: -4.90579; geodeticDatum: WGS84; **Event:** eventID: 1; samplingProtocol: Aerial; eventTime: Night
- **Type status:** Other material. **Occurrence:** individualCount: 1; sex: female; **Location:** locationID: P4; continent: Europe; country: Spain; countryCode: ES; stateProvince: Castilla y León; county: León; locality: El Canto; verbatimElevation: 943.48; decimalLatitude: 43.17227; decimalLongitude: -4.90857; geodeticDatum: WGS84; **Event:** eventID: 1; samplingProtocol: Aerial; eventTime: Night
- **Type status:** Other material. **Occurrence:** individualCount: 1; sex: female; **Location:** locationID: P4; continent: Europe; country: Spain; countryCode: ES; stateProvince: Castilla y León; county: León; locality: El Canto; verbatimElevation: 943.48; decimalLatitude: 43.17227; decimalLongitude: -4.90857; geodeticDatum: WGS84; **Event:** eventID: 2; samplingProtocol: Aerial; eventTime: Night
- **Type status:** Other material. **Occurrence:** individualCount: 1; sex: female; **Location:** locationID: P4; continent: Europe; country: Spain; countryCode: ES; stateProvince: Castilla y León; county: León; locality: El Canto; verbatimElevation: 943.48; decimalLatitude: 43.17227; decimalLongitude: -4.90857; geodeticDatum: WGS84; **Event:** eventID: 1; samplingProtocol: Beating; eventTime: Night

##### Distribution

Europe to Central Asia, Iran

#### Anyphaena sabina

L. Koch, 1866

##### Materials

- **Type status:** Other material. **Occurrence:** individualCount: 1; sex: male; **Location:** locationID: S2; continent: Europe; country: Spain; countryCode: ES; stateProvince: Andalucía; county: Granada; locality: Camarate; verbatimElevation: 1713.96; decimalLatitude: 37.18377; decimalLongitude: -3.26282; geodeticDatum: WGS84; **Event:** eventID: B; samplingProtocol: Pitfall

##### Distribution

Europe, Turkey, Russia, Georgia, Azerbaijan

#### Araneidae

Clerck, 1757

#### Araneus angulatus

Clerck, 1757

##### Materials

- **Type status:** Other material. **Occurrence:** individualCount: 1; sex: female; **Location:** locationID: C1; continent: Europe; country: Spain; countryCode: ES; stateProvince: Castilla-La Mancha; county: Ciudad Real; locality: Valle Brezoso; verbatimElevation: 756.56; decimalLatitude: 39.35663; decimalLongitude: -4.35912; geodeticDatum: WGS84; **Event:** eventID: 2; samplingProtocol: Aerial; eventTime: Night
- **Type status:** Other material. **Occurrence:** individualCount: 1; sex: female; **Location:** locationID: C1; continent: Europe; country: Spain; countryCode: ES; stateProvince: Castilla-La Mancha; county: Ciudad Real; locality: Valle Brezoso; verbatimElevation: 756.56; decimalLatitude: 39.35663; decimalLongitude: -4.35912; geodeticDatum: WGS84; **Event:** eventID: 2; samplingProtocol: Sweeping; eventTime: Night
- **Type status:** Other material. **Occurrence:** individualCount: 2; sex: female; **Location:** locationID: C2; continent: Europe; country: Spain; countryCode: ES; stateProvince: Castilla-La Mancha; county: Ciudad Real; locality: Valle Brezoso; verbatimElevation: 739.31; decimalLatitude: 39.35159; decimalLongitude: -4.3589; geodeticDatum: WGS84; **Event:** eventID: 2; samplingProtocol: Sweeping; eventTime: Night
- **Type status:** Other material. **Occurrence:** individualCount: 1; sex: female; **Location:** locationID: C3; continent: Europe; country: Spain; countryCode: ES; stateProvince: Castilla-La Mancha; county: Ciudad Real; locality: La Quesera; verbatimElevation: 767.55; decimalLatitude: 39.36177; decimalLongitude: -4.41733; geodeticDatum: WGS84; **Event:** eventID: 3; samplingProtocol: Aerial; eventTime: Night

##### Distribution

Palearctic

#### Araneus grossus

(C. L. Koch, 1844)

##### Materials

- **Type status:** Other material. **Occurrence:** individualCount: 1; sex: male; **Location:** locationID: C3; continent: Europe; country: Spain; countryCode: ES; stateProvince: Castilla-La Mancha; county: Ciudad Real; locality: La Quesera; verbatimElevation: 767.55; decimalLatitude: 39.36177; decimalLongitude: -4.41733; geodeticDatum: WGS84; **Event:** eventID: 2; samplingProtocol: Beating; eventTime: Night

##### Distribution

Europe to Central Asia

#### Araneus sturmi

(Hahn, 1831)

##### Materials

- **Type status:** Other material. **Occurrence:** individualCount: 4; sex: female; **Location:** locationID: O1; continent: Europe; country: Spain; countryCode: ES; stateProvince: Aragón; county: Huesca; locality: O Furno; verbatimElevation: 1396.73; decimalLatitude: 42.60677; decimalLongitude: 0.13135; geodeticDatum: WGS84; **Event:** eventID: 2; samplingProtocol: Aerial; eventTime: Night
- **Type status:** Other material. **Occurrence:** individualCount: 2; sex: female; **Location:** locationID: O1; continent: Europe; country: Spain; countryCode: ES; stateProvince: Aragón; county: Huesca; locality: O Furno; verbatimElevation: 1396.73; decimalLatitude: 42.60677; decimalLongitude: 0.13135; geodeticDatum: WGS84; **Event:** eventID: 1; samplingProtocol: Beating; eventTime: Day
- **Type status:** Other material. **Occurrence:** individualCount: 1; sex: female; **Location:** locationID: O1; continent: Europe; country: Spain; countryCode: ES; stateProvince: Aragón; county: Huesca; locality: O Furno; verbatimElevation: 1396.73; decimalLatitude: 42.60677; decimalLongitude: 0.13135; geodeticDatum: WGS84; **Event:** eventID: 2; samplingProtocol: Beating; eventTime: Day
- **Type status:** Other material. **Occurrence:** individualCount: 1; sex: male; **Location:** locationID: O1; continent: Europe; country: Spain; countryCode: ES; stateProvince: Aragón; county: Huesca; locality: O Furno; verbatimElevation: 1396.73; decimalLatitude: 42.60677; decimalLongitude: 0.13135; geodeticDatum: WGS84; **Event:** eventID: 1; samplingProtocol: Sweeping; eventTime: Night
- **Type status:** Other material. **Occurrence:** individualCount: 1; sex: female; **Location:** locationID: O1; continent: Europe; country: Spain; countryCode: ES; stateProvince: Aragón; county: Huesca; locality: O Furno; verbatimElevation: 1396.73; decimalLatitude: 42.60677; decimalLongitude: 0.13135; geodeticDatum: WGS84; **Event:** eventID: 1; samplingProtocol: Sweeping; eventTime: Night
- **Type status:** Other material. **Occurrence:** individualCount: 1; sex: female; **Location:** locationID: O1; continent: Europe; country: Spain; countryCode: ES; stateProvince: Aragón; county: Huesca; locality: O Furno; verbatimElevation: 1396.73; decimalLatitude: 42.60677; decimalLongitude: 0.13135; geodeticDatum: WGS84; **Event:** eventID: 2; samplingProtocol: Sweeping; eventTime: Day
- **Type status:** Other material. **Occurrence:** individualCount: 2; sex: female; **Location:** locationID: O2; continent: Europe; country: Spain; countryCode: ES; stateProvince: Aragón; county: Huesca; locality: Rebilla; verbatimElevation: 1158.13; decimalLatitude: 42.59427; decimalLongitude: 0.1529; geodeticDatum: WGS84; **Event:** eventID: 1; samplingProtocol: Beating; eventTime: Day
- **Type status:** Other material. **Occurrence:** individualCount: 2; sex: female; **Location:** locationID: O2; continent: Europe; country: Spain; countryCode: ES; stateProvince: Aragón; county: Huesca; locality: Rebilla; verbatimElevation: 1158.13; decimalLatitude: 42.59427; decimalLongitude: 0.1529; geodeticDatum: WGS84; **Event:** eventID: 1; samplingProtocol: Beating; eventTime: Night
- **Type status:** Other material. **Occurrence:** individualCount: 3; sex: female; **Location:** locationID: O2; continent: Europe; country: Spain; countryCode: ES; stateProvince: Aragón; county: Huesca; locality: Rebilla; verbatimElevation: 1158.13; decimalLatitude: 42.59427; decimalLongitude: 0.1529; geodeticDatum: WGS84; **Event:** eventID: 1; samplingProtocol: Beating; eventTime: Night
- **Type status:** Other material. **Occurrence:** individualCount: 1; sex: male; **Location:** locationID: O2; continent: Europe; country: Spain; countryCode: ES; stateProvince: Aragón; county: Huesca; locality: Rebilla; verbatimElevation: 1158.13; decimalLatitude: 42.59427; decimalLongitude: 0.1529; geodeticDatum: WGS84; **Event:** eventID: 2; samplingProtocol: Beating; eventTime: Day
- **Type status:** Other material. **Occurrence:** individualCount: 2; sex: female; **Location:** locationID: O2; continent: Europe; country: Spain; countryCode: ES; stateProvince: Aragón; county: Huesca; locality: Rebilla; verbatimElevation: 1158.13; decimalLatitude: 42.59427; decimalLongitude: 0.1529; geodeticDatum: WGS84; **Event:** eventID: 2; samplingProtocol: Beating; eventTime: Day

##### Distribution

Palearctic

#### Araneus triguttatus

(Fabricius, 1793)

##### Materials

- **Type status:** Other material. **Occurrence:** individualCount: 1; sex: female; **Location:** locationID: O1; continent: Europe; country: Spain; countryCode: ES; stateProvince: Aragón; county: Huesca; locality: O Furno; verbatimElevation: 1396.73; decimalLatitude: 42.60677; decimalLongitude: 0.13135; geodeticDatum: WGS84; **Event:** eventID: 2; samplingProtocol: Beating; eventTime: Day

##### Distribution

Europe

#### Araniella alpica

(L. Koch, 1869)

##### Materials

- **Type status:** Other material. **Occurrence:** individualCount: 1; sex: female; **Location:** locationID: A1; continent: Europe; country: Spain; countryCode: ES; stateProvince: Catalonia; county: Lleida; locality: Sola de Boi; verbatimElevation: 1759.8; decimalLatitude: 42.54958; decimalLongitude: 0.87254; geodeticDatum: WGS84; **Event:** eventID: 1; samplingProtocol: Beating; eventTime: Night
- **Type status:** Other material. **Occurrence:** individualCount: 1; sex: male; **Location:** locationID: A1; continent: Europe; country: Spain; countryCode: ES; stateProvince: Catalonia; county: Lleida; locality: Sola de Boi; verbatimElevation: 1759.8; decimalLatitude: 42.54958; decimalLongitude: 0.87254; geodeticDatum: WGS84; **Event:** eventID: 1; samplingProtocol: Sweeping; eventTime: Night
- **Type status:** Other material. **Occurrence:** individualCount: 1; sex: male; **Location:** locationID: A2; continent: Europe; country: Spain; countryCode: ES; stateProvince: Catalonia; county: Lleida; locality: Sola de Boi; verbatimElevation: 1738.7; decimalLatitude: 42.54913; decimalLongitude: 0.87137; geodeticDatum: WGS84; **Event:** eventID: 1; samplingProtocol: Sweeping; eventTime: Night
- **Type status:** Other material. **Occurrence:** individualCount: 1; sex: female; **Location:** locationID: P1; continent: Europe; country: Spain; countryCode: ES; stateProvince: Castilla y León; county: León; locality: Monte Robledo; verbatimElevation: 1071.58; decimalLatitude: 43.1445; decimalLongitude: -4.92675; geodeticDatum: WGS84; **Event:** eventID: 1; samplingProtocol: Sweeping; eventTime: Day
- **Type status:** Other material. **Occurrence:** individualCount: 1; sex: female; **Location:** locationID: A1; continent: Europe; country: Spain; countryCode: ES; stateProvince: Catalonia; county: Lleida; locality: Sola de Boi; verbatimElevation: 1759.8; decimalLatitude: 42.54958; decimalLongitude: 0.87254; geodeticDatum: WGS84; **Event:** eventID: 1; samplingProtocol: Aerial; eventTime: Night

##### Distribution

Europe to Azerbaijan

#### Araniella cucurbitina

(Clerck, 1757)

##### Materials

- **Type status:** Other material. **Occurrence:** individualCount: 1; sex: male; **Location:** locationID: A2; continent: Europe; country: Spain; countryCode: ES; stateProvince: Catalonia; county: Lleida; locality: Sola de Boi; verbatimElevation: 1738.7; decimalLatitude: 42.54913; decimalLongitude: 0.87137; geodeticDatum: WGS84; **Event:** eventID: 1; samplingProtocol: Aerial; eventTime: Night
- **Type status:** Other material. **Occurrence:** individualCount: 1; sex: male; **Location:** locationID: A2; continent: Europe; country: Spain; countryCode: ES; stateProvince: Catalonia; county: Lleida; locality: Sola de Boi; verbatimElevation: 1738.7; decimalLatitude: 42.54913; decimalLongitude: 0.87137; geodeticDatum: WGS84; **Event:** eventID: 2; samplingProtocol: Beating; eventTime: Day
- **Type status:** Other material. **Occurrence:** individualCount: 2; sex: female; **Location:** locationID: A2; continent: Europe; country: Spain; countryCode: ES; stateProvince: Catalonia; county: Lleida; locality: Sola de Boi; verbatimElevation: 1738.7; decimalLatitude: 42.54913; decimalLongitude: 0.87137; geodeticDatum: WGS84; **Event:** eventID: 2; samplingProtocol: Beating; eventTime: Day
- **Type status:** Other material. **Occurrence:** individualCount: 1; sex: male; **Location:** locationID: A2; continent: Europe; country: Spain; countryCode: ES; stateProvince: Catalonia; county: Lleida; locality: Sola de Boi; verbatimElevation: 1738.7; decimalLatitude: 42.54913; decimalLongitude: 0.87137; geodeticDatum: WGS84; **Event:** eventID: 2; samplingProtocol: Sweeping; eventTime: Day
- **Type status:** Other material. **Occurrence:** individualCount: 2; sex: female; **Location:** locationID: A2; continent: Europe; country: Spain; countryCode: ES; stateProvince: Catalonia; county: Lleida; locality: Sola de Boi; verbatimElevation: 1738.7; decimalLatitude: 42.54913; decimalLongitude: 0.87137; geodeticDatum: WGS84; **Event:** eventID: 2; samplingProtocol: Sweeping; eventTime: Day
- **Type status:** Other material. **Occurrence:** individualCount: 1; sex: male; **Location:** locationID: C3; continent: Europe; country: Spain; countryCode: ES; stateProvince: Castilla-La Mancha; county: Ciudad Real; locality: La Quesera; verbatimElevation: 767.55; decimalLatitude: 39.36177; decimalLongitude: -4.41733; geodeticDatum: WGS84; **Event:** eventID: 1; samplingProtocol: Aerial; eventTime: Night
- **Type status:** Other material. **Occurrence:** individualCount: 1; sex: female; **Location:** locationID: C3; continent: Europe; country: Spain; countryCode: ES; stateProvince: Castilla-La Mancha; county: Ciudad Real; locality: La Quesera; verbatimElevation: 767.55; decimalLatitude: 39.36177; decimalLongitude: -4.41733; geodeticDatum: WGS84; **Event:** eventID: 2; samplingProtocol: Aerial; eventTime: Night
- **Type status:** Other material. **Occurrence:** individualCount: 2; sex: female; **Location:** locationID: C4; continent: Europe; country: Spain; countryCode: ES; stateProvince: Castilla-La Mancha; county: Ciudad Real; locality: La Quesera; verbatimElevation: 772.3; decimalLatitude: 39.36337; decimalLongitude: -4.41704; geodeticDatum: WGS84; **Event:** eventID: 1; samplingProtocol: Aerial; eventTime: Night
- **Type status:** Other material. **Occurrence:** individualCount: 1; sex: female; **Location:** locationID: C4; continent: Europe; country: Spain; countryCode: ES; stateProvince: Castilla-La Mancha; county: Ciudad Real; locality: La Quesera; verbatimElevation: 772.3; decimalLatitude: 39.36337; decimalLongitude: -4.41704; geodeticDatum: WGS84; **Event:** eventID: 2; samplingProtocol: Aerial; eventTime: Night
- **Type status:** Other material. **Occurrence:** individualCount: 1; sex: female; **Location:** locationID: C4; continent: Europe; country: Spain; countryCode: ES; stateProvince: Castilla-La Mancha; county: Ciudad Real; locality: La Quesera; verbatimElevation: 772.3; decimalLatitude: 39.36337; decimalLongitude: -4.41704; geodeticDatum: WGS84; **Event:** eventID: 3; samplingProtocol: Aerial; eventTime: Night
- **Type status:** Other material. **Occurrence:** individualCount: 1; sex: female; **Location:** locationID: C4; continent: Europe; country: Spain; countryCode: ES; stateProvince: Castilla-La Mancha; county: Ciudad Real; locality: La Quesera; verbatimElevation: 772.3; decimalLatitude: 39.36337; decimalLongitude: -4.41704; geodeticDatum: WGS84; **Event:** eventID: 1; samplingProtocol: Beating; eventTime: Day
- **Type status:** Other material. **Occurrence:** individualCount: 1; sex: female; **Location:** locationID: C4; continent: Europe; country: Spain; countryCode: ES; stateProvince: Castilla-La Mancha; county: Ciudad Real; locality: La Quesera; verbatimElevation: 772.3; decimalLatitude: 39.36337; decimalLongitude: -4.41704; geodeticDatum: WGS84; **Event:** eventID: 2; samplingProtocol: Beating; eventTime: Day
- **Type status:** Other material. **Occurrence:** individualCount: 1; sex: female; **Location:** locationID: C4; continent: Europe; country: Spain; countryCode: ES; stateProvince: Castilla-La Mancha; county: Ciudad Real; locality: La Quesera; verbatimElevation: 772.3; decimalLatitude: 39.36337; decimalLongitude: -4.41704; geodeticDatum: WGS84; **Event:** eventID: 1; samplingProtocol: Sweeping; eventTime: Day
- **Type status:** Other material. **Occurrence:** individualCount: 1; sex: female; **Location:** locationID: C4; continent: Europe; country: Spain; countryCode: ES; stateProvince: Castilla-La Mancha; county: Ciudad Real; locality: La Quesera; verbatimElevation: 772.3; decimalLatitude: 39.36337; decimalLongitude: -4.41704; geodeticDatum: WGS84; **Event:** eventID: 1; samplingProtocol: Sweeping; eventTime: Night
- **Type status:** Other material. **Occurrence:** individualCount: 1; sex: female; **Location:** locationID: O1; continent: Europe; country: Spain; countryCode: ES; stateProvince: Aragón; county: Huesca; locality: O Furno; verbatimElevation: 1396.73; decimalLatitude: 42.60677; decimalLongitude: 0.13135; geodeticDatum: WGS84; **Event:** eventID: 2; samplingProtocol: Aerial; eventTime: Night
- **Type status:** Other material. **Occurrence:** individualCount: 3; sex: male; **Location:** locationID: O2; continent: Europe; country: Spain; countryCode: ES; stateProvince: Aragón; county: Huesca; locality: Rebilla; verbatimElevation: 1158.13; decimalLatitude: 42.59427; decimalLongitude: 0.1529; geodeticDatum: WGS84; **Event:** eventID: 1; samplingProtocol: Aerial; eventTime: Night
- **Type status:** Other material. **Occurrence:** individualCount: 1; sex: male; **Location:** locationID: O2; continent: Europe; country: Spain; countryCode: ES; stateProvince: Aragón; county: Huesca; locality: Rebilla; verbatimElevation: 1158.13; decimalLatitude: 42.59427; decimalLongitude: 0.1529; geodeticDatum: WGS84; **Event:** eventID: 2; samplingProtocol: Aerial; eventTime: Night
- **Type status:** Other material. **Occurrence:** individualCount: 1; sex: male; **Location:** locationID: P4; continent: Europe; country: Spain; countryCode: ES; stateProvince: Castilla y León; county: León; locality: El Canto; verbatimElevation: 943.48; decimalLatitude: 43.17227; decimalLongitude: -4.90857; geodeticDatum: WGS84; **Event:** eventID: 1; samplingProtocol: Beating; eventTime: Night
- **Type status:** Other material. **Occurrence:** individualCount: 1; sex: female; **Location:** locationID: P4; continent: Europe; country: Spain; countryCode: ES; stateProvince: Castilla y León; county: León; locality: El Canto; verbatimElevation: 943.48; decimalLatitude: 43.17227; decimalLongitude: -4.90857; geodeticDatum: WGS84; **Event:** eventID: 2; samplingProtocol: Sweeping; eventTime: Day
- **Type status:** Other material. **Occurrence:** individualCount: 2; sex: male; **Location:** locationID: S1; continent: Europe; country: Spain; countryCode: ES; stateProvince: Andalucía; county: Granada; locality: Soportujar; verbatimElevation: 1786.57; decimalLatitude: 36.96151; decimalLongitude: -3.41881; geodeticDatum: WGS84; **Event:** eventID: 2; samplingProtocol: Aerial; eventTime: Night
- **Type status:** Other material. **Occurrence:** individualCount: 1; sex: male; **Location:** locationID: S1; continent: Europe; country: Spain; countryCode: ES; stateProvince: Andalucía; county: Granada; locality: Soportujar; verbatimElevation: 1786.57; decimalLatitude: 36.96151; decimalLongitude: -3.41881; geodeticDatum: WGS84; **Event:** eventID: 2; samplingProtocol: Aerial; eventTime: Night
- **Type status:** Other material. **Occurrence:** individualCount: 2; sex: male; **Location:** locationID: S1; continent: Europe; country: Spain; countryCode: ES; stateProvince: Andalucía; county: Granada; locality: Soportujar; verbatimElevation: 1786.57; decimalLatitude: 36.96151; decimalLongitude: -3.41881; geodeticDatum: WGS84; **Event:** eventID: 1; samplingProtocol: Aerial; eventTime: Night
- **Type status:** Other material. **Occurrence:** individualCount: 2; sex: male; **Location:** locationID: S1; continent: Europe; country: Spain; countryCode: ES; stateProvince: Andalucía; county: Granada; locality: Soportujar; verbatimElevation: 1786.57; decimalLatitude: 36.96151; decimalLongitude: -3.41881; geodeticDatum: WGS84; **Event:** eventID: 3; samplingProtocol: Aerial; eventTime: Night
- **Type status:** Other material. **Occurrence:** individualCount: 1; sex: male; **Location:** locationID: S1; continent: Europe; country: Spain; countryCode: ES; stateProvince: Andalucía; county: Granada; locality: Soportujar; verbatimElevation: 1786.57; decimalLatitude: 36.96151; decimalLongitude: -3.41881; geodeticDatum: WGS84; **Event:** eventID: 2; samplingProtocol: Beating; eventTime: Night
- **Type status:** Other material. **Occurrence:** individualCount: 1; sex: female; **Location:** locationID: S1; continent: Europe; country: Spain; countryCode: ES; stateProvince: Andalucía; county: Granada; locality: Soportujar; verbatimElevation: 1786.57; decimalLatitude: 36.96151; decimalLongitude: -3.41881; geodeticDatum: WGS84; **Event:** eventID: 2; samplingProtocol: Beating; eventTime: Night
- **Type status:** Other material. **Occurrence:** individualCount: 1; sex: male; **Location:** locationID: S1; continent: Europe; country: Spain; countryCode: ES; stateProvince: Andalucía; county: Granada; locality: Soportujar; verbatimElevation: 1786.57; decimalLatitude: 36.96151; decimalLongitude: -3.41881; geodeticDatum: WGS84; **Event:** eventID: 2; samplingProtocol: Beating; eventTime: Day
- **Type status:** Other material. **Occurrence:** individualCount: 1; sex: female; **Location:** locationID: S1; continent: Europe; country: Spain; countryCode: ES; stateProvince: Andalucía; county: Granada; locality: Soportujar; verbatimElevation: 1786.57; decimalLatitude: 36.96151; decimalLongitude: -3.41881; geodeticDatum: WGS84; **Event:** eventID: 2; samplingProtocol: Sweeping; eventTime: Night
- **Type status:** Other material. **Occurrence:** individualCount: 1; sex: male; **Location:** locationID: S2; continent: Europe; country: Spain; countryCode: ES; stateProvince: Andalucía; county: Granada; locality: Camarate; verbatimElevation: 1713.96; decimalLatitude: 37.18377; decimalLongitude: -3.26282; geodeticDatum: WGS84; **Event:** eventID: 1; samplingProtocol: Aerial; eventTime: Night
- **Type status:** Other material. **Occurrence:** individualCount: 3; sex: female; **Location:** locationID: S2; continent: Europe; country: Spain; countryCode: ES; stateProvince: Andalucía; county: Granada; locality: Camarate; verbatimElevation: 1713.96; decimalLatitude: 37.18377; decimalLongitude: -3.26282; geodeticDatum: WGS84; **Event:** eventID: 1; samplingProtocol: Aerial; eventTime: Night
- **Type status:** Other material. **Occurrence:** individualCount: 1; sex: male; **Location:** locationID: S2; continent: Europe; country: Spain; countryCode: ES; stateProvince: Andalucía; county: Granada; locality: Camarate; verbatimElevation: 1713.96; decimalLatitude: 37.18377; decimalLongitude: -3.26282; geodeticDatum: WGS84; **Event:** eventID: 2; samplingProtocol: Aerial; eventTime: Night
- **Type status:** Other material. **Occurrence:** individualCount: 2; sex: female; **Location:** locationID: S2; continent: Europe; country: Spain; countryCode: ES; stateProvince: Andalucía; county: Granada; locality: Camarate; verbatimElevation: 1713.96; decimalLatitude: 37.18377; decimalLongitude: -3.26282; geodeticDatum: WGS84; **Event:** eventID: 2; samplingProtocol: Aerial; eventTime: Night
- **Type status:** Other material. **Occurrence:** individualCount: 3; sex: male; **Location:** locationID: S2; continent: Europe; country: Spain; countryCode: ES; stateProvince: Andalucía; county: Granada; locality: Camarate; verbatimElevation: 1713.96; decimalLatitude: 37.18377; decimalLongitude: -3.26282; geodeticDatum: WGS84; **Event:** eventID: 3; samplingProtocol: Aerial; eventTime: Night
- **Type status:** Other material. **Occurrence:** individualCount: 6; sex: female; **Location:** locationID: S2; continent: Europe; country: Spain; countryCode: ES; stateProvince: Andalucía; county: Granada; locality: Camarate; verbatimElevation: 1713.96; decimalLatitude: 37.18377; decimalLongitude: -3.26282; geodeticDatum: WGS84; **Event:** eventID: 3; samplingProtocol: Aerial; eventTime: Night
- **Type status:** Other material. **Occurrence:** individualCount: 2; sex: female; **Location:** locationID: S2; continent: Europe; country: Spain; countryCode: ES; stateProvince: Andalucía; county: Granada; locality: Camarate; verbatimElevation: 1713.96; decimalLatitude: 37.18377; decimalLongitude: -3.26282; geodeticDatum: WGS84; **Event:** eventID: 4; samplingProtocol: Aerial; eventTime: Night
- **Type status:** Other material. **Occurrence:** individualCount: 1; sex: female; **Location:** locationID: S2; continent: Europe; country: Spain; countryCode: ES; stateProvince: Andalucía; county: Granada; locality: Camarate; verbatimElevation: 1713.96; decimalLatitude: 37.18377; decimalLongitude: -3.26282; geodeticDatum: WGS84; **Event:** eventID: 1; samplingProtocol: Beating; eventTime: Day
- **Type status:** Other material. **Occurrence:** individualCount: 1; sex: male; **Location:** locationID: S2; continent: Europe; country: Spain; countryCode: ES; stateProvince: Andalucía; county: Granada; locality: Camarate; verbatimElevation: 1713.96; decimalLatitude: 37.18377; decimalLongitude: -3.26282; geodeticDatum: WGS84; **Event:** eventID: 1; samplingProtocol: Beating; eventTime: Night
- **Type status:** Other material. **Occurrence:** individualCount: 4; sex: female; **Location:** locationID: S2; continent: Europe; country: Spain; countryCode: ES; stateProvince: Andalucía; county: Granada; locality: Camarate; verbatimElevation: 1713.96; decimalLatitude: 37.18377; decimalLongitude: -3.26282; geodeticDatum: WGS84; **Event:** eventID: 1; samplingProtocol: Beating; eventTime: Night
- **Type status:** Other material. **Occurrence:** individualCount: 2; sex: male; **Location:** locationID: S2; continent: Europe; country: Spain; countryCode: ES; stateProvince: Andalucía; county: Granada; locality: Camarate; verbatimElevation: 1713.96; decimalLatitude: 37.18377; decimalLongitude: -3.26282; geodeticDatum: WGS84; **Event:** eventID: 2; samplingProtocol: Beating; eventTime: Night
- **Type status:** Other material. **Occurrence:** individualCount: 2; sex: female; **Location:** locationID: S2; continent: Europe; country: Spain; countryCode: ES; stateProvince: Andalucía; county: Granada; locality: Camarate; verbatimElevation: 1713.96; decimalLatitude: 37.18377; decimalLongitude: -3.26282; geodeticDatum: WGS84; **Event:** eventID: 2; samplingProtocol: Beating; eventTime: Night
- **Type status:** Other material. **Occurrence:** individualCount: 5; sex: female; **Location:** locationID: S2; continent: Europe; country: Spain; countryCode: ES; stateProvince: Andalucía; county: Granada; locality: Camarate; verbatimElevation: 1713.96; decimalLatitude: 37.18377; decimalLongitude: -3.26282; geodeticDatum: WGS84; **Event:** eventID: 2; samplingProtocol: Beating; eventTime: Day
- **Type status:** Other material. **Occurrence:** individualCount: 1; sex: male; **Location:** locationID: S2; continent: Europe; country: Spain; countryCode: ES; stateProvince: Andalucía; county: Granada; locality: Camarate; verbatimElevation: 1713.96; decimalLatitude: 37.18377; decimalLongitude: -3.26282; geodeticDatum: WGS84; **Event:** eventID: 1; samplingProtocol: Sweeping; eventTime: Day
- **Type status:** Other material. **Occurrence:** individualCount: 4; sex: male; **Location:** locationID: S2; continent: Europe; country: Spain; countryCode: ES; stateProvince: Andalucía; county: Granada; locality: Camarate; verbatimElevation: 1713.96; decimalLatitude: 37.18377; decimalLongitude: -3.26282; geodeticDatum: WGS84; **Event:** eventID: 2; samplingProtocol: Sweeping; eventTime: Night
- **Type status:** Other material. **Occurrence:** individualCount: 4; sex: female; **Location:** locationID: S2; continent: Europe; country: Spain; countryCode: ES; stateProvince: Andalucía; county: Granada; locality: Camarate; verbatimElevation: 1713.96; decimalLatitude: 37.18377; decimalLongitude: -3.26282; geodeticDatum: WGS84; **Event:** eventID: 2; samplingProtocol: Sweeping; eventTime: Night
- **Type status:** Other material. **Occurrence:** individualCount: 2; sex: male; **Location:** locationID: S2; continent: Europe; country: Spain; countryCode: ES; stateProvince: Andalucía; county: Granada; locality: Camarate; verbatimElevation: 1713.96; decimalLatitude: 37.18377; decimalLongitude: -3.26282; geodeticDatum: WGS84; **Event:** eventID: 2; samplingProtocol: Sweeping; eventTime: Day
- **Type status:** Other material. **Occurrence:** individualCount: 1; sex: female; **Location:** locationID: S2; continent: Europe; country: Spain; countryCode: ES; stateProvince: Andalucía; county: Granada; locality: Camarate; verbatimElevation: 1713.96; decimalLatitude: 37.18377; decimalLongitude: -3.26282; geodeticDatum: WGS84; **Event:** eventID: 2; samplingProtocol: Sweeping; eventTime: Day
- **Type status:** Other material. **Occurrence:** individualCount: 1; sex: male; **Location:** locationID: S2; continent: Europe; country: Spain; countryCode: ES; stateProvince: Andalucía; county: Granada; locality: Camarate; verbatimElevation: 1713.96; decimalLatitude: 37.18377; decimalLongitude: -3.26282; geodeticDatum: WGS84; **Event:** eventID: 1; samplingProtocol: Beating; eventTime: Day

##### Distribution

Palearctic

#### Araniella opisthographa

(Kulczynski, 1905)

##### Materials

- **Type status:** Other material. **Occurrence:** individualCount: 1; sex: female; **Location:** locationID: O1; continent: Europe; country: Spain; countryCode: ES; stateProvince: Aragón; county: Huesca; locality: O Furno; verbatimElevation: 1396.73; decimalLatitude: 42.60677; decimalLongitude: 0.13135; geodeticDatum: WGS84; **Event:** eventID: 2; samplingProtocol: Beating; eventTime: Day
- **Type status:** Other material. **Occurrence:** individualCount: 1; sex: male; **Location:** locationID: O1; continent: Europe; country: Spain; countryCode: ES; stateProvince: Aragón; county: Huesca; locality: O Furno; verbatimElevation: 1396.73; decimalLatitude: 42.60677; decimalLongitude: 0.13135; geodeticDatum: WGS84; **Event:** eventID: 2; samplingProtocol: Sweeping; eventTime: Day
- **Type status:** Other material. **Occurrence:** individualCount: 1; sex: male; **Location:** locationID: O2; continent: Europe; country: Spain; countryCode: ES; stateProvince: Aragón; county: Huesca; locality: Rebilla; verbatimElevation: 1158.13; decimalLatitude: 42.59427; decimalLongitude: 0.1529; geodeticDatum: WGS84; **Event:** eventID: 1; samplingProtocol: Beating; eventTime: Night
- **Type status:** Other material. **Occurrence:** individualCount: 1; sex: female; **Location:** locationID: P1; continent: Europe; country: Spain; countryCode: ES; stateProvince: Castilla y León; county: León; locality: Monte Robledo; verbatimElevation: 1071.58; decimalLatitude: 43.1445; decimalLongitude: -4.92675; geodeticDatum: WGS84; **Event:** eventID: 1; samplingProtocol: Beating; eventTime: Night
- **Type status:** Other material. **Occurrence:** individualCount: 1; sex: female; **Location:** locationID: P1; continent: Europe; country: Spain; countryCode: ES; stateProvince: Castilla y León; county: León; locality: Monte Robledo; verbatimElevation: 1071.58; decimalLatitude: 43.1445; decimalLongitude: -4.92675; geodeticDatum: WGS84; **Event:** eventID: 2; samplingProtocol: Beating; eventTime: Day
- **Type status:** Other material. **Occurrence:** individualCount: 1; sex: female; **Location:** locationID: P1; continent: Europe; country: Spain; countryCode: ES; stateProvince: Castilla y León; county: León; locality: Monte Robledo; verbatimElevation: 1071.58; decimalLatitude: 43.1445; decimalLongitude: -4.92675; geodeticDatum: WGS84; **Event:** eventID: 1; samplingProtocol: Sweeping; eventTime: Night
- **Type status:** Other material. **Occurrence:** individualCount: 1; sex: female; **Location:** locationID: P3; continent: Europe; country: Spain; countryCode: ES; stateProvince: Castilla y León; county: León; locality: Las Arroyas; verbatimElevation: 1097.1; decimalLatitude: 43.14351; decimalLongitude: -4.94878; geodeticDatum: WGS84; **Event:** eventID: 1; samplingProtocol: Aerial; eventTime: Night
- **Type status:** Other material. **Occurrence:** individualCount: 1; sex: female; **Location:** locationID: P3; continent: Europe; country: Spain; countryCode: ES; stateProvince: Castilla y León; county: León; locality: Las Arroyas; verbatimElevation: 1097.1; decimalLatitude: 43.14351; decimalLongitude: -4.94878; geodeticDatum: WGS84; **Event:** eventID: 1; samplingProtocol: Beating; eventTime: Day
- **Type status:** Other material. **Occurrence:** individualCount: 1; sex: female; **Location:** locationID: P4; continent: Europe; country: Spain; countryCode: ES; stateProvince: Castilla y León; county: León; locality: El Canto; verbatimElevation: 943.48; decimalLatitude: 43.17227; decimalLongitude: -4.90857; geodeticDatum: WGS84; **Event:** eventID: 2; samplingProtocol: Aerial; eventTime: Night
- **Type status:** Other material. **Occurrence:** individualCount: 1; sex: female; **Location:** locationID: P4; continent: Europe; country: Spain; countryCode: ES; stateProvince: Castilla y León; county: León; locality: El Canto; verbatimElevation: 943.48; decimalLatitude: 43.17227; decimalLongitude: -4.90857; geodeticDatum: WGS84; **Event:** eventID: 1; samplingProtocol: Beating; eventTime: Night
- **Type status:** Other material. **Occurrence:** individualCount: 1; sex: female; **Location:** locationID: P4; continent: Europe; country: Spain; countryCode: ES; stateProvince: Castilla y León; county: León; locality: El Canto; verbatimElevation: 943.48; decimalLatitude: 43.17227; decimalLongitude: -4.90857; geodeticDatum: WGS84; **Event:** eventID: 1; samplingProtocol: Sweeping; eventTime: Day
- **Type status:** Other material. **Occurrence:** individualCount: 2; sex: female; **Location:** locationID: P4; continent: Europe; country: Spain; countryCode: ES; stateProvince: Castilla y León; county: León; locality: El Canto; verbatimElevation: 943.48; decimalLatitude: 43.17227; decimalLongitude: -4.90857; geodeticDatum: WGS84; **Event:** eventID: 1; samplingProtocol: Sweeping; eventTime: Night
- **Type status:** Other material. **Occurrence:** individualCount: 1; sex: female; **Location:** locationID: S1; continent: Europe; country: Spain; countryCode: ES; stateProvince: Andalucía; county: Granada; locality: Soportujar; verbatimElevation: 1786.57; decimalLatitude: 36.96151; decimalLongitude: -3.41881; geodeticDatum: WGS84; **Event:** eventID: 1; samplingProtocol: Aerial; eventTime: Night
- **Type status:** Other material. **Occurrence:** individualCount: 1; sex: male; **Location:** locationID: S1; continent: Europe; country: Spain; countryCode: ES; stateProvince: Andalucía; county: Granada; locality: Soportujar; verbatimElevation: 1786.57; decimalLatitude: 36.96151; decimalLongitude: -3.41881; geodeticDatum: WGS84; **Event:** eventID: 2; samplingProtocol: Aerial; eventTime: Night
- **Type status:** Other material. **Occurrence:** individualCount: 2; sex: female; **Location:** locationID: S1; continent: Europe; country: Spain; countryCode: ES; stateProvince: Andalucía; county: Granada; locality: Soportujar; verbatimElevation: 1786.57; decimalLatitude: 36.96151; decimalLongitude: -3.41881; geodeticDatum: WGS84; **Event:** eventID: 2; samplingProtocol: Aerial; eventTime: Night
- **Type status:** Other material. **Occurrence:** individualCount: 1; sex: male; **Location:** locationID: S1; continent: Europe; country: Spain; countryCode: ES; stateProvince: Andalucía; county: Granada; locality: Soportujar; verbatimElevation: 1786.57; decimalLatitude: 36.96151; decimalLongitude: -3.41881; geodeticDatum: WGS84; **Event:** eventID: 3; samplingProtocol: Aerial; eventTime: Night
- **Type status:** Other material. **Occurrence:** individualCount: 3; sex: female; **Location:** locationID: S1; continent: Europe; country: Spain; countryCode: ES; stateProvince: Andalucía; county: Granada; locality: Soportujar; verbatimElevation: 1786.57; decimalLatitude: 36.96151; decimalLongitude: -3.41881; geodeticDatum: WGS84; **Event:** eventID: 3; samplingProtocol: Aerial; eventTime: Night
- **Type status:** Other material. **Occurrence:** individualCount: 1; sex: female; **Location:** locationID: S1; continent: Europe; country: Spain; countryCode: ES; stateProvince: Andalucía; county: Granada; locality: Soportujar; verbatimElevation: 1786.57; decimalLatitude: 36.96151; decimalLongitude: -3.41881; geodeticDatum: WGS84; **Event:** eventID: 1; samplingProtocol: Beating; eventTime: Day
- **Type status:** Other material. **Occurrence:** individualCount: 1; sex: male; **Location:** locationID: S1; continent: Europe; country: Spain; countryCode: ES; stateProvince: Andalucía; county: Granada; locality: Soportujar; verbatimElevation: 1786.57; decimalLatitude: 36.96151; decimalLongitude: -3.41881; geodeticDatum: WGS84; **Event:** eventID: 2; samplingProtocol: Beating; eventTime: Day
- **Type status:** Other material. **Occurrence:** individualCount: 1; sex: female; **Location:** locationID: S1; continent: Europe; country: Spain; countryCode: ES; stateProvince: Andalucía; county: Granada; locality: Soportujar; verbatimElevation: 1786.57; decimalLatitude: 36.96151; decimalLongitude: -3.41881; geodeticDatum: WGS84; **Event:** eventID: 1; samplingProtocol: Sweeping; eventTime: Day
- **Type status:** Other material. **Occurrence:** individualCount: 1; sex: female; **Location:** locationID: S1; continent: Europe; country: Spain; countryCode: ES; stateProvince: Andalucía; county: Granada; locality: Soportujar; verbatimElevation: 1786.57; decimalLatitude: 36.96151; decimalLongitude: -3.41881; geodeticDatum: WGS84; **Event:** eventID: 1; samplingProtocol: Sweeping; eventTime: Night
- **Type status:** Other material. **Occurrence:** individualCount: 1; sex: male; **Location:** locationID: S1; continent: Europe; country: Spain; countryCode: ES; stateProvince: Andalucía; county: Granada; locality: Soportujar; verbatimElevation: 1786.57; decimalLatitude: 36.96151; decimalLongitude: -3.41881; geodeticDatum: WGS84; **Event:** eventID: 2; samplingProtocol: Sweeping; eventTime: Night
- **Type status:** Other material. **Occurrence:** individualCount: 1; sex: male; **Location:** locationID: S2; continent: Europe; country: Spain; countryCode: ES; stateProvince: Andalucía; county: Granada; locality: Camarate; verbatimElevation: 1713.96; decimalLatitude: 37.18377; decimalLongitude: -3.26282; geodeticDatum: WGS84; **Event:** eventID: 1; samplingProtocol: Aerial; eventTime: Night
- **Type status:** Other material. **Occurrence:** individualCount: 1; sex: male; **Location:** locationID: S2; continent: Europe; country: Spain; countryCode: ES; stateProvince: Andalucía; county: Granada; locality: Camarate; verbatimElevation: 1713.96; decimalLatitude: 37.18377; decimalLongitude: -3.26282; geodeticDatum: WGS84; **Event:** eventID: 2; samplingProtocol: Aerial; eventTime: Night
- **Type status:** Other material. **Occurrence:** individualCount: 2; sex: female; **Location:** locationID: S2; continent: Europe; country: Spain; countryCode: ES; stateProvince: Andalucía; county: Granada; locality: Camarate; verbatimElevation: 1713.96; decimalLatitude: 37.18377; decimalLongitude: -3.26282; geodeticDatum: WGS84; **Event:** eventID: 2; samplingProtocol: Aerial; eventTime: Night
- **Type status:** Other material. **Occurrence:** individualCount: 2; sex: female; **Location:** locationID: S2; continent: Europe; country: Spain; countryCode: ES; stateProvince: Andalucía; county: Granada; locality: Camarate; verbatimElevation: 1713.96; decimalLatitude: 37.18377; decimalLongitude: -3.26282; geodeticDatum: WGS84; **Event:** eventID: 3; samplingProtocol: Aerial; eventTime: Night
- **Type status:** Other material. **Occurrence:** individualCount: 1; sex: female; **Location:** locationID: S2; continent: Europe; country: Spain; countryCode: ES; stateProvince: Andalucía; county: Granada; locality: Camarate; verbatimElevation: 1713.96; decimalLatitude: 37.18377; decimalLongitude: -3.26282; geodeticDatum: WGS84; **Event:** eventID: 4; samplingProtocol: Aerial; eventTime: Night
- **Type status:** Other material. **Occurrence:** individualCount: 5; sex: female; **Location:** locationID: S2; continent: Europe; country: Spain; countryCode: ES; stateProvince: Andalucía; county: Granada; locality: Camarate; verbatimElevation: 1713.96; decimalLatitude: 37.18377; decimalLongitude: -3.26282; geodeticDatum: WGS84; **Event:** eventID: 1; samplingProtocol: Beating; eventTime: Night
- **Type status:** Other material. **Occurrence:** individualCount: 1; sex: male; **Location:** locationID: S2; continent: Europe; country: Spain; countryCode: ES; stateProvince: Andalucía; county: Granada; locality: Camarate; verbatimElevation: 1713.96; decimalLatitude: 37.18377; decimalLongitude: -3.26282; geodeticDatum: WGS84; **Event:** eventID: 2; samplingProtocol: Beating; eventTime: Night
- **Type status:** Other material. **Occurrence:** individualCount: 2; sex: female; **Location:** locationID: S2; continent: Europe; country: Spain; countryCode: ES; stateProvince: Andalucía; county: Granada; locality: Camarate; verbatimElevation: 1713.96; decimalLatitude: 37.18377; decimalLongitude: -3.26282; geodeticDatum: WGS84; **Event:** eventID: 2; samplingProtocol: Beating; eventTime: Night
- **Type status:** Other material. **Occurrence:** individualCount: 2; sex: female; **Location:** locationID: S2; continent: Europe; country: Spain; countryCode: ES; stateProvince: Andalucía; county: Granada; locality: Camarate; verbatimElevation: 1713.96; decimalLatitude: 37.18377; decimalLongitude: -3.26282; geodeticDatum: WGS84; **Event:** eventID: 1; samplingProtocol: Sweeping; eventTime: Night
- **Type status:** Other material. **Occurrence:** individualCount: 1; sex: male; **Location:** locationID: S2; continent: Europe; country: Spain; countryCode: ES; stateProvince: Andalucía; county: Granada; locality: Camarate; verbatimElevation: 1713.96; decimalLatitude: 37.18377; decimalLongitude: -3.26282; geodeticDatum: WGS84; **Event:** eventID: 2; samplingProtocol: Sweeping; eventTime: Night
- **Type status:** Other material. **Occurrence:** individualCount: 1; sex: female; **Location:** locationID: S2; continent: Europe; country: Spain; countryCode: ES; stateProvince: Andalucía; county: Granada; locality: Camarate; verbatimElevation: 1713.96; decimalLatitude: 37.18377; decimalLongitude: -3.26282; geodeticDatum: WGS84; **Event:** eventID: 2; samplingProtocol: Sweeping; eventTime: Day

##### Distribution

Europe to Central Asia

#### Cercidia prominens

(Westring, 1851)

##### Materials

- **Type status:** Other material. **Occurrence:** individualCount: 1; sex: female; **Location:** locationID: O1; continent: Europe; country: Spain; countryCode: ES; stateProvince: Aragón; county: Huesca; locality: O Furno; verbatimElevation: 1396.73; decimalLatitude: 42.60677; decimalLongitude: 0.13135; geodeticDatum: WGS84; **Event:** eventID: 1; samplingProtocol: Sweeping; eventTime: Night
- **Type status:** Other material. **Occurrence:** individualCount: 1; sex: female; **Location:** locationID: O1; continent: Europe; country: Spain; countryCode: ES; stateProvince: Aragón; county: Huesca; locality: O Furno; verbatimElevation: 1396.73; decimalLatitude: 42.60677; decimalLongitude: 0.13135; geodeticDatum: WGS84; **Event:** eventID: 2; samplingProtocol: Sweeping; eventTime: Day
- **Type status:** Other material. **Occurrence:** individualCount: 1; sex: female; **Location:** locationID: P2; continent: Europe; country: Spain; countryCode: ES; stateProvince: Castilla y León; county: León; locality: Joyoguelas; verbatimElevation: 763.98; decimalLatitude: 43.17771; decimalLongitude: -4.90579; geodeticDatum: WGS84; **Event:** eventID: 1; samplingProtocol: Sweeping; eventTime: Day
- **Type status:** Other material. **Occurrence:** individualCount: 1; sex: female; **Location:** locationID: P2; continent: Europe; country: Spain; countryCode: ES; stateProvince: Castilla y León; county: León; locality: Joyoguelas; verbatimElevation: 763.98; decimalLatitude: 43.17771; decimalLongitude: -4.90579; geodeticDatum: WGS84; **Event:** eventID: 1; samplingProtocol: Sweeping; eventTime: Night
- **Type status:** Other material. **Occurrence:** individualCount: 1; sex: female; **Location:** locationID: P2; continent: Europe; country: Spain; countryCode: ES; stateProvince: Castilla y León; county: León; locality: Joyoguelas; verbatimElevation: 763.98; decimalLatitude: 43.17771; decimalLongitude: -4.90579; geodeticDatum: WGS84; **Event:** eventID: C; samplingProtocol: Pitfall
- **Type status:** Other material. **Occurrence:** individualCount: 1; sex: male; **Location:** locationID: P4; continent: Europe; country: Spain; countryCode: ES; stateProvince: Castilla y León; county: León; locality: El Canto; verbatimElevation: 943.48; decimalLatitude: 43.17227; decimalLongitude: -4.90857; geodeticDatum: WGS84; **Event:** eventID: C; samplingProtocol: Pitfall
- **Type status:** Other material. **Occurrence:** individualCount: 1; sex: female; **Location:** locationID: P4; continent: Europe; country: Spain; countryCode: ES; stateProvince: Castilla y León; county: León; locality: El Canto; verbatimElevation: 943.48; decimalLatitude: 43.17227; decimalLongitude: -4.90857; geodeticDatum: WGS84; **Event:** eventID: E; samplingProtocol: Pitfall
- **Type status:** Other material. **Occurrence:** individualCount: 1; sex: male; **Location:** locationID: P4; continent: Europe; country: Spain; countryCode: ES; stateProvince: Castilla y León; county: León; locality: El Canto; verbatimElevation: 943.48; decimalLatitude: 43.17227; decimalLongitude: -4.90857; geodeticDatum: WGS84; **Event:** eventID: H; samplingProtocol: Pitfall

##### Distribution

Holarctic

#### Cyclosa algerica

Simon, 1885

##### Materials

- **Type status:** Other material. **Occurrence:** individualCount: 1; sex: female; **Location:** locationID: C2; continent: Europe; country: Spain; countryCode: ES; stateProvince: Castilla-La Mancha; county: Ciudad Real; locality: Valle Brezoso; verbatimElevation: 739.31; decimalLatitude: 39.35159; decimalLongitude: -4.3589; geodeticDatum: WGS84; **Event:** eventID: 2; samplingProtocol: Sweeping; eventTime: Night
- **Type status:** Other material. **Occurrence:** individualCount: 2; sex: female; **Location:** locationID: C3; continent: Europe; country: Spain; countryCode: ES; stateProvince: Castilla-La Mancha; county: Ciudad Real; locality: La Quesera; verbatimElevation: 767.55; decimalLatitude: 39.36177; decimalLongitude: -4.41733; geodeticDatum: WGS84; **Event:** eventID: 1; samplingProtocol: Aerial; eventTime: Night
- **Type status:** Other material. **Occurrence:** individualCount: 1; sex: female; **Location:** locationID: C3; continent: Europe; country: Spain; countryCode: ES; stateProvince: Castilla-La Mancha; county: Ciudad Real; locality: La Quesera; verbatimElevation: 767.55; decimalLatitude: 39.36177; decimalLongitude: -4.41733; geodeticDatum: WGS84; **Event:** eventID: 1; samplingProtocol: Beating; eventTime: Night
- **Type status:** Other material. **Occurrence:** individualCount: 1; sex: female; **Location:** locationID: C3; continent: Europe; country: Spain; countryCode: ES; stateProvince: Castilla-La Mancha; county: Ciudad Real; locality: La Quesera; verbatimElevation: 767.55; decimalLatitude: 39.36177; decimalLongitude: -4.41733; geodeticDatum: WGS84; **Event:** eventID: 1; samplingProtocol: Sweeping; eventTime: Day
- **Type status:** Other material. **Occurrence:** individualCount: 1; sex: female; **Location:** locationID: C3; continent: Europe; country: Spain; countryCode: ES; stateProvince: Castilla-La Mancha; county: Ciudad Real; locality: La Quesera; verbatimElevation: 767.55; decimalLatitude: 39.36177; decimalLongitude: -4.41733; geodeticDatum: WGS84; **Event:** eventID: 2; samplingProtocol: Sweeping; eventTime: Day
- **Type status:** Other material. **Occurrence:** individualCount: 1; sex: female; **Location:** locationID: C4; continent: Europe; country: Spain; countryCode: ES; stateProvince: Castilla-La Mancha; county: Ciudad Real; locality: La Quesera; verbatimElevation: 772.3; decimalLatitude: 39.36337; decimalLongitude: -4.41704; geodeticDatum: WGS84; **Event:** eventID: 1; samplingProtocol: Aerial; eventTime: Night
- **Type status:** Other material. **Occurrence:** individualCount: 2; sex: female; **Location:** locationID: C4; continent: Europe; country: Spain; countryCode: ES; stateProvince: Castilla-La Mancha; county: Ciudad Real; locality: La Quesera; verbatimElevation: 772.3; decimalLatitude: 39.36337; decimalLongitude: -4.41704; geodeticDatum: WGS84; **Event:** eventID: 2; samplingProtocol: Beating; eventTime: Day
- **Type status:** Other material. **Occurrence:** individualCount: 1; sex: female; **Location:** locationID: S1; continent: Europe; country: Spain; countryCode: ES; stateProvince: Andalucía; county: Granada; locality: Soportujar; verbatimElevation: 1786.57; decimalLatitude: 36.96151; decimalLongitude: -3.41881; geodeticDatum: WGS84; **Event:** eventID: 1; samplingProtocol: Aerial; eventTime: Night
- **Type status:** Other material. **Occurrence:** individualCount: 3; sex: female; **Location:** locationID: S1; continent: Europe; country: Spain; countryCode: ES; stateProvince: Andalucía; county: Granada; locality: Soportujar; verbatimElevation: 1786.57; decimalLatitude: 36.96151; decimalLongitude: -3.41881; geodeticDatum: WGS84; **Event:** eventID: 2; samplingProtocol: Aerial; eventTime: Night
- **Type status:** Other material. **Occurrence:** individualCount: 1; sex: male; **Location:** locationID: S1; continent: Europe; country: Spain; countryCode: ES; stateProvince: Andalucía; county: Granada; locality: Soportujar; verbatimElevation: 1786.57; decimalLatitude: 36.96151; decimalLongitude: -3.41881; geodeticDatum: WGS84; **Event:** eventID: 1; samplingProtocol: Beating; eventTime: Day
- **Type status:** Other material. **Occurrence:** individualCount: 1; sex: female; **Location:** locationID: S1; continent: Europe; country: Spain; countryCode: ES; stateProvince: Andalucía; county: Granada; locality: Soportujar; verbatimElevation: 1786.57; decimalLatitude: 36.96151; decimalLongitude: -3.41881; geodeticDatum: WGS84; **Event:** eventID: 4; samplingProtocol: Aerial; eventTime: Night
- **Type status:** Other material. **Occurrence:** individualCount: 1; sex: female; **Location:** locationID: S2; continent: Europe; country: Spain; countryCode: ES; stateProvince: Andalucía; county: Granada; locality: Camarate; verbatimElevation: 1713.96; decimalLatitude: 37.18377; decimalLongitude: -3.26282; geodeticDatum: WGS84; **Event:** eventID: 2; samplingProtocol: Aerial; eventTime: Night
- **Type status:** Other material. **Occurrence:** individualCount: 1; sex: female; **Location:** locationID: S2; continent: Europe; country: Spain; countryCode: ES; stateProvince: Andalucía; county: Granada; locality: Camarate; verbatimElevation: 1713.96; decimalLatitude: 37.18377; decimalLongitude: -3.26282; geodeticDatum: WGS84; **Event:** eventID: 3; samplingProtocol: Aerial; eventTime: Night

##### Distribution

Mediterranean

#### Cyclosa conica

(Pallas, 1772)

##### Materials

- **Type status:** Other material. **Occurrence:** individualCount: 1; sex: male; **Location:** locationID: A1; continent: Europe; country: Spain; countryCode: ES; stateProvince: Catalonia; county: Lleida; locality: Sola de Boi; verbatimElevation: 1759.8; decimalLatitude: 42.54958; decimalLongitude: 0.87254; geodeticDatum: WGS84; **Event:** eventID: 1; samplingProtocol: Aerial; eventTime: Night
- **Type status:** Other material. **Occurrence:** individualCount: 2; sex: female; **Location:** locationID: A1; continent: Europe; country: Spain; countryCode: ES; stateProvince: Catalonia; county: Lleida; locality: Sola de Boi; verbatimElevation: 1759.8; decimalLatitude: 42.54958; decimalLongitude: 0.87254; geodeticDatum: WGS84; **Event:** eventID: 1; samplingProtocol: Aerial; eventTime: Night
- **Type status:** Other material. **Occurrence:** individualCount: 1; sex: female; **Location:** locationID: A1; continent: Europe; country: Spain; countryCode: ES; stateProvince: Catalonia; county: Lleida; locality: Sola de Boi; verbatimElevation: 1759.8; decimalLatitude: 42.54958; decimalLongitude: 0.87254; geodeticDatum: WGS84; **Event:** eventID: 1; samplingProtocol: Aerial; eventTime: Night
- **Type status:** Other material. **Occurrence:** individualCount: 2; sex: male; **Location:** locationID: A1; continent: Europe; country: Spain; countryCode: ES; stateProvince: Catalonia; county: Lleida; locality: Sola de Boi; verbatimElevation: 1759.8; decimalLatitude: 42.54958; decimalLongitude: 0.87254; geodeticDatum: WGS84; **Event:** eventID: 2; samplingProtocol: Aerial; eventTime: Night
- **Type status:** Other material. **Occurrence:** individualCount: 1; sex: female; **Location:** locationID: A1; continent: Europe; country: Spain; countryCode: ES; stateProvince: Catalonia; county: Lleida; locality: Sola de Boi; verbatimElevation: 1759.8; decimalLatitude: 42.54958; decimalLongitude: 0.87254; geodeticDatum: WGS84; **Event:** eventID: 2; samplingProtocol: Aerial; eventTime: Night
- **Type status:** Other material. **Occurrence:** individualCount: 1; sex: male; **Location:** locationID: A1; continent: Europe; country: Spain; countryCode: ES; stateProvince: Catalonia; county: Lleida; locality: Sola de Boi; verbatimElevation: 1759.8; decimalLatitude: 42.54958; decimalLongitude: 0.87254; geodeticDatum: WGS84; **Event:** eventID: 1; samplingProtocol: Beating; eventTime: Night
- **Type status:** Other material. **Occurrence:** individualCount: 1; sex: female; **Location:** locationID: A1; continent: Europe; country: Spain; countryCode: ES; stateProvince: Catalonia; county: Lleida; locality: Sola de Boi; verbatimElevation: 1759.8; decimalLatitude: 42.54958; decimalLongitude: 0.87254; geodeticDatum: WGS84; **Event:** eventID: 1; samplingProtocol: Beating; eventTime: Night
- **Type status:** Other material. **Occurrence:** individualCount: 1; sex: female; **Location:** locationID: A1; continent: Europe; country: Spain; countryCode: ES; stateProvince: Catalonia; county: Lleida; locality: Sola de Boi; verbatimElevation: 1759.8; decimalLatitude: 42.54958; decimalLongitude: 0.87254; geodeticDatum: WGS84; **Event:** eventID: 1; samplingProtocol: Sweeping; eventTime: Night
- **Type status:** Other material. **Occurrence:** individualCount: 1; sex: female; **Location:** locationID: A2; continent: Europe; country: Spain; countryCode: ES; stateProvince: Catalonia; county: Lleida; locality: Sola de Boi; verbatimElevation: 1738.7; decimalLatitude: 42.54913; decimalLongitude: 0.87137; geodeticDatum: WGS84; **Event:** eventID: 1; samplingProtocol: Aerial; eventTime: Night
- **Type status:** Other material. **Occurrence:** individualCount: 1; sex: female; **Location:** locationID: A2; continent: Europe; country: Spain; countryCode: ES; stateProvince: Catalonia; county: Lleida; locality: Sola de Boi; verbatimElevation: 1738.7; decimalLatitude: 42.54913; decimalLongitude: 0.87137; geodeticDatum: WGS84; **Event:** eventID: 1; samplingProtocol: Aerial; eventTime: Night
- **Type status:** Other material. **Occurrence:** individualCount: 1; sex: male; **Location:** locationID: A2; continent: Europe; country: Spain; countryCode: ES; stateProvince: Catalonia; county: Lleida; locality: Sola de Boi; verbatimElevation: 1738.7; decimalLatitude: 42.54913; decimalLongitude: 0.87137; geodeticDatum: WGS84; **Event:** eventID: 2; samplingProtocol: Aerial; eventTime: Night
- **Type status:** Other material. **Occurrence:** individualCount: 1; sex: female; **Location:** locationID: A2; continent: Europe; country: Spain; countryCode: ES; stateProvince: Catalonia; county: Lleida; locality: Sola de Boi; verbatimElevation: 1738.7; decimalLatitude: 42.54913; decimalLongitude: 0.87137; geodeticDatum: WGS84; **Event:** eventID: 1; samplingProtocol: Sweeping; eventTime: Day
- **Type status:** Other material. **Occurrence:** individualCount: 1; sex: female; **Location:** locationID: A2; continent: Europe; country: Spain; countryCode: ES; stateProvince: Catalonia; county: Lleida; locality: Sola de Boi; verbatimElevation: 1738.7; decimalLatitude: 42.54913; decimalLongitude: 0.87137; geodeticDatum: WGS84; **Event:** eventID: 1; samplingProtocol: Sweeping; eventTime: Night
- **Type status:** Other material. **Occurrence:** individualCount: 2; sex: female; **Location:** locationID: O1; continent: Europe; country: Spain; countryCode: ES; stateProvince: Aragón; county: Huesca; locality: O Furno; verbatimElevation: 1396.73; decimalLatitude: 42.60677; decimalLongitude: 0.13135; geodeticDatum: WGS84; **Event:** eventID: 1; samplingProtocol: Sweeping; eventTime: Night
- **Type status:** Other material. **Occurrence:** individualCount: 2; sex: female; **Location:** locationID: P1; continent: Europe; country: Spain; countryCode: ES; stateProvince: Castilla y León; county: León; locality: Monte Robledo; verbatimElevation: 1071.58; decimalLatitude: 43.1445; decimalLongitude: -4.92675; geodeticDatum: WGS84; **Event:** eventID: 1; samplingProtocol: Aerial; eventTime: Night
- **Type status:** Other material. **Occurrence:** individualCount: 2; sex: female; **Location:** locationID: P1; continent: Europe; country: Spain; countryCode: ES; stateProvince: Castilla y León; county: León; locality: Monte Robledo; verbatimElevation: 1071.58; decimalLatitude: 43.1445; decimalLongitude: -4.92675; geodeticDatum: WGS84; **Event:** eventID: 1; samplingProtocol: Aerial; eventTime: Night
- **Type status:** Other material. **Occurrence:** individualCount: 1; sex: male; **Location:** locationID: P1; continent: Europe; country: Spain; countryCode: ES; stateProvince: Castilla y León; county: León; locality: Monte Robledo; verbatimElevation: 1071.58; decimalLatitude: 43.1445; decimalLongitude: -4.92675; geodeticDatum: WGS84; **Event:** eventID: 1; samplingProtocol: Beating; eventTime: Night
- **Type status:** Other material. **Occurrence:** individualCount: 1; sex: female; **Location:** locationID: P1; continent: Europe; country: Spain; countryCode: ES; stateProvince: Castilla y León; county: León; locality: Monte Robledo; verbatimElevation: 1071.58; decimalLatitude: 43.1445; decimalLongitude: -4.92675; geodeticDatum: WGS84; **Event:** eventID: 1; samplingProtocol: Beating; eventTime: Night
- **Type status:** Other material. **Occurrence:** individualCount: 1; sex: male; **Location:** locationID: P1; continent: Europe; country: Spain; countryCode: ES; stateProvince: Castilla y León; county: León; locality: Monte Robledo; verbatimElevation: 1071.58; decimalLatitude: 43.1445; decimalLongitude: -4.92675; geodeticDatum: WGS84; **Event:** eventID: 1; samplingProtocol: Beating; eventTime: Night
- **Type status:** Other material. **Occurrence:** individualCount: 1; sex: male; **Location:** locationID: P1; continent: Europe; country: Spain; countryCode: ES; stateProvince: Castilla y León; county: León; locality: Monte Robledo; verbatimElevation: 1071.58; decimalLatitude: 43.1445; decimalLongitude: -4.92675; geodeticDatum: WGS84; **Event:** eventID: 2; samplingProtocol: Beating; eventTime: Day
- **Type status:** Other material. **Occurrence:** individualCount: 1; sex: male; **Location:** locationID: P1; continent: Europe; country: Spain; countryCode: ES; stateProvince: Castilla y León; county: León; locality: Monte Robledo; verbatimElevation: 1071.58; decimalLatitude: 43.1445; decimalLongitude: -4.92675; geodeticDatum: WGS84; **Event:** eventID: 1; samplingProtocol: Sweeping; eventTime: Night
- **Type status:** Other material. **Occurrence:** individualCount: 1; sex: female; **Location:** locationID: P1; continent: Europe; country: Spain; countryCode: ES; stateProvince: Castilla y León; county: León; locality: Monte Robledo; verbatimElevation: 1071.58; decimalLatitude: 43.1445; decimalLongitude: -4.92675; geodeticDatum: WGS84; **Event:** eventID: 1; samplingProtocol: Sweeping; eventTime: Night
- **Type status:** Other material. **Occurrence:** individualCount: 1; sex: female; **Location:** locationID: P2; continent: Europe; country: Spain; countryCode: ES; stateProvince: Castilla y León; county: León; locality: Joyoguelas; verbatimElevation: 763.98; decimalLatitude: 43.17771; decimalLongitude: -4.90579; geodeticDatum: WGS84; **Event:** eventID: 1; samplingProtocol: Aerial; eventTime: Night
- **Type status:** Other material. **Occurrence:** individualCount: 1; sex: female; **Location:** locationID: P2; continent: Europe; country: Spain; countryCode: ES; stateProvince: Castilla y León; county: León; locality: Joyoguelas; verbatimElevation: 763.98; decimalLatitude: 43.17771; decimalLongitude: -4.90579; geodeticDatum: WGS84; **Event:** eventID: 1; samplingProtocol: Beating; eventTime: Night
- **Type status:** Other material. **Occurrence:** individualCount: 1; sex: female; **Location:** locationID: P2; continent: Europe; country: Spain; countryCode: ES; stateProvince: Castilla y León; county: León; locality: Joyoguelas; verbatimElevation: 763.98; decimalLatitude: 43.17771; decimalLongitude: -4.90579; geodeticDatum: WGS84; **Event:** eventID: 1; samplingProtocol: Sweeping; eventTime: Night
- **Type status:** Other material. **Occurrence:** individualCount: 1; sex: male; **Location:** locationID: P2; continent: Europe; country: Spain; countryCode: ES; stateProvince: Castilla y León; county: León; locality: Joyoguelas; verbatimElevation: 763.98; decimalLatitude: 43.17771; decimalLongitude: -4.90579; geodeticDatum: WGS84; **Event:** eventID: 1; samplingProtocol: Sweeping; eventTime: Night
- **Type status:** Other material. **Occurrence:** individualCount: 1; sex: female; **Location:** locationID: P3; continent: Europe; country: Spain; countryCode: ES; stateProvince: Castilla y León; county: León; locality: Las Arroyas; verbatimElevation: 1097.1; decimalLatitude: 43.14351; decimalLongitude: -4.94878; geodeticDatum: WGS84; **Event:** eventID: 1; samplingProtocol: Aerial; eventTime: Night
- **Type status:** Other material. **Occurrence:** individualCount: 4; sex: female; **Location:** locationID: P3; continent: Europe; country: Spain; countryCode: ES; stateProvince: Castilla y León; county: León; locality: Las Arroyas; verbatimElevation: 1097.1; decimalLatitude: 43.14351; decimalLongitude: -4.94878; geodeticDatum: WGS84; **Event:** eventID: 1; samplingProtocol: Aerial; eventTime: Night
- **Type status:** Other material. **Occurrence:** individualCount: 1; sex: female; **Location:** locationID: P3; continent: Europe; country: Spain; countryCode: ES; stateProvince: Castilla y León; county: León; locality: Las Arroyas; verbatimElevation: 1097.1; decimalLatitude: 43.14351; decimalLongitude: -4.94878; geodeticDatum: WGS84; **Event:** eventID: 2; samplingProtocol: Aerial; eventTime: Night
- **Type status:** Other material. **Occurrence:** individualCount: 5; sex: female; **Location:** locationID: P3; continent: Europe; country: Spain; countryCode: ES; stateProvince: Castilla y León; county: León; locality: Las Arroyas; verbatimElevation: 1097.1; decimalLatitude: 43.14351; decimalLongitude: -4.94878; geodeticDatum: WGS84; **Event:** eventID: 2; samplingProtocol: Aerial; eventTime: Night
- **Type status:** Other material. **Occurrence:** individualCount: 1; sex: male; **Location:** locationID: P3; continent: Europe; country: Spain; countryCode: ES; stateProvince: Castilla y León; county: León; locality: Las Arroyas; verbatimElevation: 1097.1; decimalLatitude: 43.14351; decimalLongitude: -4.94878; geodeticDatum: WGS84; **Event:** eventID: 1; samplingProtocol: Beating; eventTime: Day
- **Type status:** Other material. **Occurrence:** individualCount: 1; sex: female; **Location:** locationID: P3; continent: Europe; country: Spain; countryCode: ES; stateProvince: Castilla y León; county: León; locality: Las Arroyas; verbatimElevation: 1097.1; decimalLatitude: 43.14351; decimalLongitude: -4.94878; geodeticDatum: WGS84; **Event:** eventID: 1; samplingProtocol: Beating; eventTime: Day
- **Type status:** Other material. **Occurrence:** individualCount: 1; sex: male; **Location:** locationID: P3; continent: Europe; country: Spain; countryCode: ES; stateProvince: Castilla y León; county: León; locality: Las Arroyas; verbatimElevation: 1097.1; decimalLatitude: 43.14351; decimalLongitude: -4.94878; geodeticDatum: WGS84; **Event:** eventID: 1; samplingProtocol: Beating; eventTime: Night
- **Type status:** Other material. **Occurrence:** individualCount: 1; sex: female; **Location:** locationID: P3; continent: Europe; country: Spain; countryCode: ES; stateProvince: Castilla y León; county: León; locality: Las Arroyas; verbatimElevation: 1097.1; decimalLatitude: 43.14351; decimalLongitude: -4.94878; geodeticDatum: WGS84; **Event:** eventID: 1; samplingProtocol: Beating; eventTime: Night
- **Type status:** Other material. **Occurrence:** individualCount: 2; sex: female; **Location:** locationID: P3; continent: Europe; country: Spain; countryCode: ES; stateProvince: Castilla y León; county: León; locality: Las Arroyas; verbatimElevation: 1097.1; decimalLatitude: 43.14351; decimalLongitude: -4.94878; geodeticDatum: WGS84; **Event:** eventID: 1; samplingProtocol: Beating; eventTime: Night
- **Type status:** Other material. **Occurrence:** individualCount: 2; sex: female; **Location:** locationID: P3; continent: Europe; country: Spain; countryCode: ES; stateProvince: Castilla y León; county: León; locality: Las Arroyas; verbatimElevation: 1097.1; decimalLatitude: 43.14351; decimalLongitude: -4.94878; geodeticDatum: WGS84; **Event:** eventID: 2; samplingProtocol: Beating; eventTime: Day
- **Type status:** Other material. **Occurrence:** individualCount: 1; sex: male; **Location:** locationID: P3; continent: Europe; country: Spain; countryCode: ES; stateProvince: Castilla y León; county: León; locality: Las Arroyas; verbatimElevation: 1097.1; decimalLatitude: 43.14351; decimalLongitude: -4.94878; geodeticDatum: WGS84; **Event:** eventID: 1; samplingProtocol: Sweeping; eventTime: Day
- **Type status:** Other material. **Occurrence:** individualCount: 1; sex: female; **Location:** locationID: P3; continent: Europe; country: Spain; countryCode: ES; stateProvince: Castilla y León; county: León; locality: Las Arroyas; verbatimElevation: 1097.1; decimalLatitude: 43.14351; decimalLongitude: -4.94878; geodeticDatum: WGS84; **Event:** eventID: 1; samplingProtocol: Sweeping; eventTime: Night
- **Type status:** Other material. **Occurrence:** individualCount: 1; sex: female; **Location:** locationID: P4; continent: Europe; country: Spain; countryCode: ES; stateProvince: Castilla y León; county: León; locality: El Canto; verbatimElevation: 943.48; decimalLatitude: 43.17227; decimalLongitude: -4.90857; geodeticDatum: WGS84; **Event:** eventID: 1; samplingProtocol: Aerial; eventTime: Night
- **Type status:** Other material. **Occurrence:** individualCount: 1; sex: female; **Location:** locationID: P4; continent: Europe; country: Spain; countryCode: ES; stateProvince: Castilla y León; county: León; locality: El Canto; verbatimElevation: 943.48; decimalLatitude: 43.17227; decimalLongitude: -4.90857; geodeticDatum: WGS84; **Event:** eventID: 2; samplingProtocol: Aerial; eventTime: Night

##### Distribution

Holarctic

#### Gibbaranea bituberculata

(Walckenaer, 1802)

##### Materials

- **Type status:** Other material. **Occurrence:** individualCount: 1; sex: female; **Location:** locationID: P2; continent: Europe; country: Spain; countryCode: ES; stateProvince: Castilla y León; county: León; locality: Joyoguelas; verbatimElevation: 763.98; decimalLatitude: 43.17771; decimalLongitude: -4.90579; geodeticDatum: WGS84; **Event:** eventID: 1; samplingProtocol: Aerial; eventTime: Night
- **Type status:** Other material. **Occurrence:** individualCount: 1; sex: female; **Location:** locationID: P2; continent: Europe; country: Spain; countryCode: ES; stateProvince: Castilla y León; county: León; locality: Joyoguelas; verbatimElevation: 763.98; decimalLatitude: 43.17771; decimalLongitude: -4.90579; geodeticDatum: WGS84; **Event:** eventID: 1; samplingProtocol: Sweeping; eventTime: Day
- **Type status:** Other material. **Occurrence:** individualCount: 1; sex: female; **Location:** locationID: P2; continent: Europe; country: Spain; countryCode: ES; stateProvince: Castilla y León; county: León; locality: Joyoguelas; verbatimElevation: 763.98; decimalLatitude: 43.17771; decimalLongitude: -4.90579; geodeticDatum: WGS84; **Event:** eventID: 1; samplingProtocol: Sweeping; eventTime: Night
- **Type status:** Other material. **Occurrence:** individualCount: 3; sex: female; **Location:** locationID: P2; continent: Europe; country: Spain; countryCode: ES; stateProvince: Castilla y León; county: León; locality: Joyoguelas; verbatimElevation: 763.98; decimalLatitude: 43.17771; decimalLongitude: -4.90579; geodeticDatum: WGS84; **Event:** eventID: 1; samplingProtocol: Sweeping; eventTime: Night
- **Type status:** Other material. **Occurrence:** individualCount: 1; sex: female; **Location:** locationID: P2; continent: Europe; country: Spain; countryCode: ES; stateProvince: Castilla y León; county: León; locality: Joyoguelas; verbatimElevation: 763.98; decimalLatitude: 43.17771; decimalLongitude: -4.90579; geodeticDatum: WGS84; **Event:** eventID: 2; samplingProtocol: Sweeping; eventTime: Day
- **Type status:** Other material. **Occurrence:** individualCount: 1; sex: female; **Location:** locationID: P4; continent: Europe; country: Spain; countryCode: ES; stateProvince: Castilla y León; county: León; locality: El Canto; verbatimElevation: 943.48; decimalLatitude: 43.17227; decimalLongitude: -4.90857; geodeticDatum: WGS84; **Event:** eventID: 1; samplingProtocol: Aerial; eventTime: Night
- **Type status:** Other material. **Occurrence:** individualCount: 1; sex: male; **Location:** locationID: P4; continent: Europe; country: Spain; countryCode: ES; stateProvince: Castilla y León; county: León; locality: El Canto; verbatimElevation: 943.48; decimalLatitude: 43.17227; decimalLongitude: -4.90857; geodeticDatum: WGS84; **Event:** eventID: 1; samplingProtocol: Sweeping; eventTime: Night
- **Type status:** Other material. **Occurrence:** individualCount: 1; sex: female; **Location:** locationID: P4; continent: Europe; country: Spain; countryCode: ES; stateProvince: Castilla y León; county: León; locality: El Canto; verbatimElevation: 943.48; decimalLatitude: 43.17227; decimalLongitude: -4.90857; geodeticDatum: WGS84; **Event:** eventID: 1; samplingProtocol: Sweeping; eventTime: Night
- **Type status:** Other material. **Occurrence:** individualCount: 1; sex: female; **Location:** locationID: P4; continent: Europe; country: Spain; countryCode: ES; stateProvince: Castilla y León; county: León; locality: El Canto; verbatimElevation: 943.48; decimalLatitude: 43.17227; decimalLongitude: -4.90857; geodeticDatum: WGS84; **Event:** eventID: 2; samplingProtocol: Sweeping; eventTime: Day

##### Distribution

Palearctic

#### Gibbaranea gibbosa

(Walckenaer, 1802)

##### Materials

- **Type status:** Other material. **Occurrence:** individualCount: 1; sex: male; **Location:** locationID: C4; continent: Europe; country: Spain; countryCode: ES; stateProvince: Castilla-La Mancha; county: Ciudad Real; locality: La Quesera; verbatimElevation: 772.3; decimalLatitude: 39.36337; decimalLongitude: -4.41704; geodeticDatum: WGS84; **Event:** eventID: 3; samplingProtocol: Aerial; eventTime: Night
- **Type status:** Other material. **Occurrence:** individualCount: 1; sex: female; **Location:** locationID: C4; continent: Europe; country: Spain; countryCode: ES; stateProvince: Castilla-La Mancha; county: Ciudad Real; locality: La Quesera; verbatimElevation: 772.3; decimalLatitude: 39.36337; decimalLongitude: -4.41704; geodeticDatum: WGS84; **Event:** eventID: 3; samplingProtocol: Aerial; eventTime: Night
- **Type status:** Other material. **Occurrence:** individualCount: 1; sex: female; **Location:** locationID: M2; continent: Europe; country: Spain; countryCode: ES; stateProvince: Extremadura; county: Cáceres; locality: Fuente del Frances; verbatimElevation: 320.72; decimalLatitude: 39.828; decimalLongitude: -6.03249; geodeticDatum: WGS84; **Event:** eventID: 1; samplingProtocol: Aerial; eventTime: Night
- **Type status:** Other material. **Occurrence:** individualCount: 2; sex: female; **Location:** locationID: O1; continent: Europe; country: Spain; countryCode: ES; stateProvince: Aragón; county: Huesca; locality: O Furno; verbatimElevation: 1396.73; decimalLatitude: 42.60677; decimalLongitude: 0.13135; geodeticDatum: WGS84; **Event:** eventID: 1; samplingProtocol: Aerial; eventTime: Night
- **Type status:** Other material. **Occurrence:** individualCount: 1; sex: male; **Location:** locationID: O1; continent: Europe; country: Spain; countryCode: ES; stateProvince: Aragón; county: Huesca; locality: O Furno; verbatimElevation: 1396.73; decimalLatitude: 42.60677; decimalLongitude: 0.13135; geodeticDatum: WGS84; **Event:** eventID: 1; samplingProtocol: Aerial; eventTime: Night
- **Type status:** Other material. **Occurrence:** individualCount: 1; sex: female; **Location:** locationID: O1; continent: Europe; country: Spain; countryCode: ES; stateProvince: Aragón; county: Huesca; locality: O Furno; verbatimElevation: 1396.73; decimalLatitude: 42.60677; decimalLongitude: 0.13135; geodeticDatum: WGS84; **Event:** eventID: 1; samplingProtocol: Aerial; eventTime: Night
- **Type status:** Other material. **Occurrence:** individualCount: 2; sex: female; **Location:** locationID: O1; continent: Europe; country: Spain; countryCode: ES; stateProvince: Aragón; county: Huesca; locality: O Furno; verbatimElevation: 1396.73; decimalLatitude: 42.60677; decimalLongitude: 0.13135; geodeticDatum: WGS84; **Event:** eventID: 2; samplingProtocol: Aerial; eventTime: Night
- **Type status:** Other material. **Occurrence:** individualCount: 1; sex: male; **Location:** locationID: O1; continent: Europe; country: Spain; countryCode: ES; stateProvince: Aragón; county: Huesca; locality: O Furno; verbatimElevation: 1396.73; decimalLatitude: 42.60677; decimalLongitude: 0.13135; geodeticDatum: WGS84; **Event:** eventID: 1; samplingProtocol: Beating; eventTime: Night
- **Type status:** Other material. **Occurrence:** individualCount: 1; sex: female; **Location:** locationID: O1; continent: Europe; country: Spain; countryCode: ES; stateProvince: Aragón; county: Huesca; locality: O Furno; verbatimElevation: 1396.73; decimalLatitude: 42.60677; decimalLongitude: 0.13135; geodeticDatum: WGS84; **Event:** eventID: 1; samplingProtocol: Beating; eventTime: Night
- **Type status:** Other material. **Occurrence:** individualCount: 1; sex: female; **Location:** locationID: O2; continent: Europe; country: Spain; countryCode: ES; stateProvince: Aragón; county: Huesca; locality: Rebilla; verbatimElevation: 1158.13; decimalLatitude: 42.59427; decimalLongitude: 0.1529; geodeticDatum: WGS84; **Event:** eventID: 1; samplingProtocol: Beating; eventTime: Day
- **Type status:** Other material. **Occurrence:** individualCount: 2; sex: female; **Location:** locationID: O2; continent: Europe; country: Spain; countryCode: ES; stateProvince: Aragón; county: Huesca; locality: Rebilla; verbatimElevation: 1158.13; decimalLatitude: 42.59427; decimalLongitude: 0.1529; geodeticDatum: WGS84; **Event:** eventID: 1; samplingProtocol: Beating; eventTime: Night
- **Type status:** Other material. **Occurrence:** individualCount: 1; sex: female; **Location:** locationID: P1; continent: Europe; country: Spain; countryCode: ES; stateProvince: Castilla y León; county: León; locality: Monte Robledo; verbatimElevation: 1071.58; decimalLatitude: 43.1445; decimalLongitude: -4.92675; geodeticDatum: WGS84; **Event:** eventID: 1; samplingProtocol: Aerial; eventTime: Night
- **Type status:** Other material. **Occurrence:** individualCount: 1; sex: female; **Location:** locationID: P2; continent: Europe; country: Spain; countryCode: ES; stateProvince: Castilla y León; county: León; locality: Joyoguelas; verbatimElevation: 763.98; decimalLatitude: 43.17771; decimalLongitude: -4.90579; geodeticDatum: WGS84; **Event:** eventID: 1; samplingProtocol: Beating; eventTime: Night
- **Type status:** Other material. **Occurrence:** individualCount: 1; sex: female; **Location:** locationID: S1; continent: Europe; country: Spain; countryCode: ES; stateProvince: Andalucía; county: Granada; locality: Soportujar; verbatimElevation: 1786.57; decimalLatitude: 36.96151; decimalLongitude: -3.41881; geodeticDatum: WGS84; **Event:** eventID: 2; samplingProtocol: Aerial; eventTime: Night
- **Type status:** Other material. **Occurrence:** individualCount: 1; sex: male; **Location:** locationID: S1; continent: Europe; country: Spain; countryCode: ES; stateProvince: Andalucía; county: Granada; locality: Soportujar; verbatimElevation: 1786.57; decimalLatitude: 36.96151; decimalLongitude: -3.41881; geodeticDatum: WGS84; **Event:** eventID: 3; samplingProtocol: Aerial; eventTime: Night
- **Type status:** Other material. **Occurrence:** individualCount: 1; sex: male; **Location:** locationID: S1; continent: Europe; country: Spain; countryCode: ES; stateProvince: Andalucía; county: Granada; locality: Soportujar; verbatimElevation: 1786.57; decimalLatitude: 36.96151; decimalLongitude: -3.41881; geodeticDatum: WGS84; **Event:** eventID: 4; samplingProtocol: Aerial; eventTime: Night
- **Type status:** Other material. **Occurrence:** individualCount: 1; sex: female; **Location:** locationID: S1; continent: Europe; country: Spain; countryCode: ES; stateProvince: Andalucía; county: Granada; locality: Soportujar; verbatimElevation: 1786.57; decimalLatitude: 36.96151; decimalLongitude: -3.41881; geodeticDatum: WGS84; **Event:** eventID: 2; samplingProtocol: Sweeping; eventTime: Night

##### Distribution

Europe to Azerbaijan

#### Hypsosinga sanguinea

(C. L. Koch, 1844)

##### Materials

- **Type status:** Other material. **Occurrence:** individualCount: 1; sex: female; **Location:** locationID: O2; continent: Europe; country: Spain; countryCode: ES; stateProvince: Aragón; county: Huesca; locality: Rebilla; verbatimElevation: 1158.13; decimalLatitude: 42.59427; decimalLongitude: 0.1529; geodeticDatum: WGS84; **Event:** eventID: 1; samplingProtocol: Sweeping; eventTime: Night

##### Distribution

Palearctic

#### Larinioides sclopetarius

(Clerck, 1757)

##### Materials

- **Type status:** Other material. **Occurrence:** individualCount: 2; sex: female; **Location:** locationID: C2; continent: Europe; country: Spain; countryCode: ES; stateProvince: Castilla-La Mancha; county: Ciudad Real; locality: Valle Brezoso; verbatimElevation: 739.31; decimalLatitude: 39.35159; decimalLongitude: -4.3589; geodeticDatum: WGS84; **Event:** eventID: 2; samplingProtocol: Aerial; eventTime: Night
- **Type status:** Other material. **Occurrence:** individualCount: 1; sex: female; **Location:** locationID: C2; continent: Europe; country: Spain; countryCode: ES; stateProvince: Castilla-La Mancha; county: Ciudad Real; locality: Valle Brezoso; verbatimElevation: 739.31; decimalLatitude: 39.35159; decimalLongitude: -4.3589; geodeticDatum: WGS84; **Event:** eventID: 4; samplingProtocol: Aerial; eventTime: Night

##### Distribution

Europe, Caucasus, Russia (Europe to Central Asia), China, Korea, introduced in North America

#### Leviellus kochi

(Thorell, 1870)

##### Materials

- **Type status:** Other material. **Occurrence:** individualCount: 2; sex: female; **Location:** locationID: S1; continent: Europe; country: Spain; countryCode: ES; stateProvince: Andalucía; county: Granada; locality: Soportujar; verbatimElevation: 1786.57; decimalLatitude: 36.96151; decimalLongitude: -3.41881; geodeticDatum: WGS84; **Event:** eventID: 3; samplingProtocol: Aerial; eventTime: Night
- **Type status:** Other material. **Occurrence:** individualCount: 1; sex: male; **Location:** locationID: S1; continent: Europe; country: Spain; countryCode: ES; stateProvince: Andalucía; county: Granada; locality: Soportujar; verbatimElevation: 1786.57; decimalLatitude: 36.96151; decimalLongitude: -3.41881; geodeticDatum: WGS84; **Event:** eventID: 2; samplingProtocol: Sweeping; eventTime: Night

##### Distribution

Southern Europe, North Africa, Central Asia

#### Mangora acalypha

(Walckenaer, 1802)

##### Materials

- **Type status:** Other material. **Occurrence:** individualCount: 1; sex: female; **Location:** locationID: C1; continent: Europe; country: Spain; countryCode: ES; stateProvince: Castilla-La Mancha; county: Ciudad Real; locality: Valle Brezoso; verbatimElevation: 756.56; decimalLatitude: 39.35663; decimalLongitude: -4.35912; geodeticDatum: WGS84; **Event:** eventID: 1; samplingProtocol: Beating; eventTime: Day
- **Type status:** Other material. **Occurrence:** individualCount: 2; sex: female; **Location:** locationID: C1; continent: Europe; country: Spain; countryCode: ES; stateProvince: Castilla-La Mancha; county: Ciudad Real; locality: Valle Brezoso; verbatimElevation: 756.56; decimalLatitude: 39.35663; decimalLongitude: -4.35912; geodeticDatum: WGS84; **Event:** eventID: 1; samplingProtocol: Sweeping; eventTime: Day
- **Type status:** Other material. **Occurrence:** individualCount: 1; sex: female; **Location:** locationID: C1; continent: Europe; country: Spain; countryCode: ES; stateProvince: Castilla-La Mancha; county: Ciudad Real; locality: Valle Brezoso; verbatimElevation: 756.56; decimalLatitude: 39.35663; decimalLongitude: -4.35912; geodeticDatum: WGS84; **Event:** eventID: 1; samplingProtocol: Sweeping; eventTime: Night
- **Type status:** Other material. **Occurrence:** individualCount: 2; sex: female; **Location:** locationID: C1; continent: Europe; country: Spain; countryCode: ES; stateProvince: Castilla-La Mancha; county: Ciudad Real; locality: Valle Brezoso; verbatimElevation: 756.56; decimalLatitude: 39.35663; decimalLongitude: -4.35912; geodeticDatum: WGS84; **Event:** eventID: 2; samplingProtocol: Sweeping; eventTime: Night
- **Type status:** Other material. **Occurrence:** individualCount: 6; sex: female; **Location:** locationID: C1; continent: Europe; country: Spain; countryCode: ES; stateProvince: Castilla-La Mancha; county: Ciudad Real; locality: Valle Brezoso; verbatimElevation: 756.56; decimalLatitude: 39.35663; decimalLongitude: -4.35912; geodeticDatum: WGS84; **Event:** eventID: 2; samplingProtocol: Sweeping; eventTime: Day
- **Type status:** Other material. **Occurrence:** individualCount: 2; sex: female; **Location:** locationID: C2; continent: Europe; country: Spain; countryCode: ES; stateProvince: Castilla-La Mancha; county: Ciudad Real; locality: Valle Brezoso; verbatimElevation: 739.31; decimalLatitude: 39.35159; decimalLongitude: -4.3589; geodeticDatum: WGS84; **Event:** eventID: 1; samplingProtocol: Aerial; eventTime: Night
- **Type status:** Other material. **Occurrence:** individualCount: 1; sex: female; **Location:** locationID: C2; continent: Europe; country: Spain; countryCode: ES; stateProvince: Castilla-La Mancha; county: Ciudad Real; locality: Valle Brezoso; verbatimElevation: 739.31; decimalLatitude: 39.35159; decimalLongitude: -4.3589; geodeticDatum: WGS84; **Event:** eventID: 2; samplingProtocol: Aerial; eventTime: Night
- **Type status:** Other material. **Occurrence:** individualCount: 1; sex: female; **Location:** locationID: C2; continent: Europe; country: Spain; countryCode: ES; stateProvince: Castilla-La Mancha; county: Ciudad Real; locality: Valle Brezoso; verbatimElevation: 739.31; decimalLatitude: 39.35159; decimalLongitude: -4.3589; geodeticDatum: WGS84; **Event:** eventID: 3; samplingProtocol: Aerial; eventTime: Night
- **Type status:** Other material. **Occurrence:** individualCount: 2; sex: female; **Location:** locationID: C2; continent: Europe; country: Spain; countryCode: ES; stateProvince: Castilla-La Mancha; county: Ciudad Real; locality: Valle Brezoso; verbatimElevation: 739.31; decimalLatitude: 39.35159; decimalLongitude: -4.3589; geodeticDatum: WGS84; **Event:** eventID: 1; samplingProtocol: Beating; eventTime: Day
- **Type status:** Other material. **Occurrence:** individualCount: 3; sex: female; **Location:** locationID: C2; continent: Europe; country: Spain; countryCode: ES; stateProvince: Castilla-La Mancha; county: Ciudad Real; locality: Valle Brezoso; verbatimElevation: 739.31; decimalLatitude: 39.35159; decimalLongitude: -4.3589; geodeticDatum: WGS84; **Event:** eventID: 2; samplingProtocol: Sweeping; eventTime: Night
- **Type status:** Other material. **Occurrence:** individualCount: 1; sex: female; **Location:** locationID: C3; continent: Europe; country: Spain; countryCode: ES; stateProvince: Castilla-La Mancha; county: Ciudad Real; locality: La Quesera; verbatimElevation: 767.55; decimalLatitude: 39.36177; decimalLongitude: -4.41733; geodeticDatum: WGS84; **Event:** eventID: 1; samplingProtocol: Aerial; eventTime: Night
- **Type status:** Other material. **Occurrence:** individualCount: 3; sex: female; **Location:** locationID: C3; continent: Europe; country: Spain; countryCode: ES; stateProvince: Castilla-La Mancha; county: Ciudad Real; locality: La Quesera; verbatimElevation: 767.55; decimalLatitude: 39.36177; decimalLongitude: -4.41733; geodeticDatum: WGS84; **Event:** eventID: 2; samplingProtocol: Aerial; eventTime: Night
- **Type status:** Other material. **Occurrence:** individualCount: 2; sex: female; **Location:** locationID: C3; continent: Europe; country: Spain; countryCode: ES; stateProvince: Castilla-La Mancha; county: Ciudad Real; locality: La Quesera; verbatimElevation: 767.55; decimalLatitude: 39.36177; decimalLongitude: -4.41733; geodeticDatum: WGS84; **Event:** eventID: 1; samplingProtocol: Beating; eventTime: Night
- **Type status:** Other material. **Occurrence:** individualCount: 4; sex: female; **Location:** locationID: C3; continent: Europe; country: Spain; countryCode: ES; stateProvince: Castilla-La Mancha; county: Ciudad Real; locality: La Quesera; verbatimElevation: 767.55; decimalLatitude: 39.36177; decimalLongitude: -4.41733; geodeticDatum: WGS84; **Event:** eventID: 2; samplingProtocol: Beating; eventTime: Day
- **Type status:** Other material. **Occurrence:** individualCount: 1; sex: female; **Location:** locationID: C3; continent: Europe; country: Spain; countryCode: ES; stateProvince: Castilla-La Mancha; county: Ciudad Real; locality: La Quesera; verbatimElevation: 767.55; decimalLatitude: 39.36177; decimalLongitude: -4.41733; geodeticDatum: WGS84; **Event:** eventID: 1; samplingProtocol: Sweeping; eventTime: Night
- **Type status:** Other material. **Occurrence:** individualCount: 3; sex: female; **Location:** locationID: C3; continent: Europe; country: Spain; countryCode: ES; stateProvince: Castilla-La Mancha; county: Ciudad Real; locality: La Quesera; verbatimElevation: 767.55; decimalLatitude: 39.36177; decimalLongitude: -4.41733; geodeticDatum: WGS84; **Event:** eventID: 2; samplingProtocol: Sweeping; eventTime: Night
- **Type status:** Other material. **Occurrence:** individualCount: 2; sex: female; **Location:** locationID: C3; continent: Europe; country: Spain; countryCode: ES; stateProvince: Castilla-La Mancha; county: Ciudad Real; locality: La Quesera; verbatimElevation: 767.55; decimalLatitude: 39.36177; decimalLongitude: -4.41733; geodeticDatum: WGS84; **Event:** eventID: 2; samplingProtocol: Sweeping; eventTime: Day
- **Type status:** Other material. **Occurrence:** individualCount: 1; sex: female; **Location:** locationID: C4; continent: Europe; country: Spain; countryCode: ES; stateProvince: Castilla-La Mancha; county: Ciudad Real; locality: La Quesera; verbatimElevation: 772.3; decimalLatitude: 39.36337; decimalLongitude: -4.41704; geodeticDatum: WGS84; **Event:** eventID: 3; samplingProtocol: Aerial; eventTime: Night
- **Type status:** Other material. **Occurrence:** individualCount: 1; sex: female; **Location:** locationID: C4; continent: Europe; country: Spain; countryCode: ES; stateProvince: Castilla-La Mancha; county: Ciudad Real; locality: La Quesera; verbatimElevation: 772.3; decimalLatitude: 39.36337; decimalLongitude: -4.41704; geodeticDatum: WGS84; **Event:** eventID: 2; samplingProtocol: Aerial; eventTime: Night
- **Type status:** Other material. **Occurrence:** individualCount: 1; sex: male; **Location:** locationID: C4; continent: Europe; country: Spain; countryCode: ES; stateProvince: Castilla-La Mancha; county: Ciudad Real; locality: La Quesera; verbatimElevation: 772.3; decimalLatitude: 39.36337; decimalLongitude: -4.41704; geodeticDatum: WGS84; **Event:** eventID: D; samplingProtocol: Pitfall
- **Type status:** Other material. **Occurrence:** individualCount: 6; sex: female; **Location:** locationID: C4; continent: Europe; country: Spain; countryCode: ES; stateProvince: Castilla-La Mancha; county: Ciudad Real; locality: La Quesera; verbatimElevation: 772.3; decimalLatitude: 39.36337; decimalLongitude: -4.41704; geodeticDatum: WGS84; **Event:** eventID: 1; samplingProtocol: Sweeping; eventTime: Day
- **Type status:** Other material. **Occurrence:** individualCount: 3; sex: female; **Location:** locationID: C4; continent: Europe; country: Spain; countryCode: ES; stateProvince: Castilla-La Mancha; county: Ciudad Real; locality: La Quesera; verbatimElevation: 772.3; decimalLatitude: 39.36337; decimalLongitude: -4.41704; geodeticDatum: WGS84; **Event:** eventID: 1; samplingProtocol: Sweeping; eventTime: Night
- **Type status:** Other material. **Occurrence:** individualCount: 1; sex: female; **Location:** locationID: C4; continent: Europe; country: Spain; countryCode: ES; stateProvince: Castilla-La Mancha; county: Ciudad Real; locality: La Quesera; verbatimElevation: 772.3; decimalLatitude: 39.36337; decimalLongitude: -4.41704; geodeticDatum: WGS84; **Event:** eventID: 2; samplingProtocol: Sweeping; eventTime: Night
- **Type status:** Other material. **Occurrence:** individualCount: 7; sex: female; **Location:** locationID: C4; continent: Europe; country: Spain; countryCode: ES; stateProvince: Castilla-La Mancha; county: Ciudad Real; locality: La Quesera; verbatimElevation: 772.3; decimalLatitude: 39.36337; decimalLongitude: -4.41704; geodeticDatum: WGS84; **Event:** eventID: 2; samplingProtocol: Sweeping; eventTime: Day
- **Type status:** Other material. **Occurrence:** individualCount: 1; sex: female; **Location:** locationID: M1; continent: Europe; country: Spain; countryCode: ES; stateProvince: Extremadura; county: Cáceres; locality: Peña Falcón; verbatimElevation: 320.6; decimalLatitude: 39.83296; decimalLongitude: -6.0641; geodeticDatum: WGS84; **Event:** eventID: 2; samplingProtocol: Aerial; eventTime: Night
- **Type status:** Other material. **Occurrence:** individualCount: 1; sex: female; **Location:** locationID: O2; continent: Europe; country: Spain; countryCode: ES; stateProvince: Aragón; county: Huesca; locality: Rebilla; verbatimElevation: 1158.13; decimalLatitude: 42.59427; decimalLongitude: 0.1529; geodeticDatum: WGS84; **Event:** eventID: 2; samplingProtocol: Aerial; eventTime: Night
- **Type status:** Other material. **Occurrence:** individualCount: 1; sex: female; **Location:** locationID: O2; continent: Europe; country: Spain; countryCode: ES; stateProvince: Aragón; county: Huesca; locality: Rebilla; verbatimElevation: 1158.13; decimalLatitude: 42.59427; decimalLongitude: 0.1529; geodeticDatum: WGS84; **Event:** eventID: 1; samplingProtocol: Sweeping; eventTime: Night
- **Type status:** Other material. **Occurrence:** individualCount: 1; sex: female; **Location:** locationID: O2; continent: Europe; country: Spain; countryCode: ES; stateProvince: Aragón; county: Huesca; locality: Rebilla; verbatimElevation: 1158.13; decimalLatitude: 42.59427; decimalLongitude: 0.1529; geodeticDatum: WGS84; **Event:** eventID: 1; samplingProtocol: Sweeping; eventTime: Night
- **Type status:** Other material. **Occurrence:** individualCount: 4; sex: male; **Location:** locationID: O2; continent: Europe; country: Spain; countryCode: ES; stateProvince: Aragón; county: Huesca; locality: Rebilla; verbatimElevation: 1158.13; decimalLatitude: 42.59427; decimalLongitude: 0.1529; geodeticDatum: WGS84; **Event:** eventID: 2; samplingProtocol: Sweeping; eventTime: Day
- **Type status:** Other material. **Occurrence:** individualCount: 2; sex: female; **Location:** locationID: O2; continent: Europe; country: Spain; countryCode: ES; stateProvince: Aragón; county: Huesca; locality: Rebilla; verbatimElevation: 1158.13; decimalLatitude: 42.59427; decimalLongitude: 0.1529; geodeticDatum: WGS84; **Event:** eventID: 2; samplingProtocol: Sweeping; eventTime: Day
- **Type status:** Other material. **Occurrence:** individualCount: 1; sex: female; **Location:** locationID: P2; continent: Europe; country: Spain; countryCode: ES; stateProvince: Castilla y León; county: León; locality: Joyoguelas; verbatimElevation: 763.98; decimalLatitude: 43.17771; decimalLongitude: -4.90579; geodeticDatum: WGS84; **Event:** eventID: 1; samplingProtocol: Sweeping; eventTime: Night
- **Type status:** Other material. **Occurrence:** individualCount: 1; sex: male; **Location:** locationID: P2; continent: Europe; country: Spain; countryCode: ES; stateProvince: Castilla y León; county: León; locality: Joyoguelas; verbatimElevation: 763.98; decimalLatitude: 43.17771; decimalLongitude: -4.90579; geodeticDatum: WGS84; **Event:** eventID: 1; samplingProtocol: Sweeping; eventTime: Night
- **Type status:** Other material. **Occurrence:** individualCount: 1; sex: female; **Location:** locationID: P2; continent: Europe; country: Spain; countryCode: ES; stateProvince: Castilla y León; county: León; locality: Joyoguelas; verbatimElevation: 763.98; decimalLatitude: 43.17771; decimalLongitude: -4.90579; geodeticDatum: WGS84; **Event:** eventID: 1; samplingProtocol: Sweeping; eventTime: Night
- **Type status:** Other material. **Occurrence:** individualCount: 1; sex: male; **Location:** locationID: P2; continent: Europe; country: Spain; countryCode: ES; stateProvince: Castilla y León; county: León; locality: Joyoguelas; verbatimElevation: 763.98; decimalLatitude: 43.17771; decimalLongitude: -4.90579; geodeticDatum: WGS84; **Event:** eventID: 2; samplingProtocol: Sweeping; eventTime: Day
- **Type status:** Other material. **Occurrence:** individualCount: 1; sex: female; **Location:** locationID: P2; continent: Europe; country: Spain; countryCode: ES; stateProvince: Castilla y León; county: León; locality: Joyoguelas; verbatimElevation: 763.98; decimalLatitude: 43.17771; decimalLongitude: -4.90579; geodeticDatum: WGS84; **Event:** eventID: 2; samplingProtocol: Sweeping; eventTime: Day
- **Type status:** Other material. **Occurrence:** individualCount: 2; sex: male; **Location:** locationID: P4; continent: Europe; country: Spain; countryCode: ES; stateProvince: Castilla y León; county: León; locality: El Canto; verbatimElevation: 943.48; decimalLatitude: 43.17227; decimalLongitude: -4.90857; geodeticDatum: WGS84; **Event:** eventID: 1; samplingProtocol: Sweeping; eventTime: Day
- **Type status:** Other material. **Occurrence:** individualCount: 1; sex: female; **Location:** locationID: P4; continent: Europe; country: Spain; countryCode: ES; stateProvince: Castilla y León; county: León; locality: El Canto; verbatimElevation: 943.48; decimalLatitude: 43.17227; decimalLongitude: -4.90857; geodeticDatum: WGS84; **Event:** eventID: 1; samplingProtocol: Sweeping; eventTime: Day
- **Type status:** Other material. **Occurrence:** individualCount: 1; sex: male; **Location:** locationID: P4; continent: Europe; country: Spain; countryCode: ES; stateProvince: Castilla y León; county: León; locality: El Canto; verbatimElevation: 943.48; decimalLatitude: 43.17227; decimalLongitude: -4.90857; geodeticDatum: WGS84; **Event:** eventID: 1; samplingProtocol: Sweeping; eventTime: Night
- **Type status:** Other material. **Occurrence:** individualCount: 1; sex: female; **Location:** locationID: P4; continent: Europe; country: Spain; countryCode: ES; stateProvince: Castilla y León; county: León; locality: El Canto; verbatimElevation: 943.48; decimalLatitude: 43.17227; decimalLongitude: -4.90857; geodeticDatum: WGS84; **Event:** eventID: 1; samplingProtocol: Sweeping; eventTime: Night
- **Type status:** Other material. **Occurrence:** individualCount: 1; sex: male; **Location:** locationID: P4; continent: Europe; country: Spain; countryCode: ES; stateProvince: Castilla y León; county: León; locality: El Canto; verbatimElevation: 943.48; decimalLatitude: 43.17227; decimalLongitude: -4.90857; geodeticDatum: WGS84; **Event:** eventID: 1; samplingProtocol: Sweeping; eventTime: Night
- **Type status:** Other material. **Occurrence:** individualCount: 3; sex: female; **Location:** locationID: P4; continent: Europe; country: Spain; countryCode: ES; stateProvince: Castilla y León; county: León; locality: El Canto; verbatimElevation: 943.48; decimalLatitude: 43.17227; decimalLongitude: -4.90857; geodeticDatum: WGS84; **Event:** eventID: 1; samplingProtocol: Sweeping; eventTime: Night
- **Type status:** Other material. **Occurrence:** individualCount: 1; sex: male; **Location:** locationID: P4; continent: Europe; country: Spain; countryCode: ES; stateProvince: Castilla y León; county: León; locality: El Canto; verbatimElevation: 943.48; decimalLatitude: 43.17227; decimalLongitude: -4.90857; geodeticDatum: WGS84; **Event:** eventID: 2; samplingProtocol: Sweeping; eventTime: Day
- **Type status:** Other material. **Occurrence:** individualCount: 7; sex: female; **Location:** locationID: P4; continent: Europe; country: Spain; countryCode: ES; stateProvince: Castilla y León; county: León; locality: El Canto; verbatimElevation: 943.48; decimalLatitude: 43.17227; decimalLongitude: -4.90857; geodeticDatum: WGS84; **Event:** eventID: 2; samplingProtocol: Sweeping; eventTime: Day
- **Type status:** Other material. **Occurrence:** individualCount: 1; sex: female; **Location:** locationID: S1; continent: Europe; country: Spain; countryCode: ES; stateProvince: Andalucía; county: Granada; locality: Soportujar; verbatimElevation: 1786.57; decimalLatitude: 36.96151; decimalLongitude: -3.41881; geodeticDatum: WGS84; **Event:** eventID: 1; samplingProtocol: Sweeping; eventTime: Night
- **Type status:** Other material. **Occurrence:** individualCount: 1; sex: female; **Location:** locationID: S1; continent: Europe; country: Spain; countryCode: ES; stateProvince: Andalucía; county: Granada; locality: Soportujar; verbatimElevation: 1786.57; decimalLatitude: 36.96151; decimalLongitude: -3.41881; geodeticDatum: WGS84; **Event:** eventID: 2; samplingProtocol: Sweeping; eventTime: Night
- **Type status:** Other material. **Occurrence:** individualCount: 1; sex: female; **Location:** locationID: S2; continent: Europe; country: Spain; countryCode: ES; stateProvince: Andalucía; county: Granada; locality: Camarate; verbatimElevation: 1713.96; decimalLatitude: 37.18377; decimalLongitude: -3.26282; geodeticDatum: WGS84; **Event:** eventID: 1; samplingProtocol: Aerial; eventTime: Night
- **Type status:** Other material. **Occurrence:** individualCount: 2; sex: female; **Location:** locationID: S2; continent: Europe; country: Spain; countryCode: ES; stateProvince: Andalucía; county: Granada; locality: Camarate; verbatimElevation: 1713.96; decimalLatitude: 37.18377; decimalLongitude: -3.26282; geodeticDatum: WGS84; **Event:** eventID: 2; samplingProtocol: Aerial; eventTime: Night
- **Type status:** Other material. **Occurrence:** individualCount: 3; sex: female; **Location:** locationID: S2; continent: Europe; country: Spain; countryCode: ES; stateProvince: Andalucía; county: Granada; locality: Camarate; verbatimElevation: 1713.96; decimalLatitude: 37.18377; decimalLongitude: -3.26282; geodeticDatum: WGS84; **Event:** eventID: 3; samplingProtocol: Aerial; eventTime: Night
- **Type status:** Other material. **Occurrence:** individualCount: 1; sex: female; **Location:** locationID: S2; continent: Europe; country: Spain; countryCode: ES; stateProvince: Andalucía; county: Granada; locality: Camarate; verbatimElevation: 1713.96; decimalLatitude: 37.18377; decimalLongitude: -3.26282; geodeticDatum: WGS84; **Event:** eventID: 2; samplingProtocol: Beating; eventTime: Night
- **Type status:** Other material. **Occurrence:** individualCount: 3; sex: female; **Location:** locationID: S2; continent: Europe; country: Spain; countryCode: ES; stateProvince: Andalucía; county: Granada; locality: Camarate; verbatimElevation: 1713.96; decimalLatitude: 37.18377; decimalLongitude: -3.26282; geodeticDatum: WGS84; **Event:** eventID: 1; samplingProtocol: Sweeping; eventTime: Day
- **Type status:** Other material. **Occurrence:** individualCount: 3; sex: female; **Location:** locationID: S2; continent: Europe; country: Spain; countryCode: ES; stateProvince: Andalucía; county: Granada; locality: Camarate; verbatimElevation: 1713.96; decimalLatitude: 37.18377; decimalLongitude: -3.26282; geodeticDatum: WGS84; **Event:** eventID: 1; samplingProtocol: Sweeping; eventTime: Night
- **Type status:** Other material. **Occurrence:** individualCount: 1; sex: female; **Location:** locationID: S2; continent: Europe; country: Spain; countryCode: ES; stateProvince: Andalucía; county: Granada; locality: Camarate; verbatimElevation: 1713.96; decimalLatitude: 37.18377; decimalLongitude: -3.26282; geodeticDatum: WGS84; **Event:** eventID: 2; samplingProtocol: Sweeping; eventTime: Night
- **Type status:** Other material. **Occurrence:** individualCount: 1; sex: female; **Location:** locationID: S2; continent: Europe; country: Spain; countryCode: ES; stateProvince: Andalucía; county: Granada; locality: Camarate; verbatimElevation: 1713.96; decimalLatitude: 37.18377; decimalLongitude: -3.26282; geodeticDatum: WGS84; **Event:** eventID: 2; samplingProtocol: Sweeping; eventTime: Day

##### Distribution

Palearctic

#### Neoscona adianta

(Walckenaer, 1802)

##### Materials

- **Type status:** Other material. **Occurrence:** individualCount: 1; sex: male; **Location:** locationID: C1; continent: Europe; country: Spain; countryCode: ES; stateProvince: Castilla-La Mancha; county: Ciudad Real; locality: Valle Brezoso; verbatimElevation: 756.56; decimalLatitude: 39.35663; decimalLongitude: -4.35912; geodeticDatum: WGS84; **Event:** eventID: 2; samplingProtocol: Sweeping; eventTime: Day
- **Type status:** Other material. **Occurrence:** individualCount: 1; sex: male; **Location:** locationID: C3; continent: Europe; country: Spain; countryCode: ES; stateProvince: Castilla-La Mancha; county: Ciudad Real; locality: La Quesera; verbatimElevation: 767.55; decimalLatitude: 39.36177; decimalLongitude: -4.41733; geodeticDatum: WGS84; **Event:** eventID: 1; samplingProtocol: Sweeping; eventTime: Night
- **Type status:** Other material. **Occurrence:** individualCount: 1; sex: male; **Location:** locationID: C4; continent: Europe; country: Spain; countryCode: ES; stateProvince: Castilla-La Mancha; county: Ciudad Real; locality: La Quesera; verbatimElevation: 772.3; decimalLatitude: 39.36337; decimalLongitude: -4.41704; geodeticDatum: WGS84; **Event:** eventID: 2; samplingProtocol: Aerial; eventTime: Night

##### Distribution

Palearctic

#### Nuctenea umbratica

(Clerck, 1757)

##### Materials

- **Type status:** Other material. **Occurrence:** individualCount: 1; sex: female; **Location:** locationID: A1; continent: Europe; country: Spain; countryCode: ES; stateProvince: Catalonia; county: Lleida; locality: Sola de Boi; verbatimElevation: 1759.8; decimalLatitude: 42.54958; decimalLongitude: 0.87254; geodeticDatum: WGS84; **Event:** eventID: 2; samplingProtocol: Aerial; eventTime: Night
- **Type status:** Other material. **Occurrence:** individualCount: 2; sex: female; **Location:** locationID: A2; continent: Europe; country: Spain; countryCode: ES; stateProvince: Catalonia; county: Lleida; locality: Sola de Boi; verbatimElevation: 1738.7; decimalLatitude: 42.54913; decimalLongitude: 0.87137; geodeticDatum: WGS84; **Event:** eventID: 1; samplingProtocol: Aerial; eventTime: Night
- **Type status:** Other material. **Occurrence:** individualCount: 1; sex: female; **Location:** locationID: A2; continent: Europe; country: Spain; countryCode: ES; stateProvince: Catalonia; county: Lleida; locality: Sola de Boi; verbatimElevation: 1738.7; decimalLatitude: 42.54913; decimalLongitude: 0.87137; geodeticDatum: WGS84; **Event:** eventID: 2; samplingProtocol: Aerial; eventTime: Night
- **Type status:** Other material. **Occurrence:** individualCount: 1; sex: male; **Location:** locationID: C4; continent: Europe; country: Spain; countryCode: ES; stateProvince: Castilla-La Mancha; county: Ciudad Real; locality: La Quesera; verbatimElevation: 772.3; decimalLatitude: 39.36337; decimalLongitude: -4.41704; geodeticDatum: WGS84; **Event:** eventID: 2; samplingProtocol: Aerial; eventTime: Night
- **Type status:** Other material. **Occurrence:** individualCount: 1; sex: female; **Location:** locationID: C4; continent: Europe; country: Spain; countryCode: ES; stateProvince: Castilla-La Mancha; county: Ciudad Real; locality: La Quesera; verbatimElevation: 772.3; decimalLatitude: 39.36337; decimalLongitude: -4.41704; geodeticDatum: WGS84; **Event:** eventID: 2; samplingProtocol: Aerial; eventTime: Night
- **Type status:** Other material. **Occurrence:** individualCount: 1; sex: female; **Location:** locationID: M1; continent: Europe; country: Spain; countryCode: ES; stateProvince: Extremadura; county: Cáceres; locality: Peña Falcón; verbatimElevation: 320.6; decimalLatitude: 39.83296; decimalLongitude: -6.0641; geodeticDatum: WGS84; **Event:** eventID: 1; samplingProtocol: Aerial; eventTime: Night
- **Type status:** Other material. **Occurrence:** individualCount: 1; sex: female; **Location:** locationID: M1; continent: Europe; country: Spain; countryCode: ES; stateProvince: Extremadura; county: Cáceres; locality: Peña Falcón; verbatimElevation: 320.6; decimalLatitude: 39.83296; decimalLongitude: -6.0641; geodeticDatum: WGS84; **Event:** eventID: 2; samplingProtocol: Sweeping; eventTime: Night
- **Type status:** Other material. **Occurrence:** individualCount: 1; sex: female; **Location:** locationID: P1; continent: Europe; country: Spain; countryCode: ES; stateProvince: Castilla y León; county: León; locality: Monte Robledo; verbatimElevation: 1071.58; decimalLatitude: 43.1445; decimalLongitude: -4.92675; geodeticDatum: WGS84; **Event:** eventID: 1; samplingProtocol: Aerial; eventTime: Night
- **Type status:** Other material. **Occurrence:** individualCount: 1; sex: female; **Location:** locationID: P2; continent: Europe; country: Spain; countryCode: ES; stateProvince: Castilla y León; county: León; locality: Joyoguelas; verbatimElevation: 763.98; decimalLatitude: 43.17771; decimalLongitude: -4.90579; geodeticDatum: WGS84; **Event:** eventID: 1; samplingProtocol: Aerial; eventTime: Night
- **Type status:** Other material. **Occurrence:** individualCount: 1; sex: female; **Location:** locationID: P2; continent: Europe; country: Spain; countryCode: ES; stateProvince: Castilla y León; county: León; locality: Joyoguelas; verbatimElevation: 763.98; decimalLatitude: 43.17771; decimalLongitude: -4.90579; geodeticDatum: WGS84; **Event:** eventID: 1; samplingProtocol: Aerial; eventTime: Night
- **Type status:** Other material. **Occurrence:** individualCount: 1; sex: female; **Location:** locationID: P4; continent: Europe; country: Spain; countryCode: ES; stateProvince: Castilla y León; county: León; locality: El Canto; verbatimElevation: 943.48; decimalLatitude: 43.17227; decimalLongitude: -4.90857; geodeticDatum: WGS84; **Event:** eventID: 1; samplingProtocol: Aerial; eventTime: Night
- **Type status:** Other material. **Occurrence:** individualCount: 1; sex: female; **Location:** locationID: P4; continent: Europe; country: Spain; countryCode: ES; stateProvince: Castilla y León; county: León; locality: El Canto; verbatimElevation: 943.48; decimalLatitude: 43.17227; decimalLongitude: -4.90857; geodeticDatum: WGS84; **Event:** eventID: 2; samplingProtocol: Aerial; eventTime: Night
- **Type status:** Other material. **Occurrence:** individualCount: 1; sex: female; **Location:** locationID: P4; continent: Europe; country: Spain; countryCode: ES; stateProvince: Castilla y León; county: León; locality: El Canto; verbatimElevation: 943.48; decimalLatitude: 43.17227; decimalLongitude: -4.90857; geodeticDatum: WGS84; **Event:** eventID: 1; samplingProtocol: Beating; eventTime: Night
- **Type status:** Other material. **Occurrence:** individualCount: 1; sex: female; **Location:** locationID: P4; continent: Europe; country: Spain; countryCode: ES; stateProvince: Castilla y León; county: León; locality: El Canto; verbatimElevation: 943.48; decimalLatitude: 43.17227; decimalLongitude: -4.90857; geodeticDatum: WGS84; **Event:** eventID: 1; samplingProtocol: Sweeping; eventTime: Night

##### Distribution

Europe to Azerbaijan

#### Zilla diodia

(Walckenaer, 1802)

##### Materials

- **Type status:** Other material. **Occurrence:** individualCount: 1; sex: male; **Location:** locationID: A2; continent: Europe; country: Spain; countryCode: ES; stateProvince: Catalonia; county: Lleida; locality: Sola de Boi; verbatimElevation: 1738.7; decimalLatitude: 42.54913; decimalLongitude: 0.87137; geodeticDatum: WGS84; **Event:** eventID: 1; samplingProtocol: Beating; eventTime: Night
- **Type status:** Other material. **Occurrence:** individualCount: 1; sex: female; **Location:** locationID: A2; continent: Europe; country: Spain; countryCode: ES; stateProvince: Catalonia; county: Lleida; locality: Sola de Boi; verbatimElevation: 1738.7; decimalLatitude: 42.54913; decimalLongitude: 0.87137; geodeticDatum: WGS84; **Event:** eventID: 1; samplingProtocol: Sweeping; eventTime: Day
- **Type status:** Other material. **Occurrence:** individualCount: 1; sex: female; **Location:** locationID: A2; continent: Europe; country: Spain; countryCode: ES; stateProvince: Catalonia; county: Lleida; locality: Sola de Boi; verbatimElevation: 1738.7; decimalLatitude: 42.54913; decimalLongitude: 0.87137; geodeticDatum: WGS84; **Event:** eventID: 2; samplingProtocol: Sweeping; eventTime: Day
- **Type status:** Other material. **Occurrence:** individualCount: 1; sex: female; **Location:** locationID: C1; continent: Europe; country: Spain; countryCode: ES; stateProvince: Castilla-La Mancha; county: Ciudad Real; locality: Valle Brezoso; verbatimElevation: 756.56; decimalLatitude: 39.35663; decimalLongitude: -4.35912; geodeticDatum: WGS84; **Event:** eventID: 2; samplingProtocol: Aerial; eventTime: Night
- **Type status:** Other material. **Occurrence:** individualCount: 1; sex: female; **Location:** locationID: C1; continent: Europe; country: Spain; countryCode: ES; stateProvince: Castilla-La Mancha; county: Ciudad Real; locality: Valle Brezoso; verbatimElevation: 756.56; decimalLatitude: 39.35663; decimalLongitude: -4.35912; geodeticDatum: WGS84; **Event:** eventID: 4; samplingProtocol: Aerial; eventTime: Night
- **Type status:** Other material. **Occurrence:** individualCount: 3; sex: male; **Location:** locationID: O1; continent: Europe; country: Spain; countryCode: ES; stateProvince: Aragón; county: Huesca; locality: O Furno; verbatimElevation: 1396.73; decimalLatitude: 42.60677; decimalLongitude: 0.13135; geodeticDatum: WGS84; **Event:** eventID: 1; samplingProtocol: Aerial; eventTime: Night
- **Type status:** Other material. **Occurrence:** individualCount: 1; sex: female; **Location:** locationID: O1; continent: Europe; country: Spain; countryCode: ES; stateProvince: Aragón; county: Huesca; locality: O Furno; verbatimElevation: 1396.73; decimalLatitude: 42.60677; decimalLongitude: 0.13135; geodeticDatum: WGS84; **Event:** eventID: 2; samplingProtocol: Beating; eventTime: Day
- **Type status:** Other material. **Occurrence:** individualCount: 1; sex: female; **Location:** locationID: O2; continent: Europe; country: Spain; countryCode: ES; stateProvince: Aragón; county: Huesca; locality: Rebilla; verbatimElevation: 1158.13; decimalLatitude: 42.59427; decimalLongitude: 0.1529; geodeticDatum: WGS84; **Event:** eventID: 1; samplingProtocol: Aerial; eventTime: Night
- **Type status:** Other material. **Occurrence:** individualCount: 1; sex: female; **Location:** locationID: O2; continent: Europe; country: Spain; countryCode: ES; stateProvince: Aragón; county: Huesca; locality: Rebilla; verbatimElevation: 1158.13; decimalLatitude: 42.59427; decimalLongitude: 0.1529; geodeticDatum: WGS84; **Event:** eventID: 1; samplingProtocol: Aerial; eventTime: Night
- **Type status:** Other material. **Occurrence:** individualCount: 1; sex: female; **Location:** locationID: O2; continent: Europe; country: Spain; countryCode: ES; stateProvince: Aragón; county: Huesca; locality: Rebilla; verbatimElevation: 1158.13; decimalLatitude: 42.59427; decimalLongitude: 0.1529; geodeticDatum: WGS84; **Event:** eventID: 2; samplingProtocol: Aerial; eventTime: Night
- **Type status:** Other material. **Occurrence:** individualCount: 2; sex: female; **Location:** locationID: P2; continent: Europe; country: Spain; countryCode: ES; stateProvince: Castilla y León; county: León; locality: Joyoguelas; verbatimElevation: 763.98; decimalLatitude: 43.17771; decimalLongitude: -4.90579; geodeticDatum: WGS84; **Event:** eventID: 1; samplingProtocol: Aerial; eventTime: Night
- **Type status:** Other material. **Occurrence:** individualCount: 2; sex: female; **Location:** locationID: P2; continent: Europe; country: Spain; countryCode: ES; stateProvince: Castilla y León; county: León; locality: Joyoguelas; verbatimElevation: 763.98; decimalLatitude: 43.17771; decimalLongitude: -4.90579; geodeticDatum: WGS84; **Event:** eventID: 2; samplingProtocol: Aerial; eventTime: Night
- **Type status:** Other material. **Occurrence:** individualCount: 1; sex: male; **Location:** locationID: P2; continent: Europe; country: Spain; countryCode: ES; stateProvince: Castilla y León; county: León; locality: Joyoguelas; verbatimElevation: 763.98; decimalLatitude: 43.17771; decimalLongitude: -4.90579; geodeticDatum: WGS84; **Event:** eventID: 2; samplingProtocol: Aerial; eventTime: Night
- **Type status:** Other material. **Occurrence:** individualCount: 3; sex: female; **Location:** locationID: P2; continent: Europe; country: Spain; countryCode: ES; stateProvince: Castilla y León; county: León; locality: Joyoguelas; verbatimElevation: 763.98; decimalLatitude: 43.17771; decimalLongitude: -4.90579; geodeticDatum: WGS84; **Event:** eventID: 2; samplingProtocol: Aerial; eventTime: Night
- **Type status:** Other material. **Occurrence:** individualCount: 3; sex: female; **Location:** locationID: P2; continent: Europe; country: Spain; countryCode: ES; stateProvince: Castilla y León; county: León; locality: Joyoguelas; verbatimElevation: 763.98; decimalLatitude: 43.17771; decimalLongitude: -4.90579; geodeticDatum: WGS84; **Event:** eventID: 1; samplingProtocol: Beating; eventTime: Day
- **Type status:** Other material. **Occurrence:** individualCount: 1; sex: female; **Location:** locationID: P2; continent: Europe; country: Spain; countryCode: ES; stateProvince: Castilla y León; county: León; locality: Joyoguelas; verbatimElevation: 763.98; decimalLatitude: 43.17771; decimalLongitude: -4.90579; geodeticDatum: WGS84; **Event:** eventID: 1; samplingProtocol: Beating; eventTime: Night
- **Type status:** Other material. **Occurrence:** individualCount: 1; sex: male; **Location:** locationID: P2; continent: Europe; country: Spain; countryCode: ES; stateProvince: Castilla y León; county: León; locality: Joyoguelas; verbatimElevation: 763.98; decimalLatitude: 43.17771; decimalLongitude: -4.90579; geodeticDatum: WGS84; **Event:** eventID: 1; samplingProtocol: Beating; eventTime: Night
- **Type status:** Other material. **Occurrence:** individualCount: 1; sex: female; **Location:** locationID: P2; continent: Europe; country: Spain; countryCode: ES; stateProvince: Castilla y León; county: León; locality: Joyoguelas; verbatimElevation: 763.98; decimalLatitude: 43.17771; decimalLongitude: -4.90579; geodeticDatum: WGS84; **Event:** eventID: 2; samplingProtocol: Beating; eventTime: Day
- **Type status:** Other material. **Occurrence:** individualCount: 1; sex: female; **Location:** locationID: P2; continent: Europe; country: Spain; countryCode: ES; stateProvince: Castilla y León; county: León; locality: Joyoguelas; verbatimElevation: 763.98; decimalLatitude: 43.17771; decimalLongitude: -4.90579; geodeticDatum: WGS84; **Event:** eventID: 1; samplingProtocol: Sweeping; eventTime: Day
- **Type status:** Other material. **Occurrence:** individualCount: 1; sex: female; **Location:** locationID: P2; continent: Europe; country: Spain; countryCode: ES; stateProvince: Castilla y León; county: León; locality: Joyoguelas; verbatimElevation: 763.98; decimalLatitude: 43.17771; decimalLongitude: -4.90579; geodeticDatum: WGS84; **Event:** eventID: 1; samplingProtocol: Sweeping; eventTime: Night
- **Type status:** Other material. **Occurrence:** individualCount: 2; sex: male; **Location:** locationID: P2; continent: Europe; country: Spain; countryCode: ES; stateProvince: Castilla y León; county: León; locality: Joyoguelas; verbatimElevation: 763.98; decimalLatitude: 43.17771; decimalLongitude: -4.90579; geodeticDatum: WGS84; **Event:** eventID: 2; samplingProtocol: Sweeping; eventTime: Day
- **Type status:** Other material. **Occurrence:** individualCount: 2; sex: female; **Location:** locationID: P2; continent: Europe; country: Spain; countryCode: ES; stateProvince: Castilla y León; county: León; locality: Joyoguelas; verbatimElevation: 763.98; decimalLatitude: 43.17771; decimalLongitude: -4.90579; geodeticDatum: WGS84; **Event:** eventID: 2; samplingProtocol: Sweeping; eventTime: Day
- **Type status:** Other material. **Occurrence:** individualCount: 1; sex: male; **Location:** locationID: P3; continent: Europe; country: Spain; countryCode: ES; stateProvince: Castilla y León; county: León; locality: Las Arroyas; verbatimElevation: 1097.1; decimalLatitude: 43.14351; decimalLongitude: -4.94878; geodeticDatum: WGS84; **Event:** eventID: 1; samplingProtocol: Beating; eventTime: Day
- **Type status:** Other material. **Occurrence:** individualCount: 1; sex: female; **Location:** locationID: P3; continent: Europe; country: Spain; countryCode: ES; stateProvince: Castilla y León; county: León; locality: Las Arroyas; verbatimElevation: 1097.1; decimalLatitude: 43.14351; decimalLongitude: -4.94878; geodeticDatum: WGS84; **Event:** eventID: 1; samplingProtocol: Beating; eventTime: Day
- **Type status:** Other material. **Occurrence:** individualCount: 2; sex: male; **Location:** locationID: P3; continent: Europe; country: Spain; countryCode: ES; stateProvince: Castilla y León; county: León; locality: Las Arroyas; verbatimElevation: 1097.1; decimalLatitude: 43.14351; decimalLongitude: -4.94878; geodeticDatum: WGS84; **Event:** eventID: 1; samplingProtocol: Beating; eventTime: Night
- **Type status:** Other material. **Occurrence:** individualCount: 1; sex: female; **Location:** locationID: P4; continent: Europe; country: Spain; countryCode: ES; stateProvince: Castilla y León; county: León; locality: El Canto; verbatimElevation: 943.48; decimalLatitude: 43.17227; decimalLongitude: -4.90857; geodeticDatum: WGS84; **Event:** eventID: 1; samplingProtocol: Aerial; eventTime: Night
- **Type status:** Other material. **Occurrence:** individualCount: 3; sex: female; **Location:** locationID: P4; continent: Europe; country: Spain; countryCode: ES; stateProvince: Castilla y León; county: León; locality: El Canto; verbatimElevation: 943.48; decimalLatitude: 43.17227; decimalLongitude: -4.90857; geodeticDatum: WGS84; **Event:** eventID: 2; samplingProtocol: Aerial; eventTime: Night
- **Type status:** Other material. **Occurrence:** individualCount: 1; sex: female; **Location:** locationID: P4; continent: Europe; country: Spain; countryCode: ES; stateProvince: Castilla y León; county: León; locality: El Canto; verbatimElevation: 943.48; decimalLatitude: 43.17227; decimalLongitude: -4.90857; geodeticDatum: WGS84; **Event:** eventID: 1; samplingProtocol: Beating; eventTime: Day
- **Type status:** Other material. **Occurrence:** individualCount: 6; sex: female; **Location:** locationID: P4; continent: Europe; country: Spain; countryCode: ES; stateProvince: Castilla y León; county: León; locality: El Canto; verbatimElevation: 943.48; decimalLatitude: 43.17227; decimalLongitude: -4.90857; geodeticDatum: WGS84; **Event:** eventID: 2; samplingProtocol: Beating; eventTime: Day
- **Type status:** Other material. **Occurrence:** individualCount: 1; sex: male; **Location:** locationID: P4; continent: Europe; country: Spain; countryCode: ES; stateProvince: Castilla y León; county: León; locality: El Canto; verbatimElevation: 943.48; decimalLatitude: 43.17227; decimalLongitude: -4.90857; geodeticDatum: WGS84; **Event:** eventID: 1; samplingProtocol: Beating; eventTime: Night
- **Type status:** Other material. **Occurrence:** individualCount: 1; sex: female; **Location:** locationID: P4; continent: Europe; country: Spain; countryCode: ES; stateProvince: Castilla y León; county: León; locality: El Canto; verbatimElevation: 943.48; decimalLatitude: 43.17227; decimalLongitude: -4.90857; geodeticDatum: WGS84; **Event:** eventID: 1; samplingProtocol: Beating; eventTime: Night
- **Type status:** Other material. **Occurrence:** individualCount: 2; sex: female; **Location:** locationID: P4; continent: Europe; country: Spain; countryCode: ES; stateProvince: Castilla y León; county: León; locality: El Canto; verbatimElevation: 943.48; decimalLatitude: 43.17227; decimalLongitude: -4.90857; geodeticDatum: WGS84; **Event:** eventID: 1; samplingProtocol: Sweeping; eventTime: Night
- **Type status:** Other material. **Occurrence:** individualCount: 1; sex: male; **Location:** locationID: P4; continent: Europe; country: Spain; countryCode: ES; stateProvince: Castilla y León; county: León; locality: El Canto; verbatimElevation: 943.48; decimalLatitude: 43.17227; decimalLongitude: -4.90857; geodeticDatum: WGS84; **Event:** eventID: 1; samplingProtocol: Sweeping; eventTime: Night
- **Type status:** Other material. **Occurrence:** individualCount: 3; sex: female; **Location:** locationID: P4; continent: Europe; country: Spain; countryCode: ES; stateProvince: Castilla y León; county: León; locality: El Canto; verbatimElevation: 943.48; decimalLatitude: 43.17227; decimalLongitude: -4.90857; geodeticDatum: WGS84; **Event:** eventID: 1; samplingProtocol: Sweeping; eventTime: Night
- **Type status:** Other material. **Occurrence:** individualCount: 3; sex: female; **Location:** locationID: P4; continent: Europe; country: Spain; countryCode: ES; stateProvince: Castilla y León; county: León; locality: El Canto; verbatimElevation: 943.48; decimalLatitude: 43.17227; decimalLongitude: -4.90857; geodeticDatum: WGS84; **Event:** eventID: 1; samplingProtocol: Sweeping; eventTime: Night
- **Type status:** Other material. **Occurrence:** individualCount: 1; sex: female; **Location:** locationID: P4; continent: Europe; country: Spain; countryCode: ES; stateProvince: Castilla y León; county: León; locality: El Canto; verbatimElevation: 943.48; decimalLatitude: 43.17227; decimalLongitude: -4.90857; geodeticDatum: WGS84; **Event:** eventID: 2; samplingProtocol: Sweeping; eventTime: Day
- **Type status:** Other material. **Occurrence:** individualCount: 2; sex: female; **Location:** locationID: S1; continent: Europe; country: Spain; countryCode: ES; stateProvince: Andalucía; county: Granada; locality: Soportujar; verbatimElevation: 1786.57; decimalLatitude: 36.96151; decimalLongitude: -3.41881; geodeticDatum: WGS84; **Event:** eventID: 1; samplingProtocol: Aerial; eventTime: Night
- **Type status:** Other material. **Occurrence:** individualCount: 1; sex: female; **Location:** locationID: S1; continent: Europe; country: Spain; countryCode: ES; stateProvince: Andalucía; county: Granada; locality: Soportujar; verbatimElevation: 1786.57; decimalLatitude: 36.96151; decimalLongitude: -3.41881; geodeticDatum: WGS84; **Event:** eventID: 4; samplingProtocol: Aerial; eventTime: Night
- **Type status:** Other material. **Occurrence:** individualCount: 1; sex: male; **Location:** locationID: S1; continent: Europe; country: Spain; countryCode: ES; stateProvince: Andalucía; county: Granada; locality: Soportujar; verbatimElevation: 1786.57; decimalLatitude: 36.96151; decimalLongitude: -3.41881; geodeticDatum: WGS84; **Event:** eventID: 1; samplingProtocol: Sweeping; eventTime: Day
- **Type status:** Other material. **Occurrence:** individualCount: 1; sex: female; **Location:** locationID: S1; continent: Europe; country: Spain; countryCode: ES; stateProvince: Andalucía; county: Granada; locality: Soportujar; verbatimElevation: 1786.57; decimalLatitude: 36.96151; decimalLongitude: -3.41881; geodeticDatum: WGS84; **Event:** eventID: 1; samplingProtocol: Sweeping; eventTime: Night
- **Type status:** Other material. **Occurrence:** individualCount: 1; sex: female; **Location:** locationID: S2; continent: Europe; country: Spain; countryCode: ES; stateProvince: Andalucía; county: Granada; locality: Camarate; verbatimElevation: 1713.96; decimalLatitude: 37.18377; decimalLongitude: -3.26282; geodeticDatum: WGS84; **Event:** eventID: 1; samplingProtocol: Aerial; eventTime: Night
- **Type status:** Other material. **Occurrence:** individualCount: 1; sex: male; **Location:** locationID: S2; continent: Europe; country: Spain; countryCode: ES; stateProvince: Andalucía; county: Granada; locality: Camarate; verbatimElevation: 1713.96; decimalLatitude: 37.18377; decimalLongitude: -3.26282; geodeticDatum: WGS84; **Event:** eventID: 2; samplingProtocol: Aerial; eventTime: Night
- **Type status:** Other material. **Occurrence:** individualCount: 2; sex: female; **Location:** locationID: S2; continent: Europe; country: Spain; countryCode: ES; stateProvince: Andalucía; county: Granada; locality: Camarate; verbatimElevation: 1713.96; decimalLatitude: 37.18377; decimalLongitude: -3.26282; geodeticDatum: WGS84; **Event:** eventID: 2; samplingProtocol: Aerial; eventTime: Night
- **Type status:** Other material. **Occurrence:** individualCount: 1; sex: female; **Location:** locationID: S2; continent: Europe; country: Spain; countryCode: ES; stateProvince: Andalucía; county: Granada; locality: Camarate; verbatimElevation: 1713.96; decimalLatitude: 37.18377; decimalLongitude: -3.26282; geodeticDatum: WGS84; **Event:** eventID: 3; samplingProtocol: Aerial; eventTime: Night
- **Type status:** Other material. **Occurrence:** individualCount: 1; sex: female; **Location:** locationID: S2; continent: Europe; country: Spain; countryCode: ES; stateProvince: Andalucía; county: Granada; locality: Camarate; verbatimElevation: 1713.96; decimalLatitude: 37.18377; decimalLongitude: -3.26282; geodeticDatum: WGS84; **Event:** eventID: 1; samplingProtocol: Sweeping; eventTime: Night

##### Distribution

Europe to Azerbaijan

#### Zygiella montana

(C. L. Koch, 1834)

##### Materials

- **Type status:** Other material. **Occurrence:** individualCount: 2; sex: female; **Location:** locationID: P1; continent: Europe; country: Spain; countryCode: ES; stateProvince: Castilla y León; county: León; locality: Monte Robledo; verbatimElevation: 1071.58; decimalLatitude: 43.1445; decimalLongitude: -4.92675; geodeticDatum: WGS84; **Event:** eventID: 1; samplingProtocol: Aerial; eventTime: Night
- **Type status:** Other material. **Occurrence:** individualCount: 1; sex: female; **Location:** locationID: P3; continent: Europe; country: Spain; countryCode: ES; stateProvince: Castilla y León; county: León; locality: Las Arroyas; verbatimElevation: 1097.1; decimalLatitude: 43.14351; decimalLongitude: -4.94878; geodeticDatum: WGS84; **Event:** eventID: 1; samplingProtocol: Aerial; eventTime: Night

##### Distribution

Palearctic

#### Clubionidae

Wagner, 1887

#### Clubiona brevipes

Blackwall, 1841

##### Materials

- **Type status:** Other material. **Occurrence:** individualCount: 1; sex: female; **Location:** locationID: A1; continent: Europe; country: Spain; countryCode: ES; stateProvince: Catalonia; county: Lleida; locality: Sola de Boi; verbatimElevation: 1759.8; decimalLatitude: 42.54958; decimalLongitude: 0.87254; geodeticDatum: WGS84; **Event:** eventID: 1; samplingProtocol: Beating; eventTime: Night
- **Type status:** Other material. **Occurrence:** individualCount: 1; sex: male; **Location:** locationID: A1; continent: Europe; country: Spain; countryCode: ES; stateProvince: Catalonia; county: Lleida; locality: Sola de Boi; verbatimElevation: 1759.8; decimalLatitude: 42.54958; decimalLongitude: 0.87254; geodeticDatum: WGS84; **Event:** eventID: 2; samplingProtocol: Beating; eventTime: Day
- **Type status:** Other material. **Occurrence:** individualCount: 1; sex: male; **Location:** locationID: A2; continent: Europe; country: Spain; countryCode: ES; stateProvince: Catalonia; county: Lleida; locality: Sola de Boi; verbatimElevation: 1738.7; decimalLatitude: 42.54913; decimalLongitude: 0.87137; geodeticDatum: WGS84; **Event:** eventID: 1; samplingProtocol: Beating; eventTime: Night
- **Type status:** Other material. **Occurrence:** individualCount: 2; sex: male; **Location:** locationID: A2; continent: Europe; country: Spain; countryCode: ES; stateProvince: Catalonia; county: Lleida; locality: Sola de Boi; verbatimElevation: 1738.7; decimalLatitude: 42.54913; decimalLongitude: 0.87137; geodeticDatum: WGS84; **Event:** eventID: 1; samplingProtocol: Beating; eventTime: Night
- **Type status:** Other material. **Occurrence:** individualCount: 1; sex: female; **Location:** locationID: A2; continent: Europe; country: Spain; countryCode: ES; stateProvince: Catalonia; county: Lleida; locality: Sola de Boi; verbatimElevation: 1738.7; decimalLatitude: 42.54913; decimalLongitude: 0.87137; geodeticDatum: WGS84; **Event:** eventID: 1; samplingProtocol: Beating; eventTime: Night
- **Type status:** Other material. **Occurrence:** individualCount: 1; sex: female; **Location:** locationID: A2; continent: Europe; country: Spain; countryCode: ES; stateProvince: Catalonia; county: Lleida; locality: Sola de Boi; verbatimElevation: 1738.7; decimalLatitude: 42.54913; decimalLongitude: 0.87137; geodeticDatum: WGS84; **Event:** eventID: 2; samplingProtocol: Beating; eventTime: Day
- **Type status:** Other material. **Occurrence:** individualCount: 1; sex: female; **Location:** locationID: A2; continent: Europe; country: Spain; countryCode: ES; stateProvince: Catalonia; county: Lleida; locality: Sola de Boi; verbatimElevation: 1738.7; decimalLatitude: 42.54913; decimalLongitude: 0.87137; geodeticDatum: WGS84; **Event:** eventID: 2; samplingProtocol: Sweeping; eventTime: Day
- **Type status:** Other material. **Occurrence:** individualCount: 2; sex: female; **Location:** locationID: O1; continent: Europe; country: Spain; countryCode: ES; stateProvince: Aragón; county: Huesca; locality: O Furno; verbatimElevation: 1396.73; decimalLatitude: 42.60677; decimalLongitude: 0.13135; geodeticDatum: WGS84; **Event:** eventID: 2; samplingProtocol: Aerial; eventTime: Night
- **Type status:** Other material. **Occurrence:** individualCount: 1; sex: male; **Location:** locationID: O1; continent: Europe; country: Spain; countryCode: ES; stateProvince: Aragón; county: Huesca; locality: O Furno; verbatimElevation: 1396.73; decimalLatitude: 42.60677; decimalLongitude: 0.13135; geodeticDatum: WGS84; **Event:** eventID: 1; samplingProtocol: Beating; eventTime: Day
- **Type status:** Other material. **Occurrence:** individualCount: 1; sex: female; **Location:** locationID: O1; continent: Europe; country: Spain; countryCode: ES; stateProvince: Aragón; county: Huesca; locality: O Furno; verbatimElevation: 1396.73; decimalLatitude: 42.60677; decimalLongitude: 0.13135; geodeticDatum: WGS84; **Event:** eventID: 1; samplingProtocol: Beating; eventTime: Day
- **Type status:** Other material. **Occurrence:** individualCount: 9; sex: female; **Location:** locationID: O1; continent: Europe; country: Spain; countryCode: ES; stateProvince: Aragón; county: Huesca; locality: O Furno; verbatimElevation: 1396.73; decimalLatitude: 42.60677; decimalLongitude: 0.13135; geodeticDatum: WGS84; **Event:** eventID: 1; samplingProtocol: Beating; eventTime: Night
- **Type status:** Other material. **Occurrence:** individualCount: 1; sex: female; **Location:** locationID: O1; continent: Europe; country: Spain; countryCode: ES; stateProvince: Aragón; county: Huesca; locality: O Furno; verbatimElevation: 1396.73; decimalLatitude: 42.60677; decimalLongitude: 0.13135; geodeticDatum: WGS84; **Event:** eventID: 1; samplingProtocol: Sweeping; eventTime: Night
- **Type status:** Other material. **Occurrence:** individualCount: 1; sex: male; **Location:** locationID: O1; continent: Europe; country: Spain; countryCode: ES; stateProvince: Aragón; county: Huesca; locality: O Furno; verbatimElevation: 1396.73; decimalLatitude: 42.60677; decimalLongitude: 0.13135; geodeticDatum: WGS84; **Event:** eventID: 2; samplingProtocol: Sweeping; eventTime: Day
- **Type status:** Other material. **Occurrence:** individualCount: 1; sex: female; **Location:** locationID: O2; continent: Europe; country: Spain; countryCode: ES; stateProvince: Aragón; county: Huesca; locality: Rebilla; verbatimElevation: 1158.13; decimalLatitude: 42.59427; decimalLongitude: 0.1529; geodeticDatum: WGS84; **Event:** eventID: 2; samplingProtocol: Aerial; eventTime: Night
- **Type status:** Other material. **Occurrence:** individualCount: 4; sex: male; **Location:** locationID: O2; continent: Europe; country: Spain; countryCode: ES; stateProvince: Aragón; county: Huesca; locality: Rebilla; verbatimElevation: 1158.13; decimalLatitude: 42.59427; decimalLongitude: 0.1529; geodeticDatum: WGS84; **Event:** eventID: 1; samplingProtocol: Beating; eventTime: Day
- **Type status:** Other material. **Occurrence:** individualCount: 4; sex: female; **Location:** locationID: O2; continent: Europe; country: Spain; countryCode: ES; stateProvince: Aragón; county: Huesca; locality: Rebilla; verbatimElevation: 1158.13; decimalLatitude: 42.59427; decimalLongitude: 0.1529; geodeticDatum: WGS84; **Event:** eventID: 1; samplingProtocol: Beating; eventTime: Day
- **Type status:** Other material. **Occurrence:** individualCount: 2; sex: male; **Location:** locationID: O2; continent: Europe; country: Spain; countryCode: ES; stateProvince: Aragón; county: Huesca; locality: Rebilla; verbatimElevation: 1158.13; decimalLatitude: 42.59427; decimalLongitude: 0.1529; geodeticDatum: WGS84; **Event:** eventID: 1; samplingProtocol: Beating; eventTime: Night
- **Type status:** Other material. **Occurrence:** individualCount: 1; sex: female; **Location:** locationID: O2; continent: Europe; country: Spain; countryCode: ES; stateProvince: Aragón; county: Huesca; locality: Rebilla; verbatimElevation: 1158.13; decimalLatitude: 42.59427; decimalLongitude: 0.1529; geodeticDatum: WGS84; **Event:** eventID: 1; samplingProtocol: Beating; eventTime: Night
- **Type status:** Other material. **Occurrence:** individualCount: 2; sex: male; **Location:** locationID: O2; continent: Europe; country: Spain; countryCode: ES; stateProvince: Aragón; county: Huesca; locality: Rebilla; verbatimElevation: 1158.13; decimalLatitude: 42.59427; decimalLongitude: 0.1529; geodeticDatum: WGS84; **Event:** eventID: 2; samplingProtocol: Beating; eventTime: Day
- **Type status:** Other material. **Occurrence:** individualCount: 3; sex: female; **Location:** locationID: O2; continent: Europe; country: Spain; countryCode: ES; stateProvince: Aragón; county: Huesca; locality: Rebilla; verbatimElevation: 1158.13; decimalLatitude: 42.59427; decimalLongitude: 0.1529; geodeticDatum: WGS84; **Event:** eventID: 2; samplingProtocol: Beating; eventTime: Day
- **Type status:** Other material. **Occurrence:** individualCount: 1; sex: male; **Location:** locationID: O2; continent: Europe; country: Spain; countryCode: ES; stateProvince: Aragón; county: Huesca; locality: Rebilla; verbatimElevation: 1158.13; decimalLatitude: 42.59427; decimalLongitude: 0.1529; geodeticDatum: WGS84; **Event:** eventID: 1; samplingProtocol: Sweeping; eventTime: Day
- **Type status:** Other material. **Occurrence:** individualCount: 1; sex: male; **Location:** locationID: P2; continent: Europe; country: Spain; countryCode: ES; stateProvince: Castilla y León; county: León; locality: Joyoguelas; verbatimElevation: 763.98; decimalLatitude: 43.17771; decimalLongitude: -4.90579; geodeticDatum: WGS84; **Event:** eventID: 1; samplingProtocol: Beating; eventTime: Day
- **Type status:** Other material. **Occurrence:** individualCount: 1; sex: female; **Location:** locationID: P2; continent: Europe; country: Spain; countryCode: ES; stateProvince: Castilla y León; county: León; locality: Joyoguelas; verbatimElevation: 763.98; decimalLatitude: 43.17771; decimalLongitude: -4.90579; geodeticDatum: WGS84; **Event:** eventID: 1; samplingProtocol: Beating; eventTime: Night
- **Type status:** Other material. **Occurrence:** individualCount: 1; sex: female; **Location:** locationID: P3; continent: Europe; country: Spain; countryCode: ES; stateProvince: Castilla y León; county: León; locality: Las Arroyas; verbatimElevation: 1097.1; decimalLatitude: 43.14351; decimalLongitude: -4.94878; geodeticDatum: WGS84; **Event:** eventID: 1; samplingProtocol: Sweeping; eventTime: Night
- **Type status:** Other material. **Occurrence:** individualCount: 1; sex: female; **Location:** locationID: P4; continent: Europe; country: Spain; countryCode: ES; stateProvince: Castilla y León; county: León; locality: El Canto; verbatimElevation: 943.48; decimalLatitude: 43.17227; decimalLongitude: -4.90857; geodeticDatum: WGS84; **Event:** eventID: 1; samplingProtocol: Aerial; eventTime: Night
- **Type status:** Other material. **Occurrence:** individualCount: 1; sex: female; **Location:** locationID: P4; continent: Europe; country: Spain; countryCode: ES; stateProvince: Castilla y León; county: León; locality: El Canto; verbatimElevation: 943.48; decimalLatitude: 43.17227; decimalLongitude: -4.90857; geodeticDatum: WGS84; **Event:** eventID: 2; samplingProtocol: Beating; eventTime: Day

##### Distribution

Palearctic

#### Clubiona comta

C. L. Koch, 1839

##### Materials

- **Type status:** Other material. **Occurrence:** individualCount: 1; sex: female; **Location:** locationID: A1; continent: Europe; country: Spain; countryCode: ES; stateProvince: Catalonia; county: Lleida; locality: Sola de Boi; verbatimElevation: 1759.8; decimalLatitude: 42.54958; decimalLongitude: 0.87254; geodeticDatum: WGS84; **Event:** eventID: 1; samplingProtocol: Beating; eventTime: Night
- **Type status:** Other material. **Occurrence:** individualCount: 1; sex: female; **Location:** locationID: A1; continent: Europe; country: Spain; countryCode: ES; stateProvince: Catalonia; county: Lleida; locality: Sola de Boi; verbatimElevation: 1759.8; decimalLatitude: 42.54958; decimalLongitude: 0.87254; geodeticDatum: WGS84; **Event:** eventID: 1; samplingProtocol: Beating; eventTime: Night
- **Type status:** Other material. **Occurrence:** individualCount: 5; sex: female; **Location:** locationID: A2; continent: Europe; country: Spain; countryCode: ES; stateProvince: Catalonia; county: Lleida; locality: Sola de Boi; verbatimElevation: 1738.7; decimalLatitude: 42.54913; decimalLongitude: 0.87137; geodeticDatum: WGS84; **Event:** eventID: 1; samplingProtocol: Beating; eventTime: Day
- **Type status:** Other material. **Occurrence:** individualCount: 1; sex: male; **Location:** locationID: A2; continent: Europe; country: Spain; countryCode: ES; stateProvince: Catalonia; county: Lleida; locality: Sola de Boi; verbatimElevation: 1738.7; decimalLatitude: 42.54913; decimalLongitude: 0.87137; geodeticDatum: WGS84; **Event:** eventID: 1; samplingProtocol: Beating; eventTime: Night
- **Type status:** Other material. **Occurrence:** individualCount: 1; sex: female; **Location:** locationID: A2; continent: Europe; country: Spain; countryCode: ES; stateProvince: Catalonia; county: Lleida; locality: Sola de Boi; verbatimElevation: 1738.7; decimalLatitude: 42.54913; decimalLongitude: 0.87137; geodeticDatum: WGS84; **Event:** eventID: 1; samplingProtocol: Beating; eventTime: Night
- **Type status:** Other material. **Occurrence:** individualCount: 3; sex: female; **Location:** locationID: A2; continent: Europe; country: Spain; countryCode: ES; stateProvince: Catalonia; county: Lleida; locality: Sola de Boi; verbatimElevation: 1738.7; decimalLatitude: 42.54913; decimalLongitude: 0.87137; geodeticDatum: WGS84; **Event:** eventID: 2; samplingProtocol: Beating; eventTime: Day
- **Type status:** Other material. **Occurrence:** individualCount: 1; sex: male; **Location:** locationID: A2; continent: Europe; country: Spain; countryCode: ES; stateProvince: Catalonia; county: Lleida; locality: Sola de Boi; verbatimElevation: 1738.7; decimalLatitude: 42.54913; decimalLongitude: 0.87137; geodeticDatum: WGS84; **Event:** eventID: 1; samplingProtocol: Sweeping; eventTime: Day
- **Type status:** Other material. **Occurrence:** individualCount: 3; sex: female; **Location:** locationID: A2; continent: Europe; country: Spain; countryCode: ES; stateProvince: Catalonia; county: Lleida; locality: Sola de Boi; verbatimElevation: 1738.7; decimalLatitude: 42.54913; decimalLongitude: 0.87137; geodeticDatum: WGS84; **Event:** eventID: 1; samplingProtocol: Sweeping; eventTime: Day
- **Type status:** Other material. **Occurrence:** individualCount: 1; sex: male; **Location:** locationID: A2; continent: Europe; country: Spain; countryCode: ES; stateProvince: Catalonia; county: Lleida; locality: Sola de Boi; verbatimElevation: 1738.7; decimalLatitude: 42.54913; decimalLongitude: 0.87137; geodeticDatum: WGS84; **Event:** eventID: 1; samplingProtocol: Sweeping; eventTime: Night
- **Type status:** Other material. **Occurrence:** individualCount: 3; sex: female; **Location:** locationID: A2; continent: Europe; country: Spain; countryCode: ES; stateProvince: Catalonia; county: Lleida; locality: Sola de Boi; verbatimElevation: 1738.7; decimalLatitude: 42.54913; decimalLongitude: 0.87137; geodeticDatum: WGS84; **Event:** eventID: 1; samplingProtocol: Sweeping; eventTime: Night
- **Type status:** Other material. **Occurrence:** individualCount: 2; sex: female; **Location:** locationID: A2; continent: Europe; country: Spain; countryCode: ES; stateProvince: Catalonia; county: Lleida; locality: Sola de Boi; verbatimElevation: 1738.7; decimalLatitude: 42.54913; decimalLongitude: 0.87137; geodeticDatum: WGS84; **Event:** eventID: 2; samplingProtocol: Sweeping; eventTime: Day
- **Type status:** Other material. **Occurrence:** individualCount: 1; sex: female; **Location:** locationID: O1; continent: Europe; country: Spain; countryCode: ES; stateProvince: Aragón; county: Huesca; locality: O Furno; verbatimElevation: 1396.73; decimalLatitude: 42.60677; decimalLongitude: 0.13135; geodeticDatum: WGS84; **Event:** eventID: 2; samplingProtocol: Aerial; eventTime: Night
- **Type status:** Other material. **Occurrence:** individualCount: 1; sex: female; **Location:** locationID: O1; continent: Europe; country: Spain; countryCode: ES; stateProvince: Aragón; county: Huesca; locality: O Furno; verbatimElevation: 1396.73; decimalLatitude: 42.60677; decimalLongitude: 0.13135; geodeticDatum: WGS84; **Event:** eventID: A; samplingProtocol: Pitfall
- **Type status:** Other material. **Occurrence:** individualCount: 1; sex: female; **Location:** locationID: O1; continent: Europe; country: Spain; countryCode: ES; stateProvince: Aragón; county: Huesca; locality: O Furno; verbatimElevation: 1396.73; decimalLatitude: 42.60677; decimalLongitude: 0.13135; geodeticDatum: WGS84; **Event:** eventID: E; samplingProtocol: Pitfall
- **Type status:** Other material. **Occurrence:** individualCount: 1; sex: female; **Location:** locationID: O1; continent: Europe; country: Spain; countryCode: ES; stateProvince: Aragón; county: Huesca; locality: O Furno; verbatimElevation: 1396.73; decimalLatitude: 42.60677; decimalLongitude: 0.13135; geodeticDatum: WGS84; **Event:** eventID: H; samplingProtocol: Pitfall
- **Type status:** Other material. **Occurrence:** individualCount: 1; sex: female; **Location:** locationID: O1; continent: Europe; country: Spain; countryCode: ES; stateProvince: Aragón; county: Huesca; locality: O Furno; verbatimElevation: 1396.73; decimalLatitude: 42.60677; decimalLongitude: 0.13135; geodeticDatum: WGS84; **Event:** eventID: I; samplingProtocol: Pitfall
- **Type status:** Other material. **Occurrence:** individualCount: 1; sex: male; **Location:** locationID: O1; continent: Europe; country: Spain; countryCode: ES; stateProvince: Aragón; county: Huesca; locality: O Furno; verbatimElevation: 1396.73; decimalLatitude: 42.60677; decimalLongitude: 0.13135; geodeticDatum: WGS84; **Event:** eventID: K; samplingProtocol: Pitfall
- **Type status:** Other material. **Occurrence:** individualCount: 1; sex: male; **Location:** locationID: O1; continent: Europe; country: Spain; countryCode: ES; stateProvince: Aragón; county: Huesca; locality: O Furno; verbatimElevation: 1396.73; decimalLatitude: 42.60677; decimalLongitude: 0.13135; geodeticDatum: WGS84; **Event:** eventID: 1; samplingProtocol: Sweeping; eventTime: Night
- **Type status:** Other material. **Occurrence:** individualCount: 1; sex: female; **Location:** locationID: O1; continent: Europe; country: Spain; countryCode: ES; stateProvince: Aragón; county: Huesca; locality: O Furno; verbatimElevation: 1396.73; decimalLatitude: 42.60677; decimalLongitude: 0.13135; geodeticDatum: WGS84; **Event:** eventID: 1; samplingProtocol: Sweeping; eventTime: Night
- **Type status:** Other material. **Occurrence:** individualCount: 1; sex: female; **Location:** locationID: P1; continent: Europe; country: Spain; countryCode: ES; stateProvince: Castilla y León; county: León; locality: Monte Robledo; verbatimElevation: 1071.58; decimalLatitude: 43.1445; decimalLongitude: -4.92675; geodeticDatum: WGS84; **Event:** eventID: 1; samplingProtocol: Beating; eventTime: Day
- **Type status:** Other material. **Occurrence:** individualCount: 3; sex: female; **Location:** locationID: P1; continent: Europe; country: Spain; countryCode: ES; stateProvince: Castilla y León; county: León; locality: Monte Robledo; verbatimElevation: 1071.58; decimalLatitude: 43.1445; decimalLongitude: -4.92675; geodeticDatum: WGS84; **Event:** eventID: 1; samplingProtocol: Beating; eventTime: Night
- **Type status:** Other material. **Occurrence:** individualCount: 1; sex: female; **Location:** locationID: P1; continent: Europe; country: Spain; countryCode: ES; stateProvince: Castilla y León; county: León; locality: Monte Robledo; verbatimElevation: 1071.58; decimalLatitude: 43.1445; decimalLongitude: -4.92675; geodeticDatum: WGS84; **Event:** eventID: 1; samplingProtocol: Sweeping; eventTime: Night
- **Type status:** Other material. **Occurrence:** individualCount: 1; sex: female; **Location:** locationID: P1; continent: Europe; country: Spain; countryCode: ES; stateProvince: Castilla y León; county: León; locality: Monte Robledo; verbatimElevation: 1071.58; decimalLatitude: 43.1445; decimalLongitude: -4.92675; geodeticDatum: WGS84; **Event:** eventID: 1; samplingProtocol: Sweeping; eventTime: Night
- **Type status:** Other material. **Occurrence:** individualCount: 2; sex: male; **Location:** locationID: P2; continent: Europe; country: Spain; countryCode: ES; stateProvince: Castilla y León; county: León; locality: Joyoguelas; verbatimElevation: 763.98; decimalLatitude: 43.17771; decimalLongitude: -4.90579; geodeticDatum: WGS84; **Event:** eventID: 2; samplingProtocol: Aerial; eventTime: Night
- **Type status:** Other material. **Occurrence:** individualCount: 2; sex: female; **Location:** locationID: P2; continent: Europe; country: Spain; countryCode: ES; stateProvince: Castilla y León; county: León; locality: Joyoguelas; verbatimElevation: 763.98; decimalLatitude: 43.17771; decimalLongitude: -4.90579; geodeticDatum: WGS84; **Event:** eventID: 2; samplingProtocol: Aerial; eventTime: Night
- **Type status:** Other material. **Occurrence:** individualCount: 8; sex: female; **Location:** locationID: P2; continent: Europe; country: Spain; countryCode: ES; stateProvince: Castilla y León; county: León; locality: Joyoguelas; verbatimElevation: 763.98; decimalLatitude: 43.17771; decimalLongitude: -4.90579; geodeticDatum: WGS84; **Event:** eventID: 1; samplingProtocol: Beating; eventTime: Day
- **Type status:** Other material. **Occurrence:** individualCount: 1; sex: female; **Location:** locationID: P2; continent: Europe; country: Spain; countryCode: ES; stateProvince: Castilla y León; county: León; locality: Joyoguelas; verbatimElevation: 763.98; decimalLatitude: 43.17771; decimalLongitude: -4.90579; geodeticDatum: WGS84; **Event:** eventID: 1; samplingProtocol: Beating; eventTime: Night
- **Type status:** Other material. **Occurrence:** individualCount: 1; sex: female; **Location:** locationID: P2; continent: Europe; country: Spain; countryCode: ES; stateProvince: Castilla y León; county: León; locality: Joyoguelas; verbatimElevation: 763.98; decimalLatitude: 43.17771; decimalLongitude: -4.90579; geodeticDatum: WGS84; **Event:** eventID: I; samplingProtocol: Pitfall
- **Type status:** Other material. **Occurrence:** individualCount: 1; sex: male; **Location:** locationID: P2; continent: Europe; country: Spain; countryCode: ES; stateProvince: Castilla y León; county: León; locality: Joyoguelas; verbatimElevation: 763.98; decimalLatitude: 43.17771; decimalLongitude: -4.90579; geodeticDatum: WGS84; **Event:** samplingProtocol: Sweeping; eventTime: Night
- **Type status:** Other material. **Occurrence:** individualCount: 1; sex: female; **Location:** locationID: P3; continent: Europe; country: Spain; countryCode: ES; stateProvince: Castilla y León; county: León; locality: Las Arroyas; verbatimElevation: 1097.1; decimalLatitude: 43.14351; decimalLongitude: -4.94878; geodeticDatum: WGS84; **Event:** eventID: 2; samplingProtocol: Aerial; eventTime: Night
- **Type status:** Other material. **Occurrence:** individualCount: 1; sex: male; **Location:** locationID: P4; continent: Europe; country: Spain; countryCode: ES; stateProvince: Castilla y León; county: León; locality: El Canto; verbatimElevation: 943.48; decimalLatitude: 43.17227; decimalLongitude: -4.90857; geodeticDatum: WGS84; **Event:** eventID: 1; samplingProtocol: Aerial; eventTime: Night
- **Type status:** Other material. **Occurrence:** individualCount: 1; sex: female; **Location:** locationID: P4; continent: Europe; country: Spain; countryCode: ES; stateProvince: Castilla y León; county: León; locality: El Canto; verbatimElevation: 943.48; decimalLatitude: 43.17227; decimalLongitude: -4.90857; geodeticDatum: WGS84; **Event:** eventID: 1; samplingProtocol: Aerial; eventTime: Night
- **Type status:** Other material. **Occurrence:** individualCount: 1; sex: male; **Location:** locationID: P4; continent: Europe; country: Spain; countryCode: ES; stateProvince: Castilla y León; county: León; locality: El Canto; verbatimElevation: 943.48; decimalLatitude: 43.17227; decimalLongitude: -4.90857; geodeticDatum: WGS84; **Event:** eventID: 2; samplingProtocol: Aerial; eventTime: Night
- **Type status:** Other material. **Occurrence:** individualCount: 1; sex: female; **Location:** locationID: P4; continent: Europe; country: Spain; countryCode: ES; stateProvince: Castilla y León; county: León; locality: El Canto; verbatimElevation: 943.48; decimalLatitude: 43.17227; decimalLongitude: -4.90857; geodeticDatum: WGS84; **Event:** eventID: 2; samplingProtocol: Aerial; eventTime: Night
- **Type status:** Other material. **Occurrence:** individualCount: 1; sex: female; **Location:** locationID: P4; continent: Europe; country: Spain; countryCode: ES; stateProvince: Castilla y León; county: León; locality: El Canto; verbatimElevation: 943.48; decimalLatitude: 43.17227; decimalLongitude: -4.90857; geodeticDatum: WGS84; **Event:** eventID: 2; samplingProtocol: Beating; eventTime: Day
- **Type status:** Other material. **Occurrence:** individualCount: 4; sex: female; **Location:** locationID: P4; continent: Europe; country: Spain; countryCode: ES; stateProvince: Castilla y León; county: León; locality: El Canto; verbatimElevation: 943.48; decimalLatitude: 43.17227; decimalLongitude: -4.90857; geodeticDatum: WGS84; **Event:** eventID: 1; samplingProtocol: Beating; eventTime: Night
- **Type status:** Other material. **Occurrence:** individualCount: 4; sex: female; **Location:** locationID: P4; continent: Europe; country: Spain; countryCode: ES; stateProvince: Castilla y León; county: León; locality: El Canto; verbatimElevation: 943.48; decimalLatitude: 43.17227; decimalLongitude: -4.90857; geodeticDatum: WGS84; **Event:** eventID: 1; samplingProtocol: Beating; eventTime: Night

##### Distribution

Europe, Russia, North Africa

#### Clubiona corticalis

(Walckenaer, 1802)

##### Materials

- **Type status:** Other material. **Occurrence:** individualCount: 1; sex: female; **Location:** locationID: P3; continent: Europe; country: Spain; countryCode: ES; stateProvince: Castilla y León; county: León; locality: Las Arroyas; verbatimElevation: 1097.1; decimalLatitude: 43.14351; decimalLongitude: -4.94878; geodeticDatum: WGS84; **Event:** eventID: 1; samplingProtocol: Aerial; eventTime: Night

##### Distribution

Europe to Central Asia

#### Clubiona diniensis

Simon, 1878

##### Materials

- **Type status:** Other material. **Occurrence:** individualCount: 1; sex: male; **Location:** locationID: C1; continent: Europe; country: Spain; countryCode: ES; stateProvince: Castilla-La Mancha; county: Ciudad Real; locality: Valle Brezoso; verbatimElevation: 756.56; decimalLatitude: 39.35663; decimalLongitude: -4.35912; geodeticDatum: WGS84; **Event:** eventID: 3; samplingProtocol: Aerial; eventTime: Night
- **Type status:** Other material. **Occurrence:** individualCount: 1; sex: female; **Location:** locationID: C1; continent: Europe; country: Spain; countryCode: ES; stateProvince: Castilla-La Mancha; county: Ciudad Real; locality: Valle Brezoso; verbatimElevation: 756.56; decimalLatitude: 39.35663; decimalLongitude: -4.35912; geodeticDatum: WGS84; **Event:** eventID: 3; samplingProtocol: Aerial; eventTime: Night
- **Type status:** Other material. **Occurrence:** individualCount: 1; sex: female; **Location:** locationID: C2; continent: Europe; country: Spain; countryCode: ES; stateProvince: Castilla-La Mancha; county: Ciudad Real; locality: Valle Brezoso; verbatimElevation: 739.31; decimalLatitude: 39.35159; decimalLongitude: -4.3589; geodeticDatum: WGS84; **Event:** eventID: 1; samplingProtocol: Beating; eventTime: Night
- **Type status:** Other material. **Occurrence:** individualCount: 1; sex: female; **Location:** locationID: C2; continent: Europe; country: Spain; countryCode: ES; stateProvince: Castilla-La Mancha; county: Ciudad Real; locality: Valle Brezoso; verbatimElevation: 739.31; decimalLatitude: 39.35159; decimalLongitude: -4.3589; geodeticDatum: WGS84; **Event:** eventID: 2; samplingProtocol: Beating; eventTime: Night
- **Type status:** Other material. **Occurrence:** individualCount: 1; sex: female; **Location:** locationID: C3; continent: Europe; country: Spain; countryCode: ES; stateProvince: Castilla-La Mancha; county: Ciudad Real; locality: La Quesera; verbatimElevation: 767.55; decimalLatitude: 39.36177; decimalLongitude: -4.41733; geodeticDatum: WGS84; **Event:** eventID: 4; samplingProtocol: Aerial; eventTime: Night
- **Type status:** Other material. **Occurrence:** individualCount: 1; sex: female; **Location:** locationID: M1; continent: Europe; country: Spain; countryCode: ES; stateProvince: Extremadura; county: Cáceres; locality: Peña Falcón; verbatimElevation: 320.6; decimalLatitude: 39.83296; decimalLongitude: -6.0641; geodeticDatum: WGS84; **Event:** eventID: 1; samplingProtocol: Sweeping; eventTime: Night

##### Distribution

Iberian Peninsula, France

#### Clubiona genevensis

L. Koch, 1866

##### Materials

- **Type status:** Other material. **Occurrence:** individualCount: 1; sex: female; **Location:** locationID: S1; continent: Europe; country: Spain; countryCode: ES; stateProvince: Andalucía; county: Granada; locality: Soportujar; verbatimElevation: 1786.57; decimalLatitude: 36.96151; decimalLongitude: -3.41881; geodeticDatum: WGS84; **Event:** eventID: 1; samplingProtocol: Sweeping; eventTime: Night

##### Distribution

Palearctic

#### Clubiona leucaspis

Simon, 1932

##### Materials

- **Type status:** Other material. **Occurrence:** individualCount: 1; sex: female; **Location:** locationID: C3; continent: Europe; country: Spain; countryCode: ES; stateProvince: Castilla-La Mancha; county: Ciudad Real; locality: La Quesera; verbatimElevation: 767.55; decimalLatitude: 39.36177; decimalLongitude: -4.41733; geodeticDatum: WGS84; **Event:** eventID: 1; samplingProtocol: Aerial; eventTime: Night
- **Type status:** Other material. **Occurrence:** individualCount: 1; sex: female; **Location:** locationID: M1; continent: Europe; country: Spain; countryCode: ES; stateProvince: Extremadura; county: Cáceres; locality: Peña Falcón; verbatimElevation: 320.6; decimalLatitude: 39.83296; decimalLongitude: -6.0641; geodeticDatum: WGS84; **Event:** eventID: 2; samplingProtocol: Beating; eventTime: Day

##### Distribution

Europe, Algeria

#### Clubiona neglecta

O. Pickard-Cambridge, 1862

##### Materials

- **Type status:** Other material. **Occurrence:** individualCount: 1; sex: male; **Location:** locationID: P4; continent: Europe; country: Spain; countryCode: ES; stateProvince: Castilla y León; county: León; locality: El Canto; verbatimElevation: 943.48; decimalLatitude: 43.17227; decimalLongitude: -4.90857; geodeticDatum: WGS84; **Event:** eventID: 1; samplingProtocol: Sweeping; eventTime: Night

##### Distribution

Palearctic

#### Clubiona terrestris

Westring, 1851

##### Materials

- **Type status:** Other material. **Occurrence:** individualCount: 1; sex: female; **Location:** locationID: O1; continent: Europe; country: Spain; countryCode: ES; stateProvince: Aragón; county: Huesca; locality: O Furno; verbatimElevation: 1396.73; decimalLatitude: 42.60677; decimalLongitude: 0.13135; geodeticDatum: WGS84; **Event:** eventID: 1; samplingProtocol: Sweeping; eventTime: Night
- **Type status:** Other material. **Occurrence:** individualCount: 2; sex: female; **Location:** locationID: P1; continent: Europe; country: Spain; countryCode: ES; stateProvince: Castilla y León; county: León; locality: Monte Robledo; verbatimElevation: 1071.58; decimalLatitude: 43.1445; decimalLongitude: -4.92675; geodeticDatum: WGS84; **Event:** eventID: 1; samplingProtocol: Beating; eventTime: Day
- **Type status:** Other material. **Occurrence:** individualCount: 1; sex: female; **Location:** locationID: P1; continent: Europe; country: Spain; countryCode: ES; stateProvince: Castilla y León; county: León; locality: Monte Robledo; verbatimElevation: 1071.58; decimalLatitude: 43.1445; decimalLongitude: -4.92675; geodeticDatum: WGS84; **Event:** eventID: D; samplingProtocol: Pitfall
- **Type status:** Other material. **Occurrence:** individualCount: 1; sex: female; **Location:** locationID: P1; continent: Europe; country: Spain; countryCode: ES; stateProvince: Castilla y León; county: León; locality: Monte Robledo; verbatimElevation: 1071.58; decimalLatitude: 43.1445; decimalLongitude: -4.92675; geodeticDatum: WGS84; **Event:** eventID: E; samplingProtocol: Pitfall
- **Type status:** Other material. **Occurrence:** individualCount: 1; sex: male; **Location:** locationID: P1; continent: Europe; country: Spain; countryCode: ES; stateProvince: Castilla y León; county: León; locality: Monte Robledo; verbatimElevation: 1071.58; decimalLatitude: 43.1445; decimalLongitude: -4.92675; geodeticDatum: WGS84; **Event:** eventID: F; samplingProtocol: Pitfall
- **Type status:** Other material. **Occurrence:** individualCount: 1; sex: male; **Location:** locationID: P1; continent: Europe; country: Spain; countryCode: ES; stateProvince: Castilla y León; county: León; locality: Monte Robledo; verbatimElevation: 1071.58; decimalLatitude: 43.1445; decimalLongitude: -4.92675; geodeticDatum: WGS84; **Event:** eventID: J; samplingProtocol: Pitfall
- **Type status:** Other material. **Occurrence:** individualCount: 2; sex: female; **Location:** locationID: P1; continent: Europe; country: Spain; countryCode: ES; stateProvince: Castilla y León; county: León; locality: Monte Robledo; verbatimElevation: 1071.58; decimalLatitude: 43.1445; decimalLongitude: -4.92675; geodeticDatum: WGS84; **Event:** eventID: 1; samplingProtocol: Sweeping; eventTime: Night
- **Type status:** Other material. **Occurrence:** individualCount: 9; sex: female; **Location:** locationID: P1; continent: Europe; country: Spain; countryCode: ES; stateProvince: Castilla y León; county: León; locality: Monte Robledo; verbatimElevation: 1071.58; decimalLatitude: 43.1445; decimalLongitude: -4.92675; geodeticDatum: WGS84; **Event:** eventID: 1; samplingProtocol: Sweeping; eventTime: Night
- **Type status:** Other material. **Occurrence:** individualCount: 1; sex: female; **Location:** locationID: P2; continent: Europe; country: Spain; countryCode: ES; stateProvince: Castilla y León; county: León; locality: Joyoguelas; verbatimElevation: 763.98; decimalLatitude: 43.17771; decimalLongitude: -4.90579; geodeticDatum: WGS84; **Event:** eventID: 2; samplingProtocol: Aerial; eventTime: Night
- **Type status:** Other material. **Occurrence:** individualCount: 1; sex: male; **Location:** locationID: P2; continent: Europe; country: Spain; countryCode: ES; stateProvince: Castilla y León; county: León; locality: Joyoguelas; verbatimElevation: 763.98; decimalLatitude: 43.17771; decimalLongitude: -4.90579; geodeticDatum: WGS84; **Event:** eventID: 1; samplingProtocol: Sweeping; eventTime: Night
- **Type status:** Other material. **Occurrence:** individualCount: 3; sex: female; **Location:** locationID: P2; continent: Europe; country: Spain; countryCode: ES; stateProvince: Castilla y León; county: León; locality: Joyoguelas; verbatimElevation: 763.98; decimalLatitude: 43.17771; decimalLongitude: -4.90579; geodeticDatum: WGS84; **Event:** eventID: 1; samplingProtocol: Sweeping; eventTime: Night
- **Type status:** Other material. **Occurrence:** individualCount: 1; sex: male; **Location:** locationID: P3; continent: Europe; country: Spain; countryCode: ES; stateProvince: Castilla y León; county: León; locality: Las Arroyas; verbatimElevation: 1097.1; decimalLatitude: 43.14351; decimalLongitude: -4.94878; geodeticDatum: WGS84; **Event:** eventID: 1; samplingProtocol: Beating; eventTime: Night
- **Type status:** Other material. **Occurrence:** individualCount: 2; sex: female; **Location:** locationID: P3; continent: Europe; country: Spain; countryCode: ES; stateProvince: Castilla y León; county: León; locality: Las Arroyas; verbatimElevation: 1097.1; decimalLatitude: 43.14351; decimalLongitude: -4.94878; geodeticDatum: WGS84; **Event:** eventID: 1; samplingProtocol: Sweeping; eventTime: Night
- **Type status:** Other material. **Occurrence:** individualCount: 4; sex: female; **Location:** locationID: P3; continent: Europe; country: Spain; countryCode: ES; stateProvince: Castilla y León; county: León; locality: Las Arroyas; verbatimElevation: 1097.1; decimalLatitude: 43.14351; decimalLongitude: -4.94878; geodeticDatum: WGS84; **Event:** eventID: 1; samplingProtocol: Sweeping; eventTime: Night
- **Type status:** Other material. **Occurrence:** individualCount: 1; sex: female; **Location:** locationID: P4; continent: Europe; country: Spain; countryCode: ES; stateProvince: Castilla y León; county: León; locality: El Canto; verbatimElevation: 943.48; decimalLatitude: 43.17227; decimalLongitude: -4.90857; geodeticDatum: WGS84; **Event:** eventID: 1; samplingProtocol: Aerial; eventTime: Night
- **Type status:** Other material. **Occurrence:** individualCount: 1; sex: male; **Location:** locationID: P4; continent: Europe; country: Spain; countryCode: ES; stateProvince: Castilla y León; county: León; locality: El Canto; verbatimElevation: 943.48; decimalLatitude: 43.17227; decimalLongitude: -4.90857; geodeticDatum: WGS84; **Event:** eventID: 1; samplingProtocol: Beating; eventTime: Night
- **Type status:** Other material. **Occurrence:** individualCount: 5; sex: female; **Location:** locationID: P4; continent: Europe; country: Spain; countryCode: ES; stateProvince: Castilla y León; county: León; locality: El Canto; verbatimElevation: 943.48; decimalLatitude: 43.17227; decimalLongitude: -4.90857; geodeticDatum: WGS84; **Event:** eventID: 1; samplingProtocol: Beating; eventTime: Night
- **Type status:** Other material. **Occurrence:** individualCount: 1; sex: female; **Location:** locationID: P4; continent: Europe; country: Spain; countryCode: ES; stateProvince: Castilla y León; county: León; locality: El Canto; verbatimElevation: 943.48; decimalLatitude: 43.17227; decimalLongitude: -4.90857; geodeticDatum: WGS84; **Event:** eventID: A; samplingProtocol: Pitfall
- **Type status:** Other material. **Occurrence:** individualCount: 2; sex: female; **Location:** locationID: P4; continent: Europe; country: Spain; countryCode: ES; stateProvince: Castilla y León; county: León; locality: El Canto; verbatimElevation: 943.48; decimalLatitude: 43.17227; decimalLongitude: -4.90857; geodeticDatum: WGS84; **Event:** eventID: G; samplingProtocol: Pitfall
- **Type status:** Other material. **Occurrence:** individualCount: 1; sex: male; **Location:** locationID: P4; continent: Europe; country: Spain; countryCode: ES; stateProvince: Castilla y León; county: León; locality: El Canto; verbatimElevation: 943.48; decimalLatitude: 43.17227; decimalLongitude: -4.90857; geodeticDatum: WGS84; **Event:** eventID: 1; samplingProtocol: Sweeping; eventTime: Night
- **Type status:** Other material. **Occurrence:** individualCount: 2; sex: female; **Location:** locationID: P4; continent: Europe; country: Spain; countryCode: ES; stateProvince: Castilla y León; county: León; locality: El Canto; verbatimElevation: 943.48; decimalLatitude: 43.17227; decimalLongitude: -4.90857; geodeticDatum: WGS84; **Event:** eventID: 1; samplingProtocol: Sweeping; eventTime: Night
- **Type status:** Other material. **Occurrence:** individualCount: 1; sex: male; **Location:** locationID: P4; continent: Europe; country: Spain; countryCode: ES; stateProvince: Castilla y León; county: León; locality: El Canto; verbatimElevation: 943.48; decimalLatitude: 43.17227; decimalLongitude: -4.90857; geodeticDatum: WGS84; **Event:** eventID: 1; samplingProtocol: Sweeping; eventTime: Night
- **Type status:** Other material. **Occurrence:** individualCount: 3; sex: female; **Location:** locationID: P4; continent: Europe; country: Spain; countryCode: ES; stateProvince: Castilla y León; county: León; locality: El Canto; verbatimElevation: 943.48; decimalLatitude: 43.17227; decimalLongitude: -4.90857; geodeticDatum: WGS84; **Event:** eventID: 1; samplingProtocol: Sweeping; eventTime: Night

##### Distribution

Europe

#### Dictynidae

O. Pickard-Cambridge, 1871

#### Brigittea civica

(Lucas, 1850)

##### Materials

- **Type status:** Other material. **Occurrence:** individualCount: 1; sex: female; **Location:** locationID: C1; continent: Europe; country: Spain; countryCode: ES; stateProvince: Castilla-La Mancha; county: Ciudad Real; locality: Valle Brezoso; verbatimElevation: 756.56; decimalLatitude: 39.35663; decimalLongitude: -4.35912; geodeticDatum: WGS84; **Event:** eventID: 3; samplingProtocol: Aerial; eventTime: Night
- **Type status:** Other material. **Occurrence:** individualCount: 2; sex: female; **Location:** locationID: C1; continent: Europe; country: Spain; countryCode: ES; stateProvince: Castilla-La Mancha; county: Ciudad Real; locality: Valle Brezoso; verbatimElevation: 756.56; decimalLatitude: 39.35663; decimalLongitude: -4.35912; geodeticDatum: WGS84; **Event:** eventID: 1; samplingProtocol: Beating; eventTime: Night
- **Type status:** Other material. **Occurrence:** individualCount: 1; sex: male; **Location:** locationID: C2; continent: Europe; country: Spain; countryCode: ES; stateProvince: Castilla-La Mancha; county: Ciudad Real; locality: Valle Brezoso; verbatimElevation: 739.31; decimalLatitude: 39.35159; decimalLongitude: -4.3589; geodeticDatum: WGS84; **Event:** eventID: 4; samplingProtocol: Aerial; eventTime: Night
- **Type status:** Other material. **Occurrence:** individualCount: 2; sex: female; **Location:** locationID: C2; continent: Europe; country: Spain; countryCode: ES; stateProvince: Castilla-La Mancha; county: Ciudad Real; locality: Valle Brezoso; verbatimElevation: 739.31; decimalLatitude: 39.35159; decimalLongitude: -4.3589; geodeticDatum: WGS84; **Event:** eventID: 1; samplingProtocol: Beating; eventTime: Night
- **Type status:** Other material. **Occurrence:** individualCount: 2; sex: female; **Location:** locationID: C2; continent: Europe; country: Spain; countryCode: ES; stateProvince: Castilla-La Mancha; county: Ciudad Real; locality: Valle Brezoso; verbatimElevation: 739.31; decimalLatitude: 39.35159; decimalLongitude: -4.3589; geodeticDatum: WGS84; **Event:** eventID: 2; samplingProtocol: Beating; eventTime: Night
- **Type status:** Other material. **Occurrence:** individualCount: 1; sex: male; **Location:** locationID: C2; continent: Europe; country: Spain; countryCode: ES; stateProvince: Castilla-La Mancha; county: Ciudad Real; locality: Valle Brezoso; verbatimElevation: 739.31; decimalLatitude: 39.35159; decimalLongitude: -4.3589; geodeticDatum: WGS84; **Event:** eventID: 2; samplingProtocol: Beating; eventTime: Day
- **Type status:** Other material. **Occurrence:** individualCount: 1; sex: female; **Location:** locationID: C3; continent: Europe; country: Spain; countryCode: ES; stateProvince: Castilla-La Mancha; county: Ciudad Real; locality: La Quesera; verbatimElevation: 767.55; decimalLatitude: 39.36177; decimalLongitude: -4.41733; geodeticDatum: WGS84; **Event:** eventID: 2; samplingProtocol: Aerial; eventTime: Night
- **Type status:** Other material. **Occurrence:** individualCount: 2; sex: female; **Location:** locationID: C3; continent: Europe; country: Spain; countryCode: ES; stateProvince: Castilla-La Mancha; county: Ciudad Real; locality: La Quesera; verbatimElevation: 767.55; decimalLatitude: 39.36177; decimalLongitude: -4.41733; geodeticDatum: WGS84; **Event:** eventID: 4; samplingProtocol: Aerial; eventTime: Night
- **Type status:** Other material. **Occurrence:** individualCount: 4; sex: male; **Location:** locationID: C3; continent: Europe; country: Spain; countryCode: ES; stateProvince: Castilla-La Mancha; county: Ciudad Real; locality: La Quesera; verbatimElevation: 767.55; decimalLatitude: 39.36177; decimalLongitude: -4.41733; geodeticDatum: WGS84; **Event:** eventID: 1; samplingProtocol: Beating; eventTime: Day
- **Type status:** Other material. **Occurrence:** individualCount: 2; sex: female; **Location:** locationID: C3; continent: Europe; country: Spain; countryCode: ES; stateProvince: Castilla-La Mancha; county: Ciudad Real; locality: La Quesera; verbatimElevation: 767.55; decimalLatitude: 39.36177; decimalLongitude: -4.41733; geodeticDatum: WGS84; **Event:** eventID: 1; samplingProtocol: Beating; eventTime: Day
- **Type status:** Other material. **Occurrence:** individualCount: 4; sex: male; **Location:** locationID: C3; continent: Europe; country: Spain; countryCode: ES; stateProvince: Castilla-La Mancha; county: Ciudad Real; locality: La Quesera; verbatimElevation: 767.55; decimalLatitude: 39.36177; decimalLongitude: -4.41733; geodeticDatum: WGS84; **Event:** eventID: 2; samplingProtocol: Beating; eventTime: Night
- **Type status:** Other material. **Occurrence:** individualCount: 4; sex: female; **Location:** locationID: C3; continent: Europe; country: Spain; countryCode: ES; stateProvince: Castilla-La Mancha; county: Ciudad Real; locality: La Quesera; verbatimElevation: 767.55; decimalLatitude: 39.36177; decimalLongitude: -4.41733; geodeticDatum: WGS84; **Event:** eventID: 2; samplingProtocol: Beating; eventTime: Night
- **Type status:** Other material. **Occurrence:** individualCount: 1; sex: male; **Location:** locationID: C3; continent: Europe; country: Spain; countryCode: ES; stateProvince: Castilla-La Mancha; county: Ciudad Real; locality: La Quesera; verbatimElevation: 767.55; decimalLatitude: 39.36177; decimalLongitude: -4.41733; geodeticDatum: WGS84; **Event:** eventID: 2; samplingProtocol: Beating; eventTime: Day
- **Type status:** Other material. **Occurrence:** individualCount: 1; sex: female; **Location:** locationID: C3; continent: Europe; country: Spain; countryCode: ES; stateProvince: Castilla-La Mancha; county: Ciudad Real; locality: La Quesera; verbatimElevation: 767.55; decimalLatitude: 39.36177; decimalLongitude: -4.41733; geodeticDatum: WGS84; **Event:** eventID: 2; samplingProtocol: Sweeping; eventTime: Night
- **Type status:** Other material. **Occurrence:** individualCount: 1; sex: female; **Location:** locationID: C4; continent: Europe; country: Spain; countryCode: ES; stateProvince: Castilla-La Mancha; county: Ciudad Real; locality: La Quesera; verbatimElevation: 772.3; decimalLatitude: 39.36337; decimalLongitude: -4.41704; geodeticDatum: WGS84; **Event:** eventID: 1; samplingProtocol: Aerial; eventTime: Night
- **Type status:** Other material. **Occurrence:** individualCount: 3; sex: male; **Location:** locationID: C4; continent: Europe; country: Spain; countryCode: ES; stateProvince: Castilla-La Mancha; county: Ciudad Real; locality: La Quesera; verbatimElevation: 772.3; decimalLatitude: 39.36337; decimalLongitude: -4.41704; geodeticDatum: WGS84; **Event:** eventID: 4; samplingProtocol: Aerial; eventTime: Night
- **Type status:** Other material. **Occurrence:** individualCount: 2; sex: female; **Location:** locationID: C4; continent: Europe; country: Spain; countryCode: ES; stateProvince: Castilla-La Mancha; county: Ciudad Real; locality: La Quesera; verbatimElevation: 772.3; decimalLatitude: 39.36337; decimalLongitude: -4.41704; geodeticDatum: WGS84; **Event:** eventID: 4; samplingProtocol: Aerial; eventTime: Night
- **Type status:** Other material. **Occurrence:** individualCount: 3; sex: male; **Location:** locationID: C4; continent: Europe; country: Spain; countryCode: ES; stateProvince: Castilla-La Mancha; county: Ciudad Real; locality: La Quesera; verbatimElevation: 772.3; decimalLatitude: 39.36337; decimalLongitude: -4.41704; geodeticDatum: WGS84; **Event:** eventID: 1; samplingProtocol: Beating; eventTime: Day
- **Type status:** Other material. **Occurrence:** individualCount: 2; sex: female; **Location:** locationID: C4; continent: Europe; country: Spain; countryCode: ES; stateProvince: Castilla-La Mancha; county: Ciudad Real; locality: La Quesera; verbatimElevation: 772.3; decimalLatitude: 39.36337; decimalLongitude: -4.41704; geodeticDatum: WGS84; **Event:** eventID: 1; samplingProtocol: Beating; eventTime: Day
- **Type status:** Other material. **Occurrence:** individualCount: 1; sex: male; **Location:** locationID: C4; continent: Europe; country: Spain; countryCode: ES; stateProvince: Castilla-La Mancha; county: Ciudad Real; locality: La Quesera; verbatimElevation: 772.3; decimalLatitude: 39.36337; decimalLongitude: -4.41704; geodeticDatum: WGS84; **Event:** eventID: 2; samplingProtocol: Beating; eventTime: Night
- **Type status:** Other material. **Occurrence:** individualCount: 1; sex: female; **Location:** locationID: C4; continent: Europe; country: Spain; countryCode: ES; stateProvince: Castilla-La Mancha; county: Ciudad Real; locality: La Quesera; verbatimElevation: 772.3; decimalLatitude: 39.36337; decimalLongitude: -4.41704; geodeticDatum: WGS84; **Event:** eventID: 2; samplingProtocol: Beating; eventTime: Night
- **Type status:** Other material. **Occurrence:** individualCount: 2; sex: female; **Location:** locationID: C4; continent: Europe; country: Spain; countryCode: ES; stateProvince: Castilla-La Mancha; county: Ciudad Real; locality: La Quesera; verbatimElevation: 772.3; decimalLatitude: 39.36337; decimalLongitude: -4.41704; geodeticDatum: WGS84; **Event:** eventID: 2; samplingProtocol: Beating; eventTime: Day
- **Type status:** Other material. **Occurrence:** individualCount: 1; sex: male; **Location:** locationID: C4; continent: Europe; country: Spain; countryCode: ES; stateProvince: Castilla-La Mancha; county: Ciudad Real; locality: La Quesera; verbatimElevation: 772.3; decimalLatitude: 39.36337; decimalLongitude: -4.41704; geodeticDatum: WGS84; **Event:** eventID: 1; samplingProtocol: Sweeping; eventTime: Night
- **Type status:** Other material. **Occurrence:** individualCount: 2; sex: male; **Location:** locationID: M1; continent: Europe; country: Spain; countryCode: ES; stateProvince: Extremadura; county: Cáceres; locality: Peña Falcón; verbatimElevation: 320.6; decimalLatitude: 39.83296; decimalLongitude: -6.0641; geodeticDatum: WGS84; **Event:** eventID: 2; samplingProtocol: Beating; eventTime: Night
- **Type status:** Other material. **Occurrence:** individualCount: 1; sex: female; **Location:** locationID: M1; continent: Europe; country: Spain; countryCode: ES; stateProvince: Extremadura; county: Cáceres; locality: Peña Falcón; verbatimElevation: 320.6; decimalLatitude: 39.83296; decimalLongitude: -6.0641; geodeticDatum: WGS84; **Event:** eventID: 2; samplingProtocol: Beating; eventTime: Day
- **Type status:** Other material. **Occurrence:** individualCount: 1; sex: male; **Location:** locationID: M1; continent: Europe; country: Spain; countryCode: ES; stateProvince: Extremadura; county: Cáceres; locality: Peña Falcón; verbatimElevation: 320.6; decimalLatitude: 39.83296; decimalLongitude: -6.0641; geodeticDatum: WGS84; **Event:** eventID: 2; samplingProtocol: Sweeping; eventTime: Night
- **Type status:** Other material. **Occurrence:** individualCount: 1; sex: male; **Location:** locationID: M2; continent: Europe; country: Spain; countryCode: ES; stateProvince: Extremadura; county: Cáceres; locality: Fuente del Frances; verbatimElevation: 320.72; decimalLatitude: 39.828; decimalLongitude: -6.03249; geodeticDatum: WGS84; **Event:** eventID: 3; samplingProtocol: Aerial; eventTime: Night
- **Type status:** Other material. **Occurrence:** individualCount: 2; sex: female; **Location:** locationID: M2; continent: Europe; country: Spain; countryCode: ES; stateProvince: Extremadura; county: Cáceres; locality: Fuente del Frances; verbatimElevation: 320.72; decimalLatitude: 39.828; decimalLongitude: -6.03249; geodeticDatum: WGS84; **Event:** eventID: 1; samplingProtocol: Beating; eventTime: Night
- **Type status:** Other material. **Occurrence:** individualCount: 1; sex: male; **Location:** locationID: M2; continent: Europe; country: Spain; countryCode: ES; stateProvince: Extremadura; county: Cáceres; locality: Fuente del Frances; verbatimElevation: 320.72; decimalLatitude: 39.828; decimalLongitude: -6.03249; geodeticDatum: WGS84; **Event:** eventID: 1; samplingProtocol: Beating; eventTime: Night
- **Type status:** Other material. **Occurrence:** individualCount: 1; sex: female; **Location:** locationID: M2; continent: Europe; country: Spain; countryCode: ES; stateProvince: Extremadura; county: Cáceres; locality: Fuente del Frances; verbatimElevation: 320.72; decimalLatitude: 39.828; decimalLongitude: -6.03249; geodeticDatum: WGS84; **Event:** eventID: 1; samplingProtocol: Beating; eventTime: Night
- **Type status:** Other material. **Occurrence:** individualCount: 1; sex: female; **Location:** locationID: M2; continent: Europe; country: Spain; countryCode: ES; stateProvince: Extremadura; county: Cáceres; locality: Fuente del Frances; verbatimElevation: 320.72; decimalLatitude: 39.828; decimalLongitude: -6.03249; geodeticDatum: WGS84; **Event:** eventID: 2; samplingProtocol: Beating; eventTime: Day
- **Type status:** Other material. **Occurrence:** individualCount: 1; sex: male; **Location:** locationID: M2; continent: Europe; country: Spain; countryCode: ES; stateProvince: Extremadura; county: Cáceres; locality: Fuente del Frances; verbatimElevation: 320.72; decimalLatitude: 39.828; decimalLongitude: -6.03249; geodeticDatum: WGS84; **Event:** eventID: 1; samplingProtocol: Sweeping; eventTime: Day
- **Type status:** Other material. **Occurrence:** individualCount: 1; sex: female; **Location:** locationID: M2; continent: Europe; country: Spain; countryCode: ES; stateProvince: Extremadura; county: Cáceres; locality: Fuente del Frances; verbatimElevation: 320.72; decimalLatitude: 39.828; decimalLongitude: -6.03249; geodeticDatum: WGS84; **Event:** eventID: 1; samplingProtocol: Sweeping; eventTime: Night
- **Type status:** Other material. **Occurrence:** individualCount: 1; sex: male; **Location:** locationID: M2; continent: Europe; country: Spain; countryCode: ES; stateProvince: Extremadura; county: Cáceres; locality: Fuente del Frances; verbatimElevation: 320.72; decimalLatitude: 39.828; decimalLongitude: -6.03249; geodeticDatum: WGS84; **Event:** eventID: 2; samplingProtocol: Sweeping; eventTime: Night
- **Type status:** Other material. **Occurrence:** individualCount: 1; sex: female; **Location:** locationID: S2; continent: Europe; country: Spain; countryCode: ES; stateProvince: Andalucía; county: Granada; locality: Camarate; verbatimElevation: 1713.96; decimalLatitude: 37.18377; decimalLongitude: -3.26282; geodeticDatum: WGS84; **Event:** eventID: 3; samplingProtocol: Aerial; eventTime: Night
- **Type status:** Other material. **Occurrence:** individualCount: 1; sex: male; **Location:** locationID: S2; continent: Europe; country: Spain; countryCode: ES; stateProvince: Andalucía; county: Granada; locality: Camarate; verbatimElevation: 1713.96; decimalLatitude: 37.18377; decimalLongitude: -3.26282; geodeticDatum: WGS84; **Event:** eventID: 1; samplingProtocol: Beating; eventTime: Day
- **Type status:** Other material. **Occurrence:** individualCount: 2; sex: female; **Location:** locationID: S2; continent: Europe; country: Spain; countryCode: ES; stateProvince: Andalucía; county: Granada; locality: Camarate; verbatimElevation: 1713.96; decimalLatitude: 37.18377; decimalLongitude: -3.26282; geodeticDatum: WGS84; **Event:** eventID: 1; samplingProtocol: Beating; eventTime: Day
- **Type status:** Other material. **Occurrence:** individualCount: 1; sex: male; **Location:** locationID: S2; continent: Europe; country: Spain; countryCode: ES; stateProvince: Andalucía; county: Granada; locality: Camarate; verbatimElevation: 1713.96; decimalLatitude: 37.18377; decimalLongitude: -3.26282; geodeticDatum: WGS84; **Event:** eventID: 2; samplingProtocol: Beating; eventTime: Day
- **Type status:** Other material. **Occurrence:** individualCount: 1; sex: female; **Location:** locationID: S2; continent: Europe; country: Spain; countryCode: ES; stateProvince: Andalucía; county: Granada; locality: Camarate; verbatimElevation: 1713.96; decimalLatitude: 37.18377; decimalLongitude: -3.26282; geodeticDatum: WGS84; **Event:** eventID: 2; samplingProtocol: Beating; eventTime: Day

##### Distribution

Europe, North Africa, Turkey, North America

##### Notes

see *Species delimitation and identification using DNA barcodes*.

#### Brigittea latens

(Fabricius, 1775)

##### Materials

- **Type status:** Other material. **Occurrence:** individualCount: 1; sex: male; **Location:** locationID: C1; continent: Europe; country: Spain; countryCode: ES; stateProvince: Castilla-La Mancha; county: Ciudad Real; locality: Valle Brezoso; verbatimElevation: 756.56; decimalLatitude: 39.35663; decimalLongitude: -4.35912; geodeticDatum: WGS84; **Event:** eventID: 2; samplingProtocol: Sweeping; eventTime: Day
- **Type status:** Other material. **Occurrence:** individualCount: 1; sex: male; **Location:** locationID: C2; continent: Europe; country: Spain; countryCode: ES; stateProvince: Castilla-La Mancha; county: Ciudad Real; locality: Valle Brezoso; verbatimElevation: 739.31; decimalLatitude: 39.35159; decimalLongitude: -4.3589; geodeticDatum: WGS84; **Event:** eventID: 4; samplingProtocol: Aerial; eventTime: Night
- **Type status:** Other material. **Occurrence:** individualCount: 1; sex: female; **Location:** locationID: C2; continent: Europe; country: Spain; countryCode: ES; stateProvince: Castilla-La Mancha; county: Ciudad Real; locality: Valle Brezoso; verbatimElevation: 739.31; decimalLatitude: 39.35159; decimalLongitude: -4.3589; geodeticDatum: WGS84; **Event:** eventID: 2; samplingProtocol: Beating; eventTime: Day
- **Type status:** Other material. **Occurrence:** individualCount: 1; sex: male; **Location:** locationID: C2; continent: Europe; country: Spain; countryCode: ES; stateProvince: Castilla-La Mancha; county: Ciudad Real; locality: Valle Brezoso; verbatimElevation: 739.31; decimalLatitude: 39.35159; decimalLongitude: -4.3589; geodeticDatum: WGS84; **Event:** eventID: 1; samplingProtocol: Sweeping; eventTime: Day
- **Type status:** Other material. **Occurrence:** individualCount: 3; sex: female; **Location:** locationID: C2; continent: Europe; country: Spain; countryCode: ES; stateProvince: Castilla-La Mancha; county: Ciudad Real; locality: Valle Brezoso; verbatimElevation: 739.31; decimalLatitude: 39.35159; decimalLongitude: -4.3589; geodeticDatum: WGS84; **Event:** eventID: 1; samplingProtocol: Sweeping; eventTime: Day
- **Type status:** Other material. **Occurrence:** individualCount: 1; sex: male; **Location:** locationID: C2; continent: Europe; country: Spain; countryCode: ES; stateProvince: Castilla-La Mancha; county: Ciudad Real; locality: Valle Brezoso; verbatimElevation: 739.31; decimalLatitude: 39.35159; decimalLongitude: -4.3589; geodeticDatum: WGS84; **Event:** eventID: 1; samplingProtocol: Sweeping; eventTime: Night
- **Type status:** Other material. **Occurrence:** individualCount: 1; sex: female; **Location:** locationID: C2; continent: Europe; country: Spain; countryCode: ES; stateProvince: Castilla-La Mancha; county: Ciudad Real; locality: Valle Brezoso; verbatimElevation: 739.31; decimalLatitude: 39.35159; decimalLongitude: -4.3589; geodeticDatum: WGS84; **Event:** eventID: 1; samplingProtocol: Sweeping; eventTime: Night
- **Type status:** Other material. **Occurrence:** individualCount: 1; sex: male; **Location:** locationID: C2; continent: Europe; country: Spain; countryCode: ES; stateProvince: Castilla-La Mancha; county: Ciudad Real; locality: Valle Brezoso; verbatimElevation: 739.31; decimalLatitude: 39.35159; decimalLongitude: -4.3589; geodeticDatum: WGS84; **Event:** eventID: 2; samplingProtocol: Sweeping; eventTime: Night
- **Type status:** Other material. **Occurrence:** individualCount: 1; sex: female; **Location:** locationID: C2; continent: Europe; country: Spain; countryCode: ES; stateProvince: Castilla-La Mancha; county: Ciudad Real; locality: Valle Brezoso; verbatimElevation: 739.31; decimalLatitude: 39.35159; decimalLongitude: -4.3589; geodeticDatum: WGS84; **Event:** eventID: 2; samplingProtocol: Sweeping; eventTime: Day
- **Type status:** Other material. **Occurrence:** individualCount: 2; sex: male; **Location:** locationID: C3; continent: Europe; country: Spain; countryCode: ES; stateProvince: Castilla-La Mancha; county: Ciudad Real; locality: La Quesera; verbatimElevation: 767.55; decimalLatitude: 39.36177; decimalLongitude: -4.41733; geodeticDatum: WGS84; **Event:** eventID: 2; samplingProtocol: Beating; eventTime: Night
- **Type status:** Other material. **Occurrence:** individualCount: 2; sex: female; **Location:** locationID: C3; continent: Europe; country: Spain; countryCode: ES; stateProvince: Castilla-La Mancha; county: Ciudad Real; locality: La Quesera; verbatimElevation: 767.55; decimalLatitude: 39.36177; decimalLongitude: -4.41733; geodeticDatum: WGS84; **Event:** eventID: 2; samplingProtocol: Beating; eventTime: Day
- **Type status:** Other material. **Occurrence:** individualCount: 2; sex: female; **Location:** locationID: C4; continent: Europe; country: Spain; countryCode: ES; stateProvince: Castilla-La Mancha; county: Ciudad Real; locality: La Quesera; verbatimElevation: 772.3; decimalLatitude: 39.36337; decimalLongitude: -4.41704; geodeticDatum: WGS84; **Event:** eventID: 1; samplingProtocol: Sweeping; eventTime: Night
- **Type status:** Other material. **Occurrence:** individualCount: 1; sex: female; **Location:** locationID: O1; continent: Europe; country: Spain; countryCode: ES; stateProvince: Aragón; county: Huesca; locality: O Furno; verbatimElevation: 1396.73; decimalLatitude: 42.60677; decimalLongitude: 0.13135; geodeticDatum: WGS84; **Event:** eventID: 1; samplingProtocol: Beating; eventTime: Day
- **Type status:** Other material. **Occurrence:** individualCount: 2; sex: male; **Location:** locationID: O1; continent: Europe; country: Spain; countryCode: ES; stateProvince: Aragón; county: Huesca; locality: O Furno; verbatimElevation: 1396.73; decimalLatitude: 42.60677; decimalLongitude: 0.13135; geodeticDatum: WGS84; **Event:** eventID: 1; samplingProtocol: Sweeping; eventTime: Day
- **Type status:** Other material. **Occurrence:** individualCount: 2; sex: female; **Location:** locationID: O1; continent: Europe; country: Spain; countryCode: ES; stateProvince: Aragón; county: Huesca; locality: O Furno; verbatimElevation: 1396.73; decimalLatitude: 42.60677; decimalLongitude: 0.13135; geodeticDatum: WGS84; **Event:** eventID: 1; samplingProtocol: Sweeping; eventTime: Day
- **Type status:** Other material. **Occurrence:** individualCount: 2; sex: male; **Location:** locationID: O1; continent: Europe; country: Spain; countryCode: ES; stateProvince: Aragón; county: Huesca; locality: O Furno; verbatimElevation: 1396.73; decimalLatitude: 42.60677; decimalLongitude: 0.13135; geodeticDatum: WGS84; **Event:** eventID: 1; samplingProtocol: Sweeping; eventTime: Night
- **Type status:** Other material. **Occurrence:** individualCount: 7; sex: male; **Location:** locationID: O1; continent: Europe; country: Spain; countryCode: ES; stateProvince: Aragón; county: Huesca; locality: O Furno; verbatimElevation: 1396.73; decimalLatitude: 42.60677; decimalLongitude: 0.13135; geodeticDatum: WGS84; **Event:** eventID: 1; samplingProtocol: Sweeping; eventTime: Night
- **Type status:** Other material. **Occurrence:** individualCount: 15; sex: female; **Location:** locationID: O1; continent: Europe; country: Spain; countryCode: ES; stateProvince: Aragón; county: Huesca; locality: O Furno; verbatimElevation: 1396.73; decimalLatitude: 42.60677; decimalLongitude: 0.13135; geodeticDatum: WGS84; **Event:** eventID: 1; samplingProtocol: Sweeping; eventTime: Night
- **Type status:** Other material. **Occurrence:** individualCount: 3; sex: male; **Location:** locationID: O1; continent: Europe; country: Spain; countryCode: ES; stateProvince: Aragón; county: Huesca; locality: O Furno; verbatimElevation: 1396.73; decimalLatitude: 42.60677; decimalLongitude: 0.13135; geodeticDatum: WGS84; **Event:** eventID: 2; samplingProtocol: Sweeping; eventTime: Day
- **Type status:** Other material. **Occurrence:** individualCount: 4; sex: female; **Location:** locationID: O1; continent: Europe; country: Spain; countryCode: ES; stateProvince: Aragón; county: Huesca; locality: O Furno; verbatimElevation: 1396.73; decimalLatitude: 42.60677; decimalLongitude: 0.13135; geodeticDatum: WGS84; **Event:** eventID: 2; samplingProtocol: Sweeping; eventTime: Day
- **Type status:** Other material. **Occurrence:** individualCount: 1; sex: female; **Location:** locationID: O2; continent: Europe; country: Spain; countryCode: ES; stateProvince: Aragón; county: Huesca; locality: Rebilla; verbatimElevation: 1158.13; decimalLatitude: 42.59427; decimalLongitude: 0.1529; geodeticDatum: WGS84; **Event:** eventID: 1; samplingProtocol: Beating; eventTime: Day
- **Type status:** Other material. **Occurrence:** individualCount: 1; sex: female; **Location:** locationID: O2; continent: Europe; country: Spain; countryCode: ES; stateProvince: Aragón; county: Huesca; locality: Rebilla; verbatimElevation: 1158.13; decimalLatitude: 42.59427; decimalLongitude: 0.1529; geodeticDatum: WGS84; **Event:** eventID: 1; samplingProtocol: Sweeping; eventTime: Day
- **Type status:** Other material. **Occurrence:** individualCount: 3; sex: male; **Location:** locationID: O2; continent: Europe; country: Spain; countryCode: ES; stateProvince: Aragón; county: Huesca; locality: Rebilla; verbatimElevation: 1158.13; decimalLatitude: 42.59427; decimalLongitude: 0.1529; geodeticDatum: WGS84; **Event:** eventID: 1; samplingProtocol: Sweeping; eventTime: Night
- **Type status:** Other material. **Occurrence:** individualCount: 1; sex: female; **Location:** locationID: O2; continent: Europe; country: Spain; countryCode: ES; stateProvince: Aragón; county: Huesca; locality: Rebilla; verbatimElevation: 1158.13; decimalLatitude: 42.59427; decimalLongitude: 0.1529; geodeticDatum: WGS84; **Event:** eventID: 1; samplingProtocol: Sweeping; eventTime: Night
- **Type status:** Other material. **Occurrence:** individualCount: 1; sex: male; **Location:** locationID: P2; continent: Europe; country: Spain; countryCode: ES; stateProvince: Castilla y León; county: León; locality: Joyoguelas; verbatimElevation: 763.98; decimalLatitude: 43.17771; decimalLongitude: -4.90579; geodeticDatum: WGS84; **Event:** eventID: 1; samplingProtocol: Sweeping; eventTime: Night
- **Type status:** Other material. **Occurrence:** individualCount: 2; sex: male; **Location:** locationID: P2; continent: Europe; country: Spain; countryCode: ES; stateProvince: Castilla y León; county: León; locality: Joyoguelas; verbatimElevation: 763.98; decimalLatitude: 43.17771; decimalLongitude: -4.90579; geodeticDatum: WGS84; **Event:** eventID: 2; samplingProtocol: Sweeping; eventTime: Day
- **Type status:** Other material. **Occurrence:** individualCount: 2; sex: female; **Location:** locationID: P2; continent: Europe; country: Spain; countryCode: ES; stateProvince: Castilla y León; county: León; locality: Joyoguelas; verbatimElevation: 763.98; decimalLatitude: 43.17771; decimalLongitude: -4.90579; geodeticDatum: WGS84; **Event:** eventID: 2; samplingProtocol: Sweeping; eventTime: Day
- **Type status:** Other material. **Occurrence:** individualCount: 1; sex: male; **Location:** locationID: P4; continent: Europe; country: Spain; countryCode: ES; stateProvince: Castilla y León; county: León; locality: El Canto; verbatimElevation: 943.48; decimalLatitude: 43.17227; decimalLongitude: -4.90857; geodeticDatum: WGS84; **Event:** eventID: 1; samplingProtocol: Beating; eventTime: Day
- **Type status:** Other material. **Occurrence:** individualCount: 3; sex: female; **Location:** locationID: P4; continent: Europe; country: Spain; countryCode: ES; stateProvince: Castilla y León; county: León; locality: El Canto; verbatimElevation: 943.48; decimalLatitude: 43.17227; decimalLongitude: -4.90857; geodeticDatum: WGS84; **Event:** eventID: 1; samplingProtocol: Sweeping; eventTime: Night
- **Type status:** Other material. **Occurrence:** individualCount: 2; sex: female; **Location:** locationID: P4; continent: Europe; country: Spain; countryCode: ES; stateProvince: Castilla y León; county: León; locality: El Canto; verbatimElevation: 943.48; decimalLatitude: 43.17227; decimalLongitude: -4.90857; geodeticDatum: WGS84; **Event:** eventID: 2; samplingProtocol: Sweeping; eventTime: Day

##### Distribution

Europe to Central Asia

##### Notes

see *Species delimitation and identification using DNA barcodes*.

#### Brigittea sp04

##### Materials

- **Type status:** Other material. **Occurrence:** individualCount: 1; sex: male; **Location:** locationID: C1; continent: Europe; country: Spain; countryCode: ES; stateProvince: Castilla-La Mancha; county: Ciudad Real; locality: Valle Brezoso; verbatimElevation: 756.56; decimalLatitude: 39.35663; decimalLongitude: -4.35912; geodeticDatum: WGS84; **Event:** eventID: 2; samplingProtocol: Sweeping; eventTime: Day
- **Type status:** Other material. **Occurrence:** individualCount: 1; sex: male; **Location:** locationID: C3; continent: Europe; country: Spain; countryCode: ES; stateProvince: Castilla-La Mancha; county: Ciudad Real; locality: La Quesera; verbatimElevation: 767.55; decimalLatitude: 39.36177; decimalLongitude: -4.41733; geodeticDatum: WGS84; **Event:** eventID: 3; samplingProtocol: Aerial; eventTime: Night
- **Type status:** Other material. **Occurrence:** individualCount: 1; sex: male; **Location:** locationID: M1; continent: Europe; country: Spain; countryCode: ES; stateProvince: Extremadura; county: Cáceres; locality: Peña Falcón; verbatimElevation: 320.6; decimalLatitude: 39.83296; decimalLongitude: -6.0641; geodeticDatum: WGS84; **Event:** eventID: 1; samplingProtocol: Aerial; eventTime: Night

##### Distribution

?

##### Notes

This is a species of *Brigittea* Lehtinen, 1967, which we were unable to identify; see *Species delimitation and identification using DNA barcodes*.

#### Dictyna pusilla

Thorell, 1856

##### Materials

- **Type status:** Other material. **Occurrence:** individualCount: 1; sex: male; **Location:** locationID: A2; continent: Europe; country: Spain; countryCode: ES; stateProvince: Catalonia; county: Lleida; locality: Sola de Boi; verbatimElevation: 1738.7; decimalLatitude: 42.54913; decimalLongitude: 0.87137; geodeticDatum: WGS84; **Event:** eventID: 1; samplingProtocol: Sweeping; eventTime: Night

##### Distribution

Palearctic

##### Notes

**Figure 4.**
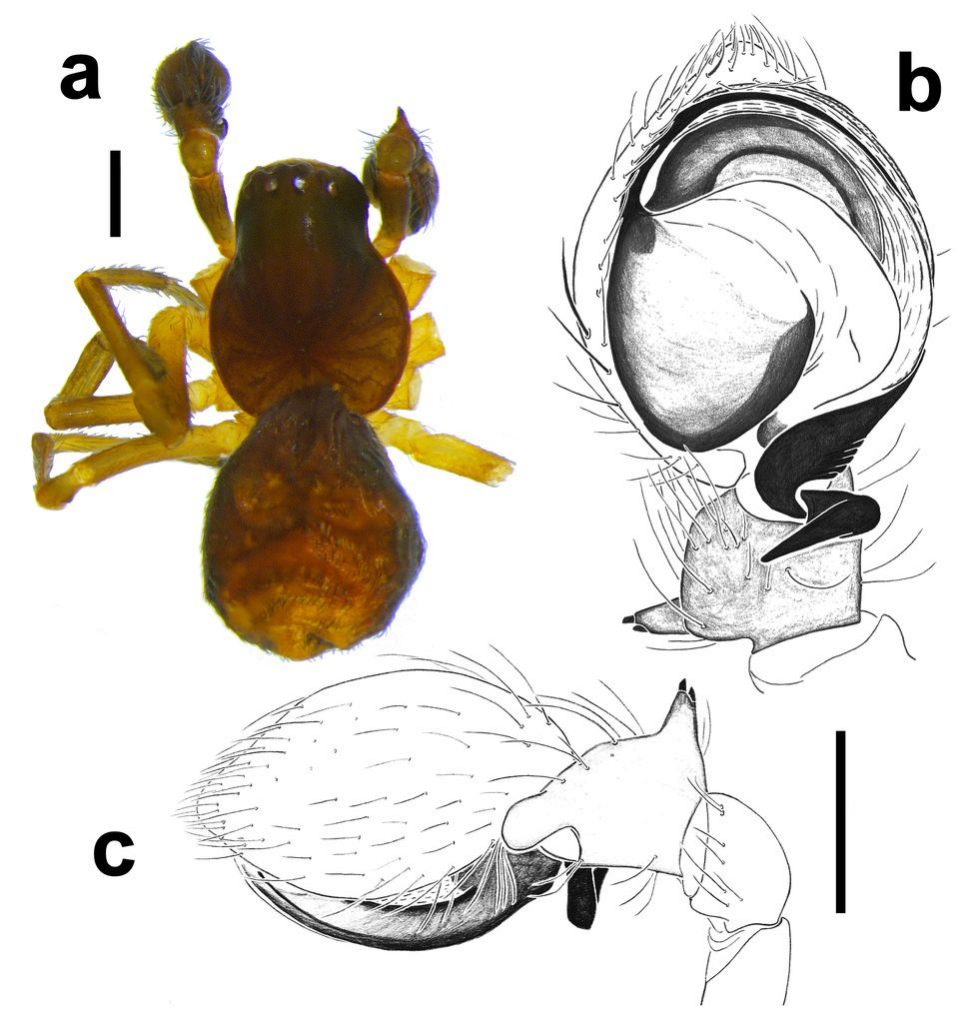
*Dictyna pusilla* Thorell, 1856. a: male, dorsal habitus, scale = 0.6 mm; b: left male palp, ventral, scale = 0.2 mm; c: left male palp, retrolateral, scale = 0.2 mm.

This is a new record for the Iberian Peninsula. See Fig. 4.

#### Lathys humilis

(Blackwall, 1855)

##### Materials

- **Type status:** Other material. **Occurrence:** individualCount: 1; sex: female; **Location:** locationID: A1; continent: Europe; country: Spain; countryCode: ES; stateProvince: Catalonia; county: Lleida; locality: Sola de Boi; verbatimElevation: 1759.8; decimalLatitude: 42.54958; decimalLongitude: 0.87254; geodeticDatum: WGS84; **Event:** eventID: 1; samplingProtocol: Beating; eventTime: Day
- **Type status:** Other material. **Occurrence:** individualCount: 1; sex: female; **Location:** locationID: A1; continent: Europe; country: Spain; countryCode: ES; stateProvince: Catalonia; county: Lleida; locality: Sola de Boi; verbatimElevation: 1759.8; decimalLatitude: 42.54958; decimalLongitude: 0.87254; geodeticDatum: WGS84; **Event:** eventID: 1; samplingProtocol: Beating; eventTime: Night
- **Type status:** Other material. **Occurrence:** individualCount: 1; sex: male; **Location:** locationID: A1; continent: Europe; country: Spain; countryCode: ES; stateProvince: Catalonia; county: Lleida; locality: Sola de Boi; verbatimElevation: 1759.8; decimalLatitude: 42.54958; decimalLongitude: 0.87254; geodeticDatum: WGS84; **Event:** eventID: 1; samplingProtocol: Beating; eventTime: Night
- **Type status:** Other material. **Occurrence:** individualCount: 1; sex: female; **Location:** locationID: A1; continent: Europe; country: Spain; countryCode: ES; stateProvince: Catalonia; county: Lleida; locality: Sola de Boi; verbatimElevation: 1759.8; decimalLatitude: 42.54958; decimalLongitude: 0.87254; geodeticDatum: WGS84; **Event:** eventID: 1; samplingProtocol: Beating; eventTime: Night
- **Type status:** Other material. **Occurrence:** individualCount: 3; sex: female; **Location:** locationID: A1; continent: Europe; country: Spain; countryCode: ES; stateProvince: Catalonia; county: Lleida; locality: Sola de Boi; verbatimElevation: 1759.8; decimalLatitude: 42.54958; decimalLongitude: 0.87254; geodeticDatum: WGS84; **Event:** eventID: 2; samplingProtocol: Beating; eventTime: Day
- **Type status:** Other material. **Occurrence:** individualCount: 1; sex: male; **Location:** locationID: A1; continent: Europe; country: Spain; countryCode: ES; stateProvince: Catalonia; county: Lleida; locality: Sola de Boi; verbatimElevation: 1759.8; decimalLatitude: 42.54958; decimalLongitude: 0.87254; geodeticDatum: WGS84; **Event:** eventID: 1; samplingProtocol: Sweeping; eventTime: Night
- **Type status:** Other material. **Occurrence:** individualCount: 1; sex: male; **Location:** locationID: A1; continent: Europe; country: Spain; countryCode: ES; stateProvince: Catalonia; county: Lleida; locality: Sola de Boi; verbatimElevation: 1759.8; decimalLatitude: 42.54958; decimalLongitude: 0.87254; geodeticDatum: WGS84; **Event:** eventID: 2; samplingProtocol: Sweeping; eventTime: Day
- **Type status:** Other material. **Occurrence:** individualCount: 5; sex: female; **Location:** locationID: A2; continent: Europe; country: Spain; countryCode: ES; stateProvince: Catalonia; county: Lleida; locality: Sola de Boi; verbatimElevation: 1738.7; decimalLatitude: 42.54913; decimalLongitude: 0.87137; geodeticDatum: WGS84; **Event:** eventID: 1; samplingProtocol: Beating; eventTime: Day
- **Type status:** Other material. **Occurrence:** individualCount: 1; sex: female; **Location:** locationID: A2; continent: Europe; country: Spain; countryCode: ES; stateProvince: Catalonia; county: Lleida; locality: Sola de Boi; verbatimElevation: 1738.7; decimalLatitude: 42.54913; decimalLongitude: 0.87137; geodeticDatum: WGS84; **Event:** eventID: 1; samplingProtocol: Beating; eventTime: Night
- **Type status:** Other material. **Occurrence:** individualCount: 1; sex: female; **Location:** locationID: A2; continent: Europe; country: Spain; countryCode: ES; stateProvince: Catalonia; county: Lleida; locality: Sola de Boi; verbatimElevation: 1738.7; decimalLatitude: 42.54913; decimalLongitude: 0.87137; geodeticDatum: WGS84; **Event:** eventID: 1; samplingProtocol: Sweeping; eventTime: Night
- **Type status:** Other material. **Occurrence:** individualCount: 1; sex: male; **Location:** locationID: O1; continent: Europe; country: Spain; countryCode: ES; stateProvince: Aragón; county: Huesca; locality: O Furno; verbatimElevation: 1396.73; decimalLatitude: 42.60677; decimalLongitude: 0.13135; geodeticDatum: WGS84; **Event:** eventID: 1; samplingProtocol: Aerial; eventTime: Night
- **Type status:** Other material. **Occurrence:** individualCount: 13; sex: female; **Location:** locationID: O1; continent: Europe; country: Spain; countryCode: ES; stateProvince: Aragón; county: Huesca; locality: O Furno; verbatimElevation: 1396.73; decimalLatitude: 42.60677; decimalLongitude: 0.13135; geodeticDatum: WGS84; **Event:** eventID: 1; samplingProtocol: Beating; eventTime: Day
- **Type status:** Other material. **Occurrence:** individualCount: 6; sex: female; **Location:** locationID: O1; continent: Europe; country: Spain; countryCode: ES; stateProvince: Aragón; county: Huesca; locality: O Furno; verbatimElevation: 1396.73; decimalLatitude: 42.60677; decimalLongitude: 0.13135; geodeticDatum: WGS84; **Event:** eventID: 1; samplingProtocol: Beating; eventTime: Night
- **Type status:** Other material. **Occurrence:** individualCount: 4; sex: female; **Location:** locationID: O1; continent: Europe; country: Spain; countryCode: ES; stateProvince: Aragón; county: Huesca; locality: O Furno; verbatimElevation: 1396.73; decimalLatitude: 42.60677; decimalLongitude: 0.13135; geodeticDatum: WGS84; **Event:** eventID: 1; samplingProtocol: Beating; eventTime: Night
- **Type status:** Other material. **Occurrence:** individualCount: 13; sex: female; **Location:** locationID: O1; continent: Europe; country: Spain; countryCode: ES; stateProvince: Aragón; county: Huesca; locality: O Furno; verbatimElevation: 1396.73; decimalLatitude: 42.60677; decimalLongitude: 0.13135; geodeticDatum: WGS84; **Event:** eventID: 2; samplingProtocol: Beating; eventTime: Day
- **Type status:** Other material. **Occurrence:** individualCount: 1; sex: female; **Location:** locationID: O1; continent: Europe; country: Spain; countryCode: ES; stateProvince: Aragón; county: Huesca; locality: O Furno; verbatimElevation: 1396.73; decimalLatitude: 42.60677; decimalLongitude: 0.13135; geodeticDatum: WGS84; **Event:** eventID: 1; samplingProtocol: Sweeping; eventTime: Day
- **Type status:** Other material. **Occurrence:** individualCount: 2; sex: female; **Location:** locationID: O1; continent: Europe; country: Spain; countryCode: ES; stateProvince: Aragón; county: Huesca; locality: O Furno; verbatimElevation: 1396.73; decimalLatitude: 42.60677; decimalLongitude: 0.13135; geodeticDatum: WGS84; **Event:** eventID: 1; samplingProtocol: Sweeping; eventTime: Night
- **Type status:** Other material. **Occurrence:** individualCount: 2; sex: female; **Location:** locationID: O1; continent: Europe; country: Spain; countryCode: ES; stateProvince: Aragón; county: Huesca; locality: O Furno; verbatimElevation: 1396.73; decimalLatitude: 42.60677; decimalLongitude: 0.13135; geodeticDatum: WGS84; **Event:** eventID: 1; samplingProtocol: Sweeping; eventTime: Night
- **Type status:** Other material. **Occurrence:** individualCount: 1; sex: female; **Location:** locationID: O1; continent: Europe; country: Spain; countryCode: ES; stateProvince: Aragón; county: Huesca; locality: O Furno; verbatimElevation: 1396.73; decimalLatitude: 42.60677; decimalLongitude: 0.13135; geodeticDatum: WGS84; **Event:** eventID: 2; samplingProtocol: Sweeping; eventTime: Day
- **Type status:** Other material. **Occurrence:** individualCount: 1; sex: female; **Location:** locationID: O2; continent: Europe; country: Spain; countryCode: ES; stateProvince: Aragón; county: Huesca; locality: Rebilla; verbatimElevation: 1158.13; decimalLatitude: 42.59427; decimalLongitude: 0.1529; geodeticDatum: WGS84; **Event:** eventID: 1; samplingProtocol: Aerial; eventTime: Night
- **Type status:** Other material. **Occurrence:** individualCount: 2; sex: female; **Location:** locationID: O2; continent: Europe; country: Spain; countryCode: ES; stateProvince: Aragón; county: Huesca; locality: Rebilla; verbatimElevation: 1158.13; decimalLatitude: 42.59427; decimalLongitude: 0.1529; geodeticDatum: WGS84; **Event:** eventID: 1; samplingProtocol: Beating; eventTime: Day
- **Type status:** Other material. **Occurrence:** individualCount: 2; sex: female; **Location:** locationID: O2; continent: Europe; country: Spain; countryCode: ES; stateProvince: Aragón; county: Huesca; locality: Rebilla; verbatimElevation: 1158.13; decimalLatitude: 42.59427; decimalLongitude: 0.1529; geodeticDatum: WGS84; **Event:** eventID: 1; samplingProtocol: Beating; eventTime: Night
- **Type status:** Other material. **Occurrence:** individualCount: 6; sex: female; **Location:** locationID: O2; continent: Europe; country: Spain; countryCode: ES; stateProvince: Aragón; county: Huesca; locality: Rebilla; verbatimElevation: 1158.13; decimalLatitude: 42.59427; decimalLongitude: 0.1529; geodeticDatum: WGS84; **Event:** eventID: 2; samplingProtocol: Beating; eventTime: Day
- **Type status:** Other material. **Occurrence:** individualCount: 1; sex: female; **Location:** locationID: O2; continent: Europe; country: Spain; countryCode: ES; stateProvince: Aragón; county: Huesca; locality: Rebilla; verbatimElevation: 1158.13; decimalLatitude: 42.59427; decimalLongitude: 0.1529; geodeticDatum: WGS84; **Event:** eventID: 2; samplingProtocol: Sweeping; eventTime: Day
- **Type status:** Other material. **Occurrence:** individualCount: 1; sex: female; **Location:** locationID: P1; continent: Europe; country: Spain; countryCode: ES; stateProvince: Castilla y León; county: León; locality: Monte Robledo; verbatimElevation: 1071.58; decimalLatitude: 43.1445; decimalLongitude: -4.92675; geodeticDatum: WGS84; **Event:** eventID: 1; samplingProtocol: Sweeping; eventTime: Day
- **Type status:** Other material. **Occurrence:** individualCount: 1; sex: female; **Location:** locationID: P3; continent: Europe; country: Spain; countryCode: ES; stateProvince: Castilla y León; county: León; locality: Las Arroyas; verbatimElevation: 1097.1; decimalLatitude: 43.14351; decimalLongitude: -4.94878; geodeticDatum: WGS84; **Event:** eventID: 2; samplingProtocol: Aerial; eventTime: Night
- **Type status:** Other material. **Occurrence:** individualCount: 1; sex: female; **Location:** locationID: P3; continent: Europe; country: Spain; countryCode: ES; stateProvince: Castilla y León; county: León; locality: Las Arroyas; verbatimElevation: 1097.1; decimalLatitude: 43.14351; decimalLongitude: -4.94878; geodeticDatum: WGS84; **Event:** eventID: 1; samplingProtocol: Beating; eventTime: Night

##### Distribution

Palearctic

#### Lathys sp01

##### Materials

- **Type status:** Other material. **Occurrence:** individualCount: 2; sex: female; **Location:** locationID: C1; continent: Europe; country: Spain; countryCode: ES; stateProvince: Castilla-La Mancha; county: Ciudad Real; locality: Valle Brezoso; verbatimElevation: 756.56; decimalLatitude: 39.35663; decimalLongitude: -4.35912; geodeticDatum: WGS84; **Event:** eventID: 1; samplingProtocol: Beating; eventTime: Night
- **Type status:** Other material. **Occurrence:** individualCount: 1; sex: female; **Location:** locationID: C1; continent: Europe; country: Spain; countryCode: ES; stateProvince: Castilla-La Mancha; county: Ciudad Real; locality: Valle Brezoso; verbatimElevation: 756.56; decimalLatitude: 39.35663; decimalLongitude: -4.35912; geodeticDatum: WGS84; **Event:** eventID: 2; samplingProtocol: Beating; eventTime: Day
- **Type status:** Other material. **Occurrence:** individualCount: 1; sex: female; **Location:** locationID: C1; continent: Europe; country: Spain; countryCode: ES; stateProvince: Castilla-La Mancha; county: Ciudad Real; locality: Valle Brezoso; verbatimElevation: 756.56; decimalLatitude: 39.35663; decimalLongitude: -4.35912; geodeticDatum: WGS84; **Event:** eventID: 2; samplingProtocol: Sweeping; eventTime: Night
- **Type status:** Other material. **Occurrence:** individualCount: 3; sex: female; **Location:** locationID: C2; continent: Europe; country: Spain; countryCode: ES; stateProvince: Castilla-La Mancha; county: Ciudad Real; locality: Valle Brezoso; verbatimElevation: 739.31; decimalLatitude: 39.35159; decimalLongitude: -4.3589; geodeticDatum: WGS84; **Event:** eventID: 2; samplingProtocol: Beating; eventTime: Day
- **Type status:** Other material. **Occurrence:** individualCount: 1; sex: female; **Location:** locationID: C2; continent: Europe; country: Spain; countryCode: ES; stateProvince: Castilla-La Mancha; county: Ciudad Real; locality: Valle Brezoso; verbatimElevation: 739.31; decimalLatitude: 39.35159; decimalLongitude: -4.3589; geodeticDatum: WGS84; **Event:** eventID: 1; samplingProtocol: Sweeping; eventTime: Day
- **Type status:** Other material. **Occurrence:** individualCount: 1; sex: female; **Location:** locationID: C2; continent: Europe; country: Spain; countryCode: ES; stateProvince: Castilla-La Mancha; county: Ciudad Real; locality: Valle Brezoso; verbatimElevation: 739.31; decimalLatitude: 39.35159; decimalLongitude: -4.3589; geodeticDatum: WGS84; **Event:** eventID: 2; samplingProtocol: Sweeping; eventTime: Day
- **Type status:** Other material. **Occurrence:** individualCount: 1; sex: female; **Location:** locationID: C3; continent: Europe; country: Spain; countryCode: ES; stateProvince: Castilla-La Mancha; county: Ciudad Real; locality: La Quesera; verbatimElevation: 767.55; decimalLatitude: 39.36177; decimalLongitude: -4.41733; geodeticDatum: WGS84; **Event:** eventID: 2; samplingProtocol: Beating; eventTime: Night
- **Type status:** Other material. **Occurrence:** individualCount: 2; sex: female; **Location:** locationID: M2; continent: Europe; country: Spain; countryCode: ES; stateProvince: Extremadura; county: Cáceres; locality: Fuente del Frances; verbatimElevation: 320.72; decimalLatitude: 39.828; decimalLongitude: -6.03249; geodeticDatum: WGS84; **Event:** eventID: 1; samplingProtocol: Aerial; eventTime: Night
- **Type status:** Other material. **Occurrence:** individualCount: 2; sex: female; **Location:** locationID: M2; continent: Europe; country: Spain; countryCode: ES; stateProvince: Extremadura; county: Cáceres; locality: Fuente del Frances; verbatimElevation: 320.72; decimalLatitude: 39.828; decimalLongitude: -6.03249; geodeticDatum: WGS84; **Event:** eventID: 1; samplingProtocol: Beating; eventTime: Day
- **Type status:** Other material. **Occurrence:** individualCount: 1; sex: female; **Location:** locationID: M2; continent: Europe; country: Spain; countryCode: ES; stateProvince: Extremadura; county: Cáceres; locality: Fuente del Frances; verbatimElevation: 320.72; decimalLatitude: 39.828; decimalLongitude: -6.03249; geodeticDatum: WGS84; **Event:** eventID: 1; samplingProtocol: Beating; eventTime: Night
- **Type status:** Other material. **Occurrence:** individualCount: 1; sex: female; **Location:** locationID: M2; continent: Europe; country: Spain; countryCode: ES; stateProvince: Extremadura; county: Cáceres; locality: Fuente del Frances; verbatimElevation: 320.72; decimalLatitude: 39.828; decimalLongitude: -6.03249; geodeticDatum: WGS84; **Event:** eventID: 1; samplingProtocol: Sweeping; eventTime: Day
- **Type status:** Other material. **Occurrence:** individualCount: 1; sex: female; **Location:** locationID: M2; continent: Europe; country: Spain; countryCode: ES; stateProvince: Extremadura; county: Cáceres; locality: Fuente del Frances; verbatimElevation: 320.72; decimalLatitude: 39.828; decimalLongitude: -6.03249; geodeticDatum: WGS84; **Event:** eventID: 1; samplingProtocol: Sweeping; eventTime: Night

##### Distribution

?

##### Notes

We could not identify this species. It might be a new species to science. See *Species delimitation using DNA barcodes*.

#### Marilynia bicolor

(Simon, 1870)

##### Materials

- **Type status:** Other material. **Occurrence:** individualCount: 3; sex: male; **Location:** locationID: C3; continent: Europe; country: Spain; countryCode: ES; stateProvince: Castilla-La Mancha; county: Ciudad Real; locality: La Quesera; verbatimElevation: 767.55; decimalLatitude: 39.36177; decimalLongitude: -4.41733; geodeticDatum: WGS84; **Event:** eventID: A; samplingProtocol: Pitfall
- **Type status:** Other material. **Occurrence:** individualCount: 2; sex: male; **Location:** locationID: C3; continent: Europe; country: Spain; countryCode: ES; stateProvince: Castilla-La Mancha; county: Ciudad Real; locality: La Quesera; verbatimElevation: 767.55; decimalLatitude: 39.36177; decimalLongitude: -4.41733; geodeticDatum: WGS84; **Event:** eventID: E; samplingProtocol: Pitfall
- **Type status:** Other material. **Occurrence:** individualCount: 1; sex: male; **Location:** locationID: C3; continent: Europe; country: Spain; countryCode: ES; stateProvince: Castilla-La Mancha; county: Ciudad Real; locality: La Quesera; verbatimElevation: 767.55; decimalLatitude: 39.36177; decimalLongitude: -4.41733; geodeticDatum: WGS84; **Event:** eventID: I; samplingProtocol: Pitfall
- **Type status:** Other material. **Occurrence:** individualCount: 2; sex: male; **Location:** locationID: C3; continent: Europe; country: Spain; countryCode: ES; stateProvince: Castilla-La Mancha; county: Ciudad Real; locality: La Quesera; verbatimElevation: 767.55; decimalLatitude: 39.36177; decimalLongitude: -4.41733; geodeticDatum: WGS84; **Event:** eventID: J; samplingProtocol: Pitfall
- **Type status:** Other material. **Occurrence:** individualCount: 1; sex: female; **Location:** locationID: C3; continent: Europe; country: Spain; countryCode: ES; stateProvince: Castilla-La Mancha; county: Ciudad Real; locality: La Quesera; verbatimElevation: 767.55; decimalLatitude: 39.36177; decimalLongitude: -4.41733; geodeticDatum: WGS84; **Event:** eventID: J; samplingProtocol: Pitfall

##### Distribution

Europe to Central Asia, North Africa

#### Mastigusa arietina

(Thorell, 1871)

##### Materials

- **Type status:** Other material. **Occurrence:** individualCount: 1; sex: male; **Location:** locationID: A1; continent: Europe; country: Spain; countryCode: ES; stateProvince: Catalonia; county: Lleida; locality: Sola de Boi; verbatimElevation: 1759.8; decimalLatitude: 42.54958; decimalLongitude: 0.87254; geodeticDatum: WGS84; **Event:** eventID: 2; samplingProtocol: Aerial
- **Type status:** Other material. **Occurrence:** individualCount: 1; sex: male; **Location:** locationID: S1; continent: Europe; country: Spain; countryCode: ES; stateProvince: Andalucía; county: Granada; locality: Soportujar; verbatimElevation: 1786.57; decimalLatitude: 36.96151; decimalLongitude: -3.41881; geodeticDatum: WGS84; **Event:** eventID: E; samplingProtocol: Pitfall

##### Distribution

Palearctic

#### Nigma gratiosa

(Simon, 1881)

##### Materials

- **Type status:** Other material. **Occurrence:** individualCount: 1; sex: male; **Location:** locationID: C4; continent: Europe; country: Spain; countryCode: ES; stateProvince: Castilla-La Mancha; county: Ciudad Real; locality: La Quesera; verbatimElevation: 772.3; decimalLatitude: 39.36337; decimalLongitude: -4.41704; geodeticDatum: WGS84; **Event:** eventID: 2; samplingProtocol: Sweeping; eventTime: Day
- **Type status:** Other material. **Occurrence:** individualCount: 1; sex: female; **Location:** locationID: C4; continent: Europe; country: Spain; countryCode: ES; stateProvince: Castilla-La Mancha; county: Ciudad Real; locality: La Quesera; verbatimElevation: 772.3; decimalLatitude: 39.36337; decimalLongitude: -4.41704; geodeticDatum: WGS84; **Event:** eventID: 1; samplingProtocol: Sweeping; eventTime: Night

##### Distribution

Iberian Peninsula, North Africa

##### Notes

**Figure 5.**
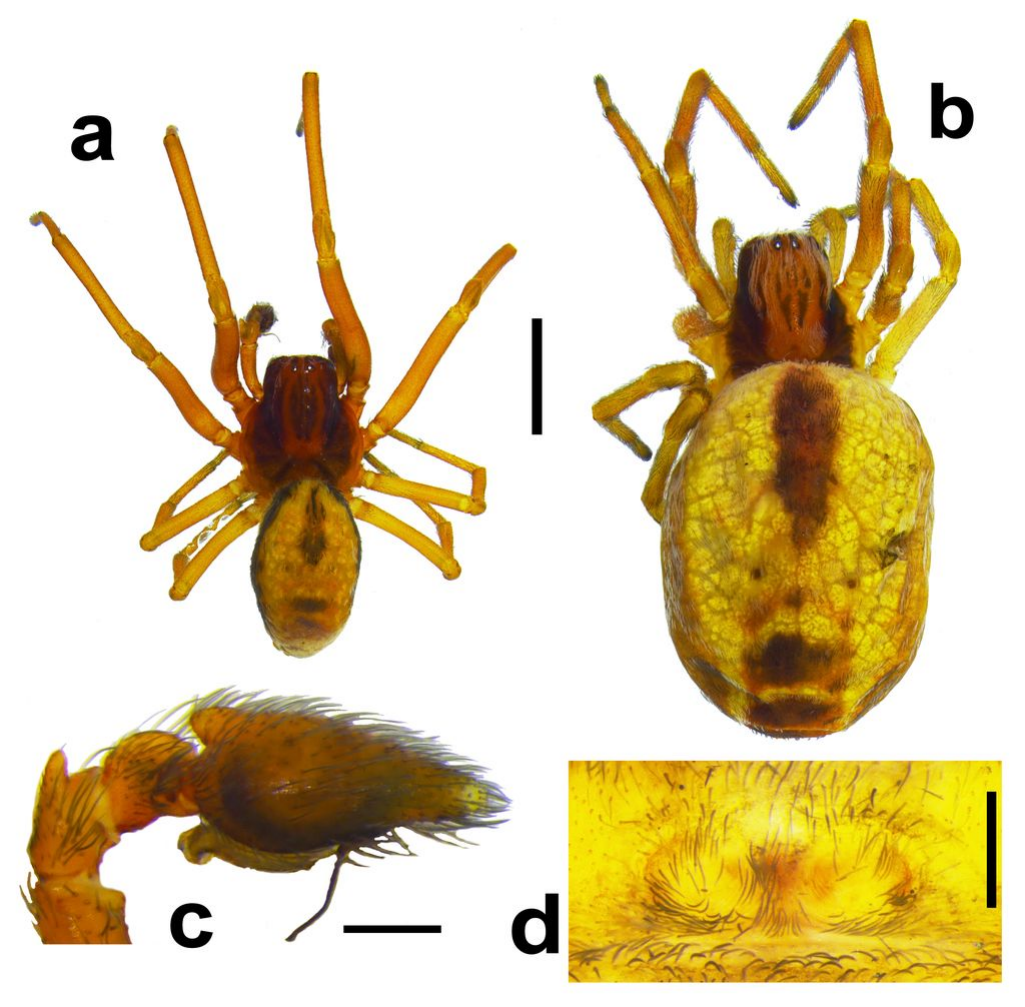
*Nigma gratiosa* (Simon, 1881). a: male, dorsal habitus, scale = 1 mm; b: female, dorsal habitus, scale = 0.1 mm; c: right male palp, retrolateral, scale = 0.2 mm; d: female epigynum, ventral, scale = 0.2 mm.

The female collected was tentatively assigned to the male based on the somatic aspect, because there are no available illustrations of the female epigyne. Although we decided not to include the formal description of the female in the present study, we nevertheless present images of the habitus and genitalia of both sexes to ease future identifications Fig. 5.

#### Nigma puella

(Simon, 1870)

##### Materials

- **Type status:** Other material. **Occurrence:** individualCount: 1; sex: female; **Location:** locationID: C1; continent: Europe; country: Spain; countryCode: ES; stateProvince: Castilla-La Mancha; county: Ciudad Real; locality: Valle Brezoso; verbatimElevation: 756.56; decimalLatitude: 39.35663; decimalLongitude: -4.35912; geodeticDatum: WGS84; **Event:** eventID: 1; samplingProtocol: Sweeping; eventTime: Day
- **Type status:** Other material. **Occurrence:** individualCount: 1; sex: female; **Location:** locationID: C2; continent: Europe; country: Spain; countryCode: ES; stateProvince: Castilla-La Mancha; county: Ciudad Real; locality: Valle Brezoso; verbatimElevation: 739.31; decimalLatitude: 39.35159; decimalLongitude: -4.3589; geodeticDatum: WGS84; **Event:** eventID: 2; samplingProtocol: Sweeping; eventTime: Day
- **Type status:** Other material. **Occurrence:** individualCount: 3; sex: female; **Location:** locationID: C3; continent: Europe; country: Spain; countryCode: ES; stateProvince: Castilla-La Mancha; county: Ciudad Real; locality: La Quesera; verbatimElevation: 767.55; decimalLatitude: 39.36177; decimalLongitude: -4.41733; geodeticDatum: WGS84; **Event:** eventID: 1; samplingProtocol: Beating; eventTime: Day
- **Type status:** Other material. **Occurrence:** individualCount: 1; sex: female; **Location:** locationID: C3; continent: Europe; country: Spain; countryCode: ES; stateProvince: Castilla-La Mancha; county: Ciudad Real; locality: La Quesera; verbatimElevation: 767.55; decimalLatitude: 39.36177; decimalLongitude: -4.41733; geodeticDatum: WGS84; **Event:** eventID: 2; samplingProtocol: Beating; eventTime: Day
- **Type status:** Other material. **Occurrence:** individualCount: 1; sex: female; **Location:** locationID: C3; continent: Europe; country: Spain; countryCode: ES; stateProvince: Castilla-La Mancha; county: Ciudad Real; locality: La Quesera; verbatimElevation: 767.55; decimalLatitude: 39.36177; decimalLongitude: -4.41733; geodeticDatum: WGS84; **Event:** eventID: 2; samplingProtocol: Sweeping; eventTime: Day
- **Type status:** Other material. **Occurrence:** individualCount: 1; sex: female; **Location:** locationID: C4; continent: Europe; country: Spain; countryCode: ES; stateProvince: Castilla-La Mancha; county: Ciudad Real; locality: La Quesera; verbatimElevation: 772.3; decimalLatitude: 39.36337; decimalLongitude: -4.41704; geodeticDatum: WGS84; **Event:** eventID: 4; samplingProtocol: Aerial; eventTime: Night
- **Type status:** Other material. **Occurrence:** individualCount: 2; sex: female; **Location:** locationID: C4; continent: Europe; country: Spain; countryCode: ES; stateProvince: Castilla-La Mancha; county: Ciudad Real; locality: La Quesera; verbatimElevation: 772.3; decimalLatitude: 39.36337; decimalLongitude: -4.41704; geodeticDatum: WGS84; **Event:** eventID: 1; samplingProtocol: Beating; eventTime: Day
- **Type status:** Other material. **Occurrence:** individualCount: 1; sex: male; **Location:** locationID: O2; continent: Europe; country: Spain; countryCode: ES; stateProvince: Aragón; county: Huesca; locality: Rebilla; verbatimElevation: 1158.13; decimalLatitude: 42.59427; decimalLongitude: 0.1529; geodeticDatum: WGS84; **Event:** eventID: 1; samplingProtocol: Aerial; eventTime: Night
- **Type status:** Other material. **Occurrence:** individualCount: 1; sex: female; **Location:** locationID: O2; continent: Europe; country: Spain; countryCode: ES; stateProvince: Aragón; county: Huesca; locality: Rebilla; verbatimElevation: 1158.13; decimalLatitude: 42.59427; decimalLongitude: 0.1529; geodeticDatum: WGS84; **Event:** eventID: 1; samplingProtocol: Aerial; eventTime: Night
- **Type status:** Other material. **Occurrence:** individualCount: 2; sex: male; **Location:** locationID: O2; continent: Europe; country: Spain; countryCode: ES; stateProvince: Aragón; county: Huesca; locality: Rebilla; verbatimElevation: 1158.13; decimalLatitude: 42.59427; decimalLongitude: 0.1529; geodeticDatum: WGS84; **Event:** eventID: 1; samplingProtocol: Beating; eventTime: Day
- **Type status:** Other material. **Occurrence:** individualCount: 8; sex: female; **Location:** locationID: O2; continent: Europe; country: Spain; countryCode: ES; stateProvince: Aragón; county: Huesca; locality: Rebilla; verbatimElevation: 1158.13; decimalLatitude: 42.59427; decimalLongitude: 0.1529; geodeticDatum: WGS84; **Event:** eventID: 1; samplingProtocol: Beating; eventTime: Day
- **Type status:** Other material. **Occurrence:** individualCount: 2; sex: male; **Location:** locationID: O2; continent: Europe; country: Spain; countryCode: ES; stateProvince: Aragón; county: Huesca; locality: Rebilla; verbatimElevation: 1158.13; decimalLatitude: 42.59427; decimalLongitude: 0.1529; geodeticDatum: WGS84; **Event:** eventID: 1; samplingProtocol: Beating; eventTime: Night
- **Type status:** Other material. **Occurrence:** individualCount: 3; sex: female; **Location:** locationID: O2; continent: Europe; country: Spain; countryCode: ES; stateProvince: Aragón; county: Huesca; locality: Rebilla; verbatimElevation: 1158.13; decimalLatitude: 42.59427; decimalLongitude: 0.1529; geodeticDatum: WGS84; **Event:** eventID: 1; samplingProtocol: Beating; eventTime: Night
- **Type status:** Other material. **Occurrence:** individualCount: 7; sex: female; **Location:** locationID: O2; continent: Europe; country: Spain; countryCode: ES; stateProvince: Aragón; county: Huesca; locality: Rebilla; verbatimElevation: 1158.13; decimalLatitude: 42.59427; decimalLongitude: 0.1529; geodeticDatum: WGS84; **Event:** eventID: 1; samplingProtocol: Beating; eventTime: Night
- **Type status:** Other material. **Occurrence:** individualCount: 1; sex: male; **Location:** locationID: O2; continent: Europe; country: Spain; countryCode: ES; stateProvince: Aragón; county: Huesca; locality: Rebilla; verbatimElevation: 1158.13; decimalLatitude: 42.59427; decimalLongitude: 0.1529; geodeticDatum: WGS84; **Event:** eventID: 2; samplingProtocol: Beating; eventTime: Day
- **Type status:** Other material. **Occurrence:** individualCount: 1; sex: female; **Location:** locationID: O2; continent: Europe; country: Spain; countryCode: ES; stateProvince: Aragón; county: Huesca; locality: Rebilla; verbatimElevation: 1158.13; decimalLatitude: 42.59427; decimalLongitude: 0.1529; geodeticDatum: WGS84; **Event:** eventID: 2; samplingProtocol: Beating; eventTime: Day
- **Type status:** Other material. **Occurrence:** individualCount: 1; sex: female; **Location:** locationID: O2; continent: Europe; country: Spain; countryCode: ES; stateProvince: Aragón; county: Huesca; locality: Rebilla; verbatimElevation: 1158.13; decimalLatitude: 42.59427; decimalLongitude: 0.1529; geodeticDatum: WGS84; **Event:** eventID: 1; samplingProtocol: Sweeping; eventTime: Night
- **Type status:** Other material. **Occurrence:** individualCount: 2; sex: female; **Location:** locationID: O2; continent: Europe; country: Spain; countryCode: ES; stateProvince: Aragón; county: Huesca; locality: Rebilla; verbatimElevation: 1158.13; decimalLatitude: 42.59427; decimalLongitude: 0.1529; geodeticDatum: WGS84; **Event:** eventID: 2; samplingProtocol: Sweeping; eventTime: Day

##### Distribution

Europe, Azores, Madeira, Canary Islands

#### Nigma sp19

##### Materials

- **Type status:** Other material. **Occurrence:** individualCount: 1; sex: female; **Location:** locationID: C1; continent: Europe; country: Spain; countryCode: ES; stateProvince: Castilla-La Mancha; county: Ciudad Real; locality: Valle Brezoso; verbatimElevation: 756.56; decimalLatitude: 39.35663; decimalLongitude: -4.35912; geodeticDatum: WGS84; **Event:** eventID: 1; samplingProtocol: Sweeping; eventTime: Day
- **Type status:** Other material. **Occurrence:** individualCount: 2; sex: female; **Location:** locationID: C2; continent: Europe; country: Spain; countryCode: ES; stateProvince: Castilla-La Mancha; county: Ciudad Real; locality: Valle Brezoso; verbatimElevation: 739.31; decimalLatitude: 39.35159; decimalLongitude: -4.3589; geodeticDatum: WGS84; **Event:** eventID: 1; samplingProtocol: Sweeping; eventTime: Day
- **Type status:** Other material. **Occurrence:** individualCount: 1; sex: female; **Location:** locationID: C4; continent: Europe; country: Spain; countryCode: ES; stateProvince: Castilla-La Mancha; county: Ciudad Real; locality: La Quesera; verbatimElevation: 772.3; decimalLatitude: 39.36337; decimalLongitude: -4.41704; geodeticDatum: WGS84; **Event:** eventID: 1; samplingProtocol: Aerial; eventTime: Night
- **Type status:** Other material. **Occurrence:** individualCount: 1; sex: female; **Location:** locationID: M2; continent: Europe; country: Spain; countryCode: ES; stateProvince: Extremadura; county: Cáceres; locality: Fuente del Frances; verbatimElevation: 320.72; decimalLatitude: 39.828; decimalLongitude: -6.03249; geodeticDatum: WGS84; **Event:** eventID: 1; samplingProtocol: Beating; eventTime: Day
- **Type status:** Other material. **Occurrence:** individualCount: 1; sex: female; **Location:** locationID: S2; continent: Europe; country: Spain; countryCode: ES; stateProvince: Andalucía; county: Granada; locality: Camarate; verbatimElevation: 1713.96; decimalLatitude: 37.18377; decimalLongitude: -3.26282; geodeticDatum: WGS84; **Event:** eventID: 2; samplingProtocol: Beating; eventTime: Night
- **Type status:** Other material. **Occurrence:** individualCount: 1; sex: female; **Location:** locationID: S2; continent: Europe; country: Spain; countryCode: ES; stateProvince: Andalucía; county: Granada; locality: Camarate; verbatimElevation: 1713.96; decimalLatitude: 37.18377; decimalLongitude: -3.26282; geodeticDatum: WGS84; **Event:** eventID: 1; samplingProtocol: Sweeping; eventTime: Night

##### Distribution

?

##### Notes

This is a species of *Nigma* Lehtninen, 1967, which we were unable to identify.

#### Dysderidae

C. L. Koch, 1837

#### Dysdera erythrina

(Walckenaer, 1802)

##### Materials

- **Type status:** Other material. **Occurrence:** individualCount: 1; sex: male; **Location:** locationID: A1; continent: Europe; country: Spain; countryCode: ES; stateProvince: Catalonia; county: Lleida; locality: Sola de Boi; verbatimElevation: 1759.8; decimalLatitude: 42.54958; decimalLongitude: 0.87254; geodeticDatum: WGS84; **Event:** eventID: A; samplingProtocol: Pitfall
- **Type status:** Other material. **Occurrence:** individualCount: 2; sex: male; **Location:** locationID: A1; continent: Europe; country: Spain; countryCode: ES; stateProvince: Catalonia; county: Lleida; locality: Sola de Boi; verbatimElevation: 1759.8; decimalLatitude: 42.54958; decimalLongitude: 0.87254; geodeticDatum: WGS84; **Event:** eventID: L; samplingProtocol: Pitfall
- **Type status:** Other material. **Occurrence:** individualCount: 1; sex: female; **Location:** locationID: A1; continent: Europe; country: Spain; countryCode: ES; stateProvince: Catalonia; county: Lleida; locality: Sola de Boi; verbatimElevation: 1759.8; decimalLatitude: 42.54958; decimalLongitude: 0.87254; geodeticDatum: WGS84; **Event:** eventID: L; samplingProtocol: Pitfall
- **Type status:** Other material. **Occurrence:** individualCount: 1; sex: male; **Location:** locationID: A2; continent: Europe; country: Spain; countryCode: ES; stateProvince: Catalonia; county: Lleida; locality: Sola de Boi; verbatimElevation: 1738.7; decimalLatitude: 42.54913; decimalLongitude: 0.87137; geodeticDatum: WGS84; **Event:** eventID: A; samplingProtocol: Pitfall
- **Type status:** Other material. **Occurrence:** individualCount: 1; sex: female; **Location:** locationID: A2; continent: Europe; country: Spain; countryCode: ES; stateProvince: Catalonia; county: Lleida; locality: Sola de Boi; verbatimElevation: 1738.7; decimalLatitude: 42.54913; decimalLongitude: 0.87137; geodeticDatum: WGS84; **Event:** eventID: L; samplingProtocol: Pitfall

##### Distribution

Europe to Georgia

#### Dysdera falciformis

Barrientos & Ferrández, 1982

##### Materials

- **Type status:** Other material. **Occurrence:** individualCount: 1; sex: male; **Location:** locationID: M2; continent: Europe; country: Spain; countryCode: ES; stateProvince: Extremadura; county: Cáceres; locality: Fuente del Frances; verbatimElevation: 320.72; decimalLatitude: 39.828; decimalLongitude: -6.03249; geodeticDatum: WGS84; **Event:** eventID: H; samplingProtocol: Pitfall

##### Distribution

Spain

#### Dysdera fuscipes

Simon, 1882

##### Materials

- **Type status:** Other material. **Occurrence:** individualCount: 1; sex: male; **Location:** locationID: P4; continent: Europe; country: Spain; countryCode: ES; stateProvince: Castilla y León; county: León; locality: El Canto; verbatimElevation: 943.48; decimalLatitude: 43.17227; decimalLongitude: -4.90857; geodeticDatum: WGS84; **Event:** eventID: J; samplingProtocol: Pitfall

##### Distribution

Iberian Peninsula, France

#### Dysdera gamarrae

Ferrández, 1984

##### Materials

- **Type status:** Other material. **Occurrence:** individualCount: 1; sex: male; **Location:** locationID: C1; continent: Europe; country: Spain; countryCode: ES; stateProvince: Castilla-La Mancha; county: Ciudad Real; locality: Valle Brezoso; verbatimElevation: 756.56; decimalLatitude: 39.35663; decimalLongitude: -4.35912; geodeticDatum: WGS84; **Event:** eventID: B; samplingProtocol: Pitfall
- **Type status:** Other material. **Occurrence:** individualCount: 1; sex: male; **Location:** locationID: C1; continent: Europe; country: Spain; countryCode: ES; stateProvince: Castilla-La Mancha; county: Ciudad Real; locality: Valle Brezoso; verbatimElevation: 756.56; decimalLatitude: 39.35663; decimalLongitude: -4.35912; geodeticDatum: WGS84; **Event:** eventID: D; samplingProtocol: Pitfall
- **Type status:** Other material. **Occurrence:** individualCount: 1; sex: male; **Location:** locationID: C4; continent: Europe; country: Spain; countryCode: ES; stateProvince: Castilla-La Mancha; county: Ciudad Real; locality: La Quesera; verbatimElevation: 772.3; decimalLatitude: 39.36337; decimalLongitude: -4.41704; geodeticDatum: WGS84; **Event:** eventID: G; samplingProtocol: Pitfall

##### Distribution

Iberian Peninsula

#### Dysdera sp03

##### Materials

- **Type status:** Other material. **Occurrence:** individualCount: 1; sex: male; **Location:** locationID: O1; continent: Europe; country: Spain; countryCode: ES; stateProvince: Aragón; county: Huesca; locality: O Furno; verbatimElevation: 1396.73; decimalLatitude: 42.60677; decimalLongitude: 0.13135; geodeticDatum: WGS84; **Event:** eventID: 1; samplingProtocol: Beating; eventTime: Night
- **Type status:** Other material. **Occurrence:** individualCount: 1; sex: male; **Location:** locationID: O1; continent: Europe; country: Spain; countryCode: ES; stateProvince: Aragón; county: Huesca; locality: O Furno; verbatimElevation: 1396.73; decimalLatitude: 42.60677; decimalLongitude: 0.13135; geodeticDatum: WGS84; **Event:** eventID: B; samplingProtocol: Pitfall
- **Type status:** Other material. **Occurrence:** individualCount: 3; sex: male; **Location:** locationID: O2; continent: Europe; country: Spain; countryCode: ES; stateProvince: Aragón; county: Huesca; locality: Rebilla; verbatimElevation: 1158.13; decimalLatitude: 42.59427; decimalLongitude: 0.1529; geodeticDatum: WGS84; **Event:** eventID: K; samplingProtocol: Pitfall

##### Distribution

?

##### Notes

This is a new species of *Dysdera* Latreille, 1804, to be described in a future publication.

#### Dysdera sp08

##### Materials

- **Type status:** Other material. **Occurrence:** individualCount: 1; sex: female; **Location:** locationID: P2; continent: Europe; country: Spain; countryCode: ES; stateProvince: Castilla y León; county: León; locality: Joyoguelas; verbatimElevation: 763.98; decimalLatitude: 43.17771; decimalLongitude: -4.90579; geodeticDatum: WGS84; **Event:** eventID: B; samplingProtocol: Pitfall
- **Type status:** Other material. **Occurrence:** individualCount: 1; sex: male; **Location:** locationID: P2; continent: Europe; country: Spain; countryCode: ES; stateProvince: Castilla y León; county: León; locality: Joyoguelas; verbatimElevation: 763.98; decimalLatitude: 43.17771; decimalLongitude: -4.90579; geodeticDatum: WGS84; **Event:** eventID: D; samplingProtocol: Pitfall
- **Type status:** Other material. **Occurrence:** individualCount: 6; sex: male; **Location:** locationID: P2; continent: Europe; country: Spain; countryCode: ES; stateProvince: Castilla y León; county: León; locality: Joyoguelas; verbatimElevation: 763.98; decimalLatitude: 43.17771; decimalLongitude: -4.90579; geodeticDatum: WGS84; **Event:** eventID: E; samplingProtocol: Pitfall
- **Type status:** Other material. **Occurrence:** individualCount: 1; sex: male; **Location:** locationID: P2; continent: Europe; country: Spain; countryCode: ES; stateProvince: Castilla y León; county: León; locality: Joyoguelas; verbatimElevation: 763.98; decimalLatitude: 43.17771; decimalLongitude: -4.90579; geodeticDatum: WGS84; **Event:** eventID: F; samplingProtocol: Pitfall
- **Type status:** Other material. **Occurrence:** individualCount: 1; sex: female; **Location:** locationID: P2; continent: Europe; country: Spain; countryCode: ES; stateProvince: Castilla y León; county: León; locality: Joyoguelas; verbatimElevation: 763.98; decimalLatitude: 43.17771; decimalLongitude: -4.90579; geodeticDatum: WGS84; **Event:** eventID: G; samplingProtocol: Pitfall
- **Type status:** Other material. **Occurrence:** individualCount: 1; sex: female; **Location:** locationID: P3; continent: Europe; country: Spain; countryCode: ES; stateProvince: Castilla y León; county: León; locality: Las Arroyas; verbatimElevation: 1097.1; decimalLatitude: 43.14351; decimalLongitude: -4.94878; geodeticDatum: WGS84; **Event:** eventID: D; samplingProtocol: Pitfall
- **Type status:** Other material. **Occurrence:** individualCount: 1; sex: male; **Location:** locationID: P3; continent: Europe; country: Spain; countryCode: ES; stateProvince: Castilla y León; county: León; locality: Las Arroyas; verbatimElevation: 1097.1; decimalLatitude: 43.14351; decimalLongitude: -4.94878; geodeticDatum: WGS84; **Event:** eventID: E; samplingProtocol: Pitfall
- **Type status:** Other material. **Occurrence:** individualCount: 1; sex: male; **Location:** locationID: P3; continent: Europe; country: Spain; countryCode: ES; stateProvince: Castilla y León; county: León; locality: Las Arroyas; verbatimElevation: 1097.1; decimalLatitude: 43.14351; decimalLongitude: -4.94878; geodeticDatum: WGS84; **Event:** eventID: H; samplingProtocol: Pitfall
- **Type status:** Other material. **Occurrence:** individualCount: 1; sex: female; **Location:** locationID: P3; continent: Europe; country: Spain; countryCode: ES; stateProvince: Castilla y León; county: León; locality: Las Arroyas; verbatimElevation: 1097.1; decimalLatitude: 43.14351; decimalLongitude: -4.94878; geodeticDatum: WGS84; **Event:** eventID: H; samplingProtocol: Pitfall
- **Type status:** Other material. **Occurrence:** individualCount: 1; sex: female; **Location:** locationID: P3; continent: Europe; country: Spain; countryCode: ES; stateProvince: Castilla y León; county: León; locality: Las Arroyas; verbatimElevation: 1097.1; decimalLatitude: 43.14351; decimalLongitude: -4.94878; geodeticDatum: WGS84; **Event:** eventID: J; samplingProtocol: Pitfall

##### Distribution

?

##### Notes

This is a new species of *Dysdera*, to be described in a future publication.

#### Dysdera sp33

##### Materials

- **Type status:** Other material. **Occurrence:** individualCount: 1; sex: female; **Location:** locationID: S1; continent: Europe; country: Spain; countryCode: ES; stateProvince: Andalucía; county: Granada; locality: Soportujar; verbatimElevation: 1786.57; decimalLatitude: 36.96151; decimalLongitude: -3.41881; geodeticDatum: WGS84; **Event:** eventID: K; samplingProtocol: Pitfall

##### Distribution

?

##### Notes

This is a species of *Dysdera*, which we were unable to identify.

#### Dysdera sp39

##### Materials

- **Type status:** Other material. **Occurrence:** individualCount: 1; sex: male; **Location:** locationID: S1; continent: Europe; country: Spain; countryCode: ES; stateProvince: Andalucía; county: Granada; locality: Soportujar; verbatimElevation: 1786.57; decimalLatitude: 36.96151; decimalLongitude: -3.41881; geodeticDatum: WGS84; **Event:** eventID: J; samplingProtocol: Pitfall

##### Distribution

?

##### Notes

This is a species of *Dysdera*, which we were unable to identify.

#### Harpactea fageli

Brignoli, 1980

##### Materials

- **Type status:** Other material. **Occurrence:** individualCount: 1; sex: male; **Location:** locationID: M2; continent: Europe; country: Spain; countryCode: ES; stateProvince: Extremadura; county: Cáceres; locality: Fuente del Frances; verbatimElevation: 320.72; decimalLatitude: 39.828; decimalLongitude: -6.03249; geodeticDatum: WGS84; **Event:** eventID: C; samplingProtocol: Pitfall
- **Type status:** Other material. **Occurrence:** individualCount: 1; sex: male; **Location:** locationID: M2; continent: Europe; country: Spain; countryCode: ES; stateProvince: Extremadura; county: Cáceres; locality: Fuente del Frances; verbatimElevation: 320.72; decimalLatitude: 39.828; decimalLongitude: -6.03249; geodeticDatum: WGS84; **Event:** eventID: F; samplingProtocol: Pitfall
- **Type status:** Other material. **Occurrence:** individualCount: 1; sex: female; **Location:** locationID: M2; continent: Europe; country: Spain; countryCode: ES; stateProvince: Extremadura; county: Cáceres; locality: Fuente del Frances; verbatimElevation: 320.72; decimalLatitude: 39.828; decimalLongitude: -6.03249; geodeticDatum: WGS84; **Event:** eventID: H; samplingProtocol: Pitfall

##### Distribution

Iberian Peninsula

#### Harpactea hombergi

(Scopoli, 1763)

##### Materials

- **Type status:** Other material. **Occurrence:** individualCount: 3; sex: female; **Location:** locationID: A1; continent: Europe; country: Spain; countryCode: ES; stateProvince: Catalonia; county: Lleida; locality: Sola de Boi; verbatimElevation: 1759.8; decimalLatitude: 42.54958; decimalLongitude: 0.87254; geodeticDatum: WGS84; **Event:** eventID: 2; samplingProtocol: Aerial; eventTime: Night
- **Type status:** Other material. **Occurrence:** individualCount: 3; sex: male; **Location:** locationID: A1; continent: Europe; country: Spain; countryCode: ES; stateProvince: Catalonia; county: Lleida; locality: Sola de Boi; verbatimElevation: 1759.8; decimalLatitude: 42.54958; decimalLongitude: 0.87254; geodeticDatum: WGS84; **Event:** eventID: 2; samplingProtocol: Ground; eventTime: Night
- **Type status:** Other material. **Occurrence:** individualCount: 1; sex: female; **Location:** locationID: A1; continent: Europe; country: Spain; countryCode: ES; stateProvince: Catalonia; county: Lleida; locality: Sola de Boi; verbatimElevation: 1759.8; decimalLatitude: 42.54958; decimalLongitude: 0.87254; geodeticDatum: WGS84; **Event:** eventID: 2; samplingProtocol: Ground; eventTime: Night
- **Type status:** Other material. **Occurrence:** individualCount: 1; sex: male; **Location:** locationID: A1; continent: Europe; country: Spain; countryCode: ES; stateProvince: Catalonia; county: Lleida; locality: Sola de Boi; verbatimElevation: 1759.8; decimalLatitude: 42.54958; decimalLongitude: 0.87254; geodeticDatum: WGS84; **Event:** eventID: A; samplingProtocol: Pitfall
- **Type status:** Other material. **Occurrence:** individualCount: 1; sex: male; **Location:** locationID: A1; continent: Europe; country: Spain; countryCode: ES; stateProvince: Catalonia; county: Lleida; locality: Sola de Boi; verbatimElevation: 1759.8; decimalLatitude: 42.54958; decimalLongitude: 0.87254; geodeticDatum: WGS84; **Event:** eventID: B; samplingProtocol: Pitfall
- **Type status:** Other material. **Occurrence:** individualCount: 1; sex: male; **Location:** locationID: A1; continent: Europe; country: Spain; countryCode: ES; stateProvince: Catalonia; county: Lleida; locality: Sola de Boi; verbatimElevation: 1759.8; decimalLatitude: 42.54958; decimalLongitude: 0.87254; geodeticDatum: WGS84; **Event:** eventID: C; samplingProtocol: Pitfall
- **Type status:** Other material. **Occurrence:** individualCount: 2; sex: male; **Location:** locationID: A1; continent: Europe; country: Spain; countryCode: ES; stateProvince: Catalonia; county: Lleida; locality: Sola de Boi; verbatimElevation: 1759.8; decimalLatitude: 42.54958; decimalLongitude: 0.87254; geodeticDatum: WGS84; **Event:** eventID: E; samplingProtocol: Pitfall
- **Type status:** Other material. **Occurrence:** individualCount: 1; sex: female; **Location:** locationID: A1; continent: Europe; country: Spain; countryCode: ES; stateProvince: Catalonia; county: Lleida; locality: Sola de Boi; verbatimElevation: 1759.8; decimalLatitude: 42.54958; decimalLongitude: 0.87254; geodeticDatum: WGS84; **Event:** eventID: G; samplingProtocol: Pitfall
- **Type status:** Other material. **Occurrence:** individualCount: 2; sex: male; **Location:** locationID: A1; continent: Europe; country: Spain; countryCode: ES; stateProvince: Catalonia; county: Lleida; locality: Sola de Boi; verbatimElevation: 1759.8; decimalLatitude: 42.54958; decimalLongitude: 0.87254; geodeticDatum: WGS84; **Event:** eventID: H; samplingProtocol: Pitfall
- **Type status:** Other material. **Occurrence:** individualCount: 1; sex: male; **Location:** locationID: A2; continent: Europe; country: Spain; countryCode: ES; stateProvince: Catalonia; county: Lleida; locality: Sola de Boi; verbatimElevation: 1738.7; decimalLatitude: 42.54913; decimalLongitude: 0.87137; geodeticDatum: WGS84; **Event:** eventID: 2; samplingProtocol: Aerial; eventTime: Night
- **Type status:** Other material. **Occurrence:** individualCount: 1; sex: male; **Location:** locationID: A2; continent: Europe; country: Spain; countryCode: ES; stateProvince: Catalonia; county: Lleida; locality: Sola de Boi; verbatimElevation: 1738.7; decimalLatitude: 42.54913; decimalLongitude: 0.87137; geodeticDatum: WGS84; **Event:** eventID: A; samplingProtocol: Pitfall
- **Type status:** Other material. **Occurrence:** individualCount: 1; sex: female; **Location:** locationID: A2; continent: Europe; country: Spain; countryCode: ES; stateProvince: Catalonia; county: Lleida; locality: Sola de Boi; verbatimElevation: 1738.7; decimalLatitude: 42.54913; decimalLongitude: 0.87137; geodeticDatum: WGS84; **Event:** eventID: B; samplingProtocol: Pitfall
- **Type status:** Other material. **Occurrence:** individualCount: 1; sex: male; **Location:** locationID: A2; continent: Europe; country: Spain; countryCode: ES; stateProvince: Catalonia; county: Lleida; locality: Sola de Boi; verbatimElevation: 1738.7; decimalLatitude: 42.54913; decimalLongitude: 0.87137; geodeticDatum: WGS84; **Event:** eventID: D; samplingProtocol: Pitfall
- **Type status:** Other material. **Occurrence:** individualCount: 1; sex: male; **Location:** locationID: A2; continent: Europe; country: Spain; countryCode: ES; stateProvince: Catalonia; county: Lleida; locality: Sola de Boi; verbatimElevation: 1738.7; decimalLatitude: 42.54913; decimalLongitude: 0.87137; geodeticDatum: WGS84; **Event:** eventID: E; samplingProtocol: Pitfall
- **Type status:** Other material. **Occurrence:** individualCount: 1; sex: male; **Location:** locationID: A2; continent: Europe; country: Spain; countryCode: ES; stateProvince: Catalonia; county: Lleida; locality: Sola de Boi; verbatimElevation: 1738.7; decimalLatitude: 42.54913; decimalLongitude: 0.87137; geodeticDatum: WGS84; **Event:** eventID: G; samplingProtocol: Pitfall
- **Type status:** Other material. **Occurrence:** individualCount: 2; sex: male; **Location:** locationID: A2; continent: Europe; country: Spain; countryCode: ES; stateProvince: Catalonia; county: Lleida; locality: Sola de Boi; verbatimElevation: 1738.7; decimalLatitude: 42.54913; decimalLongitude: 0.87137; geodeticDatum: WGS84; **Event:** eventID: L; samplingProtocol: Pitfall
- **Type status:** Other material. **Occurrence:** individualCount: 2; sex: female; **Location:** locationID: A2; continent: Europe; country: Spain; countryCode: ES; stateProvince: Catalonia; county: Lleida; locality: Sola de Boi; verbatimElevation: 1738.7; decimalLatitude: 42.54913; decimalLongitude: 0.87137; geodeticDatum: WGS84; **Event:** eventID: L; samplingProtocol: Pitfall
- **Type status:** Other material. **Occurrence:** individualCount: 2; sex: male; **Location:** locationID: O1; continent: Europe; country: Spain; countryCode: ES; stateProvince: Aragón; county: Huesca; locality: O Furno; verbatimElevation: 1396.73; decimalLatitude: 42.60677; decimalLongitude: 0.13135; geodeticDatum: WGS84; **Event:** eventID: 2; samplingProtocol: Aerial; eventTime: Night
- **Type status:** Other material. **Occurrence:** individualCount: 1; sex: male; **Location:** locationID: O2; continent: Europe; country: Spain; countryCode: ES; stateProvince: Aragón; county: Huesca; locality: Rebilla; verbatimElevation: 1158.13; decimalLatitude: 42.59427; decimalLongitude: 0.1529; geodeticDatum: WGS84; **Event:** eventID: 1; samplingProtocol: Aerial; eventTime: Night
- **Type status:** Other material. **Occurrence:** individualCount: 2; sex: male; **Location:** locationID: P1; continent: Europe; country: Spain; countryCode: ES; stateProvince: Castilla y León; county: León; locality: Monte Robledo; verbatimElevation: 1071.58; decimalLatitude: 43.1445; decimalLongitude: -4.92675; geodeticDatum: WGS84; **Event:** eventID: 1; samplingProtocol: Aerial; eventTime: Night
- **Type status:** Other material. **Occurrence:** individualCount: 1; sex: male; **Location:** locationID: P1; continent: Europe; country: Spain; countryCode: ES; stateProvince: Castilla y León; county: León; locality: Monte Robledo; verbatimElevation: 1071.58; decimalLatitude: 43.1445; decimalLongitude: -4.92675; geodeticDatum: WGS84; **Event:** eventID: 1; samplingProtocol: Aerial; eventTime: Night
- **Type status:** Other material. **Occurrence:** individualCount: 1; sex: female; **Location:** locationID: P1; continent: Europe; country: Spain; countryCode: ES; stateProvince: Castilla y León; county: León; locality: Monte Robledo; verbatimElevation: 1071.58; decimalLatitude: 43.1445; decimalLongitude: -4.92675; geodeticDatum: WGS84; **Event:** eventID: 1; samplingProtocol: Aerial; eventTime: Night
- **Type status:** Other material. **Occurrence:** individualCount: 1; sex: male; **Location:** locationID: P2; continent: Europe; country: Spain; countryCode: ES; stateProvince: Castilla y León; county: León; locality: Joyoguelas; verbatimElevation: 763.98; decimalLatitude: 43.17771; decimalLongitude: -4.90579; geodeticDatum: WGS84; **Event:** eventID: 1; samplingProtocol: Aerial; eventTime: Night
- **Type status:** Other material. **Occurrence:** individualCount: 1; sex: male; **Location:** locationID: P2; continent: Europe; country: Spain; countryCode: ES; stateProvince: Castilla y León; county: León; locality: Joyoguelas; verbatimElevation: 763.98; decimalLatitude: 43.17771; decimalLongitude: -4.90579; geodeticDatum: WGS84; **Event:** eventID: A; samplingProtocol: Pitfall
- **Type status:** Other material. **Occurrence:** individualCount: 1; sex: female; **Location:** locationID: P2; continent: Europe; country: Spain; countryCode: ES; stateProvince: Castilla y León; county: León; locality: Joyoguelas; verbatimElevation: 763.98; decimalLatitude: 43.17771; decimalLongitude: -4.90579; geodeticDatum: WGS84; **Event:** eventID: C; samplingProtocol: Pitfall
- **Type status:** Other material. **Occurrence:** individualCount: 1; sex: male; **Location:** locationID: P2; continent: Europe; country: Spain; countryCode: ES; stateProvince: Castilla y León; county: León; locality: Joyoguelas; verbatimElevation: 763.98; decimalLatitude: 43.17771; decimalLongitude: -4.90579; geodeticDatum: WGS84; **Event:** eventID: D; samplingProtocol: Pitfall
- **Type status:** Other material. **Occurrence:** individualCount: 1; sex: male; **Location:** locationID: P3; continent: Europe; country: Spain; countryCode: ES; stateProvince: Castilla y León; county: León; locality: Las Arroyas; verbatimElevation: 1097.1; decimalLatitude: 43.14351; decimalLongitude: -4.94878; geodeticDatum: WGS84; **Event:** eventID: 1; samplingProtocol: Aerial; eventTime: Night
- **Type status:** Other material. **Occurrence:** individualCount: 1; sex: male; **Location:** locationID: P3; continent: Europe; country: Spain; countryCode: ES; stateProvince: Castilla y León; county: León; locality: Las Arroyas; verbatimElevation: 1097.1; decimalLatitude: 43.14351; decimalLongitude: -4.94878; geodeticDatum: WGS84; **Event:** eventID: 2; samplingProtocol: Aerial; eventTime: Night
- **Type status:** Other material. **Occurrence:** individualCount: 1; sex: female; **Location:** locationID: P3; continent: Europe; country: Spain; countryCode: ES; stateProvince: Castilla y León; county: León; locality: Las Arroyas; verbatimElevation: 1097.1; decimalLatitude: 43.14351; decimalLongitude: -4.94878; geodeticDatum: WGS84; **Event:** eventID: 2; samplingProtocol: Aerial; eventTime: Night
- **Type status:** Other material. **Occurrence:** individualCount: 1; sex: male; **Location:** locationID: P3; continent: Europe; country: Spain; countryCode: ES; stateProvince: Castilla y León; county: León; locality: Las Arroyas; verbatimElevation: 1097.1; decimalLatitude: 43.14351; decimalLongitude: -4.94878; geodeticDatum: WGS84; **Event:** eventID: 2; samplingProtocol: Aerial; eventTime: Night
- **Type status:** Other material. **Occurrence:** individualCount: 1; sex: female; **Location:** locationID: P3; continent: Europe; country: Spain; countryCode: ES; stateProvince: Castilla y León; county: León; locality: Las Arroyas; verbatimElevation: 1097.1; decimalLatitude: 43.14351; decimalLongitude: -4.94878; geodeticDatum: WGS84; **Event:** eventID: 2; samplingProtocol: Aerial; eventTime: Night
- **Type status:** Other material. **Occurrence:** individualCount: 2; sex: male; **Location:** locationID: P4; continent: Europe; country: Spain; countryCode: ES; stateProvince: Castilla y León; county: León; locality: El Canto; verbatimElevation: 943.48; decimalLatitude: 43.17227; decimalLongitude: -4.90857; geodeticDatum: WGS84; **Event:** eventID: 2; samplingProtocol: Aerial; eventTime: Night
- **Type status:** Other material. **Occurrence:** individualCount: 1; sex: male; **Location:** locationID: P4; continent: Europe; country: Spain; countryCode: ES; stateProvince: Castilla y León; county: León; locality: El Canto; verbatimElevation: 943.48; decimalLatitude: 43.17227; decimalLongitude: -4.90857; geodeticDatum: WGS84; **Event:** eventID: D; samplingProtocol: Pitfall
- **Type status:** Other material. **Occurrence:** individualCount: 1; sex: female; **Location:** locationID: P4; continent: Europe; country: Spain; countryCode: ES; stateProvince: Castilla y León; county: León; locality: El Canto; verbatimElevation: 943.48; decimalLatitude: 43.17227; decimalLongitude: -4.90857; geodeticDatum: WGS84; **Event:** eventID: E; samplingProtocol: Pitfall
- **Type status:** Other material. **Occurrence:** individualCount: 1; sex: male; **Location:** locationID: P4; continent: Europe; country: Spain; countryCode: ES; stateProvince: Castilla y León; county: León; locality: El Canto; verbatimElevation: 943.48; decimalLatitude: 43.17227; decimalLongitude: -4.90857; geodeticDatum: WGS84; **Event:** eventID: I; samplingProtocol: Pitfall

##### Distribution

Europe to Ukraine

#### Harpactea sp16

##### Materials

- **Type status:** Other material. **Occurrence:** individualCount: 2; sex: male; **Location:** locationID: C1; continent: Europe; country: Spain; countryCode: ES; stateProvince: Castilla-La Mancha; county: Ciudad Real; locality: Valle Brezoso; verbatimElevation: 756.56; decimalLatitude: 39.35663; decimalLongitude: -4.35912; geodeticDatum: WGS84; **Event:** eventID: B; samplingProtocol: Pitfall
- **Type status:** Other material. **Occurrence:** individualCount: 1; sex: female; **Location:** locationID: C1; continent: Europe; country: Spain; countryCode: ES; stateProvince: Castilla-La Mancha; county: Ciudad Real; locality: Valle Brezoso; verbatimElevation: 756.56; decimalLatitude: 39.35663; decimalLongitude: -4.35912; geodeticDatum: WGS84; **Event:** eventID: B; samplingProtocol: Pitfall
- **Type status:** Other material. **Occurrence:** individualCount: 1; sex: male; **Location:** locationID: C1; continent: Europe; country: Spain; countryCode: ES; stateProvince: Castilla-La Mancha; county: Ciudad Real; locality: Valle Brezoso; verbatimElevation: 756.56; decimalLatitude: 39.35663; decimalLongitude: -4.35912; geodeticDatum: WGS84; **Event:** eventID: D; samplingProtocol: Pitfall
- **Type status:** Other material. **Occurrence:** individualCount: 1; sex: female; **Location:** locationID: C1; continent: Europe; country: Spain; countryCode: ES; stateProvince: Castilla-La Mancha; county: Ciudad Real; locality: Valle Brezoso; verbatimElevation: 756.56; decimalLatitude: 39.35663; decimalLongitude: -4.35912; geodeticDatum: WGS84; **Event:** eventID: E; samplingProtocol: Pitfall
- **Type status:** Other material. **Occurrence:** individualCount: 4; sex: male; **Location:** locationID: C1; continent: Europe; country: Spain; countryCode: ES; stateProvince: Castilla-La Mancha; county: Ciudad Real; locality: Valle Brezoso; verbatimElevation: 756.56; decimalLatitude: 39.35663; decimalLongitude: -4.35912; geodeticDatum: WGS84; **Event:** eventID: F; samplingProtocol: Pitfall
- **Type status:** Other material. **Occurrence:** individualCount: 10; sex: male; **Location:** locationID: C1; continent: Europe; country: Spain; countryCode: ES; stateProvince: Castilla-La Mancha; county: Ciudad Real; locality: Valle Brezoso; verbatimElevation: 756.56; decimalLatitude: 39.35663; decimalLongitude: -4.35912; geodeticDatum: WGS84; **Event:** eventID: G; samplingProtocol: Pitfall
- **Type status:** Other material. **Occurrence:** individualCount: 2; sex: female; **Location:** locationID: C1; continent: Europe; country: Spain; countryCode: ES; stateProvince: Castilla-La Mancha; county: Ciudad Real; locality: Valle Brezoso; verbatimElevation: 756.56; decimalLatitude: 39.35663; decimalLongitude: -4.35912; geodeticDatum: WGS84; **Event:** eventID: G; samplingProtocol: Pitfall
- **Type status:** Other material. **Occurrence:** individualCount: 1; sex: male; **Location:** locationID: C1; continent: Europe; country: Spain; countryCode: ES; stateProvince: Castilla-La Mancha; county: Ciudad Real; locality: Valle Brezoso; verbatimElevation: 756.56; decimalLatitude: 39.35663; decimalLongitude: -4.35912; geodeticDatum: WGS84; **Event:** eventID: I; samplingProtocol: Pitfall
- **Type status:** Other material. **Occurrence:** individualCount: 1; sex: female; **Location:** locationID: C1; continent: Europe; country: Spain; countryCode: ES; stateProvince: Castilla-La Mancha; county: Ciudad Real; locality: Valle Brezoso; verbatimElevation: 756.56; decimalLatitude: 39.35663; decimalLongitude: -4.35912; geodeticDatum: WGS84; **Event:** eventID: I; samplingProtocol: Pitfall
- **Type status:** Other material. **Occurrence:** individualCount: 1; sex: male; **Location:** locationID: C1; continent: Europe; country: Spain; countryCode: ES; stateProvince: Castilla-La Mancha; county: Ciudad Real; locality: Valle Brezoso; verbatimElevation: 756.56; decimalLatitude: 39.35663; decimalLongitude: -4.35912; geodeticDatum: WGS84; **Event:** eventID: J; samplingProtocol: Pitfall
- **Type status:** Other material. **Occurrence:** individualCount: 1; sex: female; **Location:** locationID: C1; continent: Europe; country: Spain; countryCode: ES; stateProvince: Castilla-La Mancha; county: Ciudad Real; locality: Valle Brezoso; verbatimElevation: 756.56; decimalLatitude: 39.35663; decimalLongitude: -4.35912; geodeticDatum: WGS84; **Event:** eventID: J; samplingProtocol: Pitfall
- **Type status:** Other material. **Occurrence:** individualCount: 1; sex: female; **Location:** locationID: C1; continent: Europe; country: Spain; countryCode: ES; stateProvince: Castilla-La Mancha; county: Ciudad Real; locality: Valle Brezoso; verbatimElevation: 756.56; decimalLatitude: 39.35663; decimalLongitude: -4.35912; geodeticDatum: WGS84; **Event:** eventID: K; samplingProtocol: Pitfall
- **Type status:** Other material. **Occurrence:** individualCount: 4; sex: male; **Location:** locationID: C1; continent: Europe; country: Spain; countryCode: ES; stateProvince: Castilla-La Mancha; county: Ciudad Real; locality: Valle Brezoso; verbatimElevation: 756.56; decimalLatitude: 39.35663; decimalLongitude: -4.35912; geodeticDatum: WGS84; **Event:** eventID: L; samplingProtocol: Pitfall
- **Type status:** Other material. **Occurrence:** individualCount: 3; sex: male; **Location:** locationID: C3; continent: Europe; country: Spain; countryCode: ES; stateProvince: Castilla-La Mancha; county: Ciudad Real; locality: La Quesera; verbatimElevation: 767.55; decimalLatitude: 39.36177; decimalLongitude: -4.41733; geodeticDatum: WGS84; **Event:** eventID: L; samplingProtocol: Pitfall
- **Type status:** Other material. **Occurrence:** individualCount: 1; sex: male; **Location:** locationID: C4; continent: Europe; country: Spain; countryCode: ES; stateProvince: Castilla-La Mancha; county: Ciudad Real; locality: La Quesera; verbatimElevation: 772.3; decimalLatitude: 39.36337; decimalLongitude: -4.41704; geodeticDatum: WGS84; **Event:** eventID: J; samplingProtocol: Pitfall
- **Type status:** Other material. **Occurrence:** individualCount: 2; sex: male; **Location:** locationID: S1; continent: Europe; country: Spain; countryCode: ES; stateProvince: Andalucía; county: Granada; locality: Soportujar; verbatimElevation: 1786.57; decimalLatitude: 36.96151; decimalLongitude: -3.41881; geodeticDatum: WGS84; **Event:** eventID: I; samplingProtocol: Pitfall
- **Type status:** Other material. **Occurrence:** individualCount: 1; sex: male; **Location:** locationID: S2; continent: Europe; country: Spain; countryCode: ES; stateProvince: Andalucía; county: Granada; locality: Camarate; verbatimElevation: 1713.96; decimalLatitude: 37.18377; decimalLongitude: -3.26282; geodeticDatum: WGS84; **Event:** eventID: A; samplingProtocol: Pitfall
- **Type status:** Other material. **Occurrence:** individualCount: 1; sex: male; **Location:** locationID: S2; continent: Europe; country: Spain; countryCode: ES; stateProvince: Andalucía; county: Granada; locality: Camarate; verbatimElevation: 1713.96; decimalLatitude: 37.18377; decimalLongitude: -3.26282; geodeticDatum: WGS84; **Event:** eventID: B; samplingProtocol: Pitfall
- **Type status:** Other material. **Occurrence:** individualCount: 1; sex: male; **Location:** locationID: S2; continent: Europe; country: Spain; countryCode: ES; stateProvince: Andalucía; county: Granada; locality: Camarate; verbatimElevation: 1713.96; decimalLatitude: 37.18377; decimalLongitude: -3.26282; geodeticDatum: WGS84; **Event:** eventID: D; samplingProtocol: Pitfall
- **Type status:** Other material. **Occurrence:** individualCount: 2; sex: female; **Location:** locationID: S2; continent: Europe; country: Spain; countryCode: ES; stateProvince: Andalucía; county: Granada; locality: Camarate; verbatimElevation: 1713.96; decimalLatitude: 37.18377; decimalLongitude: -3.26282; geodeticDatum: WGS84; **Event:** eventID: D; samplingProtocol: Pitfall
- **Type status:** Other material. **Occurrence:** individualCount: 2; sex: male; **Location:** locationID: S2; continent: Europe; country: Spain; countryCode: ES; stateProvince: Andalucía; county: Granada; locality: Camarate; verbatimElevation: 1713.96; decimalLatitude: 37.18377; decimalLongitude: -3.26282; geodeticDatum: WGS84; **Event:** eventID: I; samplingProtocol: Pitfall
- **Type status:** Other material. **Occurrence:** individualCount: 2; sex: male; **Location:** locationID: S2; continent: Europe; country: Spain; countryCode: ES; stateProvince: Andalucía; county: Granada; locality: Camarate; verbatimElevation: 1713.96; decimalLatitude: 37.18377; decimalLongitude: -3.26282; geodeticDatum: WGS84; **Event:** eventID: K; samplingProtocol: Pitfall

##### Distribution

?

##### Notes

This is a species of *Harpactea* Bristowe, 1939, which we were unable to identify.

#### Harpactea sp21

##### Materials

- **Type status:** Other material. **Occurrence:** individualCount: 1; sex: female; **Location:** locationID: M1; continent: Europe; country: Spain; countryCode: ES; stateProvince: Extremadura; county: Cáceres; locality: Peña Falcón; verbatimElevation: 320.6; decimalLatitude: 39.83296; decimalLongitude: -6.0641; geodeticDatum: WGS84; **Event:** eventID: I; samplingProtocol: Pitfall

##### Distribution

?

##### Notes

This is a species of *Harpactea*, which we were unable to identify.

#### Harpactea sp43

##### Materials

- **Type status:** Other material. **Occurrence:** individualCount: 1; sex: female; **Location:** locationID: S1; continent: Europe; country: Spain; countryCode: ES; stateProvince: Andalucía; county: Granada; locality: Soportujar; verbatimElevation: 1786.57; decimalLatitude: 36.96151; decimalLongitude: -3.41881; geodeticDatum: WGS84; **Event:** eventID: E; samplingProtocol: Pitfall

##### Distribution

?

##### Notes

This is a species of *Harpactea*, which we were unable to identify.

#### Harpactocrates sp10

##### Materials

- **Type status:** Other material. **Occurrence:** individualCount: 1; sex: female; **Location:** locationID: P1; continent: Europe; country: Spain; countryCode: ES; stateProvince: Castilla y León; county: León; locality: Monte Robledo; verbatimElevation: 1071.58; decimalLatitude: 43.1445; decimalLongitude: -4.92675; geodeticDatum: WGS84; **Event:** eventID: D; samplingProtocol: Pitfall
- **Type status:** Other material. **Occurrence:** individualCount: 1; sex: male; **Location:** locationID: P3; continent: Europe; country: Spain; countryCode: ES; stateProvince: Castilla y León; county: León; locality: Las Arroyas; verbatimElevation: 1097.1; decimalLatitude: 43.14351; decimalLongitude: -4.94878; geodeticDatum: WGS84; **Event:** eventID: J; samplingProtocol: Pitfall
- **Type status:** Other material. **Occurrence:** individualCount: 2; sex: male; **Location:** locationID: P3; continent: Europe; country: Spain; countryCode: ES; stateProvince: Castilla y León; county: León; locality: Las Arroyas; verbatimElevation: 1097.1; decimalLatitude: 43.14351; decimalLongitude: -4.94878; geodeticDatum: WGS84; **Event:** eventID: K; samplingProtocol: Pitfall
- **Type status:** Other material. **Occurrence:** individualCount: 1; sex: female; **Location:** locationID: P3; continent: Europe; country: Spain; countryCode: ES; stateProvince: Castilla y León; county: León; locality: Las Arroyas; verbatimElevation: 1097.1; decimalLatitude: 43.14351; decimalLongitude: -4.94878; geodeticDatum: WGS84; **Event:** eventID: 1; samplingProtocol: Aerial; eventTime: Night

##### Distribution

?

##### Notes

This is a new species of *Harpactocrates* Simon, 1914, to be described in a future publication.

#### Parachtes teruelis

(Kraus, 1955)

##### Materials

- **Type status:** Other material. **Occurrence:** individualCount: 1; sex: female; **Location:** locationID: O2; continent: Europe; country: Spain; countryCode: ES; stateProvince: Aragón; county: Huesca; locality: Rebilla; verbatimElevation: 1158.13; decimalLatitude: 42.59427; decimalLongitude: 0.1529; geodeticDatum: WGS84; **Event:** eventID: G; samplingProtocol: Pitfall
- **Type status:** Other material. **Occurrence:** individualCount: 1; sex: male; **Location:** locationID: O2; continent: Europe; country: Spain; countryCode: ES; stateProvince: Aragón; county: Huesca; locality: Rebilla; verbatimElevation: 1158.13; decimalLatitude: 42.59427; decimalLongitude: 0.1529; geodeticDatum: WGS84; **Event:** eventID: K; samplingProtocol: Pitfall
- **Type status:** Other material. **Occurrence:** individualCount: 1; sex: female; **Location:** locationID: O2; continent: Europe; country: Spain; countryCode: ES; stateProvince: Aragón; county: Huesca; locality: Rebilla; verbatimElevation: 1158.13; decimalLatitude: 42.59427; decimalLongitude: 0.1529; geodeticDatum: WGS84; **Event:** eventID: K; samplingProtocol: Pitfall

##### Distribution

Spain

#### Rhode scutiventris

Simon, 1882

##### Materials

- **Type status:** Other material. **Occurrence:** individualCount: 2; sex: female; **Location:** locationID: M1; continent: Europe; country: Spain; countryCode: ES; stateProvince: Extremadura; county: Cáceres; locality: Peña Falcón; verbatimElevation: 320.6; decimalLatitude: 39.83296; decimalLongitude: -6.0641; geodeticDatum: WGS84; **Event:** eventID: A; samplingProtocol: Pitfall
- **Type status:** Other material. **Occurrence:** individualCount: 2; sex: female; **Location:** locationID: M1; continent: Europe; country: Spain; countryCode: ES; stateProvince: Extremadura; county: Cáceres; locality: Peña Falcón; verbatimElevation: 320.6; decimalLatitude: 39.83296; decimalLongitude: -6.0641; geodeticDatum: WGS84; **Event:** eventID: B; samplingProtocol: Pitfall
- **Type status:** Other material. **Occurrence:** individualCount: 1; sex: male; **Location:** locationID: M1; continent: Europe; country: Spain; countryCode: ES; stateProvince: Extremadura; county: Cáceres; locality: Peña Falcón; verbatimElevation: 320.6; decimalLatitude: 39.83296; decimalLongitude: -6.0641; geodeticDatum: WGS84; **Event:** eventID: C; samplingProtocol: Pitfall
- **Type status:** Other material. **Occurrence:** individualCount: 1; sex: female; **Location:** locationID: M1; continent: Europe; country: Spain; countryCode: ES; stateProvince: Extremadura; county: Cáceres; locality: Peña Falcón; verbatimElevation: 320.6; decimalLatitude: 39.83296; decimalLongitude: -6.0641; geodeticDatum: WGS84; **Event:** eventID: D; samplingProtocol: Pitfall
- **Type status:** Other material. **Occurrence:** individualCount: 5; sex: female; **Location:** locationID: M1; continent: Europe; country: Spain; countryCode: ES; stateProvince: Extremadura; county: Cáceres; locality: Peña Falcón; verbatimElevation: 320.6; decimalLatitude: 39.83296; decimalLongitude: -6.0641; geodeticDatum: WGS84; **Event:** eventID: G; samplingProtocol: Pitfall
- **Type status:** Other material. **Occurrence:** individualCount: 2; sex: female; **Location:** locationID: M1; continent: Europe; country: Spain; countryCode: ES; stateProvince: Extremadura; county: Cáceres; locality: Peña Falcón; verbatimElevation: 320.6; decimalLatitude: 39.83296; decimalLongitude: -6.0641; geodeticDatum: WGS84; **Event:** eventID: H; samplingProtocol: Pitfall
- **Type status:** Other material. **Occurrence:** individualCount: 1; sex: female; **Location:** locationID: M1; continent: Europe; country: Spain; countryCode: ES; stateProvince: Extremadura; county: Cáceres; locality: Peña Falcón; verbatimElevation: 320.6; decimalLatitude: 39.83296; decimalLongitude: -6.0641; geodeticDatum: WGS84; **Event:** eventID: K; samplingProtocol: Pitfall
- **Type status:** Other material. **Occurrence:** individualCount: 2; sex: female; **Location:** locationID: M1; continent: Europe; country: Spain; countryCode: ES; stateProvince: Extremadura; county: Cáceres; locality: Peña Falcón; verbatimElevation: 320.6; decimalLatitude: 39.83296; decimalLongitude: -6.0641; geodeticDatum: WGS84; **Event:** eventID: L; samplingProtocol: Pitfall
- **Type status:** Other material. **Occurrence:** individualCount: 1; sex: male; **Location:** locationID: M2; continent: Europe; country: Spain; countryCode: ES; stateProvince: Extremadura; county: Cáceres; locality: Fuente del Frances; verbatimElevation: 320.72; decimalLatitude: 39.828; decimalLongitude: -6.03249; geodeticDatum: WGS84; **Event:** eventID: G; samplingProtocol: Pitfall
- **Type status:** Other material. **Occurrence:** individualCount: 1; sex: male; **Location:** locationID: M2; continent: Europe; country: Spain; countryCode: ES; stateProvince: Extremadura; county: Cáceres; locality: Fuente del Frances; verbatimElevation: 320.72; decimalLatitude: 39.828; decimalLongitude: -6.03249; geodeticDatum: WGS84; **Event:** eventID: J; samplingProtocol: Pitfall

##### Distribution

Iberian Peninsula, Morocco, Algeria

#### Eutichuridae

Lehtinen, 1967

#### Cheiracanthium elegans

Thorell, 1875

##### Materials

- **Type status:** Other material. **Occurrence:** individualCount: 1; sex: male; **Location:** locationID: P2; continent: Europe; country: Spain; countryCode: ES; stateProvince: Castilla y León; county: León; locality: Joyoguelas; verbatimElevation: 763.98; decimalLatitude: 43.17771; decimalLongitude: -4.90579; geodeticDatum: WGS84; **Event:** eventID: 2; samplingProtocol: Beating; eventTime: Day
- **Type status:** Other material. **Occurrence:** individualCount: 1; sex: female; **Location:** locationID: O1; continent: Europe; country: Spain; countryCode: ES; stateProvince: Aragón; county: Huesca; locality: O Furno; verbatimElevation: 1396.73; decimalLatitude: 42.60677; decimalLongitude: 0.13135; geodeticDatum: WGS84; **Event:** eventID: 2; samplingProtocol: Beating; eventTime: Day

##### Distribution

Europe to Central Asia

#### Cheiracanthium striolatum

Simon, 1878

##### Materials

- **Type status:** Other material. **Occurrence:** individualCount: 1; sex: female; **Location:** locationID: A2; continent: Europe; country: Spain; countryCode: ES; stateProvince: Catalonia; county: Lleida; locality: Sola de Boi; verbatimElevation: 1738.7; decimalLatitude: 42.54913; decimalLongitude: 0.87137; geodeticDatum: WGS84; **Event:** eventID: A; samplingProtocol: Pitfall
- **Type status:** Other material. **Occurrence:** individualCount: 1; sex: male; **Location:** locationID: S1; continent: Europe; country: Spain; countryCode: ES; stateProvince: Andalucía; county: Granada; locality: Soportujar; verbatimElevation: 1786.57; decimalLatitude: 36.96151; decimalLongitude: -3.41881; geodeticDatum: WGS84; **Event:** eventID: I; samplingProtocol: Pitfall

##### Distribution

Western Mediterranean

#### Cheiracanthium virescens

(Sundevall, 1833)

##### Materials

- **Type status:** Other material. **Occurrence:** individualCount: 1; sex: female; **Location:** locationID: P3; continent: Europe; country: Spain; countryCode: ES; stateProvince: Castilla y León; county: León; locality: Las Arroyas; verbatimElevation: 1097.1; decimalLatitude: 43.14351; decimalLongitude: -4.94878; geodeticDatum: WGS84; **Event:** eventID: 1; samplingProtocol: Beating; eventTime: Night

##### Distribution

Palearctic

#### Gnaphosidae

Pocock, 1898

#### Callilepis concolor

Simon, 1914

##### Materials

- **Type status:** Other material. **Occurrence:** individualCount: 3; sex: male; **Location:** locationID: C3; continent: Europe; country: Spain; countryCode: ES; stateProvince: Castilla-La Mancha; county: Ciudad Real; locality: La Quesera; verbatimElevation: 767.55; decimalLatitude: 39.36177; decimalLongitude: -4.41733; geodeticDatum: WGS84; **Event:** eventID: C; samplingProtocol: Pitfall
- **Type status:** Other material. **Occurrence:** individualCount: 1; sex: female; **Location:** locationID: C3; continent: Europe; country: Spain; countryCode: ES; stateProvince: Castilla-La Mancha; county: Ciudad Real; locality: La Quesera; verbatimElevation: 767.55; decimalLatitude: 39.36177; decimalLongitude: -4.41733; geodeticDatum: WGS84; **Event:** eventID: C; samplingProtocol: Pitfall
- **Type status:** Other material. **Occurrence:** individualCount: 1; sex: male; **Location:** locationID: C3; continent: Europe; country: Spain; countryCode: ES; stateProvince: Castilla-La Mancha; county: Ciudad Real; locality: La Quesera; verbatimElevation: 767.55; decimalLatitude: 39.36177; decimalLongitude: -4.41733; geodeticDatum: WGS84; **Event:** eventID: D; samplingProtocol: Pitfall
- **Type status:** Other material. **Occurrence:** individualCount: 1; sex: female; **Location:** locationID: C3; continent: Europe; country: Spain; countryCode: ES; stateProvince: Castilla-La Mancha; county: Ciudad Real; locality: La Quesera; verbatimElevation: 767.55; decimalLatitude: 39.36177; decimalLongitude: -4.41733; geodeticDatum: WGS84; **Event:** eventID: G; samplingProtocol: Pitfall
- **Type status:** Other material. **Occurrence:** individualCount: 3; sex: male; **Location:** locationID: C3; continent: Europe; country: Spain; countryCode: ES; stateProvince: Castilla-La Mancha; county: Ciudad Real; locality: La Quesera; verbatimElevation: 767.55; decimalLatitude: 39.36177; decimalLongitude: -4.41733; geodeticDatum: WGS84; **Event:** eventID: H; samplingProtocol: Pitfall
- **Type status:** Other material. **Occurrence:** individualCount: 1; sex: female; **Location:** locationID: C3; continent: Europe; country: Spain; countryCode: ES; stateProvince: Castilla-La Mancha; county: Ciudad Real; locality: La Quesera; verbatimElevation: 767.55; decimalLatitude: 39.36177; decimalLongitude: -4.41733; geodeticDatum: WGS84; **Event:** eventID: J; samplingProtocol: Pitfall
- **Type status:** Other material. **Occurrence:** individualCount: 1; sex: male; **Location:** locationID: C4; continent: Europe; country: Spain; countryCode: ES; stateProvince: Castilla-La Mancha; county: Ciudad Real; locality: La Quesera; verbatimElevation: 772.3; decimalLatitude: 39.36337; decimalLongitude: -4.41704; geodeticDatum: WGS84; **Event:** eventID: K; samplingProtocol: Pitfall
- **Type status:** Other material. **Occurrence:** individualCount: 4; sex: male; **Location:** locationID: S1; continent: Europe; country: Spain; countryCode: ES; stateProvince: Andalucía; county: Granada; locality: Soportujar; verbatimElevation: 1786.57; decimalLatitude: 36.96151; decimalLongitude: -3.41881; geodeticDatum: WGS84; **Event:** eventID: D; samplingProtocol: Pitfall
- **Type status:** Other material. **Occurrence:** individualCount: 1; sex: male; **Location:** locationID: S1; continent: Europe; country: Spain; countryCode: ES; stateProvince: Andalucía; county: Granada; locality: Soportujar; verbatimElevation: 1786.57; decimalLatitude: 36.96151; decimalLongitude: -3.41881; geodeticDatum: WGS84; **Event:** eventID: K; samplingProtocol: Pitfall

##### Distribution

Southern Europe

#### Callilepis nocturna

(Linnaeus, 1758)

##### Materials

- **Type status:** Other material. **Occurrence:** individualCount: 1; sex: male; **Location:** locationID: A1; continent: Europe; country: Spain; countryCode: ES; stateProvince: Catalonia; county: Lleida; locality: Sola de Boi; verbatimElevation: 1759.8; decimalLatitude: 42.54958; decimalLongitude: 0.87254; geodeticDatum: WGS84; **Event:** eventID: C; samplingProtocol: Pitfall
- **Type status:** Other material. **Occurrence:** individualCount: 2; sex: male; **Location:** locationID: A1; continent: Europe; country: Spain; countryCode: ES; stateProvince: Catalonia; county: Lleida; locality: Sola de Boi; verbatimElevation: 1759.8; decimalLatitude: 42.54958; decimalLongitude: 0.87254; geodeticDatum: WGS84; **Event:** eventID: E; samplingProtocol: Pitfall
- **Type status:** Other material. **Occurrence:** individualCount: 1; sex: male; **Location:** locationID: A1; continent: Europe; country: Spain; countryCode: ES; stateProvince: Catalonia; county: Lleida; locality: Sola de Boi; verbatimElevation: 1759.8; decimalLatitude: 42.54958; decimalLongitude: 0.87254; geodeticDatum: WGS84; **Event:** eventID: G; samplingProtocol: Pitfall
- **Type status:** Other material. **Occurrence:** individualCount: 1; sex: female; **Location:** locationID: A1; continent: Europe; country: Spain; countryCode: ES; stateProvince: Catalonia; county: Lleida; locality: Sola de Boi; verbatimElevation: 1759.8; decimalLatitude: 42.54958; decimalLongitude: 0.87254; geodeticDatum: WGS84; **Event:** eventID: I; samplingProtocol: Pitfall
- **Type status:** Other material. **Occurrence:** individualCount: 2; sex: male; **Location:** locationID: A2; continent: Europe; country: Spain; countryCode: ES; stateProvince: Catalonia; county: Lleida; locality: Sola de Boi; verbatimElevation: 1738.7; decimalLatitude: 42.54913; decimalLongitude: 0.87137; geodeticDatum: WGS84; **Event:** eventID: A; samplingProtocol: Pitfall
- **Type status:** Other material. **Occurrence:** individualCount: 1; sex: male; **Location:** locationID: A2; continent: Europe; country: Spain; countryCode: ES; stateProvince: Catalonia; county: Lleida; locality: Sola de Boi; verbatimElevation: 1738.7; decimalLatitude: 42.54913; decimalLongitude: 0.87137; geodeticDatum: WGS84; **Event:** eventID: B; samplingProtocol: Pitfall
- **Type status:** Other material. **Occurrence:** individualCount: 1; sex: male; **Location:** locationID: A2; continent: Europe; country: Spain; countryCode: ES; stateProvince: Catalonia; county: Lleida; locality: Sola de Boi; verbatimElevation: 1738.7; decimalLatitude: 42.54913; decimalLongitude: 0.87137; geodeticDatum: WGS84; **Event:** eventID: E; samplingProtocol: Pitfall
- **Type status:** Other material. **Occurrence:** individualCount: 1; sex: female; **Location:** locationID: A2; continent: Europe; country: Spain; countryCode: ES; stateProvince: Catalonia; county: Lleida; locality: Sola de Boi; verbatimElevation: 1738.7; decimalLatitude: 42.54913; decimalLongitude: 0.87137; geodeticDatum: WGS84; **Event:** eventID: L; samplingProtocol: Pitfall
- **Type status:** Other material. **Occurrence:** individualCount: 1; sex: male; **Location:** locationID: P2; continent: Europe; country: Spain; countryCode: ES; stateProvince: Castilla y León; county: León; locality: Joyoguelas; verbatimElevation: 763.98; decimalLatitude: 43.17771; decimalLongitude: -4.90579; geodeticDatum: WGS84; **Event:** eventID: E; samplingProtocol: Pitfall
- **Type status:** Other material. **Occurrence:** individualCount: 1; sex: male; **Location:** locationID: P4; continent: Europe; country: Spain; countryCode: ES; stateProvince: Castilla y León; county: León; locality: El Canto; verbatimElevation: 943.48; decimalLatitude: 43.17227; decimalLongitude: -4.90857; geodeticDatum: WGS84; **Event:** eventID: C; samplingProtocol: Pitfall

##### Distribution

Palearctic

#### Civizelotes civicus

(Simon, 1878)

##### Materials

- **Type status:** Other material. **Occurrence:** individualCount: 1; sex: male; **Location:** locationID: C1; continent: Europe; country: Spain; countryCode: ES; stateProvince: Castilla-La Mancha; county: Ciudad Real; locality: Valle Brezoso; verbatimElevation: 756.56; decimalLatitude: 39.35663; decimalLongitude: -4.35912; geodeticDatum: WGS84; **Event:** eventID: D; samplingProtocol: Pitfall
- **Type status:** Other material. **Occurrence:** individualCount: 1; sex: male; **Location:** locationID: C1; continent: Europe; country: Spain; countryCode: ES; stateProvince: Castilla-La Mancha; county: Ciudad Real; locality: Valle Brezoso; verbatimElevation: 756.56; decimalLatitude: 39.35663; decimalLongitude: -4.35912; geodeticDatum: WGS84; **Event:** eventID: H; samplingProtocol: Pitfall
- **Type status:** Other material. **Occurrence:** individualCount: 1; sex: male; **Location:** locationID: C2; continent: Europe; country: Spain; countryCode: ES; stateProvince: Castilla-La Mancha; county: Ciudad Real; locality: Valle Brezoso; verbatimElevation: 739.31; decimalLatitude: 39.35159; decimalLongitude: -4.3589; geodeticDatum: WGS84; **Event:** eventID: A; samplingProtocol: Pitfall
- **Type status:** Other material. **Occurrence:** individualCount: 2; sex: male; **Location:** locationID: C2; continent: Europe; country: Spain; countryCode: ES; stateProvince: Castilla-La Mancha; county: Ciudad Real; locality: Valle Brezoso; verbatimElevation: 739.31; decimalLatitude: 39.35159; decimalLongitude: -4.3589; geodeticDatum: WGS84; **Event:** eventID: C; samplingProtocol: Pitfall
- **Type status:** Other material. **Occurrence:** individualCount: 1; sex: male; **Location:** locationID: C2; continent: Europe; country: Spain; countryCode: ES; stateProvince: Castilla-La Mancha; county: Ciudad Real; locality: Valle Brezoso; verbatimElevation: 739.31; decimalLatitude: 39.35159; decimalLongitude: -4.3589; geodeticDatum: WGS84; **Event:** eventID: E; samplingProtocol: Pitfall
- **Type status:** Other material. **Occurrence:** individualCount: 1; sex: male; **Location:** locationID: C2; continent: Europe; country: Spain; countryCode: ES; stateProvince: Castilla-La Mancha; county: Ciudad Real; locality: Valle Brezoso; verbatimElevation: 739.31; decimalLatitude: 39.35159; decimalLongitude: -4.3589; geodeticDatum: WGS84; **Event:** eventID: F; samplingProtocol: Pitfall
- **Type status:** Other material. **Occurrence:** individualCount: 1; sex: male; **Location:** locationID: C2; continent: Europe; country: Spain; countryCode: ES; stateProvince: Castilla-La Mancha; county: Ciudad Real; locality: Valle Brezoso; verbatimElevation: 739.31; decimalLatitude: 39.35159; decimalLongitude: -4.3589; geodeticDatum: WGS84; **Event:** eventID: I; samplingProtocol: Pitfall
- **Type status:** Other material. **Occurrence:** individualCount: 1; sex: female; **Location:** locationID: C2; continent: Europe; country: Spain; countryCode: ES; stateProvince: Castilla-La Mancha; county: Ciudad Real; locality: Valle Brezoso; verbatimElevation: 739.31; decimalLatitude: 39.35159; decimalLongitude: -4.3589; geodeticDatum: WGS84; **Event:** eventID: I; samplingProtocol: Pitfall
- **Type status:** Other material. **Occurrence:** individualCount: 1; sex: male; **Location:** locationID: C2; continent: Europe; country: Spain; countryCode: ES; stateProvince: Castilla-La Mancha; county: Ciudad Real; locality: Valle Brezoso; verbatimElevation: 739.31; decimalLatitude: 39.35159; decimalLongitude: -4.3589; geodeticDatum: WGS84; **Event:** eventID: L; samplingProtocol: Pitfall
- **Type status:** Other material. **Occurrence:** individualCount: 1; sex: male; **Location:** locationID: C3; continent: Europe; country: Spain; countryCode: ES; stateProvince: Castilla-La Mancha; county: Ciudad Real; locality: La Quesera; verbatimElevation: 767.55; decimalLatitude: 39.36177; decimalLongitude: -4.41733; geodeticDatum: WGS84; **Event:** eventID: A; samplingProtocol: Pitfall
- **Type status:** Other material. **Occurrence:** individualCount: 1; sex: female; **Location:** locationID: C3; continent: Europe; country: Spain; countryCode: ES; stateProvince: Castilla-La Mancha; county: Ciudad Real; locality: La Quesera; verbatimElevation: 767.55; decimalLatitude: 39.36177; decimalLongitude: -4.41733; geodeticDatum: WGS84; **Event:** eventID: B; samplingProtocol: Pitfall
- **Type status:** Other material. **Occurrence:** individualCount: 4; sex: female; **Location:** locationID: C3; continent: Europe; country: Spain; countryCode: ES; stateProvince: Castilla-La Mancha; county: Ciudad Real; locality: La Quesera; verbatimElevation: 767.55; decimalLatitude: 39.36177; decimalLongitude: -4.41733; geodeticDatum: WGS84; **Event:** eventID: F; samplingProtocol: Pitfall
- **Type status:** Other material. **Occurrence:** individualCount: 1; sex: male; **Location:** locationID: C4; continent: Europe; country: Spain; countryCode: ES; stateProvince: Castilla-La Mancha; county: Ciudad Real; locality: La Quesera; verbatimElevation: 772.3; decimalLatitude: 39.36337; decimalLongitude: -4.41704; geodeticDatum: WGS84; **Event:** eventID: C; samplingProtocol: Pitfall
- **Type status:** Other material. **Occurrence:** individualCount: 1; sex: female; **Location:** locationID: C4; continent: Europe; country: Spain; countryCode: ES; stateProvince: Castilla-La Mancha; county: Ciudad Real; locality: La Quesera; verbatimElevation: 772.3; decimalLatitude: 39.36337; decimalLongitude: -4.41704; geodeticDatum: WGS84; **Event:** eventID: D; samplingProtocol: Pitfall
- **Type status:** Other material. **Occurrence:** individualCount: 1; sex: male; **Location:** locationID: C4; continent: Europe; country: Spain; countryCode: ES; stateProvince: Castilla-La Mancha; county: Ciudad Real; locality: La Quesera; verbatimElevation: 772.3; decimalLatitude: 39.36337; decimalLongitude: -4.41704; geodeticDatum: WGS84; **Event:** eventID: E; samplingProtocol: Pitfall
- **Type status:** Other material. **Occurrence:** individualCount: 1; sex: male; **Location:** locationID: C4; continent: Europe; country: Spain; countryCode: ES; stateProvince: Castilla-La Mancha; county: Ciudad Real; locality: La Quesera; verbatimElevation: 772.3; decimalLatitude: 39.36337; decimalLongitude: -4.41704; geodeticDatum: WGS84; **Event:** eventID: H; samplingProtocol: Pitfall
- **Type status:** Other material. **Occurrence:** individualCount: 1; sex: male; **Location:** locationID: C4; continent: Europe; country: Spain; countryCode: ES; stateProvince: Castilla-La Mancha; county: Ciudad Real; locality: La Quesera; verbatimElevation: 772.3; decimalLatitude: 39.36337; decimalLongitude: -4.41704; geodeticDatum: WGS84; **Event:** eventID: K; samplingProtocol: Pitfall
- **Type status:** Other material. **Occurrence:** individualCount: 1; sex: male; **Location:** locationID: S2; continent: Europe; country: Spain; countryCode: ES; stateProvince: Andalucía; county: Granada; locality: Camarate; verbatimElevation: 1713.96; decimalLatitude: 37.18377; decimalLongitude: -3.26282; geodeticDatum: WGS84; **Event:** eventID: A; samplingProtocol: Pitfall
- **Type status:** Other material. **Occurrence:** individualCount: 1; sex: male; **Location:** locationID: S2; continent: Europe; country: Spain; countryCode: ES; stateProvince: Andalucía; county: Granada; locality: Camarate; verbatimElevation: 1713.96; decimalLatitude: 37.18377; decimalLongitude: -3.26282; geodeticDatum: WGS84; **Event:** eventID: C; samplingProtocol: Pitfall
- **Type status:** Other material. **Occurrence:** individualCount: 1; sex: male; **Location:** locationID: S2; continent: Europe; country: Spain; countryCode: ES; stateProvince: Andalucía; county: Granada; locality: Camarate; verbatimElevation: 1713.96; decimalLatitude: 37.18377; decimalLongitude: -3.26282; geodeticDatum: WGS84; **Event:** eventID: D; samplingProtocol: Pitfall
- **Type status:** Other material. **Occurrence:** individualCount: 1; sex: male; **Location:** locationID: S2; continent: Europe; country: Spain; countryCode: ES; stateProvince: Andalucía; county: Granada; locality: Camarate; verbatimElevation: 1713.96; decimalLatitude: 37.18377; decimalLongitude: -3.26282; geodeticDatum: WGS84; **Event:** eventID: E; samplingProtocol: Pitfall
- **Type status:** Other material. **Occurrence:** individualCount: 1; sex: female; **Location:** locationID: S2; continent: Europe; country: Spain; countryCode: ES; stateProvince: Andalucía; county: Granada; locality: Camarate; verbatimElevation: 1713.96; decimalLatitude: 37.18377; decimalLongitude: -3.26282; geodeticDatum: WGS84; **Event:** eventID: E; samplingProtocol: Pitfall
- **Type status:** Other material. **Occurrence:** individualCount: 1; sex: male; **Location:** locationID: S2; continent: Europe; country: Spain; countryCode: ES; stateProvince: Andalucía; county: Granada; locality: Camarate; verbatimElevation: 1713.96; decimalLatitude: 37.18377; decimalLongitude: -3.26282; geodeticDatum: WGS84; **Event:** eventID: H; samplingProtocol: Pitfall
- **Type status:** Other material. **Occurrence:** individualCount: 2; sex: male; **Location:** locationID: S2; continent: Europe; country: Spain; countryCode: ES; stateProvince: Andalucía; county: Granada; locality: Camarate; verbatimElevation: 1713.96; decimalLatitude: 37.18377; decimalLongitude: -3.26282; geodeticDatum: WGS84; **Event:** eventID: I; samplingProtocol: Pitfall

##### Distribution

Europe, Madeira, Morocco

#### Civizelotes dentatidens

(Simon, 1914)

##### Materials

- **Type status:** Other material. **Occurrence:** individualCount: 1; sex: male; **Location:** locationID: P3; continent: Europe; country: Spain; countryCode: ES; stateProvince: Castilla y León; county: León; locality: Las Arroyas; verbatimElevation: 1097.1; decimalLatitude: 43.14351; decimalLongitude: -4.94878; geodeticDatum: WGS84; **Event:** eventID: D; samplingProtocol: Pitfall

##### Distribution

Iberian Peninsula, France, Sardinia

#### Civizelotes medianoides

Senglet, 2012

##### Materials

- **Type status:** Other material. **Occurrence:** individualCount: 1; sex: male; **Location:** locationID: C1; continent: Europe; country: Spain; countryCode: ES; stateProvince: Castilla-La Mancha; county: Ciudad Real; locality: Valle Brezoso; verbatimElevation: 756.56; decimalLatitude: 39.35663; decimalLongitude: -4.35912; geodeticDatum: WGS84; **Event:** eventID: H; samplingProtocol: Pitfall
- **Type status:** Other material. **Occurrence:** individualCount: 1; sex: male; **Location:** locationID: C1; continent: Europe; country: Spain; countryCode: ES; stateProvince: Castilla-La Mancha; county: Ciudad Real; locality: Valle Brezoso; verbatimElevation: 756.56; decimalLatitude: 39.35663; decimalLongitude: -4.35912; geodeticDatum: WGS84; **Event:** eventID: J; samplingProtocol: Pitfall
- **Type status:** Other material. **Occurrence:** individualCount: 1; sex: male; **Location:** locationID: C3; continent: Europe; country: Spain; countryCode: ES; stateProvince: Castilla-La Mancha; county: Ciudad Real; locality: La Quesera; verbatimElevation: 767.55; decimalLatitude: 39.36177; decimalLongitude: -4.41733; geodeticDatum: WGS84; **Event:** eventID: E; samplingProtocol: Pitfall
- **Type status:** Other material. **Occurrence:** individualCount: 1; sex: male; **Location:** locationID: C3; continent: Europe; country: Spain; countryCode: ES; stateProvince: Castilla-La Mancha; county: Ciudad Real; locality: La Quesera; verbatimElevation: 767.55; decimalLatitude: 39.36177; decimalLongitude: -4.41733; geodeticDatum: WGS84; **Event:** eventID: L; samplingProtocol: Pitfall

##### Distribution

Spain

#### Civizelotes medianus

(Denis, 1935)

##### Materials

- **Type status:** Other material. **Occurrence:** individualCount: 1; sex: male; **Location:** locationID: M1; continent: Europe; country: Spain; countryCode: ES; stateProvince: Extremadura; county: Cáceres; locality: Peña Falcón; verbatimElevation: 320.6; decimalLatitude: 39.83296; decimalLongitude: -6.0641; geodeticDatum: WGS84; **Event:** eventID: A; samplingProtocol: Pitfall
- **Type status:** Other material. **Occurrence:** individualCount: 2; sex: male; **Location:** locationID: M1; continent: Europe; country: Spain; countryCode: ES; stateProvince: Extremadura; county: Cáceres; locality: Peña Falcón; verbatimElevation: 320.6; decimalLatitude: 39.83296; decimalLongitude: -6.0641; geodeticDatum: WGS84; **Event:** eventID: B; samplingProtocol: Pitfall
- **Type status:** Other material. **Occurrence:** individualCount: 2; sex: male; **Location:** locationID: M1; continent: Europe; country: Spain; countryCode: ES; stateProvince: Extremadura; county: Cáceres; locality: Peña Falcón; verbatimElevation: 320.6; decimalLatitude: 39.83296; decimalLongitude: -6.0641; geodeticDatum: WGS84; **Event:** eventID: G; samplingProtocol: Pitfall
- **Type status:** Other material. **Occurrence:** individualCount: 1; sex: male; **Location:** locationID: M1; continent: Europe; country: Spain; countryCode: ES; stateProvince: Extremadura; county: Cáceres; locality: Peña Falcón; verbatimElevation: 320.6; decimalLatitude: 39.83296; decimalLongitude: -6.0641; geodeticDatum: WGS84; **Event:** eventID: I; samplingProtocol: Pitfall
- **Type status:** Other material. **Occurrence:** individualCount: 1; sex: male; **Location:** locationID: M2; continent: Europe; country: Spain; countryCode: ES; stateProvince: Extremadura; county: Cáceres; locality: Fuente del Frances; verbatimElevation: 320.72; decimalLatitude: 39.828; decimalLongitude: -6.03249; geodeticDatum: WGS84; **Event:** eventID: B; samplingProtocol: Pitfall
- **Type status:** Other material. **Occurrence:** individualCount: 2; sex: male; **Location:** locationID: M2; continent: Europe; country: Spain; countryCode: ES; stateProvince: Extremadura; county: Cáceres; locality: Fuente del Frances; verbatimElevation: 320.72; decimalLatitude: 39.828; decimalLongitude: -6.03249; geodeticDatum: WGS84; **Event:** eventID: C; samplingProtocol: Pitfall
- **Type status:** Other material. **Occurrence:** individualCount: 1; sex: female; **Location:** locationID: M2; continent: Europe; country: Spain; countryCode: ES; stateProvince: Extremadura; county: Cáceres; locality: Fuente del Frances; verbatimElevation: 320.72; decimalLatitude: 39.828; decimalLongitude: -6.03249; geodeticDatum: WGS84; **Event:** eventID: C; samplingProtocol: Pitfall
- **Type status:** Other material. **Occurrence:** individualCount: 2; sex: male; **Location:** locationID: M2; continent: Europe; country: Spain; countryCode: ES; stateProvince: Extremadura; county: Cáceres; locality: Fuente del Frances; verbatimElevation: 320.72; decimalLatitude: 39.828; decimalLongitude: -6.03249; geodeticDatum: WGS84; **Event:** eventID: F; samplingProtocol: Pitfall
- **Type status:** Other material. **Occurrence:** individualCount: 1; sex: male; **Location:** locationID: M2; continent: Europe; country: Spain; countryCode: ES; stateProvince: Extremadura; county: Cáceres; locality: Fuente del Frances; verbatimElevation: 320.72; decimalLatitude: 39.828; decimalLongitude: -6.03249; geodeticDatum: WGS84; **Event:** eventID: G; samplingProtocol: Pitfall
- **Type status:** Other material. **Occurrence:** individualCount: 1; sex: female; **Location:** locationID: M2; continent: Europe; country: Spain; countryCode: ES; stateProvince: Extremadura; county: Cáceres; locality: Fuente del Frances; verbatimElevation: 320.72; decimalLatitude: 39.828; decimalLongitude: -6.03249; geodeticDatum: WGS84; **Event:** eventID: J; samplingProtocol: Pitfall
- **Type status:** Other material. **Occurrence:** individualCount: 2; sex: male; **Location:** locationID: S1; continent: Europe; country: Spain; countryCode: ES; stateProvince: Andalucía; county: Granada; locality: Soportujar; verbatimElevation: 1786.57; decimalLatitude: 36.96151; decimalLongitude: -3.41881; geodeticDatum: WGS84; **Event:** eventID: G; samplingProtocol: Pitfall
- **Type status:** Other material. **Occurrence:** individualCount: 6; sex: male; **Location:** locationID: S1; continent: Europe; country: Spain; countryCode: ES; stateProvince: Andalucía; county: Granada; locality: Soportujar; verbatimElevation: 1786.57; decimalLatitude: 36.96151; decimalLongitude: -3.41881; geodeticDatum: WGS84; **Event:** eventID: I; samplingProtocol: Pitfall
- **Type status:** Other material. **Occurrence:** individualCount: 2; sex: male; **Location:** locationID: S1; continent: Europe; country: Spain; countryCode: ES; stateProvince: Andalucía; county: Granada; locality: Soportujar; verbatimElevation: 1786.57; decimalLatitude: 36.96151; decimalLongitude: -3.41881; geodeticDatum: WGS84; **Event:** eventID: J; samplingProtocol: Pitfall
- **Type status:** Other material. **Occurrence:** individualCount: 1; sex: female; **Location:** locationID: S1; continent: Europe; country: Spain; countryCode: ES; stateProvince: Andalucía; county: Granada; locality: Soportujar; verbatimElevation: 1786.57; decimalLatitude: 36.96151; decimalLongitude: -3.41881; geodeticDatum: WGS84; **Event:** eventID: J; samplingProtocol: Pitfall
- **Type status:** Other material. **Occurrence:** individualCount: 1; sex: male; **Location:** locationID: S1; continent: Europe; country: Spain; countryCode: ES; stateProvince: Andalucía; county: Granada; locality: Soportujar; verbatimElevation: 1786.57; decimalLatitude: 36.96151; decimalLongitude: -3.41881; geodeticDatum: WGS84; **Event:** eventID: K; samplingProtocol: Pitfall
- **Type status:** Other material. **Occurrence:** individualCount: 4; sex: male; **Location:** locationID: S1; continent: Europe; country: Spain; countryCode: ES; stateProvince: Andalucía; county: Granada; locality: Soportujar; verbatimElevation: 1786.57; decimalLatitude: 36.96151; decimalLongitude: -3.41881; geodeticDatum: WGS84; **Event:** eventID: L; samplingProtocol: Pitfall
- **Type status:** Other material. **Occurrence:** individualCount: 1; sex: male; **Location:** locationID: S2; continent: Europe; country: Spain; countryCode: ES; stateProvince: Andalucía; county: Granada; locality: Camarate; verbatimElevation: 1713.96; decimalLatitude: 37.18377; decimalLongitude: -3.26282; geodeticDatum: WGS84; **Event:** eventID: A; samplingProtocol: Pitfall
- **Type status:** Other material. **Occurrence:** individualCount: 6; sex: male; **Location:** locationID: S2; continent: Europe; country: Spain; countryCode: ES; stateProvince: Andalucía; county: Granada; locality: Camarate; verbatimElevation: 1713.96; decimalLatitude: 37.18377; decimalLongitude: -3.26282; geodeticDatum: WGS84; **Event:** eventID: I; samplingProtocol: Pitfall
- **Type status:** Other material. **Occurrence:** individualCount: 1; sex: female; **Location:** locationID: S2; continent: Europe; country: Spain; countryCode: ES; stateProvince: Andalucía; county: Granada; locality: Camarate; verbatimElevation: 1713.96; decimalLatitude: 37.18377; decimalLongitude: -3.26282; geodeticDatum: WGS84; **Event:** eventID: J; samplingProtocol: Pitfall
- **Type status:** Other material. **Occurrence:** individualCount: 1; sex: female; **Location:** locationID: S2; continent: Europe; country: Spain; countryCode: ES; stateProvince: Andalucía; county: Granada; locality: Camarate; verbatimElevation: 1713.96; decimalLatitude: 37.18377; decimalLongitude: -3.26282; geodeticDatum: WGS84; **Event:** eventID: K; samplingProtocol: Pitfall
- **Type status:** Other material. **Occurrence:** individualCount: 1; sex: male; **Location:** locationID: S2; continent: Europe; country: Spain; countryCode: ES; stateProvince: Andalucía; county: Granada; locality: Camarate; verbatimElevation: 1713.96; decimalLatitude: 37.18377; decimalLongitude: -3.26282; geodeticDatum: WGS84; **Event:** eventID: L; samplingProtocol: Pitfall

##### Distribution

Spain, France, Andorra

#### Drassodes lapidosus

(Walckenaer, 1802)

##### Materials

- **Type status:** Other material. **Occurrence:** individualCount: 1; sex: male; **Location:** locationID: A1; continent: Europe; country: Spain; countryCode: ES; stateProvince: Catalonia; county: Lleida; locality: Sola de Boi; verbatimElevation: 1759.8; decimalLatitude: 42.54958; decimalLongitude: 0.87254; geodeticDatum: WGS84; **Event:** eventID: L; samplingProtocol: Pitfall
- **Type status:** Other material. **Occurrence:** individualCount: 1; sex: male; **Location:** locationID: A1; continent: Europe; country: Spain; countryCode: ES; stateProvince: Catalonia; county: Lleida; locality: Sola de Boi; verbatimElevation: 1759.8; decimalLatitude: 42.54958; decimalLongitude: 0.87254; geodeticDatum: WGS84; **Event:** eventID: 1; samplingProtocol: Sweeping; eventTime: Night
- **Type status:** Other material. **Occurrence:** individualCount: 1; sex: male; **Location:** locationID: A2; continent: Europe; country: Spain; countryCode: ES; stateProvince: Catalonia; county: Lleida; locality: Sola de Boi; verbatimElevation: 1738.7; decimalLatitude: 42.54913; decimalLongitude: 0.87137; geodeticDatum: WGS84; **Event:** eventID: C; samplingProtocol: Pitfall
- **Type status:** Other material. **Occurrence:** individualCount: 1; sex: male; **Location:** locationID: A2; continent: Europe; country: Spain; countryCode: ES; stateProvince: Catalonia; county: Lleida; locality: Sola de Boi; verbatimElevation: 1738.7; decimalLatitude: 42.54913; decimalLongitude: 0.87137; geodeticDatum: WGS84; **Event:** eventID: G; samplingProtocol: Pitfall
- **Type status:** Other material. **Occurrence:** individualCount: 1; sex: male; **Location:** locationID: C1; continent: Europe; country: Spain; countryCode: ES; stateProvince: Castilla-La Mancha; county: Ciudad Real; locality: Valle Brezoso; verbatimElevation: 756.56; decimalLatitude: 39.35663; decimalLongitude: -4.35912; geodeticDatum: WGS84; **Event:** eventID: 4; samplingProtocol: Aerial; eventTime: Night
- **Type status:** Other material. **Occurrence:** individualCount: 1; sex: male; **Location:** locationID: C1; continent: Europe; country: Spain; countryCode: ES; stateProvince: Castilla-La Mancha; county: Ciudad Real; locality: Valle Brezoso; verbatimElevation: 756.56; decimalLatitude: 39.35663; decimalLongitude: -4.35912; geodeticDatum: WGS84; **Event:** eventID: C; samplingProtocol: Pitfall
- **Type status:** Other material. **Occurrence:** individualCount: 5; sex: male; **Location:** locationID: C1; continent: Europe; country: Spain; countryCode: ES; stateProvince: Castilla-La Mancha; county: Ciudad Real; locality: Valle Brezoso; verbatimElevation: 756.56; decimalLatitude: 39.35663; decimalLongitude: -4.35912; geodeticDatum: WGS84; **Event:** eventID: D; samplingProtocol: Pitfall
- **Type status:** Other material. **Occurrence:** individualCount: 1; sex: male; **Location:** locationID: C1; continent: Europe; country: Spain; countryCode: ES; stateProvince: Castilla-La Mancha; county: Ciudad Real; locality: Valle Brezoso; verbatimElevation: 756.56; decimalLatitude: 39.35663; decimalLongitude: -4.35912; geodeticDatum: WGS84; **Event:** eventID: E; samplingProtocol: Pitfall
- **Type status:** Other material. **Occurrence:** individualCount: 1; sex: male; **Location:** locationID: C1; continent: Europe; country: Spain; countryCode: ES; stateProvince: Castilla-La Mancha; county: Ciudad Real; locality: Valle Brezoso; verbatimElevation: 756.56; decimalLatitude: 39.35663; decimalLongitude: -4.35912; geodeticDatum: WGS84; **Event:** eventID: H; samplingProtocol: Pitfall
- **Type status:** Other material. **Occurrence:** individualCount: 1; sex: male; **Location:** locationID: C1; continent: Europe; country: Spain; countryCode: ES; stateProvince: Castilla-La Mancha; county: Ciudad Real; locality: Valle Brezoso; verbatimElevation: 756.56; decimalLatitude: 39.35663; decimalLongitude: -4.35912; geodeticDatum: WGS84; **Event:** eventID: I; samplingProtocol: Pitfall
- **Type status:** Other material. **Occurrence:** individualCount: 1; sex: male; **Location:** locationID: C1; continent: Europe; country: Spain; countryCode: ES; stateProvince: Castilla-La Mancha; county: Ciudad Real; locality: Valle Brezoso; verbatimElevation: 756.56; decimalLatitude: 39.35663; decimalLongitude: -4.35912; geodeticDatum: WGS84; **Event:** eventID: K; samplingProtocol: Pitfall
- **Type status:** Other material. **Occurrence:** individualCount: 1; sex: male; **Location:** locationID: C2; continent: Europe; country: Spain; countryCode: ES; stateProvince: Castilla-La Mancha; county: Ciudad Real; locality: Valle Brezoso; verbatimElevation: 739.31; decimalLatitude: 39.35159; decimalLongitude: -4.3589; geodeticDatum: WGS84; **Event:** eventID: 1; samplingProtocol: Aerial; eventTime: Night
- **Type status:** Other material. **Occurrence:** individualCount: 2; sex: male; **Location:** locationID: C2; continent: Europe; country: Spain; countryCode: ES; stateProvince: Castilla-La Mancha; county: Ciudad Real; locality: Valle Brezoso; verbatimElevation: 739.31; decimalLatitude: 39.35159; decimalLongitude: -4.3589; geodeticDatum: WGS84; **Event:** eventID: C; samplingProtocol: Pitfall
- **Type status:** Other material. **Occurrence:** individualCount: 1; sex: male; **Location:** locationID: C2; continent: Europe; country: Spain; countryCode: ES; stateProvince: Castilla-La Mancha; county: Ciudad Real; locality: Valle Brezoso; verbatimElevation: 739.31; decimalLatitude: 39.35159; decimalLongitude: -4.3589; geodeticDatum: WGS84; **Event:** eventID: D; samplingProtocol: Pitfall
- **Type status:** Other material. **Occurrence:** individualCount: 1; sex: male; **Location:** locationID: C2; continent: Europe; country: Spain; countryCode: ES; stateProvince: Castilla-La Mancha; county: Ciudad Real; locality: Valle Brezoso; verbatimElevation: 739.31; decimalLatitude: 39.35159; decimalLongitude: -4.3589; geodeticDatum: WGS84; **Event:** eventID: F; samplingProtocol: Pitfall
- **Type status:** Other material. **Occurrence:** individualCount: 1; sex: male; **Location:** locationID: C2; continent: Europe; country: Spain; countryCode: ES; stateProvince: Castilla-La Mancha; county: Ciudad Real; locality: Valle Brezoso; verbatimElevation: 739.31; decimalLatitude: 39.35159; decimalLongitude: -4.3589; geodeticDatum: WGS84; **Event:** eventID: G; samplingProtocol: Pitfall
- **Type status:** Other material. **Occurrence:** individualCount: 1; sex: male; **Location:** locationID: C2; continent: Europe; country: Spain; countryCode: ES; stateProvince: Castilla-La Mancha; county: Ciudad Real; locality: Valle Brezoso; verbatimElevation: 739.31; decimalLatitude: 39.35159; decimalLongitude: -4.3589; geodeticDatum: WGS84; **Event:** eventID: H; samplingProtocol: Pitfall
- **Type status:** Other material. **Occurrence:** individualCount: 2; sex: male; **Location:** locationID: C2; continent: Europe; country: Spain; countryCode: ES; stateProvince: Castilla-La Mancha; county: Ciudad Real; locality: Valle Brezoso; verbatimElevation: 739.31; decimalLatitude: 39.35159; decimalLongitude: -4.3589; geodeticDatum: WGS84; **Event:** eventID: I; samplingProtocol: Pitfall
- **Type status:** Other material. **Occurrence:** individualCount: 2; sex: male; **Location:** locationID: C2; continent: Europe; country: Spain; countryCode: ES; stateProvince: Castilla-La Mancha; county: Ciudad Real; locality: Valle Brezoso; verbatimElevation: 739.31; decimalLatitude: 39.35159; decimalLongitude: -4.3589; geodeticDatum: WGS84; **Event:** eventID: L; samplingProtocol: Pitfall
- **Type status:** Other material. **Occurrence:** individualCount: 1; sex: female; **Location:** locationID: C2; continent: Europe; country: Spain; countryCode: ES; stateProvince: Castilla-La Mancha; county: Ciudad Real; locality: Valle Brezoso; verbatimElevation: 739.31; decimalLatitude: 39.35159; decimalLongitude: -4.3589; geodeticDatum: WGS84; **Event:** eventID: L; samplingProtocol: Pitfall
- **Type status:** Other material. **Occurrence:** individualCount: 1; sex: female; **Location:** locationID: C3; continent: Europe; country: Spain; countryCode: ES; stateProvince: Castilla-La Mancha; county: Ciudad Real; locality: La Quesera; verbatimElevation: 767.55; decimalLatitude: 39.36177; decimalLongitude: -4.41733; geodeticDatum: WGS84; **Event:** eventID: E; samplingProtocol: Pitfall
- **Type status:** Other material. **Occurrence:** individualCount: 1; sex: male; **Location:** locationID: C4; continent: Europe; country: Spain; countryCode: ES; stateProvince: Castilla-La Mancha; county: Ciudad Real; locality: La Quesera; verbatimElevation: 772.3; decimalLatitude: 39.36337; decimalLongitude: -4.41704; geodeticDatum: WGS84; **Event:** eventID: J; samplingProtocol: Pitfall
- **Type status:** Other material. **Occurrence:** individualCount: 1; sex: male; **Location:** locationID: O2; continent: Europe; country: Spain; countryCode: ES; stateProvince: Aragón; county: Huesca; locality: Rebilla; verbatimElevation: 1158.13; decimalLatitude: 42.59427; decimalLongitude: 0.1529; geodeticDatum: WGS84; **Event:** eventID: A; samplingProtocol: Pitfall
- **Type status:** Other material. **Occurrence:** individualCount: 1; sex: female; **Location:** locationID: P2; continent: Europe; country: Spain; countryCode: ES; stateProvince: Castilla y León; county: León; locality: Joyoguelas; verbatimElevation: 763.98; decimalLatitude: 43.17771; decimalLongitude: -4.90579; geodeticDatum: WGS84; **Event:** eventID: C; samplingProtocol: Pitfall
- **Type status:** Other material. **Occurrence:** individualCount: 1; sex: female; **Location:** locationID: P2; continent: Europe; country: Spain; countryCode: ES; stateProvince: Castilla y León; county: León; locality: Joyoguelas; verbatimElevation: 763.98; decimalLatitude: 43.17771; decimalLongitude: -4.90579; geodeticDatum: WGS84; **Event:** eventID: G; samplingProtocol: Pitfall
- **Type status:** Other material. **Occurrence:** individualCount: 5; sex: female; **Location:** locationID: P2; continent: Europe; country: Spain; countryCode: ES; stateProvince: Castilla y León; county: León; locality: Joyoguelas; verbatimElevation: 763.98; decimalLatitude: 43.17771; decimalLongitude: -4.90579; geodeticDatum: WGS84; **Event:** eventID: H; samplingProtocol: Pitfall
- **Type status:** Other material. **Occurrence:** individualCount: 1; sex: female; **Location:** locationID: P4; continent: Europe; country: Spain; countryCode: ES; stateProvince: Castilla y León; county: León; locality: El Canto; verbatimElevation: 943.48; decimalLatitude: 43.17227; decimalLongitude: -4.90857; geodeticDatum: WGS84; **Event:** eventID: C; samplingProtocol: Pitfall
- **Type status:** Other material. **Occurrence:** individualCount: 1; sex: male; **Location:** locationID: S1; continent: Europe; country: Spain; countryCode: ES; stateProvince: Andalucía; county: Granada; locality: Soportujar; verbatimElevation: 1786.57; decimalLatitude: 36.96151; decimalLongitude: -3.41881; geodeticDatum: WGS84; **Event:** eventID: D; samplingProtocol: Pitfall
- **Type status:** Other material. **Occurrence:** individualCount: 1; sex: female; **Location:** locationID: S1; continent: Europe; country: Spain; countryCode: ES; stateProvince: Andalucía; county: Granada; locality: Soportujar; verbatimElevation: 1786.57; decimalLatitude: 36.96151; decimalLongitude: -3.41881; geodeticDatum: WGS84; **Event:** eventID: E; samplingProtocol: Pitfall
- **Type status:** Other material. **Occurrence:** individualCount: 1; sex: male; **Location:** locationID: S1; continent: Europe; country: Spain; countryCode: ES; stateProvince: Andalucía; county: Granada; locality: Soportujar; verbatimElevation: 1786.57; decimalLatitude: 36.96151; decimalLongitude: -3.41881; geodeticDatum: WGS84; **Event:** eventID: F; samplingProtocol: Pitfall
- **Type status:** Other material. **Occurrence:** individualCount: 1; sex: male; **Location:** locationID: S1; continent: Europe; country: Spain; countryCode: ES; stateProvince: Andalucía; county: Granada; locality: Soportujar; verbatimElevation: 1786.57; decimalLatitude: 36.96151; decimalLongitude: -3.41881; geodeticDatum: WGS84; **Event:** eventID: H; samplingProtocol: Pitfall
- **Type status:** Other material. **Occurrence:** individualCount: 1; sex: male; **Location:** locationID: S1; continent: Europe; country: Spain; countryCode: ES; stateProvince: Andalucía; county: Granada; locality: Soportujar; verbatimElevation: 1786.57; decimalLatitude: 36.96151; decimalLongitude: -3.41881; geodeticDatum: WGS84; **Event:** eventID: K; samplingProtocol: Pitfall
- **Type status:** Other material. **Occurrence:** individualCount: 1; sex: female; **Location:** locationID: S1; continent: Europe; country: Spain; countryCode: ES; stateProvince: Andalucía; county: Granada; locality: Soportujar; verbatimElevation: 1786.57; decimalLatitude: 36.96151; decimalLongitude: -3.41881; geodeticDatum: WGS84; **Event:** eventID: K; samplingProtocol: Pitfall
- **Type status:** Other material. **Occurrence:** individualCount: 1; sex: male; **Location:** locationID: S2; continent: Europe; country: Spain; countryCode: ES; stateProvince: Andalucía; county: Granada; locality: Camarate; verbatimElevation: 1713.96; decimalLatitude: 37.18377; decimalLongitude: -3.26282; geodeticDatum: WGS84; **Event:** eventID: A; samplingProtocol: Pitfall
- **Type status:** Other material. **Occurrence:** individualCount: 1; sex: female; **Location:** locationID: S2; continent: Europe; country: Spain; countryCode: ES; stateProvince: Andalucía; county: Granada; locality: Camarate; verbatimElevation: 1713.96; decimalLatitude: 37.18377; decimalLongitude: -3.26282; geodeticDatum: WGS84; **Event:** eventID: A; samplingProtocol: Pitfall
- **Type status:** Other material. **Occurrence:** individualCount: 2; sex: male; **Location:** locationID: S2; continent: Europe; country: Spain; countryCode: ES; stateProvince: Andalucía; county: Granada; locality: Camarate; verbatimElevation: 1713.96; decimalLatitude: 37.18377; decimalLongitude: -3.26282; geodeticDatum: WGS84; **Event:** eventID: C; samplingProtocol: Pitfall
- **Type status:** Other material. **Occurrence:** individualCount: 1; sex: male; **Location:** locationID: S2; continent: Europe; country: Spain; countryCode: ES; stateProvince: Andalucía; county: Granada; locality: Camarate; verbatimElevation: 1713.96; decimalLatitude: 37.18377; decimalLongitude: -3.26282; geodeticDatum: WGS84; **Event:** eventID: E; samplingProtocol: Pitfall
- **Type status:** Other material. **Occurrence:** individualCount: 1; sex: female; **Location:** locationID: S2; continent: Europe; country: Spain; countryCode: ES; stateProvince: Andalucía; county: Granada; locality: Camarate; verbatimElevation: 1713.96; decimalLatitude: 37.18377; decimalLongitude: -3.26282; geodeticDatum: WGS84; **Event:** eventID: E; samplingProtocol: Pitfall
- **Type status:** Other material. **Occurrence:** individualCount: 2; sex: female; **Location:** locationID: S2; continent: Europe; country: Spain; countryCode: ES; stateProvince: Andalucía; county: Granada; locality: Camarate; verbatimElevation: 1713.96; decimalLatitude: 37.18377; decimalLongitude: -3.26282; geodeticDatum: WGS84; **Event:** eventID: F; samplingProtocol: Pitfall

##### Distribution

Palearctic

#### Drassodes lutescens

(C. L. Koch, 1839)

##### Materials

- **Type status:** Other material. **Occurrence:** individualCount: 1; sex: male; **Location:** locationID: C3; continent: Europe; country: Spain; countryCode: ES; stateProvince: Castilla-La Mancha; county: Ciudad Real; locality: La Quesera; verbatimElevation: 767.55; decimalLatitude: 39.36177; decimalLongitude: -4.41733; geodeticDatum: WGS84; **Event:** eventID: I; samplingProtocol: Pitfall
- **Type status:** Other material. **Occurrence:** individualCount: 1; sex: female; **Location:** locationID: C4; continent: Europe; country: Spain; countryCode: ES; stateProvince: Castilla-La Mancha; county: Ciudad Real; locality: La Quesera; verbatimElevation: 772.3; decimalLatitude: 39.36337; decimalLongitude: -4.41704; geodeticDatum: WGS84; **Event:** eventID: 2; samplingProtocol: Beating; eventTime: Night
- **Type status:** Other material. **Occurrence:** individualCount: 1; sex: female; **Location:** locationID: M1; continent: Europe; country: Spain; countryCode: ES; stateProvince: Extremadura; county: Cáceres; locality: Peña Falcón; verbatimElevation: 320.6; decimalLatitude: 39.83296; decimalLongitude: -6.0641; geodeticDatum: WGS84; **Event:** eventID: E; samplingProtocol: Pitfall
- **Type status:** Other material. **Occurrence:** individualCount: 1; sex: male; **Location:** locationID: M1; continent: Europe; country: Spain; countryCode: ES; stateProvince: Extremadura; county: Cáceres; locality: Peña Falcón; verbatimElevation: 320.6; decimalLatitude: 39.83296; decimalLongitude: -6.0641; geodeticDatum: WGS84; **Event:** eventID: I; samplingProtocol: Pitfall
- **Type status:** Other material. **Occurrence:** individualCount: 1; sex: male; **Location:** locationID: M2; continent: Europe; country: Spain; countryCode: ES; stateProvince: Extremadura; county: Cáceres; locality: Fuente del Frances; verbatimElevation: 320.72; decimalLatitude: 39.828; decimalLongitude: -6.03249; geodeticDatum: WGS84; **Event:** eventID: E; samplingProtocol: Pitfall

##### Distribution

Mediterranean to Pakistan

#### Drassodes pubescens

(Thorell, 1856)

##### Materials

- **Type status:** Other material. **Occurrence:** individualCount: 1; sex: male; **Location:** locationID: O2; continent: Europe; country: Spain; countryCode: ES; stateProvince: Aragón; county: Huesca; locality: Rebilla; verbatimElevation: 1158.13; decimalLatitude: 42.59427; decimalLongitude: 0.1529; geodeticDatum: WGS84; **Event:** eventID: F; samplingProtocol: Pitfall
- **Type status:** Other material. **Occurrence:** individualCount: 1; sex: male; **Location:** locationID: P3; continent: Europe; country: Spain; countryCode: ES; stateProvince: Castilla y León; county: León; locality: Las Arroyas; verbatimElevation: 1097.1; decimalLatitude: 43.14351; decimalLongitude: -4.94878; geodeticDatum: WGS84; **Event:** eventID: A; samplingProtocol: Pitfall
- **Type status:** Other material. **Occurrence:** individualCount: 2; sex: male; **Location:** locationID: P3; continent: Europe; country: Spain; countryCode: ES; stateProvince: Castilla y León; county: León; locality: Las Arroyas; verbatimElevation: 1097.1; decimalLatitude: 43.14351; decimalLongitude: -4.94878; geodeticDatum: WGS84; **Event:** eventID: C; samplingProtocol: Pitfall
- **Type status:** Other material. **Occurrence:** individualCount: 1; sex: male; **Location:** locationID: P3; continent: Europe; country: Spain; countryCode: ES; stateProvince: Castilla y León; county: León; locality: Las Arroyas; verbatimElevation: 1097.1; decimalLatitude: 43.14351; decimalLongitude: -4.94878; geodeticDatum: WGS84; **Event:** eventID: F; samplingProtocol: Pitfall
- **Type status:** Other material. **Occurrence:** individualCount: 1; sex: male; **Location:** locationID: P3; continent: Europe; country: Spain; countryCode: ES; stateProvince: Castilla y León; county: León; locality: Las Arroyas; verbatimElevation: 1097.1; decimalLatitude: 43.14351; decimalLongitude: -4.94878; geodeticDatum: WGS84; **Event:** eventID: I; samplingProtocol: Pitfall
- **Type status:** Other material. **Occurrence:** individualCount: 1; sex: male; **Location:** locationID: P4; continent: Europe; country: Spain; countryCode: ES; stateProvince: Castilla y León; county: León; locality: El Canto; verbatimElevation: 943.48; decimalLatitude: 43.17227; decimalLongitude: -4.90857; geodeticDatum: WGS84; **Event:** eventID: B; samplingProtocol: Pitfall
- **Type status:** Other material. **Occurrence:** individualCount: 1; sex: male; **Location:** locationID: P4; continent: Europe; country: Spain; countryCode: ES; stateProvince: Castilla y León; county: León; locality: El Canto; verbatimElevation: 943.48; decimalLatitude: 43.17227; decimalLongitude: -4.90857; geodeticDatum: WGS84; **Event:** eventID: D; samplingProtocol: Pitfall
- **Type status:** Other material. **Occurrence:** individualCount: 1; sex: male; **Location:** locationID: P4; continent: Europe; country: Spain; countryCode: ES; stateProvince: Castilla y León; county: León; locality: El Canto; verbatimElevation: 943.48; decimalLatitude: 43.17227; decimalLongitude: -4.90857; geodeticDatum: WGS84; **Event:** eventID: H; samplingProtocol: Pitfall

##### Distribution

Palearctic

#### Drassyllus praeficus

(L. Koch, 1866)

##### Materials

- **Type status:** Other material. **Occurrence:** individualCount: 1; sex: male; **Location:** locationID: A1; continent: Europe; country: Spain; countryCode: ES; stateProvince: Catalonia; county: Lleida; locality: Sola de Boi; verbatimElevation: 1759.8; decimalLatitude: 42.54958; decimalLongitude: 0.87254; geodeticDatum: WGS84; **Event:** eventID: C; samplingProtocol: Pitfall
- **Type status:** Other material. **Occurrence:** individualCount: 7; sex: male; **Location:** locationID: A1; continent: Europe; country: Spain; countryCode: ES; stateProvince: Catalonia; county: Lleida; locality: Sola de Boi; verbatimElevation: 1759.8; decimalLatitude: 42.54958; decimalLongitude: 0.87254; geodeticDatum: WGS84; **Event:** eventID: E; samplingProtocol: Pitfall
- **Type status:** Other material. **Occurrence:** individualCount: 4; sex: female; **Location:** locationID: A1; continent: Europe; country: Spain; countryCode: ES; stateProvince: Catalonia; county: Lleida; locality: Sola de Boi; verbatimElevation: 1759.8; decimalLatitude: 42.54958; decimalLongitude: 0.87254; geodeticDatum: WGS84; **Event:** eventID: E; samplingProtocol: Pitfall
- **Type status:** Other material. **Occurrence:** individualCount: 2; sex: male; **Location:** locationID: A1; continent: Europe; country: Spain; countryCode: ES; stateProvince: Catalonia; county: Lleida; locality: Sola de Boi; verbatimElevation: 1759.8; decimalLatitude: 42.54958; decimalLongitude: 0.87254; geodeticDatum: WGS84; **Event:** eventID: F; samplingProtocol: Pitfall
- **Type status:** Other material. **Occurrence:** individualCount: 1; sex: male; **Location:** locationID: A1; continent: Europe; country: Spain; countryCode: ES; stateProvince: Catalonia; county: Lleida; locality: Sola de Boi; verbatimElevation: 1759.8; decimalLatitude: 42.54958; decimalLongitude: 0.87254; geodeticDatum: WGS84; **Event:** eventID: G; samplingProtocol: Pitfall
- **Type status:** Other material. **Occurrence:** individualCount: 1; sex: female; **Location:** locationID: A1; continent: Europe; country: Spain; countryCode: ES; stateProvince: Catalonia; county: Lleida; locality: Sola de Boi; verbatimElevation: 1759.8; decimalLatitude: 42.54958; decimalLongitude: 0.87254; geodeticDatum: WGS84; **Event:** eventID: I; samplingProtocol: Pitfall
- **Type status:** Other material. **Occurrence:** individualCount: 2; sex: male; **Location:** locationID: A1; continent: Europe; country: Spain; countryCode: ES; stateProvince: Catalonia; county: Lleida; locality: Sola de Boi; verbatimElevation: 1759.8; decimalLatitude: 42.54958; decimalLongitude: 0.87254; geodeticDatum: WGS84; **Event:** eventID: J; samplingProtocol: Pitfall
- **Type status:** Other material. **Occurrence:** individualCount: 2; sex: male; **Location:** locationID: A1; continent: Europe; country: Spain; countryCode: ES; stateProvince: Catalonia; county: Lleida; locality: Sola de Boi; verbatimElevation: 1759.8; decimalLatitude: 42.54958; decimalLongitude: 0.87254; geodeticDatum: WGS84; **Event:** eventID: L; samplingProtocol: Pitfall
- **Type status:** Other material. **Occurrence:** individualCount: 1; sex: female; **Location:** locationID: A2; continent: Europe; country: Spain; countryCode: ES; stateProvince: Catalonia; county: Lleida; locality: Sola de Boi; verbatimElevation: 1738.7; decimalLatitude: 42.54913; decimalLongitude: 0.87137; geodeticDatum: WGS84; **Event:** eventID: 1; samplingProtocol: Aerial; eventTime: Night
- **Type status:** Other material. **Occurrence:** individualCount: 4; sex: male; **Location:** locationID: A2; continent: Europe; country: Spain; countryCode: ES; stateProvince: Catalonia; county: Lleida; locality: Sola de Boi; verbatimElevation: 1738.7; decimalLatitude: 42.54913; decimalLongitude: 0.87137; geodeticDatum: WGS84; **Event:** eventID: A; samplingProtocol: Pitfall
- **Type status:** Other material. **Occurrence:** individualCount: 1; sex: male; **Location:** locationID: A2; continent: Europe; country: Spain; countryCode: ES; stateProvince: Catalonia; county: Lleida; locality: Sola de Boi; verbatimElevation: 1738.7; decimalLatitude: 42.54913; decimalLongitude: 0.87137; geodeticDatum: WGS84; **Event:** eventID: C; samplingProtocol: Pitfall
- **Type status:** Other material. **Occurrence:** individualCount: 1; sex: male; **Location:** locationID: A2; continent: Europe; country: Spain; countryCode: ES; stateProvince: Catalonia; county: Lleida; locality: Sola de Boi; verbatimElevation: 1738.7; decimalLatitude: 42.54913; decimalLongitude: 0.87137; geodeticDatum: WGS84; **Event:** eventID: E; samplingProtocol: Pitfall
- **Type status:** Other material. **Occurrence:** individualCount: 1; sex: male; **Location:** locationID: A2; continent: Europe; country: Spain; countryCode: ES; stateProvince: Catalonia; county: Lleida; locality: Sola de Boi; verbatimElevation: 1738.7; decimalLatitude: 42.54913; decimalLongitude: 0.87137; geodeticDatum: WGS84; **Event:** eventID: F; samplingProtocol: Pitfall
- **Type status:** Other material. **Occurrence:** individualCount: 1; sex: female; **Location:** locationID: A2; continent: Europe; country: Spain; countryCode: ES; stateProvince: Catalonia; county: Lleida; locality: Sola de Boi; verbatimElevation: 1738.7; decimalLatitude: 42.54913; decimalLongitude: 0.87137; geodeticDatum: WGS84; **Event:** eventID: H; samplingProtocol: Pitfall
- **Type status:** Other material. **Occurrence:** individualCount: 3; sex: male; **Location:** locationID: A2; continent: Europe; country: Spain; countryCode: ES; stateProvince: Catalonia; county: Lleida; locality: Sola de Boi; verbatimElevation: 1738.7; decimalLatitude: 42.54913; decimalLongitude: 0.87137; geodeticDatum: WGS84; **Event:** eventID: I; samplingProtocol: Pitfall
- **Type status:** Other material. **Occurrence:** individualCount: 1; sex: female; **Location:** locationID: A2; continent: Europe; country: Spain; countryCode: ES; stateProvince: Catalonia; county: Lleida; locality: Sola de Boi; verbatimElevation: 1738.7; decimalLatitude: 42.54913; decimalLongitude: 0.87137; geodeticDatum: WGS84; **Event:** eventID: I; samplingProtocol: Pitfall
- **Type status:** Other material. **Occurrence:** individualCount: 1; sex: male; **Location:** locationID: A2; continent: Europe; country: Spain; countryCode: ES; stateProvince: Catalonia; county: Lleida; locality: Sola de Boi; verbatimElevation: 1738.7; decimalLatitude: 42.54913; decimalLongitude: 0.87137; geodeticDatum: WGS84; **Event:** eventID: J; samplingProtocol: Pitfall
- **Type status:** Other material. **Occurrence:** individualCount: 2; sex: male; **Location:** locationID: A2; continent: Europe; country: Spain; countryCode: ES; stateProvince: Catalonia; county: Lleida; locality: Sola de Boi; verbatimElevation: 1738.7; decimalLatitude: 42.54913; decimalLongitude: 0.87137; geodeticDatum: WGS84; **Event:** eventID: K; samplingProtocol: Pitfall
- **Type status:** Other material. **Occurrence:** individualCount: 1; sex: female; **Location:** locationID: P2; continent: Europe; country: Spain; countryCode: ES; stateProvince: Castilla y León; county: León; locality: Joyoguelas; verbatimElevation: 763.98; decimalLatitude: 43.17771; decimalLongitude: -4.90579; geodeticDatum: WGS84; **Event:** eventID: A; samplingProtocol: Pitfall
- **Type status:** Other material. **Occurrence:** individualCount: 1; sex: male; **Location:** locationID: P2; continent: Europe; country: Spain; countryCode: ES; stateProvince: Castilla y León; county: León; locality: Joyoguelas; verbatimElevation: 763.98; decimalLatitude: 43.17771; decimalLongitude: -4.90579; geodeticDatum: WGS84; **Event:** eventID: B; samplingProtocol: Pitfall
- **Type status:** Other material. **Occurrence:** individualCount: 3; sex: male; **Location:** locationID: P2; continent: Europe; country: Spain; countryCode: ES; stateProvince: Castilla y León; county: León; locality: Joyoguelas; verbatimElevation: 763.98; decimalLatitude: 43.17771; decimalLongitude: -4.90579; geodeticDatum: WGS84; **Event:** eventID: C; samplingProtocol: Pitfall
- **Type status:** Other material. **Occurrence:** individualCount: 2; sex: male; **Location:** locationID: P2; continent: Europe; country: Spain; countryCode: ES; stateProvince: Castilla y León; county: León; locality: Joyoguelas; verbatimElevation: 763.98; decimalLatitude: 43.17771; decimalLongitude: -4.90579; geodeticDatum: WGS84; **Event:** eventID: D; samplingProtocol: Pitfall
- **Type status:** Other material. **Occurrence:** individualCount: 1; sex: female; **Location:** locationID: P2; continent: Europe; country: Spain; countryCode: ES; stateProvince: Castilla y León; county: León; locality: Joyoguelas; verbatimElevation: 763.98; decimalLatitude: 43.17771; decimalLongitude: -4.90579; geodeticDatum: WGS84; **Event:** eventID: D; samplingProtocol: Pitfall
- **Type status:** Other material. **Occurrence:** individualCount: 1; sex: male; **Location:** locationID: P2; continent: Europe; country: Spain; countryCode: ES; stateProvince: Castilla y León; county: León; locality: Joyoguelas; verbatimElevation: 763.98; decimalLatitude: 43.17771; decimalLongitude: -4.90579; geodeticDatum: WGS84; **Event:** eventID: E; samplingProtocol: Pitfall
- **Type status:** Other material. **Occurrence:** individualCount: 3; sex: female; **Location:** locationID: P2; continent: Europe; country: Spain; countryCode: ES; stateProvince: Castilla y León; county: León; locality: Joyoguelas; verbatimElevation: 763.98; decimalLatitude: 43.17771; decimalLongitude: -4.90579; geodeticDatum: WGS84; **Event:** eventID: H; samplingProtocol: Pitfall
- **Type status:** Other material. **Occurrence:** individualCount: 4; sex: male; **Location:** locationID: P2; continent: Europe; country: Spain; countryCode: ES; stateProvince: Castilla y León; county: León; locality: Joyoguelas; verbatimElevation: 763.98; decimalLatitude: 43.17771; decimalLongitude: -4.90579; geodeticDatum: WGS84; **Event:** eventID: H; samplingProtocol: Pitfall
- **Type status:** Other material. **Occurrence:** individualCount: 1; sex: male; **Location:** locationID: P2; continent: Europe; country: Spain; countryCode: ES; stateProvince: Castilla y León; county: León; locality: Joyoguelas; verbatimElevation: 763.98; decimalLatitude: 43.17771; decimalLongitude: -4.90579; geodeticDatum: WGS84; **Event:** eventID: I; samplingProtocol: Pitfall
- **Type status:** Other material. **Occurrence:** individualCount: 1; sex: male; **Location:** locationID: P3; continent: Europe; country: Spain; countryCode: ES; stateProvince: Castilla y León; county: León; locality: Las Arroyas; verbatimElevation: 1097.1; decimalLatitude: 43.14351; decimalLongitude: -4.94878; geodeticDatum: WGS84; **Event:** eventID: I; samplingProtocol: Pitfall
- **Type status:** Other material. **Occurrence:** individualCount: 1; sex: female; **Location:** locationID: P4; continent: Europe; country: Spain; countryCode: ES; stateProvince: Castilla y León; county: León; locality: El Canto; verbatimElevation: 943.48; decimalLatitude: 43.17227; decimalLongitude: -4.90857; geodeticDatum: WGS84; **Event:** eventID: A; samplingProtocol: Pitfall
- **Type status:** Other material. **Occurrence:** individualCount: 3; sex: male; **Location:** locationID: P4; continent: Europe; country: Spain; countryCode: ES; stateProvince: Castilla y León; county: León; locality: El Canto; verbatimElevation: 943.48; decimalLatitude: 43.17227; decimalLongitude: -4.90857; geodeticDatum: WGS84; **Event:** eventID: B; samplingProtocol: Pitfall
- **Type status:** Other material. **Occurrence:** individualCount: 7; sex: male; **Location:** locationID: P4; continent: Europe; country: Spain; countryCode: ES; stateProvince: Castilla y León; county: León; locality: El Canto; verbatimElevation: 943.48; decimalLatitude: 43.17227; decimalLongitude: -4.90857; geodeticDatum: WGS84; **Event:** eventID: C; samplingProtocol: Pitfall
- **Type status:** Other material. **Occurrence:** individualCount: 3; sex: female; **Location:** locationID: P4; continent: Europe; country: Spain; countryCode: ES; stateProvince: Castilla y León; county: León; locality: El Canto; verbatimElevation: 943.48; decimalLatitude: 43.17227; decimalLongitude: -4.90857; geodeticDatum: WGS84; **Event:** eventID: C; samplingProtocol: Pitfall
- **Type status:** Other material. **Occurrence:** individualCount: 4; sex: male; **Location:** locationID: P4; continent: Europe; country: Spain; countryCode: ES; stateProvince: Castilla y León; county: León; locality: El Canto; verbatimElevation: 943.48; decimalLatitude: 43.17227; decimalLongitude: -4.90857; geodeticDatum: WGS84; **Event:** eventID: D; samplingProtocol: Pitfall
- **Type status:** Other material. **Occurrence:** individualCount: 2; sex: male; **Location:** locationID: P4; continent: Europe; country: Spain; countryCode: ES; stateProvince: Castilla y León; county: León; locality: El Canto; verbatimElevation: 943.48; decimalLatitude: 43.17227; decimalLongitude: -4.90857; geodeticDatum: WGS84; **Event:** eventID: E; samplingProtocol: Pitfall
- **Type status:** Other material. **Occurrence:** individualCount: 6; sex: male; **Location:** locationID: P4; continent: Europe; country: Spain; countryCode: ES; stateProvince: Castilla y León; county: León; locality: El Canto; verbatimElevation: 943.48; decimalLatitude: 43.17227; decimalLongitude: -4.90857; geodeticDatum: WGS84; **Event:** eventID: F; samplingProtocol: Pitfall
- **Type status:** Other material. **Occurrence:** individualCount: 1; sex: female; **Location:** locationID: P4; continent: Europe; country: Spain; countryCode: ES; stateProvince: Castilla y León; county: León; locality: El Canto; verbatimElevation: 943.48; decimalLatitude: 43.17227; decimalLongitude: -4.90857; geodeticDatum: WGS84; **Event:** eventID: F; samplingProtocol: Pitfall
- **Type status:** Other material. **Occurrence:** individualCount: 4; sex: male; **Location:** locationID: P4; continent: Europe; country: Spain; countryCode: ES; stateProvince: Castilla y León; county: León; locality: El Canto; verbatimElevation: 943.48; decimalLatitude: 43.17227; decimalLongitude: -4.90857; geodeticDatum: WGS84; **Event:** eventID: G; samplingProtocol: Pitfall
- **Type status:** Other material. **Occurrence:** individualCount: 1; sex: female; **Location:** locationID: P4; continent: Europe; country: Spain; countryCode: ES; stateProvince: Castilla y León; county: León; locality: El Canto; verbatimElevation: 943.48; decimalLatitude: 43.17227; decimalLongitude: -4.90857; geodeticDatum: WGS84; **Event:** eventID: G; samplingProtocol: Pitfall
- **Type status:** Other material. **Occurrence:** individualCount: 1; sex: male; **Location:** locationID: P4; continent: Europe; country: Spain; countryCode: ES; stateProvince: Castilla y León; county: León; locality: El Canto; verbatimElevation: 943.48; decimalLatitude: 43.17227; decimalLongitude: -4.90857; geodeticDatum: WGS84; **Event:** eventID: H; samplingProtocol: Pitfall
- **Type status:** Other material. **Occurrence:** individualCount: 2; sex: female; **Location:** locationID: P4; continent: Europe; country: Spain; countryCode: ES; stateProvince: Castilla y León; county: León; locality: El Canto; verbatimElevation: 943.48; decimalLatitude: 43.17227; decimalLongitude: -4.90857; geodeticDatum: WGS84; **Event:** eventID: H; samplingProtocol: Pitfall
- **Type status:** Other material. **Occurrence:** individualCount: 2; sex: male; **Location:** locationID: P4; continent: Europe; country: Spain; countryCode: ES; stateProvince: Castilla y León; county: León; locality: El Canto; verbatimElevation: 943.48; decimalLatitude: 43.17227; decimalLongitude: -4.90857; geodeticDatum: WGS84; **Event:** eventID: I; samplingProtocol: Pitfall
- **Type status:** Other material. **Occurrence:** individualCount: 1; sex: male; **Location:** locationID: P4; continent: Europe; country: Spain; countryCode: ES; stateProvince: Castilla y León; county: León; locality: El Canto; verbatimElevation: 943.48; decimalLatitude: 43.17227; decimalLongitude: -4.90857; geodeticDatum: WGS84; **Event:** eventID: J; samplingProtocol: Pitfall

##### Distribution

Europe to Central Asia

#### Drassyllus villicus

(Thorell, 1875)

##### Materials

- **Type status:** Other material. **Occurrence:** individualCount: 2; sex: female; **Location:** locationID: C2; continent: Europe; country: Spain; countryCode: ES; stateProvince: Castilla-La Mancha; county: Ciudad Real; locality: Valle Brezoso; verbatimElevation: 739.31; decimalLatitude: 39.35159; decimalLongitude: -4.3589; geodeticDatum: WGS84; **Event:** eventID: B; samplingProtocol: Pitfall
- **Type status:** Other material. **Occurrence:** individualCount: 3; sex: female; **Location:** locationID: C2; continent: Europe; country: Spain; countryCode: ES; stateProvince: Castilla-La Mancha; county: Ciudad Real; locality: Valle Brezoso; verbatimElevation: 739.31; decimalLatitude: 39.35159; decimalLongitude: -4.3589; geodeticDatum: WGS84; **Event:** eventID: C; samplingProtocol: Pitfall
- **Type status:** Other material. **Occurrence:** individualCount: 1; sex: female; **Location:** locationID: C2; continent: Europe; country: Spain; countryCode: ES; stateProvince: Castilla-La Mancha; county: Ciudad Real; locality: Valle Brezoso; verbatimElevation: 739.31; decimalLatitude: 39.35159; decimalLongitude: -4.3589; geodeticDatum: WGS84; **Event:** eventID: G; samplingProtocol: Pitfall
- **Type status:** Other material. **Occurrence:** individualCount: 1; sex: female; **Location:** locationID: C2; continent: Europe; country: Spain; countryCode: ES; stateProvince: Castilla-La Mancha; county: Ciudad Real; locality: Valle Brezoso; verbatimElevation: 739.31; decimalLatitude: 39.35159; decimalLongitude: -4.3589; geodeticDatum: WGS84; **Event:** eventID: J; samplingProtocol: Pitfall
- **Type status:** Other material. **Occurrence:** individualCount: 1; sex: female; **Location:** locationID: C2; continent: Europe; country: Spain; countryCode: ES; stateProvince: Castilla-La Mancha; county: Ciudad Real; locality: Valle Brezoso; verbatimElevation: 739.31; decimalLatitude: 39.35159; decimalLongitude: -4.3589; geodeticDatum: WGS84; **Event:** eventID: K; samplingProtocol: Pitfall
- **Type status:** Other material. **Occurrence:** individualCount: 1; sex: female; **Location:** locationID: C2; continent: Europe; country: Spain; countryCode: ES; stateProvince: Castilla-La Mancha; county: Ciudad Real; locality: Valle Brezoso; verbatimElevation: 739.31; decimalLatitude: 39.35159; decimalLongitude: -4.3589; geodeticDatum: WGS84; **Event:** eventID: L; samplingProtocol: Pitfall
- **Type status:** Other material. **Occurrence:** individualCount: 1; sex: male; **Location:** locationID: O2; continent: Europe; country: Spain; countryCode: ES; stateProvince: Aragón; county: Huesca; locality: Rebilla; verbatimElevation: 1158.13; decimalLatitude: 42.59427; decimalLongitude: 0.1529; geodeticDatum: WGS84; **Event:** eventID: C; samplingProtocol: Pitfall
- **Type status:** Other material. **Occurrence:** individualCount: 1; sex: male; **Location:** locationID: O2; continent: Europe; country: Spain; countryCode: ES; stateProvince: Aragón; county: Huesca; locality: Rebilla; verbatimElevation: 1158.13; decimalLatitude: 42.59427; decimalLongitude: 0.1529; geodeticDatum: WGS84; **Event:** eventID: F; samplingProtocol: Pitfall
- **Type status:** Other material. **Occurrence:** individualCount: 1; sex: male; **Location:** locationID: O2; continent: Europe; country: Spain; countryCode: ES; stateProvince: Aragón; county: Huesca; locality: Rebilla; verbatimElevation: 1158.13; decimalLatitude: 42.59427; decimalLongitude: 0.1529; geodeticDatum: WGS84; **Event:** eventID: G; samplingProtocol: Pitfall
- **Type status:** Other material. **Occurrence:** individualCount: 3; sex: male; **Location:** locationID: O2; continent: Europe; country: Spain; countryCode: ES; stateProvince: Aragón; county: Huesca; locality: Rebilla; verbatimElevation: 1158.13; decimalLatitude: 42.59427; decimalLongitude: 0.1529; geodeticDatum: WGS84; **Event:** eventID: K; samplingProtocol: Pitfall
- **Type status:** Other material. **Occurrence:** individualCount: 1; sex: female; **Location:** locationID: O2; continent: Europe; country: Spain; countryCode: ES; stateProvince: Aragón; county: Huesca; locality: Rebilla; verbatimElevation: 1158.13; decimalLatitude: 42.59427; decimalLongitude: 0.1529; geodeticDatum: WGS84; **Event:** eventID: K; samplingProtocol: Pitfall
- **Type status:** Other material. **Occurrence:** individualCount: 1; sex: male; **Location:** locationID: O2; continent: Europe; country: Spain; countryCode: ES; stateProvince: Aragón; county: Huesca; locality: Rebilla; verbatimElevation: 1158.13; decimalLatitude: 42.59427; decimalLongitude: 0.1529; geodeticDatum: WGS84; **Event:** eventID: L; samplingProtocol: Pitfall

##### Distribution

Europe

#### Haplodrassus dalmatensis

(L. Koch, 1866)

##### Materials

- **Type status:** Other material. **Occurrence:** individualCount: 1; sex: female; **Location:** locationID: P3; continent: Europe; country: Spain; countryCode: ES; stateProvince: Castilla y León; county: León; locality: Las Arroyas; verbatimElevation: 1097.1; decimalLatitude: 43.14351; decimalLongitude: -4.94878; geodeticDatum: WGS84; **Event:** eventID: H; samplingProtocol: Pitfall

##### Distribution

Palearctic

#### Haplodrassus cf. macellinus

(Thorell, 1871)

##### Materials

- **Type status:** Other material. **Occurrence:** individualCount: 1; sex: male; **Location:** locationID: S1; continent: Europe; country: Spain; countryCode: ES; stateProvince: Andalucía; county: Granada; locality: Soportujar; verbatimElevation: 1786.57; decimalLatitude: 36.96151; decimalLongitude: -3.41881; geodeticDatum: WGS84; **Event:** eventID: E; samplingProtocol: Pitfall
- **Type status:** Other material. **Occurrence:** individualCount: 1; sex: male; **Location:** locationID: S1; continent: Europe; country: Spain; countryCode: ES; stateProvince: Andalucía; county: Granada; locality: Soportujar; verbatimElevation: 1786.57; decimalLatitude: 36.96151; decimalLongitude: -3.41881; geodeticDatum: WGS84; **Event:** eventID: F; samplingProtocol: Pitfall
- **Type status:** Other material. **Occurrence:** individualCount: 1; sex: male; **Location:** locationID: S1; continent: Europe; country: Spain; countryCode: ES; stateProvince: Andalucía; county: Granada; locality: Soportujar; verbatimElevation: 1786.57; decimalLatitude: 36.96151; decimalLongitude: -3.41881; geodeticDatum: WGS84; **Event:** eventID: L; samplingProtocol: Pitfall
- **Type status:** Other material. **Occurrence:** individualCount: 1; sex: female; **Location:** locationID: S1; continent: Europe; country: Spain; countryCode: ES; stateProvince: Andalucía; county: Granada; locality: Soportujar; verbatimElevation: 1786.57; decimalLatitude: 36.96151; decimalLongitude: -3.41881; geodeticDatum: WGS84; **Event:** eventID: L; samplingProtocol: Pitfall
- **Type status:** Other material. **Occurrence:** individualCount: 1; sex: male; **Location:** locationID: S2; continent: Europe; country: Spain; countryCode: ES; stateProvince: Andalucía; county: Granada; locality: Camarate; verbatimElevation: 1713.96; decimalLatitude: 37.18377; decimalLongitude: -3.26282; geodeticDatum: WGS84; **Event:** eventID: B; samplingProtocol: Pitfall
- **Type status:** Other material. **Occurrence:** individualCount: 1; sex: male; **Location:** locationID: S2; continent: Europe; country: Spain; countryCode: ES; stateProvince: Andalucía; county: Granada; locality: Camarate; verbatimElevation: 1713.96; decimalLatitude: 37.18377; decimalLongitude: -3.26282; geodeticDatum: WGS84; **Event:** eventID: E; samplingProtocol: Pitfall
- **Type status:** Other material. **Occurrence:** individualCount: 1; sex: male; **Location:** locationID: S2; continent: Europe; country: Spain; countryCode: ES; stateProvince: Andalucía; county: Granada; locality: Camarate; verbatimElevation: 1713.96; decimalLatitude: 37.18377; decimalLongitude: -3.26282; geodeticDatum: WGS84; **Event:** eventID: J; samplingProtocol: Pitfall

##### Distribution

Western Mediterranean

##### Notes

Though no good descriptions are available for this species, we opted to identify the specimens here cited as *H. macellinus* due to the knife-like terminal apophysis, very similar to that illustrated by Simon (Simon 1914).

#### Haplodrassus signifer

(C. L. Koch, 1839)

##### Materials

- **Type status:** Other material. **Occurrence:** individualCount: 1; sex: female; **Location:** locationID: A1; continent: Europe; country: Spain; countryCode: ES; stateProvince: Catalonia; county: Lleida; locality: Sola de Boi; verbatimElevation: 1759.8; decimalLatitude: 42.54958; decimalLongitude: 0.87254; geodeticDatum: WGS84; **Event:** eventID: 1; samplingProtocol: Aerial; eventTime: Night
- **Type status:** Other material. **Occurrence:** individualCount: 1; sex: male; **Location:** locationID: A1; continent: Europe; country: Spain; countryCode: ES; stateProvince: Catalonia; county: Lleida; locality: Sola de Boi; verbatimElevation: 1759.8; decimalLatitude: 42.54958; decimalLongitude: 0.87254; geodeticDatum: WGS84; **Event:** eventID: A; samplingProtocol: Pitfall
- **Type status:** Other material. **Occurrence:** individualCount: 1; sex: male; **Location:** locationID: A1; continent: Europe; country: Spain; countryCode: ES; stateProvince: Catalonia; county: Lleida; locality: Sola de Boi; verbatimElevation: 1759.8; decimalLatitude: 42.54958; decimalLongitude: 0.87254; geodeticDatum: WGS84; **Event:** eventID: B; samplingProtocol: Pitfall
- **Type status:** Other material. **Occurrence:** individualCount: 1; sex: male; **Location:** locationID: A1; continent: Europe; country: Spain; countryCode: ES; stateProvince: Catalonia; county: Lleida; locality: Sola de Boi; verbatimElevation: 1759.8; decimalLatitude: 42.54958; decimalLongitude: 0.87254; geodeticDatum: WGS84; **Event:** eventID: C; samplingProtocol: Pitfall
- **Type status:** Other material. **Occurrence:** individualCount: 2; sex: female; **Location:** locationID: A1; continent: Europe; country: Spain; countryCode: ES; stateProvince: Catalonia; county: Lleida; locality: Sola de Boi; verbatimElevation: 1759.8; decimalLatitude: 42.54958; decimalLongitude: 0.87254; geodeticDatum: WGS84; **Event:** eventID: C; samplingProtocol: Pitfall
- **Type status:** Other material. **Occurrence:** individualCount: 5; sex: female; **Location:** locationID: A1; continent: Europe; country: Spain; countryCode: ES; stateProvince: Catalonia; county: Lleida; locality: Sola de Boi; verbatimElevation: 1759.8; decimalLatitude: 42.54958; decimalLongitude: 0.87254; geodeticDatum: WGS84; **Event:** eventID: D; samplingProtocol: Pitfall
- **Type status:** Other material. **Occurrence:** individualCount: 3; sex: female; **Location:** locationID: A1; continent: Europe; country: Spain; countryCode: ES; stateProvince: Catalonia; county: Lleida; locality: Sola de Boi; verbatimElevation: 1759.8; decimalLatitude: 42.54958; decimalLongitude: 0.87254; geodeticDatum: WGS84; **Event:** eventID: E; samplingProtocol: Pitfall
- **Type status:** Other material. **Occurrence:** individualCount: 1; sex: male; **Location:** locationID: A1; continent: Europe; country: Spain; countryCode: ES; stateProvince: Catalonia; county: Lleida; locality: Sola de Boi; verbatimElevation: 1759.8; decimalLatitude: 42.54958; decimalLongitude: 0.87254; geodeticDatum: WGS84; **Event:** eventID: F; samplingProtocol: Pitfall
- **Type status:** Other material. **Occurrence:** individualCount: 2; sex: female; **Location:** locationID: A1; continent: Europe; country: Spain; countryCode: ES; stateProvince: Catalonia; county: Lleida; locality: Sola de Boi; verbatimElevation: 1759.8; decimalLatitude: 42.54958; decimalLongitude: 0.87254; geodeticDatum: WGS84; **Event:** eventID: F; samplingProtocol: Pitfall
- **Type status:** Other material. **Occurrence:** individualCount: 2; sex: female; **Location:** locationID: A1; continent: Europe; country: Spain; countryCode: ES; stateProvince: Catalonia; county: Lleida; locality: Sola de Boi; verbatimElevation: 1759.8; decimalLatitude: 42.54958; decimalLongitude: 0.87254; geodeticDatum: WGS84; **Event:** eventID: G; samplingProtocol: Pitfall
- **Type status:** Other material. **Occurrence:** individualCount: 1; sex: male; **Location:** locationID: A1; continent: Europe; country: Spain; countryCode: ES; stateProvince: Catalonia; county: Lleida; locality: Sola de Boi; verbatimElevation: 1759.8; decimalLatitude: 42.54958; decimalLongitude: 0.87254; geodeticDatum: WGS84; **Event:** eventID: H; samplingProtocol: Pitfall
- **Type status:** Other material. **Occurrence:** individualCount: 3; sex: female; **Location:** locationID: A1; continent: Europe; country: Spain; countryCode: ES; stateProvince: Catalonia; county: Lleida; locality: Sola de Boi; verbatimElevation: 1759.8; decimalLatitude: 42.54958; decimalLongitude: 0.87254; geodeticDatum: WGS84; **Event:** eventID: H; samplingProtocol: Pitfall
- **Type status:** Other material. **Occurrence:** individualCount: 5; sex: male; **Location:** locationID: A1; continent: Europe; country: Spain; countryCode: ES; stateProvince: Catalonia; county: Lleida; locality: Sola de Boi; verbatimElevation: 1759.8; decimalLatitude: 42.54958; decimalLongitude: 0.87254; geodeticDatum: WGS84; **Event:** eventID: I; samplingProtocol: Pitfall
- **Type status:** Other material. **Occurrence:** individualCount: 1; sex: male; **Location:** locationID: A1; continent: Europe; country: Spain; countryCode: ES; stateProvince: Catalonia; county: Lleida; locality: Sola de Boi; verbatimElevation: 1759.8; decimalLatitude: 42.54958; decimalLongitude: 0.87254; geodeticDatum: WGS84; **Event:** eventID: L; samplingProtocol: Pitfall
- **Type status:** Other material. **Occurrence:** individualCount: 3; sex: female; **Location:** locationID: A1; continent: Europe; country: Spain; countryCode: ES; stateProvince: Catalonia; county: Lleida; locality: Sola de Boi; verbatimElevation: 1759.8; decimalLatitude: 42.54958; decimalLongitude: 0.87254; geodeticDatum: WGS84; **Event:** eventID: L; samplingProtocol: Pitfall
- **Type status:** Other material. **Occurrence:** individualCount: 2; sex: male; **Location:** locationID: A2; continent: Europe; country: Spain; countryCode: ES; stateProvince: Catalonia; county: Lleida; locality: Sola de Boi; verbatimElevation: 1738.7; decimalLatitude: 42.54913; decimalLongitude: 0.87137; geodeticDatum: WGS84; **Event:** eventID: A; samplingProtocol: Pitfall
- **Type status:** Other material. **Occurrence:** individualCount: 1; sex: female; **Location:** locationID: A2; continent: Europe; country: Spain; countryCode: ES; stateProvince: Catalonia; county: Lleida; locality: Sola de Boi; verbatimElevation: 1738.7; decimalLatitude: 42.54913; decimalLongitude: 0.87137; geodeticDatum: WGS84; **Event:** eventID: A; samplingProtocol: Pitfall
- **Type status:** Other material. **Occurrence:** individualCount: 1; sex: male; **Location:** locationID: A2; continent: Europe; country: Spain; countryCode: ES; stateProvince: Catalonia; county: Lleida; locality: Sola de Boi; verbatimElevation: 1738.7; decimalLatitude: 42.54913; decimalLongitude: 0.87137; geodeticDatum: WGS84; **Event:** eventID: B; samplingProtocol: Pitfall
- **Type status:** Other material. **Occurrence:** individualCount: 1; sex: male; **Location:** locationID: A2; continent: Europe; country: Spain; countryCode: ES; stateProvince: Catalonia; county: Lleida; locality: Sola de Boi; verbatimElevation: 1738.7; decimalLatitude: 42.54913; decimalLongitude: 0.87137; geodeticDatum: WGS84; **Event:** eventID: C; samplingProtocol: Pitfall
- **Type status:** Other material. **Occurrence:** individualCount: 2; sex: female; **Location:** locationID: A2; continent: Europe; country: Spain; countryCode: ES; stateProvince: Catalonia; county: Lleida; locality: Sola de Boi; verbatimElevation: 1738.7; decimalLatitude: 42.54913; decimalLongitude: 0.87137; geodeticDatum: WGS84; **Event:** eventID: E; samplingProtocol: Pitfall
- **Type status:** Other material. **Occurrence:** individualCount: 1; sex: male; **Location:** locationID: A2; continent: Europe; country: Spain; countryCode: ES; stateProvince: Catalonia; county: Lleida; locality: Sola de Boi; verbatimElevation: 1738.7; decimalLatitude: 42.54913; decimalLongitude: 0.87137; geodeticDatum: WGS84; **Event:** eventID: F; samplingProtocol: Pitfall
- **Type status:** Other material. **Occurrence:** individualCount: 1; sex: female; **Location:** locationID: A2; continent: Europe; country: Spain; countryCode: ES; stateProvince: Catalonia; county: Lleida; locality: Sola de Boi; verbatimElevation: 1738.7; decimalLatitude: 42.54913; decimalLongitude: 0.87137; geodeticDatum: WGS84; **Event:** eventID: G; samplingProtocol: Pitfall
- **Type status:** Other material. **Occurrence:** individualCount: 2; sex: male; **Location:** locationID: A2; continent: Europe; country: Spain; countryCode: ES; stateProvince: Catalonia; county: Lleida; locality: Sola de Boi; verbatimElevation: 1738.7; decimalLatitude: 42.54913; decimalLongitude: 0.87137; geodeticDatum: WGS84; **Event:** eventID: H; samplingProtocol: Pitfall
- **Type status:** Other material. **Occurrence:** individualCount: 1; sex: female; **Location:** locationID: A2; continent: Europe; country: Spain; countryCode: ES; stateProvince: Catalonia; county: Lleida; locality: Sola de Boi; verbatimElevation: 1738.7; decimalLatitude: 42.54913; decimalLongitude: 0.87137; geodeticDatum: WGS84; **Event:** eventID: H; samplingProtocol: Pitfall
- **Type status:** Other material. **Occurrence:** individualCount: 3; sex: female; **Location:** locationID: A2; continent: Europe; country: Spain; countryCode: ES; stateProvince: Catalonia; county: Lleida; locality: Sola de Boi; verbatimElevation: 1738.7; decimalLatitude: 42.54913; decimalLongitude: 0.87137; geodeticDatum: WGS84; **Event:** eventID: I; samplingProtocol: Pitfall
- **Type status:** Other material. **Occurrence:** individualCount: 1; sex: female; **Location:** locationID: A2; continent: Europe; country: Spain; countryCode: ES; stateProvince: Catalonia; county: Lleida; locality: Sola de Boi; verbatimElevation: 1738.7; decimalLatitude: 42.54913; decimalLongitude: 0.87137; geodeticDatum: WGS84; **Event:** eventID: J; samplingProtocol: Pitfall
- **Type status:** Other material. **Occurrence:** individualCount: 1; sex: male; **Location:** locationID: A2; continent: Europe; country: Spain; countryCode: ES; stateProvince: Catalonia; county: Lleida; locality: Sola de Boi; verbatimElevation: 1738.7; decimalLatitude: 42.54913; decimalLongitude: 0.87137; geodeticDatum: WGS84; **Event:** eventID: L; samplingProtocol: Pitfall
- **Type status:** Other material. **Occurrence:** individualCount: 1; sex: male; **Location:** locationID: P2; continent: Europe; country: Spain; countryCode: ES; stateProvince: Castilla y León; county: León; locality: Joyoguelas; verbatimElevation: 763.98; decimalLatitude: 43.17771; decimalLongitude: -4.90579; geodeticDatum: WGS84; **Event:** eventID: C; samplingProtocol: Pitfall
- **Type status:** Other material. **Occurrence:** individualCount: 1; sex: female; **Location:** locationID: P4; continent: Europe; country: Spain; countryCode: ES; stateProvince: Castilla y León; county: León; locality: El Canto; verbatimElevation: 943.48; decimalLatitude: 43.17227; decimalLongitude: -4.90857; geodeticDatum: WGS84; **Event:** eventID: A; samplingProtocol: Pitfall
- **Type status:** Other material. **Occurrence:** individualCount: 2; sex: male; **Location:** locationID: P4; continent: Europe; country: Spain; countryCode: ES; stateProvince: Castilla y León; county: León; locality: El Canto; verbatimElevation: 943.48; decimalLatitude: 43.17227; decimalLongitude: -4.90857; geodeticDatum: WGS84; **Event:** eventID: C; samplingProtocol: Pitfall
- **Type status:** Other material. **Occurrence:** individualCount: 1; sex: male; **Location:** locationID: P4; continent: Europe; country: Spain; countryCode: ES; stateProvince: Castilla y León; county: León; locality: El Canto; verbatimElevation: 943.48; decimalLatitude: 43.17227; decimalLongitude: -4.90857; geodeticDatum: WGS84; **Event:** eventID: D; samplingProtocol: Pitfall

##### Distribution

Holarctic

#### Haplodrassus silvestris

(Blackwall, 1833)

##### Materials

- **Type status:** Other material. **Occurrence:** individualCount: 1; sex: male; **Location:** locationID: A1; continent: Europe; country: Spain; countryCode: ES; stateProvince: Catalonia; county: Lleida; locality: Sola de Boi; verbatimElevation: 1759.8; decimalLatitude: 42.54958; decimalLongitude: 0.87254; geodeticDatum: WGS84; **Event:** eventID: D; samplingProtocol: Pitfall
- **Type status:** Other material. **Occurrence:** individualCount: 2; sex: male; **Location:** locationID: A1; continent: Europe; country: Spain; countryCode: ES; stateProvince: Catalonia; county: Lleida; locality: Sola de Boi; verbatimElevation: 1759.8; decimalLatitude: 42.54958; decimalLongitude: 0.87254; geodeticDatum: WGS84; **Event:** eventID: L; samplingProtocol: Pitfall
- **Type status:** Other material. **Occurrence:** individualCount: 5; sex: male; **Location:** locationID: A2; continent: Europe; country: Spain; countryCode: ES; stateProvince: Catalonia; county: Lleida; locality: Sola de Boi; verbatimElevation: 1738.7; decimalLatitude: 42.54913; decimalLongitude: 0.87137; geodeticDatum: WGS84; **Event:** eventID: C; samplingProtocol: Pitfall
- **Type status:** Other material. **Occurrence:** individualCount: 1; sex: female; **Location:** locationID: A2; continent: Europe; country: Spain; countryCode: ES; stateProvince: Catalonia; county: Lleida; locality: Sola de Boi; verbatimElevation: 1738.7; decimalLatitude: 42.54913; decimalLongitude: 0.87137; geodeticDatum: WGS84; **Event:** eventID: C; samplingProtocol: Pitfall
- **Type status:** Other material. **Occurrence:** individualCount: 3; sex: male; **Location:** locationID: A2; continent: Europe; country: Spain; countryCode: ES; stateProvince: Catalonia; county: Lleida; locality: Sola de Boi; verbatimElevation: 1738.7; decimalLatitude: 42.54913; decimalLongitude: 0.87137; geodeticDatum: WGS84; **Event:** eventID: D; samplingProtocol: Pitfall
- **Type status:** Other material. **Occurrence:** individualCount: 1; sex: female; **Location:** locationID: A2; continent: Europe; country: Spain; countryCode: ES; stateProvince: Catalonia; county: Lleida; locality: Sola de Boi; verbatimElevation: 1738.7; decimalLatitude: 42.54913; decimalLongitude: 0.87137; geodeticDatum: WGS84; **Event:** eventID: D; samplingProtocol: Pitfall
- **Type status:** Other material. **Occurrence:** individualCount: 1; sex: male; **Location:** locationID: A2; continent: Europe; country: Spain; countryCode: ES; stateProvince: Catalonia; county: Lleida; locality: Sola de Boi; verbatimElevation: 1738.7; decimalLatitude: 42.54913; decimalLongitude: 0.87137; geodeticDatum: WGS84; **Event:** eventID: E; samplingProtocol: Pitfall
- **Type status:** Other material. **Occurrence:** individualCount: 3; sex: male; **Location:** locationID: A2; continent: Europe; country: Spain; countryCode: ES; stateProvince: Catalonia; county: Lleida; locality: Sola de Boi; verbatimElevation: 1738.7; decimalLatitude: 42.54913; decimalLongitude: 0.87137; geodeticDatum: WGS84; **Event:** eventID: G; samplingProtocol: Pitfall
- **Type status:** Other material. **Occurrence:** individualCount: 2; sex: female; **Location:** locationID: A2; continent: Europe; country: Spain; countryCode: ES; stateProvince: Catalonia; county: Lleida; locality: Sola de Boi; verbatimElevation: 1738.7; decimalLatitude: 42.54913; decimalLongitude: 0.87137; geodeticDatum: WGS84; **Event:** eventID: G; samplingProtocol: Pitfall
- **Type status:** Other material. **Occurrence:** individualCount: 1; sex: male; **Location:** locationID: A2; continent: Europe; country: Spain; countryCode: ES; stateProvince: Catalonia; county: Lleida; locality: Sola de Boi; verbatimElevation: 1738.7; decimalLatitude: 42.54913; decimalLongitude: 0.87137; geodeticDatum: WGS84; **Event:** eventID: H; samplingProtocol: Pitfall
- **Type status:** Other material. **Occurrence:** individualCount: 2; sex: female; **Location:** locationID: A2; continent: Europe; country: Spain; countryCode: ES; stateProvince: Catalonia; county: Lleida; locality: Sola de Boi; verbatimElevation: 1738.7; decimalLatitude: 42.54913; decimalLongitude: 0.87137; geodeticDatum: WGS84; **Event:** eventID: H; samplingProtocol: Pitfall
- **Type status:** Other material. **Occurrence:** individualCount: 1; sex: female; **Location:** locationID: A2; continent: Europe; country: Spain; countryCode: ES; stateProvince: Catalonia; county: Lleida; locality: Sola de Boi; verbatimElevation: 1738.7; decimalLatitude: 42.54913; decimalLongitude: 0.87137; geodeticDatum: WGS84; **Event:** eventID: I; samplingProtocol: Pitfall
- **Type status:** Other material. **Occurrence:** individualCount: 2; sex: female; **Location:** locationID: A2; continent: Europe; country: Spain; countryCode: ES; stateProvince: Catalonia; county: Lleida; locality: Sola de Boi; verbatimElevation: 1738.7; decimalLatitude: 42.54913; decimalLongitude: 0.87137; geodeticDatum: WGS84; **Event:** eventID: J; samplingProtocol: Pitfall
- **Type status:** Other material. **Occurrence:** individualCount: 2; sex: male; **Location:** locationID: P1; continent: Europe; country: Spain; countryCode: ES; stateProvince: Castilla y León; county: León; locality: Monte Robledo; verbatimElevation: 1071.58; decimalLatitude: 43.1445; decimalLongitude: -4.92675; geodeticDatum: WGS84; **Event:** eventID: A; samplingProtocol: Pitfall
- **Type status:** Other material. **Occurrence:** individualCount: 2; sex: female; **Location:** locationID: P1; continent: Europe; country: Spain; countryCode: ES; stateProvince: Castilla y León; county: León; locality: Monte Robledo; verbatimElevation: 1071.58; decimalLatitude: 43.1445; decimalLongitude: -4.92675; geodeticDatum: WGS84; **Event:** eventID: B; samplingProtocol: Pitfall
- **Type status:** Other material. **Occurrence:** individualCount: 2; sex: male; **Location:** locationID: P1; continent: Europe; country: Spain; countryCode: ES; stateProvince: Castilla y León; county: León; locality: Monte Robledo; verbatimElevation: 1071.58; decimalLatitude: 43.1445; decimalLongitude: -4.92675; geodeticDatum: WGS84; **Event:** eventID: E; samplingProtocol: Pitfall
- **Type status:** Other material. **Occurrence:** individualCount: 1; sex: male; **Location:** locationID: P1; continent: Europe; country: Spain; countryCode: ES; stateProvince: Castilla y León; county: León; locality: Monte Robledo; verbatimElevation: 1071.58; decimalLatitude: 43.1445; decimalLongitude: -4.92675; geodeticDatum: WGS84; **Event:** eventID: F; samplingProtocol: Pitfall
- **Type status:** Other material. **Occurrence:** individualCount: 1; sex: female; **Location:** locationID: P1; continent: Europe; country: Spain; countryCode: ES; stateProvince: Castilla y León; county: León; locality: Monte Robledo; verbatimElevation: 1071.58; decimalLatitude: 43.1445; decimalLongitude: -4.92675; geodeticDatum: WGS84; **Event:** eventID: F; samplingProtocol: Pitfall
- **Type status:** Other material. **Occurrence:** individualCount: 1; sex: male; **Location:** locationID: P1; continent: Europe; country: Spain; countryCode: ES; stateProvince: Castilla y León; county: León; locality: Monte Robledo; verbatimElevation: 1071.58; decimalLatitude: 43.1445; decimalLongitude: -4.92675; geodeticDatum: WGS84; **Event:** eventID: J; samplingProtocol: Pitfall
- **Type status:** Other material. **Occurrence:** individualCount: 1; sex: male; **Location:** locationID: P1; continent: Europe; country: Spain; countryCode: ES; stateProvince: Castilla y León; county: León; locality: Monte Robledo; verbatimElevation: 1071.58; decimalLatitude: 43.1445; decimalLongitude: -4.92675; geodeticDatum: WGS84; **Event:** eventID: K; samplingProtocol: Pitfall
- **Type status:** Other material. **Occurrence:** individualCount: 1; sex: male; **Location:** locationID: P2; continent: Europe; country: Spain; countryCode: ES; stateProvince: Castilla y León; county: León; locality: Joyoguelas; verbatimElevation: 763.98; decimalLatitude: 43.17771; decimalLongitude: -4.90579; geodeticDatum: WGS84; **Event:** eventID: E; samplingProtocol: Pitfall
- **Type status:** Other material. **Occurrence:** individualCount: 1; sex: female; **Location:** locationID: P2; continent: Europe; country: Spain; countryCode: ES; stateProvince: Castilla y León; county: León; locality: Joyoguelas; verbatimElevation: 763.98; decimalLatitude: 43.17771; decimalLongitude: -4.90579; geodeticDatum: WGS84; **Event:** eventID: E; samplingProtocol: Pitfall
- **Type status:** Other material. **Occurrence:** individualCount: 1; sex: male; **Location:** locationID: P2; continent: Europe; country: Spain; countryCode: ES; stateProvince: Castilla y León; county: León; locality: Joyoguelas; verbatimElevation: 763.98; decimalLatitude: 43.17771; decimalLongitude: -4.90579; geodeticDatum: WGS84; **Event:** eventID: F; samplingProtocol: Pitfall
- **Type status:** Other material. **Occurrence:** individualCount: 2; sex: female; **Location:** locationID: P2; continent: Europe; country: Spain; countryCode: ES; stateProvince: Castilla y León; county: León; locality: Joyoguelas; verbatimElevation: 763.98; decimalLatitude: 43.17771; decimalLongitude: -4.90579; geodeticDatum: WGS84; **Event:** eventID: F; samplingProtocol: Pitfall
- **Type status:** Other material. **Occurrence:** individualCount: 1; sex: female; **Location:** locationID: P2; continent: Europe; country: Spain; countryCode: ES; stateProvince: Castilla y León; county: León; locality: Joyoguelas; verbatimElevation: 763.98; decimalLatitude: 43.17771; decimalLongitude: -4.90579; geodeticDatum: WGS84; **Event:** eventID: G; samplingProtocol: Pitfall
- **Type status:** Other material. **Occurrence:** individualCount: 3; sex: male; **Location:** locationID: P2; continent: Europe; country: Spain; countryCode: ES; stateProvince: Castilla y León; county: León; locality: Joyoguelas; verbatimElevation: 763.98; decimalLatitude: 43.17771; decimalLongitude: -4.90579; geodeticDatum: WGS84; **Event:** eventID: K; samplingProtocol: Pitfall
- **Type status:** Other material. **Occurrence:** individualCount: 2; sex: male; **Location:** locationID: P3; continent: Europe; country: Spain; countryCode: ES; stateProvince: Castilla y León; county: León; locality: Las Arroyas; verbatimElevation: 1097.1; decimalLatitude: 43.14351; decimalLongitude: -4.94878; geodeticDatum: WGS84; **Event:** eventID: A; samplingProtocol: Pitfall
- **Type status:** Other material. **Occurrence:** individualCount: 1; sex: male; **Location:** locationID: P3; continent: Europe; country: Spain; countryCode: ES; stateProvince: Castilla y León; county: León; locality: Las Arroyas; verbatimElevation: 1097.1; decimalLatitude: 43.14351; decimalLongitude: -4.94878; geodeticDatum: WGS84; **Event:** eventID: C; samplingProtocol: Pitfall
- **Type status:** Other material. **Occurrence:** individualCount: 1; sex: male; **Location:** locationID: P3; continent: Europe; country: Spain; countryCode: ES; stateProvince: Castilla y León; county: León; locality: Las Arroyas; verbatimElevation: 1097.1; decimalLatitude: 43.14351; decimalLongitude: -4.94878; geodeticDatum: WGS84; **Event:** eventID: F; samplingProtocol: Pitfall
- **Type status:** Other material. **Occurrence:** individualCount: 1; sex: male; **Location:** locationID: P3; continent: Europe; country: Spain; countryCode: ES; stateProvince: Castilla y León; county: León; locality: Las Arroyas; verbatimElevation: 1097.1; decimalLatitude: 43.14351; decimalLongitude: -4.94878; geodeticDatum: WGS84; **Event:** eventID: H; samplingProtocol: Pitfall
- **Type status:** Other material. **Occurrence:** individualCount: 1; sex: male; **Location:** locationID: P3; continent: Europe; country: Spain; countryCode: ES; stateProvince: Castilla y León; county: León; locality: Las Arroyas; verbatimElevation: 1097.1; decimalLatitude: 43.14351; decimalLongitude: -4.94878; geodeticDatum: WGS84; **Event:** eventID: I; samplingProtocol: Pitfall
- **Type status:** Other material. **Occurrence:** individualCount: 1; sex: male; **Location:** locationID: P3; continent: Europe; country: Spain; countryCode: ES; stateProvince: Castilla y León; county: León; locality: Las Arroyas; verbatimElevation: 1097.1; decimalLatitude: 43.14351; decimalLongitude: -4.94878; geodeticDatum: WGS84; **Event:** eventID: K; samplingProtocol: Pitfall
- **Type status:** Other material. **Occurrence:** individualCount: 1; sex: male; **Location:** locationID: P4; continent: Europe; country: Spain; countryCode: ES; stateProvince: Castilla y León; county: León; locality: El Canto; verbatimElevation: 943.48; decimalLatitude: 43.17227; decimalLongitude: -4.90857; geodeticDatum: WGS84; **Event:** eventID: B; samplingProtocol: Pitfall
- **Type status:** Other material. **Occurrence:** individualCount: 2; sex: male; **Location:** locationID: P4; continent: Europe; country: Spain; countryCode: ES; stateProvince: Castilla y León; county: León; locality: El Canto; verbatimElevation: 943.48; decimalLatitude: 43.17227; decimalLongitude: -4.90857; geodeticDatum: WGS84; **Event:** eventID: C; samplingProtocol: Pitfall
- **Type status:** Other material. **Occurrence:** individualCount: 1; sex: female; **Location:** locationID: P4; continent: Europe; country: Spain; countryCode: ES; stateProvince: Castilla y León; county: León; locality: El Canto; verbatimElevation: 943.48; decimalLatitude: 43.17227; decimalLongitude: -4.90857; geodeticDatum: WGS84; **Event:** eventID: C; samplingProtocol: Pitfall
- **Type status:** Other material. **Occurrence:** individualCount: 1; sex: female; **Location:** locationID: P4; continent: Europe; country: Spain; countryCode: ES; stateProvince: Castilla y León; county: León; locality: El Canto; verbatimElevation: 943.48; decimalLatitude: 43.17227; decimalLongitude: -4.90857; geodeticDatum: WGS84; **Event:** eventID: D; samplingProtocol: Pitfall
- **Type status:** Other material. **Occurrence:** individualCount: 2; sex: male; **Location:** locationID: P4; continent: Europe; country: Spain; countryCode: ES; stateProvince: Castilla y León; county: León; locality: El Canto; verbatimElevation: 943.48; decimalLatitude: 43.17227; decimalLongitude: -4.90857; geodeticDatum: WGS84; **Event:** eventID: E; samplingProtocol: Pitfall
- **Type status:** Other material. **Occurrence:** individualCount: 1; sex: female; **Location:** locationID: P4; continent: Europe; country: Spain; countryCode: ES; stateProvince: Castilla y León; county: León; locality: El Canto; verbatimElevation: 943.48; decimalLatitude: 43.17227; decimalLongitude: -4.90857; geodeticDatum: WGS84; **Event:** eventID: G; samplingProtocol: Pitfall
- **Type status:** Other material. **Occurrence:** individualCount: 4; sex: male; **Location:** locationID: P4; continent: Europe; country: Spain; countryCode: ES; stateProvince: Castilla y León; county: León; locality: El Canto; verbatimElevation: 943.48; decimalLatitude: 43.17227; decimalLongitude: -4.90857; geodeticDatum: WGS84; **Event:** eventID: I; samplingProtocol: Pitfall
- **Type status:** Other material. **Occurrence:** individualCount: 1; sex: female; **Location:** locationID: P4; continent: Europe; country: Spain; countryCode: ES; stateProvince: Castilla y León; county: León; locality: El Canto; verbatimElevation: 943.48; decimalLatitude: 43.17227; decimalLongitude: -4.90857; geodeticDatum: WGS84; **Event:** eventID: I; samplingProtocol: Pitfall
- **Type status:** Other material. **Occurrence:** individualCount: 1; sex: male; **Location:** locationID: P4; continent: Europe; country: Spain; countryCode: ES; stateProvince: Castilla y León; county: León; locality: El Canto; verbatimElevation: 943.48; decimalLatitude: 43.17227; decimalLongitude: -4.90857; geodeticDatum: WGS84; **Event:** eventID: K; samplingProtocol: Pitfall
- **Type status:** Other material. **Occurrence:** individualCount: 1; sex: male; **Location:** locationID: P4; continent: Europe; country: Spain; countryCode: ES; stateProvince: Castilla y León; county: León; locality: El Canto; verbatimElevation: 943.48; decimalLatitude: 43.17227; decimalLongitude: -4.90857; geodeticDatum: WGS84; **Event:** eventID: L; samplingProtocol: Pitfall
- **Type status:** Other material. **Occurrence:** individualCount: 1; sex: female; **Location:** locationID: P4; continent: Europe; country: Spain; countryCode: ES; stateProvince: Castilla y León; county: León; locality: El Canto; verbatimElevation: 943.48; decimalLatitude: 43.17227; decimalLongitude: -4.90857; geodeticDatum: WGS84; **Event:** eventID: L; samplingProtocol: Pitfall

##### Distribution

Palearctic

#### Haplodrassus umbratilis

(L. Koch, 1866)

##### Materials

- **Type status:** Other material. **Occurrence:** individualCount: 1; sex: male; **Location:** locationID: A1; continent: Europe; country: Spain; countryCode: ES; stateProvince: Catalonia; county: Lleida; locality: Sola de Boi; verbatimElevation: 1759.8; decimalLatitude: 42.54958; decimalLongitude: 0.87254; geodeticDatum: WGS84; **Event:** eventID: 1; samplingProtocol: Ground; eventTime: Night
- **Type status:** Other material. **Occurrence:** individualCount: 1; sex: male; **Location:** locationID: A1; continent: Europe; country: Spain; countryCode: ES; stateProvince: Catalonia; county: Lleida; locality: Sola de Boi; verbatimElevation: 1759.8; decimalLatitude: 42.54958; decimalLongitude: 0.87254; geodeticDatum: WGS84; **Event:** eventID: 2; samplingProtocol: Ground; eventTime: Night
- **Type status:** Other material. **Occurrence:** individualCount: 1; sex: female; **Location:** locationID: A1; continent: Europe; country: Spain; countryCode: ES; stateProvince: Catalonia; county: Lleida; locality: Sola de Boi; verbatimElevation: 1759.8; decimalLatitude: 42.54958; decimalLongitude: 0.87254; geodeticDatum: WGS84; **Event:** eventID: 2; samplingProtocol: Ground; eventTime: Night
- **Type status:** Other material. **Occurrence:** individualCount: 3; sex: male; **Location:** locationID: A1; continent: Europe; country: Spain; countryCode: ES; stateProvince: Catalonia; county: Lleida; locality: Sola de Boi; verbatimElevation: 1759.8; decimalLatitude: 42.54958; decimalLongitude: 0.87254; geodeticDatum: WGS84; **Event:** eventID: A; samplingProtocol: Pitfall
- **Type status:** Other material. **Occurrence:** individualCount: 6; sex: male; **Location:** locationID: A1; continent: Europe; country: Spain; countryCode: ES; stateProvince: Catalonia; county: Lleida; locality: Sola de Boi; verbatimElevation: 1759.8; decimalLatitude: 42.54958; decimalLongitude: 0.87254; geodeticDatum: WGS84; **Event:** eventID: B; samplingProtocol: Pitfall
- **Type status:** Other material. **Occurrence:** individualCount: 8; sex: male; **Location:** locationID: A1; continent: Europe; country: Spain; countryCode: ES; stateProvince: Catalonia; county: Lleida; locality: Sola de Boi; verbatimElevation: 1759.8; decimalLatitude: 42.54958; decimalLongitude: 0.87254; geodeticDatum: WGS84; **Event:** eventID: C; samplingProtocol: Pitfall
- **Type status:** Other material. **Occurrence:** individualCount: 6; sex: female; **Location:** locationID: A1; continent: Europe; country: Spain; countryCode: ES; stateProvince: Catalonia; county: Lleida; locality: Sola de Boi; verbatimElevation: 1759.8; decimalLatitude: 42.54958; decimalLongitude: 0.87254; geodeticDatum: WGS84; **Event:** eventID: C; samplingProtocol: Pitfall
- **Type status:** Other material. **Occurrence:** individualCount: 6; sex: male; **Location:** locationID: A1; continent: Europe; country: Spain; countryCode: ES; stateProvince: Catalonia; county: Lleida; locality: Sola de Boi; verbatimElevation: 1759.8; decimalLatitude: 42.54958; decimalLongitude: 0.87254; geodeticDatum: WGS84; **Event:** eventID: D; samplingProtocol: Pitfall
- **Type status:** Other material. **Occurrence:** individualCount: 7; sex: male; **Location:** locationID: A1; continent: Europe; country: Spain; countryCode: ES; stateProvince: Catalonia; county: Lleida; locality: Sola de Boi; verbatimElevation: 1759.8; decimalLatitude: 42.54958; decimalLongitude: 0.87254; geodeticDatum: WGS84; **Event:** eventID: E; samplingProtocol: Pitfall
- **Type status:** Other material. **Occurrence:** individualCount: 1; sex: female; **Location:** locationID: A1; continent: Europe; country: Spain; countryCode: ES; stateProvince: Catalonia; county: Lleida; locality: Sola de Boi; verbatimElevation: 1759.8; decimalLatitude: 42.54958; decimalLongitude: 0.87254; geodeticDatum: WGS84; **Event:** eventID: E; samplingProtocol: Pitfall
- **Type status:** Other material. **Occurrence:** individualCount: 4; sex: male; **Location:** locationID: A1; continent: Europe; country: Spain; countryCode: ES; stateProvince: Catalonia; county: Lleida; locality: Sola de Boi; verbatimElevation: 1759.8; decimalLatitude: 42.54958; decimalLongitude: 0.87254; geodeticDatum: WGS84; **Event:** eventID: F; samplingProtocol: Pitfall
- **Type status:** Other material. **Occurrence:** individualCount: 6; sex: male; **Location:** locationID: A1; continent: Europe; country: Spain; countryCode: ES; stateProvince: Catalonia; county: Lleida; locality: Sola de Boi; verbatimElevation: 1759.8; decimalLatitude: 42.54958; decimalLongitude: 0.87254; geodeticDatum: WGS84; **Event:** eventID: G; samplingProtocol: Pitfall
- **Type status:** Other material. **Occurrence:** individualCount: 1; sex: female; **Location:** locationID: A1; continent: Europe; country: Spain; countryCode: ES; stateProvince: Catalonia; county: Lleida; locality: Sola de Boi; verbatimElevation: 1759.8; decimalLatitude: 42.54958; decimalLongitude: 0.87254; geodeticDatum: WGS84; **Event:** eventID: G; samplingProtocol: Pitfall
- **Type status:** Other material. **Occurrence:** individualCount: 4; sex: male; **Location:** locationID: A1; continent: Europe; country: Spain; countryCode: ES; stateProvince: Catalonia; county: Lleida; locality: Sola de Boi; verbatimElevation: 1759.8; decimalLatitude: 42.54958; decimalLongitude: 0.87254; geodeticDatum: WGS84; **Event:** eventID: H; samplingProtocol: Pitfall
- **Type status:** Other material. **Occurrence:** individualCount: 2; sex: female; **Location:** locationID: A1; continent: Europe; country: Spain; countryCode: ES; stateProvince: Catalonia; county: Lleida; locality: Sola de Boi; verbatimElevation: 1759.8; decimalLatitude: 42.54958; decimalLongitude: 0.87254; geodeticDatum: WGS84; **Event:** eventID: H; samplingProtocol: Pitfall
- **Type status:** Other material. **Occurrence:** individualCount: 5; sex: male; **Location:** locationID: A1; continent: Europe; country: Spain; countryCode: ES; stateProvince: Catalonia; county: Lleida; locality: Sola de Boi; verbatimElevation: 1759.8; decimalLatitude: 42.54958; decimalLongitude: 0.87254; geodeticDatum: WGS84; **Event:** eventID: I; samplingProtocol: Pitfall
- **Type status:** Other material. **Occurrence:** individualCount: 2; sex: female; **Location:** locationID: A1; continent: Europe; country: Spain; countryCode: ES; stateProvince: Catalonia; county: Lleida; locality: Sola de Boi; verbatimElevation: 1759.8; decimalLatitude: 42.54958; decimalLongitude: 0.87254; geodeticDatum: WGS84; **Event:** eventID: I; samplingProtocol: Pitfall
- **Type status:** Other material. **Occurrence:** individualCount: 3; sex: male; **Location:** locationID: A1; continent: Europe; country: Spain; countryCode: ES; stateProvince: Catalonia; county: Lleida; locality: Sola de Boi; verbatimElevation: 1759.8; decimalLatitude: 42.54958; decimalLongitude: 0.87254; geodeticDatum: WGS84; **Event:** eventID: J; samplingProtocol: Pitfall
- **Type status:** Other material. **Occurrence:** individualCount: 1; sex: female; **Location:** locationID: A1; continent: Europe; country: Spain; countryCode: ES; stateProvince: Catalonia; county: Lleida; locality: Sola de Boi; verbatimElevation: 1759.8; decimalLatitude: 42.54958; decimalLongitude: 0.87254; geodeticDatum: WGS84; **Event:** eventID: J; samplingProtocol: Pitfall
- **Type status:** Other material. **Occurrence:** individualCount: 1; sex: male; **Location:** locationID: A1; continent: Europe; country: Spain; countryCode: ES; stateProvince: Catalonia; county: Lleida; locality: Sola de Boi; verbatimElevation: 1759.8; decimalLatitude: 42.54958; decimalLongitude: 0.87254; geodeticDatum: WGS84; **Event:** eventID: K; samplingProtocol: Pitfall
- **Type status:** Other material. **Occurrence:** individualCount: 1; sex: female; **Location:** locationID: A1; continent: Europe; country: Spain; countryCode: ES; stateProvince: Catalonia; county: Lleida; locality: Sola de Boi; verbatimElevation: 1759.8; decimalLatitude: 42.54958; decimalLongitude: 0.87254; geodeticDatum: WGS84; **Event:** eventID: L; samplingProtocol: Pitfall
- **Type status:** Other material. **Occurrence:** individualCount: 4; sex: male; **Location:** locationID: A2; continent: Europe; country: Spain; countryCode: ES; stateProvince: Catalonia; county: Lleida; locality: Sola de Boi; verbatimElevation: 1738.7; decimalLatitude: 42.54913; decimalLongitude: 0.87137; geodeticDatum: WGS84; **Event:** eventID: A; samplingProtocol: Pitfall
- **Type status:** Other material. **Occurrence:** individualCount: 3; sex: female; **Location:** locationID: A2; continent: Europe; country: Spain; countryCode: ES; stateProvince: Catalonia; county: Lleida; locality: Sola de Boi; verbatimElevation: 1738.7; decimalLatitude: 42.54913; decimalLongitude: 0.87137; geodeticDatum: WGS84; **Event:** eventID: A; samplingProtocol: Pitfall
- **Type status:** Other material. **Occurrence:** individualCount: 5; sex: male; **Location:** locationID: A2; continent: Europe; country: Spain; countryCode: ES; stateProvince: Catalonia; county: Lleida; locality: Sola de Boi; verbatimElevation: 1738.7; decimalLatitude: 42.54913; decimalLongitude: 0.87137; geodeticDatum: WGS84; **Event:** eventID: B; samplingProtocol: Pitfall
- **Type status:** Other material. **Occurrence:** individualCount: 3; sex: male; **Location:** locationID: A2; continent: Europe; country: Spain; countryCode: ES; stateProvince: Catalonia; county: Lleida; locality: Sola de Boi; verbatimElevation: 1738.7; decimalLatitude: 42.54913; decimalLongitude: 0.87137; geodeticDatum: WGS84; **Event:** eventID: C; samplingProtocol: Pitfall
- **Type status:** Other material. **Occurrence:** individualCount: 1; sex: female; **Location:** locationID: A2; continent: Europe; country: Spain; countryCode: ES; stateProvince: Catalonia; county: Lleida; locality: Sola de Boi; verbatimElevation: 1738.7; decimalLatitude: 42.54913; decimalLongitude: 0.87137; geodeticDatum: WGS84; **Event:** eventID: D; samplingProtocol: Pitfall
- **Type status:** Other material. **Occurrence:** individualCount: 5; sex: male; **Location:** locationID: A2; continent: Europe; country: Spain; countryCode: ES; stateProvince: Catalonia; county: Lleida; locality: Sola de Boi; verbatimElevation: 1738.7; decimalLatitude: 42.54913; decimalLongitude: 0.87137; geodeticDatum: WGS84; **Event:** eventID: E; samplingProtocol: Pitfall
- **Type status:** Other material. **Occurrence:** individualCount: 1; sex: female; **Location:** locationID: A2; continent: Europe; country: Spain; countryCode: ES; stateProvince: Catalonia; county: Lleida; locality: Sola de Boi; verbatimElevation: 1738.7; decimalLatitude: 42.54913; decimalLongitude: 0.87137; geodeticDatum: WGS84; **Event:** eventID: E; samplingProtocol: Pitfall
- **Type status:** Other material. **Occurrence:** individualCount: 3; sex: male; **Location:** locationID: A2; continent: Europe; country: Spain; countryCode: ES; stateProvince: Catalonia; county: Lleida; locality: Sola de Boi; verbatimElevation: 1738.7; decimalLatitude: 42.54913; decimalLongitude: 0.87137; geodeticDatum: WGS84; **Event:** eventID: F; samplingProtocol: Pitfall
- **Type status:** Other material. **Occurrence:** individualCount: 2; sex: female; **Location:** locationID: A2; continent: Europe; country: Spain; countryCode: ES; stateProvince: Catalonia; county: Lleida; locality: Sola de Boi; verbatimElevation: 1738.7; decimalLatitude: 42.54913; decimalLongitude: 0.87137; geodeticDatum: WGS84; **Event:** eventID: F; samplingProtocol: Pitfall
- **Type status:** Other material. **Occurrence:** individualCount: 5; sex: male; **Location:** locationID: A2; continent: Europe; country: Spain; countryCode: ES; stateProvince: Catalonia; county: Lleida; locality: Sola de Boi; verbatimElevation: 1738.7; decimalLatitude: 42.54913; decimalLongitude: 0.87137; geodeticDatum: WGS84; **Event:** eventID: G; samplingProtocol: Pitfall
- **Type status:** Other material. **Occurrence:** individualCount: 1; sex: female; **Location:** locationID: A2; continent: Europe; country: Spain; countryCode: ES; stateProvince: Catalonia; county: Lleida; locality: Sola de Boi; verbatimElevation: 1738.7; decimalLatitude: 42.54913; decimalLongitude: 0.87137; geodeticDatum: WGS84; **Event:** eventID: G; samplingProtocol: Pitfall
- **Type status:** Other material. **Occurrence:** individualCount: 9; sex: male; **Location:** locationID: A2; continent: Europe; country: Spain; countryCode: ES; stateProvince: Catalonia; county: Lleida; locality: Sola de Boi; verbatimElevation: 1738.7; decimalLatitude: 42.54913; decimalLongitude: 0.87137; geodeticDatum: WGS84; **Event:** eventID: H; samplingProtocol: Pitfall
- **Type status:** Other material. **Occurrence:** individualCount: 1; sex: female; **Location:** locationID: A2; continent: Europe; country: Spain; countryCode: ES; stateProvince: Catalonia; county: Lleida; locality: Sola de Boi; verbatimElevation: 1738.7; decimalLatitude: 42.54913; decimalLongitude: 0.87137; geodeticDatum: WGS84; **Event:** eventID: H; samplingProtocol: Pitfall
- **Type status:** Other material. **Occurrence:** individualCount: 5; sex: male; **Location:** locationID: A2; continent: Europe; country: Spain; countryCode: ES; stateProvince: Catalonia; county: Lleida; locality: Sola de Boi; verbatimElevation: 1738.7; decimalLatitude: 42.54913; decimalLongitude: 0.87137; geodeticDatum: WGS84; **Event:** eventID: I; samplingProtocol: Pitfall
- **Type status:** Other material. **Occurrence:** individualCount: 1; sex: female; **Location:** locationID: A2; continent: Europe; country: Spain; countryCode: ES; stateProvince: Catalonia; county: Lleida; locality: Sola de Boi; verbatimElevation: 1738.7; decimalLatitude: 42.54913; decimalLongitude: 0.87137; geodeticDatum: WGS84; **Event:** eventID: I; samplingProtocol: Pitfall
- **Type status:** Other material. **Occurrence:** individualCount: 6; sex: male; **Location:** locationID: A2; continent: Europe; country: Spain; countryCode: ES; stateProvince: Catalonia; county: Lleida; locality: Sola de Boi; verbatimElevation: 1738.7; decimalLatitude: 42.54913; decimalLongitude: 0.87137; geodeticDatum: WGS84; **Event:** eventID: J; samplingProtocol: Pitfall
- **Type status:** Other material. **Occurrence:** individualCount: 1; sex: female; **Location:** locationID: A2; continent: Europe; country: Spain; countryCode: ES; stateProvince: Catalonia; county: Lleida; locality: Sola de Boi; verbatimElevation: 1738.7; decimalLatitude: 42.54913; decimalLongitude: 0.87137; geodeticDatum: WGS84; **Event:** eventID: J; samplingProtocol: Pitfall
- **Type status:** Other material. **Occurrence:** individualCount: 6; sex: male; **Location:** locationID: A2; continent: Europe; country: Spain; countryCode: ES; stateProvince: Catalonia; county: Lleida; locality: Sola de Boi; verbatimElevation: 1738.7; decimalLatitude: 42.54913; decimalLongitude: 0.87137; geodeticDatum: WGS84; **Event:** eventID: K; samplingProtocol: Pitfall
- **Type status:** Other material. **Occurrence:** individualCount: 1; sex: female; **Location:** locationID: A2; continent: Europe; country: Spain; countryCode: ES; stateProvince: Catalonia; county: Lleida; locality: Sola de Boi; verbatimElevation: 1738.7; decimalLatitude: 42.54913; decimalLongitude: 0.87137; geodeticDatum: WGS84; **Event:** eventID: K; samplingProtocol: Pitfall
- **Type status:** Other material. **Occurrence:** individualCount: 3; sex: male; **Location:** locationID: A2; continent: Europe; country: Spain; countryCode: ES; stateProvince: Catalonia; county: Lleida; locality: Sola de Boi; verbatimElevation: 1738.7; decimalLatitude: 42.54913; decimalLongitude: 0.87137; geodeticDatum: WGS84; **Event:** eventID: L; samplingProtocol: Pitfall

##### Distribution

Europe to Kazakhstan

#### Leptodrassus femineus

(Simon, 1873)

##### Materials

- **Type status:** Other material. **Occurrence:** individualCount: 1; sex: female; **Location:** locationID: C3; continent: Europe; country: Spain; countryCode: ES; stateProvince: Castilla-La Mancha; county: Ciudad Real; locality: La Quesera; verbatimElevation: 767.55; decimalLatitude: 39.36177; decimalLongitude: -4.41733; geodeticDatum: WGS84; **Event:** eventID: 1; samplingProtocol: Beating; eventTime: Night
- **Type status:** Other material. **Occurrence:** individualCount: 1; sex: female; **Location:** locationID: C3; continent: Europe; country: Spain; countryCode: ES; stateProvince: Castilla-La Mancha; county: Ciudad Real; locality: La Quesera; verbatimElevation: 767.55; decimalLatitude: 39.36177; decimalLongitude: -4.41733; geodeticDatum: WGS84; **Event:** eventID: 1; samplingProtocol: Sweeping; eventTime: Night

##### Distribution

Portugal to Crete, Israel

#### Micaria albovittata

(Lucas, 1846)

##### Materials

- **Type status:** Other material. **Occurrence:** individualCount: 1; sex: male; **Location:** locationID: S1; continent: Europe; country: Spain; countryCode: ES; stateProvince: Andalucía; county: Granada; locality: Soportujar; verbatimElevation: 1786.57; decimalLatitude: 36.96151; decimalLongitude: -3.41881; geodeticDatum: WGS84; **Event:** eventID: D; samplingProtocol: Pitfall
- **Type status:** Other material. **Occurrence:** individualCount: 1; sex: female; **Location:** locationID: S1; continent: Europe; country: Spain; countryCode: ES; stateProvince: Andalucía; county: Granada; locality: Soportujar; verbatimElevation: 1786.57; decimalLatitude: 36.96151; decimalLongitude: -3.41881; geodeticDatum: WGS84; **Event:** eventID: J; samplingProtocol: Pitfall
- **Type status:** Other material. **Occurrence:** individualCount: 2; sex: male; **Location:** locationID: S1; continent: Europe; country: Spain; countryCode: ES; stateProvince: Andalucía; county: Granada; locality: Soportujar; verbatimElevation: 1786.57; decimalLatitude: 36.96151; decimalLongitude: -3.41881; geodeticDatum: WGS84; **Event:** eventID: L; samplingProtocol: Pitfall
- **Type status:** Other material. **Occurrence:** individualCount: 2; sex: female; **Location:** locationID: S2; continent: Europe; country: Spain; countryCode: ES; stateProvince: Andalucía; county: Granada; locality: Camarate; verbatimElevation: 1713.96; decimalLatitude: 37.18377; decimalLongitude: -3.26282; geodeticDatum: WGS84; **Event:** eventID: A; samplingProtocol: Pitfall
- **Type status:** Other material. **Occurrence:** individualCount: 3; sex: male; **Location:** locationID: S2; continent: Europe; country: Spain; countryCode: ES; stateProvince: Andalucía; county: Granada; locality: Camarate; verbatimElevation: 1713.96; decimalLatitude: 37.18377; decimalLongitude: -3.26282; geodeticDatum: WGS84; **Event:** eventID: C; samplingProtocol: Pitfall
- **Type status:** Other material. **Occurrence:** individualCount: 1; sex: female; **Location:** locationID: S2; continent: Europe; country: Spain; countryCode: ES; stateProvince: Andalucía; county: Granada; locality: Camarate; verbatimElevation: 1713.96; decimalLatitude: 37.18377; decimalLongitude: -3.26282; geodeticDatum: WGS84; **Event:** eventID: C; samplingProtocol: Pitfall
- **Type status:** Other material. **Occurrence:** individualCount: 1; sex: male; **Location:** locationID: S2; continent: Europe; country: Spain; countryCode: ES; stateProvince: Andalucía; county: Granada; locality: Camarate; verbatimElevation: 1713.96; decimalLatitude: 37.18377; decimalLongitude: -3.26282; geodeticDatum: WGS84; **Event:** eventID: E; samplingProtocol: Pitfall
- **Type status:** Other material. **Occurrence:** individualCount: 1; sex: female; **Location:** locationID: S2; continent: Europe; country: Spain; countryCode: ES; stateProvince: Andalucía; county: Granada; locality: Camarate; verbatimElevation: 1713.96; decimalLatitude: 37.18377; decimalLongitude: -3.26282; geodeticDatum: WGS84; **Event:** eventID: E; samplingProtocol: Pitfall
- **Type status:** Other material. **Occurrence:** individualCount: 2; sex: male; **Location:** locationID: S2; continent: Europe; country: Spain; countryCode: ES; stateProvince: Andalucía; county: Granada; locality: Camarate; verbatimElevation: 1713.96; decimalLatitude: 37.18377; decimalLongitude: -3.26282; geodeticDatum: WGS84; **Event:** eventID: G; samplingProtocol: Pitfall
- **Type status:** Other material. **Occurrence:** individualCount: 1; sex: female; **Location:** locationID: S2; continent: Europe; country: Spain; countryCode: ES; stateProvince: Andalucía; county: Granada; locality: Camarate; verbatimElevation: 1713.96; decimalLatitude: 37.18377; decimalLongitude: -3.26282; geodeticDatum: WGS84; **Event:** eventID: G; samplingProtocol: Pitfall
- **Type status:** Other material. **Occurrence:** individualCount: 3; sex: male; **Location:** locationID: S2; continent: Europe; country: Spain; countryCode: ES; stateProvince: Andalucía; county: Granada; locality: Camarate; verbatimElevation: 1713.96; decimalLatitude: 37.18377; decimalLongitude: -3.26282; geodeticDatum: WGS84; **Event:** eventID: H; samplingProtocol: Pitfall
- **Type status:** Other material. **Occurrence:** individualCount: 1; sex: male; **Location:** locationID: S2; continent: Europe; country: Spain; countryCode: ES; stateProvince: Andalucía; county: Granada; locality: Camarate; verbatimElevation: 1713.96; decimalLatitude: 37.18377; decimalLongitude: -3.26282; geodeticDatum: WGS84; **Event:** eventID: I; samplingProtocol: Pitfall

##### Distribution

Palearctic

#### Micaria brignolii

(Bosmans & Blick, 2000)

##### Materials

- **Type status:** Other material. **Occurrence:** individualCount: 1; sex: male; **Location:** locationID: C2; continent: Europe; country: Spain; countryCode: ES; stateProvince: Castilla-La Mancha; county: Ciudad Real; locality: Valle Brezoso; verbatimElevation: 739.31; decimalLatitude: 39.35159; decimalLongitude: -4.3589; geodeticDatum: WGS84; **Event:** eventID: K; samplingProtocol: Pitfall

##### Distribution

Iberian Peninsula, France

#### Micaria fulgens

(Walckenaer, 1802)

##### Materials

- **Type status:** Other material. **Occurrence:** individualCount: 1; sex: male; **Location:** locationID: A1; continent: Europe; country: Spain; countryCode: ES; stateProvince: Catalonia; county: Lleida; locality: Sola de Boi; verbatimElevation: 1759.8; decimalLatitude: 42.54958; decimalLongitude: 0.87254; geodeticDatum: WGS84; **Event:** eventID: A; samplingProtocol: Pitfall
- **Type status:** Other material. **Occurrence:** individualCount: 2; sex: male; **Location:** locationID: A1; continent: Europe; country: Spain; countryCode: ES; stateProvince: Catalonia; county: Lleida; locality: Sola de Boi; verbatimElevation: 1759.8; decimalLatitude: 42.54958; decimalLongitude: 0.87254; geodeticDatum: WGS84; **Event:** eventID: B; samplingProtocol: Pitfall
- **Type status:** Other material. **Occurrence:** individualCount: 1; sex: female; **Location:** locationID: A1; continent: Europe; country: Spain; countryCode: ES; stateProvince: Catalonia; county: Lleida; locality: Sola de Boi; verbatimElevation: 1759.8; decimalLatitude: 42.54958; decimalLongitude: 0.87254; geodeticDatum: WGS84; **Event:** eventID: B; samplingProtocol: Pitfall
- **Type status:** Other material. **Occurrence:** individualCount: 12; sex: male; **Location:** locationID: A1; continent: Europe; country: Spain; countryCode: ES; stateProvince: Catalonia; county: Lleida; locality: Sola de Boi; verbatimElevation: 1759.8; decimalLatitude: 42.54958; decimalLongitude: 0.87254; geodeticDatum: WGS84; **Event:** eventID: C; samplingProtocol: Pitfall
- **Type status:** Other material. **Occurrence:** individualCount: 1; sex: female; **Location:** locationID: A1; continent: Europe; country: Spain; countryCode: ES; stateProvince: Catalonia; county: Lleida; locality: Sola de Boi; verbatimElevation: 1759.8; decimalLatitude: 42.54958; decimalLongitude: 0.87254; geodeticDatum: WGS84; **Event:** eventID: C; samplingProtocol: Pitfall
- **Type status:** Other material. **Occurrence:** individualCount: 2; sex: male; **Location:** locationID: A1; continent: Europe; country: Spain; countryCode: ES; stateProvince: Catalonia; county: Lleida; locality: Sola de Boi; verbatimElevation: 1759.8; decimalLatitude: 42.54958; decimalLongitude: 0.87254; geodeticDatum: WGS84; **Event:** eventID: E; samplingProtocol: Pitfall
- **Type status:** Other material. **Occurrence:** individualCount: 2; sex: male; **Location:** locationID: A1; continent: Europe; country: Spain; countryCode: ES; stateProvince: Catalonia; county: Lleida; locality: Sola de Boi; verbatimElevation: 1759.8; decimalLatitude: 42.54958; decimalLongitude: 0.87254; geodeticDatum: WGS84; **Event:** eventID: G; samplingProtocol: Pitfall
- **Type status:** Other material. **Occurrence:** individualCount: 3; sex: male; **Location:** locationID: A1; continent: Europe; country: Spain; countryCode: ES; stateProvince: Catalonia; county: Lleida; locality: Sola de Boi; verbatimElevation: 1759.8; decimalLatitude: 42.54958; decimalLongitude: 0.87254; geodeticDatum: WGS84; **Event:** eventID: H; samplingProtocol: Pitfall
- **Type status:** Other material. **Occurrence:** individualCount: 1; sex: female; **Location:** locationID: A1; continent: Europe; country: Spain; countryCode: ES; stateProvince: Catalonia; county: Lleida; locality: Sola de Boi; verbatimElevation: 1759.8; decimalLatitude: 42.54958; decimalLongitude: 0.87254; geodeticDatum: WGS84; **Event:** eventID: J; samplingProtocol: Pitfall
- **Type status:** Other material. **Occurrence:** individualCount: 1; sex: male; **Location:** locationID: A1; continent: Europe; country: Spain; countryCode: ES; stateProvince: Catalonia; county: Lleida; locality: Sola de Boi; verbatimElevation: 1759.8; decimalLatitude: 42.54958; decimalLongitude: 0.87254; geodeticDatum: WGS84; **Event:** eventID: L; samplingProtocol: Pitfall
- **Type status:** Other material. **Occurrence:** individualCount: 3; sex: female; **Location:** locationID: A1; continent: Europe; country: Spain; countryCode: ES; stateProvince: Catalonia; county: Lleida; locality: Sola de Boi; verbatimElevation: 1759.8; decimalLatitude: 42.54958; decimalLongitude: 0.87254; geodeticDatum: WGS84; **Event:** eventID: L; samplingProtocol: Pitfall
- **Type status:** Other material. **Occurrence:** individualCount: 2; sex: female; **Location:** locationID: A2; continent: Europe; country: Spain; countryCode: ES; stateProvince: Catalonia; county: Lleida; locality: Sola de Boi; verbatimElevation: 1738.7; decimalLatitude: 42.54913; decimalLongitude: 0.87137; geodeticDatum: WGS84; **Event:** eventID: A; samplingProtocol: Pitfall
- **Type status:** Other material. **Occurrence:** individualCount: 2; sex: female; **Location:** locationID: A2; continent: Europe; country: Spain; countryCode: ES; stateProvince: Catalonia; county: Lleida; locality: Sola de Boi; verbatimElevation: 1738.7; decimalLatitude: 42.54913; decimalLongitude: 0.87137; geodeticDatum: WGS84; **Event:** eventID: E; samplingProtocol: Pitfall
- **Type status:** Other material. **Occurrence:** individualCount: 1; sex: female; **Location:** locationID: A2; continent: Europe; country: Spain; countryCode: ES; stateProvince: Catalonia; county: Lleida; locality: Sola de Boi; verbatimElevation: 1738.7; decimalLatitude: 42.54913; decimalLongitude: 0.87137; geodeticDatum: WGS84; **Event:** eventID: G; samplingProtocol: Pitfall
- **Type status:** Other material. **Occurrence:** individualCount: 1; sex: female; **Location:** locationID: A2; continent: Europe; country: Spain; countryCode: ES; stateProvince: Catalonia; county: Lleida; locality: Sola de Boi; verbatimElevation: 1738.7; decimalLatitude: 42.54913; decimalLongitude: 0.87137; geodeticDatum: WGS84; **Event:** eventID: H; samplingProtocol: Pitfall
- **Type status:** Other material. **Occurrence:** individualCount: 3; sex: male; **Location:** locationID: A2; continent: Europe; country: Spain; countryCode: ES; stateProvince: Catalonia; county: Lleida; locality: Sola de Boi; verbatimElevation: 1738.7; decimalLatitude: 42.54913; decimalLongitude: 0.87137; geodeticDatum: WGS84; **Event:** eventID: I; samplingProtocol: Pitfall
- **Type status:** Other material. **Occurrence:** individualCount: 4; sex: female; **Location:** locationID: A2; continent: Europe; country: Spain; countryCode: ES; stateProvince: Catalonia; county: Lleida; locality: Sola de Boi; verbatimElevation: 1738.7; decimalLatitude: 42.54913; decimalLongitude: 0.87137; geodeticDatum: WGS84; **Event:** eventID: I; samplingProtocol: Pitfall
- **Type status:** Other material. **Occurrence:** individualCount: 2; sex: female; **Location:** locationID: A2; continent: Europe; country: Spain; countryCode: ES; stateProvince: Catalonia; county: Lleida; locality: Sola de Boi; verbatimElevation: 1738.7; decimalLatitude: 42.54913; decimalLongitude: 0.87137; geodeticDatum: WGS84; **Event:** eventID: J; samplingProtocol: Pitfall
- **Type status:** Other material. **Occurrence:** individualCount: 1; sex: male; **Location:** locationID: A2; continent: Europe; country: Spain; countryCode: ES; stateProvince: Catalonia; county: Lleida; locality: Sola de Boi; verbatimElevation: 1738.7; decimalLatitude: 42.54913; decimalLongitude: 0.87137; geodeticDatum: WGS84; **Event:** eventID: K; samplingProtocol: Pitfall
- **Type status:** Other material. **Occurrence:** individualCount: 1; sex: female; **Location:** locationID: A2; continent: Europe; country: Spain; countryCode: ES; stateProvince: Catalonia; county: Lleida; locality: Sola de Boi; verbatimElevation: 1738.7; decimalLatitude: 42.54913; decimalLongitude: 0.87137; geodeticDatum: WGS84; **Event:** eventID: K; samplingProtocol: Pitfall
- **Type status:** Other material. **Occurrence:** individualCount: 1; sex: male; **Location:** locationID: P2; continent: Europe; country: Spain; countryCode: ES; stateProvince: Castilla y León; county: León; locality: Joyoguelas; verbatimElevation: 763.98; decimalLatitude: 43.17771; decimalLongitude: -4.90579; geodeticDatum: WGS84; **Event:** eventID: C; samplingProtocol: Pitfall
- **Type status:** Other material. **Occurrence:** individualCount: 1; sex: male; **Location:** locationID: P3; continent: Europe; country: Spain; countryCode: ES; stateProvince: Castilla y León; county: León; locality: Las Arroyas; verbatimElevation: 1097.1; decimalLatitude: 43.14351; decimalLongitude: -4.94878; geodeticDatum: WGS84; **Event:** eventID: A; samplingProtocol: Pitfall
- **Type status:** Other material. **Occurrence:** individualCount: 1; sex: male; **Location:** locationID: P3; continent: Europe; country: Spain; countryCode: ES; stateProvince: Castilla y León; county: León; locality: Las Arroyas; verbatimElevation: 1097.1; decimalLatitude: 43.14351; decimalLongitude: -4.94878; geodeticDatum: WGS84; **Event:** eventID: E; samplingProtocol: Pitfall
- **Type status:** Other material. **Occurrence:** individualCount: 1; sex: female; **Location:** locationID: P4; continent: Europe; country: Spain; countryCode: ES; stateProvince: Castilla y León; county: León; locality: El Canto; verbatimElevation: 943.48; decimalLatitude: 43.17227; decimalLongitude: -4.90857; geodeticDatum: WGS84; **Event:** eventID: B; samplingProtocol: Pitfall
- **Type status:** Other material. **Occurrence:** individualCount: 1; sex: female; **Location:** locationID: P4; continent: Europe; country: Spain; countryCode: ES; stateProvince: Castilla y León; county: León; locality: El Canto; verbatimElevation: 943.48; decimalLatitude: 43.17227; decimalLongitude: -4.90857; geodeticDatum: WGS84; **Event:** eventID: D; samplingProtocol: Pitfall
- **Type status:** Other material. **Occurrence:** individualCount: 2; sex: male; **Location:** locationID: P4; continent: Europe; country: Spain; countryCode: ES; stateProvince: Castilla y León; county: León; locality: El Canto; verbatimElevation: 943.48; decimalLatitude: 43.17227; decimalLongitude: -4.90857; geodeticDatum: WGS84; **Event:** eventID: E; samplingProtocol: Pitfall
- **Type status:** Other material. **Occurrence:** individualCount: 4; sex: male; **Location:** locationID: P4; continent: Europe; country: Spain; countryCode: ES; stateProvince: Castilla y León; county: León; locality: El Canto; verbatimElevation: 943.48; decimalLatitude: 43.17227; decimalLongitude: -4.90857; geodeticDatum: WGS84; **Event:** eventID: G; samplingProtocol: Pitfall
- **Type status:** Other material. **Occurrence:** individualCount: 10; sex: male; **Location:** locationID: P4; continent: Europe; country: Spain; countryCode: ES; stateProvince: Castilla y León; county: León; locality: El Canto; verbatimElevation: 943.48; decimalLatitude: 43.17227; decimalLongitude: -4.90857; geodeticDatum: WGS84; **Event:** eventID: H; samplingProtocol: Pitfall
- **Type status:** Other material. **Occurrence:** individualCount: 1; sex: female; **Location:** locationID: P4; continent: Europe; country: Spain; countryCode: ES; stateProvince: Castilla y León; county: León; locality: El Canto; verbatimElevation: 943.48; decimalLatitude: 43.17227; decimalLongitude: -4.90857; geodeticDatum: WGS84; **Event:** eventID: H; samplingProtocol: Pitfall
- **Type status:** Other material. **Occurrence:** individualCount: 1; sex: female; **Location:** locationID: P4; continent: Europe; country: Spain; countryCode: ES; stateProvince: Castilla y León; county: León; locality: El Canto; verbatimElevation: 943.48; decimalLatitude: 43.17227; decimalLongitude: -4.90857; geodeticDatum: WGS84; **Event:** eventID: L; samplingProtocol: Pitfall

##### Distribution

Palearctic

#### Micaria guttigera

Simon, 1878

##### Materials

- **Type status:** Other material. **Occurrence:** individualCount: 1; sex: female; **Location:** locationID: C2; continent: Europe; country: Spain; countryCode: ES; stateProvince: Castilla-La Mancha; county: Ciudad Real; locality: Valle Brezoso; verbatimElevation: 739.31; decimalLatitude: 39.35159; decimalLongitude: -4.3589; geodeticDatum: WGS84; **Event:** eventID: L; samplingProtocol: Pitfall
- **Type status:** Other material. **Occurrence:** individualCount: 1; sex: male; **Location:** locationID: C3; continent: Europe; country: Spain; countryCode: ES; stateProvince: Castilla-La Mancha; county: Ciudad Real; locality: La Quesera; verbatimElevation: 767.55; decimalLatitude: 39.36177; decimalLongitude: -4.41733; geodeticDatum: WGS84; **Event:** eventID: I; samplingProtocol: Pitfall
- **Type status:** Other material. **Occurrence:** individualCount: 4; sex: male; **Location:** locationID: C4; continent: Europe; country: Spain; countryCode: ES; stateProvince: Castilla-La Mancha; county: Ciudad Real; locality: La Quesera; verbatimElevation: 772.3; decimalLatitude: 39.36337; decimalLongitude: -4.41704; geodeticDatum: WGS84; **Event:** eventID: A; samplingProtocol: Pitfall
- **Type status:** Other material. **Occurrence:** individualCount: 1; sex: female; **Location:** locationID: C4; continent: Europe; country: Spain; countryCode: ES; stateProvince: Castilla-La Mancha; county: Ciudad Real; locality: La Quesera; verbatimElevation: 772.3; decimalLatitude: 39.36337; decimalLongitude: -4.41704; geodeticDatum: WGS84; **Event:** eventID: A; samplingProtocol: Pitfall
- **Type status:** Other material. **Occurrence:** individualCount: 1; sex: female; **Location:** locationID: C4; continent: Europe; country: Spain; countryCode: ES; stateProvince: Castilla-La Mancha; county: Ciudad Real; locality: La Quesera; verbatimElevation: 772.3; decimalLatitude: 39.36337; decimalLongitude: -4.41704; geodeticDatum: WGS84; **Event:** eventID: B; samplingProtocol: Pitfall
- **Type status:** Other material. **Occurrence:** individualCount: 1; sex: male; **Location:** locationID: C4; continent: Europe; country: Spain; countryCode: ES; stateProvince: Castilla-La Mancha; county: Ciudad Real; locality: La Quesera; verbatimElevation: 772.3; decimalLatitude: 39.36337; decimalLongitude: -4.41704; geodeticDatum: WGS84; **Event:** eventID: B; samplingProtocol: Pitfall
- **Type status:** Other material. **Occurrence:** individualCount: 1; sex: male; **Location:** locationID: C4; continent: Europe; country: Spain; countryCode: ES; stateProvince: Castilla-La Mancha; county: Ciudad Real; locality: La Quesera; verbatimElevation: 772.3; decimalLatitude: 39.36337; decimalLongitude: -4.41704; geodeticDatum: WGS84; **Event:** eventID: C; samplingProtocol: Pitfall
- **Type status:** Other material. **Occurrence:** individualCount: 1; sex: male; **Location:** locationID: C4; continent: Europe; country: Spain; countryCode: ES; stateProvince: Castilla-La Mancha; county: Ciudad Real; locality: La Quesera; verbatimElevation: 772.3; decimalLatitude: 39.36337; decimalLongitude: -4.41704; geodeticDatum: WGS84; **Event:** eventID: C; samplingProtocol: Pitfall
- **Type status:** Other material. **Occurrence:** individualCount: 1; sex: female; **Location:** locationID: C4; continent: Europe; country: Spain; countryCode: ES; stateProvince: Castilla-La Mancha; county: Ciudad Real; locality: La Quesera; verbatimElevation: 772.3; decimalLatitude: 39.36337; decimalLongitude: -4.41704; geodeticDatum: WGS84; **Event:** eventID: F; samplingProtocol: Pitfall
- **Type status:** Other material. **Occurrence:** individualCount: 1; sex: female; **Location:** locationID: S1; continent: Europe; country: Spain; countryCode: ES; stateProvince: Andalucía; county: Granada; locality: Soportujar; verbatimElevation: 1786.57; decimalLatitude: 36.96151; decimalLongitude: -3.41881; geodeticDatum: WGS84; **Event:** eventID: J; samplingProtocol: Pitfall
- **Type status:** Other material. **Occurrence:** individualCount: 1; sex: male; **Location:** locationID: S1; continent: Europe; country: Spain; countryCode: ES; stateProvince: Andalucía; county: Granada; locality: Soportujar; verbatimElevation: 1786.57; decimalLatitude: 36.96151; decimalLongitude: -3.41881; geodeticDatum: WGS84; **Event:** eventID: D; samplingProtocol: Pitfall
- **Type status:** Other material. **Occurrence:** individualCount: 4; sex: female; **Location:** locationID: S2; continent: Europe; country: Spain; countryCode: ES; stateProvince: Andalucía; county: Granada; locality: Camarate; verbatimElevation: 1713.96; decimalLatitude: 37.18377; decimalLongitude: -3.26282; geodeticDatum: WGS84; **Event:** eventID: A; samplingProtocol: Pitfall
- **Type status:** Other material. **Occurrence:** individualCount: 1; sex: male; **Location:** locationID: S2; continent: Europe; country: Spain; countryCode: ES; stateProvince: Andalucía; county: Granada; locality: Camarate; verbatimElevation: 1713.96; decimalLatitude: 37.18377; decimalLongitude: -3.26282; geodeticDatum: WGS84; **Event:** eventID: A; samplingProtocol: Pitfall
- **Type status:** Other material. **Occurrence:** individualCount: 2; sex: male; **Location:** locationID: S2; continent: Europe; country: Spain; countryCode: ES; stateProvince: Andalucía; county: Granada; locality: Camarate; verbatimElevation: 1713.96; decimalLatitude: 37.18377; decimalLongitude: -3.26282; geodeticDatum: WGS84; **Event:** eventID: B; samplingProtocol: Pitfall
- **Type status:** Other material. **Occurrence:** individualCount: 3; sex: female; **Location:** locationID: S2; continent: Europe; country: Spain; countryCode: ES; stateProvince: Andalucía; county: Granada; locality: Camarate; verbatimElevation: 1713.96; decimalLatitude: 37.18377; decimalLongitude: -3.26282; geodeticDatum: WGS84; **Event:** eventID: C; samplingProtocol: Pitfall
- **Type status:** Other material. **Occurrence:** individualCount: 1; sex: male; **Location:** locationID: S2; continent: Europe; country: Spain; countryCode: ES; stateProvince: Andalucía; county: Granada; locality: Camarate; verbatimElevation: 1713.96; decimalLatitude: 37.18377; decimalLongitude: -3.26282; geodeticDatum: WGS84; **Event:** eventID: C; samplingProtocol: Pitfall
- **Type status:** Other material. **Occurrence:** individualCount: 2; sex: female; **Location:** locationID: S2; continent: Europe; country: Spain; countryCode: ES; stateProvince: Andalucía; county: Granada; locality: Camarate; verbatimElevation: 1713.96; decimalLatitude: 37.18377; decimalLongitude: -3.26282; geodeticDatum: WGS84; **Event:** eventID: D; samplingProtocol: Pitfall
- **Type status:** Other material. **Occurrence:** individualCount: 1; sex: male; **Location:** locationID: S2; continent: Europe; country: Spain; countryCode: ES; stateProvince: Andalucía; county: Granada; locality: Camarate; verbatimElevation: 1713.96; decimalLatitude: 37.18377; decimalLongitude: -3.26282; geodeticDatum: WGS84; **Event:** eventID: G; samplingProtocol: Pitfall
- **Type status:** Other material. **Occurrence:** individualCount: 1; sex: male; **Location:** locationID: S2; continent: Europe; country: Spain; countryCode: ES; stateProvince: Andalucía; county: Granada; locality: Camarate; verbatimElevation: 1713.96; decimalLatitude: 37.18377; decimalLongitude: -3.26282; geodeticDatum: WGS84; **Event:** eventID: H; samplingProtocol: Pitfall
- **Type status:** Other material. **Occurrence:** individualCount: 3; sex: female; **Location:** locationID: S2; continent: Europe; country: Spain; countryCode: ES; stateProvince: Andalucía; county: Granada; locality: Camarate; verbatimElevation: 1713.96; decimalLatitude: 37.18377; decimalLongitude: -3.26282; geodeticDatum: WGS84; **Event:** eventID: H; samplingProtocol: Pitfall
- **Type status:** Other material. **Occurrence:** individualCount: 2; sex: male; **Location:** locationID: S2; continent: Europe; country: Spain; countryCode: ES; stateProvince: Andalucía; county: Granada; locality: Camarate; verbatimElevation: 1713.96; decimalLatitude: 37.18377; decimalLongitude: -3.26282; geodeticDatum: WGS84; **Event:** eventID: I; samplingProtocol: Pitfall
- **Type status:** Other material. **Occurrence:** individualCount: 2; sex: female; **Location:** locationID: S2; continent: Europe; country: Spain; countryCode: ES; stateProvince: Andalucía; county: Granada; locality: Camarate; verbatimElevation: 1713.96; decimalLatitude: 37.18377; decimalLongitude: -3.26282; geodeticDatum: WGS84; **Event:** eventID: I; samplingProtocol: Pitfall
- **Type status:** Other material. **Occurrence:** individualCount: 1; sex: female; **Location:** locationID: S2; continent: Europe; country: Spain; countryCode: ES; stateProvince: Andalucía; county: Granada; locality: Camarate; verbatimElevation: 1713.96; decimalLatitude: 37.18377; decimalLongitude: -3.26282; geodeticDatum: WGS84; **Event:** eventID: L; samplingProtocol: Pitfall

##### Distribution

Iberian Peninsula, France

#### Nomisia celerrima

(Simon, 1914)

##### Materials

- **Type status:** Other material. **Occurrence:** individualCount: 1; sex: female; **Location:** locationID: A2; continent: Europe; country: Spain; countryCode: ES; stateProvince: Catalonia; county: Lleida; locality: Sola de Boi; verbatimElevation: 1738.7; decimalLatitude: 42.54913; decimalLongitude: 0.87137; geodeticDatum: WGS84; **Event:** eventID: 1; samplingProtocol: Aerial; eventTime: Night
- **Type status:** Other material. **Occurrence:** individualCount: 1; sex: male; **Location:** locationID: C3; continent: Europe; country: Spain; countryCode: ES; stateProvince: Castilla-La Mancha; county: Ciudad Real; locality: La Quesera; verbatimElevation: 767.55; decimalLatitude: 39.36177; decimalLongitude: -4.41733; geodeticDatum: WGS84; **Event:** eventID: H; samplingProtocol: Pitfall

##### Distribution

Spain, France

##### Notes

Uncertain identification, given the similarities between the species *N. excerpta* (O. Pickard-Cambridge, 1872) and *N. celerrima*.

#### Nomisia sp29

##### Materials

- **Type status:** Other material. **Occurrence:** individualCount: 3; sex: male; **Location:** locationID: S1; continent: Europe; country: Spain; countryCode: ES; stateProvince: Andalucía; county: Granada; locality: Soportujar; verbatimElevation: 1786.57; decimalLatitude: 36.96151; decimalLongitude: -3.41881; geodeticDatum: WGS84; **Event:** eventID: I; samplingProtocol: Pitfall
- **Type status:** Other material. **Occurrence:** individualCount: 4; sex: male; **Location:** locationID: S1; continent: Europe; country: Spain; countryCode: ES; stateProvince: Andalucía; county: Granada; locality: Soportujar; verbatimElevation: 1786.57; decimalLatitude: 36.96151; decimalLongitude: -3.41881; geodeticDatum: WGS84; **Event:** eventID: K; samplingProtocol: Pitfall
- **Type status:** Other material. **Occurrence:** individualCount: 6; sex: male; **Location:** locationID: S1; continent: Europe; country: Spain; countryCode: ES; stateProvince: Andalucía; county: Granada; locality: Soportujar; verbatimElevation: 1786.57; decimalLatitude: 36.96151; decimalLongitude: -3.41881; geodeticDatum: WGS84; **Event:** eventID: L; samplingProtocol: Pitfall
- **Type status:** Other material. **Occurrence:** individualCount: 1; sex: female; **Location:** locationID: S1; continent: Europe; country: Spain; countryCode: ES; stateProvince: Andalucía; county: Granada; locality: Soportujar; verbatimElevation: 1786.57; decimalLatitude: 36.96151; decimalLongitude: -3.41881; geodeticDatum: WGS84; **Event:** eventID: L; samplingProtocol: Pitfall

##### Distribution

?

##### Notes

This is a new species of *Nomisia* Dalmas, 1921, to be described in a future publication.

#### Poecilochroa albomaculata

(Lucas, 1846)

##### Materials

- **Type status:** Other material. **Occurrence:** individualCount: 1; sex: male; **Location:** locationID: C1; continent: Europe; country: Spain; countryCode: ES; stateProvince: Castilla-La Mancha; county: Ciudad Real; locality: Valle Brezoso; verbatimElevation: 756.56; decimalLatitude: 39.35663; decimalLongitude: -4.35912; geodeticDatum: WGS84; **Event:** eventID: I; samplingProtocol: Pitfall
- **Type status:** Other material. **Occurrence:** individualCount: 1; sex: male; **Location:** locationID: C2; continent: Europe; country: Spain; countryCode: ES; stateProvince: Castilla-La Mancha; county: Ciudad Real; locality: Valle Brezoso; verbatimElevation: 739.31; decimalLatitude: 39.35159; decimalLongitude: -4.3589; geodeticDatum: WGS84; **Event:** eventID: F; samplingProtocol: Pitfall

##### Distribution

Western Mediterranean

#### Poecilochroa variana

(C. L. Koch, 1839)

##### Materials

- **Type status:** Other material. **Occurrence:** individualCount: 1; sex: male; **Location:** locationID: O1; continent: Europe; country: Spain; countryCode: ES; stateProvince: Aragón; county: Huesca; locality: O Furno; verbatimElevation: 1396.73; decimalLatitude: 42.60677; decimalLongitude: 0.13135; geodeticDatum: WGS84; **Event:** eventID: D; samplingProtocol: Pitfall

##### Distribution

Europe to Central Asia

#### Pterotricha simoni

Dalmas, 1921

##### Materials

- **Type status:** Other material. **Occurrence:** individualCount: 2; sex: male; **Location:** locationID: S1; continent: Europe; country: Spain; countryCode: ES; stateProvince: Andalucía; county: Granada; locality: Soportujar; verbatimElevation: 1786.57; decimalLatitude: 36.96151; decimalLongitude: -3.41881; geodeticDatum: WGS84; **Event:** eventID: D; samplingProtocol: Pitfall
- **Type status:** Other material. **Occurrence:** individualCount: 2; sex: male; **Location:** locationID: S1; continent: Europe; country: Spain; countryCode: ES; stateProvince: Andalucía; county: Granada; locality: Soportujar; verbatimElevation: 1786.57; decimalLatitude: 36.96151; decimalLongitude: -3.41881; geodeticDatum: WGS84; **Event:** eventID: K; samplingProtocol: Pitfall
- **Type status:** Other material. **Occurrence:** individualCount: 1; sex: female; **Location:** locationID: S1; continent: Europe; country: Spain; countryCode: ES; stateProvince: Andalucía; county: Granada; locality: Soportujar; verbatimElevation: 1786.57; decimalLatitude: 36.96151; decimalLongitude: -3.41881; geodeticDatum: WGS84; **Event:** eventID: K; samplingProtocol: Pitfall
- **Type status:** Other material. **Occurrence:** individualCount: 1; sex: male; **Location:** locationID: S1; continent: Europe; country: Spain; countryCode: ES; stateProvince: Andalucía; county: Granada; locality: Soportujar; verbatimElevation: 1786.57; decimalLatitude: 36.96151; decimalLongitude: -3.41881; geodeticDatum: WGS84; **Event:** eventID: L; samplingProtocol: Pitfall

##### Distribution

Iberian Peninsula

#### Scotophaeus cf. blackwalli

(Thorell, 1871)

##### Materials

- **Type status:** Other material. **Occurrence:** individualCount: 1; sex: male; **Location:** locationID: C2; continent: Europe; country: Spain; countryCode: ES; stateProvince: Castilla-La Mancha; county: Ciudad Real; locality: Valle Brezoso; verbatimElevation: 739.31; decimalLatitude: 39.35159; decimalLongitude: -4.3589; geodeticDatum: WGS84; **Event:** eventID: F; samplingProtocol: Pitfall
- **Type status:** Other material. **Occurrence:** individualCount: 1; sex: female; **Location:** locationID: C3; continent: Europe; country: Spain; countryCode: ES; stateProvince: Castilla-La Mancha; county: Ciudad Real; locality: La Quesera; verbatimElevation: 767.55; decimalLatitude: 39.36177; decimalLongitude: -4.41733; geodeticDatum: WGS84; **Event:** eventID: 1; samplingProtocol: Beating; eventTime: Day
- **Type status:** Other material. **Occurrence:** individualCount: 1; sex: male; **Location:** locationID: C4; continent: Europe; country: Spain; countryCode: ES; stateProvince: Castilla-La Mancha; county: Ciudad Real; locality: La Quesera; verbatimElevation: 772.3; decimalLatitude: 39.36337; decimalLongitude: -4.41704; geodeticDatum: WGS84; **Event:** eventID: 2; samplingProtocol: Beating; eventTime: Night
- **Type status:** Other material. **Occurrence:** individualCount: 1; sex: male; **Location:** locationID: M1; continent: Europe; country: Spain; countryCode: ES; stateProvince: Extremadura; county: Cáceres; locality: Peña Falcón; verbatimElevation: 320.6; decimalLatitude: 39.83296; decimalLongitude: -6.0641; geodeticDatum: WGS84; **Event:** eventID: 1; samplingProtocol: Aerial; eventTime: Night
- **Type status:** Other material. **Occurrence:** individualCount: 1; sex: male; **Location:** locationID: S1; continent: Europe; country: Spain; countryCode: ES; stateProvince: Andalucía; county: Granada; locality: Soportujar; verbatimElevation: 1786.57; decimalLatitude: 36.96151; decimalLongitude: -3.41881; geodeticDatum: WGS84; **Event:** eventID: 4; samplingProtocol: Aerial; eventTime: Night
- **Type status:** Other material. **Occurrence:** individualCount: 1; sex: male; **Location:** locationID: S2; continent: Europe; country: Spain; countryCode: ES; stateProvince: Andalucía; county: Granada; locality: Camarate; verbatimElevation: 1713.96; decimalLatitude: 37.18377; decimalLongitude: -3.26282; geodeticDatum: WGS84; **Event:** eventID: 1; samplingProtocol: Beating; eventTime: Day

##### Distribution

Cosmopolitan

##### Notes

see *Species delimitation and identification using DNA barcodes*.

#### Scotophaeus cf. validus

(Lucas, 1846)

##### Materials

- **Type status:** Other material. **Occurrence:** individualCount: 1; sex: female; **Location:** locationID: S2; continent: Europe; country: Spain; countryCode: ES; stateProvince: Andalucía; county: Granada; locality: Camarate; verbatimElevation: 1713.96; decimalLatitude: 37.18377; decimalLongitude: -3.26282; geodeticDatum: WGS84; **Event:** eventID: 4; samplingProtocol: Aerial; eventTime: Night

##### Distribution

Southern Europe, Morocco, Algeria

##### Notes

see *Species delimitation and identification using DNA barcodes*.

#### Setaphis carmeli

(O. Pickard-Cambridge, 1872)

##### Materials

- **Type status:** Other material. **Occurrence:** individualCount: 1; sex: female; **Location:** locationID: C2; continent: Europe; country: Spain; countryCode: ES; stateProvince: Castilla-La Mancha; county: Ciudad Real; locality: Valle Brezoso; verbatimElevation: 739.31; decimalLatitude: 39.35159; decimalLongitude: -4.3589; geodeticDatum: WGS84; **Event:** eventID: C; samplingProtocol: Pitfall
- **Type status:** Other material. **Occurrence:** individualCount: 3; sex: female; **Location:** locationID: C3; continent: Europe; country: Spain; countryCode: ES; stateProvince: Castilla-La Mancha; county: Ciudad Real; locality: La Quesera; verbatimElevation: 767.55; decimalLatitude: 39.36177; decimalLongitude: -4.41733; geodeticDatum: WGS84; **Event:** eventID: A; samplingProtocol: Pitfall
- **Type status:** Other material. **Occurrence:** individualCount: 1; sex: female; **Location:** locationID: C4; continent: Europe; country: Spain; countryCode: ES; stateProvince: Castilla-La Mancha; county: Ciudad Real; locality: La Quesera; verbatimElevation: 772.3; decimalLatitude: 39.36337; decimalLongitude: -4.41704; geodeticDatum: WGS84; **Event:** eventID: C; samplingProtocol: Pitfall
- **Type status:** Other material. **Occurrence:** individualCount: 1; sex: male; **Location:** locationID: C4; continent: Europe; country: Spain; countryCode: ES; stateProvince: Castilla-La Mancha; county: Ciudad Real; locality: La Quesera; verbatimElevation: 772.3; decimalLatitude: 39.36337; decimalLongitude: -4.41704; geodeticDatum: WGS84; **Event:** eventID: K; samplingProtocol: Pitfall

##### Distribution

Mediterranean

#### Trachyzelotes mutabilis

(Simon, 1878)

##### Materials

- **Type status:** Other material. **Occurrence:** individualCount: 1; sex: male; **Location:** locationID: C3; continent: Europe; country: Spain; countryCode: ES; stateProvince: Castilla-La Mancha; county: Ciudad Real; locality: La Quesera; verbatimElevation: 767.55; decimalLatitude: 39.36177; decimalLongitude: -4.41733; geodeticDatum: WGS84; **Event:** eventID: L; samplingProtocol: Pitfall
- **Type status:** Other material. **Occurrence:** individualCount: 1; sex: male; **Location:** locationID: M1; continent: Europe; country: Spain; countryCode: ES; stateProvince: Extremadura; county: Cáceres; locality: Peña Falcón; verbatimElevation: 320.6; decimalLatitude: 39.83296; decimalLongitude: -6.0641; geodeticDatum: WGS84; **Event:** eventID: F; samplingProtocol: Pitfall
- **Type status:** Other material. **Occurrence:** individualCount: 1; sex: male; **Location:** locationID: S1; continent: Europe; country: Spain; countryCode: ES; stateProvince: Andalucía; county: Granada; locality: Soportujar; verbatimElevation: 1786.57; decimalLatitude: 36.96151; decimalLongitude: -3.41881; geodeticDatum: WGS84; **Event:** eventID: E; samplingProtocol: Pitfall
- **Type status:** Other material. **Occurrence:** individualCount: 1; sex: male; **Location:** locationID: S1; continent: Europe; country: Spain; countryCode: ES; stateProvince: Andalucía; county: Granada; locality: Soportujar; verbatimElevation: 1786.57; decimalLatitude: 36.96151; decimalLongitude: -3.41881; geodeticDatum: WGS84; **Event:** eventID: H; samplingProtocol: Pitfall

##### Distribution

Mediterranean

#### Trachyzelotes pedestris

(C. L. Koch, 1937)

##### Materials

- **Type status:** Other material. **Occurrence:** individualCount: 2; sex: male; **Location:** locationID: P2; continent: Europe; country: Spain; countryCode: ES; stateProvince: Castilla y León; county: León; locality: Joyoguelas; verbatimElevation: 763.98; decimalLatitude: 43.17771; decimalLongitude: -4.90579; geodeticDatum: WGS84; **Event:** eventID: C; samplingProtocol: Pitfall
- **Type status:** Other material. **Occurrence:** individualCount: 2; sex: male; **Location:** locationID: P4; continent: Europe; country: Spain; countryCode: ES; stateProvince: Castilla y León; county: León; locality: El Canto; verbatimElevation: 943.48; decimalLatitude: 43.17227; decimalLongitude: -4.90857; geodeticDatum: WGS84; **Event:** eventID: A; samplingProtocol: Pitfall

##### Distribution

Europe to Iran

#### Zelotes flagellans

(L. Koch, 1882)

##### Materials

- **Type status:** Other material. **Occurrence:** individualCount: 1; sex: female; **Location:** locationID: S1; continent: Europe; country: Spain; countryCode: ES; stateProvince: Andalucía; county: Granada; locality: Soportujar; verbatimElevation: 1786.57; decimalLatitude: 36.96151; decimalLongitude: -3.41881; geodeticDatum: WGS84; **Event:** eventID: D; samplingProtocol: Pitfall
- **Type status:** Other material. **Occurrence:** individualCount: 1; sex: male; **Location:** locationID: S1; continent: Europe; country: Spain; countryCode: ES; stateProvince: Andalucía; county: Granada; locality: Soportujar; verbatimElevation: 1786.57; decimalLatitude: 36.96151; decimalLongitude: -3.41881; geodeticDatum: WGS84; **Event:** eventID: E; samplingProtocol: Pitfall
- **Type status:** Other material. **Occurrence:** individualCount: 1; sex: male; **Location:** locationID: S1; continent: Europe; country: Spain; countryCode: ES; stateProvince: Andalucía; county: Granada; locality: Soportujar; verbatimElevation: 1786.57; decimalLatitude: 36.96151; decimalLongitude: -3.41881; geodeticDatum: WGS84; **Event:** eventID: F; samplingProtocol: Pitfall
- **Type status:** Other material. **Occurrence:** individualCount: 2; sex: female; **Location:** locationID: S1; continent: Europe; country: Spain; countryCode: ES; stateProvince: Andalucía; county: Granada; locality: Soportujar; verbatimElevation: 1786.57; decimalLatitude: 36.96151; decimalLongitude: -3.41881; geodeticDatum: WGS84; **Event:** eventID: F; samplingProtocol: Pitfall
- **Type status:** Other material. **Occurrence:** individualCount: 2; sex: male; **Location:** locationID: S1; continent: Europe; country: Spain; countryCode: ES; stateProvince: Andalucía; county: Granada; locality: Soportujar; verbatimElevation: 1786.57; decimalLatitude: 36.96151; decimalLongitude: -3.41881; geodeticDatum: WGS84; **Event:** eventID: H; samplingProtocol: Pitfall
- **Type status:** Other material. **Occurrence:** individualCount: 1; sex: male; **Location:** locationID: S1; continent: Europe; country: Spain; countryCode: ES; stateProvince: Andalucía; county: Granada; locality: Soportujar; verbatimElevation: 1786.57; decimalLatitude: 36.96151; decimalLongitude: -3.41881; geodeticDatum: WGS84; **Event:** eventID: K; samplingProtocol: Pitfall

##### Distribution

Spain, Balearic Islands

#### Zelotes gallicus

Simon, 1914

##### Materials

- **Type status:** Other material. **Occurrence:** individualCount: 1; sex: female; **Location:** locationID: A1; continent: Europe; country: Spain; countryCode: ES; stateProvince: Catalonia; county: Lleida; locality: Sola de Boi; verbatimElevation: 1759.8; decimalLatitude: 42.54958; decimalLongitude: 0.87254; geodeticDatum: WGS84; **Event:** eventID: C; samplingProtocol: Pitfall
- **Type status:** Other material. **Occurrence:** individualCount: 1; sex: female; **Location:** locationID: A1; continent: Europe; country: Spain; countryCode: ES; stateProvince: Catalonia; county: Lleida; locality: Sola de Boi; verbatimElevation: 1759.8; decimalLatitude: 42.54958; decimalLongitude: 0.87254; geodeticDatum: WGS84; **Event:** eventID: F; samplingProtocol: Pitfall
- **Type status:** Other material. **Occurrence:** individualCount: 1; sex: male; **Location:** locationID: A2; continent: Europe; country: Spain; countryCode: ES; stateProvince: Catalonia; county: Lleida; locality: Sola de Boi; verbatimElevation: 1738.7; decimalLatitude: 42.54913; decimalLongitude: 0.87137; geodeticDatum: WGS84; **Event:** eventID: I; samplingProtocol: Pitfall
- **Type status:** Other material. **Occurrence:** individualCount: 1; sex: male; **Location:** locationID: O1; continent: Europe; country: Spain; countryCode: ES; stateProvince: Aragón; county: Huesca; locality: O Furno; verbatimElevation: 1396.73; decimalLatitude: 42.60677; decimalLongitude: 0.13135; geodeticDatum: WGS84; **Event:** eventID: F; samplingProtocol: Pitfall
- **Type status:** Other material. **Occurrence:** individualCount: 1; sex: male; **Location:** locationID: O2; continent: Europe; country: Spain; countryCode: ES; stateProvince: Aragón; county: Huesca; locality: Rebilla; verbatimElevation: 1158.13; decimalLatitude: 42.59427; decimalLongitude: 0.1529; geodeticDatum: WGS84; **Event:** eventID: A; samplingProtocol: Pitfall
- **Type status:** Other material. **Occurrence:** individualCount: 1; sex: male; **Location:** locationID: P2; continent: Europe; country: Spain; countryCode: ES; stateProvince: Castilla y León; county: León; locality: Joyoguelas; verbatimElevation: 763.98; decimalLatitude: 43.17771; decimalLongitude: -4.90579; geodeticDatum: WGS84; **Event:** eventID: A; samplingProtocol: Pitfall
- **Type status:** Other material. **Occurrence:** individualCount: 1; sex: male; **Location:** locationID: P2; continent: Europe; country: Spain; countryCode: ES; stateProvince: Castilla y León; county: León; locality: Joyoguelas; verbatimElevation: 763.98; decimalLatitude: 43.17771; decimalLongitude: -4.90579; geodeticDatum: WGS84; **Event:** eventID: D; samplingProtocol: Pitfall
- **Type status:** Other material. **Occurrence:** individualCount: 2; sex: male; **Location:** locationID: P2; continent: Europe; country: Spain; countryCode: ES; stateProvince: Castilla y León; county: León; locality: Joyoguelas; verbatimElevation: 763.98; decimalLatitude: 43.17771; decimalLongitude: -4.90579; geodeticDatum: WGS84; **Event:** eventID: E; samplingProtocol: Pitfall
- **Type status:** Other material. **Occurrence:** individualCount: 3; sex: male; **Location:** locationID: P4; continent: Europe; country: Spain; countryCode: ES; stateProvince: Castilla y León; county: León; locality: El Canto; verbatimElevation: 943.48; decimalLatitude: 43.17227; decimalLongitude: -4.90857; geodeticDatum: WGS84; **Event:** eventID: A; samplingProtocol: Pitfall
- **Type status:** Other material. **Occurrence:** individualCount: 3; sex: female; **Location:** locationID: P4; continent: Europe; country: Spain; countryCode: ES; stateProvince: Castilla y León; county: León; locality: El Canto; verbatimElevation: 943.48; decimalLatitude: 43.17227; decimalLongitude: -4.90857; geodeticDatum: WGS84; **Event:** eventID: A; samplingProtocol: Pitfall
- **Type status:** Other material. **Occurrence:** individualCount: 1; sex: female; **Location:** locationID: P4; continent: Europe; country: Spain; countryCode: ES; stateProvince: Castilla y León; county: León; locality: El Canto; verbatimElevation: 943.48; decimalLatitude: 43.17227; decimalLongitude: -4.90857; geodeticDatum: WGS84; **Event:** eventID: C; samplingProtocol: Pitfall
- **Type status:** Other material. **Occurrence:** individualCount: 2; sex: male; **Location:** locationID: P4; continent: Europe; country: Spain; countryCode: ES; stateProvince: Castilla y León; county: León; locality: El Canto; verbatimElevation: 943.48; decimalLatitude: 43.17227; decimalLongitude: -4.90857; geodeticDatum: WGS84; **Event:** eventID: E; samplingProtocol: Pitfall
- **Type status:** Other material. **Occurrence:** individualCount: 1; sex: male; **Location:** locationID: P4; continent: Europe; country: Spain; countryCode: ES; stateProvince: Castilla y León; county: León; locality: El Canto; verbatimElevation: 943.48; decimalLatitude: 43.17227; decimalLongitude: -4.90857; geodeticDatum: WGS84; **Event:** eventID: G; samplingProtocol: Pitfall
- **Type status:** Other material. **Occurrence:** individualCount: 2; sex: female; **Location:** locationID: P4; continent: Europe; country: Spain; countryCode: ES; stateProvince: Castilla y León; county: León; locality: El Canto; verbatimElevation: 943.48; decimalLatitude: 43.17227; decimalLongitude: -4.90857; geodeticDatum: WGS84; **Event:** eventID: G; samplingProtocol: Pitfall
- **Type status:** Other material. **Occurrence:** individualCount: 3; sex: male; **Location:** locationID: P4; continent: Europe; country: Spain; countryCode: ES; stateProvince: Castilla y León; county: León; locality: El Canto; verbatimElevation: 943.48; decimalLatitude: 43.17227; decimalLongitude: -4.90857; geodeticDatum: WGS84; **Event:** eventID: H; samplingProtocol: Pitfall

##### Distribution

Europe, Russia, Kazakhstan

#### Zelotes manius

(Simon, 1878)

##### Materials

- **Type status:** Other material. **Occurrence:** individualCount: 1; sex: female; **Location:** locationID: A1; continent: Europe; country: Spain; countryCode: ES; stateProvince: Catalonia; county: Lleida; locality: Sola de Boi; verbatimElevation: 1759.8; decimalLatitude: 42.54958; decimalLongitude: 0.87254; geodeticDatum: WGS84; **Event:** eventID: 1; samplingProtocol: Ground; eventTime: Night
- **Type status:** Other material. **Occurrence:** individualCount: 1; sex: female; **Location:** locationID: A1; continent: Europe; country: Spain; countryCode: ES; stateProvince: Catalonia; county: Lleida; locality: Sola de Boi; verbatimElevation: 1759.8; decimalLatitude: 42.54958; decimalLongitude: 0.87254; geodeticDatum: WGS84; **Event:** eventID: E; samplingProtocol: Pitfall
- **Type status:** Other material. **Occurrence:** individualCount: 1; sex: female; **Location:** locationID: A1; continent: Europe; country: Spain; countryCode: ES; stateProvince: Catalonia; county: Lleida; locality: Sola de Boi; verbatimElevation: 1759.8; decimalLatitude: 42.54958; decimalLongitude: 0.87254; geodeticDatum: WGS84; **Event:** eventID: G; samplingProtocol: Pitfall
- **Type status:** Other material. **Occurrence:** individualCount: 1; sex: female; **Location:** locationID: O2; continent: Europe; country: Spain; countryCode: ES; stateProvince: Aragón; county: Huesca; locality: Rebilla; verbatimElevation: 1158.13; decimalLatitude: 42.59427; decimalLongitude: 0.1529; geodeticDatum: WGS84; **Event:** eventID: L; samplingProtocol: Pitfall

##### Distribution

Southern Europe

#### Zelotes tenuis

(L. Koch, 1866)

##### Materials

- **Type status:** Other material. **Occurrence:** individualCount: 1; sex: male; **Location:** locationID: C1; continent: Europe; country: Spain; countryCode: ES; stateProvince: Castilla-La Mancha; county: Ciudad Real; locality: Valle Brezoso; verbatimElevation: 756.56; decimalLatitude: 39.35663; decimalLongitude: -4.35912; geodeticDatum: WGS84; **Event:** eventID: B; samplingProtocol: Pitfall
- **Type status:** Other material. **Occurrence:** individualCount: 1; sex: male; **Location:** locationID: C1; continent: Europe; country: Spain; countryCode: ES; stateProvince: Castilla-La Mancha; county: Ciudad Real; locality: Valle Brezoso; verbatimElevation: 756.56; decimalLatitude: 39.35663; decimalLongitude: -4.35912; geodeticDatum: WGS84; **Event:** eventID: L; samplingProtocol: Pitfall
- **Type status:** Other material. **Occurrence:** individualCount: 1; sex: male; **Location:** locationID: C2; continent: Europe; country: Spain; countryCode: ES; stateProvince: Castilla-La Mancha; county: Ciudad Real; locality: Valle Brezoso; verbatimElevation: 739.31; decimalLatitude: 39.35159; decimalLongitude: -4.3589; geodeticDatum: WGS84; **Event:** eventID: A; samplingProtocol: Pitfall
- **Type status:** Other material. **Occurrence:** individualCount: 1; sex: male; **Location:** locationID: C2; continent: Europe; country: Spain; countryCode: ES; stateProvince: Castilla-La Mancha; county: Ciudad Real; locality: Valle Brezoso; verbatimElevation: 739.31; decimalLatitude: 39.35159; decimalLongitude: -4.3589; geodeticDatum: WGS84; **Event:** eventID: B; samplingProtocol: Pitfall
- **Type status:** Other material. **Occurrence:** individualCount: 1; sex: male; **Location:** locationID: C3; continent: Europe; country: Spain; countryCode: ES; stateProvince: Castilla-La Mancha; county: Ciudad Real; locality: La Quesera; verbatimElevation: 767.55; decimalLatitude: 39.36177; decimalLongitude: -4.41733; geodeticDatum: WGS84; **Event:** eventID: B; samplingProtocol: Pitfall
- **Type status:** Other material. **Occurrence:** individualCount: 3; sex: male; **Location:** locationID: C3; continent: Europe; country: Spain; countryCode: ES; stateProvince: Castilla-La Mancha; county: Ciudad Real; locality: La Quesera; verbatimElevation: 767.55; decimalLatitude: 39.36177; decimalLongitude: -4.41733; geodeticDatum: WGS84; **Event:** eventID: D; samplingProtocol: Pitfall
- **Type status:** Other material. **Occurrence:** individualCount: 1; sex: male; **Location:** locationID: C3; continent: Europe; country: Spain; countryCode: ES; stateProvince: Castilla-La Mancha; county: Ciudad Real; locality: La Quesera; verbatimElevation: 767.55; decimalLatitude: 39.36177; decimalLongitude: -4.41733; geodeticDatum: WGS84; **Event:** eventID: L; samplingProtocol: Pitfall
- **Type status:** Other material. **Occurrence:** individualCount: 2; sex: male; **Location:** locationID: C4; continent: Europe; country: Spain; countryCode: ES; stateProvince: Castilla-La Mancha; county: Ciudad Real; locality: La Quesera; verbatimElevation: 772.3; decimalLatitude: 39.36337; decimalLongitude: -4.41704; geodeticDatum: WGS84; **Event:** eventID: B; samplingProtocol: Pitfall
- **Type status:** Other material. **Occurrence:** individualCount: 2; sex: male; **Location:** locationID: C4; continent: Europe; country: Spain; countryCode: ES; stateProvince: Castilla-La Mancha; county: Ciudad Real; locality: La Quesera; verbatimElevation: 772.3; decimalLatitude: 39.36337; decimalLongitude: -4.41704; geodeticDatum: WGS84; **Event:** eventID: C; samplingProtocol: Pitfall
- **Type status:** Other material. **Occurrence:** individualCount: 1; sex: male; **Location:** locationID: C4; continent: Europe; country: Spain; countryCode: ES; stateProvince: Castilla-La Mancha; county: Ciudad Real; locality: La Quesera; verbatimElevation: 772.3; decimalLatitude: 39.36337; decimalLongitude: -4.41704; geodeticDatum: WGS84; **Event:** eventID: E; samplingProtocol: Pitfall
- **Type status:** Other material. **Occurrence:** individualCount: 1; sex: male; **Location:** locationID: C4; continent: Europe; country: Spain; countryCode: ES; stateProvince: Castilla-La Mancha; county: Ciudad Real; locality: La Quesera; verbatimElevation: 772.3; decimalLatitude: 39.36337; decimalLongitude: -4.41704; geodeticDatum: WGS84; **Event:** eventID: I; samplingProtocol: Pitfall
- **Type status:** Other material. **Occurrence:** individualCount: 1; sex: male; **Location:** locationID: C4; continent: Europe; country: Spain; countryCode: ES; stateProvince: Castilla-La Mancha; county: Ciudad Real; locality: La Quesera; verbatimElevation: 772.3; decimalLatitude: 39.36337; decimalLongitude: -4.41704; geodeticDatum: WGS84; **Event:** eventID: J; samplingProtocol: Pitfall

##### Distribution

Mediterranean to Ukraine, USA

#### Zelotes thorelli

Simon, 1914

##### Materials

- **Type status:** Other material. **Occurrence:** individualCount: 1; sex: female; **Location:** locationID: C1; continent: Europe; country: Spain; countryCode: ES; stateProvince: Castilla-La Mancha; county: Ciudad Real; locality: Valle Brezoso; verbatimElevation: 756.56; decimalLatitude: 39.35663; decimalLongitude: -4.35912; geodeticDatum: WGS84; **Event:** eventID: J; samplingProtocol: Pitfall
- **Type status:** Other material. **Occurrence:** individualCount: 1; sex: female; **Location:** locationID: C3; continent: Europe; country: Spain; countryCode: ES; stateProvince: Castilla-La Mancha; county: Ciudad Real; locality: La Quesera; verbatimElevation: 767.55; decimalLatitude: 39.36177; decimalLongitude: -4.41733; geodeticDatum: WGS84; **Event:** eventID: C; samplingProtocol: Pitfall
- **Type status:** Other material. **Occurrence:** individualCount: 1; sex: female; **Location:** locationID: C4; continent: Europe; country: Spain; countryCode: ES; stateProvince: Castilla-La Mancha; county: Ciudad Real; locality: La Quesera; verbatimElevation: 772.3; decimalLatitude: 39.36337; decimalLongitude: -4.41704; geodeticDatum: WGS84; **Event:** eventID: F; samplingProtocol: Pitfall
- **Type status:** Other material. **Occurrence:** individualCount: 1; sex: female; **Location:** locationID: C4; continent: Europe; country: Spain; countryCode: ES; stateProvince: Castilla-La Mancha; county: Ciudad Real; locality: La Quesera; verbatimElevation: 772.3; decimalLatitude: 39.36337; decimalLongitude: -4.41704; geodeticDatum: WGS84; **Event:** eventID: H; samplingProtocol: Pitfall
- **Type status:** Other material. **Occurrence:** individualCount: 1; sex: female; **Location:** locationID: O2; continent: Europe; country: Spain; countryCode: ES; stateProvince: Aragón; county: Huesca; locality: Rebilla; verbatimElevation: 1158.13; decimalLatitude: 42.59427; decimalLongitude: 0.1529; geodeticDatum: WGS84; **Event:** eventID: 2; samplingProtocol: Aerial; eventTime: Night
- **Type status:** Other material. **Occurrence:** individualCount: 2; sex: female; **Location:** locationID: S1; continent: Europe; country: Spain; countryCode: ES; stateProvince: Andalucía; county: Granada; locality: Soportujar; verbatimElevation: 1786.57; decimalLatitude: 36.96151; decimalLongitude: -3.41881; geodeticDatum: WGS84; **Event:** eventID: L; samplingProtocol: Pitfall
- **Type status:** Other material. **Occurrence:** individualCount: 2; sex: female; **Location:** locationID: S2; continent: Europe; country: Spain; countryCode: ES; stateProvince: Andalucía; county: Granada; locality: Camarate; verbatimElevation: 1713.96; decimalLatitude: 37.18377; decimalLongitude: -3.26282; geodeticDatum: WGS84; **Event:** eventID: A; samplingProtocol: Pitfall

##### Distribution

Southern Europe

#### Hahniidae

Bertkau, 1878

##### Materials

- **Type status:** Other material. **Occurrence:** individualCount: 1; sex: female; **Location:** locationID: P1; continent: Europe; country: Spain; countryCode: ES; stateProvince: Castilla y León; county: León; locality: Monte Robledo; verbatimElevation: 1071.58; decimalLatitude: 43.1445; decimalLongitude: -4.92675; geodeticDatum: WGS84; **Event:** eventID: 2; samplingProtocol: Ground; eventTime: Day
- **Type status:** Other material. **Occurrence:** individualCount: 1; sex: female; **Location:** locationID: P1; continent: Europe; country: Spain; countryCode: ES; stateProvince: Castilla y León; county: León; locality: Monte Robledo; verbatimElevation: 1071.58; decimalLatitude: 43.1445; decimalLongitude: -4.92675; geodeticDatum: WGS84; **Event:** eventID: E; samplingProtocol: Pitfall

#### Hahnia helveola

Simon, 1875

##### Distribution

Italy

#### Hahnia nava

(Blackwall, 1841)

##### Materials

- **Type status:** Other material. **Occurrence:** individualCount: 1; sex: male; **Location:** locationID: O1; continent: Europe; country: Spain; countryCode: ES; stateProvince: Aragón; county: Huesca; locality: O Furno; verbatimElevation: 1396.73; decimalLatitude: 42.60677; decimalLongitude: 0.13135; geodeticDatum: WGS84; **Event:** eventID: C; samplingProtocol: Pitfall
- **Type status:** Other material. **Occurrence:** individualCount: 1; sex: male; **Location:** locationID: O2; continent: Europe; country: Spain; countryCode: ES; stateProvince: Aragón; county: Huesca; locality: Rebilla; verbatimElevation: 1158.13; decimalLatitude: 42.59427; decimalLongitude: 0.1529; geodeticDatum: WGS84; **Event:** eventID: A; samplingProtocol: Pitfall
- **Type status:** Other material. **Occurrence:** individualCount: 1; sex: male; **Location:** locationID: O2; continent: Europe; country: Spain; countryCode: ES; stateProvince: Aragón; county: Huesca; locality: Rebilla; verbatimElevation: 1158.13; decimalLatitude: 42.59427; decimalLongitude: 0.1529; geodeticDatum: WGS84; **Event:** eventID: B; samplingProtocol: Pitfall
- **Type status:** Other material. **Occurrence:** individualCount: 2; sex: male; **Location:** locationID: O2; continent: Europe; country: Spain; countryCode: ES; stateProvince: Aragón; county: Huesca; locality: Rebilla; verbatimElevation: 1158.13; decimalLatitude: 42.59427; decimalLongitude: 0.1529; geodeticDatum: WGS84; **Event:** eventID: K; samplingProtocol: Pitfall

##### Distribution

Palearctic

#### Hahnia ononidum

Simon, 1875

##### Materials

- **Type status:** Other material. **Occurrence:** individualCount: 1; sex: female; **Location:** locationID: A1; continent: Europe; country: Spain; countryCode: ES; stateProvince: Catalonia; county: Lleida; locality: Sola de Boi; verbatimElevation: 1759.8; decimalLatitude: 42.54958; decimalLongitude: 0.87254; geodeticDatum: WGS84; **Event:** eventID: B; samplingProtocol: Pitfall
- **Type status:** Other material. **Occurrence:** individualCount: 1; sex: female; **Location:** locationID: A1; continent: Europe; country: Spain; countryCode: ES; stateProvince: Catalonia; county: Lleida; locality: Sola de Boi; verbatimElevation: 1759.8; decimalLatitude: 42.54958; decimalLongitude: 0.87254; geodeticDatum: WGS84; **Event:** eventID: C; samplingProtocol: Pitfall
- **Type status:** Other material. **Occurrence:** individualCount: 1; sex: female; **Location:** locationID: A1; continent: Europe; country: Spain; countryCode: ES; stateProvince: Catalonia; county: Lleida; locality: Sola de Boi; verbatimElevation: 1759.8; decimalLatitude: 42.54958; decimalLongitude: 0.87254; geodeticDatum: WGS84; **Event:** eventID: G; samplingProtocol: Pitfall
- **Type status:** Other material. **Occurrence:** individualCount: 4; sex: male; **Location:** locationID: P4; continent: Europe; country: Spain; countryCode: ES; stateProvince: Castilla y León; county: León; locality: El Canto; verbatimElevation: 943.48; decimalLatitude: 43.17227; decimalLongitude: -4.90857; geodeticDatum: WGS84; **Event:** eventID: A; samplingProtocol: Pitfall
- **Type status:** Other material. **Occurrence:** individualCount: 1; sex: male; **Location:** locationID: P4; continent: Europe; country: Spain; countryCode: ES; stateProvince: Castilla y León; county: León; locality: El Canto; verbatimElevation: 943.48; decimalLatitude: 43.17227; decimalLongitude: -4.90857; geodeticDatum: WGS84; **Event:** eventID: E; samplingProtocol: Pitfall
- **Type status:** Other material. **Occurrence:** individualCount: 1; sex: female; **Location:** locationID: P4; continent: Europe; country: Spain; countryCode: ES; stateProvince: Castilla y León; county: León; locality: El Canto; verbatimElevation: 943.48; decimalLatitude: 43.17227; decimalLongitude: -4.90857; geodeticDatum: WGS84; **Event:** eventID: G; samplingProtocol: Pitfall
- **Type status:** Other material. **Occurrence:** individualCount: 1; sex: male; **Location:** locationID: P4; continent: Europe; country: Spain; countryCode: ES; stateProvince: Castilla y León; county: León; locality: El Canto; verbatimElevation: 943.48; decimalLatitude: 43.17227; decimalLongitude: -4.90857; geodeticDatum: WGS84; **Event:** eventID: L; samplingProtocol: Pitfall

##### Distribution

USA, Canada, Europe, Russia, Turkey, Kazakhstan

#### Hahnia petrobia

Simon, 1875

##### Materials

- **Type status:** Other material. **Occurrence:** individualCount: 1; sex: female; **Location:** locationID: A1; continent: Europe; country: Spain; countryCode: ES; stateProvince: Catalonia; county: Lleida; locality: Sola de Boi; verbatimElevation: 1759.8; decimalLatitude: 42.54958; decimalLongitude: 0.87254; geodeticDatum: WGS84; **Event:** eventID: B; samplingProtocol: Pitfall

##### Distribution

Europe

#### Iberina montana

(Blackwall, 1841)

##### Materials

- **Type status:** Other material. **Occurrence:** individualCount: 1; sex: female; **Location:** locationID: O1; continent: Europe; country: Spain; countryCode: ES; stateProvince: Aragón; county: Huesca; locality: O Furno; verbatimElevation: 1396.73; decimalLatitude: 42.60677; decimalLongitude: 0.13135; geodeticDatum: WGS84; **Event:** eventID: I; samplingProtocol: Pitfall

##### Distribution

Europe, Turkey, Russia

#### Hersiliidae

Thorell, 1870

#### Tama edwardsi

(Lucas, 1846)

##### Materials

- **Type status:** Other material. **Occurrence:** individualCount: 1; sex: male; **Location:** locationID: M1; continent: Europe; country: Spain; countryCode: ES; stateProvince: Extremadura; county: Cáceres; locality: Peña Falcón; verbatimElevation: 320.6; decimalLatitude: 39.83296; decimalLongitude: -6.0641; geodeticDatum: WGS84; **Event:** eventID: G; samplingProtocol: Pitfall
- **Type status:** Other material. **Occurrence:** individualCount: 1; sex: female; **Location:** locationID: M1; continent: Europe; country: Spain; countryCode: ES; stateProvince: Extremadura; county: Cáceres; locality: Peña Falcón; verbatimElevation: 320.6; decimalLatitude: 39.83296; decimalLongitude: -6.0641; geodeticDatum: WGS84; **Event:** eventID: G; samplingProtocol: Pitfall

##### Distribution

Iberian Peninsula, Algeria

#### Leptonetidae

Simon, 1890

#### Leptoneta paroculus

Simon, 1907

##### Materials

- **Type status:** Other material. **Occurrence:** individualCount: 1; sex: male; **Location:** locationID: A1; continent: Europe; country: Spain; countryCode: ES; stateProvince: Catalonia; county: Lleida; locality: Sola de Boi; verbatimElevation: 1759.8; decimalLatitude: 42.54958; decimalLongitude: 0.87254; geodeticDatum: WGS84; **Event:** eventID: I; samplingProtocol: Pitfall
- **Type status:** Other material. **Occurrence:** individualCount: 1; sex: female; **Location:** locationID: O1; continent: Europe; country: Spain; countryCode: ES; stateProvince: Aragón; county: Huesca; locality: O Furno; verbatimElevation: 1396.73; decimalLatitude: 42.60677; decimalLongitude: 0.13135; geodeticDatum: WGS84; **Event:** eventID: I; samplingProtocol: Pitfall
- **Type status:** Other material. **Occurrence:** individualCount: 1; sex: male; **Location:** locationID: O1; continent: Europe; country: Spain; countryCode: ES; stateProvince: Aragón; county: Huesca; locality: O Furno; verbatimElevation: 1396.73; decimalLatitude: 42.60677; decimalLongitude: 0.13135; geodeticDatum: WGS84; **Event:** eventID: K; samplingProtocol: Pitfall

##### Distribution

Spain

#### Linyphiidae

Blackwall, 1859

#### Agyneta fuscipalpa

(C. L. Koch, 1836)

##### Materials

- **Type status:** Other material. **Occurrence:** individualCount: 1; sex: male; **Location:** locationID: M1; continent: Europe; country: Spain; countryCode: ES; stateProvince: Extremadura; county: Cáceres; locality: Peña Falcón; verbatimElevation: 320.6; decimalLatitude: 39.83296; decimalLongitude: -6.0641; geodeticDatum: WGS84; **Event:** eventID: 2; samplingProtocol: Beating; eventTime: Night
- **Type status:** Other material. **Occurrence:** individualCount: 1; sex: female; **Location:** locationID: P4; continent: Europe; country: Spain; countryCode: ES; stateProvince: Castilla y León; county: León; locality: El Canto; verbatimElevation: 943.48; decimalLatitude: 43.17227; decimalLongitude: -4.90857; geodeticDatum: WGS84; **Event:** eventID: 1; samplingProtocol: Beating; eventTime: Day
- **Type status:** Other material. **Occurrence:** individualCount: 1; sex: male; **Location:** locationID: S1; continent: Europe; country: Spain; countryCode: ES; stateProvince: Andalucía; county: Granada; locality: Soportujar; verbatimElevation: 1786.57; decimalLatitude: 36.96151; decimalLongitude: -3.41881; geodeticDatum: WGS84; **Event:** eventID: 2; samplingProtocol: Beating; eventTime: Day
- **Type status:** Other material. **Occurrence:** individualCount: 1; sex: male; **Location:** locationID: S1; continent: Europe; country: Spain; countryCode: ES; stateProvince: Andalucía; county: Granada; locality: Soportujar; verbatimElevation: 1786.57; decimalLatitude: 36.96151; decimalLongitude: -3.41881; geodeticDatum: WGS84; **Event:** eventID: F; samplingProtocol: Pitfall
- **Type status:** Other material. **Occurrence:** individualCount: 1; sex: male; **Location:** locationID: S2; continent: Europe; country: Spain; countryCode: ES; stateProvince: Andalucía; county: Granada; locality: Camarate; verbatimElevation: 1713.96; decimalLatitude: 37.18377; decimalLongitude: -3.26282; geodeticDatum: WGS84; **Event:** eventID: 1; samplingProtocol: Beating; eventTime: Day
- **Type status:** Other material. **Occurrence:** individualCount: 1; sex: male; **Location:** locationID: S2; continent: Europe; country: Spain; countryCode: ES; stateProvince: Andalucía; county: Granada; locality: Camarate; verbatimElevation: 1713.96; decimalLatitude: 37.18377; decimalLongitude: -3.26282; geodeticDatum: WGS84; **Event:** eventID: B; samplingProtocol: Pitfall
- **Type status:** Other material. **Occurrence:** individualCount: 2; sex: female; **Location:** locationID: S2; continent: Europe; country: Spain; countryCode: ES; stateProvince: Andalucía; county: Granada; locality: Camarate; verbatimElevation: 1713.96; decimalLatitude: 37.18377; decimalLongitude: -3.26282; geodeticDatum: WGS84; **Event:** eventID: 2; samplingProtocol: Beating; eventTime: Day

##### Distribution

Palearctic

##### Notes

We failed to find diagnostic characters to distinguish, through morphology, females of *A. fuscipalpa*, *A. pseudorurestris* Wunderlich, 1980 and *A. rurestris* (C. L. Koch, 1833). Females were assigned to *A. fuscipalpa* based on DNA barcoding. See *Species delimitation and identification using DNA barcodes*.

#### Agyneta orites

(Thorell, 1875)

##### Materials

- **Type status:** Other material. **Occurrence:** individualCount: 1; sex: male; **Location:** locationID: P2; continent: Europe; country: Spain; countryCode: ES; stateProvince: Castilla y León; county: León; locality: Joyoguelas; verbatimElevation: 763.98; decimalLatitude: 43.17771; decimalLongitude: -4.90579; geodeticDatum: WGS84; **Event:** eventID: H; samplingProtocol: Pitfall

##### Distribution

Central Europe

##### Notes

**Figure 6.**
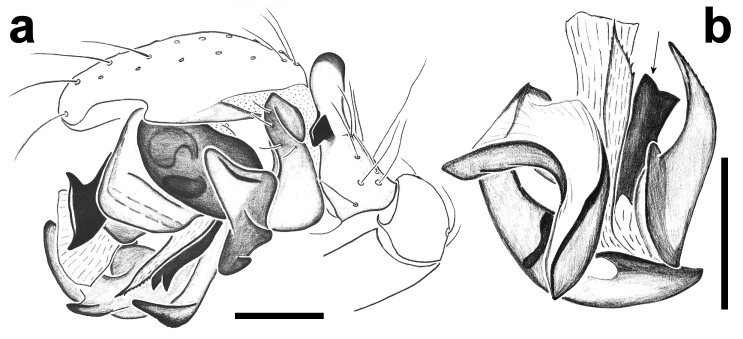
*Agyneta orites* (Thorell, 1875). a: left male palp, retrolateral, scale = 0.2 mm; b: left embolic division, ventral, scale = 0.2 mm.

First record for the Iberian Peninsula. See Fig. 6. The single male specimen collected presents slight differences to both the illustrations made by Wunderlich (Wunderlich 1973, which revised Thorell's type materials) or those by Thaler (Thaler 1983), namely, the tibial apophysis, which seems more robust and the shape of the lamella characteristica, especially the central sclerotised branch (arrow in Fig. 6b), which is bifid in our specimen, unlike the lanceolate shape depicted by Thaler. The similarities, however, outline the differences, as the small finger-like protuberance in the retrolateral branch of the lamella is present and the tibia is also dorsally protuberant, along with a well-developed retrolateral tibial apophysis. A recent citation of this species was done from France by Oger & Miquet (Oger and Miquet 2017), who show a very similar male specimen to ours, with a robust terminal apophysis. See *Species delimitation and identification using DNA barcodes*.

#### Agyneta pseudorurestris

Wunderlich, 1980

##### Materials

- **Type status:** Other material. **Occurrence:** individualCount: 1; sex: male; **Location:** locationID: C1; continent: Europe; country: Spain; countryCode: ES; stateProvince: Castilla-La Mancha; county: Ciudad Real; locality: Valle Brezoso; verbatimElevation: 756.56; decimalLatitude: 39.35663; decimalLongitude: -4.35912; geodeticDatum: WGS84; **Event:** eventID: 1; samplingProtocol: Beating; eventTime: Night
- **Type status:** Other material. **Occurrence:** individualCount: 1; sex: male; **Location:** locationID: M1; continent: Europe; country: Spain; countryCode: ES; stateProvince: Extremadura; county: Cáceres; locality: Peña Falcón; verbatimElevation: 320.6; decimalLatitude: 39.83296; decimalLongitude: -6.0641; geodeticDatum: WGS84; **Event:** eventID: E; samplingProtocol: Pitfall
- **Type status:** Other material. **Occurrence:** individualCount: 1; sex: female; **Location:** locationID: M2; continent: Europe; country: Spain; countryCode: ES; stateProvince: Extremadura; county: Cáceres; locality: Fuente del Frances; verbatimElevation: 320.72; decimalLatitude: 39.828; decimalLongitude: -6.03249; geodeticDatum: WGS84; **Event:** eventID: 1; samplingProtocol: Sweeping; eventTime: Day
- **Type status:** Other material. **Occurrence:** individualCount: 1; sex: male; **Location:** locationID: S2; continent: Europe; country: Spain; countryCode: ES; stateProvince: Andalucía; county: Granada; locality: Camarate; verbatimElevation: 1713.96; decimalLatitude: 37.18377; decimalLongitude: -3.26282; geodeticDatum: WGS84; **Event:** eventID: 1; samplingProtocol: Beating; eventTime: Day

##### Distribution

Iberian Peninsula, Sardinia, Crete, Cyprus, Algeria, Tunisia, Israel

##### Notes

Females of *A. fuscipalpa*, *A. pseudorurestris* and *A. rurestris* (C. L. Koch, 1833) could not be differentiated through morphology. Moreover, DNA identification could not distinguish *A. pseudorurestris* from *A. rurestris* (see *Species delimitation and identification using DNA barcodes*). Consequently, females were assigned to each species based on the geographical location of identified males. In the case of Cabañeros, where males of the two species were found, females were arbitrarily assigned to *A. rurestris*.

#### Agyneta rurestris

(C. L. Koch, 1836)

##### Materials

- **Type status:** Other material. **Occurrence:** individualCount: 1; sex: female; **Location:** locationID: A2; continent: Europe; country: Spain; countryCode: ES; stateProvince: Catalonia; county: Lleida; locality: Sola de Boi; verbatimElevation: 1738.7; decimalLatitude: 42.54913; decimalLongitude: 0.87137; geodeticDatum: WGS84; **Event:** eventID: 1; samplingProtocol: Beating; eventTime: Night
- **Type status:** Other material. **Occurrence:** individualCount: 1; sex: female; **Location:** locationID: A2; continent: Europe; country: Spain; countryCode: ES; stateProvince: Catalonia; county: Lleida; locality: Sola de Boi; verbatimElevation: 1738.7; decimalLatitude: 42.54913; decimalLongitude: 0.87137; geodeticDatum: WGS84; **Event:** eventID: B; samplingProtocol: Pitfall
- **Type status:** Other material. **Occurrence:** individualCount: 1; sex: female; **Location:** locationID: C1; continent: Europe; country: Spain; countryCode: ES; stateProvince: Castilla-La Mancha; county: Ciudad Real; locality: Valle Brezoso; verbatimElevation: 756.56; decimalLatitude: 39.35663; decimalLongitude: -4.35912; geodeticDatum: WGS84; **Event:** eventID: K; samplingProtocol: Pitfall
- **Type status:** Other material. **Occurrence:** individualCount: 1; sex: male; **Location:** locationID: C1; continent: Europe; country: Spain; countryCode: ES; stateProvince: Castilla-La Mancha; county: Ciudad Real; locality: Valle Brezoso; verbatimElevation: 756.56; decimalLatitude: 39.35663; decimalLongitude: -4.35912; geodeticDatum: WGS84; **Event:** eventID: K; samplingProtocol: Pitfall
- **Type status:** Other material. **Occurrence:** individualCount: 1; sex: female; **Location:** locationID: C2; continent: Europe; country: Spain; countryCode: ES; stateProvince: Castilla-La Mancha; county: Ciudad Real; locality: Valle Brezoso; verbatimElevation: 739.31; decimalLatitude: 39.35159; decimalLongitude: -4.3589; geodeticDatum: WGS84; **Event:** eventID: H; samplingProtocol: Pitfall
- **Type status:** Other material. **Occurrence:** individualCount: 1; sex: female; **Location:** locationID: C4; continent: Europe; country: Spain; countryCode: ES; stateProvince: Castilla-La Mancha; county: Ciudad Real; locality: La Quesera; verbatimElevation: 772.3; decimalLatitude: 39.36337; decimalLongitude: -4.41704; geodeticDatum: WGS84; **Event:** eventID: J; samplingProtocol: Pitfall
- **Type status:** Other material. **Occurrence:** individualCount: 1; sex: female; **Location:** locationID: O1; continent: Europe; country: Spain; countryCode: ES; stateProvince: Aragón; county: Huesca; locality: O Furno; verbatimElevation: 1396.73; decimalLatitude: 42.60677; decimalLongitude: 0.13135; geodeticDatum: WGS84; **Event:** eventID: 1; samplingProtocol: Beating; eventTime: Night
- **Type status:** Other material. **Occurrence:** individualCount: 1; sex: female; **Location:** locationID: O2; continent: Europe; country: Spain; countryCode: ES; stateProvince: Aragón; county: Huesca; locality: Rebilla; verbatimElevation: 1158.13; decimalLatitude: 42.59427; decimalLongitude: 0.1529; geodeticDatum: WGS84; **Event:** eventID: K; samplingProtocol: Pitfall
- **Type status:** Other material. **Occurrence:** individualCount: 1; sex: male; **Location:** locationID: O2; continent: Europe; country: Spain; countryCode: ES; stateProvince: Aragón; county: Huesca; locality: Rebilla; verbatimElevation: 1158.13; decimalLatitude: 42.59427; decimalLongitude: 0.1529; geodeticDatum: WGS84; **Event:** eventID: K; samplingProtocol: Pitfall
- **Type status:** Other material. **Occurrence:** individualCount: 1; sex: male; **Location:** locationID: P1; continent: Europe; country: Spain; countryCode: ES; stateProvince: Castilla y León; county: León; locality: Monte Robledo; verbatimElevation: 1071.58; decimalLatitude: 43.1445; decimalLongitude: -4.92675; geodeticDatum: WGS84; **Event:** eventID: 1; samplingProtocol: Sweeping; eventTime: Night
- **Type status:** Other material. **Occurrence:** individualCount: 1; sex: female; **Location:** locationID: P2; continent: Europe; country: Spain; countryCode: ES; stateProvince: Castilla y León; county: León; locality: Joyoguelas; verbatimElevation: 763.98; decimalLatitude: 43.17771; decimalLongitude: -4.90579; geodeticDatum: WGS84; **Event:** eventID: 1; samplingProtocol: Beating; eventTime: Day
- **Type status:** Other material. **Occurrence:** individualCount: 1; sex: female; **Location:** locationID: P2; continent: Europe; country: Spain; countryCode: ES; stateProvince: Castilla y León; county: León; locality: Joyoguelas; verbatimElevation: 763.98; decimalLatitude: 43.17771; decimalLongitude: -4.90579; geodeticDatum: WGS84; **Event:** eventID: 1; samplingProtocol: Sweeping; eventTime: Day
- **Type status:** Other material. **Occurrence:** individualCount: 1; sex: female; **Location:** locationID: P3; continent: Europe; country: Spain; countryCode: ES; stateProvince: Castilla y León; county: León; locality: Las Arroyas; verbatimElevation: 1097.1; decimalLatitude: 43.14351; decimalLongitude: -4.94878; geodeticDatum: WGS84; **Event:** eventID: 1; samplingProtocol: Beating; eventTime: Day

##### Distribution

Palearctic

##### Notes

Females of *A. fuscipalpa*, *A. pseudorurestris* and *A. rurestris* could not be differentiated through morphology. Moreover, DNA identification could not distinguish *A. pseudorurestris* from *A. rurestris* (see *Species delimitation and identification using DNA barcodes*). Consequently, females were assigned to each species based on the geographical location of identified males. In the case of Cabañeros, where males of the two species were found, females were arbitrarily assigned to *A. rurestris*.

#### Agyneta simplicitarsis

(Simon, 1884)

##### Materials

- **Type status:** Other material. **Occurrence:** individualCount: 1; sex: male; **Location:** locationID: O1; continent: Europe; country: Spain; countryCode: ES; stateProvince: Aragón; county: Huesca; locality: O Furno; verbatimElevation: 1396.73; decimalLatitude: 42.60677; decimalLongitude: 0.13135; geodeticDatum: WGS84; **Event:** eventID: D; samplingProtocol: Pitfall

##### Distribution

Europe, Russia, Kazakhstan

##### Notes

Although placed in the same genetic lineage than *A. orites* by DNA barcodes, we maintain our morphology-based identification given the clear shape of traditional diagnostic characters used in linyphiine taxonomy, namely the tibia, paracymbium and lamella characteristica, which are close to or equal to those depicted by Tanasevitch (Tanasevitch 2005).

#### Bordea negrei

(Dresco, 1951)

##### Materials

- **Type status:** Other material. **Occurrence:** individualCount: 1; sex: female; **Location:** locationID: A2; continent: Europe; country: Spain; countryCode: ES; stateProvince: Catalonia; county: Lleida; locality: Sola de Boi; verbatimElevation: 1738.7; decimalLatitude: 42.54913; decimalLongitude: 0.87137; geodeticDatum: WGS84; **Event:** eventID: J; samplingProtocol: Pitfall

##### Distribution

Spain, France

#### Canariphantes zonatus

(Simon, 1884)

##### Materials

- **Type status:** Other material. **Occurrence:** individualCount: 1; sex: male; **Location:** locationID: C1; continent: Europe; country: Spain; countryCode: ES; stateProvince: Castilla-La Mancha; county: Ciudad Real; locality: Valle Brezoso; verbatimElevation: 756.56; decimalLatitude: 39.35663; decimalLongitude: -4.35912; geodeticDatum: WGS84; **Event:** eventID: C; samplingProtocol: Pitfall
- **Type status:** Other material. **Occurrence:** individualCount: 1; sex: female; **Location:** locationID: C1; continent: Europe; country: Spain; countryCode: ES; stateProvince: Castilla-La Mancha; county: Ciudad Real; locality: Valle Brezoso; verbatimElevation: 756.56; decimalLatitude: 39.35663; decimalLongitude: -4.35912; geodeticDatum: WGS84; **Event:** eventID: C; samplingProtocol: Pitfall
- **Type status:** Other material. **Occurrence:** individualCount: 2; sex: female; **Location:** locationID: C2; continent: Europe; country: Spain; countryCode: ES; stateProvince: Castilla-La Mancha; county: Ciudad Real; locality: Valle Brezoso; verbatimElevation: 739.31; decimalLatitude: 39.35159; decimalLongitude: -4.3589; geodeticDatum: WGS84; **Event:** eventID: A; samplingProtocol: Pitfall
- **Type status:** Other material. **Occurrence:** individualCount: 2; sex: female; **Location:** locationID: C2; continent: Europe; country: Spain; countryCode: ES; stateProvince: Castilla-La Mancha; county: Ciudad Real; locality: Valle Brezoso; verbatimElevation: 739.31; decimalLatitude: 39.35159; decimalLongitude: -4.3589; geodeticDatum: WGS84; **Event:** eventID: B; samplingProtocol: Pitfall
- **Type status:** Other material. **Occurrence:** individualCount: 2; sex: female; **Location:** locationID: C2; continent: Europe; country: Spain; countryCode: ES; stateProvince: Castilla-La Mancha; county: Ciudad Real; locality: Valle Brezoso; verbatimElevation: 739.31; decimalLatitude: 39.35159; decimalLongitude: -4.3589; geodeticDatum: WGS84; **Event:** eventID: K; samplingProtocol: Pitfall
- **Type status:** Other material. **Occurrence:** individualCount: 1; sex: female; **Location:** locationID: C2; continent: Europe; country: Spain; countryCode: ES; stateProvince: Castilla-La Mancha; county: Ciudad Real; locality: Valle Brezoso; verbatimElevation: 739.31; decimalLatitude: 39.35159; decimalLongitude: -4.3589; geodeticDatum: WGS84; **Event:** eventID: L; samplingProtocol: Pitfall
- **Type status:** Other material. **Occurrence:** individualCount: 1; sex: female; **Location:** locationID: C3; continent: Europe; country: Spain; countryCode: ES; stateProvince: Castilla-La Mancha; county: Ciudad Real; locality: La Quesera; verbatimElevation: 767.55; decimalLatitude: 39.36177; decimalLongitude: -4.41733; geodeticDatum: WGS84; **Event:** eventID: A; samplingProtocol: Pitfall
- **Type status:** Other material. **Occurrence:** individualCount: 3; sex: female; **Location:** locationID: C3; continent: Europe; country: Spain; countryCode: ES; stateProvince: Castilla-La Mancha; county: Ciudad Real; locality: La Quesera; verbatimElevation: 767.55; decimalLatitude: 39.36177; decimalLongitude: -4.41733; geodeticDatum: WGS84; **Event:** eventID: D; samplingProtocol: Pitfall
- **Type status:** Other material. **Occurrence:** individualCount: 2; sex: female; **Location:** locationID: C3; continent: Europe; country: Spain; countryCode: ES; stateProvince: Castilla-La Mancha; county: Ciudad Real; locality: La Quesera; verbatimElevation: 767.55; decimalLatitude: 39.36177; decimalLongitude: -4.41733; geodeticDatum: WGS84; **Event:** eventID: E; samplingProtocol: Pitfall
- **Type status:** Other material. **Occurrence:** individualCount: 1; sex: female; **Location:** locationID: C3; continent: Europe; country: Spain; countryCode: ES; stateProvince: Castilla-La Mancha; county: Ciudad Real; locality: La Quesera; verbatimElevation: 767.55; decimalLatitude: 39.36177; decimalLongitude: -4.41733; geodeticDatum: WGS84; **Event:** eventID: F; samplingProtocol: Pitfall
- **Type status:** Other material. **Occurrence:** individualCount: 1; sex: female; **Location:** locationID: C3; continent: Europe; country: Spain; countryCode: ES; stateProvince: Castilla-La Mancha; county: Ciudad Real; locality: La Quesera; verbatimElevation: 767.55; decimalLatitude: 39.36177; decimalLongitude: -4.41733; geodeticDatum: WGS84; **Event:** eventID: H; samplingProtocol: Pitfall
- **Type status:** Other material. **Occurrence:** individualCount: 2; sex: female; **Location:** locationID: C4; continent: Europe; country: Spain; countryCode: ES; stateProvince: Castilla-La Mancha; county: Ciudad Real; locality: La Quesera; verbatimElevation: 772.3; decimalLatitude: 39.36337; decimalLongitude: -4.41704; geodeticDatum: WGS84; **Event:** eventID: E; samplingProtocol: Pitfall
- **Type status:** Other material. **Occurrence:** individualCount: 1; sex: female; **Location:** locationID: C4; continent: Europe; country: Spain; countryCode: ES; stateProvince: Castilla-La Mancha; county: Ciudad Real; locality: La Quesera; verbatimElevation: 772.3; decimalLatitude: 39.36337; decimalLongitude: -4.41704; geodeticDatum: WGS84; **Event:** eventID: F; samplingProtocol: Pitfall
- **Type status:** Other material. **Occurrence:** individualCount: 1; sex: female; **Location:** locationID: C4; continent: Europe; country: Spain; countryCode: ES; stateProvince: Castilla-La Mancha; county: Ciudad Real; locality: La Quesera; verbatimElevation: 772.3; decimalLatitude: 39.36337; decimalLongitude: -4.41704; geodeticDatum: WGS84; **Event:** eventID: G; samplingProtocol: Pitfall
- **Type status:** Other material. **Occurrence:** individualCount: 1; sex: female; **Location:** locationID: C4; continent: Europe; country: Spain; countryCode: ES; stateProvince: Castilla-La Mancha; county: Ciudad Real; locality: La Quesera; verbatimElevation: 772.3; decimalLatitude: 39.36337; decimalLongitude: -4.41704; geodeticDatum: WGS84; **Event:** eventID: J; samplingProtocol: Pitfall
- **Type status:** Other material. **Occurrence:** individualCount: 1; sex: female; **Location:** locationID: C4; continent: Europe; country: Spain; countryCode: ES; stateProvince: Castilla-La Mancha; county: Ciudad Real; locality: La Quesera; verbatimElevation: 772.3; decimalLatitude: 39.36337; decimalLongitude: -4.41704; geodeticDatum: WGS84; **Event:** eventID: K; samplingProtocol: Pitfall
- **Type status:** Other material. **Occurrence:** individualCount: 1; sex: male; **Location:** locationID: M1; continent: Europe; country: Spain; countryCode: ES; stateProvince: Extremadura; county: Cáceres; locality: Peña Falcón; verbatimElevation: 320.6; decimalLatitude: 39.83296; decimalLongitude: -6.0641; geodeticDatum: WGS84; **Event:** eventID: A; samplingProtocol: Pitfall

##### Distribution

Iberian Peninsula, France, Sardinia, Algeria, Morocco, Tunisia

#### Centromerus pabulator

(O. Pickard-Cambridge, 1875)

##### Materials

- **Type status:** Other material. **Occurrence:** individualCount: 1; sex: female; **Location:** locationID: P1; continent: Europe; country: Spain; countryCode: ES; stateProvince: Castilla y León; county: León; locality: Monte Robledo; verbatimElevation: 1071.58; decimalLatitude: 43.1445; decimalLongitude: -4.92675; geodeticDatum: WGS84; **Event:** eventID: F; samplingProtocol: Pitfall
- **Type status:** Other material. **Occurrence:** individualCount: 1; sex: female; **Location:** locationID: P1; continent: Europe; country: Spain; countryCode: ES; stateProvince: Castilla y León; county: León; locality: Monte Robledo; verbatimElevation: 1071.58; decimalLatitude: 43.1445; decimalLongitude: -4.92675; geodeticDatum: WGS84; **Event:** eventID: G; samplingProtocol: Pitfall

##### Distribution

Europe, Russia

#### Centromerus prudens

(O. Pickard-Cambridge, 1873)

##### Materials

- **Type status:** Other material. **Occurrence:** individualCount: 1; sex: female; **Location:** locationID: S1; continent: Europe; country: Spain; countryCode: ES; stateProvince: Andalucía; county: Granada; locality: Soportujar; verbatimElevation: 1786.57; decimalLatitude: 36.96151; decimalLongitude: -3.41881; geodeticDatum: WGS84; **Event:** eventID: I; samplingProtocol: Pitfall

##### Distribution

Palearctic

#### Centromerus sellarius

(Simon, 1884)

##### Materials

- **Type status:** Other material. **Occurrence:** individualCount: 1; sex: female; **Location:** locationID: P1; continent: Europe; country: Spain; countryCode: ES; stateProvince: Castilla y León; county: León; locality: Monte Robledo; verbatimElevation: 1071.58; decimalLatitude: 43.1445; decimalLongitude: -4.92675; geodeticDatum: WGS84; **Event:** eventID: E; samplingProtocol: Pitfall

##### Distribution

Europe

#### Ceratinella scabrosa

(O. Pickard-Cambridge, 1871)

##### Materials

- **Type status:** Other material. **Occurrence:** individualCount: 1; sex: male; **Location:** locationID: O1; continent: Europe; country: Spain; countryCode: ES; stateProvince: Aragón; county: Huesca; locality: O Furno; verbatimElevation: 1396.73; decimalLatitude: 42.60677; decimalLongitude: 0.13135; geodeticDatum: WGS84; **Event:** eventID: E; samplingProtocol: Pitfall
- **Type status:** Other material. **Occurrence:** individualCount: 1; sex: male; **Location:** locationID: O1; continent: Europe; country: Spain; countryCode: ES; stateProvince: Aragón; county: Huesca; locality: O Furno; verbatimElevation: 1396.73; decimalLatitude: 42.60677; decimalLongitude: 0.13135; geodeticDatum: WGS84; **Event:** eventID: F; samplingProtocol: Pitfall
- **Type status:** Other material. **Occurrence:** individualCount: 1; sex: male; **Location:** locationID: O1; continent: Europe; country: Spain; countryCode: ES; stateProvince: Aragón; county: Huesca; locality: O Furno; verbatimElevation: 1396.73; decimalLatitude: 42.60677; decimalLongitude: 0.13135; geodeticDatum: WGS84; **Event:** eventID: 1; samplingProtocol: Sweeping; eventTime: Day
- **Type status:** Other material. **Occurrence:** individualCount: 1; sex: male; **Location:** locationID: P4; continent: Europe; country: Spain; countryCode: ES; stateProvince: Castilla y León; county: León; locality: El Canto; verbatimElevation: 943.48; decimalLatitude: 43.17227; decimalLongitude: -4.90857; geodeticDatum: WGS84; **Event:** eventID: C; samplingProtocol: Pitfall
- **Type status:** Other material. **Occurrence:** individualCount: 1; sex: male; **Location:** locationID: P4; continent: Europe; country: Spain; countryCode: ES; stateProvince: Castilla y León; county: León; locality: El Canto; verbatimElevation: 943.48; decimalLatitude: 43.17227; decimalLongitude: -4.90857; geodeticDatum: WGS84; **Event:** eventID: D; samplingProtocol: Pitfall

##### Distribution

Palearctic

#### Diplocephalus picinus

(Blackwall, 1841)

##### Materials

- **Type status:** Other material. **Occurrence:** individualCount: 9; sex: male; **Location:** locationID: P1; continent: Europe; country: Spain; countryCode: ES; stateProvince: Castilla y León; county: León; locality: Monte Robledo; verbatimElevation: 1071.58; decimalLatitude: 43.1445; decimalLongitude: -4.92675; geodeticDatum: WGS84; **Event:** eventID: J; samplingProtocol: Pitfall
- **Type status:** Other material. **Occurrence:** individualCount: 1; sex: female; **Location:** locationID: P1; continent: Europe; country: Spain; countryCode: ES; stateProvince: Castilla y León; county: León; locality: Monte Robledo; verbatimElevation: 1071.58; decimalLatitude: 43.1445; decimalLongitude: -4.92675; geodeticDatum: WGS84; **Event:** eventID: J; samplingProtocol: Pitfall

##### Distribution

Palearctic

#### Entelecara acuminata

(Wider, 1834)

##### Materials

- **Type status:** Other material. **Occurrence:** individualCount: 1; sex: male; **Location:** locationID: O1; continent: Europe; country: Spain; countryCode: ES; stateProvince: Aragón; county: Huesca; locality: O Furno; verbatimElevation: 1396.73; decimalLatitude: 42.60677; decimalLongitude: 0.13135; geodeticDatum: WGS84; **Event:** eventID: 1; samplingProtocol: Beating; eventTime: Night
- **Type status:** Other material. **Occurrence:** individualCount: 1; sex: female; **Location:** locationID: O1; continent: Europe; country: Spain; countryCode: ES; stateProvince: Aragón; county: Huesca; locality: O Furno; verbatimElevation: 1396.73; decimalLatitude: 42.60677; decimalLongitude: 0.13135; geodeticDatum: WGS84; **Event:** eventID: 1; samplingProtocol: Beating; eventTime: Night
- **Type status:** Other material. **Occurrence:** individualCount: 1; sex: male; **Location:** locationID: O1; continent: Europe; country: Spain; countryCode: ES; stateProvince: Aragón; county: Huesca; locality: O Furno; verbatimElevation: 1396.73; decimalLatitude: 42.60677; decimalLongitude: 0.13135; geodeticDatum: WGS84; **Event:** eventID: 1; samplingProtocol: Sweeping; eventTime: Night
- **Type status:** Other material. **Occurrence:** individualCount: 1; sex: female; **Location:** locationID: O2; continent: Europe; country: Spain; countryCode: ES; stateProvince: Aragón; county: Huesca; locality: Rebilla; verbatimElevation: 1158.13; decimalLatitude: 42.59427; decimalLongitude: 0.1529; geodeticDatum: WGS84; **Event:** eventID: 1; samplingProtocol: Beating; eventTime: Day
- **Type status:** Other material. **Occurrence:** individualCount: 1; sex: female; **Location:** locationID: O2; continent: Europe; country: Spain; countryCode: ES; stateProvince: Aragón; county: Huesca; locality: Rebilla; verbatimElevation: 1158.13; decimalLatitude: 42.59427; decimalLongitude: 0.1529; geodeticDatum: WGS84; **Event:** eventID: 2; samplingProtocol: Beating; eventTime: Day
- **Type status:** Other material. **Occurrence:** individualCount: 1; sex: female; **Location:** locationID: O2; continent: Europe; country: Spain; countryCode: ES; stateProvince: Aragón; county: Huesca; locality: Rebilla; verbatimElevation: 1158.13; decimalLatitude: 42.59427; decimalLongitude: 0.1529; geodeticDatum: WGS84; **Event:** eventID: 2; samplingProtocol: Sweeping; eventTime: Day

##### Distribution

Holarctic

#### Erigone dentipalpis

(Wider, 1834)

##### Materials

- **Type status:** Other material. **Occurrence:** individualCount: 1; sex: male; **Location:** locationID: M2; continent: Europe; country: Spain; countryCode: ES; stateProvince: Extremadura; county: Cáceres; locality: Fuente del Frances; verbatimElevation: 320.72; decimalLatitude: 39.828; decimalLongitude: -6.03249; geodeticDatum: WGS84; **Event:** eventID: 1; samplingProtocol: Beating; eventTime: Night

##### Distribution

Holarctic

#### Frontinellina frutetorum

(C. L. Koch, 1834)

##### Materials

- **Type status:** Other material. **Occurrence:** individualCount: 1; sex: female; **Location:** locationID: C1; continent: Europe; country: Spain; countryCode: ES; stateProvince: Castilla-La Mancha; county: Ciudad Real; locality: Valle Brezoso; verbatimElevation: 756.56; decimalLatitude: 39.35663; decimalLongitude: -4.35912; geodeticDatum: WGS84; **Event:** eventID: 4; samplingProtocol: Aerial; eventTime: Night
- **Type status:** Other material. **Occurrence:** individualCount: 1; sex: female; **Location:** locationID: C1; continent: Europe; country: Spain; countryCode: ES; stateProvince: Castilla-La Mancha; county: Ciudad Real; locality: Valle Brezoso; verbatimElevation: 756.56; decimalLatitude: 39.35663; decimalLongitude: -4.35912; geodeticDatum: WGS84; **Event:** eventID: 2; samplingProtocol: Sweeping; eventTime: Day
- **Type status:** Other material. **Occurrence:** individualCount: 2; sex: female; **Location:** locationID: C2; continent: Europe; country: Spain; countryCode: ES; stateProvince: Castilla-La Mancha; county: Ciudad Real; locality: Valle Brezoso; verbatimElevation: 739.31; decimalLatitude: 39.35159; decimalLongitude: -4.3589; geodeticDatum: WGS84; **Event:** eventID: 1; samplingProtocol: Sweeping; eventTime: Day
- **Type status:** Other material. **Occurrence:** individualCount: 1; sex: female; **Location:** locationID: C2; continent: Europe; country: Spain; countryCode: ES; stateProvince: Castilla-La Mancha; county: Ciudad Real; locality: Valle Brezoso; verbatimElevation: 739.31; decimalLatitude: 39.35159; decimalLongitude: -4.3589; geodeticDatum: WGS84; **Event:** eventID: 1; samplingProtocol: Sweeping; eventTime: Night
- **Type status:** Other material. **Occurrence:** individualCount: 2; sex: female; **Location:** locationID: C2; continent: Europe; country: Spain; countryCode: ES; stateProvince: Castilla-La Mancha; county: Ciudad Real; locality: Valle Brezoso; verbatimElevation: 739.31; decimalLatitude: 39.35159; decimalLongitude: -4.3589; geodeticDatum: WGS84; **Event:** eventID: 2; samplingProtocol: Sweeping; eventTime: Day
- **Type status:** Other material. **Occurrence:** individualCount: 1; sex: female; **Location:** locationID: C3; continent: Europe; country: Spain; countryCode: ES; stateProvince: Castilla-La Mancha; county: Ciudad Real; locality: La Quesera; verbatimElevation: 767.55; decimalLatitude: 39.36177; decimalLongitude: -4.41733; geodeticDatum: WGS84; **Event:** eventID: 3; samplingProtocol: Aerial; eventTime: Night
- **Type status:** Other material. **Occurrence:** individualCount: 3; sex: female; **Location:** locationID: C3; continent: Europe; country: Spain; countryCode: ES; stateProvince: Castilla-La Mancha; county: Ciudad Real; locality: La Quesera; verbatimElevation: 767.55; decimalLatitude: 39.36177; decimalLongitude: -4.41733; geodeticDatum: WGS84; **Event:** eventID: 1; samplingProtocol: Sweeping; eventTime: Day
- **Type status:** Other material. **Occurrence:** individualCount: 1; sex: female; **Location:** locationID: C3; continent: Europe; country: Spain; countryCode: ES; stateProvince: Castilla-La Mancha; county: Ciudad Real; locality: La Quesera; verbatimElevation: 767.55; decimalLatitude: 39.36177; decimalLongitude: -4.41733; geodeticDatum: WGS84; **Event:** eventID: 1; samplingProtocol: Sweeping; eventTime: Night
- **Type status:** Other material. **Occurrence:** individualCount: 1; sex: female; **Location:** locationID: C3; continent: Europe; country: Spain; countryCode: ES; stateProvince: Castilla-La Mancha; county: Ciudad Real; locality: La Quesera; verbatimElevation: 767.55; decimalLatitude: 39.36177; decimalLongitude: -4.41733; geodeticDatum: WGS84; **Event:** eventID: 2; samplingProtocol: Sweeping; eventTime: Night
- **Type status:** Other material. **Occurrence:** individualCount: 1; sex: female; **Location:** locationID: C4; continent: Europe; country: Spain; countryCode: ES; stateProvince: Castilla-La Mancha; county: Ciudad Real; locality: La Quesera; verbatimElevation: 772.3; decimalLatitude: 39.36337; decimalLongitude: -4.41704; geodeticDatum: WGS84; **Event:** eventID: 1; samplingProtocol: Sweeping; eventTime: Day
- **Type status:** Other material. **Occurrence:** individualCount: 1; sex: female; **Location:** locationID: M2; continent: Europe; country: Spain; countryCode: ES; stateProvince: Extremadura; county: Cáceres; locality: Fuente del Frances; verbatimElevation: 320.72; decimalLatitude: 39.828; decimalLongitude: -6.03249; geodeticDatum: WGS84; **Event:** eventID: 4; samplingProtocol: Aerial; eventTime: Night
- **Type status:** Other material. **Occurrence:** individualCount: 1; sex: female; **Location:** locationID: O2; continent: Europe; country: Spain; countryCode: ES; stateProvince: Aragón; county: Huesca; locality: Rebilla; verbatimElevation: 1158.13; decimalLatitude: 42.59427; decimalLongitude: 0.1529; geodeticDatum: WGS84; **Event:** eventID: 1; samplingProtocol: Aerial; eventTime: Night
- **Type status:** Other material. **Occurrence:** individualCount: 2; sex: female; **Location:** locationID: O2; continent: Europe; country: Spain; countryCode: ES; stateProvince: Aragón; county: Huesca; locality: Rebilla; verbatimElevation: 1158.13; decimalLatitude: 42.59427; decimalLongitude: 0.1529; geodeticDatum: WGS84; **Event:** eventID: 2; samplingProtocol: Aerial; eventTime: Night
- **Type status:** Other material. **Occurrence:** individualCount: 1; sex: male; **Location:** locationID: O2; continent: Europe; country: Spain; countryCode: ES; stateProvince: Aragón; county: Huesca; locality: Rebilla; verbatimElevation: 1158.13; decimalLatitude: 42.59427; decimalLongitude: 0.1529; geodeticDatum: WGS84; **Event:** eventID: 1; samplingProtocol: Sweeping; eventTime: Night
- **Type status:** Other material. **Occurrence:** individualCount: 2; sex: female; **Location:** locationID: O2; continent: Europe; country: Spain; countryCode: ES; stateProvince: Aragón; county: Huesca; locality: Rebilla; verbatimElevation: 1158.13; decimalLatitude: 42.59427; decimalLongitude: 0.1529; geodeticDatum: WGS84; **Event:** eventID: 2; samplingProtocol: Sweeping; eventTime: Day
- **Type status:** Other material. **Occurrence:** individualCount: 4; sex: female; **Location:** locationID: P2; continent: Europe; country: Spain; countryCode: ES; stateProvince: Castilla y León; county: León; locality: Joyoguelas; verbatimElevation: 763.98; decimalLatitude: 43.17771; decimalLongitude: -4.90579; geodeticDatum: WGS84; **Event:** eventID: 1; samplingProtocol: Aerial; eventTime: Night
- **Type status:** Other material. **Occurrence:** individualCount: 1; sex: female; **Location:** locationID: P2; continent: Europe; country: Spain; countryCode: ES; stateProvince: Castilla y León; county: León; locality: Joyoguelas; verbatimElevation: 763.98; decimalLatitude: 43.17771; decimalLongitude: -4.90579; geodeticDatum: WGS84; **Event:** eventID: 2; samplingProtocol: Aerial; eventTime: Night
- **Type status:** Other material. **Occurrence:** individualCount: 1; sex: female; **Location:** locationID: P2; continent: Europe; country: Spain; countryCode: ES; stateProvince: Castilla y León; county: León; locality: Joyoguelas; verbatimElevation: 763.98; decimalLatitude: 43.17771; decimalLongitude: -4.90579; geodeticDatum: WGS84; **Event:** eventID: 2; samplingProtocol: Beating; eventTime: Day
- **Type status:** Other material. **Occurrence:** individualCount: 3; sex: female; **Location:** locationID: P2; continent: Europe; country: Spain; countryCode: ES; stateProvince: Castilla y León; county: León; locality: Joyoguelas; verbatimElevation: 763.98; decimalLatitude: 43.17771; decimalLongitude: -4.90579; geodeticDatum: WGS84; **Event:** eventID: 1; samplingProtocol: Sweeping; eventTime: Day
- **Type status:** Other material. **Occurrence:** individualCount: 5; sex: male; **Location:** locationID: P2; continent: Europe; country: Spain; countryCode: ES; stateProvince: Castilla y León; county: León; locality: Joyoguelas; verbatimElevation: 763.98; decimalLatitude: 43.17771; decimalLongitude: -4.90579; geodeticDatum: WGS84; **Event:** eventID: 1; samplingProtocol: Sweeping; eventTime: Night
- **Type status:** Other material. **Occurrence:** individualCount: 2; sex: female; **Location:** locationID: P2; continent: Europe; country: Spain; countryCode: ES; stateProvince: Castilla y León; county: León; locality: Joyoguelas; verbatimElevation: 763.98; decimalLatitude: 43.17771; decimalLongitude: -4.90579; geodeticDatum: WGS84; **Event:** eventID: 1; samplingProtocol: Sweeping; eventTime: Night
- **Type status:** Other material. **Occurrence:** individualCount: 6; sex: female; **Location:** locationID: P2; continent: Europe; country: Spain; countryCode: ES; stateProvince: Castilla y León; county: León; locality: Joyoguelas; verbatimElevation: 763.98; decimalLatitude: 43.17771; decimalLongitude: -4.90579; geodeticDatum: WGS84; **Event:** eventID: 1; samplingProtocol: Sweeping; eventTime: Night
- **Type status:** Other material. **Occurrence:** individualCount: 2; sex: male; **Location:** locationID: P2; continent: Europe; country: Spain; countryCode: ES; stateProvince: Castilla y León; county: León; locality: Joyoguelas; verbatimElevation: 763.98; decimalLatitude: 43.17771; decimalLongitude: -4.90579; geodeticDatum: WGS84; **Event:** eventID: 2; samplingProtocol: Sweeping; eventTime: Day
- **Type status:** Other material. **Occurrence:** individualCount: 4; sex: female; **Location:** locationID: P2; continent: Europe; country: Spain; countryCode: ES; stateProvince: Castilla y León; county: León; locality: Joyoguelas; verbatimElevation: 763.98; decimalLatitude: 43.17771; decimalLongitude: -4.90579; geodeticDatum: WGS84; **Event:** eventID: 2; samplingProtocol: Sweeping; eventTime: Day
- **Type status:** Other material. **Occurrence:** individualCount: 2; sex: female; **Location:** locationID: P3; continent: Europe; country: Spain; countryCode: ES; stateProvince: Castilla y León; county: León; locality: Las Arroyas; verbatimElevation: 1097.1; decimalLatitude: 43.14351; decimalLongitude: -4.94878; geodeticDatum: WGS84; **Event:** eventID: 1; samplingProtocol: Sweeping; eventTime: Day
- **Type status:** Other material. **Occurrence:** individualCount: 7; sex: female; **Location:** locationID: P4; continent: Europe; country: Spain; countryCode: ES; stateProvince: Castilla y León; county: León; locality: El Canto; verbatimElevation: 943.48; decimalLatitude: 43.17227; decimalLongitude: -4.90857; geodeticDatum: WGS84; **Event:** eventID: 1; samplingProtocol: Aerial; eventTime: Night
- **Type status:** Other material. **Occurrence:** individualCount: 1; sex: female; **Location:** locationID: P4; continent: Europe; country: Spain; countryCode: ES; stateProvince: Castilla y León; county: León; locality: El Canto; verbatimElevation: 943.48; decimalLatitude: 43.17227; decimalLongitude: -4.90857; geodeticDatum: WGS84; **Event:** eventID: 2; samplingProtocol: Aerial; eventTime: Night
- **Type status:** Other material. **Occurrence:** individualCount: 1; sex: male; **Location:** locationID: P4; continent: Europe; country: Spain; countryCode: ES; stateProvince: Castilla y León; county: León; locality: El Canto; verbatimElevation: 943.48; decimalLatitude: 43.17227; decimalLongitude: -4.90857; geodeticDatum: WGS84; **Event:** eventID: 2; samplingProtocol: Aerial; eventTime: Night
- **Type status:** Other material. **Occurrence:** individualCount: 4; sex: female; **Location:** locationID: P4; continent: Europe; country: Spain; countryCode: ES; stateProvince: Castilla y León; county: León; locality: El Canto; verbatimElevation: 943.48; decimalLatitude: 43.17227; decimalLongitude: -4.90857; geodeticDatum: WGS84; **Event:** eventID: 2; samplingProtocol: Aerial; eventTime: Night
- **Type status:** Other material. **Occurrence:** individualCount: 2; sex: male; **Location:** locationID: P4; continent: Europe; country: Spain; countryCode: ES; stateProvince: Castilla y León; county: León; locality: El Canto; verbatimElevation: 943.48; decimalLatitude: 43.17227; decimalLongitude: -4.90857; geodeticDatum: WGS84; **Event:** eventID: 1; samplingProtocol: Sweeping; eventTime: Day
- **Type status:** Other material. **Occurrence:** individualCount: 5; sex: female; **Location:** locationID: P4; continent: Europe; country: Spain; countryCode: ES; stateProvince: Castilla y León; county: León; locality: El Canto; verbatimElevation: 943.48; decimalLatitude: 43.17227; decimalLongitude: -4.90857; geodeticDatum: WGS84; **Event:** eventID: 1; samplingProtocol: Sweeping; eventTime: Day
- **Type status:** Other material. **Occurrence:** individualCount: 2; sex: female; **Location:** locationID: P4; continent: Europe; country: Spain; countryCode: ES; stateProvince: Castilla y León; county: León; locality: El Canto; verbatimElevation: 943.48; decimalLatitude: 43.17227; decimalLongitude: -4.90857; geodeticDatum: WGS84; **Event:** eventID: 1; samplingProtocol: Sweeping; eventTime: Night
- **Type status:** Other material. **Occurrence:** individualCount: 1; sex: male; **Location:** locationID: P4; continent: Europe; country: Spain; countryCode: ES; stateProvince: Castilla y León; county: León; locality: El Canto; verbatimElevation: 943.48; decimalLatitude: 43.17227; decimalLongitude: -4.90857; geodeticDatum: WGS84; **Event:** eventID: 2; samplingProtocol: Sweeping; eventTime: Day
- **Type status:** Other material. **Occurrence:** individualCount: 3; sex: female; **Location:** locationID: P4; continent: Europe; country: Spain; countryCode: ES; stateProvince: Castilla y León; county: León; locality: El Canto; verbatimElevation: 943.48; decimalLatitude: 43.17227; decimalLongitude: -4.90857; geodeticDatum: WGS84; **Event:** eventID: 2; samplingProtocol: Sweeping; eventTime: Day
- **Type status:** Other material. **Occurrence:** individualCount: 1; sex: female; **Location:** locationID: S2; continent: Europe; country: Spain; countryCode: ES; stateProvince: Andalucía; county: Granada; locality: Camarate; verbatimElevation: 1713.96; decimalLatitude: 37.18377; decimalLongitude: -3.26282; geodeticDatum: WGS84; **Event:** eventID: 1; samplingProtocol: Sweeping; eventTime: Day
- **Type status:** Other material. **Occurrence:** individualCount: 1; sex: female; **Location:** locationID: S2; continent: Europe; country: Spain; countryCode: ES; stateProvince: Andalucía; county: Granada; locality: Camarate; verbatimElevation: 1713.96; decimalLatitude: 37.18377; decimalLongitude: -3.26282; geodeticDatum: WGS84; **Event:** eventID: 2; samplingProtocol: Sweeping; eventTime: Night
- **Type status:** Other material. **Occurrence:** individualCount: 2; sex: female; **Location:** locationID: S2; continent: Europe; country: Spain; countryCode: ES; stateProvince: Andalucía; county: Granada; locality: Camarate; verbatimElevation: 1713.96; decimalLatitude: 37.18377; decimalLongitude: -3.26282; geodeticDatum: WGS84; **Event:** eventID: 2; samplingProtocol: Sweeping; eventTime: Day

##### Distribution

Palearctic

#### Gonatium rubens

(Blackwall, 1833)

##### Materials

- **Type status:** Other material. **Occurrence:** individualCount: 1; sex: female; **Location:** locationID: P2; continent: Europe; country: Spain; countryCode: ES; stateProvince: Castilla y León; county: León; locality: Joyoguelas; verbatimElevation: 763.98; decimalLatitude: 43.17771; decimalLongitude: -4.90579; geodeticDatum: WGS84; **Event:** eventID: L; samplingProtocol: Pitfall
- **Type status:** Other material. **Occurrence:** individualCount: 1; sex: female; **Location:** locationID: P3; continent: Europe; country: Spain; countryCode: ES; stateProvince: Castilla y León; county: León; locality: Las Arroyas; verbatimElevation: 1097.1; decimalLatitude: 43.14351; decimalLongitude: -4.94878; geodeticDatum: WGS84; **Event:** eventID: J; samplingProtocol: Pitfall
- **Type status:** Other material. **Occurrence:** individualCount: 1; sex: female; **Location:** locationID: P4; continent: Europe; country: Spain; countryCode: ES; stateProvince: Castilla y León; county: León; locality: El Canto; verbatimElevation: 943.48; decimalLatitude: 43.17227; decimalLongitude: -4.90857; geodeticDatum: WGS84; **Event:** eventID: E; samplingProtocol: Pitfall

##### Distribution

Palearctic

##### Notes

Uncertain identification, considering the similarities to *G. ensipotens* (Simon, 1881).

#### Gongylidiellum murcidum

Simon, 1884

##### Materials

- **Type status:** Other material. **Occurrence:** individualCount: 1; sex: male; **Location:** locationID: O1; continent: Europe; country: Spain; countryCode: ES; stateProvince: Aragón; county: Huesca; locality: O Furno; verbatimElevation: 1396.73; decimalLatitude: 42.60677; decimalLongitude: 0.13135; geodeticDatum: WGS84; **Event:** eventID: D; samplingProtocol: Pitfall
- **Type status:** Other material. **Occurrence:** individualCount: 1; sex: male; **Location:** locationID: O1; continent: Europe; country: Spain; countryCode: ES; stateProvince: Aragón; county: Huesca; locality: O Furno; verbatimElevation: 1396.73; decimalLatitude: 42.60677; decimalLongitude: 0.13135; geodeticDatum: WGS84; **Event:** eventID: L; samplingProtocol: Pitfall

##### Distribution

Palearctic

#### Labulla flahaulti

Simon, 1915

##### Materials

- **Type status:** Other material. **Occurrence:** individualCount: 1; sex: female; **Location:** locationID: P2; continent: Europe; country: Spain; countryCode: ES; stateProvince: Castilla y León; county: León; locality: Joyoguelas; verbatimElevation: 763.98; decimalLatitude: 43.17771; decimalLongitude: -4.90579; geodeticDatum: WGS84; **Event:** eventID: 1; samplingProtocol: Aerial; eventTime: Night
- **Type status:** Other material. **Occurrence:** individualCount: 1; sex: female; **Location:** locationID: P3; continent: Europe; country: Spain; countryCode: ES; stateProvince: Castilla y León; county: León; locality: Las Arroyas; verbatimElevation: 1097.1; decimalLatitude: 43.14351; decimalLongitude: -4.94878; geodeticDatum: WGS84; **Event:** eventID: 1; samplingProtocol: Aerial; eventTime: Night
- **Type status:** Other material. **Occurrence:** individualCount: 1; sex: female; **Location:** locationID: P4; continent: Europe; country: Spain; countryCode: ES; stateProvince: Castilla y León; county: León; locality: El Canto; verbatimElevation: 943.48; decimalLatitude: 43.17227; decimalLongitude: -4.90857; geodeticDatum: WGS84; **Event:** eventID: 2; samplingProtocol: Aerial; eventTime: Night

##### Distribution

Spain, France

#### Lepthyphantes minutus

(Blackwall, 1833)

##### Materials

- **Type status:** Other material. **Occurrence:** individualCount: 1; sex: female; **Location:** locationID: A1; continent: Europe; country: Spain; countryCode: ES; stateProvince: Catalonia; county: Lleida; locality: Sola de Boi; verbatimElevation: 1759.8; decimalLatitude: 42.54958; decimalLongitude: 0.87254; geodeticDatum: WGS84; **Event:** eventID: 1; samplingProtocol: Aerial; eventTime: Night
- **Type status:** Other material. **Occurrence:** individualCount: 1; sex: female; **Location:** locationID: A1; continent: Europe; country: Spain; countryCode: ES; stateProvince: Catalonia; county: Lleida; locality: Sola de Boi; verbatimElevation: 1759.8; decimalLatitude: 42.54958; decimalLongitude: 0.87254; geodeticDatum: WGS84; **Event:** eventID: 2; samplingProtocol: Aerial; eventTime: Night
- **Type status:** Other material. **Occurrence:** individualCount: 2; sex: female; **Location:** locationID: A1; continent: Europe; country: Spain; countryCode: ES; stateProvince: Catalonia; county: Lleida; locality: Sola de Boi; verbatimElevation: 1759.8; decimalLatitude: 42.54958; decimalLongitude: 0.87254; geodeticDatum: WGS84; **Event:** eventID: 2; samplingProtocol: Ground; eventTime: Night
- **Type status:** Other material. **Occurrence:** individualCount: 1; sex: female; **Location:** locationID: A1; continent: Europe; country: Spain; countryCode: ES; stateProvince: Catalonia; county: Lleida; locality: Sola de Boi; verbatimElevation: 1759.8; decimalLatitude: 42.54958; decimalLongitude: 0.87254; geodeticDatum: WGS84; **Event:** eventID: 1; samplingProtocol: Sweeping; eventTime: Night
- **Type status:** Other material. **Occurrence:** individualCount: 1; sex: female; **Location:** locationID: A2; continent: Europe; country: Spain; countryCode: ES; stateProvince: Catalonia; county: Lleida; locality: Sola de Boi; verbatimElevation: 1738.7; decimalLatitude: 42.54913; decimalLongitude: 0.87137; geodeticDatum: WGS84; **Event:** eventID: 2; samplingProtocol: Aerial; eventTime: Night
- **Type status:** Other material. **Occurrence:** individualCount: 1; sex: female; **Location:** locationID: P4; continent: Europe; country: Spain; countryCode: ES; stateProvince: Castilla y León; county: León; locality: El Canto; verbatimElevation: 943.48; decimalLatitude: 43.17227; decimalLongitude: -4.90857; geodeticDatum: WGS84; **Event:** eventID: 1; samplingProtocol: Aerial; eventTime: Night

##### Distribution

Holarctic

#### Lessertia dentichelis

(Simon, 1884)

##### Materials

- **Type status:** Other material. **Occurrence:** individualCount: 1; sex: male; **Location:** locationID: C4; continent: Europe; country: Spain; countryCode: ES; stateProvince: Castilla-La Mancha; county: Ciudad Real; locality: La Quesera; verbatimElevation: 772.3; decimalLatitude: 39.36337; decimalLongitude: -4.41704; geodeticDatum: WGS84; **Event:** eventID: C; samplingProtocol: Pitfall

##### Distribution

Europe, Canary Islands, Madeira, Canada, New Zealand

#### Linyphia hortensis

Sundevall, 1830

##### Materials

- **Type status:** Other material. **Occurrence:** individualCount: 1; sex: female; **Location:** locationID: A1; continent: Europe; country: Spain; countryCode: ES; stateProvince: Catalonia; county: Lleida; locality: Sola de Boi; verbatimElevation: 1759.8; decimalLatitude: 42.54958; decimalLongitude: 0.87254; geodeticDatum: WGS84; **Event:** eventID: 2; samplingProtocol: Ground; eventTime: Night
- **Type status:** Other material. **Occurrence:** individualCount: 1; sex: female; **Location:** locationID: A1; continent: Europe; country: Spain; countryCode: ES; stateProvince: Catalonia; county: Lleida; locality: Sola de Boi; verbatimElevation: 1759.8; decimalLatitude: 42.54958; decimalLongitude: 0.87254; geodeticDatum: WGS84; **Event:** eventID: 1; samplingProtocol: Sweeping; eventTime: Day
- **Type status:** Other material. **Occurrence:** individualCount: 2; sex: female; **Location:** locationID: A2; continent: Europe; country: Spain; countryCode: ES; stateProvince: Catalonia; county: Lleida; locality: Sola de Boi; verbatimElevation: 1738.7; decimalLatitude: 42.54913; decimalLongitude: 0.87137; geodeticDatum: WGS84; **Event:** eventID: 1; samplingProtocol: Sweeping; eventTime: Day
- **Type status:** Other material. **Occurrence:** individualCount: 1; sex: female; **Location:** locationID: P1; continent: Europe; country: Spain; countryCode: ES; stateProvince: Castilla y León; county: León; locality: Monte Robledo; verbatimElevation: 1071.58; decimalLatitude: 43.1445; decimalLongitude: -4.92675; geodeticDatum: WGS84; **Event:** eventID: 2; samplingProtocol: Aerial; eventTime: Night
- **Type status:** Other material. **Occurrence:** individualCount: 2; sex: female; **Location:** locationID: P1; continent: Europe; country: Spain; countryCode: ES; stateProvince: Castilla y León; county: León; locality: Monte Robledo; verbatimElevation: 1071.58; decimalLatitude: 43.1445; decimalLongitude: -4.92675; geodeticDatum: WGS84; **Event:** eventID: 2; samplingProtocol: Sweeping; eventTime: Day
- **Type status:** Other material. **Occurrence:** individualCount: 1; sex: male; **Location:** locationID: P2; continent: Europe; country: Spain; countryCode: ES; stateProvince: Castilla y León; county: León; locality: Joyoguelas; verbatimElevation: 763.98; decimalLatitude: 43.17771; decimalLongitude: -4.90579; geodeticDatum: WGS84; **Event:** eventID: K; samplingProtocol: Pitfall
- **Type status:** Other material. **Occurrence:** individualCount: 1; sex: female; **Location:** locationID: P2; continent: Europe; country: Spain; countryCode: ES; stateProvince: Castilla y León; county: León; locality: Joyoguelas; verbatimElevation: 763.98; decimalLatitude: 43.17771; decimalLongitude: -4.90579; geodeticDatum: WGS84; **Event:** eventID: 1; samplingProtocol: Sweeping; eventTime: Day
- **Type status:** Other material. **Occurrence:** individualCount: 1; sex: female; **Location:** locationID: P2; continent: Europe; country: Spain; countryCode: ES; stateProvince: Castilla y León; county: León; locality: Joyoguelas; verbatimElevation: 763.98; decimalLatitude: 43.17771; decimalLongitude: -4.90579; geodeticDatum: WGS84; **Event:** eventID: 2; samplingProtocol: Sweeping; eventTime: Day
- **Type status:** Other material. **Occurrence:** individualCount: 2; sex: male; **Location:** locationID: P2; continent: Europe; country: Spain; countryCode: ES; stateProvince: Castilla y León; county: León; locality: Joyoguelas; verbatimElevation: 763.98; decimalLatitude: 43.17771; decimalLongitude: -4.90579; geodeticDatum: WGS84; **Event:** eventID: 2; samplingProtocol: Sweeping; eventTime: Day
- **Type status:** Other material. **Occurrence:** individualCount: 1; sex: female; **Location:** locationID: P3; continent: Europe; country: Spain; countryCode: ES; stateProvince: Castilla y León; county: León; locality: Las Arroyas; verbatimElevation: 1097.1; decimalLatitude: 43.14351; decimalLongitude: -4.94878; geodeticDatum: WGS84; **Event:** eventID: 1; samplingProtocol: Sweeping; eventTime: Night
- **Type status:** Other material. **Occurrence:** individualCount: 2; sex: female; **Location:** locationID: P3; continent: Europe; country: Spain; countryCode: ES; stateProvince: Castilla y León; county: León; locality: Las Arroyas; verbatimElevation: 1097.1; decimalLatitude: 43.14351; decimalLongitude: -4.94878; geodeticDatum: WGS84; **Event:** eventID: 2; samplingProtocol: Sweeping; eventTime: Day

##### Distribution

Palearctic

#### Maso sundevalli

(Westring, 1851)

##### Materials

- **Type status:** Other material. **Occurrence:** individualCount: 1; sex: female; **Location:** locationID: P4; continent: Europe; country: Spain; countryCode: ES; stateProvince: Castilla y León; county: León; locality: El Canto; verbatimElevation: 943.48; decimalLatitude: 43.17227; decimalLongitude: -4.90857; geodeticDatum: WGS84; **Event:** eventID: 2; samplingProtocol: Beating; eventTime: Day
- **Type status:** Other material. **Occurrence:** individualCount: 1; sex: female; **Location:** locationID: P4; continent: Europe; country: Spain; countryCode: ES; stateProvince: Castilla y León; county: León; locality: El Canto; verbatimElevation: 943.48; decimalLatitude: 43.17227; decimalLongitude: -4.90857; geodeticDatum: WGS84; **Event:** eventID: 1; samplingProtocol: Sweeping; eventTime: Day
- **Type status:** Other material. **Occurrence:** individualCount: 6; sex: female; **Location:** locationID: P4; continent: Europe; country: Spain; countryCode: ES; stateProvince: Castilla y León; county: León; locality: El Canto; verbatimElevation: 943.48; decimalLatitude: 43.17227; decimalLongitude: -4.90857; geodeticDatum: WGS84; **Event:** eventID: 1; samplingProtocol: Sweeping; eventTime: Night
- **Type status:** Other material. **Occurrence:** individualCount: 1; sex: male; **Location:** locationID: P4; continent: Europe; country: Spain; countryCode: ES; stateProvince: Castilla y León; county: León; locality: El Canto; verbatimElevation: 943.48; decimalLatitude: 43.17227; decimalLongitude: -4.90857; geodeticDatum: WGS84; **Event:** eventID: 1; samplingProtocol: Sweeping; eventTime: Night
- **Type status:** Other material. **Occurrence:** individualCount: 2; sex: female; **Location:** locationID: P4; continent: Europe; country: Spain; countryCode: ES; stateProvince: Castilla y León; county: León; locality: El Canto; verbatimElevation: 943.48; decimalLatitude: 43.17227; decimalLongitude: -4.90857; geodeticDatum: WGS84; **Event:** eventID: 2; samplingProtocol: Sweeping; eventTime: Day

##### Distribution

Holarctic

#### Megalepthyphantes cf. collinus

(L. Koch, 1872)

##### Materials

- **Type status:** Other material. **Occurrence:** individualCount: 1; sex: female; **Location:** locationID: A2; continent: Europe; country: Spain; countryCode: ES; stateProvince: Catalonia; county: Lleida; locality: Sola de Boi; verbatimElevation: 1738.7; decimalLatitude: 42.54913; decimalLongitude: 0.87137; geodeticDatum: WGS84; **Event:** eventID: 1; samplingProtocol: Aerial; eventTime: Night
- **Type status:** Other material. **Occurrence:** individualCount: 1; sex: female; **Location:** locationID: A2; continent: Europe; country: Spain; countryCode: ES; stateProvince: Catalonia; county: Lleida; locality: Sola de Boi; verbatimElevation: 1738.7; decimalLatitude: 42.54913; decimalLongitude: 0.87137; geodeticDatum: WGS84; **Event:** eventID: 2; samplingProtocol: Aerial; eventTime: Night

##### Distribution

Europe

#### Metopobactrus prominulus

(O. Pickard-Cambridge, 1872)

##### Materials

- **Type status:** Other material. **Occurrence:** individualCount: 1; sex: male; **Location:** locationID: O1; continent: Europe; country: Spain; countryCode: ES; stateProvince: Aragón; county: Huesca; locality: O Furno; verbatimElevation: 1396.73; decimalLatitude: 42.60677; decimalLongitude: 0.13135; geodeticDatum: WGS84; **Event:** eventID: D; samplingProtocol: Pitfall
- **Type status:** Other material. **Occurrence:** individualCount: 1; sex: female; **Location:** locationID: P2; continent: Europe; country: Spain; countryCode: ES; stateProvince: Castilla y León; county: León; locality: Joyoguelas; verbatimElevation: 763.98; decimalLatitude: 43.17771; decimalLongitude: -4.90579; geodeticDatum: WGS84; **Event:** eventID: 1; samplingProtocol: Beating; eventTime: Night
- **Type status:** Other material. **Occurrence:** individualCount: 2; sex: female; **Location:** locationID: P2; continent: Europe; country: Spain; countryCode: ES; stateProvince: Castilla y León; county: León; locality: Joyoguelas; verbatimElevation: 763.98; decimalLatitude: 43.17771; decimalLongitude: -4.90579; geodeticDatum: WGS84; **Event:** eventID: C; samplingProtocol: Pitfall
- **Type status:** Other material. **Occurrence:** individualCount: 2; sex: male; **Location:** locationID: P2; continent: Europe; country: Spain; countryCode: ES; stateProvince: Castilla y León; county: León; locality: Joyoguelas; verbatimElevation: 763.98; decimalLatitude: 43.17771; decimalLongitude: -4.90579; geodeticDatum: WGS84; **Event:** eventID: C; samplingProtocol: Pitfall
- **Type status:** Other material. **Occurrence:** individualCount: 1; sex: male; **Location:** locationID: P2; continent: Europe; country: Spain; countryCode: ES; stateProvince: Castilla y León; county: León; locality: Joyoguelas; verbatimElevation: 763.98; decimalLatitude: 43.17771; decimalLongitude: -4.90579; geodeticDatum: WGS84; **Event:** eventID: F; samplingProtocol: Pitfall
- **Type status:** Other material. **Occurrence:** individualCount: 1; sex: male; **Location:** locationID: P2; continent: Europe; country: Spain; countryCode: ES; stateProvince: Castilla y León; county: León; locality: Joyoguelas; verbatimElevation: 763.98; decimalLatitude: 43.17771; decimalLongitude: -4.90579; geodeticDatum: WGS84; **Event:** eventID: I; samplingProtocol: Pitfall
- **Type status:** Other material. **Occurrence:** individualCount: 1; sex: female; **Location:** locationID: P2; continent: Europe; country: Spain; countryCode: ES; stateProvince: Castilla y León; county: León; locality: Joyoguelas; verbatimElevation: 763.98; decimalLatitude: 43.17771; decimalLongitude: -4.90579; geodeticDatum: WGS84; **Event:** eventID: J; samplingProtocol: Pitfall
- **Type status:** Other material. **Occurrence:** individualCount: 1; sex: male; **Location:** locationID: P3; continent: Europe; country: Spain; countryCode: ES; stateProvince: Castilla y León; county: León; locality: Las Arroyas; verbatimElevation: 1097.1; decimalLatitude: 43.14351; decimalLongitude: -4.94878; geodeticDatum: WGS84; **Event:** eventID: C; samplingProtocol: Pitfall
- **Type status:** Other material. **Occurrence:** individualCount: 1; sex: male; **Location:** locationID: P4; continent: Europe; country: Spain; countryCode: ES; stateProvince: Castilla y León; county: León; locality: El Canto; verbatimElevation: 943.48; decimalLatitude: 43.17227; decimalLongitude: -4.90857; geodeticDatum: WGS84; **Event:** eventID: H; samplingProtocol: Pitfall

##### Distribution

Holarctic

##### Notes

**Figure 7.**
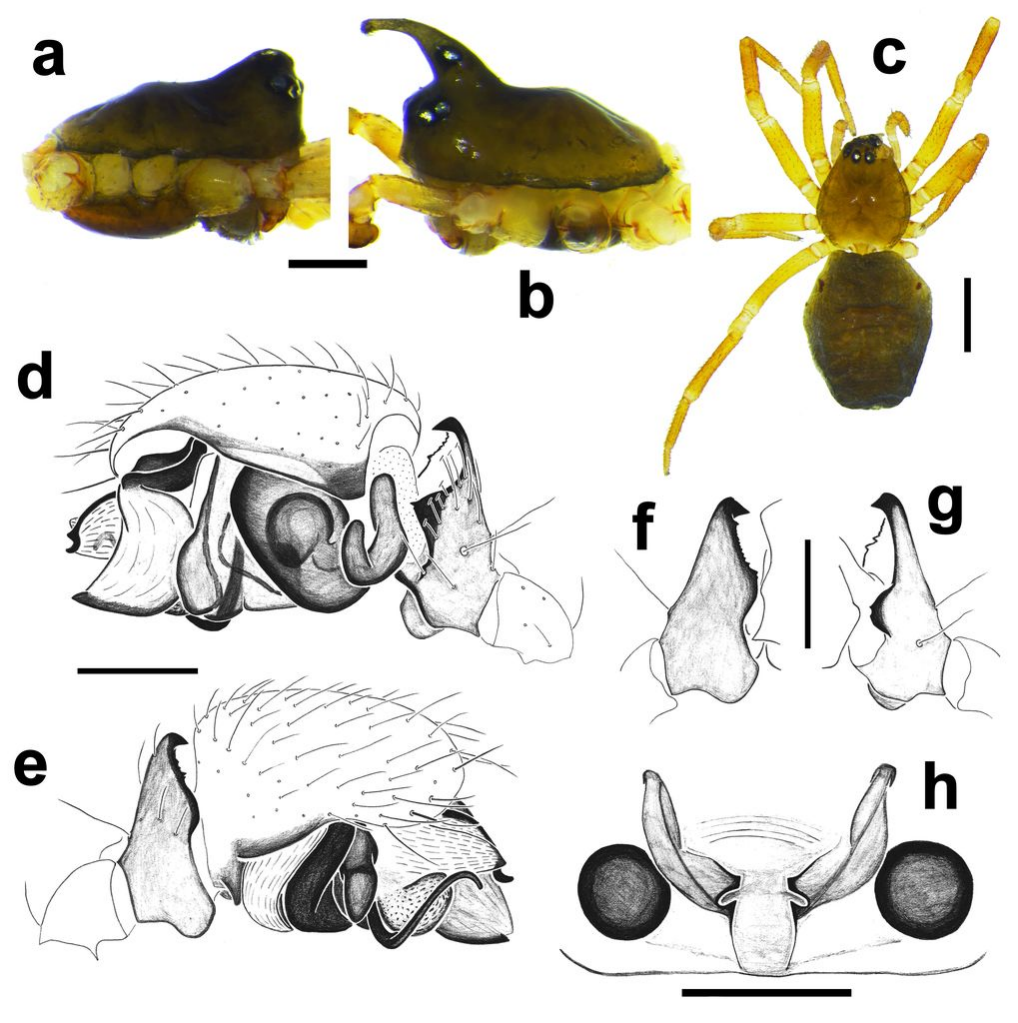
*Metopobactrus prominulus* (O. Pickard-Cambridge, 1872). a: male from Ordesa, lateral habitus, scale = 0.2 mm; b: male from Picos de Europa, lateral habitus, scale = 0.2 mm; c: female from Picos de Europa, dorsal habitus, scale = 0.5 mm; d: male from Ordesa, left palp, retrolateral, scale = 0.1 mm; d: male from Ordesa, left palp, prolateral, scale = 0.1 mm; e: male from Picos de Europa, left tibia, prolateral, scale = 0.1 mm; f: male from Picos de Europa, left tibia, retrolateral, scale = 0.1 mm; g: female from Picos de Europa, epigynum, ventral, scale = 0.1 mm.

We have uncovered an unknown case of male dimorphism. See Fig. 7 and *Species delimitation and identification using DNA barcodes*.

#### Micrargus apertus

(O. Pickard-Cambridge, 1871)

##### Materials

- **Type status:** Other material. **Occurrence:** individualCount: 1; sex: male; **Location:** locationID: P3; continent: Europe; country: Spain; countryCode: ES; stateProvince: Castilla y León; county: León; locality: Las Arroyas; verbatimElevation: 1097.1; decimalLatitude: 43.14351; decimalLongitude: -4.94878; geodeticDatum: WGS84; **Event:** eventID: 1; samplingProtocol: Sweeping; eventTime: Night

##### Distribution

Palearctic

#### Micrargus laudatus

(O. Pickard-Cambridge, 1881)

##### Materials

- **Type status:** Other material. **Occurrence:** individualCount: 1; sex: female; **Location:** locationID: A1; continent: Europe; country: Spain; countryCode: ES; stateProvince: Catalonia; county: Lleida; locality: Sola de Boi; verbatimElevation: 1759.8; decimalLatitude: 42.54958; decimalLongitude: 0.87254; geodeticDatum: WGS84; **Event:** eventID: 1; samplingProtocol: Ground; eventTime: Night
- **Type status:** Other material. **Occurrence:** individualCount: 1; sex: male; **Location:** locationID: A1; continent: Europe; country: Spain; countryCode: ES; stateProvince: Catalonia; county: Lleida; locality: Sola de Boi; verbatimElevation: 1759.8; decimalLatitude: 42.54958; decimalLongitude: 0.87254; geodeticDatum: WGS84; **Event:** eventID: E; samplingProtocol: Pitfall
- **Type status:** Other material. **Occurrence:** individualCount: 1; sex: male; **Location:** locationID: A2; continent: Europe; country: Spain; countryCode: ES; stateProvince: Catalonia; county: Lleida; locality: Sola de Boi; verbatimElevation: 1738.7; decimalLatitude: 42.54913; decimalLongitude: 0.87137; geodeticDatum: WGS84; **Event:** eventID: E; samplingProtocol: Pitfall

##### Distribution

Europe

#### Microctenonyx subitaneus

(O. Pickard-Cambridge, 1875)

##### Materials

- **Type status:** Other material. **Occurrence:** individualCount: 1; sex: male; **Location:** locationID: C1; continent: Europe; country: Spain; countryCode: ES; stateProvince: Castilla-La Mancha; county: Ciudad Real; locality: Valle Brezoso; verbatimElevation: 756.56; decimalLatitude: 39.35663; decimalLongitude: -4.35912; geodeticDatum: WGS84; **Event:** eventID: A; samplingProtocol: Pitfall
- **Type status:** Other material. **Occurrence:** individualCount: 1; sex: male; **Location:** locationID: C1; continent: Europe; country: Spain; countryCode: ES; stateProvince: Castilla-La Mancha; county: Ciudad Real; locality: Valle Brezoso; verbatimElevation: 756.56; decimalLatitude: 39.35663; decimalLongitude: -4.35912; geodeticDatum: WGS84; **Event:** eventID: C; samplingProtocol: Pitfall
- **Type status:** Other material. **Occurrence:** individualCount: 1; sex: female; **Location:** locationID: C1; continent: Europe; country: Spain; countryCode: ES; stateProvince: Castilla-La Mancha; county: Ciudad Real; locality: Valle Brezoso; verbatimElevation: 756.56; decimalLatitude: 39.35663; decimalLongitude: -4.35912; geodeticDatum: WGS84; **Event:** eventID: C; samplingProtocol: Pitfall
- **Type status:** Other material. **Occurrence:** individualCount: 1; sex: female; **Location:** locationID: C1; continent: Europe; country: Spain; countryCode: ES; stateProvince: Castilla-La Mancha; county: Ciudad Real; locality: Valle Brezoso; verbatimElevation: 756.56; decimalLatitude: 39.35663; decimalLongitude: -4.35912; geodeticDatum: WGS84; **Event:** eventID: J; samplingProtocol: Pitfall
- **Type status:** Other material. **Occurrence:** individualCount: 1; sex: female; **Location:** locationID: C2; continent: Europe; country: Spain; countryCode: ES; stateProvince: Castilla-La Mancha; county: Ciudad Real; locality: Valle Brezoso; verbatimElevation: 739.31; decimalLatitude: 39.35159; decimalLongitude: -4.3589; geodeticDatum: WGS84; **Event:** eventID: F; samplingProtocol: Pitfall
- **Type status:** Other material. **Occurrence:** individualCount: 1; sex: female; **Location:** locationID: C2; continent: Europe; country: Spain; countryCode: ES; stateProvince: Castilla-La Mancha; county: Ciudad Real; locality: Valle Brezoso; verbatimElevation: 739.31; decimalLatitude: 39.35159; decimalLongitude: -4.3589; geodeticDatum: WGS84; **Event:** eventID: G; samplingProtocol: Pitfall
- **Type status:** Other material. **Occurrence:** individualCount: 1; sex: male; **Location:** locationID: C2; continent: Europe; country: Spain; countryCode: ES; stateProvince: Castilla-La Mancha; county: Ciudad Real; locality: Valle Brezoso; verbatimElevation: 739.31; decimalLatitude: 39.35159; decimalLongitude: -4.3589; geodeticDatum: WGS84; **Event:** eventID: H; samplingProtocol: Pitfall
- **Type status:** Other material. **Occurrence:** individualCount: 1; sex: male; **Location:** locationID: C2; continent: Europe; country: Spain; countryCode: ES; stateProvince: Castilla-La Mancha; county: Ciudad Real; locality: Valle Brezoso; verbatimElevation: 739.31; decimalLatitude: 39.35159; decimalLongitude: -4.3589; geodeticDatum: WGS84; **Event:** eventID: J; samplingProtocol: Pitfall
- **Type status:** Other material. **Occurrence:** individualCount: 1; sex: female; **Location:** locationID: C2; continent: Europe; country: Spain; countryCode: ES; stateProvince: Castilla-La Mancha; county: Ciudad Real; locality: Valle Brezoso; verbatimElevation: 739.31; decimalLatitude: 39.35159; decimalLongitude: -4.3589; geodeticDatum: WGS84; **Event:** eventID: J; samplingProtocol: Pitfall
- **Type status:** Other material. **Occurrence:** individualCount: 1; sex: male; **Location:** locationID: C2; continent: Europe; country: Spain; countryCode: ES; stateProvince: Castilla-La Mancha; county: Ciudad Real; locality: Valle Brezoso; verbatimElevation: 739.31; decimalLatitude: 39.35159; decimalLongitude: -4.3589; geodeticDatum: WGS84; **Event:** eventID: K; samplingProtocol: Pitfall
- **Type status:** Other material. **Occurrence:** individualCount: 2; sex: male; **Location:** locationID: C2; continent: Europe; country: Spain; countryCode: ES; stateProvince: Castilla-La Mancha; county: Ciudad Real; locality: Valle Brezoso; verbatimElevation: 739.31; decimalLatitude: 39.35159; decimalLongitude: -4.3589; geodeticDatum: WGS84; **Event:** eventID: L; samplingProtocol: Pitfall

##### Distribution

Holarctic (elsewhere, introduced)

#### Microlinyphia pusilla

(Sundevall, 1830)

##### Materials

- **Type status:** Other material. **Occurrence:** individualCount: 1; sex: female; **Location:** locationID: P4; continent: Europe; country: Spain; countryCode: ES; stateProvince: Castilla y León; county: León; locality: El Canto; verbatimElevation: 943.48; decimalLatitude: 43.17227; decimalLongitude: -4.90857; geodeticDatum: WGS84; **Event:** eventID: 2; samplingProtocol: Sweeping; eventTime: Day

##### Distribution

Holarctic

#### Microneta viaria

(Blackwall, 1841)

##### Materials

- **Type status:** Other material. **Occurrence:** individualCount: 1; sex: male; **Location:** locationID: A1; continent: Europe; country: Spain; countryCode: ES; stateProvince: Catalonia; county: Lleida; locality: Sola de Boi; verbatimElevation: 1759.8; decimalLatitude: 42.54958; decimalLongitude: 0.87254; geodeticDatum: WGS84; **Event:** eventID: A; samplingProtocol: Pitfall
- **Type status:** Other material. **Occurrence:** individualCount: 1; sex: male; **Location:** locationID: A1; continent: Europe; country: Spain; countryCode: ES; stateProvince: Catalonia; county: Lleida; locality: Sola de Boi; verbatimElevation: 1759.8; decimalLatitude: 42.54958; decimalLongitude: 0.87254; geodeticDatum: WGS84; **Event:** eventID: B; samplingProtocol: Pitfall
- **Type status:** Other material. **Occurrence:** individualCount: 1; sex: female; **Location:** locationID: A1; continent: Europe; country: Spain; countryCode: ES; stateProvince: Catalonia; county: Lleida; locality: Sola de Boi; verbatimElevation: 1759.8; decimalLatitude: 42.54958; decimalLongitude: 0.87254; geodeticDatum: WGS84; **Event:** eventID: B; samplingProtocol: Pitfall
- **Type status:** Other material. **Occurrence:** individualCount: 1; sex: male; **Location:** locationID: A1; continent: Europe; country: Spain; countryCode: ES; stateProvince: Catalonia; county: Lleida; locality: Sola de Boi; verbatimElevation: 1759.8; decimalLatitude: 42.54958; decimalLongitude: 0.87254; geodeticDatum: WGS84; **Event:** eventID: C; samplingProtocol: Pitfall
- **Type status:** Other material. **Occurrence:** individualCount: 2; sex: male; **Location:** locationID: A1; continent: Europe; country: Spain; countryCode: ES; stateProvince: Catalonia; county: Lleida; locality: Sola de Boi; verbatimElevation: 1759.8; decimalLatitude: 42.54958; decimalLongitude: 0.87254; geodeticDatum: WGS84; **Event:** eventID: D; samplingProtocol: Pitfall
- **Type status:** Other material. **Occurrence:** individualCount: 1; sex: female; **Location:** locationID: A1; continent: Europe; country: Spain; countryCode: ES; stateProvince: Catalonia; county: Lleida; locality: Sola de Boi; verbatimElevation: 1759.8; decimalLatitude: 42.54958; decimalLongitude: 0.87254; geodeticDatum: WGS84; **Event:** eventID: D; samplingProtocol: Pitfall
- **Type status:** Other material. **Occurrence:** individualCount: 1; sex: female; **Location:** locationID: A1; continent: Europe; country: Spain; countryCode: ES; stateProvince: Catalonia; county: Lleida; locality: Sola de Boi; verbatimElevation: 1759.8; decimalLatitude: 42.54958; decimalLongitude: 0.87254; geodeticDatum: WGS84; **Event:** eventID: E; samplingProtocol: Pitfall
- **Type status:** Other material. **Occurrence:** individualCount: 1; sex: male; **Location:** locationID: A1; continent: Europe; country: Spain; countryCode: ES; stateProvince: Catalonia; county: Lleida; locality: Sola de Boi; verbatimElevation: 1759.8; decimalLatitude: 42.54958; decimalLongitude: 0.87254; geodeticDatum: WGS84; **Event:** eventID: I; samplingProtocol: Pitfall
- **Type status:** Other material. **Occurrence:** individualCount: 1; sex: female; **Location:** locationID: A1; continent: Europe; country: Spain; countryCode: ES; stateProvince: Catalonia; county: Lleida; locality: Sola de Boi; verbatimElevation: 1759.8; decimalLatitude: 42.54958; decimalLongitude: 0.87254; geodeticDatum: WGS84; **Event:** eventID: I; samplingProtocol: Pitfall
- **Type status:** Other material. **Occurrence:** individualCount: 1; sex: female; **Location:** locationID: A1; continent: Europe; country: Spain; countryCode: ES; stateProvince: Catalonia; county: Lleida; locality: Sola de Boi; verbatimElevation: 1759.8; decimalLatitude: 42.54958; decimalLongitude: 0.87254; geodeticDatum: WGS84; **Event:** eventID: J; samplingProtocol: Pitfall
- **Type status:** Other material. **Occurrence:** individualCount: 1; sex: male; **Location:** locationID: A2; continent: Europe; country: Spain; countryCode: ES; stateProvince: Catalonia; county: Lleida; locality: Sola de Boi; verbatimElevation: 1738.7; decimalLatitude: 42.54913; decimalLongitude: 0.87137; geodeticDatum: WGS84; **Event:** eventID: A; samplingProtocol: Pitfall
- **Type status:** Other material. **Occurrence:** individualCount: 2; sex: male; **Location:** locationID: A2; continent: Europe; country: Spain; countryCode: ES; stateProvince: Catalonia; county: Lleida; locality: Sola de Boi; verbatimElevation: 1738.7; decimalLatitude: 42.54913; decimalLongitude: 0.87137; geodeticDatum: WGS84; **Event:** eventID: B; samplingProtocol: Pitfall
- **Type status:** Other material. **Occurrence:** individualCount: 1; sex: male; **Location:** locationID: A2; continent: Europe; country: Spain; countryCode: ES; stateProvince: Catalonia; county: Lleida; locality: Sola de Boi; verbatimElevation: 1738.7; decimalLatitude: 42.54913; decimalLongitude: 0.87137; geodeticDatum: WGS84; **Event:** eventID: C; samplingProtocol: Pitfall
- **Type status:** Other material. **Occurrence:** individualCount: 2; sex: male; **Location:** locationID: A2; continent: Europe; country: Spain; countryCode: ES; stateProvince: Catalonia; county: Lleida; locality: Sola de Boi; verbatimElevation: 1738.7; decimalLatitude: 42.54913; decimalLongitude: 0.87137; geodeticDatum: WGS84; **Event:** eventID: D; samplingProtocol: Pitfall
- **Type status:** Other material. **Occurrence:** individualCount: 1; sex: male; **Location:** locationID: A2; continent: Europe; country: Spain; countryCode: ES; stateProvince: Catalonia; county: Lleida; locality: Sola de Boi; verbatimElevation: 1738.7; decimalLatitude: 42.54913; decimalLongitude: 0.87137; geodeticDatum: WGS84; **Event:** eventID: F; samplingProtocol: Pitfall
- **Type status:** Other material. **Occurrence:** individualCount: 2; sex: female; **Location:** locationID: A2; continent: Europe; country: Spain; countryCode: ES; stateProvince: Catalonia; county: Lleida; locality: Sola de Boi; verbatimElevation: 1738.7; decimalLatitude: 42.54913; decimalLongitude: 0.87137; geodeticDatum: WGS84; **Event:** eventID: F; samplingProtocol: Pitfall
- **Type status:** Other material. **Occurrence:** individualCount: 1; sex: male; **Location:** locationID: A2; continent: Europe; country: Spain; countryCode: ES; stateProvince: Catalonia; county: Lleida; locality: Sola de Boi; verbatimElevation: 1738.7; decimalLatitude: 42.54913; decimalLongitude: 0.87137; geodeticDatum: WGS84; **Event:** eventID: G; samplingProtocol: Pitfall
- **Type status:** Other material. **Occurrence:** individualCount: 1; sex: female; **Location:** locationID: A2; continent: Europe; country: Spain; countryCode: ES; stateProvince: Catalonia; county: Lleida; locality: Sola de Boi; verbatimElevation: 1738.7; decimalLatitude: 42.54913; decimalLongitude: 0.87137; geodeticDatum: WGS84; **Event:** eventID: G; samplingProtocol: Pitfall
- **Type status:** Other material. **Occurrence:** individualCount: 4; sex: male; **Location:** locationID: O1; continent: Europe; country: Spain; countryCode: ES; stateProvince: Aragón; county: Huesca; locality: O Furno; verbatimElevation: 1396.73; decimalLatitude: 42.60677; decimalLongitude: 0.13135; geodeticDatum: WGS84; **Event:** eventID: A; samplingProtocol: Pitfall
- **Type status:** Other material. **Occurrence:** individualCount: 3; sex: female; **Location:** locationID: O1; continent: Europe; country: Spain; countryCode: ES; stateProvince: Aragón; county: Huesca; locality: O Furno; verbatimElevation: 1396.73; decimalLatitude: 42.60677; decimalLongitude: 0.13135; geodeticDatum: WGS84; **Event:** eventID: A; samplingProtocol: Pitfall
- **Type status:** Other material. **Occurrence:** individualCount: 1; sex: male; **Location:** locationID: O1; continent: Europe; country: Spain; countryCode: ES; stateProvince: Aragón; county: Huesca; locality: O Furno; verbatimElevation: 1396.73; decimalLatitude: 42.60677; decimalLongitude: 0.13135; geodeticDatum: WGS84; **Event:** eventID: C; samplingProtocol: Pitfall
- **Type status:** Other material. **Occurrence:** individualCount: 1; sex: male; **Location:** locationID: O1; continent: Europe; country: Spain; countryCode: ES; stateProvince: Aragón; county: Huesca; locality: O Furno; verbatimElevation: 1396.73; decimalLatitude: 42.60677; decimalLongitude: 0.13135; geodeticDatum: WGS84; **Event:** eventID: E; samplingProtocol: Pitfall
- **Type status:** Other material. **Occurrence:** individualCount: 3; sex: female; **Location:** locationID: O1; continent: Europe; country: Spain; countryCode: ES; stateProvince: Aragón; county: Huesca; locality: O Furno; verbatimElevation: 1396.73; decimalLatitude: 42.60677; decimalLongitude: 0.13135; geodeticDatum: WGS84; **Event:** eventID: E; samplingProtocol: Pitfall
- **Type status:** Other material. **Occurrence:** individualCount: 2; sex: male; **Location:** locationID: O1; continent: Europe; country: Spain; countryCode: ES; stateProvince: Aragón; county: Huesca; locality: O Furno; verbatimElevation: 1396.73; decimalLatitude: 42.60677; decimalLongitude: 0.13135; geodeticDatum: WGS84; **Event:** eventID: H; samplingProtocol: Pitfall
- **Type status:** Other material. **Occurrence:** individualCount: 2; sex: male; **Location:** locationID: O1; continent: Europe; country: Spain; countryCode: ES; stateProvince: Aragón; county: Huesca; locality: O Furno; verbatimElevation: 1396.73; decimalLatitude: 42.60677; decimalLongitude: 0.13135; geodeticDatum: WGS84; **Event:** eventID: J; samplingProtocol: Pitfall
- **Type status:** Other material. **Occurrence:** individualCount: 3; sex: male; **Location:** locationID: O1; continent: Europe; country: Spain; countryCode: ES; stateProvince: Aragón; county: Huesca; locality: O Furno; verbatimElevation: 1396.73; decimalLatitude: 42.60677; decimalLongitude: 0.13135; geodeticDatum: WGS84; **Event:** eventID: K; samplingProtocol: Pitfall
- **Type status:** Other material. **Occurrence:** individualCount: 1; sex: female; **Location:** locationID: O1; continent: Europe; country: Spain; countryCode: ES; stateProvince: Aragón; county: Huesca; locality: O Furno; verbatimElevation: 1396.73; decimalLatitude: 42.60677; decimalLongitude: 0.13135; geodeticDatum: WGS84; **Event:** eventID: L; samplingProtocol: Pitfall
- **Type status:** Other material. **Occurrence:** individualCount: 2; sex: male; **Location:** locationID: O2; continent: Europe; country: Spain; countryCode: ES; stateProvince: Aragón; county: Huesca; locality: Rebilla; verbatimElevation: 1158.13; decimalLatitude: 42.59427; decimalLongitude: 0.1529; geodeticDatum: WGS84; **Event:** eventID: I; samplingProtocol: Pitfall
- **Type status:** Other material. **Occurrence:** individualCount: 2; sex: female; **Location:** locationID: O2; continent: Europe; country: Spain; countryCode: ES; stateProvince: Aragón; county: Huesca; locality: Rebilla; verbatimElevation: 1158.13; decimalLatitude: 42.59427; decimalLongitude: 0.1529; geodeticDatum: WGS84; **Event:** eventID: I; samplingProtocol: Pitfall
- **Type status:** Other material. **Occurrence:** individualCount: 1; sex: female; **Location:** locationID: O2; continent: Europe; country: Spain; countryCode: ES; stateProvince: Aragón; county: Huesca; locality: Rebilla; verbatimElevation: 1158.13; decimalLatitude: 42.59427; decimalLongitude: 0.1529; geodeticDatum: WGS84; **Event:** eventID: J; samplingProtocol: Pitfall
- **Type status:** Other material. **Occurrence:** individualCount: 1; sex: female; **Location:** locationID: P1; continent: Europe; country: Spain; countryCode: ES; stateProvince: Castilla y León; county: León; locality: Monte Robledo; verbatimElevation: 1071.58; decimalLatitude: 43.1445; decimalLongitude: -4.92675; geodeticDatum: WGS84; **Event:** eventID: B; samplingProtocol: Pitfall
- **Type status:** Other material. **Occurrence:** individualCount: 1; sex: female; **Location:** locationID: P1; continent: Europe; country: Spain; countryCode: ES; stateProvince: Castilla y León; county: León; locality: Monte Robledo; verbatimElevation: 1071.58; decimalLatitude: 43.1445; decimalLongitude: -4.92675; geodeticDatum: WGS84; **Event:** eventID: G; samplingProtocol: Pitfall
- **Type status:** Other material. **Occurrence:** individualCount: 1; sex: female; **Location:** locationID: P4; continent: Europe; country: Spain; countryCode: ES; stateProvince: Castilla y León; county: León; locality: El Canto; verbatimElevation: 943.48; decimalLatitude: 43.17227; decimalLongitude: -4.90857; geodeticDatum: WGS84; **Event:** eventID: J; samplingProtocol: Pitfall
- **Type status:** Other material. **Occurrence:** individualCount: 1; sex: female; **Location:** locationID: P4; continent: Europe; country: Spain; countryCode: ES; stateProvince: Castilla y León; county: León; locality: El Canto; verbatimElevation: 943.48; decimalLatitude: 43.17227; decimalLongitude: -4.90857; geodeticDatum: WGS84; **Event:** eventID: K; samplingProtocol: Pitfall
- **Type status:** Other material. **Occurrence:** individualCount: 1; sex: female; **Location:** locationID: P4; continent: Europe; country: Spain; countryCode: ES; stateProvince: Castilla y León; county: León; locality: El Canto; verbatimElevation: 943.48; decimalLatitude: 43.17227; decimalLongitude: -4.90857; geodeticDatum: WGS84; **Event:** eventID: L; samplingProtocol: Pitfall

##### Distribution

Holarctic

#### Midia midas

(Simon, 1884)

##### Materials

- **Type status:** Other material. **Occurrence:** individualCount: 1; sex: female; **Location:** locationID: P3; continent: Europe; country: Spain; countryCode: ES; stateProvince: Castilla y León; county: León; locality: Las Arroyas; verbatimElevation: 1097.1; decimalLatitude: 43.14351; decimalLongitude: -4.94878; geodeticDatum: WGS84; **Event:** eventID: 2; samplingProtocol: Aerial; eventTime: Night

##### Distribution

Europe

#### Minicia marginella

(Wider, 1834)

##### Materials

- **Type status:** Other material. **Occurrence:** individualCount: 1; sex: female; **Location:** locationID: O1; continent: Europe; country: Spain; countryCode: ES; stateProvince: Aragón; county: Huesca; locality: O Furno; verbatimElevation: 1396.73; decimalLatitude: 42.60677; decimalLongitude: 0.13135; geodeticDatum: WGS84; **Event:** eventID: 1; samplingProtocol: Sweeping; eventTime: Day
- **Type status:** Other material. **Occurrence:** individualCount: 2; sex: female; **Location:** locationID: O1; continent: Europe; country: Spain; countryCode: ES; stateProvince: Aragón; county: Huesca; locality: O Furno; verbatimElevation: 1396.73; decimalLatitude: 42.60677; decimalLongitude: 0.13135; geodeticDatum: WGS84; **Event:** eventID: 2; samplingProtocol: Sweeping; eventTime: Day
- **Type status:** Other material. **Occurrence:** individualCount: 1; sex: female; **Location:** locationID: O2; continent: Europe; country: Spain; countryCode: ES; stateProvince: Aragón; county: Huesca; locality: Rebilla; verbatimElevation: 1158.13; decimalLatitude: 42.59427; decimalLongitude: 0.1529; geodeticDatum: WGS84; **Event:** eventID: 1; samplingProtocol: Sweeping; eventTime: Night
- **Type status:** Other material. **Occurrence:** individualCount: 1; sex: female; **Location:** locationID: O2; continent: Europe; country: Spain; countryCode: ES; stateProvince: Aragón; county: Huesca; locality: Rebilla; verbatimElevation: 1158.13; decimalLatitude: 42.59427; decimalLongitude: 0.1529; geodeticDatum: WGS84; **Event:** eventID: 1; samplingProtocol: Sweeping; eventTime: Night

##### Distribution

Palearctic

#### Monocephalus fuscipes

(Blackwall, 1836)

##### Materials

- **Type status:** Other material. **Occurrence:** individualCount: 7; sex: male; **Location:** locationID: O1; continent: Europe; country: Spain; countryCode: ES; stateProvince: Aragón; county: Huesca; locality: O Furno; verbatimElevation: 1396.73; decimalLatitude: 42.60677; decimalLongitude: 0.13135; geodeticDatum: WGS84; **Event:** eventID: A; samplingProtocol: Pitfall
- **Type status:** Other material. **Occurrence:** individualCount: 20; sex: female; **Location:** locationID: O1; continent: Europe; country: Spain; countryCode: ES; stateProvince: Aragón; county: Huesca; locality: O Furno; verbatimElevation: 1396.73; decimalLatitude: 42.60677; decimalLongitude: 0.13135; geodeticDatum: WGS84; **Event:** eventID: A; samplingProtocol: Pitfall
- **Type status:** Other material. **Occurrence:** individualCount: 1; sex: female; **Location:** locationID: O1; continent: Europe; country: Spain; countryCode: ES; stateProvince: Aragón; county: Huesca; locality: O Furno; verbatimElevation: 1396.73; decimalLatitude: 42.60677; decimalLongitude: 0.13135; geodeticDatum: WGS84; **Event:** eventID: B; samplingProtocol: Pitfall
- **Type status:** Other material. **Occurrence:** individualCount: 1; sex: male; **Location:** locationID: O1; continent: Europe; country: Spain; countryCode: ES; stateProvince: Aragón; county: Huesca; locality: O Furno; verbatimElevation: 1396.73; decimalLatitude: 42.60677; decimalLongitude: 0.13135; geodeticDatum: WGS84; **Event:** eventID: D; samplingProtocol: Pitfall
- **Type status:** Other material. **Occurrence:** individualCount: 12; sex: male; **Location:** locationID: O1; continent: Europe; country: Spain; countryCode: ES; stateProvince: Aragón; county: Huesca; locality: O Furno; verbatimElevation: 1396.73; decimalLatitude: 42.60677; decimalLongitude: 0.13135; geodeticDatum: WGS84; **Event:** eventID: E; samplingProtocol: Pitfall
- **Type status:** Other material. **Occurrence:** individualCount: 13; sex: female; **Location:** locationID: O1; continent: Europe; country: Spain; countryCode: ES; stateProvince: Aragón; county: Huesca; locality: O Furno; verbatimElevation: 1396.73; decimalLatitude: 42.60677; decimalLongitude: 0.13135; geodeticDatum: WGS84; **Event:** eventID: E; samplingProtocol: Pitfall
- **Type status:** Other material. **Occurrence:** individualCount: 1; sex: male; **Location:** locationID: O1; continent: Europe; country: Spain; countryCode: ES; stateProvince: Aragón; county: Huesca; locality: O Furno; verbatimElevation: 1396.73; decimalLatitude: 42.60677; decimalLongitude: 0.13135; geodeticDatum: WGS84; **Event:** eventID: F; samplingProtocol: Pitfall
- **Type status:** Other material. **Occurrence:** individualCount: 1; sex: male; **Location:** locationID: O1; continent: Europe; country: Spain; countryCode: ES; stateProvince: Aragón; county: Huesca; locality: O Furno; verbatimElevation: 1396.73; decimalLatitude: 42.60677; decimalLongitude: 0.13135; geodeticDatum: WGS84; **Event:** eventID: G; samplingProtocol: Pitfall
- **Type status:** Other material. **Occurrence:** individualCount: 15; sex: male; **Location:** locationID: O1; continent: Europe; country: Spain; countryCode: ES; stateProvince: Aragón; county: Huesca; locality: O Furno; verbatimElevation: 1396.73; decimalLatitude: 42.60677; decimalLongitude: 0.13135; geodeticDatum: WGS84; **Event:** eventID: I; samplingProtocol: Pitfall
- **Type status:** Other material. **Occurrence:** individualCount: 13; sex: male; **Location:** locationID: O1; continent: Europe; country: Spain; countryCode: ES; stateProvince: Aragón; county: Huesca; locality: O Furno; verbatimElevation: 1396.73; decimalLatitude: 42.60677; decimalLongitude: 0.13135; geodeticDatum: WGS84; **Event:** eventID: I; samplingProtocol: Pitfall
- **Type status:** Other material. **Occurrence:** individualCount: 3; sex: male; **Location:** locationID: O1; continent: Europe; country: Spain; countryCode: ES; stateProvince: Aragón; county: Huesca; locality: O Furno; verbatimElevation: 1396.73; decimalLatitude: 42.60677; decimalLongitude: 0.13135; geodeticDatum: WGS84; **Event:** eventID: J; samplingProtocol: Pitfall
- **Type status:** Other material. **Occurrence:** individualCount: 4; sex: male; **Location:** locationID: O1; continent: Europe; country: Spain; countryCode: ES; stateProvince: Aragón; county: Huesca; locality: O Furno; verbatimElevation: 1396.73; decimalLatitude: 42.60677; decimalLongitude: 0.13135; geodeticDatum: WGS84; **Event:** eventID: J; samplingProtocol: Pitfall
- **Type status:** Other material. **Occurrence:** individualCount: 5; sex: male; **Location:** locationID: O1; continent: Europe; country: Spain; countryCode: ES; stateProvince: Aragón; county: Huesca; locality: O Furno; verbatimElevation: 1396.73; decimalLatitude: 42.60677; decimalLongitude: 0.13135; geodeticDatum: WGS84; **Event:** eventID: K; samplingProtocol: Pitfall
- **Type status:** Other material. **Occurrence:** individualCount: 2; sex: female; **Location:** locationID: O1; continent: Europe; country: Spain; countryCode: ES; stateProvince: Aragón; county: Huesca; locality: O Furno; verbatimElevation: 1396.73; decimalLatitude: 42.60677; decimalLongitude: 0.13135; geodeticDatum: WGS84; **Event:** eventID: K; samplingProtocol: Pitfall
- **Type status:** Other material. **Occurrence:** individualCount: 2; sex: male; **Location:** locationID: O1; continent: Europe; country: Spain; countryCode: ES; stateProvince: Aragón; county: Huesca; locality: O Furno; verbatimElevation: 1396.73; decimalLatitude: 42.60677; decimalLongitude: 0.13135; geodeticDatum: WGS84; **Event:** eventID: L; samplingProtocol: Pitfall
- **Type status:** Other material. **Occurrence:** individualCount: 2; sex: female; **Location:** locationID: O1; continent: Europe; country: Spain; countryCode: ES; stateProvince: Aragón; county: Huesca; locality: O Furno; verbatimElevation: 1396.73; decimalLatitude: 42.60677; decimalLongitude: 0.13135; geodeticDatum: WGS84; **Event:** eventID: L; samplingProtocol: Pitfall
- **Type status:** Other material. **Occurrence:** individualCount: 1; sex: male; **Location:** locationID: P1; continent: Europe; country: Spain; countryCode: ES; stateProvince: Castilla y León; county: León; locality: Monte Robledo; verbatimElevation: 1071.58; decimalLatitude: 43.1445; decimalLongitude: -4.92675; geodeticDatum: WGS84; **Event:** eventID: G; samplingProtocol: Pitfall
- **Type status:** Other material. **Occurrence:** individualCount: 2; sex: male; **Location:** locationID: P1; continent: Europe; country: Spain; countryCode: ES; stateProvince: Castilla y León; county: León; locality: Monte Robledo; verbatimElevation: 1071.58; decimalLatitude: 43.1445; decimalLongitude: -4.92675; geodeticDatum: WGS84; **Event:** eventID: J; samplingProtocol: Pitfall
- **Type status:** Other material. **Occurrence:** individualCount: 3; sex: female; **Location:** locationID: P1; continent: Europe; country: Spain; countryCode: ES; stateProvince: Castilla y León; county: León; locality: Monte Robledo; verbatimElevation: 1071.58; decimalLatitude: 43.1445; decimalLongitude: -4.92675; geodeticDatum: WGS84; **Event:** eventID: J; samplingProtocol: Pitfall
- **Type status:** Other material. **Occurrence:** individualCount: 1; sex: female; **Location:** locationID: P1; continent: Europe; country: Spain; countryCode: ES; stateProvince: Castilla y León; county: León; locality: Monte Robledo; verbatimElevation: 1071.58; decimalLatitude: 43.1445; decimalLongitude: -4.92675; geodeticDatum: WGS84; **Event:** eventID: K; samplingProtocol: Pitfall
- **Type status:** Other material. **Occurrence:** individualCount: 1; sex: male; **Location:** locationID: P1; continent: Europe; country: Spain; countryCode: ES; stateProvince: Castilla y León; county: León; locality: Monte Robledo; verbatimElevation: 1071.58; decimalLatitude: 43.1445; decimalLongitude: -4.92675; geodeticDatum: WGS84; **Event:** eventID: L; samplingProtocol: Pitfall
- **Type status:** Other material. **Occurrence:** individualCount: 2; sex: female; **Location:** locationID: P1; continent: Europe; country: Spain; countryCode: ES; stateProvince: Castilla y León; county: León; locality: Monte Robledo; verbatimElevation: 1071.58; decimalLatitude: 43.1445; decimalLongitude: -4.92675; geodeticDatum: WGS84; **Event:** eventID: L; samplingProtocol: Pitfall

##### Distribution

Europe

#### Neriene clathrata

(Sundevall, 1830)

##### Materials

- **Type status:** Other material. **Occurrence:** individualCount: 1; sex: female; **Location:** locationID: P2; continent: Europe; country: Spain; countryCode: ES; stateProvince: Castilla y León; county: León; locality: Joyoguelas; verbatimElevation: 763.98; decimalLatitude: 43.17771; decimalLongitude: -4.90579; geodeticDatum: WGS84; **Event:** eventID: 2; samplingProtocol: Aerial; eventTime: Night
- **Type status:** Other material. **Occurrence:** individualCount: 1; sex: female; **Location:** locationID: P2; continent: Europe; country: Spain; countryCode: ES; stateProvince: Castilla y León; county: León; locality: Joyoguelas; verbatimElevation: 763.98; decimalLatitude: 43.17771; decimalLongitude: -4.90579; geodeticDatum: WGS84; **Event:** eventID: C; samplingProtocol: Pitfall
- **Type status:** Other material. **Occurrence:** individualCount: 1; sex: female; **Location:** locationID: P4; continent: Europe; country: Spain; countryCode: ES; stateProvince: Castilla y León; county: León; locality: El Canto; verbatimElevation: 943.48; decimalLatitude: 43.17227; decimalLongitude: -4.90857; geodeticDatum: WGS84; **Event:** eventID: 1; samplingProtocol: Aerial; eventTime: Night

##### Distribution

Holarctic

#### Neriene peltata

(Wider, 1834)

##### Materials

- **Type status:** Other material. **Occurrence:** individualCount: 2; sex: female; **Location:** locationID: P1; continent: Europe; country: Spain; countryCode: ES; stateProvince: Castilla y León; county: León; locality: Monte Robledo; verbatimElevation: 1071.58; decimalLatitude: 43.1445; decimalLongitude: -4.92675; geodeticDatum: WGS84; **Event:** eventID: 1; samplingProtocol: Aerial; eventTime: Night
- **Type status:** Other material. **Occurrence:** individualCount: 1; sex: male; **Location:** locationID: P1; continent: Europe; country: Spain; countryCode: ES; stateProvince: Castilla y León; county: León; locality: Monte Robledo; verbatimElevation: 1071.58; decimalLatitude: 43.1445; decimalLongitude: -4.92675; geodeticDatum: WGS84; **Event:** eventID: 1; samplingProtocol: Aerial; eventTime: Night
- **Type status:** Other material. **Occurrence:** individualCount: 2; sex: female; **Location:** locationID: P1; continent: Europe; country: Spain; countryCode: ES; stateProvince: Castilla y León; county: León; locality: Monte Robledo; verbatimElevation: 1071.58; decimalLatitude: 43.1445; decimalLongitude: -4.92675; geodeticDatum: WGS84; **Event:** eventID: 2; samplingProtocol: Aerial; eventTime: Night
- **Type status:** Other material. **Occurrence:** individualCount: 1; sex: male; **Location:** locationID: P1; continent: Europe; country: Spain; countryCode: ES; stateProvince: Castilla y León; county: León; locality: Monte Robledo; verbatimElevation: 1071.58; decimalLatitude: 43.1445; decimalLongitude: -4.92675; geodeticDatum: WGS84; **Event:** eventID: 2; samplingProtocol: Aerial; eventTime: Night
- **Type status:** Other material. **Occurrence:** individualCount: 1; sex: female; **Location:** locationID: P1; continent: Europe; country: Spain; countryCode: ES; stateProvince: Castilla y León; county: León; locality: Monte Robledo; verbatimElevation: 1071.58; decimalLatitude: 43.1445; decimalLongitude: -4.92675; geodeticDatum: WGS84; **Event:** eventID: 1; samplingProtocol: Beating; eventTime: Night
- **Type status:** Other material. **Occurrence:** individualCount: 1; sex: female; **Location:** locationID: P1; continent: Europe; country: Spain; countryCode: ES; stateProvince: Castilla y León; county: León; locality: Monte Robledo; verbatimElevation: 1071.58; decimalLatitude: 43.1445; decimalLongitude: -4.92675; geodeticDatum: WGS84; **Event:** eventID: 1; samplingProtocol: Beating; eventTime: Night
- **Type status:** Other material. **Occurrence:** individualCount: 1; sex: female; **Location:** locationID: P1; continent: Europe; country: Spain; countryCode: ES; stateProvince: Castilla y León; county: León; locality: Monte Robledo; verbatimElevation: 1071.58; decimalLatitude: 43.1445; decimalLongitude: -4.92675; geodeticDatum: WGS84; **Event:** eventID: 2; samplingProtocol: Beating; eventTime: Day
- **Type status:** Other material. **Occurrence:** individualCount: 2; sex: female; **Location:** locationID: P1; continent: Europe; country: Spain; countryCode: ES; stateProvince: Castilla y León; county: León; locality: Monte Robledo; verbatimElevation: 1071.58; decimalLatitude: 43.1445; decimalLongitude: -4.92675; geodeticDatum: WGS84; **Event:** eventID: 1; samplingProtocol: Sweeping; eventTime: Day
- **Type status:** Other material. **Occurrence:** individualCount: 1; sex: female; **Location:** locationID: P1; continent: Europe; country: Spain; countryCode: ES; stateProvince: Castilla y León; county: León; locality: Monte Robledo; verbatimElevation: 1071.58; decimalLatitude: 43.1445; decimalLongitude: -4.92675; geodeticDatum: WGS84; **Event:** eventID: 1; samplingProtocol: Sweeping; eventTime: Night
- **Type status:** Other material. **Occurrence:** individualCount: 1; sex: female; **Location:** locationID: P1; continent: Europe; country: Spain; countryCode: ES; stateProvince: Castilla y León; county: León; locality: Monte Robledo; verbatimElevation: 1071.58; decimalLatitude: 43.1445; decimalLongitude: -4.92675; geodeticDatum: WGS84; **Event:** eventID: 1; samplingProtocol: Sweeping; eventTime: Night
- **Type status:** Other material. **Occurrence:** individualCount: 1; sex: female; **Location:** locationID: P1; continent: Europe; country: Spain; countryCode: ES; stateProvince: Castilla y León; county: León; locality: Monte Robledo; verbatimElevation: 1071.58; decimalLatitude: 43.1445; decimalLongitude: -4.92675; geodeticDatum: WGS84; **Event:** eventID: 2; samplingProtocol: Sweeping; eventTime: Day

##### Distribution

Greenland, Palearctic

#### Neriene radiata

(Walckenaer, 1841)

##### Materials

- **Type status:** Other material. **Occurrence:** individualCount: 1; sex: male; **Location:** locationID: A2; continent: Europe; country: Spain; countryCode: ES; stateProvince: Catalonia; county: Lleida; locality: Sola de Boi; verbatimElevation: 1738.7; decimalLatitude: 42.54913; decimalLongitude: 0.87137; geodeticDatum: WGS84; **Event:** eventID: 1; samplingProtocol: Sweeping; eventTime: Day
- **Type status:** Other material. **Occurrence:** individualCount: 1; sex: female; **Location:** locationID: A2; continent: Europe; country: Spain; countryCode: ES; stateProvince: Catalonia; county: Lleida; locality: Sola de Boi; verbatimElevation: 1738.7; decimalLatitude: 42.54913; decimalLongitude: 0.87137; geodeticDatum: WGS84; **Event:** eventID: 1; samplingProtocol: Sweeping; eventTime: Day
- **Type status:** Other material. **Occurrence:** individualCount: 1; sex: male; **Location:** locationID: A2; continent: Europe; country: Spain; countryCode: ES; stateProvince: Catalonia; county: Lleida; locality: Sola de Boi; verbatimElevation: 1738.7; decimalLatitude: 42.54913; decimalLongitude: 0.87137; geodeticDatum: WGS84; **Event:** eventID: 2; samplingProtocol: Sweeping; eventTime: Day
- **Type status:** Other material. **Occurrence:** individualCount: 2; sex: female; **Location:** locationID: O2; continent: Europe; country: Spain; countryCode: ES; stateProvince: Aragón; county: Huesca; locality: Rebilla; verbatimElevation: 1158.13; decimalLatitude: 42.59427; decimalLongitude: 0.1529; geodeticDatum: WGS84; **Event:** eventID: 1; samplingProtocol: Aerial; eventTime: Night
- **Type status:** Other material. **Occurrence:** individualCount: 4; sex: female; **Location:** locationID: O2; continent: Europe; country: Spain; countryCode: ES; stateProvince: Aragón; county: Huesca; locality: Rebilla; verbatimElevation: 1158.13; decimalLatitude: 42.59427; decimalLongitude: 0.1529; geodeticDatum: WGS84; **Event:** eventID: 2; samplingProtocol: Aerial; eventTime: Night
- **Type status:** Other material. **Occurrence:** individualCount: 1; sex: male; **Location:** locationID: O2; continent: Europe; country: Spain; countryCode: ES; stateProvince: Aragón; county: Huesca; locality: Rebilla; verbatimElevation: 1158.13; decimalLatitude: 42.59427; decimalLongitude: 0.1529; geodeticDatum: WGS84; **Event:** eventID: 1; samplingProtocol: Sweeping; eventTime: Day
- **Type status:** Other material. **Occurrence:** individualCount: 1; sex: female; **Location:** locationID: O2; continent: Europe; country: Spain; countryCode: ES; stateProvince: Aragón; county: Huesca; locality: Rebilla; verbatimElevation: 1158.13; decimalLatitude: 42.59427; decimalLongitude: 0.1529; geodeticDatum: WGS84; **Event:** eventID: 1; samplingProtocol: Sweeping; eventTime: Day
- **Type status:** Other material. **Occurrence:** individualCount: 1; sex: male; **Location:** locationID: O2; continent: Europe; country: Spain; countryCode: ES; stateProvince: Aragón; county: Huesca; locality: Rebilla; verbatimElevation: 1158.13; decimalLatitude: 42.59427; decimalLongitude: 0.1529; geodeticDatum: WGS84; **Event:** eventID: 2; samplingProtocol: Sweeping; eventTime: Day

##### Distribution

Holarctic

#### Obscuriphantes obscurus

(Blackwall, 1841)

##### Materials

- **Type status:** Other material. **Occurrence:** individualCount: 1; sex: female; **Location:** locationID: A1; continent: Europe; country: Spain; countryCode: ES; stateProvince: Catalonia; county: Lleida; locality: Sola de Boi; verbatimElevation: 1759.8; decimalLatitude: 42.54958; decimalLongitude: 0.87254; geodeticDatum: WGS84; **Event:** eventID: 1; samplingProtocol: Aerial; eventTime: Night
- **Type status:** Other material. **Occurrence:** individualCount: 1; sex: female; **Location:** locationID: O1; continent: Europe; country: Spain; countryCode: ES; stateProvince: Aragón; county: Huesca; locality: O Furno; verbatimElevation: 1396.73; decimalLatitude: 42.60677; decimalLongitude: 0.13135; geodeticDatum: WGS84; **Event:** eventID: 2; samplingProtocol: Aerial; eventTime: Night

##### Distribution

Palearctic

#### Oedothorax apicatus

(Blackwall, 1850)

##### Materials

- **Type status:** Other material. **Occurrence:** individualCount: 1; sex: male; **Location:** locationID: O2; continent: Europe; country: Spain; countryCode: ES; stateProvince: Aragón; county: Huesca; locality: Rebilla; verbatimElevation: 1158.13; decimalLatitude: 42.59427; decimalLongitude: 0.1529; geodeticDatum: WGS84; **Event:** eventID: 1; samplingProtocol: Beating; eventTime: Night

##### Distribution

Palearctic

#### Oedothorax fuscus

(Blackwall, 1834)

##### Materials

- **Type status:** Other material. **Occurrence:** individualCount: 1; sex: female; **Location:** locationID: M1; continent: Europe; country: Spain; countryCode: ES; stateProvince: Extremadura; county: Cáceres; locality: Peña Falcón; verbatimElevation: 320.6; decimalLatitude: 39.83296; decimalLongitude: -6.0641; geodeticDatum: WGS84; **Event:** eventID: H; samplingProtocol: Pitfall

##### Distribution

Europe, Mallorca, Azores, North Africa, Russia

#### Palliduphantes cernuus

(Simon, 1884)

##### Materials

- **Type status:** Other material. **Occurrence:** individualCount: 1; sex: male; **Location:** locationID: A2; continent: Europe; country: Spain; countryCode: ES; stateProvince: Catalonia; county: Lleida; locality: Sola de Boi; verbatimElevation: 1738.7; decimalLatitude: 42.54913; decimalLongitude: 0.87137; geodeticDatum: WGS84; **Event:** eventID: G; samplingProtocol: Pitfall
- **Type status:** Other material. **Occurrence:** individualCount: 1; sex: male; **Location:** locationID: A2; continent: Europe; country: Spain; countryCode: ES; stateProvince: Catalonia; county: Lleida; locality: Sola de Boi; verbatimElevation: 1738.7; decimalLatitude: 42.54913; decimalLongitude: 0.87137; geodeticDatum: WGS84; **Event:** eventID: 1; samplingProtocol: Sweeping; eventTime: Night
- **Type status:** Other material. **Occurrence:** individualCount: 2; sex: female; **Location:** locationID: O1; continent: Europe; country: Spain; countryCode: ES; stateProvince: Aragón; county: Huesca; locality: O Furno; verbatimElevation: 1396.73; decimalLatitude: 42.60677; decimalLongitude: 0.13135; geodeticDatum: WGS84; **Event:** eventID: A; samplingProtocol: Pitfall
- **Type status:** Other material. **Occurrence:** individualCount: 1; sex: male; **Location:** locationID: O1; continent: Europe; country: Spain; countryCode: ES; stateProvince: Aragón; county: Huesca; locality: O Furno; verbatimElevation: 1396.73; decimalLatitude: 42.60677; decimalLongitude: 0.13135; geodeticDatum: WGS84; **Event:** eventID: D; samplingProtocol: Pitfall
- **Type status:** Other material. **Occurrence:** individualCount: 1; sex: male; **Location:** locationID: O1; continent: Europe; country: Spain; countryCode: ES; stateProvince: Aragón; county: Huesca; locality: O Furno; verbatimElevation: 1396.73; decimalLatitude: 42.60677; decimalLongitude: 0.13135; geodeticDatum: WGS84; **Event:** eventID: L; samplingProtocol: Pitfall
- **Type status:** Other material. **Occurrence:** individualCount: 1; sex: female; **Location:** locationID: P4; continent: Europe; country: Spain; countryCode: ES; stateProvince: Castilla y León; county: León; locality: El Canto; verbatimElevation: 943.48; decimalLatitude: 43.17227; decimalLongitude: -4.90857; geodeticDatum: WGS84; **Event:** eventID: D; samplingProtocol: Pitfall
- **Type status:** Other material. **Occurrence:** individualCount: 1; sex: female; **Location:** locationID: P4; continent: Europe; country: Spain; countryCode: ES; stateProvince: Castilla y León; county: León; locality: El Canto; verbatimElevation: 943.48; decimalLatitude: 43.17227; decimalLongitude: -4.90857; geodeticDatum: WGS84; **Event:** eventID: F; samplingProtocol: Pitfall

##### Distribution

France, Spain

#### Palliduphantes sp20

##### Materials

- **Type status:** Other material. **Occurrence:** individualCount: 1; sex: female; **Location:** locationID: M1; continent: Europe; country: Spain; countryCode: ES; stateProvince: Extremadura; county: Cáceres; locality: Peña Falcón; verbatimElevation: 320.6; decimalLatitude: 39.83296; decimalLongitude: -6.0641; geodeticDatum: WGS84; **Event:** eventID: K; samplingProtocol: Pitfall

##### Distribution

?

##### Notes

This is a species of *Palliduphantes* Saaristo & Tanasevitch, 2001, which we were unable to identify.

#### Parapelecopsis nemoralis

(Blackwall, 1841)

##### Materials

- **Type status:** Other material. **Occurrence:** individualCount: 2; sex: male; **Location:** locationID: A1; continent: Europe; country: Spain; countryCode: ES; stateProvince: Catalonia; county: Lleida; locality: Sola de Boi; verbatimElevation: 1759.8; decimalLatitude: 42.54958; decimalLongitude: 0.87254; geodeticDatum: WGS84; **Event:** eventID: A; samplingProtocol: Pitfall
- **Type status:** Other material. **Occurrence:** individualCount: 1; sex: female; **Location:** locationID: A1; continent: Europe; country: Spain; countryCode: ES; stateProvince: Catalonia; county: Lleida; locality: Sola de Boi; verbatimElevation: 1759.8; decimalLatitude: 42.54958; decimalLongitude: 0.87254; geodeticDatum: WGS84; **Event:** eventID: A; samplingProtocol: Pitfall
- **Type status:** Other material. **Occurrence:** individualCount: 1; sex: male; **Location:** locationID: A2; continent: Europe; country: Spain; countryCode: ES; stateProvince: Catalonia; county: Lleida; locality: Sola de Boi; verbatimElevation: 1738.7; decimalLatitude: 42.54913; decimalLongitude: 0.87137; geodeticDatum: WGS84; **Event:** eventID: L; samplingProtocol: Pitfall
- **Type status:** Other material. **Occurrence:** individualCount: 2; sex: female; **Location:** locationID: M1; continent: Europe; country: Spain; countryCode: ES; stateProvince: Extremadura; county: Cáceres; locality: Peña Falcón; verbatimElevation: 320.6; decimalLatitude: 39.83296; decimalLongitude: -6.0641; geodeticDatum: WGS84; **Event:** eventID: H; samplingProtocol: Pitfall
- **Type status:** Other material. **Occurrence:** individualCount: 1; sex: female; **Location:** locationID: M1; continent: Europe; country: Spain; countryCode: ES; stateProvince: Extremadura; county: Cáceres; locality: Peña Falcón; verbatimElevation: 320.6; decimalLatitude: 39.83296; decimalLongitude: -6.0641; geodeticDatum: WGS84; **Event:** eventID: L; samplingProtocol: Pitfall

##### Distribution

Europe, Russia

#### Pelecopsis bucephala

(O. Pickard-Cambridge, 1875)

##### Materials

- **Type status:** Other material. **Occurrence:** individualCount: 1; sex: male; **Location:** locationID: C4; continent: Europe; country: Spain; countryCode: ES; stateProvince: Castilla-La Mancha; county: Ciudad Real; locality: La Quesera; verbatimElevation: 772.3; decimalLatitude: 39.36337; decimalLongitude: -4.41704; geodeticDatum: WGS84; **Event:** eventID: B; samplingProtocol: Pitfall
- **Type status:** Other material. **Occurrence:** individualCount: 1; sex: male; **Location:** locationID: C4; continent: Europe; country: Spain; countryCode: ES; stateProvince: Castilla-La Mancha; county: Ciudad Real; locality: La Quesera; verbatimElevation: 772.3; decimalLatitude: 39.36337; decimalLongitude: -4.41704; geodeticDatum: WGS84; **Event:** eventID: D; samplingProtocol: Pitfall

##### Distribution

Western Mediterranean

#### Pelecopsis cf. monsantensis

Bosmans & Crespo, 2010

##### Materials

- **Type status:** Other material. **Occurrence:** individualCount: 1; sex: female; **Location:** locationID: A1; continent: Europe; country: Spain; countryCode: ES; stateProvince: Catalonia; county: Lleida; locality: Sola de Boi; verbatimElevation: 1759.8; decimalLatitude: 42.54958; decimalLongitude: 0.87254; geodeticDatum: WGS84; **Event:** eventID: H; samplingProtocol: Pitfall
- **Type status:** Other material. **Occurrence:** individualCount: 1; sex: female; **Location:** locationID: A2; continent: Europe; country: Spain; countryCode: ES; stateProvince: Catalonia; county: Lleida; locality: Sola de Boi; verbatimElevation: 1738.7; decimalLatitude: 42.54913; decimalLongitude: 0.87137; geodeticDatum: WGS84; **Event:** eventID: C; samplingProtocol: Pitfall

##### Distribution

Andorra, France

##### Notes

Only the male of this species is known. In a previous work by the first author (unpublished data) from central Portugal, females such as the ones here recorded were found along with *P. monsantensis* males. We now extend the distribution of this species up north, to the Pyrenean region.

#### Piniphantes pinicola

(Simon, 1884)

##### Materials

- **Type status:** Other material. **Occurrence:** individualCount: 1; sex: female; **Location:** locationID: A1; continent: Europe; country: Spain; countryCode: ES; stateProvince: Catalonia; county: Lleida; locality: Sola de Boi; verbatimElevation: 1759.8; decimalLatitude: 42.54958; decimalLongitude: 0.87254; geodeticDatum: WGS84; **Event:** eventID: 2; samplingProtocol: Aerial; eventTime: Night
- **Type status:** Other material. **Occurrence:** individualCount: 1; sex: female; **Location:** locationID: A1; continent: Europe; country: Spain; countryCode: ES; stateProvince: Catalonia; county: Lleida; locality: Sola de Boi; verbatimElevation: 1759.8; decimalLatitude: 42.54958; decimalLongitude: 0.87254; geodeticDatum: WGS84; **Event:** eventID: B; samplingProtocol: Pitfall
- **Type status:** Other material. **Occurrence:** individualCount: 1; sex: female; **Location:** locationID: A1; continent: Europe; country: Spain; countryCode: ES; stateProvince: Catalonia; county: Lleida; locality: Sola de Boi; verbatimElevation: 1759.8; decimalLatitude: 42.54958; decimalLongitude: 0.87254; geodeticDatum: WGS84; **Event:** eventID: D; samplingProtocol: Pitfall
- **Type status:** Other material. **Occurrence:** individualCount: 2; sex: female; **Location:** locationID: A2; continent: Europe; country: Spain; countryCode: ES; stateProvince: Catalonia; county: Lleida; locality: Sola de Boi; verbatimElevation: 1738.7; decimalLatitude: 42.54913; decimalLongitude: 0.87137; geodeticDatum: WGS84; **Event:** eventID: B; samplingProtocol: Pitfall

##### Distribution

Palearctic

#### Pocadicnemis juncea

Locket & Millidge, 1953

##### Materials

- **Type status:** Other material. **Occurrence:** individualCount: 1; sex: male; **Location:** locationID: O1; continent: Europe; country: Spain; countryCode: ES; stateProvince: Aragón; county: Huesca; locality: O Furno; verbatimElevation: 1396.73; decimalLatitude: 42.60677; decimalLongitude: 0.13135; geodeticDatum: WGS84; **Event:** eventID: C; samplingProtocol: Pitfall
- **Type status:** Other material. **Occurrence:** individualCount: 2; sex: male; **Location:** locationID: O1; continent: Europe; country: Spain; countryCode: ES; stateProvince: Aragón; county: Huesca; locality: O Furno; verbatimElevation: 1396.73; decimalLatitude: 42.60677; decimalLongitude: 0.13135; geodeticDatum: WGS84; **Event:** eventID: D; samplingProtocol: Pitfall
- **Type status:** Other material. **Occurrence:** individualCount: 1; sex: male; **Location:** locationID: O1; continent: Europe; country: Spain; countryCode: ES; stateProvince: Aragón; county: Huesca; locality: O Furno; verbatimElevation: 1396.73; decimalLatitude: 42.60677; decimalLongitude: 0.13135; geodeticDatum: WGS84; **Event:** eventID: E; samplingProtocol: Pitfall
- **Type status:** Other material. **Occurrence:** individualCount: 1; sex: male; **Location:** locationID: O1; continent: Europe; country: Spain; countryCode: ES; stateProvince: Aragón; county: Huesca; locality: O Furno; verbatimElevation: 1396.73; decimalLatitude: 42.60677; decimalLongitude: 0.13135; geodeticDatum: WGS84; **Event:** eventID: G; samplingProtocol: Pitfall

##### Distribution

Europe

#### Pocadicnemis pumila

(Blackwall, 1841)

##### Materials

- **Type status:** Other material. **Occurrence:** individualCount: 1; sex: male; **Location:** locationID: O1; continent: Europe; country: Spain; countryCode: ES; stateProvince: Aragón; county: Huesca; locality: O Furno; verbatimElevation: 1396.73; decimalLatitude: 42.60677; decimalLongitude: 0.13135; geodeticDatum: WGS84; **Event:** eventID: C; samplingProtocol: Pitfall
- **Type status:** Other material. **Occurrence:** individualCount: 1; sex: female; **Location:** locationID: P3; continent: Europe; country: Spain; countryCode: ES; stateProvince: Castilla y León; county: León; locality: Las Arroyas; verbatimElevation: 1097.1; decimalLatitude: 43.14351; decimalLongitude: -4.94878; geodeticDatum: WGS84; **Event:** eventID: 1; samplingProtocol: Sweeping; eventTime: Night
- **Type status:** Other material. **Occurrence:** individualCount: 1; sex: female; **Location:** locationID: P3; continent: Europe; country: Spain; countryCode: ES; stateProvince: Castilla y León; county: León; locality: Las Arroyas; verbatimElevation: 1097.1; decimalLatitude: 43.14351; decimalLongitude: -4.94878; geodeticDatum: WGS84; **Event:** eventID: 2; samplingProtocol: Sweeping; eventTime: Day
- **Type status:** Other material. **Occurrence:** individualCount: 2; sex: male; **Location:** locationID: P4; continent: Europe; country: Spain; countryCode: ES; stateProvince: Castilla y León; county: León; locality: El Canto; verbatimElevation: 943.48; decimalLatitude: 43.17227; decimalLongitude: -4.90857; geodeticDatum: WGS84; **Event:** eventID: H; samplingProtocol: Pitfall

##### Distribution

Holarctic

#### Prinerigone vagans

(Audouin, 1826)

##### Materials

- **Type status:** Other material. **Occurrence:** individualCount: 1; sex: male; **Location:** locationID: C1; continent: Europe; country: Spain; countryCode: ES; stateProvince: Castilla-La Mancha; county: Ciudad Real; locality: Valle Brezoso; verbatimElevation: 756.56; decimalLatitude: 39.35663; decimalLongitude: -4.35912; geodeticDatum: WGS84; **Event:** eventID: K; samplingProtocol: Pitfall
- **Type status:** Other material. **Occurrence:** individualCount: 1; sex: male; **Location:** locationID: C2; continent: Europe; country: Spain; countryCode: ES; stateProvince: Castilla-La Mancha; county: Ciudad Real; locality: Valle Brezoso; verbatimElevation: 739.31; decimalLatitude: 39.35159; decimalLongitude: -4.3589; geodeticDatum: WGS84; **Event:** eventID: L; samplingProtocol: Pitfall
- **Type status:** Other material. **Occurrence:** individualCount: 1; sex: female; **Location:** locationID: C2; continent: Europe; country: Spain; countryCode: ES; stateProvince: Castilla-La Mancha; county: Ciudad Real; locality: Valle Brezoso; verbatimElevation: 739.31; decimalLatitude: 39.35159; decimalLongitude: -4.3589; geodeticDatum: WGS84; **Event:** eventID: 2; samplingProtocol: Sweeping; eventTime: Day
- **Type status:** Other material. **Occurrence:** individualCount: 3; sex: female; **Location:** locationID: C4; continent: Europe; country: Spain; countryCode: ES; stateProvince: Castilla-La Mancha; county: Ciudad Real; locality: La Quesera; verbatimElevation: 772.3; decimalLatitude: 39.36337; decimalLongitude: -4.41704; geodeticDatum: WGS84; **Event:** eventID: A; samplingProtocol: Pitfall

##### Distribution

Old World

#### Stemonyphantes lineatus

(Linnaeus, 1758)

##### Materials

- **Type status:** Other material. **Occurrence:** individualCount: 1; sex: female; **Location:** locationID: P3; continent: Europe; country: Spain; countryCode: ES; stateProvince: Castilla y León; county: León; locality: Las Arroyas; verbatimElevation: 1097.1; decimalLatitude: 43.14351; decimalLongitude: -4.94878; geodeticDatum: WGS84; **Event:** eventID: D; samplingProtocol: Pitfall

##### Distribution

Palearctic

#### Styloctetor romanus

(O. Pickard-Cambridge, 1872)

##### Materials

- **Type status:** Other material. **Occurrence:** individualCount: 1; sex: female; **Location:** locationID: C1; continent: Europe; country: Spain; countryCode: ES; stateProvince: Castilla-La Mancha; county: Ciudad Real; locality: Valle Brezoso; verbatimElevation: 756.56; decimalLatitude: 39.35663; decimalLongitude: -4.35912; geodeticDatum: WGS84; **Event:** eventID: 1; samplingProtocol: Beating; eventTime: Day
- **Type status:** Other material. **Occurrence:** individualCount: 1; sex: female; **Location:** locationID: M2; continent: Europe; country: Spain; countryCode: ES; stateProvince: Extremadura; county: Cáceres; locality: Fuente del Frances; verbatimElevation: 320.72; decimalLatitude: 39.828; decimalLongitude: -6.03249; geodeticDatum: WGS84; **Event:** eventID: 2; samplingProtocol: Sweeping; eventTime: Day

##### Distribution

Palearctic

#### Tapinocyba affinis pyrenaea

Millidge, 1979

##### Materials

- **Type status:** Other material. **Occurrence:** individualCount: 5; sex: male; **Location:** locationID: O1; continent: Europe; country: Spain; countryCode: ES; stateProvince: Aragón; county: Huesca; locality: O Furno; verbatimElevation: 1396.73; decimalLatitude: 42.60677; decimalLongitude: 0.13135; geodeticDatum: WGS84; **Event:** eventID: A; samplingProtocol: Pitfall
- **Type status:** Other material. **Occurrence:** individualCount: 1; sex: male; **Location:** locationID: O1; continent: Europe; country: Spain; countryCode: ES; stateProvince: Aragón; county: Huesca; locality: O Furno; verbatimElevation: 1396.73; decimalLatitude: 42.60677; decimalLongitude: 0.13135; geodeticDatum: WGS84; **Event:** eventID: D; samplingProtocol: Pitfall
- **Type status:** Other material. **Occurrence:** individualCount: 2; sex: male; **Location:** locationID: O1; continent: Europe; country: Spain; countryCode: ES; stateProvince: Aragón; county: Huesca; locality: O Furno; verbatimElevation: 1396.73; decimalLatitude: 42.60677; decimalLongitude: 0.13135; geodeticDatum: WGS84; **Event:** eventID: I; samplingProtocol: Pitfall
- **Type status:** Other material. **Occurrence:** individualCount: 2; sex: male; **Location:** locationID: O1; continent: Europe; country: Spain; countryCode: ES; stateProvince: Aragón; county: Huesca; locality: O Furno; verbatimElevation: 1396.73; decimalLatitude: 42.60677; decimalLongitude: 0.13135; geodeticDatum: WGS84; **Event:** eventID: K; samplingProtocol: Pitfall

##### Distribution

Spain, France

##### Notes

Although subspecies are seldom used nowadays in arachnology to address taxonomical issues, the present batch of specimens fit perfectly the cited subspecies described by Millidge; in the same publication, he remarks that of all the subspecies treated in his work, this is the most likely to represent a separate species (Millidge 1979). To properly assess this, a generic revision would be necessary. Therefore we keep a conservative approach in the identification of these specimens. See Species delimitation and identification using DNA barcodes.

#### Tapinocyba mitis

(O. Pickard-Cambridge, 1882)

##### Materials

- **Type status:** Other material. **Occurrence:** individualCount: 1; sex: male; **Location:** locationID: P1; continent: Europe; country: Spain; countryCode: ES; stateProvince: Castilla y León; county: León; locality: Monte Robledo; verbatimElevation: 1071.58; decimalLatitude: 43.1445; decimalLongitude: -4.92675; geodeticDatum: WGS84; **Event:** eventID: F; samplingProtocol: Pitfall
- **Type status:** Other material. **Occurrence:** individualCount: 1; sex: male; **Location:** locationID: P4; continent: Europe; country: Spain; countryCode: ES; stateProvince: Castilla y León; county: León; locality: El Canto; verbatimElevation: 943.48; decimalLatitude: 43.17227; decimalLongitude: -4.90857; geodeticDatum: WGS84; **Event:** eventID: L; samplingProtocol: Pitfall

##### Distribution

Britain, Spain, France, Albania, Latvia

#### Tenuiphantes flavipes

(Blackwall, 1854)

##### Materials

- **Type status:** Other material. **Occurrence:** individualCount: 1; sex: female; **Location:** locationID: A1; continent: Europe; country: Spain; countryCode: ES; stateProvince: Catalonia; county: Lleida; locality: Sola de Boi; verbatimElevation: 1759.8; decimalLatitude: 42.54958; decimalLongitude: 0.87254; geodeticDatum: WGS84; **Event:** eventID: 1; samplingProtocol: Aerial; eventTime: Night
- **Type status:** Other material. **Occurrence:** individualCount: 2; sex: female; **Location:** locationID: A1; continent: Europe; country: Spain; countryCode: ES; stateProvince: Catalonia; county: Lleida; locality: Sola de Boi; verbatimElevation: 1759.8; decimalLatitude: 42.54958; decimalLongitude: 0.87254; geodeticDatum: WGS84; **Event:** eventID: 2; samplingProtocol: Aerial; eventTime: Night
- **Type status:** Other material. **Occurrence:** individualCount: 6; sex: female; **Location:** locationID: A1; continent: Europe; country: Spain; countryCode: ES; stateProvince: Catalonia; county: Lleida; locality: Sola de Boi; verbatimElevation: 1759.8; decimalLatitude: 42.54958; decimalLongitude: 0.87254; geodeticDatum: WGS84; **Event:** eventID: 1; samplingProtocol: Ground; eventTime: Night
- **Type status:** Other material. **Occurrence:** individualCount: 1; sex: male; **Location:** locationID: A1; continent: Europe; country: Spain; countryCode: ES; stateProvince: Catalonia; county: Lleida; locality: Sola de Boi; verbatimElevation: 1759.8; decimalLatitude: 42.54958; decimalLongitude: 0.87254; geodeticDatum: WGS84; **Event:** eventID: 1; samplingProtocol: Ground; eventTime: Night
- **Type status:** Other material. **Occurrence:** individualCount: 4; sex: male; **Location:** locationID: A1; continent: Europe; country: Spain; countryCode: ES; stateProvince: Catalonia; county: Lleida; locality: Sola de Boi; verbatimElevation: 1759.8; decimalLatitude: 42.54958; decimalLongitude: 0.87254; geodeticDatum: WGS84; **Event:** eventID: 2; samplingProtocol: Ground; eventTime: Night
- **Type status:** Other material. **Occurrence:** individualCount: 21; sex: female; **Location:** locationID: A1; continent: Europe; country: Spain; countryCode: ES; stateProvince: Catalonia; county: Lleida; locality: Sola de Boi; verbatimElevation: 1759.8; decimalLatitude: 42.54958; decimalLongitude: 0.87254; geodeticDatum: WGS84; **Event:** eventID: 2; samplingProtocol: Ground; eventTime: Night
- **Type status:** Other material. **Occurrence:** individualCount: 1; sex: female; **Location:** locationID: A1; continent: Europe; country: Spain; countryCode: ES; stateProvince: Catalonia; county: Lleida; locality: Sola de Boi; verbatimElevation: 1759.8; decimalLatitude: 42.54958; decimalLongitude: 0.87254; geodeticDatum: WGS84; **Event:** eventID: B; samplingProtocol: Pitfall
- **Type status:** Other material. **Occurrence:** individualCount: 1; sex: male; **Location:** locationID: A1; continent: Europe; country: Spain; countryCode: ES; stateProvince: Catalonia; county: Lleida; locality: Sola de Boi; verbatimElevation: 1759.8; decimalLatitude: 42.54958; decimalLongitude: 0.87254; geodeticDatum: WGS84; **Event:** eventID: C; samplingProtocol: Pitfall
- **Type status:** Other material. **Occurrence:** individualCount: 1; sex: female; **Location:** locationID: A1; continent: Europe; country: Spain; countryCode: ES; stateProvince: Catalonia; county: Lleida; locality: Sola de Boi; verbatimElevation: 1759.8; decimalLatitude: 42.54958; decimalLongitude: 0.87254; geodeticDatum: WGS84; **Event:** eventID: C; samplingProtocol: Pitfall
- **Type status:** Other material. **Occurrence:** individualCount: 2; sex: male; **Location:** locationID: A1; continent: Europe; country: Spain; countryCode: ES; stateProvince: Catalonia; county: Lleida; locality: Sola de Boi; verbatimElevation: 1759.8; decimalLatitude: 42.54958; decimalLongitude: 0.87254; geodeticDatum: WGS84; **Event:** eventID: D; samplingProtocol: Pitfall
- **Type status:** Other material. **Occurrence:** individualCount: 2; sex: male; **Location:** locationID: A1; continent: Europe; country: Spain; countryCode: ES; stateProvince: Catalonia; county: Lleida; locality: Sola de Boi; verbatimElevation: 1759.8; decimalLatitude: 42.54958; decimalLongitude: 0.87254; geodeticDatum: WGS84; **Event:** eventID: E; samplingProtocol: Pitfall
- **Type status:** Other material. **Occurrence:** individualCount: 2; sex: female; **Location:** locationID: A1; continent: Europe; country: Spain; countryCode: ES; stateProvince: Catalonia; county: Lleida; locality: Sola de Boi; verbatimElevation: 1759.8; decimalLatitude: 42.54958; decimalLongitude: 0.87254; geodeticDatum: WGS84; **Event:** eventID: H; samplingProtocol: Pitfall
- **Type status:** Other material. **Occurrence:** individualCount: 2; sex: female; **Location:** locationID: A2; continent: Europe; country: Spain; countryCode: ES; stateProvince: Catalonia; county: Lleida; locality: Sola de Boi; verbatimElevation: 1738.7; decimalLatitude: 42.54913; decimalLongitude: 0.87137; geodeticDatum: WGS84; **Event:** eventID: 1; samplingProtocol: Aerial; eventTime: Night
- **Type status:** Other material. **Occurrence:** individualCount: 2; sex: male; **Location:** locationID: A2; continent: Europe; country: Spain; countryCode: ES; stateProvince: Catalonia; county: Lleida; locality: Sola de Boi; verbatimElevation: 1738.7; decimalLatitude: 42.54913; decimalLongitude: 0.87137; geodeticDatum: WGS84; **Event:** eventID: 2; samplingProtocol: Aerial; eventTime: Night
- **Type status:** Other material. **Occurrence:** individualCount: 8; sex: female; **Location:** locationID: A2; continent: Europe; country: Spain; countryCode: ES; stateProvince: Catalonia; county: Lleida; locality: Sola de Boi; verbatimElevation: 1738.7; decimalLatitude: 42.54913; decimalLongitude: 0.87137; geodeticDatum: WGS84; **Event:** eventID: 2; samplingProtocol: Aerial; eventTime: Night
- **Type status:** Other material. **Occurrence:** individualCount: 1; sex: female; **Location:** locationID: A2; continent: Europe; country: Spain; countryCode: ES; stateProvince: Catalonia; county: Lleida; locality: Sola de Boi; verbatimElevation: 1738.7; decimalLatitude: 42.54913; decimalLongitude: 0.87137; geodeticDatum: WGS84; **Event:** eventID: C; samplingProtocol: Pitfall
- **Type status:** Other material. **Occurrence:** individualCount: 2; sex: male; **Location:** locationID: A2; continent: Europe; country: Spain; countryCode: ES; stateProvince: Catalonia; county: Lleida; locality: Sola de Boi; verbatimElevation: 1738.7; decimalLatitude: 42.54913; decimalLongitude: 0.87137; geodeticDatum: WGS84; **Event:** eventID: K; samplingProtocol: Pitfall
- **Type status:** Other material. **Occurrence:** individualCount: 1; sex: male; **Location:** locationID: A2; continent: Europe; country: Spain; countryCode: ES; stateProvince: Catalonia; county: Lleida; locality: Sola de Boi; verbatimElevation: 1738.7; decimalLatitude: 42.54913; decimalLongitude: 0.87137; geodeticDatum: WGS84; **Event:** eventID: 1; samplingProtocol: Sweeping; eventTime: Night
- **Type status:** Other material. **Occurrence:** individualCount: 1; sex: male; **Location:** locationID: O1; continent: Europe; country: Spain; countryCode: ES; stateProvince: Aragón; county: Huesca; locality: O Furno; verbatimElevation: 1396.73; decimalLatitude: 42.60677; decimalLongitude: 0.13135; geodeticDatum: WGS84; **Event:** eventID: 2; samplingProtocol: Aerial; eventTime: Night
- **Type status:** Other material. **Occurrence:** individualCount: 3; sex: female; **Location:** locationID: O1; continent: Europe; country: Spain; countryCode: ES; stateProvince: Aragón; county: Huesca; locality: O Furno; verbatimElevation: 1396.73; decimalLatitude: 42.60677; decimalLongitude: 0.13135; geodeticDatum: WGS84; **Event:** eventID: 2; samplingProtocol: Aerial; eventTime: Night
- **Type status:** Other material. **Occurrence:** individualCount: 1; sex: female; **Location:** locationID: O1; continent: Europe; country: Spain; countryCode: ES; stateProvince: Aragón; county: Huesca; locality: O Furno; verbatimElevation: 1396.73; decimalLatitude: 42.60677; decimalLongitude: 0.13135; geodeticDatum: WGS84; **Event:** eventID: 1; samplingProtocol: Beating; eventTime: Night
- **Type status:** Other material. **Occurrence:** individualCount: 2; sex: male; **Location:** locationID: O1; continent: Europe; country: Spain; countryCode: ES; stateProvince: Aragón; county: Huesca; locality: O Furno; verbatimElevation: 1396.73; decimalLatitude: 42.60677; decimalLongitude: 0.13135; geodeticDatum: WGS84; **Event:** eventID: A; samplingProtocol: Pitfall
- **Type status:** Other material. **Occurrence:** individualCount: 1; sex: male; **Location:** locationID: O1; continent: Europe; country: Spain; countryCode: ES; stateProvince: Aragón; county: Huesca; locality: O Furno; verbatimElevation: 1396.73; decimalLatitude: 42.60677; decimalLongitude: 0.13135; geodeticDatum: WGS84; **Event:** eventID: D; samplingProtocol: Pitfall
- **Type status:** Other material. **Occurrence:** individualCount: 1; sex: male; **Location:** locationID: O1; continent: Europe; country: Spain; countryCode: ES; stateProvince: Aragón; county: Huesca; locality: O Furno; verbatimElevation: 1396.73; decimalLatitude: 42.60677; decimalLongitude: 0.13135; geodeticDatum: WGS84; **Event:** eventID: E; samplingProtocol: Pitfall
- **Type status:** Other material. **Occurrence:** individualCount: 1; sex: female; **Location:** locationID: O1; continent: Europe; country: Spain; countryCode: ES; stateProvince: Aragón; county: Huesca; locality: O Furno; verbatimElevation: 1396.73; decimalLatitude: 42.60677; decimalLongitude: 0.13135; geodeticDatum: WGS84; **Event:** eventID: E; samplingProtocol: Pitfall
- **Type status:** Other material. **Occurrence:** individualCount: 1; sex: male; **Location:** locationID: O1; continent: Europe; country: Spain; countryCode: ES; stateProvince: Aragón; county: Huesca; locality: O Furno; verbatimElevation: 1396.73; decimalLatitude: 42.60677; decimalLongitude: 0.13135; geodeticDatum: WGS84; **Event:** eventID: H; samplingProtocol: Pitfall
- **Type status:** Other material. **Occurrence:** individualCount: 1; sex: male; **Location:** locationID: O1; continent: Europe; country: Spain; countryCode: ES; stateProvince: Aragón; county: Huesca; locality: O Furno; verbatimElevation: 1396.73; decimalLatitude: 42.60677; decimalLongitude: 0.13135; geodeticDatum: WGS84; **Event:** eventID: I; samplingProtocol: Pitfall
- **Type status:** Other material. **Occurrence:** individualCount: 1; sex: female; **Location:** locationID: O2; continent: Europe; country: Spain; countryCode: ES; stateProvince: Aragón; county: Huesca; locality: Rebilla; verbatimElevation: 1158.13; decimalLatitude: 42.59427; decimalLongitude: 0.1529; geodeticDatum: WGS84; **Event:** eventID: 2; samplingProtocol: Aerial; eventTime: Night
- **Type status:** Other material. **Occurrence:** individualCount: 1; sex: female; **Location:** locationID: O2; continent: Europe; country: Spain; countryCode: ES; stateProvince: Aragón; county: Huesca; locality: Rebilla; verbatimElevation: 1158.13; decimalLatitude: 42.59427; decimalLongitude: 0.1529; geodeticDatum: WGS84; **Event:** eventID: A; samplingProtocol: Pitfall
- **Type status:** Other material. **Occurrence:** individualCount: 1; sex: female; **Location:** locationID: P1; continent: Europe; country: Spain; countryCode: ES; stateProvince: Castilla y León; county: León; locality: Monte Robledo; verbatimElevation: 1071.58; decimalLatitude: 43.1445; decimalLongitude: -4.92675; geodeticDatum: WGS84; **Event:** eventID: 1; samplingProtocol: Aerial; eventTime: Night
- **Type status:** Other material. **Occurrence:** individualCount: 1; sex: female; **Location:** locationID: P1; continent: Europe; country: Spain; countryCode: ES; stateProvince: Castilla y León; county: León; locality: Monte Robledo; verbatimElevation: 1071.58; decimalLatitude: 43.1445; decimalLongitude: -4.92675; geodeticDatum: WGS84; **Event:** eventID: 2; samplingProtocol: Aerial; eventTime: Night
- **Type status:** Other material. **Occurrence:** individualCount: 1; sex: male; **Location:** locationID: P2; continent: Europe; country: Spain; countryCode: ES; stateProvince: Castilla y León; county: León; locality: Joyoguelas; verbatimElevation: 763.98; decimalLatitude: 43.17771; decimalLongitude: -4.90579; geodeticDatum: WGS84; **Event:** eventID: 2; samplingProtocol: Beating; eventTime: Day
- **Type status:** Other material. **Occurrence:** individualCount: 1; sex: female; **Location:** locationID: P3; continent: Europe; country: Spain; countryCode: ES; stateProvince: Castilla y León; county: León; locality: Las Arroyas; verbatimElevation: 1097.1; decimalLatitude: 43.14351; decimalLongitude: -4.94878; geodeticDatum: WGS84; **Event:** eventID: 1; samplingProtocol: Sweeping; eventTime: Day
- **Type status:** Other material. **Occurrence:** individualCount: 1; sex: female; **Location:** locationID: P4; continent: Europe; country: Spain; countryCode: ES; stateProvince: Castilla y León; county: León; locality: El Canto; verbatimElevation: 943.48; decimalLatitude: 43.17227; decimalLongitude: -4.90857; geodeticDatum: WGS84; **Event:** eventID: G; samplingProtocol: Pitfall

##### Distribution

Europe

#### Tenuiphantes herbicola

(Simon, 1884)

##### Materials

- **Type status:** Other material. **Occurrence:** individualCount: 1; sex: male; **Location:** locationID: O1; continent: Europe; country: Spain; countryCode: ES; stateProvince: Aragón; county: Huesca; locality: O Furno; verbatimElevation: 1396.73; decimalLatitude: 42.60677; decimalLongitude: 0.13135; geodeticDatum: WGS84; **Event:** eventID: A; samplingProtocol: Pitfall
- **Type status:** Other material. **Occurrence:** individualCount: 1; sex: male; **Location:** locationID: O1; continent: Europe; country: Spain; countryCode: ES; stateProvince: Aragón; county: Huesca; locality: O Furno; verbatimElevation: 1396.73; decimalLatitude: 42.60677; decimalLongitude: 0.13135; geodeticDatum: WGS84; **Event:** eventID: F; samplingProtocol: Pitfall
- **Type status:** Other material. **Occurrence:** individualCount: 1; sex: female; **Location:** locationID: O1; continent: Europe; country: Spain; countryCode: ES; stateProvince: Aragón; county: Huesca; locality: O Furno; verbatimElevation: 1396.73; decimalLatitude: 42.60677; decimalLongitude: 0.13135; geodeticDatum: WGS84; **Event:** eventID: J; samplingProtocol: Pitfall

##### Distribution

France, Corsica, Italy, Albania, Greece, Algeria

#### Tenuiphantes tenuis

(Blackwall, 1852)

##### Materials

- **Type status:** Other material. **Occurrence:** individualCount: 1; sex: female; **Location:** locationID: A1; continent: Europe; country: Spain; countryCode: ES; stateProvince: Catalonia; county: Lleida; locality: Sola de Boi; verbatimElevation: 1759.8; decimalLatitude: 42.54958; decimalLongitude: 0.87254; geodeticDatum: WGS84; **Event:** eventID: C; samplingProtocol: Pitfall
- **Type status:** Other material. **Occurrence:** individualCount: 4; sex: female; **Location:** locationID: C1; continent: Europe; country: Spain; countryCode: ES; stateProvince: Castilla-La Mancha; county: Ciudad Real; locality: Valle Brezoso; verbatimElevation: 756.56; decimalLatitude: 39.35663; decimalLongitude: -4.35912; geodeticDatum: WGS84; **Event:** eventID: 3; samplingProtocol: Aerial; eventTime: Night
- **Type status:** Other material. **Occurrence:** individualCount: 1; sex: male; **Location:** locationID: C1; continent: Europe; country: Spain; countryCode: ES; stateProvince: Castilla-La Mancha; county: Ciudad Real; locality: Valle Brezoso; verbatimElevation: 756.56; decimalLatitude: 39.35663; decimalLongitude: -4.35912; geodeticDatum: WGS84; **Event:** eventID: 1; samplingProtocol: Beating; eventTime: Night
- **Type status:** Other material. **Occurrence:** individualCount: 1; sex: female; **Location:** locationID: C1; continent: Europe; country: Spain; countryCode: ES; stateProvince: Castilla-La Mancha; county: Ciudad Real; locality: Valle Brezoso; verbatimElevation: 756.56; decimalLatitude: 39.35663; decimalLongitude: -4.35912; geodeticDatum: WGS84; **Event:** eventID: 1; samplingProtocol: Beating; eventTime: Night
- **Type status:** Other material. **Occurrence:** individualCount: 1; sex: male; **Location:** locationID: C1; continent: Europe; country: Spain; countryCode: ES; stateProvince: Castilla-La Mancha; county: Ciudad Real; locality: Valle Brezoso; verbatimElevation: 756.56; decimalLatitude: 39.35663; decimalLongitude: -4.35912; geodeticDatum: WGS84; **Event:** eventID: 2; samplingProtocol: Beating; eventTime: Day
- **Type status:** Other material. **Occurrence:** individualCount: 2; sex: male; **Location:** locationID: C1; continent: Europe; country: Spain; countryCode: ES; stateProvince: Castilla-La Mancha; county: Ciudad Real; locality: Valle Brezoso; verbatimElevation: 756.56; decimalLatitude: 39.35663; decimalLongitude: -4.35912; geodeticDatum: WGS84; **Event:** eventID: C; samplingProtocol: Pitfall
- **Type status:** Other material. **Occurrence:** individualCount: 2; sex: female; **Location:** locationID: C1; continent: Europe; country: Spain; countryCode: ES; stateProvince: Castilla-La Mancha; county: Ciudad Real; locality: Valle Brezoso; verbatimElevation: 756.56; decimalLatitude: 39.35663; decimalLongitude: -4.35912; geodeticDatum: WGS84; **Event:** eventID: C; samplingProtocol: Pitfall
- **Type status:** Other material. **Occurrence:** individualCount: 1; sex: female; **Location:** locationID: C1; continent: Europe; country: Spain; countryCode: ES; stateProvince: Castilla-La Mancha; county: Ciudad Real; locality: Valle Brezoso; verbatimElevation: 756.56; decimalLatitude: 39.35663; decimalLongitude: -4.35912; geodeticDatum: WGS84; **Event:** eventID: D; samplingProtocol: Pitfall
- **Type status:** Other material. **Occurrence:** individualCount: 1; sex: female; **Location:** locationID: C1; continent: Europe; country: Spain; countryCode: ES; stateProvince: Castilla-La Mancha; county: Ciudad Real; locality: Valle Brezoso; verbatimElevation: 756.56; decimalLatitude: 39.35663; decimalLongitude: -4.35912; geodeticDatum: WGS84; **Event:** eventID: G; samplingProtocol: Pitfall
- **Type status:** Other material. **Occurrence:** individualCount: 1; sex: female; **Location:** locationID: C1; continent: Europe; country: Spain; countryCode: ES; stateProvince: Castilla-La Mancha; county: Ciudad Real; locality: Valle Brezoso; verbatimElevation: 756.56; decimalLatitude: 39.35663; decimalLongitude: -4.35912; geodeticDatum: WGS84; **Event:** eventID: L; samplingProtocol: Pitfall
- **Type status:** Other material. **Occurrence:** individualCount: 1; sex: female; **Location:** locationID: C1; continent: Europe; country: Spain; countryCode: ES; stateProvince: Castilla-La Mancha; county: Ciudad Real; locality: Valle Brezoso; verbatimElevation: 756.56; decimalLatitude: 39.35663; decimalLongitude: -4.35912; geodeticDatum: WGS84; **Event:** eventID: 1; samplingProtocol: Sweeping; eventTime: Day
- **Type status:** Other material. **Occurrence:** individualCount: 1; sex: female; **Location:** locationID: C1; continent: Europe; country: Spain; countryCode: ES; stateProvince: Castilla-La Mancha; county: Ciudad Real; locality: Valle Brezoso; verbatimElevation: 756.56; decimalLatitude: 39.35663; decimalLongitude: -4.35912; geodeticDatum: WGS84; **Event:** eventID: 1; samplingProtocol: Sweeping; eventTime: Night
- **Type status:** Other material. **Occurrence:** individualCount: 2; sex: female; **Location:** locationID: C1; continent: Europe; country: Spain; countryCode: ES; stateProvince: Castilla-La Mancha; county: Ciudad Real; locality: Valle Brezoso; verbatimElevation: 756.56; decimalLatitude: 39.35663; decimalLongitude: -4.35912; geodeticDatum: WGS84; **Event:** eventID: 2; samplingProtocol: Sweeping; eventTime: Day
- **Type status:** Other material. **Occurrence:** individualCount: 1; sex: female; **Location:** locationID: C2; continent: Europe; country: Spain; countryCode: ES; stateProvince: Castilla-La Mancha; county: Ciudad Real; locality: Valle Brezoso; verbatimElevation: 739.31; decimalLatitude: 39.35159; decimalLongitude: -4.3589; geodeticDatum: WGS84; **Event:** eventID: 2; samplingProtocol: Aerial; eventTime: Night
- **Type status:** Other material. **Occurrence:** individualCount: 3; sex: male; **Location:** locationID: C2; continent: Europe; country: Spain; countryCode: ES; stateProvince: Castilla-La Mancha; county: Ciudad Real; locality: Valle Brezoso; verbatimElevation: 739.31; decimalLatitude: 39.35159; decimalLongitude: -4.3589; geodeticDatum: WGS84; **Event:** eventID: 2; samplingProtocol: Aerial; eventTime: Night
- **Type status:** Other material. **Occurrence:** individualCount: 1; sex: female; **Location:** locationID: C2; continent: Europe; country: Spain; countryCode: ES; stateProvince: Castilla-La Mancha; county: Ciudad Real; locality: Valle Brezoso; verbatimElevation: 739.31; decimalLatitude: 39.35159; decimalLongitude: -4.3589; geodeticDatum: WGS84; **Event:** eventID: 1; samplingProtocol: Aerial; eventTime: Night
- **Type status:** Other material. **Occurrence:** individualCount: 2; sex: male; **Location:** locationID: C2; continent: Europe; country: Spain; countryCode: ES; stateProvince: Castilla-La Mancha; county: Ciudad Real; locality: Valle Brezoso; verbatimElevation: 739.31; decimalLatitude: 39.35159; decimalLongitude: -4.3589; geodeticDatum: WGS84; **Event:** eventID: 1; samplingProtocol: Beating; eventTime: Day
- **Type status:** Other material. **Occurrence:** individualCount: 2; sex: male; **Location:** locationID: C2; continent: Europe; country: Spain; countryCode: ES; stateProvince: Castilla-La Mancha; county: Ciudad Real; locality: Valle Brezoso; verbatimElevation: 739.31; decimalLatitude: 39.35159; decimalLongitude: -4.3589; geodeticDatum: WGS84; **Event:** eventID: 1; samplingProtocol: Beating; eventTime: Night
- **Type status:** Other material. **Occurrence:** individualCount: 1; sex: male; **Location:** locationID: C2; continent: Europe; country: Spain; countryCode: ES; stateProvince: Castilla-La Mancha; county: Ciudad Real; locality: Valle Brezoso; verbatimElevation: 739.31; decimalLatitude: 39.35159; decimalLongitude: -4.3589; geodeticDatum: WGS84; **Event:** eventID: 2; samplingProtocol: Beating; eventTime: Night
- **Type status:** Other material. **Occurrence:** individualCount: 1; sex: male; **Location:** locationID: C2; continent: Europe; country: Spain; countryCode: ES; stateProvince: Castilla-La Mancha; county: Ciudad Real; locality: Valle Brezoso; verbatimElevation: 739.31; decimalLatitude: 39.35159; decimalLongitude: -4.3589; geodeticDatum: WGS84; **Event:** eventID: 2; samplingProtocol: Beating; eventTime: Day
- **Type status:** Other material. **Occurrence:** individualCount: 2; sex: female; **Location:** locationID: C2; continent: Europe; country: Spain; countryCode: ES; stateProvince: Castilla-La Mancha; county: Ciudad Real; locality: Valle Brezoso; verbatimElevation: 739.31; decimalLatitude: 39.35159; decimalLongitude: -4.3589; geodeticDatum: WGS84; **Event:** eventID: 2; samplingProtocol: Beating; eventTime: Day
- **Type status:** Other material. **Occurrence:** individualCount: 1; sex: female; **Location:** locationID: C2; continent: Europe; country: Spain; countryCode: ES; stateProvince: Castilla-La Mancha; county: Ciudad Real; locality: Valle Brezoso; verbatimElevation: 739.31; decimalLatitude: 39.35159; decimalLongitude: -4.3589; geodeticDatum: WGS84; **Event:** eventID: A; samplingProtocol: Pitfall
- **Type status:** Other material. **Occurrence:** individualCount: 1; sex: female; **Location:** locationID: C2; continent: Europe; country: Spain; countryCode: ES; stateProvince: Castilla-La Mancha; county: Ciudad Real; locality: Valle Brezoso; verbatimElevation: 739.31; decimalLatitude: 39.35159; decimalLongitude: -4.3589; geodeticDatum: WGS84; **Event:** eventID: D; samplingProtocol: Pitfall
- **Type status:** Other material. **Occurrence:** individualCount: 1; sex: female; **Location:** locationID: C2; continent: Europe; country: Spain; countryCode: ES; stateProvince: Castilla-La Mancha; county: Ciudad Real; locality: Valle Brezoso; verbatimElevation: 739.31; decimalLatitude: 39.35159; decimalLongitude: -4.3589; geodeticDatum: WGS84; **Event:** eventID: E; samplingProtocol: Pitfall
- **Type status:** Other material. **Occurrence:** individualCount: 1; sex: male; **Location:** locationID: C2; continent: Europe; country: Spain; countryCode: ES; stateProvince: Castilla-La Mancha; county: Ciudad Real; locality: Valle Brezoso; verbatimElevation: 739.31; decimalLatitude: 39.35159; decimalLongitude: -4.3589; geodeticDatum: WGS84; **Event:** eventID: F; samplingProtocol: Pitfall
- **Type status:** Other material. **Occurrence:** individualCount: 1; sex: female; **Location:** locationID: C2; continent: Europe; country: Spain; countryCode: ES; stateProvince: Castilla-La Mancha; county: Ciudad Real; locality: Valle Brezoso; verbatimElevation: 739.31; decimalLatitude: 39.35159; decimalLongitude: -4.3589; geodeticDatum: WGS84; **Event:** eventID: F; samplingProtocol: Pitfall
- **Type status:** Other material. **Occurrence:** individualCount: 1; sex: female; **Location:** locationID: C2; continent: Europe; country: Spain; countryCode: ES; stateProvince: Castilla-La Mancha; county: Ciudad Real; locality: Valle Brezoso; verbatimElevation: 739.31; decimalLatitude: 39.35159; decimalLongitude: -4.3589; geodeticDatum: WGS84; **Event:** eventID: G; samplingProtocol: Pitfall
- **Type status:** Other material. **Occurrence:** individualCount: 1; sex: male; **Location:** locationID: C2; continent: Europe; country: Spain; countryCode: ES; stateProvince: Castilla-La Mancha; county: Ciudad Real; locality: Valle Brezoso; verbatimElevation: 739.31; decimalLatitude: 39.35159; decimalLongitude: -4.3589; geodeticDatum: WGS84; **Event:** eventID: H; samplingProtocol: Pitfall
- **Type status:** Other material. **Occurrence:** individualCount: 1; sex: female; **Location:** locationID: C2; continent: Europe; country: Spain; countryCode: ES; stateProvince: Castilla-La Mancha; county: Ciudad Real; locality: Valle Brezoso; verbatimElevation: 739.31; decimalLatitude: 39.35159; decimalLongitude: -4.3589; geodeticDatum: WGS84; **Event:** eventID: K; samplingProtocol: Pitfall
- **Type status:** Other material. **Occurrence:** individualCount: 1; sex: male; **Location:** locationID: C2; continent: Europe; country: Spain; countryCode: ES; stateProvince: Castilla-La Mancha; county: Ciudad Real; locality: Valle Brezoso; verbatimElevation: 739.31; decimalLatitude: 39.35159; decimalLongitude: -4.3589; geodeticDatum: WGS84; **Event:** eventID: 1; samplingProtocol: Sweeping; eventTime: Day
- **Type status:** Other material. **Occurrence:** individualCount: 1; sex: female; **Location:** locationID: C2; continent: Europe; country: Spain; countryCode: ES; stateProvince: Castilla-La Mancha; county: Ciudad Real; locality: Valle Brezoso; verbatimElevation: 739.31; decimalLatitude: 39.35159; decimalLongitude: -4.3589; geodeticDatum: WGS84; **Event:** eventID: 1; samplingProtocol: Sweeping; eventTime: Day
- **Type status:** Other material. **Occurrence:** individualCount: 1; sex: female; **Location:** locationID: C2; continent: Europe; country: Spain; countryCode: ES; stateProvince: Castilla-La Mancha; county: Ciudad Real; locality: Valle Brezoso; verbatimElevation: 739.31; decimalLatitude: 39.35159; decimalLongitude: -4.3589; geodeticDatum: WGS84; **Event:** eventID: 2; samplingProtocol: Sweeping; eventTime: Night
- **Type status:** Other material. **Occurrence:** individualCount: 3; sex: male; **Location:** locationID: C2; continent: Europe; country: Spain; countryCode: ES; stateProvince: Castilla-La Mancha; county: Ciudad Real; locality: Valle Brezoso; verbatimElevation: 739.31; decimalLatitude: 39.35159; decimalLongitude: -4.3589; geodeticDatum: WGS84; **Event:** eventID: 2; samplingProtocol: Sweeping; eventTime: Day
- **Type status:** Other material. **Occurrence:** individualCount: 1; sex: female; **Location:** locationID: C2; continent: Europe; country: Spain; countryCode: ES; stateProvince: Castilla-La Mancha; county: Ciudad Real; locality: Valle Brezoso; verbatimElevation: 739.31; decimalLatitude: 39.35159; decimalLongitude: -4.3589; geodeticDatum: WGS84; **Event:** eventID: 2; samplingProtocol: Sweeping; eventTime: Day
- **Type status:** Other material. **Occurrence:** individualCount: 1; sex: female; **Location:** locationID: C3; continent: Europe; country: Spain; countryCode: ES; stateProvince: Castilla-La Mancha; county: Ciudad Real; locality: La Quesera; verbatimElevation: 767.55; decimalLatitude: 39.36177; decimalLongitude: -4.41733; geodeticDatum: WGS84; **Event:** eventID: L; samplingProtocol: Pitfall
- **Type status:** Other material. **Occurrence:** individualCount: 1; sex: female; **Location:** locationID: C3; continent: Europe; country: Spain; countryCode: ES; stateProvince: Castilla-La Mancha; county: Ciudad Real; locality: La Quesera; verbatimElevation: 767.55; decimalLatitude: 39.36177; decimalLongitude: -4.41733; geodeticDatum: WGS84; **Event:** eventID: 2; samplingProtocol: Sweeping; eventTime: Night
- **Type status:** Other material. **Occurrence:** individualCount: 3; sex: male; **Location:** locationID: C4; continent: Europe; country: Spain; countryCode: ES; stateProvince: Castilla-La Mancha; county: Ciudad Real; locality: La Quesera; verbatimElevation: 772.3; decimalLatitude: 39.36337; decimalLongitude: -4.41704; geodeticDatum: WGS84; **Event:** eventID: 1; samplingProtocol: Beating; eventTime: Day
- **Type status:** Other material. **Occurrence:** individualCount: 1; sex: female; **Location:** locationID: C4; continent: Europe; country: Spain; countryCode: ES; stateProvince: Castilla-La Mancha; county: Ciudad Real; locality: La Quesera; verbatimElevation: 772.3; decimalLatitude: 39.36337; decimalLongitude: -4.41704; geodeticDatum: WGS84; **Event:** eventID: G; samplingProtocol: Pitfall
- **Type status:** Other material. **Occurrence:** individualCount: 1; sex: male; **Location:** locationID: M1; continent: Europe; country: Spain; countryCode: ES; stateProvince: Extremadura; county: Cáceres; locality: Peña Falcón; verbatimElevation: 320.6; decimalLatitude: 39.83296; decimalLongitude: -6.0641; geodeticDatum: WGS84; **Event:** eventID: 1; samplingProtocol: Beating; eventTime: Day
- **Type status:** Other material. **Occurrence:** individualCount: 1; sex: male; **Location:** locationID: M1; continent: Europe; country: Spain; countryCode: ES; stateProvince: Extremadura; county: Cáceres; locality: Peña Falcón; verbatimElevation: 320.6; decimalLatitude: 39.83296; decimalLongitude: -6.0641; geodeticDatum: WGS84; **Event:** eventID: A; samplingProtocol: Pitfall
- **Type status:** Other material. **Occurrence:** individualCount: 1; sex: female; **Location:** locationID: M1; continent: Europe; country: Spain; countryCode: ES; stateProvince: Extremadura; county: Cáceres; locality: Peña Falcón; verbatimElevation: 320.6; decimalLatitude: 39.83296; decimalLongitude: -6.0641; geodeticDatum: WGS84; **Event:** eventID: A; samplingProtocol: Pitfall
- **Type status:** Other material. **Occurrence:** individualCount: 1; sex: female; **Location:** locationID: M1; continent: Europe; country: Spain; countryCode: ES; stateProvince: Extremadura; county: Cáceres; locality: Peña Falcón; verbatimElevation: 320.6; decimalLatitude: 39.83296; decimalLongitude: -6.0641; geodeticDatum: WGS84; **Event:** eventID: I; samplingProtocol: Pitfall
- **Type status:** Other material. **Occurrence:** individualCount: 1; sex: female; **Location:** locationID: M1; continent: Europe; country: Spain; countryCode: ES; stateProvince: Extremadura; county: Cáceres; locality: Peña Falcón; verbatimElevation: 320.6; decimalLatitude: 39.83296; decimalLongitude: -6.0641; geodeticDatum: WGS84; **Event:** eventID: 1; samplingProtocol: Sweeping; eventTime: Day
- **Type status:** Other material. **Occurrence:** individualCount: 1; sex: male; **Location:** locationID: M1; continent: Europe; country: Spain; countryCode: ES; stateProvince: Extremadura; county: Cáceres; locality: Peña Falcón; verbatimElevation: 320.6; decimalLatitude: 39.83296; decimalLongitude: -6.0641; geodeticDatum: WGS84; **Event:** eventID: 1; samplingProtocol: Sweeping; eventTime: Night
- **Type status:** Other material. **Occurrence:** individualCount: 1; sex: male; **Location:** locationID: M2; continent: Europe; country: Spain; countryCode: ES; stateProvince: Extremadura; county: Cáceres; locality: Fuente del Frances; verbatimElevation: 320.72; decimalLatitude: 39.828; decimalLongitude: -6.03249; geodeticDatum: WGS84; **Event:** eventID: 1; samplingProtocol: Beating; eventTime: Night
- **Type status:** Other material. **Occurrence:** individualCount: 1; sex: female; **Location:** locationID: M2; continent: Europe; country: Spain; countryCode: ES; stateProvince: Extremadura; county: Cáceres; locality: Fuente del Frances; verbatimElevation: 320.72; decimalLatitude: 39.828; decimalLongitude: -6.03249; geodeticDatum: WGS84; **Event:** eventID: 1; samplingProtocol: Beating; eventTime: Night
- **Type status:** Other material. **Occurrence:** individualCount: 1; sex: male; **Location:** locationID: M2; continent: Europe; country: Spain; countryCode: ES; stateProvince: Extremadura; county: Cáceres; locality: Fuente del Frances; verbatimElevation: 320.72; decimalLatitude: 39.828; decimalLongitude: -6.03249; geodeticDatum: WGS84; **Event:** eventID: E; samplingProtocol: Pitfall
- **Type status:** Other material. **Occurrence:** individualCount: 1; sex: male; **Location:** locationID: M2; continent: Europe; country: Spain; countryCode: ES; stateProvince: Extremadura; county: Cáceres; locality: Fuente del Frances; verbatimElevation: 320.72; decimalLatitude: 39.828; decimalLongitude: -6.03249; geodeticDatum: WGS84; **Event:** eventID: G; samplingProtocol: Pitfall
- **Type status:** Other material. **Occurrence:** individualCount: 1; sex: female; **Location:** locationID: M2; continent: Europe; country: Spain; countryCode: ES; stateProvince: Extremadura; county: Cáceres; locality: Fuente del Frances; verbatimElevation: 320.72; decimalLatitude: 39.828; decimalLongitude: -6.03249; geodeticDatum: WGS84; **Event:** eventID: G; samplingProtocol: Pitfall
- **Type status:** Other material. **Occurrence:** individualCount: 1; sex: male; **Location:** locationID: M2; continent: Europe; country: Spain; countryCode: ES; stateProvince: Extremadura; county: Cáceres; locality: Fuente del Frances; verbatimElevation: 320.72; decimalLatitude: 39.828; decimalLongitude: -6.03249; geodeticDatum: WGS84; **Event:** eventID: J; samplingProtocol: Pitfall
- **Type status:** Other material. **Occurrence:** individualCount: 2; sex: female; **Location:** locationID: M2; continent: Europe; country: Spain; countryCode: ES; stateProvince: Extremadura; county: Cáceres; locality: Fuente del Frances; verbatimElevation: 320.72; decimalLatitude: 39.828; decimalLongitude: -6.03249; geodeticDatum: WGS84; **Event:** eventID: 1; samplingProtocol: Sweeping; eventTime: Day
- **Type status:** Other material. **Occurrence:** individualCount: 1; sex: female; **Location:** locationID: O1; continent: Europe; country: Spain; countryCode: ES; stateProvince: Aragón; county: Huesca; locality: O Furno; verbatimElevation: 1396.73; decimalLatitude: 42.60677; decimalLongitude: 0.13135; geodeticDatum: WGS84; **Event:** eventID: 1; samplingProtocol: Aerial; eventTime: Night
- **Type status:** Other material. **Occurrence:** individualCount: 1; sex: male; **Location:** locationID: O1; continent: Europe; country: Spain; countryCode: ES; stateProvince: Aragón; county: Huesca; locality: O Furno; verbatimElevation: 1396.73; decimalLatitude: 42.60677; decimalLongitude: 0.13135; geodeticDatum: WGS84; **Event:** eventID: 1; samplingProtocol: Beating; eventTime: Night
- **Type status:** Other material. **Occurrence:** individualCount: 1; sex: female; **Location:** locationID: O1; continent: Europe; country: Spain; countryCode: ES; stateProvince: Aragón; county: Huesca; locality: O Furno; verbatimElevation: 1396.73; decimalLatitude: 42.60677; decimalLongitude: 0.13135; geodeticDatum: WGS84; **Event:** eventID: 1; samplingProtocol: Sweeping; eventTime: Night
- **Type status:** Other material. **Occurrence:** individualCount: 1; sex: female; **Location:** locationID: S1; continent: Europe; country: Spain; countryCode: ES; stateProvince: Andalucía; county: Granada; locality: Soportujar; verbatimElevation: 1786.57; decimalLatitude: 36.96151; decimalLongitude: -3.41881; geodeticDatum: WGS84; **Event:** eventID: 1; samplingProtocol: Beating; eventTime: Night
- **Type status:** Other material. **Occurrence:** individualCount: 2; sex: female; **Location:** locationID: S2; continent: Europe; country: Spain; countryCode: ES; stateProvince: Andalucía; county: Granada; locality: Camarate; verbatimElevation: 1713.96; decimalLatitude: 37.18377; decimalLongitude: -3.26282; geodeticDatum: WGS84; **Event:** eventID: 1; samplingProtocol: Aerial; eventTime: Night
- **Type status:** Other material. **Occurrence:** individualCount: 2; sex: female; **Location:** locationID: S2; continent: Europe; country: Spain; countryCode: ES; stateProvince: Andalucía; county: Granada; locality: Camarate; verbatimElevation: 1713.96; decimalLatitude: 37.18377; decimalLongitude: -3.26282; geodeticDatum: WGS84; **Event:** eventID: 1; samplingProtocol: Beating; eventTime: Day
- **Type status:** Other material. **Occurrence:** individualCount: 1; sex: male; **Location:** locationID: S2; continent: Europe; country: Spain; countryCode: ES; stateProvince: Andalucía; county: Granada; locality: Camarate; verbatimElevation: 1713.96; decimalLatitude: 37.18377; decimalLongitude: -3.26282; geodeticDatum: WGS84; **Event:** eventID: 2; samplingProtocol: Beating; eventTime: Night
- **Type status:** Other material. **Occurrence:** individualCount: 1; sex: female; **Location:** locationID: S2; continent: Europe; country: Spain; countryCode: ES; stateProvince: Andalucía; county: Granada; locality: Camarate; verbatimElevation: 1713.96; decimalLatitude: 37.18377; decimalLongitude: -3.26282; geodeticDatum: WGS84; **Event:** eventID: 2; samplingProtocol: Beating; eventTime: Night
- **Type status:** Other material. **Occurrence:** individualCount: 1; sex: male; **Location:** locationID: S2; continent: Europe; country: Spain; countryCode: ES; stateProvince: Andalucía; county: Granada; locality: Camarate; verbatimElevation: 1713.96; decimalLatitude: 37.18377; decimalLongitude: -3.26282; geodeticDatum: WGS84; **Event:** eventID: 2; samplingProtocol: Beating; eventTime: Day
- **Type status:** Other material. **Occurrence:** individualCount: 3; sex: male; **Location:** locationID: S2; continent: Europe; country: Spain; countryCode: ES; stateProvince: Andalucía; county: Granada; locality: Camarate; verbatimElevation: 1713.96; decimalLatitude: 37.18377; decimalLongitude: -3.26282; geodeticDatum: WGS84; **Event:** eventID: 1; samplingProtocol: Sweeping; eventTime: Day
- **Type status:** Other material. **Occurrence:** individualCount: 1; sex: female; **Location:** locationID: S2; continent: Europe; country: Spain; countryCode: ES; stateProvince: Andalucía; county: Granada; locality: Camarate; verbatimElevation: 1713.96; decimalLatitude: 37.18377; decimalLongitude: -3.26282; geodeticDatum: WGS84; **Event:** eventID: 1; samplingProtocol: Sweeping; eventTime: Night

##### Distribution

Palearctic (elsewhere, introduced)

#### Theonina cornix

(Simon, 1881)

##### Materials

- **Type status:** Other material. **Occurrence:** individualCount: 1; sex: male; **Location:** locationID: O1; continent: Europe; country: Spain; countryCode: ES; stateProvince: Aragón; county: Huesca; locality: O Furno; verbatimElevation: 1396.73; decimalLatitude: 42.60677; decimalLongitude: 0.13135; geodeticDatum: WGS84; **Event:** eventID: D; samplingProtocol: Pitfall
- **Type status:** Other material. **Occurrence:** individualCount: 1; sex: male; **Location:** locationID: P4; continent: Europe; country: Spain; countryCode: ES; stateProvince: Castilla y León; county: León; locality: El Canto; verbatimElevation: 943.48; decimalLatitude: 43.17227; decimalLongitude: -4.90857; geodeticDatum: WGS84; **Event:** eventID: C; samplingProtocol: Pitfall
- **Type status:** Other material. **Occurrence:** individualCount: 1; sex: female; **Location:** locationID: P4; continent: Europe; country: Spain; countryCode: ES; stateProvince: Castilla y León; county: León; locality: El Canto; verbatimElevation: 943.48; decimalLatitude: 43.17227; decimalLongitude: -4.90857; geodeticDatum: WGS84; **Event:** eventID: D; samplingProtocol: Pitfall

##### Distribution

Europe, North Africa, Russia

#### Tiso vagans

(Blackwall, 1834)

##### Materials

- **Type status:** Other material. **Occurrence:** individualCount: 1; sex: male; **Location:** locationID: O1; continent: Europe; country: Spain; countryCode: ES; stateProvince: Aragón; county: Huesca; locality: O Furno; verbatimElevation: 1396.73; decimalLatitude: 42.60677; decimalLongitude: 0.13135; geodeticDatum: WGS84; **Event:** eventID: E; samplingProtocol: Pitfall
- **Type status:** Other material. **Occurrence:** individualCount: 1; sex: male; **Location:** locationID: P2; continent: Europe; country: Spain; countryCode: ES; stateProvince: Castilla y León; county: León; locality: Joyoguelas; verbatimElevation: 763.98; decimalLatitude: 43.17771; decimalLongitude: -4.90579; geodeticDatum: WGS84; **Event:** eventID: B; samplingProtocol: Pitfall
- **Type status:** Other material. **Occurrence:** individualCount: 1; sex: male; **Location:** locationID: P2; continent: Europe; country: Spain; countryCode: ES; stateProvince: Castilla y León; county: León; locality: Joyoguelas; verbatimElevation: 763.98; decimalLatitude: 43.17771; decimalLongitude: -4.90579; geodeticDatum: WGS84; **Event:** eventID: C; samplingProtocol: Pitfall
- **Type status:** Other material. **Occurrence:** individualCount: 1; sex: male; **Location:** locationID: P2; continent: Europe; country: Spain; countryCode: ES; stateProvince: Castilla y León; county: León; locality: Joyoguelas; verbatimElevation: 763.98; decimalLatitude: 43.17771; decimalLongitude: -4.90579; geodeticDatum: WGS84; **Event:** eventID: D; samplingProtocol: Pitfall
- **Type status:** Other material. **Occurrence:** individualCount: 1; sex: male; **Location:** locationID: P2; continent: Europe; country: Spain; countryCode: ES; stateProvince: Castilla y León; county: León; locality: Joyoguelas; verbatimElevation: 763.98; decimalLatitude: 43.17771; decimalLongitude: -4.90579; geodeticDatum: WGS84; **Event:** eventID: H; samplingProtocol: Pitfall
- **Type status:** Other material. **Occurrence:** individualCount: 1; sex: male; **Location:** locationID: P3; continent: Europe; country: Spain; countryCode: ES; stateProvince: Castilla y León; county: León; locality: Las Arroyas; verbatimElevation: 1097.1; decimalLatitude: 43.14351; decimalLongitude: -4.94878; geodeticDatum: WGS84; **Event:** eventID: D; samplingProtocol: Pitfall
- **Type status:** Other material. **Occurrence:** individualCount: 1; sex: male; **Location:** locationID: P3; continent: Europe; country: Spain; countryCode: ES; stateProvince: Castilla y León; county: León; locality: Las Arroyas; verbatimElevation: 1097.1; decimalLatitude: 43.14351; decimalLongitude: -4.94878; geodeticDatum: WGS84; **Event:** eventID: F; samplingProtocol: Pitfall
- **Type status:** Other material. **Occurrence:** individualCount: 1; sex: male; **Location:** locationID: P3; continent: Europe; country: Spain; countryCode: ES; stateProvince: Castilla y León; county: León; locality: Las Arroyas; verbatimElevation: 1097.1; decimalLatitude: 43.14351; decimalLongitude: -4.94878; geodeticDatum: WGS84; **Event:** eventID: L; samplingProtocol: Pitfall
- **Type status:** Other material. **Occurrence:** individualCount: 6; sex: male; **Location:** locationID: P4; continent: Europe; country: Spain; countryCode: ES; stateProvince: Castilla y León; county: León; locality: El Canto; verbatimElevation: 943.48; decimalLatitude: 43.17227; decimalLongitude: -4.90857; geodeticDatum: WGS84; **Event:** eventID: B; samplingProtocol: Pitfall
- **Type status:** Other material. **Occurrence:** individualCount: 2; sex: male; **Location:** locationID: P4; continent: Europe; country: Spain; countryCode: ES; stateProvince: Castilla y León; county: León; locality: El Canto; verbatimElevation: 943.48; decimalLatitude: 43.17227; decimalLongitude: -4.90857; geodeticDatum: WGS84; **Event:** eventID: D; samplingProtocol: Pitfall
- **Type status:** Other material. **Occurrence:** individualCount: 6; sex: male; **Location:** locationID: P4; continent: Europe; country: Spain; countryCode: ES; stateProvince: Castilla y León; county: León; locality: El Canto; verbatimElevation: 943.48; decimalLatitude: 43.17227; decimalLongitude: -4.90857; geodeticDatum: WGS84; **Event:** eventID: E; samplingProtocol: Pitfall
- **Type status:** Other material. **Occurrence:** individualCount: 13; sex: male; **Location:** locationID: P4; continent: Europe; country: Spain; countryCode: ES; stateProvince: Castilla y León; county: León; locality: El Canto; verbatimElevation: 943.48; decimalLatitude: 43.17227; decimalLongitude: -4.90857; geodeticDatum: WGS84; **Event:** eventID: F; samplingProtocol: Pitfall
- **Type status:** Other material. **Occurrence:** individualCount: 2; sex: male; **Location:** locationID: P4; continent: Europe; country: Spain; countryCode: ES; stateProvince: Castilla y León; county: León; locality: El Canto; verbatimElevation: 943.48; decimalLatitude: 43.17227; decimalLongitude: -4.90857; geodeticDatum: WGS84; **Event:** eventID: F; samplingProtocol: Pitfall
- **Type status:** Other material. **Occurrence:** individualCount: 12; sex: male; **Location:** locationID: P4; continent: Europe; country: Spain; countryCode: ES; stateProvince: Castilla y León; county: León; locality: El Canto; verbatimElevation: 943.48; decimalLatitude: 43.17227; decimalLongitude: -4.90857; geodeticDatum: WGS84; **Event:** eventID: G; samplingProtocol: Pitfall
- **Type status:** Other material. **Occurrence:** individualCount: 3; sex: male; **Location:** locationID: P4; continent: Europe; country: Spain; countryCode: ES; stateProvince: Castilla y León; county: León; locality: El Canto; verbatimElevation: 943.48; decimalLatitude: 43.17227; decimalLongitude: -4.90857; geodeticDatum: WGS84; **Event:** eventID: H; samplingProtocol: Pitfall
- **Type status:** Other material. **Occurrence:** individualCount: 3; sex: male; **Location:** locationID: P4; continent: Europe; country: Spain; countryCode: ES; stateProvince: Castilla y León; county: León; locality: El Canto; verbatimElevation: 943.48; decimalLatitude: 43.17227; decimalLongitude: -4.90857; geodeticDatum: WGS84; **Event:** eventID: J; samplingProtocol: Pitfall
- **Type status:** Other material. **Occurrence:** individualCount: 3; sex: male; **Location:** locationID: P4; continent: Europe; country: Spain; countryCode: ES; stateProvince: Castilla y León; county: León; locality: El Canto; verbatimElevation: 943.48; decimalLatitude: 43.17227; decimalLongitude: -4.90857; geodeticDatum: WGS84; **Event:** eventID: K; samplingProtocol: Pitfall
- **Type status:** Other material. **Occurrence:** individualCount: 1; sex: male; **Location:** locationID: P4; continent: Europe; country: Spain; countryCode: ES; stateProvince: Castilla y León; county: León; locality: El Canto; verbatimElevation: 943.48; decimalLatitude: 43.17227; decimalLongitude: -4.90857; geodeticDatum: WGS84; **Event:** eventID: K; samplingProtocol: Pitfall
- **Type status:** Other material. **Occurrence:** individualCount: 7; sex: male; **Location:** locationID: P4; continent: Europe; country: Spain; countryCode: ES; stateProvince: Castilla y León; county: León; locality: El Canto; verbatimElevation: 943.48; decimalLatitude: 43.17227; decimalLongitude: -4.90857; geodeticDatum: WGS84; **Event:** eventID: L; samplingProtocol: Pitfall

##### Distribution

Europe, Madeira, Russia

#### Trichoncus affinis

Kulczynski, 1894

##### Materials

- **Type status:** Other material. **Occurrence:** individualCount: 1; sex: male; **Location:** locationID: O1; continent: Europe; country: Spain; countryCode: ES; stateProvince: Aragón; county: Huesca; locality: O Furno; verbatimElevation: 1396.73; decimalLatitude: 42.60677; decimalLongitude: 0.13135; geodeticDatum: WGS84; **Event:** eventID: 1; samplingProtocol: Beating; eventTime: Day
- **Type status:** Other material. **Occurrence:** individualCount: 5; sex: male; **Location:** locationID: O1; continent: Europe; country: Spain; countryCode: ES; stateProvince: Aragón; county: Huesca; locality: O Furno; verbatimElevation: 1396.73; decimalLatitude: 42.60677; decimalLongitude: 0.13135; geodeticDatum: WGS84; **Event:** eventID: A; samplingProtocol: Pitfall
- **Type status:** Other material. **Occurrence:** individualCount: 1; sex: male; **Location:** locationID: O1; continent: Europe; country: Spain; countryCode: ES; stateProvince: Aragón; county: Huesca; locality: O Furno; verbatimElevation: 1396.73; decimalLatitude: 42.60677; decimalLongitude: 0.13135; geodeticDatum: WGS84; **Event:** eventID: D; samplingProtocol: Pitfall
- **Type status:** Other material. **Occurrence:** individualCount: 6; sex: male; **Location:** locationID: O1; continent: Europe; country: Spain; countryCode: ES; stateProvince: Aragón; county: Huesca; locality: O Furno; verbatimElevation: 1396.73; decimalLatitude: 42.60677; decimalLongitude: 0.13135; geodeticDatum: WGS84; **Event:** eventID: K; samplingProtocol: Pitfall

##### Distribution

Europe

#### Typhochrestus bogarti

Bosmans, 1990

##### Materials

- **Type status:** Other material. **Occurrence:** individualCount: 1; sex: female; **Location:** locationID: C1; continent: Europe; country: Spain; countryCode: ES; stateProvince: Castilla-La Mancha; county: Ciudad Real; locality: Valle Brezoso; verbatimElevation: 756.56; decimalLatitude: 39.35663; decimalLongitude: -4.35912; geodeticDatum: WGS84; **Event:** eventID: B; samplingProtocol: Pitfall
- **Type status:** Other material. **Occurrence:** individualCount: 1; sex: female; **Location:** locationID: C1; continent: Europe; country: Spain; countryCode: ES; stateProvince: Castilla-La Mancha; county: Ciudad Real; locality: Valle Brezoso; verbatimElevation: 756.56; decimalLatitude: 39.35663; decimalLongitude: -4.35912; geodeticDatum: WGS84; **Event:** eventID: C; samplingProtocol: Pitfall
- **Type status:** Other material. **Occurrence:** individualCount: 1; sex: female; **Location:** locationID: C1; continent: Europe; country: Spain; countryCode: ES; stateProvince: Castilla-La Mancha; county: Ciudad Real; locality: Valle Brezoso; verbatimElevation: 756.56; decimalLatitude: 39.35663; decimalLongitude: -4.35912; geodeticDatum: WGS84; **Event:** eventID: K; samplingProtocol: Pitfall
- **Type status:** Other material. **Occurrence:** individualCount: 1; sex: female; **Location:** locationID: C2; continent: Europe; country: Spain; countryCode: ES; stateProvince: Castilla-La Mancha; county: Ciudad Real; locality: Valle Brezoso; verbatimElevation: 739.31; decimalLatitude: 39.35159; decimalLongitude: -4.3589; geodeticDatum: WGS84; **Event:** eventID: J; samplingProtocol: Pitfall
- **Type status:** Other material. **Occurrence:** individualCount: 1; sex: female; **Location:** locationID: C2; continent: Europe; country: Spain; countryCode: ES; stateProvince: Castilla-La Mancha; county: Ciudad Real; locality: Valle Brezoso; verbatimElevation: 739.31; decimalLatitude: 39.35159; decimalLongitude: -4.3589; geodeticDatum: WGS84; **Event:** eventID: 2; samplingProtocol: Sweeping; eventTime: Day
- **Type status:** Other material. **Occurrence:** individualCount: 1; sex: male; **Location:** locationID: M1; continent: Europe; country: Spain; countryCode: ES; stateProvince: Extremadura; county: Cáceres; locality: Peña Falcón; verbatimElevation: 320.6; decimalLatitude: 39.83296; decimalLongitude: -6.0641; geodeticDatum: WGS84; **Event:** eventID: E; samplingProtocol: Pitfall
- **Type status:** Other material. **Occurrence:** individualCount: 1; sex: female; **Location:** locationID: M2; continent: Europe; country: Spain; countryCode: ES; stateProvince: Extremadura; county: Cáceres; locality: Fuente del Frances; verbatimElevation: 320.72; decimalLatitude: 39.828; decimalLongitude: -6.03249; geodeticDatum: WGS84; **Event:** eventID: 1; samplingProtocol: Sweeping; eventTime: Day

##### Distribution

Iberian Peninsula, France, Morocco

#### Walckenaeria acuminata

Blackwall, 1833

##### Materials

- **Type status:** Other material. **Occurrence:** individualCount: 1; sex: female; **Location:** locationID: P1; continent: Europe; country: Spain; countryCode: ES; stateProvince: Castilla y León; county: León; locality: Monte Robledo; verbatimElevation: 1071.58; decimalLatitude: 43.1445; decimalLongitude: -4.92675; geodeticDatum: WGS84; **Event:** eventID: J; samplingProtocol: Pitfall

##### Distribution

Palearctic

#### Walckenaeria antica

(Wider, 1834)

##### Materials

- **Type status:** Other material. **Occurrence:** individualCount: 1; sex: male; **Location:** locationID: P2; continent: Europe; country: Spain; countryCode: ES; stateProvince: Castilla y León; county: León; locality: Joyoguelas; verbatimElevation: 763.98; decimalLatitude: 43.17771; decimalLongitude: -4.90579; geodeticDatum: WGS84; **Event:** eventID: L; samplingProtocol: Pitfall

##### Distribution

Palearctic

#### Walckenaeria corniculans

(O. Pickard-Cambridge, 1875)

##### Materials

- **Type status:** Other material. **Occurrence:** individualCount: 1; sex: male; **Location:** locationID: O1; continent: Europe; country: Spain; countryCode: ES; stateProvince: Aragón; county: Huesca; locality: O Furno; verbatimElevation: 1396.73; decimalLatitude: 42.60677; decimalLongitude: 0.13135; geodeticDatum: WGS84; **Event:** eventID: K; samplingProtocol: Pitfall
- **Type status:** Other material. **Occurrence:** individualCount: 1; sex: female; **Location:** locationID: P1; continent: Europe; country: Spain; countryCode: ES; stateProvince: Castilla y León; county: León; locality: Monte Robledo; verbatimElevation: 1071.58; decimalLatitude: 43.1445; decimalLongitude: -4.92675; geodeticDatum: WGS84; **Event:** eventID: D; samplingProtocol: Pitfall
- **Type status:** Other material. **Occurrence:** individualCount: 1; sex: female; **Location:** locationID: P1; continent: Europe; country: Spain; countryCode: ES; stateProvince: Castilla y León; county: León; locality: Monte Robledo; verbatimElevation: 1071.58; decimalLatitude: 43.1445; decimalLongitude: -4.92675; geodeticDatum: WGS84; **Event:** eventID: E; samplingProtocol: Pitfall
- **Type status:** Other material. **Occurrence:** individualCount: 1; sex: female; **Location:** locationID: P1; continent: Europe; country: Spain; countryCode: ES; stateProvince: Castilla y León; county: León; locality: Monte Robledo; verbatimElevation: 1071.58; decimalLatitude: 43.1445; decimalLongitude: -4.92675; geodeticDatum: WGS84; **Event:** eventID: F; samplingProtocol: Pitfall
- **Type status:** Other material. **Occurrence:** individualCount: 2; sex: female; **Location:** locationID: P1; continent: Europe; country: Spain; countryCode: ES; stateProvince: Castilla y León; county: León; locality: Monte Robledo; verbatimElevation: 1071.58; decimalLatitude: 43.1445; decimalLongitude: -4.92675; geodeticDatum: WGS84; **Event:** eventID: H; samplingProtocol: Pitfall
- **Type status:** Other material. **Occurrence:** individualCount: 6; sex: female; **Location:** locationID: P1; continent: Europe; country: Spain; countryCode: ES; stateProvince: Castilla y León; county: León; locality: Monte Robledo; verbatimElevation: 1071.58; decimalLatitude: 43.1445; decimalLongitude: -4.92675; geodeticDatum: WGS84; **Event:** eventID: J; samplingProtocol: Pitfall
- **Type status:** Other material. **Occurrence:** individualCount: 1; sex: female; **Location:** locationID: P1; continent: Europe; country: Spain; countryCode: ES; stateProvince: Castilla y León; county: León; locality: Monte Robledo; verbatimElevation: 1071.58; decimalLatitude: 43.1445; decimalLongitude: -4.92675; geodeticDatum: WGS84; **Event:** eventID: K; samplingProtocol: Pitfall
- **Type status:** Other material. **Occurrence:** individualCount: 1; sex: female; **Location:** locationID: P2; continent: Europe; country: Spain; countryCode: ES; stateProvince: Castilla y León; county: León; locality: Joyoguelas; verbatimElevation: 763.98; decimalLatitude: 43.17771; decimalLongitude: -4.90579; geodeticDatum: WGS84; **Event:** eventID: D; samplingProtocol: Pitfall
- **Type status:** Other material. **Occurrence:** individualCount: 2; sex: female; **Location:** locationID: P2; continent: Europe; country: Spain; countryCode: ES; stateProvince: Castilla y León; county: León; locality: Joyoguelas; verbatimElevation: 763.98; decimalLatitude: 43.17771; decimalLongitude: -4.90579; geodeticDatum: WGS84; **Event:** eventID: G; samplingProtocol: Pitfall
- **Type status:** Other material. **Occurrence:** individualCount: 1; sex: female; **Location:** locationID: P2; continent: Europe; country: Spain; countryCode: ES; stateProvince: Castilla y León; county: León; locality: Joyoguelas; verbatimElevation: 763.98; decimalLatitude: 43.17771; decimalLongitude: -4.90579; geodeticDatum: WGS84; **Event:** eventID: I; samplingProtocol: Pitfall
- **Type status:** Other material. **Occurrence:** individualCount: 1; sex: female; **Location:** locationID: P2; continent: Europe; country: Spain; countryCode: ES; stateProvince: Castilla y León; county: León; locality: Joyoguelas; verbatimElevation: 763.98; decimalLatitude: 43.17771; decimalLongitude: -4.90579; geodeticDatum: WGS84; **Event:** eventID: J; samplingProtocol: Pitfall
- **Type status:** Other material. **Occurrence:** individualCount: 1; sex: female; **Location:** locationID: P2; continent: Europe; country: Spain; countryCode: ES; stateProvince: Castilla y León; county: León; locality: Joyoguelas; verbatimElevation: 763.98; decimalLatitude: 43.17771; decimalLongitude: -4.90579; geodeticDatum: WGS84; **Event:** eventID: K; samplingProtocol: Pitfall
- **Type status:** Other material. **Occurrence:** individualCount: 1; sex: female; **Location:** locationID: P3; continent: Europe; country: Spain; countryCode: ES; stateProvince: Castilla y León; county: León; locality: Las Arroyas; verbatimElevation: 1097.1; decimalLatitude: 43.14351; decimalLongitude: -4.94878; geodeticDatum: WGS84; **Event:** eventID: C; samplingProtocol: Pitfall
- **Type status:** Other material. **Occurrence:** individualCount: 1; sex: female; **Location:** locationID: P3; continent: Europe; country: Spain; countryCode: ES; stateProvince: Castilla y León; county: León; locality: Las Arroyas; verbatimElevation: 1097.1; decimalLatitude: 43.14351; decimalLongitude: -4.94878; geodeticDatum: WGS84; **Event:** eventID: D; samplingProtocol: Pitfall
- **Type status:** Other material. **Occurrence:** individualCount: 1; sex: female; **Location:** locationID: P3; continent: Europe; country: Spain; countryCode: ES; stateProvince: Castilla y León; county: León; locality: Las Arroyas; verbatimElevation: 1097.1; decimalLatitude: 43.14351; decimalLongitude: -4.94878; geodeticDatum: WGS84; **Event:** eventID: F; samplingProtocol: Pitfall
- **Type status:** Other material. **Occurrence:** individualCount: 1; sex: female; **Location:** locationID: P3; continent: Europe; country: Spain; countryCode: ES; stateProvince: Castilla y León; county: León; locality: Las Arroyas; verbatimElevation: 1097.1; decimalLatitude: 43.14351; decimalLongitude: -4.94878; geodeticDatum: WGS84; **Event:** eventID: J; samplingProtocol: Pitfall
- **Type status:** Other material. **Occurrence:** individualCount: 1; sex: female; **Location:** locationID: P4; continent: Europe; country: Spain; countryCode: ES; stateProvince: Castilla y León; county: León; locality: El Canto; verbatimElevation: 943.48; decimalLatitude: 43.17227; decimalLongitude: -4.90857; geodeticDatum: WGS84; **Event:** eventID: C; samplingProtocol: Pitfall
- **Type status:** Other material. **Occurrence:** individualCount: 1; sex: female; **Location:** locationID: P4; continent: Europe; country: Spain; countryCode: ES; stateProvince: Castilla y León; county: León; locality: El Canto; verbatimElevation: 943.48; decimalLatitude: 43.17227; decimalLongitude: -4.90857; geodeticDatum: WGS84; **Event:** eventID: F; samplingProtocol: Pitfall
- **Type status:** Other material. **Occurrence:** individualCount: 1; sex: female; **Location:** locationID: P4; continent: Europe; country: Spain; countryCode: ES; stateProvince: Castilla y León; county: León; locality: El Canto; verbatimElevation: 943.48; decimalLatitude: 43.17227; decimalLongitude: -4.90857; geodeticDatum: WGS84; **Event:** eventID: G; samplingProtocol: Pitfall
- **Type status:** Other material. **Occurrence:** individualCount: 1; sex: female; **Location:** locationID: P4; continent: Europe; country: Spain; countryCode: ES; stateProvince: Castilla y León; county: León; locality: El Canto; verbatimElevation: 943.48; decimalLatitude: 43.17227; decimalLongitude: -4.90857; geodeticDatum: WGS84; **Event:** eventID: L; samplingProtocol: Pitfall
- **Type status:** Other material. **Occurrence:** individualCount: 1; sex: female; **Location:** locationID: S1; continent: Europe; country: Spain; countryCode: ES; stateProvince: Andalucía; county: Granada; locality: Soportujar; verbatimElevation: 1786.57; decimalLatitude: 36.96151; decimalLongitude: -3.41881; geodeticDatum: WGS84; **Event:** eventID: G; samplingProtocol: Pitfall

##### Distribution

Europe, North Africa

#### Walckenaeria dalmasi

(Simon, 1914)

##### Materials

- **Type status:** Other material. **Occurrence:** individualCount: 1; sex: female; **Location:** locationID: A2; continent: Europe; country: Spain; countryCode: ES; stateProvince: Catalonia; county: Lleida; locality: Sola de Boi; verbatimElevation: 1738.7; decimalLatitude: 42.54913; decimalLongitude: 0.87137; geodeticDatum: WGS84; **Event:** eventID: A; samplingProtocol: Pitfall
- **Type status:** Other material. **Occurrence:** individualCount: 2; sex: female; **Location:** locationID: P1; continent: Europe; country: Spain; countryCode: ES; stateProvince: Castilla y León; county: León; locality: Monte Robledo; verbatimElevation: 1071.58; decimalLatitude: 43.1445; decimalLongitude: -4.92675; geodeticDatum: WGS84; **Event:** eventID: F; samplingProtocol: Pitfall
- **Type status:** Other material. **Occurrence:** individualCount: 1; sex: female; **Location:** locationID: P1; continent: Europe; country: Spain; countryCode: ES; stateProvince: Castilla y León; county: León; locality: Monte Robledo; verbatimElevation: 1071.58; decimalLatitude: 43.1445; decimalLongitude: -4.92675; geodeticDatum: WGS84; **Event:** eventID: G; samplingProtocol: Pitfall
- **Type status:** Other material. **Occurrence:** individualCount: 1; sex: male; **Location:** locationID: P1; continent: Europe; country: Spain; countryCode: ES; stateProvince: Castilla y León; county: León; locality: Monte Robledo; verbatimElevation: 1071.58; decimalLatitude: 43.1445; decimalLongitude: -4.92675; geodeticDatum: WGS84; **Event:** eventID: H; samplingProtocol: Pitfall
- **Type status:** Other material. **Occurrence:** individualCount: 1; sex: female; **Location:** locationID: P1; continent: Europe; country: Spain; countryCode: ES; stateProvince: Castilla y León; county: León; locality: Monte Robledo; verbatimElevation: 1071.58; decimalLatitude: 43.1445; decimalLongitude: -4.92675; geodeticDatum: WGS84; **Event:** eventID: H; samplingProtocol: Pitfall
- **Type status:** Other material. **Occurrence:** individualCount: 1; sex: female; **Location:** locationID: P1; continent: Europe; country: Spain; countryCode: ES; stateProvince: Castilla y León; county: León; locality: Monte Robledo; verbatimElevation: 1071.58; decimalLatitude: 43.1445; decimalLongitude: -4.92675; geodeticDatum: WGS84; **Event:** eventID: J; samplingProtocol: Pitfall
- **Type status:** Other material. **Occurrence:** individualCount: 1; sex: female; **Location:** locationID: P1; continent: Europe; country: Spain; countryCode: ES; stateProvince: Castilla y León; county: León; locality: Monte Robledo; verbatimElevation: 1071.58; decimalLatitude: 43.1445; decimalLongitude: -4.92675; geodeticDatum: WGS84; **Event:** eventID: K; samplingProtocol: Pitfall

##### Distribution

Iberian Peninsula, France

#### Walckenaeria dysderoides

(Wider, 1834)

##### Materials

- **Type status:** Other material. **Occurrence:** individualCount: 2; sex: male; **Location:** locationID: P1; continent: Europe; country: Spain; countryCode: ES; stateProvince: Castilla y León; county: León; locality: Monte Robledo; verbatimElevation: 1071.58; decimalLatitude: 43.1445; decimalLongitude: -4.92675; geodeticDatum: WGS84; **Event:** eventID: J; samplingProtocol: Pitfall
- **Type status:** Other material. **Occurrence:** individualCount: 1; sex: male; **Location:** locationID: P3; continent: Europe; country: Spain; countryCode: ES; stateProvince: Castilla y León; county: León; locality: Las Arroyas; verbatimElevation: 1097.1; decimalLatitude: 43.14351; decimalLongitude: -4.94878; geodeticDatum: WGS84; **Event:** eventID: B; samplingProtocol: Pitfall

##### Distribution

Palearctic

#### Walckenaeria incisa

(O. Pickard-Cambridge, 1871)

##### Materials

- **Type status:** Other material. **Occurrence:** individualCount: 1; sex: female; **Location:** locationID: S2; continent: Europe; country: Spain; countryCode: ES; stateProvince: Andalucía; county: Granada; locality: Camarate; verbatimElevation: 1713.96; decimalLatitude: 37.18377; decimalLongitude: -3.26282; geodeticDatum: WGS84; **Event:** eventID: J; samplingProtocol: Pitfall

##### Distribution

Europe

#### Walckenaeria unicornis

O. Pickard-Cambridge, 1861

##### Materials

- **Type status:** Other material. **Occurrence:** individualCount: 1; sex: female; **Location:** locationID: P4; continent: Europe; country: Spain; countryCode: ES; stateProvince: Castilla y León; county: León; locality: El Canto; verbatimElevation: 943.48; decimalLatitude: 43.17227; decimalLongitude: -4.90857; geodeticDatum: WGS84; **Event:** eventID: 1; samplingProtocol: Beating; eventTime: Night
- **Type status:** Other material. **Occurrence:** individualCount: 1; sex: male; **Location:** locationID: P4; continent: Europe; country: Spain; countryCode: ES; stateProvince: Castilla y León; county: León; locality: El Canto; verbatimElevation: 943.48; decimalLatitude: 43.17227; decimalLongitude: -4.90857; geodeticDatum: WGS84; **Event:** eventID: E; samplingProtocol: Pitfall
- **Type status:** Other material. **Occurrence:** individualCount: 1; sex: male; **Location:** locationID: P4; continent: Europe; country: Spain; countryCode: ES; stateProvince: Castilla y León; county: León; locality: El Canto; verbatimElevation: 943.48; decimalLatitude: 43.17227; decimalLongitude: -4.90857; geodeticDatum: WGS84; **Event:** eventID: G; samplingProtocol: Pitfall
- **Type status:** Other material. **Occurrence:** individualCount: 1; sex: male; **Location:** locationID: P4; continent: Europe; country: Spain; countryCode: ES; stateProvince: Castilla y León; county: León; locality: El Canto; verbatimElevation: 943.48; decimalLatitude: 43.17227; decimalLongitude: -4.90857; geodeticDatum: WGS84; **Event:** eventID: H; samplingProtocol: Pitfall
- **Type status:** Other material. **Occurrence:** individualCount: 1; sex: male; **Location:** locationID: P4; continent: Europe; country: Spain; countryCode: ES; stateProvince: Castilla y León; county: León; locality: El Canto; verbatimElevation: 943.48; decimalLatitude: 43.17227; decimalLongitude: -4.90857; geodeticDatum: WGS84; **Event:** eventID: L; samplingProtocol: Pitfall
- **Type status:** Other material. **Occurrence:** individualCount: 1; sex: female; **Location:** locationID: P4; continent: Europe; country: Spain; countryCode: ES; stateProvince: Castilla y León; county: León; locality: El Canto; verbatimElevation: 943.48; decimalLatitude: 43.17227; decimalLongitude: -4.90857; geodeticDatum: WGS84; **Event:** eventID: 2; samplingProtocol: Sweeping; eventTime: Day

##### Distribution

Palearctic

#### Linyphiidae sp05

##### Materials

- **Type status:** Other material. **Occurrence:** individualCount: 1; sex: female; **Location:** locationID: O1; continent: Europe; country: Spain; countryCode: ES; stateProvince: Aragón; county: Huesca; locality: O Furno; verbatimElevation: 1396.73; decimalLatitude: 42.60677; decimalLongitude: 0.13135; geodeticDatum: WGS84; **Event:** eventID: 2; samplingProtocol: Aerial; eventTime: Night

##### Distribution

?

##### Notes

We were unable to identify this species or even its genus.

#### Liocranidae

Simon, 1897

#### Agroeca inopina

O. Pickard-Cambridge, 1886

##### Materials

- **Type status:** Other material. **Occurrence:** individualCount: 1; sex: female; **Location:** locationID: A2; continent: Europe; country: Spain; countryCode: ES; stateProvince: Catalonia; county: Lleida; locality: Sola de Boi; verbatimElevation: 1738.7; decimalLatitude: 42.54913; decimalLongitude: 0.87137; geodeticDatum: WGS84; **Event:** eventID: C; samplingProtocol: Pitfall
- **Type status:** Other material. **Occurrence:** individualCount: 1; sex: female; **Location:** locationID: C1; continent: Europe; country: Spain; countryCode: ES; stateProvince: Castilla-La Mancha; county: Ciudad Real; locality: Valle Brezoso; verbatimElevation: 756.56; decimalLatitude: 39.35663; decimalLongitude: -4.35912; geodeticDatum: WGS84; **Event:** eventID: G; samplingProtocol: Pitfall
- **Type status:** Other material. **Occurrence:** individualCount: 1; sex: female; **Location:** locationID: C1; continent: Europe; country: Spain; countryCode: ES; stateProvince: Castilla-La Mancha; county: Ciudad Real; locality: Valle Brezoso; verbatimElevation: 756.56; decimalLatitude: 39.35663; decimalLongitude: -4.35912; geodeticDatum: WGS84; **Event:** eventID: I; samplingProtocol: Pitfall
- **Type status:** Other material. **Occurrence:** individualCount: 1; sex: female; **Location:** locationID: C2; continent: Europe; country: Spain; countryCode: ES; stateProvince: Castilla-La Mancha; county: Ciudad Real; locality: Valle Brezoso; verbatimElevation: 739.31; decimalLatitude: 39.35159; decimalLongitude: -4.3589; geodeticDatum: WGS84; **Event:** eventID: H; samplingProtocol: Pitfall
- **Type status:** Other material. **Occurrence:** individualCount: 1; sex: female; **Location:** locationID: C3; continent: Europe; country: Spain; countryCode: ES; stateProvince: Castilla-La Mancha; county: Ciudad Real; locality: La Quesera; verbatimElevation: 767.55; decimalLatitude: 39.36177; decimalLongitude: -4.41733; geodeticDatum: WGS84; **Event:** eventID: L; samplingProtocol: Pitfall
- **Type status:** Other material. **Occurrence:** individualCount: 1; sex: female; **Location:** locationID: C4; continent: Europe; country: Spain; countryCode: ES; stateProvince: Castilla-La Mancha; county: Ciudad Real; locality: La Quesera; verbatimElevation: 772.3; decimalLatitude: 39.36337; decimalLongitude: -4.41704; geodeticDatum: WGS84; **Event:** eventID: A; samplingProtocol: Pitfall
- **Type status:** Other material. **Occurrence:** individualCount: 1; sex: female; **Location:** locationID: C4; continent: Europe; country: Spain; countryCode: ES; stateProvince: Castilla-La Mancha; county: Ciudad Real; locality: La Quesera; verbatimElevation: 772.3; decimalLatitude: 39.36337; decimalLongitude: -4.41704; geodeticDatum: WGS84; **Event:** eventID: D; samplingProtocol: Pitfall
- **Type status:** Other material. **Occurrence:** individualCount: 1; sex: female; **Location:** locationID: C4; continent: Europe; country: Spain; countryCode: ES; stateProvince: Castilla-La Mancha; county: Ciudad Real; locality: La Quesera; verbatimElevation: 772.3; decimalLatitude: 39.36337; decimalLongitude: -4.41704; geodeticDatum: WGS84; **Event:** eventID: G; samplingProtocol: Pitfall
- **Type status:** Other material. **Occurrence:** individualCount: 1; sex: female; **Location:** locationID: O1; continent: Europe; country: Spain; countryCode: ES; stateProvince: Aragón; county: Huesca; locality: O Furno; verbatimElevation: 1396.73; decimalLatitude: 42.60677; decimalLongitude: 0.13135; geodeticDatum: WGS84; **Event:** eventID: D; samplingProtocol: Pitfall
- **Type status:** Other material. **Occurrence:** individualCount: 1; sex: female; **Location:** locationID: O1; continent: Europe; country: Spain; countryCode: ES; stateProvince: Aragón; county: Huesca; locality: O Furno; verbatimElevation: 1396.73; decimalLatitude: 42.60677; decimalLongitude: 0.13135; geodeticDatum: WGS84; **Event:** eventID: L; samplingProtocol: Pitfall
- **Type status:** Other material. **Occurrence:** individualCount: 1; sex: female; **Location:** locationID: O2; continent: Europe; country: Spain; countryCode: ES; stateProvince: Aragón; county: Huesca; locality: Rebilla; verbatimElevation: 1158.13; decimalLatitude: 42.59427; decimalLongitude: 0.1529; geodeticDatum: WGS84; **Event:** eventID: A; samplingProtocol: Pitfall
- **Type status:** Other material. **Occurrence:** individualCount: 1; sex: female; **Location:** locationID: P1; continent: Europe; country: Spain; countryCode: ES; stateProvince: Castilla y León; county: León; locality: Monte Robledo; verbatimElevation: 1071.58; decimalLatitude: 43.1445; decimalLongitude: -4.92675; geodeticDatum: WGS84; **Event:** eventID: L; samplingProtocol: Pitfall
- **Type status:** Other material. **Occurrence:** individualCount: 1; sex: female; **Location:** locationID: P2; continent: Europe; country: Spain; countryCode: ES; stateProvince: Castilla y León; county: León; locality: Joyoguelas; verbatimElevation: 763.98; decimalLatitude: 43.17771; decimalLongitude: -4.90579; geodeticDatum: WGS84; **Event:** eventID: D; samplingProtocol: Pitfall
- **Type status:** Other material. **Occurrence:** individualCount: 1; sex: female; **Location:** locationID: P2; continent: Europe; country: Spain; countryCode: ES; stateProvince: Castilla y León; county: León; locality: Joyoguelas; verbatimElevation: 763.98; decimalLatitude: 43.17771; decimalLongitude: -4.90579; geodeticDatum: WGS84; **Event:** eventID: E; samplingProtocol: Pitfall
- **Type status:** Other material. **Occurrence:** individualCount: 1; sex: female; **Location:** locationID: P2; continent: Europe; country: Spain; countryCode: ES; stateProvince: Castilla y León; county: León; locality: Joyoguelas; verbatimElevation: 763.98; decimalLatitude: 43.17771; decimalLongitude: -4.90579; geodeticDatum: WGS84; **Event:** eventID: K; samplingProtocol: Pitfall
- **Type status:** Other material. **Occurrence:** individualCount: 1; sex: female; **Location:** locationID: P4; continent: Europe; country: Spain; countryCode: ES; stateProvince: Castilla y León; county: León; locality: El Canto; verbatimElevation: 943.48; decimalLatitude: 43.17227; decimalLongitude: -4.90857; geodeticDatum: WGS84; **Event:** eventID: B; samplingProtocol: Pitfall
- **Type status:** Other material. **Occurrence:** individualCount: 1; sex: female; **Location:** locationID: P4; continent: Europe; country: Spain; countryCode: ES; stateProvince: Castilla y León; county: León; locality: El Canto; verbatimElevation: 943.48; decimalLatitude: 43.17227; decimalLongitude: -4.90857; geodeticDatum: WGS84; **Event:** eventID: D; samplingProtocol: Pitfall
- **Type status:** Other material. **Occurrence:** individualCount: 1; sex: female; **Location:** locationID: P4; continent: Europe; country: Spain; countryCode: ES; stateProvince: Castilla y León; county: León; locality: El Canto; verbatimElevation: 943.48; decimalLatitude: 43.17227; decimalLongitude: -4.90857; geodeticDatum: WGS84; **Event:** eventID: G; samplingProtocol: Pitfall
- **Type status:** Other material. **Occurrence:** individualCount: 1; sex: female; **Location:** locationID: P4; continent: Europe; country: Spain; countryCode: ES; stateProvince: Castilla y León; county: León; locality: El Canto; verbatimElevation: 943.48; decimalLatitude: 43.17227; decimalLongitude: -4.90857; geodeticDatum: WGS84; **Event:** eventID: H; samplingProtocol: Pitfall
- **Type status:** Other material. **Occurrence:** individualCount: 1; sex: female; **Location:** locationID: P4; continent: Europe; country: Spain; countryCode: ES; stateProvince: Castilla y León; county: León; locality: El Canto; verbatimElevation: 943.48; decimalLatitude: 43.17227; decimalLongitude: -4.90857; geodeticDatum: WGS84; **Event:** eventID: L; samplingProtocol: Pitfall
- **Type status:** Other material. **Occurrence:** individualCount: 1; sex: female; **Location:** locationID: S2; continent: Europe; country: Spain; countryCode: ES; stateProvince: Andalucía; county: Granada; locality: Camarate; verbatimElevation: 1713.96; decimalLatitude: 37.18377; decimalLongitude: -3.26282; geodeticDatum: WGS84; **Event:** eventID: I; samplingProtocol: Pitfall

##### Distribution

Europe, Algeria

#### Agroeca sp41

##### Materials

- **Type status:** Other material. **Occurrence:** individualCount: 1; sex: female; **Location:** locationID: S2; continent: Europe; country: Spain; countryCode: ES; stateProvince: Andalucía; county: Granada; locality: Camarate; verbatimElevation: 1713.96; decimalLatitude: 37.18377; decimalLongitude: -3.26282; geodeticDatum: WGS84; **Event:** eventID: L; samplingProtocol: Pitfall

##### Distribution

?

##### Notes

This is a new species of *Agroeca* Westring, 1861, to be described in a future publication.

#### Liocranum cf. majus

Simon, 1878

##### Materials

- **Type status:** Other material. **Occurrence:** individualCount: 1; sex: female; **Location:** locationID: M1; continent: Europe; country: Spain; countryCode: ES; stateProvince: Extremadura; county: Cáceres; locality: Peña Falcón; verbatimElevation: 320.6; decimalLatitude: 39.83296; decimalLongitude: -6.0641; geodeticDatum: WGS84; **Event:** eventID: C; samplingProtocol: Pitfall
- **Type status:** Other material. **Occurrence:** individualCount: 1; sex: female; **Location:** locationID: M1; continent: Europe; country: Spain; countryCode: ES; stateProvince: Extremadura; county: Cáceres; locality: Peña Falcón; verbatimElevation: 320.6; decimalLatitude: 39.83296; decimalLongitude: -6.0641; geodeticDatum: WGS84; **Event:** eventID: D; samplingProtocol: Pitfall
- **Type status:** Other material. **Occurrence:** individualCount: 1; sex: female; **Location:** locationID: M1; continent: Europe; country: Spain; countryCode: ES; stateProvince: Extremadura; county: Cáceres; locality: Peña Falcón; verbatimElevation: 320.6; decimalLatitude: 39.83296; decimalLongitude: -6.0641; geodeticDatum: WGS84; **Event:** eventID: F; samplingProtocol: Pitfall
- **Type status:** Other material. **Occurrence:** individualCount: 2; sex: female; **Location:** locationID: M1; continent: Europe; country: Spain; countryCode: ES; stateProvince: Extremadura; county: Cáceres; locality: Peña Falcón; verbatimElevation: 320.6; decimalLatitude: 39.83296; decimalLongitude: -6.0641; geodeticDatum: WGS84; **Event:** eventID: L; samplingProtocol: Pitfall

##### Distribution

Spain

##### Notes

This species is very poorly described. Its identification is based on the small diagnosis given by Simon (Simon 1932: 938), in opposition to *L. segmentatum* Simon, 1878.

#### Liocranum rupicola

(Walckenaer, 1830)

##### Materials

- **Type status:** Other material. **Occurrence:** individualCount: 1; sex: male; **Location:** locationID: A1; continent: Europe; country: Spain; countryCode: ES; stateProvince: Catalonia; county: Lleida; locality: Sola de Boi; verbatimElevation: 1759.8; decimalLatitude: 42.54958; decimalLongitude: 0.87254; geodeticDatum: WGS84; **Event:** eventID: 1; samplingProtocol: Aerial; eventTime: Night
- **Type status:** Other material. **Occurrence:** individualCount: 1; sex: female; **Location:** locationID: A1; continent: Europe; country: Spain; countryCode: ES; stateProvince: Catalonia; county: Lleida; locality: Sola de Boi; verbatimElevation: 1759.8; decimalLatitude: 42.54958; decimalLongitude: 0.87254; geodeticDatum: WGS84; **Event:** eventID: 1; samplingProtocol: Ground; eventTime: Night
- **Type status:** Other material. **Occurrence:** individualCount: 1; sex: male; **Location:** locationID: A1; continent: Europe; country: Spain; countryCode: ES; stateProvince: Catalonia; county: Lleida; locality: Sola de Boi; verbatimElevation: 1759.8; decimalLatitude: 42.54958; decimalLongitude: 0.87254; geodeticDatum: WGS84; **Event:** eventID: 2; samplingProtocol: Ground; eventTime: Night
- **Type status:** Other material. **Occurrence:** individualCount: 1; sex: male; **Location:** locationID: A1; continent: Europe; country: Spain; countryCode: ES; stateProvince: Catalonia; county: Lleida; locality: Sola de Boi; verbatimElevation: 1759.8; decimalLatitude: 42.54958; decimalLongitude: 0.87254; geodeticDatum: WGS84; **Event:** eventID: A; samplingProtocol: Pitfall
- **Type status:** Other material. **Occurrence:** individualCount: 1; sex: female; **Location:** locationID: A1; continent: Europe; country: Spain; countryCode: ES; stateProvince: Catalonia; county: Lleida; locality: Sola de Boi; verbatimElevation: 1759.8; decimalLatitude: 42.54958; decimalLongitude: 0.87254; geodeticDatum: WGS84; **Event:** eventID: J; samplingProtocol: Pitfall
- **Type status:** Other material. **Occurrence:** individualCount: 1; sex: male; **Location:** locationID: A1; continent: Europe; country: Spain; countryCode: ES; stateProvince: Catalonia; county: Lleida; locality: Sola de Boi; verbatimElevation: 1759.8; decimalLatitude: 42.54958; decimalLongitude: 0.87254; geodeticDatum: WGS84; **Event:** eventID: J; samplingProtocol: Pitfall
- **Type status:** Other material. **Occurrence:** individualCount: 1; sex: male; **Location:** locationID: A1; continent: Europe; country: Spain; countryCode: ES; stateProvince: Catalonia; county: Lleida; locality: Sola de Boi; verbatimElevation: 1759.8; decimalLatitude: 42.54958; decimalLongitude: 0.87254; geodeticDatum: WGS84; **Event:** eventID: K; samplingProtocol: Pitfall
- **Type status:** Other material. **Occurrence:** individualCount: 1; sex: male; **Location:** locationID: A2; continent: Europe; country: Spain; countryCode: ES; stateProvince: Catalonia; county: Lleida; locality: Sola de Boi; verbatimElevation: 1738.7; decimalLatitude: 42.54913; decimalLongitude: 0.87137; geodeticDatum: WGS84; **Event:** eventID: 1; samplingProtocol: Aerial; eventTime: Night
- **Type status:** Other material. **Occurrence:** individualCount: 1; sex: female; **Location:** locationID: A2; continent: Europe; country: Spain; countryCode: ES; stateProvince: Catalonia; county: Lleida; locality: Sola de Boi; verbatimElevation: 1738.7; decimalLatitude: 42.54913; decimalLongitude: 0.87137; geodeticDatum: WGS84; **Event:** eventID: 1; samplingProtocol: Aerial; eventTime: Night
- **Type status:** Other material. **Occurrence:** individualCount: 1; sex: male; **Location:** locationID: A2; continent: Europe; country: Spain; countryCode: ES; stateProvince: Catalonia; county: Lleida; locality: Sola de Boi; verbatimElevation: 1738.7; decimalLatitude: 42.54913; decimalLongitude: 0.87137; geodeticDatum: WGS84; **Event:** eventID: 2; samplingProtocol: Aerial; eventTime: Night
- **Type status:** Other material. **Occurrence:** individualCount: 1; sex: male; **Location:** locationID: A2; continent: Europe; country: Spain; countryCode: ES; stateProvince: Catalonia; county: Lleida; locality: Sola de Boi; verbatimElevation: 1738.7; decimalLatitude: 42.54913; decimalLongitude: 0.87137; geodeticDatum: WGS84; **Event:** eventID: I; samplingProtocol: Pitfall
- **Type status:** Other material. **Occurrence:** individualCount: 1; sex: female; **Location:** locationID: P2; continent: Europe; country: Spain; countryCode: ES; stateProvince: Castilla y León; county: León; locality: Joyoguelas; verbatimElevation: 763.98; decimalLatitude: 43.17771; decimalLongitude: -4.90579; geodeticDatum: WGS84; **Event:** eventID: 1; samplingProtocol: Aerial; eventTime: Night
- **Type status:** Other material. **Occurrence:** individualCount: 1; sex: male; **Location:** locationID: P2; continent: Europe; country: Spain; countryCode: ES; stateProvince: Castilla y León; county: León; locality: Joyoguelas; verbatimElevation: 763.98; decimalLatitude: 43.17771; decimalLongitude: -4.90579; geodeticDatum: WGS84; **Event:** eventID: 2; samplingProtocol: Aerial; eventTime: Night
- **Type status:** Other material. **Occurrence:** individualCount: 2; sex: female; **Location:** locationID: P4; continent: Europe; country: Spain; countryCode: ES; stateProvince: Castilla y León; county: León; locality: El Canto; verbatimElevation: 943.48; decimalLatitude: 43.17227; decimalLongitude: -4.90857; geodeticDatum: WGS84; **Event:** eventID: 1; samplingProtocol: Aerial; eventTime: Night
- **Type status:** Other material. **Occurrence:** individualCount: 2; sex: male; **Location:** locationID: P4; continent: Europe; country: Spain; countryCode: ES; stateProvince: Castilla y León; county: León; locality: El Canto; verbatimElevation: 943.48; decimalLatitude: 43.17227; decimalLongitude: -4.90857; geodeticDatum: WGS84; **Event:** eventID: 2; samplingProtocol: Aerial; eventTime: Night
- **Type status:** Other material. **Occurrence:** individualCount: 1; sex: female; **Location:** locationID: P4; continent: Europe; country: Spain; countryCode: ES; stateProvince: Castilla y León; county: León; locality: El Canto; verbatimElevation: 943.48; decimalLatitude: 43.17227; decimalLongitude: -4.90857; geodeticDatum: WGS84; **Event:** eventID: 2; samplingProtocol: Aerial; eventTime: Night

##### Distribution

Europe, Russia

#### Liocranum sp38

##### Materials

- **Type status:** Other material. **Occurrence:** individualCount: 1; sex: female; **Location:** locationID: S2; continent: Europe; country: Spain; countryCode: ES; stateProvince: Andalucía; county: Granada; locality: Camarate; verbatimElevation: 1713.96; decimalLatitude: 37.18377; decimalLongitude: -3.26282; geodeticDatum: WGS84; **Event:** eventID: J; samplingProtocol: Pitfall

##### Distribution

?

##### Notes

This is a species of *Liocranum* L. Koch, 1866, which we were unable to identify.

#### Mesiotelus mauritanicus

Simon, 1909

##### Materials

- **Type status:** Other material. **Occurrence:** individualCount: 1; sex: male; **Location:** locationID: C2; continent: Europe; country: Spain; countryCode: ES; stateProvince: Castilla-La Mancha; county: Ciudad Real; locality: Valle Brezoso; verbatimElevation: 739.31; decimalLatitude: 39.35159; decimalLongitude: -4.3589; geodeticDatum: WGS84; **Event:** eventID: E; samplingProtocol: Pitfall
- **Type status:** Other material. **Occurrence:** individualCount: 1; sex: male; **Location:** locationID: M1; continent: Europe; country: Spain; countryCode: ES; stateProvince: Extremadura; county: Cáceres; locality: Peña Falcón; verbatimElevation: 320.6; decimalLatitude: 39.83296; decimalLongitude: -6.0641; geodeticDatum: WGS84; **Event:** eventID: B; samplingProtocol: Pitfall
- **Type status:** Other material. **Occurrence:** individualCount: 1; sex: male; **Location:** locationID: M1; continent: Europe; country: Spain; countryCode: ES; stateProvince: Extremadura; county: Cáceres; locality: Peña Falcón; verbatimElevation: 320.6; decimalLatitude: 39.83296; decimalLongitude: -6.0641; geodeticDatum: WGS84; **Event:** eventID: C; samplingProtocol: Pitfall
- **Type status:** Other material. **Occurrence:** individualCount: 1; sex: male; **Location:** locationID: M1; continent: Europe; country: Spain; countryCode: ES; stateProvince: Extremadura; county: Cáceres; locality: Peña Falcón; verbatimElevation: 320.6; decimalLatitude: 39.83296; decimalLongitude: -6.0641; geodeticDatum: WGS84; **Event:** eventID: G; samplingProtocol: Pitfall
- **Type status:** Other material. **Occurrence:** individualCount: 3; sex: male; **Location:** locationID: S2; continent: Europe; country: Spain; countryCode: ES; stateProvince: Andalucía; county: Granada; locality: Camarate; verbatimElevation: 1713.96; decimalLatitude: 37.18377; decimalLongitude: -3.26282; geodeticDatum: WGS84; **Event:** eventID: A; samplingProtocol: Pitfall
- **Type status:** Other material. **Occurrence:** individualCount: 1; sex: male; **Location:** locationID: S2; continent: Europe; country: Spain; countryCode: ES; stateProvince: Andalucía; county: Granada; locality: Camarate; verbatimElevation: 1713.96; decimalLatitude: 37.18377; decimalLongitude: -3.26282; geodeticDatum: WGS84; **Event:** eventID: B; samplingProtocol: Pitfall
- **Type status:** Other material. **Occurrence:** individualCount: 1; sex: female; **Location:** locationID: S2; continent: Europe; country: Spain; countryCode: ES; stateProvince: Andalucía; county: Granada; locality: Camarate; verbatimElevation: 1713.96; decimalLatitude: 37.18377; decimalLongitude: -3.26282; geodeticDatum: WGS84; **Event:** eventID: C; samplingProtocol: Pitfall
- **Type status:** Other material. **Occurrence:** individualCount: 4; sex: male; **Location:** locationID: S2; continent: Europe; country: Spain; countryCode: ES; stateProvince: Andalucía; county: Granada; locality: Camarate; verbatimElevation: 1713.96; decimalLatitude: 37.18377; decimalLongitude: -3.26282; geodeticDatum: WGS84; **Event:** eventID: I; samplingProtocol: Pitfall
- **Type status:** Other material. **Occurrence:** individualCount: 1; sex: male; **Location:** locationID: S2; continent: Europe; country: Spain; countryCode: ES; stateProvince: Andalucía; county: Granada; locality: Camarate; verbatimElevation: 1713.96; decimalLatitude: 37.18377; decimalLongitude: -3.26282; geodeticDatum: WGS84; **Event:** eventID: J; samplingProtocol: Pitfall
- **Type status:** Other material. **Occurrence:** individualCount: 1; sex: male; **Location:** locationID: S2; continent: Europe; country: Spain; countryCode: ES; stateProvince: Andalucía; county: Granada; locality: Camarate; verbatimElevation: 1713.96; decimalLatitude: 37.18377; decimalLongitude: -3.26282; geodeticDatum: WGS84; **Event:** eventID: K; samplingProtocol: Pitfall
- **Type status:** Other material. **Occurrence:** individualCount: 1; sex: male; **Location:** locationID: S2; continent: Europe; country: Spain; countryCode: ES; stateProvince: Andalucía; county: Granada; locality: Camarate; verbatimElevation: 1713.96; decimalLatitude: 37.18377; decimalLongitude: -3.26282; geodeticDatum: WGS84; **Event:** eventID: L; samplingProtocol: Pitfall

##### Distribution

Mediterranean

#### Mesiotelus tenuissimus

(L. Koch, 1866)

##### Materials

- **Type status:** Other material. **Occurrence:** individualCount: 1; sex: female; **Location:** locationID: S1; continent: Europe; country: Spain; countryCode: ES; stateProvince: Andalucía; county: Granada; locality: Soportujar; verbatimElevation: 1786.57; decimalLatitude: 36.96151; decimalLongitude: -3.41881; geodeticDatum: WGS84; **Event:** eventID: F; samplingProtocol: Pitfall

##### Distribution

Europe, North Africa, Turkmenistan

#### Scotina celans

(Blackwall, 1841)

##### Materials

- **Type status:** Other material. **Occurrence:** individualCount: 1; sex: female; **Location:** locationID: C1; continent: Europe; country: Spain; countryCode: ES; stateProvince: Castilla-La Mancha; county: Ciudad Real; locality: Valle Brezoso; verbatimElevation: 756.56; decimalLatitude: 39.35663; decimalLongitude: -4.35912; geodeticDatum: WGS84; **Event:** eventID: B; samplingProtocol: Pitfall
- **Type status:** Other material. **Occurrence:** individualCount: 1; sex: female; **Location:** locationID: C1; continent: Europe; country: Spain; countryCode: ES; stateProvince: Castilla-La Mancha; county: Ciudad Real; locality: Valle Brezoso; verbatimElevation: 756.56; decimalLatitude: 39.35663; decimalLongitude: -4.35912; geodeticDatum: WGS84; **Event:** eventID: F; samplingProtocol: Pitfall
- **Type status:** Other material. **Occurrence:** individualCount: 1; sex: female; **Location:** locationID: C3; continent: Europe; country: Spain; countryCode: ES; stateProvince: Castilla-La Mancha; county: Ciudad Real; locality: La Quesera; verbatimElevation: 767.55; decimalLatitude: 39.36177; decimalLongitude: -4.41733; geodeticDatum: WGS84; **Event:** eventID: F; samplingProtocol: Pitfall
- **Type status:** Other material. **Occurrence:** individualCount: 1; sex: female; **Location:** locationID: S2; continent: Europe; country: Spain; countryCode: ES; stateProvince: Andalucía; county: Granada; locality: Camarate; verbatimElevation: 1713.96; decimalLatitude: 37.18377; decimalLongitude: -3.26282; geodeticDatum: WGS84; **Event:** eventID: A; samplingProtocol: Pitfall

##### Distribution

Europe, Algeria, Russia

#### Scotina palliardii

(L. Koch, 1881)

##### Materials

- **Type status:** Other material. **Occurrence:** individualCount: 1; sex: female; **Location:** locationID: O2; continent: Europe; country: Spain; countryCode: ES; stateProvince: Aragón; county: Huesca; locality: Rebilla; verbatimElevation: 1158.13; decimalLatitude: 42.59427; decimalLongitude: 0.1529; geodeticDatum: WGS84; **Event:** eventID: A; samplingProtocol: Pitfall
- **Type status:** Other material. **Occurrence:** individualCount: 1; sex: male; **Location:** locationID: O2; continent: Europe; country: Spain; countryCode: ES; stateProvince: Aragón; county: Huesca; locality: Rebilla; verbatimElevation: 1158.13; decimalLatitude: 42.59427; decimalLongitude: 0.1529; geodeticDatum: WGS84; **Event:** eventID: F; samplingProtocol: Pitfall
- **Type status:** Other material. **Occurrence:** individualCount: 1; sex: female; **Location:** locationID: P2; continent: Europe; country: Spain; countryCode: ES; stateProvince: Castilla y León; county: León; locality: Joyoguelas; verbatimElevation: 763.98; decimalLatitude: 43.17771; decimalLongitude: -4.90579; geodeticDatum: WGS84; **Event:** eventID: C; samplingProtocol: Pitfall

##### Distribution

Europe, Russia, Korea

#### Lycosidae

Sundevall, 1833

#### Alopecosa albofasciata

(Brullé, 1832)

##### Materials

- **Type status:** Other material. **Occurrence:** individualCount: 1; sex: male; **Location:** locationID: C4; continent: Europe; country: Spain; countryCode: ES; stateProvince: Castilla-La Mancha; county: Ciudad Real; locality: La Quesera; verbatimElevation: 772.3; decimalLatitude: 39.36337; decimalLongitude: -4.41704; geodeticDatum: WGS84; **Event:** eventID: C; samplingProtocol: Pitfall
- **Type status:** Other material. **Occurrence:** individualCount: 3; sex: male; **Location:** locationID: O2; continent: Europe; country: Spain; countryCode: ES; stateProvince: Aragón; county: Huesca; locality: Rebilla; verbatimElevation: 1158.13; decimalLatitude: 42.59427; decimalLongitude: 0.1529; geodeticDatum: WGS84; **Event:** eventID: A; samplingProtocol: Pitfall
- **Type status:** Other material. **Occurrence:** individualCount: 1; sex: male; **Location:** locationID: O2; continent: Europe; country: Spain; countryCode: ES; stateProvince: Aragón; county: Huesca; locality: Rebilla; verbatimElevation: 1158.13; decimalLatitude: 42.59427; decimalLongitude: 0.1529; geodeticDatum: WGS84; **Event:** eventID: B; samplingProtocol: Pitfall
- **Type status:** Other material. **Occurrence:** individualCount: 4; sex: male; **Location:** locationID: O2; continent: Europe; country: Spain; countryCode: ES; stateProvince: Aragón; county: Huesca; locality: Rebilla; verbatimElevation: 1158.13; decimalLatitude: 42.59427; decimalLongitude: 0.1529; geodeticDatum: WGS84; **Event:** eventID: L; samplingProtocol: Pitfall
- **Type status:** Other material. **Occurrence:** individualCount: 1; sex: female; **Location:** locationID: O2; continent: Europe; country: Spain; countryCode: ES; stateProvince: Aragón; county: Huesca; locality: Rebilla; verbatimElevation: 1158.13; decimalLatitude: 42.59427; decimalLongitude: 0.1529; geodeticDatum: WGS84; **Event:** eventID: L; samplingProtocol: Pitfall
- **Type status:** Other material. **Occurrence:** individualCount: 1; sex: male; **Location:** locationID: O2; continent: Europe; country: Spain; countryCode: ES; stateProvince: Aragón; county: Huesca; locality: Rebilla; verbatimElevation: 1158.13; decimalLatitude: 42.59427; decimalLongitude: 0.1529; geodeticDatum: WGS84; **Event:** eventID: 1; samplingProtocol: Sweeping; eventTime: Day
- **Type status:** Other material. **Occurrence:** individualCount: 1; sex: male; **Location:** locationID: S1; continent: Europe; country: Spain; countryCode: ES; stateProvince: Andalucía; county: Granada; locality: Soportujar; verbatimElevation: 1786.57; decimalLatitude: 36.96151; decimalLongitude: -3.41881; geodeticDatum: WGS84; **Event:** eventID: D; samplingProtocol: Pitfall
- **Type status:** Other material. **Occurrence:** individualCount: 1; sex: male; **Location:** locationID: S1; continent: Europe; country: Spain; countryCode: ES; stateProvince: Andalucía; county: Granada; locality: Soportujar; verbatimElevation: 1786.57; decimalLatitude: 36.96151; decimalLongitude: -3.41881; geodeticDatum: WGS84; **Event:** eventID: E; samplingProtocol: Pitfall
- **Type status:** Other material. **Occurrence:** individualCount: 1; sex: male; **Location:** locationID: S1; continent: Europe; country: Spain; countryCode: ES; stateProvince: Andalucía; county: Granada; locality: Soportujar; verbatimElevation: 1786.57; decimalLatitude: 36.96151; decimalLongitude: -3.41881; geodeticDatum: WGS84; **Event:** eventID: F; samplingProtocol: Pitfall
- **Type status:** Other material. **Occurrence:** individualCount: 1; sex: male; **Location:** locationID: S1; continent: Europe; country: Spain; countryCode: ES; stateProvince: Andalucía; county: Granada; locality: Soportujar; verbatimElevation: 1786.57; decimalLatitude: 36.96151; decimalLongitude: -3.41881; geodeticDatum: WGS84; **Event:** eventID: I; samplingProtocol: Pitfall
- **Type status:** Other material. **Occurrence:** individualCount: 1; sex: male; **Location:** locationID: S1; continent: Europe; country: Spain; countryCode: ES; stateProvince: Andalucía; county: Granada; locality: Soportujar; verbatimElevation: 1786.57; decimalLatitude: 36.96151; decimalLongitude: -3.41881; geodeticDatum: WGS84; **Event:** eventID: J; samplingProtocol: Pitfall
- **Type status:** Other material. **Occurrence:** individualCount: 1; sex: male; **Location:** locationID: S2; continent: Europe; country: Spain; countryCode: ES; stateProvince: Andalucía; county: Granada; locality: Camarate; verbatimElevation: 1713.96; decimalLatitude: 37.18377; decimalLongitude: -3.26282; geodeticDatum: WGS84; **Event:** eventID: D; samplingProtocol: Pitfall
- **Type status:** Other material. **Occurrence:** individualCount: 1; sex: male; **Location:** locationID: S2; continent: Europe; country: Spain; countryCode: ES; stateProvince: Andalucía; county: Granada; locality: Camarate; verbatimElevation: 1713.96; decimalLatitude: 37.18377; decimalLongitude: -3.26282; geodeticDatum: WGS84; **Event:** eventID: E; samplingProtocol: Pitfall
- **Type status:** Other material. **Occurrence:** individualCount: 1; sex: male; **Location:** locationID: S2; continent: Europe; country: Spain; countryCode: ES; stateProvince: Andalucía; county: Granada; locality: Camarate; verbatimElevation: 1713.96; decimalLatitude: 37.18377; decimalLongitude: -3.26282; geodeticDatum: WGS84; **Event:** eventID: H; samplingProtocol: Pitfall
- **Type status:** Other material. **Occurrence:** individualCount: 1; sex: male; **Location:** locationID: S2; continent: Europe; country: Spain; countryCode: ES; stateProvince: Andalucía; county: Granada; locality: Camarate; verbatimElevation: 1713.96; decimalLatitude: 37.18377; decimalLongitude: -3.26282; geodeticDatum: WGS84; **Event:** eventID: K; samplingProtocol: Pitfall

##### Distribution

Mediterranean to Central Asia

#### Alopecosa barbipes

(Sundevall, 1833)

##### Materials

- **Type status:** Other material. **Occurrence:** individualCount: 1; sex: male; **Location:** locationID: P4; continent: Europe; country: Spain; countryCode: ES; stateProvince: Castilla y León; county: León; locality: El Canto; verbatimElevation: 943.48; decimalLatitude: 43.17227; decimalLongitude: -4.90857; geodeticDatum: WGS84; **Event:** eventID: C; samplingProtocol: Pitfall
- **Type status:** Other material. **Occurrence:** individualCount: 1; sex: male; **Location:** locationID: P4; continent: Europe; country: Spain; countryCode: ES; stateProvince: Castilla y León; county: León; locality: El Canto; verbatimElevation: 943.48; decimalLatitude: 43.17227; decimalLongitude: -4.90857; geodeticDatum: WGS84; **Event:** eventID: F; samplingProtocol: Pitfall
- **Type status:** Other material. **Occurrence:** individualCount: 1; sex: male; **Location:** locationID: P4; continent: Europe; country: Spain; countryCode: ES; stateProvince: Castilla y León; county: León; locality: El Canto; verbatimElevation: 943.48; decimalLatitude: 43.17227; decimalLongitude: -4.90857; geodeticDatum: WGS84; **Event:** eventID: L; samplingProtocol: Pitfall

##### Distribution

Palearctic

#### Alopecosa cuneata

(Clerck, 1757)

##### Materials

- **Type status:** Other material. **Occurrence:** individualCount: 1; sex: male; **Location:** locationID: P4; continent: Europe; country: Spain; countryCode: ES; stateProvince: Castilla y León; county: León; locality: El Canto; verbatimElevation: 943.48; decimalLatitude: 43.17227; decimalLongitude: -4.90857; geodeticDatum: WGS84; **Event:** eventID: C; samplingProtocol: Pitfall

##### Distribution

Palearctic

#### Alopecosa pulverulenta

(Clerck, 1757)

##### Materials

- **Type status:** Other material. **Occurrence:** individualCount: 1; sex: male; **Location:** locationID: O1; continent: Europe; country: Spain; countryCode: ES; stateProvince: Aragón; county: Huesca; locality: O Furno; verbatimElevation: 1396.73; decimalLatitude: 42.60677; decimalLongitude: 0.13135; geodeticDatum: WGS84; **Event:** eventID: D; samplingProtocol: Pitfall
- **Type status:** Other material. **Occurrence:** individualCount: 6; sex: male; **Location:** locationID: P2; continent: Europe; country: Spain; countryCode: ES; stateProvince: Castilla y León; county: León; locality: Joyoguelas; verbatimElevation: 763.98; decimalLatitude: 43.17771; decimalLongitude: -4.90579; geodeticDatum: WGS84; **Event:** eventID: B; samplingProtocol: Pitfall
- **Type status:** Other material. **Occurrence:** individualCount: 5; sex: female; **Location:** locationID: P2; continent: Europe; country: Spain; countryCode: ES; stateProvince: Castilla y León; county: León; locality: Joyoguelas; verbatimElevation: 763.98; decimalLatitude: 43.17771; decimalLongitude: -4.90579; geodeticDatum: WGS84; **Event:** eventID: C; samplingProtocol: Pitfall
- **Type status:** Other material. **Occurrence:** individualCount: 10; sex: male; **Location:** locationID: P2; continent: Europe; country: Spain; countryCode: ES; stateProvince: Castilla y León; county: León; locality: Joyoguelas; verbatimElevation: 763.98; decimalLatitude: 43.17771; decimalLongitude: -4.90579; geodeticDatum: WGS84; **Event:** eventID: C; samplingProtocol: Pitfall
- **Type status:** Other material. **Occurrence:** individualCount: 2; sex: female; **Location:** locationID: P2; continent: Europe; country: Spain; countryCode: ES; stateProvince: Castilla y León; county: León; locality: Joyoguelas; verbatimElevation: 763.98; decimalLatitude: 43.17771; decimalLongitude: -4.90579; geodeticDatum: WGS84; **Event:** eventID: D; samplingProtocol: Pitfall
- **Type status:** Other material. **Occurrence:** individualCount: 6; sex: male; **Location:** locationID: P2; continent: Europe; country: Spain; countryCode: ES; stateProvince: Castilla y León; county: León; locality: Joyoguelas; verbatimElevation: 763.98; decimalLatitude: 43.17771; decimalLongitude: -4.90579; geodeticDatum: WGS84; **Event:** eventID: D; samplingProtocol: Pitfall
- **Type status:** Other material. **Occurrence:** individualCount: 1; sex: male; **Location:** locationID: P2; continent: Europe; country: Spain; countryCode: ES; stateProvince: Castilla y León; county: León; locality: Joyoguelas; verbatimElevation: 763.98; decimalLatitude: 43.17771; decimalLongitude: -4.90579; geodeticDatum: WGS84; **Event:** eventID: E; samplingProtocol: Pitfall
- **Type status:** Other material. **Occurrence:** individualCount: 1; sex: male; **Location:** locationID: P2; continent: Europe; country: Spain; countryCode: ES; stateProvince: Castilla y León; county: León; locality: Joyoguelas; verbatimElevation: 763.98; decimalLatitude: 43.17771; decimalLongitude: -4.90579; geodeticDatum: WGS84; **Event:** eventID: F; samplingProtocol: Pitfall
- **Type status:** Other material. **Occurrence:** individualCount: 5; sex: male; **Location:** locationID: P2; continent: Europe; country: Spain; countryCode: ES; stateProvince: Castilla y León; county: León; locality: Joyoguelas; verbatimElevation: 763.98; decimalLatitude: 43.17771; decimalLongitude: -4.90579; geodeticDatum: WGS84; **Event:** eventID: G; samplingProtocol: Pitfall
- **Type status:** Other material. **Occurrence:** individualCount: 7; sex: male; **Location:** locationID: P2; continent: Europe; country: Spain; countryCode: ES; stateProvince: Castilla y León; county: León; locality: Joyoguelas; verbatimElevation: 763.98; decimalLatitude: 43.17771; decimalLongitude: -4.90579; geodeticDatum: WGS84; **Event:** eventID: H; samplingProtocol: Pitfall
- **Type status:** Other material. **Occurrence:** individualCount: 4; sex: male; **Location:** locationID: P2; continent: Europe; country: Spain; countryCode: ES; stateProvince: Castilla y León; county: León; locality: Joyoguelas; verbatimElevation: 763.98; decimalLatitude: 43.17771; decimalLongitude: -4.90579; geodeticDatum: WGS84; **Event:** eventID: I; samplingProtocol: Pitfall
- **Type status:** Other material. **Occurrence:** individualCount: 6; sex: male; **Location:** locationID: P2; continent: Europe; country: Spain; countryCode: ES; stateProvince: Castilla y León; county: León; locality: Joyoguelas; verbatimElevation: 763.98; decimalLatitude: 43.17771; decimalLongitude: -4.90579; geodeticDatum: WGS84; **Event:** eventID: J; samplingProtocol: Pitfall
- **Type status:** Other material. **Occurrence:** individualCount: 3; sex: male; **Location:** locationID: P2; continent: Europe; country: Spain; countryCode: ES; stateProvince: Castilla y León; county: León; locality: Joyoguelas; verbatimElevation: 763.98; decimalLatitude: 43.17771; decimalLongitude: -4.90579; geodeticDatum: WGS84; **Event:** eventID: K; samplingProtocol: Pitfall
- **Type status:** Other material. **Occurrence:** individualCount: 1; sex: male; **Location:** locationID: P3; continent: Europe; country: Spain; countryCode: ES; stateProvince: Castilla y León; county: León; locality: Las Arroyas; verbatimElevation: 1097.1; decimalLatitude: 43.14351; decimalLongitude: -4.94878; geodeticDatum: WGS84; **Event:** eventID: L; samplingProtocol: Pitfall
- **Type status:** Other material. **Occurrence:** individualCount: 1; sex: male; **Location:** locationID: P4; continent: Europe; country: Spain; countryCode: ES; stateProvince: Castilla y León; county: León; locality: El Canto; verbatimElevation: 943.48; decimalLatitude: 43.17227; decimalLongitude: -4.90857; geodeticDatum: WGS84; **Event:** eventID: B; samplingProtocol: Pitfall
- **Type status:** Other material. **Occurrence:** individualCount: 6; sex: male; **Location:** locationID: P4; continent: Europe; country: Spain; countryCode: ES; stateProvince: Castilla y León; county: León; locality: El Canto; verbatimElevation: 943.48; decimalLatitude: 43.17227; decimalLongitude: -4.90857; geodeticDatum: WGS84; **Event:** eventID: C; samplingProtocol: Pitfall
- **Type status:** Other material. **Occurrence:** individualCount: 1; sex: male; **Location:** locationID: P4; continent: Europe; country: Spain; countryCode: ES; stateProvince: Castilla y León; county: León; locality: El Canto; verbatimElevation: 943.48; decimalLatitude: 43.17227; decimalLongitude: -4.90857; geodeticDatum: WGS84; **Event:** eventID: D; samplingProtocol: Pitfall
- **Type status:** Other material. **Occurrence:** individualCount: 3; sex: male; **Location:** locationID: P4; continent: Europe; country: Spain; countryCode: ES; stateProvince: Castilla y León; county: León; locality: El Canto; verbatimElevation: 943.48; decimalLatitude: 43.17227; decimalLongitude: -4.90857; geodeticDatum: WGS84; **Event:** eventID: E; samplingProtocol: Pitfall
- **Type status:** Other material. **Occurrence:** individualCount: 1; sex: male; **Location:** locationID: P4; continent: Europe; country: Spain; countryCode: ES; stateProvince: Castilla y León; county: León; locality: El Canto; verbatimElevation: 943.48; decimalLatitude: 43.17227; decimalLongitude: -4.90857; geodeticDatum: WGS84; **Event:** eventID: F; samplingProtocol: Pitfall
- **Type status:** Other material. **Occurrence:** individualCount: 1; sex: male; **Location:** locationID: P4; continent: Europe; country: Spain; countryCode: ES; stateProvince: Castilla y León; county: León; locality: El Canto; verbatimElevation: 943.48; decimalLatitude: 43.17227; decimalLongitude: -4.90857; geodeticDatum: WGS84; **Event:** eventID: G; samplingProtocol: Pitfall
- **Type status:** Other material. **Occurrence:** individualCount: 1; sex: male; **Location:** locationID: P4; continent: Europe; country: Spain; countryCode: ES; stateProvince: Castilla y León; county: León; locality: El Canto; verbatimElevation: 943.48; decimalLatitude: 43.17227; decimalLongitude: -4.90857; geodeticDatum: WGS84; **Event:** eventID: H; samplingProtocol: Pitfall
- **Type status:** Other material. **Occurrence:** individualCount: 1; sex: male; **Location:** locationID: P4; continent: Europe; country: Spain; countryCode: ES; stateProvince: Castilla y León; county: León; locality: El Canto; verbatimElevation: 943.48; decimalLatitude: 43.17227; decimalLongitude: -4.90857; geodeticDatum: WGS84; **Event:** eventID: J; samplingProtocol: Pitfall

##### Distribution

Palearctic

#### Alopecosa simoni

(Thorell, 1872)

##### Materials

- **Type status:** Other material. **Occurrence:** individualCount: 1; sex: female; **Location:** locationID: S1; continent: Europe; country: Spain; countryCode: ES; stateProvince: Andalucía; county: Granada; locality: Soportujar; verbatimElevation: 1786.57; decimalLatitude: 36.96151; decimalLongitude: -3.41881; geodeticDatum: WGS84; **Event:** eventID: F; samplingProtocol: Pitfall

##### Distribution

Mediterranean

#### Arctosa lacustris

(Simon, 1876)

##### Materials

- **Type status:** Other material. **Occurrence:** individualCount: 1; sex: male; **Location:** locationID: C4; continent: Europe; country: Spain; countryCode: ES; stateProvince: Castilla-La Mancha; county: Ciudad Real; locality: La Quesera; verbatimElevation: 772.3; decimalLatitude: 39.36337; decimalLongitude: -4.41704; geodeticDatum: WGS84; **Event:** eventID: A; samplingProtocol: Pitfall

##### Distribution

Canary Islands, Mallorca, Mediterranean

#### Arctosa personata

(L. Koch, 1872)

##### Materials

- **Type status:** Other material. **Occurrence:** individualCount: 1; sex: female; **Location:** locationID: O2; continent: Europe; country: Spain; countryCode: ES; stateProvince: Aragón; county: Huesca; locality: Rebilla; verbatimElevation: 1158.13; decimalLatitude: 42.59427; decimalLongitude: 0.1529; geodeticDatum: WGS84; **Event:** eventID: B; samplingProtocol: Pitfall
- **Type status:** Other material. **Occurrence:** individualCount: 1; sex: male; **Location:** locationID: O2; continent: Europe; country: Spain; countryCode: ES; stateProvince: Aragón; county: Huesca; locality: Rebilla; verbatimElevation: 1158.13; decimalLatitude: 42.59427; decimalLongitude: 0.1529; geodeticDatum: WGS84; **Event:** eventID: D; samplingProtocol: Pitfall

##### Distribution

Western Mediterranean

#### Arctosa variana

C. L. Koch, 1847

##### Materials

- **Type status:** Other material. **Occurrence:** individualCount: 1; sex: female; **Location:** locationID: C1; continent: Europe; country: Spain; countryCode: ES; stateProvince: Castilla-La Mancha; county: Ciudad Real; locality: Valle Brezoso; verbatimElevation: 756.56; decimalLatitude: 39.35663; decimalLongitude: -4.35912; geodeticDatum: WGS84; **Event:** eventID: A; samplingProtocol: Pitfall

##### Distribution

Mediterranean to Central Asia

#### Arctosa villica

(Lucas, 1846)

##### Materials

- **Type status:** Other material. **Occurrence:** individualCount: 3; sex: female; **Location:** locationID: C1; continent: Europe; country: Spain; countryCode: ES; stateProvince: Castilla-La Mancha; county: Ciudad Real; locality: Valle Brezoso; verbatimElevation: 756.56; decimalLatitude: 39.35663; decimalLongitude: -4.35912; geodeticDatum: WGS84; **Event:** eventID: C; samplingProtocol: Pitfall
- **Type status:** Other material. **Occurrence:** individualCount: 1; sex: female; **Location:** locationID: C1; continent: Europe; country: Spain; countryCode: ES; stateProvince: Castilla-La Mancha; county: Ciudad Real; locality: Valle Brezoso; verbatimElevation: 756.56; decimalLatitude: 39.35663; decimalLongitude: -4.35912; geodeticDatum: WGS84; **Event:** eventID: D; samplingProtocol: Pitfall
- **Type status:** Other material. **Occurrence:** individualCount: 1; sex: female; **Location:** locationID: C1; continent: Europe; country: Spain; countryCode: ES; stateProvince: Castilla-La Mancha; county: Ciudad Real; locality: Valle Brezoso; verbatimElevation: 756.56; decimalLatitude: 39.35663; decimalLongitude: -4.35912; geodeticDatum: WGS84; **Event:** eventID: E; samplingProtocol: Pitfall
- **Type status:** Other material. **Occurrence:** individualCount: 1; sex: female; **Location:** locationID: C1; continent: Europe; country: Spain; countryCode: ES; stateProvince: Castilla-La Mancha; county: Ciudad Real; locality: Valle Brezoso; verbatimElevation: 756.56; decimalLatitude: 39.35663; decimalLongitude: -4.35912; geodeticDatum: WGS84; **Event:** eventID: F; samplingProtocol: Pitfall
- **Type status:** Other material. **Occurrence:** individualCount: 1; sex: female; **Location:** locationID: C1; continent: Europe; country: Spain; countryCode: ES; stateProvince: Castilla-La Mancha; county: Ciudad Real; locality: Valle Brezoso; verbatimElevation: 756.56; decimalLatitude: 39.35663; decimalLongitude: -4.35912; geodeticDatum: WGS84; **Event:** eventID: G; samplingProtocol: Pitfall
- **Type status:** Other material. **Occurrence:** individualCount: 1; sex: female; **Location:** locationID: C1; continent: Europe; country: Spain; countryCode: ES; stateProvince: Castilla-La Mancha; county: Ciudad Real; locality: Valle Brezoso; verbatimElevation: 756.56; decimalLatitude: 39.35663; decimalLongitude: -4.35912; geodeticDatum: WGS84; **Event:** eventID: H; samplingProtocol: Pitfall
- **Type status:** Other material. **Occurrence:** individualCount: 2; sex: female; **Location:** locationID: C1; continent: Europe; country: Spain; countryCode: ES; stateProvince: Castilla-La Mancha; county: Ciudad Real; locality: Valle Brezoso; verbatimElevation: 756.56; decimalLatitude: 39.35663; decimalLongitude: -4.35912; geodeticDatum: WGS84; **Event:** eventID: K; samplingProtocol: Pitfall
- **Type status:** Other material. **Occurrence:** individualCount: 2; sex: female; **Location:** locationID: C1; continent: Europe; country: Spain; countryCode: ES; stateProvince: Castilla-La Mancha; county: Ciudad Real; locality: Valle Brezoso; verbatimElevation: 756.56; decimalLatitude: 39.35663; decimalLongitude: -4.35912; geodeticDatum: WGS84; **Event:** eventID: L; samplingProtocol: Pitfall
- **Type status:** Other material. **Occurrence:** individualCount: 1; sex: female; **Location:** locationID: C2; continent: Europe; country: Spain; countryCode: ES; stateProvince: Castilla-La Mancha; county: Ciudad Real; locality: Valle Brezoso; verbatimElevation: 739.31; decimalLatitude: 39.35159; decimalLongitude: -4.3589; geodeticDatum: WGS84; **Event:** eventID: L; samplingProtocol: Pitfall
- **Type status:** Other material. **Occurrence:** individualCount: 1; sex: female; **Location:** locationID: C4; continent: Europe; country: Spain; countryCode: ES; stateProvince: Castilla-La Mancha; county: Ciudad Real; locality: La Quesera; verbatimElevation: 772.3; decimalLatitude: 39.36337; decimalLongitude: -4.41704; geodeticDatum: WGS84; **Event:** eventID: A; samplingProtocol: Pitfall
- **Type status:** Other material. **Occurrence:** individualCount: 1; sex: female; **Location:** locationID: C4; continent: Europe; country: Spain; countryCode: ES; stateProvince: Castilla-La Mancha; county: Ciudad Real; locality: La Quesera; verbatimElevation: 772.3; decimalLatitude: 39.36337; decimalLongitude: -4.41704; geodeticDatum: WGS84; **Event:** eventID: B; samplingProtocol: Pitfall
- **Type status:** Other material. **Occurrence:** individualCount: 2; sex: female; **Location:** locationID: C4; continent: Europe; country: Spain; countryCode: ES; stateProvince: Castilla-La Mancha; county: Ciudad Real; locality: La Quesera; verbatimElevation: 772.3; decimalLatitude: 39.36337; decimalLongitude: -4.41704; geodeticDatum: WGS84; **Event:** eventID: H; samplingProtocol: Pitfall
- **Type status:** Other material. **Occurrence:** individualCount: 1; sex: female; **Location:** locationID: C4; continent: Europe; country: Spain; countryCode: ES; stateProvince: Castilla-La Mancha; county: Ciudad Real; locality: La Quesera; verbatimElevation: 772.3; decimalLatitude: 39.36337; decimalLongitude: -4.41704; geodeticDatum: WGS84; **Event:** eventID: K; samplingProtocol: Pitfall
- **Type status:** Other material. **Occurrence:** individualCount: 1; sex: female; **Location:** locationID: C4; continent: Europe; country: Spain; countryCode: ES; stateProvince: Castilla-La Mancha; county: Ciudad Real; locality: La Quesera; verbatimElevation: 772.3; decimalLatitude: 39.36337; decimalLongitude: -4.41704; geodeticDatum: WGS84; **Event:** eventID: L; samplingProtocol: Pitfall
- **Type status:** Other material. **Occurrence:** individualCount: 1; sex: female; **Location:** locationID: S2; continent: Europe; country: Spain; countryCode: ES; stateProvince: Andalucía; county: Granada; locality: Camarate; verbatimElevation: 1713.96; decimalLatitude: 37.18377; decimalLongitude: -3.26282; geodeticDatum: WGS84; **Event:** eventID: B; samplingProtocol: Pitfall
- **Type status:** Other material. **Occurrence:** individualCount: 1; sex: female; **Location:** locationID: S2; continent: Europe; country: Spain; countryCode: ES; stateProvince: Andalucía; county: Granada; locality: Camarate; verbatimElevation: 1713.96; decimalLatitude: 37.18377; decimalLongitude: -3.26282; geodeticDatum: WGS84; **Event:** eventID: H; samplingProtocol: Pitfall

##### Distribution

Western Mediterranean

#### Aulonia albimana

(Walckenaer, 1805)

##### Materials

- **Type status:** Other material. **Occurrence:** individualCount: 4; sex: male; **Location:** locationID: O2; continent: Europe; country: Spain; countryCode: ES; stateProvince: Aragón; county: Huesca; locality: Rebilla; verbatimElevation: 1158.13; decimalLatitude: 42.59427; decimalLongitude: 0.1529; geodeticDatum: WGS84; **Event:** eventID: I; samplingProtocol: Pitfall

##### Distribution

Palearctic

#### Pardosa bifasciata

(C. L. Koch, 1834)

##### Materials

- **Type status:** Other material. **Occurrence:** individualCount: 6; sex: male; **Location:** locationID: O1; continent: Europe; country: Spain; countryCode: ES; stateProvince: Aragón; county: Huesca; locality: O Furno; verbatimElevation: 1396.73; decimalLatitude: 42.60677; decimalLongitude: 0.13135; geodeticDatum: WGS84; **Event:** eventID: C; samplingProtocol: Pitfall

##### Distribution

Palearctic

#### Pardosa hortensis

(Thorell, 1872)

##### Materials

- **Type status:** Other material. **Occurrence:** individualCount: 1; sex: female; **Location:** locationID: C1; continent: Europe; country: Spain; countryCode: ES; stateProvince: Castilla-La Mancha; county: Ciudad Real; locality: Valle Brezoso; verbatimElevation: 756.56; decimalLatitude: 39.35663; decimalLongitude: -4.35912; geodeticDatum: WGS84; **Event:** eventID: 2; samplingProtocol: Aerial; eventTime: Night
- **Type status:** Other material. **Occurrence:** individualCount: 5; sex: female; **Location:** locationID: C1; continent: Europe; country: Spain; countryCode: ES; stateProvince: Castilla-La Mancha; county: Ciudad Real; locality: Valle Brezoso; verbatimElevation: 756.56; decimalLatitude: 39.35663; decimalLongitude: -4.35912; geodeticDatum: WGS84; **Event:** eventID: A; samplingProtocol: Pitfall
- **Type status:** Other material. **Occurrence:** individualCount: 6; sex: female; **Location:** locationID: C1; continent: Europe; country: Spain; countryCode: ES; stateProvince: Castilla-La Mancha; county: Ciudad Real; locality: Valle Brezoso; verbatimElevation: 756.56; decimalLatitude: 39.35663; decimalLongitude: -4.35912; geodeticDatum: WGS84; **Event:** eventID: B; samplingProtocol: Pitfall
- **Type status:** Other material. **Occurrence:** individualCount: 16; sex: female; **Location:** locationID: C1; continent: Europe; country: Spain; countryCode: ES; stateProvince: Castilla-La Mancha; county: Ciudad Real; locality: Valle Brezoso; verbatimElevation: 756.56; decimalLatitude: 39.35663; decimalLongitude: -4.35912; geodeticDatum: WGS84; **Event:** eventID: C; samplingProtocol: Pitfall
- **Type status:** Other material. **Occurrence:** individualCount: 3; sex: female; **Location:** locationID: C1; continent: Europe; country: Spain; countryCode: ES; stateProvince: Castilla-La Mancha; county: Ciudad Real; locality: Valle Brezoso; verbatimElevation: 756.56; decimalLatitude: 39.35663; decimalLongitude: -4.35912; geodeticDatum: WGS84; **Event:** eventID: D; samplingProtocol: Pitfall
- **Type status:** Other material. **Occurrence:** individualCount: 3; sex: female; **Location:** locationID: C1; continent: Europe; country: Spain; countryCode: ES; stateProvince: Castilla-La Mancha; county: Ciudad Real; locality: Valle Brezoso; verbatimElevation: 756.56; decimalLatitude: 39.35663; decimalLongitude: -4.35912; geodeticDatum: WGS84; **Event:** eventID: E; samplingProtocol: Pitfall
- **Type status:** Other material. **Occurrence:** individualCount: 1; sex: female; **Location:** locationID: C1; continent: Europe; country: Spain; countryCode: ES; stateProvince: Castilla-La Mancha; county: Ciudad Real; locality: Valle Brezoso; verbatimElevation: 756.56; decimalLatitude: 39.35663; decimalLongitude: -4.35912; geodeticDatum: WGS84; **Event:** eventID: F; samplingProtocol: Pitfall
- **Type status:** Other material. **Occurrence:** individualCount: 4; sex: female; **Location:** locationID: C1; continent: Europe; country: Spain; countryCode: ES; stateProvince: Castilla-La Mancha; county: Ciudad Real; locality: Valle Brezoso; verbatimElevation: 756.56; decimalLatitude: 39.35663; decimalLongitude: -4.35912; geodeticDatum: WGS84; **Event:** eventID: G; samplingProtocol: Pitfall
- **Type status:** Other material. **Occurrence:** individualCount: 4; sex: female; **Location:** locationID: C1; continent: Europe; country: Spain; countryCode: ES; stateProvince: Castilla-La Mancha; county: Ciudad Real; locality: Valle Brezoso; verbatimElevation: 756.56; decimalLatitude: 39.35663; decimalLongitude: -4.35912; geodeticDatum: WGS84; **Event:** eventID: H; samplingProtocol: Pitfall
- **Type status:** Other material. **Occurrence:** individualCount: 3; sex: female; **Location:** locationID: C1; continent: Europe; country: Spain; countryCode: ES; stateProvince: Castilla-La Mancha; county: Ciudad Real; locality: Valle Brezoso; verbatimElevation: 756.56; decimalLatitude: 39.35663; decimalLongitude: -4.35912; geodeticDatum: WGS84; **Event:** eventID: I; samplingProtocol: Pitfall
- **Type status:** Other material. **Occurrence:** individualCount: 2; sex: female; **Location:** locationID: C1; continent: Europe; country: Spain; countryCode: ES; stateProvince: Castilla-La Mancha; county: Ciudad Real; locality: Valle Brezoso; verbatimElevation: 756.56; decimalLatitude: 39.35663; decimalLongitude: -4.35912; geodeticDatum: WGS84; **Event:** eventID: J; samplingProtocol: Pitfall
- **Type status:** Other material. **Occurrence:** individualCount: 14; sex: female; **Location:** locationID: C1; continent: Europe; country: Spain; countryCode: ES; stateProvince: Castilla-La Mancha; county: Ciudad Real; locality: Valle Brezoso; verbatimElevation: 756.56; decimalLatitude: 39.35663; decimalLongitude: -4.35912; geodeticDatum: WGS84; **Event:** eventID: K; samplingProtocol: Pitfall
- **Type status:** Other material. **Occurrence:** individualCount: 6; sex: female; **Location:** locationID: C1; continent: Europe; country: Spain; countryCode: ES; stateProvince: Castilla-La Mancha; county: Ciudad Real; locality: Valle Brezoso; verbatimElevation: 756.56; decimalLatitude: 39.35663; decimalLongitude: -4.35912; geodeticDatum: WGS84; **Event:** eventID: L; samplingProtocol: Pitfall
- **Type status:** Other material. **Occurrence:** individualCount: 4; sex: female; **Location:** locationID: C2; continent: Europe; country: Spain; countryCode: ES; stateProvince: Castilla-La Mancha; county: Ciudad Real; locality: Valle Brezoso; verbatimElevation: 739.31; decimalLatitude: 39.35159; decimalLongitude: -4.3589; geodeticDatum: WGS84; **Event:** eventID: A; samplingProtocol: Pitfall
- **Type status:** Other material. **Occurrence:** individualCount: 1; sex: female; **Location:** locationID: C2; continent: Europe; country: Spain; countryCode: ES; stateProvince: Castilla-La Mancha; county: Ciudad Real; locality: Valle Brezoso; verbatimElevation: 739.31; decimalLatitude: 39.35159; decimalLongitude: -4.3589; geodeticDatum: WGS84; **Event:** eventID: C; samplingProtocol: Pitfall
- **Type status:** Other material. **Occurrence:** individualCount: 1; sex: female; **Location:** locationID: C2; continent: Europe; country: Spain; countryCode: ES; stateProvince: Castilla-La Mancha; county: Ciudad Real; locality: Valle Brezoso; verbatimElevation: 739.31; decimalLatitude: 39.35159; decimalLongitude: -4.3589; geodeticDatum: WGS84; **Event:** eventID: D; samplingProtocol: Pitfall
- **Type status:** Other material. **Occurrence:** individualCount: 3; sex: female; **Location:** locationID: C2; continent: Europe; country: Spain; countryCode: ES; stateProvince: Castilla-La Mancha; county: Ciudad Real; locality: Valle Brezoso; verbatimElevation: 739.31; decimalLatitude: 39.35159; decimalLongitude: -4.3589; geodeticDatum: WGS84; **Event:** eventID: E; samplingProtocol: Pitfall
- **Type status:** Other material. **Occurrence:** individualCount: 1; sex: female; **Location:** locationID: C2; continent: Europe; country: Spain; countryCode: ES; stateProvince: Castilla-La Mancha; county: Ciudad Real; locality: Valle Brezoso; verbatimElevation: 739.31; decimalLatitude: 39.35159; decimalLongitude: -4.3589; geodeticDatum: WGS84; **Event:** eventID: F; samplingProtocol: Pitfall
- **Type status:** Other material. **Occurrence:** individualCount: 5; sex: female; **Location:** locationID: C2; continent: Europe; country: Spain; countryCode: ES; stateProvince: Castilla-La Mancha; county: Ciudad Real; locality: Valle Brezoso; verbatimElevation: 739.31; decimalLatitude: 39.35159; decimalLongitude: -4.3589; geodeticDatum: WGS84; **Event:** eventID: G; samplingProtocol: Pitfall
- **Type status:** Other material. **Occurrence:** individualCount: 13; sex: female; **Location:** locationID: C2; continent: Europe; country: Spain; countryCode: ES; stateProvince: Castilla-La Mancha; county: Ciudad Real; locality: Valle Brezoso; verbatimElevation: 739.31; decimalLatitude: 39.35159; decimalLongitude: -4.3589; geodeticDatum: WGS84; **Event:** eventID: H; samplingProtocol: Pitfall
- **Type status:** Other material. **Occurrence:** individualCount: 2; sex: female; **Location:** locationID: C2; continent: Europe; country: Spain; countryCode: ES; stateProvince: Castilla-La Mancha; county: Ciudad Real; locality: Valle Brezoso; verbatimElevation: 739.31; decimalLatitude: 39.35159; decimalLongitude: -4.3589; geodeticDatum: WGS84; **Event:** eventID: J; samplingProtocol: Pitfall
- **Type status:** Other material. **Occurrence:** individualCount: 2; sex: female; **Location:** locationID: C2; continent: Europe; country: Spain; countryCode: ES; stateProvince: Castilla-La Mancha; county: Ciudad Real; locality: Valle Brezoso; verbatimElevation: 739.31; decimalLatitude: 39.35159; decimalLongitude: -4.3589; geodeticDatum: WGS84; **Event:** eventID: K; samplingProtocol: Pitfall
- **Type status:** Other material. **Occurrence:** individualCount: 10; sex: female; **Location:** locationID: C2; continent: Europe; country: Spain; countryCode: ES; stateProvince: Castilla-La Mancha; county: Ciudad Real; locality: Valle Brezoso; verbatimElevation: 739.31; decimalLatitude: 39.35159; decimalLongitude: -4.3589; geodeticDatum: WGS84; **Event:** eventID: L; samplingProtocol: Pitfall
- **Type status:** Other material. **Occurrence:** individualCount: 4; sex: female; **Location:** locationID: C3; continent: Europe; country: Spain; countryCode: ES; stateProvince: Castilla-La Mancha; county: Ciudad Real; locality: La Quesera; verbatimElevation: 767.55; decimalLatitude: 39.36177; decimalLongitude: -4.41733; geodeticDatum: WGS84; **Event:** eventID: A; samplingProtocol: Pitfall
- **Type status:** Other material. **Occurrence:** individualCount: 1; sex: female; **Location:** locationID: C3; continent: Europe; country: Spain; countryCode: ES; stateProvince: Castilla-La Mancha; county: Ciudad Real; locality: La Quesera; verbatimElevation: 767.55; decimalLatitude: 39.36177; decimalLongitude: -4.41733; geodeticDatum: WGS84; **Event:** eventID: F; samplingProtocol: Pitfall
- **Type status:** Other material. **Occurrence:** individualCount: 1; sex: female; **Location:** locationID: C3; continent: Europe; country: Spain; countryCode: ES; stateProvince: Castilla-La Mancha; county: Ciudad Real; locality: La Quesera; verbatimElevation: 767.55; decimalLatitude: 39.36177; decimalLongitude: -4.41733; geodeticDatum: WGS84; **Event:** eventID: I; samplingProtocol: Pitfall
- **Type status:** Other material. **Occurrence:** individualCount: 1; sex: female; **Location:** locationID: C3; continent: Europe; country: Spain; countryCode: ES; stateProvince: Castilla-La Mancha; county: Ciudad Real; locality: La Quesera; verbatimElevation: 767.55; decimalLatitude: 39.36177; decimalLongitude: -4.41733; geodeticDatum: WGS84; **Event:** eventID: J; samplingProtocol: Pitfall
- **Type status:** Other material. **Occurrence:** individualCount: 4; sex: female; **Location:** locationID: C3; continent: Europe; country: Spain; countryCode: ES; stateProvince: Castilla-La Mancha; county: Ciudad Real; locality: La Quesera; verbatimElevation: 767.55; decimalLatitude: 39.36177; decimalLongitude: -4.41733; geodeticDatum: WGS84; **Event:** eventID: L; samplingProtocol: Pitfall
- **Type status:** Other material. **Occurrence:** individualCount: 1; sex: female; **Location:** locationID: C4; continent: Europe; country: Spain; countryCode: ES; stateProvince: Castilla-La Mancha; county: Ciudad Real; locality: La Quesera; verbatimElevation: 772.3; decimalLatitude: 39.36337; decimalLongitude: -4.41704; geodeticDatum: WGS84; **Event:** eventID: A; samplingProtocol: Pitfall
- **Type status:** Other material. **Occurrence:** individualCount: 2; sex: female; **Location:** locationID: C4; continent: Europe; country: Spain; countryCode: ES; stateProvince: Castilla-La Mancha; county: Ciudad Real; locality: La Quesera; verbatimElevation: 772.3; decimalLatitude: 39.36337; decimalLongitude: -4.41704; geodeticDatum: WGS84; **Event:** eventID: D; samplingProtocol: Pitfall
- **Type status:** Other material. **Occurrence:** individualCount: 1; sex: female; **Location:** locationID: C4; continent: Europe; country: Spain; countryCode: ES; stateProvince: Castilla-La Mancha; county: Ciudad Real; locality: La Quesera; verbatimElevation: 772.3; decimalLatitude: 39.36337; decimalLongitude: -4.41704; geodeticDatum: WGS84; **Event:** eventID: F; samplingProtocol: Pitfall
- **Type status:** Other material. **Occurrence:** individualCount: 2; sex: female; **Location:** locationID: C4; continent: Europe; country: Spain; countryCode: ES; stateProvince: Castilla-La Mancha; county: Ciudad Real; locality: La Quesera; verbatimElevation: 772.3; decimalLatitude: 39.36337; decimalLongitude: -4.41704; geodeticDatum: WGS84; **Event:** eventID: G; samplingProtocol: Pitfall
- **Type status:** Other material. **Occurrence:** individualCount: 2; sex: female; **Location:** locationID: C4; continent: Europe; country: Spain; countryCode: ES; stateProvince: Castilla-La Mancha; county: Ciudad Real; locality: La Quesera; verbatimElevation: 772.3; decimalLatitude: 39.36337; decimalLongitude: -4.41704; geodeticDatum: WGS84; **Event:** eventID: K; samplingProtocol: Pitfall
- **Type status:** Other material. **Occurrence:** individualCount: 1; sex: female; **Location:** locationID: C4; continent: Europe; country: Spain; countryCode: ES; stateProvince: Castilla-La Mancha; county: Ciudad Real; locality: La Quesera; verbatimElevation: 772.3; decimalLatitude: 39.36337; decimalLongitude: -4.41704; geodeticDatum: WGS84; **Event:** eventID: L; samplingProtocol: Pitfall
- **Type status:** Other material. **Occurrence:** individualCount: 3; sex: female; **Location:** locationID: S1; continent: Europe; country: Spain; countryCode: ES; stateProvince: Andalucía; county: Granada; locality: Soportujar; verbatimElevation: 1786.57; decimalLatitude: 36.96151; decimalLongitude: -3.41881; geodeticDatum: WGS84; **Event:** eventID: D; samplingProtocol: Pitfall
- **Type status:** Other material. **Occurrence:** individualCount: 1; sex: female; **Location:** locationID: S1; continent: Europe; country: Spain; countryCode: ES; stateProvince: Andalucía; county: Granada; locality: Soportujar; verbatimElevation: 1786.57; decimalLatitude: 36.96151; decimalLongitude: -3.41881; geodeticDatum: WGS84; **Event:** eventID: E; samplingProtocol: Pitfall
- **Type status:** Other material. **Occurrence:** individualCount: 2; sex: female; **Location:** locationID: S1; continent: Europe; country: Spain; countryCode: ES; stateProvince: Andalucía; county: Granada; locality: Soportujar; verbatimElevation: 1786.57; decimalLatitude: 36.96151; decimalLongitude: -3.41881; geodeticDatum: WGS84; **Event:** eventID: F; samplingProtocol: Pitfall
- **Type status:** Other material. **Occurrence:** individualCount: 1; sex: female; **Location:** locationID: S1; continent: Europe; country: Spain; countryCode: ES; stateProvince: Andalucía; county: Granada; locality: Soportujar; verbatimElevation: 1786.57; decimalLatitude: 36.96151; decimalLongitude: -3.41881; geodeticDatum: WGS84; **Event:** eventID: G; samplingProtocol: Pitfall
- **Type status:** Other material. **Occurrence:** individualCount: 2; sex: female; **Location:** locationID: S1; continent: Europe; country: Spain; countryCode: ES; stateProvince: Andalucía; county: Granada; locality: Soportujar; verbatimElevation: 1786.57; decimalLatitude: 36.96151; decimalLongitude: -3.41881; geodeticDatum: WGS84; **Event:** eventID: H; samplingProtocol: Pitfall
- **Type status:** Other material. **Occurrence:** individualCount: 1; sex: female; **Location:** locationID: S1; continent: Europe; country: Spain; countryCode: ES; stateProvince: Andalucía; county: Granada; locality: Soportujar; verbatimElevation: 1786.57; decimalLatitude: 36.96151; decimalLongitude: -3.41881; geodeticDatum: WGS84; **Event:** eventID: I; samplingProtocol: Pitfall
- **Type status:** Other material. **Occurrence:** individualCount: 1; sex: female; **Location:** locationID: S1; continent: Europe; country: Spain; countryCode: ES; stateProvince: Andalucía; county: Granada; locality: Soportujar; verbatimElevation: 1786.57; decimalLatitude: 36.96151; decimalLongitude: -3.41881; geodeticDatum: WGS84; **Event:** eventID: J; samplingProtocol: Pitfall
- **Type status:** Other material. **Occurrence:** individualCount: 2; sex: female; **Location:** locationID: S1; continent: Europe; country: Spain; countryCode: ES; stateProvince: Andalucía; county: Granada; locality: Soportujar; verbatimElevation: 1786.57; decimalLatitude: 36.96151; decimalLongitude: -3.41881; geodeticDatum: WGS84; **Event:** eventID: K; samplingProtocol: Pitfall
- **Type status:** Other material. **Occurrence:** individualCount: 1; sex: female; **Location:** locationID: S1; continent: Europe; country: Spain; countryCode: ES; stateProvince: Andalucía; county: Granada; locality: Soportujar; verbatimElevation: 1786.57; decimalLatitude: 36.96151; decimalLongitude: -3.41881; geodeticDatum: WGS84; **Event:** eventID: L; samplingProtocol: Pitfall

##### Distribution

Palearctic

#### Pardosa lugubris

(Walckenaer, 1802)

##### Materials

- **Type status:** Other material. **Occurrence:** individualCount: 1; sex: female; **Location:** locationID: A2; continent: Europe; country: Spain; countryCode: ES; stateProvince: Catalonia; county: Lleida; locality: Sola de Boi; verbatimElevation: 1738.7; decimalLatitude: 42.54913; decimalLongitude: 0.87137; geodeticDatum: WGS84; **Event:** eventID: G; samplingProtocol: Pitfall
- **Type status:** Other material. **Occurrence:** individualCount: 2; sex: male; **Location:** locationID: A2; continent: Europe; country: Spain; countryCode: ES; stateProvince: Catalonia; county: Lleida; locality: Sola de Boi; verbatimElevation: 1738.7; decimalLatitude: 42.54913; decimalLongitude: 0.87137; geodeticDatum: WGS84; **Event:** eventID: K; samplingProtocol: Pitfall
- **Type status:** Other material. **Occurrence:** individualCount: 3; sex: male; **Location:** locationID: A2; continent: Europe; country: Spain; countryCode: ES; stateProvince: Catalonia; county: Lleida; locality: Sola de Boi; verbatimElevation: 1738.7; decimalLatitude: 42.54913; decimalLongitude: 0.87137; geodeticDatum: WGS84; **Event:** eventID: L; samplingProtocol: Pitfall
- **Type status:** Other material. **Occurrence:** individualCount: 1; sex: male; **Location:** locationID: O1; continent: Europe; country: Spain; countryCode: ES; stateProvince: Aragón; county: Huesca; locality: O Furno; verbatimElevation: 1396.73; decimalLatitude: 42.60677; decimalLongitude: 0.13135; geodeticDatum: WGS84; **Event:** eventID: A; samplingProtocol: Pitfall
- **Type status:** Other material. **Occurrence:** individualCount: 1; sex: female; **Location:** locationID: O1; continent: Europe; country: Spain; countryCode: ES; stateProvince: Aragón; county: Huesca; locality: O Furno; verbatimElevation: 1396.73; decimalLatitude: 42.60677; decimalLongitude: 0.13135; geodeticDatum: WGS84; **Event:** eventID: A; samplingProtocol: Pitfall
- **Type status:** Other material. **Occurrence:** individualCount: 1; sex: male; **Location:** locationID: O1; continent: Europe; country: Spain; countryCode: ES; stateProvince: Aragón; county: Huesca; locality: O Furno; verbatimElevation: 1396.73; decimalLatitude: 42.60677; decimalLongitude: 0.13135; geodeticDatum: WGS84; **Event:** eventID: B; samplingProtocol: Pitfall
- **Type status:** Other material. **Occurrence:** individualCount: 1; sex: female; **Location:** locationID: O1; continent: Europe; country: Spain; countryCode: ES; stateProvince: Aragón; county: Huesca; locality: O Furno; verbatimElevation: 1396.73; decimalLatitude: 42.60677; decimalLongitude: 0.13135; geodeticDatum: WGS84; **Event:** eventID: B; samplingProtocol: Pitfall
- **Type status:** Other material. **Occurrence:** individualCount: 3; sex: male; **Location:** locationID: O1; continent: Europe; country: Spain; countryCode: ES; stateProvince: Aragón; county: Huesca; locality: O Furno; verbatimElevation: 1396.73; decimalLatitude: 42.60677; decimalLongitude: 0.13135; geodeticDatum: WGS84; **Event:** eventID: C; samplingProtocol: Pitfall
- **Type status:** Other material. **Occurrence:** individualCount: 12; sex: male; **Location:** locationID: O1; continent: Europe; country: Spain; countryCode: ES; stateProvince: Aragón; county: Huesca; locality: O Furno; verbatimElevation: 1396.73; decimalLatitude: 42.60677; decimalLongitude: 0.13135; geodeticDatum: WGS84; **Event:** eventID: D; samplingProtocol: Pitfall
- **Type status:** Other material. **Occurrence:** individualCount: 6; sex: female; **Location:** locationID: O1; continent: Europe; country: Spain; countryCode: ES; stateProvince: Aragón; county: Huesca; locality: O Furno; verbatimElevation: 1396.73; decimalLatitude: 42.60677; decimalLongitude: 0.13135; geodeticDatum: WGS84; **Event:** eventID: D; samplingProtocol: Pitfall
- **Type status:** Other material. **Occurrence:** individualCount: 5; sex: male; **Location:** locationID: O1; continent: Europe; country: Spain; countryCode: ES; stateProvince: Aragón; county: Huesca; locality: O Furno; verbatimElevation: 1396.73; decimalLatitude: 42.60677; decimalLongitude: 0.13135; geodeticDatum: WGS84; **Event:** eventID: E; samplingProtocol: Pitfall
- **Type status:** Other material. **Occurrence:** individualCount: 1; sex: male; **Location:** locationID: O1; continent: Europe; country: Spain; countryCode: ES; stateProvince: Aragón; county: Huesca; locality: O Furno; verbatimElevation: 1396.73; decimalLatitude: 42.60677; decimalLongitude: 0.13135; geodeticDatum: WGS84; **Event:** eventID: G; samplingProtocol: Pitfall
- **Type status:** Other material. **Occurrence:** individualCount: 1; sex: male; **Location:** locationID: O1; continent: Europe; country: Spain; countryCode: ES; stateProvince: Aragón; county: Huesca; locality: O Furno; verbatimElevation: 1396.73; decimalLatitude: 42.60677; decimalLongitude: 0.13135; geodeticDatum: WGS84; **Event:** eventID: H; samplingProtocol: Pitfall
- **Type status:** Other material. **Occurrence:** individualCount: 3; sex: male; **Location:** locationID: O1; continent: Europe; country: Spain; countryCode: ES; stateProvince: Aragón; county: Huesca; locality: O Furno; verbatimElevation: 1396.73; decimalLatitude: 42.60677; decimalLongitude: 0.13135; geodeticDatum: WGS84; **Event:** eventID: K; samplingProtocol: Pitfall
- **Type status:** Other material. **Occurrence:** individualCount: 1; sex: female; **Location:** locationID: O1; continent: Europe; country: Spain; countryCode: ES; stateProvince: Aragón; county: Huesca; locality: O Furno; verbatimElevation: 1396.73; decimalLatitude: 42.60677; decimalLongitude: 0.13135; geodeticDatum: WGS84; **Event:** eventID: 1; samplingProtocol: Sweeping; eventTime: Day
- **Type status:** Other material. **Occurrence:** individualCount: 1; sex: female; **Location:** locationID: O2; continent: Europe; country: Spain; countryCode: ES; stateProvince: Aragón; county: Huesca; locality: Rebilla; verbatimElevation: 1158.13; decimalLatitude: 42.59427; decimalLongitude: 0.1529; geodeticDatum: WGS84; **Event:** eventID: 2; samplingProtocol: Aerial; eventTime: Night
- **Type status:** Other material. **Occurrence:** individualCount: 10; sex: male; **Location:** locationID: O2; continent: Europe; country: Spain; countryCode: ES; stateProvince: Aragón; county: Huesca; locality: Rebilla; verbatimElevation: 1158.13; decimalLatitude: 42.59427; decimalLongitude: 0.1529; geodeticDatum: WGS84; **Event:** eventID: D; samplingProtocol: Pitfall
- **Type status:** Other material. **Occurrence:** individualCount: 1; sex: female; **Location:** locationID: O2; continent: Europe; country: Spain; countryCode: ES; stateProvince: Aragón; county: Huesca; locality: Rebilla; verbatimElevation: 1158.13; decimalLatitude: 42.59427; decimalLongitude: 0.1529; geodeticDatum: WGS84; **Event:** eventID: D; samplingProtocol: Pitfall
- **Type status:** Other material. **Occurrence:** individualCount: 1; sex: male; **Location:** locationID: O2; continent: Europe; country: Spain; countryCode: ES; stateProvince: Aragón; county: Huesca; locality: Rebilla; verbatimElevation: 1158.13; decimalLatitude: 42.59427; decimalLongitude: 0.1529; geodeticDatum: WGS84; **Event:** eventID: E; samplingProtocol: Pitfall
- **Type status:** Other material. **Occurrence:** individualCount: 3; sex: male; **Location:** locationID: O2; continent: Europe; country: Spain; countryCode: ES; stateProvince: Aragón; county: Huesca; locality: Rebilla; verbatimElevation: 1158.13; decimalLatitude: 42.59427; decimalLongitude: 0.1529; geodeticDatum: WGS84; **Event:** eventID: F; samplingProtocol: Pitfall
- **Type status:** Other material. **Occurrence:** individualCount: 4; sex: female; **Location:** locationID: O2; continent: Europe; country: Spain; countryCode: ES; stateProvince: Aragón; county: Huesca; locality: Rebilla; verbatimElevation: 1158.13; decimalLatitude: 42.59427; decimalLongitude: 0.1529; geodeticDatum: WGS84; **Event:** eventID: F; samplingProtocol: Pitfall
- **Type status:** Other material. **Occurrence:** individualCount: 22; sex: male; **Location:** locationID: O2; continent: Europe; country: Spain; countryCode: ES; stateProvince: Aragón; county: Huesca; locality: Rebilla; verbatimElevation: 1158.13; decimalLatitude: 42.59427; decimalLongitude: 0.1529; geodeticDatum: WGS84; **Event:** eventID: G; samplingProtocol: Pitfall
- **Type status:** Other material. **Occurrence:** individualCount: 2; sex: female; **Location:** locationID: O2; continent: Europe; country: Spain; countryCode: ES; stateProvince: Aragón; county: Huesca; locality: Rebilla; verbatimElevation: 1158.13; decimalLatitude: 42.59427; decimalLongitude: 0.1529; geodeticDatum: WGS84; **Event:** eventID: G; samplingProtocol: Pitfall
- **Type status:** Other material. **Occurrence:** individualCount: 1; sex: female; **Location:** locationID: O2; continent: Europe; country: Spain; countryCode: ES; stateProvince: Aragón; county: Huesca; locality: Rebilla; verbatimElevation: 1158.13; decimalLatitude: 42.59427; decimalLongitude: 0.1529; geodeticDatum: WGS84; **Event:** eventID: H; samplingProtocol: Pitfall
- **Type status:** Other material. **Occurrence:** individualCount: 9; sex: male; **Location:** locationID: O2; continent: Europe; country: Spain; countryCode: ES; stateProvince: Aragón; county: Huesca; locality: Rebilla; verbatimElevation: 1158.13; decimalLatitude: 42.59427; decimalLongitude: 0.1529; geodeticDatum: WGS84; **Event:** eventID: I; samplingProtocol: Pitfall
- **Type status:** Other material. **Occurrence:** individualCount: 9; sex: female; **Location:** locationID: O2; continent: Europe; country: Spain; countryCode: ES; stateProvince: Aragón; county: Huesca; locality: Rebilla; verbatimElevation: 1158.13; decimalLatitude: 42.59427; decimalLongitude: 0.1529; geodeticDatum: WGS84; **Event:** eventID: I; samplingProtocol: Pitfall
- **Type status:** Other material. **Occurrence:** individualCount: 3; sex: male; **Location:** locationID: O2; continent: Europe; country: Spain; countryCode: ES; stateProvince: Aragón; county: Huesca; locality: Rebilla; verbatimElevation: 1158.13; decimalLatitude: 42.59427; decimalLongitude: 0.1529; geodeticDatum: WGS84; **Event:** eventID: J; samplingProtocol: Pitfall
- **Type status:** Other material. **Occurrence:** individualCount: 2; sex: female; **Location:** locationID: O2; continent: Europe; country: Spain; countryCode: ES; stateProvince: Aragón; county: Huesca; locality: Rebilla; verbatimElevation: 1158.13; decimalLatitude: 42.59427; decimalLongitude: 0.1529; geodeticDatum: WGS84; **Event:** eventID: J; samplingProtocol: Pitfall
- **Type status:** Other material. **Occurrence:** individualCount: 19; sex: male; **Location:** locationID: O2; continent: Europe; country: Spain; countryCode: ES; stateProvince: Aragón; county: Huesca; locality: Rebilla; verbatimElevation: 1158.13; decimalLatitude: 42.59427; decimalLongitude: 0.1529; geodeticDatum: WGS84; **Event:** eventID: K; samplingProtocol: Pitfall
- **Type status:** Other material. **Occurrence:** individualCount: 3; sex: female; **Location:** locationID: O2; continent: Europe; country: Spain; countryCode: ES; stateProvince: Aragón; county: Huesca; locality: Rebilla; verbatimElevation: 1158.13; decimalLatitude: 42.59427; decimalLongitude: 0.1529; geodeticDatum: WGS84; **Event:** eventID: K; samplingProtocol: Pitfall
- **Type status:** Other material. **Occurrence:** individualCount: 1; sex: male; **Location:** locationID: O2; continent: Europe; country: Spain; countryCode: ES; stateProvince: Aragón; county: Huesca; locality: Rebilla; verbatimElevation: 1158.13; decimalLatitude: 42.59427; decimalLongitude: 0.1529; geodeticDatum: WGS84; **Event:** eventID: L; samplingProtocol: Pitfall
- **Type status:** Other material. **Occurrence:** individualCount: 2; sex: female; **Location:** locationID: O2; continent: Europe; country: Spain; countryCode: ES; stateProvince: Aragón; county: Huesca; locality: Rebilla; verbatimElevation: 1158.13; decimalLatitude: 42.59427; decimalLongitude: 0.1529; geodeticDatum: WGS84; **Event:** eventID: L; samplingProtocol: Pitfall
- **Type status:** Other material. **Occurrence:** individualCount: 2; sex: female; **Location:** locationID: O2; continent: Europe; country: Spain; countryCode: ES; stateProvince: Aragón; county: Huesca; locality: Rebilla; verbatimElevation: 1158.13; decimalLatitude: 42.59427; decimalLongitude: 0.1529; geodeticDatum: WGS84; **Event:** eventID: 1; samplingProtocol: Sweeping; eventTime: Day
- **Type status:** Other material. **Occurrence:** individualCount: 2; sex: female; **Location:** locationID: O2; continent: Europe; country: Spain; countryCode: ES; stateProvince: Aragón; county: Huesca; locality: Rebilla; verbatimElevation: 1158.13; decimalLatitude: 42.59427; decimalLongitude: 0.1529; geodeticDatum: WGS84; **Event:** eventID: 1; samplingProtocol: Sweeping; eventTime: Night
- **Type status:** Other material. **Occurrence:** individualCount: 1; sex: female; **Location:** locationID: P1; continent: Europe; country: Spain; countryCode: ES; stateProvince: Castilla y León; county: León; locality: Monte Robledo; verbatimElevation: 1071.58; decimalLatitude: 43.1445; decimalLongitude: -4.92675; geodeticDatum: WGS84; **Event:** eventID: 1; samplingProtocol: Ground; eventTime: Day
- **Type status:** Other material. **Occurrence:** individualCount: 2; sex: female; **Location:** locationID: P1; continent: Europe; country: Spain; countryCode: ES; stateProvince: Castilla y León; county: León; locality: Monte Robledo; verbatimElevation: 1071.58; decimalLatitude: 43.1445; decimalLongitude: -4.92675; geodeticDatum: WGS84; **Event:** eventID: 2; samplingProtocol: Ground; eventTime: Day
- **Type status:** Other material. **Occurrence:** individualCount: 2; sex: male; **Location:** locationID: P1; continent: Europe; country: Spain; countryCode: ES; stateProvince: Castilla y León; county: León; locality: Monte Robledo; verbatimElevation: 1071.58; decimalLatitude: 43.1445; decimalLongitude: -4.92675; geodeticDatum: WGS84; **Event:** eventID: A; samplingProtocol: Pitfall
- **Type status:** Other material. **Occurrence:** individualCount: 2; sex: female; **Location:** locationID: P1; continent: Europe; country: Spain; countryCode: ES; stateProvince: Castilla y León; county: León; locality: Monte Robledo; verbatimElevation: 1071.58; decimalLatitude: 43.1445; decimalLongitude: -4.92675; geodeticDatum: WGS84; **Event:** eventID: A; samplingProtocol: Pitfall
- **Type status:** Other material. **Occurrence:** individualCount: 1; sex: male; **Location:** locationID: P1; continent: Europe; country: Spain; countryCode: ES; stateProvince: Castilla y León; county: León; locality: Monte Robledo; verbatimElevation: 1071.58; decimalLatitude: 43.1445; decimalLongitude: -4.92675; geodeticDatum: WGS84; **Event:** eventID: B; samplingProtocol: Pitfall
- **Type status:** Other material. **Occurrence:** individualCount: 1; sex: male; **Location:** locationID: P1; continent: Europe; country: Spain; countryCode: ES; stateProvince: Castilla y León; county: León; locality: Monte Robledo; verbatimElevation: 1071.58; decimalLatitude: 43.1445; decimalLongitude: -4.92675; geodeticDatum: WGS84; **Event:** eventID: C; samplingProtocol: Pitfall
- **Type status:** Other material. **Occurrence:** individualCount: 17; sex: male; **Location:** locationID: P1; continent: Europe; country: Spain; countryCode: ES; stateProvince: Castilla y León; county: León; locality: Monte Robledo; verbatimElevation: 1071.58; decimalLatitude: 43.1445; decimalLongitude: -4.92675; geodeticDatum: WGS84; **Event:** eventID: E; samplingProtocol: Pitfall
- **Type status:** Other material. **Occurrence:** individualCount: 3; sex: female; **Location:** locationID: P1; continent: Europe; country: Spain; countryCode: ES; stateProvince: Castilla y León; county: León; locality: Monte Robledo; verbatimElevation: 1071.58; decimalLatitude: 43.1445; decimalLongitude: -4.92675; geodeticDatum: WGS84; **Event:** eventID: E; samplingProtocol: Pitfall
- **Type status:** Other material. **Occurrence:** individualCount: 3; sex: male; **Location:** locationID: P1; continent: Europe; country: Spain; countryCode: ES; stateProvince: Castilla y León; county: León; locality: Monte Robledo; verbatimElevation: 1071.58; decimalLatitude: 43.1445; decimalLongitude: -4.92675; geodeticDatum: WGS84; **Event:** eventID: F; samplingProtocol: Pitfall
- **Type status:** Other material. **Occurrence:** individualCount: 5; sex: male; **Location:** locationID: P1; continent: Europe; country: Spain; countryCode: ES; stateProvince: Castilla y León; county: León; locality: Monte Robledo; verbatimElevation: 1071.58; decimalLatitude: 43.1445; decimalLongitude: -4.92675; geodeticDatum: WGS84; **Event:** eventID: G; samplingProtocol: Pitfall
- **Type status:** Other material. **Occurrence:** individualCount: 2; sex: female; **Location:** locationID: P1; continent: Europe; country: Spain; countryCode: ES; stateProvince: Castilla y León; county: León; locality: Monte Robledo; verbatimElevation: 1071.58; decimalLatitude: 43.1445; decimalLongitude: -4.92675; geodeticDatum: WGS84; **Event:** eventID: G; samplingProtocol: Pitfall
- **Type status:** Other material. **Occurrence:** individualCount: 2; sex: male; **Location:** locationID: P1; continent: Europe; country: Spain; countryCode: ES; stateProvince: Castilla y León; county: León; locality: Monte Robledo; verbatimElevation: 1071.58; decimalLatitude: 43.1445; decimalLongitude: -4.92675; geodeticDatum: WGS84; **Event:** eventID: H; samplingProtocol: Pitfall
- **Type status:** Other material. **Occurrence:** individualCount: 18; sex: male; **Location:** locationID: P1; continent: Europe; country: Spain; countryCode: ES; stateProvince: Castilla y León; county: León; locality: Monte Robledo; verbatimElevation: 1071.58; decimalLatitude: 43.1445; decimalLongitude: -4.92675; geodeticDatum: WGS84; **Event:** eventID: J; samplingProtocol: Pitfall
- **Type status:** Other material. **Occurrence:** individualCount: 2; sex: female; **Location:** locationID: P1; continent: Europe; country: Spain; countryCode: ES; stateProvince: Castilla y León; county: León; locality: Monte Robledo; verbatimElevation: 1071.58; decimalLatitude: 43.1445; decimalLongitude: -4.92675; geodeticDatum: WGS84; **Event:** eventID: J; samplingProtocol: Pitfall
- **Type status:** Other material. **Occurrence:** individualCount: 2; sex: male; **Location:** locationID: P1; continent: Europe; country: Spain; countryCode: ES; stateProvince: Castilla y León; county: León; locality: Monte Robledo; verbatimElevation: 1071.58; decimalLatitude: 43.1445; decimalLongitude: -4.92675; geodeticDatum: WGS84; **Event:** eventID: K; samplingProtocol: Pitfall
- **Type status:** Other material. **Occurrence:** individualCount: 2; sex: male; **Location:** locationID: P1; continent: Europe; country: Spain; countryCode: ES; stateProvince: Castilla y León; county: León; locality: Monte Robledo; verbatimElevation: 1071.58; decimalLatitude: 43.1445; decimalLongitude: -4.92675; geodeticDatum: WGS84; **Event:** eventID: L; samplingProtocol: Pitfall
- **Type status:** Other material. **Occurrence:** individualCount: 1; sex: female; **Location:** locationID: P1; continent: Europe; country: Spain; countryCode: ES; stateProvince: Castilla y León; county: León; locality: Monte Robledo; verbatimElevation: 1071.58; decimalLatitude: 43.1445; decimalLongitude: -4.92675; geodeticDatum: WGS84; **Event:** eventID: L; samplingProtocol: Pitfall
- **Type status:** Other material. **Occurrence:** individualCount: 2; sex: female; **Location:** locationID: P1; continent: Europe; country: Spain; countryCode: ES; stateProvince: Castilla y León; county: León; locality: Monte Robledo; verbatimElevation: 1071.58; decimalLatitude: 43.1445; decimalLongitude: -4.92675; geodeticDatum: WGS84; **Event:** eventID: 2; samplingProtocol: Sweeping; eventTime: Day
- **Type status:** Other material. **Occurrence:** individualCount: 19; sex: male; **Location:** locationID: P2; continent: Europe; country: Spain; countryCode: ES; stateProvince: Castilla y León; county: León; locality: Joyoguelas; verbatimElevation: 763.98; decimalLatitude: 43.17771; decimalLongitude: -4.90579; geodeticDatum: WGS84; **Event:** eventID: A; samplingProtocol: Pitfall
- **Type status:** Other material. **Occurrence:** individualCount: 6; sex: male; **Location:** locationID: P2; continent: Europe; country: Spain; countryCode: ES; stateProvince: Castilla y León; county: León; locality: Joyoguelas; verbatimElevation: 763.98; decimalLatitude: 43.17771; decimalLongitude: -4.90579; geodeticDatum: WGS84; **Event:** eventID: B; samplingProtocol: Pitfall
- **Type status:** Other material. **Occurrence:** individualCount: 6; sex: female; **Location:** locationID: P2; continent: Europe; country: Spain; countryCode: ES; stateProvince: Castilla y León; county: León; locality: Joyoguelas; verbatimElevation: 763.98; decimalLatitude: 43.17771; decimalLongitude: -4.90579; geodeticDatum: WGS84; **Event:** eventID: B; samplingProtocol: Pitfall
- **Type status:** Other material. **Occurrence:** individualCount: 2; sex: male; **Location:** locationID: P2; continent: Europe; country: Spain; countryCode: ES; stateProvince: Castilla y León; county: León; locality: Joyoguelas; verbatimElevation: 763.98; decimalLatitude: 43.17771; decimalLongitude: -4.90579; geodeticDatum: WGS84; **Event:** eventID: C; samplingProtocol: Pitfall
- **Type status:** Other material. **Occurrence:** individualCount: 10; sex: female; **Location:** locationID: P2; continent: Europe; country: Spain; countryCode: ES; stateProvince: Castilla y León; county: León; locality: Joyoguelas; verbatimElevation: 763.98; decimalLatitude: 43.17771; decimalLongitude: -4.90579; geodeticDatum: WGS84; **Event:** eventID: C; samplingProtocol: Pitfall
- **Type status:** Other material. **Occurrence:** individualCount: 25; sex: male; **Location:** locationID: P2; continent: Europe; country: Spain; countryCode: ES; stateProvince: Castilla y León; county: León; locality: Joyoguelas; verbatimElevation: 763.98; decimalLatitude: 43.17771; decimalLongitude: -4.90579; geodeticDatum: WGS84; **Event:** eventID: D; samplingProtocol: Pitfall
- **Type status:** Other material. **Occurrence:** individualCount: 3; sex: female; **Location:** locationID: P2; continent: Europe; country: Spain; countryCode: ES; stateProvince: Castilla y León; county: León; locality: Joyoguelas; verbatimElevation: 763.98; decimalLatitude: 43.17771; decimalLongitude: -4.90579; geodeticDatum: WGS84; **Event:** eventID: D; samplingProtocol: Pitfall
- **Type status:** Other material. **Occurrence:** individualCount: 12; sex: male; **Location:** locationID: P2; continent: Europe; country: Spain; countryCode: ES; stateProvince: Castilla y León; county: León; locality: Joyoguelas; verbatimElevation: 763.98; decimalLatitude: 43.17771; decimalLongitude: -4.90579; geodeticDatum: WGS84; **Event:** eventID: E; samplingProtocol: Pitfall
- **Type status:** Other material. **Occurrence:** individualCount: 3; sex: female; **Location:** locationID: P2; continent: Europe; country: Spain; countryCode: ES; stateProvince: Castilla y León; county: León; locality: Joyoguelas; verbatimElevation: 763.98; decimalLatitude: 43.17771; decimalLongitude: -4.90579; geodeticDatum: WGS84; **Event:** eventID: E; samplingProtocol: Pitfall
- **Type status:** Other material. **Occurrence:** individualCount: 6; sex: female; **Location:** locationID: P2; continent: Europe; country: Spain; countryCode: ES; stateProvince: Castilla y León; county: León; locality: Joyoguelas; verbatimElevation: 763.98; decimalLatitude: 43.17771; decimalLongitude: -4.90579; geodeticDatum: WGS84; **Event:** eventID: F; samplingProtocol: Pitfall
- **Type status:** Other material. **Occurrence:** individualCount: 54; sex: male; **Location:** locationID: P2; continent: Europe; country: Spain; countryCode: ES; stateProvince: Castilla y León; county: León; locality: Joyoguelas; verbatimElevation: 763.98; decimalLatitude: 43.17771; decimalLongitude: -4.90579; geodeticDatum: WGS84; **Event:** eventID: G; samplingProtocol: Pitfall
- **Type status:** Other material. **Occurrence:** individualCount: 7; sex: female; **Location:** locationID: P2; continent: Europe; country: Spain; countryCode: ES; stateProvince: Castilla y León; county: León; locality: Joyoguelas; verbatimElevation: 763.98; decimalLatitude: 43.17771; decimalLongitude: -4.90579; geodeticDatum: WGS84; **Event:** eventID: G; samplingProtocol: Pitfall
- **Type status:** Other material. **Occurrence:** individualCount: 21; sex: male; **Location:** locationID: P2; continent: Europe; country: Spain; countryCode: ES; stateProvince: Castilla y León; county: León; locality: Joyoguelas; verbatimElevation: 763.98; decimalLatitude: 43.17771; decimalLongitude: -4.90579; geodeticDatum: WGS84; **Event:** eventID: H; samplingProtocol: Pitfall
- **Type status:** Other material. **Occurrence:** individualCount: 3; sex: female; **Location:** locationID: P2; continent: Europe; country: Spain; countryCode: ES; stateProvince: Castilla y León; county: León; locality: Joyoguelas; verbatimElevation: 763.98; decimalLatitude: 43.17771; decimalLongitude: -4.90579; geodeticDatum: WGS84; **Event:** eventID: H; samplingProtocol: Pitfall
- **Type status:** Other material. **Occurrence:** individualCount: 1; sex: male; **Location:** locationID: P2; continent: Europe; country: Spain; countryCode: ES; stateProvince: Castilla y León; county: León; locality: Joyoguelas; verbatimElevation: 763.98; decimalLatitude: 43.17771; decimalLongitude: -4.90579; geodeticDatum: WGS84; **Event:** eventID: I; samplingProtocol: Pitfall
- **Type status:** Other material. **Occurrence:** individualCount: 4; sex: female; **Location:** locationID: P2; continent: Europe; country: Spain; countryCode: ES; stateProvince: Castilla y León; county: León; locality: Joyoguelas; verbatimElevation: 763.98; decimalLatitude: 43.17771; decimalLongitude: -4.90579; geodeticDatum: WGS84; **Event:** eventID: I; samplingProtocol: Pitfall
- **Type status:** Other material. **Occurrence:** individualCount: 7; sex: male; **Location:** locationID: P2; continent: Europe; country: Spain; countryCode: ES; stateProvince: Castilla y León; county: León; locality: Joyoguelas; verbatimElevation: 763.98; decimalLatitude: 43.17771; decimalLongitude: -4.90579; geodeticDatum: WGS84; **Event:** eventID: J; samplingProtocol: Pitfall
- **Type status:** Other material. **Occurrence:** individualCount: 5; sex: female; **Location:** locationID: P2; continent: Europe; country: Spain; countryCode: ES; stateProvince: Castilla y León; county: León; locality: Joyoguelas; verbatimElevation: 763.98; decimalLatitude: 43.17771; decimalLongitude: -4.90579; geodeticDatum: WGS84; **Event:** eventID: J; samplingProtocol: Pitfall
- **Type status:** Other material. **Occurrence:** individualCount: 27; sex: male; **Location:** locationID: P2; continent: Europe; country: Spain; countryCode: ES; stateProvince: Castilla y León; county: León; locality: Joyoguelas; verbatimElevation: 763.98; decimalLatitude: 43.17771; decimalLongitude: -4.90579; geodeticDatum: WGS84; **Event:** eventID: K; samplingProtocol: Pitfall
- **Type status:** Other material. **Occurrence:** individualCount: 6; sex: female; **Location:** locationID: P2; continent: Europe; country: Spain; countryCode: ES; stateProvince: Castilla y León; county: León; locality: Joyoguelas; verbatimElevation: 763.98; decimalLatitude: 43.17771; decimalLongitude: -4.90579; geodeticDatum: WGS84; **Event:** eventID: K; samplingProtocol: Pitfall
- **Type status:** Other material. **Occurrence:** individualCount: 21; sex: male; **Location:** locationID: P2; continent: Europe; country: Spain; countryCode: ES; stateProvince: Castilla y León; county: León; locality: Joyoguelas; verbatimElevation: 763.98; decimalLatitude: 43.17771; decimalLongitude: -4.90579; geodeticDatum: WGS84; **Event:** eventID: L; samplingProtocol: Pitfall
- **Type status:** Other material. **Occurrence:** individualCount: 2; sex: female; **Location:** locationID: P2; continent: Europe; country: Spain; countryCode: ES; stateProvince: Castilla y León; county: León; locality: Joyoguelas; verbatimElevation: 763.98; decimalLatitude: 43.17771; decimalLongitude: -4.90579; geodeticDatum: WGS84; **Event:** eventID: L; samplingProtocol: Pitfall
- **Type status:** Other material. **Occurrence:** individualCount: 6; sex: male; **Location:** locationID: P3; continent: Europe; country: Spain; countryCode: ES; stateProvince: Castilla y León; county: León; locality: Las Arroyas; verbatimElevation: 1097.1; decimalLatitude: 43.14351; decimalLongitude: -4.94878; geodeticDatum: WGS84; **Event:** eventID: A; samplingProtocol: Pitfall
- **Type status:** Other material. **Occurrence:** individualCount: 2; sex: female; **Location:** locationID: P3; continent: Europe; country: Spain; countryCode: ES; stateProvince: Castilla y León; county: León; locality: Las Arroyas; verbatimElevation: 1097.1; decimalLatitude: 43.14351; decimalLongitude: -4.94878; geodeticDatum: WGS84; **Event:** eventID: A; samplingProtocol: Pitfall
- **Type status:** Other material. **Occurrence:** individualCount: 1; sex: male; **Location:** locationID: P3; continent: Europe; country: Spain; countryCode: ES; stateProvince: Castilla y León; county: León; locality: Las Arroyas; verbatimElevation: 1097.1; decimalLatitude: 43.14351; decimalLongitude: -4.94878; geodeticDatum: WGS84; **Event:** eventID: B; samplingProtocol: Pitfall
- **Type status:** Other material. **Occurrence:** individualCount: 10; sex: male; **Location:** locationID: P3; continent: Europe; country: Spain; countryCode: ES; stateProvince: Castilla y León; county: León; locality: Las Arroyas; verbatimElevation: 1097.1; decimalLatitude: 43.14351; decimalLongitude: -4.94878; geodeticDatum: WGS84; **Event:** eventID: D; samplingProtocol: Pitfall
- **Type status:** Other material. **Occurrence:** individualCount: 5; sex: female; **Location:** locationID: P3; continent: Europe; country: Spain; countryCode: ES; stateProvince: Castilla y León; county: León; locality: Las Arroyas; verbatimElevation: 1097.1; decimalLatitude: 43.14351; decimalLongitude: -4.94878; geodeticDatum: WGS84; **Event:** eventID: D; samplingProtocol: Pitfall
- **Type status:** Other material. **Occurrence:** individualCount: 12; sex: male; **Location:** locationID: P3; continent: Europe; country: Spain; countryCode: ES; stateProvince: Castilla y León; county: León; locality: Las Arroyas; verbatimElevation: 1097.1; decimalLatitude: 43.14351; decimalLongitude: -4.94878; geodeticDatum: WGS84; **Event:** eventID: E; samplingProtocol: Pitfall
- **Type status:** Other material. **Occurrence:** individualCount: 6; sex: male; **Location:** locationID: P3; continent: Europe; country: Spain; countryCode: ES; stateProvince: Castilla y León; county: León; locality: Las Arroyas; verbatimElevation: 1097.1; decimalLatitude: 43.14351; decimalLongitude: -4.94878; geodeticDatum: WGS84; **Event:** eventID: F; samplingProtocol: Pitfall
- **Type status:** Other material. **Occurrence:** individualCount: 4; sex: male; **Location:** locationID: P3; continent: Europe; country: Spain; countryCode: ES; stateProvince: Castilla y León; county: León; locality: Las Arroyas; verbatimElevation: 1097.1; decimalLatitude: 43.14351; decimalLongitude: -4.94878; geodeticDatum: WGS84; **Event:** eventID: G; samplingProtocol: Pitfall
- **Type status:** Other material. **Occurrence:** individualCount: 20; sex: male; **Location:** locationID: P3; continent: Europe; country: Spain; countryCode: ES; stateProvince: Castilla y León; county: León; locality: Las Arroyas; verbatimElevation: 1097.1; decimalLatitude: 43.14351; decimalLongitude: -4.94878; geodeticDatum: WGS84; **Event:** eventID: H; samplingProtocol: Pitfall
- **Type status:** Other material. **Occurrence:** individualCount: 4; sex: female; **Location:** locationID: P3; continent: Europe; country: Spain; countryCode: ES; stateProvince: Castilla y León; county: León; locality: Las Arroyas; verbatimElevation: 1097.1; decimalLatitude: 43.14351; decimalLongitude: -4.94878; geodeticDatum: WGS84; **Event:** eventID: H; samplingProtocol: Pitfall
- **Type status:** Other material. **Occurrence:** individualCount: 5; sex: male; **Location:** locationID: P3; continent: Europe; country: Spain; countryCode: ES; stateProvince: Castilla y León; county: León; locality: Las Arroyas; verbatimElevation: 1097.1; decimalLatitude: 43.14351; decimalLongitude: -4.94878; geodeticDatum: WGS84; **Event:** eventID: I; samplingProtocol: Pitfall
- **Type status:** Other material. **Occurrence:** individualCount: 1; sex: female; **Location:** locationID: P3; continent: Europe; country: Spain; countryCode: ES; stateProvince: Castilla y León; county: León; locality: Las Arroyas; verbatimElevation: 1097.1; decimalLatitude: 43.14351; decimalLongitude: -4.94878; geodeticDatum: WGS84; **Event:** eventID: I; samplingProtocol: Pitfall
- **Type status:** Other material. **Occurrence:** individualCount: 3; sex: male; **Location:** locationID: P3; continent: Europe; country: Spain; countryCode: ES; stateProvince: Castilla y León; county: León; locality: Las Arroyas; verbatimElevation: 1097.1; decimalLatitude: 43.14351; decimalLongitude: -4.94878; geodeticDatum: WGS84; **Event:** eventID: J; samplingProtocol: Pitfall
- **Type status:** Other material. **Occurrence:** individualCount: 3; sex: female; **Location:** locationID: P3; continent: Europe; country: Spain; countryCode: ES; stateProvince: Castilla y León; county: León; locality: Las Arroyas; verbatimElevation: 1097.1; decimalLatitude: 43.14351; decimalLongitude: -4.94878; geodeticDatum: WGS84; **Event:** eventID: J; samplingProtocol: Pitfall
- **Type status:** Other material. **Occurrence:** individualCount: 6; sex: male; **Location:** locationID: P3; continent: Europe; country: Spain; countryCode: ES; stateProvince: Castilla y León; county: León; locality: Las Arroyas; verbatimElevation: 1097.1; decimalLatitude: 43.14351; decimalLongitude: -4.94878; geodeticDatum: WGS84; **Event:** eventID: K; samplingProtocol: Pitfall
- **Type status:** Other material. **Occurrence:** individualCount: 2; sex: female; **Location:** locationID: P3; continent: Europe; country: Spain; countryCode: ES; stateProvince: Castilla y León; county: León; locality: Las Arroyas; verbatimElevation: 1097.1; decimalLatitude: 43.14351; decimalLongitude: -4.94878; geodeticDatum: WGS84; **Event:** eventID: K; samplingProtocol: Pitfall
- **Type status:** Other material. **Occurrence:** individualCount: 20; sex: male; **Location:** locationID: P3; continent: Europe; country: Spain; countryCode: ES; stateProvince: Castilla y León; county: León; locality: Las Arroyas; verbatimElevation: 1097.1; decimalLatitude: 43.14351; decimalLongitude: -4.94878; geodeticDatum: WGS84; **Event:** eventID: L; samplingProtocol: Pitfall
- **Type status:** Other material. **Occurrence:** individualCount: 1; sex: female; **Location:** locationID: P3; continent: Europe; country: Spain; countryCode: ES; stateProvince: Castilla y León; county: León; locality: Las Arroyas; verbatimElevation: 1097.1; decimalLatitude: 43.14351; decimalLongitude: -4.94878; geodeticDatum: WGS84; **Event:** eventID: L; samplingProtocol: Pitfall
- **Type status:** Other material. **Occurrence:** individualCount: 1; sex: female; **Location:** locationID: P3; continent: Europe; country: Spain; countryCode: ES; stateProvince: Castilla y León; county: León; locality: Las Arroyas; verbatimElevation: 1097.1; decimalLatitude: 43.14351; decimalLongitude: -4.94878; geodeticDatum: WGS84; **Event:** eventID: 1; samplingProtocol: Sweeping; eventTime: Day
- **Type status:** Other material. **Occurrence:** individualCount: 2; sex: male; **Location:** locationID: P4; continent: Europe; country: Spain; countryCode: ES; stateProvince: Castilla y León; county: León; locality: El Canto; verbatimElevation: 943.48; decimalLatitude: 43.17227; decimalLongitude: -4.90857; geodeticDatum: WGS84; **Event:** eventID: A; samplingProtocol: Pitfall
- **Type status:** Other material. **Occurrence:** individualCount: 6; sex: male; **Location:** locationID: P4; continent: Europe; country: Spain; countryCode: ES; stateProvince: Castilla y León; county: León; locality: El Canto; verbatimElevation: 943.48; decimalLatitude: 43.17227; decimalLongitude: -4.90857; geodeticDatum: WGS84; **Event:** eventID: B; samplingProtocol: Pitfall
- **Type status:** Other material. **Occurrence:** individualCount: 3; sex: female; **Location:** locationID: P4; continent: Europe; country: Spain; countryCode: ES; stateProvince: Castilla y León; county: León; locality: El Canto; verbatimElevation: 943.48; decimalLatitude: 43.17227; decimalLongitude: -4.90857; geodeticDatum: WGS84; **Event:** eventID: B; samplingProtocol: Pitfall
- **Type status:** Other material. **Occurrence:** individualCount: 4; sex: male; **Location:** locationID: P4; continent: Europe; country: Spain; countryCode: ES; stateProvince: Castilla y León; county: León; locality: El Canto; verbatimElevation: 943.48; decimalLatitude: 43.17227; decimalLongitude: -4.90857; geodeticDatum: WGS84; **Event:** eventID: D; samplingProtocol: Pitfall
- **Type status:** Other material. **Occurrence:** individualCount: 9; sex: male; **Location:** locationID: P4; continent: Europe; country: Spain; countryCode: ES; stateProvince: Castilla y León; county: León; locality: El Canto; verbatimElevation: 943.48; decimalLatitude: 43.17227; decimalLongitude: -4.90857; geodeticDatum: WGS84; **Event:** eventID: E; samplingProtocol: Pitfall
- **Type status:** Other material. **Occurrence:** individualCount: 2; sex: female; **Location:** locationID: P4; continent: Europe; country: Spain; countryCode: ES; stateProvince: Castilla y León; county: León; locality: El Canto; verbatimElevation: 943.48; decimalLatitude: 43.17227; decimalLongitude: -4.90857; geodeticDatum: WGS84; **Event:** eventID: E; samplingProtocol: Pitfall
- **Type status:** Other material. **Occurrence:** individualCount: 1; sex: male; **Location:** locationID: P4; continent: Europe; country: Spain; countryCode: ES; stateProvince: Castilla y León; county: León; locality: El Canto; verbatimElevation: 943.48; decimalLatitude: 43.17227; decimalLongitude: -4.90857; geodeticDatum: WGS84; **Event:** eventID: F; samplingProtocol: Pitfall
- **Type status:** Other material. **Occurrence:** individualCount: 1; sex: male; **Location:** locationID: P4; continent: Europe; country: Spain; countryCode: ES; stateProvince: Castilla y León; county: León; locality: El Canto; verbatimElevation: 943.48; decimalLatitude: 43.17227; decimalLongitude: -4.90857; geodeticDatum: WGS84; **Event:** eventID: G; samplingProtocol: Pitfall
- **Type status:** Other material. **Occurrence:** individualCount: 3; sex: male; **Location:** locationID: P4; continent: Europe; country: Spain; countryCode: ES; stateProvince: Castilla y León; county: León; locality: El Canto; verbatimElevation: 943.48; decimalLatitude: 43.17227; decimalLongitude: -4.90857; geodeticDatum: WGS84; **Event:** eventID: H; samplingProtocol: Pitfall
- **Type status:** Other material. **Occurrence:** individualCount: 1; sex: female; **Location:** locationID: P4; continent: Europe; country: Spain; countryCode: ES; stateProvince: Castilla y León; county: León; locality: El Canto; verbatimElevation: 943.48; decimalLatitude: 43.17227; decimalLongitude: -4.90857; geodeticDatum: WGS84; **Event:** eventID: H; samplingProtocol: Pitfall
- **Type status:** Other material. **Occurrence:** individualCount: 12; sex: male; **Location:** locationID: P4; continent: Europe; country: Spain; countryCode: ES; stateProvince: Castilla y León; county: León; locality: El Canto; verbatimElevation: 943.48; decimalLatitude: 43.17227; decimalLongitude: -4.90857; geodeticDatum: WGS84; **Event:** eventID: I; samplingProtocol: Pitfall
- **Type status:** Other material. **Occurrence:** individualCount: 4; sex: male; **Location:** locationID: P4; continent: Europe; country: Spain; countryCode: ES; stateProvince: Castilla y León; county: León; locality: El Canto; verbatimElevation: 943.48; decimalLatitude: 43.17227; decimalLongitude: -4.90857; geodeticDatum: WGS84; **Event:** eventID: J; samplingProtocol: Pitfall
- **Type status:** Other material. **Occurrence:** individualCount: 1; sex: male; **Location:** locationID: P4; continent: Europe; country: Spain; countryCode: ES; stateProvince: Castilla y León; county: León; locality: El Canto; verbatimElevation: 943.48; decimalLatitude: 43.17227; decimalLongitude: -4.90857; geodeticDatum: WGS84; **Event:** eventID: K; samplingProtocol: Pitfall
- **Type status:** Other material. **Occurrence:** individualCount: 9; sex: male; **Location:** locationID: P4; continent: Europe; country: Spain; countryCode: ES; stateProvince: Castilla y León; county: León; locality: El Canto; verbatimElevation: 943.48; decimalLatitude: 43.17227; decimalLongitude: -4.90857; geodeticDatum: WGS84; **Event:** eventID: L; samplingProtocol: Pitfall
- **Type status:** Other material. **Occurrence:** individualCount: 2; sex: female; **Location:** locationID: P4; continent: Europe; country: Spain; countryCode: ES; stateProvince: Castilla y León; county: León; locality: El Canto; verbatimElevation: 943.48; decimalLatitude: 43.17227; decimalLongitude: -4.90857; geodeticDatum: WGS84; **Event:** eventID: L; samplingProtocol: Pitfall

##### Distribution

Palearctic

#### Pardosa nigriceps

(Thorell, 1856)

##### Materials

- **Type status:** Other material. **Occurrence:** individualCount: 1; sex: male; **Location:** locationID: P2; continent: Europe; country: Spain; countryCode: ES; stateProvince: Castilla y León; county: León; locality: Joyoguelas; verbatimElevation: 763.98; decimalLatitude: 43.17771; decimalLongitude: -4.90579; geodeticDatum: WGS84; **Event:** eventID: C; samplingProtocol: Pitfall
- **Type status:** Other material. **Occurrence:** individualCount: 1; sex: male; **Location:** locationID: P4; continent: Europe; country: Spain; countryCode: ES; stateProvince: Castilla y León; county: León; locality: El Canto; verbatimElevation: 943.48; decimalLatitude: 43.17227; decimalLongitude: -4.90857; geodeticDatum: WGS84; **Event:** eventID: L; samplingProtocol: Pitfall

##### Distribution

Europe

#### Pardosa proxima

(C. L. Koch, 1847)

##### Materials

- **Type status:** Other material. **Occurrence:** individualCount: 1; sex: male; **Location:** locationID: C1; continent: Europe; country: Spain; countryCode: ES; stateProvince: Castilla-La Mancha; county: Ciudad Real; locality: Valle Brezoso; verbatimElevation: 756.56; decimalLatitude: 39.35663; decimalLongitude: -4.35912; geodeticDatum: WGS84; **Event:** eventID: B; samplingProtocol: Pitfall
- **Type status:** Other material. **Occurrence:** individualCount: 2; sex: female; **Location:** locationID: C1; continent: Europe; country: Spain; countryCode: ES; stateProvince: Castilla-La Mancha; county: Ciudad Real; locality: Valle Brezoso; verbatimElevation: 756.56; decimalLatitude: 39.35663; decimalLongitude: -4.35912; geodeticDatum: WGS84; **Event:** eventID: B; samplingProtocol: Pitfall
- **Type status:** Other material. **Occurrence:** individualCount: 2; sex: male; **Location:** locationID: C1; continent: Europe; country: Spain; countryCode: ES; stateProvince: Castilla-La Mancha; county: Ciudad Real; locality: Valle Brezoso; verbatimElevation: 756.56; decimalLatitude: 39.35663; decimalLongitude: -4.35912; geodeticDatum: WGS84; **Event:** eventID: C; samplingProtocol: Pitfall
- **Type status:** Other material. **Occurrence:** individualCount: 1; sex: female; **Location:** locationID: C1; continent: Europe; country: Spain; countryCode: ES; stateProvince: Castilla-La Mancha; county: Ciudad Real; locality: Valle Brezoso; verbatimElevation: 756.56; decimalLatitude: 39.35663; decimalLongitude: -4.35912; geodeticDatum: WGS84; **Event:** eventID: C; samplingProtocol: Pitfall
- **Type status:** Other material. **Occurrence:** individualCount: 1; sex: female; **Location:** locationID: C1; continent: Europe; country: Spain; countryCode: ES; stateProvince: Castilla-La Mancha; county: Ciudad Real; locality: Valle Brezoso; verbatimElevation: 756.56; decimalLatitude: 39.35663; decimalLongitude: -4.35912; geodeticDatum: WGS84; **Event:** eventID: D; samplingProtocol: Pitfall
- **Type status:** Other material. **Occurrence:** individualCount: 1; sex: female; **Location:** locationID: C1; continent: Europe; country: Spain; countryCode: ES; stateProvince: Castilla-La Mancha; county: Ciudad Real; locality: Valle Brezoso; verbatimElevation: 756.56; decimalLatitude: 39.35663; decimalLongitude: -4.35912; geodeticDatum: WGS84; **Event:** eventID: E; samplingProtocol: Pitfall
- **Type status:** Other material. **Occurrence:** individualCount: 1; sex: male; **Location:** locationID: C1; continent: Europe; country: Spain; countryCode: ES; stateProvince: Castilla-La Mancha; county: Ciudad Real; locality: Valle Brezoso; verbatimElevation: 756.56; decimalLatitude: 39.35663; decimalLongitude: -4.35912; geodeticDatum: WGS84; **Event:** eventID: G; samplingProtocol: Pitfall
- **Type status:** Other material. **Occurrence:** individualCount: 1; sex: female; **Location:** locationID: C1; continent: Europe; country: Spain; countryCode: ES; stateProvince: Castilla-La Mancha; county: Ciudad Real; locality: Valle Brezoso; verbatimElevation: 756.56; decimalLatitude: 39.35663; decimalLongitude: -4.35912; geodeticDatum: WGS84; **Event:** eventID: H; samplingProtocol: Pitfall
- **Type status:** Other material. **Occurrence:** individualCount: 1; sex: male; **Location:** locationID: C1; continent: Europe; country: Spain; countryCode: ES; stateProvince: Castilla-La Mancha; county: Ciudad Real; locality: Valle Brezoso; verbatimElevation: 756.56; decimalLatitude: 39.35663; decimalLongitude: -4.35912; geodeticDatum: WGS84; **Event:** eventID: J; samplingProtocol: Pitfall
- **Type status:** Other material. **Occurrence:** individualCount: 1; sex: male; **Location:** locationID: C1; continent: Europe; country: Spain; countryCode: ES; stateProvince: Castilla-La Mancha; county: Ciudad Real; locality: Valle Brezoso; verbatimElevation: 756.56; decimalLatitude: 39.35663; decimalLongitude: -4.35912; geodeticDatum: WGS84; **Event:** eventID: K; samplingProtocol: Pitfall
- **Type status:** Other material. **Occurrence:** individualCount: 1; sex: female; **Location:** locationID: C1; continent: Europe; country: Spain; countryCode: ES; stateProvince: Castilla-La Mancha; county: Ciudad Real; locality: Valle Brezoso; verbatimElevation: 756.56; decimalLatitude: 39.35663; decimalLongitude: -4.35912; geodeticDatum: WGS84; **Event:** eventID: K; samplingProtocol: Pitfall
- **Type status:** Other material. **Occurrence:** individualCount: 2; sex: female; **Location:** locationID: C1; continent: Europe; country: Spain; countryCode: ES; stateProvince: Castilla-La Mancha; county: Ciudad Real; locality: Valle Brezoso; verbatimElevation: 756.56; decimalLatitude: 39.35663; decimalLongitude: -4.35912; geodeticDatum: WGS84; **Event:** eventID: K; samplingProtocol: Pitfall
- **Type status:** Other material. **Occurrence:** individualCount: 1; sex: male; **Location:** locationID: C1; continent: Europe; country: Spain; countryCode: ES; stateProvince: Castilla-La Mancha; county: Ciudad Real; locality: Valle Brezoso; verbatimElevation: 756.56; decimalLatitude: 39.35663; decimalLongitude: -4.35912; geodeticDatum: WGS84; **Event:** eventID: L; samplingProtocol: Pitfall
- **Type status:** Other material. **Occurrence:** individualCount: 1; sex: male; **Location:** locationID: C2; continent: Europe; country: Spain; countryCode: ES; stateProvince: Castilla-La Mancha; county: Ciudad Real; locality: Valle Brezoso; verbatimElevation: 739.31; decimalLatitude: 39.35159; decimalLongitude: -4.3589; geodeticDatum: WGS84; **Event:** eventID: A; samplingProtocol: Pitfall
- **Type status:** Other material. **Occurrence:** individualCount: 1; sex: female; **Location:** locationID: C2; continent: Europe; country: Spain; countryCode: ES; stateProvince: Castilla-La Mancha; county: Ciudad Real; locality: Valle Brezoso; verbatimElevation: 739.31; decimalLatitude: 39.35159; decimalLongitude: -4.3589; geodeticDatum: WGS84; **Event:** eventID: B; samplingProtocol: Pitfall
- **Type status:** Other material. **Occurrence:** individualCount: 1; sex: female; **Location:** locationID: C2; continent: Europe; country: Spain; countryCode: ES; stateProvince: Castilla-La Mancha; county: Ciudad Real; locality: Valle Brezoso; verbatimElevation: 739.31; decimalLatitude: 39.35159; decimalLongitude: -4.3589; geodeticDatum: WGS84; **Event:** eventID: C; samplingProtocol: Pitfall
- **Type status:** Other material. **Occurrence:** individualCount: 1; sex: male; **Location:** locationID: C2; continent: Europe; country: Spain; countryCode: ES; stateProvince: Castilla-La Mancha; county: Ciudad Real; locality: Valle Brezoso; verbatimElevation: 739.31; decimalLatitude: 39.35159; decimalLongitude: -4.3589; geodeticDatum: WGS84; **Event:** eventID: D; samplingProtocol: Pitfall
- **Type status:** Other material. **Occurrence:** individualCount: 1; sex: male; **Location:** locationID: C2; continent: Europe; country: Spain; countryCode: ES; stateProvince: Castilla-La Mancha; county: Ciudad Real; locality: Valle Brezoso; verbatimElevation: 739.31; decimalLatitude: 39.35159; decimalLongitude: -4.3589; geodeticDatum: WGS84; **Event:** eventID: E; samplingProtocol: Pitfall
- **Type status:** Other material. **Occurrence:** individualCount: 1; sex: male; **Location:** locationID: C2; continent: Europe; country: Spain; countryCode: ES; stateProvince: Castilla-La Mancha; county: Ciudad Real; locality: Valle Brezoso; verbatimElevation: 739.31; decimalLatitude: 39.35159; decimalLongitude: -4.3589; geodeticDatum: WGS84; **Event:** eventID: G; samplingProtocol: Pitfall
- **Type status:** Other material. **Occurrence:** individualCount: 1; sex: male; **Location:** locationID: C2; continent: Europe; country: Spain; countryCode: ES; stateProvince: Castilla-La Mancha; county: Ciudad Real; locality: Valle Brezoso; verbatimElevation: 739.31; decimalLatitude: 39.35159; decimalLongitude: -4.3589; geodeticDatum: WGS84; **Event:** eventID: I; samplingProtocol: Pitfall
- **Type status:** Other material. **Occurrence:** individualCount: 4; sex: female; **Location:** locationID: C2; continent: Europe; country: Spain; countryCode: ES; stateProvince: Castilla-La Mancha; county: Ciudad Real; locality: Valle Brezoso; verbatimElevation: 739.31; decimalLatitude: 39.35159; decimalLongitude: -4.3589; geodeticDatum: WGS84; **Event:** eventID: I; samplingProtocol: Pitfall
- **Type status:** Other material. **Occurrence:** individualCount: 1; sex: male; **Location:** locationID: C2; continent: Europe; country: Spain; countryCode: ES; stateProvince: Castilla-La Mancha; county: Ciudad Real; locality: Valle Brezoso; verbatimElevation: 739.31; decimalLatitude: 39.35159; decimalLongitude: -4.3589; geodeticDatum: WGS84; **Event:** eventID: K; samplingProtocol: Pitfall
- **Type status:** Other material. **Occurrence:** individualCount: 1; sex: male; **Location:** locationID: C2; continent: Europe; country: Spain; countryCode: ES; stateProvince: Castilla-La Mancha; county: Ciudad Real; locality: Valle Brezoso; verbatimElevation: 739.31; decimalLatitude: 39.35159; decimalLongitude: -4.3589; geodeticDatum: WGS84; **Event:** eventID: L; samplingProtocol: Pitfall
- **Type status:** Other material. **Occurrence:** individualCount: 1; sex: female; **Location:** locationID: C2; continent: Europe; country: Spain; countryCode: ES; stateProvince: Castilla-La Mancha; county: Ciudad Real; locality: Valle Brezoso; verbatimElevation: 739.31; decimalLatitude: 39.35159; decimalLongitude: -4.3589; geodeticDatum: WGS84; **Event:** eventID: K; samplingProtocol: Pitfall
- **Type status:** Other material. **Occurrence:** individualCount: 1; sex: male; **Location:** locationID: C3; continent: Europe; country: Spain; countryCode: ES; stateProvince: Castilla-La Mancha; county: Ciudad Real; locality: La Quesera; verbatimElevation: 767.55; decimalLatitude: 39.36177; decimalLongitude: -4.41733; geodeticDatum: WGS84; **Event:** eventID: A; samplingProtocol: Pitfall
- **Type status:** Other material. **Occurrence:** individualCount: 1; sex: male; **Location:** locationID: C3; continent: Europe; country: Spain; countryCode: ES; stateProvince: Castilla-La Mancha; county: Ciudad Real; locality: La Quesera; verbatimElevation: 767.55; decimalLatitude: 39.36177; decimalLongitude: -4.41733; geodeticDatum: WGS84; **Event:** eventID: D; samplingProtocol: Pitfall
- **Type status:** Other material. **Occurrence:** individualCount: 2; sex: male; **Location:** locationID: C3; continent: Europe; country: Spain; countryCode: ES; stateProvince: Castilla-La Mancha; county: Ciudad Real; locality: La Quesera; verbatimElevation: 767.55; decimalLatitude: 39.36177; decimalLongitude: -4.41733; geodeticDatum: WGS84; **Event:** eventID: H; samplingProtocol: Pitfall
- **Type status:** Other material. **Occurrence:** individualCount: 1; sex: female; **Location:** locationID: C3; continent: Europe; country: Spain; countryCode: ES; stateProvince: Castilla-La Mancha; county: Ciudad Real; locality: La Quesera; verbatimElevation: 767.55; decimalLatitude: 39.36177; decimalLongitude: -4.41733; geodeticDatum: WGS84; **Event:** eventID: I; samplingProtocol: Pitfall
- **Type status:** Other material. **Occurrence:** individualCount: 3; sex: female; **Location:** locationID: C4; continent: Europe; country: Spain; countryCode: ES; stateProvince: Castilla-La Mancha; county: Ciudad Real; locality: La Quesera; verbatimElevation: 772.3; decimalLatitude: 39.36337; decimalLongitude: -4.41704; geodeticDatum: WGS84; **Event:** eventID: A; samplingProtocol: Pitfall
- **Type status:** Other material. **Occurrence:** individualCount: 20; sex: male; **Location:** locationID: C4; continent: Europe; country: Spain; countryCode: ES; stateProvince: Castilla-La Mancha; county: Ciudad Real; locality: La Quesera; verbatimElevation: 772.3; decimalLatitude: 39.36337; decimalLongitude: -4.41704; geodeticDatum: WGS84; **Event:** eventID: A; samplingProtocol: Pitfall
- **Type status:** Other material. **Occurrence:** individualCount: 1; sex: female; **Location:** locationID: C4; continent: Europe; country: Spain; countryCode: ES; stateProvince: Castilla-La Mancha; county: Ciudad Real; locality: La Quesera; verbatimElevation: 772.3; decimalLatitude: 39.36337; decimalLongitude: -4.41704; geodeticDatum: WGS84; **Event:** eventID: F; samplingProtocol: Pitfall
- **Type status:** Other material. **Occurrence:** individualCount: 1; sex: male; **Location:** locationID: C4; continent: Europe; country: Spain; countryCode: ES; stateProvince: Castilla-La Mancha; county: Ciudad Real; locality: La Quesera; verbatimElevation: 772.3; decimalLatitude: 39.36337; decimalLongitude: -4.41704; geodeticDatum: WGS84; **Event:** eventID: F; samplingProtocol: Pitfall
- **Type status:** Other material. **Occurrence:** individualCount: 1; sex: female; **Location:** locationID: C4; continent: Europe; country: Spain; countryCode: ES; stateProvince: Castilla-La Mancha; county: Ciudad Real; locality: La Quesera; verbatimElevation: 772.3; decimalLatitude: 39.36337; decimalLongitude: -4.41704; geodeticDatum: WGS84; **Event:** eventID: H; samplingProtocol: Pitfall
- **Type status:** Other material. **Occurrence:** individualCount: 4; sex: male; **Location:** locationID: C4; continent: Europe; country: Spain; countryCode: ES; stateProvince: Castilla-La Mancha; county: Ciudad Real; locality: La Quesera; verbatimElevation: 772.3; decimalLatitude: 39.36337; decimalLongitude: -4.41704; geodeticDatum: WGS84; **Event:** eventID: H; samplingProtocol: Pitfall
- **Type status:** Other material. **Occurrence:** individualCount: 1; sex: male; **Location:** locationID: C4; continent: Europe; country: Spain; countryCode: ES; stateProvince: Castilla-La Mancha; county: Ciudad Real; locality: La Quesera; verbatimElevation: 772.3; decimalLatitude: 39.36337; decimalLongitude: -4.41704; geodeticDatum: WGS84; **Event:** eventID: J; samplingProtocol: Pitfall
- **Type status:** Other material. **Occurrence:** individualCount: 1; sex: male; **Location:** locationID: C4; continent: Europe; country: Spain; countryCode: ES; stateProvince: Castilla-La Mancha; county: Ciudad Real; locality: La Quesera; verbatimElevation: 772.3; decimalLatitude: 39.36337; decimalLongitude: -4.41704; geodeticDatum: WGS84; **Event:** eventID: L; samplingProtocol: Pitfall
- **Type status:** Other material. **Occurrence:** individualCount: 1; sex: female; **Location:** locationID: C4; continent: Europe; country: Spain; countryCode: ES; stateProvince: Castilla-La Mancha; county: Ciudad Real; locality: La Quesera; verbatimElevation: 772.3; decimalLatitude: 39.36337; decimalLongitude: -4.41704; geodeticDatum: WGS84; **Event:** eventID: 1; samplingProtocol: Sweeping; eventTime: Day

##### Distribution

Palearctic, Canary Islands, Azores

#### Pardosa pullata

(Clerck, 1757)

##### Materials

- **Type status:** Other material. **Occurrence:** individualCount: 1; sex: female; **Location:** locationID: P4; continent: Europe; country: Spain; countryCode: ES; stateProvince: Castilla y León; county: León; locality: El Canto; verbatimElevation: 943.48; decimalLatitude: 43.17227; decimalLongitude: -4.90857; geodeticDatum: WGS84; **Event:** eventID: 1; samplingProtocol: Sweeping; eventTime: Night

##### Distribution

Europe, Russia, Central Asia

#### Pirata piraticus

(Clerck, 1757)

##### Materials

- **Type status:** Other material. **Occurrence:** individualCount: 1; sex: female; **Location:** locationID: C4; continent: Europe; country: Spain; countryCode: ES; stateProvince: Castilla-La Mancha; county: Ciudad Real; locality: La Quesera; verbatimElevation: 772.3; decimalLatitude: 39.36337; decimalLongitude: -4.41704; geodeticDatum: WGS84; **Event:** eventID: A; samplingProtocol: Pitfall

##### Distribution

Holarctic

#### Piratula latitans

(Blackwall, 1841)

##### Materials

- **Type status:** Other material. **Occurrence:** individualCount: 1; sex: male; **Location:** locationID: C1; continent: Europe; country: Spain; countryCode: ES; stateProvince: Castilla-La Mancha; county: Ciudad Real; locality: Valle Brezoso; verbatimElevation: 756.56; decimalLatitude: 39.35663; decimalLongitude: -4.35912; geodeticDatum: WGS84; **Event:** eventID: C; samplingProtocol: Pitfall

##### Distribution

Europe to Azerbaijan

#### Trabea cazorla

Snazell, 1983

##### Materials

- **Type status:** Other material. **Occurrence:** individualCount: 2; sex: male; **Location:** locationID: C1; continent: Europe; country: Spain; countryCode: ES; stateProvince: Castilla-La Mancha; county: Ciudad Real; locality: Valle Brezoso; verbatimElevation: 756.56; decimalLatitude: 39.35663; decimalLongitude: -4.35912; geodeticDatum: WGS84; **Event:** eventID: B; samplingProtocol: Pitfall
- **Type status:** Other material. **Occurrence:** individualCount: 1; sex: male; **Location:** locationID: C1; continent: Europe; country: Spain; countryCode: ES; stateProvince: Castilla-La Mancha; county: Ciudad Real; locality: Valle Brezoso; verbatimElevation: 756.56; decimalLatitude: 39.35663; decimalLongitude: -4.35912; geodeticDatum: WGS84; **Event:** eventID: J; samplingProtocol: Pitfall
- **Type status:** Other material. **Occurrence:** individualCount: 1; sex: female; **Location:** locationID: C4; continent: Europe; country: Spain; countryCode: ES; stateProvince: Castilla-La Mancha; county: Ciudad Real; locality: La Quesera; verbatimElevation: 772.3; decimalLatitude: 39.36337; decimalLongitude: -4.41704; geodeticDatum: WGS84; **Event:** eventID: A; samplingProtocol: Pitfall
- **Type status:** Other material. **Occurrence:** individualCount: 1; sex: male; **Location:** locationID: C4; continent: Europe; country: Spain; countryCode: ES; stateProvince: Castilla-La Mancha; county: Ciudad Real; locality: La Quesera; verbatimElevation: 772.3; decimalLatitude: 39.36337; decimalLongitude: -4.41704; geodeticDatum: WGS84; **Event:** eventID: E; samplingProtocol: Pitfall
- **Type status:** Other material. **Occurrence:** individualCount: 1; sex: male; **Location:** locationID: O2; continent: Europe; country: Spain; countryCode: ES; stateProvince: Aragón; county: Huesca; locality: Rebilla; verbatimElevation: 1158.13; decimalLatitude: 42.59427; decimalLongitude: 0.1529; geodeticDatum: WGS84; **Event:** eventID: G; samplingProtocol: Pitfall
- **Type status:** Other material. **Occurrence:** individualCount: 1; sex: female; **Location:** locationID: O2; continent: Europe; country: Spain; countryCode: ES; stateProvince: Aragón; county: Huesca; locality: Rebilla; verbatimElevation: 1158.13; decimalLatitude: 42.59427; decimalLongitude: 0.1529; geodeticDatum: WGS84; **Event:** eventID: I; samplingProtocol: Pitfall
- **Type status:** Other material. **Occurrence:** individualCount: 1; sex: male; **Location:** locationID: S1; continent: Europe; country: Spain; countryCode: ES; stateProvince: Andalucía; county: Granada; locality: Soportujar; verbatimElevation: 1786.57; decimalLatitude: 36.96151; decimalLongitude: -3.41881; geodeticDatum: WGS84; **Event:** eventID: J; samplingProtocol: Pitfall

##### Distribution

Spain, Morocco, Algeria

#### Trochosa robusta

(Simon, 1876)

##### Materials

- **Type status:** Other material. **Occurrence:** individualCount: 2; sex: male; **Location:** locationID: C1; continent: Europe; country: Spain; countryCode: ES; stateProvince: Castilla-La Mancha; county: Ciudad Real; locality: Valle Brezoso; verbatimElevation: 756.56; decimalLatitude: 39.35663; decimalLongitude: -4.35912; geodeticDatum: WGS84; **Event:** eventID: C; samplingProtocol: Pitfall
- **Type status:** Other material. **Occurrence:** individualCount: 1; sex: female; **Location:** locationID: C1; continent: Europe; country: Spain; countryCode: ES; stateProvince: Castilla-La Mancha; county: Ciudad Real; locality: Valle Brezoso; verbatimElevation: 756.56; decimalLatitude: 39.35663; decimalLongitude: -4.35912; geodeticDatum: WGS84; **Event:** eventID: C; samplingProtocol: Pitfall
- **Type status:** Other material. **Occurrence:** individualCount: 1; sex: male; **Location:** locationID: C4; continent: Europe; country: Spain; countryCode: ES; stateProvince: Castilla-La Mancha; county: Ciudad Real; locality: La Quesera; verbatimElevation: 772.3; decimalLatitude: 39.36337; decimalLongitude: -4.41704; geodeticDatum: WGS84; **Event:** eventID: A; samplingProtocol: Pitfall

##### Distribution

Palearctic

#### Trochosa terricola

Thorell, 1856

##### Materials

- **Type status:** Other material. **Occurrence:** individualCount: 2; sex: male; **Location:** locationID: O1; continent: Europe; country: Spain; countryCode: ES; stateProvince: Aragón; county: Huesca; locality: O Furno; verbatimElevation: 1396.73; decimalLatitude: 42.60677; decimalLongitude: 0.13135; geodeticDatum: WGS84; **Event:** eventID: C; samplingProtocol: Pitfall
- **Type status:** Other material. **Occurrence:** individualCount: 1; sex: male; **Location:** locationID: O1; continent: Europe; country: Spain; countryCode: ES; stateProvince: Aragón; county: Huesca; locality: O Furno; verbatimElevation: 1396.73; decimalLatitude: 42.60677; decimalLongitude: 0.13135; geodeticDatum: WGS84; **Event:** eventID: D; samplingProtocol: Pitfall
- **Type status:** Other material. **Occurrence:** individualCount: 1; sex: male; **Location:** locationID: O2; continent: Europe; country: Spain; countryCode: ES; stateProvince: Aragón; county: Huesca; locality: Rebilla; verbatimElevation: 1158.13; decimalLatitude: 42.59427; decimalLongitude: 0.1529; geodeticDatum: WGS84; **Event:** eventID: E; samplingProtocol: Pitfall
- **Type status:** Other material. **Occurrence:** individualCount: 2; sex: female; **Location:** locationID: P2; continent: Europe; country: Spain; countryCode: ES; stateProvince: Castilla y León; county: León; locality: Joyoguelas; verbatimElevation: 763.98; decimalLatitude: 43.17771; decimalLongitude: -4.90579; geodeticDatum: WGS84; **Event:** eventID: B; samplingProtocol: Pitfall
- **Type status:** Other material. **Occurrence:** individualCount: 4; sex: female; **Location:** locationID: P2; continent: Europe; country: Spain; countryCode: ES; stateProvince: Castilla y León; county: León; locality: Joyoguelas; verbatimElevation: 763.98; decimalLatitude: 43.17771; decimalLongitude: -4.90579; geodeticDatum: WGS84; **Event:** eventID: C; samplingProtocol: Pitfall
- **Type status:** Other material. **Occurrence:** individualCount: 2; sex: male; **Location:** locationID: P2; continent: Europe; country: Spain; countryCode: ES; stateProvince: Castilla y León; county: León; locality: Joyoguelas; verbatimElevation: 763.98; decimalLatitude: 43.17771; decimalLongitude: -4.90579; geodeticDatum: WGS84; **Event:** eventID: C; samplingProtocol: Pitfall
- **Type status:** Other material. **Occurrence:** individualCount: 1; sex: female; **Location:** locationID: P2; continent: Europe; country: Spain; countryCode: ES; stateProvince: Castilla y León; county: León; locality: Joyoguelas; verbatimElevation: 763.98; decimalLatitude: 43.17771; decimalLongitude: -4.90579; geodeticDatum: WGS84; **Event:** eventID: D; samplingProtocol: Pitfall
- **Type status:** Other material. **Occurrence:** individualCount: 3; sex: male; **Location:** locationID: P2; continent: Europe; country: Spain; countryCode: ES; stateProvince: Castilla y León; county: León; locality: Joyoguelas; verbatimElevation: 763.98; decimalLatitude: 43.17771; decimalLongitude: -4.90579; geodeticDatum: WGS84; **Event:** eventID: D; samplingProtocol: Pitfall
- **Type status:** Other material. **Occurrence:** individualCount: 1; sex: female; **Location:** locationID: P2; continent: Europe; country: Spain; countryCode: ES; stateProvince: Castilla y León; county: León; locality: Joyoguelas; verbatimElevation: 763.98; decimalLatitude: 43.17771; decimalLongitude: -4.90579; geodeticDatum: WGS84; **Event:** eventID: F; samplingProtocol: Pitfall
- **Type status:** Other material. **Occurrence:** individualCount: 1; sex: male; **Location:** locationID: P2; continent: Europe; country: Spain; countryCode: ES; stateProvince: Castilla y León; county: León; locality: Joyoguelas; verbatimElevation: 763.98; decimalLatitude: 43.17771; decimalLongitude: -4.90579; geodeticDatum: WGS84; **Event:** eventID: G; samplingProtocol: Pitfall
- **Type status:** Other material. **Occurrence:** individualCount: 1; sex: female; **Location:** locationID: P2; continent: Europe; country: Spain; countryCode: ES; stateProvince: Castilla y León; county: León; locality: Joyoguelas; verbatimElevation: 763.98; decimalLatitude: 43.17771; decimalLongitude: -4.90579; geodeticDatum: WGS84; **Event:** eventID: H; samplingProtocol: Pitfall
- **Type status:** Other material. **Occurrence:** individualCount: 2; sex: male; **Location:** locationID: P2; continent: Europe; country: Spain; countryCode: ES; stateProvince: Castilla y León; county: León; locality: Joyoguelas; verbatimElevation: 763.98; decimalLatitude: 43.17771; decimalLongitude: -4.90579; geodeticDatum: WGS84; **Event:** eventID: H; samplingProtocol: Pitfall
- **Type status:** Other material. **Occurrence:** individualCount: 2; sex: female; **Location:** locationID: P2; continent: Europe; country: Spain; countryCode: ES; stateProvince: Castilla y León; county: León; locality: Joyoguelas; verbatimElevation: 763.98; decimalLatitude: 43.17771; decimalLongitude: -4.90579; geodeticDatum: WGS84; **Event:** eventID: I; samplingProtocol: Pitfall
- **Type status:** Other material. **Occurrence:** individualCount: 3; sex: male; **Location:** locationID: P2; continent: Europe; country: Spain; countryCode: ES; stateProvince: Castilla y León; county: León; locality: Joyoguelas; verbatimElevation: 763.98; decimalLatitude: 43.17771; decimalLongitude: -4.90579; geodeticDatum: WGS84; **Event:** eventID: I; samplingProtocol: Pitfall
- **Type status:** Other material. **Occurrence:** individualCount: 1; sex: female; **Location:** locationID: P2; continent: Europe; country: Spain; countryCode: ES; stateProvince: Castilla y León; county: León; locality: Joyoguelas; verbatimElevation: 763.98; decimalLatitude: 43.17771; decimalLongitude: -4.90579; geodeticDatum: WGS84; **Event:** eventID: J; samplingProtocol: Pitfall
- **Type status:** Other material. **Occurrence:** individualCount: 1; sex: male; **Location:** locationID: P2; continent: Europe; country: Spain; countryCode: ES; stateProvince: Castilla y León; county: León; locality: Joyoguelas; verbatimElevation: 763.98; decimalLatitude: 43.17771; decimalLongitude: -4.90579; geodeticDatum: WGS84; **Event:** eventID: J; samplingProtocol: Pitfall
- **Type status:** Other material. **Occurrence:** individualCount: 1; sex: male; **Location:** locationID: P3; continent: Europe; country: Spain; countryCode: ES; stateProvince: Castilla y León; county: León; locality: Las Arroyas; verbatimElevation: 1097.1; decimalLatitude: 43.14351; decimalLongitude: -4.94878; geodeticDatum: WGS84; **Event:** eventID: J; samplingProtocol: Pitfall
- **Type status:** Other material. **Occurrence:** individualCount: 2; sex: male; **Location:** locationID: P4; continent: Europe; country: Spain; countryCode: ES; stateProvince: Castilla y León; county: León; locality: El Canto; verbatimElevation: 943.48; decimalLatitude: 43.17227; decimalLongitude: -4.90857; geodeticDatum: WGS84; **Event:** eventID: A; samplingProtocol: Pitfall
- **Type status:** Other material. **Occurrence:** individualCount: 1; sex: male; **Location:** locationID: P4; continent: Europe; country: Spain; countryCode: ES; stateProvince: Castilla y León; county: León; locality: El Canto; verbatimElevation: 943.48; decimalLatitude: 43.17227; decimalLongitude: -4.90857; geodeticDatum: WGS84; **Event:** eventID: D; samplingProtocol: Pitfall
- **Type status:** Other material. **Occurrence:** individualCount: 1; sex: male; **Location:** locationID: P4; continent: Europe; country: Spain; countryCode: ES; stateProvince: Castilla y León; county: León; locality: El Canto; verbatimElevation: 943.48; decimalLatitude: 43.17227; decimalLongitude: -4.90857; geodeticDatum: WGS84; **Event:** eventID: E; samplingProtocol: Pitfall
- **Type status:** Other material. **Occurrence:** individualCount: 1; sex: female; **Location:** locationID: P4; continent: Europe; country: Spain; countryCode: ES; stateProvince: Castilla y León; county: León; locality: El Canto; verbatimElevation: 943.48; decimalLatitude: 43.17227; decimalLongitude: -4.90857; geodeticDatum: WGS84; **Event:** eventID: I; samplingProtocol: Pitfall
- **Type status:** Other material. **Occurrence:** individualCount: 1; sex: male; **Location:** locationID: P4; continent: Europe; country: Spain; countryCode: ES; stateProvince: Castilla y León; county: León; locality: El Canto; verbatimElevation: 943.48; decimalLatitude: 43.17227; decimalLongitude: -4.90857; geodeticDatum: WGS84; **Event:** eventID: I; samplingProtocol: Pitfall

##### Distribution

Holarctic

#### Xerolycosa nemoralis

(Westring, 1861)

##### Materials

- **Type status:** Other material. **Occurrence:** individualCount: 1; sex: male; **Location:** locationID: A1; continent: Europe; country: Spain; countryCode: ES; stateProvince: Catalonia; county: Lleida; locality: Sola de Boi; verbatimElevation: 1759.8; decimalLatitude: 42.54958; decimalLongitude: 0.87254; geodeticDatum: WGS84; **Event:** eventID: 2; samplingProtocol: Ground; eventTime: Night
- **Type status:** Other material. **Occurrence:** individualCount: 3; sex: female; **Location:** locationID: A1; continent: Europe; country: Spain; countryCode: ES; stateProvince: Catalonia; county: Lleida; locality: Sola de Boi; verbatimElevation: 1759.8; decimalLatitude: 42.54958; decimalLongitude: 0.87254; geodeticDatum: WGS84; **Event:** eventID: C; samplingProtocol: Pitfall
- **Type status:** Other material. **Occurrence:** individualCount: 1; sex: female; **Location:** locationID: A1; continent: Europe; country: Spain; countryCode: ES; stateProvince: Catalonia; county: Lleida; locality: Sola de Boi; verbatimElevation: 1759.8; decimalLatitude: 42.54958; decimalLongitude: 0.87254; geodeticDatum: WGS84; **Event:** eventID: F; samplingProtocol: Pitfall
- **Type status:** Other material. **Occurrence:** individualCount: 2; sex: male; **Location:** locationID: A1; continent: Europe; country: Spain; countryCode: ES; stateProvince: Catalonia; county: Lleida; locality: Sola de Boi; verbatimElevation: 1759.8; decimalLatitude: 42.54958; decimalLongitude: 0.87254; geodeticDatum: WGS84; **Event:** eventID: G; samplingProtocol: Pitfall
- **Type status:** Other material. **Occurrence:** individualCount: 1; sex: female; **Location:** locationID: A1; continent: Europe; country: Spain; countryCode: ES; stateProvince: Catalonia; county: Lleida; locality: Sola de Boi; verbatimElevation: 1759.8; decimalLatitude: 42.54958; decimalLongitude: 0.87254; geodeticDatum: WGS84; **Event:** eventID: G; samplingProtocol: Pitfall
- **Type status:** Other material. **Occurrence:** individualCount: 1; sex: female; **Location:** locationID: A2; continent: Europe; country: Spain; countryCode: ES; stateProvince: Catalonia; county: Lleida; locality: Sola de Boi; verbatimElevation: 1738.7; decimalLatitude: 42.54913; decimalLongitude: 0.87137; geodeticDatum: WGS84; **Event:** eventID: 1; samplingProtocol: Aerial; eventTime: Night
- **Type status:** Other material. **Occurrence:** individualCount: 2; sex: male; **Location:** locationID: A2; continent: Europe; country: Spain; countryCode: ES; stateProvince: Catalonia; county: Lleida; locality: Sola de Boi; verbatimElevation: 1738.7; decimalLatitude: 42.54913; decimalLongitude: 0.87137; geodeticDatum: WGS84; **Event:** eventID: A; samplingProtocol: Pitfall
- **Type status:** Other material. **Occurrence:** individualCount: 2; sex: female; **Location:** locationID: A2; continent: Europe; country: Spain; countryCode: ES; stateProvince: Catalonia; county: Lleida; locality: Sola de Boi; verbatimElevation: 1738.7; decimalLatitude: 42.54913; decimalLongitude: 0.87137; geodeticDatum: WGS84; **Event:** eventID: A; samplingProtocol: Pitfall
- **Type status:** Other material. **Occurrence:** individualCount: 1; sex: female; **Location:** locationID: A2; continent: Europe; country: Spain; countryCode: ES; stateProvince: Catalonia; county: Lleida; locality: Sola de Boi; verbatimElevation: 1738.7; decimalLatitude: 42.54913; decimalLongitude: 0.87137; geodeticDatum: WGS84; **Event:** eventID: B; samplingProtocol: Pitfall
- **Type status:** Other material. **Occurrence:** individualCount: 3; sex: male; **Location:** locationID: A2; continent: Europe; country: Spain; countryCode: ES; stateProvince: Catalonia; county: Lleida; locality: Sola de Boi; verbatimElevation: 1738.7; decimalLatitude: 42.54913; decimalLongitude: 0.87137; geodeticDatum: WGS84; **Event:** eventID: C; samplingProtocol: Pitfall
- **Type status:** Other material. **Occurrence:** individualCount: 1; sex: female; **Location:** locationID: A2; continent: Europe; country: Spain; countryCode: ES; stateProvince: Catalonia; county: Lleida; locality: Sola de Boi; verbatimElevation: 1738.7; decimalLatitude: 42.54913; decimalLongitude: 0.87137; geodeticDatum: WGS84; **Event:** eventID: D; samplingProtocol: Pitfall
- **Type status:** Other material. **Occurrence:** individualCount: 3; sex: male; **Location:** locationID: A2; continent: Europe; country: Spain; countryCode: ES; stateProvince: Catalonia; county: Lleida; locality: Sola de Boi; verbatimElevation: 1738.7; decimalLatitude: 42.54913; decimalLongitude: 0.87137; geodeticDatum: WGS84; **Event:** eventID: E; samplingProtocol: Pitfall
- **Type status:** Other material. **Occurrence:** individualCount: 1; sex: female; **Location:** locationID: A2; continent: Europe; country: Spain; countryCode: ES; stateProvince: Catalonia; county: Lleida; locality: Sola de Boi; verbatimElevation: 1738.7; decimalLatitude: 42.54913; decimalLongitude: 0.87137; geodeticDatum: WGS84; **Event:** eventID: E; samplingProtocol: Pitfall
- **Type status:** Other material. **Occurrence:** individualCount: 1; sex: female; **Location:** locationID: A2; continent: Europe; country: Spain; countryCode: ES; stateProvince: Catalonia; county: Lleida; locality: Sola de Boi; verbatimElevation: 1738.7; decimalLatitude: 42.54913; decimalLongitude: 0.87137; geodeticDatum: WGS84; **Event:** eventID: F; samplingProtocol: Pitfall
- **Type status:** Other material. **Occurrence:** individualCount: 6; sex: male; **Location:** locationID: A2; continent: Europe; country: Spain; countryCode: ES; stateProvince: Catalonia; county: Lleida; locality: Sola de Boi; verbatimElevation: 1738.7; decimalLatitude: 42.54913; decimalLongitude: 0.87137; geodeticDatum: WGS84; **Event:** eventID: H; samplingProtocol: Pitfall
- **Type status:** Other material. **Occurrence:** individualCount: 2; sex: female; **Location:** locationID: A2; continent: Europe; country: Spain; countryCode: ES; stateProvince: Catalonia; county: Lleida; locality: Sola de Boi; verbatimElevation: 1738.7; decimalLatitude: 42.54913; decimalLongitude: 0.87137; geodeticDatum: WGS84; **Event:** eventID: H; samplingProtocol: Pitfall
- **Type status:** Other material. **Occurrence:** individualCount: 1; sex: male; **Location:** locationID: A2; continent: Europe; country: Spain; countryCode: ES; stateProvince: Catalonia; county: Lleida; locality: Sola de Boi; verbatimElevation: 1738.7; decimalLatitude: 42.54913; decimalLongitude: 0.87137; geodeticDatum: WGS84; **Event:** eventID: K; samplingProtocol: Pitfall
- **Type status:** Other material. **Occurrence:** individualCount: 2; sex: male; **Location:** locationID: A2; continent: Europe; country: Spain; countryCode: ES; stateProvince: Catalonia; county: Lleida; locality: Sola de Boi; verbatimElevation: 1738.7; decimalLatitude: 42.54913; decimalLongitude: 0.87137; geodeticDatum: WGS84; **Event:** eventID: L; samplingProtocol: Pitfall
- **Type status:** Other material. **Occurrence:** individualCount: 1; sex: female; **Location:** locationID: A2; continent: Europe; country: Spain; countryCode: ES; stateProvince: Catalonia; county: Lleida; locality: Sola de Boi; verbatimElevation: 1738.7; decimalLatitude: 42.54913; decimalLongitude: 0.87137; geodeticDatum: WGS84; **Event:** eventID: 1; samplingProtocol: Sweeping; eventTime: Day

##### Distribution

Palearctic

#### Mimetidae

Simon, 1881

#### Ero aphana

(Walckenaer, 1802)

##### Materials

- **Type status:** Other material. **Occurrence:** individualCount: 2; sex: female; **Location:** locationID: C1; continent: Europe; country: Spain; countryCode: ES; stateProvince: Castilla-La Mancha; county: Ciudad Real; locality: Valle Brezoso; verbatimElevation: 756.56; decimalLatitude: 39.35663; decimalLongitude: -4.35912; geodeticDatum: WGS84; **Event:** eventID: 3; samplingProtocol: Aerial; eventTime: Night
- **Type status:** Other material. **Occurrence:** individualCount: 1; sex: male; **Location:** locationID: C1; continent: Europe; country: Spain; countryCode: ES; stateProvince: Castilla-La Mancha; county: Ciudad Real; locality: Valle Brezoso; verbatimElevation: 756.56; decimalLatitude: 39.35663; decimalLongitude: -4.35912; geodeticDatum: WGS84; **Event:** eventID: 1; samplingProtocol: Beating; eventTime: Day
- **Type status:** Other material. **Occurrence:** individualCount: 2; sex: male; **Location:** locationID: C1; continent: Europe; country: Spain; countryCode: ES; stateProvince: Castilla-La Mancha; county: Ciudad Real; locality: Valle Brezoso; verbatimElevation: 756.56; decimalLatitude: 39.35663; decimalLongitude: -4.35912; geodeticDatum: WGS84; **Event:** eventID: 2; samplingProtocol: Beating; eventTime: Day
- **Type status:** Other material. **Occurrence:** individualCount: 3; sex: female; **Location:** locationID: C1; continent: Europe; country: Spain; countryCode: ES; stateProvince: Castilla-La Mancha; county: Ciudad Real; locality: Valle Brezoso; verbatimElevation: 756.56; decimalLatitude: 39.35663; decimalLongitude: -4.35912; geodeticDatum: WGS84; **Event:** eventID: 2; samplingProtocol: Beating; eventTime: Day
- **Type status:** Other material. **Occurrence:** individualCount: 1; sex: female; **Location:** locationID: C1; continent: Europe; country: Spain; countryCode: ES; stateProvince: Castilla-La Mancha; county: Ciudad Real; locality: Valle Brezoso; verbatimElevation: 756.56; decimalLatitude: 39.35663; decimalLongitude: -4.35912; geodeticDatum: WGS84; **Event:** eventID: 2; samplingProtocol: Beating; eventTime: Night
- **Type status:** Other material. **Occurrence:** individualCount: 1; sex: female; **Location:** locationID: C1; continent: Europe; country: Spain; countryCode: ES; stateProvince: Castilla-La Mancha; county: Ciudad Real; locality: Valle Brezoso; verbatimElevation: 756.56; decimalLatitude: 39.35663; decimalLongitude: -4.35912; geodeticDatum: WGS84; **Event:** eventID: 2; samplingProtocol: Sweeping; eventTime: Day
- **Type status:** Other material. **Occurrence:** individualCount: 1; sex: female; **Location:** locationID: C1; continent: Europe; country: Spain; countryCode: ES; stateProvince: Castilla-La Mancha; county: Ciudad Real; locality: Valle Brezoso; verbatimElevation: 756.56; decimalLatitude: 39.35663; decimalLongitude: -4.35912; geodeticDatum: WGS84; **Event:** eventID: 1; samplingProtocol: Sweeping; eventTime: Night
- **Type status:** Other material. **Occurrence:** individualCount: 1; sex: female; **Location:** locationID: C1; continent: Europe; country: Spain; countryCode: ES; stateProvince: Castilla-La Mancha; county: Ciudad Real; locality: Valle Brezoso; verbatimElevation: 756.56; decimalLatitude: 39.35663; decimalLongitude: -4.35912; geodeticDatum: WGS84; **Event:** eventID: 2; samplingProtocol: Sweeping; eventTime: Night
- **Type status:** Other material. **Occurrence:** individualCount: 1; sex: male; **Location:** locationID: C1; continent: Europe; country: Spain; countryCode: ES; stateProvince: Castilla-La Mancha; county: Ciudad Real; locality: Valle Brezoso; verbatimElevation: 756.56; decimalLatitude: 39.35663; decimalLongitude: -4.35912; geodeticDatum: WGS84; **Event:** eventID: 2; samplingProtocol: Sweeping; eventTime: Night
- **Type status:** Other material. **Occurrence:** individualCount: 1; sex: male; **Location:** locationID: C2; continent: Europe; country: Spain; countryCode: ES; stateProvince: Castilla-La Mancha; county: Ciudad Real; locality: Valle Brezoso; verbatimElevation: 739.31; decimalLatitude: 39.35159; decimalLongitude: -4.3589; geodeticDatum: WGS84; **Event:** eventID: 2; samplingProtocol: Aerial; eventTime: Night
- **Type status:** Other material. **Occurrence:** individualCount: 1; sex: female; **Location:** locationID: C4; continent: Europe; country: Spain; countryCode: ES; stateProvince: Castilla-La Mancha; county: Ciudad Real; locality: La Quesera; verbatimElevation: 772.3; decimalLatitude: 39.36337; decimalLongitude: -4.41704; geodeticDatum: WGS84; **Event:** eventID: 1; samplingProtocol: Beating; eventTime: Day
- **Type status:** Other material. **Occurrence:** individualCount: 1; sex: female; **Location:** locationID: C4; continent: Europe; country: Spain; countryCode: ES; stateProvince: Castilla-La Mancha; county: Ciudad Real; locality: La Quesera; verbatimElevation: 772.3; decimalLatitude: 39.36337; decimalLongitude: -4.41704; geodeticDatum: WGS84; **Event:** eventID: 1; samplingProtocol: Sweeping; eventTime: Day
- **Type status:** Other material. **Occurrence:** individualCount: 1; sex: male; **Location:** locationID: O2; continent: Europe; country: Spain; countryCode: ES; stateProvince: Aragón; county: Huesca; locality: Rebilla; verbatimElevation: 1158.13; decimalLatitude: 42.59427; decimalLongitude: 0.1529; geodeticDatum: WGS84; **Event:** eventID: 1; samplingProtocol: Sweeping; eventTime: Night
- **Type status:** Other material. **Occurrence:** individualCount: 1; sex: male; **Location:** locationID: P2; continent: Europe; country: Spain; countryCode: ES; stateProvince: Castilla y León; county: León; locality: Joyoguelas; verbatimElevation: 763.98; decimalLatitude: 43.17771; decimalLongitude: -4.90579; geodeticDatum: WGS84; **Event:** eventID: 1; samplingProtocol: Aerial; eventTime: Night
- **Type status:** Other material. **Occurrence:** individualCount: 1; sex: male; **Location:** locationID: P2; continent: Europe; country: Spain; countryCode: ES; stateProvince: Castilla y León; county: León; locality: Joyoguelas; verbatimElevation: 763.98; decimalLatitude: 43.17771; decimalLongitude: -4.90579; geodeticDatum: WGS84; **Event:** eventID: 2; samplingProtocol: Aerial; eventTime: Night
- **Type status:** Other material. **Occurrence:** individualCount: 1; sex: female; **Location:** locationID: P2; continent: Europe; country: Spain; countryCode: ES; stateProvince: Castilla y León; county: León; locality: Joyoguelas; verbatimElevation: 763.98; decimalLatitude: 43.17771; decimalLongitude: -4.90579; geodeticDatum: WGS84; **Event:** eventID: 1; samplingProtocol: Sweeping; eventTime: Night
- **Type status:** Other material. **Occurrence:** individualCount: 1; sex: male; **Location:** locationID: P4; continent: Europe; country: Spain; countryCode: ES; stateProvince: Castilla y León; county: León; locality: El Canto; verbatimElevation: 943.48; decimalLatitude: 43.17227; decimalLongitude: -4.90857; geodeticDatum: WGS84; **Event:** eventID: 1; samplingProtocol: Aerial; eventTime: Night
- **Type status:** Other material. **Occurrence:** individualCount: 1; sex: male; **Location:** locationID: P4; continent: Europe; country: Spain; countryCode: ES; stateProvince: Castilla y León; county: León; locality: El Canto; verbatimElevation: 943.48; decimalLatitude: 43.17227; decimalLongitude: -4.90857; geodeticDatum: WGS84; **Event:** eventID: 2; samplingProtocol: Aerial; eventTime: Night
- **Type status:** Other material. **Occurrence:** individualCount: 2; sex: male; **Location:** locationID: P4; continent: Europe; country: Spain; countryCode: ES; stateProvince: Castilla y León; county: León; locality: El Canto; verbatimElevation: 943.48; decimalLatitude: 43.17227; decimalLongitude: -4.90857; geodeticDatum: WGS84; **Event:** eventID: 2; samplingProtocol: Aerial; eventTime: Night
- **Type status:** Other material. **Occurrence:** individualCount: 1; sex: female; **Location:** locationID: P4; continent: Europe; country: Spain; countryCode: ES; stateProvince: Castilla y León; county: León; locality: El Canto; verbatimElevation: 943.48; decimalLatitude: 43.17227; decimalLongitude: -4.90857; geodeticDatum: WGS84; **Event:** eventID: 1; samplingProtocol: Beating; eventTime: Night

##### Distribution

Palearctic (elsewhere, introduced)

#### Ero tuberculata

(De Geer, 1778)

##### Materials

- **Type status:** Other material. **Occurrence:** individualCount: 1; sex: female; **Location:** locationID: C1; continent: Europe; country: Spain; countryCode: ES; stateProvince: Castilla-La Mancha; county: Ciudad Real; locality: Valle Brezoso; verbatimElevation: 756.56; decimalLatitude: 39.35663; decimalLongitude: -4.35912; geodeticDatum: WGS84; **Event:** eventID: 2; samplingProtocol: Beating; eventTime: Night
- **Type status:** Other material. **Occurrence:** individualCount: 1; sex: female; **Location:** locationID: C1; continent: Europe; country: Spain; countryCode: ES; stateProvince: Castilla-La Mancha; county: Ciudad Real; locality: Valle Brezoso; verbatimElevation: 756.56; decimalLatitude: 39.35663; decimalLongitude: -4.35912; geodeticDatum: WGS84; **Event:** eventID: 2; samplingProtocol: Sweeping; eventTime: Day

##### Distribution

Palearctic

#### Mimetus laevigatus

(Keyserling, 1863)

##### Materials

- **Type status:** Other material. **Occurrence:** individualCount: 1; sex: male; **Location:** locationID: C1; continent: Europe; country: Spain; countryCode: ES; stateProvince: Castilla-La Mancha; county: Ciudad Real; locality: Valle Brezoso; verbatimElevation: 756.56; decimalLatitude: 39.35663; decimalLongitude: -4.35912; geodeticDatum: WGS84; **Event:** eventID: 2; samplingProtocol: Beating; eventTime: Night
- **Type status:** Other material. **Occurrence:** individualCount: 1; sex: male; **Location:** locationID: C1; continent: Europe; country: Spain; countryCode: ES; stateProvince: Castilla-La Mancha; county: Ciudad Real; locality: Valle Brezoso; verbatimElevation: 756.56; decimalLatitude: 39.35663; decimalLongitude: -4.35912; geodeticDatum: WGS84; **Event:** eventID: 1; samplingProtocol: Sweeping; eventTime: Night
- **Type status:** Other material. **Occurrence:** individualCount: 1; sex: female; **Location:** locationID: C2; continent: Europe; country: Spain; countryCode: ES; stateProvince: Castilla-La Mancha; county: Ciudad Real; locality: Valle Brezoso; verbatimElevation: 739.31; decimalLatitude: 39.35159; decimalLongitude: -4.3589; geodeticDatum: WGS84; **Event:** eventID: 3; samplingProtocol: Aerial; eventTime: Night
- **Type status:** Other material. **Occurrence:** individualCount: 1; sex: female; **Location:** locationID: C2; continent: Europe; country: Spain; countryCode: ES; stateProvince: Castilla-La Mancha; county: Ciudad Real; locality: Valle Brezoso; verbatimElevation: 739.31; decimalLatitude: 39.35159; decimalLongitude: -4.3589; geodeticDatum: WGS84; **Event:** eventID: 1; samplingProtocol: Beating; eventTime: Night
- **Type status:** Other material. **Occurrence:** individualCount: 1; sex: male; **Location:** locationID: C3; continent: Europe; country: Spain; countryCode: ES; stateProvince: Castilla-La Mancha; county: Ciudad Real; locality: La Quesera; verbatimElevation: 767.55; decimalLatitude: 39.36177; decimalLongitude: -4.41733; geodeticDatum: WGS84; **Event:** eventID: 3; samplingProtocol: Aerial; eventTime: Night
- **Type status:** Other material. **Occurrence:** individualCount: 1; sex: male; **Location:** locationID: C4; continent: Europe; country: Spain; countryCode: ES; stateProvince: Castilla-La Mancha; county: Ciudad Real; locality: La Quesera; verbatimElevation: 772.3; decimalLatitude: 39.36337; decimalLongitude: -4.41704; geodeticDatum: WGS84; **Event:** eventID: 2; samplingProtocol: Aerial; eventTime: Night
- **Type status:** Other material. **Occurrence:** individualCount: 1; sex: female; **Location:** locationID: C4; continent: Europe; country: Spain; countryCode: ES; stateProvince: Castilla-La Mancha; county: Ciudad Real; locality: La Quesera; verbatimElevation: 772.3; decimalLatitude: 39.36337; decimalLongitude: -4.41704; geodeticDatum: WGS84; **Event:** eventID: 1; samplingProtocol: Beating; eventTime: Day
- **Type status:** Other material. **Occurrence:** individualCount: 1; sex: male; **Location:** locationID: M1; continent: Europe; country: Spain; countryCode: ES; stateProvince: Extremadura; county: Cáceres; locality: Peña Falcón; verbatimElevation: 320.6; decimalLatitude: 39.83296; decimalLongitude: -6.0641; geodeticDatum: WGS84; **Event:** eventID: 2; samplingProtocol: Beating; eventTime: Night
- **Type status:** Other material. **Occurrence:** individualCount: 2; sex: male; **Location:** locationID: M2; continent: Europe; country: Spain; countryCode: ES; stateProvince: Extremadura; county: Cáceres; locality: Fuente del Frances; verbatimElevation: 320.72; decimalLatitude: 39.828; decimalLongitude: -6.03249; geodeticDatum: WGS84; **Event:** eventID: 2; samplingProtocol: Aerial; eventTime: Night
- **Type status:** Other material. **Occurrence:** individualCount: 2; sex: female; **Location:** locationID: M2; continent: Europe; country: Spain; countryCode: ES; stateProvince: Extremadura; county: Cáceres; locality: Fuente del Frances; verbatimElevation: 320.72; decimalLatitude: 39.828; decimalLongitude: -6.03249; geodeticDatum: WGS84; **Event:** eventID: 2; samplingProtocol: Aerial; eventTime: Night
- **Type status:** Other material. **Occurrence:** individualCount: 1; sex: male; **Location:** locationID: M2; continent: Europe; country: Spain; countryCode: ES; stateProvince: Extremadura; county: Cáceres; locality: Fuente del Frances; verbatimElevation: 320.72; decimalLatitude: 39.828; decimalLongitude: -6.03249; geodeticDatum: WGS84; **Event:** eventID: 3; samplingProtocol: Aerial; eventTime: Night
- **Type status:** Other material. **Occurrence:** individualCount: 1; sex: male; **Location:** locationID: M2; continent: Europe; country: Spain; countryCode: ES; stateProvince: Extremadura; county: Cáceres; locality: Fuente del Frances; verbatimElevation: 320.72; decimalLatitude: 39.828; decimalLongitude: -6.03249; geodeticDatum: WGS84; **Event:** eventID: 4; samplingProtocol: Aerial; eventTime: Night
- **Type status:** Other material. **Occurrence:** individualCount: 1; sex: female; **Location:** locationID: M2; continent: Europe; country: Spain; countryCode: ES; stateProvince: Extremadura; county: Cáceres; locality: Fuente del Frances; verbatimElevation: 320.72; decimalLatitude: 39.828; decimalLongitude: -6.03249; geodeticDatum: WGS84; **Event:** eventID: 4; samplingProtocol: Aerial; eventTime: Night
- **Type status:** Other material. **Occurrence:** individualCount: 1; sex: male; **Location:** locationID: M2; continent: Europe; country: Spain; countryCode: ES; stateProvince: Extremadura; county: Cáceres; locality: Fuente del Frances; verbatimElevation: 320.72; decimalLatitude: 39.828; decimalLongitude: -6.03249; geodeticDatum: WGS84; **Event:** eventID: 1; samplingProtocol: Beating; eventTime: Night
- **Type status:** Other material. **Occurrence:** individualCount: 3; sex: female; **Location:** locationID: M2; continent: Europe; country: Spain; countryCode: ES; stateProvince: Extremadura; county: Cáceres; locality: Fuente del Frances; verbatimElevation: 320.72; decimalLatitude: 39.828; decimalLongitude: -6.03249; geodeticDatum: WGS84; **Event:** eventID: 1; samplingProtocol: Beating; eventTime: Night
- **Type status:** Other material. **Occurrence:** individualCount: 1; sex: female; **Location:** locationID: M2; continent: Europe; country: Spain; countryCode: ES; stateProvince: Extremadura; county: Cáceres; locality: Fuente del Frances; verbatimElevation: 320.72; decimalLatitude: 39.828; decimalLongitude: -6.03249; geodeticDatum: WGS84; **Event:** eventID: 1; samplingProtocol: Sweeping; eventTime: Night
- **Type status:** Other material. **Occurrence:** individualCount: 1; sex: male; **Location:** locationID: M2; continent: Europe; country: Spain; countryCode: ES; stateProvince: Extremadura; county: Cáceres; locality: Fuente del Frances; verbatimElevation: 320.72; decimalLatitude: 39.828; decimalLongitude: -6.03249; geodeticDatum: WGS84; **Event:** eventID: 2; samplingProtocol: Sweeping; eventTime: Night
- **Type status:** Other material. **Occurrence:** individualCount: 2; sex: female; **Location:** locationID: M2; continent: Europe; country: Spain; countryCode: ES; stateProvince: Extremadura; county: Cáceres; locality: Fuente del Frances; verbatimElevation: 320.72; decimalLatitude: 39.828; decimalLongitude: -6.03249; geodeticDatum: WGS84; **Event:** eventID: 2; samplingProtocol: Sweeping; eventTime: Night

##### Distribution

Mediterranean to Central Asia

#### Miturgidae

Simon, 1886

#### Zora manicata

Simon, 1878

##### Materials

- **Type status:** Other material. **Occurrence:** individualCount: 1; sex: male; **Location:** locationID: A1; continent: Europe; country: Spain; countryCode: ES; stateProvince: Catalonia; county: Lleida; locality: Sola de Boi; verbatimElevation: 1759.8; decimalLatitude: 42.54958; decimalLongitude: 0.87254; geodeticDatum: WGS84; **Event:** eventID: B; samplingProtocol: Pitfall
- **Type status:** Other material. **Occurrence:** individualCount: 2; sex: male; **Location:** locationID: A1; continent: Europe; country: Spain; countryCode: ES; stateProvince: Catalonia; county: Lleida; locality: Sola de Boi; verbatimElevation: 1759.8; decimalLatitude: 42.54958; decimalLongitude: 0.87254; geodeticDatum: WGS84; **Event:** eventID: E; samplingProtocol: Pitfall
- **Type status:** Other material. **Occurrence:** individualCount: 3; sex: male; **Location:** locationID: A1; continent: Europe; country: Spain; countryCode: ES; stateProvince: Catalonia; county: Lleida; locality: Sola de Boi; verbatimElevation: 1759.8; decimalLatitude: 42.54958; decimalLongitude: 0.87254; geodeticDatum: WGS84; **Event:** eventID: F; samplingProtocol: Pitfall
- **Type status:** Other material. **Occurrence:** individualCount: 2; sex: male; **Location:** locationID: A1; continent: Europe; country: Spain; countryCode: ES; stateProvince: Catalonia; county: Lleida; locality: Sola de Boi; verbatimElevation: 1759.8; decimalLatitude: 42.54958; decimalLongitude: 0.87254; geodeticDatum: WGS84; **Event:** eventID: H; samplingProtocol: Pitfall
- **Type status:** Other material. **Occurrence:** individualCount: 1; sex: male; **Location:** locationID: A1; continent: Europe; country: Spain; countryCode: ES; stateProvince: Catalonia; county: Lleida; locality: Sola de Boi; verbatimElevation: 1759.8; decimalLatitude: 42.54958; decimalLongitude: 0.87254; geodeticDatum: WGS84; **Event:** eventID: K; samplingProtocol: Pitfall
- **Type status:** Other material. **Occurrence:** individualCount: 2; sex: male; **Location:** locationID: A2; continent: Europe; country: Spain; countryCode: ES; stateProvince: Catalonia; county: Lleida; locality: Sola de Boi; verbatimElevation: 1738.7; decimalLatitude: 42.54913; decimalLongitude: 0.87137; geodeticDatum: WGS84; **Event:** eventID: B; samplingProtocol: Pitfall
- **Type status:** Other material. **Occurrence:** individualCount: 1; sex: male; **Location:** locationID: A2; continent: Europe; country: Spain; countryCode: ES; stateProvince: Catalonia; county: Lleida; locality: Sola de Boi; verbatimElevation: 1738.7; decimalLatitude: 42.54913; decimalLongitude: 0.87137; geodeticDatum: WGS84; **Event:** eventID: H; samplingProtocol: Pitfall
- **Type status:** Other material. **Occurrence:** individualCount: 1; sex: male; **Location:** locationID: A2; continent: Europe; country: Spain; countryCode: ES; stateProvince: Catalonia; county: Lleida; locality: Sola de Boi; verbatimElevation: 1738.7; decimalLatitude: 42.54913; decimalLongitude: 0.87137; geodeticDatum: WGS84; **Event:** eventID: I; samplingProtocol: Pitfall
- **Type status:** Other material. **Occurrence:** individualCount: 1; sex: male; **Location:** locationID: A2; continent: Europe; country: Spain; countryCode: ES; stateProvince: Catalonia; county: Lleida; locality: Sola de Boi; verbatimElevation: 1738.7; decimalLatitude: 42.54913; decimalLongitude: 0.87137; geodeticDatum: WGS84; **Event:** eventID: J; samplingProtocol: Pitfall
- **Type status:** Other material. **Occurrence:** individualCount: 1; sex: male; **Location:** locationID: A2; continent: Europe; country: Spain; countryCode: ES; stateProvince: Catalonia; county: Lleida; locality: Sola de Boi; verbatimElevation: 1738.7; decimalLatitude: 42.54913; decimalLongitude: 0.87137; geodeticDatum: WGS84; **Event:** eventID: L; samplingProtocol: Pitfall
- **Type status:** Other material. **Occurrence:** individualCount: 2; sex: female; **Location:** locationID: C4; continent: Europe; country: Spain; countryCode: ES; stateProvince: Castilla-La Mancha; county: Ciudad Real; locality: La Quesera; verbatimElevation: 772.3; decimalLatitude: 39.36337; decimalLongitude: -4.41704; geodeticDatum: WGS84; **Event:** eventID: C; samplingProtocol: Pitfall
- **Type status:** Other material. **Occurrence:** individualCount: 1; sex: male; **Location:** locationID: C4; continent: Europe; country: Spain; countryCode: ES; stateProvince: Castilla-La Mancha; county: Ciudad Real; locality: La Quesera; verbatimElevation: 772.3; decimalLatitude: 39.36337; decimalLongitude: -4.41704; geodeticDatum: WGS84; **Event:** eventID: D; samplingProtocol: Pitfall
- **Type status:** Other material. **Occurrence:** individualCount: 1; sex: male; **Location:** locationID: C4; continent: Europe; country: Spain; countryCode: ES; stateProvince: Castilla-La Mancha; county: Ciudad Real; locality: La Quesera; verbatimElevation: 772.3; decimalLatitude: 39.36337; decimalLongitude: -4.41704; geodeticDatum: WGS84; **Event:** eventID: E; samplingProtocol: Pitfall
- **Type status:** Other material. **Occurrence:** individualCount: 1; sex: female; **Location:** locationID: C4; continent: Europe; country: Spain; countryCode: ES; stateProvince: Castilla-La Mancha; county: Ciudad Real; locality: La Quesera; verbatimElevation: 772.3; decimalLatitude: 39.36337; decimalLongitude: -4.41704; geodeticDatum: WGS84; **Event:** eventID: G; samplingProtocol: Pitfall
- **Type status:** Other material. **Occurrence:** individualCount: 2; sex: male; **Location:** locationID: C4; continent: Europe; country: Spain; countryCode: ES; stateProvince: Castilla-La Mancha; county: Ciudad Real; locality: La Quesera; verbatimElevation: 772.3; decimalLatitude: 39.36337; decimalLongitude: -4.41704; geodeticDatum: WGS84; **Event:** eventID: G; samplingProtocol: Pitfall
- **Type status:** Other material. **Occurrence:** individualCount: 2; sex: male; **Location:** locationID: C4; continent: Europe; country: Spain; countryCode: ES; stateProvince: Castilla-La Mancha; county: Ciudad Real; locality: La Quesera; verbatimElevation: 772.3; decimalLatitude: 39.36337; decimalLongitude: -4.41704; geodeticDatum: WGS84; **Event:** eventID: H; samplingProtocol: Pitfall
- **Type status:** Other material. **Occurrence:** individualCount: 1; sex: male; **Location:** locationID: C4; continent: Europe; country: Spain; countryCode: ES; stateProvince: Castilla-La Mancha; county: Ciudad Real; locality: La Quesera; verbatimElevation: 772.3; decimalLatitude: 39.36337; decimalLongitude: -4.41704; geodeticDatum: WGS84; **Event:** eventID: I; samplingProtocol: Pitfall
- **Type status:** Other material. **Occurrence:** individualCount: 1; sex: male; **Location:** locationID: C4; continent: Europe; country: Spain; countryCode: ES; stateProvince: Castilla-La Mancha; county: Ciudad Real; locality: La Quesera; verbatimElevation: 772.3; decimalLatitude: 39.36337; decimalLongitude: -4.41704; geodeticDatum: WGS84; **Event:** eventID: K; samplingProtocol: Pitfall
- **Type status:** Other material. **Occurrence:** individualCount: 1; sex: male; **Location:** locationID: O1; continent: Europe; country: Spain; countryCode: ES; stateProvince: Aragón; county: Huesca; locality: O Furno; verbatimElevation: 1396.73; decimalLatitude: 42.60677; decimalLongitude: 0.13135; geodeticDatum: WGS84; **Event:** eventID: C; samplingProtocol: Pitfall
- **Type status:** Other material. **Occurrence:** individualCount: 1; sex: male; **Location:** locationID: O1; continent: Europe; country: Spain; countryCode: ES; stateProvince: Aragón; county: Huesca; locality: O Furno; verbatimElevation: 1396.73; decimalLatitude: 42.60677; decimalLongitude: 0.13135; geodeticDatum: WGS84; **Event:** eventID: G; samplingProtocol: Pitfall
- **Type status:** Other material. **Occurrence:** individualCount: 1; sex: male; **Location:** locationID: O2; continent: Europe; country: Spain; countryCode: ES; stateProvince: Aragón; county: Huesca; locality: Rebilla; verbatimElevation: 1158.13; decimalLatitude: 42.59427; decimalLongitude: 0.1529; geodeticDatum: WGS84; **Event:** eventID: A; samplingProtocol: Pitfall
- **Type status:** Other material. **Occurrence:** individualCount: 1; sex: male; **Location:** locationID: O2; continent: Europe; country: Spain; countryCode: ES; stateProvince: Aragón; county: Huesca; locality: Rebilla; verbatimElevation: 1158.13; decimalLatitude: 42.59427; decimalLongitude: 0.1529; geodeticDatum: WGS84; **Event:** eventID: C; samplingProtocol: Pitfall
- **Type status:** Other material. **Occurrence:** individualCount: 1; sex: female; **Location:** locationID: O2; continent: Europe; country: Spain; countryCode: ES; stateProvince: Aragón; county: Huesca; locality: Rebilla; verbatimElevation: 1158.13; decimalLatitude: 42.59427; decimalLongitude: 0.1529; geodeticDatum: WGS84; **Event:** eventID: K; samplingProtocol: Pitfall
- **Type status:** Other material. **Occurrence:** individualCount: 2; sex: male; **Location:** locationID: O2; continent: Europe; country: Spain; countryCode: ES; stateProvince: Aragón; county: Huesca; locality: Rebilla; verbatimElevation: 1158.13; decimalLatitude: 42.59427; decimalLongitude: 0.1529; geodeticDatum: WGS84; **Event:** eventID: L; samplingProtocol: Pitfall
- **Type status:** Other material. **Occurrence:** individualCount: 1; sex: female; **Location:** locationID: S2; continent: Europe; country: Spain; countryCode: ES; stateProvince: Andalucía; county: Granada; locality: Camarate; verbatimElevation: 1713.96; decimalLatitude: 37.18377; decimalLongitude: -3.26282; geodeticDatum: WGS84; **Event:** eventID: A; samplingProtocol: Pitfall

##### Distribution

Europe, Ukraine, Israel

#### Zora pardalis

Simon, 1878

##### Materials

- **Type status:** Other material. **Occurrence:** individualCount: 1; sex: female; **Location:** locationID: P4; continent: Europe; country: Spain; countryCode: ES; stateProvince: Castilla y León; county: León; locality: El Canto; verbatimElevation: 943.48; decimalLatitude: 43.17227; decimalLongitude: -4.90857; geodeticDatum: WGS84; **Event:** eventID: C; samplingProtocol: Pitfall
- **Type status:** Other material. **Occurrence:** individualCount: 1; sex: male; **Location:** locationID: P4; continent: Europe; country: Spain; countryCode: ES; stateProvince: Castilla y León; county: León; locality: El Canto; verbatimElevation: 943.48; decimalLatitude: 43.17227; decimalLongitude: -4.90857; geodeticDatum: WGS84; **Event:** eventID: C; samplingProtocol: Pitfall
- **Type status:** Other material. **Occurrence:** individualCount: 2; sex: male; **Location:** locationID: P4; continent: Europe; country: Spain; countryCode: ES; stateProvince: Castilla y León; county: León; locality: El Canto; verbatimElevation: 943.48; decimalLatitude: 43.17227; decimalLongitude: -4.90857; geodeticDatum: WGS84; **Event:** eventID: D; samplingProtocol: Pitfall

##### Distribution

Europe to Kazakhstan

#### Zora silvestris

Kulczynski, 1897

##### Materials

- **Type status:** Other material. **Occurrence:** individualCount: 4; sex: male; **Location:** locationID: A1; continent: Europe; country: Spain; countryCode: ES; stateProvince: Catalonia; county: Lleida; locality: Sola de Boi; verbatimElevation: 1759.8; decimalLatitude: 42.54958; decimalLongitude: 0.87254; geodeticDatum: WGS84; **Event:** eventID: 2; samplingProtocol: Ground; eventTime: Night
- **Type status:** Other material. **Occurrence:** individualCount: 1; sex: male; **Location:** locationID: A1; continent: Europe; country: Spain; countryCode: ES; stateProvince: Catalonia; county: Lleida; locality: Sola de Boi; verbatimElevation: 1759.8; decimalLatitude: 42.54958; decimalLongitude: 0.87254; geodeticDatum: WGS84; **Event:** eventID: D; samplingProtocol: Pitfall
- **Type status:** Other material. **Occurrence:** individualCount: 1; sex: female; **Location:** locationID: A1; continent: Europe; country: Spain; countryCode: ES; stateProvince: Catalonia; county: Lleida; locality: Sola de Boi; verbatimElevation: 1759.8; decimalLatitude: 42.54958; decimalLongitude: 0.87254; geodeticDatum: WGS84; **Event:** eventID: E; samplingProtocol: Pitfall
- **Type status:** Other material. **Occurrence:** individualCount: 1; sex: female; **Location:** locationID: A1; continent: Europe; country: Spain; countryCode: ES; stateProvince: Catalonia; county: Lleida; locality: Sola de Boi; verbatimElevation: 1759.8; decimalLatitude: 42.54958; decimalLongitude: 0.87254; geodeticDatum: WGS84; **Event:** eventID: I; samplingProtocol: Pitfall
- **Type status:** Other material. **Occurrence:** individualCount: 1; sex: male; **Location:** locationID: A2; continent: Europe; country: Spain; countryCode: ES; stateProvince: Catalonia; county: Lleida; locality: Sola de Boi; verbatimElevation: 1738.7; decimalLatitude: 42.54913; decimalLongitude: 0.87137; geodeticDatum: WGS84; **Event:** eventID: 2; samplingProtocol: Aerial; eventTime: Night
- **Type status:** Other material. **Occurrence:** individualCount: 1; sex: male; **Location:** locationID: A2; continent: Europe; country: Spain; countryCode: ES; stateProvince: Catalonia; county: Lleida; locality: Sola de Boi; verbatimElevation: 1738.7; decimalLatitude: 42.54913; decimalLongitude: 0.87137; geodeticDatum: WGS84; **Event:** eventID: 1; samplingProtocol: Sweeping; eventTime: Night

##### Distribution

Europe to Central Asia

##### Notes

**Figure 8.**
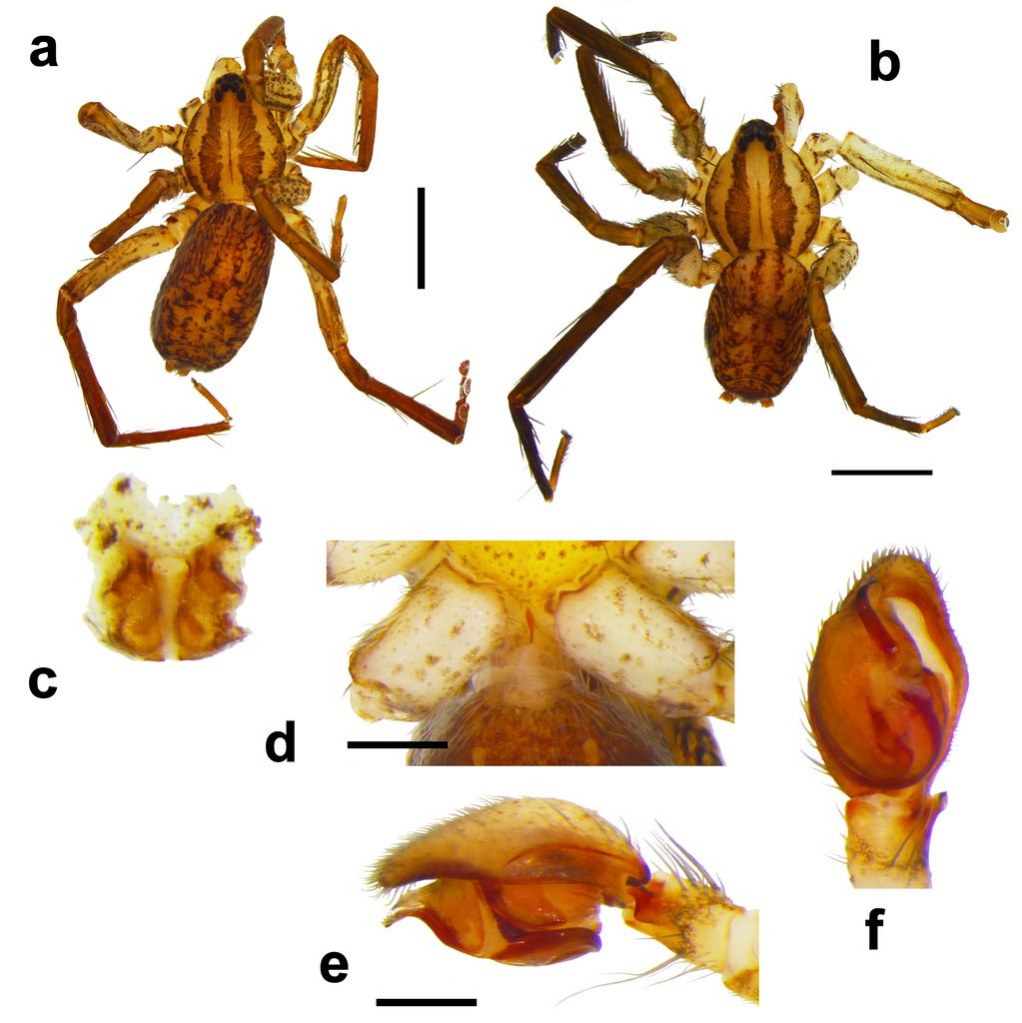
*Zora silvestris* Kulczynski, 1897. a: female, dorsal habitus, scale = 2 mm; b: male, dorsal habitus, scale = 1 mm; c: female epigynum, ventral, scale = 0.2 mm; d: male coxae IV (no special setae), ventral, scale = 0.4 mm; e: left male palp, retrolateral, scale = 0.2 mm; f: left male palp, ventral, scale = 0.2 mm.

This is a new record for Spain. See Fig. 8. The occurrence of this species was presumed dubious in a previous revisionary study of the genus (Urones 2005). We now confirm its presence in the Pyrenean region.

#### Zora spinimana

(Sundevall, 1833)

##### Materials

- **Type status:** Other material. **Occurrence:** individualCount: 1; sex: female; **Location:** locationID: A2; continent: Europe; country: Spain; countryCode: ES; stateProvince: Catalonia; county: Lleida; locality: Sola de Boi; verbatimElevation: 1738.7; decimalLatitude: 42.54913; decimalLongitude: 0.87137; geodeticDatum: WGS84; **Event:** eventID: 1; samplingProtocol: Aerial; eventTime: Night
- **Type status:** Other material. **Occurrence:** individualCount: 1; sex: female; **Location:** locationID: O1; continent: Europe; country: Spain; countryCode: ES; stateProvince: Aragón; county: Huesca; locality: O Furno; verbatimElevation: 1396.73; decimalLatitude: 42.60677; decimalLongitude: 0.13135; geodeticDatum: WGS84; **Event:** eventID: A; samplingProtocol: Pitfall
- **Type status:** Other material. **Occurrence:** individualCount: 1; sex: female; **Location:** locationID: O1; continent: Europe; country: Spain; countryCode: ES; stateProvince: Aragón; county: Huesca; locality: O Furno; verbatimElevation: 1396.73; decimalLatitude: 42.60677; decimalLongitude: 0.13135; geodeticDatum: WGS84; **Event:** eventID: D; samplingProtocol: Pitfall
- **Type status:** Other material. **Occurrence:** individualCount: 1; sex: male; **Location:** locationID: O1; continent: Europe; country: Spain; countryCode: ES; stateProvince: Aragón; county: Huesca; locality: O Furno; verbatimElevation: 1396.73; decimalLatitude: 42.60677; decimalLongitude: 0.13135; geodeticDatum: WGS84; **Event:** eventID: G; samplingProtocol: Pitfall
- **Type status:** Other material. **Occurrence:** individualCount: 1; sex: male; **Location:** locationID: O1; continent: Europe; country: Spain; countryCode: ES; stateProvince: Aragón; county: Huesca; locality: O Furno; verbatimElevation: 1396.73; decimalLatitude: 42.60677; decimalLongitude: 0.13135; geodeticDatum: WGS84; **Event:** eventID: K; samplingProtocol: Pitfall
- **Type status:** Other material. **Occurrence:** individualCount: 2; sex: male; **Location:** locationID: P2; continent: Europe; country: Spain; countryCode: ES; stateProvince: Castilla y León; county: León; locality: Joyoguelas; verbatimElevation: 763.98; decimalLatitude: 43.17771; decimalLongitude: -4.90579; geodeticDatum: WGS84; **Event:** eventID: K; samplingProtocol: Pitfall
- **Type status:** Other material. **Occurrence:** individualCount: 1; sex: female; **Location:** locationID: P3; continent: Europe; country: Spain; countryCode: ES; stateProvince: Castilla y León; county: León; locality: Las Arroyas; verbatimElevation: 1097.1; decimalLatitude: 43.14351; decimalLongitude: -4.94878; geodeticDatum: WGS84; **Event:** eventID: D; samplingProtocol: Pitfall
- **Type status:** Other material. **Occurrence:** individualCount: 1; sex: male; **Location:** locationID: P3; continent: Europe; country: Spain; countryCode: ES; stateProvince: Castilla y León; county: León; locality: Las Arroyas; verbatimElevation: 1097.1; decimalLatitude: 43.14351; decimalLongitude: -4.94878; geodeticDatum: WGS84; **Event:** eventID: F; samplingProtocol: Pitfall
- **Type status:** Other material. **Occurrence:** individualCount: 1; sex: male; **Location:** locationID: P3; continent: Europe; country: Spain; countryCode: ES; stateProvince: Castilla y León; county: León; locality: Las Arroyas; verbatimElevation: 1097.1; decimalLatitude: 43.14351; decimalLongitude: -4.94878; geodeticDatum: WGS84; **Event:** eventID: K; samplingProtocol: Pitfall
- **Type status:** Other material. **Occurrence:** individualCount: 1; sex: female; **Location:** locationID: P4; continent: Europe; country: Spain; countryCode: ES; stateProvince: Castilla y León; county: León; locality: El Canto; verbatimElevation: 943.48; decimalLatitude: 43.17227; decimalLongitude: -4.90857; geodeticDatum: WGS84; **Event:** eventID: B; samplingProtocol: Pitfall
- **Type status:** Other material. **Occurrence:** individualCount: 1; sex: male; **Location:** locationID: P4; continent: Europe; country: Spain; countryCode: ES; stateProvince: Castilla y León; county: León; locality: El Canto; verbatimElevation: 943.48; decimalLatitude: 43.17227; decimalLongitude: -4.90857; geodeticDatum: WGS84; **Event:** eventID: C; samplingProtocol: Pitfall
- **Type status:** Other material. **Occurrence:** individualCount: 1; sex: female; **Location:** locationID: P4; continent: Europe; country: Spain; countryCode: ES; stateProvince: Castilla y León; county: León; locality: El Canto; verbatimElevation: 943.48; decimalLatitude: 43.17227; decimalLongitude: -4.90857; geodeticDatum: WGS84; **Event:** eventID: E; samplingProtocol: Pitfall
- **Type status:** Other material. **Occurrence:** individualCount: 1; sex: male; **Location:** locationID: P4; continent: Europe; country: Spain; countryCode: ES; stateProvince: Castilla y León; county: León; locality: El Canto; verbatimElevation: 943.48; decimalLatitude: 43.17227; decimalLongitude: -4.90857; geodeticDatum: WGS84; **Event:** eventID: J; samplingProtocol: Pitfall
- **Type status:** Other material. **Occurrence:** individualCount: 1; sex: male; **Location:** locationID: P4; continent: Europe; country: Spain; countryCode: ES; stateProvince: Castilla y León; county: León; locality: El Canto; verbatimElevation: 943.48; decimalLatitude: 43.17227; decimalLongitude: -4.90857; geodeticDatum: WGS84; **Event:** eventID: L; samplingProtocol: Pitfall
- **Type status:** Other material. **Occurrence:** individualCount: 1; sex: female; **Location:** locationID: P4; continent: Europe; country: Spain; countryCode: ES; stateProvince: Castilla y León; county: León; locality: El Canto; verbatimElevation: 943.48; decimalLatitude: 43.17227; decimalLongitude: -4.90857; geodeticDatum: WGS84; **Event:** eventID: L; samplingProtocol: Pitfall

##### Distribution

Palearctic

#### Mysmenidae

Petrunkevitch, 1928

#### Mysmenidae sp26

##### Materials

- **Type status:** Other material. **Occurrence:** individualCount: 1; sex: female; **Location:** locationID: S1; continent: Europe; country: Spain; countryCode: ES; stateProvince: Andalucía; county: Granada; locality: Soportujar; verbatimElevation: 1786.57; decimalLatitude: 36.96151; decimalLongitude: -3.41881; geodeticDatum: WGS84; **Event:** eventID: 1; samplingProtocol: Sweeping; eventTime: Day

##### Distribution

?

##### Notes

We were unable to identify the genus of this specimen.

#### Nemesiidae

Simon, 1889

#### Nemesia sp14

##### Materials

- **Type status:** Other material. **Occurrence:** individualCount: 1; sex: female; **Location:** locationID: O2; continent: Europe; country: Spain; countryCode: ES; stateProvince: Aragón; county: Huesca; locality: Rebilla; verbatimElevation: 1158.13; decimalLatitude: 42.59427; decimalLongitude: 0.1529; geodeticDatum: WGS84; **Event:** eventID: F; samplingProtocol: Pitfall

##### Distribution

?

##### Notes

This is a species of *Nemesia* Audouin, 1826, which we were unable to identify.

#### Oecobiidae

Blackwall, 1862

#### Oecobius machadoi

Wunderlich, 1995

##### Materials

- **Type status:** Other material. **Occurrence:** individualCount: 1; sex: male; **Location:** locationID: C1; continent: Europe; country: Spain; countryCode: ES; stateProvince: Castilla-La Mancha; county: Ciudad Real; locality: Valle Brezoso; verbatimElevation: 756.56; decimalLatitude: 39.35663; decimalLongitude: -4.35912; geodeticDatum: WGS84; **Event:** eventID: B; samplingProtocol: Pitfall

##### Distribution

Iberian Peninsula

#### Oonopidae

Simon, 1890

#### Oonops pulcher

Templeton, 1835

##### Materials

- **Type status:** Other material. **Occurrence:** individualCount: 1; sex: male; **Location:** locationID: P4; continent: Europe; country: Spain; countryCode: ES; stateProvince: Castilla y León; county: León; locality: El Canto; verbatimElevation: 943.48; decimalLatitude: 43.17227; decimalLongitude: -4.90857; geodeticDatum: WGS84; **Event:** eventID: G; samplingProtocol: Pitfall
- **Type status:** Other material. **Occurrence:** individualCount: 1; sex: female; **Location:** locationID: P3; continent: Europe; country: Spain; countryCode: ES; stateProvince: Castilla y León; county: León; locality: Las Arroyas; verbatimElevation: 1097.1; decimalLatitude: 43.14351; decimalLongitude: -4.94878; geodeticDatum: WGS84; **Event:** eventID: 1; samplingProtocol: Aerial; eventTime: Night

##### Distribution

Europe to Ukraine, probably Madeira, North Africa, Tasmania

#### Oonops sp17

##### Materials

- **Type status:** Other material. **Occurrence:** individualCount: 1; sex: male; **Location:** locationID: C1; continent: Europe; country: Spain; countryCode: ES; stateProvince: Castilla-La Mancha; county: Ciudad Real; locality: Valle Brezoso; verbatimElevation: 756.56; decimalLatitude: 39.35663; decimalLongitude: -4.35912; geodeticDatum: WGS84; **Event:** eventID: A; samplingProtocol: Pitfall
- **Type status:** Other material. **Occurrence:** individualCount: 2; sex: female; **Location:** locationID: C1; continent: Europe; country: Spain; countryCode: ES; stateProvince: Castilla-La Mancha; county: Ciudad Real; locality: Valle Brezoso; verbatimElevation: 756.56; decimalLatitude: 39.35663; decimalLongitude: -4.35912; geodeticDatum: WGS84; **Event:** eventID: A; samplingProtocol: Pitfall
- **Type status:** Other material. **Occurrence:** individualCount: 1; sex: male; **Location:** locationID: C1; continent: Europe; country: Spain; countryCode: ES; stateProvince: Castilla-La Mancha; county: Ciudad Real; locality: Valle Brezoso; verbatimElevation: 756.56; decimalLatitude: 39.35663; decimalLongitude: -4.35912; geodeticDatum: WGS84; **Event:** eventID: J; samplingProtocol: Pitfall

##### Distribution

?

##### Notes

This is a new species of *Oonops* Templeton, 1835, to be described in a future publication.

#### Oonops sp22

##### Materials

- **Type status:** Other material. **Occurrence:** individualCount: 1; sex: female; **Location:** locationID: M2; continent: Europe; country: Spain; countryCode: ES; stateProvince: Extremadura; county: Cáceres; locality: Fuente del Frances; verbatimElevation: 320.72; decimalLatitude: 39.828; decimalLongitude: -6.03249; geodeticDatum: WGS84; **Event:** eventID: I; samplingProtocol: Pitfall

##### Distribution

?

##### Notes

We suspect this single specimen might be *Oonops* sp. 17, but we were unable to use genetic data to confirm this. Given that many undescribed species of *Oonops* are known to occur in the Iberian Peninsula, we opted for assignment of the present specimen to a separate morphospecies until further specimens are collected.

#### Oonops sp27

##### Materials

- **Type status:** Other material. **Occurrence:** individualCount: 1; sex: male; **Location:** locationID: S1; continent: Europe; country: Spain; countryCode: ES; stateProvince: Andalucía; county: Granada; locality: Soportujar; verbatimElevation: 1786.57; decimalLatitude: 36.96151; decimalLongitude: -3.41881; geodeticDatum: WGS84; **Event:** eventID: L; samplingProtocol: Pitfall

##### Distribution

?

##### Notes

This is a new species of *Oonops*, to be described in a future publication.

#### Oonops sp44

##### Materials

- **Type status:** Other material. **Occurrence:** individualCount: 1; sex: female; **Location:** locationID: C3; continent: Europe; country: Spain; countryCode: ES; stateProvince: Castilla-La Mancha; county: Ciudad Real; locality: La Quesera; verbatimElevation: 767.55; decimalLatitude: 39.36177; decimalLongitude: -4.41733; geodeticDatum: WGS84; **Event:** eventID: E; samplingProtocol: Pitfall

##### Distribution

?

##### Notes

This is a new species of *Oonops*, to be described in a future publication.

#### Orchestina sp34

##### Materials

- **Type status:** Other material. **Occurrence:** individualCount: 1; sex: female; **Location:** locationID: C1; continent: Europe; country: Spain; countryCode: ES; stateProvince: Castilla-La Mancha; county: Ciudad Real; locality: Valle Brezoso; verbatimElevation: 756.56; decimalLatitude: 39.35663; decimalLongitude: -4.35912; geodeticDatum: WGS84; **Event:** eventID: 2; samplingProtocol: Sweeping; eventTime: Night
- **Type status:** Other material. **Occurrence:** individualCount: 1; sex: female; **Location:** locationID: C3; continent: Europe; country: Spain; countryCode: ES; stateProvince: Castilla-La Mancha; county: Ciudad Real; locality: La Quesera; verbatimElevation: 767.55; decimalLatitude: 39.36177; decimalLongitude: -4.41733; geodeticDatum: WGS84; **Event:** eventID: 4; samplingProtocol: Aerial; eventTime: Night
- **Type status:** Other material. **Occurrence:** individualCount: 1; sex: female; **Location:** locationID: M2; continent: Europe; country: Spain; countryCode: ES; stateProvince: Extremadura; county: Cáceres; locality: Fuente del Frances; verbatimElevation: 320.72; decimalLatitude: 39.828; decimalLongitude: -6.03249; geodeticDatum: WGS84; **Event:** eventID: 3; samplingProtocol: Aerial; eventTime: Night
- **Type status:** Other material. **Occurrence:** individualCount: 1; sex: female; **Location:** locationID: M2; continent: Europe; country: Spain; countryCode: ES; stateProvince: Extremadura; county: Cáceres; locality: Fuente del Frances; verbatimElevation: 320.72; decimalLatitude: 39.828; decimalLongitude: -6.03249; geodeticDatum: WGS84; **Event:** eventID: 4; samplingProtocol: Aerial; eventTime: Night
- **Type status:** Other material. **Occurrence:** individualCount: 1; sex: male; **Location:** locationID: M2; continent: Europe; country: Spain; countryCode: ES; stateProvince: Extremadura; county: Cáceres; locality: Fuente del Frances; verbatimElevation: 320.72; decimalLatitude: 39.828; decimalLongitude: -6.03249; geodeticDatum: WGS84; **Event:** eventID: 1; samplingProtocol: Beating; eventTime: Night
- **Type status:** Other material. **Occurrence:** individualCount: 1; sex: male; **Location:** locationID: M2; continent: Europe; country: Spain; countryCode: ES; stateProvince: Extremadura; county: Cáceres; locality: Fuente del Frances; verbatimElevation: 320.72; decimalLatitude: 39.828; decimalLongitude: -6.03249; geodeticDatum: WGS84; **Event:** eventID: 1; samplingProtocol: Beating; eventTime: Night
- **Type status:** Other material. **Occurrence:** individualCount: 2; sex: female; **Location:** locationID: M2; continent: Europe; country: Spain; countryCode: ES; stateProvince: Extremadura; county: Cáceres; locality: Fuente del Frances; verbatimElevation: 320.72; decimalLatitude: 39.828; decimalLongitude: -6.03249; geodeticDatum: WGS84; **Event:** eventID: 1; samplingProtocol: Beating; eventTime: Night
- **Type status:** Other material. **Occurrence:** individualCount: 1; sex: male; **Location:** locationID: M2; continent: Europe; country: Spain; countryCode: ES; stateProvince: Extremadura; county: Cáceres; locality: Fuente del Frances; verbatimElevation: 320.72; decimalLatitude: 39.828; decimalLongitude: -6.03249; geodeticDatum: WGS84; **Event:** eventID: 1; samplingProtocol: Sweeping; eventTime: Night

##### Distribution

?

##### Notes

This is a new species of *Orchestina* Simon, 1882, under study in an ongoing work .

#### Oxyopidae

Thorell, 1870

#### Oxyopes heterophthalmus

(Latreille, 1804)

##### Materials

- **Type status:** Other material. **Occurrence:** individualCount: 1; sex: female; **Location:** locationID: S1; continent: Europe; country: Spain; countryCode: ES; stateProvince: Andalucía; county: Granada; locality: Soportujar; verbatimElevation: 1786.57; decimalLatitude: 36.96151; decimalLongitude: -3.41881; geodeticDatum: WGS84; **Event:** eventID: 2; samplingProtocol: Beating; eventTime: Night
- **Type status:** Other material. **Occurrence:** individualCount: 1; sex: female; **Location:** locationID: S2; continent: Europe; country: Spain; countryCode: ES; stateProvince: Andalucía; county: Granada; locality: Camarate; verbatimElevation: 1713.96; decimalLatitude: 37.18377; decimalLongitude: -3.26282; geodeticDatum: WGS84; **Event:** eventID: 1; samplingProtocol: Sweeping; eventTime: Night

##### Distribution

Palearctic

#### Oxyopes lineatus

Latreille, 1806

##### Materials

- **Type status:** Other material. **Occurrence:** individualCount: 1; sex: male; **Location:** locationID: C3; continent: Europe; country: Spain; countryCode: ES; stateProvince: Castilla-La Mancha; county: Ciudad Real; locality: La Quesera; verbatimElevation: 767.55; decimalLatitude: 39.36177; decimalLongitude: -4.41733; geodeticDatum: WGS84; **Event:** eventID: 1; samplingProtocol: Aerial; eventTime: Night
- **Type status:** Other material. **Occurrence:** individualCount: 1; sex: male; **Location:** locationID: C3; continent: Europe; country: Spain; countryCode: ES; stateProvince: Castilla-La Mancha; county: Ciudad Real; locality: La Quesera; verbatimElevation: 767.55; decimalLatitude: 39.36177; decimalLongitude: -4.41733; geodeticDatum: WGS84; **Event:** eventID: 1; samplingProtocol: Sweeping; eventTime: Night
- **Type status:** Other material. **Occurrence:** individualCount: 2; sex: female; **Location:** locationID: C3; continent: Europe; country: Spain; countryCode: ES; stateProvince: Castilla-La Mancha; county: Ciudad Real; locality: La Quesera; verbatimElevation: 767.55; decimalLatitude: 39.36177; decimalLongitude: -4.41733; geodeticDatum: WGS84; **Event:** eventID: 1; samplingProtocol: Sweeping; eventTime: Night
- **Type status:** Other material. **Occurrence:** individualCount: 4; sex: male; **Location:** locationID: C4; continent: Europe; country: Spain; countryCode: ES; stateProvince: Castilla-La Mancha; county: Ciudad Real; locality: La Quesera; verbatimElevation: 772.3; decimalLatitude: 39.36337; decimalLongitude: -4.41704; geodeticDatum: WGS84; **Event:** eventID: 1; samplingProtocol: Aerial; eventTime: Night
- **Type status:** Other material. **Occurrence:** individualCount: 1; sex: female; **Location:** locationID: C4; continent: Europe; country: Spain; countryCode: ES; stateProvince: Castilla-La Mancha; county: Ciudad Real; locality: La Quesera; verbatimElevation: 772.3; decimalLatitude: 39.36337; decimalLongitude: -4.41704; geodeticDatum: WGS84; **Event:** eventID: 1; samplingProtocol: Aerial; eventTime: Night
- **Type status:** Other material. **Occurrence:** individualCount: 2; sex: male; **Location:** locationID: C4; continent: Europe; country: Spain; countryCode: ES; stateProvince: Castilla-La Mancha; county: Ciudad Real; locality: La Quesera; verbatimElevation: 772.3; decimalLatitude: 39.36337; decimalLongitude: -4.41704; geodeticDatum: WGS84; **Event:** eventID: 2; samplingProtocol: Aerial; eventTime: Night
- **Type status:** Other material. **Occurrence:** individualCount: 1; sex: male; **Location:** locationID: C4; continent: Europe; country: Spain; countryCode: ES; stateProvince: Castilla-La Mancha; county: Ciudad Real; locality: La Quesera; verbatimElevation: 772.3; decimalLatitude: 39.36337; decimalLongitude: -4.41704; geodeticDatum: WGS84; **Event:** eventID: 3; samplingProtocol: Aerial; eventTime: Night
- **Type status:** Other material. **Occurrence:** individualCount: 1; sex: male; **Location:** locationID: C4; continent: Europe; country: Spain; countryCode: ES; stateProvince: Castilla-La Mancha; county: Ciudad Real; locality: La Quesera; verbatimElevation: 772.3; decimalLatitude: 39.36337; decimalLongitude: -4.41704; geodeticDatum: WGS84; **Event:** eventID: 1; samplingProtocol: Beating; eventTime: Night
- **Type status:** Other material. **Occurrence:** individualCount: 2; sex: female; **Location:** locationID: C4; continent: Europe; country: Spain; countryCode: ES; stateProvince: Castilla-La Mancha; county: Ciudad Real; locality: La Quesera; verbatimElevation: 772.3; decimalLatitude: 39.36337; decimalLongitude: -4.41704; geodeticDatum: WGS84; **Event:** eventID: 2; samplingProtocol: Beating; eventTime: Day
- **Type status:** Other material. **Occurrence:** individualCount: 16; sex: male; **Location:** locationID: C4; continent: Europe; country: Spain; countryCode: ES; stateProvince: Castilla-La Mancha; county: Ciudad Real; locality: La Quesera; verbatimElevation: 772.3; decimalLatitude: 39.36337; decimalLongitude: -4.41704; geodeticDatum: WGS84; **Event:** eventID: 1; samplingProtocol: Sweeping; eventTime: Day
- **Type status:** Other material. **Occurrence:** individualCount: 11; sex: female; **Location:** locationID: C4; continent: Europe; country: Spain; countryCode: ES; stateProvince: Castilla-La Mancha; county: Ciudad Real; locality: La Quesera; verbatimElevation: 772.3; decimalLatitude: 39.36337; decimalLongitude: -4.41704; geodeticDatum: WGS84; **Event:** eventID: 1; samplingProtocol: Sweeping; eventTime: Day
- **Type status:** Other material. **Occurrence:** individualCount: 4; sex: male; **Location:** locationID: C4; continent: Europe; country: Spain; countryCode: ES; stateProvince: Castilla-La Mancha; county: Ciudad Real; locality: La Quesera; verbatimElevation: 772.3; decimalLatitude: 39.36337; decimalLongitude: -4.41704; geodeticDatum: WGS84; **Event:** eventID: 2; samplingProtocol: Sweeping; eventTime: Day
- **Type status:** Other material. **Occurrence:** individualCount: 4; sex: female; **Location:** locationID: C4; continent: Europe; country: Spain; countryCode: ES; stateProvince: Castilla-La Mancha; county: Ciudad Real; locality: La Quesera; verbatimElevation: 772.3; decimalLatitude: 39.36337; decimalLongitude: -4.41704; geodeticDatum: WGS84; **Event:** eventID: 2; samplingProtocol: Sweeping; eventTime: Day
- **Type status:** Other material. **Occurrence:** individualCount: 7; sex: male; **Location:** locationID: C4; continent: Europe; country: Spain; countryCode: ES; stateProvince: Castilla-La Mancha; county: Ciudad Real; locality: La Quesera; verbatimElevation: 772.3; decimalLatitude: 39.36337; decimalLongitude: -4.41704; geodeticDatum: WGS84; **Event:** eventID: 1; samplingProtocol: Sweeping; eventTime: Night
- **Type status:** Other material. **Occurrence:** individualCount: 4; sex: female; **Location:** locationID: C4; continent: Europe; country: Spain; countryCode: ES; stateProvince: Castilla-La Mancha; county: Ciudad Real; locality: La Quesera; verbatimElevation: 772.3; decimalLatitude: 39.36337; decimalLongitude: -4.41704; geodeticDatum: WGS84; **Event:** eventID: 1; samplingProtocol: Sweeping; eventTime: Night
- **Type status:** Other material. **Occurrence:** individualCount: 8; sex: male; **Location:** locationID: C4; continent: Europe; country: Spain; countryCode: ES; stateProvince: Castilla-La Mancha; county: Ciudad Real; locality: La Quesera; verbatimElevation: 772.3; decimalLatitude: 39.36337; decimalLongitude: -4.41704; geodeticDatum: WGS84; **Event:** eventID: 2; samplingProtocol: Sweeping; eventTime: Night
- **Type status:** Other material. **Occurrence:** individualCount: 7; sex: female; **Location:** locationID: C4; continent: Europe; country: Spain; countryCode: ES; stateProvince: Castilla-La Mancha; county: Ciudad Real; locality: La Quesera; verbatimElevation: 772.3; decimalLatitude: 39.36337; decimalLongitude: -4.41704; geodeticDatum: WGS84; **Event:** eventID: 2; samplingProtocol: Sweeping; eventTime: Night
- **Type status:** Other material. **Occurrence:** individualCount: 1; sex: male; **Location:** locationID: O2; continent: Europe; country: Spain; countryCode: ES; stateProvince: Aragón; county: Huesca; locality: Rebilla; verbatimElevation: 1158.13; decimalLatitude: 42.59427; decimalLongitude: 0.1529; geodeticDatum: WGS84; **Event:** eventID: 2; samplingProtocol: Sweeping; eventTime: Day

##### Distribution

Palearctic

#### Oxyopes nigripalpis

Kulczynski, 1891

##### Materials

- **Type status:** Other material. **Occurrence:** individualCount: 1; sex: male; **Location:** locationID: C1; continent: Europe; country: Spain; countryCode: ES; stateProvince: Castilla-La Mancha; county: Ciudad Real; locality: Valle Brezoso; verbatimElevation: 756.56; decimalLatitude: 39.35663; decimalLongitude: -4.35912; geodeticDatum: WGS84; **Event:** eventID: 2; samplingProtocol: Aerial; eventTime: Night
- **Type status:** Other material. **Occurrence:** individualCount: 1; sex: male; **Location:** locationID: C1; continent: Europe; country: Spain; countryCode: ES; stateProvince: Castilla-La Mancha; county: Ciudad Real; locality: Valle Brezoso; verbatimElevation: 756.56; decimalLatitude: 39.35663; decimalLongitude: -4.35912; geodeticDatum: WGS84; **Event:** eventID: 4; samplingProtocol: Aerial; eventTime: Night
- **Type status:** Other material. **Occurrence:** individualCount: 1; sex: male; **Location:** locationID: C1; continent: Europe; country: Spain; countryCode: ES; stateProvince: Castilla-La Mancha; county: Ciudad Real; locality: Valle Brezoso; verbatimElevation: 756.56; decimalLatitude: 39.35663; decimalLongitude: -4.35912; geodeticDatum: WGS84; **Event:** eventID: 2; samplingProtocol: Beating; eventTime: Night
- **Type status:** Other material. **Occurrence:** individualCount: 1; sex: female; **Location:** locationID: C1; continent: Europe; country: Spain; countryCode: ES; stateProvince: Castilla-La Mancha; county: Ciudad Real; locality: Valle Brezoso; verbatimElevation: 756.56; decimalLatitude: 39.35663; decimalLongitude: -4.35912; geodeticDatum: WGS84; **Event:** eventID: 2; samplingProtocol: Beating; eventTime: Night
- **Type status:** Other material. **Occurrence:** individualCount: 1; sex: male; **Location:** locationID: C1; continent: Europe; country: Spain; countryCode: ES; stateProvince: Castilla-La Mancha; county: Ciudad Real; locality: Valle Brezoso; verbatimElevation: 756.56; decimalLatitude: 39.35663; decimalLongitude: -4.35912; geodeticDatum: WGS84; **Event:** eventID: 2; samplingProtocol: Sweeping; eventTime: Night
- **Type status:** Other material. **Occurrence:** individualCount: 1; sex: female; **Location:** locationID: C1; continent: Europe; country: Spain; countryCode: ES; stateProvince: Castilla-La Mancha; county: Ciudad Real; locality: Valle Brezoso; verbatimElevation: 756.56; decimalLatitude: 39.35663; decimalLongitude: -4.35912; geodeticDatum: WGS84; **Event:** eventID: 1; samplingProtocol: Sweeping; eventTime: Night
- **Type status:** Other material. **Occurrence:** individualCount: 1; sex: male; **Location:** locationID: C1; continent: Europe; country: Spain; countryCode: ES; stateProvince: Castilla-La Mancha; county: Ciudad Real; locality: Valle Brezoso; verbatimElevation: 756.56; decimalLatitude: 39.35663; decimalLongitude: -4.35912; geodeticDatum: WGS84; **Event:** eventID: 1; samplingProtocol: Sweeping; eventTime: Night
- **Type status:** Other material. **Occurrence:** individualCount: 2; sex: female; **Location:** locationID: C1; continent: Europe; country: Spain; countryCode: ES; stateProvince: Castilla-La Mancha; county: Ciudad Real; locality: Valle Brezoso; verbatimElevation: 756.56; decimalLatitude: 39.35663; decimalLongitude: -4.35912; geodeticDatum: WGS84; **Event:** eventID: 2; samplingProtocol: Sweeping; eventTime: Night
- **Type status:** Other material. **Occurrence:** individualCount: 2; sex: male; **Location:** locationID: C1; continent: Europe; country: Spain; countryCode: ES; stateProvince: Castilla-La Mancha; county: Ciudad Real; locality: Valle Brezoso; verbatimElevation: 756.56; decimalLatitude: 39.35663; decimalLongitude: -4.35912; geodeticDatum: WGS84; **Event:** eventID: 2; samplingProtocol: Sweeping; eventTime: Day
- **Type status:** Other material. **Occurrence:** individualCount: 1; sex: male; **Location:** locationID: C2; continent: Europe; country: Spain; countryCode: ES; stateProvince: Castilla-La Mancha; county: Ciudad Real; locality: Valle Brezoso; verbatimElevation: 739.31; decimalLatitude: 39.35159; decimalLongitude: -4.3589; geodeticDatum: WGS84; **Event:** eventID: 1; samplingProtocol: Beating; eventTime: Night
- **Type status:** Other material. **Occurrence:** individualCount: 2; sex: male; **Location:** locationID: C2; continent: Europe; country: Spain; countryCode: ES; stateProvince: Castilla-La Mancha; county: Ciudad Real; locality: Valle Brezoso; verbatimElevation: 739.31; decimalLatitude: 39.35159; decimalLongitude: -4.3589; geodeticDatum: WGS84; **Event:** eventID: 2; samplingProtocol: Beating; eventTime: Night
- **Type status:** Other material. **Occurrence:** individualCount: 1; sex: female; **Location:** locationID: C2; continent: Europe; country: Spain; countryCode: ES; stateProvince: Castilla-La Mancha; county: Ciudad Real; locality: Valle Brezoso; verbatimElevation: 739.31; decimalLatitude: 39.35159; decimalLongitude: -4.3589; geodeticDatum: WGS84; **Event:** eventID: 1; samplingProtocol: Beating; eventTime: Day
- **Type status:** Other material. **Occurrence:** individualCount: 1; sex: female; **Location:** locationID: C2; continent: Europe; country: Spain; countryCode: ES; stateProvince: Castilla-La Mancha; county: Ciudad Real; locality: Valle Brezoso; verbatimElevation: 739.31; decimalLatitude: 39.35159; decimalLongitude: -4.3589; geodeticDatum: WGS84; **Event:** eventID: 1; samplingProtocol: Sweeping; eventTime: Day
- **Type status:** Other material. **Occurrence:** individualCount: 1; sex: female; **Location:** locationID: C3; continent: Europe; country: Spain; countryCode: ES; stateProvince: Castilla-La Mancha; county: Ciudad Real; locality: La Quesera; verbatimElevation: 767.55; decimalLatitude: 39.36177; decimalLongitude: -4.41733; geodeticDatum: WGS84; **Event:** eventID: 2; samplingProtocol: Aerial; eventTime: Night
- **Type status:** Other material. **Occurrence:** individualCount: 1; sex: male; **Location:** locationID: C3; continent: Europe; country: Spain; countryCode: ES; stateProvince: Castilla-La Mancha; county: Ciudad Real; locality: La Quesera; verbatimElevation: 767.55; decimalLatitude: 39.36177; decimalLongitude: -4.41733; geodeticDatum: WGS84; **Event:** eventID: 1; samplingProtocol: Aerial; eventTime: Night
- **Type status:** Other material. **Occurrence:** individualCount: 4; sex: female; **Location:** locationID: C3; continent: Europe; country: Spain; countryCode: ES; stateProvince: Castilla-La Mancha; county: Ciudad Real; locality: La Quesera; verbatimElevation: 767.55; decimalLatitude: 39.36177; decimalLongitude: -4.41733; geodeticDatum: WGS84; **Event:** eventID: 1; samplingProtocol: Beating; eventTime: Day
- **Type status:** Other material. **Occurrence:** individualCount: 1; sex: male; **Location:** locationID: C3; continent: Europe; country: Spain; countryCode: ES; stateProvince: Castilla-La Mancha; county: Ciudad Real; locality: La Quesera; verbatimElevation: 767.55; decimalLatitude: 39.36177; decimalLongitude: -4.41733; geodeticDatum: WGS84; **Event:** eventID: 1; samplingProtocol: Beating; eventTime: Day
- **Type status:** Other material. **Occurrence:** individualCount: 4; sex: female; **Location:** locationID: C3; continent: Europe; country: Spain; countryCode: ES; stateProvince: Castilla-La Mancha; county: Ciudad Real; locality: La Quesera; verbatimElevation: 767.55; decimalLatitude: 39.36177; decimalLongitude: -4.41733; geodeticDatum: WGS84; **Event:** eventID: 2; samplingProtocol: Beating; eventTime: Day
- **Type status:** Other material. **Occurrence:** individualCount: 1; sex: male; **Location:** locationID: C3; continent: Europe; country: Spain; countryCode: ES; stateProvince: Castilla-La Mancha; county: Ciudad Real; locality: La Quesera; verbatimElevation: 767.55; decimalLatitude: 39.36177; decimalLongitude: -4.41733; geodeticDatum: WGS84; **Event:** eventID: 2; samplingProtocol: Beating; eventTime: Day
- **Type status:** Other material. **Occurrence:** individualCount: 2; sex: male; **Location:** locationID: C3; continent: Europe; country: Spain; countryCode: ES; stateProvince: Castilla-La Mancha; county: Ciudad Real; locality: La Quesera; verbatimElevation: 767.55; decimalLatitude: 39.36177; decimalLongitude: -4.41733; geodeticDatum: WGS84; **Event:** eventID: 1; samplingProtocol: Beating; eventTime: Night
- **Type status:** Other material. **Occurrence:** individualCount: 1; sex: female; **Location:** locationID: C3; continent: Europe; country: Spain; countryCode: ES; stateProvince: Castilla-La Mancha; county: Ciudad Real; locality: La Quesera; verbatimElevation: 767.55; decimalLatitude: 39.36177; decimalLongitude: -4.41733; geodeticDatum: WGS84; **Event:** eventID: 2; samplingProtocol: Beating; eventTime: Night
- **Type status:** Other material. **Occurrence:** individualCount: 3; sex: female; **Location:** locationID: C3; continent: Europe; country: Spain; countryCode: ES; stateProvince: Castilla-La Mancha; county: Ciudad Real; locality: La Quesera; verbatimElevation: 767.55; decimalLatitude: 39.36177; decimalLongitude: -4.41733; geodeticDatum: WGS84; **Event:** eventID: 1; samplingProtocol: Sweeping; eventTime: Day
- **Type status:** Other material. **Occurrence:** individualCount: 1; sex: male; **Location:** locationID: C3; continent: Europe; country: Spain; countryCode: ES; stateProvince: Castilla-La Mancha; county: Ciudad Real; locality: La Quesera; verbatimElevation: 767.55; decimalLatitude: 39.36177; decimalLongitude: -4.41733; geodeticDatum: WGS84; **Event:** eventID: 1; samplingProtocol: Sweeping; eventTime: Day
- **Type status:** Other material. **Occurrence:** individualCount: 3; sex: female; **Location:** locationID: C3; continent: Europe; country: Spain; countryCode: ES; stateProvince: Castilla-La Mancha; county: Ciudad Real; locality: La Quesera; verbatimElevation: 767.55; decimalLatitude: 39.36177; decimalLongitude: -4.41733; geodeticDatum: WGS84; **Event:** eventID: 2; samplingProtocol: Sweeping; eventTime: Day
- **Type status:** Other material. **Occurrence:** individualCount: 4; sex: male; **Location:** locationID: C3; continent: Europe; country: Spain; countryCode: ES; stateProvince: Castilla-La Mancha; county: Ciudad Real; locality: La Quesera; verbatimElevation: 767.55; decimalLatitude: 39.36177; decimalLongitude: -4.41733; geodeticDatum: WGS84; **Event:** eventID: 2; samplingProtocol: Sweeping; eventTime: Day
- **Type status:** Other material. **Occurrence:** individualCount: 7; sex: female; **Location:** locationID: C3; continent: Europe; country: Spain; countryCode: ES; stateProvince: Castilla-La Mancha; county: Ciudad Real; locality: La Quesera; verbatimElevation: 767.55; decimalLatitude: 39.36177; decimalLongitude: -4.41733; geodeticDatum: WGS84; **Event:** eventID: 1; samplingProtocol: Sweeping; eventTime: Night
- **Type status:** Other material. **Occurrence:** individualCount: 4; sex: male; **Location:** locationID: C3; continent: Europe; country: Spain; countryCode: ES; stateProvince: Castilla-La Mancha; county: Ciudad Real; locality: La Quesera; verbatimElevation: 767.55; decimalLatitude: 39.36177; decimalLongitude: -4.41733; geodeticDatum: WGS84; **Event:** eventID: 1; samplingProtocol: Sweeping; eventTime: Night
- **Type status:** Other material. **Occurrence:** individualCount: 6; sex: female; **Location:** locationID: C3; continent: Europe; country: Spain; countryCode: ES; stateProvince: Castilla-La Mancha; county: Ciudad Real; locality: La Quesera; verbatimElevation: 767.55; decimalLatitude: 39.36177; decimalLongitude: -4.41733; geodeticDatum: WGS84; **Event:** eventID: 2; samplingProtocol: Sweeping; eventTime: Night
- **Type status:** Other material. **Occurrence:** individualCount: 3; sex: male; **Location:** locationID: C3; continent: Europe; country: Spain; countryCode: ES; stateProvince: Castilla-La Mancha; county: Ciudad Real; locality: La Quesera; verbatimElevation: 767.55; decimalLatitude: 39.36177; decimalLongitude: -4.41733; geodeticDatum: WGS84; **Event:** eventID: 2; samplingProtocol: Sweeping; eventTime: Night
- **Type status:** Other material. **Occurrence:** individualCount: 1; sex: female; **Location:** locationID: C4; continent: Europe; country: Spain; countryCode: ES; stateProvince: Castilla-La Mancha; county: Ciudad Real; locality: La Quesera; verbatimElevation: 772.3; decimalLatitude: 39.36337; decimalLongitude: -4.41704; geodeticDatum: WGS84; **Event:** eventID: 3; samplingProtocol: Aerial; eventTime: Night
- **Type status:** Other material. **Occurrence:** individualCount: 2; sex: female; **Location:** locationID: C4; continent: Europe; country: Spain; countryCode: ES; stateProvince: Castilla-La Mancha; county: Ciudad Real; locality: La Quesera; verbatimElevation: 772.3; decimalLatitude: 39.36337; decimalLongitude: -4.41704; geodeticDatum: WGS84; **Event:** eventID: 1; samplingProtocol: Beating; eventTime: Day
- **Type status:** Other material. **Occurrence:** individualCount: 1; sex: female; **Location:** locationID: C4; continent: Europe; country: Spain; countryCode: ES; stateProvince: Castilla-La Mancha; county: Ciudad Real; locality: La Quesera; verbatimElevation: 772.3; decimalLatitude: 39.36337; decimalLongitude: -4.41704; geodeticDatum: WGS84; **Event:** eventID: 1; samplingProtocol: Beating; eventTime: Night
- **Type status:** Other material. **Occurrence:** individualCount: 2; sex: male; **Location:** locationID: C4; continent: Europe; country: Spain; countryCode: ES; stateProvince: Castilla-La Mancha; county: Ciudad Real; locality: La Quesera; verbatimElevation: 772.3; decimalLatitude: 39.36337; decimalLongitude: -4.41704; geodeticDatum: WGS84; **Event:** eventID: 1; samplingProtocol: Beating; eventTime: Night
- **Type status:** Other material. **Occurrence:** individualCount: 1; sex: male; **Location:** locationID: C4; continent: Europe; country: Spain; countryCode: ES; stateProvince: Castilla-La Mancha; county: Ciudad Real; locality: La Quesera; verbatimElevation: 772.3; decimalLatitude: 39.36337; decimalLongitude: -4.41704; geodeticDatum: WGS84; **Event:** eventID: E; samplingProtocol: Pitfall
- **Type status:** Other material. **Occurrence:** individualCount: 5; sex: female; **Location:** locationID: C4; continent: Europe; country: Spain; countryCode: ES; stateProvince: Castilla-La Mancha; county: Ciudad Real; locality: La Quesera; verbatimElevation: 772.3; decimalLatitude: 39.36337; decimalLongitude: -4.41704; geodeticDatum: WGS84; **Event:** eventID: 1; samplingProtocol: Sweeping; eventTime: Day
- **Type status:** Other material. **Occurrence:** individualCount: 3; sex: male; **Location:** locationID: C4; continent: Europe; country: Spain; countryCode: ES; stateProvince: Castilla-La Mancha; county: Ciudad Real; locality: La Quesera; verbatimElevation: 772.3; decimalLatitude: 39.36337; decimalLongitude: -4.41704; geodeticDatum: WGS84; **Event:** eventID: 1; samplingProtocol: Sweeping; eventTime: Day
- **Type status:** Other material. **Occurrence:** individualCount: 1; sex: female; **Location:** locationID: C4; continent: Europe; country: Spain; countryCode: ES; stateProvince: Castilla-La Mancha; county: Ciudad Real; locality: La Quesera; verbatimElevation: 772.3; decimalLatitude: 39.36337; decimalLongitude: -4.41704; geodeticDatum: WGS84; **Event:** eventID: 2; samplingProtocol: Sweeping; eventTime: Day
- **Type status:** Other material. **Occurrence:** individualCount: 4; sex: male; **Location:** locationID: C4; continent: Europe; country: Spain; countryCode: ES; stateProvince: Castilla-La Mancha; county: Ciudad Real; locality: La Quesera; verbatimElevation: 772.3; decimalLatitude: 39.36337; decimalLongitude: -4.41704; geodeticDatum: WGS84; **Event:** eventID: 2; samplingProtocol: Sweeping; eventTime: Day
- **Type status:** Other material. **Occurrence:** individualCount: 2; sex: female; **Location:** locationID: C4; continent: Europe; country: Spain; countryCode: ES; stateProvince: Castilla-La Mancha; county: Ciudad Real; locality: La Quesera; verbatimElevation: 772.3; decimalLatitude: 39.36337; decimalLongitude: -4.41704; geodeticDatum: WGS84; **Event:** eventID: 1; samplingProtocol: Sweeping; eventTime: Night
- **Type status:** Other material. **Occurrence:** individualCount: 2; sex: male; **Location:** locationID: C4; continent: Europe; country: Spain; countryCode: ES; stateProvince: Castilla-La Mancha; county: Ciudad Real; locality: La Quesera; verbatimElevation: 772.3; decimalLatitude: 39.36337; decimalLongitude: -4.41704; geodeticDatum: WGS84; **Event:** eventID: 1; samplingProtocol: Sweeping; eventTime: Night
- **Type status:** Other material. **Occurrence:** individualCount: 3; sex: female; **Location:** locationID: C4; continent: Europe; country: Spain; countryCode: ES; stateProvince: Castilla-La Mancha; county: Ciudad Real; locality: La Quesera; verbatimElevation: 772.3; decimalLatitude: 39.36337; decimalLongitude: -4.41704; geodeticDatum: WGS84; **Event:** eventID: 2; samplingProtocol: Sweeping; eventTime: Night
- **Type status:** Other material. **Occurrence:** individualCount: 4; sex: male; **Location:** locationID: C4; continent: Europe; country: Spain; countryCode: ES; stateProvince: Castilla-La Mancha; county: Ciudad Real; locality: La Quesera; verbatimElevation: 772.3; decimalLatitude: 39.36337; decimalLongitude: -4.41704; geodeticDatum: WGS84; **Event:** eventID: 2; samplingProtocol: Sweeping; eventTime: Night
- **Type status:** Other material. **Occurrence:** individualCount: 1; sex: female; **Location:** locationID: M1; continent: Europe; country: Spain; countryCode: ES; stateProvince: Extremadura; county: Cáceres; locality: Peña Falcón; verbatimElevation: 320.6; decimalLatitude: 39.83296; decimalLongitude: -6.0641; geodeticDatum: WGS84; **Event:** eventID: 2; samplingProtocol: Sweeping; eventTime: Day
- **Type status:** Other material. **Occurrence:** individualCount: 1; sex: male; **Location:** locationID: M1; continent: Europe; country: Spain; countryCode: ES; stateProvince: Extremadura; county: Cáceres; locality: Peña Falcón; verbatimElevation: 320.6; decimalLatitude: 39.83296; decimalLongitude: -6.0641; geodeticDatum: WGS84; **Event:** eventID: 2; samplingProtocol: Sweeping; eventTime: Day
- **Type status:** Other material. **Occurrence:** individualCount: 3; sex: male; **Location:** locationID: S1; continent: Europe; country: Spain; countryCode: ES; stateProvince: Andalucía; county: Granada; locality: Soportujar; verbatimElevation: 1786.57; decimalLatitude: 36.96151; decimalLongitude: -3.41881; geodeticDatum: WGS84; **Event:** eventID: 1; samplingProtocol: Aerial; eventTime: Night
- **Type status:** Other material. **Occurrence:** individualCount: 1; sex: female; **Location:** locationID: S1; continent: Europe; country: Spain; countryCode: ES; stateProvince: Andalucía; county: Granada; locality: Soportujar; verbatimElevation: 1786.57; decimalLatitude: 36.96151; decimalLongitude: -3.41881; geodeticDatum: WGS84; **Event:** eventID: 2; samplingProtocol: Aerial; eventTime: Night
- **Type status:** Other material. **Occurrence:** individualCount: 1; sex: male; **Location:** locationID: S1; continent: Europe; country: Spain; countryCode: ES; stateProvince: Andalucía; county: Granada; locality: Soportujar; verbatimElevation: 1786.57; decimalLatitude: 36.96151; decimalLongitude: -3.41881; geodeticDatum: WGS84; **Event:** eventID: 2; samplingProtocol: Aerial; eventTime: Night
- **Type status:** Other material. **Occurrence:** individualCount: 1; sex: female; **Location:** locationID: S1; continent: Europe; country: Spain; countryCode: ES; stateProvince: Andalucía; county: Granada; locality: Soportujar; verbatimElevation: 1786.57; decimalLatitude: 36.96151; decimalLongitude: -3.41881; geodeticDatum: WGS84; **Event:** eventID: 3; samplingProtocol: Aerial; eventTime: Night
- **Type status:** Other material. **Occurrence:** individualCount: 2; sex: male; **Location:** locationID: S1; continent: Europe; country: Spain; countryCode: ES; stateProvince: Andalucía; county: Granada; locality: Soportujar; verbatimElevation: 1786.57; decimalLatitude: 36.96151; decimalLongitude: -3.41881; geodeticDatum: WGS84; **Event:** eventID: 1; samplingProtocol: Beating; eventTime: Night
- **Type status:** Other material. **Occurrence:** individualCount: 1; sex: male; **Location:** locationID: S1; continent: Europe; country: Spain; countryCode: ES; stateProvince: Andalucía; county: Granada; locality: Soportujar; verbatimElevation: 1786.57; decimalLatitude: 36.96151; decimalLongitude: -3.41881; geodeticDatum: WGS84; **Event:** eventID: 2; samplingProtocol: Beating; eventTime: Day
- **Type status:** Other material. **Occurrence:** individualCount: 2; sex: female; **Location:** locationID: S1; continent: Europe; country: Spain; countryCode: ES; stateProvince: Andalucía; county: Granada; locality: Soportujar; verbatimElevation: 1786.57; decimalLatitude: 36.96151; decimalLongitude: -3.41881; geodeticDatum: WGS84; **Event:** eventID: 2; samplingProtocol: Sweeping; eventTime: Day
- **Type status:** Other material. **Occurrence:** individualCount: 1; sex: male; **Location:** locationID: S1; continent: Europe; country: Spain; countryCode: ES; stateProvince: Andalucía; county: Granada; locality: Soportujar; verbatimElevation: 1786.57; decimalLatitude: 36.96151; decimalLongitude: -3.41881; geodeticDatum: WGS84; **Event:** eventID: 2; samplingProtocol: Sweeping; eventTime: Day
- **Type status:** Other material. **Occurrence:** individualCount: 2; sex: male; **Location:** locationID: S1; continent: Europe; country: Spain; countryCode: ES; stateProvince: Andalucía; county: Granada; locality: Soportujar; verbatimElevation: 1786.57; decimalLatitude: 36.96151; decimalLongitude: -3.41881; geodeticDatum: WGS84; **Event:** eventID: 1; samplingProtocol: Sweeping; eventTime: Night
- **Type status:** Other material. **Occurrence:** individualCount: 1; sex: male; **Location:** locationID: S1; continent: Europe; country: Spain; countryCode: ES; stateProvince: Andalucía; county: Granada; locality: Soportujar; verbatimElevation: 1786.57; decimalLatitude: 36.96151; decimalLongitude: -3.41881; geodeticDatum: WGS84; **Event:** eventID: 2; samplingProtocol: Sweeping; eventTime: Night
- **Type status:** Other material. **Occurrence:** individualCount: 1; sex: male; **Location:** locationID: S2; continent: Europe; country: Spain; countryCode: ES; stateProvince: Andalucía; county: Granada; locality: Camarate; verbatimElevation: 1713.96; decimalLatitude: 37.18377; decimalLongitude: -3.26282; geodeticDatum: WGS84; **Event:** eventID: 2; samplingProtocol: Aerial; eventTime: Night
- **Type status:** Other material. **Occurrence:** individualCount: 2; sex: female; **Location:** locationID: S2; continent: Europe; country: Spain; countryCode: ES; stateProvince: Andalucía; county: Granada; locality: Camarate; verbatimElevation: 1713.96; decimalLatitude: 37.18377; decimalLongitude: -3.26282; geodeticDatum: WGS84; **Event:** eventID: 3; samplingProtocol: Aerial; eventTime: Night
- **Type status:** Other material. **Occurrence:** individualCount: 5; sex: male; **Location:** locationID: S2; continent: Europe; country: Spain; countryCode: ES; stateProvince: Andalucía; county: Granada; locality: Camarate; verbatimElevation: 1713.96; decimalLatitude: 37.18377; decimalLongitude: -3.26282; geodeticDatum: WGS84; **Event:** eventID: 3; samplingProtocol: Aerial; eventTime: Night
- **Type status:** Other material. **Occurrence:** individualCount: 1; sex: female; **Location:** locationID: S2; continent: Europe; country: Spain; countryCode: ES; stateProvince: Andalucía; county: Granada; locality: Camarate; verbatimElevation: 1713.96; decimalLatitude: 37.18377; decimalLongitude: -3.26282; geodeticDatum: WGS84; **Event:** eventID: 4; samplingProtocol: Aerial; eventTime: Night
- **Type status:** Other material. **Occurrence:** individualCount: 2; sex: male; **Location:** locationID: S2; continent: Europe; country: Spain; countryCode: ES; stateProvince: Andalucía; county: Granada; locality: Camarate; verbatimElevation: 1713.96; decimalLatitude: 37.18377; decimalLongitude: -3.26282; geodeticDatum: WGS84; **Event:** eventID: 4; samplingProtocol: Aerial; eventTime: Night
- **Type status:** Other material. **Occurrence:** individualCount: 1; sex: female; **Location:** locationID: S2; continent: Europe; country: Spain; countryCode: ES; stateProvince: Andalucía; county: Granada; locality: Camarate; verbatimElevation: 1713.96; decimalLatitude: 37.18377; decimalLongitude: -3.26282; geodeticDatum: WGS84; **Event:** eventID: 1; samplingProtocol: Beating; eventTime: Night
- **Type status:** Other material. **Occurrence:** individualCount: 1; sex: female; **Location:** locationID: S2; continent: Europe; country: Spain; countryCode: ES; stateProvince: Andalucía; county: Granada; locality: Camarate; verbatimElevation: 1713.96; decimalLatitude: 37.18377; decimalLongitude: -3.26282; geodeticDatum: WGS84; **Event:** eventID: 2; samplingProtocol: Beating; eventTime: Night
- **Type status:** Other material. **Occurrence:** individualCount: 5; sex: male; **Location:** locationID: S2; continent: Europe; country: Spain; countryCode: ES; stateProvince: Andalucía; county: Granada; locality: Camarate; verbatimElevation: 1713.96; decimalLatitude: 37.18377; decimalLongitude: -3.26282; geodeticDatum: WGS84; **Event:** eventID: 2; samplingProtocol: Beating; eventTime: Night
- **Type status:** Other material. **Occurrence:** individualCount: 2; sex: female; **Location:** locationID: S2; continent: Europe; country: Spain; countryCode: ES; stateProvince: Andalucía; county: Granada; locality: Camarate; verbatimElevation: 1713.96; decimalLatitude: 37.18377; decimalLongitude: -3.26282; geodeticDatum: WGS84; **Event:** eventID: 1; samplingProtocol: Sweeping; eventTime: Day
- **Type status:** Other material. **Occurrence:** individualCount: 1; sex: male; **Location:** locationID: S2; continent: Europe; country: Spain; countryCode: ES; stateProvince: Andalucía; county: Granada; locality: Camarate; verbatimElevation: 1713.96; decimalLatitude: 37.18377; decimalLongitude: -3.26282; geodeticDatum: WGS84; **Event:** eventID: 1; samplingProtocol: Sweeping; eventTime: Day
- **Type status:** Other material. **Occurrence:** individualCount: 2; sex: male; **Location:** locationID: S2; continent: Europe; country: Spain; countryCode: ES; stateProvince: Andalucía; county: Granada; locality: Camarate; verbatimElevation: 1713.96; decimalLatitude: 37.18377; decimalLongitude: -3.26282; geodeticDatum: WGS84; **Event:** eventID: 2; samplingProtocol: Sweeping; eventTime: Day
- **Type status:** Other material. **Occurrence:** individualCount: 3; sex: female; **Location:** locationID: S2; continent: Europe; country: Spain; countryCode: ES; stateProvince: Andalucía; county: Granada; locality: Camarate; verbatimElevation: 1713.96; decimalLatitude: 37.18377; decimalLongitude: -3.26282; geodeticDatum: WGS84; **Event:** eventID: 1; samplingProtocol: Sweeping; eventTime: Night
- **Type status:** Other material. **Occurrence:** individualCount: 5; sex: male; **Location:** locationID: S2; continent: Europe; country: Spain; countryCode: ES; stateProvince: Andalucía; county: Granada; locality: Camarate; verbatimElevation: 1713.96; decimalLatitude: 37.18377; decimalLongitude: -3.26282; geodeticDatum: WGS84; **Event:** eventID: 1; samplingProtocol: Sweeping; eventTime: Night
- **Type status:** Other material. **Occurrence:** individualCount: 2; sex: female; **Location:** locationID: S2; continent: Europe; country: Spain; countryCode: ES; stateProvince: Andalucía; county: Granada; locality: Camarate; verbatimElevation: 1713.96; decimalLatitude: 37.18377; decimalLongitude: -3.26282; geodeticDatum: WGS84; **Event:** eventID: 2; samplingProtocol: Sweeping; eventTime: Night
- **Type status:** Other material. **Occurrence:** individualCount: 1; sex: male; **Location:** locationID: S2; continent: Europe; country: Spain; countryCode: ES; stateProvince: Andalucía; county: Granada; locality: Camarate; verbatimElevation: 1713.96; decimalLatitude: 37.18377; decimalLongitude: -3.26282; geodeticDatum: WGS84; **Event:** eventID: 2; samplingProtocol: Sweeping; eventTime: Night

##### Distribution

Mediterranean

#### Palpimanidae

Thorell, 1870

#### Palpimanus gibbulus

Dufour, 1820

##### Materials

- **Type status:** Other material. **Occurrence:** individualCount: 1; sex: female; **Location:** locationID: S1; continent: Europe; country: Spain; countryCode: ES; stateProvince: Andalucía; county: Granada; locality: Soportujar; verbatimElevation: 1786.57; decimalLatitude: 36.96151; decimalLongitude: -3.41881; geodeticDatum: WGS84; **Event:** eventID: H; samplingProtocol: Pitfall
- **Type status:** Other material. **Occurrence:** individualCount: 1; sex: male; **Location:** locationID: S1; continent: Europe; country: Spain; countryCode: ES; stateProvince: Andalucía; county: Granada; locality: Soportujar; verbatimElevation: 1786.57; decimalLatitude: 36.96151; decimalLongitude: -3.41881; geodeticDatum: WGS84; **Event:** eventID: K; samplingProtocol: Pitfall
- **Type status:** Other material. **Occurrence:** individualCount: 1; sex: female; **Location:** locationID: S1; continent: Europe; country: Spain; countryCode: ES; stateProvince: Andalucía; county: Granada; locality: Soportujar; verbatimElevation: 1786.57; decimalLatitude: 36.96151; decimalLongitude: -3.41881; geodeticDatum: WGS84; **Event:** eventID: L; samplingProtocol: Pitfall

##### Distribution

Mediterranean to Central Asia

#### Philodromidae

Thorell, 1870

#### Philodromus albidus

Kulczynski, 1911

##### Materials

- **Type status:** Other material. **Occurrence:** individualCount: 2; sex: female; **Location:** locationID: O1; continent: Europe; country: Spain; countryCode: ES; stateProvince: Aragón; county: Huesca; locality: O Furno; verbatimElevation: 1396.73; decimalLatitude: 42.60677; decimalLongitude: 0.13135; geodeticDatum: WGS84; **Event:** eventID: 1; samplingProtocol: Beating; eventTime: Day
- **Type status:** Other material. **Occurrence:** individualCount: 3; sex: female; **Location:** locationID: O1; continent: Europe; country: Spain; countryCode: ES; stateProvince: Aragón; county: Huesca; locality: O Furno; verbatimElevation: 1396.73; decimalLatitude: 42.60677; decimalLongitude: 0.13135; geodeticDatum: WGS84; **Event:** eventID: 2; samplingProtocol: Beating; eventTime: Day
- **Type status:** Other material. **Occurrence:** individualCount: 1; sex: female; **Location:** locationID: O1; continent: Europe; country: Spain; countryCode: ES; stateProvince: Aragón; county: Huesca; locality: O Furno; verbatimElevation: 1396.73; decimalLatitude: 42.60677; decimalLongitude: 0.13135; geodeticDatum: WGS84; **Event:** eventID: 1; samplingProtocol: Beating; eventTime: Night
- **Type status:** Other material. **Occurrence:** individualCount: 1; sex: female; **Location:** locationID: P1; continent: Europe; country: Spain; countryCode: ES; stateProvince: Castilla y León; county: León; locality: Monte Robledo; verbatimElevation: 1071.58; decimalLatitude: 43.1445; decimalLongitude: -4.92675; geodeticDatum: WGS84; **Event:** eventID: 1; samplingProtocol: Beating; eventTime: Day
- **Type status:** Other material. **Occurrence:** individualCount: 1; sex: female; **Location:** locationID: P1; continent: Europe; country: Spain; countryCode: ES; stateProvince: Castilla y León; county: León; locality: Monte Robledo; verbatimElevation: 1071.58; decimalLatitude: 43.1445; decimalLongitude: -4.92675; geodeticDatum: WGS84; **Event:** eventID: 1; samplingProtocol: Sweeping; eventTime: Day
- **Type status:** Other material. **Occurrence:** individualCount: 1; sex: female; **Location:** locationID: P1; continent: Europe; country: Spain; countryCode: ES; stateProvince: Castilla y León; county: León; locality: Monte Robledo; verbatimElevation: 1071.58; decimalLatitude: 43.1445; decimalLongitude: -4.92675; geodeticDatum: WGS84; **Event:** eventID: 1; samplingProtocol: Sweeping; eventTime: Day
- **Type status:** Other material. **Occurrence:** individualCount: 1; sex: female; **Location:** locationID: P1; continent: Europe; country: Spain; countryCode: ES; stateProvince: Castilla y León; county: León; locality: Monte Robledo; verbatimElevation: 1071.58; decimalLatitude: 43.1445; decimalLongitude: -4.92675; geodeticDatum: WGS84; **Event:** eventID: 2; samplingProtocol: Sweeping; eventTime: Day
- **Type status:** Other material. **Occurrence:** individualCount: 1; sex: female; **Location:** locationID: P1; continent: Europe; country: Spain; countryCode: ES; stateProvince: Castilla y León; county: León; locality: Monte Robledo; verbatimElevation: 1071.58; decimalLatitude: 43.1445; decimalLongitude: -4.92675; geodeticDatum: WGS84; **Event:** eventID: 1; samplingProtocol: Sweeping; eventTime: Night
- **Type status:** Other material. **Occurrence:** individualCount: 1; sex: female; **Location:** locationID: P2; continent: Europe; country: Spain; countryCode: ES; stateProvince: Castilla y León; county: León; locality: Joyoguelas; verbatimElevation: 763.98; decimalLatitude: 43.17771; decimalLongitude: -4.90579; geodeticDatum: WGS84; **Event:** eventID: 2; samplingProtocol: Sweeping; eventTime: Night

##### Distribution

Europe

#### Philodromus aureolus

(Clerck, 1757)

##### Materials

- **Type status:** Other material. **Occurrence:** individualCount: 2; sex: female; **Location:** locationID: A1; continent: Europe; country: Spain; countryCode: ES; stateProvince: Catalonia; county: Lleida; locality: Sola de Boi; verbatimElevation: 1759.8; decimalLatitude: 42.54958; decimalLongitude: 0.87254; geodeticDatum: WGS84; **Event:** eventID: 1; samplingProtocol: Beating; eventTime: Day
- **Type status:** Other material. **Occurrence:** individualCount: 2; sex: female; **Location:** locationID: A1; continent: Europe; country: Spain; countryCode: ES; stateProvince: Catalonia; county: Lleida; locality: Sola de Boi; verbatimElevation: 1759.8; decimalLatitude: 42.54958; decimalLongitude: 0.87254; geodeticDatum: WGS84; **Event:** eventID: 2; samplingProtocol: Beating; eventTime: Day
- **Type status:** Other material. **Occurrence:** individualCount: 5; sex: female; **Location:** locationID: A1; continent: Europe; country: Spain; countryCode: ES; stateProvince: Catalonia; county: Lleida; locality: Sola de Boi; verbatimElevation: 1759.8; decimalLatitude: 42.54958; decimalLongitude: 0.87254; geodeticDatum: WGS84; **Event:** eventID: 1; samplingProtocol: Beating; eventTime: Night
- **Type status:** Other material. **Occurrence:** individualCount: 1; sex: male; **Location:** locationID: A1; continent: Europe; country: Spain; countryCode: ES; stateProvince: Catalonia; county: Lleida; locality: Sola de Boi; verbatimElevation: 1759.8; decimalLatitude: 42.54958; decimalLongitude: 0.87254; geodeticDatum: WGS84; **Event:** eventID: 1; samplingProtocol: Beating; eventTime: Night
- **Type status:** Other material. **Occurrence:** individualCount: 3; sex: female; **Location:** locationID: A1; continent: Europe; country: Spain; countryCode: ES; stateProvince: Catalonia; county: Lleida; locality: Sola de Boi; verbatimElevation: 1759.8; decimalLatitude: 42.54958; decimalLongitude: 0.87254; geodeticDatum: WGS84; **Event:** eventID: 1; samplingProtocol: Beating; eventTime: Night
- **Type status:** Other material. **Occurrence:** individualCount: 1; sex: male; **Location:** locationID: A1; continent: Europe; country: Spain; countryCode: ES; stateProvince: Catalonia; county: Lleida; locality: Sola de Boi; verbatimElevation: 1759.8; decimalLatitude: 42.54958; decimalLongitude: 0.87254; geodeticDatum: WGS84; **Event:** eventID: 1; samplingProtocol: Beating; eventTime: Night
- **Type status:** Other material. **Occurrence:** individualCount: 2; sex: female; **Location:** locationID: A1; continent: Europe; country: Spain; countryCode: ES; stateProvince: Catalonia; county: Lleida; locality: Sola de Boi; verbatimElevation: 1759.8; decimalLatitude: 42.54958; decimalLongitude: 0.87254; geodeticDatum: WGS84; **Event:** eventID: 1; samplingProtocol: Sweeping; eventTime: Day
- **Type status:** Other material. **Occurrence:** individualCount: 2; sex: female; **Location:** locationID: A1; continent: Europe; country: Spain; countryCode: ES; stateProvince: Catalonia; county: Lleida; locality: Sola de Boi; verbatimElevation: 1759.8; decimalLatitude: 42.54958; decimalLongitude: 0.87254; geodeticDatum: WGS84; **Event:** eventID: 1; samplingProtocol: Sweeping; eventTime: Night
- **Type status:** Other material. **Occurrence:** individualCount: 1; sex: female; **Location:** locationID: A2; continent: Europe; country: Spain; countryCode: ES; stateProvince: Catalonia; county: Lleida; locality: Sola de Boi; verbatimElevation: 1738.7; decimalLatitude: 42.54913; decimalLongitude: 0.87137; geodeticDatum: WGS84; **Event:** eventID: 1; samplingProtocol: Aerial; eventTime: Night
- **Type status:** Other material. **Occurrence:** individualCount: 3; sex: female; **Location:** locationID: A2; continent: Europe; country: Spain; countryCode: ES; stateProvince: Catalonia; county: Lleida; locality: Sola de Boi; verbatimElevation: 1738.7; decimalLatitude: 42.54913; decimalLongitude: 0.87137; geodeticDatum: WGS84; **Event:** eventID: 1; samplingProtocol: Beating; eventTime: Day
- **Type status:** Other material. **Occurrence:** individualCount: 1; sex: male; **Location:** locationID: A2; continent: Europe; country: Spain; countryCode: ES; stateProvince: Catalonia; county: Lleida; locality: Sola de Boi; verbatimElevation: 1738.7; decimalLatitude: 42.54913; decimalLongitude: 0.87137; geodeticDatum: WGS84; **Event:** eventID: 1; samplingProtocol: Beating; eventTime: Day
- **Type status:** Other material. **Occurrence:** individualCount: 1; sex: male; **Location:** locationID: A2; continent: Europe; country: Spain; countryCode: ES; stateProvince: Catalonia; county: Lleida; locality: Sola de Boi; verbatimElevation: 1738.7; decimalLatitude: 42.54913; decimalLongitude: 0.87137; geodeticDatum: WGS84; **Event:** eventID: 2; samplingProtocol: Beating; eventTime: Day
- **Type status:** Other material. **Occurrence:** individualCount: 2; sex: female; **Location:** locationID: A2; continent: Europe; country: Spain; countryCode: ES; stateProvince: Catalonia; county: Lleida; locality: Sola de Boi; verbatimElevation: 1738.7; decimalLatitude: 42.54913; decimalLongitude: 0.87137; geodeticDatum: WGS84; **Event:** eventID: 1; samplingProtocol: Beating; eventTime: Night
- **Type status:** Other material. **Occurrence:** individualCount: 1; sex: female; **Location:** locationID: A2; continent: Europe; country: Spain; countryCode: ES; stateProvince: Catalonia; county: Lleida; locality: Sola de Boi; verbatimElevation: 1738.7; decimalLatitude: 42.54913; decimalLongitude: 0.87137; geodeticDatum: WGS84; **Event:** eventID: 1; samplingProtocol: Beating; eventTime: Night
- **Type status:** Other material. **Occurrence:** individualCount: 1; sex: female; **Location:** locationID: C1; continent: Europe; country: Spain; countryCode: ES; stateProvince: Castilla-La Mancha; county: Ciudad Real; locality: Valle Brezoso; verbatimElevation: 756.56; decimalLatitude: 39.35663; decimalLongitude: -4.35912; geodeticDatum: WGS84; **Event:** eventID: 1; samplingProtocol: Beating; eventTime: Day
- **Type status:** Other material. **Occurrence:** individualCount: 1; sex: female; **Location:** locationID: C1; continent: Europe; country: Spain; countryCode: ES; stateProvince: Castilla-La Mancha; county: Ciudad Real; locality: Valle Brezoso; verbatimElevation: 756.56; decimalLatitude: 39.35663; decimalLongitude: -4.35912; geodeticDatum: WGS84; **Event:** eventID: 2; samplingProtocol: Beating; eventTime: Day
- **Type status:** Other material. **Occurrence:** individualCount: 1; sex: female; **Location:** locationID: C1; continent: Europe; country: Spain; countryCode: ES; stateProvince: Castilla-La Mancha; county: Ciudad Real; locality: Valle Brezoso; verbatimElevation: 756.56; decimalLatitude: 39.35663; decimalLongitude: -4.35912; geodeticDatum: WGS84; **Event:** eventID: 2; samplingProtocol: Beating; eventTime: Night
- **Type status:** Other material. **Occurrence:** individualCount: 1; sex: female; **Location:** locationID: O1; continent: Europe; country: Spain; countryCode: ES; stateProvince: Aragón; county: Huesca; locality: O Furno; verbatimElevation: 1396.73; decimalLatitude: 42.60677; decimalLongitude: 0.13135; geodeticDatum: WGS84; **Event:** eventID: 1; samplingProtocol: Beating; eventTime: Day
- **Type status:** Other material. **Occurrence:** individualCount: 1; sex: male; **Location:** locationID: O1; continent: Europe; country: Spain; countryCode: ES; stateProvince: Aragón; county: Huesca; locality: O Furno; verbatimElevation: 1396.73; decimalLatitude: 42.60677; decimalLongitude: 0.13135; geodeticDatum: WGS84; **Event:** eventID: 1; samplingProtocol: Beating; eventTime: Day
- **Type status:** Other material. **Occurrence:** individualCount: 2; sex: female; **Location:** locationID: O2; continent: Europe; country: Spain; countryCode: ES; stateProvince: Aragón; county: Huesca; locality: Rebilla; verbatimElevation: 1158.13; decimalLatitude: 42.59427; decimalLongitude: 0.1529; geodeticDatum: WGS84; **Event:** eventID: 1; samplingProtocol: Beating; eventTime: Night
- **Type status:** Other material. **Occurrence:** individualCount: 2; sex: female; **Location:** locationID: P1; continent: Europe; country: Spain; countryCode: ES; stateProvince: Castilla y León; county: León; locality: Monte Robledo; verbatimElevation: 1071.58; decimalLatitude: 43.1445; decimalLongitude: -4.92675; geodeticDatum: WGS84; **Event:** eventID: 1; samplingProtocol: Beating; eventTime: Day
- **Type status:** Other material. **Occurrence:** individualCount: 1; sex: male; **Location:** locationID: P4; continent: Europe; country: Spain; countryCode: ES; stateProvince: Castilla y León; county: León; locality: El Canto; verbatimElevation: 943.48; decimalLatitude: 43.17227; decimalLongitude: -4.90857; geodeticDatum: WGS84; **Event:** eventID: 2; samplingProtocol: Aerial; eventTime: Night
- **Type status:** Other material. **Occurrence:** individualCount: 1; sex: female; **Location:** locationID: P4; continent: Europe; country: Spain; countryCode: ES; stateProvince: Castilla y León; county: León; locality: El Canto; verbatimElevation: 943.48; decimalLatitude: 43.17227; decimalLongitude: -4.90857; geodeticDatum: WGS84; **Event:** eventID: 1; samplingProtocol: Beating; eventTime: Day
- **Type status:** Other material. **Occurrence:** individualCount: 1; sex: female; **Location:** locationID: P4; continent: Europe; country: Spain; countryCode: ES; stateProvince: Castilla y León; county: León; locality: El Canto; verbatimElevation: 943.48; decimalLatitude: 43.17227; decimalLongitude: -4.90857; geodeticDatum: WGS84; **Event:** eventID: 1; samplingProtocol: Beating; eventTime: Night

##### Distribution

Europe

#### Philodromus buchari

Kubcová, 2004

##### Materials

- **Type status:** Other material. **Occurrence:** individualCount: 1; sex: female; **Location:** locationID: C1; continent: Europe; country: Spain; countryCode: ES; stateProvince: Castilla-La Mancha; county: Ciudad Real; locality: Valle Brezoso; verbatimElevation: 756.56; decimalLatitude: 39.35663; decimalLongitude: -4.35912; geodeticDatum: WGS84; **Event:** eventID: 2; samplingProtocol: Beating; eventTime: Night
- **Type status:** Other material. **Occurrence:** individualCount: 1; sex: female; **Location:** locationID: P4; continent: Europe; country: Spain; countryCode: ES; stateProvince: Castilla y León; county: León; locality: El Canto; verbatimElevation: 943.48; decimalLatitude: 43.17227; decimalLongitude: -4.90857; geodeticDatum: WGS84; **Event:** eventID: 1; samplingProtocol: Beating; eventTime: Day
- **Type status:** Other material. **Occurrence:** individualCount: 1; sex: male; **Location:** locationID: S1; continent: Europe; country: Spain; countryCode: ES; stateProvince: Andalucía; county: Granada; locality: Soportujar; verbatimElevation: 1786.57; decimalLatitude: 36.96151; decimalLongitude: -3.41881; geodeticDatum: WGS84; **Event:** eventID: 1; samplingProtocol: Beating; eventTime: Day
- **Type status:** Other material. **Occurrence:** individualCount: 2; sex: female; **Location:** locationID: S1; continent: Europe; country: Spain; countryCode: ES; stateProvince: Andalucía; county: Granada; locality: Soportujar; verbatimElevation: 1786.57; decimalLatitude: 36.96151; decimalLongitude: -3.41881; geodeticDatum: WGS84; **Event:** eventID: 1; samplingProtocol: Beating; eventTime: Day
- **Type status:** Other material. **Occurrence:** individualCount: 1; sex: male; **Location:** locationID: S2; continent: Europe; country: Spain; countryCode: ES; stateProvince: Andalucía; county: Granada; locality: Camarate; verbatimElevation: 1713.96; decimalLatitude: 37.18377; decimalLongitude: -3.26282; geodeticDatum: WGS84; **Event:** eventID: 1; samplingProtocol: Sweeping; eventTime: Day
- **Type status:** Other material. **Occurrence:** individualCount: 1; sex: female; **Location:** locationID: S2; continent: Europe; country: Spain; countryCode: ES; stateProvince: Andalucía; county: Granada; locality: Camarate; verbatimElevation: 1713.96; decimalLatitude: 37.18377; decimalLongitude: -3.26282; geodeticDatum: WGS84; **Event:** eventID: 1; samplingProtocol: Sweeping; eventTime: Day

##### Distribution

Europe

##### Notes

**Figure 9.**
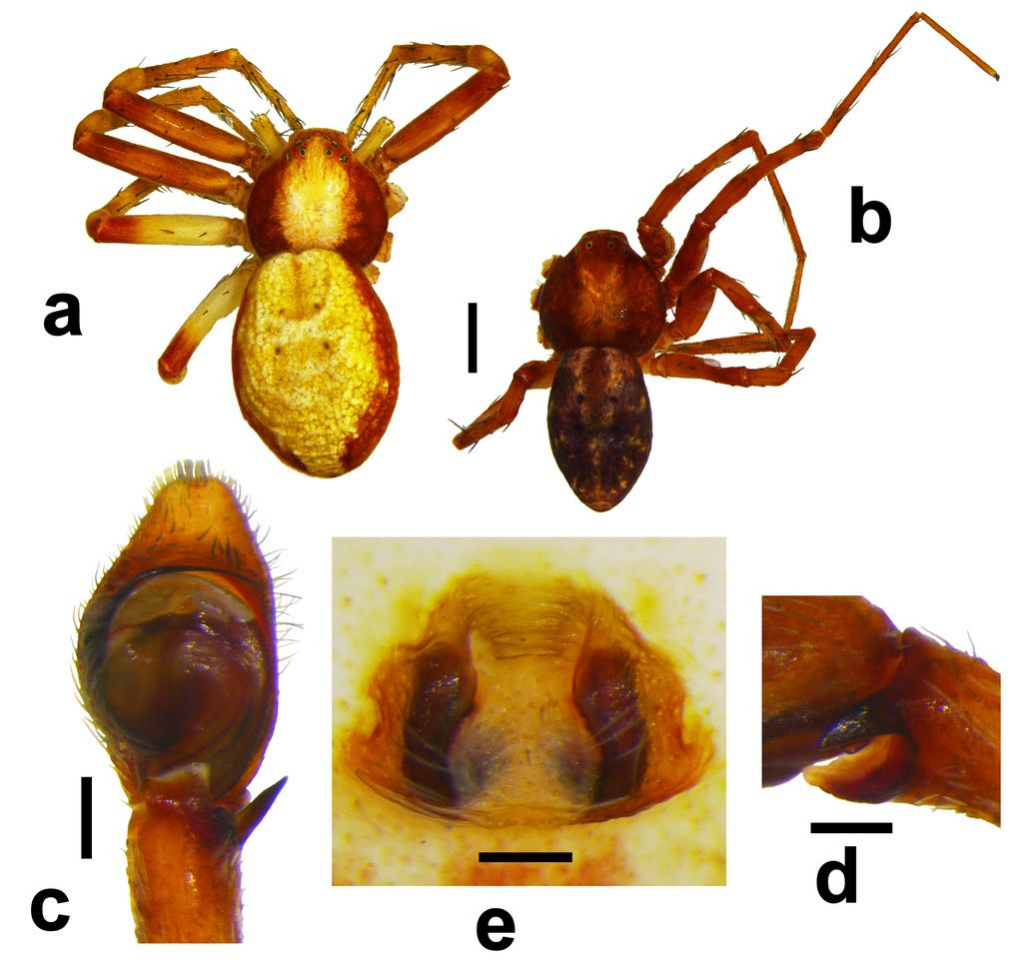
*Philodromus buchari* Kubcová, 2004. a: female, dorsal habitus, scale = 1 mm; b: male, dorsal habitus, scale = 1 mm; c: left male palp, ventral, scale = 0.2 mm; d: left male tibial apophysis, retrolateral, scale = 0.2 mm; e: female epigynum, ventral, scale = 0.2 mm.

First record for the Iberian Peninsula. See Fig. 9.

#### Philodromus buxi

Simon, 1884

##### Materials

- **Type status:** Other material. **Occurrence:** individualCount: 1; sex: female; **Location:** locationID: C1; continent: Europe; country: Spain; countryCode: ES; stateProvince: Castilla-La Mancha; county: Ciudad Real; locality: Valle Brezoso; verbatimElevation: 756.56; decimalLatitude: 39.35663; decimalLongitude: -4.35912; geodeticDatum: WGS84; **Event:** eventID: 2; samplingProtocol: Beating; eventTime: Night
- **Type status:** Other material. **Occurrence:** individualCount: 1; sex: female; **Location:** locationID: C2; continent: Europe; country: Spain; countryCode: ES; stateProvince: Castilla-La Mancha; county: Ciudad Real; locality: Valle Brezoso; verbatimElevation: 739.31; decimalLatitude: 39.35159; decimalLongitude: -4.3589; geodeticDatum: WGS84; **Event:** eventID: 4; samplingProtocol: Aerial; eventTime: Night
- **Type status:** Other material. **Occurrence:** individualCount: 1; sex: female; **Location:** locationID: C4; continent: Europe; country: Spain; countryCode: ES; stateProvince: Castilla-La Mancha; county: Ciudad Real; locality: La Quesera; verbatimElevation: 772.3; decimalLatitude: 39.36337; decimalLongitude: -4.41704; geodeticDatum: WGS84; **Event:** eventID: 1; samplingProtocol: Beating; eventTime: Night
- **Type status:** Other material. **Occurrence:** individualCount: 1; sex: male; **Location:** locationID: M2; continent: Europe; country: Spain; countryCode: ES; stateProvince: Extremadura; county: Cáceres; locality: Fuente del Frances; verbatimElevation: 320.72; decimalLatitude: 39.828; decimalLongitude: -6.03249; geodeticDatum: WGS84; **Event:** eventID: G; samplingProtocol: Pitfall
- **Type status:** Other material. **Occurrence:** individualCount: 1; sex: female; **Location:** locationID: S1; continent: Europe; country: Spain; countryCode: ES; stateProvince: Andalucía; county: Granada; locality: Soportujar; verbatimElevation: 1786.57; decimalLatitude: 36.96151; decimalLongitude: -3.41881; geodeticDatum: WGS84; **Event:** eventID: 1; samplingProtocol: Beating; eventTime: Day
- **Type status:** Other material. **Occurrence:** individualCount: 2; sex: female; **Location:** locationID: S1; continent: Europe; country: Spain; countryCode: ES; stateProvince: Andalucía; county: Granada; locality: Soportujar; verbatimElevation: 1786.57; decimalLatitude: 36.96151; decimalLongitude: -3.41881; geodeticDatum: WGS84; **Event:** eventID: 2; samplingProtocol: Beating; eventTime: Day
- **Type status:** Other material. **Occurrence:** individualCount: 1; sex: male; **Location:** locationID: S1; continent: Europe; country: Spain; countryCode: ES; stateProvince: Andalucía; county: Granada; locality: Soportujar; verbatimElevation: 1786.57; decimalLatitude: 36.96151; decimalLongitude: -3.41881; geodeticDatum: WGS84; **Event:** eventID: 1; samplingProtocol: Beating; eventTime: Night
- **Type status:** Other material. **Occurrence:** individualCount: 1; sex: female; **Location:** locationID: S1; continent: Europe; country: Spain; countryCode: ES; stateProvince: Andalucía; county: Granada; locality: Soportujar; verbatimElevation: 1786.57; decimalLatitude: 36.96151; decimalLongitude: -3.41881; geodeticDatum: WGS84; **Event:** eventID: 1; samplingProtocol: Sweeping; eventTime: Night
- **Type status:** Other material. **Occurrence:** individualCount: 1; sex: female; **Location:** locationID: S2; continent: Europe; country: Spain; countryCode: ES; stateProvince: Andalucía; county: Granada; locality: Camarate; verbatimElevation: 1713.96; decimalLatitude: 37.18377; decimalLongitude: -3.26282; geodeticDatum: WGS84; **Event:** eventID: 1; samplingProtocol: Beating; eventTime: Day
- **Type status:** Other material. **Occurrence:** individualCount: 1; sex: female; **Location:** locationID: S2; continent: Europe; country: Spain; countryCode: ES; stateProvince: Andalucía; county: Granada; locality: Camarate; verbatimElevation: 1713.96; decimalLatitude: 37.18377; decimalLongitude: -3.26282; geodeticDatum: WGS84; **Event:** eventID: 2; samplingProtocol: Beating; eventTime: Day

##### Distribution

Europe to Kazakhstan

#### Philodromus dispar

Walckenaer, 1826

##### Materials

- **Type status:** Other material. **Occurrence:** individualCount: 2; sex: female; **Location:** locationID: A1; continent: Europe; country: Spain; countryCode: ES; stateProvince: Catalonia; county: Lleida; locality: Sola de Boi; verbatimElevation: 1759.8; decimalLatitude: 42.54958; decimalLongitude: 0.87254; geodeticDatum: WGS84; **Event:** eventID: 2; samplingProtocol: Beating; eventTime: Day
- **Type status:** Other material. **Occurrence:** individualCount: 1; sex: female; **Location:** locationID: A1; continent: Europe; country: Spain; countryCode: ES; stateProvince: Catalonia; county: Lleida; locality: Sola de Boi; verbatimElevation: 1759.8; decimalLatitude: 42.54958; decimalLongitude: 0.87254; geodeticDatum: WGS84; **Event:** eventID: 1; samplingProtocol: Beating; eventTime: Night
- **Type status:** Other material. **Occurrence:** individualCount: 1; sex: male; **Location:** locationID: A1; continent: Europe; country: Spain; countryCode: ES; stateProvince: Catalonia; county: Lleida; locality: Sola de Boi; verbatimElevation: 1759.8; decimalLatitude: 42.54958; decimalLongitude: 0.87254; geodeticDatum: WGS84; **Event:** eventID: 2; samplingProtocol: Beating; eventTime: Night
- **Type status:** Other material. **Occurrence:** individualCount: 1; sex: female; **Location:** locationID: A1; continent: Europe; country: Spain; countryCode: ES; stateProvince: Catalonia; county: Lleida; locality: Sola de Boi; verbatimElevation: 1759.8; decimalLatitude: 42.54958; decimalLongitude: 0.87254; geodeticDatum: WGS84; **Event:** eventID: 2; samplingProtocol: Beating; eventTime: Night
- **Type status:** Other material. **Occurrence:** individualCount: 1; sex: female; **Location:** locationID: A1; continent: Europe; country: Spain; countryCode: ES; stateProvince: Catalonia; county: Lleida; locality: Sola de Boi; verbatimElevation: 1759.8; decimalLatitude: 42.54958; decimalLongitude: 0.87254; geodeticDatum: WGS84; **Event:** eventID: 2; samplingProtocol: Sweeping; eventTime: Day
- **Type status:** Other material. **Occurrence:** individualCount: 2; sex: male; **Location:** locationID: A1; continent: Europe; country: Spain; countryCode: ES; stateProvince: Catalonia; county: Lleida; locality: Sola de Boi; verbatimElevation: 1759.8; decimalLatitude: 42.54958; decimalLongitude: 0.87254; geodeticDatum: WGS84; **Event:** eventID: 2; samplingProtocol: Sweeping; eventTime: Night
- **Type status:** Other material. **Occurrence:** individualCount: 1; sex: male; **Location:** locationID: A2; continent: Europe; country: Spain; countryCode: ES; stateProvince: Catalonia; county: Lleida; locality: Sola de Boi; verbatimElevation: 1738.7; decimalLatitude: 42.54913; decimalLongitude: 0.87137; geodeticDatum: WGS84; **Event:** eventID: 1; samplingProtocol: Aerial; eventTime: Night
- **Type status:** Other material. **Occurrence:** individualCount: 1; sex: male; **Location:** locationID: A2; continent: Europe; country: Spain; countryCode: ES; stateProvince: Catalonia; county: Lleida; locality: Sola de Boi; verbatimElevation: 1738.7; decimalLatitude: 42.54913; decimalLongitude: 0.87137; geodeticDatum: WGS84; **Event:** eventID: 1; samplingProtocol: Beating; eventTime: Day
- **Type status:** Other material. **Occurrence:** individualCount: 1; sex: female; **Location:** locationID: A2; continent: Europe; country: Spain; countryCode: ES; stateProvince: Catalonia; county: Lleida; locality: Sola de Boi; verbatimElevation: 1738.7; decimalLatitude: 42.54913; decimalLongitude: 0.87137; geodeticDatum: WGS84; **Event:** eventID: 2; samplingProtocol: Beating; eventTime: Day
- **Type status:** Other material. **Occurrence:** individualCount: 2; sex: male; **Location:** locationID: A2; continent: Europe; country: Spain; countryCode: ES; stateProvince: Catalonia; county: Lleida; locality: Sola de Boi; verbatimElevation: 1738.7; decimalLatitude: 42.54913; decimalLongitude: 0.87137; geodeticDatum: WGS84; **Event:** eventID: 2; samplingProtocol: Beating; eventTime: Night
- **Type status:** Other material. **Occurrence:** individualCount: 1; sex: female; **Location:** locationID: A2; continent: Europe; country: Spain; countryCode: ES; stateProvince: Catalonia; county: Lleida; locality: Sola de Boi; verbatimElevation: 1738.7; decimalLatitude: 42.54913; decimalLongitude: 0.87137; geodeticDatum: WGS84; **Event:** eventID: 1; samplingProtocol: Sweeping; eventTime: Day
- **Type status:** Other material. **Occurrence:** individualCount: 1; sex: male; **Location:** locationID: A2; continent: Europe; country: Spain; countryCode: ES; stateProvince: Catalonia; county: Lleida; locality: Sola de Boi; verbatimElevation: 1738.7; decimalLatitude: 42.54913; decimalLongitude: 0.87137; geodeticDatum: WGS84; **Event:** eventID: 2; samplingProtocol: Sweeping; eventTime: Day
- **Type status:** Other material. **Occurrence:** individualCount: 2; sex: male; **Location:** locationID: A2; continent: Europe; country: Spain; countryCode: ES; stateProvince: Catalonia; county: Lleida; locality: Sola de Boi; verbatimElevation: 1738.7; decimalLatitude: 42.54913; decimalLongitude: 0.87137; geodeticDatum: WGS84; **Event:** eventID: 1; samplingProtocol: Sweeping; eventTime: Night
- **Type status:** Other material. **Occurrence:** individualCount: 3; sex: female; **Location:** locationID: A2; continent: Europe; country: Spain; countryCode: ES; stateProvince: Catalonia; county: Lleida; locality: Sola de Boi; verbatimElevation: 1738.7; decimalLatitude: 42.54913; decimalLongitude: 0.87137; geodeticDatum: WGS84; **Event:** eventID: 1; samplingProtocol: Sweeping; eventTime: Night
- **Type status:** Other material. **Occurrence:** individualCount: 2; sex: male; **Location:** locationID: A2; continent: Europe; country: Spain; countryCode: ES; stateProvince: Catalonia; county: Lleida; locality: Sola de Boi; verbatimElevation: 1738.7; decimalLatitude: 42.54913; decimalLongitude: 0.87137; geodeticDatum: WGS84; **Event:** eventID: 2; samplingProtocol: Sweeping; eventTime: Night
- **Type status:** Other material. **Occurrence:** individualCount: 4; sex: female; **Location:** locationID: A2; continent: Europe; country: Spain; countryCode: ES; stateProvince: Catalonia; county: Lleida; locality: Sola de Boi; verbatimElevation: 1738.7; decimalLatitude: 42.54913; decimalLongitude: 0.87137; geodeticDatum: WGS84; **Event:** eventID: 2; samplingProtocol: Sweeping; eventTime: Night
- **Type status:** Other material. **Occurrence:** individualCount: 1; sex: female; **Location:** locationID: O1; continent: Europe; country: Spain; countryCode: ES; stateProvince: Aragón; county: Huesca; locality: O Furno; verbatimElevation: 1396.73; decimalLatitude: 42.60677; decimalLongitude: 0.13135; geodeticDatum: WGS84; **Event:** eventID: 1; samplingProtocol: Aerial; eventTime: Night
- **Type status:** Other material. **Occurrence:** individualCount: 1; sex: female; **Location:** locationID: O1; continent: Europe; country: Spain; countryCode: ES; stateProvince: Aragón; county: Huesca; locality: O Furno; verbatimElevation: 1396.73; decimalLatitude: 42.60677; decimalLongitude: 0.13135; geodeticDatum: WGS84; **Event:** eventID: 1; samplingProtocol: Beating; eventTime: Day
- **Type status:** Other material. **Occurrence:** individualCount: 1; sex: female; **Location:** locationID: O1; continent: Europe; country: Spain; countryCode: ES; stateProvince: Aragón; county: Huesca; locality: O Furno; verbatimElevation: 1396.73; decimalLatitude: 42.60677; decimalLongitude: 0.13135; geodeticDatum: WGS84; **Event:** eventID: 1; samplingProtocol: Sweeping; eventTime: Night
- **Type status:** Other material. **Occurrence:** individualCount: 1; sex: male; **Location:** locationID: O1; continent: Europe; country: Spain; countryCode: ES; stateProvince: Aragón; county: Huesca; locality: O Furno; verbatimElevation: 1396.73; decimalLatitude: 42.60677; decimalLongitude: 0.13135; geodeticDatum: WGS84; **Event:** eventID: 2; samplingProtocol: Sweeping; eventTime: Night
- **Type status:** Other material. **Occurrence:** individualCount: 1; sex: female; **Location:** locationID: O2; continent: Europe; country: Spain; countryCode: ES; stateProvince: Aragón; county: Huesca; locality: Rebilla; verbatimElevation: 1158.13; decimalLatitude: 42.59427; decimalLongitude: 0.1529; geodeticDatum: WGS84; **Event:** eventID: 1; samplingProtocol: Beating; eventTime: Day
- **Type status:** Other material. **Occurrence:** individualCount: 1; sex: female; **Location:** locationID: O2; continent: Europe; country: Spain; countryCode: ES; stateProvince: Aragón; county: Huesca; locality: Rebilla; verbatimElevation: 1158.13; decimalLatitude: 42.59427; decimalLongitude: 0.1529; geodeticDatum: WGS84; **Event:** eventID: 2; samplingProtocol: Beating; eventTime: Day
- **Type status:** Other material. **Occurrence:** individualCount: 1; sex: male; **Location:** locationID: O2; continent: Europe; country: Spain; countryCode: ES; stateProvince: Aragón; county: Huesca; locality: Rebilla; verbatimElevation: 1158.13; decimalLatitude: 42.59427; decimalLongitude: 0.1529; geodeticDatum: WGS84; **Event:** eventID: 2; samplingProtocol: Beating; eventTime: Night
- **Type status:** Other material. **Occurrence:** individualCount: 1; sex: female; **Location:** locationID: O2; continent: Europe; country: Spain; countryCode: ES; stateProvince: Aragón; county: Huesca; locality: Rebilla; verbatimElevation: 1158.13; decimalLatitude: 42.59427; decimalLongitude: 0.1529; geodeticDatum: WGS84; **Event:** eventID: 1; samplingProtocol: Sweeping; eventTime: Night
- **Type status:** Other material. **Occurrence:** individualCount: 1; sex: female; **Location:** locationID: P1; continent: Europe; country: Spain; countryCode: ES; stateProvince: Castilla y León; county: León; locality: Monte Robledo; verbatimElevation: 1071.58; decimalLatitude: 43.1445; decimalLongitude: -4.92675; geodeticDatum: WGS84; **Event:** eventID: 1; samplingProtocol: Beating; eventTime: Day
- **Type status:** Other material. **Occurrence:** individualCount: 2; sex: female; **Location:** locationID: P1; continent: Europe; country: Spain; countryCode: ES; stateProvince: Castilla y León; county: León; locality: Monte Robledo; verbatimElevation: 1071.58; decimalLatitude: 43.1445; decimalLongitude: -4.92675; geodeticDatum: WGS84; **Event:** eventID: 2; samplingProtocol: Beating; eventTime: Day
- **Type status:** Other material. **Occurrence:** individualCount: 2; sex: male; **Location:** locationID: P1; continent: Europe; country: Spain; countryCode: ES; stateProvince: Castilla y León; county: León; locality: Monte Robledo; verbatimElevation: 1071.58; decimalLatitude: 43.1445; decimalLongitude: -4.92675; geodeticDatum: WGS84; **Event:** eventID: 1; samplingProtocol: Beating; eventTime: Night
- **Type status:** Other material. **Occurrence:** individualCount: 4; sex: female; **Location:** locationID: P1; continent: Europe; country: Spain; countryCode: ES; stateProvince: Castilla y León; county: León; locality: Monte Robledo; verbatimElevation: 1071.58; decimalLatitude: 43.1445; decimalLongitude: -4.92675; geodeticDatum: WGS84; **Event:** eventID: 1; samplingProtocol: Beating; eventTime: Night
- **Type status:** Other material. **Occurrence:** individualCount: 3; sex: male; **Location:** locationID: P1; continent: Europe; country: Spain; countryCode: ES; stateProvince: Castilla y León; county: León; locality: Monte Robledo; verbatimElevation: 1071.58; decimalLatitude: 43.1445; decimalLongitude: -4.92675; geodeticDatum: WGS84; **Event:** eventID: 2; samplingProtocol: Beating; eventTime: Night
- **Type status:** Other material. **Occurrence:** individualCount: 4; sex: female; **Location:** locationID: P1; continent: Europe; country: Spain; countryCode: ES; stateProvince: Castilla y León; county: León; locality: Monte Robledo; verbatimElevation: 1071.58; decimalLatitude: 43.1445; decimalLongitude: -4.92675; geodeticDatum: WGS84; **Event:** eventID: 2; samplingProtocol: Beating; eventTime: Night
- **Type status:** Other material. **Occurrence:** individualCount: 1; sex: female; **Location:** locationID: P1; continent: Europe; country: Spain; countryCode: ES; stateProvince: Castilla y León; county: León; locality: Monte Robledo; verbatimElevation: 1071.58; decimalLatitude: 43.1445; decimalLongitude: -4.92675; geodeticDatum: WGS84; **Event:** eventID: 2; samplingProtocol: Ground; eventTime: Day
- **Type status:** Other material. **Occurrence:** individualCount: 1; sex: male; **Location:** locationID: P1; continent: Europe; country: Spain; countryCode: ES; stateProvince: Castilla y León; county: León; locality: Monte Robledo; verbatimElevation: 1071.58; decimalLatitude: 43.1445; decimalLongitude: -4.92675; geodeticDatum: WGS84; **Event:** eventID: A; samplingProtocol: Pitfall
- **Type status:** Other material. **Occurrence:** individualCount: 1; sex: female; **Location:** locationID: P1; continent: Europe; country: Spain; countryCode: ES; stateProvince: Castilla y León; county: León; locality: Monte Robledo; verbatimElevation: 1071.58; decimalLatitude: 43.1445; decimalLongitude: -4.92675; geodeticDatum: WGS84; **Event:** eventID: 1; samplingProtocol: Sweeping; eventTime: Day
- **Type status:** Other material. **Occurrence:** individualCount: 2; sex: male; **Location:** locationID: P1; continent: Europe; country: Spain; countryCode: ES; stateProvince: Castilla y León; county: León; locality: Monte Robledo; verbatimElevation: 1071.58; decimalLatitude: 43.1445; decimalLongitude: -4.92675; geodeticDatum: WGS84; **Event:** eventID: 2; samplingProtocol: Sweeping; eventTime: Day
- **Type status:** Other material. **Occurrence:** individualCount: 3; sex: female; **Location:** locationID: P1; continent: Europe; country: Spain; countryCode: ES; stateProvince: Castilla y León; county: León; locality: Monte Robledo; verbatimElevation: 1071.58; decimalLatitude: 43.1445; decimalLongitude: -4.92675; geodeticDatum: WGS84; **Event:** eventID: 2; samplingProtocol: Sweeping; eventTime: Day
- **Type status:** Other material. **Occurrence:** individualCount: 1; sex: male; **Location:** locationID: P1; continent: Europe; country: Spain; countryCode: ES; stateProvince: Castilla y León; county: León; locality: Monte Robledo; verbatimElevation: 1071.58; decimalLatitude: 43.1445; decimalLongitude: -4.92675; geodeticDatum: WGS84; **Event:** eventID: 1; samplingProtocol: Sweeping; eventTime: Night
- **Type status:** Other material. **Occurrence:** individualCount: 2; sex: male; **Location:** locationID: P1; continent: Europe; country: Spain; countryCode: ES; stateProvince: Castilla y León; county: León; locality: Monte Robledo; verbatimElevation: 1071.58; decimalLatitude: 43.1445; decimalLongitude: -4.92675; geodeticDatum: WGS84; **Event:** eventID: 2; samplingProtocol: Sweeping; eventTime: Night
- **Type status:** Other material. **Occurrence:** individualCount: 6; sex: female; **Location:** locationID: P1; continent: Europe; country: Spain; countryCode: ES; stateProvince: Castilla y León; county: León; locality: Monte Robledo; verbatimElevation: 1071.58; decimalLatitude: 43.1445; decimalLongitude: -4.92675; geodeticDatum: WGS84; **Event:** eventID: 2; samplingProtocol: Sweeping; eventTime: Night
- **Type status:** Other material. **Occurrence:** individualCount: 2; sex: female; **Location:** locationID: P2; continent: Europe; country: Spain; countryCode: ES; stateProvince: Castilla y León; county: León; locality: Joyoguelas; verbatimElevation: 763.98; decimalLatitude: 43.17771; decimalLongitude: -4.90579; geodeticDatum: WGS84; **Event:** eventID: 1; samplingProtocol: Beating; eventTime: Day
- **Type status:** Other material. **Occurrence:** individualCount: 1; sex: female; **Location:** locationID: P2; continent: Europe; country: Spain; countryCode: ES; stateProvince: Castilla y León; county: León; locality: Joyoguelas; verbatimElevation: 763.98; decimalLatitude: 43.17771; decimalLongitude: -4.90579; geodeticDatum: WGS84; **Event:** eventID: 1; samplingProtocol: Beating; eventTime: Night
- **Type status:** Other material. **Occurrence:** individualCount: 1; sex: male; **Location:** locationID: P2; continent: Europe; country: Spain; countryCode: ES; stateProvince: Castilla y León; county: León; locality: Joyoguelas; verbatimElevation: 763.98; decimalLatitude: 43.17771; decimalLongitude: -4.90579; geodeticDatum: WGS84; **Event:** eventID: 1; samplingProtocol: Sweeping; eventTime: Day
- **Type status:** Other material. **Occurrence:** individualCount: 1; sex: female; **Location:** locationID: P2; continent: Europe; country: Spain; countryCode: ES; stateProvince: Castilla y León; county: León; locality: Joyoguelas; verbatimElevation: 763.98; decimalLatitude: 43.17771; decimalLongitude: -4.90579; geodeticDatum: WGS84; **Event:** eventID: 1; samplingProtocol: Sweeping; eventTime: Day
- **Type status:** Other material. **Occurrence:** individualCount: 1; sex: female; **Location:** locationID: P2; continent: Europe; country: Spain; countryCode: ES; stateProvince: Castilla y León; county: León; locality: Joyoguelas; verbatimElevation: 763.98; decimalLatitude: 43.17771; decimalLongitude: -4.90579; geodeticDatum: WGS84; **Event:** eventID: 2; samplingProtocol: Sweeping; eventTime: Day
- **Type status:** Other material. **Occurrence:** individualCount: 1; sex: female; **Location:** locationID: P2; continent: Europe; country: Spain; countryCode: ES; stateProvince: Castilla y León; county: León; locality: Joyoguelas; verbatimElevation: 763.98; decimalLatitude: 43.17771; decimalLongitude: -4.90579; geodeticDatum: WGS84; **Event:** eventID: 1; samplingProtocol: Sweeping; eventTime: Night
- **Type status:** Other material. **Occurrence:** individualCount: 1; sex: male; **Location:** locationID: P2; continent: Europe; country: Spain; countryCode: ES; stateProvince: Castilla y León; county: León; locality: Joyoguelas; verbatimElevation: 763.98; decimalLatitude: 43.17771; decimalLongitude: -4.90579; geodeticDatum: WGS84; **Event:** eventID: 2; samplingProtocol: Sweeping; eventTime: Night
- **Type status:** Other material. **Occurrence:** individualCount: 3; sex: female; **Location:** locationID: P2; continent: Europe; country: Spain; countryCode: ES; stateProvince: Castilla y León; county: León; locality: Joyoguelas; verbatimElevation: 763.98; decimalLatitude: 43.17771; decimalLongitude: -4.90579; geodeticDatum: WGS84; **Event:** eventID: 2; samplingProtocol: Sweeping; eventTime: Night
- **Type status:** Other material. **Occurrence:** individualCount: 2; sex: male; **Location:** locationID: P3; continent: Europe; country: Spain; countryCode: ES; stateProvince: Castilla y León; county: León; locality: Las Arroyas; verbatimElevation: 1097.1; decimalLatitude: 43.14351; decimalLongitude: -4.94878; geodeticDatum: WGS84; **Event:** eventID: 1; samplingProtocol: Beating; eventTime: Day
- **Type status:** Other material. **Occurrence:** individualCount: 5; sex: female; **Location:** locationID: P3; continent: Europe; country: Spain; countryCode: ES; stateProvince: Castilla y León; county: León; locality: Las Arroyas; verbatimElevation: 1097.1; decimalLatitude: 43.14351; decimalLongitude: -4.94878; geodeticDatum: WGS84; **Event:** eventID: 1; samplingProtocol: Beating; eventTime: Day
- **Type status:** Other material. **Occurrence:** individualCount: 1; sex: female; **Location:** locationID: P3; continent: Europe; country: Spain; countryCode: ES; stateProvince: Castilla y León; county: León; locality: Las Arroyas; verbatimElevation: 1097.1; decimalLatitude: 43.14351; decimalLongitude: -4.94878; geodeticDatum: WGS84; **Event:** eventID: 1; samplingProtocol: Beating; eventTime: Night
- **Type status:** Other material. **Occurrence:** individualCount: 1; sex: male; **Location:** locationID: P3; continent: Europe; country: Spain; countryCode: ES; stateProvince: Castilla y León; county: León; locality: Las Arroyas; verbatimElevation: 1097.1; decimalLatitude: 43.14351; decimalLongitude: -4.94878; geodeticDatum: WGS84; **Event:** eventID: 2; samplingProtocol: Beating; eventTime: Night
- **Type status:** Other material. **Occurrence:** individualCount: 1; sex: female; **Location:** locationID: P3; continent: Europe; country: Spain; countryCode: ES; stateProvince: Castilla y León; county: León; locality: Las Arroyas; verbatimElevation: 1097.1; decimalLatitude: 43.14351; decimalLongitude: -4.94878; geodeticDatum: WGS84; **Event:** eventID: 2; samplingProtocol: Beating; eventTime: Night
- **Type status:** Other material. **Occurrence:** individualCount: 2; sex: female; **Location:** locationID: P3; continent: Europe; country: Spain; countryCode: ES; stateProvince: Castilla y León; county: León; locality: Las Arroyas; verbatimElevation: 1097.1; decimalLatitude: 43.14351; decimalLongitude: -4.94878; geodeticDatum: WGS84; **Event:** eventID: 2; samplingProtocol: Sweeping; eventTime: Day
- **Type status:** Other material. **Occurrence:** individualCount: 2; sex: male; **Location:** locationID: P3; continent: Europe; country: Spain; countryCode: ES; stateProvince: Castilla y León; county: León; locality: Las Arroyas; verbatimElevation: 1097.1; decimalLatitude: 43.14351; decimalLongitude: -4.94878; geodeticDatum: WGS84; **Event:** eventID: 1; samplingProtocol: Sweeping; eventTime: Night
- **Type status:** Other material. **Occurrence:** individualCount: 1; sex: female; **Location:** locationID: P3; continent: Europe; country: Spain; countryCode: ES; stateProvince: Castilla y León; county: León; locality: Las Arroyas; verbatimElevation: 1097.1; decimalLatitude: 43.14351; decimalLongitude: -4.94878; geodeticDatum: WGS84; **Event:** eventID: 1; samplingProtocol: Sweeping; eventTime: Night
- **Type status:** Other material. **Occurrence:** individualCount: 2; sex: female; **Location:** locationID: P3; continent: Europe; country: Spain; countryCode: ES; stateProvince: Castilla y León; county: León; locality: Las Arroyas; verbatimElevation: 1097.1; decimalLatitude: 43.14351; decimalLongitude: -4.94878; geodeticDatum: WGS84; **Event:** eventID: 2; samplingProtocol: Sweeping; eventTime: Night
- **Type status:** Other material. **Occurrence:** individualCount: 1; sex: female; **Location:** locationID: P4; continent: Europe; country: Spain; countryCode: ES; stateProvince: Castilla y León; county: León; locality: El Canto; verbatimElevation: 943.48; decimalLatitude: 43.17227; decimalLongitude: -4.90857; geodeticDatum: WGS84; **Event:** eventID: 1; samplingProtocol: Beating; eventTime: Day
- **Type status:** Other material. **Occurrence:** individualCount: 1; sex: female; **Location:** locationID: P4; continent: Europe; country: Spain; countryCode: ES; stateProvince: Castilla y León; county: León; locality: El Canto; verbatimElevation: 943.48; decimalLatitude: 43.17227; decimalLongitude: -4.90857; geodeticDatum: WGS84; **Event:** eventID: 2; samplingProtocol: Beating; eventTime: Day
- **Type status:** Other material. **Occurrence:** individualCount: 2; sex: male; **Location:** locationID: P4; continent: Europe; country: Spain; countryCode: ES; stateProvince: Castilla y León; county: León; locality: El Canto; verbatimElevation: 943.48; decimalLatitude: 43.17227; decimalLongitude: -4.90857; geodeticDatum: WGS84; **Event:** eventID: 1; samplingProtocol: Beating; eventTime: Night
- **Type status:** Other material. **Occurrence:** individualCount: 1; sex: female; **Location:** locationID: P4; continent: Europe; country: Spain; countryCode: ES; stateProvince: Castilla y León; county: León; locality: El Canto; verbatimElevation: 943.48; decimalLatitude: 43.17227; decimalLongitude: -4.90857; geodeticDatum: WGS84; **Event:** eventID: 1; samplingProtocol: Beating; eventTime: Night
- **Type status:** Other material. **Occurrence:** individualCount: 1; sex: female; **Location:** locationID: P4; continent: Europe; country: Spain; countryCode: ES; stateProvince: Castilla y León; county: León; locality: El Canto; verbatimElevation: 943.48; decimalLatitude: 43.17227; decimalLongitude: -4.90857; geodeticDatum: WGS84; **Event:** eventID: 1; samplingProtocol: Sweeping; eventTime: Day
- **Type status:** Other material. **Occurrence:** individualCount: 1; sex: female; **Location:** locationID: P4; continent: Europe; country: Spain; countryCode: ES; stateProvince: Castilla y León; county: León; locality: El Canto; verbatimElevation: 943.48; decimalLatitude: 43.17227; decimalLongitude: -4.90857; geodeticDatum: WGS84; **Event:** eventID: 1; samplingProtocol: Sweeping; eventTime: Night
- **Type status:** Other material. **Occurrence:** individualCount: 1; sex: female; **Location:** locationID: S2; continent: Europe; country: Spain; countryCode: ES; stateProvince: Andalucía; county: Granada; locality: Camarate; verbatimElevation: 1713.96; decimalLatitude: 37.18377; decimalLongitude: -3.26282; geodeticDatum: WGS84; **Event:** eventID: 1; samplingProtocol: Sweeping; eventTime: Night

##### Distribution

Europe to Central Asia (USA, Canada, introduced)

#### Philodromus emarginatus

(Schrank, 1803)

##### Materials

- **Type status:** Other material. **Occurrence:** individualCount: 1; sex: female; **Location:** locationID: C1; continent: Europe; country: Spain; countryCode: ES; stateProvince: Castilla-La Mancha; county: Ciudad Real; locality: Valle Brezoso; verbatimElevation: 756.56; decimalLatitude: 39.35663; decimalLongitude: -4.35912; geodeticDatum: WGS84; **Event:** eventID: 2; samplingProtocol: Beating; eventTime: Night
- **Type status:** Other material. **Occurrence:** individualCount: 1; sex: female; **Location:** locationID: C1; continent: Europe; country: Spain; countryCode: ES; stateProvince: Castilla-La Mancha; county: Ciudad Real; locality: Valle Brezoso; verbatimElevation: 756.56; decimalLatitude: 39.35663; decimalLongitude: -4.35912; geodeticDatum: WGS84; **Event:** eventID: 1; samplingProtocol: Sweeping; eventTime: Day
- **Type status:** Other material. **Occurrence:** individualCount: 1; sex: male; **Location:** locationID: C1; continent: Europe; country: Spain; countryCode: ES; stateProvince: Castilla-La Mancha; county: Ciudad Real; locality: Valle Brezoso; verbatimElevation: 756.56; decimalLatitude: 39.35663; decimalLongitude: -4.35912; geodeticDatum: WGS84; **Event:** eventID: 2; samplingProtocol: Sweeping; eventTime: Day
- **Type status:** Other material. **Occurrence:** individualCount: 1; sex: female; **Location:** locationID: C1; continent: Europe; country: Spain; countryCode: ES; stateProvince: Castilla-La Mancha; county: Ciudad Real; locality: Valle Brezoso; verbatimElevation: 756.56; decimalLatitude: 39.35663; decimalLongitude: -4.35912; geodeticDatum: WGS84; **Event:** eventID: 2; samplingProtocol: Sweeping; eventTime: Day

##### Distribution

Palearctic

#### Philodromus fuscolimbatus

Lucas, 1846

##### Materials

- **Type status:** Other material. **Occurrence:** individualCount: 1; sex: male; **Location:** locationID: A2; continent: Europe; country: Spain; countryCode: ES; stateProvince: Catalonia; county: Lleida; locality: Sola de Boi; verbatimElevation: 1738.7; decimalLatitude: 42.54913; decimalLongitude: 0.87137; geodeticDatum: WGS84; **Event:** eventID: 2; samplingProtocol: Sweeping; eventTime: Day
- **Type status:** Other material. **Occurrence:** individualCount: 1; sex: female; **Location:** locationID: A2; continent: Europe; country: Spain; countryCode: ES; stateProvince: Catalonia; county: Lleida; locality: Sola de Boi; verbatimElevation: 1738.7; decimalLatitude: 42.54913; decimalLongitude: 0.87137; geodeticDatum: WGS84; **Event:** eventID: 2; samplingProtocol: Sweeping; eventTime: Day
- **Type status:** Other material. **Occurrence:** individualCount: 1; sex: male; **Location:** locationID: C1; continent: Europe; country: Spain; countryCode: ES; stateProvince: Castilla-La Mancha; county: Ciudad Real; locality: Valle Brezoso; verbatimElevation: 756.56; decimalLatitude: 39.35663; decimalLongitude: -4.35912; geodeticDatum: WGS84; **Event:** eventID: 1; samplingProtocol: Aerial; eventTime: Night
- **Type status:** Other material. **Occurrence:** individualCount: 1; sex: female; **Location:** locationID: C1; continent: Europe; country: Spain; countryCode: ES; stateProvince: Castilla-La Mancha; county: Ciudad Real; locality: Valle Brezoso; verbatimElevation: 756.56; decimalLatitude: 39.35663; decimalLongitude: -4.35912; geodeticDatum: WGS84; **Event:** eventID: 2; samplingProtocol: Aerial; eventTime: Night
- **Type status:** Other material. **Occurrence:** individualCount: 2; sex: male; **Location:** locationID: C1; continent: Europe; country: Spain; countryCode: ES; stateProvince: Castilla-La Mancha; county: Ciudad Real; locality: Valle Brezoso; verbatimElevation: 756.56; decimalLatitude: 39.35663; decimalLongitude: -4.35912; geodeticDatum: WGS84; **Event:** eventID: 1; samplingProtocol: Beating; eventTime: Day
- **Type status:** Other material. **Occurrence:** individualCount: 1; sex: male; **Location:** locationID: C1; continent: Europe; country: Spain; countryCode: ES; stateProvince: Castilla-La Mancha; county: Ciudad Real; locality: Valle Brezoso; verbatimElevation: 756.56; decimalLatitude: 39.35663; decimalLongitude: -4.35912; geodeticDatum: WGS84; **Event:** eventID: 2; samplingProtocol: Beating; eventTime: Day
- **Type status:** Other material. **Occurrence:** individualCount: 1; sex: female; **Location:** locationID: C1; continent: Europe; country: Spain; countryCode: ES; stateProvince: Castilla-La Mancha; county: Ciudad Real; locality: Valle Brezoso; verbatimElevation: 756.56; decimalLatitude: 39.35663; decimalLongitude: -4.35912; geodeticDatum: WGS84; **Event:** eventID: 2; samplingProtocol: Beating; eventTime: Day
- **Type status:** Other material. **Occurrence:** individualCount: 1; sex: male; **Location:** locationID: C1; continent: Europe; country: Spain; countryCode: ES; stateProvince: Castilla-La Mancha; county: Ciudad Real; locality: Valle Brezoso; verbatimElevation: 756.56; decimalLatitude: 39.35663; decimalLongitude: -4.35912; geodeticDatum: WGS84; **Event:** eventID: 1; samplingProtocol: Beating; eventTime: Night
- **Type status:** Other material. **Occurrence:** individualCount: 4; sex: male; **Location:** locationID: C1; continent: Europe; country: Spain; countryCode: ES; stateProvince: Castilla-La Mancha; county: Ciudad Real; locality: Valle Brezoso; verbatimElevation: 756.56; decimalLatitude: 39.35663; decimalLongitude: -4.35912; geodeticDatum: WGS84; **Event:** eventID: 2; samplingProtocol: Beating; eventTime: Night
- **Type status:** Other material. **Occurrence:** individualCount: 6; sex: female; **Location:** locationID: C1; continent: Europe; country: Spain; countryCode: ES; stateProvince: Castilla-La Mancha; county: Ciudad Real; locality: Valle Brezoso; verbatimElevation: 756.56; decimalLatitude: 39.35663; decimalLongitude: -4.35912; geodeticDatum: WGS84; **Event:** eventID: 2; samplingProtocol: Beating; eventTime: Night
- **Type status:** Other material. **Occurrence:** individualCount: 3; sex: male; **Location:** locationID: C1; continent: Europe; country: Spain; countryCode: ES; stateProvince: Castilla-La Mancha; county: Ciudad Real; locality: Valle Brezoso; verbatimElevation: 756.56; decimalLatitude: 39.35663; decimalLongitude: -4.35912; geodeticDatum: WGS84; **Event:** eventID: 1; samplingProtocol: Sweeping; eventTime: Day
- **Type status:** Other material. **Occurrence:** individualCount: 2; sex: female; **Location:** locationID: C1; continent: Europe; country: Spain; countryCode: ES; stateProvince: Castilla-La Mancha; county: Ciudad Real; locality: Valle Brezoso; verbatimElevation: 756.56; decimalLatitude: 39.35663; decimalLongitude: -4.35912; geodeticDatum: WGS84; **Event:** eventID: 1; samplingProtocol: Sweeping; eventTime: Day
- **Type status:** Other material. **Occurrence:** individualCount: 1; sex: male; **Location:** locationID: C1; continent: Europe; country: Spain; countryCode: ES; stateProvince: Castilla-La Mancha; county: Ciudad Real; locality: Valle Brezoso; verbatimElevation: 756.56; decimalLatitude: 39.35663; decimalLongitude: -4.35912; geodeticDatum: WGS84; **Event:** eventID: 2; samplingProtocol: Sweeping; eventTime: Day
- **Type status:** Other material. **Occurrence:** individualCount: 6; sex: female; **Location:** locationID: C1; continent: Europe; country: Spain; countryCode: ES; stateProvince: Castilla-La Mancha; county: Ciudad Real; locality: Valle Brezoso; verbatimElevation: 756.56; decimalLatitude: 39.35663; decimalLongitude: -4.35912; geodeticDatum: WGS84; **Event:** eventID: 2; samplingProtocol: Sweeping; eventTime: Day
- **Type status:** Other material. **Occurrence:** individualCount: 1; sex: male; **Location:** locationID: C1; continent: Europe; country: Spain; countryCode: ES; stateProvince: Castilla-La Mancha; county: Ciudad Real; locality: Valle Brezoso; verbatimElevation: 756.56; decimalLatitude: 39.35663; decimalLongitude: -4.35912; geodeticDatum: WGS84; **Event:** eventID: 1; samplingProtocol: Sweeping; eventTime: Night
- **Type status:** Other material. **Occurrence:** individualCount: 2; sex: female; **Location:** locationID: C1; continent: Europe; country: Spain; countryCode: ES; stateProvince: Castilla-La Mancha; county: Ciudad Real; locality: Valle Brezoso; verbatimElevation: 756.56; decimalLatitude: 39.35663; decimalLongitude: -4.35912; geodeticDatum: WGS84; **Event:** eventID: 1; samplingProtocol: Sweeping; eventTime: Night
- **Type status:** Other material. **Occurrence:** individualCount: 1; sex: female; **Location:** locationID: C2; continent: Europe; country: Spain; countryCode: ES; stateProvince: Castilla-La Mancha; county: Ciudad Real; locality: Valle Brezoso; verbatimElevation: 739.31; decimalLatitude: 39.35159; decimalLongitude: -4.3589; geodeticDatum: WGS84; **Event:** eventID: 1; samplingProtocol: Beating; eventTime: Night
- **Type status:** Other material. **Occurrence:** individualCount: 2; sex: male; **Location:** locationID: C2; continent: Europe; country: Spain; countryCode: ES; stateProvince: Castilla-La Mancha; county: Ciudad Real; locality: Valle Brezoso; verbatimElevation: 739.31; decimalLatitude: 39.35159; decimalLongitude: -4.3589; geodeticDatum: WGS84; **Event:** eventID: 2; samplingProtocol: Beating; eventTime: Night
- **Type status:** Other material. **Occurrence:** individualCount: 1; sex: male; **Location:** locationID: C2; continent: Europe; country: Spain; countryCode: ES; stateProvince: Castilla-La Mancha; county: Ciudad Real; locality: Valle Brezoso; verbatimElevation: 739.31; decimalLatitude: 39.35159; decimalLongitude: -4.3589; geodeticDatum: WGS84; **Event:** eventID: 1; samplingProtocol: Sweeping; eventTime: Day
- **Type status:** Other material. **Occurrence:** individualCount: 2; sex: female; **Location:** locationID: C2; continent: Europe; country: Spain; countryCode: ES; stateProvince: Castilla-La Mancha; county: Ciudad Real; locality: Valle Brezoso; verbatimElevation: 739.31; decimalLatitude: 39.35159; decimalLongitude: -4.3589; geodeticDatum: WGS84; **Event:** eventID: 1; samplingProtocol: Sweeping; eventTime: Day
- **Type status:** Other material. **Occurrence:** individualCount: 2; sex: male; **Location:** locationID: C2; continent: Europe; country: Spain; countryCode: ES; stateProvince: Castilla-La Mancha; county: Ciudad Real; locality: Valle Brezoso; verbatimElevation: 739.31; decimalLatitude: 39.35159; decimalLongitude: -4.3589; geodeticDatum: WGS84; **Event:** eventID: 1; samplingProtocol: Sweeping; eventTime: Night
- **Type status:** Other material. **Occurrence:** individualCount: 1; sex: male; **Location:** locationID: C3; continent: Europe; country: Spain; countryCode: ES; stateProvince: Castilla-La Mancha; county: Ciudad Real; locality: La Quesera; verbatimElevation: 767.55; decimalLatitude: 39.36177; decimalLongitude: -4.41733; geodeticDatum: WGS84; **Event:** eventID: 1; samplingProtocol: Beating; eventTime: Day
- **Type status:** Other material. **Occurrence:** individualCount: 1; sex: female; **Location:** locationID: C3; continent: Europe; country: Spain; countryCode: ES; stateProvince: Castilla-La Mancha; county: Ciudad Real; locality: La Quesera; verbatimElevation: 767.55; decimalLatitude: 39.36177; decimalLongitude: -4.41733; geodeticDatum: WGS84; **Event:** eventID: 1; samplingProtocol: Beating; eventTime: Day
- **Type status:** Other material. **Occurrence:** individualCount: 1; sex: male; **Location:** locationID: C3; continent: Europe; country: Spain; countryCode: ES; stateProvince: Castilla-La Mancha; county: Ciudad Real; locality: La Quesera; verbatimElevation: 767.55; decimalLatitude: 39.36177; decimalLongitude: -4.41733; geodeticDatum: WGS84; **Event:** eventID: 2; samplingProtocol: Beating; eventTime: Day
- **Type status:** Other material. **Occurrence:** individualCount: 2; sex: female; **Location:** locationID: C3; continent: Europe; country: Spain; countryCode: ES; stateProvince: Castilla-La Mancha; county: Ciudad Real; locality: La Quesera; verbatimElevation: 767.55; decimalLatitude: 39.36177; decimalLongitude: -4.41733; geodeticDatum: WGS84; **Event:** eventID: 2; samplingProtocol: Beating; eventTime: Day
- **Type status:** Other material. **Occurrence:** individualCount: 1; sex: male; **Location:** locationID: C3; continent: Europe; country: Spain; countryCode: ES; stateProvince: Castilla-La Mancha; county: Ciudad Real; locality: La Quesera; verbatimElevation: 767.55; decimalLatitude: 39.36177; decimalLongitude: -4.41733; geodeticDatum: WGS84; **Event:** eventID: 1; samplingProtocol: Beating; eventTime: Night
- **Type status:** Other material. **Occurrence:** individualCount: 2; sex: female; **Location:** locationID: C3; continent: Europe; country: Spain; countryCode: ES; stateProvince: Castilla-La Mancha; county: Ciudad Real; locality: La Quesera; verbatimElevation: 767.55; decimalLatitude: 39.36177; decimalLongitude: -4.41733; geodeticDatum: WGS84; **Event:** eventID: 2; samplingProtocol: Beating; eventTime: Night
- **Type status:** Other material. **Occurrence:** individualCount: 1; sex: male; **Location:** locationID: C3; continent: Europe; country: Spain; countryCode: ES; stateProvince: Castilla-La Mancha; county: Ciudad Real; locality: La Quesera; verbatimElevation: 767.55; decimalLatitude: 39.36177; decimalLongitude: -4.41733; geodeticDatum: WGS84; **Event:** eventID: 1; samplingProtocol: Sweeping; eventTime: Day
- **Type status:** Other material. **Occurrence:** individualCount: 1; sex: female; **Location:** locationID: C3; continent: Europe; country: Spain; countryCode: ES; stateProvince: Castilla-La Mancha; county: Ciudad Real; locality: La Quesera; verbatimElevation: 767.55; decimalLatitude: 39.36177; decimalLongitude: -4.41733; geodeticDatum: WGS84; **Event:** eventID: 1; samplingProtocol: Sweeping; eventTime: Day
- **Type status:** Other material. **Occurrence:** individualCount: 1; sex: male; **Location:** locationID: C3; continent: Europe; country: Spain; countryCode: ES; stateProvince: Castilla-La Mancha; county: Ciudad Real; locality: La Quesera; verbatimElevation: 767.55; decimalLatitude: 39.36177; decimalLongitude: -4.41733; geodeticDatum: WGS84; **Event:** eventID: 1; samplingProtocol: Sweeping; eventTime: Night
- **Type status:** Other material. **Occurrence:** individualCount: 1; sex: male; **Location:** locationID: C3; continent: Europe; country: Spain; countryCode: ES; stateProvince: Castilla-La Mancha; county: Ciudad Real; locality: La Quesera; verbatimElevation: 767.55; decimalLatitude: 39.36177; decimalLongitude: -4.41733; geodeticDatum: WGS84; **Event:** eventID: 2; samplingProtocol: Sweeping; eventTime: Night
- **Type status:** Other material. **Occurrence:** individualCount: 1; sex: male; **Location:** locationID: C4; continent: Europe; country: Spain; countryCode: ES; stateProvince: Castilla-La Mancha; county: Ciudad Real; locality: La Quesera; verbatimElevation: 772.3; decimalLatitude: 39.36337; decimalLongitude: -4.41704; geodeticDatum: WGS84; **Event:** eventID: 4; samplingProtocol: Aerial; eventTime: Night
- **Type status:** Other material. **Occurrence:** individualCount: 1; sex: male; **Location:** locationID: C4; continent: Europe; country: Spain; countryCode: ES; stateProvince: Castilla-La Mancha; county: Ciudad Real; locality: La Quesera; verbatimElevation: 772.3; decimalLatitude: 39.36337; decimalLongitude: -4.41704; geodeticDatum: WGS84; **Event:** eventID: 1; samplingProtocol: Beating; eventTime: Day
- **Type status:** Other material. **Occurrence:** individualCount: 1; sex: male; **Location:** locationID: C4; continent: Europe; country: Spain; countryCode: ES; stateProvince: Castilla-La Mancha; county: Ciudad Real; locality: La Quesera; verbatimElevation: 772.3; decimalLatitude: 39.36337; decimalLongitude: -4.41704; geodeticDatum: WGS84; **Event:** eventID: 2; samplingProtocol: Beating; eventTime: Day
- **Type status:** Other material. **Occurrence:** individualCount: 1; sex: male; **Location:** locationID: C4; continent: Europe; country: Spain; countryCode: ES; stateProvince: Castilla-La Mancha; county: Ciudad Real; locality: La Quesera; verbatimElevation: 772.3; decimalLatitude: 39.36337; decimalLongitude: -4.41704; geodeticDatum: WGS84; **Event:** eventID: 2; samplingProtocol: Sweeping; eventTime: Day
- **Type status:** Other material. **Occurrence:** individualCount: 2; sex: male; **Location:** locationID: M1; continent: Europe; country: Spain; countryCode: ES; stateProvince: Extremadura; county: Cáceres; locality: Peña Falcón; verbatimElevation: 320.6; decimalLatitude: 39.83296; decimalLongitude: -6.0641; geodeticDatum: WGS84; **Event:** eventID: 2; samplingProtocol: Beating; eventTime: Day
- **Type status:** Other material. **Occurrence:** individualCount: 1; sex: female; **Location:** locationID: M1; continent: Europe; country: Spain; countryCode: ES; stateProvince: Extremadura; county: Cáceres; locality: Peña Falcón; verbatimElevation: 320.6; decimalLatitude: 39.83296; decimalLongitude: -6.0641; geodeticDatum: WGS84; **Event:** eventID: C; samplingProtocol: Pitfall
- **Type status:** Other material. **Occurrence:** individualCount: 1; sex: male; **Location:** locationID: M2; continent: Europe; country: Spain; countryCode: ES; stateProvince: Extremadura; county: Cáceres; locality: Fuente del Frances; verbatimElevation: 320.72; decimalLatitude: 39.828; decimalLongitude: -6.03249; geodeticDatum: WGS84; **Event:** eventID: L; samplingProtocol: Pitfall
- **Type status:** Other material. **Occurrence:** individualCount: 2; sex: female; **Location:** locationID: O1; continent: Europe; country: Spain; countryCode: ES; stateProvince: Aragón; county: Huesca; locality: O Furno; verbatimElevation: 1396.73; decimalLatitude: 42.60677; decimalLongitude: 0.13135; geodeticDatum: WGS84; **Event:** eventID: 1; samplingProtocol: Beating; eventTime: Night
- **Type status:** Other material. **Occurrence:** individualCount: 2; sex: male; **Location:** locationID: O1; continent: Europe; country: Spain; countryCode: ES; stateProvince: Aragón; county: Huesca; locality: O Furno; verbatimElevation: 1396.73; decimalLatitude: 42.60677; decimalLongitude: 0.13135; geodeticDatum: WGS84; **Event:** eventID: 2; samplingProtocol: Sweeping; eventTime: Day
- **Type status:** Other material. **Occurrence:** individualCount: 1; sex: female; **Location:** locationID: O1; continent: Europe; country: Spain; countryCode: ES; stateProvince: Aragón; county: Huesca; locality: O Furno; verbatimElevation: 1396.73; decimalLatitude: 42.60677; decimalLongitude: 0.13135; geodeticDatum: WGS84; **Event:** eventID: 2; samplingProtocol: Sweeping; eventTime: Day
- **Type status:** Other material. **Occurrence:** individualCount: 1; sex: male; **Location:** locationID: O1; continent: Europe; country: Spain; countryCode: ES; stateProvince: Aragón; county: Huesca; locality: O Furno; verbatimElevation: 1396.73; decimalLatitude: 42.60677; decimalLongitude: 0.13135; geodeticDatum: WGS84; **Event:** eventID: 2; samplingProtocol: Sweeping; eventTime: Night
- **Type status:** Other material. **Occurrence:** individualCount: 1; sex: male; **Location:** locationID: O2; continent: Europe; country: Spain; countryCode: ES; stateProvince: Aragón; county: Huesca; locality: Rebilla; verbatimElevation: 1158.13; decimalLatitude: 42.59427; decimalLongitude: 0.1529; geodeticDatum: WGS84; **Event:** eventID: 2; samplingProtocol: Aerial; eventTime: Night
- **Type status:** Other material. **Occurrence:** individualCount: 1; sex: male; **Location:** locationID: S1; continent: Europe; country: Spain; countryCode: ES; stateProvince: Andalucía; county: Granada; locality: Soportujar; verbatimElevation: 1786.57; decimalLatitude: 36.96151; decimalLongitude: -3.41881; geodeticDatum: WGS84; **Event:** eventID: 1; samplingProtocol: Beating; eventTime: Night
- **Type status:** Other material. **Occurrence:** individualCount: 2; sex: female; **Location:** locationID: S1; continent: Europe; country: Spain; countryCode: ES; stateProvince: Andalucía; county: Granada; locality: Soportujar; verbatimElevation: 1786.57; decimalLatitude: 36.96151; decimalLongitude: -3.41881; geodeticDatum: WGS84; **Event:** eventID: 1; samplingProtocol: Beating; eventTime: Night
- **Type status:** Other material. **Occurrence:** individualCount: 1; sex: female; **Location:** locationID: S1; continent: Europe; country: Spain; countryCode: ES; stateProvince: Andalucía; county: Granada; locality: Soportujar; verbatimElevation: 1786.57; decimalLatitude: 36.96151; decimalLongitude: -3.41881; geodeticDatum: WGS84; **Event:** eventID: 1; samplingProtocol: Sweeping; eventTime: Day
- **Type status:** Other material. **Occurrence:** individualCount: 1; sex: female; **Location:** locationID: S1; continent: Europe; country: Spain; countryCode: ES; stateProvince: Andalucía; county: Granada; locality: Soportujar; verbatimElevation: 1786.57; decimalLatitude: 36.96151; decimalLongitude: -3.41881; geodeticDatum: WGS84; **Event:** eventID: 2; samplingProtocol: Sweeping; eventTime: Day

##### Distribution

Mediterranean

#### Philodromus lividus

Simon, 1875

##### Materials

- **Type status:** Other material. **Occurrence:** individualCount: 1; sex: male; **Location:** locationID: C1; continent: Europe; country: Spain; countryCode: ES; stateProvince: Castilla-La Mancha; county: Ciudad Real; locality: Valle Brezoso; verbatimElevation: 756.56; decimalLatitude: 39.35663; decimalLongitude: -4.35912; geodeticDatum: WGS84; **Event:** eventID: 1; samplingProtocol: Aerial; eventTime: Night
- **Type status:** Other material. **Occurrence:** individualCount: 1; sex: female; **Location:** locationID: C1; continent: Europe; country: Spain; countryCode: ES; stateProvince: Castilla-La Mancha; county: Ciudad Real; locality: Valle Brezoso; verbatimElevation: 756.56; decimalLatitude: 39.35663; decimalLongitude: -4.35912; geodeticDatum: WGS84; **Event:** eventID: 1; samplingProtocol: Beating; eventTime: Night
- **Type status:** Other material. **Occurrence:** individualCount: 1; sex: female; **Location:** locationID: C1; continent: Europe; country: Spain; countryCode: ES; stateProvince: Castilla-La Mancha; county: Ciudad Real; locality: Valle Brezoso; verbatimElevation: 756.56; decimalLatitude: 39.35663; decimalLongitude: -4.35912; geodeticDatum: WGS84; **Event:** eventID: 2; samplingProtocol: Beating; eventTime: Night
- **Type status:** Other material. **Occurrence:** individualCount: 1; sex: male; **Location:** locationID: C1; continent: Europe; country: Spain; countryCode: ES; stateProvince: Castilla-La Mancha; county: Ciudad Real; locality: Valle Brezoso; verbatimElevation: 756.56; decimalLatitude: 39.35663; decimalLongitude: -4.35912; geodeticDatum: WGS84; **Event:** eventID: 2; samplingProtocol: Sweeping; eventTime: Night
- **Type status:** Other material. **Occurrence:** individualCount: 1; sex: male; **Location:** locationID: C2; continent: Europe; country: Spain; countryCode: ES; stateProvince: Castilla-La Mancha; county: Ciudad Real; locality: Valle Brezoso; verbatimElevation: 739.31; decimalLatitude: 39.35159; decimalLongitude: -4.3589; geodeticDatum: WGS84; **Event:** eventID: 2; samplingProtocol: Beating; eventTime: Day
- **Type status:** Other material. **Occurrence:** individualCount: 2; sex: female; **Location:** locationID: C2; continent: Europe; country: Spain; countryCode: ES; stateProvince: Castilla-La Mancha; county: Ciudad Real; locality: Valle Brezoso; verbatimElevation: 739.31; decimalLatitude: 39.35159; decimalLongitude: -4.3589; geodeticDatum: WGS84; **Event:** eventID: 1; samplingProtocol: Beating; eventTime: Night
- **Type status:** Other material. **Occurrence:** individualCount: 1; sex: female; **Location:** locationID: C2; continent: Europe; country: Spain; countryCode: ES; stateProvince: Castilla-La Mancha; county: Ciudad Real; locality: Valle Brezoso; verbatimElevation: 739.31; decimalLatitude: 39.35159; decimalLongitude: -4.3589; geodeticDatum: WGS84; **Event:** eventID: 2; samplingProtocol: Beating; eventTime: Night
- **Type status:** Other material. **Occurrence:** individualCount: 1; sex: female; **Location:** locationID: C2; continent: Europe; country: Spain; countryCode: ES; stateProvince: Castilla-La Mancha; county: Ciudad Real; locality: Valle Brezoso; verbatimElevation: 739.31; decimalLatitude: 39.35159; decimalLongitude: -4.3589; geodeticDatum: WGS84; **Event:** eventID: 2; samplingProtocol: Sweeping; eventTime: Day
- **Type status:** Other material. **Occurrence:** individualCount: 1; sex: male; **Location:** locationID: C3; continent: Europe; country: Spain; countryCode: ES; stateProvince: Castilla-La Mancha; county: Ciudad Real; locality: La Quesera; verbatimElevation: 767.55; decimalLatitude: 39.36177; decimalLongitude: -4.41733; geodeticDatum: WGS84; **Event:** eventID: 1; samplingProtocol: Aerial; eventTime: Night
- **Type status:** Other material. **Occurrence:** individualCount: 1; sex: male; **Location:** locationID: C3; continent: Europe; country: Spain; countryCode: ES; stateProvince: Castilla-La Mancha; county: Ciudad Real; locality: La Quesera; verbatimElevation: 767.55; decimalLatitude: 39.36177; decimalLongitude: -4.41733; geodeticDatum: WGS84; **Event:** eventID: 4; samplingProtocol: Aerial; eventTime: Night
- **Type status:** Other material. **Occurrence:** individualCount: 4; sex: male; **Location:** locationID: C3; continent: Europe; country: Spain; countryCode: ES; stateProvince: Castilla-La Mancha; county: Ciudad Real; locality: La Quesera; verbatimElevation: 767.55; decimalLatitude: 39.36177; decimalLongitude: -4.41733; geodeticDatum: WGS84; **Event:** eventID: 1; samplingProtocol: Beating; eventTime: Day
- **Type status:** Other material. **Occurrence:** individualCount: 1; sex: female; **Location:** locationID: C3; continent: Europe; country: Spain; countryCode: ES; stateProvince: Castilla-La Mancha; county: Ciudad Real; locality: La Quesera; verbatimElevation: 767.55; decimalLatitude: 39.36177; decimalLongitude: -4.41733; geodeticDatum: WGS84; **Event:** eventID: 1; samplingProtocol: Beating; eventTime: Day
- **Type status:** Other material. **Occurrence:** individualCount: 1; sex: male; **Location:** locationID: C3; continent: Europe; country: Spain; countryCode: ES; stateProvince: Castilla-La Mancha; county: Ciudad Real; locality: La Quesera; verbatimElevation: 767.55; decimalLatitude: 39.36177; decimalLongitude: -4.41733; geodeticDatum: WGS84; **Event:** eventID: 2; samplingProtocol: Beating; eventTime: Day
- **Type status:** Other material. **Occurrence:** individualCount: 1; sex: female; **Location:** locationID: C3; continent: Europe; country: Spain; countryCode: ES; stateProvince: Castilla-La Mancha; county: Ciudad Real; locality: La Quesera; verbatimElevation: 767.55; decimalLatitude: 39.36177; decimalLongitude: -4.41733; geodeticDatum: WGS84; **Event:** eventID: 2; samplingProtocol: Beating; eventTime: Day
- **Type status:** Other material. **Occurrence:** individualCount: 3; sex: male; **Location:** locationID: C3; continent: Europe; country: Spain; countryCode: ES; stateProvince: Castilla-La Mancha; county: Ciudad Real; locality: La Quesera; verbatimElevation: 767.55; decimalLatitude: 39.36177; decimalLongitude: -4.41733; geodeticDatum: WGS84; **Event:** eventID: 1; samplingProtocol: Beating; eventTime: Night
- **Type status:** Other material. **Occurrence:** individualCount: 2; sex: female; **Location:** locationID: C3; continent: Europe; country: Spain; countryCode: ES; stateProvince: Castilla-La Mancha; county: Ciudad Real; locality: La Quesera; verbatimElevation: 767.55; decimalLatitude: 39.36177; decimalLongitude: -4.41733; geodeticDatum: WGS84; **Event:** eventID: 2; samplingProtocol: Beating; eventTime: Night
- **Type status:** Other material. **Occurrence:** individualCount: 1; sex: male; **Location:** locationID: C3; continent: Europe; country: Spain; countryCode: ES; stateProvince: Castilla-La Mancha; county: Ciudad Real; locality: La Quesera; verbatimElevation: 767.55; decimalLatitude: 39.36177; decimalLongitude: -4.41733; geodeticDatum: WGS84; **Event:** eventID: 1; samplingProtocol: Sweeping; eventTime: Day
- **Type status:** Other material. **Occurrence:** individualCount: 1; sex: female; **Location:** locationID: C3; continent: Europe; country: Spain; countryCode: ES; stateProvince: Castilla-La Mancha; county: Ciudad Real; locality: La Quesera; verbatimElevation: 767.55; decimalLatitude: 39.36177; decimalLongitude: -4.41733; geodeticDatum: WGS84; **Event:** eventID: 1; samplingProtocol: Sweeping; eventTime: Day
- **Type status:** Other material. **Occurrence:** individualCount: 2; sex: male; **Location:** locationID: C3; continent: Europe; country: Spain; countryCode: ES; stateProvince: Castilla-La Mancha; county: Ciudad Real; locality: La Quesera; verbatimElevation: 767.55; decimalLatitude: 39.36177; decimalLongitude: -4.41733; geodeticDatum: WGS84; **Event:** eventID: 2; samplingProtocol: Sweeping; eventTime: Day
- **Type status:** Other material. **Occurrence:** individualCount: 1; sex: male; **Location:** locationID: C3; continent: Europe; country: Spain; countryCode: ES; stateProvince: Castilla-La Mancha; county: Ciudad Real; locality: La Quesera; verbatimElevation: 767.55; decimalLatitude: 39.36177; decimalLongitude: -4.41733; geodeticDatum: WGS84; **Event:** eventID: 1; samplingProtocol: Sweeping; eventTime: Night
- **Type status:** Other material. **Occurrence:** individualCount: 1; sex: male; **Location:** locationID: C3; continent: Europe; country: Spain; countryCode: ES; stateProvince: Castilla-La Mancha; county: Ciudad Real; locality: La Quesera; verbatimElevation: 767.55; decimalLatitude: 39.36177; decimalLongitude: -4.41733; geodeticDatum: WGS84; **Event:** eventID: 2; samplingProtocol: Sweeping; eventTime: Night
- **Type status:** Other material. **Occurrence:** individualCount: 3; sex: female; **Location:** locationID: C3; continent: Europe; country: Spain; countryCode: ES; stateProvince: Castilla-La Mancha; county: Ciudad Real; locality: La Quesera; verbatimElevation: 767.55; decimalLatitude: 39.36177; decimalLongitude: -4.41733; geodeticDatum: WGS84; **Event:** eventID: 2; samplingProtocol: Sweeping; eventTime: Night
- **Type status:** Other material. **Occurrence:** individualCount: 2; sex: male; **Location:** locationID: C4; continent: Europe; country: Spain; countryCode: ES; stateProvince: Castilla-La Mancha; county: Ciudad Real; locality: La Quesera; verbatimElevation: 772.3; decimalLatitude: 39.36337; decimalLongitude: -4.41704; geodeticDatum: WGS84; **Event:** eventID: 1; samplingProtocol: Aerial; eventTime: Night
- **Type status:** Other material. **Occurrence:** individualCount: 2; sex: female; **Location:** locationID: C4; continent: Europe; country: Spain; countryCode: ES; stateProvince: Castilla-La Mancha; county: Ciudad Real; locality: La Quesera; verbatimElevation: 772.3; decimalLatitude: 39.36337; decimalLongitude: -4.41704; geodeticDatum: WGS84; **Event:** eventID: 1; samplingProtocol: Aerial; eventTime: Night
- **Type status:** Other material. **Occurrence:** individualCount: 3; sex: male; **Location:** locationID: C4; continent: Europe; country: Spain; countryCode: ES; stateProvince: Castilla-La Mancha; county: Ciudad Real; locality: La Quesera; verbatimElevation: 772.3; decimalLatitude: 39.36337; decimalLongitude: -4.41704; geodeticDatum: WGS84; **Event:** eventID: 2; samplingProtocol: Aerial; eventTime: Night
- **Type status:** Other material. **Occurrence:** individualCount: 1; sex: female; **Location:** locationID: C4; continent: Europe; country: Spain; countryCode: ES; stateProvince: Castilla-La Mancha; county: Ciudad Real; locality: La Quesera; verbatimElevation: 772.3; decimalLatitude: 39.36337; decimalLongitude: -4.41704; geodeticDatum: WGS84; **Event:** eventID: 4; samplingProtocol: Aerial; eventTime: Night
- **Type status:** Other material. **Occurrence:** individualCount: 2; sex: female; **Location:** locationID: C4; continent: Europe; country: Spain; countryCode: ES; stateProvince: Castilla-La Mancha; county: Ciudad Real; locality: La Quesera; verbatimElevation: 772.3; decimalLatitude: 39.36337; decimalLongitude: -4.41704; geodeticDatum: WGS84; **Event:** eventID: 1; samplingProtocol: Beating; eventTime: Night
- **Type status:** Other material. **Occurrence:** individualCount: 1; sex: female; **Location:** locationID: C4; continent: Europe; country: Spain; countryCode: ES; stateProvince: Castilla-La Mancha; county: Ciudad Real; locality: La Quesera; verbatimElevation: 772.3; decimalLatitude: 39.36337; decimalLongitude: -4.41704; geodeticDatum: WGS84; **Event:** eventID: 1; samplingProtocol: Sweeping; eventTime: Day
- **Type status:** Other material. **Occurrence:** individualCount: 1; sex: male; **Location:** locationID: C4; continent: Europe; country: Spain; countryCode: ES; stateProvince: Castilla-La Mancha; county: Ciudad Real; locality: La Quesera; verbatimElevation: 772.3; decimalLatitude: 39.36337; decimalLongitude: -4.41704; geodeticDatum: WGS84; **Event:** eventID: 2; samplingProtocol: Sweeping; eventTime: Day
- **Type status:** Other material. **Occurrence:** individualCount: 1; sex: female; **Location:** locationID: C4; continent: Europe; country: Spain; countryCode: ES; stateProvince: Castilla-La Mancha; county: Ciudad Real; locality: La Quesera; verbatimElevation: 772.3; decimalLatitude: 39.36337; decimalLongitude: -4.41704; geodeticDatum: WGS84; **Event:** eventID: 2; samplingProtocol: Sweeping; eventTime: Day
- **Type status:** Other material. **Occurrence:** individualCount: 1; sex: female; **Location:** locationID: C4; continent: Europe; country: Spain; countryCode: ES; stateProvince: Castilla-La Mancha; county: Ciudad Real; locality: La Quesera; verbatimElevation: 772.3; decimalLatitude: 39.36337; decimalLongitude: -4.41704; geodeticDatum: WGS84; **Event:** eventID: 1; samplingProtocol: Sweeping; eventTime: Night
- **Type status:** Other material. **Occurrence:** individualCount: 1; sex: male; **Location:** locationID: C4; continent: Europe; country: Spain; countryCode: ES; stateProvince: Castilla-La Mancha; county: Ciudad Real; locality: La Quesera; verbatimElevation: 772.3; decimalLatitude: 39.36337; decimalLongitude: -4.41704; geodeticDatum: WGS84; **Event:** eventID: 2; samplingProtocol: Sweeping; eventTime: Night
- **Type status:** Other material. **Occurrence:** individualCount: 1; sex: female; **Location:** locationID: C4; continent: Europe; country: Spain; countryCode: ES; stateProvince: Castilla-La Mancha; county: Ciudad Real; locality: La Quesera; verbatimElevation: 772.3; decimalLatitude: 39.36337; decimalLongitude: -4.41704; geodeticDatum: WGS84; **Event:** eventID: 2; samplingProtocol: Sweeping; eventTime: Night
- **Type status:** Other material. **Occurrence:** individualCount: 1; sex: female; **Location:** locationID: M1; continent: Europe; country: Spain; countryCode: ES; stateProvince: Extremadura; county: Cáceres; locality: Peña Falcón; verbatimElevation: 320.6; decimalLatitude: 39.83296; decimalLongitude: -6.0641; geodeticDatum: WGS84; **Event:** eventID: 3; samplingProtocol: Aerial; eventTime: Night
- **Type status:** Other material. **Occurrence:** individualCount: 2; sex: female; **Location:** locationID: M1; continent: Europe; country: Spain; countryCode: ES; stateProvince: Extremadura; county: Cáceres; locality: Peña Falcón; verbatimElevation: 320.6; decimalLatitude: 39.83296; decimalLongitude: -6.0641; geodeticDatum: WGS84; **Event:** eventID: 2; samplingProtocol: Beating; eventTime: Night
- **Type status:** Other material. **Occurrence:** individualCount: 1; sex: female; **Location:** locationID: M1; continent: Europe; country: Spain; countryCode: ES; stateProvince: Extremadura; county: Cáceres; locality: Peña Falcón; verbatimElevation: 320.6; decimalLatitude: 39.83296; decimalLongitude: -6.0641; geodeticDatum: WGS84; **Event:** eventID: 1; samplingProtocol: Sweeping; eventTime: Day
- **Type status:** Other material. **Occurrence:** individualCount: 1; sex: female; **Location:** locationID: M1; continent: Europe; country: Spain; countryCode: ES; stateProvince: Extremadura; county: Cáceres; locality: Peña Falcón; verbatimElevation: 320.6; decimalLatitude: 39.83296; decimalLongitude: -6.0641; geodeticDatum: WGS84; **Event:** eventID: 2; samplingProtocol: Sweeping; eventTime: Night
- **Type status:** Other material. **Occurrence:** individualCount: 1; sex: male; **Location:** locationID: M1; continent: Europe; country: Spain; countryCode: ES; stateProvince: Extremadura; county: Cáceres; locality: Peña Falcón; verbatimElevation: 320.6; decimalLatitude: 39.83296; decimalLongitude: -6.0641; geodeticDatum: WGS84; **Event:** eventID: 1; samplingProtocol: Sweeping; eventTime: Night
- **Type status:** Other material. **Occurrence:** individualCount: 1; sex: male; **Location:** locationID: M2; continent: Europe; country: Spain; countryCode: ES; stateProvince: Extremadura; county: Cáceres; locality: Fuente del Frances; verbatimElevation: 320.72; decimalLatitude: 39.828; decimalLongitude: -6.03249; geodeticDatum: WGS84; **Event:** eventID: 1; samplingProtocol: Aerial; eventTime: Night
- **Type status:** Other material. **Occurrence:** individualCount: 1; sex: female; **Location:** locationID: M2; continent: Europe; country: Spain; countryCode: ES; stateProvince: Extremadura; county: Cáceres; locality: Fuente del Frances; verbatimElevation: 320.72; decimalLatitude: 39.828; decimalLongitude: -6.03249; geodeticDatum: WGS84; **Event:** eventID: 2; samplingProtocol: Aerial; eventTime: Night
- **Type status:** Other material. **Occurrence:** individualCount: 1; sex: female; **Location:** locationID: M2; continent: Europe; country: Spain; countryCode: ES; stateProvince: Extremadura; county: Cáceres; locality: Fuente del Frances; verbatimElevation: 320.72; decimalLatitude: 39.828; decimalLongitude: -6.03249; geodeticDatum: WGS84; **Event:** eventID: 3; samplingProtocol: Aerial; eventTime: Night
- **Type status:** Other material. **Occurrence:** individualCount: 4; sex: male; **Location:** locationID: M2; continent: Europe; country: Spain; countryCode: ES; stateProvince: Extremadura; county: Cáceres; locality: Fuente del Frances; verbatimElevation: 320.72; decimalLatitude: 39.828; decimalLongitude: -6.03249; geodeticDatum: WGS84; **Event:** eventID: 1; samplingProtocol: Beating; eventTime: Day
- **Type status:** Other material. **Occurrence:** individualCount: 1; sex: female; **Location:** locationID: M2; continent: Europe; country: Spain; countryCode: ES; stateProvince: Extremadura; county: Cáceres; locality: Fuente del Frances; verbatimElevation: 320.72; decimalLatitude: 39.828; decimalLongitude: -6.03249; geodeticDatum: WGS84; **Event:** eventID: 1; samplingProtocol: Beating; eventTime: Day
- **Type status:** Other material. **Occurrence:** individualCount: 1; sex: male; **Location:** locationID: M2; continent: Europe; country: Spain; countryCode: ES; stateProvince: Extremadura; county: Cáceres; locality: Fuente del Frances; verbatimElevation: 320.72; decimalLatitude: 39.828; decimalLongitude: -6.03249; geodeticDatum: WGS84; **Event:** eventID: 2; samplingProtocol: Beating; eventTime: Day
- **Type status:** Other material. **Occurrence:** individualCount: 2; sex: male; **Location:** locationID: M2; continent: Europe; country: Spain; countryCode: ES; stateProvince: Extremadura; county: Cáceres; locality: Fuente del Frances; verbatimElevation: 320.72; decimalLatitude: 39.828; decimalLongitude: -6.03249; geodeticDatum: WGS84; **Event:** eventID: 1; samplingProtocol: Beating; eventTime: Night
- **Type status:** Other material. **Occurrence:** individualCount: 2; sex: female; **Location:** locationID: M2; continent: Europe; country: Spain; countryCode: ES; stateProvince: Extremadura; county: Cáceres; locality: Fuente del Frances; verbatimElevation: 320.72; decimalLatitude: 39.828; decimalLongitude: -6.03249; geodeticDatum: WGS84; **Event:** eventID: 1; samplingProtocol: Beating; eventTime: Night
- **Type status:** Other material. **Occurrence:** individualCount: 1; sex: male; **Location:** locationID: M2; continent: Europe; country: Spain; countryCode: ES; stateProvince: Extremadura; county: Cáceres; locality: Fuente del Frances; verbatimElevation: 320.72; decimalLatitude: 39.828; decimalLongitude: -6.03249; geodeticDatum: WGS84; **Event:** eventID: 2; samplingProtocol: Beating; eventTime: Night
- **Type status:** Other material. **Occurrence:** individualCount: 2; sex: female; **Location:** locationID: M2; continent: Europe; country: Spain; countryCode: ES; stateProvince: Extremadura; county: Cáceres; locality: Fuente del Frances; verbatimElevation: 320.72; decimalLatitude: 39.828; decimalLongitude: -6.03249; geodeticDatum: WGS84; **Event:** eventID: 2; samplingProtocol: Beating; eventTime: Night
- **Type status:** Other material. **Occurrence:** individualCount: 3; sex: female; **Location:** locationID: M2; continent: Europe; country: Spain; countryCode: ES; stateProvince: Extremadura; county: Cáceres; locality: Fuente del Frances; verbatimElevation: 320.72; decimalLatitude: 39.828; decimalLongitude: -6.03249; geodeticDatum: WGS84; **Event:** eventID: 1; samplingProtocol: Sweeping; eventTime: Day
- **Type status:** Other material. **Occurrence:** individualCount: 1; sex: female; **Location:** locationID: M2; continent: Europe; country: Spain; countryCode: ES; stateProvince: Extremadura; county: Cáceres; locality: Fuente del Frances; verbatimElevation: 320.72; decimalLatitude: 39.828; decimalLongitude: -6.03249; geodeticDatum: WGS84; **Event:** eventID: 2; samplingProtocol: Sweeping; eventTime: Night

##### Distribution

Iberian Peninsula, France, Morocco, Algeria, Italy, Croatia

#### Philodromus pinetorum

Muster, 2009

##### Materials

- **Type status:** Other material. **Occurrence:** individualCount: 1; sex: female; **Location:** locationID: P2; continent: Europe; country: Spain; countryCode: ES; stateProvince: Castilla y León; county: León; locality: Joyoguelas; verbatimElevation: 763.98; decimalLatitude: 43.17771; decimalLongitude: -4.90579; geodeticDatum: WGS84; **Event:** eventID: 2; samplingProtocol: Aerial; eventTime: Night

##### Distribution

Portugal to Turkey

#### Philodromus praedatus

O. Pickard-Cambridge, 1871

##### Materials

- **Type status:** Other material. **Occurrence:** individualCount: 1; sex: female; **Location:** locationID: C1; continent: Europe; country: Spain; countryCode: ES; stateProvince: Castilla-La Mancha; county: Ciudad Real; locality: Valle Brezoso; verbatimElevation: 756.56; decimalLatitude: 39.35663; decimalLongitude: -4.35912; geodeticDatum: WGS84; **Event:** eventID: 1; samplingProtocol: Beating; eventTime: Day
- **Type status:** Other material. **Occurrence:** individualCount: 1; sex: female; **Location:** locationID: C1; continent: Europe; country: Spain; countryCode: ES; stateProvince: Castilla-La Mancha; county: Ciudad Real; locality: Valle Brezoso; verbatimElevation: 756.56; decimalLatitude: 39.35663; decimalLongitude: -4.35912; geodeticDatum: WGS84; **Event:** eventID: 2; samplingProtocol: Beating; eventTime: Day
- **Type status:** Other material. **Occurrence:** individualCount: 1; sex: male; **Location:** locationID: C1; continent: Europe; country: Spain; countryCode: ES; stateProvince: Castilla-La Mancha; county: Ciudad Real; locality: Valle Brezoso; verbatimElevation: 756.56; decimalLatitude: 39.35663; decimalLongitude: -4.35912; geodeticDatum: WGS84; **Event:** eventID: 1; samplingProtocol: Beating; eventTime: Night
- **Type status:** Other material. **Occurrence:** individualCount: 1; sex: female; **Location:** locationID: C1; continent: Europe; country: Spain; countryCode: ES; stateProvince: Castilla-La Mancha; county: Ciudad Real; locality: Valle Brezoso; verbatimElevation: 756.56; decimalLatitude: 39.35663; decimalLongitude: -4.35912; geodeticDatum: WGS84; **Event:** eventID: 2; samplingProtocol: Beating; eventTime: Night
- **Type status:** Other material. **Occurrence:** individualCount: 1; sex: female; **Location:** locationID: C1; continent: Europe; country: Spain; countryCode: ES; stateProvince: Castilla-La Mancha; county: Ciudad Real; locality: Valle Brezoso; verbatimElevation: 756.56; decimalLatitude: 39.35663; decimalLongitude: -4.35912; geodeticDatum: WGS84; **Event:** eventID: 2; samplingProtocol: Sweeping; eventTime: Night
- **Type status:** Other material. **Occurrence:** individualCount: 1; sex: female; **Location:** locationID: C2; continent: Europe; country: Spain; countryCode: ES; stateProvince: Castilla-La Mancha; county: Ciudad Real; locality: Valle Brezoso; verbatimElevation: 739.31; decimalLatitude: 39.35159; decimalLongitude: -4.3589; geodeticDatum: WGS84; **Event:** eventID: 4; samplingProtocol: Aerial; eventTime: Night
- **Type status:** Other material. **Occurrence:** individualCount: 1; sex: male; **Location:** locationID: C2; continent: Europe; country: Spain; countryCode: ES; stateProvince: Castilla-La Mancha; county: Ciudad Real; locality: Valle Brezoso; verbatimElevation: 739.31; decimalLatitude: 39.35159; decimalLongitude: -4.3589; geodeticDatum: WGS84; **Event:** eventID: 1; samplingProtocol: Beating; eventTime: Day
- **Type status:** Other material. **Occurrence:** individualCount: 1; sex: female; **Location:** locationID: C2; continent: Europe; country: Spain; countryCode: ES; stateProvince: Castilla-La Mancha; county: Ciudad Real; locality: Valle Brezoso; verbatimElevation: 739.31; decimalLatitude: 39.35159; decimalLongitude: -4.3589; geodeticDatum: WGS84; **Event:** eventID: 2; samplingProtocol: Beating; eventTime: Day
- **Type status:** Other material. **Occurrence:** individualCount: 1; sex: female; **Location:** locationID: C3; continent: Europe; country: Spain; countryCode: ES; stateProvince: Castilla-La Mancha; county: Ciudad Real; locality: La Quesera; verbatimElevation: 767.55; decimalLatitude: 39.36177; decimalLongitude: -4.41733; geodeticDatum: WGS84; **Event:** eventID: 1; samplingProtocol: Beating; eventTime: Day
- **Type status:** Other material. **Occurrence:** individualCount: 3; sex: female; **Location:** locationID: C4; continent: Europe; country: Spain; countryCode: ES; stateProvince: Castilla-La Mancha; county: Ciudad Real; locality: La Quesera; verbatimElevation: 772.3; decimalLatitude: 39.36337; decimalLongitude: -4.41704; geodeticDatum: WGS84; **Event:** eventID: 4; samplingProtocol: Aerial; eventTime: Night
- **Type status:** Other material. **Occurrence:** individualCount: 2; sex: male; **Location:** locationID: C4; continent: Europe; country: Spain; countryCode: ES; stateProvince: Castilla-La Mancha; county: Ciudad Real; locality: La Quesera; verbatimElevation: 772.3; decimalLatitude: 39.36337; decimalLongitude: -4.41704; geodeticDatum: WGS84; **Event:** eventID: 1; samplingProtocol: Beating; eventTime: Night
- **Type status:** Other material. **Occurrence:** individualCount: 1; sex: female; **Location:** locationID: C4; continent: Europe; country: Spain; countryCode: ES; stateProvince: Castilla-La Mancha; county: Ciudad Real; locality: La Quesera; verbatimElevation: 772.3; decimalLatitude: 39.36337; decimalLongitude: -4.41704; geodeticDatum: WGS84; **Event:** eventID: 1; samplingProtocol: Beating; eventTime: Night
- **Type status:** Other material. **Occurrence:** individualCount: 1; sex: female; **Location:** locationID: C4; continent: Europe; country: Spain; countryCode: ES; stateProvince: Castilla-La Mancha; county: Ciudad Real; locality: La Quesera; verbatimElevation: 772.3; decimalLatitude: 39.36337; decimalLongitude: -4.41704; geodeticDatum: WGS84; **Event:** eventID: 1; samplingProtocol: Sweeping; eventTime: Day
- **Type status:** Other material. **Occurrence:** individualCount: 1; sex: male; **Location:** locationID: C4; continent: Europe; country: Spain; countryCode: ES; stateProvince: Castilla-La Mancha; county: Ciudad Real; locality: La Quesera; verbatimElevation: 772.3; decimalLatitude: 39.36337; decimalLongitude: -4.41704; geodeticDatum: WGS84; **Event:** eventID: 2; samplingProtocol: Sweeping; eventTime: Night
- **Type status:** Other material. **Occurrence:** individualCount: 1; sex: female; **Location:** locationID: P4; continent: Europe; country: Spain; countryCode: ES; stateProvince: Castilla y León; county: León; locality: El Canto; verbatimElevation: 943.48; decimalLatitude: 43.17227; decimalLongitude: -4.90857; geodeticDatum: WGS84; **Event:** eventID: 1; samplingProtocol: Beating; eventTime: Night
- **Type status:** Other material. **Occurrence:** individualCount: 1; sex: female; **Location:** locationID: S1; continent: Europe; country: Spain; countryCode: ES; stateProvince: Andalucía; county: Granada; locality: Soportujar; verbatimElevation: 1786.57; decimalLatitude: 36.96151; decimalLongitude: -3.41881; geodeticDatum: WGS84; **Event:** eventID: 1; samplingProtocol: Aerial; eventTime: Night

##### Distribution

Europe, Russia, Azerbaijan

#### Philodromus rufus

Walckenaer, 1826

##### Materials

- **Type status:** Other material. **Occurrence:** individualCount: 1; sex: female; **Location:** locationID: C4; continent: Europe; country: Spain; countryCode: ES; stateProvince: Castilla-La Mancha; county: Ciudad Real; locality: La Quesera; verbatimElevation: 772.3; decimalLatitude: 39.36337; decimalLongitude: -4.41704; geodeticDatum: WGS84; **Event:** eventID: 1; samplingProtocol: Sweeping; eventTime: Night
- **Type status:** Other material. **Occurrence:** individualCount: 1; sex: female; **Location:** locationID: O2; continent: Europe; country: Spain; countryCode: ES; stateProvince: Aragón; county: Huesca; locality: Rebilla; verbatimElevation: 1158.13; decimalLatitude: 42.59427; decimalLongitude: 0.1529; geodeticDatum: WGS84; **Event:** eventID: 2; samplingProtocol: Beating; eventTime: Night
- **Type status:** Other material. **Occurrence:** individualCount: 1; sex: female; **Location:** locationID: P2; continent: Europe; country: Spain; countryCode: ES; stateProvince: Castilla y León; county: León; locality: Joyoguelas; verbatimElevation: 763.98; decimalLatitude: 43.17771; decimalLongitude: -4.90579; geodeticDatum: WGS84; **Event:** eventID: 2; samplingProtocol: Beating; eventTime: Day
- **Type status:** Other material. **Occurrence:** individualCount: 2; sex: female; **Location:** locationID: P4; continent: Europe; country: Spain; countryCode: ES; stateProvince: Castilla y León; county: León; locality: El Canto; verbatimElevation: 943.48; decimalLatitude: 43.17227; decimalLongitude: -4.90857; geodeticDatum: WGS84; **Event:** eventID: 1; samplingProtocol: Beating; eventTime: Day
- **Type status:** Other material. **Occurrence:** individualCount: 1; sex: female; **Location:** locationID: P4; continent: Europe; country: Spain; countryCode: ES; stateProvince: Castilla y León; county: León; locality: El Canto; verbatimElevation: 943.48; decimalLatitude: 43.17227; decimalLongitude: -4.90857; geodeticDatum: WGS84; **Event:** eventID: 1; samplingProtocol: Beating; eventTime: Night

##### Distribution

Holarctic

#### Philodromus sp18

##### Materials

- **Type status:** Other material. **Occurrence:** individualCount: 1; sex: female; **Location:** locationID: C1; continent: Europe; country: Spain; countryCode: ES; stateProvince: Castilla-La Mancha; county: Ciudad Real; locality: Valle Brezoso; verbatimElevation: 756.56; decimalLatitude: 39.35663; decimalLongitude: -4.35912; geodeticDatum: WGS84; **Event:** eventID: 1; samplingProtocol: Beating; eventTime: Night
- **Type status:** Other material. **Occurrence:** individualCount: 1; sex: female; **Location:** locationID: C4; continent: Europe; country: Spain; countryCode: ES; stateProvince: Castilla-La Mancha; county: Ciudad Real; locality: La Quesera; verbatimElevation: 772.3; decimalLatitude: 39.36337; decimalLongitude: -4.41704; geodeticDatum: WGS84; **Event:** eventID: 2; samplingProtocol: Aerial; eventTime: Night
- **Type status:** Other material. **Occurrence:** individualCount: 1; sex: female; **Location:** locationID: O2; continent: Europe; country: Spain; countryCode: ES; stateProvince: Aragón; county: Huesca; locality: Rebilla; verbatimElevation: 1158.13; decimalLatitude: 42.59427; decimalLongitude: 0.1529; geodeticDatum: WGS84; **Event:** eventID: 2; samplingProtocol: Sweeping; eventTime: Day
- **Type status:** Other material. **Occurrence:** individualCount: 1; sex: female; **Location:** locationID: S1; continent: Europe; country: Spain; countryCode: ES; stateProvince: Andalucía; county: Granada; locality: Soportujar; verbatimElevation: 1786.57; decimalLatitude: 36.96151; decimalLongitude: -3.41881; geodeticDatum: WGS84; **Event:** eventID: 1; samplingProtocol: Beating; eventTime: Day
- **Type status:** Other material. **Occurrence:** individualCount: 1; sex: female; **Location:** locationID: S1; continent: Europe; country: Spain; countryCode: ES; stateProvince: Andalucía; county: Granada; locality: Soportujar; verbatimElevation: 1786.57; decimalLatitude: 36.96151; decimalLongitude: -3.41881; geodeticDatum: WGS84; **Event:** eventID: 2; samplingProtocol: Beating; eventTime: Day
- **Type status:** Other material. **Occurrence:** individualCount: 1; sex: female; **Location:** locationID: S1; continent: Europe; country: Spain; countryCode: ES; stateProvince: Andalucía; county: Granada; locality: Soportujar; verbatimElevation: 1786.57; decimalLatitude: 36.96151; decimalLongitude: -3.41881; geodeticDatum: WGS84; **Event:** eventID: 2; samplingProtocol: Beating; eventTime: Night
- **Type status:** Other material. **Occurrence:** individualCount: 1; sex: female; **Location:** locationID: S2; continent: Europe; country: Spain; countryCode: ES; stateProvince: Andalucía; county: Granada; locality: Camarate; verbatimElevation: 1713.96; decimalLatitude: 37.18377; decimalLongitude: -3.26282; geodeticDatum: WGS84; **Event:** eventID: 2; samplingProtocol: Aerial; eventTime: Night
- **Type status:** Other material. **Occurrence:** individualCount: 2; sex: female; **Location:** locationID: S2; continent: Europe; country: Spain; countryCode: ES; stateProvince: Andalucía; county: Granada; locality: Camarate; verbatimElevation: 1713.96; decimalLatitude: 37.18377; decimalLongitude: -3.26282; geodeticDatum: WGS84; **Event:** eventID: 1; samplingProtocol: Beating; eventTime: Day
- **Type status:** Other material. **Occurrence:** individualCount: 1; sex: female; **Location:** locationID: S2; continent: Europe; country: Spain; countryCode: ES; stateProvince: Andalucía; county: Granada; locality: Camarate; verbatimElevation: 1713.96; decimalLatitude: 37.18377; decimalLongitude: -3.26282; geodeticDatum: WGS84; **Event:** eventID: 2; samplingProtocol: Beating; eventTime: Day
- **Type status:** Other material. **Occurrence:** individualCount: 2; sex: female; **Location:** locationID: S2; continent: Europe; country: Spain; countryCode: ES; stateProvince: Andalucía; county: Granada; locality: Camarate; verbatimElevation: 1713.96; decimalLatitude: 37.18377; decimalLongitude: -3.26282; geodeticDatum: WGS84; **Event:** eventID: 2; samplingProtocol: Beating; eventTime: Night
- **Type status:** Other material. **Occurrence:** individualCount: 1; sex: female; **Location:** locationID: S2; continent: Europe; country: Spain; countryCode: ES; stateProvince: Andalucía; county: Granada; locality: Camarate; verbatimElevation: 1713.96; decimalLatitude: 37.18377; decimalLongitude: -3.26282; geodeticDatum: WGS84; **Event:** eventID: 1; samplingProtocol: Sweeping; eventTime: Night

##### Distribution

?

##### Notes

This morph of *Philodromus* Thorell, 1870, for which only female specimens were available, defied identification based on morphology. However, these specimens formed a distinct genetic cluster (see *Species delimitation and identification using DNA barcodes*). We tentatively refer to this morph as sp. 18 although further scrutiny may reveal it actually corresponds to either a nominal species not reported in our study or a new species.

#### Pulchellodromus bistigma

(Simon, 1870)

##### Materials

- **Type status:** Other material. **Occurrence:** individualCount: 1; sex: male; **Location:** locationID: C1; continent: Europe; country: Spain; countryCode: ES; stateProvince: Castilla-La Mancha; county: Ciudad Real; locality: Valle Brezoso; verbatimElevation: 756.56; decimalLatitude: 39.35663; decimalLongitude: -4.35912; geodeticDatum: WGS84; **Event:** eventID: J; samplingProtocol: Pitfall
- **Type status:** Other material. **Occurrence:** individualCount: 1; sex: male; **Location:** locationID: M1; continent: Europe; country: Spain; countryCode: ES; stateProvince: Extremadura; county: Cáceres; locality: Peña Falcón; verbatimElevation: 320.6; decimalLatitude: 39.83296; decimalLongitude: -6.0641; geodeticDatum: WGS84; **Event:** eventID: B; samplingProtocol: Pitfall
- **Type status:** Other material. **Occurrence:** individualCount: 2; sex: male; **Location:** locationID: M1; continent: Europe; country: Spain; countryCode: ES; stateProvince: Extremadura; county: Cáceres; locality: Peña Falcón; verbatimElevation: 320.6; decimalLatitude: 39.83296; decimalLongitude: -6.0641; geodeticDatum: WGS84; **Event:** eventID: C; samplingProtocol: Pitfall
- **Type status:** Other material. **Occurrence:** individualCount: 2; sex: male; **Location:** locationID: M1; continent: Europe; country: Spain; countryCode: ES; stateProvince: Extremadura; county: Cáceres; locality: Peña Falcón; verbatimElevation: 320.6; decimalLatitude: 39.83296; decimalLongitude: -6.0641; geodeticDatum: WGS84; **Event:** eventID: D; samplingProtocol: Pitfall
- **Type status:** Other material. **Occurrence:** individualCount: 1; sex: male; **Location:** locationID: M1; continent: Europe; country: Spain; countryCode: ES; stateProvince: Extremadura; county: Cáceres; locality: Peña Falcón; verbatimElevation: 320.6; decimalLatitude: 39.83296; decimalLongitude: -6.0641; geodeticDatum: WGS84; **Event:** eventID: E; samplingProtocol: Pitfall
- **Type status:** Other material. **Occurrence:** individualCount: 1; sex: male; **Location:** locationID: M1; continent: Europe; country: Spain; countryCode: ES; stateProvince: Extremadura; county: Cáceres; locality: Peña Falcón; verbatimElevation: 320.6; decimalLatitude: 39.83296; decimalLongitude: -6.0641; geodeticDatum: WGS84; **Event:** eventID: F; samplingProtocol: Pitfall
- **Type status:** Other material. **Occurrence:** individualCount: 1; sex: male; **Location:** locationID: M1; continent: Europe; country: Spain; countryCode: ES; stateProvince: Extremadura; county: Cáceres; locality: Peña Falcón; verbatimElevation: 320.6; decimalLatitude: 39.83296; decimalLongitude: -6.0641; geodeticDatum: WGS84; **Event:** eventID: J; samplingProtocol: Pitfall
- **Type status:** Other material. **Occurrence:** individualCount: 1; sex: male; **Location:** locationID: M2; continent: Europe; country: Spain; countryCode: ES; stateProvince: Extremadura; county: Cáceres; locality: Fuente del Frances; verbatimElevation: 320.72; decimalLatitude: 39.828; decimalLongitude: -6.03249; geodeticDatum: WGS84; **Event:** eventID: B; samplingProtocol: Pitfall
- **Type status:** Other material. **Occurrence:** individualCount: 1; sex: male; **Location:** locationID: M2; continent: Europe; country: Spain; countryCode: ES; stateProvince: Extremadura; county: Cáceres; locality: Fuente del Frances; verbatimElevation: 320.72; decimalLatitude: 39.828; decimalLongitude: -6.03249; geodeticDatum: WGS84; **Event:** eventID: F; samplingProtocol: Pitfall
- **Type status:** Other material. **Occurrence:** individualCount: 1; sex: male; **Location:** locationID: M2; continent: Europe; country: Spain; countryCode: ES; stateProvince: Extremadura; county: Cáceres; locality: Fuente del Frances; verbatimElevation: 320.72; decimalLatitude: 39.828; decimalLongitude: -6.03249; geodeticDatum: WGS84; **Event:** eventID: H; samplingProtocol: Pitfall
- **Type status:** Other material. **Occurrence:** individualCount: 1; sex: male; **Location:** locationID: S2; continent: Europe; country: Spain; countryCode: ES; stateProvince: Andalucía; county: Granada; locality: Camarate; verbatimElevation: 1713.96; decimalLatitude: 37.18377; decimalLongitude: -3.26282; geodeticDatum: WGS84; **Event:** eventID: B; samplingProtocol: Pitfall
- **Type status:** Other material. **Occurrence:** individualCount: 2; sex: male; **Location:** locationID: S2; continent: Europe; country: Spain; countryCode: ES; stateProvince: Andalucía; county: Granada; locality: Camarate; verbatimElevation: 1713.96; decimalLatitude: 37.18377; decimalLongitude: -3.26282; geodeticDatum: WGS84; **Event:** eventID: L; samplingProtocol: Pitfall

##### Distribution

Mediterranean

#### Pulchellodromus glaucinus

(Simon, 1870)

##### Materials

- **Type status:** Other material. **Occurrence:** individualCount: 1; sex: female; **Location:** locationID: C3; continent: Europe; country: Spain; countryCode: ES; stateProvince: Castilla-La Mancha; county: Ciudad Real; locality: La Quesera; verbatimElevation: 767.55; decimalLatitude: 39.36177; decimalLongitude: -4.41733; geodeticDatum: WGS84; **Event:** eventID: 1; samplingProtocol: Sweeping; eventTime: Day
- **Type status:** Other material. **Occurrence:** individualCount: 1; sex: female; **Location:** locationID: C4; continent: Europe; country: Spain; countryCode: ES; stateProvince: Castilla-La Mancha; county: Ciudad Real; locality: La Quesera; verbatimElevation: 772.3; decimalLatitude: 39.36337; decimalLongitude: -4.41704; geodeticDatum: WGS84; **Event:** eventID: 1; samplingProtocol: Sweeping; eventTime: Day
- **Type status:** Other material. **Occurrence:** individualCount: 1; sex: female; **Location:** locationID: C4; continent: Europe; country: Spain; countryCode: ES; stateProvince: Castilla-La Mancha; county: Ciudad Real; locality: La Quesera; verbatimElevation: 772.3; decimalLatitude: 39.36337; decimalLongitude: -4.41704; geodeticDatum: WGS84; **Event:** eventID: 2; samplingProtocol: Sweeping; eventTime: Day

##### Distribution

Mediterranean

#### Pulchellodromus pulchellus

(Lucas, 1846)

##### Materials

- **Type status:** Other material. **Occurrence:** individualCount: 1; sex: female; **Location:** locationID: S2; continent: Europe; country: Spain; countryCode: ES; stateProvince: Andalucía; county: Granada; locality: Camarate; verbatimElevation: 1713.96; decimalLatitude: 37.18377; decimalLongitude: -3.26282; geodeticDatum: WGS84; **Event:** eventID: 1; samplingProtocol: Sweeping; eventTime: Day
- **Type status:** Other material. **Occurrence:** individualCount: 1; sex: male; **Location:** locationID: S2; continent: Europe; country: Spain; countryCode: ES; stateProvince: Andalucía; county: Granada; locality: Camarate; verbatimElevation: 1713.96; decimalLatitude: 37.18377; decimalLongitude: -3.26282; geodeticDatum: WGS84; **Event:** eventID: 1; samplingProtocol: Sweeping; eventTime: Day

##### Distribution

Mediterranean

#### Pulchellodromus simoni

(Mello-Leitão, 1929)

##### Materials

- **Type status:** Other material. **Occurrence:** individualCount: 1; sex: female; **Location:** locationID: C2; continent: Europe; country: Spain; countryCode: ES; stateProvince: Castilla-La Mancha; county: Ciudad Real; locality: Valle Brezoso; verbatimElevation: 739.31; decimalLatitude: 39.35159; decimalLongitude: -4.3589; geodeticDatum: WGS84; **Event:** eventID: F; samplingProtocol: Pitfall
- **Type status:** Other material. **Occurrence:** individualCount: 1; sex: female; **Location:** locationID: C2; continent: Europe; country: Spain; countryCode: ES; stateProvince: Castilla-La Mancha; county: Ciudad Real; locality: Valle Brezoso; verbatimElevation: 739.31; decimalLatitude: 39.35159; decimalLongitude: -4.3589; geodeticDatum: WGS84; **Event:** eventID: 1; samplingProtocol: Sweeping; eventTime: Day
- **Type status:** Other material. **Occurrence:** individualCount: 1; sex: female; **Location:** locationID: C3; continent: Europe; country: Spain; countryCode: ES; stateProvince: Castilla-La Mancha; county: Ciudad Real; locality: La Quesera; verbatimElevation: 767.55; decimalLatitude: 39.36177; decimalLongitude: -4.41733; geodeticDatum: WGS84; **Event:** eventID: 1; samplingProtocol: Beating; eventTime: Day
- **Type status:** Other material. **Occurrence:** individualCount: 1; sex: female; **Location:** locationID: C4; continent: Europe; country: Spain; countryCode: ES; stateProvince: Castilla-La Mancha; county: Ciudad Real; locality: La Quesera; verbatimElevation: 772.3; decimalLatitude: 39.36337; decimalLongitude: -4.41704; geodeticDatum: WGS84; **Event:** eventID: 4; samplingProtocol: Aerial; eventTime: Night
- **Type status:** Other material. **Occurrence:** individualCount: 1; sex: female; **Location:** locationID: C4; continent: Europe; country: Spain; countryCode: ES; stateProvince: Castilla-La Mancha; county: Ciudad Real; locality: La Quesera; verbatimElevation: 772.3; decimalLatitude: 39.36337; decimalLongitude: -4.41704; geodeticDatum: WGS84; **Event:** eventID: 1; samplingProtocol: Beating; eventTime: Day
- **Type status:** Other material. **Occurrence:** individualCount: 1; sex: female; **Location:** locationID: C4; continent: Europe; country: Spain; countryCode: ES; stateProvince: Castilla-La Mancha; county: Ciudad Real; locality: La Quesera; verbatimElevation: 772.3; decimalLatitude: 39.36337; decimalLongitude: -4.41704; geodeticDatum: WGS84; **Event:** eventID: 1; samplingProtocol: Beating; eventTime: Night

##### Distribution

Spain, Algeria

#### Thanatus atratus

Simon, 1875

##### Materials

- **Type status:** Other material. **Occurrence:** individualCount: 2; sex: male; **Location:** locationID: S1; continent: Europe; country: Spain; countryCode: ES; stateProvince: Andalucía; county: Granada; locality: Soportujar; verbatimElevation: 1786.57; decimalLatitude: 36.96151; decimalLongitude: -3.41881; geodeticDatum: WGS84; **Event:** eventID: E; samplingProtocol: Pitfall
- **Type status:** Other material. **Occurrence:** individualCount: 7; sex: male; **Location:** locationID: S1; continent: Europe; country: Spain; countryCode: ES; stateProvince: Andalucía; county: Granada; locality: Soportujar; verbatimElevation: 1786.57; decimalLatitude: 36.96151; decimalLongitude: -3.41881; geodeticDatum: WGS84; **Event:** eventID: F; samplingProtocol: Pitfall
- **Type status:** Other material. **Occurrence:** individualCount: 4; sex: male; **Location:** locationID: S1; continent: Europe; country: Spain; countryCode: ES; stateProvince: Andalucía; county: Granada; locality: Soportujar; verbatimElevation: 1786.57; decimalLatitude: 36.96151; decimalLongitude: -3.41881; geodeticDatum: WGS84; **Event:** eventID: H; samplingProtocol: Pitfall
- **Type status:** Other material. **Occurrence:** individualCount: 1; sex: female; **Location:** locationID: S1; continent: Europe; country: Spain; countryCode: ES; stateProvince: Andalucía; county: Granada; locality: Soportujar; verbatimElevation: 1786.57; decimalLatitude: 36.96151; decimalLongitude: -3.41881; geodeticDatum: WGS84; **Event:** eventID: K; samplingProtocol: Pitfall
- **Type status:** Other material. **Occurrence:** individualCount: 3; sex: male; **Location:** locationID: S1; continent: Europe; country: Spain; countryCode: ES; stateProvince: Andalucía; county: Granada; locality: Soportujar; verbatimElevation: 1786.57; decimalLatitude: 36.96151; decimalLongitude: -3.41881; geodeticDatum: WGS84; **Event:** eventID: K; samplingProtocol: Pitfall
- **Type status:** Other material. **Occurrence:** individualCount: 1; sex: female; **Location:** locationID: S1; continent: Europe; country: Spain; countryCode: ES; stateProvince: Andalucía; county: Granada; locality: Soportujar; verbatimElevation: 1786.57; decimalLatitude: 36.96151; decimalLongitude: -3.41881; geodeticDatum: WGS84; **Event:** eventID: L; samplingProtocol: Pitfall
- **Type status:** Other material. **Occurrence:** individualCount: 5; sex: male; **Location:** locationID: S1; continent: Europe; country: Spain; countryCode: ES; stateProvince: Andalucía; county: Granada; locality: Soportujar; verbatimElevation: 1786.57; decimalLatitude: 36.96151; decimalLongitude: -3.41881; geodeticDatum: WGS84; **Event:** eventID: L; samplingProtocol: Pitfall
- **Type status:** Other material. **Occurrence:** individualCount: 2; sex: female; **Location:** locationID: S2; continent: Europe; country: Spain; countryCode: ES; stateProvince: Andalucía; county: Granada; locality: Camarate; verbatimElevation: 1713.96; decimalLatitude: 37.18377; decimalLongitude: -3.26282; geodeticDatum: WGS84; **Event:** eventID: 2; samplingProtocol: Beating; eventTime: Night
- **Type status:** Other material. **Occurrence:** individualCount: 1; sex: female; **Location:** locationID: S2; continent: Europe; country: Spain; countryCode: ES; stateProvince: Andalucía; county: Granada; locality: Camarate; verbatimElevation: 1713.96; decimalLatitude: 37.18377; decimalLongitude: -3.26282; geodeticDatum: WGS84; **Event:** eventID: C; samplingProtocol: Pitfall
- **Type status:** Other material. **Occurrence:** individualCount: 1; sex: male; **Location:** locationID: S2; continent: Europe; country: Spain; countryCode: ES; stateProvince: Andalucía; county: Granada; locality: Camarate; verbatimElevation: 1713.96; decimalLatitude: 37.18377; decimalLongitude: -3.26282; geodeticDatum: WGS84; **Event:** eventID: L; samplingProtocol: Pitfall
- **Type status:** Other material. **Occurrence:** individualCount: 1; sex: female; **Location:** locationID: S2; continent: Europe; country: Spain; countryCode: ES; stateProvince: Andalucía; county: Granada; locality: Camarate; verbatimElevation: 1713.96; decimalLatitude: 37.18377; decimalLongitude: -3.26282; geodeticDatum: WGS84; **Event:** eventID: 1; samplingProtocol: Sweeping; eventTime: Night
- **Type status:** Other material. **Occurrence:** individualCount: 3; sex: male; **Location:** locationID: S2; continent: Europe; country: Spain; countryCode: ES; stateProvince: Andalucía; county: Granada; locality: Camarate; verbatimElevation: 1713.96; decimalLatitude: 37.18377; decimalLongitude: -3.26282; geodeticDatum: WGS84; **Event:** eventID: 1; samplingProtocol: Sweeping; eventTime: Night
- **Type status:** Other material. **Occurrence:** individualCount: 1; sex: female; **Location:** locationID: S2; continent: Europe; country: Spain; countryCode: ES; stateProvince: Andalucía; county: Granada; locality: Camarate; verbatimElevation: 1713.96; decimalLatitude: 37.18377; decimalLongitude: -3.26282; geodeticDatum: WGS84; **Event:** eventID: 2; samplingProtocol: Sweeping; eventTime: Night
- **Type status:** Other material. **Occurrence:** individualCount: 1; sex: male; **Location:** locationID: S2; continent: Europe; country: Spain; countryCode: ES; stateProvince: Andalucía; county: Granada; locality: Camarate; verbatimElevation: 1713.96; decimalLatitude: 37.18377; decimalLongitude: -3.26282; geodeticDatum: WGS84; **Event:** eventID: 2; samplingProtocol: Sweeping; eventTime: Night

##### Distribution

Palearctic

#### Thanatus oblongiusculus

(Lucas, 1846)

##### Materials

- **Type status:** Other material. **Occurrence:** individualCount: 1; sex: female; **Location:** locationID: S2; continent: Europe; country: Spain; countryCode: ES; stateProvince: Andalucía; county: Granada; locality: Camarate; verbatimElevation: 1713.96; decimalLatitude: 37.18377; decimalLongitude: -3.26282; geodeticDatum: WGS84; **Event:** eventID: 4; samplingProtocol: Aerial; eventTime: Night
- **Type status:** Other material. **Occurrence:** individualCount: 1; sex: male; **Location:** locationID: S2; continent: Europe; country: Spain; countryCode: ES; stateProvince: Andalucía; county: Granada; locality: Camarate; verbatimElevation: 1713.96; decimalLatitude: 37.18377; decimalLongitude: -3.26282; geodeticDatum: WGS84; **Event:** eventID: 1; samplingProtocol: Sweeping; eventTime: Day
- **Type status:** Other material. **Occurrence:** individualCount: 1; sex: female; **Location:** locationID: S2; continent: Europe; country: Spain; countryCode: ES; stateProvince: Andalucía; county: Granada; locality: Camarate; verbatimElevation: 1713.96; decimalLatitude: 37.18377; decimalLongitude: -3.26282; geodeticDatum: WGS84; **Event:** eventID: 1; samplingProtocol: Sweeping; eventTime: Night
- **Type status:** Other material. **Occurrence:** individualCount: 1; sex: male; **Location:** locationID: S2; continent: Europe; country: Spain; countryCode: ES; stateProvince: Andalucía; county: Granada; locality: Camarate; verbatimElevation: 1713.96; decimalLatitude: 37.18377; decimalLongitude: -3.26282; geodeticDatum: WGS84; **Event:** eventID: 1; samplingProtocol: Sweeping; eventTime: Night

##### Distribution

Mediterranean

#### Thanatus vulgaris

Simon, 1870

##### Materials

- **Type status:** Other material. **Occurrence:** individualCount: 1; sex: female; **Location:** locationID: C3; continent: Europe; country: Spain; countryCode: ES; stateProvince: Castilla-La Mancha; county: Ciudad Real; locality: La Quesera; verbatimElevation: 767.55; decimalLatitude: 39.36177; decimalLongitude: -4.41733; geodeticDatum: WGS84; **Event:** eventID: A; samplingProtocol: Pitfall
- **Type status:** Other material. **Occurrence:** individualCount: 3; sex: male; **Location:** locationID: C3; continent: Europe; country: Spain; countryCode: ES; stateProvince: Castilla-La Mancha; county: Ciudad Real; locality: La Quesera; verbatimElevation: 767.55; decimalLatitude: 39.36177; decimalLongitude: -4.41733; geodeticDatum: WGS84; **Event:** eventID: A; samplingProtocol: Pitfall
- **Type status:** Other material. **Occurrence:** individualCount: 3; sex: male; **Location:** locationID: C3; continent: Europe; country: Spain; countryCode: ES; stateProvince: Castilla-La Mancha; county: Ciudad Real; locality: La Quesera; verbatimElevation: 767.55; decimalLatitude: 39.36177; decimalLongitude: -4.41733; geodeticDatum: WGS84; **Event:** eventID: C; samplingProtocol: Pitfall
- **Type status:** Other material. **Occurrence:** individualCount: 3; sex: male; **Location:** locationID: C3; continent: Europe; country: Spain; countryCode: ES; stateProvince: Castilla-La Mancha; county: Ciudad Real; locality: La Quesera; verbatimElevation: 767.55; decimalLatitude: 39.36177; decimalLongitude: -4.41733; geodeticDatum: WGS84; **Event:** eventID: G; samplingProtocol: Pitfall
- **Type status:** Other material. **Occurrence:** individualCount: 2; sex: male; **Location:** locationID: C3; continent: Europe; country: Spain; countryCode: ES; stateProvince: Castilla-La Mancha; county: Ciudad Real; locality: La Quesera; verbatimElevation: 767.55; decimalLatitude: 39.36177; decimalLongitude: -4.41733; geodeticDatum: WGS84; **Event:** eventID: I; samplingProtocol: Pitfall
- **Type status:** Other material. **Occurrence:** individualCount: 1; sex: male; **Location:** locationID: C4; continent: Europe; country: Spain; countryCode: ES; stateProvince: Castilla-La Mancha; county: Ciudad Real; locality: La Quesera; verbatimElevation: 772.3; decimalLatitude: 39.36337; decimalLongitude: -4.41704; geodeticDatum: WGS84; **Event:** eventID: B; samplingProtocol: Pitfall
- **Type status:** Other material. **Occurrence:** individualCount: 1; sex: female; **Location:** locationID: C4; continent: Europe; country: Spain; countryCode: ES; stateProvince: Castilla-La Mancha; county: Ciudad Real; locality: La Quesera; verbatimElevation: 772.3; decimalLatitude: 39.36337; decimalLongitude: -4.41704; geodeticDatum: WGS84; **Event:** eventID: 2; samplingProtocol: Sweeping; eventTime: Day

##### Distribution

Holarctic

#### Tibellus oblongus

(Walckenaer, 1802)

##### Materials

- **Type status:** Other material. **Occurrence:** individualCount: 1; sex: female; **Location:** locationID: C4; continent: Europe; country: Spain; countryCode: ES; stateProvince: Castilla-La Mancha; county: Ciudad Real; locality: La Quesera; verbatimElevation: 772.3; decimalLatitude: 39.36337; decimalLongitude: -4.41704; geodeticDatum: WGS84; **Event:** eventID: 1; samplingProtocol: Aerial; eventTime: Night
- **Type status:** Other material. **Occurrence:** individualCount: 1; sex: female; **Location:** locationID: C4; continent: Europe; country: Spain; countryCode: ES; stateProvince: Castilla-La Mancha; county: Ciudad Real; locality: La Quesera; verbatimElevation: 772.3; decimalLatitude: 39.36337; decimalLongitude: -4.41704; geodeticDatum: WGS84; **Event:** eventID: 1; samplingProtocol: Sweeping; eventTime: Day
- **Type status:** Other material. **Occurrence:** individualCount: 1; sex: male; **Location:** locationID: C4; continent: Europe; country: Spain; countryCode: ES; stateProvince: Castilla-La Mancha; county: Ciudad Real; locality: La Quesera; verbatimElevation: 772.3; decimalLatitude: 39.36337; decimalLongitude: -4.41704; geodeticDatum: WGS84; **Event:** eventID: 1; samplingProtocol: Sweeping; eventTime: Day
- **Type status:** Other material. **Occurrence:** individualCount: 1; sex: female; **Location:** locationID: C4; continent: Europe; country: Spain; countryCode: ES; stateProvince: Castilla-La Mancha; county: Ciudad Real; locality: La Quesera; verbatimElevation: 772.3; decimalLatitude: 39.36337; decimalLongitude: -4.41704; geodeticDatum: WGS84; **Event:** eventID: 2; samplingProtocol: Sweeping; eventTime: Day
- **Type status:** Other material. **Occurrence:** individualCount: 2; sex: female; **Location:** locationID: C4; continent: Europe; country: Spain; countryCode: ES; stateProvince: Castilla-La Mancha; county: Ciudad Real; locality: La Quesera; verbatimElevation: 772.3; decimalLatitude: 39.36337; decimalLongitude: -4.41704; geodeticDatum: WGS84; **Event:** eventID: 1; samplingProtocol: Sweeping; eventTime: Night
- **Type status:** Other material. **Occurrence:** individualCount: 1; sex: male; **Location:** locationID: C4; continent: Europe; country: Spain; countryCode: ES; stateProvince: Castilla-La Mancha; county: Ciudad Real; locality: La Quesera; verbatimElevation: 772.3; decimalLatitude: 39.36337; decimalLongitude: -4.41704; geodeticDatum: WGS84; **Event:** eventID: 2; samplingProtocol: Sweeping; eventTime: Night
- **Type status:** Other material. **Occurrence:** individualCount: 1; sex: male; **Location:** locationID: P2; continent: Europe; country: Spain; countryCode: ES; stateProvince: Castilla y León; county: León; locality: Joyoguelas; verbatimElevation: 763.98; decimalLatitude: 43.17771; decimalLongitude: -4.90579; geodeticDatum: WGS84; **Event:** eventID: 1; samplingProtocol: Sweeping; eventTime: Night
- **Type status:** Other material. **Occurrence:** individualCount: 1; sex: female; **Location:** locationID: P4; continent: Europe; country: Spain; countryCode: ES; stateProvince: Castilla y León; county: León; locality: El Canto; verbatimElevation: 943.48; decimalLatitude: 43.17227; decimalLongitude: -4.90857; geodeticDatum: WGS84; **Event:** eventID: 1; samplingProtocol: Beating; eventTime: Night

##### Distribution

Holarctic

#### Pholcidae

C. L. Koch, 1850

#### Holocnemus caudatus

(Dufour, 1820)

##### Materials

- **Type status:** Other material. **Occurrence:** individualCount: 1; sex: female; **Location:** locationID: S1; continent: Europe; country: Spain; countryCode: ES; stateProvince: Andalucía; county: Granada; locality: Soportujar; verbatimElevation: 1786.57; decimalLatitude: 36.96151; decimalLongitude: -3.41881; geodeticDatum: WGS84; **Event:** eventID: 3; samplingProtocol: Aerial; eventTime: Night

##### Distribution

Spain, Sicily

#### Holocnemus hispanicus

Wiehle, 1933

##### Materials

- **Type status:** Other material. **Occurrence:** individualCount: 1; sex: male; **Location:** locationID: M1; continent: Europe; country: Spain; countryCode: ES; stateProvince: Extremadura; county: Cáceres; locality: Peña Falcón; verbatimElevation: 320.6; decimalLatitude: 39.83296; decimalLongitude: -6.0641; geodeticDatum: WGS84; **Event:** eventID: 3; samplingProtocol: Aerial; eventTime: Night
- **Type status:** Other material. **Occurrence:** individualCount: 1; sex: male; **Location:** locationID: M2; continent: Europe; country: Spain; countryCode: ES; stateProvince: Extremadura; county: Cáceres; locality: Fuente del Frances; verbatimElevation: 320.72; decimalLatitude: 39.828; decimalLongitude: -6.03249; geodeticDatum: WGS84; **Event:** eventID: C; samplingProtocol: Pitfall

##### Distribution

Iberian Peninsula

#### Pholcus opilionoides

(Schrank, 1781)

##### Materials

- **Type status:** Other material. **Occurrence:** individualCount: 1; sex: male; **Location:** locationID: O2; continent: Europe; country: Spain; countryCode: ES; stateProvince: Aragón; county: Huesca; locality: Rebilla; verbatimElevation: 1158.13; decimalLatitude: 42.59427; decimalLongitude: 0.1529; geodeticDatum: WGS84; **Event:** eventID: 2; samplingProtocol: Aerial; eventTime: Night

##### Distribution

Europe to Azerbaijan

#### Phrurolithidae

Banks, 1892

#### Liophrurillus flavitarsis

(Lucas, 1846)

##### Materials

- **Type status:** Other material. **Occurrence:** individualCount: 1; sex: female; **Location:** locationID: C1; continent: Europe; country: Spain; countryCode: ES; stateProvince: Castilla-La Mancha; county: Ciudad Real; locality: Valle Brezoso; verbatimElevation: 756.56; decimalLatitude: 39.35663; decimalLongitude: -4.35912; geodeticDatum: WGS84; **Event:** eventID: B; samplingProtocol: Pitfall
- **Type status:** Other material. **Occurrence:** individualCount: 1; sex: female; **Location:** locationID: C1; continent: Europe; country: Spain; countryCode: ES; stateProvince: Castilla-La Mancha; county: Ciudad Real; locality: Valle Brezoso; verbatimElevation: 756.56; decimalLatitude: 39.35663; decimalLongitude: -4.35912; geodeticDatum: WGS84; **Event:** eventID: C; samplingProtocol: Pitfall
- **Type status:** Other material. **Occurrence:** individualCount: 2; sex: female; **Location:** locationID: C1; continent: Europe; country: Spain; countryCode: ES; stateProvince: Castilla-La Mancha; county: Ciudad Real; locality: Valle Brezoso; verbatimElevation: 756.56; decimalLatitude: 39.35663; decimalLongitude: -4.35912; geodeticDatum: WGS84; **Event:** eventID: D; samplingProtocol: Pitfall
- **Type status:** Other material. **Occurrence:** individualCount: 1; sex: female; **Location:** locationID: C1; continent: Europe; country: Spain; countryCode: ES; stateProvince: Castilla-La Mancha; county: Ciudad Real; locality: Valle Brezoso; verbatimElevation: 756.56; decimalLatitude: 39.35663; decimalLongitude: -4.35912; geodeticDatum: WGS84; **Event:** eventID: E; samplingProtocol: Pitfall
- **Type status:** Other material. **Occurrence:** individualCount: 2; sex: female; **Location:** locationID: C1; continent: Europe; country: Spain; countryCode: ES; stateProvince: Castilla-La Mancha; county: Ciudad Real; locality: Valle Brezoso; verbatimElevation: 756.56; decimalLatitude: 39.35663; decimalLongitude: -4.35912; geodeticDatum: WGS84; **Event:** eventID: F; samplingProtocol: Pitfall
- **Type status:** Other material. **Occurrence:** individualCount: 2; sex: female; **Location:** locationID: C1; continent: Europe; country: Spain; countryCode: ES; stateProvince: Castilla-La Mancha; county: Ciudad Real; locality: Valle Brezoso; verbatimElevation: 756.56; decimalLatitude: 39.35663; decimalLongitude: -4.35912; geodeticDatum: WGS84; **Event:** eventID: G; samplingProtocol: Pitfall
- **Type status:** Other material. **Occurrence:** individualCount: 2; sex: female; **Location:** locationID: C1; continent: Europe; country: Spain; countryCode: ES; stateProvince: Castilla-La Mancha; county: Ciudad Real; locality: Valle Brezoso; verbatimElevation: 756.56; decimalLatitude: 39.35663; decimalLongitude: -4.35912; geodeticDatum: WGS84; **Event:** eventID: H; samplingProtocol: Pitfall
- **Type status:** Other material. **Occurrence:** individualCount: 2; sex: female; **Location:** locationID: C1; continent: Europe; country: Spain; countryCode: ES; stateProvince: Castilla-La Mancha; county: Ciudad Real; locality: Valle Brezoso; verbatimElevation: 756.56; decimalLatitude: 39.35663; decimalLongitude: -4.35912; geodeticDatum: WGS84; **Event:** eventID: I; samplingProtocol: Pitfall
- **Type status:** Other material. **Occurrence:** individualCount: 10; sex: female; **Location:** locationID: C1; continent: Europe; country: Spain; countryCode: ES; stateProvince: Castilla-La Mancha; county: Ciudad Real; locality: Valle Brezoso; verbatimElevation: 756.56; decimalLatitude: 39.35663; decimalLongitude: -4.35912; geodeticDatum: WGS84; **Event:** eventID: J; samplingProtocol: Pitfall
- **Type status:** Other material. **Occurrence:** individualCount: 2; sex: female; **Location:** locationID: C1; continent: Europe; country: Spain; countryCode: ES; stateProvince: Castilla-La Mancha; county: Ciudad Real; locality: Valle Brezoso; verbatimElevation: 756.56; decimalLatitude: 39.35663; decimalLongitude: -4.35912; geodeticDatum: WGS84; **Event:** eventID: K; samplingProtocol: Pitfall
- **Type status:** Other material. **Occurrence:** individualCount: 3; sex: female; **Location:** locationID: C1; continent: Europe; country: Spain; countryCode: ES; stateProvince: Castilla-La Mancha; county: Ciudad Real; locality: Valle Brezoso; verbatimElevation: 756.56; decimalLatitude: 39.35663; decimalLongitude: -4.35912; geodeticDatum: WGS84; **Event:** eventID: L; samplingProtocol: Pitfall
- **Type status:** Other material. **Occurrence:** individualCount: 1; sex: male; **Location:** locationID: C1; continent: Europe; country: Spain; countryCode: ES; stateProvince: Castilla-La Mancha; county: Ciudad Real; locality: Valle Brezoso; verbatimElevation: 756.56; decimalLatitude: 39.35663; decimalLongitude: -4.35912; geodeticDatum: WGS84; **Event:** eventID: L; samplingProtocol: Pitfall
- **Type status:** Other material. **Occurrence:** individualCount: 5; sex: female; **Location:** locationID: C2; continent: Europe; country: Spain; countryCode: ES; stateProvince: Castilla-La Mancha; county: Ciudad Real; locality: Valle Brezoso; verbatimElevation: 739.31; decimalLatitude: 39.35159; decimalLongitude: -4.3589; geodeticDatum: WGS84; **Event:** eventID: A; samplingProtocol: Pitfall
- **Type status:** Other material. **Occurrence:** individualCount: 2; sex: female; **Location:** locationID: C2; continent: Europe; country: Spain; countryCode: ES; stateProvince: Castilla-La Mancha; county: Ciudad Real; locality: Valle Brezoso; verbatimElevation: 739.31; decimalLatitude: 39.35159; decimalLongitude: -4.3589; geodeticDatum: WGS84; **Event:** eventID: B; samplingProtocol: Pitfall
- **Type status:** Other material. **Occurrence:** individualCount: 2; sex: female; **Location:** locationID: C2; continent: Europe; country: Spain; countryCode: ES; stateProvince: Castilla-La Mancha; county: Ciudad Real; locality: Valle Brezoso; verbatimElevation: 739.31; decimalLatitude: 39.35159; decimalLongitude: -4.3589; geodeticDatum: WGS84; **Event:** eventID: D; samplingProtocol: Pitfall
- **Type status:** Other material. **Occurrence:** individualCount: 2; sex: female; **Location:** locationID: C2; continent: Europe; country: Spain; countryCode: ES; stateProvince: Castilla-La Mancha; county: Ciudad Real; locality: Valle Brezoso; verbatimElevation: 739.31; decimalLatitude: 39.35159; decimalLongitude: -4.3589; geodeticDatum: WGS84; **Event:** eventID: E; samplingProtocol: Pitfall
- **Type status:** Other material. **Occurrence:** individualCount: 1; sex: female; **Location:** locationID: C2; continent: Europe; country: Spain; countryCode: ES; stateProvince: Castilla-La Mancha; county: Ciudad Real; locality: Valle Brezoso; verbatimElevation: 739.31; decimalLatitude: 39.35159; decimalLongitude: -4.3589; geodeticDatum: WGS84; **Event:** eventID: F; samplingProtocol: Pitfall
- **Type status:** Other material. **Occurrence:** individualCount: 2; sex: female; **Location:** locationID: C2; continent: Europe; country: Spain; countryCode: ES; stateProvince: Castilla-La Mancha; county: Ciudad Real; locality: Valle Brezoso; verbatimElevation: 739.31; decimalLatitude: 39.35159; decimalLongitude: -4.3589; geodeticDatum: WGS84; **Event:** eventID: H; samplingProtocol: Pitfall
- **Type status:** Other material. **Occurrence:** individualCount: 2; sex: female; **Location:** locationID: C2; continent: Europe; country: Spain; countryCode: ES; stateProvince: Castilla-La Mancha; county: Ciudad Real; locality: Valle Brezoso; verbatimElevation: 739.31; decimalLatitude: 39.35159; decimalLongitude: -4.3589; geodeticDatum: WGS84; **Event:** eventID: K; samplingProtocol: Pitfall
- **Type status:** Other material. **Occurrence:** individualCount: 1; sex: female; **Location:** locationID: C3; continent: Europe; country: Spain; countryCode: ES; stateProvince: Castilla-La Mancha; county: Ciudad Real; locality: La Quesera; verbatimElevation: 767.55; decimalLatitude: 39.36177; decimalLongitude: -4.41733; geodeticDatum: WGS84; **Event:** eventID: A; samplingProtocol: Pitfall
- **Type status:** Other material. **Occurrence:** individualCount: 2; sex: female; **Location:** locationID: C3; continent: Europe; country: Spain; countryCode: ES; stateProvince: Castilla-La Mancha; county: Ciudad Real; locality: La Quesera; verbatimElevation: 767.55; decimalLatitude: 39.36177; decimalLongitude: -4.41733; geodeticDatum: WGS84; **Event:** eventID: C; samplingProtocol: Pitfall
- **Type status:** Other material. **Occurrence:** individualCount: 2; sex: female; **Location:** locationID: C3; continent: Europe; country: Spain; countryCode: ES; stateProvince: Castilla-La Mancha; county: Ciudad Real; locality: La Quesera; verbatimElevation: 767.55; decimalLatitude: 39.36177; decimalLongitude: -4.41733; geodeticDatum: WGS84; **Event:** eventID: E; samplingProtocol: Pitfall
- **Type status:** Other material. **Occurrence:** individualCount: 1; sex: female; **Location:** locationID: C3; continent: Europe; country: Spain; countryCode: ES; stateProvince: Castilla-La Mancha; county: Ciudad Real; locality: La Quesera; verbatimElevation: 767.55; decimalLatitude: 39.36177; decimalLongitude: -4.41733; geodeticDatum: WGS84; **Event:** eventID: F; samplingProtocol: Pitfall
- **Type status:** Other material. **Occurrence:** individualCount: 2; sex: female; **Location:** locationID: C3; continent: Europe; country: Spain; countryCode: ES; stateProvince: Castilla-La Mancha; county: Ciudad Real; locality: La Quesera; verbatimElevation: 767.55; decimalLatitude: 39.36177; decimalLongitude: -4.41733; geodeticDatum: WGS84; **Event:** eventID: H; samplingProtocol: Pitfall
- **Type status:** Other material. **Occurrence:** individualCount: 2; sex: female; **Location:** locationID: C3; continent: Europe; country: Spain; countryCode: ES; stateProvince: Castilla-La Mancha; county: Ciudad Real; locality: La Quesera; verbatimElevation: 767.55; decimalLatitude: 39.36177; decimalLongitude: -4.41733; geodeticDatum: WGS84; **Event:** eventID: I; samplingProtocol: Pitfall
- **Type status:** Other material. **Occurrence:** individualCount: 2; sex: female; **Location:** locationID: C3; continent: Europe; country: Spain; countryCode: ES; stateProvince: Castilla-La Mancha; county: Ciudad Real; locality: La Quesera; verbatimElevation: 767.55; decimalLatitude: 39.36177; decimalLongitude: -4.41733; geodeticDatum: WGS84; **Event:** eventID: J; samplingProtocol: Pitfall
- **Type status:** Other material. **Occurrence:** individualCount: 1; sex: female; **Location:** locationID: C3; continent: Europe; country: Spain; countryCode: ES; stateProvince: Castilla-La Mancha; county: Ciudad Real; locality: La Quesera; verbatimElevation: 767.55; decimalLatitude: 39.36177; decimalLongitude: -4.41733; geodeticDatum: WGS84; **Event:** eventID: K; samplingProtocol: Pitfall
- **Type status:** Other material. **Occurrence:** individualCount: 2; sex: female; **Location:** locationID: C3; continent: Europe; country: Spain; countryCode: ES; stateProvince: Castilla-La Mancha; county: Ciudad Real; locality: La Quesera; verbatimElevation: 767.55; decimalLatitude: 39.36177; decimalLongitude: -4.41733; geodeticDatum: WGS84; **Event:** eventID: L; samplingProtocol: Pitfall
- **Type status:** Other material. **Occurrence:** individualCount: 1; sex: female; **Location:** locationID: C4; continent: Europe; country: Spain; countryCode: ES; stateProvince: Castilla-La Mancha; county: Ciudad Real; locality: La Quesera; verbatimElevation: 772.3; decimalLatitude: 39.36337; decimalLongitude: -4.41704; geodeticDatum: WGS84; **Event:** eventID: C; samplingProtocol: Pitfall
- **Type status:** Other material. **Occurrence:** individualCount: 1; sex: female; **Location:** locationID: C4; continent: Europe; country: Spain; countryCode: ES; stateProvince: Castilla-La Mancha; county: Ciudad Real; locality: La Quesera; verbatimElevation: 772.3; decimalLatitude: 39.36337; decimalLongitude: -4.41704; geodeticDatum: WGS84; **Event:** eventID: D; samplingProtocol: Pitfall
- **Type status:** Other material. **Occurrence:** individualCount: 1; sex: female; **Location:** locationID: C4; continent: Europe; country: Spain; countryCode: ES; stateProvince: Castilla-La Mancha; county: Ciudad Real; locality: La Quesera; verbatimElevation: 772.3; decimalLatitude: 39.36337; decimalLongitude: -4.41704; geodeticDatum: WGS84; **Event:** eventID: E; samplingProtocol: Pitfall
- **Type status:** Other material. **Occurrence:** individualCount: 3; sex: female; **Location:** locationID: C4; continent: Europe; country: Spain; countryCode: ES; stateProvince: Castilla-La Mancha; county: Ciudad Real; locality: La Quesera; verbatimElevation: 772.3; decimalLatitude: 39.36337; decimalLongitude: -4.41704; geodeticDatum: WGS84; **Event:** eventID: F; samplingProtocol: Pitfall
- **Type status:** Other material. **Occurrence:** individualCount: 1; sex: female; **Location:** locationID: C4; continent: Europe; country: Spain; countryCode: ES; stateProvince: Castilla-La Mancha; county: Ciudad Real; locality: La Quesera; verbatimElevation: 772.3; decimalLatitude: 39.36337; decimalLongitude: -4.41704; geodeticDatum: WGS84; **Event:** eventID: H; samplingProtocol: Pitfall
- **Type status:** Other material. **Occurrence:** individualCount: 1; sex: female; **Location:** locationID: C4; continent: Europe; country: Spain; countryCode: ES; stateProvince: Castilla-La Mancha; county: Ciudad Real; locality: La Quesera; verbatimElevation: 772.3; decimalLatitude: 39.36337; decimalLongitude: -4.41704; geodeticDatum: WGS84; **Event:** eventID: J; samplingProtocol: Pitfall
- **Type status:** Other material. **Occurrence:** individualCount: 2; sex: female; **Location:** locationID: C4; continent: Europe; country: Spain; countryCode: ES; stateProvince: Castilla-La Mancha; county: Ciudad Real; locality: La Quesera; verbatimElevation: 772.3; decimalLatitude: 39.36337; decimalLongitude: -4.41704; geodeticDatum: WGS84; **Event:** eventID: K; samplingProtocol: Pitfall

##### Distribution

Europe, Madeira, North Africa

#### Phrurolinillus lisboensis

Wunderlich, 1995

##### Materials

- **Type status:** Other material. **Occurrence:** individualCount: 1; sex: male; **Location:** locationID: C1; continent: Europe; country: Spain; countryCode: ES; stateProvince: Castilla-La Mancha; county: Ciudad Real; locality: Valle Brezoso; verbatimElevation: 756.56; decimalLatitude: 39.35663; decimalLongitude: -4.35912; geodeticDatum: WGS84; **Event:** eventID: B; samplingProtocol: Pitfall
- **Type status:** Other material. **Occurrence:** individualCount: 1; sex: female; **Location:** locationID: C1; continent: Europe; country: Spain; countryCode: ES; stateProvince: Castilla-La Mancha; county: Ciudad Real; locality: Valle Brezoso; verbatimElevation: 756.56; decimalLatitude: 39.35663; decimalLongitude: -4.35912; geodeticDatum: WGS84; **Event:** eventID: C; samplingProtocol: Pitfall
- **Type status:** Other material. **Occurrence:** individualCount: 1; sex: male; **Location:** locationID: C1; continent: Europe; country: Spain; countryCode: ES; stateProvince: Castilla-La Mancha; county: Ciudad Real; locality: Valle Brezoso; verbatimElevation: 756.56; decimalLatitude: 39.35663; decimalLongitude: -4.35912; geodeticDatum: WGS84; **Event:** eventID: E; samplingProtocol: Pitfall
- **Type status:** Other material. **Occurrence:** individualCount: 1; sex: male; **Location:** locationID: C1; continent: Europe; country: Spain; countryCode: ES; stateProvince: Castilla-La Mancha; county: Ciudad Real; locality: Valle Brezoso; verbatimElevation: 756.56; decimalLatitude: 39.35663; decimalLongitude: -4.35912; geodeticDatum: WGS84; **Event:** eventID: J; samplingProtocol: Pitfall
- **Type status:** Other material. **Occurrence:** individualCount: 1; sex: female; **Location:** locationID: C1; continent: Europe; country: Spain; countryCode: ES; stateProvince: Castilla-La Mancha; county: Ciudad Real; locality: Valle Brezoso; verbatimElevation: 756.56; decimalLatitude: 39.35663; decimalLongitude: -4.35912; geodeticDatum: WGS84; **Event:** eventID: K; samplingProtocol: Pitfall
- **Type status:** Other material. **Occurrence:** individualCount: 1; sex: female; **Location:** locationID: C1; continent: Europe; country: Spain; countryCode: ES; stateProvince: Castilla-La Mancha; county: Ciudad Real; locality: Valle Brezoso; verbatimElevation: 756.56; decimalLatitude: 39.35663; decimalLongitude: -4.35912; geodeticDatum: WGS84; **Event:** eventID: L; samplingProtocol: Pitfall

##### Distribution

Iberian Peninsula

#### Phrurolinillus tibialis

(Simon, 1878)

##### Materials

- **Type status:** Other material. **Occurrence:** individualCount: 1; sex: female; **Location:** locationID: S1; continent: Europe; country: Spain; countryCode: ES; stateProvince: Andalucía; county: Granada; locality: Soportujar; verbatimElevation: 1786.57; decimalLatitude: 36.96151; decimalLongitude: -3.41881; geodeticDatum: WGS84; **Event:** eventID: G; samplingProtocol: Pitfall
- **Type status:** Other material. **Occurrence:** individualCount: 2; sex: male; **Location:** locationID: S1; continent: Europe; country: Spain; countryCode: ES; stateProvince: Andalucía; county: Granada; locality: Soportujar; verbatimElevation: 1786.57; decimalLatitude: 36.96151; decimalLongitude: -3.41881; geodeticDatum: WGS84; **Event:** eventID: G; samplingProtocol: Pitfall
- **Type status:** Other material. **Occurrence:** individualCount: 2; sex: male; **Location:** locationID: S1; continent: Europe; country: Spain; countryCode: ES; stateProvince: Andalucía; county: Granada; locality: Soportujar; verbatimElevation: 1786.57; decimalLatitude: 36.96151; decimalLongitude: -3.41881; geodeticDatum: WGS84; **Event:** eventID: I; samplingProtocol: Pitfall
- **Type status:** Other material. **Occurrence:** individualCount: 1; sex: female; **Location:** locationID: S1; continent: Europe; country: Spain; countryCode: ES; stateProvince: Andalucía; county: Granada; locality: Soportujar; verbatimElevation: 1786.57; decimalLatitude: 36.96151; decimalLongitude: -3.41881; geodeticDatum: WGS84; **Event:** eventID: J; samplingProtocol: Pitfall
- **Type status:** Other material. **Occurrence:** individualCount: 4; sex: male; **Location:** locationID: S1; continent: Europe; country: Spain; countryCode: ES; stateProvince: Andalucía; county: Granada; locality: Soportujar; verbatimElevation: 1786.57; decimalLatitude: 36.96151; decimalLongitude: -3.41881; geodeticDatum: WGS84; **Event:** eventID: J; samplingProtocol: Pitfall
- **Type status:** Other material. **Occurrence:** individualCount: 2; sex: male; **Location:** locationID: S1; continent: Europe; country: Spain; countryCode: ES; stateProvince: Andalucía; county: Granada; locality: Soportujar; verbatimElevation: 1786.57; decimalLatitude: 36.96151; decimalLongitude: -3.41881; geodeticDatum: WGS84; **Event:** eventID: K; samplingProtocol: Pitfall
- **Type status:** Other material. **Occurrence:** individualCount: 5; sex: male; **Location:** locationID: S1; continent: Europe; country: Spain; countryCode: ES; stateProvince: Andalucía; county: Granada; locality: Soportujar; verbatimElevation: 1786.57; decimalLatitude: 36.96151; decimalLongitude: -3.41881; geodeticDatum: WGS84; **Event:** eventID: L; samplingProtocol: Pitfall
- **Type status:** Other material. **Occurrence:** individualCount: 1; sex: male; **Location:** locationID: S2; continent: Europe; country: Spain; countryCode: ES; stateProvince: Andalucía; county: Granada; locality: Camarate; verbatimElevation: 1713.96; decimalLatitude: 37.18377; decimalLongitude: -3.26282; geodeticDatum: WGS84; **Event:** eventID: D; samplingProtocol: Pitfall
- **Type status:** Other material. **Occurrence:** individualCount: 2; sex: male; **Location:** locationID: S2; continent: Europe; country: Spain; countryCode: ES; stateProvince: Andalucía; county: Granada; locality: Camarate; verbatimElevation: 1713.96; decimalLatitude: 37.18377; decimalLongitude: -3.26282; geodeticDatum: WGS84; **Event:** eventID: E; samplingProtocol: Pitfall
- **Type status:** Other material. **Occurrence:** individualCount: 2; sex: male; **Location:** locationID: S2; continent: Europe; country: Spain; countryCode: ES; stateProvince: Andalucía; county: Granada; locality: Camarate; verbatimElevation: 1713.96; decimalLatitude: 37.18377; decimalLongitude: -3.26282; geodeticDatum: WGS84; **Event:** eventID: H; samplingProtocol: Pitfall
- **Type status:** Other material. **Occurrence:** individualCount: 1; sex: male; **Location:** locationID: S2; continent: Europe; country: Spain; countryCode: ES; stateProvince: Andalucía; county: Granada; locality: Camarate; verbatimElevation: 1713.96; decimalLatitude: 37.18377; decimalLongitude: -3.26282; geodeticDatum: WGS84; **Event:** eventID: I; samplingProtocol: Pitfall

##### Distribution

Iberian Peninsula

##### Notes

**Figure 10.**
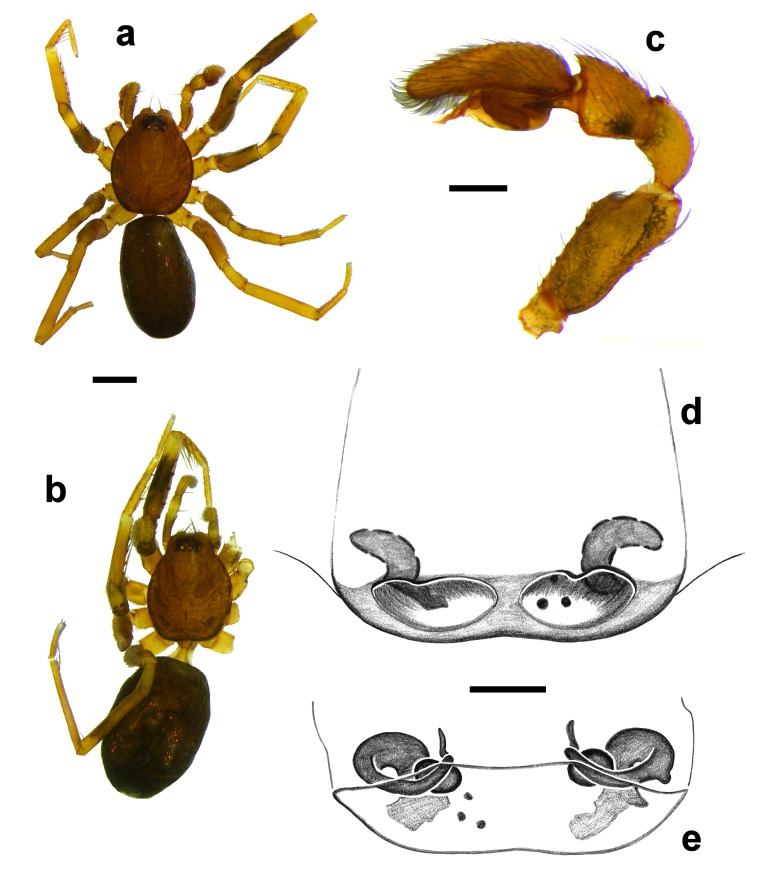
*Phrurolinillus tibialis* (Simon, 1878). a: male, dorsal habitus, scale = 0.5 mm; b: female, dorsal habitus, scale = 0.5 mm; c: left male palp, retrolateral, scale = 0.2 mm; d: female epigynum, ventral, scale = 0.1 mm; e: female vulva, dorsal, scale = 0.1 mm.

This is the first occurrence of females of this species. We hereby illustrate the epigyne for the first time (Fig. 10d), composed of two roughly ellipsoid and only lightly sclerotised depressions (several irregularly shaped exudates can be seen) and the vulva (Fig. 10), composed by two copulatory ducts that coil dorsally towards small spermathecae, from which the fertilisation ducts can also be seen.

#### Phrurolithus festivus

(C. L. Koch, 1835)

##### Materials

- **Type status:** Other material. **Occurrence:** individualCount: 1; sex: male; **Location:** locationID: A1; continent: Europe; country: Spain; countryCode: ES; stateProvince: Catalonia; county: Lleida; locality: Sola de Boi; verbatimElevation: 1759.8; decimalLatitude: 42.54958; decimalLongitude: 0.87254; geodeticDatum: WGS84; **Event:** eventID: 2; samplingProtocol: Beating; eventTime: Day
- **Type status:** Other material. **Occurrence:** individualCount: 2; sex: male; **Location:** locationID: A1; continent: Europe; country: Spain; countryCode: ES; stateProvince: Catalonia; county: Lleida; locality: Sola de Boi; verbatimElevation: 1759.8; decimalLatitude: 42.54958; decimalLongitude: 0.87254; geodeticDatum: WGS84; **Event:** eventID: 1; samplingProtocol: Ground; eventTime: Night
- **Type status:** Other material. **Occurrence:** individualCount: 1; sex: female; **Location:** locationID: A1; continent: Europe; country: Spain; countryCode: ES; stateProvince: Catalonia; county: Lleida; locality: Sola de Boi; verbatimElevation: 1759.8; decimalLatitude: 42.54958; decimalLongitude: 0.87254; geodeticDatum: WGS84; **Event:** eventID: 1; samplingProtocol: Ground; eventTime: Night
- **Type status:** Other material. **Occurrence:** individualCount: 2; sex: male; **Location:** locationID: A1; continent: Europe; country: Spain; countryCode: ES; stateProvince: Catalonia; county: Lleida; locality: Sola de Boi; verbatimElevation: 1759.8; decimalLatitude: 42.54958; decimalLongitude: 0.87254; geodeticDatum: WGS84; **Event:** eventID: 2; samplingProtocol: Ground; eventTime: Night
- **Type status:** Other material. **Occurrence:** individualCount: 1; sex: female; **Location:** locationID: A1; continent: Europe; country: Spain; countryCode: ES; stateProvince: Catalonia; county: Lleida; locality: Sola de Boi; verbatimElevation: 1759.8; decimalLatitude: 42.54958; decimalLongitude: 0.87254; geodeticDatum: WGS84; **Event:** eventID: 2; samplingProtocol: Ground; eventTime: Night
- **Type status:** Other material. **Occurrence:** individualCount: 4; sex: male; **Location:** locationID: A1; continent: Europe; country: Spain; countryCode: ES; stateProvince: Catalonia; county: Lleida; locality: Sola de Boi; verbatimElevation: 1759.8; decimalLatitude: 42.54958; decimalLongitude: 0.87254; geodeticDatum: WGS84; **Event:** eventID: A; samplingProtocol: Pitfall
- **Type status:** Other material. **Occurrence:** individualCount: 3; sex: male; **Location:** locationID: A1; continent: Europe; country: Spain; countryCode: ES; stateProvince: Catalonia; county: Lleida; locality: Sola de Boi; verbatimElevation: 1759.8; decimalLatitude: 42.54958; decimalLongitude: 0.87254; geodeticDatum: WGS84; **Event:** eventID: B; samplingProtocol: Pitfall
- **Type status:** Other material. **Occurrence:** individualCount: 3; sex: male; **Location:** locationID: A1; continent: Europe; country: Spain; countryCode: ES; stateProvince: Catalonia; county: Lleida; locality: Sola de Boi; verbatimElevation: 1759.8; decimalLatitude: 42.54958; decimalLongitude: 0.87254; geodeticDatum: WGS84; **Event:** eventID: C; samplingProtocol: Pitfall
- **Type status:** Other material. **Occurrence:** individualCount: 2; sex: female; **Location:** locationID: A1; continent: Europe; country: Spain; countryCode: ES; stateProvince: Catalonia; county: Lleida; locality: Sola de Boi; verbatimElevation: 1759.8; decimalLatitude: 42.54958; decimalLongitude: 0.87254; geodeticDatum: WGS84; **Event:** eventID: E; samplingProtocol: Pitfall
- **Type status:** Other material. **Occurrence:** individualCount: 1; sex: male; **Location:** locationID: A1; continent: Europe; country: Spain; countryCode: ES; stateProvince: Catalonia; county: Lleida; locality: Sola de Boi; verbatimElevation: 1759.8; decimalLatitude: 42.54958; decimalLongitude: 0.87254; geodeticDatum: WGS84; **Event:** eventID: E; samplingProtocol: Pitfall
- **Type status:** Other material. **Occurrence:** individualCount: 1; sex: female; **Location:** locationID: A1; continent: Europe; country: Spain; countryCode: ES; stateProvince: Catalonia; county: Lleida; locality: Sola de Boi; verbatimElevation: 1759.8; decimalLatitude: 42.54958; decimalLongitude: 0.87254; geodeticDatum: WGS84; **Event:** eventID: F; samplingProtocol: Pitfall
- **Type status:** Other material. **Occurrence:** individualCount: 1; sex: male; **Location:** locationID: A1; continent: Europe; country: Spain; countryCode: ES; stateProvince: Catalonia; county: Lleida; locality: Sola de Boi; verbatimElevation: 1759.8; decimalLatitude: 42.54958; decimalLongitude: 0.87254; geodeticDatum: WGS84; **Event:** eventID: F; samplingProtocol: Pitfall
- **Type status:** Other material. **Occurrence:** individualCount: 2; sex: male; **Location:** locationID: A1; continent: Europe; country: Spain; countryCode: ES; stateProvince: Catalonia; county: Lleida; locality: Sola de Boi; verbatimElevation: 1759.8; decimalLatitude: 42.54958; decimalLongitude: 0.87254; geodeticDatum: WGS84; **Event:** eventID: G; samplingProtocol: Pitfall
- **Type status:** Other material. **Occurrence:** individualCount: 1; sex: female; **Location:** locationID: A1; continent: Europe; country: Spain; countryCode: ES; stateProvince: Catalonia; county: Lleida; locality: Sola de Boi; verbatimElevation: 1759.8; decimalLatitude: 42.54958; decimalLongitude: 0.87254; geodeticDatum: WGS84; **Event:** eventID: H; samplingProtocol: Pitfall
- **Type status:** Other material. **Occurrence:** individualCount: 1; sex: male; **Location:** locationID: A1; continent: Europe; country: Spain; countryCode: ES; stateProvince: Catalonia; county: Lleida; locality: Sola de Boi; verbatimElevation: 1759.8; decimalLatitude: 42.54958; decimalLongitude: 0.87254; geodeticDatum: WGS84; **Event:** eventID: I; samplingProtocol: Pitfall
- **Type status:** Other material. **Occurrence:** individualCount: 1; sex: male; **Location:** locationID: A1; continent: Europe; country: Spain; countryCode: ES; stateProvince: Catalonia; county: Lleida; locality: Sola de Boi; verbatimElevation: 1759.8; decimalLatitude: 42.54958; decimalLongitude: 0.87254; geodeticDatum: WGS84; **Event:** eventID: J; samplingProtocol: Pitfall
- **Type status:** Other material. **Occurrence:** individualCount: 3; sex: male; **Location:** locationID: A1; continent: Europe; country: Spain; countryCode: ES; stateProvince: Catalonia; county: Lleida; locality: Sola de Boi; verbatimElevation: 1759.8; decimalLatitude: 42.54958; decimalLongitude: 0.87254; geodeticDatum: WGS84; **Event:** eventID: L; samplingProtocol: Pitfall
- **Type status:** Other material. **Occurrence:** individualCount: 2; sex: male; **Location:** locationID: A2; continent: Europe; country: Spain; countryCode: ES; stateProvince: Catalonia; county: Lleida; locality: Sola de Boi; verbatimElevation: 1738.7; decimalLatitude: 42.54913; decimalLongitude: 0.87137; geodeticDatum: WGS84; **Event:** eventID: A; samplingProtocol: Pitfall
- **Type status:** Other material. **Occurrence:** individualCount: 1; sex: male; **Location:** locationID: A2; continent: Europe; country: Spain; countryCode: ES; stateProvince: Catalonia; county: Lleida; locality: Sola de Boi; verbatimElevation: 1738.7; decimalLatitude: 42.54913; decimalLongitude: 0.87137; geodeticDatum: WGS84; **Event:** eventID: C; samplingProtocol: Pitfall
- **Type status:** Other material. **Occurrence:** individualCount: 1; sex: male; **Location:** locationID: A2; continent: Europe; country: Spain; countryCode: ES; stateProvince: Catalonia; county: Lleida; locality: Sola de Boi; verbatimElevation: 1738.7; decimalLatitude: 42.54913; decimalLongitude: 0.87137; geodeticDatum: WGS84; **Event:** eventID: D; samplingProtocol: Pitfall
- **Type status:** Other material. **Occurrence:** individualCount: 1; sex: male; **Location:** locationID: A2; continent: Europe; country: Spain; countryCode: ES; stateProvince: Catalonia; county: Lleida; locality: Sola de Boi; verbatimElevation: 1738.7; decimalLatitude: 42.54913; decimalLongitude: 0.87137; geodeticDatum: WGS84; **Event:** eventID: E; samplingProtocol: Pitfall
- **Type status:** Other material. **Occurrence:** individualCount: 1; sex: male; **Location:** locationID: A2; continent: Europe; country: Spain; countryCode: ES; stateProvince: Catalonia; county: Lleida; locality: Sola de Boi; verbatimElevation: 1738.7; decimalLatitude: 42.54913; decimalLongitude: 0.87137; geodeticDatum: WGS84; **Event:** eventID: G; samplingProtocol: Pitfall
- **Type status:** Other material. **Occurrence:** individualCount: 5; sex: male; **Location:** locationID: A2; continent: Europe; country: Spain; countryCode: ES; stateProvince: Catalonia; county: Lleida; locality: Sola de Boi; verbatimElevation: 1738.7; decimalLatitude: 42.54913; decimalLongitude: 0.87137; geodeticDatum: WGS84; **Event:** eventID: J; samplingProtocol: Pitfall
- **Type status:** Other material. **Occurrence:** individualCount: 2; sex: male; **Location:** locationID: A2; continent: Europe; country: Spain; countryCode: ES; stateProvince: Catalonia; county: Lleida; locality: Sola de Boi; verbatimElevation: 1738.7; decimalLatitude: 42.54913; decimalLongitude: 0.87137; geodeticDatum: WGS84; **Event:** eventID: L; samplingProtocol: Pitfall
- **Type status:** Other material. **Occurrence:** individualCount: 1; sex: male; **Location:** locationID: P2; continent: Europe; country: Spain; countryCode: ES; stateProvince: Castilla y León; county: León; locality: Joyoguelas; verbatimElevation: 763.98; decimalLatitude: 43.17771; decimalLongitude: -4.90579; geodeticDatum: WGS84; **Event:** eventID: I; samplingProtocol: Pitfall

##### Distribution

Palearctic

#### Phrurolithus minimus

C. L. Koch, 1839

##### Materials

- **Type status:** Other material. **Occurrence:** individualCount: 1; sex: female; **Location:** locationID: P2; continent: Europe; country: Spain; countryCode: ES; stateProvince: Castilla y León; county: León; locality: Joyoguelas; verbatimElevation: 763.98; decimalLatitude: 43.17771; decimalLongitude: -4.90579; geodeticDatum: WGS84; **Event:** eventID: 1; samplingProtocol: Aerial; eventTime: Night
- **Type status:** Other material. **Occurrence:** individualCount: 1; sex: male; **Location:** locationID: P2; continent: Europe; country: Spain; countryCode: ES; stateProvince: Castilla y León; county: León; locality: Joyoguelas; verbatimElevation: 763.98; decimalLatitude: 43.17771; decimalLongitude: -4.90579; geodeticDatum: WGS84; **Event:** eventID: D; samplingProtocol: Pitfall
- **Type status:** Other material. **Occurrence:** individualCount: 1; sex: male; **Location:** locationID: P2; continent: Europe; country: Spain; countryCode: ES; stateProvince: Castilla y León; county: León; locality: Joyoguelas; verbatimElevation: 763.98; decimalLatitude: 43.17771; decimalLongitude: -4.90579; geodeticDatum: WGS84; **Event:** eventID: H; samplingProtocol: Pitfall
- **Type status:** Other material. **Occurrence:** individualCount: 1; sex: female; **Location:** locationID: P3; continent: Europe; country: Spain; countryCode: ES; stateProvince: Castilla y León; county: León; locality: Las Arroyas; verbatimElevation: 1097.1; decimalLatitude: 43.14351; decimalLongitude: -4.94878; geodeticDatum: WGS84; **Event:** eventID: E; samplingProtocol: Pitfall
- **Type status:** Other material. **Occurrence:** individualCount: 2; sex: male; **Location:** locationID: P3; continent: Europe; country: Spain; countryCode: ES; stateProvince: Castilla y León; county: León; locality: Las Arroyas; verbatimElevation: 1097.1; decimalLatitude: 43.14351; decimalLongitude: -4.94878; geodeticDatum: WGS84; **Event:** eventID: I; samplingProtocol: Pitfall
- **Type status:** Other material. **Occurrence:** individualCount: 1; sex: male; **Location:** locationID: P4; continent: Europe; country: Spain; countryCode: ES; stateProvince: Castilla y León; county: León; locality: El Canto; verbatimElevation: 943.48; decimalLatitude: 43.17227; decimalLongitude: -4.90857; geodeticDatum: WGS84; **Event:** eventID: A; samplingProtocol: Pitfall
- **Type status:** Other material. **Occurrence:** individualCount: 1; sex: male; **Location:** locationID: P4; continent: Europe; country: Spain; countryCode: ES; stateProvince: Castilla y León; county: León; locality: El Canto; verbatimElevation: 943.48; decimalLatitude: 43.17227; decimalLongitude: -4.90857; geodeticDatum: WGS84; **Event:** eventID: B; samplingProtocol: Pitfall
- **Type status:** Other material. **Occurrence:** individualCount: 1; sex: female; **Location:** locationID: P4; continent: Europe; country: Spain; countryCode: ES; stateProvince: Castilla y León; county: León; locality: El Canto; verbatimElevation: 943.48; decimalLatitude: 43.17227; decimalLongitude: -4.90857; geodeticDatum: WGS84; **Event:** eventID: E; samplingProtocol: Pitfall
- **Type status:** Other material. **Occurrence:** individualCount: 6; sex: male; **Location:** locationID: P4; continent: Europe; country: Spain; countryCode: ES; stateProvince: Castilla y León; county: León; locality: El Canto; verbatimElevation: 943.48; decimalLatitude: 43.17227; decimalLongitude: -4.90857; geodeticDatum: WGS84; **Event:** eventID: E; samplingProtocol: Pitfall
- **Type status:** Other material. **Occurrence:** individualCount: 1; sex: female; **Location:** locationID: P4; continent: Europe; country: Spain; countryCode: ES; stateProvince: Castilla y León; county: León; locality: El Canto; verbatimElevation: 943.48; decimalLatitude: 43.17227; decimalLongitude: -4.90857; geodeticDatum: WGS84; **Event:** eventID: F; samplingProtocol: Pitfall
- **Type status:** Other material. **Occurrence:** individualCount: 1; sex: female; **Location:** locationID: P4; continent: Europe; country: Spain; countryCode: ES; stateProvince: Castilla y León; county: León; locality: El Canto; verbatimElevation: 943.48; decimalLatitude: 43.17227; decimalLongitude: -4.90857; geodeticDatum: WGS84; **Event:** eventID: G; samplingProtocol: Pitfall
- **Type status:** Other material. **Occurrence:** individualCount: 1; sex: male; **Location:** locationID: P4; continent: Europe; country: Spain; countryCode: ES; stateProvince: Castilla y León; county: León; locality: El Canto; verbatimElevation: 943.48; decimalLatitude: 43.17227; decimalLongitude: -4.90857; geodeticDatum: WGS84; **Event:** eventID: H; samplingProtocol: Pitfall
- **Type status:** Other material. **Occurrence:** individualCount: 1; sex: female; **Location:** locationID: P4; continent: Europe; country: Spain; countryCode: ES; stateProvince: Castilla y León; county: León; locality: El Canto; verbatimElevation: 943.48; decimalLatitude: 43.17227; decimalLongitude: -4.90857; geodeticDatum: WGS84; **Event:** eventID: L; samplingProtocol: Pitfall

##### Distribution

Europe

#### Phrurolithus nigrinus

(Simon, 1878)

##### Materials

- **Type status:** Other material. **Occurrence:** individualCount: 1; sex: female; **Location:** locationID: O2; continent: Europe; country: Spain; countryCode: ES; stateProvince: Aragón; county: Huesca; locality: Rebilla; verbatimElevation: 1158.13; decimalLatitude: 42.59427; decimalLongitude: 0.1529; geodeticDatum: WGS84; **Event:** eventID: K; samplingProtocol: Pitfall

##### Distribution

Central and Southern Europe

#### Phrurolithus szilyi

Herman, 1879

##### Materials

- **Type status:** Other material. **Occurrence:** individualCount: 1; sex: male; **Location:** locationID: A1; continent: Europe; country: Spain; countryCode: ES; stateProvince: Catalonia; county: Lleida; locality: Sola de Boi; verbatimElevation: 1759.8; decimalLatitude: 42.54958; decimalLongitude: 0.87254; geodeticDatum: WGS84; **Event:** eventID: E; samplingProtocol: Pitfall
- **Type status:** Other material. **Occurrence:** individualCount: 1; sex: male; **Location:** locationID: A1; continent: Europe; country: Spain; countryCode: ES; stateProvince: Catalonia; county: Lleida; locality: Sola de Boi; verbatimElevation: 1759.8; decimalLatitude: 42.54958; decimalLongitude: 0.87254; geodeticDatum: WGS84; **Event:** eventID: F; samplingProtocol: Pitfall
- **Type status:** Other material. **Occurrence:** individualCount: 2; sex: male; **Location:** locationID: A1; continent: Europe; country: Spain; countryCode: ES; stateProvince: Catalonia; county: Lleida; locality: Sola de Boi; verbatimElevation: 1759.8; decimalLatitude: 42.54958; decimalLongitude: 0.87254; geodeticDatum: WGS84; **Event:** eventID: G; samplingProtocol: Pitfall
- **Type status:** Other material. **Occurrence:** individualCount: 1; sex: male; **Location:** locationID: A1; continent: Europe; country: Spain; countryCode: ES; stateProvince: Catalonia; county: Lleida; locality: Sola de Boi; verbatimElevation: 1759.8; decimalLatitude: 42.54958; decimalLongitude: 0.87254; geodeticDatum: WGS84; **Event:** eventID: I; samplingProtocol: Pitfall
- **Type status:** Other material. **Occurrence:** individualCount: 1; sex: male; **Location:** locationID: A2; continent: Europe; country: Spain; countryCode: ES; stateProvince: Catalonia; county: Lleida; locality: Sola de Boi; verbatimElevation: 1738.7; decimalLatitude: 42.54913; decimalLongitude: 0.87137; geodeticDatum: WGS84; **Event:** eventID: A; samplingProtocol: Pitfall
- **Type status:** Other material. **Occurrence:** individualCount: 1; sex: male; **Location:** locationID: A2; continent: Europe; country: Spain; countryCode: ES; stateProvince: Catalonia; county: Lleida; locality: Sola de Boi; verbatimElevation: 1738.7; decimalLatitude: 42.54913; decimalLongitude: 0.87137; geodeticDatum: WGS84; **Event:** eventID: H; samplingProtocol: Pitfall
- **Type status:** Other material. **Occurrence:** individualCount: 3; sex: male; **Location:** locationID: C1; continent: Europe; country: Spain; countryCode: ES; stateProvince: Castilla-La Mancha; county: Ciudad Real; locality: Valle Brezoso; verbatimElevation: 756.56; decimalLatitude: 39.35663; decimalLongitude: -4.35912; geodeticDatum: WGS84; **Event:** eventID: B; samplingProtocol: Pitfall
- **Type status:** Other material. **Occurrence:** individualCount: 2; sex: male; **Location:** locationID: C1; continent: Europe; country: Spain; countryCode: ES; stateProvince: Castilla-La Mancha; county: Ciudad Real; locality: Valle Brezoso; verbatimElevation: 756.56; decimalLatitude: 39.35663; decimalLongitude: -4.35912; geodeticDatum: WGS84; **Event:** eventID: C; samplingProtocol: Pitfall
- **Type status:** Other material. **Occurrence:** individualCount: 1; sex: male; **Location:** locationID: C1; continent: Europe; country: Spain; countryCode: ES; stateProvince: Castilla-La Mancha; county: Ciudad Real; locality: Valle Brezoso; verbatimElevation: 756.56; decimalLatitude: 39.35663; decimalLongitude: -4.35912; geodeticDatum: WGS84; **Event:** eventID: D; samplingProtocol: Pitfall
- **Type status:** Other material. **Occurrence:** individualCount: 5; sex: male; **Location:** locationID: C1; continent: Europe; country: Spain; countryCode: ES; stateProvince: Castilla-La Mancha; county: Ciudad Real; locality: Valle Brezoso; verbatimElevation: 756.56; decimalLatitude: 39.35663; decimalLongitude: -4.35912; geodeticDatum: WGS84; **Event:** eventID: E; samplingProtocol: Pitfall
- **Type status:** Other material. **Occurrence:** individualCount: 1; sex: female; **Location:** locationID: C1; continent: Europe; country: Spain; countryCode: ES; stateProvince: Castilla-La Mancha; county: Ciudad Real; locality: Valle Brezoso; verbatimElevation: 756.56; decimalLatitude: 39.35663; decimalLongitude: -4.35912; geodeticDatum: WGS84; **Event:** eventID: F; samplingProtocol: Pitfall
- **Type status:** Other material. **Occurrence:** individualCount: 1; sex: male; **Location:** locationID: C1; continent: Europe; country: Spain; countryCode: ES; stateProvince: Castilla-La Mancha; county: Ciudad Real; locality: Valle Brezoso; verbatimElevation: 756.56; decimalLatitude: 39.35663; decimalLongitude: -4.35912; geodeticDatum: WGS84; **Event:** eventID: F; samplingProtocol: Pitfall
- **Type status:** Other material. **Occurrence:** individualCount: 3; sex: male; **Location:** locationID: C1; continent: Europe; country: Spain; countryCode: ES; stateProvince: Castilla-La Mancha; county: Ciudad Real; locality: Valle Brezoso; verbatimElevation: 756.56; decimalLatitude: 39.35663; decimalLongitude: -4.35912; geodeticDatum: WGS84; **Event:** eventID: G; samplingProtocol: Pitfall
- **Type status:** Other material. **Occurrence:** individualCount: 1; sex: female; **Location:** locationID: C1; continent: Europe; country: Spain; countryCode: ES; stateProvince: Castilla-La Mancha; county: Ciudad Real; locality: Valle Brezoso; verbatimElevation: 756.56; decimalLatitude: 39.35663; decimalLongitude: -4.35912; geodeticDatum: WGS84; **Event:** eventID: H; samplingProtocol: Pitfall
- **Type status:** Other material. **Occurrence:** individualCount: 8; sex: male; **Location:** locationID: C1; continent: Europe; country: Spain; countryCode: ES; stateProvince: Castilla-La Mancha; county: Ciudad Real; locality: Valle Brezoso; verbatimElevation: 756.56; decimalLatitude: 39.35663; decimalLongitude: -4.35912; geodeticDatum: WGS84; **Event:** eventID: H; samplingProtocol: Pitfall
- **Type status:** Other material. **Occurrence:** individualCount: 4; sex: male; **Location:** locationID: C1; continent: Europe; country: Spain; countryCode: ES; stateProvince: Castilla-La Mancha; county: Ciudad Real; locality: Valle Brezoso; verbatimElevation: 756.56; decimalLatitude: 39.35663; decimalLongitude: -4.35912; geodeticDatum: WGS84; **Event:** eventID: I; samplingProtocol: Pitfall
- **Type status:** Other material. **Occurrence:** individualCount: 2; sex: male; **Location:** locationID: C1; continent: Europe; country: Spain; countryCode: ES; stateProvince: Castilla-La Mancha; county: Ciudad Real; locality: Valle Brezoso; verbatimElevation: 756.56; decimalLatitude: 39.35663; decimalLongitude: -4.35912; geodeticDatum: WGS84; **Event:** eventID: J; samplingProtocol: Pitfall
- **Type status:** Other material. **Occurrence:** individualCount: 5; sex: male; **Location:** locationID: C1; continent: Europe; country: Spain; countryCode: ES; stateProvince: Castilla-La Mancha; county: Ciudad Real; locality: Valle Brezoso; verbatimElevation: 756.56; decimalLatitude: 39.35663; decimalLongitude: -4.35912; geodeticDatum: WGS84; **Event:** eventID: K; samplingProtocol: Pitfall
- **Type status:** Other material. **Occurrence:** individualCount: 2; sex: male; **Location:** locationID: C1; continent: Europe; country: Spain; countryCode: ES; stateProvince: Castilla-La Mancha; county: Ciudad Real; locality: Valle Brezoso; verbatimElevation: 756.56; decimalLatitude: 39.35663; decimalLongitude: -4.35912; geodeticDatum: WGS84; **Event:** eventID: L; samplingProtocol: Pitfall
- **Type status:** Other material. **Occurrence:** individualCount: 5; sex: male; **Location:** locationID: C2; continent: Europe; country: Spain; countryCode: ES; stateProvince: Castilla-La Mancha; county: Ciudad Real; locality: Valle Brezoso; verbatimElevation: 739.31; decimalLatitude: 39.35159; decimalLongitude: -4.3589; geodeticDatum: WGS84; **Event:** eventID: A; samplingProtocol: Pitfall
- **Type status:** Other material. **Occurrence:** individualCount: 3; sex: male; **Location:** locationID: C2; continent: Europe; country: Spain; countryCode: ES; stateProvince: Castilla-La Mancha; county: Ciudad Real; locality: Valle Brezoso; verbatimElevation: 739.31; decimalLatitude: 39.35159; decimalLongitude: -4.3589; geodeticDatum: WGS84; **Event:** eventID: B; samplingProtocol: Pitfall
- **Type status:** Other material. **Occurrence:** individualCount: 1; sex: male; **Location:** locationID: C2; continent: Europe; country: Spain; countryCode: ES; stateProvince: Castilla-La Mancha; county: Ciudad Real; locality: Valle Brezoso; verbatimElevation: 739.31; decimalLatitude: 39.35159; decimalLongitude: -4.3589; geodeticDatum: WGS84; **Event:** eventID: C; samplingProtocol: Pitfall
- **Type status:** Other material. **Occurrence:** individualCount: 2; sex: male; **Location:** locationID: C2; continent: Europe; country: Spain; countryCode: ES; stateProvince: Castilla-La Mancha; county: Ciudad Real; locality: Valle Brezoso; verbatimElevation: 739.31; decimalLatitude: 39.35159; decimalLongitude: -4.3589; geodeticDatum: WGS84; **Event:** eventID: F; samplingProtocol: Pitfall
- **Type status:** Other material. **Occurrence:** individualCount: 2; sex: male; **Location:** locationID: C2; continent: Europe; country: Spain; countryCode: ES; stateProvince: Castilla-La Mancha; county: Ciudad Real; locality: Valle Brezoso; verbatimElevation: 739.31; decimalLatitude: 39.35159; decimalLongitude: -4.3589; geodeticDatum: WGS84; **Event:** eventID: G; samplingProtocol: Pitfall
- **Type status:** Other material. **Occurrence:** individualCount: 1; sex: male; **Location:** locationID: C2; continent: Europe; country: Spain; countryCode: ES; stateProvince: Castilla-La Mancha; county: Ciudad Real; locality: Valle Brezoso; verbatimElevation: 739.31; decimalLatitude: 39.35159; decimalLongitude: -4.3589; geodeticDatum: WGS84; **Event:** eventID: H; samplingProtocol: Pitfall
- **Type status:** Other material. **Occurrence:** individualCount: 1; sex: male; **Location:** locationID: C2; continent: Europe; country: Spain; countryCode: ES; stateProvince: Castilla-La Mancha; county: Ciudad Real; locality: Valle Brezoso; verbatimElevation: 739.31; decimalLatitude: 39.35159; decimalLongitude: -4.3589; geodeticDatum: WGS84; **Event:** eventID: I; samplingProtocol: Pitfall
- **Type status:** Other material. **Occurrence:** individualCount: 3; sex: male; **Location:** locationID: C2; continent: Europe; country: Spain; countryCode: ES; stateProvince: Castilla-La Mancha; county: Ciudad Real; locality: Valle Brezoso; verbatimElevation: 739.31; decimalLatitude: 39.35159; decimalLongitude: -4.3589; geodeticDatum: WGS84; **Event:** eventID: J; samplingProtocol: Pitfall
- **Type status:** Other material. **Occurrence:** individualCount: 1; sex: male; **Location:** locationID: C2; continent: Europe; country: Spain; countryCode: ES; stateProvince: Castilla-La Mancha; county: Ciudad Real; locality: Valle Brezoso; verbatimElevation: 739.31; decimalLatitude: 39.35159; decimalLongitude: -4.3589; geodeticDatum: WGS84; **Event:** eventID: K; samplingProtocol: Pitfall
- **Type status:** Other material. **Occurrence:** individualCount: 4; sex: male; **Location:** locationID: C2; continent: Europe; country: Spain; countryCode: ES; stateProvince: Castilla-La Mancha; county: Ciudad Real; locality: Valle Brezoso; verbatimElevation: 739.31; decimalLatitude: 39.35159; decimalLongitude: -4.3589; geodeticDatum: WGS84; **Event:** eventID: L; samplingProtocol: Pitfall
- **Type status:** Other material. **Occurrence:** individualCount: 1; sex: male; **Location:** locationID: C3; continent: Europe; country: Spain; countryCode: ES; stateProvince: Castilla-La Mancha; county: Ciudad Real; locality: La Quesera; verbatimElevation: 767.55; decimalLatitude: 39.36177; decimalLongitude: -4.41733; geodeticDatum: WGS84; **Event:** eventID: E; samplingProtocol: Pitfall
- **Type status:** Other material. **Occurrence:** individualCount: 1; sex: female; **Location:** locationID: C3; continent: Europe; country: Spain; countryCode: ES; stateProvince: Castilla-La Mancha; county: Ciudad Real; locality: La Quesera; verbatimElevation: 767.55; decimalLatitude: 39.36177; decimalLongitude: -4.41733; geodeticDatum: WGS84; **Event:** eventID: G; samplingProtocol: Pitfall
- **Type status:** Other material. **Occurrence:** individualCount: 1; sex: female; **Location:** locationID: C4; continent: Europe; country: Spain; countryCode: ES; stateProvince: Castilla-La Mancha; county: Ciudad Real; locality: La Quesera; verbatimElevation: 772.3; decimalLatitude: 39.36337; decimalLongitude: -4.41704; geodeticDatum: WGS84; **Event:** eventID: A; samplingProtocol: Pitfall
- **Type status:** Other material. **Occurrence:** individualCount: 2; sex: male; **Location:** locationID: C4; continent: Europe; country: Spain; countryCode: ES; stateProvince: Castilla-La Mancha; county: Ciudad Real; locality: La Quesera; verbatimElevation: 772.3; decimalLatitude: 39.36337; decimalLongitude: -4.41704; geodeticDatum: WGS84; **Event:** eventID: C; samplingProtocol: Pitfall
- **Type status:** Other material. **Occurrence:** individualCount: 1; sex: male; **Location:** locationID: C4; continent: Europe; country: Spain; countryCode: ES; stateProvince: Castilla-La Mancha; county: Ciudad Real; locality: La Quesera; verbatimElevation: 772.3; decimalLatitude: 39.36337; decimalLongitude: -4.41704; geodeticDatum: WGS84; **Event:** eventID: G; samplingProtocol: Pitfall
- **Type status:** Other material. **Occurrence:** individualCount: 1; sex: male; **Location:** locationID: C4; continent: Europe; country: Spain; countryCode: ES; stateProvince: Castilla-La Mancha; county: Ciudad Real; locality: La Quesera; verbatimElevation: 772.3; decimalLatitude: 39.36337; decimalLongitude: -4.41704; geodeticDatum: WGS84; **Event:** eventID: J; samplingProtocol: Pitfall
- **Type status:** Other material. **Occurrence:** individualCount: 1; sex: female; **Location:** locationID: C4; continent: Europe; country: Spain; countryCode: ES; stateProvince: Castilla-La Mancha; county: Ciudad Real; locality: La Quesera; verbatimElevation: 772.3; decimalLatitude: 39.36337; decimalLongitude: -4.41704; geodeticDatum: WGS84; **Event:** eventID: K; samplingProtocol: Pitfall
- **Type status:** Other material. **Occurrence:** individualCount: 1; sex: male; **Location:** locationID: C4; continent: Europe; country: Spain; countryCode: ES; stateProvince: Castilla-La Mancha; county: Ciudad Real; locality: La Quesera; verbatimElevation: 772.3; decimalLatitude: 39.36337; decimalLongitude: -4.41704; geodeticDatum: WGS84; **Event:** eventID: K; samplingProtocol: Pitfall
- **Type status:** Other material. **Occurrence:** individualCount: 1; sex: female; **Location:** locationID: O2; continent: Europe; country: Spain; countryCode: ES; stateProvince: Aragón; county: Huesca; locality: Rebilla; verbatimElevation: 1158.13; decimalLatitude: 42.59427; decimalLongitude: 0.1529; geodeticDatum: WGS84; **Event:** eventID: J; samplingProtocol: Pitfall
- **Type status:** Other material. **Occurrence:** individualCount: 1; sex: male; **Location:** locationID: O2; continent: Europe; country: Spain; countryCode: ES; stateProvince: Aragón; county: Huesca; locality: Rebilla; verbatimElevation: 1158.13; decimalLatitude: 42.59427; decimalLongitude: 0.1529; geodeticDatum: WGS84; **Event:** eventID: L; samplingProtocol: Pitfall
- **Type status:** Other material. **Occurrence:** individualCount: 1; sex: male; **Location:** locationID: S1; continent: Europe; country: Spain; countryCode: ES; stateProvince: Andalucía; county: Granada; locality: Soportujar; verbatimElevation: 1786.57; decimalLatitude: 36.96151; decimalLongitude: -3.41881; geodeticDatum: WGS84; **Event:** eventID: F; samplingProtocol: Pitfall
- **Type status:** Other material. **Occurrence:** individualCount: 2; sex: female; **Location:** locationID: S1; continent: Europe; country: Spain; countryCode: ES; stateProvince: Andalucía; county: Granada; locality: Soportujar; verbatimElevation: 1786.57; decimalLatitude: 36.96151; decimalLongitude: -3.41881; geodeticDatum: WGS84; **Event:** eventID: F; samplingProtocol: Pitfall
- **Type status:** Other material. **Occurrence:** individualCount: 1; sex: male; **Location:** locationID: S1; continent: Europe; country: Spain; countryCode: ES; stateProvince: Andalucía; county: Granada; locality: Soportujar; verbatimElevation: 1786.57; decimalLatitude: 36.96151; decimalLongitude: -3.41881; geodeticDatum: WGS84; **Event:** eventID: L; samplingProtocol: Pitfall
- **Type status:** Other material. **Occurrence:** individualCount: 4; sex: male; **Location:** locationID: S2; continent: Europe; country: Spain; countryCode: ES; stateProvince: Andalucía; county: Granada; locality: Camarate; verbatimElevation: 1713.96; decimalLatitude: 37.18377; decimalLongitude: -3.26282; geodeticDatum: WGS84; **Event:** eventID: A; samplingProtocol: Pitfall
- **Type status:** Other material. **Occurrence:** individualCount: 1; sex: female; **Location:** locationID: S2; continent: Europe; country: Spain; countryCode: ES; stateProvince: Andalucía; county: Granada; locality: Camarate; verbatimElevation: 1713.96; decimalLatitude: 37.18377; decimalLongitude: -3.26282; geodeticDatum: WGS84; **Event:** eventID: B; samplingProtocol: Pitfall
- **Type status:** Other material. **Occurrence:** individualCount: 2; sex: male; **Location:** locationID: S2; continent: Europe; country: Spain; countryCode: ES; stateProvince: Andalucía; county: Granada; locality: Camarate; verbatimElevation: 1713.96; decimalLatitude: 37.18377; decimalLongitude: -3.26282; geodeticDatum: WGS84; **Event:** eventID: C; samplingProtocol: Pitfall
- **Type status:** Other material. **Occurrence:** individualCount: 1; sex: female; **Location:** locationID: S2; continent: Europe; country: Spain; countryCode: ES; stateProvince: Andalucía; county: Granada; locality: Camarate; verbatimElevation: 1713.96; decimalLatitude: 37.18377; decimalLongitude: -3.26282; geodeticDatum: WGS84; **Event:** eventID: C; samplingProtocol: Pitfall
- **Type status:** Other material. **Occurrence:** individualCount: 2; sex: male; **Location:** locationID: S2; continent: Europe; country: Spain; countryCode: ES; stateProvince: Andalucía; county: Granada; locality: Camarate; verbatimElevation: 1713.96; decimalLatitude: 37.18377; decimalLongitude: -3.26282; geodeticDatum: WGS84; **Event:** eventID: D; samplingProtocol: Pitfall
- **Type status:** Other material. **Occurrence:** individualCount: 3; sex: male; **Location:** locationID: S2; continent: Europe; country: Spain; countryCode: ES; stateProvince: Andalucía; county: Granada; locality: Camarate; verbatimElevation: 1713.96; decimalLatitude: 37.18377; decimalLongitude: -3.26282; geodeticDatum: WGS84; **Event:** eventID: E; samplingProtocol: Pitfall
- **Type status:** Other material. **Occurrence:** individualCount: 1; sex: female; **Location:** locationID: S2; continent: Europe; country: Spain; countryCode: ES; stateProvince: Andalucía; county: Granada; locality: Camarate; verbatimElevation: 1713.96; decimalLatitude: 37.18377; decimalLongitude: -3.26282; geodeticDatum: WGS84; **Event:** eventID: E; samplingProtocol: Pitfall
- **Type status:** Other material. **Occurrence:** individualCount: 3; sex: male; **Location:** locationID: S2; continent: Europe; country: Spain; countryCode: ES; stateProvince: Andalucía; county: Granada; locality: Camarate; verbatimElevation: 1713.96; decimalLatitude: 37.18377; decimalLongitude: -3.26282; geodeticDatum: WGS84; **Event:** eventID: I; samplingProtocol: Pitfall
- **Type status:** Other material. **Occurrence:** individualCount: 1; sex: female; **Location:** locationID: S2; continent: Europe; country: Spain; countryCode: ES; stateProvince: Andalucía; county: Granada; locality: Camarate; verbatimElevation: 1713.96; decimalLatitude: 37.18377; decimalLongitude: -3.26282; geodeticDatum: WGS84; **Event:** eventID: I; samplingProtocol: Pitfall
- **Type status:** Other material. **Occurrence:** individualCount: 1; sex: male; **Location:** locationID: S2; continent: Europe; country: Spain; countryCode: ES; stateProvince: Andalucía; county: Granada; locality: Camarate; verbatimElevation: 1713.96; decimalLatitude: 37.18377; decimalLongitude: -3.26282; geodeticDatum: WGS84; **Event:** eventID: J; samplingProtocol: Pitfall
- **Type status:** Other material. **Occurrence:** individualCount: 1; sex: male; **Location:** locationID: S2; continent: Europe; country: Spain; countryCode: ES; stateProvince: Andalucía; county: Granada; locality: Camarate; verbatimElevation: 1713.96; decimalLatitude: 37.18377; decimalLongitude: -3.26282; geodeticDatum: WGS84; **Event:** eventID: K; samplingProtocol: Pitfall

##### Distribution

Europe

#### Pimoidae

Wunderlich, 1986

#### Pimoa breuili

(Fage, 1931)

##### Materials

- **Type status:** Other material. **Occurrence:** individualCount: 1; sex: female; **Location:** locationID: P4; continent: Europe; country: Spain; countryCode: ES; stateProvince: Castilla y León; county: León; locality: El Canto; verbatimElevation: 943.48; decimalLatitude: 43.17227; decimalLongitude: -4.90857; geodeticDatum: WGS84; **Event:** eventID: 1; samplingProtocol: Aerial; eventTime: Night

##### Distribution

Spain

#### Pisauridae

Simon, 1890

#### Pisaura mirabilis

(Clerck, 1757)

##### Materials

- **Type status:** Other material. **Occurrence:** individualCount: 1; sex: female; **Location:** locationID: C4; continent: Europe; country: Spain; countryCode: ES; stateProvince: Castilla-La Mancha; county: Ciudad Real; locality: La Quesera; verbatimElevation: 772.3; decimalLatitude: 39.36337; decimalLongitude: -4.41704; geodeticDatum: WGS84; **Event:** eventID: 1; samplingProtocol: Sweeping; eventTime: Night
- **Type status:** Other material. **Occurrence:** individualCount: 1; sex: female; **Location:** locationID: O1; continent: Europe; country: Spain; countryCode: ES; stateProvince: Aragón; county: Huesca; locality: O Furno; verbatimElevation: 1396.73; decimalLatitude: 42.60677; decimalLongitude: 0.13135; geodeticDatum: WGS84; **Event:** eventID: 1; samplingProtocol: Sweeping; eventTime: Night
- **Type status:** Other material. **Occurrence:** individualCount: 1; sex: female; **Location:** locationID: O2; continent: Europe; country: Spain; countryCode: ES; stateProvince: Aragón; county: Huesca; locality: Rebilla; verbatimElevation: 1158.13; decimalLatitude: 42.59427; decimalLongitude: 0.1529; geodeticDatum: WGS84; **Event:** eventID: 1; samplingProtocol: Aerial; eventTime: Night
- **Type status:** Other material. **Occurrence:** individualCount: 1; sex: female; **Location:** locationID: P1; continent: Europe; country: Spain; countryCode: ES; stateProvince: Castilla y León; county: León; locality: Monte Robledo; verbatimElevation: 1071.58; decimalLatitude: 43.1445; decimalLongitude: -4.92675; geodeticDatum: WGS84; **Event:** eventID: E; samplingProtocol: Pitfall

##### Distribution

Palearctic

#### Salticidae

Blackwall, 1841

#### Aelurillus luctuosus

(Lucas, 1846)

##### Materials

- **Type status:** Other material. **Occurrence:** individualCount: 1; sex: male; **Location:** locationID: C3; continent: Europe; country: Spain; countryCode: ES; stateProvince: Castilla-La Mancha; county: Ciudad Real; locality: La Quesera; verbatimElevation: 767.55; decimalLatitude: 39.36177; decimalLongitude: -4.41733; geodeticDatum: WGS84; **Event:** eventID: G; samplingProtocol: Pitfall
- **Type status:** Other material. **Occurrence:** individualCount: 1; sex: male; **Location:** locationID: S1; continent: Europe; country: Spain; countryCode: ES; stateProvince: Andalucía; county: Granada; locality: Soportujar; verbatimElevation: 1786.57; decimalLatitude: 36.96151; decimalLongitude: -3.41881; geodeticDatum: WGS84; **Event:** eventID: H; samplingProtocol: Pitfall
- **Type status:** Other material. **Occurrence:** individualCount: 1; sex: male; **Location:** locationID: S1; continent: Europe; country: Spain; countryCode: ES; stateProvince: Andalucía; county: Granada; locality: Soportujar; verbatimElevation: 1786.57; decimalLatitude: 36.96151; decimalLongitude: -3.41881; geodeticDatum: WGS84; **Event:** eventID: J; samplingProtocol: Pitfall
- **Type status:** Other material. **Occurrence:** individualCount: 1; sex: male; **Location:** locationID: S2; continent: Europe; country: Spain; countryCode: ES; stateProvince: Andalucía; county: Granada; locality: Camarate; verbatimElevation: 1713.96; decimalLatitude: 37.18377; decimalLongitude: -3.26282; geodeticDatum: WGS84; **Event:** eventID: E; samplingProtocol: Pitfall
- **Type status:** Other material. **Occurrence:** individualCount: 1; sex: male; **Location:** locationID: S2; continent: Europe; country: Spain; countryCode: ES; stateProvince: Andalucía; county: Granada; locality: Camarate; verbatimElevation: 1713.96; decimalLatitude: 37.18377; decimalLongitude: -3.26282; geodeticDatum: WGS84; **Event:** eventID: F; samplingProtocol: Pitfall

##### Distribution

Mediterranean to Turkmenistan

#### Ballus chalybeius

(Walckenaer, 1802)

##### Materials

- **Type status:** Other material. **Occurrence:** individualCount: 1; sex: female; **Location:** locationID: A1; continent: Europe; country: Spain; countryCode: ES; stateProvince: Catalonia; county: Lleida; locality: Sola de Boi; verbatimElevation: 1759.8; decimalLatitude: 42.54958; decimalLongitude: 0.87254; geodeticDatum: WGS84; **Event:** eventID: 1; samplingProtocol: Beating; eventTime: Day
- **Type status:** Other material. **Occurrence:** individualCount: 2; sex: female; **Location:** locationID: A2; continent: Europe; country: Spain; countryCode: ES; stateProvince: Catalonia; county: Lleida; locality: Sola de Boi; verbatimElevation: 1738.7; decimalLatitude: 42.54913; decimalLongitude: 0.87137; geodeticDatum: WGS84; **Event:** eventID: 1; samplingProtocol: Beating; eventTime: Day
- **Type status:** Other material. **Occurrence:** individualCount: 1; sex: female; **Location:** locationID: A2; continent: Europe; country: Spain; countryCode: ES; stateProvince: Catalonia; county: Lleida; locality: Sola de Boi; verbatimElevation: 1738.7; decimalLatitude: 42.54913; decimalLongitude: 0.87137; geodeticDatum: WGS84; **Event:** eventID: 1; samplingProtocol: Beating; eventTime: Night
- **Type status:** Other material. **Occurrence:** individualCount: 1; sex: male; **Location:** locationID: A2; continent: Europe; country: Spain; countryCode: ES; stateProvince: Catalonia; county: Lleida; locality: Sola de Boi; verbatimElevation: 1738.7; decimalLatitude: 42.54913; decimalLongitude: 0.87137; geodeticDatum: WGS84; **Event:** eventID: L; samplingProtocol: Pitfall
- **Type status:** Other material. **Occurrence:** individualCount: 1; sex: female; **Location:** locationID: A2; continent: Europe; country: Spain; countryCode: ES; stateProvince: Catalonia; county: Lleida; locality: Sola de Boi; verbatimElevation: 1738.7; decimalLatitude: 42.54913; decimalLongitude: 0.87137; geodeticDatum: WGS84; **Event:** eventID: 1; samplingProtocol: Sweeping; eventTime: Day
- **Type status:** Other material. **Occurrence:** individualCount: 1; sex: female; **Location:** locationID: C1; continent: Europe; country: Spain; countryCode: ES; stateProvince: Castilla-La Mancha; county: Ciudad Real; locality: Valle Brezoso; verbatimElevation: 756.56; decimalLatitude: 39.35663; decimalLongitude: -4.35912; geodeticDatum: WGS84; **Event:** eventID: 2; samplingProtocol: Beating; eventTime: Day
- **Type status:** Other material. **Occurrence:** individualCount: 1; sex: female; **Location:** locationID: C2; continent: Europe; country: Spain; countryCode: ES; stateProvince: Castilla-La Mancha; county: Ciudad Real; locality: Valle Brezoso; verbatimElevation: 739.31; decimalLatitude: 39.35159; decimalLongitude: -4.3589; geodeticDatum: WGS84; **Event:** eventID: 2; samplingProtocol: Beating; eventTime: Night
- **Type status:** Other material. **Occurrence:** individualCount: 1; sex: male; **Location:** locationID: C3; continent: Europe; country: Spain; countryCode: ES; stateProvince: Castilla-La Mancha; county: Ciudad Real; locality: La Quesera; verbatimElevation: 767.55; decimalLatitude: 39.36177; decimalLongitude: -4.41733; geodeticDatum: WGS84; **Event:** eventID: H; samplingProtocol: Pitfall
- **Type status:** Other material. **Occurrence:** individualCount: 1; sex: female; **Location:** locationID: C4; continent: Europe; country: Spain; countryCode: ES; stateProvince: Castilla-La Mancha; county: Ciudad Real; locality: La Quesera; verbatimElevation: 772.3; decimalLatitude: 39.36337; decimalLongitude: -4.41704; geodeticDatum: WGS84; **Event:** eventID: 1; samplingProtocol: Sweeping; eventTime: Night
- **Type status:** Other material. **Occurrence:** individualCount: 1; sex: female; **Location:** locationID: M1; continent: Europe; country: Spain; countryCode: ES; stateProvince: Extremadura; county: Cáceres; locality: Peña Falcón; verbatimElevation: 320.6; decimalLatitude: 39.83296; decimalLongitude: -6.0641; geodeticDatum: WGS84; **Event:** eventID: 1; samplingProtocol: Beating; eventTime: Day
- **Type status:** Other material. **Occurrence:** individualCount: 1; sex: female; **Location:** locationID: M1; continent: Europe; country: Spain; countryCode: ES; stateProvince: Extremadura; county: Cáceres; locality: Peña Falcón; verbatimElevation: 320.6; decimalLatitude: 39.83296; decimalLongitude: -6.0641; geodeticDatum: WGS84; **Event:** eventID: 2; samplingProtocol: Beating; eventTime: Day
- **Type status:** Other material. **Occurrence:** individualCount: 1; sex: female; **Location:** locationID: M2; continent: Europe; country: Spain; countryCode: ES; stateProvince: Extremadura; county: Cáceres; locality: Fuente del Frances; verbatimElevation: 320.72; decimalLatitude: 39.828; decimalLongitude: -6.03249; geodeticDatum: WGS84; **Event:** eventID: 1; samplingProtocol: Sweeping; eventTime: Day
- **Type status:** Other material. **Occurrence:** individualCount: 1; sex: female; **Location:** locationID: O1; continent: Europe; country: Spain; countryCode: ES; stateProvince: Aragón; county: Huesca; locality: O Furno; verbatimElevation: 1396.73; decimalLatitude: 42.60677; decimalLongitude: 0.13135; geodeticDatum: WGS84; **Event:** eventID: 1; samplingProtocol: Beating; eventTime: Day
- **Type status:** Other material. **Occurrence:** individualCount: 1; sex: male; **Location:** locationID: O1; continent: Europe; country: Spain; countryCode: ES; stateProvince: Aragón; county: Huesca; locality: O Furno; verbatimElevation: 1396.73; decimalLatitude: 42.60677; decimalLongitude: 0.13135; geodeticDatum: WGS84; **Event:** eventID: 2; samplingProtocol: Beating; eventTime: Day
- **Type status:** Other material. **Occurrence:** individualCount: 1; sex: male; **Location:** locationID: O1; continent: Europe; country: Spain; countryCode: ES; stateProvince: Aragón; county: Huesca; locality: O Furno; verbatimElevation: 1396.73; decimalLatitude: 42.60677; decimalLongitude: 0.13135; geodeticDatum: WGS84; **Event:** eventID: 1; samplingProtocol: Beating; eventTime: Night
- **Type status:** Other material. **Occurrence:** individualCount: 1; sex: female; **Location:** locationID: O2; continent: Europe; country: Spain; countryCode: ES; stateProvince: Aragón; county: Huesca; locality: Rebilla; verbatimElevation: 1158.13; decimalLatitude: 42.59427; decimalLongitude: 0.1529; geodeticDatum: WGS84; **Event:** eventID: 1; samplingProtocol: Beating; eventTime: Day
- **Type status:** Other material. **Occurrence:** individualCount: 3; sex: female; **Location:** locationID: P2; continent: Europe; country: Spain; countryCode: ES; stateProvince: Castilla y León; county: León; locality: Joyoguelas; verbatimElevation: 763.98; decimalLatitude: 43.17771; decimalLongitude: -4.90579; geodeticDatum: WGS84; **Event:** eventID: 1; samplingProtocol: Beating; eventTime: Day
- **Type status:** Other material. **Occurrence:** individualCount: 2; sex: female; **Location:** locationID: P2; continent: Europe; country: Spain; countryCode: ES; stateProvince: Castilla y León; county: León; locality: Joyoguelas; verbatimElevation: 763.98; decimalLatitude: 43.17771; decimalLongitude: -4.90579; geodeticDatum: WGS84; **Event:** eventID: 2; samplingProtocol: Beating; eventTime: Day
- **Type status:** Other material. **Occurrence:** individualCount: 1; sex: female; **Location:** locationID: P2; continent: Europe; country: Spain; countryCode: ES; stateProvince: Castilla y León; county: León; locality: Joyoguelas; verbatimElevation: 763.98; decimalLatitude: 43.17771; decimalLongitude: -4.90579; geodeticDatum: WGS84; **Event:** eventID: 1; samplingProtocol: Beating; eventTime: Night
- **Type status:** Other material. **Occurrence:** individualCount: 1; sex: female; **Location:** locationID: P2; continent: Europe; country: Spain; countryCode: ES; stateProvince: Castilla y León; county: León; locality: Joyoguelas; verbatimElevation: 763.98; decimalLatitude: 43.17771; decimalLongitude: -4.90579; geodeticDatum: WGS84; **Event:** eventID: 1; samplingProtocol: Sweeping; eventTime: Night
- **Type status:** Other material. **Occurrence:** individualCount: 1; sex: male; **Location:** locationID: P3; continent: Europe; country: Spain; countryCode: ES; stateProvince: Castilla y León; county: León; locality: Las Arroyas; verbatimElevation: 1097.1; decimalLatitude: 43.14351; decimalLongitude: -4.94878; geodeticDatum: WGS84; **Event:** eventID: 1; samplingProtocol: Beating; eventTime: Day
- **Type status:** Other material. **Occurrence:** individualCount: 1; sex: female; **Location:** locationID: P3; continent: Europe; country: Spain; countryCode: ES; stateProvince: Castilla y León; county: León; locality: Las Arroyas; verbatimElevation: 1097.1; decimalLatitude: 43.14351; decimalLongitude: -4.94878; geodeticDatum: WGS84; **Event:** eventID: H; samplingProtocol: Pitfall
- **Type status:** Other material. **Occurrence:** individualCount: 1; sex: female; **Location:** locationID: P3; continent: Europe; country: Spain; countryCode: ES; stateProvince: Castilla y León; county: León; locality: Las Arroyas; verbatimElevation: 1097.1; decimalLatitude: 43.14351; decimalLongitude: -4.94878; geodeticDatum: WGS84; **Event:** eventID: 1; samplingProtocol: Sweeping; eventTime: Night
- **Type status:** Other material. **Occurrence:** individualCount: 1; sex: female; **Location:** locationID: P4; continent: Europe; country: Spain; countryCode: ES; stateProvince: Castilla y León; county: León; locality: El Canto; verbatimElevation: 943.48; decimalLatitude: 43.17227; decimalLongitude: -4.90857; geodeticDatum: WGS84; **Event:** eventID: 1; samplingProtocol: Beating; eventTime: Day
- **Type status:** Other material. **Occurrence:** individualCount: 2; sex: female; **Location:** locationID: P4; continent: Europe; country: Spain; countryCode: ES; stateProvince: Castilla y León; county: León; locality: El Canto; verbatimElevation: 943.48; decimalLatitude: 43.17227; decimalLongitude: -4.90857; geodeticDatum: WGS84; **Event:** eventID: 1; samplingProtocol: Beating; eventTime: Night
- **Type status:** Other material. **Occurrence:** individualCount: 1; sex: female; **Location:** locationID: S2; continent: Europe; country: Spain; countryCode: ES; stateProvince: Andalucía; county: Granada; locality: Camarate; verbatimElevation: 1713.96; decimalLatitude: 37.18377; decimalLongitude: -3.26282; geodeticDatum: WGS84; **Event:** eventID: 2; samplingProtocol: Beating; eventTime: Night
- **Type status:** Other material. **Occurrence:** individualCount: 1; sex: female; **Location:** locationID: S2; continent: Europe; country: Spain; countryCode: ES; stateProvince: Andalucía; county: Granada; locality: Camarate; verbatimElevation: 1713.96; decimalLatitude: 37.18377; decimalLongitude: -3.26282; geodeticDatum: WGS84; **Event:** eventID: 2; samplingProtocol: Beating; eventTime: Day

##### Distribution

Europe, North Africa to Central Asia

#### Chalcoscirtus alpicola

(L. Koch, 1876)

##### Materials

- **Type status:** Other material. **Occurrence:** individualCount: 1; sex: female; **Location:** locationID: M1; continent: Europe; country: Spain; countryCode: ES; stateProvince: Extremadura; county: Cáceres; locality: Peña Falcón; verbatimElevation: 320.6; decimalLatitude: 39.83296; decimalLongitude: -6.0641; geodeticDatum: WGS84; **Event:** eventID: F; samplingProtocol: Pitfall
- **Type status:** Other material. **Occurrence:** individualCount: 1; sex: female; **Location:** locationID: M1; continent: Europe; country: Spain; countryCode: ES; stateProvince: Extremadura; county: Cáceres; locality: Peña Falcón; verbatimElevation: 320.6; decimalLatitude: 39.83296; decimalLongitude: -6.0641; geodeticDatum: WGS84; **Event:** eventID: K; samplingProtocol: Pitfall

##### Distribution

Holarctic

#### Chalcoscirtus infimus

(Simon, 1868)

##### Materials

- **Type status:** Other material. **Occurrence:** individualCount: 1; sex: male; **Location:** locationID: C2; continent: Europe; country: Spain; countryCode: ES; stateProvince: Castilla-La Mancha; county: Ciudad Real; locality: Valle Brezoso; verbatimElevation: 739.31; decimalLatitude: 39.35159; decimalLongitude: -4.3589; geodeticDatum: WGS84; **Event:** eventID: L; samplingProtocol: Pitfall
- **Type status:** Other material. **Occurrence:** individualCount: 1; sex: female; **Location:** locationID: C2; continent: Europe; country: Spain; countryCode: ES; stateProvince: Castilla-La Mancha; county: Ciudad Real; locality: Valle Brezoso; verbatimElevation: 739.31; decimalLatitude: 39.35159; decimalLongitude: -4.3589; geodeticDatum: WGS84; **Event:** eventID: 1; samplingProtocol: Sweeping; eventTime: Day
- **Type status:** Other material. **Occurrence:** individualCount: 1; sex: male; **Location:** locationID: C3; continent: Europe; country: Spain; countryCode: ES; stateProvince: Castilla-La Mancha; county: Ciudad Real; locality: La Quesera; verbatimElevation: 767.55; decimalLatitude: 39.36177; decimalLongitude: -4.41733; geodeticDatum: WGS84; **Event:** eventID: C; samplingProtocol: Pitfall
- **Type status:** Other material. **Occurrence:** individualCount: 1; sex: female; **Location:** locationID: C3; continent: Europe; country: Spain; countryCode: ES; stateProvince: Castilla-La Mancha; county: Ciudad Real; locality: La Quesera; verbatimElevation: 767.55; decimalLatitude: 39.36177; decimalLongitude: -4.41733; geodeticDatum: WGS84; **Event:** eventID: C; samplingProtocol: Pitfall
- **Type status:** Other material. **Occurrence:** individualCount: 1; sex: male; **Location:** locationID: C3; continent: Europe; country: Spain; countryCode: ES; stateProvince: Castilla-La Mancha; county: Ciudad Real; locality: La Quesera; verbatimElevation: 767.55; decimalLatitude: 39.36177; decimalLongitude: -4.41733; geodeticDatum: WGS84; **Event:** eventID: F; samplingProtocol: Pitfall
- **Type status:** Other material. **Occurrence:** individualCount: 1; sex: female; **Location:** locationID: C3; continent: Europe; country: Spain; countryCode: ES; stateProvince: Castilla-La Mancha; county: Ciudad Real; locality: La Quesera; verbatimElevation: 767.55; decimalLatitude: 39.36177; decimalLongitude: -4.41733; geodeticDatum: WGS84; **Event:** eventID: G; samplingProtocol: Pitfall
- **Type status:** Other material. **Occurrence:** individualCount: 1; sex: female; **Location:** locationID: S1; continent: Europe; country: Spain; countryCode: ES; stateProvince: Andalucía; county: Granada; locality: Soportujar; verbatimElevation: 1786.57; decimalLatitude: 36.96151; decimalLongitude: -3.41881; geodeticDatum: WGS84; **Event:** eventID: F; samplingProtocol: Pitfall
- **Type status:** Other material. **Occurrence:** individualCount: 3; sex: male; **Location:** locationID: S2; continent: Europe; country: Spain; countryCode: ES; stateProvince: Andalucía; county: Granada; locality: Camarate; verbatimElevation: 1713.96; decimalLatitude: 37.18377; decimalLongitude: -3.26282; geodeticDatum: WGS84; **Event:** eventID: C; samplingProtocol: Pitfall
- **Type status:** Other material. **Occurrence:** individualCount: 1; sex: male; **Location:** locationID: S2; continent: Europe; country: Spain; countryCode: ES; stateProvince: Andalucía; county: Granada; locality: Camarate; verbatimElevation: 1713.96; decimalLatitude: 37.18377; decimalLongitude: -3.26282; geodeticDatum: WGS84; **Event:** eventID: L; samplingProtocol: Pitfall
- **Type status:** Other material. **Occurrence:** individualCount: 1; sex: male; **Location:** locationID: S2; continent: Europe; country: Spain; countryCode: ES; stateProvince: Andalucía; county: Granada; locality: Camarate; verbatimElevation: 1713.96; decimalLatitude: 37.18377; decimalLongitude: -3.26282; geodeticDatum: WGS84; **Event:** eventID: L; samplingProtocol: Pitfall

##### Distribution

Southern, Central Europe to Central Asia

#### Euophrys frontalis

(Walckenaer, 1802)

##### Materials

- **Type status:** Other material. **Occurrence:** individualCount: 1; sex: male; **Location:** locationID: A1; continent: Europe; country: Spain; countryCode: ES; stateProvince: Catalonia; county: Lleida; locality: Sola de Boi; verbatimElevation: 1759.8; decimalLatitude: 42.54958; decimalLongitude: 0.87254; geodeticDatum: WGS84; **Event:** eventID: F; samplingProtocol: Pitfall
- **Type status:** Other material. **Occurrence:** individualCount: 1; sex: male; **Location:** locationID: A1; continent: Europe; country: Spain; countryCode: ES; stateProvince: Catalonia; county: Lleida; locality: Sola de Boi; verbatimElevation: 1759.8; decimalLatitude: 42.54958; decimalLongitude: 0.87254; geodeticDatum: WGS84; **Event:** eventID: I; samplingProtocol: Pitfall
- **Type status:** Other material. **Occurrence:** individualCount: 1; sex: female; **Location:** locationID: A1; continent: Europe; country: Spain; countryCode: ES; stateProvince: Catalonia; county: Lleida; locality: Sola de Boi; verbatimElevation: 1759.8; decimalLatitude: 42.54958; decimalLongitude: 0.87254; geodeticDatum: WGS84; **Event:** eventID: I; samplingProtocol: Pitfall
- **Type status:** Other material. **Occurrence:** individualCount: 1; sex: male; **Location:** locationID: A2; continent: Europe; country: Spain; countryCode: ES; stateProvince: Catalonia; county: Lleida; locality: Sola de Boi; verbatimElevation: 1738.7; decimalLatitude: 42.54913; decimalLongitude: 0.87137; geodeticDatum: WGS84; **Event:** eventID: A; samplingProtocol: Pitfall
- **Type status:** Other material. **Occurrence:** individualCount: 1; sex: female; **Location:** locationID: A2; continent: Europe; country: Spain; countryCode: ES; stateProvince: Catalonia; county: Lleida; locality: Sola de Boi; verbatimElevation: 1738.7; decimalLatitude: 42.54913; decimalLongitude: 0.87137; geodeticDatum: WGS84; **Event:** eventID: J; samplingProtocol: Pitfall
- **Type status:** Other material. **Occurrence:** individualCount: 1; sex: female; **Location:** locationID: A2; continent: Europe; country: Spain; countryCode: ES; stateProvince: Catalonia; county: Lleida; locality: Sola de Boi; verbatimElevation: 1738.7; decimalLatitude: 42.54913; decimalLongitude: 0.87137; geodeticDatum: WGS84; **Event:** eventID: 1; samplingProtocol: Sweeping; eventTime: Night
- **Type status:** Other material. **Occurrence:** individualCount: 1; sex: female; **Location:** locationID: A2; continent: Europe; country: Spain; countryCode: ES; stateProvince: Catalonia; county: Lleida; locality: Sola de Boi; verbatimElevation: 1738.7; decimalLatitude: 42.54913; decimalLongitude: 0.87137; geodeticDatum: WGS84; **Event:** eventID: 1; samplingProtocol: Sweeping; eventTime: Night

##### Distribution

Palearctic

#### Euophrys herbigrada

(Simon, 1871)

##### Materials

- **Type status:** Other material. **Occurrence:** individualCount: 1; sex: male; **Location:** locationID: C2; continent: Europe; country: Spain; countryCode: ES; stateProvince: Castilla-La Mancha; county: Ciudad Real; locality: Valle Brezoso; verbatimElevation: 739.31; decimalLatitude: 39.35159; decimalLongitude: -4.3589; geodeticDatum: WGS84; **Event:** eventID: K; samplingProtocol: Pitfall

##### Distribution

Europe

#### Euophrys sp30

##### Materials

- **Type status:** Other material. **Occurrence:** individualCount: 1; sex: male; **Location:** locationID: S1; continent: Europe; country: Spain; countryCode: ES; stateProvince: Andalucía; county: Granada; locality: Soportujar; verbatimElevation: 1786.57; decimalLatitude: 36.96151; decimalLongitude: -3.41881; geodeticDatum: WGS84; **Event:** eventID: I; samplingProtocol: Pitfall

##### Distribution

?

##### Notes

This is a species of *Euophrys* C. L. Koch, 1838, which we could not identify.

#### Evarcha arcuata

(Clerck, 1757)

##### Materials

- **Type status:** Other material. **Occurrence:** individualCount: 2; sex: male; **Location:** locationID: O1; continent: Europe; country: Spain; countryCode: ES; stateProvince: Aragón; county: Huesca; locality: O Furno; verbatimElevation: 1396.73; decimalLatitude: 42.60677; decimalLongitude: 0.13135; geodeticDatum: WGS84; **Event:** eventID: 2; samplingProtocol: Beating; eventTime: Day
- **Type status:** Other material. **Occurrence:** individualCount: 2; sex: female; **Location:** locationID: O1; continent: Europe; country: Spain; countryCode: ES; stateProvince: Aragón; county: Huesca; locality: O Furno; verbatimElevation: 1396.73; decimalLatitude: 42.60677; decimalLongitude: 0.13135; geodeticDatum: WGS84; **Event:** eventID: 1; samplingProtocol: Sweeping; eventTime: Night

##### Distribution

Palearctic

#### Evarcha falcata

(Clerck, 1757)

##### Materials

- **Type status:** Other material. **Occurrence:** individualCount: 1; sex: female; **Location:** locationID: A1; continent: Europe; country: Spain; countryCode: ES; stateProvince: Catalonia; county: Lleida; locality: Sola de Boi; verbatimElevation: 1759.8; decimalLatitude: 42.54958; decimalLongitude: 0.87254; geodeticDatum: WGS84; **Event:** eventID: 2; samplingProtocol: Sweeping; eventTime: Day
- **Type status:** Other material. **Occurrence:** individualCount: 3; sex: male; **Location:** locationID: A1; continent: Europe; country: Spain; countryCode: ES; stateProvince: Catalonia; county: Lleida; locality: Sola de Boi; verbatimElevation: 1759.8; decimalLatitude: 42.54958; decimalLongitude: 0.87254; geodeticDatum: WGS84; **Event:** eventID: 1; samplingProtocol: Sweeping; eventTime: Night
- **Type status:** Other material. **Occurrence:** individualCount: 2; sex: female; **Location:** locationID: A1; continent: Europe; country: Spain; countryCode: ES; stateProvince: Catalonia; county: Lleida; locality: Sola de Boi; verbatimElevation: 1759.8; decimalLatitude: 42.54958; decimalLongitude: 0.87254; geodeticDatum: WGS84; **Event:** eventID: 1; samplingProtocol: Sweeping; eventTime: Night
- **Type status:** Other material. **Occurrence:** individualCount: 1; sex: female; **Location:** locationID: P2; continent: Europe; country: Spain; countryCode: ES; stateProvince: Castilla y León; county: León; locality: Joyoguelas; verbatimElevation: 763.98; decimalLatitude: 43.17771; decimalLongitude: -4.90579; geodeticDatum: WGS84; **Event:** eventID: 1; samplingProtocol: Sweeping; eventTime: Day
- **Type status:** Other material. **Occurrence:** individualCount: 1; sex: male; **Location:** locationID: P2; continent: Europe; country: Spain; countryCode: ES; stateProvince: Castilla y León; county: León; locality: Joyoguelas; verbatimElevation: 763.98; decimalLatitude: 43.17771; decimalLongitude: -4.90579; geodeticDatum: WGS84; **Event:** eventID: 1; samplingProtocol: Sweeping; eventTime: Night
- **Type status:** Other material. **Occurrence:** individualCount: 2; sex: female; **Location:** locationID: P3; continent: Europe; country: Spain; countryCode: ES; stateProvince: Castilla y León; county: León; locality: Las Arroyas; verbatimElevation: 1097.1; decimalLatitude: 43.14351; decimalLongitude: -4.94878; geodeticDatum: WGS84; **Event:** eventID: 1; samplingProtocol: Sweeping; eventTime: Night
- **Type status:** Other material. **Occurrence:** individualCount: 1; sex: male; **Location:** locationID: P4; continent: Europe; country: Spain; countryCode: ES; stateProvince: Castilla y León; county: León; locality: El Canto; verbatimElevation: 943.48; decimalLatitude: 43.17227; decimalLongitude: -4.90857; geodeticDatum: WGS84; **Event:** eventID: 1; samplingProtocol: Beating; eventTime: Night
- **Type status:** Other material. **Occurrence:** individualCount: 2; sex: female; **Location:** locationID: P4; continent: Europe; country: Spain; countryCode: ES; stateProvince: Castilla y León; county: León; locality: El Canto; verbatimElevation: 943.48; decimalLatitude: 43.17227; decimalLongitude: -4.90857; geodeticDatum: WGS84; **Event:** eventID: 1; samplingProtocol: Sweeping; eventTime: Night
- **Type status:** Other material. **Occurrence:** individualCount: 1; sex: male; **Location:** locationID: P4; continent: Europe; country: Spain; countryCode: ES; stateProvince: Castilla y León; county: León; locality: El Canto; verbatimElevation: 943.48; decimalLatitude: 43.17227; decimalLongitude: -4.90857; geodeticDatum: WGS84; **Event:** eventID: 2; samplingProtocol: Sweeping; eventTime: Day

##### Distribution

Palearctic

#### Evarcha jucunda

(Lucas, 1846)

##### Materials

- **Type status:** Other material. **Occurrence:** individualCount: 1; sex: male; **Location:** locationID: C4; continent: Europe; country: Spain; countryCode: ES; stateProvince: Castilla-La Mancha; county: Ciudad Real; locality: La Quesera; verbatimElevation: 772.3; decimalLatitude: 39.36337; decimalLongitude: -4.41704; geodeticDatum: WGS84; **Event:** eventID: L; samplingProtocol: Pitfall

##### Distribution

Mediterranean, introduced in Belgium

#### Heliophanus cupreus

(Walckenaer, 1802)

##### Materials

- **Type status:** Other material. **Occurrence:** individualCount: 1; sex: male; **Location:** locationID: A1; continent: Europe; country: Spain; countryCode: ES; stateProvince: Catalonia; county: Lleida; locality: Sola de Boi; verbatimElevation: 1759.8; decimalLatitude: 42.54958; decimalLongitude: 0.87254; geodeticDatum: WGS84; **Event:** eventID: 1; samplingProtocol: Aerial; eventTime: Night
- **Type status:** Other material. **Occurrence:** individualCount: 4; sex: female; **Location:** locationID: A1; continent: Europe; country: Spain; countryCode: ES; stateProvince: Catalonia; county: Lleida; locality: Sola de Boi; verbatimElevation: 1759.8; decimalLatitude: 42.54958; decimalLongitude: 0.87254; geodeticDatum: WGS84; **Event:** eventID: 1; samplingProtocol: Beating; eventTime: Day
- **Type status:** Other material. **Occurrence:** individualCount: 2; sex: male; **Location:** locationID: A1; continent: Europe; country: Spain; countryCode: ES; stateProvince: Catalonia; county: Lleida; locality: Sola de Boi; verbatimElevation: 1759.8; decimalLatitude: 42.54958; decimalLongitude: 0.87254; geodeticDatum: WGS84; **Event:** eventID: 1; samplingProtocol: Beating; eventTime: Day
- **Type status:** Other material. **Occurrence:** individualCount: 1; sex: female; **Location:** locationID: A1; continent: Europe; country: Spain; countryCode: ES; stateProvince: Catalonia; county: Lleida; locality: Sola de Boi; verbatimElevation: 1759.8; decimalLatitude: 42.54958; decimalLongitude: 0.87254; geodeticDatum: WGS84; **Event:** eventID: 2; samplingProtocol: Beating; eventTime: Day
- **Type status:** Other material. **Occurrence:** individualCount: 2; sex: female; **Location:** locationID: A1; continent: Europe; country: Spain; countryCode: ES; stateProvince: Catalonia; county: Lleida; locality: Sola de Boi; verbatimElevation: 1759.8; decimalLatitude: 42.54958; decimalLongitude: 0.87254; geodeticDatum: WGS84; **Event:** eventID: 1; samplingProtocol: Beating; eventTime: Night
- **Type status:** Other material. **Occurrence:** individualCount: 1; sex: male; **Location:** locationID: A1; continent: Europe; country: Spain; countryCode: ES; stateProvince: Catalonia; county: Lleida; locality: Sola de Boi; verbatimElevation: 1759.8; decimalLatitude: 42.54958; decimalLongitude: 0.87254; geodeticDatum: WGS84; **Event:** eventID: 1; samplingProtocol: Beating; eventTime: Night
- **Type status:** Other material. **Occurrence:** individualCount: 1; sex: female; **Location:** locationID: A1; continent: Europe; country: Spain; countryCode: ES; stateProvince: Catalonia; county: Lleida; locality: Sola de Boi; verbatimElevation: 1759.8; decimalLatitude: 42.54958; decimalLongitude: 0.87254; geodeticDatum: WGS84; **Event:** eventID: 1; samplingProtocol: Beating; eventTime: Night
- **Type status:** Other material. **Occurrence:** individualCount: 2; sex: female; **Location:** locationID: A1; continent: Europe; country: Spain; countryCode: ES; stateProvince: Catalonia; county: Lleida; locality: Sola de Boi; verbatimElevation: 1759.8; decimalLatitude: 42.54958; decimalLongitude: 0.87254; geodeticDatum: WGS84; **Event:** eventID: 1; samplingProtocol: Sweeping; eventTime: Day
- **Type status:** Other material. **Occurrence:** individualCount: 1; sex: male; **Location:** locationID: A1; continent: Europe; country: Spain; countryCode: ES; stateProvince: Catalonia; county: Lleida; locality: Sola de Boi; verbatimElevation: 1759.8; decimalLatitude: 42.54958; decimalLongitude: 0.87254; geodeticDatum: WGS84; **Event:** eventID: 1; samplingProtocol: Sweeping; eventTime: Day
- **Type status:** Other material. **Occurrence:** individualCount: 1; sex: female; **Location:** locationID: A1; continent: Europe; country: Spain; countryCode: ES; stateProvince: Catalonia; county: Lleida; locality: Sola de Boi; verbatimElevation: 1759.8; decimalLatitude: 42.54958; decimalLongitude: 0.87254; geodeticDatum: WGS84; **Event:** eventID: 1; samplingProtocol: Sweeping; eventTime: Night
- **Type status:** Other material. **Occurrence:** individualCount: 3; sex: female; **Location:** locationID: A2; continent: Europe; country: Spain; countryCode: ES; stateProvince: Catalonia; county: Lleida; locality: Sola de Boi; verbatimElevation: 1738.7; decimalLatitude: 42.54913; decimalLongitude: 0.87137; geodeticDatum: WGS84; **Event:** eventID: 1; samplingProtocol: Beating; eventTime: Day
- **Type status:** Other material. **Occurrence:** individualCount: 6; sex: female; **Location:** locationID: A2; continent: Europe; country: Spain; countryCode: ES; stateProvince: Catalonia; county: Lleida; locality: Sola de Boi; verbatimElevation: 1738.7; decimalLatitude: 42.54913; decimalLongitude: 0.87137; geodeticDatum: WGS84; **Event:** eventID: 2; samplingProtocol: Beating; eventTime: Day
- **Type status:** Other material. **Occurrence:** individualCount: 2; sex: male; **Location:** locationID: A2; continent: Europe; country: Spain; countryCode: ES; stateProvince: Catalonia; county: Lleida; locality: Sola de Boi; verbatimElevation: 1738.7; decimalLatitude: 42.54913; decimalLongitude: 0.87137; geodeticDatum: WGS84; **Event:** eventID: 2; samplingProtocol: Beating; eventTime: Day
- **Type status:** Other material. **Occurrence:** individualCount: 2; sex: female; **Location:** locationID: A2; continent: Europe; country: Spain; countryCode: ES; stateProvince: Catalonia; county: Lleida; locality: Sola de Boi; verbatimElevation: 1738.7; decimalLatitude: 42.54913; decimalLongitude: 0.87137; geodeticDatum: WGS84; **Event:** eventID: 1; samplingProtocol: Beating; eventTime: Night
- **Type status:** Other material. **Occurrence:** individualCount: 2; sex: female; **Location:** locationID: A2; continent: Europe; country: Spain; countryCode: ES; stateProvince: Catalonia; county: Lleida; locality: Sola de Boi; verbatimElevation: 1738.7; decimalLatitude: 42.54913; decimalLongitude: 0.87137; geodeticDatum: WGS84; **Event:** eventID: 1; samplingProtocol: Sweeping; eventTime: Day
- **Type status:** Other material. **Occurrence:** individualCount: 1; sex: male; **Location:** locationID: A2; continent: Europe; country: Spain; countryCode: ES; stateProvince: Catalonia; county: Lleida; locality: Sola de Boi; verbatimElevation: 1738.7; decimalLatitude: 42.54913; decimalLongitude: 0.87137; geodeticDatum: WGS84; **Event:** eventID: 1; samplingProtocol: Sweeping; eventTime: Day
- **Type status:** Other material. **Occurrence:** individualCount: 1; sex: female; **Location:** locationID: C1; continent: Europe; country: Spain; countryCode: ES; stateProvince: Castilla-La Mancha; county: Ciudad Real; locality: Valle Brezoso; verbatimElevation: 756.56; decimalLatitude: 39.35663; decimalLongitude: -4.35912; geodeticDatum: WGS84; **Event:** eventID: 2; samplingProtocol: Beating; eventTime: Night
- **Type status:** Other material. **Occurrence:** individualCount: 2; sex: female; **Location:** locationID: C1; continent: Europe; country: Spain; countryCode: ES; stateProvince: Castilla-La Mancha; county: Ciudad Real; locality: Valle Brezoso; verbatimElevation: 756.56; decimalLatitude: 39.35663; decimalLongitude: -4.35912; geodeticDatum: WGS84; **Event:** eventID: 2; samplingProtocol: Sweeping; eventTime: Day
- **Type status:** Other material. **Occurrence:** individualCount: 2; sex: male; **Location:** locationID: C1; continent: Europe; country: Spain; countryCode: ES; stateProvince: Castilla-La Mancha; county: Ciudad Real; locality: Valle Brezoso; verbatimElevation: 756.56; decimalLatitude: 39.35663; decimalLongitude: -4.35912; geodeticDatum: WGS84; **Event:** eventID: 2; samplingProtocol: Sweeping; eventTime: Day
- **Type status:** Other material. **Occurrence:** individualCount: 1; sex: female; **Location:** locationID: O1; continent: Europe; country: Spain; countryCode: ES; stateProvince: Aragón; county: Huesca; locality: O Furno; verbatimElevation: 1396.73; decimalLatitude: 42.60677; decimalLongitude: 0.13135; geodeticDatum: WGS84; **Event:** eventID: 1; samplingProtocol: Beating; eventTime: Day
- **Type status:** Other material. **Occurrence:** individualCount: 1; sex: female; **Location:** locationID: O1; continent: Europe; country: Spain; countryCode: ES; stateProvince: Aragón; county: Huesca; locality: O Furno; verbatimElevation: 1396.73; decimalLatitude: 42.60677; decimalLongitude: 0.13135; geodeticDatum: WGS84; **Event:** eventID: D; samplingProtocol: Pitfall
- **Type status:** Other material. **Occurrence:** individualCount: 1; sex: female; **Location:** locationID: O1; continent: Europe; country: Spain; countryCode: ES; stateProvince: Aragón; county: Huesca; locality: O Furno; verbatimElevation: 1396.73; decimalLatitude: 42.60677; decimalLongitude: 0.13135; geodeticDatum: WGS84; **Event:** eventID: 1; samplingProtocol: Sweeping; eventTime: Day
- **Type status:** Other material. **Occurrence:** individualCount: 1; sex: female; **Location:** locationID: O1; continent: Europe; country: Spain; countryCode: ES; stateProvince: Aragón; county: Huesca; locality: O Furno; verbatimElevation: 1396.73; decimalLatitude: 42.60677; decimalLongitude: 0.13135; geodeticDatum: WGS84; **Event:** eventID: 1; samplingProtocol: Sweeping; eventTime: Night
- **Type status:** Other material. **Occurrence:** individualCount: 1; sex: male; **Location:** locationID: O2; continent: Europe; country: Spain; countryCode: ES; stateProvince: Aragón; county: Huesca; locality: Rebilla; verbatimElevation: 1158.13; decimalLatitude: 42.59427; decimalLongitude: 0.1529; geodeticDatum: WGS84; **Event:** eventID: 1; samplingProtocol: Beating; eventTime: Day
- **Type status:** Other material. **Occurrence:** individualCount: 3; sex: female; **Location:** locationID: O2; continent: Europe; country: Spain; countryCode: ES; stateProvince: Aragón; county: Huesca; locality: Rebilla; verbatimElevation: 1158.13; decimalLatitude: 42.59427; decimalLongitude: 0.1529; geodeticDatum: WGS84; **Event:** eventID: 1; samplingProtocol: Sweeping; eventTime: Night
- **Type status:** Other material. **Occurrence:** individualCount: 1; sex: female; **Location:** locationID: O2; continent: Europe; country: Spain; countryCode: ES; stateProvince: Aragón; county: Huesca; locality: Rebilla; verbatimElevation: 1158.13; decimalLatitude: 42.59427; decimalLongitude: 0.1529; geodeticDatum: WGS84; **Event:** eventID: 2; samplingProtocol: Sweeping; eventTime: Day
- **Type status:** Other material. **Occurrence:** individualCount: 1; sex: female; **Location:** locationID: P2; continent: Europe; country: Spain; countryCode: ES; stateProvince: Castilla y León; county: León; locality: Joyoguelas; verbatimElevation: 763.98; decimalLatitude: 43.17771; decimalLongitude: -4.90579; geodeticDatum: WGS84; **Event:** eventID: 1; samplingProtocol: Beating; eventTime: Day
- **Type status:** Other material. **Occurrence:** individualCount: 2; sex: female; **Location:** locationID: P2; continent: Europe; country: Spain; countryCode: ES; stateProvince: Castilla y León; county: León; locality: Joyoguelas; verbatimElevation: 763.98; decimalLatitude: 43.17771; decimalLongitude: -4.90579; geodeticDatum: WGS84; **Event:** eventID: 1; samplingProtocol: Beating; eventTime: Night
- **Type status:** Other material. **Occurrence:** individualCount: 1; sex: male; **Location:** locationID: P2; continent: Europe; country: Spain; countryCode: ES; stateProvince: Castilla y León; county: León; locality: Joyoguelas; verbatimElevation: 763.98; decimalLatitude: 43.17771; decimalLongitude: -4.90579; geodeticDatum: WGS84; **Event:** eventID: 1; samplingProtocol: Beating; eventTime: Night
- **Type status:** Other material. **Occurrence:** individualCount: 3; sex: female; **Location:** locationID: P2; continent: Europe; country: Spain; countryCode: ES; stateProvince: Castilla y León; county: León; locality: Joyoguelas; verbatimElevation: 763.98; decimalLatitude: 43.17771; decimalLongitude: -4.90579; geodeticDatum: WGS84; **Event:** eventID: 1; samplingProtocol: Sweeping; eventTime: Day
- **Type status:** Other material. **Occurrence:** individualCount: 1; sex: male; **Location:** locationID: P2; continent: Europe; country: Spain; countryCode: ES; stateProvince: Castilla y León; county: León; locality: Joyoguelas; verbatimElevation: 763.98; decimalLatitude: 43.17771; decimalLongitude: -4.90579; geodeticDatum: WGS84; **Event:** eventID: 1; samplingProtocol: Sweeping; eventTime: Day
- **Type status:** Other material. **Occurrence:** individualCount: 2; sex: female; **Location:** locationID: P2; continent: Europe; country: Spain; countryCode: ES; stateProvince: Castilla y León; county: León; locality: Joyoguelas; verbatimElevation: 763.98; decimalLatitude: 43.17771; decimalLongitude: -4.90579; geodeticDatum: WGS84; **Event:** eventID: 2; samplingProtocol: Sweeping; eventTime: Day
- **Type status:** Other material. **Occurrence:** individualCount: 2; sex: male; **Location:** locationID: P2; continent: Europe; country: Spain; countryCode: ES; stateProvince: Castilla y León; county: León; locality: Joyoguelas; verbatimElevation: 763.98; decimalLatitude: 43.17771; decimalLongitude: -4.90579; geodeticDatum: WGS84; **Event:** eventID: 2; samplingProtocol: Sweeping; eventTime: Day
- **Type status:** Other material. **Occurrence:** individualCount: 2; sex: female; **Location:** locationID: P2; continent: Europe; country: Spain; countryCode: ES; stateProvince: Castilla y León; county: León; locality: Joyoguelas; verbatimElevation: 763.98; decimalLatitude: 43.17771; decimalLongitude: -4.90579; geodeticDatum: WGS84; **Event:** eventID: 1; samplingProtocol: Sweeping; eventTime: Night
- **Type status:** Other material. **Occurrence:** individualCount: 1; sex: male; **Location:** locationID: P2; continent: Europe; country: Spain; countryCode: ES; stateProvince: Castilla y León; county: León; locality: Joyoguelas; verbatimElevation: 763.98; decimalLatitude: 43.17771; decimalLongitude: -4.90579; geodeticDatum: WGS84; **Event:** eventID: 1; samplingProtocol: Sweeping; eventTime: Night
- **Type status:** Other material. **Occurrence:** individualCount: 1; sex: male; **Location:** locationID: P3; continent: Europe; country: Spain; countryCode: ES; stateProvince: Castilla y León; county: León; locality: Las Arroyas; verbatimElevation: 1097.1; decimalLatitude: 43.14351; decimalLongitude: -4.94878; geodeticDatum: WGS84; **Event:** eventID: 1; samplingProtocol: Sweeping; eventTime: Day
- **Type status:** Other material. **Occurrence:** individualCount: 1; sex: female; **Location:** locationID: P4; continent: Europe; country: Spain; countryCode: ES; stateProvince: Castilla y León; county: León; locality: El Canto; verbatimElevation: 943.48; decimalLatitude: 43.17227; decimalLongitude: -4.90857; geodeticDatum: WGS84; **Event:** eventID: 2; samplingProtocol: Aerial; eventTime: Night
- **Type status:** Other material. **Occurrence:** individualCount: 1; sex: female; **Location:** locationID: P4; continent: Europe; country: Spain; countryCode: ES; stateProvince: Castilla y León; county: León; locality: El Canto; verbatimElevation: 943.48; decimalLatitude: 43.17227; decimalLongitude: -4.90857; geodeticDatum: WGS84; **Event:** eventID: 2; samplingProtocol: Beating; eventTime: Day
- **Type status:** Other material. **Occurrence:** individualCount: 2; sex: male; **Location:** locationID: P4; continent: Europe; country: Spain; countryCode: ES; stateProvince: Castilla y León; county: León; locality: El Canto; verbatimElevation: 943.48; decimalLatitude: 43.17227; decimalLongitude: -4.90857; geodeticDatum: WGS84; **Event:** eventID: 1; samplingProtocol: Sweeping; eventTime: Day
- **Type status:** Other material. **Occurrence:** individualCount: 5; sex: female; **Location:** locationID: P4; continent: Europe; country: Spain; countryCode: ES; stateProvince: Castilla y León; county: León; locality: El Canto; verbatimElevation: 943.48; decimalLatitude: 43.17227; decimalLongitude: -4.90857; geodeticDatum: WGS84; **Event:** eventID: 2; samplingProtocol: Sweeping; eventTime: Day
- **Type status:** Other material. **Occurrence:** individualCount: 2; sex: male; **Location:** locationID: P4; continent: Europe; country: Spain; countryCode: ES; stateProvince: Castilla y León; county: León; locality: El Canto; verbatimElevation: 943.48; decimalLatitude: 43.17227; decimalLongitude: -4.90857; geodeticDatum: WGS84; **Event:** eventID: 2; samplingProtocol: Sweeping; eventTime: Day
- **Type status:** Other material. **Occurrence:** individualCount: 1; sex: female; **Location:** locationID: S1; continent: Europe; country: Spain; countryCode: ES; stateProvince: Andalucía; county: Granada; locality: Soportujar; verbatimElevation: 1786.57; decimalLatitude: 36.96151; decimalLongitude: -3.41881; geodeticDatum: WGS84; **Event:** eventID: 1; samplingProtocol: Sweeping; eventTime: Day
- **Type status:** Other material. **Occurrence:** individualCount: 1; sex: female; **Location:** locationID: S2; continent: Europe; country: Spain; countryCode: ES; stateProvince: Andalucía; county: Granada; locality: Camarate; verbatimElevation: 1713.96; decimalLatitude: 37.18377; decimalLongitude: -3.26282; geodeticDatum: WGS84; **Event:** eventID: 1; samplingProtocol: Beating; eventTime: Day
- **Type status:** Other material. **Occurrence:** individualCount: 1; sex: male; **Location:** locationID: S2; continent: Europe; country: Spain; countryCode: ES; stateProvince: Andalucía; county: Granada; locality: Camarate; verbatimElevation: 1713.96; decimalLatitude: 37.18377; decimalLongitude: -3.26282; geodeticDatum: WGS84; **Event:** eventID: E; samplingProtocol: Pitfall
- **Type status:** Other material. **Occurrence:** individualCount: 2; sex: female; **Location:** locationID: S2; continent: Europe; country: Spain; countryCode: ES; stateProvince: Andalucía; county: Granada; locality: Camarate; verbatimElevation: 1713.96; decimalLatitude: 37.18377; decimalLongitude: -3.26282; geodeticDatum: WGS84; **Event:** eventID: 1; samplingProtocol: Sweeping; eventTime: Day

##### Distribution

Palearctic

#### Heliophanus tribulosus

Simon, 1868

##### Materials

- **Type status:** Other material. **Occurrence:** individualCount: 1; sex: female; **Location:** locationID: P4; continent: Europe; country: Spain; countryCode: ES; stateProvince: Castilla y León; county: León; locality: El Canto; verbatimElevation: 943.48; decimalLatitude: 43.17227; decimalLongitude: -4.90857; geodeticDatum: WGS84; **Event:** eventID: 1; samplingProtocol: Beating; eventTime: Day

##### Distribution

Europe to Kazakhstan

#### Heliophanus sp24

##### Materials

- **Type status:** Other material. **Occurrence:** individualCount: 1; sex: female; **Location:** locationID: M1; continent: Europe; country: Spain; countryCode: ES; stateProvince: Extremadura; county: Cáceres; locality: Peña Falcón; verbatimElevation: 320.6; decimalLatitude: 39.83296; decimalLongitude: -6.0641; geodeticDatum: WGS84; **Event:** eventID: 2; samplingProtocol: Beating; eventTime: Day
- **Type status:** Other material. **Occurrence:** individualCount: 1; sex: female; **Location:** locationID: M1; continent: Europe; country: Spain; countryCode: ES; stateProvince: Extremadura; county: Cáceres; locality: Peña Falcón; verbatimElevation: 320.6; decimalLatitude: 39.83296; decimalLongitude: -6.0641; geodeticDatum: WGS84; **Event:** eventID: 2; samplingProtocol: Sweeping; eventTime: Day
- **Type status:** Other material. **Occurrence:** individualCount: 1; sex: female; **Location:** locationID: M2; continent: Europe; country: Spain; countryCode: ES; stateProvince: Extremadura; county: Cáceres; locality: Fuente del Frances; verbatimElevation: 320.72; decimalLatitude: 39.828; decimalLongitude: -6.03249; geodeticDatum: WGS84; **Event:** eventID: 1; samplingProtocol: Beating; eventTime: Night

##### Distribution

?

##### Notes

This is a species of *Heliophanus* C. L. Koch, 1833, which we could not identify. We suspect it might be the undescribed female of *H. ibericus* Wesolowska, 1986, since its type locality is roughly 170 km away from the two sampling sites where our specimens were found.

#### Iberattus semiglabratus

(Simon, 1868)

##### Materials

- **Type status:** Other material. **Occurrence:** individualCount: 1; sex: male; **Location:** locationID: M1; continent: Europe; country: Spain; countryCode: ES; stateProvince: Extremadura; county: Cáceres; locality: Peña Falcón; verbatimElevation: 320.6; decimalLatitude: 39.83296; decimalLongitude: -6.0641; geodeticDatum: WGS84; **Event:** eventID: G; samplingProtocol: Pitfall
- **Type status:** Other material. **Occurrence:** individualCount: 1; sex: female; **Location:** locationID: P1; continent: Europe; country: Spain; countryCode: ES; stateProvince: Castilla y León; county: León; locality: Monte Robledo; verbatimElevation: 1071.58; decimalLatitude: 43.1445; decimalLongitude: -4.92675; geodeticDatum: WGS84; **Event:** eventID: 2; samplingProtocol: Ground; eventTime: Day
- **Type status:** Other material. **Occurrence:** individualCount: 1; sex: female; **Location:** locationID: P2; continent: Europe; country: Spain; countryCode: ES; stateProvince: Castilla y León; county: León; locality: Joyoguelas; verbatimElevation: 763.98; decimalLatitude: 43.17771; decimalLongitude: -4.90579; geodeticDatum: WGS84; **Event:** eventID: 1; samplingProtocol: Sweeping; eventTime: Day

##### Distribution

Iberian Peninsula, France

#### Icius hamatus

(C. L. Koch, 1846)

##### Materials

- **Type status:** Other material. **Occurrence:** individualCount: 1; sex: male; **Location:** locationID: C3; continent: Europe; country: Spain; countryCode: ES; stateProvince: Castilla-La Mancha; county: Ciudad Real; locality: La Quesera; verbatimElevation: 767.55; decimalLatitude: 39.36177; decimalLongitude: -4.41733; geodeticDatum: WGS84; **Event:** eventID: 1; samplingProtocol: Aerial; eventTime: Night
- **Type status:** Other material. **Occurrence:** individualCount: 1; sex: female; **Location:** locationID: C3; continent: Europe; country: Spain; countryCode: ES; stateProvince: Castilla-La Mancha; county: Ciudad Real; locality: La Quesera; verbatimElevation: 767.55; decimalLatitude: 39.36177; decimalLongitude: -4.41733; geodeticDatum: WGS84; **Event:** eventID: 1; samplingProtocol: Sweeping; eventTime: Day
- **Type status:** Other material. **Occurrence:** individualCount: 1; sex: female; **Location:** locationID: C3; continent: Europe; country: Spain; countryCode: ES; stateProvince: Castilla-La Mancha; county: Ciudad Real; locality: La Quesera; verbatimElevation: 767.55; decimalLatitude: 39.36177; decimalLongitude: -4.41733; geodeticDatum: WGS84; **Event:** eventID: 2; samplingProtocol: Sweeping; eventTime: Day
- **Type status:** Other material. **Occurrence:** individualCount: 1; sex: female; **Location:** locationID: M1; continent: Europe; country: Spain; countryCode: ES; stateProvince: Extremadura; county: Cáceres; locality: Peña Falcón; verbatimElevation: 320.6; decimalLatitude: 39.83296; decimalLongitude: -6.0641; geodeticDatum: WGS84; **Event:** eventID: 2; samplingProtocol: Aerial; eventTime: Night
- **Type status:** Other material. **Occurrence:** individualCount: 1; sex: female; **Location:** locationID: M1; continent: Europe; country: Spain; countryCode: ES; stateProvince: Extremadura; county: Cáceres; locality: Peña Falcón; verbatimElevation: 320.6; decimalLatitude: 39.83296; decimalLongitude: -6.0641; geodeticDatum: WGS84; **Event:** eventID: 1; samplingProtocol: Beating; eventTime: Day
- **Type status:** Other material. **Occurrence:** individualCount: 1; sex: female; **Location:** locationID: M1; continent: Europe; country: Spain; countryCode: ES; stateProvince: Extremadura; county: Cáceres; locality: Peña Falcón; verbatimElevation: 320.6; decimalLatitude: 39.83296; decimalLongitude: -6.0641; geodeticDatum: WGS84; **Event:** eventID: 2; samplingProtocol: Beating; eventTime: Day
- **Type status:** Other material. **Occurrence:** individualCount: 1; sex: male; **Location:** locationID: M1; continent: Europe; country: Spain; countryCode: ES; stateProvince: Extremadura; county: Cáceres; locality: Peña Falcón; verbatimElevation: 320.6; decimalLatitude: 39.83296; decimalLongitude: -6.0641; geodeticDatum: WGS84; **Event:** eventID: 1; samplingProtocol: Sweeping; eventTime: Night
- **Type status:** Other material. **Occurrence:** individualCount: 1; sex: female; **Location:** locationID: M2; continent: Europe; country: Spain; countryCode: ES; stateProvince: Extremadura; county: Cáceres; locality: Fuente del Frances; verbatimElevation: 320.72; decimalLatitude: 39.828; decimalLongitude: -6.03249; geodeticDatum: WGS84; **Event:** eventID: 1; samplingProtocol: Beating; eventTime: Night

##### Distribution

Palearctic

#### Macaroeris nidicolens

(Walckenaer, 1802)

##### Materials

- **Type status:** Other material. **Occurrence:** individualCount: 1; sex: female; **Location:** locationID: C1; continent: Europe; country: Spain; countryCode: ES; stateProvince: Castilla-La Mancha; county: Ciudad Real; locality: Valle Brezoso; verbatimElevation: 756.56; decimalLatitude: 39.35663; decimalLongitude: -4.35912; geodeticDatum: WGS84; **Event:** eventID: 1; samplingProtocol: Sweeping; eventTime: Day
- **Type status:** Other material. **Occurrence:** individualCount: 1; sex: female; **Location:** locationID: C2; continent: Europe; country: Spain; countryCode: ES; stateProvince: Castilla-La Mancha; county: Ciudad Real; locality: Valle Brezoso; verbatimElevation: 739.31; decimalLatitude: 39.35159; decimalLongitude: -4.3589; geodeticDatum: WGS84; **Event:** eventID: 1; samplingProtocol: Beating; eventTime: Day
- **Type status:** Other material. **Occurrence:** individualCount: 2; sex: female; **Location:** locationID: C3; continent: Europe; country: Spain; countryCode: ES; stateProvince: Castilla-La Mancha; county: Ciudad Real; locality: La Quesera; verbatimElevation: 767.55; decimalLatitude: 39.36177; decimalLongitude: -4.41733; geodeticDatum: WGS84; **Event:** eventID: 1; samplingProtocol: Beating; eventTime: Day
- **Type status:** Other material. **Occurrence:** individualCount: 1; sex: female; **Location:** locationID: C4; continent: Europe; country: Spain; countryCode: ES; stateProvince: Castilla-La Mancha; county: Ciudad Real; locality: La Quesera; verbatimElevation: 772.3; decimalLatitude: 39.36337; decimalLongitude: -4.41704; geodeticDatum: WGS84; **Event:** eventID: 1; samplingProtocol: Beating; eventTime: Day
- **Type status:** Other material. **Occurrence:** individualCount: 3; sex: female; **Location:** locationID: O1; continent: Europe; country: Spain; countryCode: ES; stateProvince: Aragón; county: Huesca; locality: O Furno; verbatimElevation: 1396.73; decimalLatitude: 42.60677; decimalLongitude: 0.13135; geodeticDatum: WGS84; **Event:** eventID: 1; samplingProtocol: Beating; eventTime: Day
- **Type status:** Other material. **Occurrence:** individualCount: 1; sex: female; **Location:** locationID: O2; continent: Europe; country: Spain; countryCode: ES; stateProvince: Aragón; county: Huesca; locality: Rebilla; verbatimElevation: 1158.13; decimalLatitude: 42.59427; decimalLongitude: 0.1529; geodeticDatum: WGS84; **Event:** eventID: 1; samplingProtocol: Beating; eventTime: Day
- **Type status:** Other material. **Occurrence:** individualCount: 2; sex: female; **Location:** locationID: O2; continent: Europe; country: Spain; countryCode: ES; stateProvince: Aragón; county: Huesca; locality: Rebilla; verbatimElevation: 1158.13; decimalLatitude: 42.59427; decimalLongitude: 0.1529; geodeticDatum: WGS84; **Event:** eventID: 2; samplingProtocol: Beating; eventTime: Day
- **Type status:** Other material. **Occurrence:** individualCount: 1; sex: male; **Location:** locationID: O2; continent: Europe; country: Spain; countryCode: ES; stateProvince: Aragón; county: Huesca; locality: Rebilla; verbatimElevation: 1158.13; decimalLatitude: 42.59427; decimalLongitude: 0.1529; geodeticDatum: WGS84; **Event:** eventID: 1; samplingProtocol: Beating; eventTime: Night
- **Type status:** Other material. **Occurrence:** individualCount: 1; sex: male; **Location:** locationID: S2; continent: Europe; country: Spain; countryCode: ES; stateProvince: Andalucía; county: Granada; locality: Camarate; verbatimElevation: 1713.96; decimalLatitude: 37.18377; decimalLongitude: -3.26282; geodeticDatum: WGS84; **Event:** eventID: 2; samplingProtocol: Beating; eventTime: Night

##### Distribution

Portugal to Cyprus, Turkey

#### Pellenes nigrociliatus

(Simon, 1875)

##### Materials

- **Type status:** Other material. **Occurrence:** individualCount: 1; sex: male; **Location:** locationID: O2; continent: Europe; country: Spain; countryCode: ES; stateProvince: Aragón; county: Huesca; locality: Rebilla; verbatimElevation: 1158.13; decimalLatitude: 42.59427; decimalLongitude: 0.1529; geodeticDatum: WGS84; **Event:** eventID: F; samplingProtocol: Pitfall

##### Distribution

Palearctic

#### Phlegra fasciata

(Hahn, 1826)

##### Materials

- **Type status:** Other material. **Occurrence:** individualCount: 1; sex: male; **Location:** locationID: P1; continent: Europe; country: Spain; countryCode: ES; stateProvince: Castilla y León; county: León; locality: Monte Robledo; verbatimElevation: 1071.58; decimalLatitude: 43.1445; decimalLongitude: -4.92675; geodeticDatum: WGS84; **Event:** eventID: D; samplingProtocol: Pitfall

##### Distribution

Palearctic

#### Pseudeuophrys erratica

(Walckenaer, 1826)

##### Materials

- **Type status:** Other material. **Occurrence:** individualCount: 2; sex: male; **Location:** locationID: P1; continent: Europe; country: Spain; countryCode: ES; stateProvince: Castilla y León; county: León; locality: Monte Robledo; verbatimElevation: 1071.58; decimalLatitude: 43.1445; decimalLongitude: -4.92675; geodeticDatum: WGS84; **Event:** eventID: 1; samplingProtocol: Sweeping; eventTime: Night

##### Distribution

Palearctic (USA, introduced)

#### Pseudeuophrys nebrodensis

Alicata & Cantarella, 2000

##### Materials

- **Type status:** Other material. **Occurrence:** individualCount: 1; sex: male; **Location:** locationID: S1; continent: Europe; country: Spain; countryCode: ES; stateProvince: Andalucía; county: Granada; locality: Soportujar; verbatimElevation: 1786.57; decimalLatitude: 36.96151; decimalLongitude: -3.41881; geodeticDatum: WGS84; **Event:** eventID: D; samplingProtocol: Pitfall

##### Distribution

Sicily

##### Notes

**Figure 11.**
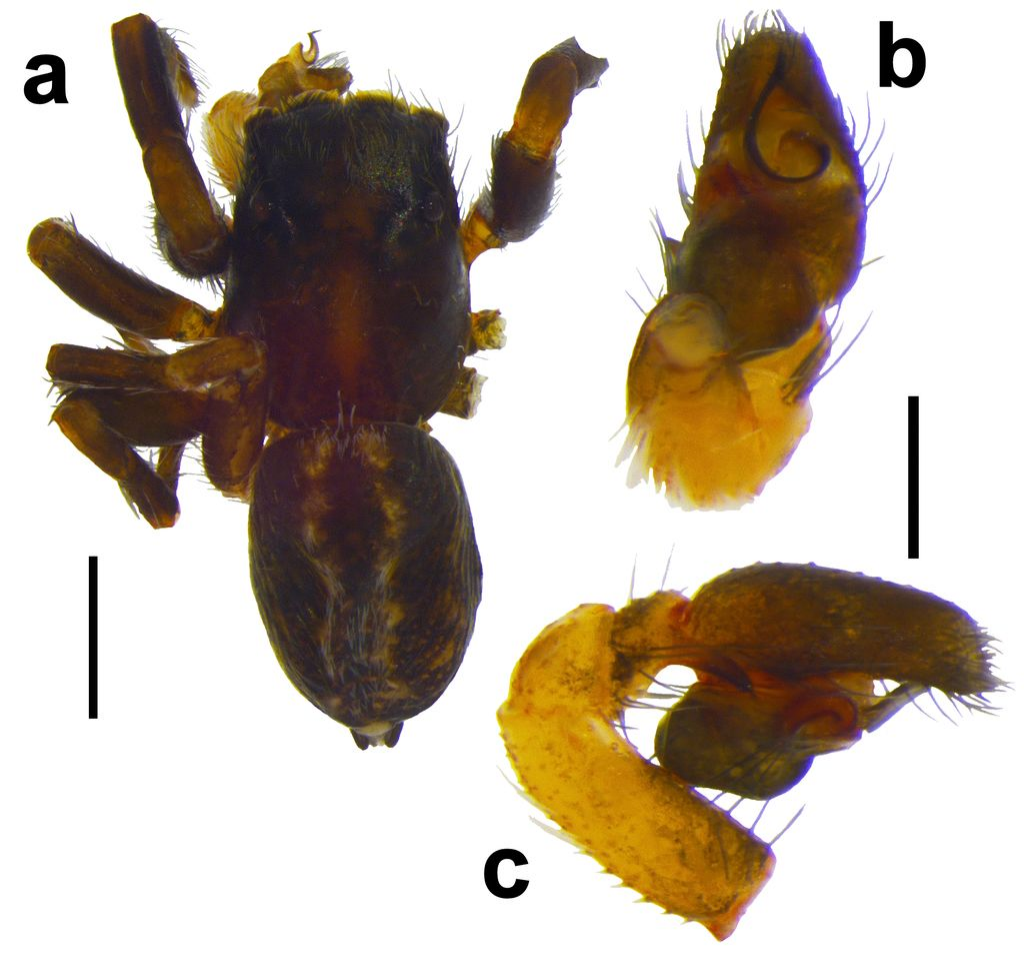
*Pseudeuophrys nebrodensis* Alicata & Cantarella, 2000. a: male, dorsal habitus, scale = 0.8 mm; b: left male palp, ventral, scale = 0.2 mm; c: left male palp, retrolateral, scale = 0.2 mm.

First record for the Iberian Peninsula. See Fig. 11.

#### Salticus scenicus

(Clerck, 1757)

##### Materials

- **Type status:** Other material. **Occurrence:** individualCount: 1; sex: female; **Location:** locationID: A1; continent: Europe; country: Spain; countryCode: ES; stateProvince: Catalonia; county: Lleida; locality: Sola de Boi; verbatimElevation: 1759.8; decimalLatitude: 42.54958; decimalLongitude: 0.87254; geodeticDatum: WGS84; **Event:** eventID: 1; samplingProtocol: Sweeping; eventTime: Night
- **Type status:** Other material. **Occurrence:** individualCount: 1; sex: female; **Location:** locationID: C2; continent: Europe; country: Spain; countryCode: ES; stateProvince: Castilla-La Mancha; county: Ciudad Real; locality: Valle Brezoso; verbatimElevation: 739.31; decimalLatitude: 39.35159; decimalLongitude: -4.3589; geodeticDatum: WGS84; **Event:** eventID: 4; samplingProtocol: Aerial; eventTime: Night
- **Type status:** Other material. **Occurrence:** individualCount: 1; sex: male; **Location:** locationID: C2; continent: Europe; country: Spain; countryCode: ES; stateProvince: Castilla-La Mancha; county: Ciudad Real; locality: Valle Brezoso; verbatimElevation: 739.31; decimalLatitude: 39.35159; decimalLongitude: -4.3589; geodeticDatum: WGS84; **Event:** eventID: 2; samplingProtocol: Beating; eventTime: Night
- **Type status:** Other material. **Occurrence:** individualCount: 2; sex: female; **Location:** locationID: C2; continent: Europe; country: Spain; countryCode: ES; stateProvince: Castilla-La Mancha; county: Ciudad Real; locality: Valle Brezoso; verbatimElevation: 739.31; decimalLatitude: 39.35159; decimalLongitude: -4.3589; geodeticDatum: WGS84; **Event:** eventID: 2; samplingProtocol: Beating; eventTime: Day
- **Type status:** Other material. **Occurrence:** individualCount: 1; sex: female; **Location:** locationID: C2; continent: Europe; country: Spain; countryCode: ES; stateProvince: Castilla-La Mancha; county: Ciudad Real; locality: Valle Brezoso; verbatimElevation: 739.31; decimalLatitude: 39.35159; decimalLongitude: -4.3589; geodeticDatum: WGS84; **Event:** eventID: 1; samplingProtocol: Sweeping; eventTime: Day
- **Type status:** Other material. **Occurrence:** individualCount: 1; sex: male; **Location:** locationID: C3; continent: Europe; country: Spain; countryCode: ES; stateProvince: Castilla-La Mancha; county: Ciudad Real; locality: La Quesera; verbatimElevation: 767.55; decimalLatitude: 39.36177; decimalLongitude: -4.41733; geodeticDatum: WGS84; **Event:** eventID: 1; samplingProtocol: Aerial; eventTime: Night
- **Type status:** Other material. **Occurrence:** individualCount: 1; sex: male; **Location:** locationID: C3; continent: Europe; country: Spain; countryCode: ES; stateProvince: Castilla-La Mancha; county: Ciudad Real; locality: La Quesera; verbatimElevation: 767.55; decimalLatitude: 39.36177; decimalLongitude: -4.41733; geodeticDatum: WGS84; **Event:** eventID: 2; samplingProtocol: Beating; eventTime: Day
- **Type status:** Other material. **Occurrence:** individualCount: 1; sex: female; **Location:** locationID: C4; continent: Europe; country: Spain; countryCode: ES; stateProvince: Castilla-La Mancha; county: Ciudad Real; locality: La Quesera; verbatimElevation: 772.3; decimalLatitude: 39.36337; decimalLongitude: -4.41704; geodeticDatum: WGS84; **Event:** eventID: 1; samplingProtocol: Aerial; eventTime: Night
- **Type status:** Other material. **Occurrence:** individualCount: 1; sex: male; **Location:** locationID: C4; continent: Europe; country: Spain; countryCode: ES; stateProvince: Castilla-La Mancha; county: Ciudad Real; locality: La Quesera; verbatimElevation: 772.3; decimalLatitude: 39.36337; decimalLongitude: -4.41704; geodeticDatum: WGS84; **Event:** eventID: 2; samplingProtocol: Aerial; eventTime: Night
- **Type status:** Other material. **Occurrence:** individualCount: 2; sex: female; **Location:** locationID: C4; continent: Europe; country: Spain; countryCode: ES; stateProvince: Castilla-La Mancha; county: Ciudad Real; locality: La Quesera; verbatimElevation: 772.3; decimalLatitude: 39.36337; decimalLongitude: -4.41704; geodeticDatum: WGS84; **Event:** eventID: 1; samplingProtocol: Beating; eventTime: Day
- **Type status:** Other material. **Occurrence:** individualCount: 1; sex: male; **Location:** locationID: C4; continent: Europe; country: Spain; countryCode: ES; stateProvince: Castilla-La Mancha; county: Ciudad Real; locality: La Quesera; verbatimElevation: 772.3; decimalLatitude: 39.36337; decimalLongitude: -4.41704; geodeticDatum: WGS84; **Event:** eventID: 1; samplingProtocol: Beating; eventTime: Day
- **Type status:** Other material. **Occurrence:** individualCount: 2; sex: male; **Location:** locationID: M1; continent: Europe; country: Spain; countryCode: ES; stateProvince: Extremadura; county: Cáceres; locality: Peña Falcón; verbatimElevation: 320.6; decimalLatitude: 39.83296; decimalLongitude: -6.0641; geodeticDatum: WGS84; **Event:** eventID: 1; samplingProtocol: Beating; eventTime: Day
- **Type status:** Other material. **Occurrence:** individualCount: 2; sex: female; **Location:** locationID: M1; continent: Europe; country: Spain; countryCode: ES; stateProvince: Extremadura; county: Cáceres; locality: Peña Falcón; verbatimElevation: 320.6; decimalLatitude: 39.83296; decimalLongitude: -6.0641; geodeticDatum: WGS84; **Event:** eventID: 2; samplingProtocol: Beating; eventTime: Day
- **Type status:** Other material. **Occurrence:** individualCount: 1; sex: male; **Location:** locationID: M1; continent: Europe; country: Spain; countryCode: ES; stateProvince: Extremadura; county: Cáceres; locality: Peña Falcón; verbatimElevation: 320.6; decimalLatitude: 39.83296; decimalLongitude: -6.0641; geodeticDatum: WGS84; **Event:** eventID: E; samplingProtocol: Pitfall
- **Type status:** Other material. **Occurrence:** individualCount: 1; sex: male; **Location:** locationID: M2; continent: Europe; country: Spain; countryCode: ES; stateProvince: Extremadura; county: Cáceres; locality: Fuente del Frances; verbatimElevation: 320.72; decimalLatitude: 39.828; decimalLongitude: -6.03249; geodeticDatum: WGS84; **Event:** eventID: 1; samplingProtocol: Aerial; eventTime: Night
- **Type status:** Other material. **Occurrence:** individualCount: 1; sex: female; **Location:** locationID: M2; continent: Europe; country: Spain; countryCode: ES; stateProvince: Extremadura; county: Cáceres; locality: Fuente del Frances; verbatimElevation: 320.72; decimalLatitude: 39.828; decimalLongitude: -6.03249; geodeticDatum: WGS84; **Event:** eventID: 1; samplingProtocol: Beating; eventTime: Day
- **Type status:** Other material. **Occurrence:** individualCount: 1; sex: female; **Location:** locationID: M2; continent: Europe; country: Spain; countryCode: ES; stateProvince: Extremadura; county: Cáceres; locality: Fuente del Frances; verbatimElevation: 320.72; decimalLatitude: 39.828; decimalLongitude: -6.03249; geodeticDatum: WGS84; **Event:** eventID: 2; samplingProtocol: Beating; eventTime: Day
- **Type status:** Other material. **Occurrence:** individualCount: 1; sex: female; **Location:** locationID: O2; continent: Europe; country: Spain; countryCode: ES; stateProvince: Aragón; county: Huesca; locality: Rebilla; verbatimElevation: 1158.13; decimalLatitude: 42.59427; decimalLongitude: 0.1529; geodeticDatum: WGS84; **Event:** eventID: 1; samplingProtocol: Aerial; eventTime: Night
- **Type status:** Other material. **Occurrence:** individualCount: 1; sex: male; **Location:** locationID: O2; continent: Europe; country: Spain; countryCode: ES; stateProvince: Aragón; county: Huesca; locality: Rebilla; verbatimElevation: 1158.13; decimalLatitude: 42.59427; decimalLongitude: 0.1529; geodeticDatum: WGS84; **Event:** eventID: 2; samplingProtocol: Sweeping; eventTime: Day
- **Type status:** Other material. **Occurrence:** individualCount: 2; sex: female; **Location:** locationID: P4; continent: Europe; country: Spain; countryCode: ES; stateProvince: Castilla y León; county: León; locality: El Canto; verbatimElevation: 943.48; decimalLatitude: 43.17227; decimalLongitude: -4.90857; geodeticDatum: WGS84; **Event:** eventID: 1; samplingProtocol: Beating; eventTime: Day
- **Type status:** Other material. **Occurrence:** individualCount: 1; sex: male; **Location:** locationID: P4; continent: Europe; country: Spain; countryCode: ES; stateProvince: Castilla y León; county: León; locality: El Canto; verbatimElevation: 943.48; decimalLatitude: 43.17227; decimalLongitude: -4.90857; geodeticDatum: WGS84; **Event:** eventID: 1; samplingProtocol: Beating; eventTime: Day
- **Type status:** Other material. **Occurrence:** individualCount: 1; sex: male; **Location:** locationID: S1; continent: Europe; country: Spain; countryCode: ES; stateProvince: Andalucía; county: Granada; locality: Soportujar; verbatimElevation: 1786.57; decimalLatitude: 36.96151; decimalLongitude: -3.41881; geodeticDatum: WGS84; **Event:** eventID: 1; samplingProtocol: Beating; eventTime: Day
- **Type status:** Other material. **Occurrence:** individualCount: 3; sex: female; **Location:** locationID: S1; continent: Europe; country: Spain; countryCode: ES; stateProvince: Andalucía; county: Granada; locality: Soportujar; verbatimElevation: 1786.57; decimalLatitude: 36.96151; decimalLongitude: -3.41881; geodeticDatum: WGS84; **Event:** eventID: 2; samplingProtocol: Beating; eventTime: Night

##### Distribution

Holarctic

#### Salticus zebraneus

(C. L. Koch, 1837)

##### Materials

- **Type status:** Other material. **Occurrence:** individualCount: 1; sex: female; **Location:** locationID: O1; continent: Europe; country: Spain; countryCode: ES; stateProvince: Aragón; county: Huesca; locality: O Furno; verbatimElevation: 1396.73; decimalLatitude: 42.60677; decimalLongitude: 0.13135; geodeticDatum: WGS84; **Event:** eventID: 2; samplingProtocol: Beating; eventTime: Day
- **Type status:** Other material. **Occurrence:** individualCount: 1; sex: male; **Location:** locationID: O2; continent: Europe; country: Spain; countryCode: ES; stateProvince: Aragón; county: Huesca; locality: Rebilla; verbatimElevation: 1158.13; decimalLatitude: 42.59427; decimalLongitude: 0.1529; geodeticDatum: WGS84; **Event:** eventID: 2; samplingProtocol: Aerial; eventTime: Night
- **Type status:** Other material. **Occurrence:** individualCount: 1; sex: male; **Location:** locationID: S2; continent: Europe; country: Spain; countryCode: ES; stateProvince: Andalucía; county: Granada; locality: Camarate; verbatimElevation: 1713.96; decimalLatitude: 37.18377; decimalLongitude: -3.26282; geodeticDatum: WGS84; **Event:** eventID: 2; samplingProtocol: Beating; eventTime: Day

##### Distribution

Palearctic

#### Sittipub pubescens

(Fabricius, 1775)

##### Materials

- **Type status:** Other material. **Occurrence:** individualCount: 1; sex: female; **Location:** locationID: A2; continent: Europe; country: Spain; countryCode: ES; stateProvince: Catalonia; county: Lleida; locality: Sola de Boi; verbatimElevation: 1738.7; decimalLatitude: 42.54913; decimalLongitude: 0.87137; geodeticDatum: WGS84; **Event:** eventID: 1; samplingProtocol: Aerial; eventTime: Night

##### Distribution

Europe, Turkey, Morocco, Russia, USA

#### Scytodidae

Blackwall, 1864

#### Scytodes velutina

Heineken & Lowe, 1832

##### Materials

- **Type status:** Other material. **Occurrence:** individualCount: 1; sex: male; **Location:** locationID: C3; continent: Europe; country: Spain; countryCode: ES; stateProvince: Castilla-La Mancha; county: Ciudad Real; locality: La Quesera; verbatimElevation: 767.55; decimalLatitude: 39.36177; decimalLongitude: -4.41733; geodeticDatum: WGS84; **Event:** eventID: C; samplingProtocol: Pitfall
- **Type status:** Other material. **Occurrence:** individualCount: 1; sex: male; **Location:** locationID: C3; continent: Europe; country: Spain; countryCode: ES; stateProvince: Castilla-La Mancha; county: Ciudad Real; locality: La Quesera; verbatimElevation: 767.55; decimalLatitude: 39.36177; decimalLongitude: -4.41733; geodeticDatum: WGS84; **Event:** eventID: E; samplingProtocol: Pitfall
- **Type status:** Other material. **Occurrence:** individualCount: 2; sex: male; **Location:** locationID: C3; continent: Europe; country: Spain; countryCode: ES; stateProvince: Castilla-La Mancha; county: Ciudad Real; locality: La Quesera; verbatimElevation: 767.55; decimalLatitude: 39.36177; decimalLongitude: -4.41733; geodeticDatum: WGS84; **Event:** eventID: G; samplingProtocol: Pitfall
- **Type status:** Other material. **Occurrence:** individualCount: 1; sex: male; **Location:** locationID: C4; continent: Europe; country: Spain; countryCode: ES; stateProvince: Castilla-La Mancha; county: Ciudad Real; locality: La Quesera; verbatimElevation: 772.3; decimalLatitude: 39.36337; decimalLongitude: -4.41704; geodeticDatum: WGS84; **Event:** eventID: C; samplingProtocol: Pitfall
- **Type status:** Other material. **Occurrence:** individualCount: 1; sex: male; **Location:** locationID: M1; continent: Europe; country: Spain; countryCode: ES; stateProvince: Extremadura; county: Cáceres; locality: Peña Falcón; verbatimElevation: 320.6; decimalLatitude: 39.83296; decimalLongitude: -6.0641; geodeticDatum: WGS84; **Event:** eventID: 4; samplingProtocol: Aerial; eventTime: Night
- **Type status:** Other material. **Occurrence:** individualCount: 1; sex: male; **Location:** locationID: M1; continent: Europe; country: Spain; countryCode: ES; stateProvince: Extremadura; county: Cáceres; locality: Peña Falcón; verbatimElevation: 320.6; decimalLatitude: 39.83296; decimalLongitude: -6.0641; geodeticDatum: WGS84; **Event:** eventID: E; samplingProtocol: Pitfall
- **Type status:** Other material. **Occurrence:** individualCount: 2; sex: male; **Location:** locationID: M1; continent: Europe; country: Spain; countryCode: ES; stateProvince: Extremadura; county: Cáceres; locality: Peña Falcón; verbatimElevation: 320.6; decimalLatitude: 39.83296; decimalLongitude: -6.0641; geodeticDatum: WGS84; **Event:** eventID: F; samplingProtocol: Pitfall
- **Type status:** Other material. **Occurrence:** individualCount: 1; sex: male; **Location:** locationID: M1; continent: Europe; country: Spain; countryCode: ES; stateProvince: Extremadura; county: Cáceres; locality: Peña Falcón; verbatimElevation: 320.6; decimalLatitude: 39.83296; decimalLongitude: -6.0641; geodeticDatum: WGS84; **Event:** eventID: G; samplingProtocol: Pitfall
- **Type status:** Other material. **Occurrence:** individualCount: 1; sex: female; **Location:** locationID: M1; continent: Europe; country: Spain; countryCode: ES; stateProvince: Extremadura; county: Cáceres; locality: Peña Falcón; verbatimElevation: 320.6; decimalLatitude: 39.83296; decimalLongitude: -6.0641; geodeticDatum: WGS84; **Event:** eventID: H; samplingProtocol: Pitfall
- **Type status:** Other material. **Occurrence:** individualCount: 1; sex: male; **Location:** locationID: M1; continent: Europe; country: Spain; countryCode: ES; stateProvince: Extremadura; county: Cáceres; locality: Peña Falcón; verbatimElevation: 320.6; decimalLatitude: 39.83296; decimalLongitude: -6.0641; geodeticDatum: WGS84; **Event:** eventID: L; samplingProtocol: Pitfall
- **Type status:** Other material. **Occurrence:** individualCount: 1; sex: male; **Location:** locationID: M2; continent: Europe; country: Spain; countryCode: ES; stateProvince: Extremadura; county: Cáceres; locality: Fuente del Frances; verbatimElevation: 320.72; decimalLatitude: 39.828; decimalLongitude: -6.03249; geodeticDatum: WGS84; **Event:** eventID: D; samplingProtocol: Pitfall
- **Type status:** Other material. **Occurrence:** individualCount: 1; sex: male; **Location:** locationID: M2; continent: Europe; country: Spain; countryCode: ES; stateProvince: Extremadura; county: Cáceres; locality: Fuente del Frances; verbatimElevation: 320.72; decimalLatitude: 39.828; decimalLongitude: -6.03249; geodeticDatum: WGS84; **Event:** eventID: L; samplingProtocol: Pitfall

##### Distribution

Mediterranean, Cape Verde Islands, Seychelles

#### Segestriidae

Simon, 1893

#### Segestria bavarica

C. L. Koch, 1843

##### Materials

- **Type status:** Other material. **Occurrence:** individualCount: 1; sex: female; **Location:** locationID: A2; continent: Europe; country: Spain; countryCode: ES; stateProvince: Catalonia; county: Lleida; locality: Sola de Boi; verbatimElevation: 1738.7; decimalLatitude: 42.54913; decimalLongitude: 0.87137; geodeticDatum: WGS84; **Event:** eventID: 1; samplingProtocol: Aerial; eventTime: Night
- **Type status:** Other material. **Occurrence:** individualCount: 1; sex: female; **Location:** locationID: P4; continent: Europe; country: Spain; countryCode: ES; stateProvince: Castilla y León; county: León; locality: El Canto; verbatimElevation: 943.48; decimalLatitude: 43.17227; decimalLongitude: -4.90857; geodeticDatum: WGS84; **Event:** eventID: 2; samplingProtocol: Aerial; eventTime: Night

##### Distribution

Europe to Azerbaijan

#### Segestria florentina

(Rossi, 1790)

##### Materials

- **Type status:** Other material. **Occurrence:** individualCount: 1; sex: female; **Location:** locationID: C3; continent: Europe; country: Spain; countryCode: ES; stateProvince: Castilla-La Mancha; county: Ciudad Real; locality: La Quesera; verbatimElevation: 767.55; decimalLatitude: 39.36177; decimalLongitude: -4.41733; geodeticDatum: WGS84; **Event:** eventID: 3; samplingProtocol: Aerial; eventTime: Night

##### Distribution

Europe to Georgia, Brazil, Uruguay, Argentina

#### Segestria senoculata

(Linnaeus, 1758)

##### Materials

- **Type status:** Other material. **Occurrence:** individualCount: 3; sex: male; **Location:** locationID: A1; continent: Europe; country: Spain; countryCode: ES; stateProvince: Catalonia; county: Lleida; locality: Sola de Boi; verbatimElevation: 1759.8; decimalLatitude: 42.54958; decimalLongitude: 0.87254; geodeticDatum: WGS84; **Event:** eventID: 1; samplingProtocol: Aerial; eventTime: Night
- **Type status:** Other material. **Occurrence:** individualCount: 1; sex: female; **Location:** locationID: A1; continent: Europe; country: Spain; countryCode: ES; stateProvince: Catalonia; county: Lleida; locality: Sola de Boi; verbatimElevation: 1759.8; decimalLatitude: 42.54958; decimalLongitude: 0.87254; geodeticDatum: WGS84; **Event:** eventID: 1; samplingProtocol: Ground; eventTime: Night
- **Type status:** Other material. **Occurrence:** individualCount: 1; sex: female; **Location:** locationID: A1; continent: Europe; country: Spain; countryCode: ES; stateProvince: Catalonia; county: Lleida; locality: Sola de Boi; verbatimElevation: 1759.8; decimalLatitude: 42.54958; decimalLongitude: 0.87254; geodeticDatum: WGS84; **Event:** eventID: C; samplingProtocol: Pitfall
- **Type status:** Other material. **Occurrence:** individualCount: 2; sex: male; **Location:** locationID: A1; continent: Europe; country: Spain; countryCode: ES; stateProvince: Catalonia; county: Lleida; locality: Sola de Boi; verbatimElevation: 1759.8; decimalLatitude: 42.54958; decimalLongitude: 0.87254; geodeticDatum: WGS84; **Event:** eventID: H; samplingProtocol: Pitfall
- **Type status:** Other material. **Occurrence:** individualCount: 1; sex: female; **Location:** locationID: O2; continent: Europe; country: Spain; countryCode: ES; stateProvince: Aragón; county: Huesca; locality: Rebilla; verbatimElevation: 1158.13; decimalLatitude: 42.59427; decimalLongitude: 0.1529; geodeticDatum: WGS84; **Event:** eventID: 1; samplingProtocol: Aerial; eventTime: Night
- **Type status:** Other material. **Occurrence:** individualCount: 1; sex: female; **Location:** locationID: P1; continent: Europe; country: Spain; countryCode: ES; stateProvince: Castilla y León; county: León; locality: Monte Robledo; verbatimElevation: 1071.58; decimalLatitude: 43.1445; decimalLongitude: -4.92675; geodeticDatum: WGS84; **Event:** eventID: 2; samplingProtocol: Ground; eventTime: Day
- **Type status:** Other material. **Occurrence:** individualCount: 1; sex: male; **Location:** locationID: P1; continent: Europe; country: Spain; countryCode: ES; stateProvince: Castilla y León; county: León; locality: Monte Robledo; verbatimElevation: 1071.58; decimalLatitude: 43.1445; decimalLongitude: -4.92675; geodeticDatum: WGS84; **Event:** eventID: C; samplingProtocol: Pitfall
- **Type status:** Other material. **Occurrence:** individualCount: 1; sex: male; **Location:** locationID: P1; continent: Europe; country: Spain; countryCode: ES; stateProvince: Castilla y León; county: León; locality: Monte Robledo; verbatimElevation: 1071.58; decimalLatitude: 43.1445; decimalLongitude: -4.92675; geodeticDatum: WGS84; **Event:** eventID: D; samplingProtocol: Pitfall
- **Type status:** Other material. **Occurrence:** individualCount: 2; sex: male; **Location:** locationID: P3; continent: Europe; country: Spain; countryCode: ES; stateProvince: Castilla y León; county: León; locality: Las Arroyas; verbatimElevation: 1097.1; decimalLatitude: 43.14351; decimalLongitude: -4.94878; geodeticDatum: WGS84; **Event:** eventID: 1; samplingProtocol: Aerial; eventTime: Night
- **Type status:** Other material. **Occurrence:** individualCount: 2; sex: female; **Location:** locationID: P3; continent: Europe; country: Spain; countryCode: ES; stateProvince: Castilla y León; county: León; locality: Las Arroyas; verbatimElevation: 1097.1; decimalLatitude: 43.14351; decimalLongitude: -4.94878; geodeticDatum: WGS84; **Event:** eventID: 2; samplingProtocol: Aerial; eventTime: Night

##### Distribution

Palearctic

#### Sicariidae

Keyserling, 1880

#### Loxosceles rufescens

(Dufour, 1820)

##### Materials

- **Type status:** Other material. **Occurrence:** individualCount: 1; sex: male; **Location:** locationID: M1; continent: Europe; country: Spain; countryCode: ES; stateProvince: Extremadura; county: Cáceres; locality: Peña Falcón; verbatimElevation: 320.6; decimalLatitude: 39.83296; decimalLongitude: -6.0641; geodeticDatum: WGS84; **Event:** eventID: A; samplingProtocol: Pitfall
- **Type status:** Other material. **Occurrence:** individualCount: 1; sex: female; **Location:** locationID: M1; continent: Europe; country: Spain; countryCode: ES; stateProvince: Extremadura; county: Cáceres; locality: Peña Falcón; verbatimElevation: 320.6; decimalLatitude: 39.83296; decimalLongitude: -6.0641; geodeticDatum: WGS84; **Event:** eventID: B; samplingProtocol: Pitfall
- **Type status:** Other material. **Occurrence:** individualCount: 1; sex: male; **Location:** locationID: M1; continent: Europe; country: Spain; countryCode: ES; stateProvince: Extremadura; county: Cáceres; locality: Peña Falcón; verbatimElevation: 320.6; decimalLatitude: 39.83296; decimalLongitude: -6.0641; geodeticDatum: WGS84; **Event:** eventID: C; samplingProtocol: Pitfall
- **Type status:** Other material. **Occurrence:** individualCount: 1; sex: female; **Location:** locationID: M1; continent: Europe; country: Spain; countryCode: ES; stateProvince: Extremadura; county: Cáceres; locality: Peña Falcón; verbatimElevation: 320.6; decimalLatitude: 39.83296; decimalLongitude: -6.0641; geodeticDatum: WGS84; **Event:** eventID: D; samplingProtocol: Pitfall
- **Type status:** Other material. **Occurrence:** individualCount: 1; sex: male; **Location:** locationID: M1; continent: Europe; country: Spain; countryCode: ES; stateProvince: Extremadura; county: Cáceres; locality: Peña Falcón; verbatimElevation: 320.6; decimalLatitude: 39.83296; decimalLongitude: -6.0641; geodeticDatum: WGS84; **Event:** eventID: D; samplingProtocol: Pitfall
- **Type status:** Other material. **Occurrence:** individualCount: 1; sex: male; **Location:** locationID: M1; continent: Europe; country: Spain; countryCode: ES; stateProvince: Extremadura; county: Cáceres; locality: Peña Falcón; verbatimElevation: 320.6; decimalLatitude: 39.83296; decimalLongitude: -6.0641; geodeticDatum: WGS84; **Event:** eventID: E; samplingProtocol: Pitfall
- **Type status:** Other material. **Occurrence:** individualCount: 3; sex: male; **Location:** locationID: M1; continent: Europe; country: Spain; countryCode: ES; stateProvince: Extremadura; county: Cáceres; locality: Peña Falcón; verbatimElevation: 320.6; decimalLatitude: 39.83296; decimalLongitude: -6.0641; geodeticDatum: WGS84; **Event:** eventID: G; samplingProtocol: Pitfall
- **Type status:** Other material. **Occurrence:** individualCount: 3; sex: male; **Location:** locationID: M1; continent: Europe; country: Spain; countryCode: ES; stateProvince: Extremadura; county: Cáceres; locality: Peña Falcón; verbatimElevation: 320.6; decimalLatitude: 39.83296; decimalLongitude: -6.0641; geodeticDatum: WGS84; **Event:** eventID: H; samplingProtocol: Pitfall
- **Type status:** Other material. **Occurrence:** individualCount: 1; sex: male; **Location:** locationID: M1; continent: Europe; country: Spain; countryCode: ES; stateProvince: Extremadura; county: Cáceres; locality: Peña Falcón; verbatimElevation: 320.6; decimalLatitude: 39.83296; decimalLongitude: -6.0641; geodeticDatum: WGS84; **Event:** eventID: I; samplingProtocol: Pitfall
- **Type status:** Other material. **Occurrence:** individualCount: 1; sex: female; **Location:** locationID: M1; continent: Europe; country: Spain; countryCode: ES; stateProvince: Extremadura; county: Cáceres; locality: Peña Falcón; verbatimElevation: 320.6; decimalLatitude: 39.83296; decimalLongitude: -6.0641; geodeticDatum: WGS84; **Event:** eventID: J; samplingProtocol: Pitfall
- **Type status:** Other material. **Occurrence:** individualCount: 1; sex: male; **Location:** locationID: M1; continent: Europe; country: Spain; countryCode: ES; stateProvince: Extremadura; county: Cáceres; locality: Peña Falcón; verbatimElevation: 320.6; decimalLatitude: 39.83296; decimalLongitude: -6.0641; geodeticDatum: WGS84; **Event:** eventID: J; samplingProtocol: Pitfall
- **Type status:** Other material. **Occurrence:** individualCount: 1; sex: male; **Location:** locationID: M1; continent: Europe; country: Spain; countryCode: ES; stateProvince: Extremadura; county: Cáceres; locality: Peña Falcón; verbatimElevation: 320.6; decimalLatitude: 39.83296; decimalLongitude: -6.0641; geodeticDatum: WGS84; **Event:** eventID: K; samplingProtocol: Pitfall
- **Type status:** Other material. **Occurrence:** individualCount: 1; sex: male; **Location:** locationID: M2; continent: Europe; country: Spain; countryCode: ES; stateProvince: Extremadura; county: Cáceres; locality: Fuente del Frances; verbatimElevation: 320.72; decimalLatitude: 39.828; decimalLongitude: -6.03249; geodeticDatum: WGS84; **Event:** eventID: H; samplingProtocol: Pitfall

##### Distribution

Cosmopolitan

#### Sparassidae

Bertkau, 1872

#### Eusparassus dufouri

Simon, 1932

##### Materials

- **Type status:** Other material. **Occurrence:** individualCount: 1; sex: female; **Location:** locationID: M1; continent: Europe; country: Spain; countryCode: ES; stateProvince: Extremadura; county: Cáceres; locality: Peña Falcón; verbatimElevation: 320.6; decimalLatitude: 39.83296; decimalLongitude: -6.0641; geodeticDatum: WGS84; **Event:** eventID: 1; samplingProtocol: Aerial; eventTime: Night
- **Type status:** Other material. **Occurrence:** individualCount: 1; sex: male; **Location:** locationID: M1; continent: Europe; country: Spain; countryCode: ES; stateProvince: Extremadura; county: Cáceres; locality: Peña Falcón; verbatimElevation: 320.6; decimalLatitude: 39.83296; decimalLongitude: -6.0641; geodeticDatum: WGS84; **Event:** eventID: I; samplingProtocol: Pitfall
- **Type status:** Other material. **Occurrence:** individualCount: 1; sex: male; **Location:** locationID: M2; continent: Europe; country: Spain; countryCode: ES; stateProvince: Extremadura; county: Cáceres; locality: Fuente del Frances; verbatimElevation: 320.72; decimalLatitude: 39.828; decimalLongitude: -6.03249; geodeticDatum: WGS84; **Event:** eventID: 2; samplingProtocol: Aerial; eventTime: Night
- **Type status:** Other material. **Occurrence:** individualCount: 1; sex: female; **Location:** locationID: M2; continent: Europe; country: Spain; countryCode: ES; stateProvince: Extremadura; county: Cáceres; locality: Fuente del Frances; verbatimElevation: 320.72; decimalLatitude: 39.828; decimalLongitude: -6.03249; geodeticDatum: WGS84; **Event:** eventID: 3; samplingProtocol: Aerial; eventTime: Night

##### Distribution

Iberian Peninsula

#### Micrommata aljibica

Urones, 2004

##### Materials

- **Type status:** Other material. **Occurrence:** individualCount: 1; sex: female; **Location:** locationID: S2; continent: Europe; country: Spain; countryCode: ES; stateProvince: Andalucía; county: Granada; locality: Camarate; verbatimElevation: 1713.96; decimalLatitude: 37.18377; decimalLongitude: -3.26282; geodeticDatum: WGS84; **Event:** eventID: 4; samplingProtocol: Aerial; eventTime: Night
- **Type status:** Other material. **Occurrence:** individualCount: 1; sex: male; **Location:** locationID: S2; continent: Europe; country: Spain; countryCode: ES; stateProvince: Andalucía; county: Granada; locality: Camarate; verbatimElevation: 1713.96; decimalLatitude: 37.18377; decimalLongitude: -3.26282; geodeticDatum: WGS84; **Event:** eventID: D; samplingProtocol: Pitfall

##### Distribution

Spain

#### Micrommata virescens

(Clerck, 1757)

##### Materials

- **Type status:** Other material. **Occurrence:** individualCount: 1; sex: male; **Location:** locationID: O1; continent: Europe; country: Spain; countryCode: ES; stateProvince: Aragón; county: Huesca; locality: O Furno; verbatimElevation: 1396.73; decimalLatitude: 42.60677; decimalLongitude: 0.13135; geodeticDatum: WGS84; **Event:** eventID: 1; samplingProtocol: Sweeping; eventTime: Day
- **Type status:** Other material. **Occurrence:** individualCount: 1; sex: female; **Location:** locationID: P4; continent: Europe; country: Spain; countryCode: ES; stateProvince: Castilla y León; county: León; locality: El Canto; verbatimElevation: 943.48; decimalLatitude: 43.17227; decimalLongitude: -4.90857; geodeticDatum: WGS84; **Event:** eventID: 2; samplingProtocol: Aerial; eventTime: Night

##### Distribution

Palearctic

#### Olios argelasius

(Walckenaer, 1805)

##### Materials

- **Type status:** Other material. **Occurrence:** individualCount: 1; sex: male; **Location:** locationID: C4; continent: Europe; country: Spain; countryCode: ES; stateProvince: Castilla-La Mancha; county: Ciudad Real; locality: La Quesera; verbatimElevation: 772.3; decimalLatitude: 39.36337; decimalLongitude: -4.41704; geodeticDatum: WGS84; **Event:** eventID: 2; samplingProtocol: Aerial; eventTime: Night
- **Type status:** Other material. **Occurrence:** individualCount: 1; sex: male; **Location:** locationID: O2; continent: Europe; country: Spain; countryCode: ES; stateProvince: Aragón; county: Huesca; locality: Rebilla; verbatimElevation: 1158.13; decimalLatitude: 42.59427; decimalLongitude: 0.1529; geodeticDatum: WGS84; **Event:** eventID: 1; samplingProtocol: Aerial; eventTime: Night

##### Distribution

Mediterranean

#### Tetragnathidae

Menge, 1866

#### Meta menardi

(Latreille, 1804)

##### Materials

- **Type status:** Other material. **Occurrence:** individualCount: 1; sex: male; **Location:** locationID: A1; continent: Europe; country: Spain; countryCode: ES; stateProvince: Catalonia; county: Lleida; locality: Sola de Boi; verbatimElevation: 1759.8; decimalLatitude: 42.54958; decimalLongitude: 0.87254; geodeticDatum: WGS84; **Event:** eventID: 2; samplingProtocol: Ground; eventTime: Night

##### Distribution

Europe to Korea(?)

#### Metellina mengei

(Blackwall, 1869)

##### Materials

- **Type status:** Other material. **Occurrence:** individualCount: 1; sex: female; **Location:** locationID: A2; continent: Europe; country: Spain; countryCode: ES; stateProvince: Catalonia; county: Lleida; locality: Sola de Boi; verbatimElevation: 1738.7; decimalLatitude: 42.54913; decimalLongitude: 0.87137; geodeticDatum: WGS84; **Event:** eventID: 1; samplingProtocol: Sweeping; eventTime: Day
- **Type status:** Other material. **Occurrence:** individualCount: 1; sex: male; **Location:** locationID: P2; continent: Europe; country: Spain; countryCode: ES; stateProvince: Castilla y León; county: León; locality: Joyoguelas; verbatimElevation: 763.98; decimalLatitude: 43.17771; decimalLongitude: -4.90579; geodeticDatum: WGS84; **Event:** eventID: 1; samplingProtocol: Sweeping; eventTime: Night

##### Distribution

Europe to Caucasus, Altai, Iran

#### Metellina merianae

(Scopoli, 1763)

##### Materials

- **Type status:** Other material. **Occurrence:** individualCount: 1; sex: female; **Location:** locationID: C2; continent: Europe; country: Spain; countryCode: ES; stateProvince: Castilla-La Mancha; county: Ciudad Real; locality: Valle Brezoso; verbatimElevation: 739.31; decimalLatitude: 39.35159; decimalLongitude: -4.3589; geodeticDatum: WGS84; **Event:** eventID: 4; samplingProtocol: Aerial; eventTime: Night
- **Type status:** Other material. **Occurrence:** individualCount: 2; sex: female; **Location:** locationID: O1; continent: Europe; country: Spain; countryCode: ES; stateProvince: Aragón; county: Huesca; locality: O Furno; verbatimElevation: 1396.73; decimalLatitude: 42.60677; decimalLongitude: 0.13135; geodeticDatum: WGS84; **Event:** eventID: 2; samplingProtocol: Aerial; eventTime: Night

##### Distribution

Europe, Ural, Caucasus, Turkey, Iran

#### Tetragnatha extensa

(Linnaeus, 1758)

##### Materials

- **Type status:** Other material. **Occurrence:** individualCount: 1; sex: female; **Location:** locationID: C1; continent: Europe; country: Spain; countryCode: ES; stateProvince: Castilla-La Mancha; county: Ciudad Real; locality: Valle Brezoso; verbatimElevation: 756.56; decimalLatitude: 39.35663; decimalLongitude: -4.35912; geodeticDatum: WGS84; **Event:** eventID: 2; samplingProtocol: Aerial; eventTime: Night
- **Type status:** Other material. **Occurrence:** individualCount: 1; sex: female; **Location:** locationID: C4; continent: Europe; country: Spain; countryCode: ES; stateProvince: Castilla-La Mancha; county: Ciudad Real; locality: La Quesera; verbatimElevation: 772.3; decimalLatitude: 39.36337; decimalLongitude: -4.41704; geodeticDatum: WGS84; **Event:** eventID: 1; samplingProtocol: Aerial; eventTime: Night
- **Type status:** Other material. **Occurrence:** individualCount: 1; sex: male; **Location:** locationID: C4; continent: Europe; country: Spain; countryCode: ES; stateProvince: Castilla-La Mancha; county: Ciudad Real; locality: La Quesera; verbatimElevation: 772.3; decimalLatitude: 39.36337; decimalLongitude: -4.41704; geodeticDatum: WGS84; **Event:** eventID: 1; samplingProtocol: Aerial; eventTime: Night
- **Type status:** Other material. **Occurrence:** individualCount: 1; sex: female; **Location:** locationID: C4; continent: Europe; country: Spain; countryCode: ES; stateProvince: Castilla-La Mancha; county: Ciudad Real; locality: La Quesera; verbatimElevation: 772.3; decimalLatitude: 39.36337; decimalLongitude: -4.41704; geodeticDatum: WGS84; **Event:** eventID: 3; samplingProtocol: Aerial; eventTime: Night
- **Type status:** Other material. **Occurrence:** individualCount: 1; sex: female; **Location:** locationID: C4; continent: Europe; country: Spain; countryCode: ES; stateProvince: Castilla-La Mancha; county: Ciudad Real; locality: La Quesera; verbatimElevation: 772.3; decimalLatitude: 39.36337; decimalLongitude: -4.41704; geodeticDatum: WGS84; **Event:** eventID: 1; samplingProtocol: Sweeping; eventTime: Day
- **Type status:** Other material. **Occurrence:** individualCount: 1; sex: male; **Location:** locationID: C4; continent: Europe; country: Spain; countryCode: ES; stateProvince: Castilla-La Mancha; county: Ciudad Real; locality: La Quesera; verbatimElevation: 772.3; decimalLatitude: 39.36337; decimalLongitude: -4.41704; geodeticDatum: WGS84; **Event:** eventID: 1; samplingProtocol: Sweeping; eventTime: Day

##### Distribution

Holarctic, Madeira

#### Tetragnatha montana

Simon, 1874

##### Materials

- **Type status:** Other material. **Occurrence:** individualCount: 1; sex: female; **Location:** locationID: P4; continent: Europe; country: Spain; countryCode: ES; stateProvince: Castilla y León; county: León; locality: El Canto; verbatimElevation: 943.48; decimalLatitude: 43.17227; decimalLongitude: -4.90857; geodeticDatum: WGS84; **Event:** eventID: 1; samplingProtocol: Beating; eventTime: Night
- **Type status:** Other material. **Occurrence:** individualCount: 1; sex: male; **Location:** locationID: P4; continent: Europe; country: Spain; countryCode: ES; stateProvince: Castilla y León; county: León; locality: El Canto; verbatimElevation: 943.48; decimalLatitude: 43.17227; decimalLongitude: -4.90857; geodeticDatum: WGS84; **Event:** eventID: 1; samplingProtocol: Sweeping; eventTime: Night
- **Type status:** Other material. **Occurrence:** individualCount: 1; sex: female; **Location:** locationID: P4; continent: Europe; country: Spain; countryCode: ES; stateProvince: Castilla y León; county: León; locality: El Canto; verbatimElevation: 943.48; decimalLatitude: 43.17227; decimalLongitude: -4.90857; geodeticDatum: WGS84; **Event:** eventID: 2; samplingProtocol: Sweeping; eventTime: Day

##### Distribution

Palearctic

#### Tetragnatha obtusa

C. L. Koch, 1837

##### Materials

- **Type status:** Other material. **Occurrence:** individualCount: 1; sex: male; **Location:** locationID: P2; continent: Europe; country: Spain; countryCode: ES; stateProvince: Castilla y León; county: León; locality: Joyoguelas; verbatimElevation: 763.98; decimalLatitude: 43.17771; decimalLongitude: -4.90579; geodeticDatum: WGS84; **Event:** eventID: 1; samplingProtocol: Aerial; eventTime: Night
- **Type status:** Other material. **Occurrence:** individualCount: 2; sex: female; **Location:** locationID: P2; continent: Europe; country: Spain; countryCode: ES; stateProvince: Castilla y León; county: León; locality: Joyoguelas; verbatimElevation: 763.98; decimalLatitude: 43.17771; decimalLongitude: -4.90579; geodeticDatum: WGS84; **Event:** eventID: 2; samplingProtocol: Aerial; eventTime: Night
- **Type status:** Other material. **Occurrence:** individualCount: 1; sex: male; **Location:** locationID: P2; continent: Europe; country: Spain; countryCode: ES; stateProvince: Castilla y León; county: León; locality: Joyoguelas; verbatimElevation: 763.98; decimalLatitude: 43.17771; decimalLongitude: -4.90579; geodeticDatum: WGS84; **Event:** eventID: 2; samplingProtocol: Aerial; eventTime: Night
- **Type status:** Other material. **Occurrence:** individualCount: 2; sex: male; **Location:** locationID: P2; continent: Europe; country: Spain; countryCode: ES; stateProvince: Castilla y León; county: León; locality: Joyoguelas; verbatimElevation: 763.98; decimalLatitude: 43.17771; decimalLongitude: -4.90579; geodeticDatum: WGS84; **Event:** eventID: 2; samplingProtocol: Aerial; eventTime: Night
- **Type status:** Other material. **Occurrence:** individualCount: 1; sex: male; **Location:** locationID: P2; continent: Europe; country: Spain; countryCode: ES; stateProvince: Castilla y León; county: León; locality: Joyoguelas; verbatimElevation: 763.98; decimalLatitude: 43.17771; decimalLongitude: -4.90579; geodeticDatum: WGS84; **Event:** eventID: 1; samplingProtocol: Beating; eventTime: Night
- **Type status:** Other material. **Occurrence:** individualCount: 3; sex: female; **Location:** locationID: P2; continent: Europe; country: Spain; countryCode: ES; stateProvince: Castilla y León; county: León; locality: Joyoguelas; verbatimElevation: 763.98; decimalLatitude: 43.17771; decimalLongitude: -4.90579; geodeticDatum: WGS84; **Event:** eventID: 1; samplingProtocol: Beating; eventTime: Night
- **Type status:** Other material. **Occurrence:** individualCount: 1; sex: male; **Location:** locationID: P2; continent: Europe; country: Spain; countryCode: ES; stateProvince: Castilla y León; county: León; locality: Joyoguelas; verbatimElevation: 763.98; decimalLatitude: 43.17771; decimalLongitude: -4.90579; geodeticDatum: WGS84; **Event:** eventID: 1; samplingProtocol: Beating; eventTime: Night
- **Type status:** Other material. **Occurrence:** individualCount: 1; sex: female; **Location:** locationID: P2; continent: Europe; country: Spain; countryCode: ES; stateProvince: Castilla y León; county: León; locality: Joyoguelas; verbatimElevation: 763.98; decimalLatitude: 43.17771; decimalLongitude: -4.90579; geodeticDatum: WGS84; **Event:** eventID: 2; samplingProtocol: Beating; eventTime: Day
- **Type status:** Other material. **Occurrence:** individualCount: 1; sex: female; **Location:** locationID: P2; continent: Europe; country: Spain; countryCode: ES; stateProvince: Castilla y León; county: León; locality: Joyoguelas; verbatimElevation: 763.98; decimalLatitude: 43.17771; decimalLongitude: -4.90579; geodeticDatum: WGS84; **Event:** eventID: 1; samplingProtocol: Sweeping; eventTime: Day
- **Type status:** Other material. **Occurrence:** individualCount: 1; sex: male; **Location:** locationID: P2; continent: Europe; country: Spain; countryCode: ES; stateProvince: Castilla y León; county: León; locality: Joyoguelas; verbatimElevation: 763.98; decimalLatitude: 43.17771; decimalLongitude: -4.90579; geodeticDatum: WGS84; **Event:** eventID: 1; samplingProtocol: Sweeping; eventTime: Night
- **Type status:** Other material. **Occurrence:** individualCount: 2; sex: female; **Location:** locationID: P4; continent: Europe; country: Spain; countryCode: ES; stateProvince: Castilla y León; county: León; locality: El Canto; verbatimElevation: 943.48; decimalLatitude: 43.17227; decimalLongitude: -4.90857; geodeticDatum: WGS84; **Event:** eventID: 1; samplingProtocol: Aerial; eventTime: Night
- **Type status:** Other material. **Occurrence:** individualCount: 1; sex: female; **Location:** locationID: P4; continent: Europe; country: Spain; countryCode: ES; stateProvince: Castilla y León; county: León; locality: El Canto; verbatimElevation: 943.48; decimalLatitude: 43.17227; decimalLongitude: -4.90857; geodeticDatum: WGS84; **Event:** eventID: 2; samplingProtocol: Aerial; eventTime: Night
- **Type status:** Other material. **Occurrence:** individualCount: 2; sex: female; **Location:** locationID: P4; continent: Europe; country: Spain; countryCode: ES; stateProvince: Castilla y León; county: León; locality: El Canto; verbatimElevation: 943.48; decimalLatitude: 43.17227; decimalLongitude: -4.90857; geodeticDatum: WGS84; **Event:** eventID: 1; samplingProtocol: Beating; eventTime: Day
- **Type status:** Other material. **Occurrence:** individualCount: 1; sex: female; **Location:** locationID: P4; continent: Europe; country: Spain; countryCode: ES; stateProvince: Castilla y León; county: León; locality: El Canto; verbatimElevation: 943.48; decimalLatitude: 43.17227; decimalLongitude: -4.90857; geodeticDatum: WGS84; **Event:** eventID: 2; samplingProtocol: Beating; eventTime: Day
- **Type status:** Other material. **Occurrence:** individualCount: 2; sex: female; **Location:** locationID: P4; continent: Europe; country: Spain; countryCode: ES; stateProvince: Castilla y León; county: León; locality: El Canto; verbatimElevation: 943.48; decimalLatitude: 43.17227; decimalLongitude: -4.90857; geodeticDatum: WGS84; **Event:** eventID: 1; samplingProtocol: Beating; eventTime: Night
- **Type status:** Other material. **Occurrence:** individualCount: 1; sex: male; **Location:** locationID: P4; continent: Europe; country: Spain; countryCode: ES; stateProvince: Castilla y León; county: León; locality: El Canto; verbatimElevation: 943.48; decimalLatitude: 43.17227; decimalLongitude: -4.90857; geodeticDatum: WGS84; **Event:** eventID: 1; samplingProtocol: Beating; eventTime: Night

##### Distribution

Palearctic

#### Tetragnatha pinicola

L. Koch, 1870

##### Materials

- **Type status:** Other material. **Occurrence:** individualCount: 1; sex: female; **Location:** locationID: P3; continent: Europe; country: Spain; countryCode: ES; stateProvince: Castilla y León; county: León; locality: Las Arroyas; verbatimElevation: 1097.1; decimalLatitude: 43.14351; decimalLongitude: -4.94878; geodeticDatum: WGS84; **Event:** eventID: 1; samplingProtocol: Aerial; eventTime: Night

##### Distribution

Palearctic

#### Theridiidae

Sundevall, 1833

#### Anelosimus vittatus

(C. L. Koch, 1836)

##### Materials

- **Type status:** Other material. **Occurrence:** individualCount: 1; sex: female; **Location:** locationID: P1; continent: Europe; country: Spain; countryCode: ES; stateProvince: Castilla y León; county: León; locality: Monte Robledo; verbatimElevation: 1071.58; decimalLatitude: 43.1445; decimalLongitude: -4.92675; geodeticDatum: WGS84; **Event:** eventID: 2; samplingProtocol: Aerial; eventTime: Night
- **Type status:** Other material. **Occurrence:** individualCount: 1; sex: male; **Location:** locationID: P1; continent: Europe; country: Spain; countryCode: ES; stateProvince: Castilla y León; county: León; locality: Monte Robledo; verbatimElevation: 1071.58; decimalLatitude: 43.1445; decimalLongitude: -4.92675; geodeticDatum: WGS84; **Event:** eventID: 1; samplingProtocol: Beating; eventTime: Day
- **Type status:** Other material. **Occurrence:** individualCount: 1; sex: female; **Location:** locationID: P1; continent: Europe; country: Spain; countryCode: ES; stateProvince: Castilla y León; county: León; locality: Monte Robledo; verbatimElevation: 1071.58; decimalLatitude: 43.1445; decimalLongitude: -4.92675; geodeticDatum: WGS84; **Event:** eventID: 2; samplingProtocol: Beating; eventTime: Day
- **Type status:** Other material. **Occurrence:** individualCount: 1; sex: female; **Location:** locationID: P1; continent: Europe; country: Spain; countryCode: ES; stateProvince: Castilla y León; county: León; locality: Monte Robledo; verbatimElevation: 1071.58; decimalLatitude: 43.1445; decimalLongitude: -4.92675; geodeticDatum: WGS84; **Event:** eventID: 1; samplingProtocol: Ground; eventTime: Day
- **Type status:** Other material. **Occurrence:** individualCount: 1; sex: male; **Location:** locationID: P1; continent: Europe; country: Spain; countryCode: ES; stateProvince: Castilla y León; county: León; locality: Monte Robledo; verbatimElevation: 1071.58; decimalLatitude: 43.1445; decimalLongitude: -4.92675; geodeticDatum: WGS84; **Event:** eventID: 1; samplingProtocol: Sweeping; eventTime: Day
- **Type status:** Other material. **Occurrence:** individualCount: 1; sex: female; **Location:** locationID: P1; continent: Europe; country: Spain; countryCode: ES; stateProvince: Castilla y León; county: León; locality: Monte Robledo; verbatimElevation: 1071.58; decimalLatitude: 43.1445; decimalLongitude: -4.92675; geodeticDatum: WGS84; **Event:** eventID: 2; samplingProtocol: Sweeping; eventTime: Day
- **Type status:** Other material. **Occurrence:** individualCount: 1; sex: male; **Location:** locationID: P1; continent: Europe; country: Spain; countryCode: ES; stateProvince: Castilla y León; county: León; locality: Monte Robledo; verbatimElevation: 1071.58; decimalLatitude: 43.1445; decimalLongitude: -4.92675; geodeticDatum: WGS84; **Event:** eventID: 2; samplingProtocol: Sweeping; eventTime: Day
- **Type status:** Other material. **Occurrence:** individualCount: 2; sex: male; **Location:** locationID: P1; continent: Europe; country: Spain; countryCode: ES; stateProvince: Castilla y León; county: León; locality: Monte Robledo; verbatimElevation: 1071.58; decimalLatitude: 43.1445; decimalLongitude: -4.92675; geodeticDatum: WGS84; **Event:** eventID: 1; samplingProtocol: Sweeping; eventTime: Night
- **Type status:** Other material. **Occurrence:** individualCount: 2; sex: female; **Location:** locationID: P2; continent: Europe; country: Spain; countryCode: ES; stateProvince: Castilla y León; county: León; locality: Joyoguelas; verbatimElevation: 763.98; decimalLatitude: 43.17771; decimalLongitude: -4.90579; geodeticDatum: WGS84; **Event:** eventID: 1; samplingProtocol: Aerial; eventTime: Night
- **Type status:** Other material. **Occurrence:** individualCount: 1; sex: female; **Location:** locationID: P2; continent: Europe; country: Spain; countryCode: ES; stateProvince: Castilla y León; county: León; locality: Joyoguelas; verbatimElevation: 763.98; decimalLatitude: 43.17771; decimalLongitude: -4.90579; geodeticDatum: WGS84; **Event:** eventID: 2; samplingProtocol: Aerial; eventTime: Night
- **Type status:** Other material. **Occurrence:** individualCount: 1; sex: female; **Location:** locationID: P2; continent: Europe; country: Spain; countryCode: ES; stateProvince: Castilla y León; county: León; locality: Joyoguelas; verbatimElevation: 763.98; decimalLatitude: 43.17771; decimalLongitude: -4.90579; geodeticDatum: WGS84; **Event:** eventID: 2; samplingProtocol: Aerial; eventTime: Night
- **Type status:** Other material. **Occurrence:** individualCount: 1; sex: male; **Location:** locationID: P2; continent: Europe; country: Spain; countryCode: ES; stateProvince: Castilla y León; county: León; locality: Joyoguelas; verbatimElevation: 763.98; decimalLatitude: 43.17771; decimalLongitude: -4.90579; geodeticDatum: WGS84; **Event:** eventID: 1; samplingProtocol: Beating; eventTime: Day
- **Type status:** Other material. **Occurrence:** individualCount: 1; sex: female; **Location:** locationID: P2; continent: Europe; country: Spain; countryCode: ES; stateProvince: Castilla y León; county: León; locality: Joyoguelas; verbatimElevation: 763.98; decimalLatitude: 43.17771; decimalLongitude: -4.90579; geodeticDatum: WGS84; **Event:** eventID: 2; samplingProtocol: Beating; eventTime: Day
- **Type status:** Other material. **Occurrence:** individualCount: 2; sex: male; **Location:** locationID: P2; continent: Europe; country: Spain; countryCode: ES; stateProvince: Castilla y León; county: León; locality: Joyoguelas; verbatimElevation: 763.98; decimalLatitude: 43.17771; decimalLongitude: -4.90579; geodeticDatum: WGS84; **Event:** eventID: 1; samplingProtocol: Beating; eventTime: Night
- **Type status:** Other material. **Occurrence:** individualCount: 1; sex: female; **Location:** locationID: P2; continent: Europe; country: Spain; countryCode: ES; stateProvince: Castilla y León; county: León; locality: Joyoguelas; verbatimElevation: 763.98; decimalLatitude: 43.17771; decimalLongitude: -4.90579; geodeticDatum: WGS84; **Event:** eventID: 1; samplingProtocol: Beating; eventTime: Night
- **Type status:** Other material. **Occurrence:** individualCount: 1; sex: female; **Location:** locationID: P4; continent: Europe; country: Spain; countryCode: ES; stateProvince: Castilla y León; county: León; locality: El Canto; verbatimElevation: 943.48; decimalLatitude: 43.17227; decimalLongitude: -4.90857; geodeticDatum: WGS84; **Event:** eventID: 2; samplingProtocol: Aerial; eventTime: Night
- **Type status:** Other material. **Occurrence:** individualCount: 7; sex: female; **Location:** locationID: P4; continent: Europe; country: Spain; countryCode: ES; stateProvince: Castilla y León; county: León; locality: El Canto; verbatimElevation: 943.48; decimalLatitude: 43.17227; decimalLongitude: -4.90857; geodeticDatum: WGS84; **Event:** eventID: 2; samplingProtocol: Beating; eventTime: Day
- **Type status:** Other material. **Occurrence:** individualCount: 2; sex: male; **Location:** locationID: P4; continent: Europe; country: Spain; countryCode: ES; stateProvince: Castilla y León; county: León; locality: El Canto; verbatimElevation: 943.48; decimalLatitude: 43.17227; decimalLongitude: -4.90857; geodeticDatum: WGS84; **Event:** eventID: 2; samplingProtocol: Beating; eventTime: Day
- **Type status:** Other material. **Occurrence:** individualCount: 3; sex: female; **Location:** locationID: P4; continent: Europe; country: Spain; countryCode: ES; stateProvince: Castilla y León; county: León; locality: El Canto; verbatimElevation: 943.48; decimalLatitude: 43.17227; decimalLongitude: -4.90857; geodeticDatum: WGS84; **Event:** eventID: 1; samplingProtocol: Beating; eventTime: Night
- **Type status:** Other material. **Occurrence:** individualCount: 1; sex: male; **Location:** locationID: P4; continent: Europe; country: Spain; countryCode: ES; stateProvince: Castilla y León; county: León; locality: El Canto; verbatimElevation: 943.48; decimalLatitude: 43.17227; decimalLongitude: -4.90857; geodeticDatum: WGS84; **Event:** eventID: 1; samplingProtocol: Sweeping; eventTime: Day

##### Distribution

Palearctic

#### Asagena phalerata

(Panzer, 1801)

##### Materials

- **Type status:** Other material. **Occurrence:** individualCount: 1; sex: male; **Location:** locationID: S2; continent: Europe; country: Spain; countryCode: ES; stateProvince: Andalucía; county: Granada; locality: Camarate; verbatimElevation: 1713.96; decimalLatitude: 37.18377; decimalLongitude: -3.26282; geodeticDatum: WGS84; **Event:** eventID: E; samplingProtocol: Pitfall

##### Distribution

Palearctic

#### Crustulina guttata

(Wider, 1834)

##### Materials

- **Type status:** Other material. **Occurrence:** individualCount: 1; sex: female; **Location:** locationID: P2; continent: Europe; country: Spain; countryCode: ES; stateProvince: Castilla y León; county: León; locality: Joyoguelas; verbatimElevation: 763.98; decimalLatitude: 43.17771; decimalLongitude: -4.90579; geodeticDatum: WGS84; **Event:** eventID: 1; samplingProtocol: Beating; eventTime: Night
- **Type status:** Other material. **Occurrence:** individualCount: 1; sex: male; **Location:** locationID: P2; continent: Europe; country: Spain; countryCode: ES; stateProvince: Castilla y León; county: León; locality: Joyoguelas; verbatimElevation: 763.98; decimalLatitude: 43.17771; decimalLongitude: -4.90579; geodeticDatum: WGS84; **Event:** eventID: 1; samplingProtocol: Sweeping; eventTime: Night
- **Type status:** Other material. **Occurrence:** individualCount: 1; sex: male; **Location:** locationID: P2; continent: Europe; country: Spain; countryCode: ES; stateProvince: Castilla y León; county: León; locality: Joyoguelas; verbatimElevation: 763.98; decimalLatitude: 43.17771; decimalLongitude: -4.90579; geodeticDatum: WGS84; **Event:** eventID: 1; samplingProtocol: Sweeping; eventTime: Night

##### Distribution

Palearctic

#### Dipoena erythropus

(Simon, 1881)

##### Materials

- **Type status:** Other material. **Occurrence:** individualCount: 1; sex: male; **Location:** locationID: O2; continent: Europe; country: Spain; countryCode: ES; stateProvince: Aragón; county: Huesca; locality: Rebilla; verbatimElevation: 1158.13; decimalLatitude: 42.59427; decimalLongitude: 0.1529; geodeticDatum: WGS84; **Event:** eventID: 1; samplingProtocol: Aerial; eventTime: Night
- **Type status:** Other material. **Occurrence:** individualCount: 1; sex: female; **Location:** locationID: O2; continent: Europe; country: Spain; countryCode: ES; stateProvince: Aragón; county: Huesca; locality: Rebilla; verbatimElevation: 1158.13; decimalLatitude: 42.59427; decimalLongitude: 0.1529; geodeticDatum: WGS84; **Event:** eventID: 1; samplingProtocol: Beating; eventTime: Day
- **Type status:** Other material. **Occurrence:** individualCount: 1; sex: male; **Location:** locationID: O2; continent: Europe; country: Spain; countryCode: ES; stateProvince: Aragón; county: Huesca; locality: Rebilla; verbatimElevation: 1158.13; decimalLatitude: 42.59427; decimalLongitude: 0.1529; geodeticDatum: WGS84; **Event:** eventID: 1; samplingProtocol: Sweeping; eventTime: Day
- **Type status:** Other material. **Occurrence:** individualCount: 2; sex: male; **Location:** locationID: O2; continent: Europe; country: Spain; countryCode: ES; stateProvince: Aragón; county: Huesca; locality: Rebilla; verbatimElevation: 1158.13; decimalLatitude: 42.59427; decimalLongitude: 0.1529; geodeticDatum: WGS84; **Event:** eventID: 1; samplingProtocol: Sweeping; eventTime: Night

##### Distribution

Europe

#### Dipoena melanogaster

(C. L. Koch, 1837)

##### Materials

- **Type status:** Other material. **Occurrence:** individualCount: 2; sex: female; **Location:** locationID: A1; continent: Europe; country: Spain; countryCode: ES; stateProvince: Catalonia; county: Lleida; locality: Sola de Boi; verbatimElevation: 1759.8; decimalLatitude: 42.54958; decimalLongitude: 0.87254; geodeticDatum: WGS84; **Event:** eventID: 1; samplingProtocol: Aerial; eventTime: Night
- **Type status:** Other material. **Occurrence:** individualCount: 1; sex: male; **Location:** locationID: A1; continent: Europe; country: Spain; countryCode: ES; stateProvince: Catalonia; county: Lleida; locality: Sola de Boi; verbatimElevation: 1759.8; decimalLatitude: 42.54958; decimalLongitude: 0.87254; geodeticDatum: WGS84; **Event:** eventID: 1; samplingProtocol: Aerial; eventTime: Night
- **Type status:** Other material. **Occurrence:** individualCount: 2; sex: female; **Location:** locationID: A1; continent: Europe; country: Spain; countryCode: ES; stateProvince: Catalonia; county: Lleida; locality: Sola de Boi; verbatimElevation: 1759.8; decimalLatitude: 42.54958; decimalLongitude: 0.87254; geodeticDatum: WGS84; **Event:** eventID: 1; samplingProtocol: Aerial; eventTime: Night
- **Type status:** Other material. **Occurrence:** individualCount: 1; sex: male; **Location:** locationID: A1; continent: Europe; country: Spain; countryCode: ES; stateProvince: Catalonia; county: Lleida; locality: Sola de Boi; verbatimElevation: 1759.8; decimalLatitude: 42.54958; decimalLongitude: 0.87254; geodeticDatum: WGS84; **Event:** eventID: 1; samplingProtocol: Aerial; eventTime: Night
- **Type status:** Other material. **Occurrence:** individualCount: 2; sex: female; **Location:** locationID: A1; continent: Europe; country: Spain; countryCode: ES; stateProvince: Catalonia; county: Lleida; locality: Sola de Boi; verbatimElevation: 1759.8; decimalLatitude: 42.54958; decimalLongitude: 0.87254; geodeticDatum: WGS84; **Event:** eventID: 2; samplingProtocol: Aerial; eventTime: Night
- **Type status:** Other material. **Occurrence:** individualCount: 1; sex: male; **Location:** locationID: A1; continent: Europe; country: Spain; countryCode: ES; stateProvince: Catalonia; county: Lleida; locality: Sola de Boi; verbatimElevation: 1759.8; decimalLatitude: 42.54958; decimalLongitude: 0.87254; geodeticDatum: WGS84; **Event:** eventID: 2; samplingProtocol: Aerial; eventTime: Night
- **Type status:** Other material. **Occurrence:** individualCount: 1; sex: male; **Location:** locationID: A1; continent: Europe; country: Spain; countryCode: ES; stateProvince: Catalonia; county: Lleida; locality: Sola de Boi; verbatimElevation: 1759.8; decimalLatitude: 42.54958; decimalLongitude: 0.87254; geodeticDatum: WGS84; **Event:** eventID: 2; samplingProtocol: Aerial; eventTime: Night
- **Type status:** Other material. **Occurrence:** individualCount: 2; sex: female; **Location:** locationID: A1; continent: Europe; country: Spain; countryCode: ES; stateProvince: Catalonia; county: Lleida; locality: Sola de Boi; verbatimElevation: 1759.8; decimalLatitude: 42.54958; decimalLongitude: 0.87254; geodeticDatum: WGS84; **Event:** eventID: 1; samplingProtocol: Beating; eventTime: Day
- **Type status:** Other material. **Occurrence:** individualCount: 1; sex: male; **Location:** locationID: A1; continent: Europe; country: Spain; countryCode: ES; stateProvince: Catalonia; county: Lleida; locality: Sola de Boi; verbatimElevation: 1759.8; decimalLatitude: 42.54958; decimalLongitude: 0.87254; geodeticDatum: WGS84; **Event:** eventID: 1; samplingProtocol: Beating; eventTime: Day
- **Type status:** Other material. **Occurrence:** individualCount: 3; sex: female; **Location:** locationID: A1; continent: Europe; country: Spain; countryCode: ES; stateProvince: Catalonia; county: Lleida; locality: Sola de Boi; verbatimElevation: 1759.8; decimalLatitude: 42.54958; decimalLongitude: 0.87254; geodeticDatum: WGS84; **Event:** eventID: 2; samplingProtocol: Beating; eventTime: Day
- **Type status:** Other material. **Occurrence:** individualCount: 2; sex: female; **Location:** locationID: A1; continent: Europe; country: Spain; countryCode: ES; stateProvince: Catalonia; county: Lleida; locality: Sola de Boi; verbatimElevation: 1759.8; decimalLatitude: 42.54958; decimalLongitude: 0.87254; geodeticDatum: WGS84; **Event:** eventID: 1; samplingProtocol: Beating; eventTime: Night
- **Type status:** Other material. **Occurrence:** individualCount: 1; sex: male; **Location:** locationID: A1; continent: Europe; country: Spain; countryCode: ES; stateProvince: Catalonia; county: Lleida; locality: Sola de Boi; verbatimElevation: 1759.8; decimalLatitude: 42.54958; decimalLongitude: 0.87254; geodeticDatum: WGS84; **Event:** eventID: 1; samplingProtocol: Beating; eventTime: Night
- **Type status:** Other material. **Occurrence:** individualCount: 1; sex: male; **Location:** locationID: A1; continent: Europe; country: Spain; countryCode: ES; stateProvince: Catalonia; county: Lleida; locality: Sola de Boi; verbatimElevation: 1759.8; decimalLatitude: 42.54958; decimalLongitude: 0.87254; geodeticDatum: WGS84; **Event:** eventID: 1; samplingProtocol: Beating; eventTime: Night
- **Type status:** Other material. **Occurrence:** individualCount: 1; sex: female; **Location:** locationID: A1; continent: Europe; country: Spain; countryCode: ES; stateProvince: Catalonia; county: Lleida; locality: Sola de Boi; verbatimElevation: 1759.8; decimalLatitude: 42.54958; decimalLongitude: 0.87254; geodeticDatum: WGS84; **Event:** eventID: 2; samplingProtocol: Ground; eventTime: Night
- **Type status:** Other material. **Occurrence:** individualCount: 1; sex: female; **Location:** locationID: A1; continent: Europe; country: Spain; countryCode: ES; stateProvince: Catalonia; county: Lleida; locality: Sola de Boi; verbatimElevation: 1759.8; decimalLatitude: 42.54958; decimalLongitude: 0.87254; geodeticDatum: WGS84; **Event:** eventID: 1; samplingProtocol: Sweeping; eventTime: Night
- **Type status:** Other material. **Occurrence:** individualCount: 1; sex: female; **Location:** locationID: A2; continent: Europe; country: Spain; countryCode: ES; stateProvince: Catalonia; county: Lleida; locality: Sola de Boi; verbatimElevation: 1738.7; decimalLatitude: 42.54913; decimalLongitude: 0.87137; geodeticDatum: WGS84; **Event:** eventID: 1; samplingProtocol: Aerial; eventTime: Night
- **Type status:** Other material. **Occurrence:** individualCount: 1; sex: male; **Location:** locationID: A2; continent: Europe; country: Spain; countryCode: ES; stateProvince: Catalonia; county: Lleida; locality: Sola de Boi; verbatimElevation: 1738.7; decimalLatitude: 42.54913; decimalLongitude: 0.87137; geodeticDatum: WGS84; **Event:** eventID: 2; samplingProtocol: Aerial; eventTime: Night
- **Type status:** Other material. **Occurrence:** individualCount: 1; sex: male; **Location:** locationID: A2; continent: Europe; country: Spain; countryCode: ES; stateProvince: Catalonia; county: Lleida; locality: Sola de Boi; verbatimElevation: 1738.7; decimalLatitude: 42.54913; decimalLongitude: 0.87137; geodeticDatum: WGS84; **Event:** eventID: 2; samplingProtocol: Aerial; eventTime: Night
- **Type status:** Other material. **Occurrence:** individualCount: 1; sex: female; **Location:** locationID: A2; continent: Europe; country: Spain; countryCode: ES; stateProvince: Catalonia; county: Lleida; locality: Sola de Boi; verbatimElevation: 1738.7; decimalLatitude: 42.54913; decimalLongitude: 0.87137; geodeticDatum: WGS84; **Event:** eventID: 1; samplingProtocol: Beating; eventTime: Day
- **Type status:** Other material. **Occurrence:** individualCount: 1; sex: female; **Location:** locationID: A2; continent: Europe; country: Spain; countryCode: ES; stateProvince: Catalonia; county: Lleida; locality: Sola de Boi; verbatimElevation: 1738.7; decimalLatitude: 42.54913; decimalLongitude: 0.87137; geodeticDatum: WGS84; **Event:** eventID: 2; samplingProtocol: Beating; eventTime: Day
- **Type status:** Other material. **Occurrence:** individualCount: 1; sex: male; **Location:** locationID: A2; continent: Europe; country: Spain; countryCode: ES; stateProvince: Catalonia; county: Lleida; locality: Sola de Boi; verbatimElevation: 1738.7; decimalLatitude: 42.54913; decimalLongitude: 0.87137; geodeticDatum: WGS84; **Event:** eventID: 2; samplingProtocol: Beating; eventTime: Day
- **Type status:** Other material. **Occurrence:** individualCount: 1; sex: female; **Location:** locationID: A2; continent: Europe; country: Spain; countryCode: ES; stateProvince: Catalonia; county: Lleida; locality: Sola de Boi; verbatimElevation: 1738.7; decimalLatitude: 42.54913; decimalLongitude: 0.87137; geodeticDatum: WGS84; **Event:** eventID: 1; samplingProtocol: Beating; eventTime: Night
- **Type status:** Other material. **Occurrence:** individualCount: 1; sex: female; **Location:** locationID: A2; continent: Europe; country: Spain; countryCode: ES; stateProvince: Catalonia; county: Lleida; locality: Sola de Boi; verbatimElevation: 1738.7; decimalLatitude: 42.54913; decimalLongitude: 0.87137; geodeticDatum: WGS84; **Event:** eventID: 1; samplingProtocol: Sweeping; eventTime: Night
- **Type status:** Other material. **Occurrence:** individualCount: 8; sex: female; **Location:** locationID: C1; continent: Europe; country: Spain; countryCode: ES; stateProvince: Castilla-La Mancha; county: Ciudad Real; locality: Valle Brezoso; verbatimElevation: 756.56; decimalLatitude: 39.35663; decimalLongitude: -4.35912; geodeticDatum: WGS84; **Event:** eventID: 1; samplingProtocol: Aerial; eventTime: Night
- **Type status:** Other material. **Occurrence:** individualCount: 1; sex: female; **Location:** locationID: C1; continent: Europe; country: Spain; countryCode: ES; stateProvince: Castilla-La Mancha; county: Ciudad Real; locality: Valle Brezoso; verbatimElevation: 756.56; decimalLatitude: 39.35663; decimalLongitude: -4.35912; geodeticDatum: WGS84; **Event:** eventID: 2; samplingProtocol: Aerial; eventTime: Night
- **Type status:** Other material. **Occurrence:** individualCount: 1; sex: male; **Location:** locationID: C1; continent: Europe; country: Spain; countryCode: ES; stateProvince: Castilla-La Mancha; county: Ciudad Real; locality: Valle Brezoso; verbatimElevation: 756.56; decimalLatitude: 39.35663; decimalLongitude: -4.35912; geodeticDatum: WGS84; **Event:** eventID: 2; samplingProtocol: Aerial; eventTime: Night
- **Type status:** Other material. **Occurrence:** individualCount: 3; sex: female; **Location:** locationID: C1; continent: Europe; country: Spain; countryCode: ES; stateProvince: Castilla-La Mancha; county: Ciudad Real; locality: Valle Brezoso; verbatimElevation: 756.56; decimalLatitude: 39.35663; decimalLongitude: -4.35912; geodeticDatum: WGS84; **Event:** eventID: 3; samplingProtocol: Aerial; eventTime: Night
- **Type status:** Other material. **Occurrence:** individualCount: 14; sex: female; **Location:** locationID: C1; continent: Europe; country: Spain; countryCode: ES; stateProvince: Castilla-La Mancha; county: Ciudad Real; locality: Valle Brezoso; verbatimElevation: 756.56; decimalLatitude: 39.35663; decimalLongitude: -4.35912; geodeticDatum: WGS84; **Event:** eventID: 4; samplingProtocol: Aerial; eventTime: Night
- **Type status:** Other material. **Occurrence:** individualCount: 2; sex: male; **Location:** locationID: C1; continent: Europe; country: Spain; countryCode: ES; stateProvince: Castilla-La Mancha; county: Ciudad Real; locality: Valle Brezoso; verbatimElevation: 756.56; decimalLatitude: 39.35663; decimalLongitude: -4.35912; geodeticDatum: WGS84; **Event:** eventID: 4; samplingProtocol: Aerial; eventTime: Night
- **Type status:** Other material. **Occurrence:** individualCount: 1; sex: female; **Location:** locationID: C1; continent: Europe; country: Spain; countryCode: ES; stateProvince: Castilla-La Mancha; county: Ciudad Real; locality: Valle Brezoso; verbatimElevation: 756.56; decimalLatitude: 39.35663; decimalLongitude: -4.35912; geodeticDatum: WGS84; **Event:** eventID: 1; samplingProtocol: Beating; eventTime: Day
- **Type status:** Other material. **Occurrence:** individualCount: 4; sex: female; **Location:** locationID: C1; continent: Europe; country: Spain; countryCode: ES; stateProvince: Castilla-La Mancha; county: Ciudad Real; locality: Valle Brezoso; verbatimElevation: 756.56; decimalLatitude: 39.35663; decimalLongitude: -4.35912; geodeticDatum: WGS84; **Event:** eventID: 2; samplingProtocol: Beating; eventTime: Day
- **Type status:** Other material. **Occurrence:** individualCount: 1; sex: female; **Location:** locationID: C1; continent: Europe; country: Spain; countryCode: ES; stateProvince: Castilla-La Mancha; county: Ciudad Real; locality: Valle Brezoso; verbatimElevation: 756.56; decimalLatitude: 39.35663; decimalLongitude: -4.35912; geodeticDatum: WGS84; **Event:** eventID: 1; samplingProtocol: Beating; eventTime: Night
- **Type status:** Other material. **Occurrence:** individualCount: 4; sex: female; **Location:** locationID: C1; continent: Europe; country: Spain; countryCode: ES; stateProvince: Castilla-La Mancha; county: Ciudad Real; locality: Valle Brezoso; verbatimElevation: 756.56; decimalLatitude: 39.35663; decimalLongitude: -4.35912; geodeticDatum: WGS84; **Event:** eventID: 2; samplingProtocol: Beating; eventTime: Night
- **Type status:** Other material. **Occurrence:** individualCount: 1; sex: female; **Location:** locationID: C1; continent: Europe; country: Spain; countryCode: ES; stateProvince: Castilla-La Mancha; county: Ciudad Real; locality: Valle Brezoso; verbatimElevation: 756.56; decimalLatitude: 39.35663; decimalLongitude: -4.35912; geodeticDatum: WGS84; **Event:** eventID: 1; samplingProtocol: Sweeping; eventTime: Day
- **Type status:** Other material. **Occurrence:** individualCount: 1; sex: female; **Location:** locationID: C1; continent: Europe; country: Spain; countryCode: ES; stateProvince: Castilla-La Mancha; county: Ciudad Real; locality: Valle Brezoso; verbatimElevation: 756.56; decimalLatitude: 39.35663; decimalLongitude: -4.35912; geodeticDatum: WGS84; **Event:** eventID: 2; samplingProtocol: Sweeping; eventTime: Day
- **Type status:** Other material. **Occurrence:** individualCount: 2; sex: female; **Location:** locationID: C1; continent: Europe; country: Spain; countryCode: ES; stateProvince: Castilla-La Mancha; county: Ciudad Real; locality: Valle Brezoso; verbatimElevation: 756.56; decimalLatitude: 39.35663; decimalLongitude: -4.35912; geodeticDatum: WGS84; **Event:** eventID: 1; samplingProtocol: Sweeping; eventTime: Night
- **Type status:** Other material. **Occurrence:** individualCount: 6; sex: female; **Location:** locationID: C1; continent: Europe; country: Spain; countryCode: ES; stateProvince: Castilla-La Mancha; county: Ciudad Real; locality: Valle Brezoso; verbatimElevation: 756.56; decimalLatitude: 39.35663; decimalLongitude: -4.35912; geodeticDatum: WGS84; **Event:** eventID: 2; samplingProtocol: Sweeping; eventTime: Night
- **Type status:** Other material. **Occurrence:** individualCount: 15; sex: female; **Location:** locationID: C2; continent: Europe; country: Spain; countryCode: ES; stateProvince: Castilla-La Mancha; county: Ciudad Real; locality: Valle Brezoso; verbatimElevation: 739.31; decimalLatitude: 39.35159; decimalLongitude: -4.3589; geodeticDatum: WGS84; **Event:** eventID: 1; samplingProtocol: Aerial; eventTime: Night
- **Type status:** Other material. **Occurrence:** individualCount: 3; sex: male; **Location:** locationID: C2; continent: Europe; country: Spain; countryCode: ES; stateProvince: Castilla-La Mancha; county: Ciudad Real; locality: Valle Brezoso; verbatimElevation: 739.31; decimalLatitude: 39.35159; decimalLongitude: -4.3589; geodeticDatum: WGS84; **Event:** eventID: 1; samplingProtocol: Aerial; eventTime: Night
- **Type status:** Other material. **Occurrence:** individualCount: 12; sex: female; **Location:** locationID: C2; continent: Europe; country: Spain; countryCode: ES; stateProvince: Castilla-La Mancha; county: Ciudad Real; locality: Valle Brezoso; verbatimElevation: 739.31; decimalLatitude: 39.35159; decimalLongitude: -4.3589; geodeticDatum: WGS84; **Event:** eventID: 2; samplingProtocol: Aerial; eventTime: Night
- **Type status:** Other material. **Occurrence:** individualCount: 1; sex: male; **Location:** locationID: C2; continent: Europe; country: Spain; countryCode: ES; stateProvince: Castilla-La Mancha; county: Ciudad Real; locality: Valle Brezoso; verbatimElevation: 739.31; decimalLatitude: 39.35159; decimalLongitude: -4.3589; geodeticDatum: WGS84; **Event:** eventID: 2; samplingProtocol: Aerial; eventTime: Night
- **Type status:** Other material. **Occurrence:** individualCount: 4; sex: female; **Location:** locationID: C2; continent: Europe; country: Spain; countryCode: ES; stateProvince: Castilla-La Mancha; county: Ciudad Real; locality: Valle Brezoso; verbatimElevation: 739.31; decimalLatitude: 39.35159; decimalLongitude: -4.3589; geodeticDatum: WGS84; **Event:** eventID: 3; samplingProtocol: Aerial; eventTime: Night
- **Type status:** Other material. **Occurrence:** individualCount: 5; sex: female; **Location:** locationID: C2; continent: Europe; country: Spain; countryCode: ES; stateProvince: Castilla-La Mancha; county: Ciudad Real; locality: Valle Brezoso; verbatimElevation: 739.31; decimalLatitude: 39.35159; decimalLongitude: -4.3589; geodeticDatum: WGS84; **Event:** eventID: 4; samplingProtocol: Aerial; eventTime: Night
- **Type status:** Other material. **Occurrence:** individualCount: 1; sex: male; **Location:** locationID: C2; continent: Europe; country: Spain; countryCode: ES; stateProvince: Castilla-La Mancha; county: Ciudad Real; locality: Valle Brezoso; verbatimElevation: 739.31; decimalLatitude: 39.35159; decimalLongitude: -4.3589; geodeticDatum: WGS84; **Event:** eventID: 4; samplingProtocol: Aerial; eventTime: Night
- **Type status:** Other material. **Occurrence:** individualCount: 3; sex: female; **Location:** locationID: C2; continent: Europe; country: Spain; countryCode: ES; stateProvince: Castilla-La Mancha; county: Ciudad Real; locality: Valle Brezoso; verbatimElevation: 739.31; decimalLatitude: 39.35159; decimalLongitude: -4.3589; geodeticDatum: WGS84; **Event:** eventID: 1; samplingProtocol: Beating; eventTime: Day
- **Type status:** Other material. **Occurrence:** individualCount: 5; sex: female; **Location:** locationID: C2; continent: Europe; country: Spain; countryCode: ES; stateProvince: Castilla-La Mancha; county: Ciudad Real; locality: Valle Brezoso; verbatimElevation: 739.31; decimalLatitude: 39.35159; decimalLongitude: -4.3589; geodeticDatum: WGS84; **Event:** eventID: 2; samplingProtocol: Beating; eventTime: Day
- **Type status:** Other material. **Occurrence:** individualCount: 4; sex: female; **Location:** locationID: C2; continent: Europe; country: Spain; countryCode: ES; stateProvince: Castilla-La Mancha; county: Ciudad Real; locality: Valle Brezoso; verbatimElevation: 739.31; decimalLatitude: 39.35159; decimalLongitude: -4.3589; geodeticDatum: WGS84; **Event:** eventID: 1; samplingProtocol: Beating; eventTime: Night
- **Type status:** Other material. **Occurrence:** individualCount: 5; sex: female; **Location:** locationID: C2; continent: Europe; country: Spain; countryCode: ES; stateProvince: Castilla-La Mancha; county: Ciudad Real; locality: Valle Brezoso; verbatimElevation: 739.31; decimalLatitude: 39.35159; decimalLongitude: -4.3589; geodeticDatum: WGS84; **Event:** eventID: 2; samplingProtocol: Beating; eventTime: Night
- **Type status:** Other material. **Occurrence:** individualCount: 1; sex: female; **Location:** locationID: C2; continent: Europe; country: Spain; countryCode: ES; stateProvince: Castilla-La Mancha; county: Ciudad Real; locality: Valle Brezoso; verbatimElevation: 739.31; decimalLatitude: 39.35159; decimalLongitude: -4.3589; geodeticDatum: WGS84; **Event:** eventID: 1; samplingProtocol: Sweeping; eventTime: Day
- **Type status:** Other material. **Occurrence:** individualCount: 1; sex: male; **Location:** locationID: C2; continent: Europe; country: Spain; countryCode: ES; stateProvince: Castilla-La Mancha; county: Ciudad Real; locality: Valle Brezoso; verbatimElevation: 739.31; decimalLatitude: 39.35159; decimalLongitude: -4.3589; geodeticDatum: WGS84; **Event:** eventID: 2; samplingProtocol: Sweeping; eventTime: Day
- **Type status:** Other material. **Occurrence:** individualCount: 1; sex: female; **Location:** locationID: C2; continent: Europe; country: Spain; countryCode: ES; stateProvince: Castilla-La Mancha; county: Ciudad Real; locality: Valle Brezoso; verbatimElevation: 739.31; decimalLatitude: 39.35159; decimalLongitude: -4.3589; geodeticDatum: WGS84; **Event:** eventID: 1; samplingProtocol: Sweeping; eventTime: Night
- **Type status:** Other material. **Occurrence:** individualCount: 4; sex: female; **Location:** locationID: C3; continent: Europe; country: Spain; countryCode: ES; stateProvince: Castilla-La Mancha; county: Ciudad Real; locality: La Quesera; verbatimElevation: 767.55; decimalLatitude: 39.36177; decimalLongitude: -4.41733; geodeticDatum: WGS84; **Event:** eventID: 1; samplingProtocol: Aerial; eventTime: Night
- **Type status:** Other material. **Occurrence:** individualCount: 3; sex: female; **Location:** locationID: C3; continent: Europe; country: Spain; countryCode: ES; stateProvince: Castilla-La Mancha; county: Ciudad Real; locality: La Quesera; verbatimElevation: 767.55; decimalLatitude: 39.36177; decimalLongitude: -4.41733; geodeticDatum: WGS84; **Event:** eventID: 3; samplingProtocol: Aerial; eventTime: Night
- **Type status:** Other material. **Occurrence:** individualCount: 2; sex: female; **Location:** locationID: C3; continent: Europe; country: Spain; countryCode: ES; stateProvince: Castilla-La Mancha; county: Ciudad Real; locality: La Quesera; verbatimElevation: 767.55; decimalLatitude: 39.36177; decimalLongitude: -4.41733; geodeticDatum: WGS84; **Event:** eventID: 4; samplingProtocol: Aerial; eventTime: Night
- **Type status:** Other material. **Occurrence:** individualCount: 1; sex: female; **Location:** locationID: C3; continent: Europe; country: Spain; countryCode: ES; stateProvince: Castilla-La Mancha; county: Ciudad Real; locality: La Quesera; verbatimElevation: 767.55; decimalLatitude: 39.36177; decimalLongitude: -4.41733; geodeticDatum: WGS84; **Event:** eventID: 2; samplingProtocol: Beating; eventTime: Night
- **Type status:** Other material. **Occurrence:** individualCount: 1; sex: female; **Location:** locationID: C3; continent: Europe; country: Spain; countryCode: ES; stateProvince: Castilla-La Mancha; county: Ciudad Real; locality: La Quesera; verbatimElevation: 767.55; decimalLatitude: 39.36177; decimalLongitude: -4.41733; geodeticDatum: WGS84; **Event:** eventID: 2; samplingProtocol: Beating; eventTime: Day
- **Type status:** Other material. **Occurrence:** individualCount: 1; sex: male; **Location:** locationID: C4; continent: Europe; country: Spain; countryCode: ES; stateProvince: Castilla-La Mancha; county: Ciudad Real; locality: La Quesera; verbatimElevation: 772.3; decimalLatitude: 39.36337; decimalLongitude: -4.41704; geodeticDatum: WGS84; **Event:** eventID: 1; samplingProtocol: Aerial; eventTime: Night
- **Type status:** Other material. **Occurrence:** individualCount: 1; sex: female; **Location:** locationID: C4; continent: Europe; country: Spain; countryCode: ES; stateProvince: Castilla-La Mancha; county: Ciudad Real; locality: La Quesera; verbatimElevation: 772.3; decimalLatitude: 39.36337; decimalLongitude: -4.41704; geodeticDatum: WGS84; **Event:** eventID: 2; samplingProtocol: Aerial; eventTime: Night
- **Type status:** Other material. **Occurrence:** individualCount: 2; sex: female; **Location:** locationID: C4; continent: Europe; country: Spain; countryCode: ES; stateProvince: Castilla-La Mancha; county: Ciudad Real; locality: La Quesera; verbatimElevation: 772.3; decimalLatitude: 39.36337; decimalLongitude: -4.41704; geodeticDatum: WGS84; **Event:** eventID: 4; samplingProtocol: Aerial; eventTime: Night
- **Type status:** Other material. **Occurrence:** individualCount: 1; sex: male; **Location:** locationID: C4; continent: Europe; country: Spain; countryCode: ES; stateProvince: Castilla-La Mancha; county: Ciudad Real; locality: La Quesera; verbatimElevation: 772.3; decimalLatitude: 39.36337; decimalLongitude: -4.41704; geodeticDatum: WGS84; **Event:** eventID: 1; samplingProtocol: Beating; eventTime: Day
- **Type status:** Other material. **Occurrence:** individualCount: 8; sex: female; **Location:** locationID: O1; continent: Europe; country: Spain; countryCode: ES; stateProvince: Aragón; county: Huesca; locality: O Furno; verbatimElevation: 1396.73; decimalLatitude: 42.60677; decimalLongitude: 0.13135; geodeticDatum: WGS84; **Event:** eventID: 1; samplingProtocol: Aerial; eventTime: Night
- **Type status:** Other material. **Occurrence:** individualCount: 4; sex: male; **Location:** locationID: O1; continent: Europe; country: Spain; countryCode: ES; stateProvince: Aragón; county: Huesca; locality: O Furno; verbatimElevation: 1396.73; decimalLatitude: 42.60677; decimalLongitude: 0.13135; geodeticDatum: WGS84; **Event:** eventID: 1; samplingProtocol: Aerial; eventTime: Night
- **Type status:** Other material. **Occurrence:** individualCount: 3; sex: female; **Location:** locationID: O1; continent: Europe; country: Spain; countryCode: ES; stateProvince: Aragón; county: Huesca; locality: O Furno; verbatimElevation: 1396.73; decimalLatitude: 42.60677; decimalLongitude: 0.13135; geodeticDatum: WGS84; **Event:** eventID: 2; samplingProtocol: Aerial; eventTime: Night
- **Type status:** Other material. **Occurrence:** individualCount: 1; sex: male; **Location:** locationID: O1; continent: Europe; country: Spain; countryCode: ES; stateProvince: Aragón; county: Huesca; locality: O Furno; verbatimElevation: 1396.73; decimalLatitude: 42.60677; decimalLongitude: 0.13135; geodeticDatum: WGS84; **Event:** eventID: 2; samplingProtocol: Aerial; eventTime: Night
- **Type status:** Other material. **Occurrence:** individualCount: 2; sex: female; **Location:** locationID: O1; continent: Europe; country: Spain; countryCode: ES; stateProvince: Aragón; county: Huesca; locality: O Furno; verbatimElevation: 1396.73; decimalLatitude: 42.60677; decimalLongitude: 0.13135; geodeticDatum: WGS84; **Event:** eventID: 2; samplingProtocol: Aerial; eventTime: Night
- **Type status:** Other material. **Occurrence:** individualCount: 2; sex: male; **Location:** locationID: O1; continent: Europe; country: Spain; countryCode: ES; stateProvince: Aragón; county: Huesca; locality: O Furno; verbatimElevation: 1396.73; decimalLatitude: 42.60677; decimalLongitude: 0.13135; geodeticDatum: WGS84; **Event:** eventID: 2; samplingProtocol: Aerial; eventTime: Night
- **Type status:** Other material. **Occurrence:** individualCount: 1; sex: male; **Location:** locationID: O1; continent: Europe; country: Spain; countryCode: ES; stateProvince: Aragón; county: Huesca; locality: O Furno; verbatimElevation: 1396.73; decimalLatitude: 42.60677; decimalLongitude: 0.13135; geodeticDatum: WGS84; **Event:** eventID: 1; samplingProtocol: Beating; eventTime: Day
- **Type status:** Other material. **Occurrence:** individualCount: 1; sex: male; **Location:** locationID: O1; continent: Europe; country: Spain; countryCode: ES; stateProvince: Aragón; county: Huesca; locality: O Furno; verbatimElevation: 1396.73; decimalLatitude: 42.60677; decimalLongitude: 0.13135; geodeticDatum: WGS84; **Event:** eventID: 2; samplingProtocol: Beating; eventTime: Day
- **Type status:** Other material. **Occurrence:** individualCount: 3; sex: female; **Location:** locationID: O1; continent: Europe; country: Spain; countryCode: ES; stateProvince: Aragón; county: Huesca; locality: O Furno; verbatimElevation: 1396.73; decimalLatitude: 42.60677; decimalLongitude: 0.13135; geodeticDatum: WGS84; **Event:** eventID: 1; samplingProtocol: Beating; eventTime: Night
- **Type status:** Other material. **Occurrence:** individualCount: 4; sex: male; **Location:** locationID: O1; continent: Europe; country: Spain; countryCode: ES; stateProvince: Aragón; county: Huesca; locality: O Furno; verbatimElevation: 1396.73; decimalLatitude: 42.60677; decimalLongitude: 0.13135; geodeticDatum: WGS84; **Event:** eventID: 1; samplingProtocol: Beating; eventTime: Night
- **Type status:** Other material. **Occurrence:** individualCount: 1; sex: male; **Location:** locationID: O1; continent: Europe; country: Spain; countryCode: ES; stateProvince: Aragón; county: Huesca; locality: O Furno; verbatimElevation: 1396.73; decimalLatitude: 42.60677; decimalLongitude: 0.13135; geodeticDatum: WGS84; **Event:** eventID: 1; samplingProtocol: Beating; eventTime: Night
- **Type status:** Other material. **Occurrence:** individualCount: 1; sex: male; **Location:** locationID: O1; continent: Europe; country: Spain; countryCode: ES; stateProvince: Aragón; county: Huesca; locality: O Furno; verbatimElevation: 1396.73; decimalLatitude: 42.60677; decimalLongitude: 0.13135; geodeticDatum: WGS84; **Event:** eventID: 1; samplingProtocol: Sweeping; eventTime: Night
- **Type status:** Other material. **Occurrence:** individualCount: 11; sex: female; **Location:** locationID: O2; continent: Europe; country: Spain; countryCode: ES; stateProvince: Aragón; county: Huesca; locality: Rebilla; verbatimElevation: 1158.13; decimalLatitude: 42.59427; decimalLongitude: 0.1529; geodeticDatum: WGS84; **Event:** eventID: 1; samplingProtocol: Aerial; eventTime: Night
- **Type status:** Other material. **Occurrence:** individualCount: 4; sex: male; **Location:** locationID: O2; continent: Europe; country: Spain; countryCode: ES; stateProvince: Aragón; county: Huesca; locality: Rebilla; verbatimElevation: 1158.13; decimalLatitude: 42.59427; decimalLongitude: 0.1529; geodeticDatum: WGS84; **Event:** eventID: 1; samplingProtocol: Aerial; eventTime: Night
- **Type status:** Other material. **Occurrence:** individualCount: 3; sex: female; **Location:** locationID: O2; continent: Europe; country: Spain; countryCode: ES; stateProvince: Aragón; county: Huesca; locality: Rebilla; verbatimElevation: 1158.13; decimalLatitude: 42.59427; decimalLongitude: 0.1529; geodeticDatum: WGS84; **Event:** eventID: 1; samplingProtocol: Aerial; eventTime: Night
- **Type status:** Other material. **Occurrence:** individualCount: 1; sex: male; **Location:** locationID: O2; continent: Europe; country: Spain; countryCode: ES; stateProvince: Aragón; county: Huesca; locality: Rebilla; verbatimElevation: 1158.13; decimalLatitude: 42.59427; decimalLongitude: 0.1529; geodeticDatum: WGS84; **Event:** eventID: 1; samplingProtocol: Aerial; eventTime: Night
- **Type status:** Other material. **Occurrence:** individualCount: 1; sex: female; **Location:** locationID: O2; continent: Europe; country: Spain; countryCode: ES; stateProvince: Aragón; county: Huesca; locality: Rebilla; verbatimElevation: 1158.13; decimalLatitude: 42.59427; decimalLongitude: 0.1529; geodeticDatum: WGS84; **Event:** eventID: 2; samplingProtocol: Aerial; eventTime: Night
- **Type status:** Other material. **Occurrence:** individualCount: 6; sex: female; **Location:** locationID: O2; continent: Europe; country: Spain; countryCode: ES; stateProvince: Aragón; county: Huesca; locality: Rebilla; verbatimElevation: 1158.13; decimalLatitude: 42.59427; decimalLongitude: 0.1529; geodeticDatum: WGS84; **Event:** eventID: 2; samplingProtocol: Aerial; eventTime: Night
- **Type status:** Other material. **Occurrence:** individualCount: 6; sex: male; **Location:** locationID: O2; continent: Europe; country: Spain; countryCode: ES; stateProvince: Aragón; county: Huesca; locality: Rebilla; verbatimElevation: 1158.13; decimalLatitude: 42.59427; decimalLongitude: 0.1529; geodeticDatum: WGS84; **Event:** eventID: 2; samplingProtocol: Aerial; eventTime: Night
- **Type status:** Other material. **Occurrence:** individualCount: 3; sex: female; **Location:** locationID: O2; continent: Europe; country: Spain; countryCode: ES; stateProvince: Aragón; county: Huesca; locality: Rebilla; verbatimElevation: 1158.13; decimalLatitude: 42.59427; decimalLongitude: 0.1529; geodeticDatum: WGS84; **Event:** eventID: 1; samplingProtocol: Beating; eventTime: Day
- **Type status:** Other material. **Occurrence:** individualCount: 2; sex: male; **Location:** locationID: O2; continent: Europe; country: Spain; countryCode: ES; stateProvince: Aragón; county: Huesca; locality: Rebilla; verbatimElevation: 1158.13; decimalLatitude: 42.59427; decimalLongitude: 0.1529; geodeticDatum: WGS84; **Event:** eventID: 1; samplingProtocol: Beating; eventTime: Day
- **Type status:** Other material. **Occurrence:** individualCount: 3; sex: female; **Location:** locationID: O2; continent: Europe; country: Spain; countryCode: ES; stateProvince: Aragón; county: Huesca; locality: Rebilla; verbatimElevation: 1158.13; decimalLatitude: 42.59427; decimalLongitude: 0.1529; geodeticDatum: WGS84; **Event:** eventID: 2; samplingProtocol: Beating; eventTime: Day
- **Type status:** Other material. **Occurrence:** individualCount: 1; sex: male; **Location:** locationID: O2; continent: Europe; country: Spain; countryCode: ES; stateProvince: Aragón; county: Huesca; locality: Rebilla; verbatimElevation: 1158.13; decimalLatitude: 42.59427; decimalLongitude: 0.1529; geodeticDatum: WGS84; **Event:** eventID: 2; samplingProtocol: Beating; eventTime: Day
- **Type status:** Other material. **Occurrence:** individualCount: 3; sex: female; **Location:** locationID: O2; continent: Europe; country: Spain; countryCode: ES; stateProvince: Aragón; county: Huesca; locality: Rebilla; verbatimElevation: 1158.13; decimalLatitude: 42.59427; decimalLongitude: 0.1529; geodeticDatum: WGS84; **Event:** eventID: 1; samplingProtocol: Beating; eventTime: Night
- **Type status:** Other material. **Occurrence:** individualCount: 2; sex: male; **Location:** locationID: O2; continent: Europe; country: Spain; countryCode: ES; stateProvince: Aragón; county: Huesca; locality: Rebilla; verbatimElevation: 1158.13; decimalLatitude: 42.59427; decimalLongitude: 0.1529; geodeticDatum: WGS84; **Event:** eventID: 1; samplingProtocol: Beating; eventTime: Night
- **Type status:** Other material. **Occurrence:** individualCount: 1; sex: female; **Location:** locationID: O2; continent: Europe; country: Spain; countryCode: ES; stateProvince: Aragón; county: Huesca; locality: Rebilla; verbatimElevation: 1158.13; decimalLatitude: 42.59427; decimalLongitude: 0.1529; geodeticDatum: WGS84; **Event:** eventID: 1; samplingProtocol: Sweeping; eventTime: Night
- **Type status:** Other material. **Occurrence:** individualCount: 1; sex: female; **Location:** locationID: O2; continent: Europe; country: Spain; countryCode: ES; stateProvince: Aragón; county: Huesca; locality: Rebilla; verbatimElevation: 1158.13; decimalLatitude: 42.59427; decimalLongitude: 0.1529; geodeticDatum: WGS84; **Event:** eventID: 1; samplingProtocol: Sweeping; eventTime: Night
- **Type status:** Other material. **Occurrence:** individualCount: 2; sex: male; **Location:** locationID: O2; continent: Europe; country: Spain; countryCode: ES; stateProvince: Aragón; county: Huesca; locality: Rebilla; verbatimElevation: 1158.13; decimalLatitude: 42.59427; decimalLongitude: 0.1529; geodeticDatum: WGS84; **Event:** eventID: 1; samplingProtocol: Sweeping; eventTime: Night
- **Type status:** Other material. **Occurrence:** individualCount: 1; sex: male; **Location:** locationID: O2; continent: Europe; country: Spain; countryCode: ES; stateProvince: Aragón; county: Huesca; locality: Rebilla; verbatimElevation: 1158.13; decimalLatitude: 42.59427; decimalLongitude: 0.1529; geodeticDatum: WGS84; **Event:** eventID: 2; samplingProtocol: Sweeping; eventTime: Day
- **Type status:** Other material. **Occurrence:** individualCount: 1; sex: female; **Location:** locationID: P1; continent: Europe; country: Spain; countryCode: ES; stateProvince: Castilla y León; county: León; locality: Monte Robledo; verbatimElevation: 1071.58; decimalLatitude: 43.1445; decimalLongitude: -4.92675; geodeticDatum: WGS84; **Event:** eventID: 1; samplingProtocol: Aerial; eventTime: Night
- **Type status:** Other material. **Occurrence:** individualCount: 1; sex: male; **Location:** locationID: P1; continent: Europe; country: Spain; countryCode: ES; stateProvince: Castilla y León; county: León; locality: Monte Robledo; verbatimElevation: 1071.58; decimalLatitude: 43.1445; decimalLongitude: -4.92675; geodeticDatum: WGS84; **Event:** eventID: 1; samplingProtocol: Aerial; eventTime: Night
- **Type status:** Other material. **Occurrence:** individualCount: 1; sex: male; **Location:** locationID: P1; continent: Europe; country: Spain; countryCode: ES; stateProvince: Castilla y León; county: León; locality: Monte Robledo; verbatimElevation: 1071.58; decimalLatitude: 43.1445; decimalLongitude: -4.92675; geodeticDatum: WGS84; **Event:** eventID: 1; samplingProtocol: Aerial; eventTime: Night
- **Type status:** Other material. **Occurrence:** individualCount: 1; sex: female; **Location:** locationID: P1; continent: Europe; country: Spain; countryCode: ES; stateProvince: Castilla y León; county: León; locality: Monte Robledo; verbatimElevation: 1071.58; decimalLatitude: 43.1445; decimalLongitude: -4.92675; geodeticDatum: WGS84; **Event:** eventID: 1; samplingProtocol: Beating; eventTime: Day
- **Type status:** Other material. **Occurrence:** individualCount: 1; sex: male; **Location:** locationID: P1; continent: Europe; country: Spain; countryCode: ES; stateProvince: Castilla y León; county: León; locality: Monte Robledo; verbatimElevation: 1071.58; decimalLatitude: 43.1445; decimalLongitude: -4.92675; geodeticDatum: WGS84; **Event:** eventID: 1; samplingProtocol: Beating; eventTime: Day
- **Type status:** Other material. **Occurrence:** individualCount: 1; sex: female; **Location:** locationID: P1; continent: Europe; country: Spain; countryCode: ES; stateProvince: Castilla y León; county: León; locality: Monte Robledo; verbatimElevation: 1071.58; decimalLatitude: 43.1445; decimalLongitude: -4.92675; geodeticDatum: WGS84; **Event:** eventID: 2; samplingProtocol: Beating; eventTime: Day
- **Type status:** Other material. **Occurrence:** individualCount: 2; sex: female; **Location:** locationID: P1; continent: Europe; country: Spain; countryCode: ES; stateProvince: Castilla y León; county: León; locality: Monte Robledo; verbatimElevation: 1071.58; decimalLatitude: 43.1445; decimalLongitude: -4.92675; geodeticDatum: WGS84; **Event:** eventID: 1; samplingProtocol: Beating; eventTime: Night
- **Type status:** Other material. **Occurrence:** individualCount: 1; sex: male; **Location:** locationID: P1; continent: Europe; country: Spain; countryCode: ES; stateProvince: Castilla y León; county: León; locality: Monte Robledo; verbatimElevation: 1071.58; decimalLatitude: 43.1445; decimalLongitude: -4.92675; geodeticDatum: WGS84; **Event:** eventID: 1; samplingProtocol: Beating; eventTime: Night
- **Type status:** Other material. **Occurrence:** individualCount: 5; sex: female; **Location:** locationID: P2; continent: Europe; country: Spain; countryCode: ES; stateProvince: Castilla y León; county: León; locality: Joyoguelas; verbatimElevation: 763.98; decimalLatitude: 43.17771; decimalLongitude: -4.90579; geodeticDatum: WGS84; **Event:** eventID: 1; samplingProtocol: Aerial; eventTime: Night
- **Type status:** Other material. **Occurrence:** individualCount: 1; sex: male; **Location:** locationID: P2; continent: Europe; country: Spain; countryCode: ES; stateProvince: Castilla y León; county: León; locality: Joyoguelas; verbatimElevation: 763.98; decimalLatitude: 43.17771; decimalLongitude: -4.90579; geodeticDatum: WGS84; **Event:** eventID: 1; samplingProtocol: Aerial; eventTime: Night
- **Type status:** Other material. **Occurrence:** individualCount: 1; sex: female; **Location:** locationID: P2; continent: Europe; country: Spain; countryCode: ES; stateProvince: Castilla y León; county: León; locality: Joyoguelas; verbatimElevation: 763.98; decimalLatitude: 43.17771; decimalLongitude: -4.90579; geodeticDatum: WGS84; **Event:** eventID: 1; samplingProtocol: Aerial; eventTime: Night
- **Type status:** Other material. **Occurrence:** individualCount: 5; sex: female; **Location:** locationID: P2; continent: Europe; country: Spain; countryCode: ES; stateProvince: Castilla y León; county: León; locality: Joyoguelas; verbatimElevation: 763.98; decimalLatitude: 43.17771; decimalLongitude: -4.90579; geodeticDatum: WGS84; **Event:** eventID: 2; samplingProtocol: Aerial; eventTime: Night
- **Type status:** Other material. **Occurrence:** individualCount: 2; sex: female; **Location:** locationID: P2; continent: Europe; country: Spain; countryCode: ES; stateProvince: Castilla y León; county: León; locality: Joyoguelas; verbatimElevation: 763.98; decimalLatitude: 43.17771; decimalLongitude: -4.90579; geodeticDatum: WGS84; **Event:** eventID: 2; samplingProtocol: Aerial; eventTime: Night
- **Type status:** Other material. **Occurrence:** individualCount: 3; sex: female; **Location:** locationID: P2; continent: Europe; country: Spain; countryCode: ES; stateProvince: Castilla y León; county: León; locality: Joyoguelas; verbatimElevation: 763.98; decimalLatitude: 43.17771; decimalLongitude: -4.90579; geodeticDatum: WGS84; **Event:** eventID: 1; samplingProtocol: Beating; eventTime: Day
- **Type status:** Other material. **Occurrence:** individualCount: 2; sex: female; **Location:** locationID: P2; continent: Europe; country: Spain; countryCode: ES; stateProvince: Castilla y León; county: León; locality: Joyoguelas; verbatimElevation: 763.98; decimalLatitude: 43.17771; decimalLongitude: -4.90579; geodeticDatum: WGS84; **Event:** eventID: 1; samplingProtocol: Beating; eventTime: Night
- **Type status:** Other material. **Occurrence:** individualCount: 2; sex: male; **Location:** locationID: P2; continent: Europe; country: Spain; countryCode: ES; stateProvince: Castilla y León; county: León; locality: Joyoguelas; verbatimElevation: 763.98; decimalLatitude: 43.17771; decimalLongitude: -4.90579; geodeticDatum: WGS84; **Event:** eventID: 1; samplingProtocol: Beating; eventTime: Night
- **Type status:** Other material. **Occurrence:** individualCount: 1; sex: male; **Location:** locationID: P2; continent: Europe; country: Spain; countryCode: ES; stateProvince: Castilla y León; county: León; locality: Joyoguelas; verbatimElevation: 763.98; decimalLatitude: 43.17771; decimalLongitude: -4.90579; geodeticDatum: WGS84; **Event:** eventID: 1; samplingProtocol: Beating; eventTime: Night
- **Type status:** Other material. **Occurrence:** individualCount: 1; sex: female; **Location:** locationID: P2; continent: Europe; country: Spain; countryCode: ES; stateProvince: Castilla y León; county: León; locality: Joyoguelas; verbatimElevation: 763.98; decimalLatitude: 43.17771; decimalLongitude: -4.90579; geodeticDatum: WGS84; **Event:** eventID: 1; samplingProtocol: Sweeping; eventTime: Night
- **Type status:** Other material. **Occurrence:** individualCount: 2; sex: female; **Location:** locationID: P3; continent: Europe; country: Spain; countryCode: ES; stateProvince: Castilla y León; county: León; locality: Las Arroyas; verbatimElevation: 1097.1; decimalLatitude: 43.14351; decimalLongitude: -4.94878; geodeticDatum: WGS84; **Event:** eventID: 2; samplingProtocol: Aerial; eventTime: Night
- **Type status:** Other material. **Occurrence:** individualCount: 1; sex: female; **Location:** locationID: P3; continent: Europe; country: Spain; countryCode: ES; stateProvince: Castilla y León; county: León; locality: Las Arroyas; verbatimElevation: 1097.1; decimalLatitude: 43.14351; decimalLongitude: -4.94878; geodeticDatum: WGS84; **Event:** eventID: 2; samplingProtocol: Aerial; eventTime: Night
- **Type status:** Other material. **Occurrence:** individualCount: 2; sex: male; **Location:** locationID: P3; continent: Europe; country: Spain; countryCode: ES; stateProvince: Castilla y León; county: León; locality: Las Arroyas; verbatimElevation: 1097.1; decimalLatitude: 43.14351; decimalLongitude: -4.94878; geodeticDatum: WGS84; **Event:** eventID: 2; samplingProtocol: Aerial; eventTime: Night
- **Type status:** Other material. **Occurrence:** individualCount: 1; sex: female; **Location:** locationID: P3; continent: Europe; country: Spain; countryCode: ES; stateProvince: Castilla y León; county: León; locality: Las Arroyas; verbatimElevation: 1097.1; decimalLatitude: 43.14351; decimalLongitude: -4.94878; geodeticDatum: WGS84; **Event:** eventID: 1; samplingProtocol: Beating; eventTime: Day
- **Type status:** Other material. **Occurrence:** individualCount: 1; sex: female; **Location:** locationID: P3; continent: Europe; country: Spain; countryCode: ES; stateProvince: Castilla y León; county: León; locality: Las Arroyas; verbatimElevation: 1097.1; decimalLatitude: 43.14351; decimalLongitude: -4.94878; geodeticDatum: WGS84; **Event:** eventID: 1; samplingProtocol: Sweeping; eventTime: Night
- **Type status:** Other material. **Occurrence:** individualCount: 1; sex: female; **Location:** locationID: P3; continent: Europe; country: Spain; countryCode: ES; stateProvince: Castilla y León; county: León; locality: Las Arroyas; verbatimElevation: 1097.1; decimalLatitude: 43.14351; decimalLongitude: -4.94878; geodeticDatum: WGS84; **Event:** eventID: 1; samplingProtocol: Sweeping; eventTime: Night
- **Type status:** Other material. **Occurrence:** individualCount: 1; sex: female; **Location:** locationID: P4; continent: Europe; country: Spain; countryCode: ES; stateProvince: Castilla y León; county: León; locality: El Canto; verbatimElevation: 943.48; decimalLatitude: 43.17227; decimalLongitude: -4.90857; geodeticDatum: WGS84; **Event:** eventID: 1; samplingProtocol: Aerial; eventTime: Night
- **Type status:** Other material. **Occurrence:** individualCount: 7; sex: female; **Location:** locationID: P4; continent: Europe; country: Spain; countryCode: ES; stateProvince: Castilla y León; county: León; locality: El Canto; verbatimElevation: 943.48; decimalLatitude: 43.17227; decimalLongitude: -4.90857; geodeticDatum: WGS84; **Event:** eventID: 1; samplingProtocol: Aerial; eventTime: Night
- **Type status:** Other material. **Occurrence:** individualCount: 4; sex: male; **Location:** locationID: P4; continent: Europe; country: Spain; countryCode: ES; stateProvince: Castilla y León; county: León; locality: El Canto; verbatimElevation: 943.48; decimalLatitude: 43.17227; decimalLongitude: -4.90857; geodeticDatum: WGS84; **Event:** eventID: 1; samplingProtocol: Aerial; eventTime: Night
- **Type status:** Other material. **Occurrence:** individualCount: 6; sex: female; **Location:** locationID: P4; continent: Europe; country: Spain; countryCode: ES; stateProvince: Castilla y León; county: León; locality: El Canto; verbatimElevation: 943.48; decimalLatitude: 43.17227; decimalLongitude: -4.90857; geodeticDatum: WGS84; **Event:** eventID: 2; samplingProtocol: Aerial; eventTime: Night
- **Type status:** Other material. **Occurrence:** individualCount: 4; sex: male; **Location:** locationID: P4; continent: Europe; country: Spain; countryCode: ES; stateProvince: Castilla y León; county: León; locality: El Canto; verbatimElevation: 943.48; decimalLatitude: 43.17227; decimalLongitude: -4.90857; geodeticDatum: WGS84; **Event:** eventID: 2; samplingProtocol: Aerial; eventTime: Night
- **Type status:** Other material. **Occurrence:** individualCount: 10; sex: female; **Location:** locationID: P4; continent: Europe; country: Spain; countryCode: ES; stateProvince: Castilla y León; county: León; locality: El Canto; verbatimElevation: 943.48; decimalLatitude: 43.17227; decimalLongitude: -4.90857; geodeticDatum: WGS84; **Event:** eventID: 2; samplingProtocol: Aerial; eventTime: Night
- **Type status:** Other material. **Occurrence:** individualCount: 6; sex: male; **Location:** locationID: P4; continent: Europe; country: Spain; countryCode: ES; stateProvince: Castilla y León; county: León; locality: El Canto; verbatimElevation: 943.48; decimalLatitude: 43.17227; decimalLongitude: -4.90857; geodeticDatum: WGS84; **Event:** eventID: 2; samplingProtocol: Aerial; eventTime: Night
- **Type status:** Other material. **Occurrence:** individualCount: 7; sex: female; **Location:** locationID: P4; continent: Europe; country: Spain; countryCode: ES; stateProvince: Castilla y León; county: León; locality: El Canto; verbatimElevation: 943.48; decimalLatitude: 43.17227; decimalLongitude: -4.90857; geodeticDatum: WGS84; **Event:** eventID: 1; samplingProtocol: Beating; eventTime: Day
- **Type status:** Other material. **Occurrence:** individualCount: 1; sex: male; **Location:** locationID: P4; continent: Europe; country: Spain; countryCode: ES; stateProvince: Castilla y León; county: León; locality: El Canto; verbatimElevation: 943.48; decimalLatitude: 43.17227; decimalLongitude: -4.90857; geodeticDatum: WGS84; **Event:** eventID: 1; samplingProtocol: Beating; eventTime: Day
- **Type status:** Other material. **Occurrence:** individualCount: 10; sex: female; **Location:** locationID: P4; continent: Europe; country: Spain; countryCode: ES; stateProvince: Castilla y León; county: León; locality: El Canto; verbatimElevation: 943.48; decimalLatitude: 43.17227; decimalLongitude: -4.90857; geodeticDatum: WGS84; **Event:** eventID: 2; samplingProtocol: Beating; eventTime: Day
- **Type status:** Other material. **Occurrence:** individualCount: 5; sex: male; **Location:** locationID: P4; continent: Europe; country: Spain; countryCode: ES; stateProvince: Castilla y León; county: León; locality: El Canto; verbatimElevation: 943.48; decimalLatitude: 43.17227; decimalLongitude: -4.90857; geodeticDatum: WGS84; **Event:** eventID: 2; samplingProtocol: Beating; eventTime: Day
- **Type status:** Other material. **Occurrence:** individualCount: 7; sex: female; **Location:** locationID: P4; continent: Europe; country: Spain; countryCode: ES; stateProvince: Castilla y León; county: León; locality: El Canto; verbatimElevation: 943.48; decimalLatitude: 43.17227; decimalLongitude: -4.90857; geodeticDatum: WGS84; **Event:** eventID: 1; samplingProtocol: Beating; eventTime: Night
- **Type status:** Other material. **Occurrence:** individualCount: 6; sex: male; **Location:** locationID: P4; continent: Europe; country: Spain; countryCode: ES; stateProvince: Castilla y León; county: León; locality: El Canto; verbatimElevation: 943.48; decimalLatitude: 43.17227; decimalLongitude: -4.90857; geodeticDatum: WGS84; **Event:** eventID: 1; samplingProtocol: Beating; eventTime: Night
- **Type status:** Other material. **Occurrence:** individualCount: 2; sex: female; **Location:** locationID: P4; continent: Europe; country: Spain; countryCode: ES; stateProvince: Castilla y León; county: León; locality: El Canto; verbatimElevation: 943.48; decimalLatitude: 43.17227; decimalLongitude: -4.90857; geodeticDatum: WGS84; **Event:** eventID: 1; samplingProtocol: Beating; eventTime: Night
- **Type status:** Other material. **Occurrence:** individualCount: 2; sex: male; **Location:** locationID: P4; continent: Europe; country: Spain; countryCode: ES; stateProvince: Castilla y León; county: León; locality: El Canto; verbatimElevation: 943.48; decimalLatitude: 43.17227; decimalLongitude: -4.90857; geodeticDatum: WGS84; **Event:** eventID: 1; samplingProtocol: Beating; eventTime: Night
- **Type status:** Other material. **Occurrence:** individualCount: 1; sex: female; **Location:** locationID: P4; continent: Europe; country: Spain; countryCode: ES; stateProvince: Castilla y León; county: León; locality: El Canto; verbatimElevation: 943.48; decimalLatitude: 43.17227; decimalLongitude: -4.90857; geodeticDatum: WGS84; **Event:** eventID: 1; samplingProtocol: Sweeping; eventTime: Day
- **Type status:** Other material. **Occurrence:** individualCount: 1; sex: male; **Location:** locationID: P4; continent: Europe; country: Spain; countryCode: ES; stateProvince: Castilla y León; county: León; locality: El Canto; verbatimElevation: 943.48; decimalLatitude: 43.17227; decimalLongitude: -4.90857; geodeticDatum: WGS84; **Event:** eventID: 2; samplingProtocol: Sweeping; eventTime: Day
- **Type status:** Other material. **Occurrence:** individualCount: 2; sex: male; **Location:** locationID: P4; continent: Europe; country: Spain; countryCode: ES; stateProvince: Castilla y León; county: León; locality: El Canto; verbatimElevation: 943.48; decimalLatitude: 43.17227; decimalLongitude: -4.90857; geodeticDatum: WGS84; **Event:** eventID: 1; samplingProtocol: Sweeping; eventTime: Night
- **Type status:** Other material. **Occurrence:** individualCount: 1; sex: female; **Location:** locationID: P4; continent: Europe; country: Spain; countryCode: ES; stateProvince: Castilla y León; county: León; locality: El Canto; verbatimElevation: 943.48; decimalLatitude: 43.17227; decimalLongitude: -4.90857; geodeticDatum: WGS84; **Event:** eventID: 1; samplingProtocol: Sweeping; eventTime: Night
- **Type status:** Other material. **Occurrence:** individualCount: 1; sex: male; **Location:** locationID: P4; continent: Europe; country: Spain; countryCode: ES; stateProvince: Castilla y León; county: León; locality: El Canto; verbatimElevation: 943.48; decimalLatitude: 43.17227; decimalLongitude: -4.90857; geodeticDatum: WGS84; **Event:** eventID: 1; samplingProtocol: Sweeping; eventTime: Night
- **Type status:** Other material. **Occurrence:** individualCount: 9; sex: female; **Location:** locationID: S1; continent: Europe; country: Spain; countryCode: ES; stateProvince: Andalucía; county: Granada; locality: Soportujar; verbatimElevation: 1786.57; decimalLatitude: 36.96151; decimalLongitude: -3.41881; geodeticDatum: WGS84; **Event:** eventID: 1; samplingProtocol: Aerial; eventTime: Night
- **Type status:** Other material. **Occurrence:** individualCount: 3; sex: male; **Location:** locationID: S1; continent: Europe; country: Spain; countryCode: ES; stateProvince: Andalucía; county: Granada; locality: Soportujar; verbatimElevation: 1786.57; decimalLatitude: 36.96151; decimalLongitude: -3.41881; geodeticDatum: WGS84; **Event:** eventID: 1; samplingProtocol: Aerial; eventTime: Night
- **Type status:** Other material. **Occurrence:** individualCount: 3; sex: female; **Location:** locationID: S1; continent: Europe; country: Spain; countryCode: ES; stateProvince: Andalucía; county: Granada; locality: Soportujar; verbatimElevation: 1786.57; decimalLatitude: 36.96151; decimalLongitude: -3.41881; geodeticDatum: WGS84; **Event:** eventID: 2; samplingProtocol: Aerial; eventTime: Night
- **Type status:** Other material. **Occurrence:** individualCount: 1; sex: male; **Location:** locationID: S1; continent: Europe; country: Spain; countryCode: ES; stateProvince: Andalucía; county: Granada; locality: Soportujar; verbatimElevation: 1786.57; decimalLatitude: 36.96151; decimalLongitude: -3.41881; geodeticDatum: WGS84; **Event:** eventID: 2; samplingProtocol: Aerial; eventTime: Night
- **Type status:** Other material. **Occurrence:** individualCount: 2; sex: female; **Location:** locationID: S1; continent: Europe; country: Spain; countryCode: ES; stateProvince: Andalucía; county: Granada; locality: Soportujar; verbatimElevation: 1786.57; decimalLatitude: 36.96151; decimalLongitude: -3.41881; geodeticDatum: WGS84; **Event:** eventID: 3; samplingProtocol: Aerial; eventTime: Night
- **Type status:** Other material. **Occurrence:** individualCount: 5; sex: male; **Location:** locationID: S1; continent: Europe; country: Spain; countryCode: ES; stateProvince: Andalucía; county: Granada; locality: Soportujar; verbatimElevation: 1786.57; decimalLatitude: 36.96151; decimalLongitude: -3.41881; geodeticDatum: WGS84; **Event:** eventID: 3; samplingProtocol: Aerial; eventTime: Night
- **Type status:** Other material. **Occurrence:** individualCount: 8; sex: female; **Location:** locationID: S1; continent: Europe; country: Spain; countryCode: ES; stateProvince: Andalucía; county: Granada; locality: Soportujar; verbatimElevation: 1786.57; decimalLatitude: 36.96151; decimalLongitude: -3.41881; geodeticDatum: WGS84; **Event:** eventID: 4; samplingProtocol: Aerial; eventTime: Night
- **Type status:** Other material. **Occurrence:** individualCount: 2; sex: male; **Location:** locationID: S1; continent: Europe; country: Spain; countryCode: ES; stateProvince: Andalucía; county: Granada; locality: Soportujar; verbatimElevation: 1786.57; decimalLatitude: 36.96151; decimalLongitude: -3.41881; geodeticDatum: WGS84; **Event:** eventID: 4; samplingProtocol: Aerial; eventTime: Night
- **Type status:** Other material. **Occurrence:** individualCount: 3; sex: female; **Location:** locationID: S1; continent: Europe; country: Spain; countryCode: ES; stateProvince: Andalucía; county: Granada; locality: Soportujar; verbatimElevation: 1786.57; decimalLatitude: 36.96151; decimalLongitude: -3.41881; geodeticDatum: WGS84; **Event:** eventID: 1; samplingProtocol: Beating; eventTime: Day
- **Type status:** Other material. **Occurrence:** individualCount: 3; sex: male; **Location:** locationID: S1; continent: Europe; country: Spain; countryCode: ES; stateProvince: Andalucía; county: Granada; locality: Soportujar; verbatimElevation: 1786.57; decimalLatitude: 36.96151; decimalLongitude: -3.41881; geodeticDatum: WGS84; **Event:** eventID: 1; samplingProtocol: Beating; eventTime: Day
- **Type status:** Other material. **Occurrence:** individualCount: 1; sex: female; **Location:** locationID: S1; continent: Europe; country: Spain; countryCode: ES; stateProvince: Andalucía; county: Granada; locality: Soportujar; verbatimElevation: 1786.57; decimalLatitude: 36.96151; decimalLongitude: -3.41881; geodeticDatum: WGS84; **Event:** eventID: 2; samplingProtocol: Beating; eventTime: Day
- **Type status:** Other material. **Occurrence:** individualCount: 5; sex: male; **Location:** locationID: S1; continent: Europe; country: Spain; countryCode: ES; stateProvince: Andalucía; county: Granada; locality: Soportujar; verbatimElevation: 1786.57; decimalLatitude: 36.96151; decimalLongitude: -3.41881; geodeticDatum: WGS84; **Event:** eventID: 1; samplingProtocol: Beating; eventTime: Night
- **Type status:** Other material. **Occurrence:** individualCount: 2; sex: female; **Location:** locationID: S1; continent: Europe; country: Spain; countryCode: ES; stateProvince: Andalucía; county: Granada; locality: Soportujar; verbatimElevation: 1786.57; decimalLatitude: 36.96151; decimalLongitude: -3.41881; geodeticDatum: WGS84; **Event:** eventID: 2; samplingProtocol: Beating; eventTime: Night
- **Type status:** Other material. **Occurrence:** individualCount: 2; sex: male; **Location:** locationID: S1; continent: Europe; country: Spain; countryCode: ES; stateProvince: Andalucía; county: Granada; locality: Soportujar; verbatimElevation: 1786.57; decimalLatitude: 36.96151; decimalLongitude: -3.41881; geodeticDatum: WGS84; **Event:** eventID: 2; samplingProtocol: Beating; eventTime: Night
- **Type status:** Other material. **Occurrence:** individualCount: 1; sex: female; **Location:** locationID: S1; continent: Europe; country: Spain; countryCode: ES; stateProvince: Andalucía; county: Granada; locality: Soportujar; verbatimElevation: 1786.57; decimalLatitude: 36.96151; decimalLongitude: -3.41881; geodeticDatum: WGS84; **Event:** eventID: 1; samplingProtocol: Sweeping; eventTime: Night
- **Type status:** Other material. **Occurrence:** individualCount: 1; sex: male; **Location:** locationID: S1; continent: Europe; country: Spain; countryCode: ES; stateProvince: Andalucía; county: Granada; locality: Soportujar; verbatimElevation: 1786.57; decimalLatitude: 36.96151; decimalLongitude: -3.41881; geodeticDatum: WGS84; **Event:** eventID: 1; samplingProtocol: Sweeping; eventTime: Night
- **Type status:** Other material. **Occurrence:** individualCount: 1; sex: male; **Location:** locationID: S1; continent: Europe; country: Spain; countryCode: ES; stateProvince: Andalucía; county: Granada; locality: Soportujar; verbatimElevation: 1786.57; decimalLatitude: 36.96151; decimalLongitude: -3.41881; geodeticDatum: WGS84; **Event:** eventID: 2; samplingProtocol: Sweeping; eventTime: Night
- **Type status:** Other material. **Occurrence:** individualCount: 15; sex: female; **Location:** locationID: S2; continent: Europe; country: Spain; countryCode: ES; stateProvince: Andalucía; county: Granada; locality: Camarate; verbatimElevation: 1713.96; decimalLatitude: 37.18377; decimalLongitude: -3.26282; geodeticDatum: WGS84; **Event:** eventID: 1; samplingProtocol: Aerial; eventTime: Night
- **Type status:** Other material. **Occurrence:** individualCount: 3; sex: male; **Location:** locationID: S2; continent: Europe; country: Spain; countryCode: ES; stateProvince: Andalucía; county: Granada; locality: Camarate; verbatimElevation: 1713.96; decimalLatitude: 37.18377; decimalLongitude: -3.26282; geodeticDatum: WGS84; **Event:** eventID: 1; samplingProtocol: Aerial; eventTime: Night
- **Type status:** Other material. **Occurrence:** individualCount: 4; sex: female; **Location:** locationID: S2; continent: Europe; country: Spain; countryCode: ES; stateProvince: Andalucía; county: Granada; locality: Camarate; verbatimElevation: 1713.96; decimalLatitude: 37.18377; decimalLongitude: -3.26282; geodeticDatum: WGS84; **Event:** eventID: 2; samplingProtocol: Aerial; eventTime: Night
- **Type status:** Other material. **Occurrence:** individualCount: 2; sex: female; **Location:** locationID: S2; continent: Europe; country: Spain; countryCode: ES; stateProvince: Andalucía; county: Granada; locality: Camarate; verbatimElevation: 1713.96; decimalLatitude: 37.18377; decimalLongitude: -3.26282; geodeticDatum: WGS84; **Event:** eventID: 3; samplingProtocol: Aerial; eventTime: Night
- **Type status:** Other material. **Occurrence:** individualCount: 4; sex: female; **Location:** locationID: S2; continent: Europe; country: Spain; countryCode: ES; stateProvince: Andalucía; county: Granada; locality: Camarate; verbatimElevation: 1713.96; decimalLatitude: 37.18377; decimalLongitude: -3.26282; geodeticDatum: WGS84; **Event:** eventID: 4; samplingProtocol: Aerial; eventTime: Night
- **Type status:** Other material. **Occurrence:** individualCount: 5; sex: male; **Location:** locationID: S2; continent: Europe; country: Spain; countryCode: ES; stateProvince: Andalucía; county: Granada; locality: Camarate; verbatimElevation: 1713.96; decimalLatitude: 37.18377; decimalLongitude: -3.26282; geodeticDatum: WGS84; **Event:** eventID: 4; samplingProtocol: Aerial; eventTime: Night
- **Type status:** Other material. **Occurrence:** individualCount: 2; sex: female; **Location:** locationID: S2; continent: Europe; country: Spain; countryCode: ES; stateProvince: Andalucía; county: Granada; locality: Camarate; verbatimElevation: 1713.96; decimalLatitude: 37.18377; decimalLongitude: -3.26282; geodeticDatum: WGS84; **Event:** eventID: 1; samplingProtocol: Beating; eventTime: Day
- **Type status:** Other material. **Occurrence:** individualCount: 2; sex: female; **Location:** locationID: S2; continent: Europe; country: Spain; countryCode: ES; stateProvince: Andalucía; county: Granada; locality: Camarate; verbatimElevation: 1713.96; decimalLatitude: 37.18377; decimalLongitude: -3.26282; geodeticDatum: WGS84; **Event:** eventID: 2; samplingProtocol: Beating; eventTime: Day
- **Type status:** Other material. **Occurrence:** individualCount: 2; sex: male; **Location:** locationID: S2; continent: Europe; country: Spain; countryCode: ES; stateProvince: Andalucía; county: Granada; locality: Camarate; verbatimElevation: 1713.96; decimalLatitude: 37.18377; decimalLongitude: -3.26282; geodeticDatum: WGS84; **Event:** eventID: 2; samplingProtocol: Beating; eventTime: Day
- **Type status:** Other material. **Occurrence:** individualCount: 1; sex: female; **Location:** locationID: S2; continent: Europe; country: Spain; countryCode: ES; stateProvince: Andalucía; county: Granada; locality: Camarate; verbatimElevation: 1713.96; decimalLatitude: 37.18377; decimalLongitude: -3.26282; geodeticDatum: WGS84; **Event:** eventID: 1; samplingProtocol: Beating; eventTime: Night
- **Type status:** Other material. **Occurrence:** individualCount: 2; sex: female; **Location:** locationID: S2; continent: Europe; country: Spain; countryCode: ES; stateProvince: Andalucía; county: Granada; locality: Camarate; verbatimElevation: 1713.96; decimalLatitude: 37.18377; decimalLongitude: -3.26282; geodeticDatum: WGS84; **Event:** eventID: 2; samplingProtocol: Beating; eventTime: Night
- **Type status:** Other material. **Occurrence:** individualCount: 1; sex: female; **Location:** locationID: S2; continent: Europe; country: Spain; countryCode: ES; stateProvince: Andalucía; county: Granada; locality: Camarate; verbatimElevation: 1713.96; decimalLatitude: 37.18377; decimalLongitude: -3.26282; geodeticDatum: WGS84; **Event:** eventID: 2; samplingProtocol: Sweeping; eventTime: Day
- **Type status:** Other material. **Occurrence:** individualCount: 1; sex: male; **Location:** locationID: S2; continent: Europe; country: Spain; countryCode: ES; stateProvince: Andalucía; county: Granada; locality: Camarate; verbatimElevation: 1713.96; decimalLatitude: 37.18377; decimalLongitude: -3.26282; geodeticDatum: WGS84; **Event:** eventID: 1; samplingProtocol: Sweeping; eventTime: Night
- **Type status:** Other material. **Occurrence:** individualCount: 2; sex: female; **Location:** locationID: S2; continent: Europe; country: Spain; countryCode: ES; stateProvince: Andalucía; county: Granada; locality: Camarate; verbatimElevation: 1713.96; decimalLatitude: 37.18377; decimalLongitude: -3.26282; geodeticDatum: WGS84; **Event:** eventID: 2; samplingProtocol: Sweeping; eventTime: Night

##### Distribution

Europe, North Africa to Azerbaijan, Iran

#### Dipoena nigroreticulata

(Simon, 1879)

##### Materials

- **Type status:** Other material. **Occurrence:** individualCount: 2; sex: male; **Location:** locationID: P1; continent: Europe; country: Spain; countryCode: ES; stateProvince: Castilla y León; county: León; locality: Monte Robledo; verbatimElevation: 1071.58; decimalLatitude: 43.1445; decimalLongitude: -4.92675; geodeticDatum: WGS84; **Event:** eventID: 2; samplingProtocol: Aerial; eventTime: Night
- **Type status:** Other material. **Occurrence:** individualCount: 1; sex: male; **Location:** locationID: P1; continent: Europe; country: Spain; countryCode: ES; stateProvince: Castilla y León; county: León; locality: Monte Robledo; verbatimElevation: 1071.58; decimalLatitude: 43.1445; decimalLongitude: -4.92675; geodeticDatum: WGS84; **Event:** eventID: 2; samplingProtocol: Beating; eventTime: Day
- **Type status:** Other material. **Occurrence:** individualCount: 2; sex: male; **Location:** locationID: P2; continent: Europe; country: Spain; countryCode: ES; stateProvince: Castilla y León; county: León; locality: Joyoguelas; verbatimElevation: 763.98; decimalLatitude: 43.17771; decimalLongitude: -4.90579; geodeticDatum: WGS84; **Event:** eventID: 1; samplingProtocol: Aerial; eventTime: Night
- **Type status:** Other material. **Occurrence:** individualCount: 2; sex: female; **Location:** locationID: P2; continent: Europe; country: Spain; countryCode: ES; stateProvince: Castilla y León; county: León; locality: Joyoguelas; verbatimElevation: 763.98; decimalLatitude: 43.17771; decimalLongitude: -4.90579; geodeticDatum: WGS84; **Event:** eventID: 1; samplingProtocol: Aerial; eventTime: Night
- **Type status:** Other material. **Occurrence:** individualCount: 3; sex: male; **Location:** locationID: P2; continent: Europe; country: Spain; countryCode: ES; stateProvince: Castilla y León; county: León; locality: Joyoguelas; verbatimElevation: 763.98; decimalLatitude: 43.17771; decimalLongitude: -4.90579; geodeticDatum: WGS84; **Event:** eventID: 1; samplingProtocol: Aerial; eventTime: Night
- **Type status:** Other material. **Occurrence:** individualCount: 2; sex: female; **Location:** locationID: P2; continent: Europe; country: Spain; countryCode: ES; stateProvince: Castilla y León; county: León; locality: Joyoguelas; verbatimElevation: 763.98; decimalLatitude: 43.17771; decimalLongitude: -4.90579; geodeticDatum: WGS84; **Event:** eventID: 2; samplingProtocol: Aerial; eventTime: Night
- **Type status:** Other material. **Occurrence:** individualCount: 1; sex: male; **Location:** locationID: P2; continent: Europe; country: Spain; countryCode: ES; stateProvince: Castilla y León; county: León; locality: Joyoguelas; verbatimElevation: 763.98; decimalLatitude: 43.17771; decimalLongitude: -4.90579; geodeticDatum: WGS84; **Event:** eventID: 2; samplingProtocol: Aerial; eventTime: Night

##### Distribution

Europe to Azerbaijan

#### Dipoena torva

(Thorell, 1875)

##### Materials

- **Type status:** Other material. **Occurrence:** individualCount: 1; sex: male; **Location:** locationID: A1; continent: Europe; country: Spain; countryCode: ES; stateProvince: Catalonia; county: Lleida; locality: Sola de Boi; verbatimElevation: 1759.8; decimalLatitude: 42.54958; decimalLongitude: 0.87254; geodeticDatum: WGS84; **Event:** eventID: 1; samplingProtocol: Beating; eventTime: Day
- **Type status:** Other material. **Occurrence:** individualCount: 1; sex: female; **Location:** locationID: A2; continent: Europe; country: Spain; countryCode: ES; stateProvince: Catalonia; county: Lleida; locality: Sola de Boi; verbatimElevation: 1738.7; decimalLatitude: 42.54913; decimalLongitude: 0.87137; geodeticDatum: WGS84; **Event:** eventID: 1; samplingProtocol: Aerial; eventTime: Night
- **Type status:** Other material. **Occurrence:** individualCount: 1; sex: male; **Location:** locationID: O2; continent: Europe; country: Spain; countryCode: ES; stateProvince: Aragón; county: Huesca; locality: Rebilla; verbatimElevation: 1158.13; decimalLatitude: 42.59427; decimalLongitude: 0.1529; geodeticDatum: WGS84; **Event:** eventID: 2; samplingProtocol: Aerial; eventTime: Night

##### Distribution

Palearctic

##### Notes

**Figure 12.**
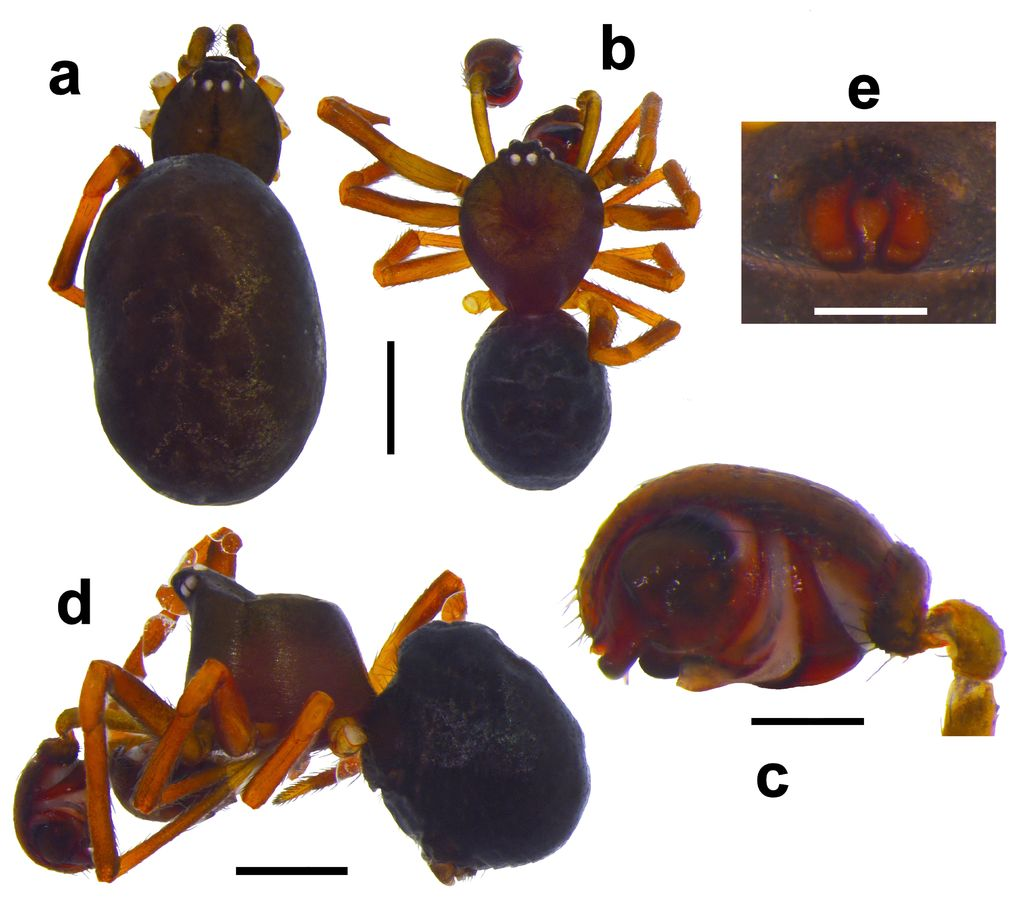
*Dipoena torva* (Thorell, 1875). a: female, dorsal habitus, scale = 0.8 mm; b: male, dorsal habitus, scale = 0.8 mm; c: male, lateral habitus, scale = 0.6 mm; d: female epigynum, ventral, scale = 0.2 mm; e: left male palp, retrolateral, scale = 0.2 mm.

First record for Spain. See Fig. 12.

#### Dipoena umbratilis

(Simon, 1873)

##### Materials

- **Type status:** Other material. **Occurrence:** individualCount: 1; sex: male; **Location:** locationID: S1; continent: Europe; country: Spain; countryCode: ES; stateProvince: Andalucía; county: Granada; locality: Soportujar; verbatimElevation: 1786.57; decimalLatitude: 36.96151; decimalLongitude: -3.41881; geodeticDatum: WGS84; **Event:** eventID: 4; samplingProtocol: Aerial; eventTime: Night
- **Type status:** Other material. **Occurrence:** individualCount: 1; sex: male; **Location:** locationID: S2; continent: Europe; country: Spain; countryCode: ES; stateProvince: Andalucía; county: Granada; locality: Camarate; verbatimElevation: 1713.96; decimalLatitude: 37.18377; decimalLongitude: -3.26282; geodeticDatum: WGS84; **Event:** eventID: 1; samplingProtocol: Aerial; eventTime: Night

##### Distribution

Western Mediterranean

#### Enoplognatha thoracica

(Hahn, 1833)

##### Materials

- **Type status:** Other material. **Occurrence:** individualCount: 1; sex: male; **Location:** locationID: A1; continent: Europe; country: Spain; countryCode: ES; stateProvince: Catalonia; county: Lleida; locality: Sola de Boi; verbatimElevation: 1759.8; decimalLatitude: 42.54958; decimalLongitude: 0.87254; geodeticDatum: WGS84; **Event:** eventID: D; samplingProtocol: Pitfall
- **Type status:** Other material. **Occurrence:** individualCount: 1; sex: male; **Location:** locationID: A1; continent: Europe; country: Spain; countryCode: ES; stateProvince: Catalonia; county: Lleida; locality: Sola de Boi; verbatimElevation: 1759.8; decimalLatitude: 42.54958; decimalLongitude: 0.87254; geodeticDatum: WGS84; **Event:** eventID: I; samplingProtocol: Pitfall
- **Type status:** Other material. **Occurrence:** individualCount: 1; sex: female; **Location:** locationID: A2; continent: Europe; country: Spain; countryCode: ES; stateProvince: Catalonia; county: Lleida; locality: Sola de Boi; verbatimElevation: 1738.7; decimalLatitude: 42.54913; decimalLongitude: 0.87137; geodeticDatum: WGS84; **Event:** eventID: F; samplingProtocol: Pitfall
- **Type status:** Other material. **Occurrence:** individualCount: 1; sex: male; **Location:** locationID: A2; continent: Europe; country: Spain; countryCode: ES; stateProvince: Catalonia; county: Lleida; locality: Sola de Boi; verbatimElevation: 1738.7; decimalLatitude: 42.54913; decimalLongitude: 0.87137; geodeticDatum: WGS84; **Event:** eventID: F; samplingProtocol: Pitfall
- **Type status:** Other material. **Occurrence:** individualCount: 1; sex: male; **Location:** locationID: P1; continent: Europe; country: Spain; countryCode: ES; stateProvince: Castilla y León; county: León; locality: Monte Robledo; verbatimElevation: 1071.58; decimalLatitude: 43.1445; decimalLongitude: -4.92675; geodeticDatum: WGS84; **Event:** eventID: B; samplingProtocol: Pitfall
- **Type status:** Other material. **Occurrence:** individualCount: 1; sex: male; **Location:** locationID: P2; continent: Europe; country: Spain; countryCode: ES; stateProvince: Castilla y León; county: León; locality: Joyoguelas; verbatimElevation: 763.98; decimalLatitude: 43.17771; decimalLongitude: -4.90579; geodeticDatum: WGS84; **Event:** eventID: G; samplingProtocol: Pitfall
- **Type status:** Other material. **Occurrence:** individualCount: 1; sex: male; **Location:** locationID: P3; continent: Europe; country: Spain; countryCode: ES; stateProvince: Castilla y León; county: León; locality: Las Arroyas; verbatimElevation: 1097.1; decimalLatitude: 43.14351; decimalLongitude: -4.94878; geodeticDatum: WGS84; **Event:** eventID: 2; samplingProtocol: Aerial; eventTime: Night
- **Type status:** Other material. **Occurrence:** individualCount: 1; sex: male; **Location:** locationID: P3; continent: Europe; country: Spain; countryCode: ES; stateProvince: Castilla y León; county: León; locality: Las Arroyas; verbatimElevation: 1097.1; decimalLatitude: 43.14351; decimalLongitude: -4.94878; geodeticDatum: WGS84; **Event:** eventID: B; samplingProtocol: Pitfall
- **Type status:** Other material. **Occurrence:** individualCount: 1; sex: male; **Location:** locationID: P3; continent: Europe; country: Spain; countryCode: ES; stateProvince: Castilla y León; county: León; locality: Las Arroyas; verbatimElevation: 1097.1; decimalLatitude: 43.14351; decimalLongitude: -4.94878; geodeticDatum: WGS84; **Event:** eventID: E; samplingProtocol: Pitfall
- **Type status:** Other material. **Occurrence:** individualCount: 1; sex: male; **Location:** locationID: P3; continent: Europe; country: Spain; countryCode: ES; stateProvince: Castilla y León; county: León; locality: Las Arroyas; verbatimElevation: 1097.1; decimalLatitude: 43.14351; decimalLongitude: -4.94878; geodeticDatum: WGS84; **Event:** eventID: I; samplingProtocol: Pitfall
- **Type status:** Other material. **Occurrence:** individualCount: 1; sex: male; **Location:** locationID: P3; continent: Europe; country: Spain; countryCode: ES; stateProvince: Castilla y León; county: León; locality: Las Arroyas; verbatimElevation: 1097.1; decimalLatitude: 43.14351; decimalLongitude: -4.94878; geodeticDatum: WGS84; **Event:** eventID: K; samplingProtocol: Pitfall
- **Type status:** Other material. **Occurrence:** individualCount: 1; sex: male; **Location:** locationID: P3; continent: Europe; country: Spain; countryCode: ES; stateProvince: Castilla y León; county: León; locality: Las Arroyas; verbatimElevation: 1097.1; decimalLatitude: 43.14351; decimalLongitude: -4.94878; geodeticDatum: WGS84; **Event:** eventID: 1; samplingProtocol: Sweeping; eventTime: Night
- **Type status:** Other material. **Occurrence:** individualCount: 1; sex: male; **Location:** locationID: P4; continent: Europe; country: Spain; countryCode: ES; stateProvince: Castilla y León; county: León; locality: El Canto; verbatimElevation: 943.48; decimalLatitude: 43.17227; decimalLongitude: -4.90857; geodeticDatum: WGS84; **Event:** eventID: C; samplingProtocol: Pitfall
- **Type status:** Other material. **Occurrence:** individualCount: 1; sex: male; **Location:** locationID: P4; continent: Europe; country: Spain; countryCode: ES; stateProvince: Castilla y León; county: León; locality: El Canto; verbatimElevation: 943.48; decimalLatitude: 43.17227; decimalLongitude: -4.90857; geodeticDatum: WGS84; **Event:** eventID: E; samplingProtocol: Pitfall
- **Type status:** Other material. **Occurrence:** individualCount: 2; sex: male; **Location:** locationID: P4; continent: Europe; country: Spain; countryCode: ES; stateProvince: Castilla y León; county: León; locality: El Canto; verbatimElevation: 943.48; decimalLatitude: 43.17227; decimalLongitude: -4.90857; geodeticDatum: WGS84; **Event:** eventID: H; samplingProtocol: Pitfall
- **Type status:** Other material. **Occurrence:** individualCount: 2; sex: male; **Location:** locationID: P4; continent: Europe; country: Spain; countryCode: ES; stateProvince: Castilla y León; county: León; locality: El Canto; verbatimElevation: 943.48; decimalLatitude: 43.17227; decimalLongitude: -4.90857; geodeticDatum: WGS84; **Event:** eventID: K; samplingProtocol: Pitfall
- **Type status:** Other material. **Occurrence:** individualCount: 1; sex: female; **Location:** locationID: P4; continent: Europe; country: Spain; countryCode: ES; stateProvince: Castilla y León; county: León; locality: El Canto; verbatimElevation: 943.48; decimalLatitude: 43.17227; decimalLongitude: -4.90857; geodeticDatum: WGS84; **Event:** eventID: 1; samplingProtocol: Sweeping; eventTime: Night

##### Distribution

Holarctic

#### Episinus algiricus

Lucas, 1846

##### Materials

- **Type status:** Other material. **Occurrence:** individualCount: 1; sex: female; **Location:** locationID: C1; continent: Europe; country: Spain; countryCode: ES; stateProvince: Castilla-La Mancha; county: Ciudad Real; locality: Valle Brezoso; verbatimElevation: 756.56; decimalLatitude: 39.35663; decimalLongitude: -4.35912; geodeticDatum: WGS84; **Event:** eventID: 1; samplingProtocol: Aerial; eventTime: Night
- **Type status:** Other material. **Occurrence:** individualCount: 1; sex: male; **Location:** locationID: C1; continent: Europe; country: Spain; countryCode: ES; stateProvince: Castilla-La Mancha; county: Ciudad Real; locality: Valle Brezoso; verbatimElevation: 756.56; decimalLatitude: 39.35663; decimalLongitude: -4.35912; geodeticDatum: WGS84; **Event:** eventID: 2; samplingProtocol: Beating; eventTime: Night
- **Type status:** Other material. **Occurrence:** individualCount: 1; sex: male; **Location:** locationID: C3; continent: Europe; country: Spain; countryCode: ES; stateProvince: Castilla-La Mancha; county: Ciudad Real; locality: La Quesera; verbatimElevation: 767.55; decimalLatitude: 39.36177; decimalLongitude: -4.41733; geodeticDatum: WGS84; **Event:** eventID: 1; samplingProtocol: Aerial; eventTime: Night
- **Type status:** Other material. **Occurrence:** individualCount: 1; sex: male; **Location:** locationID: C3; continent: Europe; country: Spain; countryCode: ES; stateProvince: Castilla-La Mancha; county: Ciudad Real; locality: La Quesera; verbatimElevation: 767.55; decimalLatitude: 39.36177; decimalLongitude: -4.41733; geodeticDatum: WGS84; **Event:** eventID: 1; samplingProtocol: Beating; eventTime: Night
- **Type status:** Other material. **Occurrence:** individualCount: 1; sex: female; **Location:** locationID: C3; continent: Europe; country: Spain; countryCode: ES; stateProvince: Castilla-La Mancha; county: Ciudad Real; locality: La Quesera; verbatimElevation: 767.55; decimalLatitude: 39.36177; decimalLongitude: -4.41733; geodeticDatum: WGS84; **Event:** eventID: 1; samplingProtocol: Sweeping; eventTime: Day
- **Type status:** Other material. **Occurrence:** individualCount: 2; sex: female; **Location:** locationID: M1; continent: Europe; country: Spain; countryCode: ES; stateProvince: Extremadura; county: Cáceres; locality: Peña Falcón; verbatimElevation: 320.6; decimalLatitude: 39.83296; decimalLongitude: -6.0641; geodeticDatum: WGS84; **Event:** eventID: 1; samplingProtocol: Aerial; eventTime: Night
- **Type status:** Other material. **Occurrence:** individualCount: 1; sex: male; **Location:** locationID: M1; continent: Europe; country: Spain; countryCode: ES; stateProvince: Extremadura; county: Cáceres; locality: Peña Falcón; verbatimElevation: 320.6; decimalLatitude: 39.83296; decimalLongitude: -6.0641; geodeticDatum: WGS84; **Event:** eventID: 1; samplingProtocol: Aerial; eventTime: Night
- **Type status:** Other material. **Occurrence:** individualCount: 1; sex: male; **Location:** locationID: M1; continent: Europe; country: Spain; countryCode: ES; stateProvince: Extremadura; county: Cáceres; locality: Peña Falcón; verbatimElevation: 320.6; decimalLatitude: 39.83296; decimalLongitude: -6.0641; geodeticDatum: WGS84; **Event:** eventID: 4; samplingProtocol: Aerial; eventTime: Night
- **Type status:** Other material. **Occurrence:** individualCount: 1; sex: male; **Location:** locationID: M1; continent: Europe; country: Spain; countryCode: ES; stateProvince: Extremadura; county: Cáceres; locality: Peña Falcón; verbatimElevation: 320.6; decimalLatitude: 39.83296; decimalLongitude: -6.0641; geodeticDatum: WGS84; **Event:** eventID: 3; samplingProtocol: Aerial; eventTime: Night
- **Type status:** Other material. **Occurrence:** individualCount: 2; sex: male; **Location:** locationID: M1; continent: Europe; country: Spain; countryCode: ES; stateProvince: Extremadura; county: Cáceres; locality: Peña Falcón; verbatimElevation: 320.6; decimalLatitude: 39.83296; decimalLongitude: -6.0641; geodeticDatum: WGS84; **Event:** eventID: 2; samplingProtocol: Beating; eventTime: Night
- **Type status:** Other material. **Occurrence:** individualCount: 1; sex: female; **Location:** locationID: M1; continent: Europe; country: Spain; countryCode: ES; stateProvince: Extremadura; county: Cáceres; locality: Peña Falcón; verbatimElevation: 320.6; decimalLatitude: 39.83296; decimalLongitude: -6.0641; geodeticDatum: WGS84; **Event:** eventID: 2; samplingProtocol: Beating; eventTime: Day
- **Type status:** Other material. **Occurrence:** individualCount: 1; sex: male; **Location:** locationID: M2; continent: Europe; country: Spain; countryCode: ES; stateProvince: Extremadura; county: Cáceres; locality: Fuente del Frances; verbatimElevation: 320.72; decimalLatitude: 39.828; decimalLongitude: -6.03249; geodeticDatum: WGS84; **Event:** eventID: 1; samplingProtocol: Aerial; eventTime: Night
- **Type status:** Other material. **Occurrence:** individualCount: 1; sex: female; **Location:** locationID: M2; continent: Europe; country: Spain; countryCode: ES; stateProvince: Extremadura; county: Cáceres; locality: Fuente del Frances; verbatimElevation: 320.72; decimalLatitude: 39.828; decimalLongitude: -6.03249; geodeticDatum: WGS84; **Event:** eventID: 1; samplingProtocol: Beating; eventTime: Night

##### Distribution

Iberian Peninsula, France, Italy, Northwest Africa

#### Episinus maculipes

Cavanna, 1876

##### Materials

- **Type status:** Other material. **Occurrence:** individualCount: 1; sex: male; **Location:** locationID: A1; continent: Europe; country: Spain; countryCode: ES; stateProvince: Catalonia; county: Lleida; locality: Sola de Boi; verbatimElevation: 1759.8; decimalLatitude: 42.54958; decimalLongitude: 0.87254; geodeticDatum: WGS84; **Event:** eventID: 2; samplingProtocol: Aerial; eventTime: Night
- **Type status:** Other material. **Occurrence:** individualCount: 1; sex: male; **Location:** locationID: A1; continent: Europe; country: Spain; countryCode: ES; stateProvince: Catalonia; county: Lleida; locality: Sola de Boi; verbatimElevation: 1759.8; decimalLatitude: 42.54958; decimalLongitude: 0.87254; geodeticDatum: WGS84; **Event:** eventID: 2; samplingProtocol: Aerial; eventTime: Night
- **Type status:** Other material. **Occurrence:** individualCount: 1; sex: female; **Location:** locationID: O1; continent: Europe; country: Spain; countryCode: ES; stateProvince: Aragón; county: Huesca; locality: O Furno; verbatimElevation: 1396.73; decimalLatitude: 42.60677; decimalLongitude: 0.13135; geodeticDatum: WGS84; **Event:** eventID: 2; samplingProtocol: Aerial; eventTime: Night
- **Type status:** Other material. **Occurrence:** individualCount: 1; sex: female; **Location:** locationID: O2; continent: Europe; country: Spain; countryCode: ES; stateProvince: Aragón; county: Huesca; locality: Rebilla; verbatimElevation: 1158.13; decimalLatitude: 42.59427; decimalLongitude: 0.1529; geodeticDatum: WGS84; **Event:** eventID: 1; samplingProtocol: Aerial; eventTime: Night
- **Type status:** Other material. **Occurrence:** individualCount: 1; sex: female; **Location:** locationID: O2; continent: Europe; country: Spain; countryCode: ES; stateProvince: Aragón; county: Huesca; locality: Rebilla; verbatimElevation: 1158.13; decimalLatitude: 42.59427; decimalLongitude: 0.1529; geodeticDatum: WGS84; **Event:** eventID: 2; samplingProtocol: Aerial; eventTime: Night
- **Type status:** Other material. **Occurrence:** individualCount: 1; sex: female; **Location:** locationID: O2; continent: Europe; country: Spain; countryCode: ES; stateProvince: Aragón; county: Huesca; locality: Rebilla; verbatimElevation: 1158.13; decimalLatitude: 42.59427; decimalLongitude: 0.1529; geodeticDatum: WGS84; **Event:** eventID: 2; samplingProtocol: Aerial; eventTime: Night
- **Type status:** Other material. **Occurrence:** individualCount: 1; sex: male; **Location:** locationID: O2; continent: Europe; country: Spain; countryCode: ES; stateProvince: Aragón; county: Huesca; locality: Rebilla; verbatimElevation: 1158.13; decimalLatitude: 42.59427; decimalLongitude: 0.1529; geodeticDatum: WGS84; **Event:** eventID: 2; samplingProtocol: Aerial; eventTime: Night

##### Distribution

Great Britain to Algeria, Ukraine, Russia

#### Episinus theridioides

Simon, 1873

##### Materials

- **Type status:** Other material. **Occurrence:** individualCount: 1; sex: female; **Location:** locationID: P1; continent: Europe; country: Spain; countryCode: ES; stateProvince: Castilla y León; county: León; locality: Monte Robledo; verbatimElevation: 1071.58; decimalLatitude: 43.1445; decimalLongitude: -4.92675; geodeticDatum: WGS84; **Event:** eventID: 1; samplingProtocol: Sweeping; eventTime: Night
- **Type status:** Other material. **Occurrence:** individualCount: 1; sex: male; **Location:** locationID: P1; continent: Europe; country: Spain; countryCode: ES; stateProvince: Castilla y León; county: León; locality: Monte Robledo; verbatimElevation: 1071.58; decimalLatitude: 43.1445; decimalLongitude: -4.92675; geodeticDatum: WGS84; **Event:** eventID: 1; samplingProtocol: Sweeping; eventTime: Night
- **Type status:** Other material. **Occurrence:** individualCount: 1; sex: female; **Location:** locationID: P2; continent: Europe; country: Spain; countryCode: ES; stateProvince: Castilla y León; county: León; locality: Joyoguelas; verbatimElevation: 763.98; decimalLatitude: 43.17771; decimalLongitude: -4.90579; geodeticDatum: WGS84; **Event:** eventID: 2; samplingProtocol: Aerial; eventTime: Night
- **Type status:** Other material. **Occurrence:** individualCount: 1; sex: female; **Location:** locationID: P2; continent: Europe; country: Spain; countryCode: ES; stateProvince: Castilla y León; county: León; locality: Joyoguelas; verbatimElevation: 763.98; decimalLatitude: 43.17771; decimalLongitude: -4.90579; geodeticDatum: WGS84; **Event:** eventID: 1; samplingProtocol: Sweeping; eventTime: Night
- **Type status:** Other material. **Occurrence:** individualCount: 1; sex: male; **Location:** locationID: P4; continent: Europe; country: Spain; countryCode: ES; stateProvince: Castilla y León; county: León; locality: El Canto; verbatimElevation: 943.48; decimalLatitude: 43.17227; decimalLongitude: -4.90857; geodeticDatum: WGS84; **Event:** eventID: 1; samplingProtocol: Aerial; eventTime: Night
- **Type status:** Other material. **Occurrence:** individualCount: 1; sex: male; **Location:** locationID: P4; continent: Europe; country: Spain; countryCode: ES; stateProvince: Castilla y León; county: León; locality: El Canto; verbatimElevation: 943.48; decimalLatitude: 43.17227; decimalLongitude: -4.90857; geodeticDatum: WGS84; **Event:** eventID: 1; samplingProtocol: Aerial; eventTime: Night
- **Type status:** Other material. **Occurrence:** individualCount: 1; sex: female; **Location:** locationID: P4; continent: Europe; country: Spain; countryCode: ES; stateProvince: Castilla y León; county: León; locality: El Canto; verbatimElevation: 943.48; decimalLatitude: 43.17227; decimalLongitude: -4.90857; geodeticDatum: WGS84; **Event:** eventID: 2; samplingProtocol: Aerial; eventTime: Night

##### Distribution

Spain, France, Corsica, Sardinia

#### Episinus truncatus

Latreille, 1809

##### Materials

- **Type status:** Other material. **Occurrence:** individualCount: 1; sex: female; **Location:** locationID: A2; continent: Europe; country: Spain; countryCode: ES; stateProvince: Catalonia; county: Lleida; locality: Sola de Boi; verbatimElevation: 1738.7; decimalLatitude: 42.54913; decimalLongitude: 0.87137; geodeticDatum: WGS84; **Event:** eventID: 1; samplingProtocol: Aerial; eventTime: Night
- **Type status:** Other material. **Occurrence:** individualCount: 1; sex: female; **Location:** locationID: C1; continent: Europe; country: Spain; countryCode: ES; stateProvince: Castilla-La Mancha; county: Ciudad Real; locality: Valle Brezoso; verbatimElevation: 756.56; decimalLatitude: 39.35663; decimalLongitude: -4.35912; geodeticDatum: WGS84; **Event:** eventID: B; samplingProtocol: Pitfall
- **Type status:** Other material. **Occurrence:** individualCount: 1; sex: male; **Location:** locationID: O2; continent: Europe; country: Spain; countryCode: ES; stateProvince: Aragón; county: Huesca; locality: Rebilla; verbatimElevation: 1158.13; decimalLatitude: 42.59427; decimalLongitude: 0.1529; geodeticDatum: WGS84; **Event:** eventID: K; samplingProtocol: Pitfall

##### Distribution

Palearctic

#### Euryopis flavomaculata

(C. L. Koch, 1836)

##### Materials

- **Type status:** Other material. **Occurrence:** individualCount: 1; sex: male; **Location:** locationID: P2; continent: Europe; country: Spain; countryCode: ES; stateProvince: Castilla y León; county: León; locality: Joyoguelas; verbatimElevation: 763.98; decimalLatitude: 43.17771; decimalLongitude: -4.90579; geodeticDatum: WGS84; **Event:** eventID: B; samplingProtocol: Pitfall
- **Type status:** Other material. **Occurrence:** individualCount: 2; sex: male; **Location:** locationID: P2; continent: Europe; country: Spain; countryCode: ES; stateProvince: Castilla y León; county: León; locality: Joyoguelas; verbatimElevation: 763.98; decimalLatitude: 43.17771; decimalLongitude: -4.90579; geodeticDatum: WGS84; **Event:** eventID: D; samplingProtocol: Pitfall
- **Type status:** Other material. **Occurrence:** individualCount: 2; sex: male; **Location:** locationID: P2; continent: Europe; country: Spain; countryCode: ES; stateProvince: Castilla y León; county: León; locality: Joyoguelas; verbatimElevation: 763.98; decimalLatitude: 43.17771; decimalLongitude: -4.90579; geodeticDatum: WGS84; **Event:** eventID: F; samplingProtocol: Pitfall
- **Type status:** Other material. **Occurrence:** individualCount: 2; sex: male; **Location:** locationID: P2; continent: Europe; country: Spain; countryCode: ES; stateProvince: Castilla y León; county: León; locality: Joyoguelas; verbatimElevation: 763.98; decimalLatitude: 43.17771; decimalLongitude: -4.90579; geodeticDatum: WGS84; **Event:** eventID: G; samplingProtocol: Pitfall
- **Type status:** Other material. **Occurrence:** individualCount: 4; sex: male; **Location:** locationID: P2; continent: Europe; country: Spain; countryCode: ES; stateProvince: Castilla y León; county: León; locality: Joyoguelas; verbatimElevation: 763.98; decimalLatitude: 43.17771; decimalLongitude: -4.90579; geodeticDatum: WGS84; **Event:** eventID: H; samplingProtocol: Pitfall
- **Type status:** Other material. **Occurrence:** individualCount: 1; sex: male; **Location:** locationID: P2; continent: Europe; country: Spain; countryCode: ES; stateProvince: Castilla y León; county: León; locality: Joyoguelas; verbatimElevation: 763.98; decimalLatitude: 43.17771; decimalLongitude: -4.90579; geodeticDatum: WGS84; **Event:** eventID: I; samplingProtocol: Pitfall
- **Type status:** Other material. **Occurrence:** individualCount: 2; sex: male; **Location:** locationID: P2; continent: Europe; country: Spain; countryCode: ES; stateProvince: Castilla y León; county: León; locality: Joyoguelas; verbatimElevation: 763.98; decimalLatitude: 43.17771; decimalLongitude: -4.90579; geodeticDatum: WGS84; **Event:** eventID: J; samplingProtocol: Pitfall
- **Type status:** Other material. **Occurrence:** individualCount: 1; sex: male; **Location:** locationID: P3; continent: Europe; country: Spain; countryCode: ES; stateProvince: Castilla y León; county: León; locality: Las Arroyas; verbatimElevation: 1097.1; decimalLatitude: 43.14351; decimalLongitude: -4.94878; geodeticDatum: WGS84; **Event:** eventID: J; samplingProtocol: Pitfall
- **Type status:** Other material. **Occurrence:** individualCount: 1; sex: female; **Location:** locationID: P4; continent: Europe; country: Spain; countryCode: ES; stateProvince: Castilla y León; county: León; locality: El Canto; verbatimElevation: 943.48; decimalLatitude: 43.17227; decimalLongitude: -4.90857; geodeticDatum: WGS84; **Event:** eventID: A; samplingProtocol: Pitfall
- **Type status:** Other material. **Occurrence:** individualCount: 5; sex: male; **Location:** locationID: P4; continent: Europe; country: Spain; countryCode: ES; stateProvince: Castilla y León; county: León; locality: El Canto; verbatimElevation: 943.48; decimalLatitude: 43.17227; decimalLongitude: -4.90857; geodeticDatum: WGS84; **Event:** eventID: A; samplingProtocol: Pitfall
- **Type status:** Other material. **Occurrence:** individualCount: 4; sex: male; **Location:** locationID: P4; continent: Europe; country: Spain; countryCode: ES; stateProvince: Castilla y León; county: León; locality: El Canto; verbatimElevation: 943.48; decimalLatitude: 43.17227; decimalLongitude: -4.90857; geodeticDatum: WGS84; **Event:** eventID: B; samplingProtocol: Pitfall
- **Type status:** Other material. **Occurrence:** individualCount: 1; sex: male; **Location:** locationID: P4; continent: Europe; country: Spain; countryCode: ES; stateProvince: Castilla y León; county: León; locality: El Canto; verbatimElevation: 943.48; decimalLatitude: 43.17227; decimalLongitude: -4.90857; geodeticDatum: WGS84; **Event:** eventID: C; samplingProtocol: Pitfall
- **Type status:** Other material. **Occurrence:** individualCount: 8; sex: male; **Location:** locationID: P4; continent: Europe; country: Spain; countryCode: ES; stateProvince: Castilla y León; county: León; locality: El Canto; verbatimElevation: 943.48; decimalLatitude: 43.17227; decimalLongitude: -4.90857; geodeticDatum: WGS84; **Event:** eventID: E; samplingProtocol: Pitfall
- **Type status:** Other material. **Occurrence:** individualCount: 8; sex: male; **Location:** locationID: P4; continent: Europe; country: Spain; countryCode: ES; stateProvince: Castilla y León; county: León; locality: El Canto; verbatimElevation: 943.48; decimalLatitude: 43.17227; decimalLongitude: -4.90857; geodeticDatum: WGS84; **Event:** eventID: F; samplingProtocol: Pitfall
- **Type status:** Other material. **Occurrence:** individualCount: 2; sex: male; **Location:** locationID: P4; continent: Europe; country: Spain; countryCode: ES; stateProvince: Castilla y León; county: León; locality: El Canto; verbatimElevation: 943.48; decimalLatitude: 43.17227; decimalLongitude: -4.90857; geodeticDatum: WGS84; **Event:** eventID: G; samplingProtocol: Pitfall
- **Type status:** Other material. **Occurrence:** individualCount: 3; sex: male; **Location:** locationID: P4; continent: Europe; country: Spain; countryCode: ES; stateProvince: Castilla y León; county: León; locality: El Canto; verbatimElevation: 943.48; decimalLatitude: 43.17227; decimalLongitude: -4.90857; geodeticDatum: WGS84; **Event:** eventID: H; samplingProtocol: Pitfall
- **Type status:** Other material. **Occurrence:** individualCount: 5; sex: male; **Location:** locationID: P4; continent: Europe; country: Spain; countryCode: ES; stateProvince: Castilla y León; county: León; locality: El Canto; verbatimElevation: 943.48; decimalLatitude: 43.17227; decimalLongitude: -4.90857; geodeticDatum: WGS84; **Event:** eventID: I; samplingProtocol: Pitfall
- **Type status:** Other material. **Occurrence:** individualCount: 3; sex: male; **Location:** locationID: P4; continent: Europe; country: Spain; countryCode: ES; stateProvince: Castilla y León; county: León; locality: El Canto; verbatimElevation: 943.48; decimalLatitude: 43.17227; decimalLongitude: -4.90857; geodeticDatum: WGS84; **Event:** eventID: J; samplingProtocol: Pitfall
- **Type status:** Other material. **Occurrence:** individualCount: 1; sex: male; **Location:** locationID: P4; continent: Europe; country: Spain; countryCode: ES; stateProvince: Castilla y León; county: León; locality: El Canto; verbatimElevation: 943.48; decimalLatitude: 43.17227; decimalLongitude: -4.90857; geodeticDatum: WGS84; **Event:** eventID: L; samplingProtocol: Pitfall

##### Distribution

Palearctic

##### Notes

**Figure 13.**
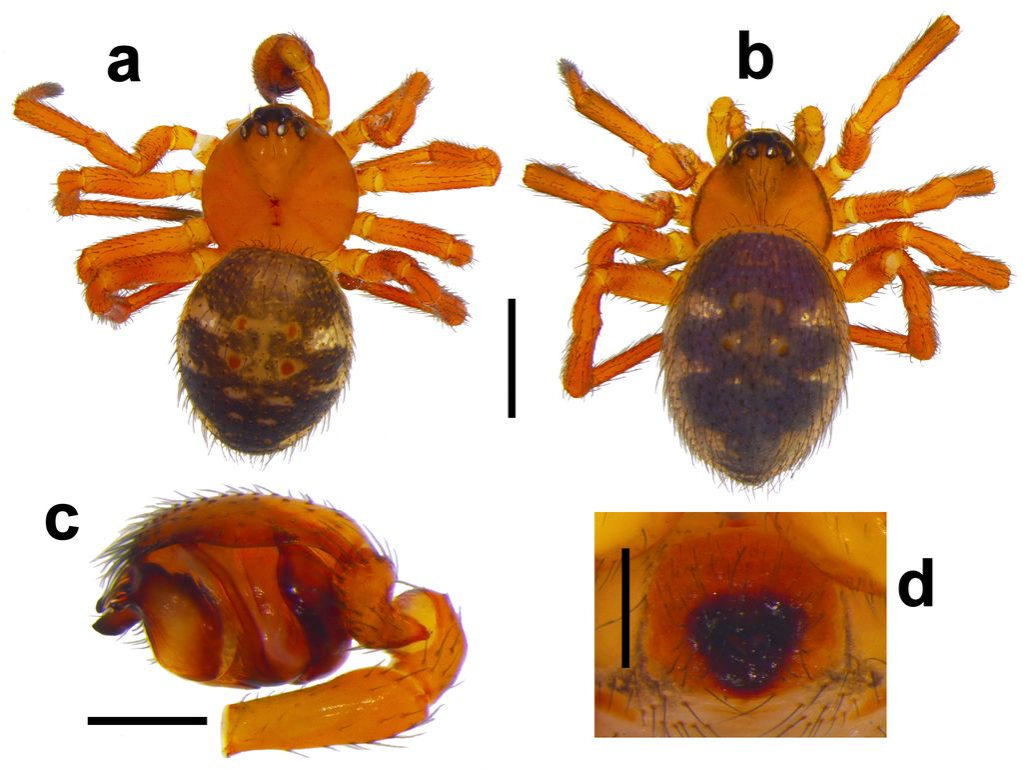
*Euryopis flavomaculata* (C. L. Koch, 1836). a: male, dorsal habitus, scale = 1 mm; b: female, dorsal habitus, scale = 1 mm; c: left male palp, retrolateral, scale = 0.3 mm; d: female epigynum, ventral, scale = 0.3 mm.

First record for the Iberian Peninsula. See Fig. 13.

#### Euryopis sexalbomaculata

(Lucas, 1846)

##### Materials

- **Type status:** Other material. **Occurrence:** individualCount: 1; sex: male; **Location:** locationID: C3; continent: Europe; country: Spain; countryCode: ES; stateProvince: Castilla-La Mancha; county: Ciudad Real; locality: La Quesera; verbatimElevation: 767.55; decimalLatitude: 39.36177; decimalLongitude: -4.41733; geodeticDatum: WGS84; **Event:** eventID: H; samplingProtocol: Pitfall
- **Type status:** Other material. **Occurrence:** individualCount: 1; sex: male; **Location:** locationID: M2; continent: Europe; country: Spain; countryCode: ES; stateProvince: Extremadura; county: Cáceres; locality: Fuente del Frances; verbatimElevation: 320.72; decimalLatitude: 39.828; decimalLongitude: -6.03249; geodeticDatum: WGS84; **Event:** eventID: A; samplingProtocol: Pitfall
- **Type status:** Other material. **Occurrence:** individualCount: 2; sex: male; **Location:** locationID: S1; continent: Europe; country: Spain; countryCode: ES; stateProvince: Andalucía; county: Granada; locality: Soportujar; verbatimElevation: 1786.57; decimalLatitude: 36.96151; decimalLongitude: -3.41881; geodeticDatum: WGS84; **Event:** eventID: L; samplingProtocol: Pitfall
- **Type status:** Other material. **Occurrence:** individualCount: 1; sex: female; **Location:** locationID: S2; continent: Europe; country: Spain; countryCode: ES; stateProvince: Andalucía; county: Granada; locality: Camarate; verbatimElevation: 1713.96; decimalLatitude: 37.18377; decimalLongitude: -3.26282; geodeticDatum: WGS84; **Event:** eventID: A; samplingProtocol: Pitfall
- **Type status:** Other material. **Occurrence:** individualCount: 1; sex: male; **Location:** locationID: S2; continent: Europe; country: Spain; countryCode: ES; stateProvince: Andalucía; county: Granada; locality: Camarate; verbatimElevation: 1713.96; decimalLatitude: 37.18377; decimalLongitude: -3.26282; geodeticDatum: WGS84; **Event:** eventID: A; samplingProtocol: Pitfall
- **Type status:** Other material. **Occurrence:** individualCount: 2; sex: male; **Location:** locationID: S2; continent: Europe; country: Spain; countryCode: ES; stateProvince: Andalucía; county: Granada; locality: Camarate; verbatimElevation: 1713.96; decimalLatitude: 37.18377; decimalLongitude: -3.26282; geodeticDatum: WGS84; **Event:** eventID: C; samplingProtocol: Pitfall
- **Type status:** Other material. **Occurrence:** individualCount: 1; sex: male; **Location:** locationID: S2; continent: Europe; country: Spain; countryCode: ES; stateProvince: Andalucía; county: Granada; locality: Camarate; verbatimElevation: 1713.96; decimalLatitude: 37.18377; decimalLongitude: -3.26282; geodeticDatum: WGS84; **Event:** eventID: E; samplingProtocol: Pitfall

##### Distribution

Mediterranean, Ukraine, Russia

##### Notes

**Figure 14.**
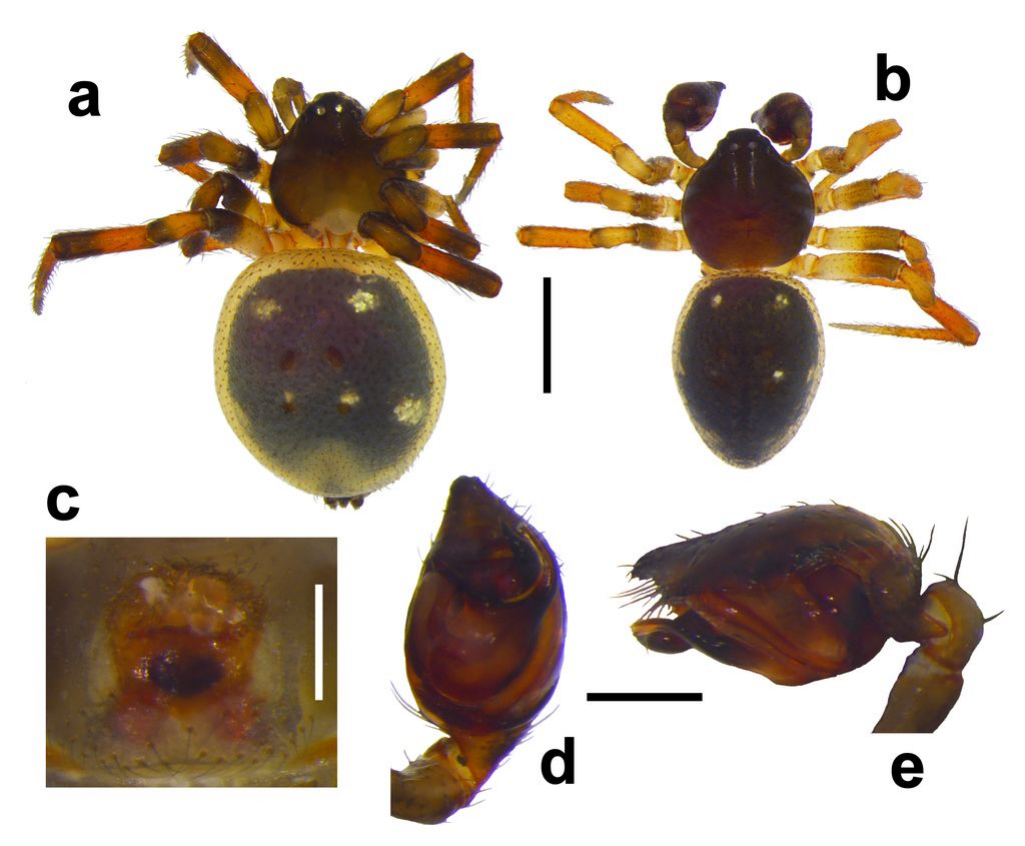
*Euryopis sexalbomaculata* (Lucas, 1846). a: female, dorsal habitus, scale = 0.8 mm; b: male, dorsal habitus, scale = 0.8 mm; c: female epigynum, ventral, scale = 0.2 mm; d: left male palp, ventral, scale = 0.2 mm; e: left male palp, retrolateral, scale = 0.2 mm.

First record for the Iberian Peninsula. See Fig. 14.

#### Heterotheridion nigrovariegatum

(Simon, 1873)

##### Materials

- **Type status:** Other material. **Occurrence:** individualCount: 1; sex: male; **Location:** locationID: O2; continent: Europe; country: Spain; countryCode: ES; stateProvince: Aragón; county: Huesca; locality: Rebilla; verbatimElevation: 1158.13; decimalLatitude: 42.59427; decimalLongitude: 0.1529; geodeticDatum: WGS84; **Event:** eventID: 1; samplingProtocol: Aerial; eventTime: Night
- **Type status:** Other material. **Occurrence:** individualCount: 1; sex: male; **Location:** locationID: O2; continent: Europe; country: Spain; countryCode: ES; stateProvince: Aragón; county: Huesca; locality: Rebilla; verbatimElevation: 1158.13; decimalLatitude: 42.59427; decimalLongitude: 0.1529; geodeticDatum: WGS84; **Event:** eventID: 2; samplingProtocol: Aerial; eventTime: Night
- **Type status:** Other material. **Occurrence:** individualCount: 2; sex: female; **Location:** locationID: O2; continent: Europe; country: Spain; countryCode: ES; stateProvince: Aragón; county: Huesca; locality: Rebilla; verbatimElevation: 1158.13; decimalLatitude: 42.59427; decimalLongitude: 0.1529; geodeticDatum: WGS84; **Event:** eventID: 1; samplingProtocol: Beating; eventTime: Day
- **Type status:** Other material. **Occurrence:** individualCount: 2; sex: male; **Location:** locationID: O2; continent: Europe; country: Spain; countryCode: ES; stateProvince: Aragón; county: Huesca; locality: Rebilla; verbatimElevation: 1158.13; decimalLatitude: 42.59427; decimalLongitude: 0.1529; geodeticDatum: WGS84; **Event:** eventID: 1; samplingProtocol: Beating; eventTime: Day
- **Type status:** Other material. **Occurrence:** individualCount: 1; sex: female; **Location:** locationID: O2; continent: Europe; country: Spain; countryCode: ES; stateProvince: Aragón; county: Huesca; locality: Rebilla; verbatimElevation: 1158.13; decimalLatitude: 42.59427; decimalLongitude: 0.1529; geodeticDatum: WGS84; **Event:** eventID: 1; samplingProtocol: Beating; eventTime: Night
- **Type status:** Other material. **Occurrence:** individualCount: 1; sex: male; **Location:** locationID: O2; continent: Europe; country: Spain; countryCode: ES; stateProvince: Aragón; county: Huesca; locality: Rebilla; verbatimElevation: 1158.13; decimalLatitude: 42.59427; decimalLongitude: 0.1529; geodeticDatum: WGS84; **Event:** eventID: 1; samplingProtocol: Beating; eventTime: Night
- **Type status:** Other material. **Occurrence:** individualCount: 1; sex: male; **Location:** locationID: O2; continent: Europe; country: Spain; countryCode: ES; stateProvince: Aragón; county: Huesca; locality: Rebilla; verbatimElevation: 1158.13; decimalLatitude: 42.59427; decimalLongitude: 0.1529; geodeticDatum: WGS84; **Event:** eventID: 1; samplingProtocol: Sweeping; eventTime: Night
- **Type status:** Other material. **Occurrence:** individualCount: 1; sex: female; **Location:** locationID: O2; continent: Europe; country: Spain; countryCode: ES; stateProvince: Aragón; county: Huesca; locality: Rebilla; verbatimElevation: 1158.13; decimalLatitude: 42.59427; decimalLongitude: 0.1529; geodeticDatum: WGS84; **Event:** eventID: 2; samplingProtocol: Sweeping; eventTime: Day
- **Type status:** Other material. **Occurrence:** individualCount: 2; sex: male; **Location:** locationID: O2; continent: Europe; country: Spain; countryCode: ES; stateProvince: Aragón; county: Huesca; locality: Rebilla; verbatimElevation: 1158.13; decimalLatitude: 42.59427; decimalLongitude: 0.1529; geodeticDatum: WGS84; **Event:** eventID: 2; samplingProtocol: Sweeping; eventTime: Day
- **Type status:** Other material. **Occurrence:** individualCount: 4; sex: female; **Location:** locationID: S1; continent: Europe; country: Spain; countryCode: ES; stateProvince: Andalucía; county: Granada; locality: Soportujar; verbatimElevation: 1786.57; decimalLatitude: 36.96151; decimalLongitude: -3.41881; geodeticDatum: WGS84; **Event:** eventID: 1; samplingProtocol: Aerial; eventTime: Night
- **Type status:** Other material. **Occurrence:** individualCount: 2; sex: male; **Location:** locationID: S1; continent: Europe; country: Spain; countryCode: ES; stateProvince: Andalucía; county: Granada; locality: Soportujar; verbatimElevation: 1786.57; decimalLatitude: 36.96151; decimalLongitude: -3.41881; geodeticDatum: WGS84; **Event:** eventID: 1; samplingProtocol: Aerial; eventTime: Night
- **Type status:** Other material. **Occurrence:** individualCount: 2; sex: female; **Location:** locationID: S1; continent: Europe; country: Spain; countryCode: ES; stateProvince: Andalucía; county: Granada; locality: Soportujar; verbatimElevation: 1786.57; decimalLatitude: 36.96151; decimalLongitude: -3.41881; geodeticDatum: WGS84; **Event:** eventID: 2; samplingProtocol: Aerial; eventTime: Night
- **Type status:** Other material. **Occurrence:** individualCount: 1; sex: male; **Location:** locationID: S1; continent: Europe; country: Spain; countryCode: ES; stateProvince: Andalucía; county: Granada; locality: Soportujar; verbatimElevation: 1786.57; decimalLatitude: 36.96151; decimalLongitude: -3.41881; geodeticDatum: WGS84; **Event:** eventID: 2; samplingProtocol: Aerial; eventTime: Night
- **Type status:** Other material. **Occurrence:** individualCount: 1; sex: female; **Location:** locationID: S1; continent: Europe; country: Spain; countryCode: ES; stateProvince: Andalucía; county: Granada; locality: Soportujar; verbatimElevation: 1786.57; decimalLatitude: 36.96151; decimalLongitude: -3.41881; geodeticDatum: WGS84; **Event:** eventID: 3; samplingProtocol: Aerial; eventTime: Night
- **Type status:** Other material. **Occurrence:** individualCount: 2; sex: male; **Location:** locationID: S1; continent: Europe; country: Spain; countryCode: ES; stateProvince: Andalucía; county: Granada; locality: Soportujar; verbatimElevation: 1786.57; decimalLatitude: 36.96151; decimalLongitude: -3.41881; geodeticDatum: WGS84; **Event:** eventID: 3; samplingProtocol: Aerial; eventTime: Night
- **Type status:** Other material. **Occurrence:** individualCount: 1; sex: female; **Location:** locationID: S1; continent: Europe; country: Spain; countryCode: ES; stateProvince: Andalucía; county: Granada; locality: Soportujar; verbatimElevation: 1786.57; decimalLatitude: 36.96151; decimalLongitude: -3.41881; geodeticDatum: WGS84; **Event:** eventID: 4; samplingProtocol: Aerial; eventTime: Night
- **Type status:** Other material. **Occurrence:** individualCount: 1; sex: female; **Location:** locationID: S1; continent: Europe; country: Spain; countryCode: ES; stateProvince: Andalucía; county: Granada; locality: Soportujar; verbatimElevation: 1786.57; decimalLatitude: 36.96151; decimalLongitude: -3.41881; geodeticDatum: WGS84; **Event:** eventID: 1; samplingProtocol: Beating; eventTime: Day
- **Type status:** Other material. **Occurrence:** individualCount: 7; sex: female; **Location:** locationID: S1; continent: Europe; country: Spain; countryCode: ES; stateProvince: Andalucía; county: Granada; locality: Soportujar; verbatimElevation: 1786.57; decimalLatitude: 36.96151; decimalLongitude: -3.41881; geodeticDatum: WGS84; **Event:** eventID: 2; samplingProtocol: Beating; eventTime: Day
- **Type status:** Other material. **Occurrence:** individualCount: 10; sex: male; **Location:** locationID: S1; continent: Europe; country: Spain; countryCode: ES; stateProvince: Andalucía; county: Granada; locality: Soportujar; verbatimElevation: 1786.57; decimalLatitude: 36.96151; decimalLongitude: -3.41881; geodeticDatum: WGS84; **Event:** eventID: 2; samplingProtocol: Beating; eventTime: Day
- **Type status:** Other material. **Occurrence:** individualCount: 2; sex: female; **Location:** locationID: S1; continent: Europe; country: Spain; countryCode: ES; stateProvince: Andalucía; county: Granada; locality: Soportujar; verbatimElevation: 1786.57; decimalLatitude: 36.96151; decimalLongitude: -3.41881; geodeticDatum: WGS84; **Event:** eventID: 1; samplingProtocol: Beating; eventTime: Night
- **Type status:** Other material. **Occurrence:** individualCount: 2; sex: male; **Location:** locationID: S1; continent: Europe; country: Spain; countryCode: ES; stateProvince: Andalucía; county: Granada; locality: Soportujar; verbatimElevation: 1786.57; decimalLatitude: 36.96151; decimalLongitude: -3.41881; geodeticDatum: WGS84; **Event:** eventID: 1; samplingProtocol: Beating; eventTime: Night
- **Type status:** Other material. **Occurrence:** individualCount: 6; sex: female; **Location:** locationID: S1; continent: Europe; country: Spain; countryCode: ES; stateProvince: Andalucía; county: Granada; locality: Soportujar; verbatimElevation: 1786.57; decimalLatitude: 36.96151; decimalLongitude: -3.41881; geodeticDatum: WGS84; **Event:** eventID: 2; samplingProtocol: Beating; eventTime: Night
- **Type status:** Other material. **Occurrence:** individualCount: 5; sex: male; **Location:** locationID: S1; continent: Europe; country: Spain; countryCode: ES; stateProvince: Andalucía; county: Granada; locality: Soportujar; verbatimElevation: 1786.57; decimalLatitude: 36.96151; decimalLongitude: -3.41881; geodeticDatum: WGS84; **Event:** eventID: 2; samplingProtocol: Beating; eventTime: Night
- **Type status:** Other material. **Occurrence:** individualCount: 1; sex: male; **Location:** locationID: S1; continent: Europe; country: Spain; countryCode: ES; stateProvince: Andalucía; county: Granada; locality: Soportujar; verbatimElevation: 1786.57; decimalLatitude: 36.96151; decimalLongitude: -3.41881; geodeticDatum: WGS84; **Event:** eventID: 1; samplingProtocol: Sweeping; eventTime: Day
- **Type status:** Other material. **Occurrence:** individualCount: 1; sex: female; **Location:** locationID: S1; continent: Europe; country: Spain; countryCode: ES; stateProvince: Andalucía; county: Granada; locality: Soportujar; verbatimElevation: 1786.57; decimalLatitude: 36.96151; decimalLongitude: -3.41881; geodeticDatum: WGS84; **Event:** eventID: 2; samplingProtocol: Sweeping; eventTime: Day
- **Type status:** Other material. **Occurrence:** individualCount: 4; sex: male; **Location:** locationID: S1; continent: Europe; country: Spain; countryCode: ES; stateProvince: Andalucía; county: Granada; locality: Soportujar; verbatimElevation: 1786.57; decimalLatitude: 36.96151; decimalLongitude: -3.41881; geodeticDatum: WGS84; **Event:** eventID: 2; samplingProtocol: Sweeping; eventTime: Day
- **Type status:** Other material. **Occurrence:** individualCount: 1; sex: female; **Location:** locationID: S1; continent: Europe; country: Spain; countryCode: ES; stateProvince: Andalucía; county: Granada; locality: Soportujar; verbatimElevation: 1786.57; decimalLatitude: 36.96151; decimalLongitude: -3.41881; geodeticDatum: WGS84; **Event:** eventID: 1; samplingProtocol: Sweeping; eventTime: Night
- **Type status:** Other material. **Occurrence:** individualCount: 4; sex: male; **Location:** locationID: S1; continent: Europe; country: Spain; countryCode: ES; stateProvince: Andalucía; county: Granada; locality: Soportujar; verbatimElevation: 1786.57; decimalLatitude: 36.96151; decimalLongitude: -3.41881; geodeticDatum: WGS84; **Event:** eventID: 1; samplingProtocol: Sweeping; eventTime: Night
- **Type status:** Other material. **Occurrence:** individualCount: 1; sex: female; **Location:** locationID: S1; continent: Europe; country: Spain; countryCode: ES; stateProvince: Andalucía; county: Granada; locality: Soportujar; verbatimElevation: 1786.57; decimalLatitude: 36.96151; decimalLongitude: -3.41881; geodeticDatum: WGS84; **Event:** eventID: 2; samplingProtocol: Sweeping; eventTime: Night
- **Type status:** Other material. **Occurrence:** individualCount: 1; sex: male; **Location:** locationID: S1; continent: Europe; country: Spain; countryCode: ES; stateProvince: Andalucía; county: Granada; locality: Soportujar; verbatimElevation: 1786.57; decimalLatitude: 36.96151; decimalLongitude: -3.41881; geodeticDatum: WGS84; **Event:** eventID: 2; samplingProtocol: Sweeping; eventTime: Night
- **Type status:** Other material. **Occurrence:** individualCount: 1; sex: male; **Location:** locationID: S2; continent: Europe; country: Spain; countryCode: ES; stateProvince: Andalucía; county: Granada; locality: Camarate; verbatimElevation: 1713.96; decimalLatitude: 37.18377; decimalLongitude: -3.26282; geodeticDatum: WGS84; **Event:** eventID: 1; samplingProtocol: Aerial; eventTime: Night
- **Type status:** Other material. **Occurrence:** individualCount: 1; sex: male; **Location:** locationID: S2; continent: Europe; country: Spain; countryCode: ES; stateProvince: Andalucía; county: Granada; locality: Camarate; verbatimElevation: 1713.96; decimalLatitude: 37.18377; decimalLongitude: -3.26282; geodeticDatum: WGS84; **Event:** eventID: 2; samplingProtocol: Aerial; eventTime: Night
- **Type status:** Other material. **Occurrence:** individualCount: 1; sex: female; **Location:** locationID: S2; continent: Europe; country: Spain; countryCode: ES; stateProvince: Andalucía; county: Granada; locality: Camarate; verbatimElevation: 1713.96; decimalLatitude: 37.18377; decimalLongitude: -3.26282; geodeticDatum: WGS84; **Event:** eventID: 3; samplingProtocol: Aerial; eventTime: Night
- **Type status:** Other material. **Occurrence:** individualCount: 1; sex: male; **Location:** locationID: S2; continent: Europe; country: Spain; countryCode: ES; stateProvince: Andalucía; county: Granada; locality: Camarate; verbatimElevation: 1713.96; decimalLatitude: 37.18377; decimalLongitude: -3.26282; geodeticDatum: WGS84; **Event:** eventID: 3; samplingProtocol: Aerial; eventTime: Night
- **Type status:** Other material. **Occurrence:** individualCount: 1; sex: female; **Location:** locationID: S2; continent: Europe; country: Spain; countryCode: ES; stateProvince: Andalucía; county: Granada; locality: Camarate; verbatimElevation: 1713.96; decimalLatitude: 37.18377; decimalLongitude: -3.26282; geodeticDatum: WGS84; **Event:** eventID: 4; samplingProtocol: Aerial; eventTime: Night
- **Type status:** Other material. **Occurrence:** individualCount: 1; sex: male; **Location:** locationID: S2; continent: Europe; country: Spain; countryCode: ES; stateProvince: Andalucía; county: Granada; locality: Camarate; verbatimElevation: 1713.96; decimalLatitude: 37.18377; decimalLongitude: -3.26282; geodeticDatum: WGS84; **Event:** eventID: 4; samplingProtocol: Aerial; eventTime: Night
- **Type status:** Other material. **Occurrence:** individualCount: 1; sex: male; **Location:** locationID: S2; continent: Europe; country: Spain; countryCode: ES; stateProvince: Andalucía; county: Granada; locality: Camarate; verbatimElevation: 1713.96; decimalLatitude: 37.18377; decimalLongitude: -3.26282; geodeticDatum: WGS84; **Event:** eventID: 2; samplingProtocol: Beating; eventTime: Night
- **Type status:** Other material. **Occurrence:** individualCount: 1; sex: male; **Location:** locationID: S2; continent: Europe; country: Spain; countryCode: ES; stateProvince: Andalucía; county: Granada; locality: Camarate; verbatimElevation: 1713.96; decimalLatitude: 37.18377; decimalLongitude: -3.26282; geodeticDatum: WGS84; **Event:** eventID: J; samplingProtocol: Pitfall
- **Type status:** Other material. **Occurrence:** individualCount: 3; sex: female; **Location:** locationID: S2; continent: Europe; country: Spain; countryCode: ES; stateProvince: Andalucía; county: Granada; locality: Camarate; verbatimElevation: 1713.96; decimalLatitude: 37.18377; decimalLongitude: -3.26282; geodeticDatum: WGS84; **Event:** eventID: 1; samplingProtocol: Sweeping; eventTime: Day
- **Type status:** Other material. **Occurrence:** individualCount: 2; sex: male; **Location:** locationID: S2; continent: Europe; country: Spain; countryCode: ES; stateProvince: Andalucía; county: Granada; locality: Camarate; verbatimElevation: 1713.96; decimalLatitude: 37.18377; decimalLongitude: -3.26282; geodeticDatum: WGS84; **Event:** eventID: 1; samplingProtocol: Sweeping; eventTime: Day
- **Type status:** Other material. **Occurrence:** individualCount: 1; sex: female; **Location:** locationID: S2; continent: Europe; country: Spain; countryCode: ES; stateProvince: Andalucía; county: Granada; locality: Camarate; verbatimElevation: 1713.96; decimalLatitude: 37.18377; decimalLongitude: -3.26282; geodeticDatum: WGS84; **Event:** eventID: 2; samplingProtocol: Sweeping; eventTime: Day
- **Type status:** Other material. **Occurrence:** individualCount: 1; sex: male; **Location:** locationID: S2; continent: Europe; country: Spain; countryCode: ES; stateProvince: Andalucía; county: Granada; locality: Camarate; verbatimElevation: 1713.96; decimalLatitude: 37.18377; decimalLongitude: -3.26282; geodeticDatum: WGS84; **Event:** eventID: 2; samplingProtocol: Sweeping; eventTime: Day
- **Type status:** Other material. **Occurrence:** individualCount: 2; sex: male; **Location:** locationID: S2; continent: Europe; country: Spain; countryCode: ES; stateProvince: Andalucía; county: Granada; locality: Camarate; verbatimElevation: 1713.96; decimalLatitude: 37.18377; decimalLongitude: -3.26282; geodeticDatum: WGS84; **Event:** eventID: 1; samplingProtocol: Sweeping; eventTime: Night
- **Type status:** Other material. **Occurrence:** individualCount: 1; sex: male; **Location:** locationID: S2; continent: Europe; country: Spain; countryCode: ES; stateProvince: Andalucía; county: Granada; locality: Camarate; verbatimElevation: 1713.96; decimalLatitude: 37.18377; decimalLongitude: -3.26282; geodeticDatum: WGS84; **Event:** eventID: 2; samplingProtocol: Sweeping; eventTime: Night

##### Distribution

Palearctic

#### Kochiura aulica

(C. L. Koch, 1838)

##### Materials

- **Type status:** Other material. **Occurrence:** individualCount: 1; sex: male; **Location:** locationID: C3; continent: Europe; country: Spain; countryCode: ES; stateProvince: Castilla-La Mancha; county: Ciudad Real; locality: La Quesera; verbatimElevation: 767.55; decimalLatitude: 39.36177; decimalLongitude: -4.41733; geodeticDatum: WGS84; **Event:** eventID: 3; samplingProtocol: Aerial; eventTime: Night
- **Type status:** Other material. **Occurrence:** individualCount: 1; sex: female; **Location:** locationID: C3; continent: Europe; country: Spain; countryCode: ES; stateProvince: Castilla-La Mancha; county: Ciudad Real; locality: La Quesera; verbatimElevation: 767.55; decimalLatitude: 39.36177; decimalLongitude: -4.41733; geodeticDatum: WGS84; **Event:** eventID: 1; samplingProtocol: Beating; eventTime: Night
- **Type status:** Other material. **Occurrence:** individualCount: 1; sex: female; **Location:** locationID: C4; continent: Europe; country: Spain; countryCode: ES; stateProvince: Castilla-La Mancha; county: Ciudad Real; locality: La Quesera; verbatimElevation: 772.3; decimalLatitude: 39.36337; decimalLongitude: -4.41704; geodeticDatum: WGS84; **Event:** eventID: 2; samplingProtocol: Sweeping; eventTime: Day

##### Distribution

Canary Islands, Cape Verde Islands to Azerbaijan

#### Lasaeola convexa

(Blackwall, 1870)

##### Materials

- **Type status:** Other material. **Occurrence:** individualCount: 1; sex: male; **Location:** locationID: M1; continent: Europe; country: Spain; countryCode: ES; stateProvince: Extremadura; county: Cáceres; locality: Peña Falcón; verbatimElevation: 320.6; decimalLatitude: 39.83296; decimalLongitude: -6.0641; geodeticDatum: WGS84; **Event:** eventID: 1; samplingProtocol: Aerial; eventTime: Night
- **Type status:** Other material. **Occurrence:** individualCount: 4; sex: female; **Location:** locationID: M1; continent: Europe; country: Spain; countryCode: ES; stateProvince: Extremadura; county: Cáceres; locality: Peña Falcón; verbatimElevation: 320.6; decimalLatitude: 39.83296; decimalLongitude: -6.0641; geodeticDatum: WGS84; **Event:** eventID: 3; samplingProtocol: Aerial; eventTime: Night
- **Type status:** Other material. **Occurrence:** individualCount: 1; sex: male; **Location:** locationID: M1; continent: Europe; country: Spain; countryCode: ES; stateProvince: Extremadura; county: Cáceres; locality: Peña Falcón; verbatimElevation: 320.6; decimalLatitude: 39.83296; decimalLongitude: -6.0641; geodeticDatum: WGS84; **Event:** eventID: 3; samplingProtocol: Aerial; eventTime: Night
- **Type status:** Other material. **Occurrence:** individualCount: 1; sex: male; **Location:** locationID: M1; continent: Europe; country: Spain; countryCode: ES; stateProvince: Extremadura; county: Cáceres; locality: Peña Falcón; verbatimElevation: 320.6; decimalLatitude: 39.83296; decimalLongitude: -6.0641; geodeticDatum: WGS84; **Event:** eventID: 2; samplingProtocol: Beating; eventTime: Day
- **Type status:** Other material. **Occurrence:** individualCount: 1; sex: female; **Location:** locationID: M2; continent: Europe; country: Spain; countryCode: ES; stateProvince: Extremadura; county: Cáceres; locality: Fuente del Frances; verbatimElevation: 320.72; decimalLatitude: 39.828; decimalLongitude: -6.03249; geodeticDatum: WGS84; **Event:** eventID: 2; samplingProtocol: Aerial; eventTime: Night
- **Type status:** Other material. **Occurrence:** individualCount: 1; sex: male; **Location:** locationID: M2; continent: Europe; country: Spain; countryCode: ES; stateProvince: Extremadura; county: Cáceres; locality: Fuente del Frances; verbatimElevation: 320.72; decimalLatitude: 39.828; decimalLongitude: -6.03249; geodeticDatum: WGS84; **Event:** eventID: 2; samplingProtocol: Aerial; eventTime: Night
- **Type status:** Other material. **Occurrence:** individualCount: 2; sex: female; **Location:** locationID: M2; continent: Europe; country: Spain; countryCode: ES; stateProvince: Extremadura; county: Cáceres; locality: Fuente del Frances; verbatimElevation: 320.72; decimalLatitude: 39.828; decimalLongitude: -6.03249; geodeticDatum: WGS84; **Event:** eventID: 3; samplingProtocol: Aerial; eventTime: Night
- **Type status:** Other material. **Occurrence:** individualCount: 1; sex: male; **Location:** locationID: M2; continent: Europe; country: Spain; countryCode: ES; stateProvince: Extremadura; county: Cáceres; locality: Fuente del Frances; verbatimElevation: 320.72; decimalLatitude: 39.828; decimalLongitude: -6.03249; geodeticDatum: WGS84; **Event:** eventID: 3; samplingProtocol: Aerial; eventTime: Night
- **Type status:** Other material. **Occurrence:** individualCount: 3; sex: male; **Location:** locationID: M2; continent: Europe; country: Spain; countryCode: ES; stateProvince: Extremadura; county: Cáceres; locality: Fuente del Frances; verbatimElevation: 320.72; decimalLatitude: 39.828; decimalLongitude: -6.03249; geodeticDatum: WGS84; **Event:** eventID: 4; samplingProtocol: Aerial; eventTime: Night
- **Type status:** Other material. **Occurrence:** individualCount: 1; sex: female; **Location:** locationID: M2; continent: Europe; country: Spain; countryCode: ES; stateProvince: Extremadura; county: Cáceres; locality: Fuente del Frances; verbatimElevation: 320.72; decimalLatitude: 39.828; decimalLongitude: -6.03249; geodeticDatum: WGS84; **Event:** eventID: 1; samplingProtocol: Beating; eventTime: Night
- **Type status:** Other material. **Occurrence:** individualCount: 1; sex: male; **Location:** locationID: M2; continent: Europe; country: Spain; countryCode: ES; stateProvince: Extremadura; county: Cáceres; locality: Fuente del Frances; verbatimElevation: 320.72; decimalLatitude: 39.828; decimalLongitude: -6.03249; geodeticDatum: WGS84; **Event:** eventID: 1; samplingProtocol: Beating; eventTime: Night

##### Distribution

Mediterranean

#### Lasaeola tristis

(Hahn, 1833)

##### Materials

- **Type status:** Other material. **Occurrence:** individualCount: 9; sex: female; **Location:** locationID: C1; continent: Europe; country: Spain; countryCode: ES; stateProvince: Castilla-La Mancha; county: Ciudad Real; locality: Valle Brezoso; verbatimElevation: 756.56; decimalLatitude: 39.35663; decimalLongitude: -4.35912; geodeticDatum: WGS84; **Event:** eventID: 1; samplingProtocol: Aerial; eventTime: Night
- **Type status:** Other material. **Occurrence:** individualCount: 4; sex: male; **Location:** locationID: C1; continent: Europe; country: Spain; countryCode: ES; stateProvince: Castilla-La Mancha; county: Ciudad Real; locality: Valle Brezoso; verbatimElevation: 756.56; decimalLatitude: 39.35663; decimalLongitude: -4.35912; geodeticDatum: WGS84; **Event:** eventID: 1; samplingProtocol: Aerial; eventTime: Night
- **Type status:** Other material. **Occurrence:** individualCount: 1; sex: female; **Location:** locationID: C1; continent: Europe; country: Spain; countryCode: ES; stateProvince: Castilla-La Mancha; county: Ciudad Real; locality: Valle Brezoso; verbatimElevation: 756.56; decimalLatitude: 39.35663; decimalLongitude: -4.35912; geodeticDatum: WGS84; **Event:** eventID: 2; samplingProtocol: Aerial; eventTime: Night
- **Type status:** Other material. **Occurrence:** individualCount: 1; sex: male; **Location:** locationID: C1; continent: Europe; country: Spain; countryCode: ES; stateProvince: Castilla-La Mancha; county: Ciudad Real; locality: Valle Brezoso; verbatimElevation: 756.56; decimalLatitude: 39.35663; decimalLongitude: -4.35912; geodeticDatum: WGS84; **Event:** eventID: 2; samplingProtocol: Aerial; eventTime: Night
- **Type status:** Other material. **Occurrence:** individualCount: 10; sex: female; **Location:** locationID: C1; continent: Europe; country: Spain; countryCode: ES; stateProvince: Castilla-La Mancha; county: Ciudad Real; locality: Valle Brezoso; verbatimElevation: 756.56; decimalLatitude: 39.35663; decimalLongitude: -4.35912; geodeticDatum: WGS84; **Event:** eventID: 3; samplingProtocol: Aerial; eventTime: Night
- **Type status:** Other material. **Occurrence:** individualCount: 3; sex: male; **Location:** locationID: C1; continent: Europe; country: Spain; countryCode: ES; stateProvince: Castilla-La Mancha; county: Ciudad Real; locality: Valle Brezoso; verbatimElevation: 756.56; decimalLatitude: 39.35663; decimalLongitude: -4.35912; geodeticDatum: WGS84; **Event:** eventID: 3; samplingProtocol: Aerial; eventTime: Night
- **Type status:** Other material. **Occurrence:** individualCount: 5; sex: female; **Location:** locationID: C1; continent: Europe; country: Spain; countryCode: ES; stateProvince: Castilla-La Mancha; county: Ciudad Real; locality: Valle Brezoso; verbatimElevation: 756.56; decimalLatitude: 39.35663; decimalLongitude: -4.35912; geodeticDatum: WGS84; **Event:** eventID: 4; samplingProtocol: Aerial; eventTime: Night
- **Type status:** Other material. **Occurrence:** individualCount: 3; sex: male; **Location:** locationID: C1; continent: Europe; country: Spain; countryCode: ES; stateProvince: Castilla-La Mancha; county: Ciudad Real; locality: Valle Brezoso; verbatimElevation: 756.56; decimalLatitude: 39.35663; decimalLongitude: -4.35912; geodeticDatum: WGS84; **Event:** eventID: 4; samplingProtocol: Aerial; eventTime: Night
- **Type status:** Other material. **Occurrence:** individualCount: 5; sex: female; **Location:** locationID: C1; continent: Europe; country: Spain; countryCode: ES; stateProvince: Castilla-La Mancha; county: Ciudad Real; locality: Valle Brezoso; verbatimElevation: 756.56; decimalLatitude: 39.35663; decimalLongitude: -4.35912; geodeticDatum: WGS84; **Event:** eventID: 1; samplingProtocol: Beating; eventTime: Day
- **Type status:** Other material. **Occurrence:** individualCount: 1; sex: male; **Location:** locationID: C1; continent: Europe; country: Spain; countryCode: ES; stateProvince: Castilla-La Mancha; county: Ciudad Real; locality: Valle Brezoso; verbatimElevation: 756.56; decimalLatitude: 39.35663; decimalLongitude: -4.35912; geodeticDatum: WGS84; **Event:** eventID: 1; samplingProtocol: Beating; eventTime: Day
- **Type status:** Other material. **Occurrence:** individualCount: 15; sex: female; **Location:** locationID: C1; continent: Europe; country: Spain; countryCode: ES; stateProvince: Castilla-La Mancha; county: Ciudad Real; locality: Valle Brezoso; verbatimElevation: 756.56; decimalLatitude: 39.35663; decimalLongitude: -4.35912; geodeticDatum: WGS84; **Event:** eventID: 2; samplingProtocol: Beating; eventTime: Day
- **Type status:** Other material. **Occurrence:** individualCount: 8; sex: female; **Location:** locationID: C1; continent: Europe; country: Spain; countryCode: ES; stateProvince: Castilla-La Mancha; county: Ciudad Real; locality: Valle Brezoso; verbatimElevation: 756.56; decimalLatitude: 39.35663; decimalLongitude: -4.35912; geodeticDatum: WGS84; **Event:** eventID: 1; samplingProtocol: Beating; eventTime: Night
- **Type status:** Other material. **Occurrence:** individualCount: 1; sex: male; **Location:** locationID: C1; continent: Europe; country: Spain; countryCode: ES; stateProvince: Castilla-La Mancha; county: Ciudad Real; locality: Valle Brezoso; verbatimElevation: 756.56; decimalLatitude: 39.35663; decimalLongitude: -4.35912; geodeticDatum: WGS84; **Event:** eventID: 1; samplingProtocol: Beating; eventTime: Night
- **Type status:** Other material. **Occurrence:** individualCount: 13; sex: female; **Location:** locationID: C1; continent: Europe; country: Spain; countryCode: ES; stateProvince: Castilla-La Mancha; county: Ciudad Real; locality: Valle Brezoso; verbatimElevation: 756.56; decimalLatitude: 39.35663; decimalLongitude: -4.35912; geodeticDatum: WGS84; **Event:** eventID: 2; samplingProtocol: Beating; eventTime: Night
- **Type status:** Other material. **Occurrence:** individualCount: 2; sex: male; **Location:** locationID: C1; continent: Europe; country: Spain; countryCode: ES; stateProvince: Castilla-La Mancha; county: Ciudad Real; locality: Valle Brezoso; verbatimElevation: 756.56; decimalLatitude: 39.35663; decimalLongitude: -4.35912; geodeticDatum: WGS84; **Event:** eventID: 2; samplingProtocol: Beating; eventTime: Night
- **Type status:** Other material. **Occurrence:** individualCount: 1; sex: male; **Location:** locationID: C1; continent: Europe; country: Spain; countryCode: ES; stateProvince: Castilla-La Mancha; county: Ciudad Real; locality: Valle Brezoso; verbatimElevation: 756.56; decimalLatitude: 39.35663; decimalLongitude: -4.35912; geodeticDatum: WGS84; **Event:** eventID: 1; samplingProtocol: Sweeping; eventTime: Day
- **Type status:** Other material. **Occurrence:** individualCount: 6; sex: female; **Location:** locationID: C1; continent: Europe; country: Spain; countryCode: ES; stateProvince: Castilla-La Mancha; county: Ciudad Real; locality: Valle Brezoso; verbatimElevation: 756.56; decimalLatitude: 39.35663; decimalLongitude: -4.35912; geodeticDatum: WGS84; **Event:** eventID: 2; samplingProtocol: Sweeping; eventTime: Day
- **Type status:** Other material. **Occurrence:** individualCount: 1; sex: male; **Location:** locationID: C1; continent: Europe; country: Spain; countryCode: ES; stateProvince: Castilla-La Mancha; county: Ciudad Real; locality: Valle Brezoso; verbatimElevation: 756.56; decimalLatitude: 39.35663; decimalLongitude: -4.35912; geodeticDatum: WGS84; **Event:** eventID: 2; samplingProtocol: Sweeping; eventTime: Day
- **Type status:** Other material. **Occurrence:** individualCount: 12; sex: female; **Location:** locationID: C1; continent: Europe; country: Spain; countryCode: ES; stateProvince: Castilla-La Mancha; county: Ciudad Real; locality: Valle Brezoso; verbatimElevation: 756.56; decimalLatitude: 39.35663; decimalLongitude: -4.35912; geodeticDatum: WGS84; **Event:** eventID: 2; samplingProtocol: Sweeping; eventTime: Night
- **Type status:** Other material. **Occurrence:** individualCount: 3; sex: male; **Location:** locationID: C1; continent: Europe; country: Spain; countryCode: ES; stateProvince: Castilla-La Mancha; county: Ciudad Real; locality: Valle Brezoso; verbatimElevation: 756.56; decimalLatitude: 39.35663; decimalLongitude: -4.35912; geodeticDatum: WGS84; **Event:** eventID: 2; samplingProtocol: Sweeping; eventTime: Night
- **Type status:** Other material. **Occurrence:** individualCount: 4; sex: female; **Location:** locationID: C2; continent: Europe; country: Spain; countryCode: ES; stateProvince: Castilla-La Mancha; county: Ciudad Real; locality: Valle Brezoso; verbatimElevation: 739.31; decimalLatitude: 39.35159; decimalLongitude: -4.3589; geodeticDatum: WGS84; **Event:** eventID: 1; samplingProtocol: Aerial; eventTime: Night
- **Type status:** Other material. **Occurrence:** individualCount: 5; sex: female; **Location:** locationID: C2; continent: Europe; country: Spain; countryCode: ES; stateProvince: Castilla-La Mancha; county: Ciudad Real; locality: Valle Brezoso; verbatimElevation: 739.31; decimalLatitude: 39.35159; decimalLongitude: -4.3589; geodeticDatum: WGS84; **Event:** eventID: 2; samplingProtocol: Aerial; eventTime: Night
- **Type status:** Other material. **Occurrence:** individualCount: 1; sex: female; **Location:** locationID: C2; continent: Europe; country: Spain; countryCode: ES; stateProvince: Castilla-La Mancha; county: Ciudad Real; locality: Valle Brezoso; verbatimElevation: 739.31; decimalLatitude: 39.35159; decimalLongitude: -4.3589; geodeticDatum: WGS84; **Event:** eventID: 3; samplingProtocol: Aerial; eventTime: Night
- **Type status:** Other material. **Occurrence:** individualCount: 2; sex: female; **Location:** locationID: C2; continent: Europe; country: Spain; countryCode: ES; stateProvince: Castilla-La Mancha; county: Ciudad Real; locality: Valle Brezoso; verbatimElevation: 739.31; decimalLatitude: 39.35159; decimalLongitude: -4.3589; geodeticDatum: WGS84; **Event:** eventID: 4; samplingProtocol: Aerial; eventTime: Night
- **Type status:** Other material. **Occurrence:** individualCount: 3; sex: male; **Location:** locationID: C2; continent: Europe; country: Spain; countryCode: ES; stateProvince: Castilla-La Mancha; county: Ciudad Real; locality: Valle Brezoso; verbatimElevation: 739.31; decimalLatitude: 39.35159; decimalLongitude: -4.3589; geodeticDatum: WGS84; **Event:** eventID: 4; samplingProtocol: Aerial; eventTime: Night
- **Type status:** Other material. **Occurrence:** individualCount: 10; sex: female; **Location:** locationID: C2; continent: Europe; country: Spain; countryCode: ES; stateProvince: Castilla-La Mancha; county: Ciudad Real; locality: Valle Brezoso; verbatimElevation: 739.31; decimalLatitude: 39.35159; decimalLongitude: -4.3589; geodeticDatum: WGS84; **Event:** eventID: 1; samplingProtocol: Beating; eventTime: Day
- **Type status:** Other material. **Occurrence:** individualCount: 1; sex: male; **Location:** locationID: C2; continent: Europe; country: Spain; countryCode: ES; stateProvince: Castilla-La Mancha; county: Ciudad Real; locality: Valle Brezoso; verbatimElevation: 739.31; decimalLatitude: 39.35159; decimalLongitude: -4.3589; geodeticDatum: WGS84; **Event:** eventID: 1; samplingProtocol: Beating; eventTime: Day
- **Type status:** Other material. **Occurrence:** individualCount: 10; sex: female; **Location:** locationID: C2; continent: Europe; country: Spain; countryCode: ES; stateProvince: Castilla-La Mancha; county: Ciudad Real; locality: Valle Brezoso; verbatimElevation: 739.31; decimalLatitude: 39.35159; decimalLongitude: -4.3589; geodeticDatum: WGS84; **Event:** eventID: 2; samplingProtocol: Beating; eventTime: Day
- **Type status:** Other material. **Occurrence:** individualCount: 1; sex: male; **Location:** locationID: C2; continent: Europe; country: Spain; countryCode: ES; stateProvince: Castilla-La Mancha; county: Ciudad Real; locality: Valle Brezoso; verbatimElevation: 739.31; decimalLatitude: 39.35159; decimalLongitude: -4.3589; geodeticDatum: WGS84; **Event:** eventID: 2; samplingProtocol: Beating; eventTime: Day
- **Type status:** Other material. **Occurrence:** individualCount: 6; sex: female; **Location:** locationID: C2; continent: Europe; country: Spain; countryCode: ES; stateProvince: Castilla-La Mancha; county: Ciudad Real; locality: Valle Brezoso; verbatimElevation: 739.31; decimalLatitude: 39.35159; decimalLongitude: -4.3589; geodeticDatum: WGS84; **Event:** eventID: 1; samplingProtocol: Beating; eventTime: Night
- **Type status:** Other material. **Occurrence:** individualCount: 1; sex: male; **Location:** locationID: C2; continent: Europe; country: Spain; countryCode: ES; stateProvince: Castilla-La Mancha; county: Ciudad Real; locality: Valle Brezoso; verbatimElevation: 739.31; decimalLatitude: 39.35159; decimalLongitude: -4.3589; geodeticDatum: WGS84; **Event:** eventID: 1; samplingProtocol: Beating; eventTime: Night
- **Type status:** Other material. **Occurrence:** individualCount: 2; sex: female; **Location:** locationID: C2; continent: Europe; country: Spain; countryCode: ES; stateProvince: Castilla-La Mancha; county: Ciudad Real; locality: Valle Brezoso; verbatimElevation: 739.31; decimalLatitude: 39.35159; decimalLongitude: -4.3589; geodeticDatum: WGS84; **Event:** eventID: 2; samplingProtocol: Beating; eventTime: Night
- **Type status:** Other material. **Occurrence:** individualCount: 3; sex: female; **Location:** locationID: C2; continent: Europe; country: Spain; countryCode: ES; stateProvince: Castilla-La Mancha; county: Ciudad Real; locality: Valle Brezoso; verbatimElevation: 739.31; decimalLatitude: 39.35159; decimalLongitude: -4.3589; geodeticDatum: WGS84; **Event:** eventID: 1; samplingProtocol: Sweeping; eventTime: Day
- **Type status:** Other material. **Occurrence:** individualCount: 2; sex: female; **Location:** locationID: C2; continent: Europe; country: Spain; countryCode: ES; stateProvince: Castilla-La Mancha; county: Ciudad Real; locality: Valle Brezoso; verbatimElevation: 739.31; decimalLatitude: 39.35159; decimalLongitude: -4.3589; geodeticDatum: WGS84; **Event:** eventID: 2; samplingProtocol: Sweeping; eventTime: Day
- **Type status:** Other material. **Occurrence:** individualCount: 1; sex: male; **Location:** locationID: C2; continent: Europe; country: Spain; countryCode: ES; stateProvince: Castilla-La Mancha; county: Ciudad Real; locality: Valle Brezoso; verbatimElevation: 739.31; decimalLatitude: 39.35159; decimalLongitude: -4.3589; geodeticDatum: WGS84; **Event:** eventID: 1; samplingProtocol: Sweeping; eventTime: Night
- **Type status:** Other material. **Occurrence:** individualCount: 1; sex: female; **Location:** locationID: C3; continent: Europe; country: Spain; countryCode: ES; stateProvince: Castilla-La Mancha; county: Ciudad Real; locality: La Quesera; verbatimElevation: 767.55; decimalLatitude: 39.36177; decimalLongitude: -4.41733; geodeticDatum: WGS84; **Event:** eventID: 1; samplingProtocol: Aerial; eventTime: Night
- **Type status:** Other material. **Occurrence:** individualCount: 4; sex: female; **Location:** locationID: C3; continent: Europe; country: Spain; countryCode: ES; stateProvince: Castilla-La Mancha; county: Ciudad Real; locality: La Quesera; verbatimElevation: 767.55; decimalLatitude: 39.36177; decimalLongitude: -4.41733; geodeticDatum: WGS84; **Event:** eventID: 3; samplingProtocol: Aerial; eventTime: Night
- **Type status:** Other material. **Occurrence:** individualCount: 7; sex: female; **Location:** locationID: C3; continent: Europe; country: Spain; countryCode: ES; stateProvince: Castilla-La Mancha; county: Ciudad Real; locality: La Quesera; verbatimElevation: 767.55; decimalLatitude: 39.36177; decimalLongitude: -4.41733; geodeticDatum: WGS84; **Event:** eventID: 1; samplingProtocol: Beating; eventTime: Day
- **Type status:** Other material. **Occurrence:** individualCount: 1; sex: female; **Location:** locationID: C3; continent: Europe; country: Spain; countryCode: ES; stateProvince: Castilla-La Mancha; county: Ciudad Real; locality: La Quesera; verbatimElevation: 767.55; decimalLatitude: 39.36177; decimalLongitude: -4.41733; geodeticDatum: WGS84; **Event:** eventID: 2; samplingProtocol: Beating; eventTime: Day
- **Type status:** Other material. **Occurrence:** individualCount: 2; sex: female; **Location:** locationID: C3; continent: Europe; country: Spain; countryCode: ES; stateProvince: Castilla-La Mancha; county: Ciudad Real; locality: La Quesera; verbatimElevation: 767.55; decimalLatitude: 39.36177; decimalLongitude: -4.41733; geodeticDatum: WGS84; **Event:** eventID: 2; samplingProtocol: Beating; eventTime: Night
- **Type status:** Other material. **Occurrence:** individualCount: 1; sex: female; **Location:** locationID: C4; continent: Europe; country: Spain; countryCode: ES; stateProvince: Castilla-La Mancha; county: Ciudad Real; locality: La Quesera; verbatimElevation: 772.3; decimalLatitude: 39.36337; decimalLongitude: -4.41704; geodeticDatum: WGS84; **Event:** eventID: 1; samplingProtocol: Aerial; eventTime: Night
- **Type status:** Other material. **Occurrence:** individualCount: 4; sex: female; **Location:** locationID: C4; continent: Europe; country: Spain; countryCode: ES; stateProvince: Castilla-La Mancha; county: Ciudad Real; locality: La Quesera; verbatimElevation: 772.3; decimalLatitude: 39.36337; decimalLongitude: -4.41704; geodeticDatum: WGS84; **Event:** eventID: 4; samplingProtocol: Aerial; eventTime: Night
- **Type status:** Other material. **Occurrence:** individualCount: 3; sex: female; **Location:** locationID: C4; continent: Europe; country: Spain; countryCode: ES; stateProvince: Castilla-La Mancha; county: Ciudad Real; locality: La Quesera; verbatimElevation: 772.3; decimalLatitude: 39.36337; decimalLongitude: -4.41704; geodeticDatum: WGS84; **Event:** eventID: 1; samplingProtocol: Beating; eventTime: Day
- **Type status:** Other material. **Occurrence:** individualCount: 1; sex: female; **Location:** locationID: C4; continent: Europe; country: Spain; countryCode: ES; stateProvince: Castilla-La Mancha; county: Ciudad Real; locality: La Quesera; verbatimElevation: 772.3; decimalLatitude: 39.36337; decimalLongitude: -4.41704; geodeticDatum: WGS84; **Event:** eventID: 2; samplingProtocol: Beating; eventTime: Night
- **Type status:** Other material. **Occurrence:** individualCount: 1; sex: male; **Location:** locationID: C4; continent: Europe; country: Spain; countryCode: ES; stateProvince: Castilla-La Mancha; county: Ciudad Real; locality: La Quesera; verbatimElevation: 772.3; decimalLatitude: 39.36337; decimalLongitude: -4.41704; geodeticDatum: WGS84; **Event:** eventID: 2; samplingProtocol: Beating; eventTime: Night
- **Type status:** Other material. **Occurrence:** individualCount: 3; sex: female; **Location:** locationID: M1; continent: Europe; country: Spain; countryCode: ES; stateProvince: Extremadura; county: Cáceres; locality: Peña Falcón; verbatimElevation: 320.6; decimalLatitude: 39.83296; decimalLongitude: -6.0641; geodeticDatum: WGS84; **Event:** eventID: 1; samplingProtocol: Aerial; eventTime: Night
- **Type status:** Other material. **Occurrence:** individualCount: 1; sex: female; **Location:** locationID: M1; continent: Europe; country: Spain; countryCode: ES; stateProvince: Extremadura; county: Cáceres; locality: Peña Falcón; verbatimElevation: 320.6; decimalLatitude: 39.83296; decimalLongitude: -6.0641; geodeticDatum: WGS84; **Event:** eventID: 2; samplingProtocol: Aerial; eventTime: Night
- **Type status:** Other material. **Occurrence:** individualCount: 1; sex: male; **Location:** locationID: M1; continent: Europe; country: Spain; countryCode: ES; stateProvince: Extremadura; county: Cáceres; locality: Peña Falcón; verbatimElevation: 320.6; decimalLatitude: 39.83296; decimalLongitude: -6.0641; geodeticDatum: WGS84; **Event:** eventID: 4; samplingProtocol: Aerial; eventTime: Night
- **Type status:** Other material. **Occurrence:** individualCount: 2; sex: female; **Location:** locationID: M1; continent: Europe; country: Spain; countryCode: ES; stateProvince: Extremadura; county: Cáceres; locality: Peña Falcón; verbatimElevation: 320.6; decimalLatitude: 39.83296; decimalLongitude: -6.0641; geodeticDatum: WGS84; **Event:** eventID: 1; samplingProtocol: Beating; eventTime: Day
- **Type status:** Other material. **Occurrence:** individualCount: 1; sex: female; **Location:** locationID: M1; continent: Europe; country: Spain; countryCode: ES; stateProvince: Extremadura; county: Cáceres; locality: Peña Falcón; verbatimElevation: 320.6; decimalLatitude: 39.83296; decimalLongitude: -6.0641; geodeticDatum: WGS84; **Event:** eventID: 1; samplingProtocol: Beating; eventTime: Night
- **Type status:** Other material. **Occurrence:** individualCount: 4; sex: female; **Location:** locationID: M2; continent: Europe; country: Spain; countryCode: ES; stateProvince: Extremadura; county: Cáceres; locality: Fuente del Frances; verbatimElevation: 320.72; decimalLatitude: 39.828; decimalLongitude: -6.03249; geodeticDatum: WGS84; **Event:** eventID: 2; samplingProtocol: Aerial; eventTime: Night
- **Type status:** Other material. **Occurrence:** individualCount: 5; sex: female; **Location:** locationID: O2; continent: Europe; country: Spain; countryCode: ES; stateProvince: Aragón; county: Huesca; locality: Rebilla; verbatimElevation: 1158.13; decimalLatitude: 42.59427; decimalLongitude: 0.1529; geodeticDatum: WGS84; **Event:** eventID: 1; samplingProtocol: Aerial; eventTime: Night
- **Type status:** Other material. **Occurrence:** individualCount: 5; sex: male; **Location:** locationID: O2; continent: Europe; country: Spain; countryCode: ES; stateProvince: Aragón; county: Huesca; locality: Rebilla; verbatimElevation: 1158.13; decimalLatitude: 42.59427; decimalLongitude: 0.1529; geodeticDatum: WGS84; **Event:** eventID: 1; samplingProtocol: Aerial; eventTime: Night
- **Type status:** Other material. **Occurrence:** individualCount: 4; sex: female; **Location:** locationID: O2; continent: Europe; country: Spain; countryCode: ES; stateProvince: Aragón; county: Huesca; locality: Rebilla; verbatimElevation: 1158.13; decimalLatitude: 42.59427; decimalLongitude: 0.1529; geodeticDatum: WGS84; **Event:** eventID: 1; samplingProtocol: Aerial; eventTime: Night
- **Type status:** Other material. **Occurrence:** individualCount: 3; sex: male; **Location:** locationID: O2; continent: Europe; country: Spain; countryCode: ES; stateProvince: Aragón; county: Huesca; locality: Rebilla; verbatimElevation: 1158.13; decimalLatitude: 42.59427; decimalLongitude: 0.1529; geodeticDatum: WGS84; **Event:** eventID: 1; samplingProtocol: Aerial; eventTime: Night
- **Type status:** Other material. **Occurrence:** individualCount: 2; sex: female; **Location:** locationID: O2; continent: Europe; country: Spain; countryCode: ES; stateProvince: Aragón; county: Huesca; locality: Rebilla; verbatimElevation: 1158.13; decimalLatitude: 42.59427; decimalLongitude: 0.1529; geodeticDatum: WGS84; **Event:** eventID: 2; samplingProtocol: Aerial; eventTime: Night
- **Type status:** Other material. **Occurrence:** individualCount: 10; sex: female; **Location:** locationID: O2; continent: Europe; country: Spain; countryCode: ES; stateProvince: Aragón; county: Huesca; locality: Rebilla; verbatimElevation: 1158.13; decimalLatitude: 42.59427; decimalLongitude: 0.1529; geodeticDatum: WGS84; **Event:** eventID: 2; samplingProtocol: Aerial; eventTime: Night
- **Type status:** Other material. **Occurrence:** individualCount: 1; sex: male; **Location:** locationID: O2; continent: Europe; country: Spain; countryCode: ES; stateProvince: Aragón; county: Huesca; locality: Rebilla; verbatimElevation: 1158.13; decimalLatitude: 42.59427; decimalLongitude: 0.1529; geodeticDatum: WGS84; **Event:** eventID: 2; samplingProtocol: Aerial; eventTime: Night
- **Type status:** Other material. **Occurrence:** individualCount: 15; sex: female; **Location:** locationID: O2; continent: Europe; country: Spain; countryCode: ES; stateProvince: Aragón; county: Huesca; locality: Rebilla; verbatimElevation: 1158.13; decimalLatitude: 42.59427; decimalLongitude: 0.1529; geodeticDatum: WGS84; **Event:** eventID: 1; samplingProtocol: Beating; eventTime: Day
- **Type status:** Other material. **Occurrence:** individualCount: 5; sex: male; **Location:** locationID: O2; continent: Europe; country: Spain; countryCode: ES; stateProvince: Aragón; county: Huesca; locality: Rebilla; verbatimElevation: 1158.13; decimalLatitude: 42.59427; decimalLongitude: 0.1529; geodeticDatum: WGS84; **Event:** eventID: 1; samplingProtocol: Beating; eventTime: Day
- **Type status:** Other material. **Occurrence:** individualCount: 12; sex: female; **Location:** locationID: O2; continent: Europe; country: Spain; countryCode: ES; stateProvince: Aragón; county: Huesca; locality: Rebilla; verbatimElevation: 1158.13; decimalLatitude: 42.59427; decimalLongitude: 0.1529; geodeticDatum: WGS84; **Event:** eventID: 2; samplingProtocol: Beating; eventTime: Day
- **Type status:** Other material. **Occurrence:** individualCount: 4; sex: male; **Location:** locationID: O2; continent: Europe; country: Spain; countryCode: ES; stateProvince: Aragón; county: Huesca; locality: Rebilla; verbatimElevation: 1158.13; decimalLatitude: 42.59427; decimalLongitude: 0.1529; geodeticDatum: WGS84; **Event:** eventID: 2; samplingProtocol: Beating; eventTime: Day
- **Type status:** Other material. **Occurrence:** individualCount: 9; sex: female; **Location:** locationID: O2; continent: Europe; country: Spain; countryCode: ES; stateProvince: Aragón; county: Huesca; locality: Rebilla; verbatimElevation: 1158.13; decimalLatitude: 42.59427; decimalLongitude: 0.1529; geodeticDatum: WGS84; **Event:** eventID: 1; samplingProtocol: Beating; eventTime: Night
- **Type status:** Other material. **Occurrence:** individualCount: 1; sex: male; **Location:** locationID: O2; continent: Europe; country: Spain; countryCode: ES; stateProvince: Aragón; county: Huesca; locality: Rebilla; verbatimElevation: 1158.13; decimalLatitude: 42.59427; decimalLongitude: 0.1529; geodeticDatum: WGS84; **Event:** eventID: 1; samplingProtocol: Beating; eventTime: Night
- **Type status:** Other material. **Occurrence:** individualCount: 16; sex: female; **Location:** locationID: O2; continent: Europe; country: Spain; countryCode: ES; stateProvince: Aragón; county: Huesca; locality: Rebilla; verbatimElevation: 1158.13; decimalLatitude: 42.59427; decimalLongitude: 0.1529; geodeticDatum: WGS84; **Event:** eventID: 1; samplingProtocol: Beating; eventTime: Night
- **Type status:** Other material. **Occurrence:** individualCount: 5; sex: male; **Location:** locationID: O2; continent: Europe; country: Spain; countryCode: ES; stateProvince: Aragón; county: Huesca; locality: Rebilla; verbatimElevation: 1158.13; decimalLatitude: 42.59427; decimalLongitude: 0.1529; geodeticDatum: WGS84; **Event:** eventID: 1; samplingProtocol: Beating; eventTime: Night
- **Type status:** Other material. **Occurrence:** individualCount: 1; sex: female; **Location:** locationID: O2; continent: Europe; country: Spain; countryCode: ES; stateProvince: Aragón; county: Huesca; locality: Rebilla; verbatimElevation: 1158.13; decimalLatitude: 42.59427; decimalLongitude: 0.1529; geodeticDatum: WGS84; **Event:** eventID: 1; samplingProtocol: Sweeping; eventTime: Day
- **Type status:** Other material. **Occurrence:** individualCount: 4; sex: female; **Location:** locationID: O2; continent: Europe; country: Spain; countryCode: ES; stateProvince: Aragón; county: Huesca; locality: Rebilla; verbatimElevation: 1158.13; decimalLatitude: 42.59427; decimalLongitude: 0.1529; geodeticDatum: WGS84; **Event:** eventID: 2; samplingProtocol: Sweeping; eventTime: Day
- **Type status:** Other material. **Occurrence:** individualCount: 3; sex: female; **Location:** locationID: O2; continent: Europe; country: Spain; countryCode: ES; stateProvince: Aragón; county: Huesca; locality: Rebilla; verbatimElevation: 1158.13; decimalLatitude: 42.59427; decimalLongitude: 0.1529; geodeticDatum: WGS84; **Event:** eventID: 1; samplingProtocol: Sweeping; eventTime: Night
- **Type status:** Other material. **Occurrence:** individualCount: 1; sex: male; **Location:** locationID: O2; continent: Europe; country: Spain; countryCode: ES; stateProvince: Aragón; county: Huesca; locality: Rebilla; verbatimElevation: 1158.13; decimalLatitude: 42.59427; decimalLongitude: 0.1529; geodeticDatum: WGS84; **Event:** eventID: 1; samplingProtocol: Sweeping; eventTime: Night
- **Type status:** Other material. **Occurrence:** individualCount: 5; sex: female; **Location:** locationID: O2; continent: Europe; country: Spain; countryCode: ES; stateProvince: Aragón; county: Huesca; locality: Rebilla; verbatimElevation: 1158.13; decimalLatitude: 42.59427; decimalLongitude: 0.1529; geodeticDatum: WGS84; **Event:** eventID: 1; samplingProtocol: Sweeping; eventTime: Night
- **Type status:** Other material. **Occurrence:** individualCount: 4; sex: male; **Location:** locationID: O2; continent: Europe; country: Spain; countryCode: ES; stateProvince: Aragón; county: Huesca; locality: Rebilla; verbatimElevation: 1158.13; decimalLatitude: 42.59427; decimalLongitude: 0.1529; geodeticDatum: WGS84; **Event:** eventID: 1; samplingProtocol: Sweeping; eventTime: Night
- **Type status:** Other material. **Occurrence:** individualCount: 2; sex: male; **Location:** locationID: P2; continent: Europe; country: Spain; countryCode: ES; stateProvince: Castilla y León; county: León; locality: Joyoguelas; verbatimElevation: 763.98; decimalLatitude: 43.17771; decimalLongitude: -4.90579; geodeticDatum: WGS84; **Event:** eventID: 1; samplingProtocol: Aerial; eventTime: Night
- **Type status:** Other material. **Occurrence:** individualCount: 1; sex: male; **Location:** locationID: P2; continent: Europe; country: Spain; countryCode: ES; stateProvince: Castilla y León; county: León; locality: Joyoguelas; verbatimElevation: 763.98; decimalLatitude: 43.17771; decimalLongitude: -4.90579; geodeticDatum: WGS84; **Event:** eventID: 2; samplingProtocol: Aerial; eventTime: Night
- **Type status:** Other material. **Occurrence:** individualCount: 1; sex: male; **Location:** locationID: P3; continent: Europe; country: Spain; countryCode: ES; stateProvince: Castilla y León; county: León; locality: Las Arroyas; verbatimElevation: 1097.1; decimalLatitude: 43.14351; decimalLongitude: -4.94878; geodeticDatum: WGS84; **Event:** eventID: 1; samplingProtocol: Sweeping; eventTime: Day
- **Type status:** Other material. **Occurrence:** individualCount: 1; sex: male; **Location:** locationID: P4; continent: Europe; country: Spain; countryCode: ES; stateProvince: Castilla y León; county: León; locality: El Canto; verbatimElevation: 943.48; decimalLatitude: 43.17227; decimalLongitude: -4.90857; geodeticDatum: WGS84; **Event:** eventID: 2; samplingProtocol: Beating; eventTime: Day
- **Type status:** Other material. **Occurrence:** individualCount: 1; sex: female; **Location:** locationID: P4; continent: Europe; country: Spain; countryCode: ES; stateProvince: Castilla y León; county: León; locality: El Canto; verbatimElevation: 943.48; decimalLatitude: 43.17227; decimalLongitude: -4.90857; geodeticDatum: WGS84; **Event:** eventID: 1; samplingProtocol: Sweeping; eventTime: Night

##### Distribution

Europe to Central Asia

#### Lasaeola sp23

##### Materials

- **Type status:** Other material. **Occurrence:** individualCount: 1; sex: male; **Location:** locationID: C3; continent: Europe; country: Spain; countryCode: ES; stateProvince: Castilla-La Mancha; county: Ciudad Real; locality: La Quesera; verbatimElevation: 767.55; decimalLatitude: 39.36177; decimalLongitude: -4.41733; geodeticDatum: WGS84; **Event:** eventID: 3; samplingProtocol: Aerial; eventTime: Night
- **Type status:** Other material. **Occurrence:** individualCount: 4; sex: male; **Location:** locationID: M2; continent: Europe; country: Spain; countryCode: ES; stateProvince: Extremadura; county: Cáceres; locality: Fuente del Frances; verbatimElevation: 320.72; decimalLatitude: 39.828; decimalLongitude: -6.03249; geodeticDatum: WGS84; **Event:** eventID: 2; samplingProtocol: Aerial; eventTime: Night
- **Type status:** Other material. **Occurrence:** individualCount: 4; sex: male; **Location:** locationID: O2; continent: Europe; country: Spain; countryCode: ES; stateProvince: Aragón; county: Huesca; locality: Rebilla; verbatimElevation: 1158.13; decimalLatitude: 42.59427; decimalLongitude: 0.1529; geodeticDatum: WGS84; **Event:** eventID: 2; samplingProtocol: Aerial; eventTime: Night

##### Distribution

?

##### Notes

This is a species of *Lasaeola* Simon, 1881, which we were unable to identify.

#### Lasaeola sp36

##### Materials

- **Type status:** Other material. **Occurrence:** individualCount: 2; sex: female; **Location:** locationID: C2; continent: Europe; country: Spain; countryCode: ES; stateProvince: Castilla-La Mancha; county: Ciudad Real; locality: Valle Brezoso; verbatimElevation: 739.31; decimalLatitude: 39.35159; decimalLongitude: -4.3589; geodeticDatum: WGS84; **Event:** eventID: 2; samplingProtocol: Beating; eventTime: Night
- **Type status:** Other material. **Occurrence:** individualCount: 1; sex: female; **Location:** locationID: C4; continent: Europe; country: Spain; countryCode: ES; stateProvince: Castilla-La Mancha; county: Ciudad Real; locality: La Quesera; verbatimElevation: 772.3; decimalLatitude: 39.36337; decimalLongitude: -4.41704; geodeticDatum: WGS84; **Event:** eventID: 3; samplingProtocol: Aerial; eventTime: Night

##### Distribution

?

##### Notes

This is a species of *Lasaeola*, which we were unable to identify.

#### Neottiura bimaculata

(Linnaeus, 1767)

##### Materials

- **Type status:** Other material. **Occurrence:** individualCount: 1; sex: male; **Location:** locationID: O2; continent: Europe; country: Spain; countryCode: ES; stateProvince: Aragón; county: Huesca; locality: Rebilla; verbatimElevation: 1158.13; decimalLatitude: 42.59427; decimalLongitude: 0.1529; geodeticDatum: WGS84; **Event:** eventID: J; samplingProtocol: Pitfall
- **Type status:** Other material. **Occurrence:** individualCount: 1; sex: male; **Location:** locationID: O2; continent: Europe; country: Spain; countryCode: ES; stateProvince: Aragón; county: Huesca; locality: Rebilla; verbatimElevation: 1158.13; decimalLatitude: 42.59427; decimalLongitude: 0.1529; geodeticDatum: WGS84; **Event:** eventID: 1; samplingProtocol: Sweeping; eventTime: Night
- **Type status:** Other material. **Occurrence:** individualCount: 1; sex: female; **Location:** locationID: O2; continent: Europe; country: Spain; countryCode: ES; stateProvince: Aragón; county: Huesca; locality: Rebilla; verbatimElevation: 1158.13; decimalLatitude: 42.59427; decimalLongitude: 0.1529; geodeticDatum: WGS84; **Event:** eventID: 1; samplingProtocol: Sweeping; eventTime: Night
- **Type status:** Other material. **Occurrence:** individualCount: 1; sex: female; **Location:** locationID: P4; continent: Europe; country: Spain; countryCode: ES; stateProvince: Castilla y León; county: León; locality: El Canto; verbatimElevation: 943.48; decimalLatitude: 43.17227; decimalLongitude: -4.90857; geodeticDatum: WGS84; **Event:** eventID: 1; samplingProtocol: Sweeping; eventTime: Night

##### Distribution

Holarctic

#### Paidiscura pallens

(Blackwall, 1834)

##### Materials

- **Type status:** Other material. **Occurrence:** individualCount: 1; sex: female; **Location:** locationID: A1; continent: Europe; country: Spain; countryCode: ES; stateProvince: Catalonia; county: Lleida; locality: Sola de Boi; verbatimElevation: 1759.8; decimalLatitude: 42.54958; decimalLongitude: 0.87254; geodeticDatum: WGS84; **Event:** eventID: 1; samplingProtocol: Beating; eventTime: Day
- **Type status:** Other material. **Occurrence:** individualCount: 1; sex: female; **Location:** locationID: A1; continent: Europe; country: Spain; countryCode: ES; stateProvince: Catalonia; county: Lleida; locality: Sola de Boi; verbatimElevation: 1759.8; decimalLatitude: 42.54958; decimalLongitude: 0.87254; geodeticDatum: WGS84; **Event:** eventID: 2; samplingProtocol: Beating; eventTime: Day
- **Type status:** Other material. **Occurrence:** individualCount: 1; sex: male; **Location:** locationID: A1; continent: Europe; country: Spain; countryCode: ES; stateProvince: Catalonia; county: Lleida; locality: Sola de Boi; verbatimElevation: 1759.8; decimalLatitude: 42.54958; decimalLongitude: 0.87254; geodeticDatum: WGS84; **Event:** eventID: 1; samplingProtocol: Beating; eventTime: Night
- **Type status:** Other material. **Occurrence:** individualCount: 1; sex: female; **Location:** locationID: A2; continent: Europe; country: Spain; countryCode: ES; stateProvince: Catalonia; county: Lleida; locality: Sola de Boi; verbatimElevation: 1738.7; decimalLatitude: 42.54913; decimalLongitude: 0.87137; geodeticDatum: WGS84; **Event:** eventID: 1; samplingProtocol: Beating; eventTime: Day
- **Type status:** Other material. **Occurrence:** individualCount: 2; sex: male; **Location:** locationID: A2; continent: Europe; country: Spain; countryCode: ES; stateProvince: Catalonia; county: Lleida; locality: Sola de Boi; verbatimElevation: 1738.7; decimalLatitude: 42.54913; decimalLongitude: 0.87137; geodeticDatum: WGS84; **Event:** eventID: 1; samplingProtocol: Beating; eventTime: Day
- **Type status:** Other material. **Occurrence:** individualCount: 4; sex: female; **Location:** locationID: A2; continent: Europe; country: Spain; countryCode: ES; stateProvince: Catalonia; county: Lleida; locality: Sola de Boi; verbatimElevation: 1738.7; decimalLatitude: 42.54913; decimalLongitude: 0.87137; geodeticDatum: WGS84; **Event:** eventID: 2; samplingProtocol: Beating; eventTime: Day
- **Type status:** Other material. **Occurrence:** individualCount: 2; sex: female; **Location:** locationID: A2; continent: Europe; country: Spain; countryCode: ES; stateProvince: Catalonia; county: Lleida; locality: Sola de Boi; verbatimElevation: 1738.7; decimalLatitude: 42.54913; decimalLongitude: 0.87137; geodeticDatum: WGS84; **Event:** eventID: 1; samplingProtocol: Beating; eventTime: Night
- **Type status:** Other material. **Occurrence:** individualCount: 2; sex: female; **Location:** locationID: A2; continent: Europe; country: Spain; countryCode: ES; stateProvince: Catalonia; county: Lleida; locality: Sola de Boi; verbatimElevation: 1738.7; decimalLatitude: 42.54913; decimalLongitude: 0.87137; geodeticDatum: WGS84; **Event:** eventID: 1; samplingProtocol: Beating; eventTime: Night
- **Type status:** Other material. **Occurrence:** individualCount: 1; sex: female; **Location:** locationID: C4; continent: Europe; country: Spain; countryCode: ES; stateProvince: Castilla-La Mancha; county: Ciudad Real; locality: La Quesera; verbatimElevation: 772.3; decimalLatitude: 39.36337; decimalLongitude: -4.41704; geodeticDatum: WGS84; **Event:** eventID: 3; samplingProtocol: Aerial; eventTime: Night
- **Type status:** Other material. **Occurrence:** individualCount: 1; sex: female; **Location:** locationID: C4; continent: Europe; country: Spain; countryCode: ES; stateProvince: Castilla-La Mancha; county: Ciudad Real; locality: La Quesera; verbatimElevation: 772.3; decimalLatitude: 39.36337; decimalLongitude: -4.41704; geodeticDatum: WGS84; **Event:** eventID: 1; samplingProtocol: Beating; eventTime: Day
- **Type status:** Other material. **Occurrence:** individualCount: 1; sex: female; **Location:** locationID: M2; continent: Europe; country: Spain; countryCode: ES; stateProvince: Extremadura; county: Cáceres; locality: Fuente del Frances; verbatimElevation: 320.72; decimalLatitude: 39.828; decimalLongitude: -6.03249; geodeticDatum: WGS84; **Event:** eventID: 1; samplingProtocol: Beating; eventTime: Night
- **Type status:** Other material. **Occurrence:** individualCount: 1; sex: female; **Location:** locationID: O1; continent: Europe; country: Spain; countryCode: ES; stateProvince: Aragón; county: Huesca; locality: O Furno; verbatimElevation: 1396.73; decimalLatitude: 42.60677; decimalLongitude: 0.13135; geodeticDatum: WGS84; **Event:** eventID: 1; samplingProtocol: Aerial; eventTime: Night
- **Type status:** Other material. **Occurrence:** individualCount: 2; sex: female; **Location:** locationID: O1; continent: Europe; country: Spain; countryCode: ES; stateProvince: Aragón; county: Huesca; locality: O Furno; verbatimElevation: 1396.73; decimalLatitude: 42.60677; decimalLongitude: 0.13135; geodeticDatum: WGS84; **Event:** eventID: 2; samplingProtocol: Aerial; eventTime: Night
- **Type status:** Other material. **Occurrence:** individualCount: 10; sex: female; **Location:** locationID: O1; continent: Europe; country: Spain; countryCode: ES; stateProvince: Aragón; county: Huesca; locality: O Furno; verbatimElevation: 1396.73; decimalLatitude: 42.60677; decimalLongitude: 0.13135; geodeticDatum: WGS84; **Event:** eventID: 1; samplingProtocol: Beating; eventTime: Day
- **Type status:** Other material. **Occurrence:** individualCount: 2; sex: female; **Location:** locationID: O1; continent: Europe; country: Spain; countryCode: ES; stateProvince: Aragón; county: Huesca; locality: O Furno; verbatimElevation: 1396.73; decimalLatitude: 42.60677; decimalLongitude: 0.13135; geodeticDatum: WGS84; **Event:** eventID: 2; samplingProtocol: Beating; eventTime: Day
- **Type status:** Other material. **Occurrence:** individualCount: 1; sex: female; **Location:** locationID: O1; continent: Europe; country: Spain; countryCode: ES; stateProvince: Aragón; county: Huesca; locality: O Furno; verbatimElevation: 1396.73; decimalLatitude: 42.60677; decimalLongitude: 0.13135; geodeticDatum: WGS84; **Event:** eventID: 1; samplingProtocol: Beating; eventTime: Night
- **Type status:** Other material. **Occurrence:** individualCount: 1; sex: female; **Location:** locationID: O1; continent: Europe; country: Spain; countryCode: ES; stateProvince: Aragón; county: Huesca; locality: O Furno; verbatimElevation: 1396.73; decimalLatitude: 42.60677; decimalLongitude: 0.13135; geodeticDatum: WGS84; **Event:** eventID: 1; samplingProtocol: Beating; eventTime: Night
- **Type status:** Other material. **Occurrence:** individualCount: 2; sex: female; **Location:** locationID: O1; continent: Europe; country: Spain; countryCode: ES; stateProvince: Aragón; county: Huesca; locality: O Furno; verbatimElevation: 1396.73; decimalLatitude: 42.60677; decimalLongitude: 0.13135; geodeticDatum: WGS84; **Event:** eventID: 1; samplingProtocol: Sweeping; eventTime: Day
- **Type status:** Other material. **Occurrence:** individualCount: 1; sex: female; **Location:** locationID: O2; continent: Europe; country: Spain; countryCode: ES; stateProvince: Aragón; county: Huesca; locality: Rebilla; verbatimElevation: 1158.13; decimalLatitude: 42.59427; decimalLongitude: 0.1529; geodeticDatum: WGS84; **Event:** eventID: 1; samplingProtocol: Aerial; eventTime: Night
- **Type status:** Other material. **Occurrence:** individualCount: 1; sex: female; **Location:** locationID: O2; continent: Europe; country: Spain; countryCode: ES; stateProvince: Aragón; county: Huesca; locality: Rebilla; verbatimElevation: 1158.13; decimalLatitude: 42.59427; decimalLongitude: 0.1529; geodeticDatum: WGS84; **Event:** eventID: 1; samplingProtocol: Beating; eventTime: Day
- **Type status:** Other material. **Occurrence:** individualCount: 3; sex: female; **Location:** locationID: O2; continent: Europe; country: Spain; countryCode: ES; stateProvince: Aragón; county: Huesca; locality: Rebilla; verbatimElevation: 1158.13; decimalLatitude: 42.59427; decimalLongitude: 0.1529; geodeticDatum: WGS84; **Event:** eventID: 2; samplingProtocol: Beating; eventTime: Day
- **Type status:** Other material. **Occurrence:** individualCount: 1; sex: female; **Location:** locationID: O2; continent: Europe; country: Spain; countryCode: ES; stateProvince: Aragón; county: Huesca; locality: Rebilla; verbatimElevation: 1158.13; decimalLatitude: 42.59427; decimalLongitude: 0.1529; geodeticDatum: WGS84; **Event:** eventID: 2; samplingProtocol: Sweeping; eventTime: Day
- **Type status:** Other material. **Occurrence:** individualCount: 1; sex: female; **Location:** locationID: P1; continent: Europe; country: Spain; countryCode: ES; stateProvince: Castilla y León; county: León; locality: Monte Robledo; verbatimElevation: 1071.58; decimalLatitude: 43.1445; decimalLongitude: -4.92675; geodeticDatum: WGS84; **Event:** eventID: 2; samplingProtocol: Aerial; eventTime: Night
- **Type status:** Other material. **Occurrence:** individualCount: 2; sex: female; **Location:** locationID: P1; continent: Europe; country: Spain; countryCode: ES; stateProvince: Castilla y León; county: León; locality: Monte Robledo; verbatimElevation: 1071.58; decimalLatitude: 43.1445; decimalLongitude: -4.92675; geodeticDatum: WGS84; **Event:** eventID: 2; samplingProtocol: Aerial; eventTime: Night
- **Type status:** Other material. **Occurrence:** individualCount: 5; sex: female; **Location:** locationID: P1; continent: Europe; country: Spain; countryCode: ES; stateProvince: Castilla y León; county: León; locality: Monte Robledo; verbatimElevation: 1071.58; decimalLatitude: 43.1445; decimalLongitude: -4.92675; geodeticDatum: WGS84; **Event:** eventID: 1; samplingProtocol: Beating; eventTime: Day
- **Type status:** Other material. **Occurrence:** individualCount: 1; sex: male; **Location:** locationID: P1; continent: Europe; country: Spain; countryCode: ES; stateProvince: Castilla y León; county: León; locality: Monte Robledo; verbatimElevation: 1071.58; decimalLatitude: 43.1445; decimalLongitude: -4.92675; geodeticDatum: WGS84; **Event:** eventID: 1; samplingProtocol: Beating; eventTime: Day
- **Type status:** Other material. **Occurrence:** individualCount: 13; sex: female; **Location:** locationID: P1; continent: Europe; country: Spain; countryCode: ES; stateProvince: Castilla y León; county: León; locality: Monte Robledo; verbatimElevation: 1071.58; decimalLatitude: 43.1445; decimalLongitude: -4.92675; geodeticDatum: WGS84; **Event:** eventID: 2; samplingProtocol: Beating; eventTime: Day
- **Type status:** Other material. **Occurrence:** individualCount: 2; sex: male; **Location:** locationID: P1; continent: Europe; country: Spain; countryCode: ES; stateProvince: Castilla y León; county: León; locality: Monte Robledo; verbatimElevation: 1071.58; decimalLatitude: 43.1445; decimalLongitude: -4.92675; geodeticDatum: WGS84; **Event:** eventID: 2; samplingProtocol: Beating; eventTime: Day
- **Type status:** Other material. **Occurrence:** individualCount: 1; sex: male; **Location:** locationID: P1; continent: Europe; country: Spain; countryCode: ES; stateProvince: Castilla y León; county: León; locality: Monte Robledo; verbatimElevation: 1071.58; decimalLatitude: 43.1445; decimalLongitude: -4.92675; geodeticDatum: WGS84; **Event:** eventID: 1; samplingProtocol: Beating; eventTime: Night
- **Type status:** Other material. **Occurrence:** individualCount: 1; sex: female; **Location:** locationID: P1; continent: Europe; country: Spain; countryCode: ES; stateProvince: Castilla y León; county: León; locality: Monte Robledo; verbatimElevation: 1071.58; decimalLatitude: 43.1445; decimalLongitude: -4.92675; geodeticDatum: WGS84; **Event:** eventID: 1; samplingProtocol: Beating; eventTime: Night
- **Type status:** Other material. **Occurrence:** individualCount: 3; sex: male; **Location:** locationID: P1; continent: Europe; country: Spain; countryCode: ES; stateProvince: Castilla y León; county: León; locality: Monte Robledo; verbatimElevation: 1071.58; decimalLatitude: 43.1445; decimalLongitude: -4.92675; geodeticDatum: WGS84; **Event:** eventID: 1; samplingProtocol: Beating; eventTime: Night
- **Type status:** Other material. **Occurrence:** individualCount: 1; sex: female; **Location:** locationID: P1; continent: Europe; country: Spain; countryCode: ES; stateProvince: Castilla y León; county: León; locality: Monte Robledo; verbatimElevation: 1071.58; decimalLatitude: 43.1445; decimalLongitude: -4.92675; geodeticDatum: WGS84; **Event:** eventID: 1; samplingProtocol: Sweeping; eventTime: Day
- **Type status:** Other material. **Occurrence:** individualCount: 1; sex: male; **Location:** locationID: P1; continent: Europe; country: Spain; countryCode: ES; stateProvince: Castilla y León; county: León; locality: Monte Robledo; verbatimElevation: 1071.58; decimalLatitude: 43.1445; decimalLongitude: -4.92675; geodeticDatum: WGS84; **Event:** eventID: 1; samplingProtocol: Sweeping; eventTime: Day
- **Type status:** Other material. **Occurrence:** individualCount: 1; sex: female; **Location:** locationID: P1; continent: Europe; country: Spain; countryCode: ES; stateProvince: Castilla y León; county: León; locality: Monte Robledo; verbatimElevation: 1071.58; decimalLatitude: 43.1445; decimalLongitude: -4.92675; geodeticDatum: WGS84; **Event:** eventID: 2; samplingProtocol: Sweeping; eventTime: Day
- **Type status:** Other material. **Occurrence:** individualCount: 1; sex: male; **Location:** locationID: P1; continent: Europe; country: Spain; countryCode: ES; stateProvince: Castilla y León; county: León; locality: Monte Robledo; verbatimElevation: 1071.58; decimalLatitude: 43.1445; decimalLongitude: -4.92675; geodeticDatum: WGS84; **Event:** eventID: 2; samplingProtocol: Sweeping; eventTime: Day
- **Type status:** Other material. **Occurrence:** individualCount: 2; sex: female; **Location:** locationID: P1; continent: Europe; country: Spain; countryCode: ES; stateProvince: Castilla y León; county: León; locality: Monte Robledo; verbatimElevation: 1071.58; decimalLatitude: 43.1445; decimalLongitude: -4.92675; geodeticDatum: WGS84; **Event:** eventID: 1; samplingProtocol: Sweeping; eventTime: Night
- **Type status:** Other material. **Occurrence:** individualCount: 1; sex: male; **Location:** locationID: P1; continent: Europe; country: Spain; countryCode: ES; stateProvince: Castilla y León; county: León; locality: Monte Robledo; verbatimElevation: 1071.58; decimalLatitude: 43.1445; decimalLongitude: -4.92675; geodeticDatum: WGS84; **Event:** eventID: 1; samplingProtocol: Sweeping; eventTime: Night
- **Type status:** Other material. **Occurrence:** individualCount: 8; sex: female; **Location:** locationID: P1; continent: Europe; country: Spain; countryCode: ES; stateProvince: Castilla y León; county: León; locality: Monte Robledo; verbatimElevation: 1071.58; decimalLatitude: 43.1445; decimalLongitude: -4.92675; geodeticDatum: WGS84; **Event:** eventID: 1; samplingProtocol: Sweeping; eventTime: Night
- **Type status:** Other material. **Occurrence:** individualCount: 7; sex: male; **Location:** locationID: P1; continent: Europe; country: Spain; countryCode: ES; stateProvince: Castilla y León; county: León; locality: Monte Robledo; verbatimElevation: 1071.58; decimalLatitude: 43.1445; decimalLongitude: -4.92675; geodeticDatum: WGS84; **Event:** eventID: 1; samplingProtocol: Sweeping; eventTime: Night
- **Type status:** Other material. **Occurrence:** individualCount: 1; sex: female; **Location:** locationID: P2; continent: Europe; country: Spain; countryCode: ES; stateProvince: Castilla y León; county: León; locality: Joyoguelas; verbatimElevation: 763.98; decimalLatitude: 43.17771; decimalLongitude: -4.90579; geodeticDatum: WGS84; **Event:** eventID: 1; samplingProtocol: Aerial; eventTime: Night
- **Type status:** Other material. **Occurrence:** individualCount: 1; sex: female; **Location:** locationID: P2; continent: Europe; country: Spain; countryCode: ES; stateProvince: Castilla y León; county: León; locality: Joyoguelas; verbatimElevation: 763.98; decimalLatitude: 43.17771; decimalLongitude: -4.90579; geodeticDatum: WGS84; **Event:** eventID: 1; samplingProtocol: Aerial; eventTime: Night
- **Type status:** Other material. **Occurrence:** individualCount: 1; sex: female; **Location:** locationID: P2; continent: Europe; country: Spain; countryCode: ES; stateProvince: Castilla y León; county: León; locality: Joyoguelas; verbatimElevation: 763.98; decimalLatitude: 43.17771; decimalLongitude: -4.90579; geodeticDatum: WGS84; **Event:** eventID: 2; samplingProtocol: Aerial; eventTime: Night
- **Type status:** Other material. **Occurrence:** individualCount: 2; sex: female; **Location:** locationID: P2; continent: Europe; country: Spain; countryCode: ES; stateProvince: Castilla y León; county: León; locality: Joyoguelas; verbatimElevation: 763.98; decimalLatitude: 43.17771; decimalLongitude: -4.90579; geodeticDatum: WGS84; **Event:** eventID: 2; samplingProtocol: Aerial; eventTime: Night
- **Type status:** Other material. **Occurrence:** individualCount: 5; sex: female; **Location:** locationID: P2; continent: Europe; country: Spain; countryCode: ES; stateProvince: Castilla y León; county: León; locality: Joyoguelas; verbatimElevation: 763.98; decimalLatitude: 43.17771; decimalLongitude: -4.90579; geodeticDatum: WGS84; **Event:** eventID: 1; samplingProtocol: Beating; eventTime: Day
- **Type status:** Other material. **Occurrence:** individualCount: 14; sex: female; **Location:** locationID: P2; continent: Europe; country: Spain; countryCode: ES; stateProvince: Castilla y León; county: León; locality: Joyoguelas; verbatimElevation: 763.98; decimalLatitude: 43.17771; decimalLongitude: -4.90579; geodeticDatum: WGS84; **Event:** eventID: 2; samplingProtocol: Beating; eventTime: Day
- **Type status:** Other material. **Occurrence:** individualCount: 1; sex: male; **Location:** locationID: P2; continent: Europe; country: Spain; countryCode: ES; stateProvince: Castilla y León; county: León; locality: Joyoguelas; verbatimElevation: 763.98; decimalLatitude: 43.17771; decimalLongitude: -4.90579; geodeticDatum: WGS84; **Event:** eventID: 2; samplingProtocol: Beating; eventTime: Day
- **Type status:** Other material. **Occurrence:** individualCount: 6; sex: female; **Location:** locationID: P2; continent: Europe; country: Spain; countryCode: ES; stateProvince: Castilla y León; county: León; locality: Joyoguelas; verbatimElevation: 763.98; decimalLatitude: 43.17771; decimalLongitude: -4.90579; geodeticDatum: WGS84; **Event:** eventID: 1; samplingProtocol: Beating; eventTime: Night
- **Type status:** Other material. **Occurrence:** individualCount: 1; sex: male; **Location:** locationID: P2; continent: Europe; country: Spain; countryCode: ES; stateProvince: Castilla y León; county: León; locality: Joyoguelas; verbatimElevation: 763.98; decimalLatitude: 43.17771; decimalLongitude: -4.90579; geodeticDatum: WGS84; **Event:** eventID: 1; samplingProtocol: Beating; eventTime: Night
- **Type status:** Other material. **Occurrence:** individualCount: 1; sex: female; **Location:** locationID: P2; continent: Europe; country: Spain; countryCode: ES; stateProvince: Castilla y León; county: León; locality: Joyoguelas; verbatimElevation: 763.98; decimalLatitude: 43.17771; decimalLongitude: -4.90579; geodeticDatum: WGS84; **Event:** eventID: 1; samplingProtocol: Beating; eventTime: Night
- **Type status:** Other material. **Occurrence:** individualCount: 1; sex: female; **Location:** locationID: P2; continent: Europe; country: Spain; countryCode: ES; stateProvince: Castilla y León; county: León; locality: Joyoguelas; verbatimElevation: 763.98; decimalLatitude: 43.17771; decimalLongitude: -4.90579; geodeticDatum: WGS84; **Event:** eventID: 1; samplingProtocol: Sweeping; eventTime: Night
- **Type status:** Other material. **Occurrence:** individualCount: 2; sex: female; **Location:** locationID: P2; continent: Europe; country: Spain; countryCode: ES; stateProvince: Castilla y León; county: León; locality: Joyoguelas; verbatimElevation: 763.98; decimalLatitude: 43.17771; decimalLongitude: -4.90579; geodeticDatum: WGS84; **Event:** eventID: 1; samplingProtocol: Sweeping; eventTime: Night
- **Type status:** Other material. **Occurrence:** individualCount: 1; sex: female; **Location:** locationID: P3; continent: Europe; country: Spain; countryCode: ES; stateProvince: Castilla y León; county: León; locality: Las Arroyas; verbatimElevation: 1097.1; decimalLatitude: 43.14351; decimalLongitude: -4.94878; geodeticDatum: WGS84; **Event:** eventID: 1; samplingProtocol: Aerial; eventTime: Night
- **Type status:** Other material. **Occurrence:** individualCount: 3; sex: female; **Location:** locationID: P3; continent: Europe; country: Spain; countryCode: ES; stateProvince: Castilla y León; county: León; locality: Las Arroyas; verbatimElevation: 1097.1; decimalLatitude: 43.14351; decimalLongitude: -4.94878; geodeticDatum: WGS84; **Event:** eventID: 1; samplingProtocol: Aerial; eventTime: Night
- **Type status:** Other material. **Occurrence:** individualCount: 4; sex: female; **Location:** locationID: P3; continent: Europe; country: Spain; countryCode: ES; stateProvince: Castilla y León; county: León; locality: Las Arroyas; verbatimElevation: 1097.1; decimalLatitude: 43.14351; decimalLongitude: -4.94878; geodeticDatum: WGS84; **Event:** eventID: 2; samplingProtocol: Aerial; eventTime: Night
- **Type status:** Other material. **Occurrence:** individualCount: 1; sex: male; **Location:** locationID: P3; continent: Europe; country: Spain; countryCode: ES; stateProvince: Castilla y León; county: León; locality: Las Arroyas; verbatimElevation: 1097.1; decimalLatitude: 43.14351; decimalLongitude: -4.94878; geodeticDatum: WGS84; **Event:** eventID: 2; samplingProtocol: Aerial; eventTime: Night
- **Type status:** Other material. **Occurrence:** individualCount: 3; sex: female; **Location:** locationID: P3; continent: Europe; country: Spain; countryCode: ES; stateProvince: Castilla y León; county: León; locality: Las Arroyas; verbatimElevation: 1097.1; decimalLatitude: 43.14351; decimalLongitude: -4.94878; geodeticDatum: WGS84; **Event:** eventID: 1; samplingProtocol: Beating; eventTime: Day
- **Type status:** Other material. **Occurrence:** individualCount: 1; sex: male; **Location:** locationID: P3; continent: Europe; country: Spain; countryCode: ES; stateProvince: Castilla y León; county: León; locality: Las Arroyas; verbatimElevation: 1097.1; decimalLatitude: 43.14351; decimalLongitude: -4.94878; geodeticDatum: WGS84; **Event:** eventID: 1; samplingProtocol: Beating; eventTime: Day
- **Type status:** Other material. **Occurrence:** individualCount: 6; sex: female; **Location:** locationID: P3; continent: Europe; country: Spain; countryCode: ES; stateProvince: Castilla y León; county: León; locality: Las Arroyas; verbatimElevation: 1097.1; decimalLatitude: 43.14351; decimalLongitude: -4.94878; geodeticDatum: WGS84; **Event:** eventID: 2; samplingProtocol: Beating; eventTime: Day
- **Type status:** Other material. **Occurrence:** individualCount: 2; sex: male; **Location:** locationID: P3; continent: Europe; country: Spain; countryCode: ES; stateProvince: Castilla y León; county: León; locality: Las Arroyas; verbatimElevation: 1097.1; decimalLatitude: 43.14351; decimalLongitude: -4.94878; geodeticDatum: WGS84; **Event:** eventID: 2; samplingProtocol: Beating; eventTime: Day
- **Type status:** Other material. **Occurrence:** individualCount: 2; sex: female; **Location:** locationID: P3; continent: Europe; country: Spain; countryCode: ES; stateProvince: Castilla y León; county: León; locality: Las Arroyas; verbatimElevation: 1097.1; decimalLatitude: 43.14351; decimalLongitude: -4.94878; geodeticDatum: WGS84; **Event:** eventID: 1; samplingProtocol: Beating; eventTime: Night
- **Type status:** Other material. **Occurrence:** individualCount: 13; sex: female; **Location:** locationID: P3; continent: Europe; country: Spain; countryCode: ES; stateProvince: Castilla y León; county: León; locality: Las Arroyas; verbatimElevation: 1097.1; decimalLatitude: 43.14351; decimalLongitude: -4.94878; geodeticDatum: WGS84; **Event:** eventID: 1; samplingProtocol: Beating; eventTime: Night
- **Type status:** Other material. **Occurrence:** individualCount: 1; sex: female; **Location:** locationID: P3; continent: Europe; country: Spain; countryCode: ES; stateProvince: Castilla y León; county: León; locality: Las Arroyas; verbatimElevation: 1097.1; decimalLatitude: 43.14351; decimalLongitude: -4.94878; geodeticDatum: WGS84; **Event:** eventID: 1; samplingProtocol: Sweeping; eventTime: Day
- **Type status:** Other material. **Occurrence:** individualCount: 5; sex: female; **Location:** locationID: P3; continent: Europe; country: Spain; countryCode: ES; stateProvince: Castilla y León; county: León; locality: Las Arroyas; verbatimElevation: 1097.1; decimalLatitude: 43.14351; decimalLongitude: -4.94878; geodeticDatum: WGS84; **Event:** eventID: 2; samplingProtocol: Sweeping; eventTime: Day
- **Type status:** Other material. **Occurrence:** individualCount: 1; sex: male; **Location:** locationID: P3; continent: Europe; country: Spain; countryCode: ES; stateProvince: Castilla y León; county: León; locality: Las Arroyas; verbatimElevation: 1097.1; decimalLatitude: 43.14351; decimalLongitude: -4.94878; geodeticDatum: WGS84; **Event:** eventID: 2; samplingProtocol: Sweeping; eventTime: Day
- **Type status:** Other material. **Occurrence:** individualCount: 3; sex: female; **Location:** locationID: P3; continent: Europe; country: Spain; countryCode: ES; stateProvince: Castilla y León; county: León; locality: Las Arroyas; verbatimElevation: 1097.1; decimalLatitude: 43.14351; decimalLongitude: -4.94878; geodeticDatum: WGS84; **Event:** eventID: 1; samplingProtocol: Sweeping; eventTime: Night
- **Type status:** Other material. **Occurrence:** individualCount: 1; sex: male; **Location:** locationID: P3; continent: Europe; country: Spain; countryCode: ES; stateProvince: Castilla y León; county: León; locality: Las Arroyas; verbatimElevation: 1097.1; decimalLatitude: 43.14351; decimalLongitude: -4.94878; geodeticDatum: WGS84; **Event:** eventID: 1; samplingProtocol: Sweeping; eventTime: Night
- **Type status:** Other material. **Occurrence:** individualCount: 1; sex: female; **Location:** locationID: P3; continent: Europe; country: Spain; countryCode: ES; stateProvince: Castilla y León; county: León; locality: Las Arroyas; verbatimElevation: 1097.1; decimalLatitude: 43.14351; decimalLongitude: -4.94878; geodeticDatum: WGS84; **Event:** eventID: 1; samplingProtocol: Sweeping; eventTime: Night
- **Type status:** Other material. **Occurrence:** individualCount: 3; sex: female; **Location:** locationID: P4; continent: Europe; country: Spain; countryCode: ES; stateProvince: Castilla y León; county: León; locality: El Canto; verbatimElevation: 943.48; decimalLatitude: 43.17227; decimalLongitude: -4.90857; geodeticDatum: WGS84; **Event:** eventID: 2; samplingProtocol: Aerial; eventTime: Night
- **Type status:** Other material. **Occurrence:** individualCount: 2; sex: female; **Location:** locationID: P4; continent: Europe; country: Spain; countryCode: ES; stateProvince: Castilla y León; county: León; locality: El Canto; verbatimElevation: 943.48; decimalLatitude: 43.17227; decimalLongitude: -4.90857; geodeticDatum: WGS84; **Event:** eventID: 1; samplingProtocol: Beating; eventTime: Day
- **Type status:** Other material. **Occurrence:** individualCount: 18; sex: female; **Location:** locationID: P4; continent: Europe; country: Spain; countryCode: ES; stateProvince: Castilla y León; county: León; locality: El Canto; verbatimElevation: 943.48; decimalLatitude: 43.17227; decimalLongitude: -4.90857; geodeticDatum: WGS84; **Event:** eventID: 2; samplingProtocol: Beating; eventTime: Day
- **Type status:** Other material. **Occurrence:** individualCount: 2; sex: male; **Location:** locationID: P4; continent: Europe; country: Spain; countryCode: ES; stateProvince: Castilla y León; county: León; locality: El Canto; verbatimElevation: 943.48; decimalLatitude: 43.17227; decimalLongitude: -4.90857; geodeticDatum: WGS84; **Event:** eventID: 2; samplingProtocol: Beating; eventTime: Day
- **Type status:** Other material. **Occurrence:** individualCount: 1; sex: female; **Location:** locationID: P4; continent: Europe; country: Spain; countryCode: ES; stateProvince: Castilla y León; county: León; locality: El Canto; verbatimElevation: 943.48; decimalLatitude: 43.17227; decimalLongitude: -4.90857; geodeticDatum: WGS84; **Event:** eventID: 1; samplingProtocol: Beating; eventTime: Night
- **Type status:** Other material. **Occurrence:** individualCount: 1; sex: male; **Location:** locationID: P4; continent: Europe; country: Spain; countryCode: ES; stateProvince: Castilla y León; county: León; locality: El Canto; verbatimElevation: 943.48; decimalLatitude: 43.17227; decimalLongitude: -4.90857; geodeticDatum: WGS84; **Event:** eventID: 1; samplingProtocol: Beating; eventTime: Night

##### Distribution

Europe, Algeria, Russia

#### Parasteatoda lunata

(Clerck, 1757)

##### Materials

- **Type status:** Other material. **Occurrence:** individualCount: 2; sex: male; **Location:** locationID: A1; continent: Europe; country: Spain; countryCode: ES; stateProvince: Catalonia; county: Lleida; locality: Sola de Boi; verbatimElevation: 1759.8; decimalLatitude: 42.54958; decimalLongitude: 0.87254; geodeticDatum: WGS84; **Event:** eventID: 1; samplingProtocol: Aerial; eventTime: Night
- **Type status:** Other material. **Occurrence:** individualCount: 2; sex: female; **Location:** locationID: A1; continent: Europe; country: Spain; countryCode: ES; stateProvince: Catalonia; county: Lleida; locality: Sola de Boi; verbatimElevation: 1759.8; decimalLatitude: 42.54958; decimalLongitude: 0.87254; geodeticDatum: WGS84; **Event:** eventID: 1; samplingProtocol: Aerial; eventTime: Night
- **Type status:** Other material. **Occurrence:** individualCount: 1; sex: male; **Location:** locationID: A1; continent: Europe; country: Spain; countryCode: ES; stateProvince: Catalonia; county: Lleida; locality: Sola de Boi; verbatimElevation: 1759.8; decimalLatitude: 42.54958; decimalLongitude: 0.87254; geodeticDatum: WGS84; **Event:** eventID: 1; samplingProtocol: Aerial; eventTime: Night
- **Type status:** Other material. **Occurrence:** individualCount: 1; sex: male; **Location:** locationID: A1; continent: Europe; country: Spain; countryCode: ES; stateProvince: Catalonia; county: Lleida; locality: Sola de Boi; verbatimElevation: 1759.8; decimalLatitude: 42.54958; decimalLongitude: 0.87254; geodeticDatum: WGS84; **Event:** eventID: 2; samplingProtocol: Aerial; eventTime: Night
- **Type status:** Other material. **Occurrence:** individualCount: 1; sex: male; **Location:** locationID: A1; continent: Europe; country: Spain; countryCode: ES; stateProvince: Catalonia; county: Lleida; locality: Sola de Boi; verbatimElevation: 1759.8; decimalLatitude: 42.54958; decimalLongitude: 0.87254; geodeticDatum: WGS84; **Event:** eventID: 1; samplingProtocol: Beating; eventTime: Night
- **Type status:** Other material. **Occurrence:** individualCount: 1; sex: female; **Location:** locationID: A1; continent: Europe; country: Spain; countryCode: ES; stateProvince: Catalonia; county: Lleida; locality: Sola de Boi; verbatimElevation: 1759.8; decimalLatitude: 42.54958; decimalLongitude: 0.87254; geodeticDatum: WGS84; **Event:** eventID: 2; samplingProtocol: Ground; eventTime: Night
- **Type status:** Other material. **Occurrence:** individualCount: 1; sex: female; **Location:** locationID: A2; continent: Europe; country: Spain; countryCode: ES; stateProvince: Catalonia; county: Lleida; locality: Sola de Boi; verbatimElevation: 1738.7; decimalLatitude: 42.54913; decimalLongitude: 0.87137; geodeticDatum: WGS84; **Event:** eventID: 1; samplingProtocol: Aerial; eventTime: Night
- **Type status:** Other material. **Occurrence:** individualCount: 1; sex: male; **Location:** locationID: A2; continent: Europe; country: Spain; countryCode: ES; stateProvince: Catalonia; county: Lleida; locality: Sola de Boi; verbatimElevation: 1738.7; decimalLatitude: 42.54913; decimalLongitude: 0.87137; geodeticDatum: WGS84; **Event:** eventID: 1; samplingProtocol: Aerial; eventTime: Night
- **Type status:** Other material. **Occurrence:** individualCount: 2; sex: female; **Location:** locationID: A2; continent: Europe; country: Spain; countryCode: ES; stateProvince: Catalonia; county: Lleida; locality: Sola de Boi; verbatimElevation: 1738.7; decimalLatitude: 42.54913; decimalLongitude: 0.87137; geodeticDatum: WGS84; **Event:** eventID: 2; samplingProtocol: Aerial; eventTime: Night
- **Type status:** Other material. **Occurrence:** individualCount: 1; sex: male; **Location:** locationID: A2; continent: Europe; country: Spain; countryCode: ES; stateProvince: Catalonia; county: Lleida; locality: Sola de Boi; verbatimElevation: 1738.7; decimalLatitude: 42.54913; decimalLongitude: 0.87137; geodeticDatum: WGS84; **Event:** eventID: 2; samplingProtocol: Aerial; eventTime: Night
- **Type status:** Other material. **Occurrence:** individualCount: 1; sex: female; **Location:** locationID: A2; continent: Europe; country: Spain; countryCode: ES; stateProvince: Catalonia; county: Lleida; locality: Sola de Boi; verbatimElevation: 1738.7; decimalLatitude: 42.54913; decimalLongitude: 0.87137; geodeticDatum: WGS84; **Event:** eventID: 1; samplingProtocol: Sweeping; eventTime: Night
- **Type status:** Other material. **Occurrence:** individualCount: 1; sex: male; **Location:** locationID: O1; continent: Europe; country: Spain; countryCode: ES; stateProvince: Aragón; county: Huesca; locality: O Furno; verbatimElevation: 1396.73; decimalLatitude: 42.60677; decimalLongitude: 0.13135; geodeticDatum: WGS84; **Event:** eventID: 1; samplingProtocol: Sweeping; eventTime: Night
- **Type status:** Other material. **Occurrence:** individualCount: 1; sex: female; **Location:** locationID: O2; continent: Europe; country: Spain; countryCode: ES; stateProvince: Aragón; county: Huesca; locality: Rebilla; verbatimElevation: 1158.13; decimalLatitude: 42.59427; decimalLongitude: 0.1529; geodeticDatum: WGS84; **Event:** eventID: 2; samplingProtocol: Aerial; eventTime: Night
- **Type status:** Other material. **Occurrence:** individualCount: 2; sex: female; **Location:** locationID: P1; continent: Europe; country: Spain; countryCode: ES; stateProvince: Castilla y León; county: León; locality: Monte Robledo; verbatimElevation: 1071.58; decimalLatitude: 43.1445; decimalLongitude: -4.92675; geodeticDatum: WGS84; **Event:** eventID: 1; samplingProtocol: Aerial; eventTime: Night
- **Type status:** Other material. **Occurrence:** individualCount: 2; sex: male; **Location:** locationID: P1; continent: Europe; country: Spain; countryCode: ES; stateProvince: Castilla y León; county: León; locality: Monte Robledo; verbatimElevation: 1071.58; decimalLatitude: 43.1445; decimalLongitude: -4.92675; geodeticDatum: WGS84; **Event:** eventID: 1; samplingProtocol: Aerial; eventTime: Night
- **Type status:** Other material. **Occurrence:** individualCount: 1; sex: male; **Location:** locationID: P1; continent: Europe; country: Spain; countryCode: ES; stateProvince: Castilla y León; county: León; locality: Monte Robledo; verbatimElevation: 1071.58; decimalLatitude: 43.1445; decimalLongitude: -4.92675; geodeticDatum: WGS84; **Event:** eventID: 1; samplingProtocol: Aerial; eventTime: Night
- **Type status:** Other material. **Occurrence:** individualCount: 1; sex: male; **Location:** locationID: P1; continent: Europe; country: Spain; countryCode: ES; stateProvince: Castilla y León; county: León; locality: Monte Robledo; verbatimElevation: 1071.58; decimalLatitude: 43.1445; decimalLongitude: -4.92675; geodeticDatum: WGS84; **Event:** eventID: 1; samplingProtocol: Beating; eventTime: Night
- **Type status:** Other material. **Occurrence:** individualCount: 7; sex: female; **Location:** locationID: P2; continent: Europe; country: Spain; countryCode: ES; stateProvince: Castilla y León; county: León; locality: Joyoguelas; verbatimElevation: 763.98; decimalLatitude: 43.17771; decimalLongitude: -4.90579; geodeticDatum: WGS84; **Event:** eventID: 1; samplingProtocol: Aerial; eventTime: Night
- **Type status:** Other material. **Occurrence:** individualCount: 1; sex: female; **Location:** locationID: P2; continent: Europe; country: Spain; countryCode: ES; stateProvince: Castilla y León; county: León; locality: Joyoguelas; verbatimElevation: 763.98; decimalLatitude: 43.17771; decimalLongitude: -4.90579; geodeticDatum: WGS84; **Event:** eventID: 1; samplingProtocol: Aerial; eventTime: Night
- **Type status:** Other material. **Occurrence:** individualCount: 3; sex: female; **Location:** locationID: P2; continent: Europe; country: Spain; countryCode: ES; stateProvince: Castilla y León; county: León; locality: Joyoguelas; verbatimElevation: 763.98; decimalLatitude: 43.17771; decimalLongitude: -4.90579; geodeticDatum: WGS84; **Event:** eventID: 2; samplingProtocol: Aerial; eventTime: Night
- **Type status:** Other material. **Occurrence:** individualCount: 1; sex: female; **Location:** locationID: P2; continent: Europe; country: Spain; countryCode: ES; stateProvince: Castilla y León; county: León; locality: Joyoguelas; verbatimElevation: 763.98; decimalLatitude: 43.17771; decimalLongitude: -4.90579; geodeticDatum: WGS84; **Event:** eventID: 2; samplingProtocol: Aerial; eventTime: Night
- **Type status:** Other material. **Occurrence:** individualCount: 1; sex: female; **Location:** locationID: P3; continent: Europe; country: Spain; countryCode: ES; stateProvince: Castilla y León; county: León; locality: Las Arroyas; verbatimElevation: 1097.1; decimalLatitude: 43.14351; decimalLongitude: -4.94878; geodeticDatum: WGS84; **Event:** eventID: 2; samplingProtocol: Aerial; eventTime: Night
- **Type status:** Other material. **Occurrence:** individualCount: 3; sex: female; **Location:** locationID: P4; continent: Europe; country: Spain; countryCode: ES; stateProvince: Castilla y León; county: León; locality: El Canto; verbatimElevation: 943.48; decimalLatitude: 43.17227; decimalLongitude: -4.90857; geodeticDatum: WGS84; **Event:** eventID: 1; samplingProtocol: Aerial; eventTime: Night
- **Type status:** Other material. **Occurrence:** individualCount: 1; sex: male; **Location:** locationID: P4; continent: Europe; country: Spain; countryCode: ES; stateProvince: Castilla y León; county: León; locality: El Canto; verbatimElevation: 943.48; decimalLatitude: 43.17227; decimalLongitude: -4.90857; geodeticDatum: WGS84; **Event:** eventID: 1; samplingProtocol: Aerial; eventTime: Night
- **Type status:** Other material. **Occurrence:** individualCount: 1; sex: female; **Location:** locationID: P4; continent: Europe; country: Spain; countryCode: ES; stateProvince: Castilla y León; county: León; locality: El Canto; verbatimElevation: 943.48; decimalLatitude: 43.17227; decimalLongitude: -4.90857; geodeticDatum: WGS84; **Event:** eventID: 2; samplingProtocol: Aerial; eventTime: Night

##### Distribution

Palearctic

#### Pholcomma gibbum

(Westring, 1851)

##### Materials

- **Type status:** Other material. **Occurrence:** individualCount: 1; sex: male; **Location:** locationID: O1; continent: Europe; country: Spain; countryCode: ES; stateProvince: Aragón; county: Huesca; locality: O Furno; verbatimElevation: 1396.73; decimalLatitude: 42.60677; decimalLongitude: 0.13135; geodeticDatum: WGS84; **Event:** eventID: A; samplingProtocol: Pitfall
- **Type status:** Other material. **Occurrence:** individualCount: 1; sex: male; **Location:** locationID: O1; continent: Europe; country: Spain; countryCode: ES; stateProvince: Aragón; county: Huesca; locality: O Furno; verbatimElevation: 1396.73; decimalLatitude: 42.60677; decimalLongitude: 0.13135; geodeticDatum: WGS84; **Event:** eventID: E; samplingProtocol: Pitfall
- **Type status:** Other material. **Occurrence:** individualCount: 1; sex: male; **Location:** locationID: O1; continent: Europe; country: Spain; countryCode: ES; stateProvince: Aragón; county: Huesca; locality: O Furno; verbatimElevation: 1396.73; decimalLatitude: 42.60677; decimalLongitude: 0.13135; geodeticDatum: WGS84; **Event:** eventID: I; samplingProtocol: Pitfall
- **Type status:** Other material. **Occurrence:** individualCount: 1; sex: female; **Location:** locationID: P2; continent: Europe; country: Spain; countryCode: ES; stateProvince: Castilla y León; county: León; locality: Joyoguelas; verbatimElevation: 763.98; decimalLatitude: 43.17771; decimalLongitude: -4.90579; geodeticDatum: WGS84; **Event:** eventID: B; samplingProtocol: Pitfall

##### Distribution

Europe, North Africa to Azerbaijan

#### Phoroncidia paradoxa

(Lucas, 1846)

##### Materials

- **Type status:** Other material. **Occurrence:** individualCount: 1; sex: female; **Location:** locationID: C3; continent: Europe; country: Spain; countryCode: ES; stateProvince: Castilla-La Mancha; county: Ciudad Real; locality: La Quesera; verbatimElevation: 767.55; decimalLatitude: 39.36177; decimalLongitude: -4.41733; geodeticDatum: WGS84; **Event:** eventID: 4; samplingProtocol: Aerial; eventTime: Night
- **Type status:** Other material. **Occurrence:** individualCount: 1; sex: female; **Location:** locationID: O2; continent: Europe; country: Spain; countryCode: ES; stateProvince: Aragón; county: Huesca; locality: Rebilla; verbatimElevation: 1158.13; decimalLatitude: 42.59427; decimalLongitude: 0.1529; geodeticDatum: WGS84; **Event:** eventID: 2; samplingProtocol: Aerial; eventTime: Night

##### Distribution

Europe, North Africa

#### Phycosoma inornatum

(O. Pickard-Cambridge, 1861)

##### Materials

- **Type status:** Other material. **Occurrence:** individualCount: 1; sex: male; **Location:** locationID: A1; continent: Europe; country: Spain; countryCode: ES; stateProvince: Catalonia; county: Lleida; locality: Sola de Boi; verbatimElevation: 1759.8; decimalLatitude: 42.54958; decimalLongitude: 0.87254; geodeticDatum: WGS84; **Event:** eventID: 1; samplingProtocol: Ground; eventTime: Night
- **Type status:** Other material. **Occurrence:** individualCount: 1; sex: male; **Location:** locationID: A1; continent: Europe; country: Spain; countryCode: ES; stateProvince: Catalonia; county: Lleida; locality: Sola de Boi; verbatimElevation: 1759.8; decimalLatitude: 42.54958; decimalLongitude: 0.87254; geodeticDatum: WGS84; **Event:** eventID: 1; samplingProtocol: Sweeping; eventTime: Day
- **Type status:** Other material. **Occurrence:** individualCount: 1; sex: female; **Location:** locationID: A2; continent: Europe; country: Spain; countryCode: ES; stateProvince: Catalonia; county: Lleida; locality: Sola de Boi; verbatimElevation: 1738.7; decimalLatitude: 42.54913; decimalLongitude: 0.87137; geodeticDatum: WGS84; **Event:** eventID: 1; samplingProtocol: Aerial; eventTime: Night
- **Type status:** Other material. **Occurrence:** individualCount: 1; sex: male; **Location:** locationID: A2; continent: Europe; country: Spain; countryCode: ES; stateProvince: Catalonia; county: Lleida; locality: Sola de Boi; verbatimElevation: 1738.7; decimalLatitude: 42.54913; decimalLongitude: 0.87137; geodeticDatum: WGS84; **Event:** eventID: 1; samplingProtocol: Aerial; eventTime: Night

##### Distribution

Europe to Azerbaijan

#### Phylloneta impressa

(L. Koch, 1881)

##### Materials

- **Type status:** Other material. **Occurrence:** individualCount: 1; sex: male; **Location:** locationID: C4; continent: Europe; country: Spain; countryCode: ES; stateProvince: Castilla-La Mancha; county: Ciudad Real; locality: La Quesera; verbatimElevation: 772.3; decimalLatitude: 39.36337; decimalLongitude: -4.41704; geodeticDatum: WGS84; **Event:** eventID: 2; samplingProtocol: Aerial; eventTime: Night
- **Type status:** Other material. **Occurrence:** individualCount: 1; sex: male; **Location:** locationID: O2; continent: Europe; country: Spain; countryCode: ES; stateProvince: Aragón; county: Huesca; locality: Rebilla; verbatimElevation: 1158.13; decimalLatitude: 42.59427; decimalLongitude: 0.1529; geodeticDatum: WGS84; **Event:** eventID: 1; samplingProtocol: Beating; eventTime: Night
- **Type status:** Other material. **Occurrence:** individualCount: 1; sex: male; **Location:** locationID: O2; continent: Europe; country: Spain; countryCode: ES; stateProvince: Aragón; county: Huesca; locality: Rebilla; verbatimElevation: 1158.13; decimalLatitude: 42.59427; decimalLongitude: 0.1529; geodeticDatum: WGS84; **Event:** eventID: 2; samplingProtocol: Sweeping; eventTime: Day

##### Distribution

Holarctic

#### Phylloneta sisyphia

(Clerck, 1757)

##### Materials

- **Type status:** Other material. **Occurrence:** individualCount: 2; sex: male; **Location:** locationID: A1; continent: Europe; country: Spain; countryCode: ES; stateProvince: Catalonia; county: Lleida; locality: Sola de Boi; verbatimElevation: 1759.8; decimalLatitude: 42.54958; decimalLongitude: 0.87254; geodeticDatum: WGS84; **Event:** eventID: 1; samplingProtocol: Beating; eventTime: Day
- **Type status:** Other material. **Occurrence:** individualCount: 2; sex: female; **Location:** locationID: A1; continent: Europe; country: Spain; countryCode: ES; stateProvince: Catalonia; county: Lleida; locality: Sola de Boi; verbatimElevation: 1759.8; decimalLatitude: 42.54958; decimalLongitude: 0.87254; geodeticDatum: WGS84; **Event:** eventID: 2; samplingProtocol: Beating; eventTime: Day
- **Type status:** Other material. **Occurrence:** individualCount: 1; sex: male; **Location:** locationID: A1; continent: Europe; country: Spain; countryCode: ES; stateProvince: Catalonia; county: Lleida; locality: Sola de Boi; verbatimElevation: 1759.8; decimalLatitude: 42.54958; decimalLongitude: 0.87254; geodeticDatum: WGS84; **Event:** eventID: 1; samplingProtocol: Beating; eventTime: Night
- **Type status:** Other material. **Occurrence:** individualCount: 1; sex: female; **Location:** locationID: A1; continent: Europe; country: Spain; countryCode: ES; stateProvince: Catalonia; county: Lleida; locality: Sola de Boi; verbatimElevation: 1759.8; decimalLatitude: 42.54958; decimalLongitude: 0.87254; geodeticDatum: WGS84; **Event:** eventID: 1; samplingProtocol: Sweeping; eventTime: Night
- **Type status:** Other material. **Occurrence:** individualCount: 1; sex: female; **Location:** locationID: A2; continent: Europe; country: Spain; countryCode: ES; stateProvince: Catalonia; county: Lleida; locality: Sola de Boi; verbatimElevation: 1738.7; decimalLatitude: 42.54913; decimalLongitude: 0.87137; geodeticDatum: WGS84; **Event:** eventID: 1; samplingProtocol: Aerial; eventTime: Night
- **Type status:** Other material. **Occurrence:** individualCount: 1; sex: male; **Location:** locationID: A2; continent: Europe; country: Spain; countryCode: ES; stateProvince: Catalonia; county: Lleida; locality: Sola de Boi; verbatimElevation: 1738.7; decimalLatitude: 42.54913; decimalLongitude: 0.87137; geodeticDatum: WGS84; **Event:** eventID: 1; samplingProtocol: Beating; eventTime: Day
- **Type status:** Other material. **Occurrence:** individualCount: 1; sex: female; **Location:** locationID: A2; continent: Europe; country: Spain; countryCode: ES; stateProvince: Catalonia; county: Lleida; locality: Sola de Boi; verbatimElevation: 1738.7; decimalLatitude: 42.54913; decimalLongitude: 0.87137; geodeticDatum: WGS84; **Event:** eventID: 2; samplingProtocol: Sweeping; eventTime: Day
- **Type status:** Other material. **Occurrence:** individualCount: 3; sex: male; **Location:** locationID: A2; continent: Europe; country: Spain; countryCode: ES; stateProvince: Catalonia; county: Lleida; locality: Sola de Boi; verbatimElevation: 1738.7; decimalLatitude: 42.54913; decimalLongitude: 0.87137; geodeticDatum: WGS84; **Event:** eventID: 2; samplingProtocol: Sweeping; eventTime: Day
- **Type status:** Other material. **Occurrence:** individualCount: 1; sex: female; **Location:** locationID: A2; continent: Europe; country: Spain; countryCode: ES; stateProvince: Catalonia; county: Lleida; locality: Sola de Boi; verbatimElevation: 1738.7; decimalLatitude: 42.54913; decimalLongitude: 0.87137; geodeticDatum: WGS84; **Event:** eventID: 1; samplingProtocol: Sweeping; eventTime: Night
- **Type status:** Other material. **Occurrence:** individualCount: 1; sex: male; **Location:** locationID: A2; continent: Europe; country: Spain; countryCode: ES; stateProvince: Catalonia; county: Lleida; locality: Sola de Boi; verbatimElevation: 1738.7; decimalLatitude: 42.54913; decimalLongitude: 0.87137; geodeticDatum: WGS84; **Event:** eventID: 1; samplingProtocol: Sweeping; eventTime: Night
- **Type status:** Other material. **Occurrence:** individualCount: 1; sex: female; **Location:** locationID: A2; continent: Europe; country: Spain; countryCode: ES; stateProvince: Catalonia; county: Lleida; locality: Sola de Boi; verbatimElevation: 1738.7; decimalLatitude: 42.54913; decimalLongitude: 0.87137; geodeticDatum: WGS84; **Event:** eventID: 1; samplingProtocol: Sweeping; eventTime: Night
- **Type status:** Other material. **Occurrence:** individualCount: 1; sex: male; **Location:** locationID: O1; continent: Europe; country: Spain; countryCode: ES; stateProvince: Aragón; county: Huesca; locality: O Furno; verbatimElevation: 1396.73; decimalLatitude: 42.60677; decimalLongitude: 0.13135; geodeticDatum: WGS84; **Event:** eventID: 1; samplingProtocol: Beating; eventTime: Night

##### Distribution

Palearctic

#### Platnickina tincta

(Walckenaer, 1802)

##### Materials

- **Type status:** Other material. **Occurrence:** individualCount: 1; sex: male; **Location:** locationID: A1; continent: Europe; country: Spain; countryCode: ES; stateProvince: Catalonia; county: Lleida; locality: Sola de Boi; verbatimElevation: 1759.8; decimalLatitude: 42.54958; decimalLongitude: 0.87254; geodeticDatum: WGS84; **Event:** eventID: 1; samplingProtocol: Beating; eventTime: Day
- **Type status:** Other material. **Occurrence:** individualCount: 2; sex: female; **Location:** locationID: C1; continent: Europe; country: Spain; countryCode: ES; stateProvince: Castilla-La Mancha; county: Ciudad Real; locality: Valle Brezoso; verbatimElevation: 756.56; decimalLatitude: 39.35663; decimalLongitude: -4.35912; geodeticDatum: WGS84; **Event:** eventID: 3; samplingProtocol: Aerial; eventTime: Night
- **Type status:** Other material. **Occurrence:** individualCount: 2; sex: female; **Location:** locationID: C1; continent: Europe; country: Spain; countryCode: ES; stateProvince: Castilla-La Mancha; county: Ciudad Real; locality: Valle Brezoso; verbatimElevation: 756.56; decimalLatitude: 39.35663; decimalLongitude: -4.35912; geodeticDatum: WGS84; **Event:** eventID: 1; samplingProtocol: Beating; eventTime: Night
- **Type status:** Other material. **Occurrence:** individualCount: 3; sex: female; **Location:** locationID: C1; continent: Europe; country: Spain; countryCode: ES; stateProvince: Castilla-La Mancha; county: Ciudad Real; locality: Valle Brezoso; verbatimElevation: 756.56; decimalLatitude: 39.35663; decimalLongitude: -4.35912; geodeticDatum: WGS84; **Event:** eventID: 2; samplingProtocol: Beating; eventTime: Night
- **Type status:** Other material. **Occurrence:** individualCount: 1; sex: male; **Location:** locationID: C1; continent: Europe; country: Spain; countryCode: ES; stateProvince: Castilla-La Mancha; county: Ciudad Real; locality: Valle Brezoso; verbatimElevation: 756.56; decimalLatitude: 39.35663; decimalLongitude: -4.35912; geodeticDatum: WGS84; **Event:** eventID: 1; samplingProtocol: Sweeping; eventTime: Day
- **Type status:** Other material. **Occurrence:** individualCount: 1; sex: female; **Location:** locationID: C2; continent: Europe; country: Spain; countryCode: ES; stateProvince: Castilla-La Mancha; county: Ciudad Real; locality: Valle Brezoso; verbatimElevation: 739.31; decimalLatitude: 39.35159; decimalLongitude: -4.3589; geodeticDatum: WGS84; **Event:** eventID: 1; samplingProtocol: Aerial; eventTime: Night
- **Type status:** Other material. **Occurrence:** individualCount: 1; sex: female; **Location:** locationID: C2; continent: Europe; country: Spain; countryCode: ES; stateProvince: Castilla-La Mancha; county: Ciudad Real; locality: Valle Brezoso; verbatimElevation: 739.31; decimalLatitude: 39.35159; decimalLongitude: -4.3589; geodeticDatum: WGS84; **Event:** eventID: 2; samplingProtocol: Aerial; eventTime: Night
- **Type status:** Other material. **Occurrence:** individualCount: 1; sex: female; **Location:** locationID: C2; continent: Europe; country: Spain; countryCode: ES; stateProvince: Castilla-La Mancha; county: Ciudad Real; locality: Valle Brezoso; verbatimElevation: 739.31; decimalLatitude: 39.35159; decimalLongitude: -4.3589; geodeticDatum: WGS84; **Event:** eventID: 4; samplingProtocol: Aerial; eventTime: Night
- **Type status:** Other material. **Occurrence:** individualCount: 1; sex: male; **Location:** locationID: C2; continent: Europe; country: Spain; countryCode: ES; stateProvince: Castilla-La Mancha; county: Ciudad Real; locality: Valle Brezoso; verbatimElevation: 739.31; decimalLatitude: 39.35159; decimalLongitude: -4.3589; geodeticDatum: WGS84; **Event:** eventID: 4; samplingProtocol: Aerial; eventTime: Night
- **Type status:** Other material. **Occurrence:** individualCount: 2; sex: female; **Location:** locationID: C2; continent: Europe; country: Spain; countryCode: ES; stateProvince: Castilla-La Mancha; county: Ciudad Real; locality: Valle Brezoso; verbatimElevation: 739.31; decimalLatitude: 39.35159; decimalLongitude: -4.3589; geodeticDatum: WGS84; **Event:** eventID: 1; samplingProtocol: Beating; eventTime: Day
- **Type status:** Other material. **Occurrence:** individualCount: 1; sex: male; **Location:** locationID: C2; continent: Europe; country: Spain; countryCode: ES; stateProvince: Castilla-La Mancha; county: Ciudad Real; locality: Valle Brezoso; verbatimElevation: 739.31; decimalLatitude: 39.35159; decimalLongitude: -4.3589; geodeticDatum: WGS84; **Event:** eventID: 1; samplingProtocol: Beating; eventTime: Day
- **Type status:** Other material. **Occurrence:** individualCount: 3; sex: female; **Location:** locationID: C2; continent: Europe; country: Spain; countryCode: ES; stateProvince: Castilla-La Mancha; county: Ciudad Real; locality: Valle Brezoso; verbatimElevation: 739.31; decimalLatitude: 39.35159; decimalLongitude: -4.3589; geodeticDatum: WGS84; **Event:** eventID: 2; samplingProtocol: Beating; eventTime: Day
- **Type status:** Other material. **Occurrence:** individualCount: 1; sex: female; **Location:** locationID: C2; continent: Europe; country: Spain; countryCode: ES; stateProvince: Castilla-La Mancha; county: Ciudad Real; locality: Valle Brezoso; verbatimElevation: 739.31; decimalLatitude: 39.35159; decimalLongitude: -4.3589; geodeticDatum: WGS84; **Event:** eventID: 1; samplingProtocol: Beating; eventTime: Night
- **Type status:** Other material. **Occurrence:** individualCount: 2; sex: female; **Location:** locationID: C2; continent: Europe; country: Spain; countryCode: ES; stateProvince: Castilla-La Mancha; county: Ciudad Real; locality: Valle Brezoso; verbatimElevation: 739.31; decimalLatitude: 39.35159; decimalLongitude: -4.3589; geodeticDatum: WGS84; **Event:** eventID: 1; samplingProtocol: Sweeping; eventTime: Day
- **Type status:** Other material. **Occurrence:** individualCount: 1; sex: male; **Location:** locationID: C2; continent: Europe; country: Spain; countryCode: ES; stateProvince: Castilla-La Mancha; county: Ciudad Real; locality: Valle Brezoso; verbatimElevation: 739.31; decimalLatitude: 39.35159; decimalLongitude: -4.3589; geodeticDatum: WGS84; **Event:** eventID: 2; samplingProtocol: Sweeping; eventTime: Day
- **Type status:** Other material. **Occurrence:** individualCount: 2; sex: female; **Location:** locationID: C2; continent: Europe; country: Spain; countryCode: ES; stateProvince: Castilla-La Mancha; county: Ciudad Real; locality: Valle Brezoso; verbatimElevation: 739.31; decimalLatitude: 39.35159; decimalLongitude: -4.3589; geodeticDatum: WGS84; **Event:** eventID: 2; samplingProtocol: Sweeping; eventTime: Night
- **Type status:** Other material. **Occurrence:** individualCount: 1; sex: female; **Location:** locationID: C3; continent: Europe; country: Spain; countryCode: ES; stateProvince: Castilla-La Mancha; county: Ciudad Real; locality: La Quesera; verbatimElevation: 767.55; decimalLatitude: 39.36177; decimalLongitude: -4.41733; geodeticDatum: WGS84; **Event:** eventID: 1; samplingProtocol: Aerial; eventTime: Night
- **Type status:** Other material. **Occurrence:** individualCount: 1; sex: female; **Location:** locationID: C3; continent: Europe; country: Spain; countryCode: ES; stateProvince: Castilla-La Mancha; county: Ciudad Real; locality: La Quesera; verbatimElevation: 767.55; decimalLatitude: 39.36177; decimalLongitude: -4.41733; geodeticDatum: WGS84; **Event:** eventID: 2; samplingProtocol: Beating; eventTime: Day
- **Type status:** Other material. **Occurrence:** individualCount: 2; sex: female; **Location:** locationID: C3; continent: Europe; country: Spain; countryCode: ES; stateProvince: Castilla-La Mancha; county: Ciudad Real; locality: La Quesera; verbatimElevation: 767.55; decimalLatitude: 39.36177; decimalLongitude: -4.41733; geodeticDatum: WGS84; **Event:** eventID: 2; samplingProtocol: Beating; eventTime: Night
- **Type status:** Other material. **Occurrence:** individualCount: 1; sex: male; **Location:** locationID: C3; continent: Europe; country: Spain; countryCode: ES; stateProvince: Castilla-La Mancha; county: Ciudad Real; locality: La Quesera; verbatimElevation: 767.55; decimalLatitude: 39.36177; decimalLongitude: -4.41733; geodeticDatum: WGS84; **Event:** eventID: 2; samplingProtocol: Beating; eventTime: Night
- **Type status:** Other material. **Occurrence:** individualCount: 2; sex: female; **Location:** locationID: C4; continent: Europe; country: Spain; countryCode: ES; stateProvince: Castilla-La Mancha; county: Ciudad Real; locality: La Quesera; verbatimElevation: 772.3; decimalLatitude: 39.36337; decimalLongitude: -4.41704; geodeticDatum: WGS84; **Event:** eventID: 4; samplingProtocol: Aerial; eventTime: Night
- **Type status:** Other material. **Occurrence:** individualCount: 1; sex: male; **Location:** locationID: C4; continent: Europe; country: Spain; countryCode: ES; stateProvince: Castilla-La Mancha; county: Ciudad Real; locality: La Quesera; verbatimElevation: 772.3; decimalLatitude: 39.36337; decimalLongitude: -4.41704; geodeticDatum: WGS84; **Event:** eventID: 1; samplingProtocol: Beating; eventTime: Day
- **Type status:** Other material. **Occurrence:** individualCount: 1; sex: female; **Location:** locationID: C4; continent: Europe; country: Spain; countryCode: ES; stateProvince: Castilla-La Mancha; county: Ciudad Real; locality: La Quesera; verbatimElevation: 772.3; decimalLatitude: 39.36337; decimalLongitude: -4.41704; geodeticDatum: WGS84; **Event:** eventID: 2; samplingProtocol: Beating; eventTime: Day
- **Type status:** Other material. **Occurrence:** individualCount: 1; sex: female; **Location:** locationID: M2; continent: Europe; country: Spain; countryCode: ES; stateProvince: Extremadura; county: Cáceres; locality: Fuente del Frances; verbatimElevation: 320.72; decimalLatitude: 39.828; decimalLongitude: -6.03249; geodeticDatum: WGS84; **Event:** eventID: 1; samplingProtocol: Beating; eventTime: Night
- **Type status:** Other material. **Occurrence:** individualCount: 1; sex: female; **Location:** locationID: O1; continent: Europe; country: Spain; countryCode: ES; stateProvince: Aragón; county: Huesca; locality: O Furno; verbatimElevation: 1396.73; decimalLatitude: 42.60677; decimalLongitude: 0.13135; geodeticDatum: WGS84; **Event:** eventID: 1; samplingProtocol: Aerial; eventTime: Night
- **Type status:** Other material. **Occurrence:** individualCount: 5; sex: male; **Location:** locationID: O1; continent: Europe; country: Spain; countryCode: ES; stateProvince: Aragón; county: Huesca; locality: O Furno; verbatimElevation: 1396.73; decimalLatitude: 42.60677; decimalLongitude: 0.13135; geodeticDatum: WGS84; **Event:** eventID: 1; samplingProtocol: Aerial; eventTime: Night
- **Type status:** Other material. **Occurrence:** individualCount: 1; sex: male; **Location:** locationID: O1; continent: Europe; country: Spain; countryCode: ES; stateProvince: Aragón; county: Huesca; locality: O Furno; verbatimElevation: 1396.73; decimalLatitude: 42.60677; decimalLongitude: 0.13135; geodeticDatum: WGS84; **Event:** eventID: 1; samplingProtocol: Aerial; eventTime: Night
- **Type status:** Other material. **Occurrence:** individualCount: 2; sex: male; **Location:** locationID: O1; continent: Europe; country: Spain; countryCode: ES; stateProvince: Aragón; county: Huesca; locality: O Furno; verbatimElevation: 1396.73; decimalLatitude: 42.60677; decimalLongitude: 0.13135; geodeticDatum: WGS84; **Event:** eventID: 2; samplingProtocol: Aerial; eventTime: Night
- **Type status:** Other material. **Occurrence:** individualCount: 1; sex: female; **Location:** locationID: O1; continent: Europe; country: Spain; countryCode: ES; stateProvince: Aragón; county: Huesca; locality: O Furno; verbatimElevation: 1396.73; decimalLatitude: 42.60677; decimalLongitude: 0.13135; geodeticDatum: WGS84; **Event:** eventID: 2; samplingProtocol: Beating; eventTime: Day
- **Type status:** Other material. **Occurrence:** individualCount: 1; sex: male; **Location:** locationID: O1; continent: Europe; country: Spain; countryCode: ES; stateProvince: Aragón; county: Huesca; locality: O Furno; verbatimElevation: 1396.73; decimalLatitude: 42.60677; decimalLongitude: 0.13135; geodeticDatum: WGS84; **Event:** eventID: 2; samplingProtocol: Beating; eventTime: Day
- **Type status:** Other material. **Occurrence:** individualCount: 1; sex: female; **Location:** locationID: O1; continent: Europe; country: Spain; countryCode: ES; stateProvince: Aragón; county: Huesca; locality: O Furno; verbatimElevation: 1396.73; decimalLatitude: 42.60677; decimalLongitude: 0.13135; geodeticDatum: WGS84; **Event:** eventID: 1; samplingProtocol: Beating; eventTime: Night
- **Type status:** Other material. **Occurrence:** individualCount: 1; sex: female; **Location:** locationID: O2; continent: Europe; country: Spain; countryCode: ES; stateProvince: Aragón; county: Huesca; locality: Rebilla; verbatimElevation: 1158.13; decimalLatitude: 42.59427; decimalLongitude: 0.1529; geodeticDatum: WGS84; **Event:** eventID: 1; samplingProtocol: Aerial; eventTime: Night
- **Type status:** Other material. **Occurrence:** individualCount: 1; sex: male; **Location:** locationID: O2; continent: Europe; country: Spain; countryCode: ES; stateProvince: Aragón; county: Huesca; locality: Rebilla; verbatimElevation: 1158.13; decimalLatitude: 42.59427; decimalLongitude: 0.1529; geodeticDatum: WGS84; **Event:** eventID: 1; samplingProtocol: Aerial; eventTime: Night
- **Type status:** Other material. **Occurrence:** individualCount: 5; sex: female; **Location:** locationID: O2; continent: Europe; country: Spain; countryCode: ES; stateProvince: Aragón; county: Huesca; locality: Rebilla; verbatimElevation: 1158.13; decimalLatitude: 42.59427; decimalLongitude: 0.1529; geodeticDatum: WGS84; **Event:** eventID: 1; samplingProtocol: Aerial; eventTime: Night
- **Type status:** Other material. **Occurrence:** individualCount: 1; sex: male; **Location:** locationID: O2; continent: Europe; country: Spain; countryCode: ES; stateProvince: Aragón; county: Huesca; locality: Rebilla; verbatimElevation: 1158.13; decimalLatitude: 42.59427; decimalLongitude: 0.1529; geodeticDatum: WGS84; **Event:** eventID: 1; samplingProtocol: Aerial; eventTime: Night
- **Type status:** Other material. **Occurrence:** individualCount: 1; sex: female; **Location:** locationID: O2; continent: Europe; country: Spain; countryCode: ES; stateProvince: Aragón; county: Huesca; locality: Rebilla; verbatimElevation: 1158.13; decimalLatitude: 42.59427; decimalLongitude: 0.1529; geodeticDatum: WGS84; **Event:** eventID: 2; samplingProtocol: Aerial; eventTime: Night
- **Type status:** Other material. **Occurrence:** individualCount: 1; sex: female; **Location:** locationID: O2; continent: Europe; country: Spain; countryCode: ES; stateProvince: Aragón; county: Huesca; locality: Rebilla; verbatimElevation: 1158.13; decimalLatitude: 42.59427; decimalLongitude: 0.1529; geodeticDatum: WGS84; **Event:** eventID: 2; samplingProtocol: Aerial; eventTime: Night
- **Type status:** Other material. **Occurrence:** individualCount: 1; sex: female; **Location:** locationID: O2; continent: Europe; country: Spain; countryCode: ES; stateProvince: Aragón; county: Huesca; locality: Rebilla; verbatimElevation: 1158.13; decimalLatitude: 42.59427; decimalLongitude: 0.1529; geodeticDatum: WGS84; **Event:** eventID: 1; samplingProtocol: Beating; eventTime: Day
- **Type status:** Other material. **Occurrence:** individualCount: 2; sex: male; **Location:** locationID: O2; continent: Europe; country: Spain; countryCode: ES; stateProvince: Aragón; county: Huesca; locality: Rebilla; verbatimElevation: 1158.13; decimalLatitude: 42.59427; decimalLongitude: 0.1529; geodeticDatum: WGS84; **Event:** eventID: 1; samplingProtocol: Beating; eventTime: Day
- **Type status:** Other material. **Occurrence:** individualCount: 3; sex: female; **Location:** locationID: O2; continent: Europe; country: Spain; countryCode: ES; stateProvince: Aragón; county: Huesca; locality: Rebilla; verbatimElevation: 1158.13; decimalLatitude: 42.59427; decimalLongitude: 0.1529; geodeticDatum: WGS84; **Event:** eventID: 2; samplingProtocol: Beating; eventTime: Day
- **Type status:** Other material. **Occurrence:** individualCount: 1; sex: male; **Location:** locationID: O2; continent: Europe; country: Spain; countryCode: ES; stateProvince: Aragón; county: Huesca; locality: Rebilla; verbatimElevation: 1158.13; decimalLatitude: 42.59427; decimalLongitude: 0.1529; geodeticDatum: WGS84; **Event:** eventID: 2; samplingProtocol: Beating; eventTime: Day
- **Type status:** Other material. **Occurrence:** individualCount: 1; sex: female; **Location:** locationID: O2; continent: Europe; country: Spain; countryCode: ES; stateProvince: Aragón; county: Huesca; locality: Rebilla; verbatimElevation: 1158.13; decimalLatitude: 42.59427; decimalLongitude: 0.1529; geodeticDatum: WGS84; **Event:** eventID: 1; samplingProtocol: Beating; eventTime: Night
- **Type status:** Other material. **Occurrence:** individualCount: 1; sex: female; **Location:** locationID: O2; continent: Europe; country: Spain; countryCode: ES; stateProvince: Aragón; county: Huesca; locality: Rebilla; verbatimElevation: 1158.13; decimalLatitude: 42.59427; decimalLongitude: 0.1529; geodeticDatum: WGS84; **Event:** eventID: 1; samplingProtocol: Beating; eventTime: Night
- **Type status:** Other material. **Occurrence:** individualCount: 1; sex: female; **Location:** locationID: O2; continent: Europe; country: Spain; countryCode: ES; stateProvince: Aragón; county: Huesca; locality: Rebilla; verbatimElevation: 1158.13; decimalLatitude: 42.59427; decimalLongitude: 0.1529; geodeticDatum: WGS84; **Event:** eventID: 1; samplingProtocol: Sweeping; eventTime: Day
- **Type status:** Other material. **Occurrence:** individualCount: 1; sex: female; **Location:** locationID: O2; continent: Europe; country: Spain; countryCode: ES; stateProvince: Aragón; county: Huesca; locality: Rebilla; verbatimElevation: 1158.13; decimalLatitude: 42.59427; decimalLongitude: 0.1529; geodeticDatum: WGS84; **Event:** eventID: 1; samplingProtocol: Sweeping; eventTime: Night
- **Type status:** Other material. **Occurrence:** individualCount: 1; sex: male; **Location:** locationID: O2; continent: Europe; country: Spain; countryCode: ES; stateProvince: Aragón; county: Huesca; locality: Rebilla; verbatimElevation: 1158.13; decimalLatitude: 42.59427; decimalLongitude: 0.1529; geodeticDatum: WGS84; **Event:** eventID: 1; samplingProtocol: Sweeping; eventTime: Night
- **Type status:** Other material. **Occurrence:** individualCount: 2; sex: female; **Location:** locationID: P2; continent: Europe; country: Spain; countryCode: ES; stateProvince: Castilla y León; county: León; locality: Joyoguelas; verbatimElevation: 763.98; decimalLatitude: 43.17771; decimalLongitude: -4.90579; geodeticDatum: WGS84; **Event:** eventID: 1; samplingProtocol: Aerial; eventTime: Night
- **Type status:** Other material. **Occurrence:** individualCount: 2; sex: male; **Location:** locationID: P2; continent: Europe; country: Spain; countryCode: ES; stateProvince: Castilla y León; county: León; locality: Joyoguelas; verbatimElevation: 763.98; decimalLatitude: 43.17771; decimalLongitude: -4.90579; geodeticDatum: WGS84; **Event:** eventID: 1; samplingProtocol: Aerial; eventTime: Night
- **Type status:** Other material. **Occurrence:** individualCount: 1; sex: male; **Location:** locationID: P2; continent: Europe; country: Spain; countryCode: ES; stateProvince: Castilla y León; county: León; locality: Joyoguelas; verbatimElevation: 763.98; decimalLatitude: 43.17771; decimalLongitude: -4.90579; geodeticDatum: WGS84; **Event:** eventID: 2; samplingProtocol: Aerial; eventTime: Night
- **Type status:** Other material. **Occurrence:** individualCount: 1; sex: male; **Location:** locationID: P2; continent: Europe; country: Spain; countryCode: ES; stateProvince: Castilla y León; county: León; locality: Joyoguelas; verbatimElevation: 763.98; decimalLatitude: 43.17771; decimalLongitude: -4.90579; geodeticDatum: WGS84; **Event:** eventID: 2; samplingProtocol: Aerial; eventTime: Night
- **Type status:** Other material. **Occurrence:** individualCount: 1; sex: male; **Location:** locationID: P2; continent: Europe; country: Spain; countryCode: ES; stateProvince: Castilla y León; county: León; locality: Joyoguelas; verbatimElevation: 763.98; decimalLatitude: 43.17771; decimalLongitude: -4.90579; geodeticDatum: WGS84; **Event:** eventID: 1; samplingProtocol: Beating; eventTime: Day
- **Type status:** Other material. **Occurrence:** individualCount: 3; sex: male; **Location:** locationID: P2; continent: Europe; country: Spain; countryCode: ES; stateProvince: Castilla y León; county: León; locality: Joyoguelas; verbatimElevation: 763.98; decimalLatitude: 43.17771; decimalLongitude: -4.90579; geodeticDatum: WGS84; **Event:** eventID: 2; samplingProtocol: Beating; eventTime: Day
- **Type status:** Other material. **Occurrence:** individualCount: 1; sex: male; **Location:** locationID: P2; continent: Europe; country: Spain; countryCode: ES; stateProvince: Castilla y León; county: León; locality: Joyoguelas; verbatimElevation: 763.98; decimalLatitude: 43.17771; decimalLongitude: -4.90579; geodeticDatum: WGS84; **Event:** eventID: 1; samplingProtocol: Beating; eventTime: Night
- **Type status:** Other material. **Occurrence:** individualCount: 1; sex: male; **Location:** locationID: P2; continent: Europe; country: Spain; countryCode: ES; stateProvince: Castilla y León; county: León; locality: Joyoguelas; verbatimElevation: 763.98; decimalLatitude: 43.17771; decimalLongitude: -4.90579; geodeticDatum: WGS84; **Event:** eventID: 1; samplingProtocol: Sweeping; eventTime: Night
- **Type status:** Other material. **Occurrence:** individualCount: 3; sex: female; **Location:** locationID: P4; continent: Europe; country: Spain; countryCode: ES; stateProvince: Castilla y León; county: León; locality: El Canto; verbatimElevation: 943.48; decimalLatitude: 43.17227; decimalLongitude: -4.90857; geodeticDatum: WGS84; **Event:** eventID: 1; samplingProtocol: Aerial; eventTime: Night
- **Type status:** Other material. **Occurrence:** individualCount: 1; sex: male; **Location:** locationID: P4; continent: Europe; country: Spain; countryCode: ES; stateProvince: Castilla y León; county: León; locality: El Canto; verbatimElevation: 943.48; decimalLatitude: 43.17227; decimalLongitude: -4.90857; geodeticDatum: WGS84; **Event:** eventID: 2; samplingProtocol: Aerial; eventTime: Night
- **Type status:** Other material. **Occurrence:** individualCount: 3; sex: female; **Location:** locationID: P4; continent: Europe; country: Spain; countryCode: ES; stateProvince: Castilla y León; county: León; locality: El Canto; verbatimElevation: 943.48; decimalLatitude: 43.17227; decimalLongitude: -4.90857; geodeticDatum: WGS84; **Event:** eventID: 1; samplingProtocol: Beating; eventTime: Day
- **Type status:** Other material. **Occurrence:** individualCount: 1; sex: male; **Location:** locationID: P4; continent: Europe; country: Spain; countryCode: ES; stateProvince: Castilla y León; county: León; locality: El Canto; verbatimElevation: 943.48; decimalLatitude: 43.17227; decimalLongitude: -4.90857; geodeticDatum: WGS84; **Event:** eventID: 1; samplingProtocol: Beating; eventTime: Day
- **Type status:** Other material. **Occurrence:** individualCount: 1; sex: female; **Location:** locationID: P4; continent: Europe; country: Spain; countryCode: ES; stateProvince: Castilla y León; county: León; locality: El Canto; verbatimElevation: 943.48; decimalLatitude: 43.17227; decimalLongitude: -4.90857; geodeticDatum: WGS84; **Event:** eventID: 2; samplingProtocol: Beating; eventTime: Day
- **Type status:** Other material. **Occurrence:** individualCount: 2; sex: female; **Location:** locationID: P4; continent: Europe; country: Spain; countryCode: ES; stateProvince: Castilla y León; county: León; locality: El Canto; verbatimElevation: 943.48; decimalLatitude: 43.17227; decimalLongitude: -4.90857; geodeticDatum: WGS84; **Event:** eventID: 1; samplingProtocol: Beating; eventTime: Night
- **Type status:** Other material. **Occurrence:** individualCount: 1; sex: male; **Location:** locationID: P4; continent: Europe; country: Spain; countryCode: ES; stateProvince: Castilla y León; county: León; locality: El Canto; verbatimElevation: 943.48; decimalLatitude: 43.17227; decimalLongitude: -4.90857; geodeticDatum: WGS84; **Event:** eventID: 1; samplingProtocol: Beating; eventTime: Night
- **Type status:** Other material. **Occurrence:** individualCount: 1; sex: female; **Location:** locationID: P4; continent: Europe; country: Spain; countryCode: ES; stateProvince: Castilla y León; county: León; locality: El Canto; verbatimElevation: 943.48; decimalLatitude: 43.17227; decimalLongitude: -4.90857; geodeticDatum: WGS84; **Event:** eventID: 1; samplingProtocol: Beating; eventTime: Night
- **Type status:** Other material. **Occurrence:** individualCount: 2; sex: male; **Location:** locationID: P4; continent: Europe; country: Spain; countryCode: ES; stateProvince: Castilla y León; county: León; locality: El Canto; verbatimElevation: 943.48; decimalLatitude: 43.17227; decimalLongitude: -4.90857; geodeticDatum: WGS84; **Event:** eventID: 1; samplingProtocol: Beating; eventTime: Night
- **Type status:** Other material. **Occurrence:** individualCount: 1; sex: male; **Location:** locationID: P4; continent: Europe; country: Spain; countryCode: ES; stateProvince: Castilla y León; county: León; locality: El Canto; verbatimElevation: 943.48; decimalLatitude: 43.17227; decimalLongitude: -4.90857; geodeticDatum: WGS84; **Event:** eventID: 1; samplingProtocol: Sweeping; eventTime: Day

##### Distribution

Holarctic

#### Robertus arundineti

(O. Pickard-Cambridge, 1871)

##### Materials

- **Type status:** Other material. **Occurrence:** individualCount: 1; sex: male; **Location:** locationID: C2; continent: Europe; country: Spain; countryCode: ES; stateProvince: Castilla-La Mancha; county: Ciudad Real; locality: Valle Brezoso; verbatimElevation: 739.31; decimalLatitude: 39.35159; decimalLongitude: -4.3589; geodeticDatum: WGS84; **Event:** eventID: H; samplingProtocol: Pitfall
- **Type status:** Other material. **Occurrence:** individualCount: 1; sex: female; **Location:** locationID: C4; continent: Europe; country: Spain; countryCode: ES; stateProvince: Castilla-La Mancha; county: Ciudad Real; locality: La Quesera; verbatimElevation: 772.3; decimalLatitude: 39.36337; decimalLongitude: -4.41704; geodeticDatum: WGS84; **Event:** eventID: L; samplingProtocol: Pitfall
- **Type status:** Other material. **Occurrence:** individualCount: 1; sex: female; **Location:** locationID: P4; continent: Europe; country: Spain; countryCode: ES; stateProvince: Castilla y León; county: León; locality: El Canto; verbatimElevation: 943.48; decimalLatitude: 43.17227; decimalLongitude: -4.90857; geodeticDatum: WGS84; **Event:** eventID: J; samplingProtocol: Pitfall

##### Distribution

Palearctic

#### Sardinidion blackwalli

(O. Pickard-Cambridge, 1871)

##### Materials

- **Type status:** Other material. **Occurrence:** individualCount: 3; sex: female; **Location:** locationID: C3; continent: Europe; country: Spain; countryCode: ES; stateProvince: Castilla-La Mancha; county: Ciudad Real; locality: La Quesera; verbatimElevation: 767.55; decimalLatitude: 39.36177; decimalLongitude: -4.41733; geodeticDatum: WGS84; **Event:** eventID: 1; samplingProtocol: Beating; eventTime: Day
- **Type status:** Other material. **Occurrence:** individualCount: 1; sex: male; **Location:** locationID: C3; continent: Europe; country: Spain; countryCode: ES; stateProvince: Castilla-La Mancha; county: Ciudad Real; locality: La Quesera; verbatimElevation: 767.55; decimalLatitude: 39.36177; decimalLongitude: -4.41733; geodeticDatum: WGS84; **Event:** eventID: 1; samplingProtocol: Beating; eventTime: Day
- **Type status:** Other material. **Occurrence:** individualCount: 1; sex: male; **Location:** locationID: C3; continent: Europe; country: Spain; countryCode: ES; stateProvince: Castilla-La Mancha; county: Ciudad Real; locality: La Quesera; verbatimElevation: 767.55; decimalLatitude: 39.36177; decimalLongitude: -4.41733; geodeticDatum: WGS84; **Event:** eventID: 2; samplingProtocol: Sweeping; eventTime: Night
- **Type status:** Other material. **Occurrence:** individualCount: 1; sex: male; **Location:** locationID: S2; continent: Europe; country: Spain; countryCode: ES; stateProvince: Andalucía; county: Granada; locality: Camarate; verbatimElevation: 1713.96; decimalLatitude: 37.18377; decimalLongitude: -3.26282; geodeticDatum: WGS84; **Event:** eventID: 1; samplingProtocol: Sweeping; eventTime: Day

##### Distribution

Europe, Russia, Ukraine, North Africa

##### Notes

**Figure 15.**
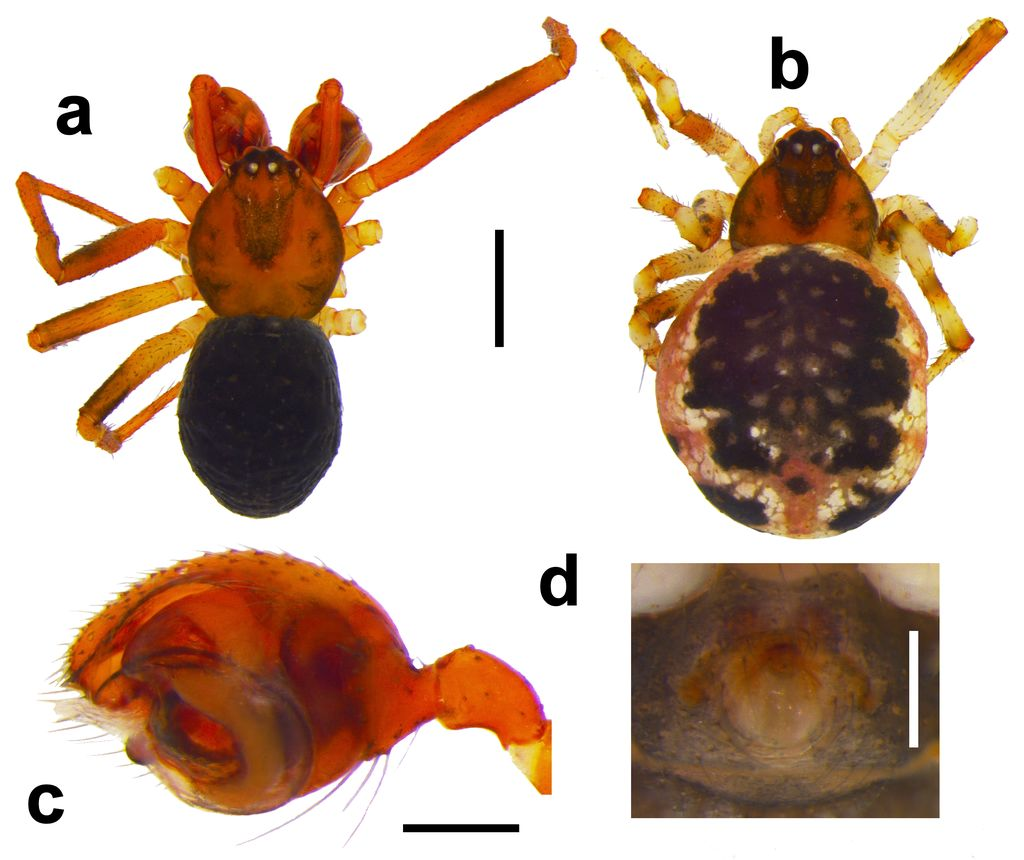
*Sardinidion blackwalli* (O. Pickard-Cambridge, 1871). a: male, dorsal habitus, scale = 0.7 mm; b: female, dorsal habitus, scale = 0.7 mm; c: left male palp, retrolateral, scale = 0.2 mm; d: female epigynum, ventral, scale = 0.2 mm.

First record for Spain. See Fig. 15.

#### Simitidion simile

(C. L. Koch, 1836)

##### Materials

- **Type status:** Other material. **Occurrence:** individualCount: 1; sex: female; **Location:** locationID: C1; continent: Europe; country: Spain; countryCode: ES; stateProvince: Castilla-La Mancha; county: Ciudad Real; locality: Valle Brezoso; verbatimElevation: 756.56; decimalLatitude: 39.35663; decimalLongitude: -4.35912; geodeticDatum: WGS84; **Event:** eventID: 1; samplingProtocol: Sweeping; eventTime: Day
- **Type status:** Other material. **Occurrence:** individualCount: 1; sex: male; **Location:** locationID: C1; continent: Europe; country: Spain; countryCode: ES; stateProvince: Castilla-La Mancha; county: Ciudad Real; locality: Valle Brezoso; verbatimElevation: 756.56; decimalLatitude: 39.35663; decimalLongitude: -4.35912; geodeticDatum: WGS84; **Event:** eventID: 2; samplingProtocol: Sweeping; eventTime: Day
- **Type status:** Other material. **Occurrence:** individualCount: 1; sex: female; **Location:** locationID: C2; continent: Europe; country: Spain; countryCode: ES; stateProvince: Castilla-La Mancha; county: Ciudad Real; locality: Valle Brezoso; verbatimElevation: 739.31; decimalLatitude: 39.35159; decimalLongitude: -4.3589; geodeticDatum: WGS84; **Event:** eventID: 1; samplingProtocol: Sweeping; eventTime: Day
- **Type status:** Other material. **Occurrence:** individualCount: 3; sex: female; **Location:** locationID: C3; continent: Europe; country: Spain; countryCode: ES; stateProvince: Castilla-La Mancha; county: Ciudad Real; locality: La Quesera; verbatimElevation: 767.55; decimalLatitude: 39.36177; decimalLongitude: -4.41733; geodeticDatum: WGS84; **Event:** eventID: 2; samplingProtocol: Beating; eventTime: Day
- **Type status:** Other material. **Occurrence:** individualCount: 2; sex: female; **Location:** locationID: C4; continent: Europe; country: Spain; countryCode: ES; stateProvince: Castilla-La Mancha; county: Ciudad Real; locality: La Quesera; verbatimElevation: 772.3; decimalLatitude: 39.36337; decimalLongitude: -4.41704; geodeticDatum: WGS84; **Event:** eventID: 2; samplingProtocol: Aerial; eventTime: Night
- **Type status:** Other material. **Occurrence:** individualCount: 1; sex: female; **Location:** locationID: C4; continent: Europe; country: Spain; countryCode: ES; stateProvince: Castilla-La Mancha; county: Ciudad Real; locality: La Quesera; verbatimElevation: 772.3; decimalLatitude: 39.36337; decimalLongitude: -4.41704; geodeticDatum: WGS84; **Event:** eventID: 1; samplingProtocol: Beating; eventTime: Day
- **Type status:** Other material. **Occurrence:** individualCount: 1; sex: male; **Location:** locationID: C4; continent: Europe; country: Spain; countryCode: ES; stateProvince: Castilla-La Mancha; county: Ciudad Real; locality: La Quesera; verbatimElevation: 772.3; decimalLatitude: 39.36337; decimalLongitude: -4.41704; geodeticDatum: WGS84; **Event:** eventID: 1; samplingProtocol: Beating; eventTime: Night
- **Type status:** Other material. **Occurrence:** individualCount: 1; sex: male; **Location:** locationID: C4; continent: Europe; country: Spain; countryCode: ES; stateProvince: Castilla-La Mancha; county: Ciudad Real; locality: La Quesera; verbatimElevation: 772.3; decimalLatitude: 39.36337; decimalLongitude: -4.41704; geodeticDatum: WGS84; **Event:** eventID: 2; samplingProtocol: Beating; eventTime: Night
- **Type status:** Other material. **Occurrence:** individualCount: 3; sex: female; **Location:** locationID: C4; continent: Europe; country: Spain; countryCode: ES; stateProvince: Castilla-La Mancha; county: Ciudad Real; locality: La Quesera; verbatimElevation: 772.3; decimalLatitude: 39.36337; decimalLongitude: -4.41704; geodeticDatum: WGS84; **Event:** eventID: 1; samplingProtocol: Sweeping; eventTime: Day
- **Type status:** Other material. **Occurrence:** individualCount: 1; sex: female; **Location:** locationID: C4; continent: Europe; country: Spain; countryCode: ES; stateProvince: Castilla-La Mancha; county: Ciudad Real; locality: La Quesera; verbatimElevation: 772.3; decimalLatitude: 39.36337; decimalLongitude: -4.41704; geodeticDatum: WGS84; **Event:** eventID: 2; samplingProtocol: Sweeping; eventTime: Day
- **Type status:** Other material. **Occurrence:** individualCount: 1; sex: male; **Location:** locationID: O2; continent: Europe; country: Spain; countryCode: ES; stateProvince: Aragón; county: Huesca; locality: Rebilla; verbatimElevation: 1158.13; decimalLatitude: 42.59427; decimalLongitude: 0.1529; geodeticDatum: WGS84; **Event:** eventID: 1; samplingProtocol: Sweeping; eventTime: Night
- **Type status:** Other material. **Occurrence:** individualCount: 1; sex: male; **Location:** locationID: O2; continent: Europe; country: Spain; countryCode: ES; stateProvince: Aragón; county: Huesca; locality: Rebilla; verbatimElevation: 1158.13; decimalLatitude: 42.59427; decimalLongitude: 0.1529; geodeticDatum: WGS84; **Event:** eventID: 1; samplingProtocol: Sweeping; eventTime: Night
- **Type status:** Other material. **Occurrence:** individualCount: 1; sex: female; **Location:** locationID: P2; continent: Europe; country: Spain; countryCode: ES; stateProvince: Castilla y León; county: León; locality: Joyoguelas; verbatimElevation: 763.98; decimalLatitude: 43.17771; decimalLongitude: -4.90579; geodeticDatum: WGS84; **Event:** eventID: 1; samplingProtocol: Sweeping; eventTime: Night
- **Type status:** Other material. **Occurrence:** individualCount: 1; sex: female; **Location:** locationID: S1; continent: Europe; country: Spain; countryCode: ES; stateProvince: Andalucía; county: Granada; locality: Soportujar; verbatimElevation: 1786.57; decimalLatitude: 36.96151; decimalLongitude: -3.41881; geodeticDatum: WGS84; **Event:** eventID: 1; samplingProtocol: Sweeping; eventTime: Night

##### Distribution

Holarctic

#### Steatoda triangulosa

(Walckenaer, 1802)

##### Materials

- **Type status:** Other material. **Occurrence:** individualCount: 1; sex: male; **Location:** locationID: O2; continent: Europe; country: Spain; countryCode: ES; stateProvince: Aragón; county: Huesca; locality: Rebilla; verbatimElevation: 1158.13; decimalLatitude: 42.59427; decimalLongitude: 0.1529; geodeticDatum: WGS84; **Event:** eventID: 2; samplingProtocol: Aerial; eventTime: Night

##### Distribution

Cosmopolitan

#### Theridion harmsi

Wunderlich, 2011

##### Materials

- **Type status:** Other material. **Occurrence:** individualCount: 3; sex: female; **Location:** locationID: A1; continent: Europe; country: Spain; countryCode: ES; stateProvince: Catalonia; county: Lleida; locality: Sola de Boi; verbatimElevation: 1759.8; decimalLatitude: 42.54958; decimalLongitude: 0.87254; geodeticDatum: WGS84; **Event:** eventID: 1; samplingProtocol: Aerial; eventTime: Night
- **Type status:** Other material. **Occurrence:** individualCount: 2; sex: male; **Location:** locationID: A1; continent: Europe; country: Spain; countryCode: ES; stateProvince: Catalonia; county: Lleida; locality: Sola de Boi; verbatimElevation: 1759.8; decimalLatitude: 42.54958; decimalLongitude: 0.87254; geodeticDatum: WGS84; **Event:** eventID: 1; samplingProtocol: Aerial; eventTime: Night
- **Type status:** Other material. **Occurrence:** individualCount: 1; sex: female; **Location:** locationID: A1; continent: Europe; country: Spain; countryCode: ES; stateProvince: Catalonia; county: Lleida; locality: Sola de Boi; verbatimElevation: 1759.8; decimalLatitude: 42.54958; decimalLongitude: 0.87254; geodeticDatum: WGS84; **Event:** eventID: 1; samplingProtocol: Aerial; eventTime: Night
- **Type status:** Other material. **Occurrence:** individualCount: 2; sex: male; **Location:** locationID: A1; continent: Europe; country: Spain; countryCode: ES; stateProvince: Catalonia; county: Lleida; locality: Sola de Boi; verbatimElevation: 1759.8; decimalLatitude: 42.54958; decimalLongitude: 0.87254; geodeticDatum: WGS84; **Event:** eventID: 1; samplingProtocol: Aerial; eventTime: Night
- **Type status:** Other material. **Occurrence:** individualCount: 1; sex: male; **Location:** locationID: A1; continent: Europe; country: Spain; countryCode: ES; stateProvince: Catalonia; county: Lleida; locality: Sola de Boi; verbatimElevation: 1759.8; decimalLatitude: 42.54958; decimalLongitude: 0.87254; geodeticDatum: WGS84; **Event:** eventID: 2; samplingProtocol: Aerial; eventTime: Night
- **Type status:** Other material. **Occurrence:** individualCount: 1; sex: female; **Location:** locationID: A1; continent: Europe; country: Spain; countryCode: ES; stateProvince: Catalonia; county: Lleida; locality: Sola de Boi; verbatimElevation: 1759.8; decimalLatitude: 42.54958; decimalLongitude: 0.87254; geodeticDatum: WGS84; **Event:** eventID: 2; samplingProtocol: Aerial; eventTime: Night
- **Type status:** Other material. **Occurrence:** individualCount: 1; sex: male; **Location:** locationID: A1; continent: Europe; country: Spain; countryCode: ES; stateProvince: Catalonia; county: Lleida; locality: Sola de Boi; verbatimElevation: 1759.8; decimalLatitude: 42.54958; decimalLongitude: 0.87254; geodeticDatum: WGS84; **Event:** eventID: 2; samplingProtocol: Aerial; eventTime: Night
- **Type status:** Other material. **Occurrence:** individualCount: 2; sex: female; **Location:** locationID: A1; continent: Europe; country: Spain; countryCode: ES; stateProvince: Catalonia; county: Lleida; locality: Sola de Boi; verbatimElevation: 1759.8; decimalLatitude: 42.54958; decimalLongitude: 0.87254; geodeticDatum: WGS84; **Event:** eventID: 1; samplingProtocol: Beating; eventTime: Day
- **Type status:** Other material. **Occurrence:** individualCount: 1; sex: female; **Location:** locationID: A1; continent: Europe; country: Spain; countryCode: ES; stateProvince: Catalonia; county: Lleida; locality: Sola de Boi; verbatimElevation: 1759.8; decimalLatitude: 42.54958; decimalLongitude: 0.87254; geodeticDatum: WGS84; **Event:** eventID: 2; samplingProtocol: Beating; eventTime: Day
- **Type status:** Other material. **Occurrence:** individualCount: 2; sex: male; **Location:** locationID: A1; continent: Europe; country: Spain; countryCode: ES; stateProvince: Catalonia; county: Lleida; locality: Sola de Boi; verbatimElevation: 1759.8; decimalLatitude: 42.54958; decimalLongitude: 0.87254; geodeticDatum: WGS84; **Event:** eventID: 2; samplingProtocol: Beating; eventTime: Day
- **Type status:** Other material. **Occurrence:** individualCount: 2; sex: female; **Location:** locationID: A1; continent: Europe; country: Spain; countryCode: ES; stateProvince: Catalonia; county: Lleida; locality: Sola de Boi; verbatimElevation: 1759.8; decimalLatitude: 42.54958; decimalLongitude: 0.87254; geodeticDatum: WGS84; **Event:** eventID: 1; samplingProtocol: Beating; eventTime: Night
- **Type status:** Other material. **Occurrence:** individualCount: 1; sex: male; **Location:** locationID: A1; continent: Europe; country: Spain; countryCode: ES; stateProvince: Catalonia; county: Lleida; locality: Sola de Boi; verbatimElevation: 1759.8; decimalLatitude: 42.54958; decimalLongitude: 0.87254; geodeticDatum: WGS84; **Event:** eventID: 1; samplingProtocol: Beating; eventTime: Night
- **Type status:** Other material. **Occurrence:** individualCount: 3; sex: female; **Location:** locationID: A1; continent: Europe; country: Spain; countryCode: ES; stateProvince: Catalonia; county: Lleida; locality: Sola de Boi; verbatimElevation: 1759.8; decimalLatitude: 42.54958; decimalLongitude: 0.87254; geodeticDatum: WGS84; **Event:** eventID: 1; samplingProtocol: Beating; eventTime: Night
- **Type status:** Other material. **Occurrence:** individualCount: 2; sex: female; **Location:** locationID: A1; continent: Europe; country: Spain; countryCode: ES; stateProvince: Catalonia; county: Lleida; locality: Sola de Boi; verbatimElevation: 1759.8; decimalLatitude: 42.54958; decimalLongitude: 0.87254; geodeticDatum: WGS84; **Event:** eventID: 2; samplingProtocol: Ground; eventTime: Night
- **Type status:** Other material. **Occurrence:** individualCount: 2; sex: female; **Location:** locationID: A1; continent: Europe; country: Spain; countryCode: ES; stateProvince: Catalonia; county: Lleida; locality: Sola de Boi; verbatimElevation: 1759.8; decimalLatitude: 42.54958; decimalLongitude: 0.87254; geodeticDatum: WGS84; **Event:** eventID: 1; samplingProtocol: Sweeping; eventTime: Night
- **Type status:** Other material. **Occurrence:** individualCount: 1; sex: male; **Location:** locationID: A1; continent: Europe; country: Spain; countryCode: ES; stateProvince: Catalonia; county: Lleida; locality: Sola de Boi; verbatimElevation: 1759.8; decimalLatitude: 42.54958; decimalLongitude: 0.87254; geodeticDatum: WGS84; **Event:** eventID: 1; samplingProtocol: Sweeping; eventTime: Night
- **Type status:** Other material. **Occurrence:** individualCount: 1; sex: male; **Location:** locationID: A1; continent: Europe; country: Spain; countryCode: ES; stateProvince: Catalonia; county: Lleida; locality: Sola de Boi; verbatimElevation: 1759.8; decimalLatitude: 42.54958; decimalLongitude: 0.87254; geodeticDatum: WGS84; **Event:** eventID: 1; samplingProtocol: Sweeping; eventTime: Night
- **Type status:** Other material. **Occurrence:** individualCount: 1; sex: female; **Location:** locationID: A2; continent: Europe; country: Spain; countryCode: ES; stateProvince: Catalonia; county: Lleida; locality: Sola de Boi; verbatimElevation: 1738.7; decimalLatitude: 42.54913; decimalLongitude: 0.87137; geodeticDatum: WGS84; **Event:** eventID: 1; samplingProtocol: Aerial; eventTime: Night
- **Type status:** Other material. **Occurrence:** individualCount: 3; sex: male; **Location:** locationID: A2; continent: Europe; country: Spain; countryCode: ES; stateProvince: Catalonia; county: Lleida; locality: Sola de Boi; verbatimElevation: 1738.7; decimalLatitude: 42.54913; decimalLongitude: 0.87137; geodeticDatum: WGS84; **Event:** eventID: 2; samplingProtocol: Aerial; eventTime: Night
- **Type status:** Other material. **Occurrence:** individualCount: 1; sex: female; **Location:** locationID: A2; continent: Europe; country: Spain; countryCode: ES; stateProvince: Catalonia; county: Lleida; locality: Sola de Boi; verbatimElevation: 1738.7; decimalLatitude: 42.54913; decimalLongitude: 0.87137; geodeticDatum: WGS84; **Event:** eventID: 1; samplingProtocol: Beating; eventTime: Day
- **Type status:** Other material. **Occurrence:** individualCount: 3; sex: female; **Location:** locationID: A2; continent: Europe; country: Spain; countryCode: ES; stateProvince: Catalonia; county: Lleida; locality: Sola de Boi; verbatimElevation: 1738.7; decimalLatitude: 42.54913; decimalLongitude: 0.87137; geodeticDatum: WGS84; **Event:** eventID: 2; samplingProtocol: Beating; eventTime: Day
- **Type status:** Other material. **Occurrence:** individualCount: 1; sex: female; **Location:** locationID: A2; continent: Europe; country: Spain; countryCode: ES; stateProvince: Catalonia; county: Lleida; locality: Sola de Boi; verbatimElevation: 1738.7; decimalLatitude: 42.54913; decimalLongitude: 0.87137; geodeticDatum: WGS84; **Event:** eventID: 1; samplingProtocol: Beating; eventTime: Night
- **Type status:** Other material. **Occurrence:** individualCount: 1; sex: male; **Location:** locationID: A2; continent: Europe; country: Spain; countryCode: ES; stateProvince: Catalonia; county: Lleida; locality: Sola de Boi; verbatimElevation: 1738.7; decimalLatitude: 42.54913; decimalLongitude: 0.87137; geodeticDatum: WGS84; **Event:** eventID: 1; samplingProtocol: Beating; eventTime: Night
- **Type status:** Other material. **Occurrence:** individualCount: 1; sex: female; **Location:** locationID: A2; continent: Europe; country: Spain; countryCode: ES; stateProvince: Catalonia; county: Lleida; locality: Sola de Boi; verbatimElevation: 1738.7; decimalLatitude: 42.54913; decimalLongitude: 0.87137; geodeticDatum: WGS84; **Event:** eventID: 1; samplingProtocol: Beating; eventTime: Night
- **Type status:** Other material. **Occurrence:** individualCount: 1; sex: female; **Location:** locationID: A2; continent: Europe; country: Spain; countryCode: ES; stateProvince: Catalonia; county: Lleida; locality: Sola de Boi; verbatimElevation: 1738.7; decimalLatitude: 42.54913; decimalLongitude: 0.87137; geodeticDatum: WGS84; **Event:** eventID: 1; samplingProtocol: Sweeping; eventTime: Night
- **Type status:** Other material. **Occurrence:** individualCount: 1; sex: female; **Location:** locationID: C1; continent: Europe; country: Spain; countryCode: ES; stateProvince: Castilla-La Mancha; county: Ciudad Real; locality: Valle Brezoso; verbatimElevation: 756.56; decimalLatitude: 39.35663; decimalLongitude: -4.35912; geodeticDatum: WGS84; **Event:** eventID: 3; samplingProtocol: Aerial; eventTime: Night
- **Type status:** Other material. **Occurrence:** individualCount: 1; sex: male; **Location:** locationID: C1; continent: Europe; country: Spain; countryCode: ES; stateProvince: Castilla-La Mancha; county: Ciudad Real; locality: Valle Brezoso; verbatimElevation: 756.56; decimalLatitude: 39.35663; decimalLongitude: -4.35912; geodeticDatum: WGS84; **Event:** eventID: 4; samplingProtocol: Aerial; eventTime: Night
- **Type status:** Other material. **Occurrence:** individualCount: 1; sex: female; **Location:** locationID: C1; continent: Europe; country: Spain; countryCode: ES; stateProvince: Castilla-La Mancha; county: Ciudad Real; locality: Valle Brezoso; verbatimElevation: 756.56; decimalLatitude: 39.35663; decimalLongitude: -4.35912; geodeticDatum: WGS84; **Event:** eventID: 1; samplingProtocol: Beating; eventTime: Day
- **Type status:** Other material. **Occurrence:** individualCount: 2; sex: male; **Location:** locationID: C1; continent: Europe; country: Spain; countryCode: ES; stateProvince: Castilla-La Mancha; county: Ciudad Real; locality: Valle Brezoso; verbatimElevation: 756.56; decimalLatitude: 39.35663; decimalLongitude: -4.35912; geodeticDatum: WGS84; **Event:** eventID: 2; samplingProtocol: Beating; eventTime: Night
- **Type status:** Other material. **Occurrence:** individualCount: 1; sex: female; **Location:** locationID: C1; continent: Europe; country: Spain; countryCode: ES; stateProvince: Castilla-La Mancha; county: Ciudad Real; locality: Valle Brezoso; verbatimElevation: 756.56; decimalLatitude: 39.35663; decimalLongitude: -4.35912; geodeticDatum: WGS84; **Event:** eventID: 2; samplingProtocol: Sweeping; eventTime: Day
- **Type status:** Other material. **Occurrence:** individualCount: 2; sex: female; **Location:** locationID: C2; continent: Europe; country: Spain; countryCode: ES; stateProvince: Castilla-La Mancha; county: Ciudad Real; locality: Valle Brezoso; verbatimElevation: 739.31; decimalLatitude: 39.35159; decimalLongitude: -4.3589; geodeticDatum: WGS84; **Event:** eventID: 1; samplingProtocol: Beating; eventTime: Day
- **Type status:** Other material. **Occurrence:** individualCount: 1; sex: female; **Location:** locationID: C2; continent: Europe; country: Spain; countryCode: ES; stateProvince: Castilla-La Mancha; county: Ciudad Real; locality: Valle Brezoso; verbatimElevation: 739.31; decimalLatitude: 39.35159; decimalLongitude: -4.3589; geodeticDatum: WGS84; **Event:** eventID: 2; samplingProtocol: Beating; eventTime: Day
- **Type status:** Other material. **Occurrence:** individualCount: 1; sex: female; **Location:** locationID: C2; continent: Europe; country: Spain; countryCode: ES; stateProvince: Castilla-La Mancha; county: Ciudad Real; locality: Valle Brezoso; verbatimElevation: 739.31; decimalLatitude: 39.35159; decimalLongitude: -4.3589; geodeticDatum: WGS84; **Event:** eventID: 1; samplingProtocol: Beating; eventTime: Night
- **Type status:** Other material. **Occurrence:** individualCount: 2; sex: female; **Location:** locationID: C2; continent: Europe; country: Spain; countryCode: ES; stateProvince: Castilla-La Mancha; county: Ciudad Real; locality: Valle Brezoso; verbatimElevation: 739.31; decimalLatitude: 39.35159; decimalLongitude: -4.3589; geodeticDatum: WGS84; **Event:** eventID: 2; samplingProtocol: Beating; eventTime: Night
- **Type status:** Other material. **Occurrence:** individualCount: 1; sex: female; **Location:** locationID: C2; continent: Europe; country: Spain; countryCode: ES; stateProvince: Castilla-La Mancha; county: Ciudad Real; locality: Valle Brezoso; verbatimElevation: 739.31; decimalLatitude: 39.35159; decimalLongitude: -4.3589; geodeticDatum: WGS84; **Event:** eventID: 1; samplingProtocol: Sweeping; eventTime: Night
- **Type status:** Other material. **Occurrence:** individualCount: 1; sex: male; **Location:** locationID: C2; continent: Europe; country: Spain; countryCode: ES; stateProvince: Castilla-La Mancha; county: Ciudad Real; locality: Valle Brezoso; verbatimElevation: 739.31; decimalLatitude: 39.35159; decimalLongitude: -4.3589; geodeticDatum: WGS84; **Event:** eventID: 1; samplingProtocol: Sweeping; eventTime: Night
- **Type status:** Other material. **Occurrence:** individualCount: 1; sex: female; **Location:** locationID: C3; continent: Europe; country: Spain; countryCode: ES; stateProvince: Castilla-La Mancha; county: Ciudad Real; locality: La Quesera; verbatimElevation: 767.55; decimalLatitude: 39.36177; decimalLongitude: -4.41733; geodeticDatum: WGS84; **Event:** eventID: 1; samplingProtocol: Aerial; eventTime: Night
- **Type status:** Other material. **Occurrence:** individualCount: 1; sex: female; **Location:** locationID: C3; continent: Europe; country: Spain; countryCode: ES; stateProvince: Castilla-La Mancha; county: Ciudad Real; locality: La Quesera; verbatimElevation: 767.55; decimalLatitude: 39.36177; decimalLongitude: -4.41733; geodeticDatum: WGS84; **Event:** eventID: 4; samplingProtocol: Aerial; eventTime: Night
- **Type status:** Other material. **Occurrence:** individualCount: 3; sex: female; **Location:** locationID: C3; continent: Europe; country: Spain; countryCode: ES; stateProvince: Castilla-La Mancha; county: Ciudad Real; locality: La Quesera; verbatimElevation: 767.55; decimalLatitude: 39.36177; decimalLongitude: -4.41733; geodeticDatum: WGS84; **Event:** eventID: 1; samplingProtocol: Beating; eventTime: Day
- **Type status:** Other material. **Occurrence:** individualCount: 1; sex: female; **Location:** locationID: C3; continent: Europe; country: Spain; countryCode: ES; stateProvince: Castilla-La Mancha; county: Ciudad Real; locality: La Quesera; verbatimElevation: 767.55; decimalLatitude: 39.36177; decimalLongitude: -4.41733; geodeticDatum: WGS84; **Event:** eventID: 2; samplingProtocol: Beating; eventTime: Day
- **Type status:** Other material. **Occurrence:** individualCount: 2; sex: female; **Location:** locationID: C3; continent: Europe; country: Spain; countryCode: ES; stateProvince: Castilla-La Mancha; county: Ciudad Real; locality: La Quesera; verbatimElevation: 767.55; decimalLatitude: 39.36177; decimalLongitude: -4.41733; geodeticDatum: WGS84; **Event:** eventID: 2; samplingProtocol: Beating; eventTime: Night
- **Type status:** Other material. **Occurrence:** individualCount: 1; sex: female; **Location:** locationID: C4; continent: Europe; country: Spain; countryCode: ES; stateProvince: Castilla-La Mancha; county: Ciudad Real; locality: La Quesera; verbatimElevation: 772.3; decimalLatitude: 39.36337; decimalLongitude: -4.41704; geodeticDatum: WGS84; **Event:** eventID: 2; samplingProtocol: Aerial; eventTime: Night
- **Type status:** Other material. **Occurrence:** individualCount: 1; sex: female; **Location:** locationID: C4; continent: Europe; country: Spain; countryCode: ES; stateProvince: Castilla-La Mancha; county: Ciudad Real; locality: La Quesera; verbatimElevation: 772.3; decimalLatitude: 39.36337; decimalLongitude: -4.41704; geodeticDatum: WGS84; **Event:** eventID: 4; samplingProtocol: Aerial; eventTime: Night
- **Type status:** Other material. **Occurrence:** individualCount: 2; sex: female; **Location:** locationID: C4; continent: Europe; country: Spain; countryCode: ES; stateProvince: Castilla-La Mancha; county: Ciudad Real; locality: La Quesera; verbatimElevation: 772.3; decimalLatitude: 39.36337; decimalLongitude: -4.41704; geodeticDatum: WGS84; **Event:** eventID: 1; samplingProtocol: Beating; eventTime: Day
- **Type status:** Other material. **Occurrence:** individualCount: 1; sex: female; **Location:** locationID: C4; continent: Europe; country: Spain; countryCode: ES; stateProvince: Castilla-La Mancha; county: Ciudad Real; locality: La Quesera; verbatimElevation: 772.3; decimalLatitude: 39.36337; decimalLongitude: -4.41704; geodeticDatum: WGS84; **Event:** eventID: 2; samplingProtocol: Sweeping; eventTime: Day
- **Type status:** Other material. **Occurrence:** individualCount: 1; sex: female; **Location:** locationID: M1; continent: Europe; country: Spain; countryCode: ES; stateProvince: Extremadura; county: Cáceres; locality: Peña Falcón; verbatimElevation: 320.6; decimalLatitude: 39.83296; decimalLongitude: -6.0641; geodeticDatum: WGS84; **Event:** eventID: 1; samplingProtocol: Aerial; eventTime: Night
- **Type status:** Other material. **Occurrence:** individualCount: 1; sex: female; **Location:** locationID: M1; continent: Europe; country: Spain; countryCode: ES; stateProvince: Extremadura; county: Cáceres; locality: Peña Falcón; verbatimElevation: 320.6; decimalLatitude: 39.83296; decimalLongitude: -6.0641; geodeticDatum: WGS84; **Event:** eventID: 3; samplingProtocol: Aerial; eventTime: Night
- **Type status:** Other material. **Occurrence:** individualCount: 5; sex: female; **Location:** locationID: M1; continent: Europe; country: Spain; countryCode: ES; stateProvince: Extremadura; county: Cáceres; locality: Peña Falcón; verbatimElevation: 320.6; decimalLatitude: 39.83296; decimalLongitude: -6.0641; geodeticDatum: WGS84; **Event:** eventID: 1; samplingProtocol: Beating; eventTime: Day
- **Type status:** Other material. **Occurrence:** individualCount: 2; sex: female; **Location:** locationID: M1; continent: Europe; country: Spain; countryCode: ES; stateProvince: Extremadura; county: Cáceres; locality: Peña Falcón; verbatimElevation: 320.6; decimalLatitude: 39.83296; decimalLongitude: -6.0641; geodeticDatum: WGS84; **Event:** eventID: 1; samplingProtocol: Beating; eventTime: Night
- **Type status:** Other material. **Occurrence:** individualCount: 1; sex: female; **Location:** locationID: M1; continent: Europe; country: Spain; countryCode: ES; stateProvince: Extremadura; county: Cáceres; locality: Peña Falcón; verbatimElevation: 320.6; decimalLatitude: 39.83296; decimalLongitude: -6.0641; geodeticDatum: WGS84; **Event:** eventID: 2; samplingProtocol: Beating; eventTime: Night
- **Type status:** Other material. **Occurrence:** individualCount: 4; sex: female; **Location:** locationID: M1; continent: Europe; country: Spain; countryCode: ES; stateProvince: Extremadura; county: Cáceres; locality: Peña Falcón; verbatimElevation: 320.6; decimalLatitude: 39.83296; decimalLongitude: -6.0641; geodeticDatum: WGS84; **Event:** eventID: 2; samplingProtocol: Beating; eventTime: Day
- **Type status:** Other material. **Occurrence:** individualCount: 1; sex: female; **Location:** locationID: M1; continent: Europe; country: Spain; countryCode: ES; stateProvince: Extremadura; county: Cáceres; locality: Peña Falcón; verbatimElevation: 320.6; decimalLatitude: 39.83296; decimalLongitude: -6.0641; geodeticDatum: WGS84; **Event:** eventID: 2; samplingProtocol: Sweeping; eventTime: Day
- **Type status:** Other material. **Occurrence:** individualCount: 4; sex: female; **Location:** locationID: M2; continent: Europe; country: Spain; countryCode: ES; stateProvince: Extremadura; county: Cáceres; locality: Fuente del Frances; verbatimElevation: 320.72; decimalLatitude: 39.828; decimalLongitude: -6.03249; geodeticDatum: WGS84; **Event:** eventID: 1; samplingProtocol: Aerial; eventTime: Night
- **Type status:** Other material. **Occurrence:** individualCount: 1; sex: female; **Location:** locationID: M2; continent: Europe; country: Spain; countryCode: ES; stateProvince: Extremadura; county: Cáceres; locality: Fuente del Frances; verbatimElevation: 320.72; decimalLatitude: 39.828; decimalLongitude: -6.03249; geodeticDatum: WGS84; **Event:** eventID: 3; samplingProtocol: Aerial; eventTime: Night
- **Type status:** Other material. **Occurrence:** individualCount: 1; sex: female; **Location:** locationID: M2; continent: Europe; country: Spain; countryCode: ES; stateProvince: Extremadura; county: Cáceres; locality: Fuente del Frances; verbatimElevation: 320.72; decimalLatitude: 39.828; decimalLongitude: -6.03249; geodeticDatum: WGS84; **Event:** eventID: 4; samplingProtocol: Aerial; eventTime: Night
- **Type status:** Other material. **Occurrence:** individualCount: 2; sex: female; **Location:** locationID: M2; continent: Europe; country: Spain; countryCode: ES; stateProvince: Extremadura; county: Cáceres; locality: Fuente del Frances; verbatimElevation: 320.72; decimalLatitude: 39.828; decimalLongitude: -6.03249; geodeticDatum: WGS84; **Event:** eventID: 1; samplingProtocol: Beating; eventTime: Day
- **Type status:** Other material. **Occurrence:** individualCount: 2; sex: female; **Location:** locationID: M2; continent: Europe; country: Spain; countryCode: ES; stateProvince: Extremadura; county: Cáceres; locality: Fuente del Frances; verbatimElevation: 320.72; decimalLatitude: 39.828; decimalLongitude: -6.03249; geodeticDatum: WGS84; **Event:** eventID: 1; samplingProtocol: Beating; eventTime: Night
- **Type status:** Other material. **Occurrence:** individualCount: 1; sex: female; **Location:** locationID: M2; continent: Europe; country: Spain; countryCode: ES; stateProvince: Extremadura; county: Cáceres; locality: Fuente del Frances; verbatimElevation: 320.72; decimalLatitude: 39.828; decimalLongitude: -6.03249; geodeticDatum: WGS84; **Event:** eventID: 1; samplingProtocol: Beating; eventTime: Night
- **Type status:** Other material. **Occurrence:** individualCount: 2; sex: female; **Location:** locationID: M2; continent: Europe; country: Spain; countryCode: ES; stateProvince: Extremadura; county: Cáceres; locality: Fuente del Frances; verbatimElevation: 320.72; decimalLatitude: 39.828; decimalLongitude: -6.03249; geodeticDatum: WGS84; **Event:** eventID: 2; samplingProtocol: Beating; eventTime: Day
- **Type status:** Other material. **Occurrence:** individualCount: 2; sex: female; **Location:** locationID: M2; continent: Europe; country: Spain; countryCode: ES; stateProvince: Extremadura; county: Cáceres; locality: Fuente del Frances; verbatimElevation: 320.72; decimalLatitude: 39.828; decimalLongitude: -6.03249; geodeticDatum: WGS84; **Event:** eventID: 1; samplingProtocol: Sweeping; eventTime: Night
- **Type status:** Other material. **Occurrence:** individualCount: 3; sex: male; **Location:** locationID: O1; continent: Europe; country: Spain; countryCode: ES; stateProvince: Aragón; county: Huesca; locality: O Furno; verbatimElevation: 1396.73; decimalLatitude: 42.60677; decimalLongitude: 0.13135; geodeticDatum: WGS84; **Event:** eventID: 1; samplingProtocol: Aerial; eventTime: Night
- **Type status:** Other material. **Occurrence:** individualCount: 2; sex: female; **Location:** locationID: O1; continent: Europe; country: Spain; countryCode: ES; stateProvince: Aragón; county: Huesca; locality: O Furno; verbatimElevation: 1396.73; decimalLatitude: 42.60677; decimalLongitude: 0.13135; geodeticDatum: WGS84; **Event:** eventID: 1; samplingProtocol: Aerial; eventTime: Night
- **Type status:** Other material. **Occurrence:** individualCount: 2; sex: male; **Location:** locationID: O1; continent: Europe; country: Spain; countryCode: ES; stateProvince: Aragón; county: Huesca; locality: O Furno; verbatimElevation: 1396.73; decimalLatitude: 42.60677; decimalLongitude: 0.13135; geodeticDatum: WGS84; **Event:** eventID: 1; samplingProtocol: Aerial; eventTime: Night
- **Type status:** Other material. **Occurrence:** individualCount: 1; sex: male; **Location:** locationID: O1; continent: Europe; country: Spain; countryCode: ES; stateProvince: Aragón; county: Huesca; locality: O Furno; verbatimElevation: 1396.73; decimalLatitude: 42.60677; decimalLongitude: 0.13135; geodeticDatum: WGS84; **Event:** eventID: 1; samplingProtocol: Sweeping; eventTime: Night
- **Type status:** Other material. **Occurrence:** individualCount: 2; sex: female; **Location:** locationID: O1; continent: Europe; country: Spain; countryCode: ES; stateProvince: Aragón; county: Huesca; locality: O Furno; verbatimElevation: 1396.73; decimalLatitude: 42.60677; decimalLongitude: 0.13135; geodeticDatum: WGS84; **Event:** eventID: 1; samplingProtocol: Sweeping; eventTime: Night
- **Type status:** Other material. **Occurrence:** individualCount: 1; sex: male; **Location:** locationID: O2; continent: Europe; country: Spain; countryCode: ES; stateProvince: Aragón; county: Huesca; locality: Rebilla; verbatimElevation: 1158.13; decimalLatitude: 42.59427; decimalLongitude: 0.1529; geodeticDatum: WGS84; **Event:** eventID: 1; samplingProtocol: Aerial; eventTime: Night
- **Type status:** Other material. **Occurrence:** individualCount: 1; sex: male; **Location:** locationID: O2; continent: Europe; country: Spain; countryCode: ES; stateProvince: Aragón; county: Huesca; locality: Rebilla; verbatimElevation: 1158.13; decimalLatitude: 42.59427; decimalLongitude: 0.1529; geodeticDatum: WGS84; **Event:** eventID: 1; samplingProtocol: Aerial; eventTime: Night
- **Type status:** Other material. **Occurrence:** individualCount: 3; sex: male; **Location:** locationID: O2; continent: Europe; country: Spain; countryCode: ES; stateProvince: Aragón; county: Huesca; locality: Rebilla; verbatimElevation: 1158.13; decimalLatitude: 42.59427; decimalLongitude: 0.1529; geodeticDatum: WGS84; **Event:** eventID: 2; samplingProtocol: Aerial; eventTime: Night
- **Type status:** Other material. **Occurrence:** individualCount: 4; sex: female; **Location:** locationID: O2; continent: Europe; country: Spain; countryCode: ES; stateProvince: Aragón; county: Huesca; locality: Rebilla; verbatimElevation: 1158.13; decimalLatitude: 42.59427; decimalLongitude: 0.1529; geodeticDatum: WGS84; **Event:** eventID: 1; samplingProtocol: Beating; eventTime: Day
- **Type status:** Other material. **Occurrence:** individualCount: 1; sex: female; **Location:** locationID: O2; continent: Europe; country: Spain; countryCode: ES; stateProvince: Aragón; county: Huesca; locality: Rebilla; verbatimElevation: 1158.13; decimalLatitude: 42.59427; decimalLongitude: 0.1529; geodeticDatum: WGS84; **Event:** eventID: 2; samplingProtocol: Beating; eventTime: Day
- **Type status:** Other material. **Occurrence:** individualCount: 1; sex: female; **Location:** locationID: O2; continent: Europe; country: Spain; countryCode: ES; stateProvince: Aragón; county: Huesca; locality: Rebilla; verbatimElevation: 1158.13; decimalLatitude: 42.59427; decimalLongitude: 0.1529; geodeticDatum: WGS84; **Event:** eventID: 1; samplingProtocol: Beating; eventTime: Night
- **Type status:** Other material. **Occurrence:** individualCount: 2; sex: female; **Location:** locationID: O2; continent: Europe; country: Spain; countryCode: ES; stateProvince: Aragón; county: Huesca; locality: Rebilla; verbatimElevation: 1158.13; decimalLatitude: 42.59427; decimalLongitude: 0.1529; geodeticDatum: WGS84; **Event:** eventID: 1; samplingProtocol: Beating; eventTime: Night
- **Type status:** Other material. **Occurrence:** individualCount: 1; sex: female; **Location:** locationID: O2; continent: Europe; country: Spain; countryCode: ES; stateProvince: Aragón; county: Huesca; locality: Rebilla; verbatimElevation: 1158.13; decimalLatitude: 42.59427; decimalLongitude: 0.1529; geodeticDatum: WGS84; **Event:** eventID: 1; samplingProtocol: Sweeping; eventTime: Day
- **Type status:** Other material. **Occurrence:** individualCount: 1; sex: female; **Location:** locationID: O2; continent: Europe; country: Spain; countryCode: ES; stateProvince: Aragón; county: Huesca; locality: Rebilla; verbatimElevation: 1158.13; decimalLatitude: 42.59427; decimalLongitude: 0.1529; geodeticDatum: WGS84; **Event:** eventID: 1; samplingProtocol: Sweeping; eventTime: Day
- **Type status:** Other material. **Occurrence:** individualCount: 1; sex: female; **Location:** locationID: O2; continent: Europe; country: Spain; countryCode: ES; stateProvince: Aragón; county: Huesca; locality: Rebilla; verbatimElevation: 1158.13; decimalLatitude: 42.59427; decimalLongitude: 0.1529; geodeticDatum: WGS84; **Event:** eventID: 1; samplingProtocol: Sweeping; eventTime: Night
- **Type status:** Other material. **Occurrence:** individualCount: 3; sex: female; **Location:** locationID: O2; continent: Europe; country: Spain; countryCode: ES; stateProvince: Aragón; county: Huesca; locality: Rebilla; verbatimElevation: 1158.13; decimalLatitude: 42.59427; decimalLongitude: 0.1529; geodeticDatum: WGS84; **Event:** eventID: 1; samplingProtocol: Sweeping; eventTime: Night
- **Type status:** Other material. **Occurrence:** individualCount: 1; sex: female; **Location:** locationID: P2; continent: Europe; country: Spain; countryCode: ES; stateProvince: Castilla y León; county: León; locality: Joyoguelas; verbatimElevation: 763.98; decimalLatitude: 43.17771; decimalLongitude: -4.90579; geodeticDatum: WGS84; **Event:** eventID: 2; samplingProtocol: Aerial; eventTime: Night
- **Type status:** Other material. **Occurrence:** individualCount: 1; sex: female; **Location:** locationID: P2; continent: Europe; country: Spain; countryCode: ES; stateProvince: Castilla y León; county: León; locality: Joyoguelas; verbatimElevation: 763.98; decimalLatitude: 43.17771; decimalLongitude: -4.90579; geodeticDatum: WGS84; **Event:** eventID: 1; samplingProtocol: Sweeping; eventTime: Night
- **Type status:** Other material. **Occurrence:** individualCount: 1; sex: female; **Location:** locationID: P2; continent: Europe; country: Spain; countryCode: ES; stateProvince: Castilla y León; county: León; locality: Joyoguelas; verbatimElevation: 763.98; decimalLatitude: 43.17771; decimalLongitude: -4.90579; geodeticDatum: WGS84; **Event:** eventID: 1; samplingProtocol: Sweeping; eventTime: Night
- **Type status:** Other material. **Occurrence:** individualCount: 1; sex: female; **Location:** locationID: P3; continent: Europe; country: Spain; countryCode: ES; stateProvince: Castilla y León; county: León; locality: Joyoguelas; verbatimElevation: 763.98; decimalLatitude: 43.17771; decimalLongitude: -4.90579; geodeticDatum: WGS84; **Event:** eventID: 1; samplingProtocol: Beating; eventTime: Night
- **Type status:** Other material. **Occurrence:** individualCount: 2; sex: male; **Location:** locationID: P4; continent: Europe; country: Spain; countryCode: ES; stateProvince: Castilla y León; county: León; locality: Joyoguelas; verbatimElevation: 763.98; decimalLatitude: 43.17771; decimalLongitude: -4.90579; geodeticDatum: WGS84; **Event:** eventID: 1; samplingProtocol: Beating; eventTime: Night
- **Type status:** Other material. **Occurrence:** individualCount: 2; sex: female; **Location:** locationID: P4; continent: Europe; country: Spain; countryCode: ES; stateProvince: Castilla y León; county: León; locality: Joyoguelas; verbatimElevation: 763.98; decimalLatitude: 43.17771; decimalLongitude: -4.90579; geodeticDatum: WGS84; **Event:** eventID: 1; samplingProtocol: Beating; eventTime: Night
- **Type status:** Other material. **Occurrence:** individualCount: 3; sex: female; **Location:** locationID: S1; continent: Europe; country: Spain; countryCode: ES; stateProvince: Andalucía; county: Granada; locality: Soportujar; verbatimElevation: 1786.57; decimalLatitude: 36.96151; decimalLongitude: -3.41881; geodeticDatum: WGS84; **Event:** eventID: 3; samplingProtocol: Aerial; eventTime: Night
- **Type status:** Other material. **Occurrence:** individualCount: 2; sex: male; **Location:** locationID: S1; continent: Europe; country: Spain; countryCode: ES; stateProvince: Andalucía; county: Granada; locality: Soportujar; verbatimElevation: 1786.57; decimalLatitude: 36.96151; decimalLongitude: -3.41881; geodeticDatum: WGS84; **Event:** eventID: 3; samplingProtocol: Aerial; eventTime: Night
- **Type status:** Other material. **Occurrence:** individualCount: 5; sex: female; **Location:** locationID: S1; continent: Europe; country: Spain; countryCode: ES; stateProvince: Andalucía; county: Granada; locality: Soportujar; verbatimElevation: 1786.57; decimalLatitude: 36.96151; decimalLongitude: -3.41881; geodeticDatum: WGS84; **Event:** eventID: 2; samplingProtocol: Beating; eventTime: Night
- **Type status:** Other material. **Occurrence:** individualCount: 1; sex: male; **Location:** locationID: S1; continent: Europe; country: Spain; countryCode: ES; stateProvince: Andalucía; county: Granada; locality: Soportujar; verbatimElevation: 1786.57; decimalLatitude: 36.96151; decimalLongitude: -3.41881; geodeticDatum: WGS84; **Event:** eventID: 2; samplingProtocol: Beating; eventTime: Day
- **Type status:** Other material. **Occurrence:** individualCount: 1; sex: female; **Location:** locationID: S1; continent: Europe; country: Spain; countryCode: ES; stateProvince: Andalucía; county: Granada; locality: Soportujar; verbatimElevation: 1786.57; decimalLatitude: 36.96151; decimalLongitude: -3.41881; geodeticDatum: WGS84; **Event:** eventID: 2; samplingProtocol: Sweeping; eventTime: Night
- **Type status:** Other material. **Occurrence:** individualCount: 8; sex: female; **Location:** locationID: S1; continent: Europe; country: Spain; countryCode: ES; stateProvince: Andalucía; county: Granada; locality: Soportujar; verbatimElevation: 1786.57; decimalLatitude: 36.96151; decimalLongitude: -3.41881; geodeticDatum: WGS84; **Event:** eventID: 2; samplingProtocol: Sweeping; eventTime: Day
- **Type status:** Other material. **Occurrence:** individualCount: 2; sex: male; **Location:** locationID: S2; continent: Europe; country: Spain; countryCode: ES; stateProvince: Andalucía; county: Granada; locality: Camarate; verbatimElevation: 1713.96; decimalLatitude: 37.18377; decimalLongitude: -3.26282; geodeticDatum: WGS84; **Event:** eventID: 1; samplingProtocol: Aerial; eventTime: Night
- **Type status:** Other material. **Occurrence:** individualCount: 3; sex: female; **Location:** locationID: S2; continent: Europe; country: Spain; countryCode: ES; stateProvince: Andalucía; county: Granada; locality: Camarate; verbatimElevation: 1713.96; decimalLatitude: 37.18377; decimalLongitude: -3.26282; geodeticDatum: WGS84; **Event:** eventID: 1; samplingProtocol: Aerial; eventTime: Night
- **Type status:** Other material. **Occurrence:** individualCount: 1; sex: female; **Location:** locationID: S2; continent: Europe; country: Spain; countryCode: ES; stateProvince: Andalucía; county: Granada; locality: Camarate; verbatimElevation: 1713.96; decimalLatitude: 37.18377; decimalLongitude: -3.26282; geodeticDatum: WGS84; **Event:** eventID: 4; samplingProtocol: Aerial; eventTime: Night
- **Type status:** Other material. **Occurrence:** individualCount: 1; sex: female; **Location:** locationID: S2; continent: Europe; country: Spain; countryCode: ES; stateProvince: Andalucía; county: Granada; locality: Camarate; verbatimElevation: 1713.96; decimalLatitude: 37.18377; decimalLongitude: -3.26282; geodeticDatum: WGS84; **Event:** eventID: 1; samplingProtocol: Beating; eventTime: Day
- **Type status:** Other material. **Occurrence:** individualCount: 3; sex: male; **Location:** locationID: S2; continent: Europe; country: Spain; countryCode: ES; stateProvince: Andalucía; county: Granada; locality: Camarate; verbatimElevation: 1713.96; decimalLatitude: 37.18377; decimalLongitude: -3.26282; geodeticDatum: WGS84; **Event:** eventID: 1; samplingProtocol: Beating; eventTime: Day
- **Type status:** Other material. **Occurrence:** individualCount: 1; sex: female; **Location:** locationID: S2; continent: Europe; country: Spain; countryCode: ES; stateProvince: Andalucía; county: Granada; locality: Camarate; verbatimElevation: 1713.96; decimalLatitude: 37.18377; decimalLongitude: -3.26282; geodeticDatum: WGS84; **Event:** eventID: 2; samplingProtocol: Beating; eventTime: Day
- **Type status:** Other material. **Occurrence:** individualCount: 2; sex: female; **Location:** locationID: S2; continent: Europe; country: Spain; countryCode: ES; stateProvince: Andalucía; county: Granada; locality: Camarate; verbatimElevation: 1713.96; decimalLatitude: 37.18377; decimalLongitude: -3.26282; geodeticDatum: WGS84; **Event:** eventID: 2; samplingProtocol: Beating; eventTime: Night
- **Type status:** Other material. **Occurrence:** individualCount: 1; sex: female; **Location:** locationID: S2; continent: Europe; country: Spain; countryCode: ES; stateProvince: Andalucía; county: Granada; locality: Camarate; verbatimElevation: 1713.96; decimalLatitude: 37.18377; decimalLongitude: -3.26282; geodeticDatum: WGS84; **Event:** eventID: 1; samplingProtocol: Beating; eventTime: Night
- **Type status:** Other material. **Occurrence:** individualCount: 1; sex: female; **Location:** locationID: S2; continent: Europe; country: Spain; countryCode: ES; stateProvince: Andalucía; county: Granada; locality: Camarate; verbatimElevation: 1713.96; decimalLatitude: 37.18377; decimalLongitude: -3.26282; geodeticDatum: WGS84; **Event:** eventID: 1; samplingProtocol: Sweeping; eventTime: Night
- **Type status:** Other material. **Occurrence:** individualCount: 3; sex: female; **Location:** locationID: S2; continent: Europe; country: Spain; countryCode: ES; stateProvince: Andalucía; county: Granada; locality: Camarate; verbatimElevation: 1713.96; decimalLatitude: 37.18377; decimalLongitude: -3.26282; geodeticDatum: WGS84; **Event:** eventID: 1; samplingProtocol: Sweeping; eventTime: Night
- **Type status:** Other material. **Occurrence:** individualCount: 2; sex: female; **Location:** locationID: S2; continent: Europe; country: Spain; countryCode: ES; stateProvince: Andalucía; county: Granada; locality: Camarate; verbatimElevation: 1713.96; decimalLatitude: 37.18377; decimalLongitude: -3.26282; geodeticDatum: WGS84; **Event:** eventID: 2; samplingProtocol: Sweeping; eventTime: Night
- **Type status:** Other material. **Occurrence:** individualCount: 1; sex: female; **Location:** locationID: S2; continent: Europe; country: Spain; countryCode: ES; stateProvince: Andalucía; county: Granada; locality: Camarate; verbatimElevation: 1713.96; decimalLatitude: 37.18377; decimalLongitude: -3.26282; geodeticDatum: WGS84; **Event:** eventID: 2; samplingProtocol: Sweeping; eventTime: Day

##### Distribution

Iberian Peninsula, France

#### Theridion mystaceum

L. Koch, 1870

##### Materials

- **Type status:** Other material. **Occurrence:** individualCount: 3; sex: female; **Location:** locationID: A1; continent: Europe; country: Spain; countryCode: ES; stateProvince: Catalonia; county: Lleida; locality: Sola de Boi; verbatimElevation: 1759.8; decimalLatitude: 42.54958; decimalLongitude: 0.87254; geodeticDatum: WGS84; **Event:** eventID: 1; samplingProtocol: Aerial; eventTime: Night
- **Type status:** Other material. **Occurrence:** individualCount: 2; sex: female; **Location:** locationID: A1; continent: Europe; country: Spain; countryCode: ES; stateProvince: Catalonia; county: Lleida; locality: Sola de Boi; verbatimElevation: 1759.8; decimalLatitude: 42.54958; decimalLongitude: 0.87254; geodeticDatum: WGS84; **Event:** eventID: 2; samplingProtocol: Aerial; eventTime: Night
- **Type status:** Other material. **Occurrence:** individualCount: 2; sex: female; **Location:** locationID: A1; continent: Europe; country: Spain; countryCode: ES; stateProvince: Catalonia; county: Lleida; locality: Sola de Boi; verbatimElevation: 1759.8; decimalLatitude: 42.54958; decimalLongitude: 0.87254; geodeticDatum: WGS84; **Event:** eventID: 2; samplingProtocol: Ground; eventTime: Night
- **Type status:** Other material. **Occurrence:** individualCount: 1; sex: male; **Location:** locationID: A1; continent: Europe; country: Spain; countryCode: ES; stateProvince: Catalonia; county: Lleida; locality: Sola de Boi; verbatimElevation: 1759.8; decimalLatitude: 42.54958; decimalLongitude: 0.87254; geodeticDatum: WGS84; **Event:** eventID: 1; samplingProtocol: Sweeping; eventTime: Night
- **Type status:** Other material. **Occurrence:** individualCount: 1; sex: male; **Location:** locationID: A1; continent: Europe; country: Spain; countryCode: ES; stateProvince: Catalonia; county: Lleida; locality: Sola de Boi; verbatimElevation: 1759.8; decimalLatitude: 42.54958; decimalLongitude: 0.87254; geodeticDatum: WGS84; **Event:** eventID: 1; samplingProtocol: Sweeping; eventTime: Night
- **Type status:** Other material. **Occurrence:** individualCount: 1; sex: female; **Location:** locationID: A2; continent: Europe; country: Spain; countryCode: ES; stateProvince: Catalonia; county: Lleida; locality: Sola de Boi; verbatimElevation: 1738.7; decimalLatitude: 42.54913; decimalLongitude: 0.87137; geodeticDatum: WGS84; **Event:** eventID: 2; samplingProtocol: Aerial; eventTime: Night
- **Type status:** Other material. **Occurrence:** individualCount: 2; sex: male; **Location:** locationID: O1; continent: Europe; country: Spain; countryCode: ES; stateProvince: Aragón; county: Huesca; locality: O Furno; verbatimElevation: 1396.73; decimalLatitude: 42.60677; decimalLongitude: 0.13135; geodeticDatum: WGS84; **Event:** eventID: 1; samplingProtocol: Aerial; eventTime: Night
- **Type status:** Other material. **Occurrence:** individualCount: 3; sex: female; **Location:** locationID: O1; continent: Europe; country: Spain; countryCode: ES; stateProvince: Aragón; county: Huesca; locality: O Furno; verbatimElevation: 1396.73; decimalLatitude: 42.60677; decimalLongitude: 0.13135; geodeticDatum: WGS84; **Event:** eventID: 1; samplingProtocol: Aerial; eventTime: Night
- **Type status:** Other material. **Occurrence:** individualCount: 2; sex: male; **Location:** locationID: O1; continent: Europe; country: Spain; countryCode: ES; stateProvince: Aragón; county: Huesca; locality: O Furno; verbatimElevation: 1396.73; decimalLatitude: 42.60677; decimalLongitude: 0.13135; geodeticDatum: WGS84; **Event:** eventID: 1; samplingProtocol: Aerial; eventTime: Night
- **Type status:** Other material. **Occurrence:** individualCount: 2; sex: female; **Location:** locationID: O1; continent: Europe; country: Spain; countryCode: ES; stateProvince: Aragón; county: Huesca; locality: O Furno; verbatimElevation: 1396.73; decimalLatitude: 42.60677; decimalLongitude: 0.13135; geodeticDatum: WGS84; **Event:** eventID: 1; samplingProtocol: Aerial; eventTime: Night
- **Type status:** Other material. **Occurrence:** individualCount: 1; sex: male; **Location:** locationID: O1; continent: Europe; country: Spain; countryCode: ES; stateProvince: Aragón; county: Huesca; locality: O Furno; verbatimElevation: 1396.73; decimalLatitude: 42.60677; decimalLongitude: 0.13135; geodeticDatum: WGS84; **Event:** eventID: 2; samplingProtocol: Aerial; eventTime: Night
- **Type status:** Other material. **Occurrence:** individualCount: 2; sex: female; **Location:** locationID: O1; continent: Europe; country: Spain; countryCode: ES; stateProvince: Aragón; county: Huesca; locality: O Furno; verbatimElevation: 1396.73; decimalLatitude: 42.60677; decimalLongitude: 0.13135; geodeticDatum: WGS84; **Event:** eventID: 2; samplingProtocol: Aerial; eventTime: Night
- **Type status:** Other material. **Occurrence:** individualCount: 3; sex: male; **Location:** locationID: O1; continent: Europe; country: Spain; countryCode: ES; stateProvince: Aragón; county: Huesca; locality: O Furno; verbatimElevation: 1396.73; decimalLatitude: 42.60677; decimalLongitude: 0.13135; geodeticDatum: WGS84; **Event:** eventID: 2; samplingProtocol: Aerial; eventTime: Night
- **Type status:** Other material. **Occurrence:** individualCount: 1; sex: male; **Location:** locationID: O1; continent: Europe; country: Spain; countryCode: ES; stateProvince: Aragón; county: Huesca; locality: O Furno; verbatimElevation: 1396.73; decimalLatitude: 42.60677; decimalLongitude: 0.13135; geodeticDatum: WGS84; **Event:** eventID: 1; samplingProtocol: Beating; eventTime: Day
- **Type status:** Other material. **Occurrence:** individualCount: 1; sex: female; **Location:** locationID: O1; continent: Europe; country: Spain; countryCode: ES; stateProvince: Aragón; county: Huesca; locality: O Furno; verbatimElevation: 1396.73; decimalLatitude: 42.60677; decimalLongitude: 0.13135; geodeticDatum: WGS84; **Event:** eventID: 1; samplingProtocol: Beating; eventTime: Night
- **Type status:** Other material. **Occurrence:** individualCount: 1; sex: male; **Location:** locationID: O1; continent: Europe; country: Spain; countryCode: ES; stateProvince: Aragón; county: Huesca; locality: O Furno; verbatimElevation: 1396.73; decimalLatitude: 42.60677; decimalLongitude: 0.13135; geodeticDatum: WGS84; **Event:** eventID: 1; samplingProtocol: Beating; eventTime: Night
- **Type status:** Other material. **Occurrence:** individualCount: 1; sex: male; **Location:** locationID: O2; continent: Europe; country: Spain; countryCode: ES; stateProvince: Aragón; county: Huesca; locality: O Furno; verbatimElevation: 1396.73; decimalLatitude: 42.60677; decimalLongitude: 0.13135; geodeticDatum: WGS84; **Event:** eventID: 2; samplingProtocol: Aerial; eventTime: Night
- **Type status:** Other material. **Occurrence:** individualCount: 1; sex: male; **Location:** locationID: O2; continent: Europe; country: Spain; countryCode: ES; stateProvince: Aragón; county: Huesca; locality: O Furno; verbatimElevation: 1396.73; decimalLatitude: 42.60677; decimalLongitude: 0.13135; geodeticDatum: WGS84; **Event:** eventID: 4; samplingProtocol: Aerial; eventTime: Night
- **Type status:** Other material. **Occurrence:** individualCount: 1; sex: male; **Location:** locationID: P1; continent: Europe; country: Spain; countryCode: ES; stateProvince: Castilla y León; county: León; locality: Monte Robledo; verbatimElevation: 1071.58; decimalLatitude: 43.1445; decimalLongitude: -4.92675; geodeticDatum: WGS84; **Event:** eventID: 1; samplingProtocol: Aerial; eventTime: Night
- **Type status:** Other material. **Occurrence:** individualCount: 1; sex: female; **Location:** locationID: P1; continent: Europe; country: Spain; countryCode: ES; stateProvince: Castilla y León; county: León; locality: Monte Robledo; verbatimElevation: 1071.58; decimalLatitude: 43.1445; decimalLongitude: -4.92675; geodeticDatum: WGS84; **Event:** eventID: 1; samplingProtocol: Aerial; eventTime: Night
- **Type status:** Other material. **Occurrence:** individualCount: 3; sex: female; **Location:** locationID: P1; continent: Europe; country: Spain; countryCode: ES; stateProvince: Castilla y León; county: León; locality: Monte Robledo; verbatimElevation: 1071.58; decimalLatitude: 43.1445; decimalLongitude: -4.92675; geodeticDatum: WGS84; **Event:** eventID: 1; samplingProtocol: Aerial; eventTime: Night
- **Type status:** Other material. **Occurrence:** individualCount: 7; sex: female; **Location:** locationID: P1; continent: Europe; country: Spain; countryCode: ES; stateProvince: Castilla y León; county: León; locality: Monte Robledo; verbatimElevation: 1071.58; decimalLatitude: 43.1445; decimalLongitude: -4.92675; geodeticDatum: WGS84; **Event:** eventID: 1; samplingProtocol: Aerial; eventTime: Night
- **Type status:** Other material. **Occurrence:** individualCount: 6; sex: male; **Location:** locationID: P1; continent: Europe; country: Spain; countryCode: ES; stateProvince: Castilla y León; county: León; locality: Monte Robledo; verbatimElevation: 1071.58; decimalLatitude: 43.1445; decimalLongitude: -4.92675; geodeticDatum: WGS84; **Event:** eventID: 2; samplingProtocol: Aerial; eventTime: Night
- **Type status:** Other material. **Occurrence:** individualCount: 1; sex: female; **Location:** locationID: P1; continent: Europe; country: Spain; countryCode: ES; stateProvince: Castilla y León; county: León; locality: Monte Robledo; verbatimElevation: 1071.58; decimalLatitude: 43.1445; decimalLongitude: -4.92675; geodeticDatum: WGS84; **Event:** eventID: 2; samplingProtocol: Aerial; eventTime: Night
- **Type status:** Other material. **Occurrence:** individualCount: 2; sex: male; **Location:** locationID: P1; continent: Europe; country: Spain; countryCode: ES; stateProvince: Castilla y León; county: León; locality: Monte Robledo; verbatimElevation: 1071.58; decimalLatitude: 43.1445; decimalLongitude: -4.92675; geodeticDatum: WGS84; **Event:** eventID: 2; samplingProtocol: Aerial; eventTime: Night
- **Type status:** Other material. **Occurrence:** individualCount: 11; sex: female; **Location:** locationID: P1; continent: Europe; country: Spain; countryCode: ES; stateProvince: Castilla y León; county: León; locality: Monte Robledo; verbatimElevation: 1071.58; decimalLatitude: 43.1445; decimalLongitude: -4.92675; geodeticDatum: WGS84; **Event:** eventID: 2; samplingProtocol: Aerial; eventTime: Night
- **Type status:** Other material. **Occurrence:** individualCount: 6; sex: male; **Location:** locationID: P1; continent: Europe; country: Spain; countryCode: ES; stateProvince: Castilla y León; county: León; locality: Monte Robledo; verbatimElevation: 1071.58; decimalLatitude: 43.1445; decimalLongitude: -4.92675; geodeticDatum: WGS84; **Event:** eventID: 1; samplingProtocol: Beating; eventTime: Day
- **Type status:** Other material. **Occurrence:** individualCount: 3; sex: female; **Location:** locationID: P1; continent: Europe; country: Spain; countryCode: ES; stateProvince: Castilla y León; county: León; locality: Monte Robledo; verbatimElevation: 1071.58; decimalLatitude: 43.1445; decimalLongitude: -4.92675; geodeticDatum: WGS84; **Event:** eventID: 1; samplingProtocol: Beating; eventTime: Day
- **Type status:** Other material. **Occurrence:** individualCount: 2; sex: male; **Location:** locationID: P1; continent: Europe; country: Spain; countryCode: ES; stateProvince: Castilla y León; county: León; locality: Monte Robledo; verbatimElevation: 1071.58; decimalLatitude: 43.1445; decimalLongitude: -4.92675; geodeticDatum: WGS84; **Event:** eventID: 2; samplingProtocol: Beating; eventTime: Day
- **Type status:** Other material. **Occurrence:** individualCount: 1; sex: female; **Location:** locationID: P1; continent: Europe; country: Spain; countryCode: ES; stateProvince: Castilla y León; county: León; locality: Monte Robledo; verbatimElevation: 1071.58; decimalLatitude: 43.1445; decimalLongitude: -4.92675; geodeticDatum: WGS84; **Event:** eventID: 2; samplingProtocol: Beating; eventTime: Day
- **Type status:** Other material. **Occurrence:** individualCount: 2; sex: male; **Location:** locationID: P1; continent: Europe; country: Spain; countryCode: ES; stateProvince: Castilla y León; county: León; locality: Monte Robledo; verbatimElevation: 1071.58; decimalLatitude: 43.1445; decimalLongitude: -4.92675; geodeticDatum: WGS84; **Event:** eventID: 1; samplingProtocol: Beating; eventTime: Night
- **Type status:** Other material. **Occurrence:** individualCount: 12; sex: female; **Location:** locationID: P1; continent: Europe; country: Spain; countryCode: ES; stateProvince: Castilla y León; county: León; locality: Monte Robledo; verbatimElevation: 1071.58; decimalLatitude: 43.1445; decimalLongitude: -4.92675; geodeticDatum: WGS84; **Event:** eventID: 1; samplingProtocol: Beating; eventTime: Night
- **Type status:** Other material. **Occurrence:** individualCount: 2; sex: male; **Location:** locationID: P1; continent: Europe; country: Spain; countryCode: ES; stateProvince: Castilla y León; county: León; locality: Monte Robledo; verbatimElevation: 1071.58; decimalLatitude: 43.1445; decimalLongitude: -4.92675; geodeticDatum: WGS84; **Event:** eventID: 1; samplingProtocol: Beating; eventTime: Night
- **Type status:** Other material. **Occurrence:** individualCount: 1; sex: female; **Location:** locationID: P1; continent: Europe; country: Spain; countryCode: ES; stateProvince: Castilla y León; county: León; locality: Monte Robledo; verbatimElevation: 1071.58; decimalLatitude: 43.1445; decimalLongitude: -4.92675; geodeticDatum: WGS84; **Event:** eventID: 1; samplingProtocol: Beating; eventTime: Night
- **Type status:** Other material. **Occurrence:** individualCount: 1; sex: male; **Location:** locationID: P1; continent: Europe; country: Spain; countryCode: ES; stateProvince: Castilla y León; county: León; locality: Monte Robledo; verbatimElevation: 1071.58; decimalLatitude: 43.1445; decimalLongitude: -4.92675; geodeticDatum: WGS84; **Event:** eventID: 1; samplingProtocol: Sweeping; eventTime: Day
- **Type status:** Other material. **Occurrence:** individualCount: 1; sex: male; **Location:** locationID: P1; continent: Europe; country: Spain; countryCode: ES; stateProvince: Castilla y León; county: León; locality: Monte Robledo; verbatimElevation: 1071.58; decimalLatitude: 43.1445; decimalLongitude: -4.92675; geodeticDatum: WGS84; **Event:** eventID: 2; samplingProtocol: Sweeping; eventTime: Day
- **Type status:** Other material. **Occurrence:** individualCount: 8; sex: female; **Location:** locationID: P1; continent: Europe; country: Spain; countryCode: ES; stateProvince: Castilla y León; county: León; locality: Monte Robledo; verbatimElevation: 1071.58; decimalLatitude: 43.1445; decimalLongitude: -4.92675; geodeticDatum: WGS84; **Event:** eventID: 1; samplingProtocol: Sweeping; eventTime: Night
- **Type status:** Other material. **Occurrence:** individualCount: 2; sex: male; **Location:** locationID: P2; continent: Europe; country: Spain; countryCode: ES; stateProvince: Castilla y León; county: León; locality: Joyoguelas; verbatimElevation: 763.98; decimalLatitude: 43.17771; decimalLongitude: -4.90579; geodeticDatum: WGS84; **Event:** eventID: 1; samplingProtocol: Aerial; eventTime: Night
- **Type status:** Other material. **Occurrence:** individualCount: 1; sex: male; **Location:** locationID: P2; continent: Europe; country: Spain; countryCode: ES; stateProvince: Castilla y León; county: León; locality: Joyoguelas; verbatimElevation: 763.98; decimalLatitude: 43.17771; decimalLongitude: -4.90579; geodeticDatum: WGS84; **Event:** eventID: 1; samplingProtocol: Aerial; eventTime: Night
- **Type status:** Other material. **Occurrence:** individualCount: 2; sex: male; **Location:** locationID: P2; continent: Europe; country: Spain; countryCode: ES; stateProvince: Castilla y León; county: León; locality: Joyoguelas; verbatimElevation: 763.98; decimalLatitude: 43.17771; decimalLongitude: -4.90579; geodeticDatum: WGS84; **Event:** eventID: 1; samplingProtocol: Aerial; eventTime: Night
- **Type status:** Other material. **Occurrence:** individualCount: 1; sex: female; **Location:** locationID: P2; continent: Europe; country: Spain; countryCode: ES; stateProvince: Castilla y León; county: León; locality: Joyoguelas; verbatimElevation: 763.98; decimalLatitude: 43.17771; decimalLongitude: -4.90579; geodeticDatum: WGS84; **Event:** eventID: 2; samplingProtocol: Aerial; eventTime: Night
- **Type status:** Other material. **Occurrence:** individualCount: 1; sex: male; **Location:** locationID: P2; continent: Europe; country: Spain; countryCode: ES; stateProvince: Castilla y León; county: León; locality: Joyoguelas; verbatimElevation: 763.98; decimalLatitude: 43.17771; decimalLongitude: -4.90579; geodeticDatum: WGS84; **Event:** eventID: 2; samplingProtocol: Aerial; eventTime: Night
- **Type status:** Other material. **Occurrence:** individualCount: 4; sex: female; **Location:** locationID: P2; continent: Europe; country: Spain; countryCode: ES; stateProvince: Castilla y León; county: León; locality: Joyoguelas; verbatimElevation: 763.98; decimalLatitude: 43.17771; decimalLongitude: -4.90579; geodeticDatum: WGS84; **Event:** eventID: 1; samplingProtocol: Beating; eventTime: Day
- **Type status:** Other material. **Occurrence:** individualCount: 4; sex: female; **Location:** locationID: P2; continent: Europe; country: Spain; countryCode: ES; stateProvince: Castilla y León; county: León; locality: Joyoguelas; verbatimElevation: 763.98; decimalLatitude: 43.17771; decimalLongitude: -4.90579; geodeticDatum: WGS84; **Event:** eventID: 2; samplingProtocol: Beating; eventTime: Day
- **Type status:** Other material. **Occurrence:** individualCount: 1; sex: female; **Location:** locationID: P2; continent: Europe; country: Spain; countryCode: ES; stateProvince: Castilla y León; county: León; locality: Joyoguelas; verbatimElevation: 763.98; decimalLatitude: 43.17771; decimalLongitude: -4.90579; geodeticDatum: WGS84; **Event:** eventID: 1; samplingProtocol: Beating; eventTime: Night
- **Type status:** Other material. **Occurrence:** individualCount: 2; sex: male; **Location:** locationID: P2; continent: Europe; country: Spain; countryCode: ES; stateProvince: Castilla y León; county: León; locality: Joyoguelas; verbatimElevation: 763.98; decimalLatitude: 43.17771; decimalLongitude: -4.90579; geodeticDatum: WGS84; **Event:** eventID: 1; samplingProtocol: Beating; eventTime: Night
- **Type status:** Other material. **Occurrence:** individualCount: 8; sex: female; **Location:** locationID: P3; continent: Europe; country: Spain; countryCode: ES; stateProvince: Castilla y León; county: León; locality: Las Arroyas; verbatimElevation: 1097.1; decimalLatitude: 43.14351; decimalLongitude: -4.94878; geodeticDatum: WGS84; **Event:** eventID: 1; samplingProtocol: Aerial; eventTime: Night
- **Type status:** Other material. **Occurrence:** individualCount: 5; sex: female; **Location:** locationID: P3; continent: Europe; country: Spain; countryCode: ES; stateProvince: Castilla y León; county: León; locality: Las Arroyas; verbatimElevation: 1097.1; decimalLatitude: 43.14351; decimalLongitude: -4.94878; geodeticDatum: WGS84; **Event:** eventID: 2; samplingProtocol: Aerial; eventTime: Night
- **Type status:** Other material. **Occurrence:** individualCount: 1; sex: male; **Location:** locationID: P3; continent: Europe; country: Spain; countryCode: ES; stateProvince: Castilla y León; county: León; locality: Las Arroyas; verbatimElevation: 1097.1; decimalLatitude: 43.14351; decimalLongitude: -4.94878; geodeticDatum: WGS84; **Event:** eventID: 3; samplingProtocol: Aerial; eventTime: Night
- **Type status:** Other material. **Occurrence:** individualCount: 2; sex: female; **Location:** locationID: P3; continent: Europe; country: Spain; countryCode: ES; stateProvince: Castilla y León; county: León; locality: Las Arroyas; verbatimElevation: 1097.1; decimalLatitude: 43.14351; decimalLongitude: -4.94878; geodeticDatum: WGS84; **Event:** eventID: 3; samplingProtocol: Aerial; eventTime: Night
- **Type status:** Other material. **Occurrence:** individualCount: 9; sex: male; **Location:** locationID: P3; continent: Europe; country: Spain; countryCode: ES; stateProvince: Castilla y León; county: León; locality: Las Arroyas; verbatimElevation: 1097.1; decimalLatitude: 43.14351; decimalLongitude: -4.94878; geodeticDatum: WGS84; **Event:** eventID: 4; samplingProtocol: Aerial; eventTime: Night
- **Type status:** Other material. **Occurrence:** individualCount: 1; sex: female; **Location:** locationID: P3; continent: Europe; country: Spain; countryCode: ES; stateProvince: Castilla y León; county: León; locality: Las Arroyas; verbatimElevation: 1097.1; decimalLatitude: 43.14351; decimalLongitude: -4.94878; geodeticDatum: WGS84; **Event:** eventID: 4; samplingProtocol: Aerial; eventTime: Night
- **Type status:** Other material. **Occurrence:** individualCount: 1; sex: male; **Location:** locationID: P3; continent: Europe; country: Spain; countryCode: ES; stateProvince: Castilla y León; county: León; locality: Las Arroyas; verbatimElevation: 1097.1; decimalLatitude: 43.14351; decimalLongitude: -4.94878; geodeticDatum: WGS84; **Event:** eventID: 1; samplingProtocol: Beating; eventTime: Day
- **Type status:** Other material. **Occurrence:** individualCount: 6; sex: female; **Location:** locationID: P3; continent: Europe; country: Spain; countryCode: ES; stateProvince: Castilla y León; county: León; locality: Las Arroyas; verbatimElevation: 1097.1; decimalLatitude: 43.14351; decimalLongitude: -4.94878; geodeticDatum: WGS84; **Event:** eventID: 1; samplingProtocol: Beating; eventTime: Day
- **Type status:** Other material. **Occurrence:** individualCount: 1; sex: male; **Location:** locationID: P3; continent: Europe; country: Spain; countryCode: ES; stateProvince: Castilla y León; county: León; locality: Las Arroyas; verbatimElevation: 1097.1; decimalLatitude: 43.14351; decimalLongitude: -4.94878; geodeticDatum: WGS84; **Event:** eventID: 2; samplingProtocol: Beating; eventTime: Day
- **Type status:** Other material. **Occurrence:** individualCount: 5; sex: female; **Location:** locationID: P3; continent: Europe; country: Spain; countryCode: ES; stateProvince: Castilla y León; county: León; locality: Las Arroyas; verbatimElevation: 1097.1; decimalLatitude: 43.14351; decimalLongitude: -4.94878; geodeticDatum: WGS84; **Event:** eventID: 2; samplingProtocol: Beating; eventTime: Day
- **Type status:** Other material. **Occurrence:** individualCount: 1; sex: male; **Location:** locationID: P3; continent: Europe; country: Spain; countryCode: ES; stateProvince: Castilla y León; county: León; locality: Las Arroyas; verbatimElevation: 1097.1; decimalLatitude: 43.14351; decimalLongitude: -4.94878; geodeticDatum: WGS84; **Event:** eventID: 1; samplingProtocol: Beating; eventTime: Night
- **Type status:** Other material. **Occurrence:** individualCount: 1; sex: female; **Location:** locationID: P3; continent: Europe; country: Spain; countryCode: ES; stateProvince: Castilla y León; county: León; locality: Las Arroyas; verbatimElevation: 1097.1; decimalLatitude: 43.14351; decimalLongitude: -4.94878; geodeticDatum: WGS84; **Event:** eventID: 1; samplingProtocol: Beating; eventTime: Night
- **Type status:** Other material. **Occurrence:** individualCount: 1; sex: male; **Location:** locationID: P3; continent: Europe; country: Spain; countryCode: ES; stateProvince: Castilla y León; county: León; locality: Las Arroyas; verbatimElevation: 1097.1; decimalLatitude: 43.14351; decimalLongitude: -4.94878; geodeticDatum: WGS84; **Event:** eventID: 1; samplingProtocol: Beating; eventTime: Night
- **Type status:** Other material. **Occurrence:** individualCount: 1; sex: female; **Location:** locationID: P3; continent: Europe; country: Spain; countryCode: ES; stateProvince: Castilla y León; county: León; locality: Las Arroyas; verbatimElevation: 1097.1; decimalLatitude: 43.14351; decimalLongitude: -4.94878; geodeticDatum: WGS84; **Event:** eventID: 1; samplingProtocol: Beating; eventTime: Night
- **Type status:** Other material. **Occurrence:** individualCount: 1; sex: female; **Location:** locationID: P3; continent: Europe; country: Spain; countryCode: ES; stateProvince: Castilla y León; county: León; locality: Las Arroyas; verbatimElevation: 1097.1; decimalLatitude: 43.14351; decimalLongitude: -4.94878; geodeticDatum: WGS84; **Event:** eventID: 2; samplingProtocol: Sweeping; eventTime: Day
- **Type status:** Other material. **Occurrence:** individualCount: 1; sex: male; **Location:** locationID: P3; continent: Europe; country: Spain; countryCode: ES; stateProvince: Castilla y León; county: León; locality: Las Arroyas; verbatimElevation: 1097.1; decimalLatitude: 43.14351; decimalLongitude: -4.94878; geodeticDatum: WGS84; **Event:** eventID: 1; samplingProtocol: Sweeping; eventTime: Night
- **Type status:** Other material. **Occurrence:** individualCount: 2; sex: female; **Location:** locationID: P3; continent: Europe; country: Spain; countryCode: ES; stateProvince: Castilla y León; county: León; locality: Las Arroyas; verbatimElevation: 1097.1; decimalLatitude: 43.14351; decimalLongitude: -4.94878; geodeticDatum: WGS84; **Event:** eventID: 1; samplingProtocol: Sweeping; eventTime: Night
- **Type status:** Other material. **Occurrence:** individualCount: 1; sex: male; **Location:** locationID: P4; continent: Europe; country: Spain; countryCode: ES; stateProvince: Castilla y León; county: León; locality: El Canto; verbatimElevation: 943.48; decimalLatitude: 43.17227; decimalLongitude: -4.90857; geodeticDatum: WGS84; **Event:** eventID: 1; samplingProtocol: Aerial; eventTime: Night
- **Type status:** Other material. **Occurrence:** individualCount: 2; sex: male; **Location:** locationID: P4; continent: Europe; country: Spain; countryCode: ES; stateProvince: Castilla y León; county: León; locality: El Canto; verbatimElevation: 943.48; decimalLatitude: 43.17227; decimalLongitude: -4.90857; geodeticDatum: WGS84; **Event:** eventID: 1; samplingProtocol: Aerial; eventTime: Night
- **Type status:** Other material. **Occurrence:** individualCount: 3; sex: female; **Location:** locationID: P4; continent: Europe; country: Spain; countryCode: ES; stateProvince: Castilla y León; county: León; locality: El Canto; verbatimElevation: 943.48; decimalLatitude: 43.17227; decimalLongitude: -4.90857; geodeticDatum: WGS84; **Event:** eventID: 1; samplingProtocol: Aerial; eventTime: Night
- **Type status:** Other material. **Occurrence:** individualCount: 1; sex: male; **Location:** locationID: P4; continent: Europe; country: Spain; countryCode: ES; stateProvince: Castilla y León; county: León; locality: El Canto; verbatimElevation: 943.48; decimalLatitude: 43.17227; decimalLongitude: -4.90857; geodeticDatum: WGS84; **Event:** eventID: 3; samplingProtocol: Aerial; eventTime: Night
- **Type status:** Other material. **Occurrence:** individualCount: 3; sex: female; **Location:** locationID: P4; continent: Europe; country: Spain; countryCode: ES; stateProvince: Castilla y León; county: León; locality: El Canto; verbatimElevation: 943.48; decimalLatitude: 43.17227; decimalLongitude: -4.90857; geodeticDatum: WGS84; **Event:** eventID: 3; samplingProtocol: Aerial; eventTime: Night
- **Type status:** Other material. **Occurrence:** individualCount: 1; sex: female; **Location:** locationID: P4; continent: Europe; country: Spain; countryCode: ES; stateProvince: Castilla y León; county: León; locality: El Canto; verbatimElevation: 943.48; decimalLatitude: 43.17227; decimalLongitude: -4.90857; geodeticDatum: WGS84; **Event:** eventID: 4; samplingProtocol: Aerial; eventTime: Night
- **Type status:** Other material. **Occurrence:** individualCount: 6; sex: female; **Location:** locationID: P4; continent: Europe; country: Spain; countryCode: ES; stateProvince: Castilla y León; county: León; locality: El Canto; verbatimElevation: 943.48; decimalLatitude: 43.17227; decimalLongitude: -4.90857; geodeticDatum: WGS84; **Event:** eventID: 1; samplingProtocol: Beating; eventTime: Day
- **Type status:** Other material. **Occurrence:** individualCount: 1; sex: female; **Location:** locationID: P4; continent: Europe; country: Spain; countryCode: ES; stateProvince: Castilla y León; county: León; locality: El Canto; verbatimElevation: 943.48; decimalLatitude: 43.17227; decimalLongitude: -4.90857; geodeticDatum: WGS84; **Event:** eventID: 1; samplingProtocol: Beating; eventTime: Night

##### Distribution

Europe, Turkey, China

#### Theridion pinastri

L. Koch, 1872

##### Materials

- **Type status:** Other material. **Occurrence:** individualCount: 1; sex: male; **Location:** locationID: O2; continent: Europe; country: Spain; countryCode: ES; stateProvince: Aragón; county: Huesca; locality: Rebilla; verbatimElevation: 1158.13; decimalLatitude: 42.59427; decimalLongitude: 0.1529; geodeticDatum: WGS84; **Event:** eventID: 1; samplingProtocol: Aerial; eventTime: Night
- **Type status:** Other material. **Occurrence:** individualCount: 1; sex: male; **Location:** locationID: O2; continent: Europe; country: Spain; countryCode: ES; stateProvince: Aragón; county: Huesca; locality: Rebilla; verbatimElevation: 1158.13; decimalLatitude: 42.59427; decimalLongitude: 0.1529; geodeticDatum: WGS84; **Event:** eventID: 2; samplingProtocol: Aerial; eventTime: Night

##### Distribution

Palearctic

#### Theridion varians

Hahn, 1833

##### Materials

- **Type status:** Other material. **Occurrence:** individualCount: 1; sex: female; **Location:** locationID: A1; continent: Europe; country: Spain; countryCode: ES; stateProvince: Catalonia; county: Lleida; locality: Sola de Boi; verbatimElevation: 1759.8; decimalLatitude: 42.54958; decimalLongitude: 0.87254; geodeticDatum: WGS84; **Event:** eventID: 2; samplingProtocol: Sweeping; eventTime: Day
- **Type status:** Other material. **Occurrence:** individualCount: 2; sex: female; **Location:** locationID: A2; continent: Europe; country: Spain; countryCode: ES; stateProvince: Catalonia; county: Lleida; locality: Sola de Boi; verbatimElevation: 1738.7; decimalLatitude: 42.54913; decimalLongitude: 0.87137; geodeticDatum: WGS84; **Event:** eventID: 1; samplingProtocol: Beating; eventTime: Day
- **Type status:** Other material. **Occurrence:** individualCount: 1; sex: male; **Location:** locationID: A2; continent: Europe; country: Spain; countryCode: ES; stateProvince: Catalonia; county: Lleida; locality: Sola de Boi; verbatimElevation: 1738.7; decimalLatitude: 42.54913; decimalLongitude: 0.87137; geodeticDatum: WGS84; **Event:** eventID: 1; samplingProtocol: Beating; eventTime: Day
- **Type status:** Other material. **Occurrence:** individualCount: 2; sex: female; **Location:** locationID: A2; continent: Europe; country: Spain; countryCode: ES; stateProvince: Catalonia; county: Lleida; locality: Sola de Boi; verbatimElevation: 1738.7; decimalLatitude: 42.54913; decimalLongitude: 0.87137; geodeticDatum: WGS84; **Event:** eventID: 2; samplingProtocol: Beating; eventTime: Day
- **Type status:** Other material. **Occurrence:** individualCount: 1; sex: male; **Location:** locationID: A2; continent: Europe; country: Spain; countryCode: ES; stateProvince: Catalonia; county: Lleida; locality: Sola de Boi; verbatimElevation: 1738.7; decimalLatitude: 42.54913; decimalLongitude: 0.87137; geodeticDatum: WGS84; **Event:** eventID: 2; samplingProtocol: Beating; eventTime: Day
- **Type status:** Other material. **Occurrence:** individualCount: 1; sex: male; **Location:** locationID: A2; continent: Europe; country: Spain; countryCode: ES; stateProvince: Catalonia; county: Lleida; locality: Sola de Boi; verbatimElevation: 1738.7; decimalLatitude: 42.54913; decimalLongitude: 0.87137; geodeticDatum: WGS84; **Event:** eventID: 1; samplingProtocol: Sweeping; eventTime: Night
- **Type status:** Other material. **Occurrence:** individualCount: 1; sex: female; **Location:** locationID: A2; continent: Europe; country: Spain; countryCode: ES; stateProvince: Catalonia; county: Lleida; locality: Sola de Boi; verbatimElevation: 1738.7; decimalLatitude: 42.54913; decimalLongitude: 0.87137; geodeticDatum: WGS84; **Event:** eventID: 2; samplingProtocol: Sweeping; eventTime: Day
- **Type status:** Other material. **Occurrence:** individualCount: 1; sex: male; **Location:** locationID: O1; continent: Europe; country: Spain; countryCode: ES; stateProvince: Aragón; county: Huesca; locality: O Furno; verbatimElevation: 1396.73; decimalLatitude: 42.60677; decimalLongitude: 0.13135; geodeticDatum: WGS84; **Event:** eventID: 1; samplingProtocol: Aerial; eventTime: Night
- **Type status:** Other material. **Occurrence:** individualCount: 1; sex: male; **Location:** locationID: O1; continent: Europe; country: Spain; countryCode: ES; stateProvince: Aragón; county: Huesca; locality: O Furno; verbatimElevation: 1396.73; decimalLatitude: 42.60677; decimalLongitude: 0.13135; geodeticDatum: WGS84; **Event:** eventID: 1; samplingProtocol: Beating; eventTime: Day
- **Type status:** Other material. **Occurrence:** individualCount: 1; sex: male; **Location:** locationID: O2; continent: Europe; country: Spain; countryCode: ES; stateProvince: Aragón; county: Huesca; locality: Rebilla; verbatimElevation: 1158.13; decimalLatitude: 42.59427; decimalLongitude: 0.1529; geodeticDatum: WGS84; **Event:** eventID: 1; samplingProtocol: Aerial; eventTime: Night
- **Type status:** Other material. **Occurrence:** individualCount: 1; sex: female; **Location:** locationID: P1; continent: Europe; country: Spain; countryCode: ES; stateProvince: Castilla y León; county: León; locality: Monte Robledo; verbatimElevation: 1071.58; decimalLatitude: 43.1445; decimalLongitude: -4.92675; geodeticDatum: WGS84; **Event:** eventID: 1; samplingProtocol: Beating; eventTime: Night
- **Type status:** Other material. **Occurrence:** individualCount: 1; sex: female; **Location:** locationID: P2; continent: Europe; country: Spain; countryCode: ES; stateProvince: Castilla y León; county: León; locality: Joyoguelas; verbatimElevation: 763.98; decimalLatitude: 43.17771; decimalLongitude: -4.90579; geodeticDatum: WGS84; **Event:** eventID: 1; samplingProtocol: Aerial; eventTime: Night
- **Type status:** Other material. **Occurrence:** individualCount: 2; sex: male; **Location:** locationID: P2; continent: Europe; country: Spain; countryCode: ES; stateProvince: Castilla y León; county: León; locality: Joyoguelas; verbatimElevation: 763.98; decimalLatitude: 43.17771; decimalLongitude: -4.90579; geodeticDatum: WGS84; **Event:** eventID: 1; samplingProtocol: Aerial; eventTime: Night
- **Type status:** Other material. **Occurrence:** individualCount: 1; sex: male; **Location:** locationID: P2; continent: Europe; country: Spain; countryCode: ES; stateProvince: Castilla y León; county: León; locality: Joyoguelas; verbatimElevation: 763.98; decimalLatitude: 43.17771; decimalLongitude: -4.90579; geodeticDatum: WGS84; **Event:** eventID: 1; samplingProtocol: Aerial; eventTime: Night
- **Type status:** Other material. **Occurrence:** individualCount: 1; sex: male; **Location:** locationID: P2; continent: Europe; country: Spain; countryCode: ES; stateProvince: Castilla y León; county: León; locality: Joyoguelas; verbatimElevation: 763.98; decimalLatitude: 43.17771; decimalLongitude: -4.90579; geodeticDatum: WGS84; **Event:** eventID: 2; samplingProtocol: Aerial; eventTime: Night
- **Type status:** Other material. **Occurrence:** individualCount: 1; sex: male; **Location:** locationID: P2; continent: Europe; country: Spain; countryCode: ES; stateProvince: Castilla y León; county: León; locality: Joyoguelas; verbatimElevation: 763.98; decimalLatitude: 43.17771; decimalLongitude: -4.90579; geodeticDatum: WGS84; **Event:** eventID: 1; samplingProtocol: Beating; eventTime: Day
- **Type status:** Other material. **Occurrence:** individualCount: 1; sex: male; **Location:** locationID: P2; continent: Europe; country: Spain; countryCode: ES; stateProvince: Castilla y León; county: León; locality: Joyoguelas; verbatimElevation: 763.98; decimalLatitude: 43.17771; decimalLongitude: -4.90579; geodeticDatum: WGS84; **Event:** eventID: 1; samplingProtocol: Sweeping; eventTime: Night
- **Type status:** Other material. **Occurrence:** individualCount: 1; sex: male; **Location:** locationID: P2; continent: Europe; country: Spain; countryCode: ES; stateProvince: Castilla y León; county: León; locality: Joyoguelas; verbatimElevation: 763.98; decimalLatitude: 43.17771; decimalLongitude: -4.90579; geodeticDatum: WGS84; **Event:** eventID: 1; samplingProtocol: Sweeping; eventTime: Night
- **Type status:** Other material. **Occurrence:** individualCount: 1; sex: female; **Location:** locationID: P4; continent: Europe; country: Spain; countryCode: ES; stateProvince: Castilla y León; county: León; locality: El Canto; verbatimElevation: 943.48; decimalLatitude: 43.17227; decimalLongitude: -4.90857; geodeticDatum: WGS84; **Event:** eventID: 1; samplingProtocol: Beating; eventTime: Day
- **Type status:** Other material. **Occurrence:** individualCount: 1; sex: male; **Location:** locationID: P4; continent: Europe; country: Spain; countryCode: ES; stateProvince: Castilla y León; county: León; locality: El Canto; verbatimElevation: 943.48; decimalLatitude: 43.17227; decimalLongitude: -4.90857; geodeticDatum: WGS84; **Event:** eventID: 1; samplingProtocol: Beating; eventTime: Day
- **Type status:** Other material. **Occurrence:** individualCount: 1; sex: female; **Location:** locationID: P4; continent: Europe; country: Spain; countryCode: ES; stateProvince: Castilla y León; county: León; locality: El Canto; verbatimElevation: 943.48; decimalLatitude: 43.17227; decimalLongitude: -4.90857; geodeticDatum: WGS84; **Event:** eventID: 2; samplingProtocol: Beating; eventTime: Day

##### Distribution

Holarctic

#### Theridion sp06

##### Materials

- **Type status:** Other material. **Occurrence:** individualCount: 1; sex: male; **Location:** locationID: A1; continent: Europe; country: Spain; countryCode: ES; stateProvince: Catalonia; county: Lleida; locality: Sola de Boi; verbatimElevation: 1759.8; decimalLatitude: 42.54958; decimalLongitude: 0.87254; geodeticDatum: WGS84; **Event:** eventID: 1; samplingProtocol: Aerial; eventTime: Night
- **Type status:** Other material. **Occurrence:** individualCount: 1; sex: female; **Location:** locationID: A1; continent: Europe; country: Spain; countryCode: ES; stateProvince: Catalonia; county: Lleida; locality: Sola de Boi; verbatimElevation: 1759.8; decimalLatitude: 42.54958; decimalLongitude: 0.87254; geodeticDatum: WGS84; **Event:** eventID: 1; samplingProtocol: Aerial; eventTime: Night
- **Type status:** Other material. **Occurrence:** individualCount: 1; sex: male; **Location:** locationID: A1; continent: Europe; country: Spain; countryCode: ES; stateProvince: Catalonia; county: Lleida; locality: Sola de Boi; verbatimElevation: 1759.8; decimalLatitude: 42.54958; decimalLongitude: 0.87254; geodeticDatum: WGS84; **Event:** eventID: 1; samplingProtocol: Aerial; eventTime: Night
- **Type status:** Other material. **Occurrence:** individualCount: 1; sex: female; **Location:** locationID: A1; continent: Europe; country: Spain; countryCode: ES; stateProvince: Catalonia; county: Lleida; locality: Sola de Boi; verbatimElevation: 1759.8; decimalLatitude: 42.54958; decimalLongitude: 0.87254; geodeticDatum: WGS84; **Event:** eventID: 2; samplingProtocol: Aerial; eventTime: Night
- **Type status:** Other material. **Occurrence:** individualCount: 1; sex: male; **Location:** locationID: A1; continent: Europe; country: Spain; countryCode: ES; stateProvince: Catalonia; county: Lleida; locality: Sola de Boi; verbatimElevation: 1759.8; decimalLatitude: 42.54958; decimalLongitude: 0.87254; geodeticDatum: WGS84; **Event:** eventID: 2; samplingProtocol: Aerial; eventTime: Night
- **Type status:** Other material. **Occurrence:** individualCount: 1; sex: female; **Location:** locationID: A1; continent: Europe; country: Spain; countryCode: ES; stateProvince: Catalonia; county: Lleida; locality: Sola de Boi; verbatimElevation: 1759.8; decimalLatitude: 42.54958; decimalLongitude: 0.87254; geodeticDatum: WGS84; **Event:** eventID: 2; samplingProtocol: Aerial; eventTime: Night
- **Type status:** Other material. **Occurrence:** individualCount: 1; sex: male; **Location:** locationID: A1; continent: Europe; country: Spain; countryCode: ES; stateProvince: Catalonia; county: Lleida; locality: Sola de Boi; verbatimElevation: 1759.8; decimalLatitude: 42.54958; decimalLongitude: 0.87254; geodeticDatum: WGS84; **Event:** eventID: 2; samplingProtocol: Aerial; eventTime: Night
- **Type status:** Other material. **Occurrence:** individualCount: 1; sex: female; **Location:** locationID: A1; continent: Europe; country: Spain; countryCode: ES; stateProvince: Catalonia; county: Lleida; locality: Sola de Boi; verbatimElevation: 1759.8; decimalLatitude: 42.54958; decimalLongitude: 0.87254; geodeticDatum: WGS84; **Event:** eventID: 2; samplingProtocol: Beating; eventTime: Day
- **Type status:** Other material. **Occurrence:** individualCount: 1; sex: male; **Location:** locationID: A1; continent: Europe; country: Spain; countryCode: ES; stateProvince: Catalonia; county: Lleida; locality: Sola de Boi; verbatimElevation: 1759.8; decimalLatitude: 42.54958; decimalLongitude: 0.87254; geodeticDatum: WGS84; **Event:** eventID: 2; samplingProtocol: Beating; eventTime: Day
- **Type status:** Other material. **Occurrence:** individualCount: 1; sex: female; **Location:** locationID: A1; continent: Europe; country: Spain; countryCode: ES; stateProvince: Catalonia; county: Lleida; locality: Sola de Boi; verbatimElevation: 1759.8; decimalLatitude: 42.54958; decimalLongitude: 0.87254; geodeticDatum: WGS84; **Event:** eventID: 1; samplingProtocol: Sweeping; eventTime: Night
- **Type status:** Other material. **Occurrence:** individualCount: 1; sex: male; **Location:** locationID: A1; continent: Europe; country: Spain; countryCode: ES; stateProvince: Catalonia; county: Lleida; locality: Sola de Boi; verbatimElevation: 1759.8; decimalLatitude: 42.54958; decimalLongitude: 0.87254; geodeticDatum: WGS84; **Event:** eventID: 1; samplingProtocol: Sweeping; eventTime: Night
- **Type status:** Other material. **Occurrence:** individualCount: 1; sex: male; **Location:** locationID: A2; continent: Europe; country: Spain; countryCode: ES; stateProvince: Catalonia; county: Lleida; locality: Sola de Boi; verbatimElevation: 1738.7; decimalLatitude: 42.54913; decimalLongitude: 0.87137; geodeticDatum: WGS84; **Event:** eventID: 1; samplingProtocol: Beating; eventTime: Day

##### Distribution

?

##### Notes

This species is part of the *melanurum*-group, which would also include *T. harmsi* and *T. mystaceum*, but differs from both these species and anything else we could find in the literature. DNA barcodes (results not shown) suggested a potential case of hybridisation amongst the three *melanurum* species collected in this project. A specific study on species boundaries and the possible introgressive hybridisation amongst species in the *melanurum*-group is underway.

#### Theridion sp15

##### Materials

- **Type status:** Other material. **Occurrence:** individualCount: 1; sex: male; **Location:** locationID: O2; continent: Europe; country: Spain; countryCode: ES; stateProvince: Aragón; county: Huesca; locality: Rebilla; verbatimElevation: 1158.13; decimalLatitude: 42.59427; decimalLongitude: 0.1529; geodeticDatum: WGS84; **Event:** eventID: 2; samplingProtocol: Aerial; eventTime: Night

##### Distribution

?

##### Notes

This specimen is very close to the species *T. cinereum* Thorell, 1875, *T. petraeum* L. Koch, 1872, *T. furfuraceum* Simon, 1914, *T. pyrenaeum* Denis, 1944 and *T. wiehlei* Schenkel, 1938. Due to the small genitalic differences in all males of these species, we could not make a differential diagnosis on our single male specimen. Identification based on DNA does not pair this specimen with central European *T. petraeum* populations, which is also supported by morphological data, such as a different shape in the base of the embolus and a longer median apophysis.

#### Thomisidae

Sundevall, 1833

#### Cozyptila blackwalli

(Simon, 1875)

##### Materials

- **Type status:** Other material. **Occurrence:** individualCount: 1; sex: female; **Location:** locationID: O1; continent: Europe; country: Spain; countryCode: ES; stateProvince: Aragón; county: Huesca; locality: O Furno; verbatimElevation: 1396.73; decimalLatitude: 42.60677; decimalLongitude: 0.13135; geodeticDatum: WGS84; **Event:** eventID: A; samplingProtocol: Pitfall
- **Type status:** Other material. **Occurrence:** individualCount: 1; sex: male; **Location:** locationID: O1; continent: Europe; country: Spain; countryCode: ES; stateProvince: Aragón; county: Huesca; locality: O Furno; verbatimElevation: 1396.73; decimalLatitude: 42.60677; decimalLongitude: 0.13135; geodeticDatum: WGS84; **Event:** eventID: A; samplingProtocol: Pitfall
- **Type status:** Other material. **Occurrence:** individualCount: 1; sex: female; **Location:** locationID: P2; continent: Europe; country: Spain; countryCode: ES; stateProvince: Castilla y León; county: León; locality: Joyoguelas; verbatimElevation: 763.98; decimalLatitude: 43.17771; decimalLongitude: -4.90579; geodeticDatum: WGS84; **Event:** eventID: A; samplingProtocol: Pitfall
- **Type status:** Other material. **Occurrence:** individualCount: 1; sex: male; **Location:** locationID: P2; continent: Europe; country: Spain; countryCode: ES; stateProvince: Castilla y León; county: León; locality: Joyoguelas; verbatimElevation: 763.98; decimalLatitude: 43.17771; decimalLongitude: -4.90579; geodeticDatum: WGS84; **Event:** eventID: I; samplingProtocol: Pitfall
- **Type status:** Other material. **Occurrence:** individualCount: 1; sex: female; **Location:** locationID: P2; continent: Europe; country: Spain; countryCode: ES; stateProvince: Castilla y León; county: León; locality: Joyoguelas; verbatimElevation: 763.98; decimalLatitude: 43.17771; decimalLongitude: -4.90579; geodeticDatum: WGS84; **Event:** eventID: J; samplingProtocol: Pitfall
- **Type status:** Other material. **Occurrence:** individualCount: 1; sex: male; **Location:** locationID: P4; continent: Europe; country: Spain; countryCode: ES; stateProvince: Castilla y León; county: León; locality: El Canto; verbatimElevation: 943.48; decimalLatitude: 43.17227; decimalLongitude: -4.90857; geodeticDatum: WGS84; **Event:** eventID: H; samplingProtocol: Pitfall
- **Type status:** Other material. **Occurrence:** individualCount: 1; sex: male; **Location:** locationID: P4; continent: Europe; country: Spain; countryCode: ES; stateProvince: Castilla y León; county: León; locality: El Canto; verbatimElevation: 943.48; decimalLatitude: 43.17227; decimalLongitude: -4.90857; geodeticDatum: WGS84; **Event:** eventID: I; samplingProtocol: Pitfall
- **Type status:** Other material. **Occurrence:** individualCount: 1; sex: male; **Location:** locationID: P4; continent: Europe; country: Spain; countryCode: ES; stateProvince: Castilla y León; county: León; locality: El Canto; verbatimElevation: 943.48; decimalLatitude: 43.17227; decimalLongitude: -4.90857; geodeticDatum: WGS84; **Event:** eventID: L; samplingProtocol: Pitfall

##### Distribution

Europe

#### Diaea dorsata

(Fabricius, 1777)

##### Materials

- **Type status:** Other material. **Occurrence:** individualCount: 3; sex: male; **Location:** locationID: A1; continent: Europe; country: Spain; countryCode: ES; stateProvince: Catalonia; county: Lleida; locality: Sola de Boi; verbatimElevation: 1759.8; decimalLatitude: 42.54958; decimalLongitude: 0.87254; geodeticDatum: WGS84; **Event:** eventID: 1; samplingProtocol: Beating; eventTime: Day
- **Type status:** Other material. **Occurrence:** individualCount: 1; sex: male; **Location:** locationID: A2; continent: Europe; country: Spain; countryCode: ES; stateProvince: Catalonia; county: Lleida; locality: Sola de Boi; verbatimElevation: 1738.7; decimalLatitude: 42.54913; decimalLongitude: 0.87137; geodeticDatum: WGS84; **Event:** eventID: 1; samplingProtocol: Aerial; eventTime: Night
- **Type status:** Other material. **Occurrence:** individualCount: 1; sex: female; **Location:** locationID: A2; continent: Europe; country: Spain; countryCode: ES; stateProvince: Catalonia; county: Lleida; locality: Sola de Boi; verbatimElevation: 1738.7; decimalLatitude: 42.54913; decimalLongitude: 0.87137; geodeticDatum: WGS84; **Event:** eventID: 1; samplingProtocol: Beating; eventTime: Day
- **Type status:** Other material. **Occurrence:** individualCount: 1; sex: male; **Location:** locationID: A2; continent: Europe; country: Spain; countryCode: ES; stateProvince: Catalonia; county: Lleida; locality: Sola de Boi; verbatimElevation: 1738.7; decimalLatitude: 42.54913; decimalLongitude: 0.87137; geodeticDatum: WGS84; **Event:** eventID: 1; samplingProtocol: Beating; eventTime: Day
- **Type status:** Other material. **Occurrence:** individualCount: 2; sex: female; **Location:** locationID: A2; continent: Europe; country: Spain; countryCode: ES; stateProvince: Catalonia; county: Lleida; locality: Sola de Boi; verbatimElevation: 1738.7; decimalLatitude: 42.54913; decimalLongitude: 0.87137; geodeticDatum: WGS84; **Event:** eventID: 2; samplingProtocol: Beating; eventTime: Day
- **Type status:** Other material. **Occurrence:** individualCount: 1; sex: female; **Location:** locationID: A2; continent: Europe; country: Spain; countryCode: ES; stateProvince: Catalonia; county: Lleida; locality: Sola de Boi; verbatimElevation: 1738.7; decimalLatitude: 42.54913; decimalLongitude: 0.87137; geodeticDatum: WGS84; **Event:** eventID: 1; samplingProtocol: Beating; eventTime: Night
- **Type status:** Other material. **Occurrence:** individualCount: 1; sex: male; **Location:** locationID: A2; continent: Europe; country: Spain; countryCode: ES; stateProvince: Catalonia; county: Lleida; locality: Sola de Boi; verbatimElevation: 1738.7; decimalLatitude: 42.54913; decimalLongitude: 0.87137; geodeticDatum: WGS84; **Event:** eventID: 1; samplingProtocol: Sweeping; eventTime: Night
- **Type status:** Other material. **Occurrence:** individualCount: 1; sex: female; **Location:** locationID: A2; continent: Europe; country: Spain; countryCode: ES; stateProvince: Catalonia; county: Lleida; locality: Sola de Boi; verbatimElevation: 1738.7; decimalLatitude: 42.54913; decimalLongitude: 0.87137; geodeticDatum: WGS84; **Event:** eventID: 2; samplingProtocol: Sweeping; eventTime: Day
- **Type status:** Other material. **Occurrence:** individualCount: 1; sex: female; **Location:** locationID: O1; continent: Europe; country: Spain; countryCode: ES; stateProvince: Aragón; county: Huesca; locality: O Furno; verbatimElevation: 1396.73; decimalLatitude: 42.60677; decimalLongitude: 0.13135; geodeticDatum: WGS84; **Event:** eventID: 1; samplingProtocol: Aerial; eventTime: Night
- **Type status:** Other material. **Occurrence:** individualCount: 1; sex: female; **Location:** locationID: O1; continent: Europe; country: Spain; countryCode: ES; stateProvince: Aragón; county: Huesca; locality: O Furno; verbatimElevation: 1396.73; decimalLatitude: 42.60677; decimalLongitude: 0.13135; geodeticDatum: WGS84; **Event:** eventID: 1; samplingProtocol: Beating; eventTime: Day
- **Type status:** Other material. **Occurrence:** individualCount: 1; sex: male; **Location:** locationID: O1; continent: Europe; country: Spain; countryCode: ES; stateProvince: Aragón; county: Huesca; locality: O Furno; verbatimElevation: 1396.73; decimalLatitude: 42.60677; decimalLongitude: 0.13135; geodeticDatum: WGS84; **Event:** eventID: 1; samplingProtocol: Beating; eventTime: Day
- **Type status:** Other material. **Occurrence:** individualCount: 4; sex: female; **Location:** locationID: O1; continent: Europe; country: Spain; countryCode: ES; stateProvince: Aragón; county: Huesca; locality: O Furno; verbatimElevation: 1396.73; decimalLatitude: 42.60677; decimalLongitude: 0.13135; geodeticDatum: WGS84; **Event:** eventID: 1; samplingProtocol: Beating; eventTime: Night
- **Type status:** Other material. **Occurrence:** individualCount: 2; sex: male; **Location:** locationID: O1; continent: Europe; country: Spain; countryCode: ES; stateProvince: Aragón; county: Huesca; locality: O Furno; verbatimElevation: 1396.73; decimalLatitude: 42.60677; decimalLongitude: 0.13135; geodeticDatum: WGS84; **Event:** eventID: 1; samplingProtocol: Beating; eventTime: Night
- **Type status:** Other material. **Occurrence:** individualCount: 1; sex: female; **Location:** locationID: O1; continent: Europe; country: Spain; countryCode: ES; stateProvince: Aragón; county: Huesca; locality: O Furno; verbatimElevation: 1396.73; decimalLatitude: 42.60677; decimalLongitude: 0.13135; geodeticDatum: WGS84; **Event:** eventID: 1; samplingProtocol: Beating; eventTime: Night
- **Type status:** Other material. **Occurrence:** individualCount: 1; sex: male; **Location:** locationID: O1; continent: Europe; country: Spain; countryCode: ES; stateProvince: Aragón; county: Huesca; locality: O Furno; verbatimElevation: 1396.73; decimalLatitude: 42.60677; decimalLongitude: 0.13135; geodeticDatum: WGS84; **Event:** eventID: 1; samplingProtocol: Beating; eventTime: Night
- **Type status:** Other material. **Occurrence:** individualCount: 1; sex: female; **Location:** locationID: O1; continent: Europe; country: Spain; countryCode: ES; stateProvince: Aragón; county: Huesca; locality: O Furno; verbatimElevation: 1396.73; decimalLatitude: 42.60677; decimalLongitude: 0.13135; geodeticDatum: WGS84; **Event:** eventID: 1; samplingProtocol: Sweeping; eventTime: Day
- **Type status:** Other material. **Occurrence:** individualCount: 1; sex: male; **Location:** locationID: O1; continent: Europe; country: Spain; countryCode: ES; stateProvince: Aragón; county: Huesca; locality: O Furno; verbatimElevation: 1396.73; decimalLatitude: 42.60677; decimalLongitude: 0.13135; geodeticDatum: WGS84; **Event:** eventID: 1; samplingProtocol: Sweeping; eventTime: Night
- **Type status:** Other material. **Occurrence:** individualCount: 1; sex: female; **Location:** locationID: P2; continent: Europe; country: Spain; countryCode: ES; stateProvince: Castilla y León; county: León; locality: Joyoguelas; verbatimElevation: 763.98; decimalLatitude: 43.17771; decimalLongitude: -4.90579; geodeticDatum: WGS84; **Event:** eventID: 1; samplingProtocol: Beating; eventTime: Day
- **Type status:** Other material. **Occurrence:** individualCount: 1; sex: male; **Location:** locationID: P3; continent: Europe; country: Spain; countryCode: ES; stateProvince: Castilla y León; county: León; locality: Las Arroyas; verbatimElevation: 1097.1; decimalLatitude: 43.14351; decimalLongitude: -4.94878; geodeticDatum: WGS84; **Event:** eventID: 1; samplingProtocol: Beating; eventTime: Day
- **Type status:** Other material. **Occurrence:** individualCount: 1; sex: female; **Location:** locationID: P3; continent: Europe; country: Spain; countryCode: ES; stateProvince: Castilla y León; county: León; locality: Las Arroyas; verbatimElevation: 1097.1; decimalLatitude: 43.14351; decimalLongitude: -4.94878; geodeticDatum: WGS84; **Event:** eventID: 1; samplingProtocol: Sweeping; eventTime: Day
- **Type status:** Other material. **Occurrence:** individualCount: 1; sex: female; **Location:** locationID: P4; continent: Europe; country: Spain; countryCode: ES; stateProvince: Castilla y León; county: León; locality: El Canto; verbatimElevation: 943.48; decimalLatitude: 43.17227; decimalLongitude: -4.90857; geodeticDatum: WGS84; **Event:** eventID: 1; samplingProtocol: Beating; eventTime: Night

##### Distribution

Palearctic

#### Heriaeus oblongus

Simon, 1918

##### Materials

- **Type status:** Other material. **Occurrence:** individualCount: 1; sex: female; **Location:** locationID: S1; continent: Europe; country: Spain; countryCode: ES; stateProvince: Andalucía; county: Granada; locality: Soportujar; verbatimElevation: 1786.57; decimalLatitude: 36.96151; decimalLongitude: -3.41881; geodeticDatum: WGS84; **Event:** eventID: 1; samplingProtocol: Aerial; eventTime: Night
- **Type status:** Other material. **Occurrence:** individualCount: 1; sex: female; **Location:** locationID: S1; continent: Europe; country: Spain; countryCode: ES; stateProvince: Andalucía; county: Granada; locality: Soportujar; verbatimElevation: 1786.57; decimalLatitude: 36.96151; decimalLongitude: -3.41881; geodeticDatum: WGS84; **Event:** eventID: 2; samplingProtocol: Aerial; eventTime: Night
- **Type status:** Other material. **Occurrence:** individualCount: 1; sex: male; **Location:** locationID: S1; continent: Europe; country: Spain; countryCode: ES; stateProvince: Andalucía; county: Granada; locality: Soportujar; verbatimElevation: 1786.57; decimalLatitude: 36.96151; decimalLongitude: -3.41881; geodeticDatum: WGS84; **Event:** eventID: 2; samplingProtocol: Sweeping; eventTime: Day

##### Distribution

Palearctic

#### Misumena vatia

(Clerck, 1757)

##### Materials

- **Type status:** Other material. **Occurrence:** individualCount: 1; sex: male; **Location:** locationID: C1; continent: Europe; country: Spain; countryCode: ES; stateProvince: Castilla-La Mancha; county: Ciudad Real; locality: Valle Brezoso; verbatimElevation: 756.56; decimalLatitude: 39.35663; decimalLongitude: -4.35912; geodeticDatum: WGS84; **Event:** eventID: 1; samplingProtocol: Beating; eventTime: Day
- **Type status:** Other material. **Occurrence:** individualCount: 1; sex: male; **Location:** locationID: C4; continent: Europe; country: Spain; countryCode: ES; stateProvince: Castilla-La Mancha; county: Ciudad Real; locality: La Quesera; verbatimElevation: 772.3; decimalLatitude: 39.36337; decimalLongitude: -4.41704; geodeticDatum: WGS84; **Event:** eventID: 1; samplingProtocol: Aerial; eventTime: Night
- **Type status:** Other material. **Occurrence:** individualCount: 2; sex: male; **Location:** locationID: C4; continent: Europe; country: Spain; countryCode: ES; stateProvince: Castilla-La Mancha; county: Ciudad Real; locality: La Quesera; verbatimElevation: 772.3; decimalLatitude: 39.36337; decimalLongitude: -4.41704; geodeticDatum: WGS84; **Event:** eventID: 1; samplingProtocol: Beating; eventTime: Night
- **Type status:** Other material. **Occurrence:** individualCount: 1; sex: male; **Location:** locationID: C4; continent: Europe; country: Spain; countryCode: ES; stateProvince: Castilla-La Mancha; county: Ciudad Real; locality: La Quesera; verbatimElevation: 772.3; decimalLatitude: 39.36337; decimalLongitude: -4.41704; geodeticDatum: WGS84; **Event:** eventID: 2; samplingProtocol: Sweeping; eventTime: Day
- **Type status:** Other material. **Occurrence:** individualCount: 1; sex: male; **Location:** locationID: M1; continent: Europe; country: Spain; countryCode: ES; stateProvince: Extremadura; county: Cáceres; locality: Peña Falcón; verbatimElevation: 320.6; decimalLatitude: 39.83296; decimalLongitude: -6.0641; geodeticDatum: WGS84; **Event:** eventID: 2; samplingProtocol: Sweeping; eventTime: Day
- **Type status:** Other material. **Occurrence:** individualCount: 1; sex: male; **Location:** locationID: O2; continent: Europe; country: Spain; countryCode: ES; stateProvince: Aragón; county: Huesca; locality: Rebilla; verbatimElevation: 1158.13; decimalLatitude: 42.59427; decimalLongitude: 0.1529; geodeticDatum: WGS84; **Event:** eventID: 1; samplingProtocol: Beating; eventTime: Night
- **Type status:** Other material. **Occurrence:** individualCount: 1; sex: female; **Location:** locationID: O2; continent: Europe; country: Spain; countryCode: ES; stateProvince: Aragón; county: Huesca; locality: Rebilla; verbatimElevation: 1158.13; decimalLatitude: 42.59427; decimalLongitude: 0.1529; geodeticDatum: WGS84; **Event:** eventID: 2; samplingProtocol: Beating; eventTime: Day
- **Type status:** Other material. **Occurrence:** individualCount: 1; sex: female; **Location:** locationID: P4; continent: Europe; country: Spain; countryCode: ES; stateProvince: Castilla y León; county: León; locality: El Canto; verbatimElevation: 943.48; decimalLatitude: 43.17227; decimalLongitude: -4.90857; geodeticDatum: WGS84; **Event:** eventID: 2; samplingProtocol: Sweeping; eventTime: Day
- **Type status:** Other material. **Occurrence:** individualCount: 1; sex: male; **Location:** locationID: S1; continent: Europe; country: Spain; countryCode: ES; stateProvince: Andalucía; county: Granada; locality: Soportujar; verbatimElevation: 1786.57; decimalLatitude: 36.96151; decimalLongitude: -3.41881; geodeticDatum: WGS84; **Event:** eventID: 1; samplingProtocol: Aerial; eventTime: Night
- **Type status:** Other material. **Occurrence:** individualCount: 1; sex: female; **Location:** locationID: S2; continent: Europe; country: Spain; countryCode: ES; stateProvince: Andalucía; county: Granada; locality: Camarate; verbatimElevation: 1713.96; decimalLatitude: 37.18377; decimalLongitude: -3.26282; geodeticDatum: WGS84; **Event:** eventID: 2; samplingProtocol: Beating; eventTime: Day

##### Distribution

Holarctic

#### Ozyptila atomaria

(Panzer, 1801)

##### Materials

- **Type status:** Other material. **Occurrence:** individualCount: 1; sex: female; **Location:** locationID: A1; continent: Europe; country: Spain; countryCode: ES; stateProvince: Catalonia; county: Lleida; locality: Sola de Boi; verbatimElevation: 1759.8; decimalLatitude: 42.54958; decimalLongitude: 0.87254; geodeticDatum: WGS84; **Event:** eventID: B; samplingProtocol: Pitfall

##### Distribution

Palearctic

#### Pistius truncatus

(Pallas, 1772)

##### Materials

- **Type status:** Other material. **Occurrence:** individualCount: 1; sex: male; **Location:** locationID: O2; continent: Europe; country: Spain; countryCode: ES; stateProvince: Aragón; county: Huesca; locality: Rebilla; verbatimElevation: 1158.13; decimalLatitude: 42.59427; decimalLongitude: 0.1529; geodeticDatum: WGS84; **Event:** eventID: 2; samplingProtocol: Beating; eventTime: Day
- **Type status:** Other material. **Occurrence:** individualCount: 1; sex: female; **Location:** locationID: P2; continent: Europe; country: Spain; countryCode: ES; stateProvince: Castilla y León; county: León; locality: Joyoguelas; verbatimElevation: 763.98; decimalLatitude: 43.17771; decimalLongitude: -4.90579; geodeticDatum: WGS84; **Event:** eventID: 1; samplingProtocol: Beating; eventTime: Night

##### Distribution

Palearctic

#### Runcinia grammica

(C. L. Koch, 1837)

##### Materials

- **Type status:** Other material. **Occurrence:** individualCount: 1; sex: male; **Location:** locationID: C3; continent: Europe; country: Spain; countryCode: ES; stateProvince: Castilla-La Mancha; county: Ciudad Real; locality: La Quesera; verbatimElevation: 767.55; decimalLatitude: 39.36177; decimalLongitude: -4.41733; geodeticDatum: WGS84; **Event:** eventID: 2; samplingProtocol: Sweeping; eventTime: Day
- **Type status:** Other material. **Occurrence:** individualCount: 1; sex: male; **Location:** locationID: C4; continent: Europe; country: Spain; countryCode: ES; stateProvince: Castilla-La Mancha; county: Ciudad Real; locality: La Quesera; verbatimElevation: 772.3; decimalLatitude: 39.36337; decimalLongitude: -4.41704; geodeticDatum: WGS84; **Event:** eventID: 1; samplingProtocol: Sweeping; eventTime: Day
- **Type status:** Other material. **Occurrence:** individualCount: 2; sex: male; **Location:** locationID: C4; continent: Europe; country: Spain; countryCode: ES; stateProvince: Castilla-La Mancha; county: Ciudad Real; locality: La Quesera; verbatimElevation: 772.3; decimalLatitude: 39.36337; decimalLongitude: -4.41704; geodeticDatum: WGS84; **Event:** eventID: 2; samplingProtocol: Sweeping; eventTime: Day
- **Type status:** Other material. **Occurrence:** individualCount: 1; sex: male; **Location:** locationID: M1; continent: Europe; country: Spain; countryCode: ES; stateProvince: Extremadura; county: Cáceres; locality: Peña Falcón; verbatimElevation: 320.6; decimalLatitude: 39.83296; decimalLongitude: -6.0641; geodeticDatum: WGS84; **Event:** eventID: 2; samplingProtocol: Sweeping; eventTime: Day

##### Distribution

Palearctic, St. Helena, South Africa, Lesotho

#### Synema globosum

(Fabricius, 1775)

##### Materials

- **Type status:** Other material. **Occurrence:** individualCount: 1; sex: female; **Location:** locationID: C3; continent: Europe; country: Spain; countryCode: ES; stateProvince: Castilla-La Mancha; county: Ciudad Real; locality: La Quesera; verbatimElevation: 767.55; decimalLatitude: 39.36177; decimalLongitude: -4.41733; geodeticDatum: WGS84; **Event:** eventID: 2; samplingProtocol: Sweeping; eventTime: Day
- **Type status:** Other material. **Occurrence:** individualCount: 1; sex: female; **Location:** locationID: C4; continent: Europe; country: Spain; countryCode: ES; stateProvince: Castilla-La Mancha; county: Ciudad Real; locality: La Quesera; verbatimElevation: 772.3; decimalLatitude: 39.36337; decimalLongitude: -4.41704; geodeticDatum: WGS84; **Event:** eventID: 1; samplingProtocol: Beating; eventTime: Day
- **Type status:** Other material. **Occurrence:** individualCount: 1; sex: female; **Location:** locationID: C4; continent: Europe; country: Spain; countryCode: ES; stateProvince: Castilla-La Mancha; county: Ciudad Real; locality: La Quesera; verbatimElevation: 772.3; decimalLatitude: 39.36337; decimalLongitude: -4.41704; geodeticDatum: WGS84; **Event:** eventID: 1; samplingProtocol: Sweeping; eventTime: Day
- **Type status:** Other material. **Occurrence:** individualCount: 1; sex: male; **Location:** locationID: C4; continent: Europe; country: Spain; countryCode: ES; stateProvince: Castilla-La Mancha; county: Ciudad Real; locality: La Quesera; verbatimElevation: 772.3; decimalLatitude: 39.36337; decimalLongitude: -4.41704; geodeticDatum: WGS84; **Event:** eventID: 1; samplingProtocol: Sweeping; eventTime: Day
- **Type status:** Other material. **Occurrence:** individualCount: 1; sex: female; **Location:** locationID: C4; continent: Europe; country: Spain; countryCode: ES; stateProvince: Castilla-La Mancha; county: Ciudad Real; locality: La Quesera; verbatimElevation: 772.3; decimalLatitude: 39.36337; decimalLongitude: -4.41704; geodeticDatum: WGS84; **Event:** eventID: 2; samplingProtocol: Sweeping; eventTime: Day
- **Type status:** Other material. **Occurrence:** individualCount: 1; sex: female; **Location:** locationID: C4; continent: Europe; country: Spain; countryCode: ES; stateProvince: Castilla-La Mancha; county: Ciudad Real; locality: La Quesera; verbatimElevation: 772.3; decimalLatitude: 39.36337; decimalLongitude: -4.41704; geodeticDatum: WGS84; **Event:** eventID: 1; samplingProtocol: Sweeping; eventTime: Night
- **Type status:** Other material. **Occurrence:** individualCount: 1; sex: male; **Location:** locationID: O1; continent: Europe; country: Spain; countryCode: ES; stateProvince: Aragón; county: Huesca; locality: O Furno; verbatimElevation: 1396.73; decimalLatitude: 42.60677; decimalLongitude: 0.13135; geodeticDatum: WGS84; **Event:** eventID: 2; samplingProtocol: Sweeping; eventTime: Day
- **Type status:** Other material. **Occurrence:** individualCount: 1; sex: male; **Location:** locationID: O2; continent: Europe; country: Spain; countryCode: ES; stateProvince: Aragón; county: Huesca; locality: Rebilla; verbatimElevation: 1158.13; decimalLatitude: 42.59427; decimalLongitude: 0.1529; geodeticDatum: WGS84; **Event:** eventID: 1; samplingProtocol: Sweeping; eventTime: Night
- **Type status:** Other material. **Occurrence:** individualCount: 1; sex: female; **Location:** locationID: O2; continent: Europe; country: Spain; countryCode: ES; stateProvince: Aragón; county: Huesca; locality: Rebilla; verbatimElevation: 1158.13; decimalLatitude: 42.59427; decimalLongitude: 0.1529; geodeticDatum: WGS84; **Event:** eventID: 2; samplingProtocol: Sweeping; eventTime: Day
- **Type status:** Other material. **Occurrence:** individualCount: 1; sex: male; **Location:** locationID: O2; continent: Europe; country: Spain; countryCode: ES; stateProvince: Aragón; county: Huesca; locality: Rebilla; verbatimElevation: 1158.13; decimalLatitude: 42.59427; decimalLongitude: 0.1529; geodeticDatum: WGS84; **Event:** eventID: 2; samplingProtocol: Sweeping; eventTime: Day

##### Distribution

Palearctic

#### Thomisus onustus

Walckenaer, 1805

##### Materials

- **Type status:** Other material. **Occurrence:** individualCount: 1; sex: male; **Location:** locationID: C4; continent: Europe; country: Spain; countryCode: ES; stateProvince: Castilla-La Mancha; county: Ciudad Real; locality: La Quesera; verbatimElevation: 772.3; decimalLatitude: 39.36337; decimalLongitude: -4.41704; geodeticDatum: WGS84; **Event:** eventID: 1; samplingProtocol: Sweeping; eventTime: Night
- **Type status:** Other material. **Occurrence:** individualCount: 1; sex: male; **Location:** locationID: S2; continent: Europe; country: Spain; countryCode: ES; stateProvince: Andalucía; county: Granada; locality: Camarate; verbatimElevation: 1713.96; decimalLatitude: 37.18377; decimalLongitude: -3.26282; geodeticDatum: WGS84; **Event:** eventID: 1; samplingProtocol: Sweeping; eventTime: Night

##### Distribution

Palearctic

#### Tmarus punctatissimus

(Simon, 1870)

##### Materials

- **Type status:** Other material. **Occurrence:** individualCount: 1; sex: male; **Location:** locationID: O2; continent: Europe; country: Spain; countryCode: ES; stateProvince: Aragón; county: Huesca; locality: Rebilla; verbatimElevation: 1158.13; decimalLatitude: 42.59427; decimalLongitude: 0.1529; geodeticDatum: WGS84; **Event:** eventID: 1; samplingProtocol: Beating; eventTime: Night
- **Type status:** Other material. **Occurrence:** individualCount: 1; sex: male; **Location:** locationID: O2; continent: Europe; country: Spain; countryCode: ES; stateProvince: Aragón; county: Huesca; locality: Rebilla; verbatimElevation: 1158.13; decimalLatitude: 42.59427; decimalLongitude: 0.1529; geodeticDatum: WGS84; **Event:** eventID: 1; samplingProtocol: Sweeping; eventTime: Night
- **Type status:** Other material. **Occurrence:** individualCount: 1; sex: female; **Location:** locationID: O2; continent: Europe; country: Spain; countryCode: ES; stateProvince: Aragón; county: Huesca; locality: Rebilla; verbatimElevation: 1158.13; decimalLatitude: 42.59427; decimalLongitude: 0.1529; geodeticDatum: WGS84; **Event:** eventID: 2; samplingProtocol: Sweeping; eventTime: Day
- **Type status:** Other material. **Occurrence:** individualCount: 2; sex: male; **Location:** locationID: O2; continent: Europe; country: Spain; countryCode: ES; stateProvince: Aragón; county: Huesca; locality: Rebilla; verbatimElevation: 1158.13; decimalLatitude: 42.59427; decimalLongitude: 0.1529; geodeticDatum: WGS84; **Event:** eventID: 2; samplingProtocol: Sweeping; eventTime: Day

##### Distribution

Palearctic

#### Tmarus staintoni

(O. Pickard-Cambridge, 1873)

##### Materials

- **Type status:** Other material. **Occurrence:** individualCount: 1; sex: female; **Location:** locationID: C3; continent: Europe; country: Spain; countryCode: ES; stateProvince: Castilla-La Mancha; county: Ciudad Real; locality: La Quesera; verbatimElevation: 767.55; decimalLatitude: 39.36177; decimalLongitude: -4.41733; geodeticDatum: WGS84; **Event:** eventID: 1; samplingProtocol: Beating; eventTime: Night
- **Type status:** Other material. **Occurrence:** individualCount: 1; sex: male; **Location:** locationID: C3; continent: Europe; country: Spain; countryCode: ES; stateProvince: Castilla-La Mancha; county: Ciudad Real; locality: La Quesera; verbatimElevation: 767.55; decimalLatitude: 39.36177; decimalLongitude: -4.41733; geodeticDatum: WGS84; **Event:** eventID: 1; samplingProtocol: Sweeping; eventTime: Day
- **Type status:** Other material. **Occurrence:** individualCount: 1; sex: female; **Location:** locationID: C3; continent: Europe; country: Spain; countryCode: ES; stateProvince: Castilla-La Mancha; county: Ciudad Real; locality: La Quesera; verbatimElevation: 767.55; decimalLatitude: 39.36177; decimalLongitude: -4.41733; geodeticDatum: WGS84; **Event:** eventID: 2; samplingProtocol: Sweeping; eventTime: Day
- **Type status:** Other material. **Occurrence:** individualCount: 2; sex: male; **Location:** locationID: C3; continent: Europe; country: Spain; countryCode: ES; stateProvince: Castilla-La Mancha; county: Ciudad Real; locality: La Quesera; verbatimElevation: 767.55; decimalLatitude: 39.36177; decimalLongitude: -4.41733; geodeticDatum: WGS84; **Event:** eventID: 2; samplingProtocol: Sweeping; eventTime: Day
- **Type status:** Other material. **Occurrence:** individualCount: 1; sex: male; **Location:** locationID: C3; continent: Europe; country: Spain; countryCode: ES; stateProvince: Castilla-La Mancha; county: Ciudad Real; locality: La Quesera; verbatimElevation: 767.55; decimalLatitude: 39.36177; decimalLongitude: -4.41733; geodeticDatum: WGS84; **Event:** eventID: 1; samplingProtocol: Aerial; eventTime: Night
- **Type status:** Other material. **Occurrence:** individualCount: 2; sex: male; **Location:** locationID: C4; continent: Europe; country: Spain; countryCode: ES; stateProvince: Castilla-La Mancha; county: Ciudad Real; locality: La Quesera; verbatimElevation: 772.3; decimalLatitude: 39.36337; decimalLongitude: -4.41704; geodeticDatum: WGS84; **Event:** eventID: 2; samplingProtocol: Aerial; eventTime: Night
- **Type status:** Other material. **Occurrence:** individualCount: 2; sex: male; **Location:** locationID: C4; continent: Europe; country: Spain; countryCode: ES; stateProvince: Castilla-La Mancha; county: Ciudad Real; locality: La Quesera; verbatimElevation: 772.3; decimalLatitude: 39.36337; decimalLongitude: -4.41704; geodeticDatum: WGS84; **Event:** eventID: 1; samplingProtocol: Sweeping; eventTime: Night
- **Type status:** Other material. **Occurrence:** individualCount: 1; sex: female; **Location:** locationID: M1; continent: Europe; country: Spain; countryCode: ES; stateProvince: Extremadura; county: Cáceres; locality: Peña Falcón; verbatimElevation: 320.6; decimalLatitude: 39.83296; decimalLongitude: -6.0641; geodeticDatum: WGS84; **Event:** eventID: 1; samplingProtocol: Sweeping; eventTime: Day
- **Type status:** Other material. **Occurrence:** individualCount: 1; sex: female; **Location:** locationID: M2; continent: Europe; country: Spain; countryCode: ES; stateProvince: Extremadura; county: Cáceres; locality: Fuente del Frances; verbatimElevation: 320.72; decimalLatitude: 39.828; decimalLongitude: -6.03249; geodeticDatum: WGS84; **Event:** eventID: 1; samplingProtocol: Beating; eventTime: Night
- **Type status:** Other material. **Occurrence:** individualCount: 1; sex: female; **Location:** locationID: M2; continent: Europe; country: Spain; countryCode: ES; stateProvince: Extremadura; county: Cáceres; locality: Fuente del Frances; verbatimElevation: 320.72; decimalLatitude: 39.828; decimalLongitude: -6.03249; geodeticDatum: WGS84; **Event:** eventID: 2; samplingProtocol: Beating; eventTime: Day
- **Type status:** Other material. **Occurrence:** individualCount: 2; sex: female; **Location:** locationID: M2; continent: Europe; country: Spain; countryCode: ES; stateProvince: Extremadura; county: Cáceres; locality: Fuente del Frances; verbatimElevation: 320.72; decimalLatitude: 39.828; decimalLongitude: -6.03249; geodeticDatum: WGS84; **Event:** eventID: 1; samplingProtocol: Sweeping; eventTime: Day
- **Type status:** Other material. **Occurrence:** individualCount: 1; sex: male; **Location:** locationID: M2; continent: Europe; country: Spain; countryCode: ES; stateProvince: Extremadura; county: Cáceres; locality: Fuente del Frances; verbatimElevation: 320.72; decimalLatitude: 39.828; decimalLongitude: -6.03249; geodeticDatum: WGS84; **Event:** eventID: 1; samplingProtocol: Beating; eventTime: Night
- **Type status:** Other material. **Occurrence:** individualCount: 1; sex: female; **Location:** locationID: O2; continent: Europe; country: Spain; countryCode: ES; stateProvince: Aragón; county: Huesca; locality: Rebilla; verbatimElevation: 1158.13; decimalLatitude: 42.59427; decimalLongitude: 0.1529; geodeticDatum: WGS84; **Event:** eventID: 1; samplingProtocol: Aerial; eventTime: Night
- **Type status:** Other material. **Occurrence:** individualCount: 2; sex: male; **Location:** locationID: O2; continent: Europe; country: Spain; countryCode: ES; stateProvince: Aragón; county: Huesca; locality: Rebilla; verbatimElevation: 1158.13; decimalLatitude: 42.59427; decimalLongitude: 0.1529; geodeticDatum: WGS84; **Event:** eventID: 1; samplingProtocol: Aerial; eventTime: Night

##### Distribution

Iberian Peninsula, France, Algeria

#### Tmarus stellio

Simon, 1875

##### Materials

- **Type status:** Other material. **Occurrence:** individualCount: 1; sex: female; **Location:** locationID: O2; continent: Europe; country: Spain; countryCode: ES; stateProvince: Aragón; county: Huesca; locality: Rebilla; verbatimElevation: 1158.13; decimalLatitude: 42.59427; decimalLongitude: 0.1529; geodeticDatum: WGS84; **Event:** eventID: 1; samplingProtocol: Aerial; eventTime: Night
- **Type status:** Other material. **Occurrence:** individualCount: 2; sex: female; **Location:** locationID: O2; continent: Europe; country: Spain; countryCode: ES; stateProvince: Aragón; county: Huesca; locality: Rebilla; verbatimElevation: 1158.13; decimalLatitude: 42.59427; decimalLongitude: 0.1529; geodeticDatum: WGS84; **Event:** eventID: 2; samplingProtocol: Aerial; eventTime: Night
- **Type status:** Other material. **Occurrence:** individualCount: 2; sex: female; **Location:** locationID: O2; continent: Europe; country: Spain; countryCode: ES; stateProvince: Aragón; county: Huesca; locality: Rebilla; verbatimElevation: 1158.13; decimalLatitude: 42.59427; decimalLongitude: 0.1529; geodeticDatum: WGS84; **Event:** eventID: 1; samplingProtocol: Beating; eventTime: Night
- **Type status:** Other material. **Occurrence:** individualCount: 1; sex: female; **Location:** locationID: O2; continent: Europe; country: Spain; countryCode: ES; stateProvince: Aragón; county: Huesca; locality: Rebilla; verbatimElevation: 1158.13; decimalLatitude: 42.59427; decimalLongitude: 0.1529; geodeticDatum: WGS84; **Event:** eventID: 1; samplingProtocol: Beating; eventTime: Night
- **Type status:** Other material. **Occurrence:** individualCount: 1; sex: male; **Location:** locationID: O2; continent: Europe; country: Spain; countryCode: ES; stateProvince: Aragón; county: Huesca; locality: Rebilla; verbatimElevation: 1158.13; decimalLatitude: 42.59427; decimalLongitude: 0.1529; geodeticDatum: WGS84; **Event:** eventID: 1; samplingProtocol: Beating; eventTime: Night
- **Type status:** Other material. **Occurrence:** individualCount: 1; sex: male; **Location:** locationID: O2; continent: Europe; country: Spain; countryCode: ES; stateProvince: Aragón; county: Huesca; locality: Rebilla; verbatimElevation: 1158.13; decimalLatitude: 42.59427; decimalLongitude: 0.1529; geodeticDatum: WGS84; **Event:** eventID: 2; samplingProtocol: Beating; eventTime: Day
- **Type status:** Other material. **Occurrence:** individualCount: 1; sex: female; **Location:** locationID: P2; continent: Europe; country: Spain; countryCode: ES; stateProvince: Castilla y León; county: León; locality: Joyoguelas; verbatimElevation: 763.98; decimalLatitude: 43.17771; decimalLongitude: -4.90579; geodeticDatum: WGS84; **Event:** eventID: 1; samplingProtocol: Beating; eventTime: Day
- **Type status:** Other material. **Occurrence:** individualCount: 1; sex: female; **Location:** locationID: P2; continent: Europe; country: Spain; countryCode: ES; stateProvince: Castilla y León; county: León; locality: Joyoguelas; verbatimElevation: 763.98; decimalLatitude: 43.17771; decimalLongitude: -4.90579; geodeticDatum: WGS84; **Event:** eventID: 2; samplingProtocol: Beating; eventTime: Day
- **Type status:** Other material. **Occurrence:** individualCount: 1; sex: male; **Location:** locationID: P2; continent: Europe; country: Spain; countryCode: ES; stateProvince: Castilla y León; county: León; locality: Joyoguelas; verbatimElevation: 763.98; decimalLatitude: 43.17771; decimalLongitude: -4.90579; geodeticDatum: WGS84; **Event:** eventID: 1; samplingProtocol: Beating; eventTime: Night
- **Type status:** Other material. **Occurrence:** individualCount: 1; sex: female; **Location:** locationID: P2; continent: Europe; country: Spain; countryCode: ES; stateProvince: Castilla y León; county: León; locality: Joyoguelas; verbatimElevation: 763.98; decimalLatitude: 43.17771; decimalLongitude: -4.90579; geodeticDatum: WGS84; **Event:** eventID: 1; samplingProtocol: Sweeping; eventTime: Night

##### Distribution

Palearctic

#### Xysticus audax

(Schrank, 1803)

##### Materials

- **Type status:** Other material. **Occurrence:** individualCount: 1; sex: male; **Location:** locationID: A1; continent: Europe; country: Spain; countryCode: ES; stateProvince: Catalonia; county: Lleida; locality: Sola de Boi; verbatimElevation: 1759.8; decimalLatitude: 42.54958; decimalLongitude: 0.87254; geodeticDatum: WGS84; **Event:** eventID: A; samplingProtocol: Pitfall
- **Type status:** Other material. **Occurrence:** individualCount: 1; sex: male; **Location:** locationID: A1; continent: Europe; country: Spain; countryCode: ES; stateProvince: Catalonia; county: Lleida; locality: Sola de Boi; verbatimElevation: 1759.8; decimalLatitude: 42.54958; decimalLongitude: 0.87254; geodeticDatum: WGS84; **Event:** eventID: B; samplingProtocol: Pitfall
- **Type status:** Other material. **Occurrence:** individualCount: 1; sex: male; **Location:** locationID: A1; continent: Europe; country: Spain; countryCode: ES; stateProvince: Catalonia; county: Lleida; locality: Sola de Boi; verbatimElevation: 1759.8; decimalLatitude: 42.54958; decimalLongitude: 0.87254; geodeticDatum: WGS84; **Event:** eventID: D; samplingProtocol: Pitfall
- **Type status:** Other material. **Occurrence:** individualCount: 1; sex: male; **Location:** locationID: A2; continent: Europe; country: Spain; countryCode: ES; stateProvince: Catalonia; county: Lleida; locality: Sola de Boi; verbatimElevation: 1738.7; decimalLatitude: 42.54913; decimalLongitude: 0.87137; geodeticDatum: WGS84; **Event:** eventID: 1; samplingProtocol: Beating; eventTime: Day

##### Distribution

Palearctic

#### Xysticus cristatus

(Clerck, 1757)

##### Materials

- **Type status:** Other material. **Occurrence:** individualCount: 1; sex: female; **Location:** locationID: P4; continent: Europe; country: Spain; countryCode: ES; stateProvince: Castilla y León; county: León; locality: El Canto; verbatimElevation: 943.48; decimalLatitude: 43.17227; decimalLongitude: -4.90857; geodeticDatum: WGS84; **Event:** eventID: 1; samplingProtocol: Sweeping; eventTime: Day
- **Type status:** Other material. **Occurrence:** individualCount: 1; sex: female; **Location:** locationID: P4; continent: Europe; country: Spain; countryCode: ES; stateProvince: Castilla y León; county: León; locality: El Canto; verbatimElevation: 943.48; decimalLatitude: 43.17227; decimalLongitude: -4.90857; geodeticDatum: WGS84; **Event:** eventID: 1; samplingProtocol: Sweeping; eventTime: Night

##### Distribution

Palearctic

#### Xysticus erraticus

(Blackwall, 1834)

##### Materials

- **Type status:** Other material. **Occurrence:** individualCount: 1; sex: male; **Location:** locationID: O1; continent: Europe; country: Spain; countryCode: ES; stateProvince: Aragón; county: Huesca; locality: O Furno; verbatimElevation: 1396.73; decimalLatitude: 42.60677; decimalLongitude: 0.13135; geodeticDatum: WGS84; **Event:** eventID: H; samplingProtocol: Pitfall
- **Type status:** Other material. **Occurrence:** individualCount: 1; sex: male; **Location:** locationID: P1; continent: Europe; country: Spain; countryCode: ES; stateProvince: Castilla y León; county: León; locality: Monte Robledo; verbatimElevation: 1071.58; decimalLatitude: 43.1445; decimalLongitude: -4.92675; geodeticDatum: WGS84; **Event:** eventID: B; samplingProtocol: Pitfall
- **Type status:** Other material. **Occurrence:** individualCount: 1; sex: male; **Location:** locationID: P1; continent: Europe; country: Spain; countryCode: ES; stateProvince: Castilla y León; county: León; locality: Monte Robledo; verbatimElevation: 1071.58; decimalLatitude: 43.1445; decimalLongitude: -4.92675; geodeticDatum: WGS84; **Event:** eventID: F; samplingProtocol: Pitfall
- **Type status:** Other material. **Occurrence:** individualCount: 1; sex: female; **Location:** locationID: P1; continent: Europe; country: Spain; countryCode: ES; stateProvince: Castilla y León; county: León; locality: Monte Robledo; verbatimElevation: 1071.58; decimalLatitude: 43.1445; decimalLongitude: -4.92675; geodeticDatum: WGS84; **Event:** eventID: H; samplingProtocol: Pitfall
- **Type status:** Other material. **Occurrence:** individualCount: 1; sex: male; **Location:** locationID: P1; continent: Europe; country: Spain; countryCode: ES; stateProvince: Castilla y León; county: León; locality: Monte Robledo; verbatimElevation: 1071.58; decimalLatitude: 43.1445; decimalLongitude: -4.92675; geodeticDatum: WGS84; **Event:** eventID: H; samplingProtocol: Pitfall
- **Type status:** Other material. **Occurrence:** individualCount: 2; sex: male; **Location:** locationID: P1; continent: Europe; country: Spain; countryCode: ES; stateProvince: Castilla y León; county: León; locality: Monte Robledo; verbatimElevation: 1071.58; decimalLatitude: 43.1445; decimalLongitude: -4.92675; geodeticDatum: WGS84; **Event:** eventID: J; samplingProtocol: Pitfall
- **Type status:** Other material. **Occurrence:** individualCount: 1; sex: female; **Location:** locationID: P1; continent: Europe; country: Spain; countryCode: ES; stateProvince: Castilla y León; county: León; locality: Monte Robledo; verbatimElevation: 1071.58; decimalLatitude: 43.1445; decimalLongitude: -4.92675; geodeticDatum: WGS84; **Event:** eventID: 1; samplingProtocol: Sweeping; eventTime: Night
- **Type status:** Other material. **Occurrence:** individualCount: 2; sex: male; **Location:** locationID: P2; continent: Europe; country: Spain; countryCode: ES; stateProvince: Castilla y León; county: León; locality: Joyoguelas; verbatimElevation: 763.98; decimalLatitude: 43.17771; decimalLongitude: -4.90579; geodeticDatum: WGS84; **Event:** eventID: B; samplingProtocol: Pitfall
- **Type status:** Other material. **Occurrence:** individualCount: 1; sex: female; **Location:** locationID: P2; continent: Europe; country: Spain; countryCode: ES; stateProvince: Castilla y León; county: León; locality: Joyoguelas; verbatimElevation: 763.98; decimalLatitude: 43.17771; decimalLongitude: -4.90579; geodeticDatum: WGS84; **Event:** eventID: C; samplingProtocol: Pitfall
- **Type status:** Other material. **Occurrence:** individualCount: 1; sex: male; **Location:** locationID: P2; continent: Europe; country: Spain; countryCode: ES; stateProvince: Castilla y León; county: León; locality: Joyoguelas; verbatimElevation: 763.98; decimalLatitude: 43.17771; decimalLongitude: -4.90579; geodeticDatum: WGS84; **Event:** eventID: C; samplingProtocol: Pitfall
- **Type status:** Other material. **Occurrence:** individualCount: 1; sex: female; **Location:** locationID: P2; continent: Europe; country: Spain; countryCode: ES; stateProvince: Castilla y León; county: León; locality: Joyoguelas; verbatimElevation: 763.98; decimalLatitude: 43.17771; decimalLongitude: -4.90579; geodeticDatum: WGS84; **Event:** eventID: D; samplingProtocol: Pitfall
- **Type status:** Other material. **Occurrence:** individualCount: 3; sex: male; **Location:** locationID: P2; continent: Europe; country: Spain; countryCode: ES; stateProvince: Castilla y León; county: León; locality: Joyoguelas; verbatimElevation: 763.98; decimalLatitude: 43.17771; decimalLongitude: -4.90579; geodeticDatum: WGS84; **Event:** eventID: D; samplingProtocol: Pitfall
- **Type status:** Other material. **Occurrence:** individualCount: 1; sex: male; **Location:** locationID: P2; continent: Europe; country: Spain; countryCode: ES; stateProvince: Castilla y León; county: León; locality: Joyoguelas; verbatimElevation: 763.98; decimalLatitude: 43.17771; decimalLongitude: -4.90579; geodeticDatum: WGS84; **Event:** eventID: F; samplingProtocol: Pitfall
- **Type status:** Other material. **Occurrence:** individualCount: 2; sex: male; **Location:** locationID: P2; continent: Europe; country: Spain; countryCode: ES; stateProvince: Castilla y León; county: León; locality: Joyoguelas; verbatimElevation: 763.98; decimalLatitude: 43.17771; decimalLongitude: -4.90579; geodeticDatum: WGS84; **Event:** eventID: G; samplingProtocol: Pitfall
- **Type status:** Other material. **Occurrence:** individualCount: 1; sex: male; **Location:** locationID: P2; continent: Europe; country: Spain; countryCode: ES; stateProvince: Castilla y León; county: León; locality: Joyoguelas; verbatimElevation: 763.98; decimalLatitude: 43.17771; decimalLongitude: -4.90579; geodeticDatum: WGS84; **Event:** eventID: H; samplingProtocol: Pitfall
- **Type status:** Other material. **Occurrence:** individualCount: 3; sex: male; **Location:** locationID: P2; continent: Europe; country: Spain; countryCode: ES; stateProvince: Castilla y León; county: León; locality: Joyoguelas; verbatimElevation: 763.98; decimalLatitude: 43.17771; decimalLongitude: -4.90579; geodeticDatum: WGS84; **Event:** eventID: J; samplingProtocol: Pitfall
- **Type status:** Other material. **Occurrence:** individualCount: 1; sex: male; **Location:** locationID: P2; continent: Europe; country: Spain; countryCode: ES; stateProvince: Castilla y León; county: León; locality: Joyoguelas; verbatimElevation: 763.98; decimalLatitude: 43.17771; decimalLongitude: -4.90579; geodeticDatum: WGS84; **Event:** eventID: L; samplingProtocol: Pitfall
- **Type status:** Other material. **Occurrence:** individualCount: 1; sex: male; **Location:** locationID: P2; continent: Europe; country: Spain; countryCode: ES; stateProvince: Castilla y León; county: León; locality: Joyoguelas; verbatimElevation: 763.98; decimalLatitude: 43.17771; decimalLongitude: -4.90579; geodeticDatum: WGS84; **Event:** eventID: 2; samplingProtocol: Sweeping; eventTime: Day
- **Type status:** Other material. **Occurrence:** individualCount: 1; sex: female; **Location:** locationID: P3; continent: Europe; country: Spain; countryCode: ES; stateProvince: Castilla y León; county: León; locality: Las Arroyas; verbatimElevation: 1097.1; decimalLatitude: 43.14351; decimalLongitude: -4.94878; geodeticDatum: WGS84; **Event:** eventID: A; samplingProtocol: Pitfall
- **Type status:** Other material. **Occurrence:** individualCount: 3; sex: male; **Location:** locationID: P3; continent: Europe; country: Spain; countryCode: ES; stateProvince: Castilla y León; county: León; locality: Las Arroyas; verbatimElevation: 1097.1; decimalLatitude: 43.14351; decimalLongitude: -4.94878; geodeticDatum: WGS84; **Event:** eventID: A; samplingProtocol: Pitfall
- **Type status:** Other material. **Occurrence:** individualCount: 1; sex: male; **Location:** locationID: P3; continent: Europe; country: Spain; countryCode: ES; stateProvince: Castilla y León; county: León; locality: Las Arroyas; verbatimElevation: 1097.1; decimalLatitude: 43.14351; decimalLongitude: -4.94878; geodeticDatum: WGS84; **Event:** eventID: B; samplingProtocol: Pitfall
- **Type status:** Other material. **Occurrence:** individualCount: 2; sex: male; **Location:** locationID: P3; continent: Europe; country: Spain; countryCode: ES; stateProvince: Castilla y León; county: León; locality: Las Arroyas; verbatimElevation: 1097.1; decimalLatitude: 43.14351; decimalLongitude: -4.94878; geodeticDatum: WGS84; **Event:** eventID: C; samplingProtocol: Pitfall
- **Type status:** Other material. **Occurrence:** individualCount: 1; sex: female; **Location:** locationID: P3; continent: Europe; country: Spain; countryCode: ES; stateProvince: Castilla y León; county: León; locality: Las Arroyas; verbatimElevation: 1097.1; decimalLatitude: 43.14351; decimalLongitude: -4.94878; geodeticDatum: WGS84; **Event:** eventID: D; samplingProtocol: Pitfall
- **Type status:** Other material. **Occurrence:** individualCount: 3; sex: male; **Location:** locationID: P3; continent: Europe; country: Spain; countryCode: ES; stateProvince: Castilla y León; county: León; locality: Las Arroyas; verbatimElevation: 1097.1; decimalLatitude: 43.14351; decimalLongitude: -4.94878; geodeticDatum: WGS84; **Event:** eventID: D; samplingProtocol: Pitfall
- **Type status:** Other material. **Occurrence:** individualCount: 2; sex: male; **Location:** locationID: P3; continent: Europe; country: Spain; countryCode: ES; stateProvince: Castilla y León; county: León; locality: Las Arroyas; verbatimElevation: 1097.1; decimalLatitude: 43.14351; decimalLongitude: -4.94878; geodeticDatum: WGS84; **Event:** eventID: E; samplingProtocol: Pitfall
- **Type status:** Other material. **Occurrence:** individualCount: 1; sex: male; **Location:** locationID: P3; continent: Europe; country: Spain; countryCode: ES; stateProvince: Castilla y León; county: León; locality: Las Arroyas; verbatimElevation: 1097.1; decimalLatitude: 43.14351; decimalLongitude: -4.94878; geodeticDatum: WGS84; **Event:** eventID: H; samplingProtocol: Pitfall
- **Type status:** Other material. **Occurrence:** individualCount: 3; sex: male; **Location:** locationID: P3; continent: Europe; country: Spain; countryCode: ES; stateProvince: Castilla y León; county: León; locality: Las Arroyas; verbatimElevation: 1097.1; decimalLatitude: 43.14351; decimalLongitude: -4.94878; geodeticDatum: WGS84; **Event:** eventID: I; samplingProtocol: Pitfall
- **Type status:** Other material. **Occurrence:** individualCount: 3; sex: male; **Location:** locationID: P3; continent: Europe; country: Spain; countryCode: ES; stateProvince: Castilla y León; county: León; locality: Las Arroyas; verbatimElevation: 1097.1; decimalLatitude: 43.14351; decimalLongitude: -4.94878; geodeticDatum: WGS84; **Event:** eventID: J; samplingProtocol: Pitfall
- **Type status:** Other material. **Occurrence:** individualCount: 1; sex: male; **Location:** locationID: P3; continent: Europe; country: Spain; countryCode: ES; stateProvince: Castilla y León; county: León; locality: Las Arroyas; verbatimElevation: 1097.1; decimalLatitude: 43.14351; decimalLongitude: -4.94878; geodeticDatum: WGS84; **Event:** eventID: K; samplingProtocol: Pitfall
- **Type status:** Other material. **Occurrence:** individualCount: 3; sex: male; **Location:** locationID: P4; continent: Europe; country: Spain; countryCode: ES; stateProvince: Castilla y León; county: León; locality: El Canto; verbatimElevation: 943.48; decimalLatitude: 43.17227; decimalLongitude: -4.90857; geodeticDatum: WGS84; **Event:** eventID: C; samplingProtocol: Pitfall
- **Type status:** Other material. **Occurrence:** individualCount: 4; sex: male; **Location:** locationID: P4; continent: Europe; country: Spain; countryCode: ES; stateProvince: Castilla y León; county: León; locality: El Canto; verbatimElevation: 943.48; decimalLatitude: 43.17227; decimalLongitude: -4.90857; geodeticDatum: WGS84; **Event:** eventID: D; samplingProtocol: Pitfall
- **Type status:** Other material. **Occurrence:** individualCount: 3; sex: male; **Location:** locationID: P4; continent: Europe; country: Spain; countryCode: ES; stateProvince: Castilla y León; county: León; locality: El Canto; verbatimElevation: 943.48; decimalLatitude: 43.17227; decimalLongitude: -4.90857; geodeticDatum: WGS84; **Event:** eventID: G; samplingProtocol: Pitfall
- **Type status:** Other material. **Occurrence:** individualCount: 1; sex: female; **Location:** locationID: P4; continent: Europe; country: Spain; countryCode: ES; stateProvince: Castilla y León; county: León; locality: El Canto; verbatimElevation: 943.48; decimalLatitude: 43.17227; decimalLongitude: -4.90857; geodeticDatum: WGS84; **Event:** eventID: H; samplingProtocol: Pitfall
- **Type status:** Other material. **Occurrence:** individualCount: 1; sex: male; **Location:** locationID: P4; continent: Europe; country: Spain; countryCode: ES; stateProvince: Castilla y León; county: León; locality: El Canto; verbatimElevation: 943.48; decimalLatitude: 43.17227; decimalLongitude: -4.90857; geodeticDatum: WGS84; **Event:** eventID: H; samplingProtocol: Pitfall
- **Type status:** Other material. **Occurrence:** individualCount: 1; sex: male; **Location:** locationID: P4; continent: Europe; country: Spain; countryCode: ES; stateProvince: Castilla y León; county: León; locality: El Canto; verbatimElevation: 943.48; decimalLatitude: 43.17227; decimalLongitude: -4.90857; geodeticDatum: WGS84; **Event:** eventID: I; samplingProtocol: Pitfall
- **Type status:** Other material. **Occurrence:** individualCount: 1; sex: male; **Location:** locationID: P4; continent: Europe; country: Spain; countryCode: ES; stateProvince: Castilla y León; county: León; locality: El Canto; verbatimElevation: 943.48; decimalLatitude: 43.17227; decimalLongitude: -4.90857; geodeticDatum: WGS84; **Event:** eventID: L; samplingProtocol: Pitfall
- **Type status:** Other material. **Occurrence:** individualCount: 1; sex: female; **Location:** locationID: P4; continent: Europe; country: Spain; countryCode: ES; stateProvince: Castilla y León; county: León; locality: El Canto; verbatimElevation: 943.48; decimalLatitude: 43.17227; decimalLongitude: -4.90857; geodeticDatum: WGS84; **Event:** eventID: 1; samplingProtocol: Sweeping; eventTime: Night

##### Distribution

Europe, Russia

#### Xysticus ferrugineus

Menge, 1876

##### Materials

- **Type status:** Other material. **Occurrence:** individualCount: 1; sex: female; **Location:** locationID: S1; continent: Europe; country: Spain; countryCode: ES; stateProvince: Andalucía; county: Granada; locality: Soportujar; verbatimElevation: 1786.57; decimalLatitude: 36.96151; decimalLongitude: -3.41881; geodeticDatum: WGS84; **Event:** eventID: F; samplingProtocol: Pitfall

##### Distribution

Palearctic

#### Xysticus kempeleni

Thorell, 1872

##### Materials

- **Type status:** Other material. **Occurrence:** individualCount: 1; sex: male; **Location:** locationID: P4; continent: Europe; country: Spain; countryCode: ES; stateProvince: Castilla y León; county: León; locality: El Canto; verbatimElevation: 943.48; decimalLatitude: 43.17227; decimalLongitude: -4.90857; geodeticDatum: WGS84; **Event:** eventID: A; samplingProtocol: Pitfall
- **Type status:** Other material. **Occurrence:** individualCount: 1; sex: male; **Location:** locationID: P4; continent: Europe; country: Spain; countryCode: ES; stateProvince: Castilla y León; county: León; locality: El Canto; verbatimElevation: 943.48; decimalLatitude: 43.17227; decimalLongitude: -4.90857; geodeticDatum: WGS84; **Event:** eventID: E; samplingProtocol: Pitfall

##### Distribution

Europe to Central Asia

#### Xysticus lanio

C. L. Koch, 1835

##### Materials

- **Type status:** Other material. **Occurrence:** individualCount: 1; sex: female; **Location:** locationID: O1; continent: Europe; country: Spain; countryCode: ES; stateProvince: Aragón; county: Huesca; locality: O Furno; verbatimElevation: 1396.73; decimalLatitude: 42.60677; decimalLongitude: 0.13135; geodeticDatum: WGS84; **Event:** eventID: 1; samplingProtocol: Beating; eventTime: Day
- **Type status:** Other material. **Occurrence:** individualCount: 2; sex: male; **Location:** locationID: O1; continent: Europe; country: Spain; countryCode: ES; stateProvince: Aragón; county: Huesca; locality: O Furno; verbatimElevation: 1396.73; decimalLatitude: 42.60677; decimalLongitude: 0.13135; geodeticDatum: WGS84; **Event:** eventID: 1; samplingProtocol: Beating; eventTime: Day
- **Type status:** Other material. **Occurrence:** individualCount: 1; sex: male; **Location:** locationID: O1; continent: Europe; country: Spain; countryCode: ES; stateProvince: Aragón; county: Huesca; locality: O Furno; verbatimElevation: 1396.73; decimalLatitude: 42.60677; decimalLongitude: 0.13135; geodeticDatum: WGS84; **Event:** eventID: 1; samplingProtocol: Sweeping; eventTime: Day
- **Type status:** Other material. **Occurrence:** individualCount: 1; sex: female; **Location:** locationID: O1; continent: Europe; country: Spain; countryCode: ES; stateProvince: Aragón; county: Huesca; locality: O Furno; verbatimElevation: 1396.73; decimalLatitude: 42.60677; decimalLongitude: 0.13135; geodeticDatum: WGS84; **Event:** eventID: 1; samplingProtocol: Sweeping; eventTime: Night
- **Type status:** Other material. **Occurrence:** individualCount: 1; sex: female; **Location:** locationID: O2; continent: Europe; country: Spain; countryCode: ES; stateProvince: Aragón; county: Huesca; locality: Rebilla; verbatimElevation: 1158.13; decimalLatitude: 42.59427; decimalLongitude: 0.1529; geodeticDatum: WGS84; **Event:** eventID: 1; samplingProtocol: Beating; eventTime: Night
- **Type status:** Other material. **Occurrence:** individualCount: 1; sex: female; **Location:** locationID: O2; continent: Europe; country: Spain; countryCode: ES; stateProvince: Aragón; county: Huesca; locality: Rebilla; verbatimElevation: 1158.13; decimalLatitude: 42.59427; decimalLongitude: 0.1529; geodeticDatum: WGS84; **Event:** eventID: 1; samplingProtocol: Sweeping; eventTime: Day

##### Distribution

Palearctic

#### Xysticus ninnii

Thorell, 1872

##### Materials

- **Type status:** Other material. **Occurrence:** individualCount: 1; sex: female; **Location:** locationID: S2; continent: Europe; country: Spain; countryCode: ES; stateProvince: Andalucía; county: Granada; locality: Camarate; verbatimElevation: 1713.96; decimalLatitude: 37.18377; decimalLongitude: -3.26282; geodeticDatum: WGS84; **Event:** eventID: 2; samplingProtocol: Beating; eventTime: Night
- **Type status:** Other material. **Occurrence:** individualCount: 1; sex: male; **Location:** locationID: S2; continent: Europe; country: Spain; countryCode: ES; stateProvince: Andalucía; county: Granada; locality: Camarate; verbatimElevation: 1713.96; decimalLatitude: 37.18377; decimalLongitude: -3.26282; geodeticDatum: WGS84; **Event:** eventID: C; samplingProtocol: Pitfall
- **Type status:** Other material. **Occurrence:** individualCount: 1; sex: male; **Location:** locationID: S2; continent: Europe; country: Spain; countryCode: ES; stateProvince: Andalucía; county: Granada; locality: Camarate; verbatimElevation: 1713.96; decimalLatitude: 37.18377; decimalLongitude: -3.26282; geodeticDatum: WGS84; **Event:** eventID: F; samplingProtocol: Pitfall
- **Type status:** Other material. **Occurrence:** individualCount: 1; sex: male; **Location:** locationID: S2; continent: Europe; country: Spain; countryCode: ES; stateProvince: Andalucía; county: Granada; locality: Camarate; verbatimElevation: 1713.96; decimalLatitude: 37.18377; decimalLongitude: -3.26282; geodeticDatum: WGS84; **Event:** eventID: J; samplingProtocol: Pitfall
- **Type status:** Other material. **Occurrence:** individualCount: 1; sex: male; **Location:** locationID: S2; continent: Europe; country: Spain; countryCode: ES; stateProvince: Andalucía; county: Granada; locality: Camarate; verbatimElevation: 1713.96; decimalLatitude: 37.18377; decimalLongitude: -3.26282; geodeticDatum: WGS84; **Event:** eventID: L; samplingProtocol: Pitfall

##### Distribution

Palearctic

#### Xysticus sp31

##### Materials

- **Type status:** Other material. **Occurrence:** individualCount: 2; sex: male; **Location:** locationID: S1; continent: Europe; country: Spain; countryCode: ES; stateProvince: Andalucía; county: Granada; locality: Soportujar; verbatimElevation: 1786.57; decimalLatitude: 36.96151; decimalLongitude: -3.41881; geodeticDatum: WGS84; **Event:** eventID: H; samplingProtocol: Pitfall
- **Type status:** Other material. **Occurrence:** individualCount: 1; sex: male; **Location:** locationID: S1; continent: Europe; country: Spain; countryCode: ES; stateProvince: Andalucía; county: Granada; locality: Soportujar; verbatimElevation: 1786.57; decimalLatitude: 36.96151; decimalLongitude: -3.41881; geodeticDatum: WGS84; **Event:** eventID: L; samplingProtocol: Pitfall

##### Distribution

?

##### Notes

This species belongs in the *Xysticus cribratus* group of species (also *Bassaniodes* sensu Lehtinen 2002), but we could not identify the available specimens.

#### Xysticus sp42

##### Materials

- **Type status:** Other material. **Occurrence:** individualCount: 1; sex: male; **Location:** locationID: C4; continent: Europe; country: Spain; countryCode: ES; stateProvince: Castilla-La Mancha; county: Ciudad Real; locality: La Quesera; verbatimElevation: 772.3; decimalLatitude: 39.36337; decimalLongitude: -4.41704; geodeticDatum: WGS84; **Event:** eventID: 4; samplingProtocol: Aerial; eventTime: Night
- **Type status:** Other material. **Occurrence:** individualCount: 1; sex: female; **Location:** locationID: C4; continent: Europe; country: Spain; countryCode: ES; stateProvince: Castilla-La Mancha; county: Ciudad Real; locality: La Quesera; verbatimElevation: 772.3; decimalLatitude: 39.36337; decimalLongitude: -4.41704; geodeticDatum: WGS84; **Event:** eventID: 4; samplingProtocol: Aerial; eventTime: Night

##### Distribution

?

##### Notes

This is a species of *Xysticus* C. L. Koch, 1835, which we were unable to identify.

#### Titanoecidae

Lehtinen, 1967

#### Titanoeca schineri

L. Koch, 1872

##### Materials

- **Type status:** Other material. **Occurrence:** individualCount: 1; sex: male; **Location:** locationID: S2; continent: Europe; country: Spain; countryCode: ES; stateProvince: Andalucía; county: Granada; locality: Camarate; verbatimElevation: 1713.96; decimalLatitude: 37.18377; decimalLongitude: -3.26282; geodeticDatum: WGS84; **Event:** eventID: A; samplingProtocol: Pitfall

##### Distribution

Palearctic

##### Notes

New record for the Iberian Peninsula.

#### Uloboridae

Thorell, 1869

#### Hyptiotes flavidus

(Blackwall, 1862)

##### Materials

- **Type status:** Other material. **Occurrence:** individualCount: 1; sex: female; **Location:** locationID: C2; continent: Europe; country: Spain; countryCode: ES; stateProvince: Castilla-La Mancha; county: Ciudad Real; locality: Valle Brezoso; verbatimElevation: 739.31; decimalLatitude: 39.35159; decimalLongitude: -4.3589; geodeticDatum: WGS84; **Event:** eventID: 2; samplingProtocol: Beating; eventTime: Day
- **Type status:** Other material. **Occurrence:** individualCount: 1; sex: female; **Location:** locationID: M2; continent: Europe; country: Spain; countryCode: ES; stateProvince: Extremadura; county: Cáceres; locality: Fuente del Frances; verbatimElevation: 320.72; decimalLatitude: 39.828; decimalLongitude: -6.03249; geodeticDatum: WGS84; **Event:** eventID: 1; samplingProtocol: Beating; eventTime: Day

##### Distribution

Mediterranean to Caucasus

#### Polenecia producta

(Simon, 1873)

##### Materials

- **Type status:** Other material. **Occurrence:** individualCount: 1; sex: female; **Location:** locationID: C2; continent: Europe; country: Spain; countryCode: ES; stateProvince: Castilla-La Mancha; county: Ciudad Real; locality: Valle Brezoso; verbatimElevation: 739.31; decimalLatitude: 39.35159; decimalLongitude: -4.3589; geodeticDatum: WGS84; **Event:** eventID: 2; samplingProtocol: Aerial; eventTime: Night

##### Distribution

Mediterranean to Azerbaijan

#### Uloborus walckenaerius

Latreille, 1806

##### Materials

- **Type status:** Other material. **Occurrence:** individualCount: 1; sex: male; **Location:** locationID: C2; continent: Europe; country: Spain; countryCode: ES; stateProvince: Castilla-La Mancha; county: Ciudad Real; locality: Valle Brezoso; verbatimElevation: 739.31; decimalLatitude: 39.35159; decimalLongitude: -4.3589; geodeticDatum: WGS84; **Event:** eventID: 1; samplingProtocol: Aerial; eventTime: Night
- **Type status:** Other material. **Occurrence:** individualCount: 1; sex: female; **Location:** locationID: C2; continent: Europe; country: Spain; countryCode: ES; stateProvince: Castilla-La Mancha; county: Ciudad Real; locality: Valle Brezoso; verbatimElevation: 739.31; decimalLatitude: 39.35159; decimalLongitude: -4.3589; geodeticDatum: WGS84; **Event:** eventID: 2; samplingProtocol: Sweeping; eventTime: Day
- **Type status:** Other material. **Occurrence:** individualCount: 2; sex: male; **Location:** locationID: C3; continent: Europe; country: Spain; countryCode: ES; stateProvince: Castilla-La Mancha; county: Ciudad Real; locality: La Quesera; verbatimElevation: 767.55; decimalLatitude: 39.36177; decimalLongitude: -4.41733; geodeticDatum: WGS84; **Event:** eventID: 1; samplingProtocol: Aerial; eventTime: Night
- **Type status:** Other material. **Occurrence:** individualCount: 1; sex: female; **Location:** locationID: C3; continent: Europe; country: Spain; countryCode: ES; stateProvince: Castilla-La Mancha; county: Ciudad Real; locality: La Quesera; verbatimElevation: 767.55; decimalLatitude: 39.36177; decimalLongitude: -4.41733; geodeticDatum: WGS84; **Event:** eventID: 2; samplingProtocol: Aerial; eventTime: Night
- **Type status:** Other material. **Occurrence:** individualCount: 1; sex: male; **Location:** locationID: C3; continent: Europe; country: Spain; countryCode: ES; stateProvince: Castilla-La Mancha; county: Ciudad Real; locality: La Quesera; verbatimElevation: 767.55; decimalLatitude: 39.36177; decimalLongitude: -4.41733; geodeticDatum: WGS84; **Event:** eventID: 3; samplingProtocol: Aerial; eventTime: Night
- **Type status:** Other material. **Occurrence:** individualCount: 1; sex: male; **Location:** locationID: C3; continent: Europe; country: Spain; countryCode: ES; stateProvince: Castilla-La Mancha; county: Ciudad Real; locality: La Quesera; verbatimElevation: 767.55; decimalLatitude: 39.36177; decimalLongitude: -4.41733; geodeticDatum: WGS84; **Event:** eventID: 4; samplingProtocol: Aerial; eventTime: Night
- **Type status:** Other material. **Occurrence:** individualCount: 1; sex: male; **Location:** locationID: C3; continent: Europe; country: Spain; countryCode: ES; stateProvince: Castilla-La Mancha; county: Ciudad Real; locality: La Quesera; verbatimElevation: 767.55; decimalLatitude: 39.36177; decimalLongitude: -4.41733; geodeticDatum: WGS84; **Event:** eventID: 2; samplingProtocol: Sweeping; eventTime: Night
- **Type status:** Other material. **Occurrence:** individualCount: 2; sex: male; **Location:** locationID: C4; continent: Europe; country: Spain; countryCode: ES; stateProvince: Castilla-La Mancha; county: Ciudad Real; locality: La Quesera; verbatimElevation: 772.3; decimalLatitude: 39.36337; decimalLongitude: -4.41704; geodeticDatum: WGS84; **Event:** eventID: 1; samplingProtocol: Aerial; eventTime: Night
- **Type status:** Other material. **Occurrence:** individualCount: 1; sex: female; **Location:** locationID: C4; continent: Europe; country: Spain; countryCode: ES; stateProvince: Castilla-La Mancha; county: Ciudad Real; locality: La Quesera; verbatimElevation: 772.3; decimalLatitude: 39.36337; decimalLongitude: -4.41704; geodeticDatum: WGS84; **Event:** eventID: 1; samplingProtocol: Sweeping; eventTime: Day
- **Type status:** Other material. **Occurrence:** individualCount: 2; sex: female; **Location:** locationID: C4; continent: Europe; country: Spain; countryCode: ES; stateProvince: Castilla-La Mancha; county: Ciudad Real; locality: La Quesera; verbatimElevation: 772.3; decimalLatitude: 39.36337; decimalLongitude: -4.41704; geodeticDatum: WGS84; **Event:** eventID: 2; samplingProtocol: Sweeping; eventTime: Day
- **Type status:** Other material. **Occurrence:** individualCount: 1; sex: female; **Location:** locationID: O2; continent: Europe; country: Spain; countryCode: ES; stateProvince: Aragón; county: Huesca; locality: Rebilla; verbatimElevation: 1158.13; decimalLatitude: 42.59427; decimalLongitude: 0.1529; geodeticDatum: WGS84; **Event:** eventID: 2; samplingProtocol: Aerial; eventTime: Night
- **Type status:** Other material. **Occurrence:** individualCount: 1; sex: male; **Location:** locationID: S1; continent: Europe; country: Spain; countryCode: ES; stateProvince: Andalucía; county: Granada; locality: Soportujar; verbatimElevation: 1786.57; decimalLatitude: 36.96151; decimalLongitude: -3.41881; geodeticDatum: WGS84; **Event:** eventID: 2; samplingProtocol: Beating; eventTime: Night
- **Type status:** Other material. **Occurrence:** individualCount: 2; sex: male; **Location:** locationID: S1; continent: Europe; country: Spain; countryCode: ES; stateProvince: Andalucía; county: Granada; locality: Soportujar; verbatimElevation: 1786.57; decimalLatitude: 36.96151; decimalLongitude: -3.41881; geodeticDatum: WGS84; **Event:** eventID: 1; samplingProtocol: Sweeping; eventTime: Day
- **Type status:** Other material. **Occurrence:** individualCount: 2; sex: male; **Location:** locationID: S1; continent: Europe; country: Spain; countryCode: ES; stateProvince: Andalucía; county: Granada; locality: Soportujar; verbatimElevation: 1786.57; decimalLatitude: 36.96151; decimalLongitude: -3.41881; geodeticDatum: WGS84; **Event:** eventID: 2; samplingProtocol: Sweeping; eventTime: Day

##### Distribution

Palearctic

#### Zodariidae

Thorell, 1881

#### Amphiledorus sp28

##### Materials

- **Type status:** Other material. **Occurrence:** individualCount: 1; sex: male; **Location:** locationID: S1; continent: Europe; country: Spain; countryCode: ES; stateProvince: Andalucía; county: Granada; locality: Soportujar; verbatimElevation: 1786.57; decimalLatitude: 36.96151; decimalLongitude: -3.41881; geodeticDatum: WGS84; **Event:** eventID: K; samplingProtocol: Pitfall

##### Distribution

?

##### Notes

This is a new species of *Amphiledorus* Jocqué & Bosmans, 2001, to be described in a future publication.

#### Amphiledorus sp40

##### Materials

- **Type status:** Other material. **Occurrence:** individualCount: 1; sex: male; **Location:** locationID: S2; continent: Europe; country: Spain; countryCode: ES; stateProvince: Andalucía; county: Granada; locality: Camarate; verbatimElevation: 1713.96; decimalLatitude: 37.18377; decimalLongitude: -3.26282; geodeticDatum: WGS84; **Event:** eventID: C; samplingProtocol: Pitfall
- **Type status:** Other material. **Occurrence:** individualCount: 1; sex: female; **Location:** locationID: S2; continent: Europe; country: Spain; countryCode: ES; stateProvince: Andalucía; county: Granada; locality: Camarate; verbatimElevation: 1713.96; decimalLatitude: 37.18377; decimalLongitude: -3.26282; geodeticDatum: WGS84; **Event:** eventID: L; samplingProtocol: Pitfall

##### Distribution

?

##### Notes

This is a new species of *Amphiledorus*, to be described in a future publication.

#### Selamia reticulata

(Simon, 1870)

##### Materials

- **Type status:** Other material. **Occurrence:** individualCount: 1; sex: male; **Location:** locationID: C1; continent: Europe; country: Spain; countryCode: ES; stateProvince: Castilla-La Mancha; county: Ciudad Real; locality: Valle Brezoso; verbatimElevation: 756.56; decimalLatitude: 39.35663; decimalLongitude: -4.35912; geodeticDatum: WGS84; **Event:** eventID: H; samplingProtocol: Pitfall
- **Type status:** Other material. **Occurrence:** individualCount: 1; sex: male; **Location:** locationID: C2; continent: Europe; country: Spain; countryCode: ES; stateProvince: Castilla-La Mancha; county: Ciudad Real; locality: Valle Brezoso; verbatimElevation: 739.31; decimalLatitude: 39.35159; decimalLongitude: -4.3589; geodeticDatum: WGS84; **Event:** eventID: C; samplingProtocol: Pitfall
- **Type status:** Other material. **Occurrence:** individualCount: 1; sex: male; **Location:** locationID: C4; continent: Europe; country: Spain; countryCode: ES; stateProvince: Castilla-La Mancha; county: Ciudad Real; locality: La Quesera; verbatimElevation: 772.3; decimalLatitude: 39.36337; decimalLongitude: -4.41704; geodeticDatum: WGS84; **Event:** eventID: D; samplingProtocol: Pitfall
- **Type status:** Other material. **Occurrence:** individualCount: 1; sex: male; **Location:** locationID: C4; continent: Europe; country: Spain; countryCode: ES; stateProvince: Castilla-La Mancha; county: Ciudad Real; locality: La Quesera; verbatimElevation: 772.3; decimalLatitude: 39.36337; decimalLongitude: -4.41704; geodeticDatum: WGS84; **Event:** eventID: E; samplingProtocol: Pitfall
- **Type status:** Other material. **Occurrence:** individualCount: 2; sex: male; **Location:** locationID: M1; continent: Europe; country: Spain; countryCode: ES; stateProvince: Extremadura; county: Cáceres; locality: Peña Falcón; verbatimElevation: 320.6; decimalLatitude: 39.83296; decimalLongitude: -6.0641; geodeticDatum: WGS84; **Event:** eventID: A; samplingProtocol: Pitfall
- **Type status:** Other material. **Occurrence:** individualCount: 5; sex: male; **Location:** locationID: M1; continent: Europe; country: Spain; countryCode: ES; stateProvince: Extremadura; county: Cáceres; locality: Peña Falcón; verbatimElevation: 320.6; decimalLatitude: 39.83296; decimalLongitude: -6.0641; geodeticDatum: WGS84; **Event:** eventID: B; samplingProtocol: Pitfall
- **Type status:** Other material. **Occurrence:** individualCount: 7; sex: male; **Location:** locationID: M1; continent: Europe; country: Spain; countryCode: ES; stateProvince: Extremadura; county: Cáceres; locality: Peña Falcón; verbatimElevation: 320.6; decimalLatitude: 39.83296; decimalLongitude: -6.0641; geodeticDatum: WGS84; **Event:** eventID: C; samplingProtocol: Pitfall
- **Type status:** Other material. **Occurrence:** individualCount: 11; sex: male; **Location:** locationID: M1; continent: Europe; country: Spain; countryCode: ES; stateProvince: Extremadura; county: Cáceres; locality: Peña Falcón; verbatimElevation: 320.6; decimalLatitude: 39.83296; decimalLongitude: -6.0641; geodeticDatum: WGS84; **Event:** eventID: D; samplingProtocol: Pitfall
- **Type status:** Other material. **Occurrence:** individualCount: 2; sex: female; **Location:** locationID: M1; continent: Europe; country: Spain; countryCode: ES; stateProvince: Extremadura; county: Cáceres; locality: Peña Falcón; verbatimElevation: 320.6; decimalLatitude: 39.83296; decimalLongitude: -6.0641; geodeticDatum: WGS84; **Event:** eventID: E; samplingProtocol: Pitfall
- **Type status:** Other material. **Occurrence:** individualCount: 2; sex: male; **Location:** locationID: M1; continent: Europe; country: Spain; countryCode: ES; stateProvince: Extremadura; county: Cáceres; locality: Peña Falcón; verbatimElevation: 320.6; decimalLatitude: 39.83296; decimalLongitude: -6.0641; geodeticDatum: WGS84; **Event:** eventID: E; samplingProtocol: Pitfall
- **Type status:** Other material. **Occurrence:** individualCount: 4; sex: male; **Location:** locationID: M1; continent: Europe; country: Spain; countryCode: ES; stateProvince: Extremadura; county: Cáceres; locality: Peña Falcón; verbatimElevation: 320.6; decimalLatitude: 39.83296; decimalLongitude: -6.0641; geodeticDatum: WGS84; **Event:** eventID: F; samplingProtocol: Pitfall
- **Type status:** Other material. **Occurrence:** individualCount: 1; sex: female; **Location:** locationID: M1; continent: Europe; country: Spain; countryCode: ES; stateProvince: Extremadura; county: Cáceres; locality: Peña Falcón; verbatimElevation: 320.6; decimalLatitude: 39.83296; decimalLongitude: -6.0641; geodeticDatum: WGS84; **Event:** eventID: G; samplingProtocol: Pitfall
- **Type status:** Other material. **Occurrence:** individualCount: 12; sex: male; **Location:** locationID: M1; continent: Europe; country: Spain; countryCode: ES; stateProvince: Extremadura; county: Cáceres; locality: Peña Falcón; verbatimElevation: 320.6; decimalLatitude: 39.83296; decimalLongitude: -6.0641; geodeticDatum: WGS84; **Event:** eventID: G; samplingProtocol: Pitfall
- **Type status:** Other material. **Occurrence:** individualCount: 9; sex: male; **Location:** locationID: M1; continent: Europe; country: Spain; countryCode: ES; stateProvince: Extremadura; county: Cáceres; locality: Peña Falcón; verbatimElevation: 320.6; decimalLatitude: 39.83296; decimalLongitude: -6.0641; geodeticDatum: WGS84; **Event:** eventID: H; samplingProtocol: Pitfall
- **Type status:** Other material. **Occurrence:** individualCount: 5; sex: male; **Location:** locationID: M1; continent: Europe; country: Spain; countryCode: ES; stateProvince: Extremadura; county: Cáceres; locality: Peña Falcón; verbatimElevation: 320.6; decimalLatitude: 39.83296; decimalLongitude: -6.0641; geodeticDatum: WGS84; **Event:** eventID: I; samplingProtocol: Pitfall
- **Type status:** Other material. **Occurrence:** individualCount: 2; sex: male; **Location:** locationID: M1; continent: Europe; country: Spain; countryCode: ES; stateProvince: Extremadura; county: Cáceres; locality: Peña Falcón; verbatimElevation: 320.6; decimalLatitude: 39.83296; decimalLongitude: -6.0641; geodeticDatum: WGS84; **Event:** eventID: J; samplingProtocol: Pitfall
- **Type status:** Other material. **Occurrence:** individualCount: 1; sex: female; **Location:** locationID: M1; continent: Europe; country: Spain; countryCode: ES; stateProvince: Extremadura; county: Cáceres; locality: Peña Falcón; verbatimElevation: 320.6; decimalLatitude: 39.83296; decimalLongitude: -6.0641; geodeticDatum: WGS84; **Event:** eventID: K; samplingProtocol: Pitfall
- **Type status:** Other material. **Occurrence:** individualCount: 4; sex: male; **Location:** locationID: M1; continent: Europe; country: Spain; countryCode: ES; stateProvince: Extremadura; county: Cáceres; locality: Peña Falcón; verbatimElevation: 320.6; decimalLatitude: 39.83296; decimalLongitude: -6.0641; geodeticDatum: WGS84; **Event:** eventID: K; samplingProtocol: Pitfall
- **Type status:** Other material. **Occurrence:** individualCount: 1; sex: male; **Location:** locationID: M1; continent: Europe; country: Spain; countryCode: ES; stateProvince: Extremadura; county: Cáceres; locality: Peña Falcón; verbatimElevation: 320.6; decimalLatitude: 39.83296; decimalLongitude: -6.0641; geodeticDatum: WGS84; **Event:** eventID: L; samplingProtocol: Pitfall
- **Type status:** Other material. **Occurrence:** individualCount: 2; sex: male; **Location:** locationID: M1; continent: Europe; country: Spain; countryCode: ES; stateProvince: Extremadura; county: Cáceres; locality: Peña Falcón; verbatimElevation: 320.6; decimalLatitude: 39.83296; decimalLongitude: -6.0641; geodeticDatum: WGS84; **Event:** eventID: L; samplingProtocol: Pitfall
- **Type status:** Other material. **Occurrence:** individualCount: 1; sex: male; **Location:** locationID: M2; continent: Europe; country: Spain; countryCode: ES; stateProvince: Extremadura; county: Cáceres; locality: Fuente del Frances; verbatimElevation: 320.72; decimalLatitude: 39.828; decimalLongitude: -6.03249; geodeticDatum: WGS84; **Event:** eventID: A; samplingProtocol: Pitfall
- **Type status:** Other material. **Occurrence:** individualCount: 2; sex: male; **Location:** locationID: M2; continent: Europe; country: Spain; countryCode: ES; stateProvince: Extremadura; county: Cáceres; locality: Fuente del Frances; verbatimElevation: 320.72; decimalLatitude: 39.828; decimalLongitude: -6.03249; geodeticDatum: WGS84; **Event:** eventID: C; samplingProtocol: Pitfall
- **Type status:** Other material. **Occurrence:** individualCount: 1; sex: female; **Location:** locationID: M2; continent: Europe; country: Spain; countryCode: ES; stateProvince: Extremadura; county: Cáceres; locality: Fuente del Frances; verbatimElevation: 320.72; decimalLatitude: 39.828; decimalLongitude: -6.03249; geodeticDatum: WGS84; **Event:** eventID: E; samplingProtocol: Pitfall
- **Type status:** Other material. **Occurrence:** individualCount: 8; sex: male; **Location:** locationID: M2; continent: Europe; country: Spain; countryCode: ES; stateProvince: Extremadura; county: Cáceres; locality: Fuente del Frances; verbatimElevation: 320.72; decimalLatitude: 39.828; decimalLongitude: -6.03249; geodeticDatum: WGS84; **Event:** eventID: E; samplingProtocol: Pitfall
- **Type status:** Other material. **Occurrence:** individualCount: 7; sex: male; **Location:** locationID: M2; continent: Europe; country: Spain; countryCode: ES; stateProvince: Extremadura; county: Cáceres; locality: Fuente del Frances; verbatimElevation: 320.72; decimalLatitude: 39.828; decimalLongitude: -6.03249; geodeticDatum: WGS84; **Event:** eventID: F; samplingProtocol: Pitfall
- **Type status:** Other material. **Occurrence:** individualCount: 1; sex: male; **Location:** locationID: M2; continent: Europe; country: Spain; countryCode: ES; stateProvince: Extremadura; county: Cáceres; locality: Fuente del Frances; verbatimElevation: 320.72; decimalLatitude: 39.828; decimalLongitude: -6.03249; geodeticDatum: WGS84; **Event:** eventID: G; samplingProtocol: Pitfall
- **Type status:** Other material. **Occurrence:** individualCount: 2; sex: male; **Location:** locationID: M2; continent: Europe; country: Spain; countryCode: ES; stateProvince: Extremadura; county: Cáceres; locality: Fuente del Frances; verbatimElevation: 320.72; decimalLatitude: 39.828; decimalLongitude: -6.03249; geodeticDatum: WGS84; **Event:** eventID: J; samplingProtocol: Pitfall
- **Type status:** Other material. **Occurrence:** individualCount: 4; sex: male; **Location:** locationID: M2; continent: Europe; country: Spain; countryCode: ES; stateProvince: Extremadura; county: Cáceres; locality: Fuente del Frances; verbatimElevation: 320.72; decimalLatitude: 39.828; decimalLongitude: -6.03249; geodeticDatum: WGS84; **Event:** eventID: L; samplingProtocol: Pitfall
- **Type status:** Other material. **Occurrence:** individualCount: 2; sex: male; **Location:** locationID: S1; continent: Europe; country: Spain; countryCode: ES; stateProvince: Andalucía; county: Granada; locality: Soportujar; verbatimElevation: 1786.57; decimalLatitude: 36.96151; decimalLongitude: -3.41881; geodeticDatum: WGS84; **Event:** eventID: K; samplingProtocol: Pitfall
- **Type status:** Other material. **Occurrence:** individualCount: 1; sex: male; **Location:** locationID: S2; continent: Europe; country: Spain; countryCode: ES; stateProvince: Andalucía; county: Granada; locality: Camarate; verbatimElevation: 1713.96; decimalLatitude: 37.18377; decimalLongitude: -3.26282; geodeticDatum: WGS84; **Event:** eventID: A; samplingProtocol: Pitfall
- **Type status:** Other material. **Occurrence:** individualCount: 1; sex: male; **Location:** locationID: S2; continent: Europe; country: Spain; countryCode: ES; stateProvince: Andalucía; county: Granada; locality: Camarate; verbatimElevation: 1713.96; decimalLatitude: 37.18377; decimalLongitude: -3.26282; geodeticDatum: WGS84; **Event:** eventID: C; samplingProtocol: Pitfall
- **Type status:** Other material. **Occurrence:** individualCount: 1; sex: male; **Location:** locationID: S2; continent: Europe; country: Spain; countryCode: ES; stateProvince: Andalucía; county: Granada; locality: Camarate; verbatimElevation: 1713.96; decimalLatitude: 37.18377; decimalLongitude: -3.26282; geodeticDatum: WGS84; **Event:** eventID: E; samplingProtocol: Pitfall
- **Type status:** Other material. **Occurrence:** individualCount: 2; sex: male; **Location:** locationID: S2; continent: Europe; country: Spain; countryCode: ES; stateProvince: Andalucía; county: Granada; locality: Camarate; verbatimElevation: 1713.96; decimalLatitude: 37.18377; decimalLongitude: -3.26282; geodeticDatum: WGS84; **Event:** eventID: F; samplingProtocol: Pitfall
- **Type status:** Other material. **Occurrence:** individualCount: 1; sex: male; **Location:** locationID: S2; continent: Europe; country: Spain; countryCode: ES; stateProvince: Andalucía; county: Granada; locality: Camarate; verbatimElevation: 1713.96; decimalLatitude: 37.18377; decimalLongitude: -3.26282; geodeticDatum: WGS84; **Event:** eventID: J; samplingProtocol: Pitfall
- **Type status:** Other material. **Occurrence:** individualCount: 1; sex: female; **Location:** locationID: S2; continent: Europe; country: Spain; countryCode: ES; stateProvince: Andalucía; county: Granada; locality: Camarate; verbatimElevation: 1713.96; decimalLatitude: 37.18377; decimalLongitude: -3.26282; geodeticDatum: WGS84; **Event:** eventID: K; samplingProtocol: Pitfall
- **Type status:** Other material. **Occurrence:** individualCount: 3; sex: male; **Location:** locationID: S2; continent: Europe; country: Spain; countryCode: ES; stateProvince: Andalucía; county: Granada; locality: Camarate; verbatimElevation: 1713.96; decimalLatitude: 37.18377; decimalLongitude: -3.26282; geodeticDatum: WGS84; **Event:** eventID: K; samplingProtocol: Pitfall
- **Type status:** Other material. **Occurrence:** individualCount: 4; sex: male; **Location:** locationID: S2; continent: Europe; country: Spain; countryCode: ES; stateProvince: Andalucía; county: Granada; locality: Camarate; verbatimElevation: 1713.96; decimalLatitude: 37.18377; decimalLongitude: -3.26282; geodeticDatum: WGS84; **Event:** eventID: L; samplingProtocol: Pitfall

##### Distribution

Western Mediterranean

#### Zodarion alacre

(Simon, 1870)

##### Materials

- **Type status:** Other material. **Occurrence:** individualCount: 1; sex: male; **Location:** locationID: C1; continent: Europe; country: Spain; countryCode: ES; stateProvince: Castilla-La Mancha; county: Ciudad Real; locality: Valle Brezoso; verbatimElevation: 756.56; decimalLatitude: 39.35663; decimalLongitude: -4.35912; geodeticDatum: WGS84; **Event:** eventID: B; samplingProtocol: Pitfall
- **Type status:** Other material. **Occurrence:** individualCount: 1; sex: male; **Location:** locationID: C1; continent: Europe; country: Spain; countryCode: ES; stateProvince: Castilla-La Mancha; county: Ciudad Real; locality: Valle Brezoso; verbatimElevation: 756.56; decimalLatitude: 39.35663; decimalLongitude: -4.35912; geodeticDatum: WGS84; **Event:** eventID: D; samplingProtocol: Pitfall
- **Type status:** Other material. **Occurrence:** individualCount: 1; sex: male; **Location:** locationID: C1; continent: Europe; country: Spain; countryCode: ES; stateProvince: Castilla-La Mancha; county: Ciudad Real; locality: Valle Brezoso; verbatimElevation: 756.56; decimalLatitude: 39.35663; decimalLongitude: -4.35912; geodeticDatum: WGS84; **Event:** eventID: E; samplingProtocol: Pitfall
- **Type status:** Other material. **Occurrence:** individualCount: 3; sex: male; **Location:** locationID: C1; continent: Europe; country: Spain; countryCode: ES; stateProvince: Castilla-La Mancha; county: Ciudad Real; locality: Valle Brezoso; verbatimElevation: 756.56; decimalLatitude: 39.35663; decimalLongitude: -4.35912; geodeticDatum: WGS84; **Event:** eventID: H; samplingProtocol: Pitfall
- **Type status:** Other material. **Occurrence:** individualCount: 1; sex: male; **Location:** locationID: C1; continent: Europe; country: Spain; countryCode: ES; stateProvince: Castilla-La Mancha; county: Ciudad Real; locality: Valle Brezoso; verbatimElevation: 756.56; decimalLatitude: 39.35663; decimalLongitude: -4.35912; geodeticDatum: WGS84; **Event:** eventID: J; samplingProtocol: Pitfall
- **Type status:** Other material. **Occurrence:** individualCount: 1; sex: female; **Location:** locationID: C2; continent: Europe; country: Spain; countryCode: ES; stateProvince: Castilla-La Mancha; county: Ciudad Real; locality: Valle Brezoso; verbatimElevation: 739.31; decimalLatitude: 39.35159; decimalLongitude: -4.3589; geodeticDatum: WGS84; **Event:** eventID: A; samplingProtocol: Pitfall
- **Type status:** Other material. **Occurrence:** individualCount: 1; sex: male; **Location:** locationID: C2; continent: Europe; country: Spain; countryCode: ES; stateProvince: Castilla-La Mancha; county: Ciudad Real; locality: Valle Brezoso; verbatimElevation: 739.31; decimalLatitude: 39.35159; decimalLongitude: -4.3589; geodeticDatum: WGS84; **Event:** eventID: A; samplingProtocol: Pitfall
- **Type status:** Other material. **Occurrence:** individualCount: 1; sex: male; **Location:** locationID: C2; continent: Europe; country: Spain; countryCode: ES; stateProvince: Castilla-La Mancha; county: Ciudad Real; locality: Valle Brezoso; verbatimElevation: 739.31; decimalLatitude: 39.35159; decimalLongitude: -4.3589; geodeticDatum: WGS84; **Event:** eventID: E; samplingProtocol: Pitfall
- **Type status:** Other material. **Occurrence:** individualCount: 2; sex: male; **Location:** locationID: C2; continent: Europe; country: Spain; countryCode: ES; stateProvince: Castilla-La Mancha; county: Ciudad Real; locality: Valle Brezoso; verbatimElevation: 739.31; decimalLatitude: 39.35159; decimalLongitude: -4.3589; geodeticDatum: WGS84; **Event:** eventID: F; samplingProtocol: Pitfall
- **Type status:** Other material. **Occurrence:** individualCount: 3; sex: male; **Location:** locationID: C2; continent: Europe; country: Spain; countryCode: ES; stateProvince: Castilla-La Mancha; county: Ciudad Real; locality: Valle Brezoso; verbatimElevation: 739.31; decimalLatitude: 39.35159; decimalLongitude: -4.3589; geodeticDatum: WGS84; **Event:** eventID: G; samplingProtocol: Pitfall
- **Type status:** Other material. **Occurrence:** individualCount: 2; sex: male; **Location:** locationID: C2; continent: Europe; country: Spain; countryCode: ES; stateProvince: Castilla-La Mancha; county: Ciudad Real; locality: Valle Brezoso; verbatimElevation: 739.31; decimalLatitude: 39.35159; decimalLongitude: -4.3589; geodeticDatum: WGS84; **Event:** eventID: H; samplingProtocol: Pitfall
- **Type status:** Other material. **Occurrence:** individualCount: 1; sex: male; **Location:** locationID: C2; continent: Europe; country: Spain; countryCode: ES; stateProvince: Castilla-La Mancha; county: Ciudad Real; locality: Valle Brezoso; verbatimElevation: 739.31; decimalLatitude: 39.35159; decimalLongitude: -4.3589; geodeticDatum: WGS84; **Event:** eventID: I; samplingProtocol: Pitfall
- **Type status:** Other material. **Occurrence:** individualCount: 2; sex: male; **Location:** locationID: C2; continent: Europe; country: Spain; countryCode: ES; stateProvince: Castilla-La Mancha; county: Ciudad Real; locality: Valle Brezoso; verbatimElevation: 739.31; decimalLatitude: 39.35159; decimalLongitude: -4.3589; geodeticDatum: WGS84; **Event:** eventID: J; samplingProtocol: Pitfall
- **Type status:** Other material. **Occurrence:** individualCount: 2; sex: male; **Location:** locationID: C2; continent: Europe; country: Spain; countryCode: ES; stateProvince: Castilla-La Mancha; county: Ciudad Real; locality: Valle Brezoso; verbatimElevation: 739.31; decimalLatitude: 39.35159; decimalLongitude: -4.3589; geodeticDatum: WGS84; **Event:** eventID: K; samplingProtocol: Pitfall
- **Type status:** Other material. **Occurrence:** individualCount: 1; sex: female; **Location:** locationID: C2; continent: Europe; country: Spain; countryCode: ES; stateProvince: Castilla-La Mancha; county: Ciudad Real; locality: Valle Brezoso; verbatimElevation: 739.31; decimalLatitude: 39.35159; decimalLongitude: -4.3589; geodeticDatum: WGS84; **Event:** eventID: L; samplingProtocol: Pitfall
- **Type status:** Other material. **Occurrence:** individualCount: 4; sex: male; **Location:** locationID: C2; continent: Europe; country: Spain; countryCode: ES; stateProvince: Castilla-La Mancha; county: Ciudad Real; locality: Valle Brezoso; verbatimElevation: 739.31; decimalLatitude: 39.35159; decimalLongitude: -4.3589; geodeticDatum: WGS84; **Event:** eventID: L; samplingProtocol: Pitfall
- **Type status:** Other material. **Occurrence:** individualCount: 2; sex: male; **Location:** locationID: C3; continent: Europe; country: Spain; countryCode: ES; stateProvince: Castilla-La Mancha; county: Ciudad Real; locality: La Quesera; verbatimElevation: 767.55; decimalLatitude: 39.36177; decimalLongitude: -4.41733; geodeticDatum: WGS84; **Event:** eventID: A; samplingProtocol: Pitfall
- **Type status:** Other material. **Occurrence:** individualCount: 3; sex: male; **Location:** locationID: C3; continent: Europe; country: Spain; countryCode: ES; stateProvince: Castilla-La Mancha; county: Ciudad Real; locality: La Quesera; verbatimElevation: 767.55; decimalLatitude: 39.36177; decimalLongitude: -4.41733; geodeticDatum: WGS84; **Event:** eventID: C; samplingProtocol: Pitfall
- **Type status:** Other material. **Occurrence:** individualCount: 1; sex: male; **Location:** locationID: C3; continent: Europe; country: Spain; countryCode: ES; stateProvince: Castilla-La Mancha; county: Ciudad Real; locality: La Quesera; verbatimElevation: 767.55; decimalLatitude: 39.36177; decimalLongitude: -4.41733; geodeticDatum: WGS84; **Event:** eventID: G; samplingProtocol: Pitfall
- **Type status:** Other material. **Occurrence:** individualCount: 1; sex: male; **Location:** locationID: C4; continent: Europe; country: Spain; countryCode: ES; stateProvince: Castilla-La Mancha; county: Ciudad Real; locality: La Quesera; verbatimElevation: 772.3; decimalLatitude: 39.36337; decimalLongitude: -4.41704; geodeticDatum: WGS84; **Event:** eventID: A; samplingProtocol: Pitfall
- **Type status:** Other material. **Occurrence:** individualCount: 1; sex: male; **Location:** locationID: C4; continent: Europe; country: Spain; countryCode: ES; stateProvince: Castilla-La Mancha; county: Ciudad Real; locality: La Quesera; verbatimElevation: 772.3; decimalLatitude: 39.36337; decimalLongitude: -4.41704; geodeticDatum: WGS84; **Event:** eventID: C; samplingProtocol: Pitfall
- **Type status:** Other material. **Occurrence:** individualCount: 1; sex: male; **Location:** locationID: C4; continent: Europe; country: Spain; countryCode: ES; stateProvince: Castilla-La Mancha; county: Ciudad Real; locality: La Quesera; verbatimElevation: 772.3; decimalLatitude: 39.36337; decimalLongitude: -4.41704; geodeticDatum: WGS84; **Event:** eventID: E; samplingProtocol: Pitfall
- **Type status:** Other material. **Occurrence:** individualCount: 1; sex: female; **Location:** locationID: C4; continent: Europe; country: Spain; countryCode: ES; stateProvince: Castilla-La Mancha; county: Ciudad Real; locality: La Quesera; verbatimElevation: 772.3; decimalLatitude: 39.36337; decimalLongitude: -4.41704; geodeticDatum: WGS84; **Event:** eventID: F; samplingProtocol: Pitfall
- **Type status:** Other material. **Occurrence:** individualCount: 1; sex: male; **Location:** locationID: C4; continent: Europe; country: Spain; countryCode: ES; stateProvince: Castilla-La Mancha; county: Ciudad Real; locality: La Quesera; verbatimElevation: 772.3; decimalLatitude: 39.36337; decimalLongitude: -4.41704; geodeticDatum: WGS84; **Event:** eventID: H; samplingProtocol: Pitfall
- **Type status:** Other material. **Occurrence:** individualCount: 1; sex: male; **Location:** locationID: C4; continent: Europe; country: Spain; countryCode: ES; stateProvince: Castilla-La Mancha; county: Ciudad Real; locality: La Quesera; verbatimElevation: 772.3; decimalLatitude: 39.36337; decimalLongitude: -4.41704; geodeticDatum: WGS84; **Event:** eventID: J; samplingProtocol: Pitfall
- **Type status:** Other material. **Occurrence:** individualCount: 1; sex: male; **Location:** locationID: C4; continent: Europe; country: Spain; countryCode: ES; stateProvince: Castilla-La Mancha; county: Ciudad Real; locality: La Quesera; verbatimElevation: 772.3; decimalLatitude: 39.36337; decimalLongitude: -4.41704; geodeticDatum: WGS84; **Event:** eventID: L; samplingProtocol: Pitfall
- **Type status:** Other material. **Occurrence:** individualCount: 1; sex: female; **Location:** locationID: M1; continent: Europe; country: Spain; countryCode: ES; stateProvince: Extremadura; county: Cáceres; locality: Peña Falcón; verbatimElevation: 320.6; decimalLatitude: 39.83296; decimalLongitude: -6.0641; geodeticDatum: WGS84; **Event:** eventID: E; samplingProtocol: Pitfall

##### Distribution

Iberian Peninsula

#### Zodarion beticum

Denis, 1957

##### Materials

- **Type status:** Other material. **Occurrence:** individualCount: 45; sex: male; **Location:** locationID: S2; continent: Europe; country: Spain; countryCode: ES; stateProvince: Andalucía; county: Granada; locality: Camarate; verbatimElevation: 1713.96; decimalLatitude: 37.18377; decimalLongitude: -3.26282; geodeticDatum: WGS84; **Event:** eventID: A; samplingProtocol: Pitfall
- **Type status:** Other material. **Occurrence:** individualCount: 1; sex: female; **Location:** locationID: S2; continent: Europe; country: Spain; countryCode: ES; stateProvince: Andalucía; county: Granada; locality: Camarate; verbatimElevation: 1713.96; decimalLatitude: 37.18377; decimalLongitude: -3.26282; geodeticDatum: WGS84; **Event:** eventID: B; samplingProtocol: Pitfall
- **Type status:** Other material. **Occurrence:** individualCount: 21; sex: male; **Location:** locationID: S2; continent: Europe; country: Spain; countryCode: ES; stateProvince: Andalucía; county: Granada; locality: Camarate; verbatimElevation: 1713.96; decimalLatitude: 37.18377; decimalLongitude: -3.26282; geodeticDatum: WGS84; **Event:** eventID: B; samplingProtocol: Pitfall
- **Type status:** Other material. **Occurrence:** individualCount: 1; sex: female; **Location:** locationID: S2; continent: Europe; country: Spain; countryCode: ES; stateProvince: Andalucía; county: Granada; locality: Camarate; verbatimElevation: 1713.96; decimalLatitude: 37.18377; decimalLongitude: -3.26282; geodeticDatum: WGS84; **Event:** eventID: C; samplingProtocol: Pitfall
- **Type status:** Other material. **Occurrence:** individualCount: 4; sex: male; **Location:** locationID: S2; continent: Europe; country: Spain; countryCode: ES; stateProvince: Andalucía; county: Granada; locality: Camarate; verbatimElevation: 1713.96; decimalLatitude: 37.18377; decimalLongitude: -3.26282; geodeticDatum: WGS84; **Event:** eventID: C; samplingProtocol: Pitfall
- **Type status:** Other material. **Occurrence:** individualCount: 3; sex: male; **Location:** locationID: S2; continent: Europe; country: Spain; countryCode: ES; stateProvince: Andalucía; county: Granada; locality: Camarate; verbatimElevation: 1713.96; decimalLatitude: 37.18377; decimalLongitude: -3.26282; geodeticDatum: WGS84; **Event:** eventID: D; samplingProtocol: Pitfall
- **Type status:** Other material. **Occurrence:** individualCount: 10; sex: male; **Location:** locationID: S2; continent: Europe; country: Spain; countryCode: ES; stateProvince: Andalucía; county: Granada; locality: Camarate; verbatimElevation: 1713.96; decimalLatitude: 37.18377; decimalLongitude: -3.26282; geodeticDatum: WGS84; **Event:** eventID: E; samplingProtocol: Pitfall
- **Type status:** Other material. **Occurrence:** individualCount: 3; sex: male; **Location:** locationID: S2; continent: Europe; country: Spain; countryCode: ES; stateProvince: Andalucía; county: Granada; locality: Camarate; verbatimElevation: 1713.96; decimalLatitude: 37.18377; decimalLongitude: -3.26282; geodeticDatum: WGS84; **Event:** eventID: G; samplingProtocol: Pitfall
- **Type status:** Other material. **Occurrence:** individualCount: 1; sex: female; **Location:** locationID: S2; continent: Europe; country: Spain; countryCode: ES; stateProvince: Andalucía; county: Granada; locality: Camarate; verbatimElevation: 1713.96; decimalLatitude: 37.18377; decimalLongitude: -3.26282; geodeticDatum: WGS84; **Event:** eventID: H; samplingProtocol: Pitfall
- **Type status:** Other material. **Occurrence:** individualCount: 2; sex: male; **Location:** locationID: S2; continent: Europe; country: Spain; countryCode: ES; stateProvince: Andalucía; county: Granada; locality: Camarate; verbatimElevation: 1713.96; decimalLatitude: 37.18377; decimalLongitude: -3.26282; geodeticDatum: WGS84; **Event:** eventID: I; samplingProtocol: Pitfall
- **Type status:** Other material. **Occurrence:** individualCount: 5; sex: male; **Location:** locationID: S2; continent: Europe; country: Spain; countryCode: ES; stateProvince: Andalucía; county: Granada; locality: Camarate; verbatimElevation: 1713.96; decimalLatitude: 37.18377; decimalLongitude: -3.26282; geodeticDatum: WGS84; **Event:** eventID: J; samplingProtocol: Pitfall
- **Type status:** Other material. **Occurrence:** individualCount: 3; sex: male; **Location:** locationID: S2; continent: Europe; country: Spain; countryCode: ES; stateProvince: Andalucía; county: Granada; locality: Camarate; verbatimElevation: 1713.96; decimalLatitude: 37.18377; decimalLongitude: -3.26282; geodeticDatum: WGS84; **Event:** eventID: K; samplingProtocol: Pitfall

##### Distribution

Spain

#### Zodarion fuscum

(Simon, 1870)

##### Materials

- **Type status:** Other material. **Occurrence:** individualCount: 1; sex: female; **Location:** locationID: P1; continent: Europe; country: Spain; countryCode: ES; stateProvince: Castilla y León; county: León; locality: Monte Robledo; verbatimElevation: 1071.58; decimalLatitude: 43.1445; decimalLongitude: -4.92675; geodeticDatum: WGS84; **Event:** eventID: 2; samplingProtocol: Ground; eventTime: Day
- **Type status:** Other material. **Occurrence:** individualCount: 1; sex: male; **Location:** locationID: P2; continent: Europe; country: Spain; countryCode: ES; stateProvince: Castilla y León; county: León; locality: Joyoguelas; verbatimElevation: 763.98; decimalLatitude: 43.17771; decimalLongitude: -4.90579; geodeticDatum: WGS84; **Event:** eventID: A; samplingProtocol: Pitfall
- **Type status:** Other material. **Occurrence:** individualCount: 1; sex: male; **Location:** locationID: P2; continent: Europe; country: Spain; countryCode: ES; stateProvince: Castilla y León; county: León; locality: Joyoguelas; verbatimElevation: 763.98; decimalLatitude: 43.17771; decimalLongitude: -4.90579; geodeticDatum: WGS84; **Event:** eventID: B; samplingProtocol: Pitfall
- **Type status:** Other material. **Occurrence:** individualCount: 1; sex: male; **Location:** locationID: P2; continent: Europe; country: Spain; countryCode: ES; stateProvince: Castilla y León; county: León; locality: Joyoguelas; verbatimElevation: 763.98; decimalLatitude: 43.17771; decimalLongitude: -4.90579; geodeticDatum: WGS84; **Event:** eventID: C; samplingProtocol: Pitfall
- **Type status:** Other material. **Occurrence:** individualCount: 4; sex: male; **Location:** locationID: P4; continent: Europe; country: Spain; countryCode: ES; stateProvince: Castilla y León; county: León; locality: El Canto; verbatimElevation: 943.48; decimalLatitude: 43.17227; decimalLongitude: -4.90857; geodeticDatum: WGS84; **Event:** eventID: C; samplingProtocol: Pitfall
- **Type status:** Other material. **Occurrence:** individualCount: 1; sex: male; **Location:** locationID: P4; continent: Europe; country: Spain; countryCode: ES; stateProvince: Castilla y León; county: León; locality: El Canto; verbatimElevation: 943.48; decimalLatitude: 43.17227; decimalLongitude: -4.90857; geodeticDatum: WGS84; **Event:** eventID: D; samplingProtocol: Pitfall
- **Type status:** Other material. **Occurrence:** individualCount: 1; sex: male; **Location:** locationID: P4; continent: Europe; country: Spain; countryCode: ES; stateProvince: Castilla y León; county: León; locality: El Canto; verbatimElevation: 943.48; decimalLatitude: 43.17227; decimalLongitude: -4.90857; geodeticDatum: WGS84; **Event:** eventID: E; samplingProtocol: Pitfall
- **Type status:** Other material. **Occurrence:** individualCount: 8; sex: male; **Location:** locationID: P4; continent: Europe; country: Spain; countryCode: ES; stateProvince: Castilla y León; county: León; locality: El Canto; verbatimElevation: 943.48; decimalLatitude: 43.17227; decimalLongitude: -4.90857; geodeticDatum: WGS84; **Event:** eventID: F; samplingProtocol: Pitfall
- **Type status:** Other material. **Occurrence:** individualCount: 1; sex: male; **Location:** locationID: P4; continent: Europe; country: Spain; countryCode: ES; stateProvince: Castilla y León; county: León; locality: El Canto; verbatimElevation: 943.48; decimalLatitude: 43.17227; decimalLongitude: -4.90857; geodeticDatum: WGS84; **Event:** eventID: G; samplingProtocol: Pitfall
- **Type status:** Other material. **Occurrence:** individualCount: 1; sex: male; **Location:** locationID: P4; continent: Europe; country: Spain; countryCode: ES; stateProvince: Castilla y León; county: León; locality: El Canto; verbatimElevation: 943.48; decimalLatitude: 43.17227; decimalLongitude: -4.90857; geodeticDatum: WGS84; **Event:** eventID: H; samplingProtocol: Pitfall
- **Type status:** Other material. **Occurrence:** individualCount: 2; sex: male; **Location:** locationID: P4; continent: Europe; country: Spain; countryCode: ES; stateProvince: Castilla y León; county: León; locality: El Canto; verbatimElevation: 943.48; decimalLatitude: 43.17227; decimalLongitude: -4.90857; geodeticDatum: WGS84; **Event:** eventID: I; samplingProtocol: Pitfall

##### Distribution

Britain, Iberian Peninsula, France

#### Zodarion marginiceps

Simon, 1914

##### Materials

- **Type status:** Other material. **Occurrence:** individualCount: 1; sex: female; **Location:** locationID: A1; continent: Europe; country: Spain; countryCode: ES; stateProvince: Catalonia; county: Lleida; locality: Sola de Boi; verbatimElevation: 1759.8; decimalLatitude: 42.54958; decimalLongitude: 0.87254; geodeticDatum: WGS84; **Event:** eventID: A; samplingProtocol: Pitfall
- **Type status:** Other material. **Occurrence:** individualCount: 2; sex: male; **Location:** locationID: A1; continent: Europe; country: Spain; countryCode: ES; stateProvince: Catalonia; county: Lleida; locality: Sola de Boi; verbatimElevation: 1759.8; decimalLatitude: 42.54958; decimalLongitude: 0.87254; geodeticDatum: WGS84; **Event:** eventID: C; samplingProtocol: Pitfall
- **Type status:** Other material. **Occurrence:** individualCount: 1; sex: female; **Location:** locationID: A1; continent: Europe; country: Spain; countryCode: ES; stateProvince: Catalonia; county: Lleida; locality: Sola de Boi; verbatimElevation: 1759.8; decimalLatitude: 42.54958; decimalLongitude: 0.87254; geodeticDatum: WGS84; **Event:** eventID: E; samplingProtocol: Pitfall
- **Type status:** Other material. **Occurrence:** individualCount: 2; sex: male; **Location:** locationID: A1; continent: Europe; country: Spain; countryCode: ES; stateProvince: Catalonia; county: Lleida; locality: Sola de Boi; verbatimElevation: 1759.8; decimalLatitude: 42.54958; decimalLongitude: 0.87254; geodeticDatum: WGS84; **Event:** eventID: E; samplingProtocol: Pitfall
- **Type status:** Other material. **Occurrence:** individualCount: 1; sex: male; **Location:** locationID: A1; continent: Europe; country: Spain; countryCode: ES; stateProvince: Catalonia; county: Lleida; locality: Sola de Boi; verbatimElevation: 1759.8; decimalLatitude: 42.54958; decimalLongitude: 0.87254; geodeticDatum: WGS84; **Event:** eventID: F; samplingProtocol: Pitfall
- **Type status:** Other material. **Occurrence:** individualCount: 4; sex: male; **Location:** locationID: A1; continent: Europe; country: Spain; countryCode: ES; stateProvince: Catalonia; county: Lleida; locality: Sola de Boi; verbatimElevation: 1759.8; decimalLatitude: 42.54958; decimalLongitude: 0.87254; geodeticDatum: WGS84; **Event:** eventID: G; samplingProtocol: Pitfall
- **Type status:** Other material. **Occurrence:** individualCount: 3; sex: male; **Location:** locationID: A1; continent: Europe; country: Spain; countryCode: ES; stateProvince: Catalonia; county: Lleida; locality: Sola de Boi; verbatimElevation: 1759.8; decimalLatitude: 42.54958; decimalLongitude: 0.87254; geodeticDatum: WGS84; **Event:** eventID: H; samplingProtocol: Pitfall
- **Type status:** Other material. **Occurrence:** individualCount: 2; sex: male; **Location:** locationID: A1; continent: Europe; country: Spain; countryCode: ES; stateProvince: Catalonia; county: Lleida; locality: Sola de Boi; verbatimElevation: 1759.8; decimalLatitude: 42.54958; decimalLongitude: 0.87254; geodeticDatum: WGS84; **Event:** eventID: L; samplingProtocol: Pitfall
- **Type status:** Other material. **Occurrence:** individualCount: 1; sex: male; **Location:** locationID: A2; continent: Europe; country: Spain; countryCode: ES; stateProvince: Catalonia; county: Lleida; locality: Sola de Boi; verbatimElevation: 1738.7; decimalLatitude: 42.54913; decimalLongitude: 0.87137; geodeticDatum: WGS84; **Event:** eventID: A; samplingProtocol: Pitfall
- **Type status:** Other material. **Occurrence:** individualCount: 3; sex: male; **Location:** locationID: A2; continent: Europe; country: Spain; countryCode: ES; stateProvince: Catalonia; county: Lleida; locality: Sola de Boi; verbatimElevation: 1738.7; decimalLatitude: 42.54913; decimalLongitude: 0.87137; geodeticDatum: WGS84; **Event:** eventID: E; samplingProtocol: Pitfall
- **Type status:** Other material. **Occurrence:** individualCount: 1; sex: male; **Location:** locationID: A2; continent: Europe; country: Spain; countryCode: ES; stateProvince: Catalonia; county: Lleida; locality: Sola de Boi; verbatimElevation: 1738.7; decimalLatitude: 42.54913; decimalLongitude: 0.87137; geodeticDatum: WGS84; **Event:** eventID: H; samplingProtocol: Pitfall
- **Type status:** Other material. **Occurrence:** individualCount: 5; sex: male; **Location:** locationID: A2; continent: Europe; country: Spain; countryCode: ES; stateProvince: Catalonia; county: Lleida; locality: Sola de Boi; verbatimElevation: 1738.7; decimalLatitude: 42.54913; decimalLongitude: 0.87137; geodeticDatum: WGS84; **Event:** eventID: I; samplingProtocol: Pitfall
- **Type status:** Other material. **Occurrence:** individualCount: 1; sex: female; **Location:** locationID: A2; continent: Europe; country: Spain; countryCode: ES; stateProvince: Catalonia; county: Lleida; locality: Sola de Boi; verbatimElevation: 1738.7; decimalLatitude: 42.54913; decimalLongitude: 0.87137; geodeticDatum: WGS84; **Event:** eventID: J; samplingProtocol: Pitfall
- **Type status:** Other material. **Occurrence:** individualCount: 10; sex: male; **Location:** locationID: A2; continent: Europe; country: Spain; countryCode: ES; stateProvince: Catalonia; county: Lleida; locality: Sola de Boi; verbatimElevation: 1738.7; decimalLatitude: 42.54913; decimalLongitude: 0.87137; geodeticDatum: WGS84; **Event:** eventID: J; samplingProtocol: Pitfall
- **Type status:** Other material. **Occurrence:** individualCount: 1; sex: male; **Location:** locationID: A2; continent: Europe; country: Spain; countryCode: ES; stateProvince: Catalonia; county: Lleida; locality: Sola de Boi; verbatimElevation: 1738.7; decimalLatitude: 42.54913; decimalLongitude: 0.87137; geodeticDatum: WGS84; **Event:** eventID: L; samplingProtocol: Pitfall
- **Type status:** Other material. **Occurrence:** individualCount: 1; sex: female; **Location:** locationID: O2; continent: Europe; country: Spain; countryCode: ES; stateProvince: Aragón; county: Huesca; locality: Rebilla; verbatimElevation: 1158.13; decimalLatitude: 42.59427; decimalLongitude: 0.1529; geodeticDatum: WGS84; **Event:** eventID: 2; samplingProtocol: Aerial; eventTime: Night
- **Type status:** Other material. **Occurrence:** individualCount: 1; sex: male; **Location:** locationID: O2; continent: Europe; country: Spain; countryCode: ES; stateProvince: Aragón; county: Huesca; locality: Rebilla; verbatimElevation: 1158.13; decimalLatitude: 42.59427; decimalLongitude: 0.1529; geodeticDatum: WGS84; **Event:** eventID: 2; samplingProtocol: Aerial; eventTime: Night
- **Type status:** Other material. **Occurrence:** individualCount: 1; sex: female; **Location:** locationID: O2; continent: Europe; country: Spain; countryCode: ES; stateProvince: Aragón; county: Huesca; locality: Rebilla; verbatimElevation: 1158.13; decimalLatitude: 42.59427; decimalLongitude: 0.1529; geodeticDatum: WGS84; **Event:** eventID: L; samplingProtocol: Pitfall
- **Type status:** Other material. **Occurrence:** individualCount: 1; sex: male; **Location:** locationID: O2; continent: Europe; country: Spain; countryCode: ES; stateProvince: Aragón; county: Huesca; locality: Rebilla; verbatimElevation: 1158.13; decimalLatitude: 42.59427; decimalLongitude: 0.1529; geodeticDatum: WGS84; **Event:** eventID: L; samplingProtocol: Pitfall

##### Distribution

Spain, France

#### Zodarion modestum

(Simon, 1870)

##### Materials

- **Type status:** Other material. **Occurrence:** individualCount: 1; sex: female; **Location:** locationID: S1; continent: Europe; country: Spain; countryCode: ES; stateProvince: Andalucía; county: Granada; locality: Soportujar; verbatimElevation: 1786.57; decimalLatitude: 36.96151; decimalLongitude: -3.41881; geodeticDatum: WGS84; **Event:** eventID: G; samplingProtocol: Pitfall
- **Type status:** Other material. **Occurrence:** individualCount: 2; sex: male; **Location:** locationID: S1; continent: Europe; country: Spain; countryCode: ES; stateProvince: Andalucía; county: Granada; locality: Soportujar; verbatimElevation: 1786.57; decimalLatitude: 36.96151; decimalLongitude: -3.41881; geodeticDatum: WGS84; **Event:** eventID: G; samplingProtocol: Pitfall
- **Type status:** Other material. **Occurrence:** individualCount: 1; sex: male; **Location:** locationID: S1; continent: Europe; country: Spain; countryCode: ES; stateProvince: Andalucía; county: Granada; locality: Soportujar; verbatimElevation: 1786.57; decimalLatitude: 36.96151; decimalLongitude: -3.41881; geodeticDatum: WGS84; **Event:** eventID: I; samplingProtocol: Pitfall
- **Type status:** Other material. **Occurrence:** individualCount: 1; sex: male; **Location:** locationID: S1; continent: Europe; country: Spain; countryCode: ES; stateProvince: Andalucía; county: Granada; locality: Soportujar; verbatimElevation: 1786.57; decimalLatitude: 36.96151; decimalLongitude: -3.41881; geodeticDatum: WGS84; **Event:** eventID: J; samplingProtocol: Pitfall
- **Type status:** Other material. **Occurrence:** individualCount: 1; sex: male; **Location:** locationID: S1; continent: Europe; country: Spain; countryCode: ES; stateProvince: Andalucía; county: Granada; locality: Soportujar; verbatimElevation: 1786.57; decimalLatitude: 36.96151; decimalLongitude: -3.41881; geodeticDatum: WGS84; **Event:** eventID: K; samplingProtocol: Pitfall
- **Type status:** Other material. **Occurrence:** individualCount: 1; sex: female; **Location:** locationID: S1; continent: Europe; country: Spain; countryCode: ES; stateProvince: Andalucía; county: Granada; locality: Soportujar; verbatimElevation: 1786.57; decimalLatitude: 36.96151; decimalLongitude: -3.41881; geodeticDatum: WGS84; **Event:** eventID: L; samplingProtocol: Pitfall
- **Type status:** Other material. **Occurrence:** individualCount: 3; sex: male; **Location:** locationID: S1; continent: Europe; country: Spain; countryCode: ES; stateProvince: Andalucía; county: Granada; locality: Soportujar; verbatimElevation: 1786.57; decimalLatitude: 36.96151; decimalLongitude: -3.41881; geodeticDatum: WGS84; **Event:** eventID: L; samplingProtocol: Pitfall
- **Type status:** Other material. **Occurrence:** individualCount: 1; sex: male; **Location:** locationID: S2; continent: Europe; country: Spain; countryCode: ES; stateProvince: Andalucía; county: Granada; locality: Camarate; verbatimElevation: 1713.96; decimalLatitude: 37.18377; decimalLongitude: -3.26282; geodeticDatum: WGS84; **Event:** eventID: A; samplingProtocol: Pitfall
- **Type status:** Other material. **Occurrence:** individualCount: 1; sex: female; **Location:** locationID: S2; continent: Europe; country: Spain; countryCode: ES; stateProvince: Andalucía; county: Granada; locality: Camarate; verbatimElevation: 1713.96; decimalLatitude: 37.18377; decimalLongitude: -3.26282; geodeticDatum: WGS84; **Event:** eventID: D; samplingProtocol: Pitfall
- **Type status:** Other material. **Occurrence:** individualCount: 1; sex: male; **Location:** locationID: S2; continent: Europe; country: Spain; countryCode: ES; stateProvince: Andalucía; county: Granada; locality: Camarate; verbatimElevation: 1713.96; decimalLatitude: 37.18377; decimalLongitude: -3.26282; geodeticDatum: WGS84; **Event:** eventID: D; samplingProtocol: Pitfall
- **Type status:** Other material. **Occurrence:** individualCount: 2; sex: male; **Location:** locationID: S2; continent: Europe; country: Spain; countryCode: ES; stateProvince: Andalucía; county: Granada; locality: Camarate; verbatimElevation: 1713.96; decimalLatitude: 37.18377; decimalLongitude: -3.26282; geodeticDatum: WGS84; **Event:** eventID: E; samplingProtocol: Pitfall
- **Type status:** Other material. **Occurrence:** individualCount: 1; sex: female; **Location:** locationID: S2; continent: Europe; country: Spain; countryCode: ES; stateProvince: Andalucía; county: Granada; locality: Camarate; verbatimElevation: 1713.96; decimalLatitude: 37.18377; decimalLongitude: -3.26282; geodeticDatum: WGS84; **Event:** eventID: H; samplingProtocol: Pitfall
- **Type status:** Other material. **Occurrence:** individualCount: 3; sex: male; **Location:** locationID: S2; continent: Europe; country: Spain; countryCode: ES; stateProvince: Andalucía; county: Granada; locality: Camarate; verbatimElevation: 1713.96; decimalLatitude: 37.18377; decimalLongitude: -3.26282; geodeticDatum: WGS84; **Event:** eventID: I; samplingProtocol: Pitfall
- **Type status:** Other material. **Occurrence:** individualCount: 3; sex: male; **Location:** locationID: S2; continent: Europe; country: Spain; countryCode: ES; stateProvince: Andalucía; county: Granada; locality: Camarate; verbatimElevation: 1713.96; decimalLatitude: 37.18377; decimalLongitude: -3.26282; geodeticDatum: WGS84; **Event:** eventID: J; samplingProtocol: Pitfall
- **Type status:** Other material. **Occurrence:** individualCount: 1; sex: male; **Location:** locationID: S2; continent: Europe; country: Spain; countryCode: ES; stateProvince: Andalucía; county: Granada; locality: Camarate; verbatimElevation: 1713.96; decimalLatitude: 37.18377; decimalLongitude: -3.26282; geodeticDatum: WGS84; **Event:** eventID: L; samplingProtocol: Pitfall

##### Distribution

Spain

#### Zodarion rudyi

Bosmans, 1994

##### Materials

- **Type status:** Other material. **Occurrence:** individualCount: 1; sex: female; **Location:** locationID: S1; continent: Europe; country: Spain; countryCode: ES; stateProvince: Andalucía; county: Granada; locality: Soportujar; verbatimElevation: 1786.57; decimalLatitude: 36.96151; decimalLongitude: -3.41881; geodeticDatum: WGS84; **Event:** eventID: F; samplingProtocol: Pitfall
- **Type status:** Other material. **Occurrence:** individualCount: 1; sex: male; **Location:** locationID: S1; continent: Europe; country: Spain; countryCode: ES; stateProvince: Andalucía; county: Granada; locality: Soportujar; verbatimElevation: 1786.57; decimalLatitude: 36.96151; decimalLongitude: -3.41881; geodeticDatum: WGS84; **Event:** eventID: F; samplingProtocol: Pitfall
- **Type status:** Other material. **Occurrence:** individualCount: 1; sex: female; **Location:** locationID: S1; continent: Europe; country: Spain; countryCode: ES; stateProvince: Andalucía; county: Granada; locality: Soportujar; verbatimElevation: 1786.57; decimalLatitude: 36.96151; decimalLongitude: -3.41881; geodeticDatum: WGS84; **Event:** eventID: G; samplingProtocol: Pitfall
- **Type status:** Other material. **Occurrence:** individualCount: 2; sex: male; **Location:** locationID: S1; continent: Europe; country: Spain; countryCode: ES; stateProvince: Andalucía; county: Granada; locality: Soportujar; verbatimElevation: 1786.57; decimalLatitude: 36.96151; decimalLongitude: -3.41881; geodeticDatum: WGS84; **Event:** eventID: G; samplingProtocol: Pitfall
- **Type status:** Other material. **Occurrence:** individualCount: 2; sex: male; **Location:** locationID: S1; continent: Europe; country: Spain; countryCode: ES; stateProvince: Andalucía; county: Granada; locality: Soportujar; verbatimElevation: 1786.57; decimalLatitude: 36.96151; decimalLongitude: -3.41881; geodeticDatum: WGS84; **Event:** eventID: H; samplingProtocol: Pitfall
- **Type status:** Other material. **Occurrence:** individualCount: 1; sex: male; **Location:** locationID: S1; continent: Europe; country: Spain; countryCode: ES; stateProvince: Andalucía; county: Granada; locality: Soportujar; verbatimElevation: 1786.57; decimalLatitude: 36.96151; decimalLongitude: -3.41881; geodeticDatum: WGS84; **Event:** eventID: J; samplingProtocol: Pitfall
- **Type status:** Other material. **Occurrence:** individualCount: 1; sex: female; **Location:** locationID: S1; continent: Europe; country: Spain; countryCode: ES; stateProvince: Andalucía; county: Granada; locality: Soportujar; verbatimElevation: 1786.57; decimalLatitude: 36.96151; decimalLongitude: -3.41881; geodeticDatum: WGS84; **Event:** eventID: K; samplingProtocol: Pitfall
- **Type status:** Other material. **Occurrence:** individualCount: 2; sex: male; **Location:** locationID: S1; continent: Europe; country: Spain; countryCode: ES; stateProvince: Andalucía; county: Granada; locality: Soportujar; verbatimElevation: 1786.57; decimalLatitude: 36.96151; decimalLongitude: -3.41881; geodeticDatum: WGS84; **Event:** eventID: K; samplingProtocol: Pitfall
- **Type status:** Other material. **Occurrence:** individualCount: 1; sex: female; **Location:** locationID: S1; continent: Europe; country: Spain; countryCode: ES; stateProvince: Andalucía; county: Granada; locality: Soportujar; verbatimElevation: 1786.57; decimalLatitude: 36.96151; decimalLongitude: -3.41881; geodeticDatum: WGS84; **Event:** eventID: L; samplingProtocol: Pitfall

##### Distribution

Iberian Peninsula

#### Zodarion styliferum

(Simon, 1870)

##### Materials

- **Type status:** Other material. **Occurrence:** individualCount: 4; sex: male; **Location:** locationID: C1; continent: Europe; country: Spain; countryCode: ES; stateProvince: Castilla-La Mancha; county: Ciudad Real; locality: Valle Brezoso; verbatimElevation: 756.56; decimalLatitude: 39.35663; decimalLongitude: -4.35912; geodeticDatum: WGS84; **Event:** eventID: D; samplingProtocol: Pitfall
- **Type status:** Other material. **Occurrence:** individualCount: 1; sex: male; **Location:** locationID: C1; continent: Europe; country: Spain; countryCode: ES; stateProvince: Castilla-La Mancha; county: Ciudad Real; locality: Valle Brezoso; verbatimElevation: 756.56; decimalLatitude: 39.35663; decimalLongitude: -4.35912; geodeticDatum: WGS84; **Event:** eventID: G; samplingProtocol: Pitfall
- **Type status:** Other material. **Occurrence:** individualCount: 1; sex: male; **Location:** locationID: C1; continent: Europe; country: Spain; countryCode: ES; stateProvince: Castilla-La Mancha; county: Ciudad Real; locality: Valle Brezoso; verbatimElevation: 756.56; decimalLatitude: 39.35663; decimalLongitude: -4.35912; geodeticDatum: WGS84; **Event:** eventID: H; samplingProtocol: Pitfall
- **Type status:** Other material. **Occurrence:** individualCount: 1; sex: male; **Location:** locationID: C2; continent: Europe; country: Spain; countryCode: ES; stateProvince: Castilla-La Mancha; county: Ciudad Real; locality: Valle Brezoso; verbatimElevation: 739.31; decimalLatitude: 39.35159; decimalLongitude: -4.3589; geodeticDatum: WGS84; **Event:** eventID: 4; samplingProtocol: Aerial; eventTime: Night
- **Type status:** Other material. **Occurrence:** individualCount: 1; sex: male; **Location:** locationID: C2; continent: Europe; country: Spain; countryCode: ES; stateProvince: Castilla-La Mancha; county: Ciudad Real; locality: Valle Brezoso; verbatimElevation: 739.31; decimalLatitude: 39.35159; decimalLongitude: -4.3589; geodeticDatum: WGS84; **Event:** eventID: C; samplingProtocol: Pitfall
- **Type status:** Other material. **Occurrence:** individualCount: 15; sex: male; **Location:** locationID: C2; continent: Europe; country: Spain; countryCode: ES; stateProvince: Castilla-La Mancha; county: Ciudad Real; locality: Valle Brezoso; verbatimElevation: 739.31; decimalLatitude: 39.35159; decimalLongitude: -4.3589; geodeticDatum: WGS84; **Event:** eventID: E; samplingProtocol: Pitfall
- **Type status:** Other material. **Occurrence:** individualCount: 1; sex: female; **Location:** locationID: C2; continent: Europe; country: Spain; countryCode: ES; stateProvince: Castilla-La Mancha; county: Ciudad Real; locality: Valle Brezoso; verbatimElevation: 739.31; decimalLatitude: 39.35159; decimalLongitude: -4.3589; geodeticDatum: WGS84; **Event:** eventID: G; samplingProtocol: Pitfall
- **Type status:** Other material. **Occurrence:** individualCount: 2; sex: male; **Location:** locationID: C2; continent: Europe; country: Spain; countryCode: ES; stateProvince: Castilla-La Mancha; county: Ciudad Real; locality: Valle Brezoso; verbatimElevation: 739.31; decimalLatitude: 39.35159; decimalLongitude: -4.3589; geodeticDatum: WGS84; **Event:** eventID: I; samplingProtocol: Pitfall
- **Type status:** Other material. **Occurrence:** individualCount: 1; sex: male; **Location:** locationID: C2; continent: Europe; country: Spain; countryCode: ES; stateProvince: Castilla-La Mancha; county: Ciudad Real; locality: Valle Brezoso; verbatimElevation: 739.31; decimalLatitude: 39.35159; decimalLongitude: -4.3589; geodeticDatum: WGS84; **Event:** eventID: K; samplingProtocol: Pitfall
- **Type status:** Other material. **Occurrence:** individualCount: 3; sex: male; **Location:** locationID: C2; continent: Europe; country: Spain; countryCode: ES; stateProvince: Castilla-La Mancha; county: Ciudad Real; locality: Valle Brezoso; verbatimElevation: 739.31; decimalLatitude: 39.35159; decimalLongitude: -4.3589; geodeticDatum: WGS84; **Event:** eventID: L; samplingProtocol: Pitfall
- **Type status:** Other material. **Occurrence:** individualCount: 1; sex: male; **Location:** locationID: C3; continent: Europe; country: Spain; countryCode: ES; stateProvince: Castilla-La Mancha; county: Ciudad Real; locality: La Quesera; verbatimElevation: 767.55; decimalLatitude: 39.36177; decimalLongitude: -4.41733; geodeticDatum: WGS84; **Event:** eventID: A; samplingProtocol: Pitfall
- **Type status:** Other material. **Occurrence:** individualCount: 2; sex: female; **Location:** locationID: C3; continent: Europe; country: Spain; countryCode: ES; stateProvince: Castilla-La Mancha; county: Ciudad Real; locality: La Quesera; verbatimElevation: 767.55; decimalLatitude: 39.36177; decimalLongitude: -4.41733; geodeticDatum: WGS84; **Event:** eventID: C; samplingProtocol: Pitfall
- **Type status:** Other material. **Occurrence:** individualCount: 1; sex: male; **Location:** locationID: C3; continent: Europe; country: Spain; countryCode: ES; stateProvince: Castilla-La Mancha; county: Ciudad Real; locality: La Quesera; verbatimElevation: 767.55; decimalLatitude: 39.36177; decimalLongitude: -4.41733; geodeticDatum: WGS84; **Event:** eventID: C; samplingProtocol: Pitfall
- **Type status:** Other material. **Occurrence:** individualCount: 2; sex: male; **Location:** locationID: C3; continent: Europe; country: Spain; countryCode: ES; stateProvince: Castilla-La Mancha; county: Ciudad Real; locality: La Quesera; verbatimElevation: 767.55; decimalLatitude: 39.36177; decimalLongitude: -4.41733; geodeticDatum: WGS84; **Event:** eventID: E; samplingProtocol: Pitfall
- **Type status:** Other material. **Occurrence:** individualCount: 1; sex: male; **Location:** locationID: C3; continent: Europe; country: Spain; countryCode: ES; stateProvince: Castilla-La Mancha; county: Ciudad Real; locality: La Quesera; verbatimElevation: 767.55; decimalLatitude: 39.36177; decimalLongitude: -4.41733; geodeticDatum: WGS84; **Event:** eventID: F; samplingProtocol: Pitfall
- **Type status:** Other material. **Occurrence:** individualCount: 2; sex: male; **Location:** locationID: C3; continent: Europe; country: Spain; countryCode: ES; stateProvince: Castilla-La Mancha; county: Ciudad Real; locality: La Quesera; verbatimElevation: 767.55; decimalLatitude: 39.36177; decimalLongitude: -4.41733; geodeticDatum: WGS84; **Event:** eventID: H; samplingProtocol: Pitfall
- **Type status:** Other material. **Occurrence:** individualCount: 1; sex: female; **Location:** locationID: C3; continent: Europe; country: Spain; countryCode: ES; stateProvince: Castilla-La Mancha; county: Ciudad Real; locality: La Quesera; verbatimElevation: 767.55; decimalLatitude: 39.36177; decimalLongitude: -4.41733; geodeticDatum: WGS84; **Event:** eventID: K; samplingProtocol: Pitfall
- **Type status:** Other material. **Occurrence:** individualCount: 1; sex: male; **Location:** locationID: C3; continent: Europe; country: Spain; countryCode: ES; stateProvince: Castilla-La Mancha; county: Ciudad Real; locality: La Quesera; verbatimElevation: 767.55; decimalLatitude: 39.36177; decimalLongitude: -4.41733; geodeticDatum: WGS84; **Event:** eventID: K; samplingProtocol: Pitfall
- **Type status:** Other material. **Occurrence:** individualCount: 2; sex: male; **Location:** locationID: C3; continent: Europe; country: Spain; countryCode: ES; stateProvince: Castilla-La Mancha; county: Ciudad Real; locality: La Quesera; verbatimElevation: 767.55; decimalLatitude: 39.36177; decimalLongitude: -4.41733; geodeticDatum: WGS84; **Event:** eventID: L; samplingProtocol: Pitfall
- **Type status:** Other material. **Occurrence:** individualCount: 3; sex: male; **Location:** locationID: C4; continent: Europe; country: Spain; countryCode: ES; stateProvince: Castilla-La Mancha; county: Ciudad Real; locality: La Quesera; verbatimElevation: 772.3; decimalLatitude: 39.36337; decimalLongitude: -4.41704; geodeticDatum: WGS84; **Event:** eventID: D; samplingProtocol: Pitfall
- **Type status:** Other material. **Occurrence:** individualCount: 4; sex: male; **Location:** locationID: C4; continent: Europe; country: Spain; countryCode: ES; stateProvince: Castilla-La Mancha; county: Ciudad Real; locality: La Quesera; verbatimElevation: 772.3; decimalLatitude: 39.36337; decimalLongitude: -4.41704; geodeticDatum: WGS84; **Event:** eventID: H; samplingProtocol: Pitfall
- **Type status:** Other material. **Occurrence:** individualCount: 1; sex: female; **Location:** locationID: M1; continent: Europe; country: Spain; countryCode: ES; stateProvince: Extremadura; county: Cáceres; locality: Peña Falcón; verbatimElevation: 320.6; decimalLatitude: 39.83296; decimalLongitude: -6.0641; geodeticDatum: WGS84; **Event:** eventID: I; samplingProtocol: Pitfall
- **Type status:** Other material. **Occurrence:** individualCount: 1; sex: female; **Location:** locationID: M2; continent: Europe; country: Spain; countryCode: ES; stateProvince: Extremadura; county: Cáceres; locality: Fuente del Frances; verbatimElevation: 320.72; decimalLatitude: 39.828; decimalLongitude: -6.03249; geodeticDatum: WGS84; **Event:** eventID: G; samplingProtocol: Pitfall
- **Type status:** Other material. **Occurrence:** individualCount: 1; sex: male; **Location:** locationID: S2; continent: Europe; country: Spain; countryCode: ES; stateProvince: Andalucía; county: Granada; locality: Camarate; verbatimElevation: 1713.96; decimalLatitude: 37.18377; decimalLongitude: -3.26282; geodeticDatum: WGS84; **Event:** eventID: A; samplingProtocol: Pitfall
- **Type status:** Other material. **Occurrence:** individualCount: 1; sex: female; **Location:** locationID: S2; continent: Europe; country: Spain; countryCode: ES; stateProvince: Andalucía; county: Granada; locality: Camarate; verbatimElevation: 1713.96; decimalLatitude: 37.18377; decimalLongitude: -3.26282; geodeticDatum: WGS84; **Event:** eventID: C; samplingProtocol: Pitfall
- **Type status:** Other material. **Occurrence:** individualCount: 1; sex: male; **Location:** locationID: S2; continent: Europe; country: Spain; countryCode: ES; stateProvince: Andalucía; county: Granada; locality: Camarate; verbatimElevation: 1713.96; decimalLatitude: 37.18377; decimalLongitude: -3.26282; geodeticDatum: WGS84; **Event:** eventID: C; samplingProtocol: Pitfall
- **Type status:** Other material. **Occurrence:** individualCount: 1; sex: male; **Location:** locationID: S2; continent: Europe; country: Spain; countryCode: ES; stateProvince: Andalucía; county: Granada; locality: Camarate; verbatimElevation: 1713.96; decimalLatitude: 37.18377; decimalLongitude: -3.26282; geodeticDatum: WGS84; **Event:** eventID: J; samplingProtocol: Pitfall

##### Distribution

Iberian Peninsula, Madeira

#### Zoropsidae

Bertkau, 1882

#### Zoropsis media

Simon, 1878

##### Materials

- **Type status:** Other material. **Occurrence:** individualCount: 3; sex: male; **Location:** locationID: C1; continent: Europe; country: Spain; countryCode: ES; stateProvince: Castilla-La Mancha; county: Ciudad Real; locality: Valle Brezoso; verbatimElevation: 756.56; decimalLatitude: 39.35663; decimalLongitude: -4.35912; geodeticDatum: WGS84; **Event:** eventID: D; samplingProtocol: Pitfall
- **Type status:** Other material. **Occurrence:** individualCount: 1; sex: female; **Location:** locationID: C1; continent: Europe; country: Spain; countryCode: ES; stateProvince: Castilla-La Mancha; county: Ciudad Real; locality: Valle Brezoso; verbatimElevation: 756.56; decimalLatitude: 39.35663; decimalLongitude: -4.35912; geodeticDatum: WGS84; **Event:** eventID: E; samplingProtocol: Pitfall
- **Type status:** Other material. **Occurrence:** individualCount: 1; sex: male; **Location:** locationID: C1; continent: Europe; country: Spain; countryCode: ES; stateProvince: Castilla-La Mancha; county: Ciudad Real; locality: Valle Brezoso; verbatimElevation: 756.56; decimalLatitude: 39.35663; decimalLongitude: -4.35912; geodeticDatum: WGS84; **Event:** eventID: E; samplingProtocol: Pitfall
- **Type status:** Other material. **Occurrence:** individualCount: 2; sex: male; **Location:** locationID: C1; continent: Europe; country: Spain; countryCode: ES; stateProvince: Castilla-La Mancha; county: Ciudad Real; locality: Valle Brezoso; verbatimElevation: 756.56; decimalLatitude: 39.35663; decimalLongitude: -4.35912; geodeticDatum: WGS84; **Event:** eventID: F; samplingProtocol: Pitfall
- **Type status:** Other material. **Occurrence:** individualCount: 1; sex: male; **Location:** locationID: C1; continent: Europe; country: Spain; countryCode: ES; stateProvince: Castilla-La Mancha; county: Ciudad Real; locality: Valle Brezoso; verbatimElevation: 756.56; decimalLatitude: 39.35663; decimalLongitude: -4.35912; geodeticDatum: WGS84; **Event:** eventID: G; samplingProtocol: Pitfall
- **Type status:** Other material. **Occurrence:** individualCount: 3; sex: male; **Location:** locationID: C1; continent: Europe; country: Spain; countryCode: ES; stateProvince: Castilla-La Mancha; county: Ciudad Real; locality: Valle Brezoso; verbatimElevation: 756.56; decimalLatitude: 39.35663; decimalLongitude: -4.35912; geodeticDatum: WGS84; **Event:** eventID: I; samplingProtocol: Pitfall
- **Type status:** Other material. **Occurrence:** individualCount: 2; sex: male; **Location:** locationID: C1; continent: Europe; country: Spain; countryCode: ES; stateProvince: Castilla-La Mancha; county: Ciudad Real; locality: Valle Brezoso; verbatimElevation: 756.56; decimalLatitude: 39.35663; decimalLongitude: -4.35912; geodeticDatum: WGS84; **Event:** eventID: J; samplingProtocol: Pitfall
- **Type status:** Other material. **Occurrence:** individualCount: 1; sex: male; **Location:** locationID: C1; continent: Europe; country: Spain; countryCode: ES; stateProvince: Castilla-La Mancha; county: Ciudad Real; locality: Valle Brezoso; verbatimElevation: 756.56; decimalLatitude: 39.35663; decimalLongitude: -4.35912; geodeticDatum: WGS84; **Event:** eventID: K; samplingProtocol: Pitfall
- **Type status:** Other material. **Occurrence:** individualCount: 1; sex: male; **Location:** locationID: C1; continent: Europe; country: Spain; countryCode: ES; stateProvince: Castilla-La Mancha; county: Ciudad Real; locality: Valle Brezoso; verbatimElevation: 756.56; decimalLatitude: 39.35663; decimalLongitude: -4.35912; geodeticDatum: WGS84; **Event:** eventID: L; samplingProtocol: Pitfall
- **Type status:** Other material. **Occurrence:** individualCount: 1; sex: male; **Location:** locationID: C3; continent: Europe; country: Spain; countryCode: ES; stateProvince: Castilla-La Mancha; county: Ciudad Real; locality: La Quesera; verbatimElevation: 767.55; decimalLatitude: 39.36177; decimalLongitude: -4.41733; geodeticDatum: WGS84; **Event:** eventID: L; samplingProtocol: Pitfall
- **Type status:** Other material. **Occurrence:** individualCount: 1; sex: male; **Location:** locationID: C4; continent: Europe; country: Spain; countryCode: ES; stateProvince: Castilla-La Mancha; county: Ciudad Real; locality: La Quesera; verbatimElevation: 772.3; decimalLatitude: 39.36337; decimalLongitude: -4.41704; geodeticDatum: WGS84; **Event:** eventID: I; samplingProtocol: Pitfall
- **Type status:** Other material. **Occurrence:** individualCount: 1; sex: male; **Location:** locationID: C4; continent: Europe; country: Spain; countryCode: ES; stateProvince: Castilla-La Mancha; county: Ciudad Real; locality: La Quesera; verbatimElevation: 772.3; decimalLatitude: 39.36337; decimalLongitude: -4.41704; geodeticDatum: WGS84; **Event:** eventID: J; samplingProtocol: Pitfall
- **Type status:** Other material. **Occurrence:** individualCount: 1; sex: male; **Location:** locationID: M1; continent: Europe; country: Spain; countryCode: ES; stateProvince: Extremadura; county: Cáceres; locality: Peña Falcón; verbatimElevation: 320.6; decimalLatitude: 39.83296; decimalLongitude: -6.0641; geodeticDatum: WGS84; **Event:** eventID: B; samplingProtocol: Pitfall
- **Type status:** Other material. **Occurrence:** individualCount: 2; sex: male; **Location:** locationID: M1; continent: Europe; country: Spain; countryCode: ES; stateProvince: Extremadura; county: Cáceres; locality: Peña Falcón; verbatimElevation: 320.6; decimalLatitude: 39.83296; decimalLongitude: -6.0641; geodeticDatum: WGS84; **Event:** eventID: D; samplingProtocol: Pitfall
- **Type status:** Other material. **Occurrence:** individualCount: 1; sex: male; **Location:** locationID: M1; continent: Europe; country: Spain; countryCode: ES; stateProvince: Extremadura; county: Cáceres; locality: Peña Falcón; verbatimElevation: 320.6; decimalLatitude: 39.83296; decimalLongitude: -6.0641; geodeticDatum: WGS84; **Event:** eventID: H; samplingProtocol: Pitfall
- **Type status:** Other material. **Occurrence:** individualCount: 1; sex: male; **Location:** locationID: M1; continent: Europe; country: Spain; countryCode: ES; stateProvince: Extremadura; county: Cáceres; locality: Peña Falcón; verbatimElevation: 320.6; decimalLatitude: 39.83296; decimalLongitude: -6.0641; geodeticDatum: WGS84; **Event:** eventID: K; samplingProtocol: Pitfall
- **Type status:** Other material. **Occurrence:** individualCount: 1; sex: female; **Location:** locationID: M1; continent: Europe; country: Spain; countryCode: ES; stateProvince: Extremadura; county: Cáceres; locality: Peña Falcón; verbatimElevation: 320.6; decimalLatitude: 39.83296; decimalLongitude: -6.0641; geodeticDatum: WGS84; **Event:** eventID: L; samplingProtocol: Pitfall
- **Type status:** Other material. **Occurrence:** individualCount: 2; sex: male; **Location:** locationID: M1; continent: Europe; country: Spain; countryCode: ES; stateProvince: Extremadura; county: Cáceres; locality: Peña Falcón; verbatimElevation: 320.6; decimalLatitude: 39.83296; decimalLongitude: -6.0641; geodeticDatum: WGS84; **Event:** eventID: L; samplingProtocol: Pitfall
- **Type status:** Other material. **Occurrence:** individualCount: 1; sex: female; **Location:** locationID: O2; continent: Europe; country: Spain; countryCode: ES; stateProvince: Aragón; county: Huesca; locality: Rebilla; verbatimElevation: 1158.13; decimalLatitude: 42.59427; decimalLongitude: 0.1529; geodeticDatum: WGS84; **Event:** eventID: K; samplingProtocol: Pitfall
- **Type status:** Other material. **Occurrence:** individualCount: 1; sex: female; **Location:** locationID: S1; continent: Europe; country: Spain; countryCode: ES; stateProvince: Andalucía; county: Granada; locality: Soportujar; verbatimElevation: 1786.57; decimalLatitude: 36.96151; decimalLongitude: -3.41881; geodeticDatum: WGS84; **Event:** eventID: 1; samplingProtocol: Aerial; eventTime: Night
- **Type status:** Other material. **Occurrence:** individualCount: 1; sex: male; **Location:** locationID: S1; continent: Europe; country: Spain; countryCode: ES; stateProvince: Andalucía; county: Granada; locality: Soportujar; verbatimElevation: 1786.57; decimalLatitude: 36.96151; decimalLongitude: -3.41881; geodeticDatum: WGS84; **Event:** eventID: 1; samplingProtocol: Aerial; eventTime: Night
- **Type status:** Other material. **Occurrence:** individualCount: 1; sex: female; **Location:** locationID: S1; continent: Europe; country: Spain; countryCode: ES; stateProvince: Andalucía; county: Granada; locality: Soportujar; verbatimElevation: 1786.57; decimalLatitude: 36.96151; decimalLongitude: -3.41881; geodeticDatum: WGS84; **Event:** eventID: 4; samplingProtocol: Aerial; eventTime: Night
- **Type status:** Other material. **Occurrence:** individualCount: 1; sex: male; **Location:** locationID: S1; continent: Europe; country: Spain; countryCode: ES; stateProvince: Andalucía; county: Granada; locality: Soportujar; verbatimElevation: 1786.57; decimalLatitude: 36.96151; decimalLongitude: -3.41881; geodeticDatum: WGS84; **Event:** eventID: G; samplingProtocol: Pitfall
- **Type status:** Other material. **Occurrence:** individualCount: 1; sex: male; **Location:** locationID: S1; continent: Europe; country: Spain; countryCode: ES; stateProvince: Andalucía; county: Granada; locality: Soportujar; verbatimElevation: 1786.57; decimalLatitude: 36.96151; decimalLongitude: -3.41881; geodeticDatum: WGS84; **Event:** eventID: I; samplingProtocol: Pitfall
- **Type status:** Other material. **Occurrence:** individualCount: 1; sex: male; **Location:** locationID: S1; continent: Europe; country: Spain; countryCode: ES; stateProvince: Andalucía; county: Granada; locality: Soportujar; verbatimElevation: 1786.57; decimalLatitude: 36.96151; decimalLongitude: -3.41881; geodeticDatum: WGS84; **Event:** eventID: J; samplingProtocol: Pitfall
- **Type status:** Other material. **Occurrence:** individualCount: 1; sex: female; **Location:** locationID: S1; continent: Europe; country: Spain; countryCode: ES; stateProvince: Andalucía; county: Granada; locality: Soportujar; verbatimElevation: 1786.57; decimalLatitude: 36.96151; decimalLongitude: -3.41881; geodeticDatum: WGS84; **Event:** eventID: K; samplingProtocol: Pitfall
- **Type status:** Other material. **Occurrence:** individualCount: 2; sex: male; **Location:** locationID: S2; continent: Europe; country: Spain; countryCode: ES; stateProvince: Andalucía; county: Granada; locality: Camarate; verbatimElevation: 1713.96; decimalLatitude: 37.18377; decimalLongitude: -3.26282; geodeticDatum: WGS84; **Event:** eventID: B; samplingProtocol: Pitfall
- **Type status:** Other material. **Occurrence:** individualCount: 1; sex: male; **Location:** locationID: S2; continent: Europe; country: Spain; countryCode: ES; stateProvince: Andalucía; county: Granada; locality: Camarate; verbatimElevation: 1713.96; decimalLatitude: 37.18377; decimalLongitude: -3.26282; geodeticDatum: WGS84; **Event:** eventID: G; samplingProtocol: Pitfall
- **Type status:** Other material. **Occurrence:** individualCount: 1; sex: male; **Location:** locationID: S2; continent: Europe; country: Spain; countryCode: ES; stateProvince: Andalucía; county: Granada; locality: Camarate; verbatimElevation: 1713.96; decimalLatitude: 37.18377; decimalLongitude: -3.26282; geodeticDatum: WGS84; **Event:** eventID: I; samplingProtocol: Pitfall
- **Type status:** Other material. **Occurrence:** individualCount: 1; sex: male; **Location:** locationID: S2; continent: Europe; country: Spain; countryCode: ES; stateProvince: Andalucía; county: Granada; locality: Camarate; verbatimElevation: 1713.96; decimalLatitude: 37.18377; decimalLongitude: -3.26282; geodeticDatum: WGS84; **Event:** eventID: K; samplingProtocol: Pitfall

##### Distribution

Western Mediterranean

## Analysis

### Biogeographic composition

After sorting 20,539 spiders, we identified 376 species based on 8,521 adult specimens, corresponding to 190 genera in 39 families. Eight species, *Dictyna pusilla*, *Agyneta orites*, *Zora silvestris*, *Philodromus buchari*, *Pseudeuophrys nebrodensis*, *Euryopis flavomaculata*, *Euryopis sexalbomaculata* and *Titanoeca schineri* are new citations for the Iberian Peninsula, while two species, *Dipoena torva* and *Sardinidion blackwalli*, are first citations for Spain. Thirteen putative new species to science and 20 additional unidentified species, some of them candidates to new species, were found.

**Figure 16.**
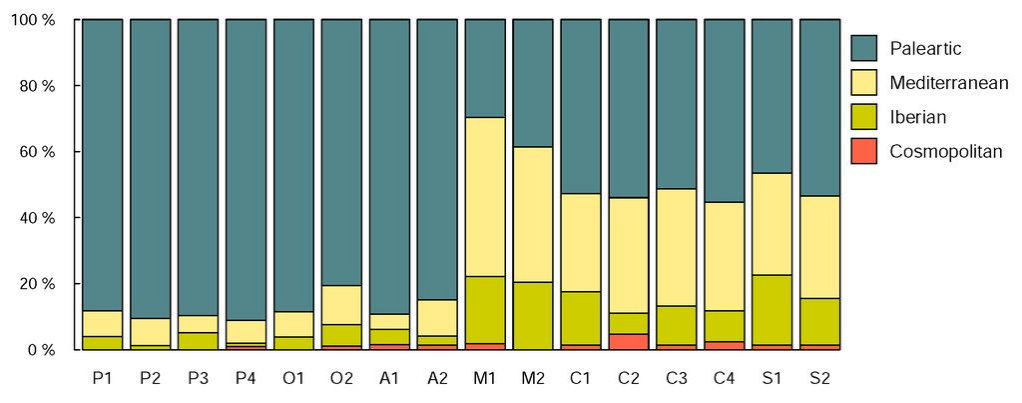
Plot with percentages of species of different biogeographic categories, namely Palearctic, Mediterranean, Iberian, Cosmopolitan, per National Park.

The percentage of occurrence of species from the different groups in each park (Fig. 16) indicated that the northern parks (Picos de Europa (P1, P2, P3, P4), Ordesa (O1, O2) and Aigüestortes (A1, A2)), where the Palearctic species were dominant, have lower percentages of Mediterranean and Iberian endemic species than southern parks (Monfragüe (M1, M2), Cabañeros (C1, C2, C3, C4) and Sierra Nevada (S1, S2)). Quantitative analyses of the ecological patterns in the spider communities and the drivers behind them are presented elsewhere (Malumbres-Olarte et al., in prep).

### Species delimitation and identification using DNA barcodes

For some individuals in certain families, identification solely based on morphology was doubtful or insufficient. In these cases, we used DNA barcode information to support or further refine species delimitation and identification. The sequences generated and analysed in the present study are avilable at GenBank under accession numbers MH630465-MH630994.

**Family Dictynidae**

**Figure 17.**
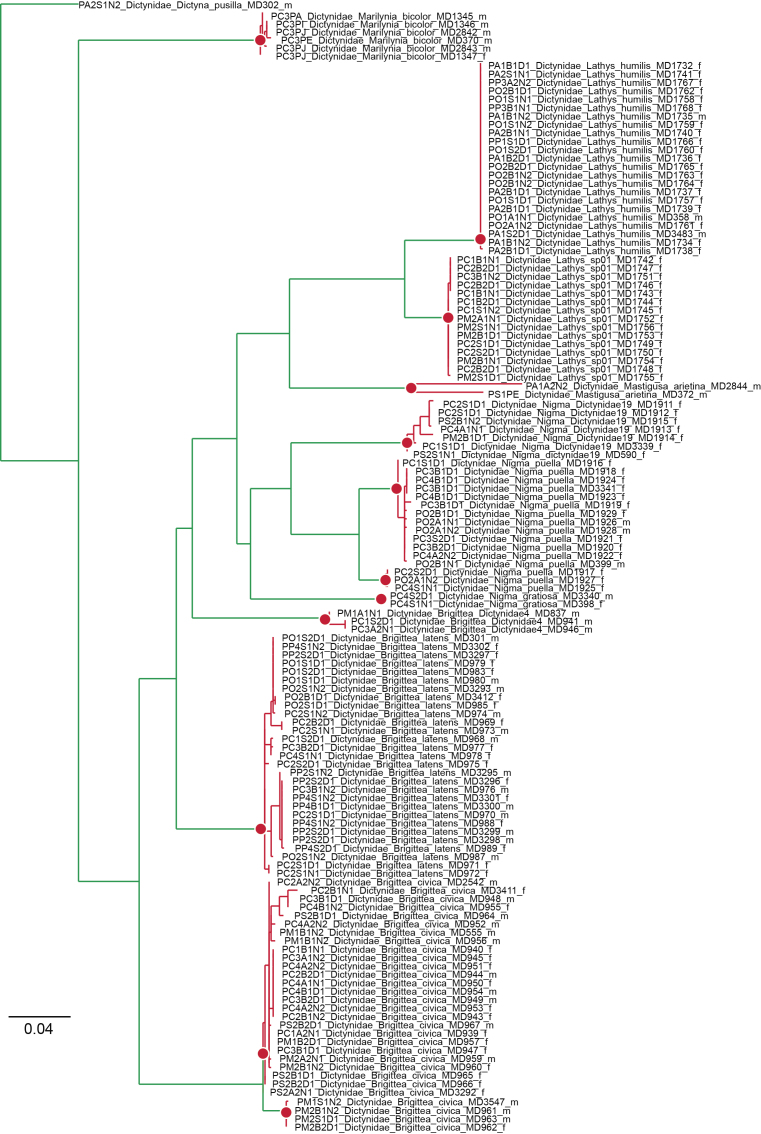
Maximum likelihood tree of the Dictynidae DNA barcode sequences analysed, using a codon partition model with IQ-tree. Red coloured clusters correspond to candidate species as delimited using the mPTP algorithm (see text for details).

Due to the possibility of overlooked Mediterranean *Brigittea* species, based on earlier observations by the first author and some dubious citations of *Brigittea innocens* (O. Pickard-Cambridge, 1872) from Italy in the literature (as recognised by Ijland et al. 2012: 6), as well as the finding of two poorly known species of the genus *Nigma*, we decided to further explore Dictynidae identifications with DNA barcodes. We included 134 specimens in the molecular analyses, which yielded 83 unique DNA barcodes corresponding to species belonging to 6 genera, namely *Brigittea, Dictyna*, *Lathys*, *Nigma*, *Marilynia* and *Mastigusa* (now placed in the Hahniidae). The mPTP algorithm identified 13 groups (Fig. 17). Three of the groups corresponded to *Dictyna*, *Marilynia* and *Mastigusa* (for each of which only one species was found, respectively *D. pusilla*, *M. bicolor* and *M. arietina*). The two *Mastigusa arietina* sequences showed high levels of intraspecific genetic divergence (10%). The sequence of *Dictyna pusilla* was correctly identified in BOLD with a 100% match. There were no representatives of either *Marilynia* or *Mastigusa* in the BOLD database.

In the case of the genus *Brigittea*, the morphology-based identifications indicated the presence of the two most widespread species in Europe, namely *B. civica* and *B. latens*. However, the delimitation analysis of the DNA barcodes suggested the presence of four different clusters, one including all individuals of *B. latens*, two corresponding to *B. civica* and a fourth, composed of 3 males from sites M1, C1 and C3, initially identified as *B. civica*. Genetic divergences within each cluster were low (from 0.1 to 1%). Two of the clusters identified as *B. civica* also showed low genetic divergences between them (2.3%). However, divergences between these clusters and the third group were much higher (13.3-14.2%) and similar to those observed between the nominal species *B. civica* and *B. latens* (12.4-13%). Automatic identification in BOLD returned correct identification for *B. latens* and the two genetically similar *B. civica* clusters (99.23%, 99.85% and 98.15% similarity, respectively). However, the divergent *B. civica* cluster found no match. We revised the specimens in this group and found some previously overlooked somatic differences, for example in the abdominal colour as well as slight difference in the male palp and hence it may correspond to an overlooked species (*Brigittea* sp04).

The mPTP analysis grouped the *Nigma* sequences into 4 clusters (Fig. 17), one corresponding to a male and a female of *N. gratiosa* (species in which the female is very poorly known, see Fig. 5), Fig. 5, a second one including the sequences of unidentified individuals (*Nigma* sp19) and two clusters with sequences of the widely distributed nominal species *N. puella*, with no clear geographic structure. Within the group, divergences were low (0.2-1.2%). Divergences were high between the nominal species (12.3-13.1%) and between these species and the unidentified cluster (*Nigma* sp19, 11.4-14%), while divergences between the two *N. puella* were lower (4%). Only one of the clusters, corresponding to *N. puella*, found a match in the BOLD database and was correctly identified with 99.84% similarity to *N. puella* sequences from northern and central Europe. We revised morphological identification of females in both clusters containing *N. puella* and found no differences.

The DNA barcodes of the specimens of the genus *Lathys* clustered into two different groups, with moderate levels of genetic divergence (7.5%), corresponding to *L. humilis* from the northern parks (Aigüestortes, Ordesa and Picos de Europa) and a putative new species, *Lathys* sp01, from two of the southern parks (Cabañeros and Monfragüe), respectively. The northern parks were correctly identified (100%) and clustered with *L. humilis* sequences from Germany available in BOLD. The *Lathys* sp01 cluster returned no match. Additionally, sequences available in BOLD from Slovenia formed a separate cluster from those from Germany and the northern parks.

**Family Gnaphosidae**

**Figure 18.**
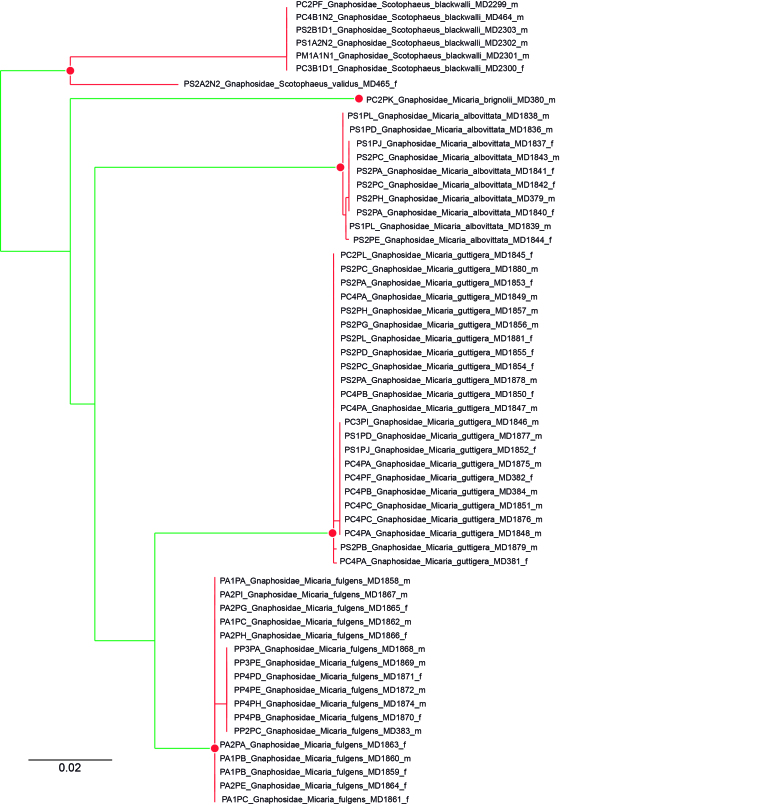
Maximum likelihood tree of the Gnaphosidae DNA barcode sequences analysed, using a codon partition model with IQ-tree. Red coloured clusters correspond to candidate species as delimited using the mPTP algorithm (see text for details).

Gnaphosids are highly diverse in the Iberian Peninsula. Although we did discover a morphologically diagnosable putative new species of *Nomisia*, we decided to further scrutinise other local species that closely resemble well-known widespread species in the genera *Micaria* and *Scotophaeus*. We included 58 specimens in the molecular analysis, which yielded 26 unique DNA barcodes. The mPTP algorithm identified 5 groups (Fig. 18). All *Scotophaeus* sequences were clustered into a single cluster, while *Micaria* resolved into four groups, corresponding to the nominal species *M. brignoli, M. albovittata*, *M. fulgens* and *M. guttigera*, respectively. Genetic divergences between the *Micaria* species in our study ranged from 10.3%, between *M. fulgens* and *M. albovittata* to 18.5%, between *M. guttigera* and *M. brignoli*. We could not assess intraspecific divergences of the latter species because only a single specimen was found. Divergences within the *M. guttigera* were as low as 0.3%. Unfortunately, no DNA barcodes for *M. guttigera* or closely related species were available in public repositories for comparison. The closest match to the *M. guttigera* cluster returned in BOLD was *Micaria corvina* (92.17% similarity). *M. albovittata* was the only *Micaria* species in our study to have conspecific sequences available in BOLD. However, although our query sequence clustered with other *M. albovittata* sequences from Italy, Bulgaria and France, genetic divergences were too great for an unequivocal assignment (94.25%).

The genus *Scotophaeus* was represented in our samples by two species, *S. blackwalli* and S. cf. validus. Surprisingly, the species delimitation analyses clustered the sequences of the two species in a single cluster. The genetic divergence between the *S. blackwalli* and S. cf. validus sequences, however, was similar to those estimated for the *Micaria* species (12%), while all the sequences of *S. blackwalli* (n=6) were identical. Although *S. blackwalli* is represented in public repositories by several sequences, our query did not return any identification. The closest match corresponded to *S. blackwalli* from Germany (90.48% similarity), which in turn showed similar low levels of similarity to *S. blackwalli* from Canada and the USA. The single female specimen identified as S. cf. validus could not be assigned to any species in BOLD. The closest match returned sequences assigned to *S. scutulatus* (L. Koch, 1866) from Germany, Greece and Turkey, but with low similarity (87.83 to 87.99%).

**Family Linyphiidae**

**Figure 19.**
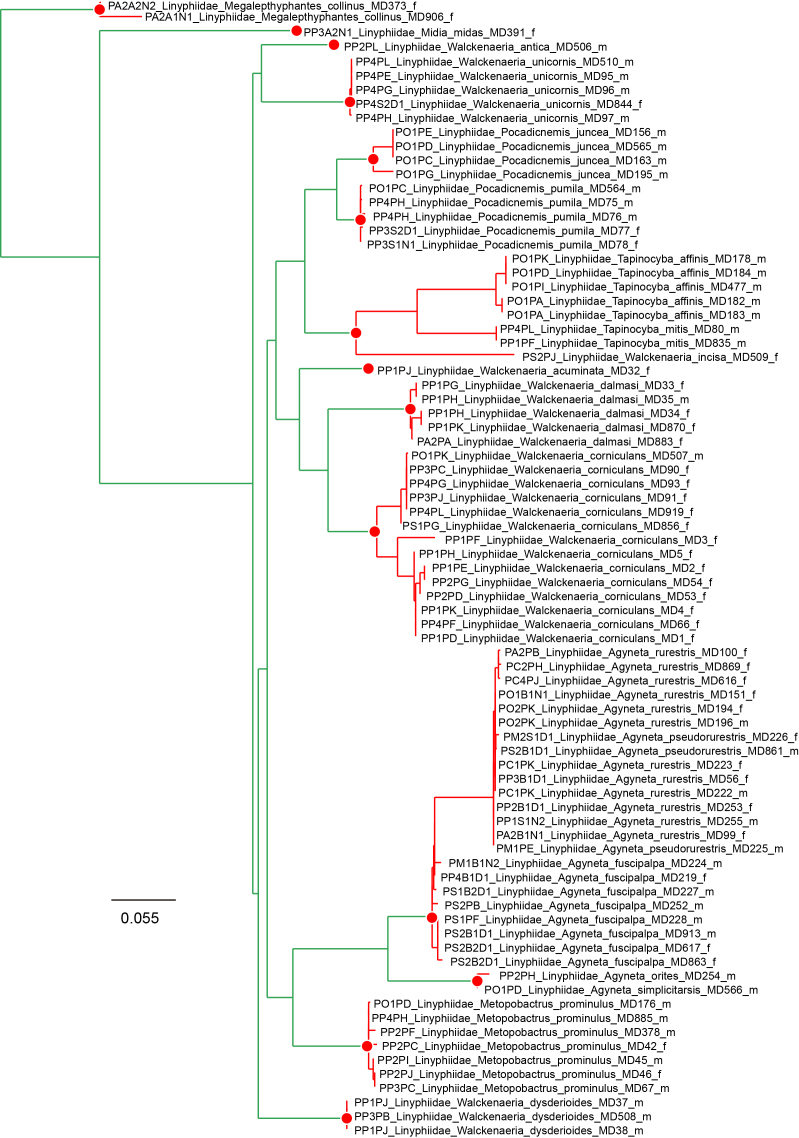
Maximum likelihood tree of the Linyphiidae DNA barcode sequences analysed, using a codon partition model with IQ-tree. Red coloured clusters correspond to candidate species as delimited using the mPTP algorithm (see text for details).

Some observations made during the morphological identification called for a cross-validation of some linyphiids using DNA barcodes. We included 81 specimens in the molecular analysis, which yielded 76 unique DNA barcodes corresponding to 7 genera, namely *Agyneta*, *Metopobactrus*, *Megaleptyphantes*, *Midas*, *Pocadicnemis*, *Tapinocyba* and*Walckenaeria*. The mPTP algorithm yielded 14 clusters (Fig. 19). Ten of the groups had a one-to-one fit to the nominal species identified in the study, while the remaining four included more than one species. In the genus *Metopobactrus*, we discovered a case of male polymorphism. Eleven specimens of this genus were collected from two different national parks. One male from Ordesa was identified as *M. prominulus* (Fig. 7a), while the remaining males were collected in Picos de Europa and presented a remarkable turret-like cephalic lobe (Fig. 7b), similar to the one reported in the congeneric species *M. falcifrons* Simon, 1884, from France and Spain. Although slight differences were observed in the tibial apophyses between the single specimen from Ordesa and the specimens from Picos de Europa (Fig. 7d-g), these were not greater than the variation observed amongst the Picos specimens. Identification of females from Picos was inconclusive, while no females were collected from Ordesa. The DNA barcodes of the 5 *Metopobactrus* adult specimens available revealed low levels of intraspecific divergence (0.8%). The singleton identified as *A. orites* was clustered together with a singleton of *A. simplicitarsis*, which was odd considering that both males have conspicuous morphological differences. Although the remaining *Agyneta* individuals were all clustered together, two distinct lineages were apparent within this cluster, one including males identified as *A. rurestris* and *A. pseudorurestris* and a second one including males identified as *A. fuscipalpa*. Female specimens of this genus are very difficult to identify by morphology alone. The genetic delimitation assigned three females to the lineage including the three *A. fuscipalpa* males, while the remaining females were included in the *A. rurestris/A. pseudorurestris* males group. The most extremes case of interspecific clustering was that of the grouping of specimens belonging to the two *Tapinocyba* species (*T. affinis* and *T. mitis*), which also included the single specimen of *Walckenaeria incisa*.

The largest intraspecific divergences corresponded to *P. juncea* (2.4%), while remaining divergences were below 1%. The interspecific divergences, on the other hand, were all above 10%. The nominal species *Tapinocyba affinis*, *T. mitis* and *Walckenaeria incisa* delimited in a single cluster, showed interspecific divergences between 11%, for the congeneric comparison, and >15% between genera. The genetic divergence between the two distinct groups within the large *Agyneta* cluster was 4.8%.

The BOLD automatic identification algorithm correctly identified the following species: *Metopobactrus prominulus*, *Walckenaeria acuminata*, *Walckenaeria antica*, *Walckenaeria dysderoides* and *Walckenaeria unicornis* (similarity >98.92%). The closest match for *Tapinocyba affinis* corresponded to an unidentified *Tapinocyba* (similarity 98.78), and the tree based identification clustered it close to sequences identified as *T. affinis* and *T. pallens* (O. Pickard-Cambridge, 1873). Surprisingly, our *P. juncea* sequences were assigned to *P. pumila* (similarity 99.85%), while sequences identified in our study as *P. pumila*, were assigned to *P. americana* (95.44%). The *Agyneta* specimens identified as *A. orites* and *A. simplicitarsis* were both assigned by BOLD to *A. simplicitarsis* (98.92 and 100%, respectively). The group including *A. rurestris*, both males of *A. pseudorurestris* and several problematic females, was assigned to *A. rurestris* (100%), while the lineage with *A. fuscipalpa* males and other problematic females was assigned to *A. fuscipalpa* (99.8%). The two *Megaleptyphantes* species identified in our samples were assigned by BOLD to *M. pseudocollinus* (97.14 and 97.69% respectively), and after carefully revising these specimens, we opted to classify them as M. cf. collinus. The species *Walckenaeria corniculans* did not return any match but did cluster with available conspecifics (94.8%, Germany and Turkey), and the grouping also included a *W. monoceros* (Germany). The remaining query sequences had no conspecifics in BOLD and did not return any match.

**Family *Philodromidae***

The Philodromidae are among the most difficult spider families to identify in the Mediterranean, especially female specimens (Muster et al. 2007). Species commonly present indistinct genitalia which, coupled with intraspecific variability, often clouds the decision of morphology based identification. Therefore, the Philodromidae provided an ideal test case to integrate DNA barcode information with traditional morphological approaches to species delimitation and identification. The genera *Philodromus, Pulchellodromus* and *Thanatus* include species with similar genitalia and females are usually difficult to identify. We sequenced 259 specimens belonging to the *Philodromus* plus the single species of *Tibellus* collected, which yielded 149 unique DNA barcodes. The single-gene species delimitation method resolved 21 putative species (Fig. 20), which neatly corresponded to our species identified using male morphological characters and in few cases on females. However, in some instances morphological assignations of females did not match the genetic clusters. Specifically, some females originally assigned by morphology to different collected species belonging to the *Philodromus-aureolus* group (as defined in Muster and Thaler 2004), turned out to form a distinct genetic cluster, to which we refer as *Philodromus* sp18. Within cluster genetic divergences were low, below 1% except for clusters corresponding to *Philodromus rufus, Pulchellodromus simoni*, exclusively formed by female specimens, and *Thanatus vulgaris*, which showed 1.5, 1.02 and 1.6%, respectively. The lowest between-cluster divergence observed was between two *Philodromus* clusters (3%), both identified as *Philodromus aureolus.* The remaining intra-cluster divergences were all above 10%, except for some within-genus comparisons that dropped to between 5.3 to 8% (e.g. 5.3% between *Pulchellodromus glaucinus* and *Pulchellodromus pulchellus*). The sequences of *Thanatus oblongiusculus* were more similar to those of *Tibellus oblongus* (11.6%) than to any of the two other *Thanatus* species sampled (15.1 and 15.5%).

Automatic identification in BOLD returned matches (>97.38% similarity) for 15 out of the 21 clusters. The closest match of the species *Philodromus emarginatus* was a conspecific (similarity 93.52%, Finland, Norway and Russia). *Pulchellodromus simoni* returned a 98.5% similarity with an unidentified Philodromidae from Egypt. The following species had no conspecific available in BOLD: *Philodromus fuscolimbatus*, *Philodromus buchari*, *Philodromus lividus*, *Pulchellodromus glaucinus*, *Tanatus oblongiusculus* or *Philodromus pinetorum*. Interestingly sp. 18 was assigned to *Philodromus praedatus* (>99%, similarity). However, after visualising the identification tree, we observed that sequences identified as *Philodromus praedatus* in BOLD formed two independent clusters, one formed by 2 specimens from Germany, 1 from Slovenia and one identified as of *Philodromus longipalpis*, identical to the Slovenian specimen, closer to our sp. 18 and a second cluster that included the bulk of *Philodromus praedatus* specimens (Germany, Bulgaria, Switzerland and the Netherlands), to which our sequences identified as *Philodromus praedatus* clustered.

## Discussion

The taxonomic and ecological data gathered in semi-quantitative inventories, such as the one conducted here, provide essential information on the diversity and health of ecosystems and set the ground for subsequent monitoring programmes that track changes in space and time for conservation and management purposes (Barnosky et al. 2013). Arthropods and other highly diverse invertebrates are excellent indicators of the health of ecosystems. On one hand, they provide information on biodiversity patterns and provide the data to infer the processes behind them at finer spatial and shorter temporal scales than vertebrates or plants (Kremen et al. 1993). On the other hand, because of the high abundance and short generation time, they are more sensitive to environmental perturbations (Yen and Butcher 1997). Despite their advantages for tracking ecosystem changes, arthropods and invertebrates, in general, are often neglected in biodiversity studies and conservation programmes due to the Linnean, Wallacean, Prestonian and Hutchinsonian shortfalls (Cardoso et al. 2011). Optimised sampling protocols (Oliver and Beattie 1996), especially those based on taxon sampling curves (Colwell and Coddington 1994, Coddington et al. 1991), offer unparallelled opportunities to study communities of mega-diverse groups. Our study confirms the productivity of semi-quantitative protocols such as COBRA, through which we were able to recover three-quarters of the families, half of the genera and close to one-third of the species known to be present in the Iberian Peninsula.

Traditionally, inventorying of arthropods has also been hampered by poor taxonomy (e.g. uninformative diagnosis, lack of illustrations) and the lack of taxonomic expertise. Molecular approaches may provide a more time efficient and informative at finer scales alternative to morphologically based identification (e.g. Monaghan et al. 2009). However, the exclusive use of molecular information for species delimitation and identification, especially when it is based exclusively on single, haplotypic markers, may be challenged by a number of events: e.g. coalescent processes associated with large population sizes and recent species divergences (Maddison 1997), hybridisation between close relatives (Whitworth et al. 2007) or deep population structuring in low dispersal species (Kuo and Avise 2005). Additionally, the female inheritance of mitochondrial markers may compromise their ability to delimit species boundaries in species with male-biased gene-flow (Petit and Excoffier 2009). Ideally, molecular data should be used in combination with morphological identification to alleviate the former limitations in species identification – by facilitating sorting and assignment of sex, stages and remains and democratising identification (Hajibabaei et al. 2007, Ratnasingham and Hebert 2007, Hebert et al. 2003) – and in species delimitation – by reinterpreting phenotypic polymorphism (Hebert et al. 2004, Teske et al. 2007) or revealing introgressive hybridisation (Rougerie et al. 2012).

Our study stands out as a clear example of the benefits of incorporating DNA barcode information to contrast and refine preliminary sorting and identification solely based on morphological observations and vice-versa. First, DNA barcodes revealed previously overlooked diversity in some of the investigated groups. Specifically, deeply divergent genetic lineages that could not be assigned to any nominal species, using either morphology or BOLD identification, were found within the Dictynidae genera *Brigittea, Nigma* and *Lathys*. This indicates a lack of knowledge even for a taxon – dyctinids – that is not particularly species-rich in the region. Putative new species of *Brigittea* (sp04), *Lathys* (sp01, only females) and *Nigma* (sp19, only females), were found in parks with Mediterranean characteristics (Cabañeros and Monfragüe and sp. 19 also in Sierra Nevada). These data point to the existence of overlooked diversity within a species with a continental-wide distribution in Europe and call for further phylogeographic scrutiny. Other Dictynidae species may show a similar pattern. For instance, *Mastigusa arietina* specimens, sampled from Aigüestortes and Sierra Nevada, showed even deeper genetic divergences than *Lathys*, although the small sample size (2 individuals) prevented us from taking any further decisions.

Second, DNA barcodes provided evidence to assign unknown or poorly diagnosable specimens to species. For example within Dictynidae, the DNA barcodes confirmed that one female identified as *Nigma* (Dictynidae) was conspecific to the male identified as *N. gratiosa*, a poorly known species for which females had not been previously documented. Here we illustrate the female of this species for the first time (see Fig. 5). A similar situation was observed in Philodromidae, which are amongst the most difficult spider families to identify in the Mediterranean, especially regarding female specimens (Muster et al. 2007). After the first round of morphological identifications, DNA barcoding clusters revealed several mismatches to morphological identifications, in all instances involving female specimens. Morphological re-examination of the conflicting specimens suggested that mismatches were the result of either poorly interpreted or lack of diagnostic characters. In the Philodromidae, for example, two individuals first considered as a different morphospecies were later assigned by DNA barcodes to *Philodromus lividus* and the specimens re-interpreted as immatures with a protoepigyne (but with apparently complete spermathecae). Similarly, in the Linyphiidae, several female specimens of the linyphiid genus *Agyneta*, tentatively assigned to morphospecies *Agyneta* sp02, were shown to belong to two distinct genetic lineages, probably corresponding to the nominal species *A. fuscipalpa, A. rurestris* and *A. pseudorurestris*, respectively.

Third, DNA barcodes merged individuals that had been tentatively assigned to different morphotypes during sorting, suggesting that the supposedly diagnostic traits were better interpreted as intraspecific polymorphisms. The morphological study of the Gnaphosidae genus *Micaria* indicated the presence of three different nominal species belonging to the *formicaria* species group, namely *M. coarctata* (Lucas, 1846), *M. formicaria* (Sundevall, 1831) and *M. guttigera*, only distinguishable by either characters such as prosoma measurements, or colouration pattern. However, all the analysed individuals were merged in a single genetic lineage with low genetic divergences (0.3%). After revising all male specimens, we noticed that, although smaller males had different prosoma proportions, their somatic aspect and male palp were all extremely similar. Therefore, we opted to lump all these specimens together as a single species tentatively referred as *M. guttigera*, given that their colouration pattern and prosoma measurements could exclude them from *M. coarctata* and *M. formicaria*, respectively. Reliable characters to clearly diagnose the species of the *formicaria*-group, in which all share the typical two small tibial apophyses and a simple bulbus, are missing.

In the family Linyphiidae, *Metopobactrus prominulus* constitutes a good example of the power of an integrative approach to species delimitation and identification. If we based our data solely on morphology-based taxonomy, we would think an additional species was present in our samples because erigonine species are usually diagnosed, amongst other characters, by the shape of the male cephalic modifications. Given the addition of the species delimitation analysis using DNA barcodes, we, therefore, concluded that all our specimens belonged to *M. prominulus* and that the remarkable turret-like carapace lobe of the males found in Picos de Europa is an undescribed dimorphism within this species. This is clearly exemplified by a recent study (Barrientos et al. 2014) that reports for the first time the presence of *Thaumatoncus indicator* Simon, 1884 in the Iberian Peninsula, based on specimens that most likely belong to the *M. prominulus* with the turret-like cephalic lobe. Similarly, the recent record of *M. falcifrons* in the northern side of Picos de Europa (Jimenez-Segura et al. 2017), most likely also corresponds to *M. prominulus*. Cephalic modifications are common in many erigonines species and are frequently associated to pheromone secreting glands involved in sexual behaviour (Michalik and Uhl 2011). The polymorphisms in the development of the cephalic modification are not exclusive to *M. prominulus*. It was first reported and subsequently studied in another erigonine, *Oedothorax gibbosus* (Blackwall, 1841) (de Keer and Maelfait 1988, Heinemann and Uhl 2000) and has been subsequently discovered in other erigonines (Bosmans and Oger 2018).

In a few cases, we decided to resolve the disagreement on species delimitation between phenotypes and DNA barcodes in favour of the morphological evidence. This was the case in the dictynid *Brigittea civica* and the philodromid *Philodromus aureolus*, which were both split into two lineages with low between-lineage divergence (2.3% and 3%, respectively) and no clear geographic pattern. Additionally, the two lineages of each species returned the same automatic identification in BOLD. Therefore, we assigned the two clusters to the corresponding nominal species and considered their genetic differences as intraspecific divergence. More complex is the case observed in the linyphiidae genus *Agyneta*, where molecular delimitation merged together individuals identified as belonging to *A. orites* and *A. simplicitarsis* (1.2% interspecific divergence), while individuals identified as *A. pseudorurestris*, were mixed with individuals belonging to *A. rurestris*. All these mismatched specimens were revised and their initial morphological identification confirmed, which leads us to suggest that this was probably the result of non-exclusivity of the DNA barcodes, either due to recent speciation, introgression or incomplete lineage sorting. Additional nuclear data and a more thorough geographic sampling should be conducted to select amongst the possible alternatives.

It is also important to highlight that some of the mismatches between the morphological sorting and the molecular delimitation may be actually due to limitations in the algorithm implanted to establish species boundaries. Surprisingly, the mPTP method was unable to split individuals of well-defined nominal species, in some cases even belonging to different genera, despite the significant genetic divergences. In the Linyphiidae, the most obvious case was the grouping of specimens belonging to the two *Tapinocyba* species (*T. affinis* and *T. mitis*), which also included one specimen of *Walckenaeria incisa*, which show divergences above 10% amongst them. Similarly in the Gnaphosidae, the two *Scotophaeus* species were clustered together despite showing genetic divergence above ~12%. The reasons for the rare lumping of divergent sequences (it was only observed in 2 out of the 53 groupings generated) are difficult to assess, but it clearly exemplifies that the method is not error-proof and recommends exerting some quality control on the automatic molecular assignment.

The genetic data was further used to interrogate the generic status of *Thanatus oblongiusculus*, which has been a matter of debate. Depending on the source, the species has been cited as a *Philodromus, Thanatus, Tibellus* or even assigned to its own monotypic genus, *Paratibellus* Simon, 1932 (World Spider Catalog 2018). We identified 4 specimens from Sierra Nevada as belonging to this species. Molecular analyses revealed that genetic divergences between *Thanatus oblongiusculus* and *Tibellus oblongus* were lower than those of any of these species to the other *Thanatus* species sampled. Additionally, the inferred gene trees were compatible with the sister group relationship between *Thanatus oblongiusculus* and *Tibellus*. Future studies based on a more thorough taxon sampling and additional markers may provide further evidence for the transfer of *Thanatus oblongiusculus* to the genus *Tibellus*.

### BOLD identifications

The disagreements between DNA barcodes and morphological data were not only restricted to the specimen sorting and species delimitation. Some mismatches were observed between the morphological identification and the automatic molecular assignment conducted in BOLD. Wrong identifications are not necessarily the result of a lack of expertise and may instead be the reflection of conflicting taxonomy, poor diagnoses or missing informative characters, either due to the stage or preservation of the specimens. In some cases, such apparent misidentifications revealed additional cases of potential new species, but in others, they were interpreted as errors in the BOLD reference library. Several individuals (5 males and 1 female) from the southern parks, identified as belonging to the gnaphosid *Scotophaeus blackwalli*, were slightly smaller than the cosmopolitan form. Although sequences of *S. blackwalli* from Germany and North America were available for comparison, the molecular identification in BOLD did not return any species level identification, which may suggest that our *S. blackwalli* specimens may, in fact, correspond to a different species. It should be noted that a recently described species, endemic to the Iberian Peninsula, *S. nanoides* Wunderlich, 2011, bears similar genitalia to our *S. blackwalli* specimens. However, the reported chaetotaxy of the two species did not match. Availability of DNA barcodes for specimens identified as *S. nanoides* will help to elucidate the taxonomic status of our specimens. A second *Scotophaeus* species, represented by a single female specimen, greatly resembled *S. scutulatus* and *S. validus* (Lucas, 1846) in the genitalia. The BOLD identification tool confirmed that our specimen most likely did not belong to the *S. scutulatus*, because, despite the fact that *S. scutulatus* from Germany, Greece and Turkey were available in BOLD, it neither clustered with these specimens nor resulted in a correct assignment (similarity 87.83 to 87.99%). We provisionally refer to this specimen as Scotophaeus cf. validus.

In the Philodromidae, the *Philodromus praedatus* species group nicely illustrates the challenge of identifying species that display a remarkable degree of genitalic plasticity. The 29 DNA barcodes available in BOLD belonged to two different genetic clusters (as generated by BOLD own clustering algorithm), with a mean genetic divergence of 5.5%, which closely matches the intraspecific divergences of other nominal species within species complexes found in our study. The two clusters included specimens from Germany and Slovenia. The first cluster also included specimens from the Netherlands and Switzerland and the second an additional specimen identified as *Philodromus longipalpis* from Greece. Our specimens identified as *Philodromus praedatus* as illustrated by Segers (1990), matched the first cluster, while an unknown morph referred to as *Philodromus* sp18, clustered with the second group. Due to the mixed identifications in the second cluster and the lack of male specimens, we were reluctant to assign our sp. 18 morph to any nominal species.

Finally, a surprising result was to find out that the specimens identified in our study as the linyphiid *Pocadicnemis juncea*, returned *P. pumila* as the best match in BOLD, while our specimens identified as *P. pumila* could not be assigned. Interestingly, *P. pumila* in BOLD included several genetic clusters, some of which include specimens identified as *P. juncea* in other studies. Our working explanation is a misidentification of *P. pumila* in the reference DNA barcodes in BOLD.

### Closing remarks

Prior to this study, little was known on the spider communities of the Spanish Network of National Parks despite their relevance for ensuring the preservation of exceptional landscapes and their value for future generations. Until now, spider surveys have been conducted in Picos de Europa (Jimenez-Segura et al. 2017) and Cabañeros (Barriga et al. 2007). Here, we present, for the first time, checklists for four additional parks, including abundance information and site preferences. In addition, the DNA barcodes, generated in the framework of the IBERCODING project, will serve as the reference dataset and the basis for an automatic identification system that will ease the monitoring of the studied plots. Finally, some of the species recorded showed morphological variability and deep intraspecific divergences that hint at additional overlooked diversity. Although our study has gone beyond the tip of the iceberg, it may have still fallen short of uncovering the entire iceberg.

## Supplementary Material

Supplementary material 1 Species assignemnt to biogeographic categories Data type: biogeographic categories
File: oo_238705.csv Jagoba Malubres-Olarte

## Figures and Tables

**Figure 2a.**
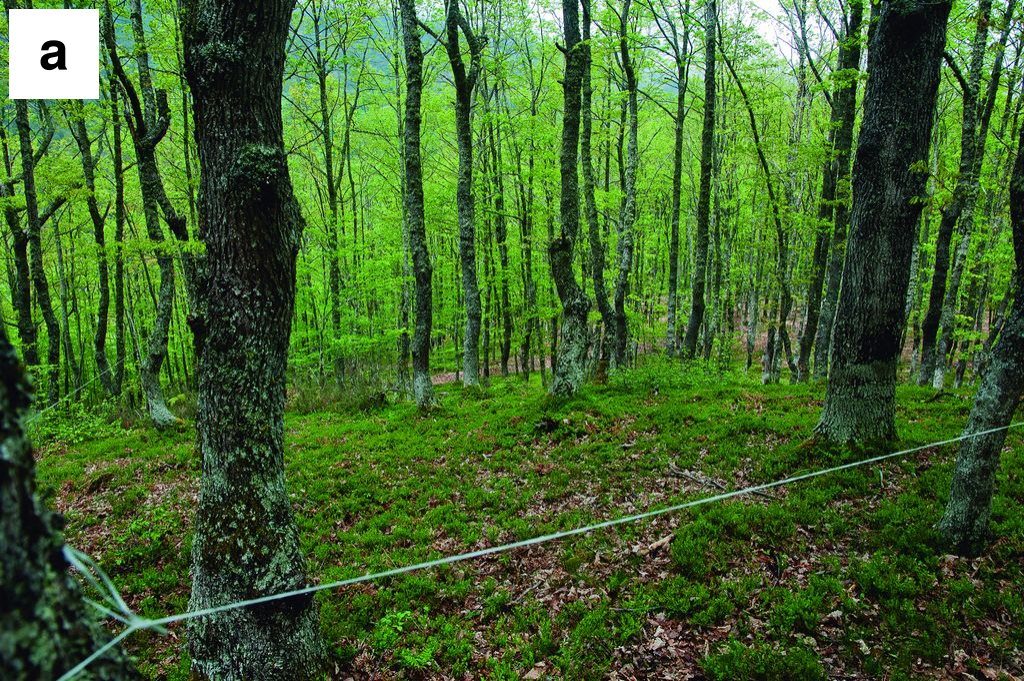
P1 plot Monte Robledo, *Quercus petraea* forest

**Figure 2b.**
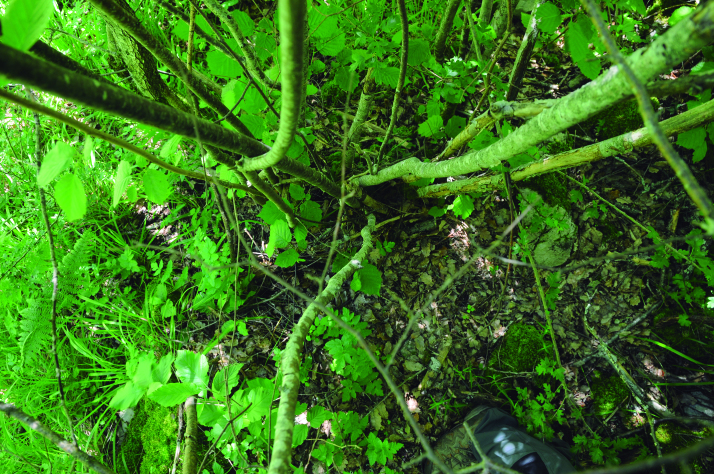
P4 plot El Canto, *Q. faginea* forest (detail leaflitter)

**Figure 2c.**
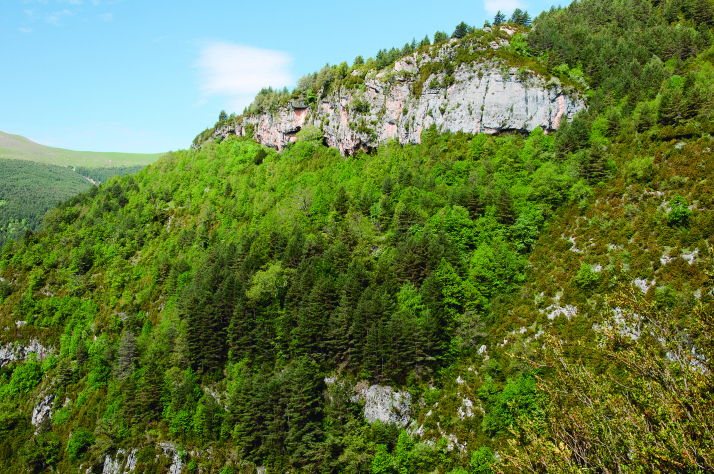
O1 plot O Furno, *Q. subpyrenaica* forest

**Figure 2d.**
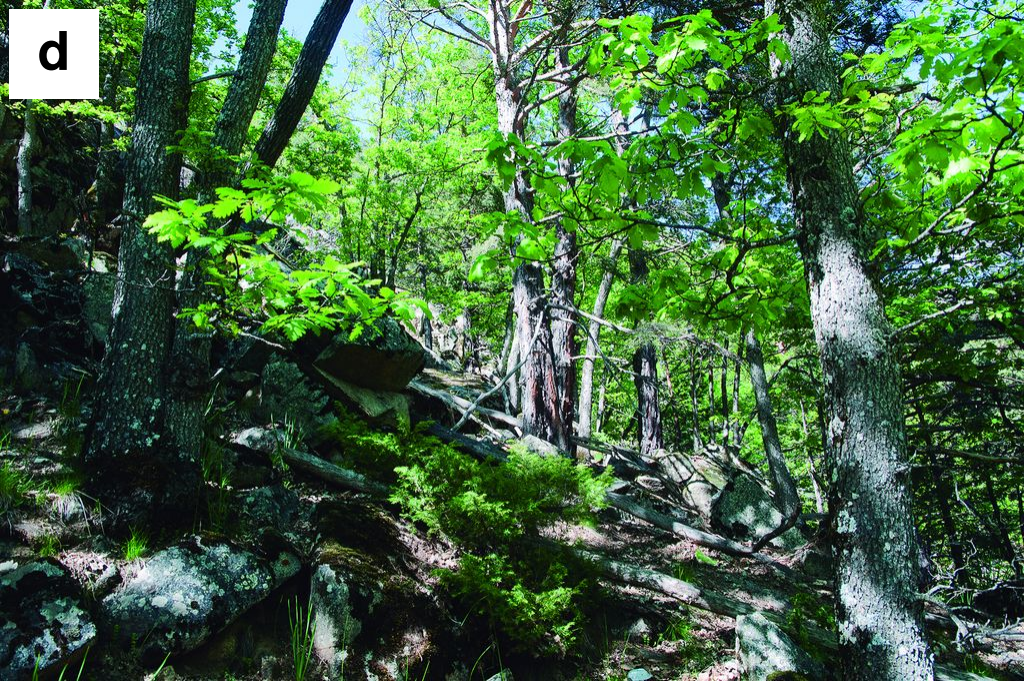
A2 plot Sola de Boi, *Q. pubescens* forest

**Figure 3a.**
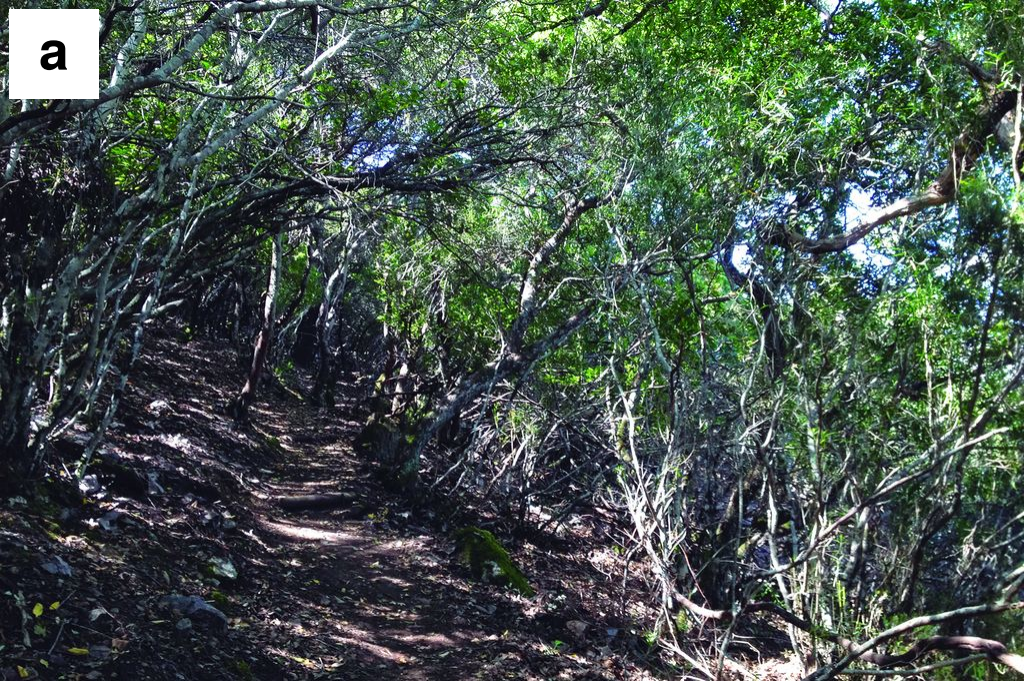
M2 plot Fuente del Frances, *Quercus faginea* forest

**Figure 3b.**
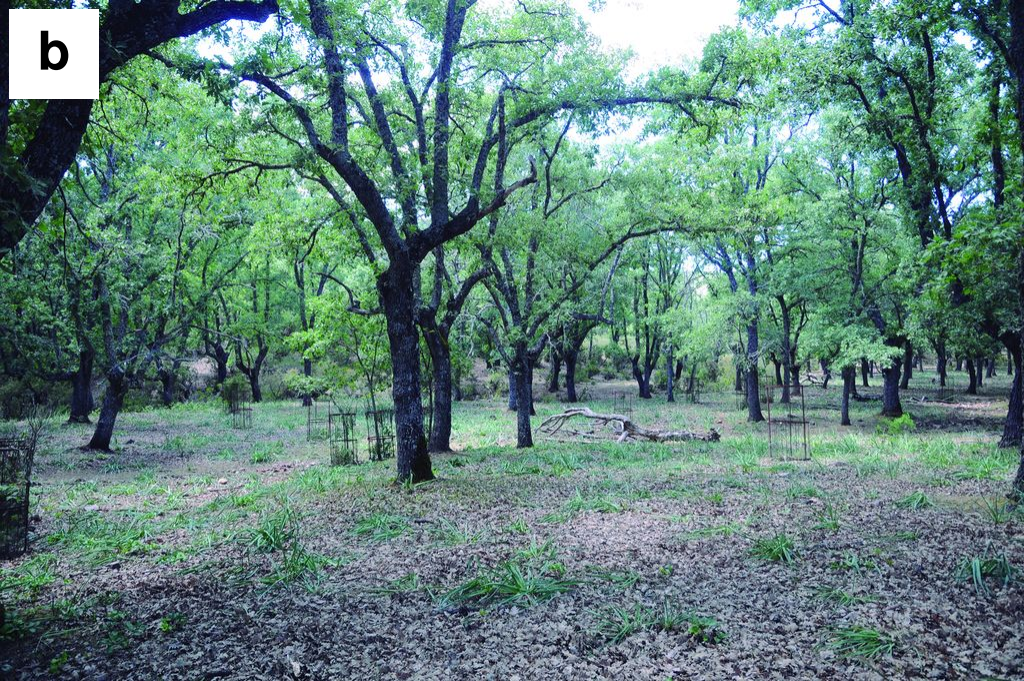
C1 plot Valle Brezoso, *Q. pirenaica*

**Figure 3c.**
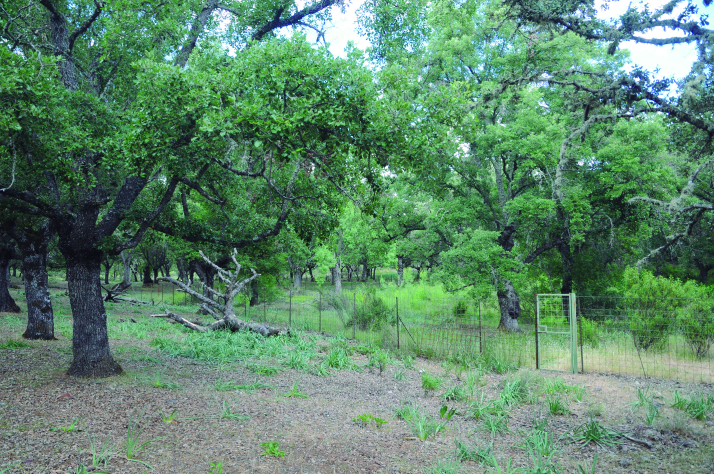
C3 plot La Quesera, *Q. faginea* forest

**Figure 3d.**
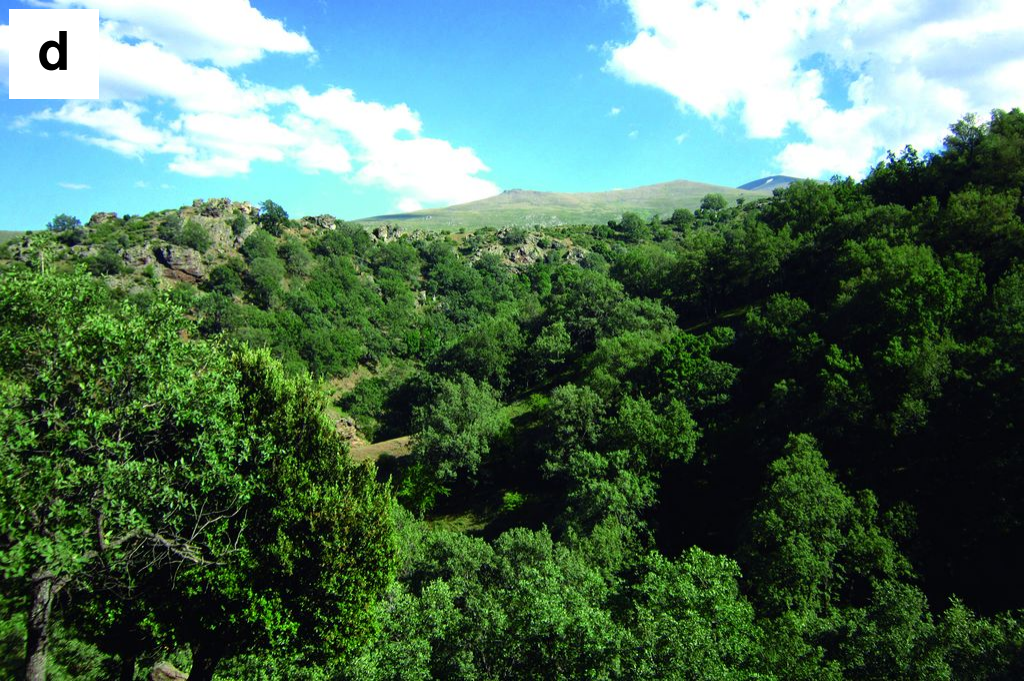
S2 plot Camarate, *Q. pyrenaica*

**Figure 20a.**
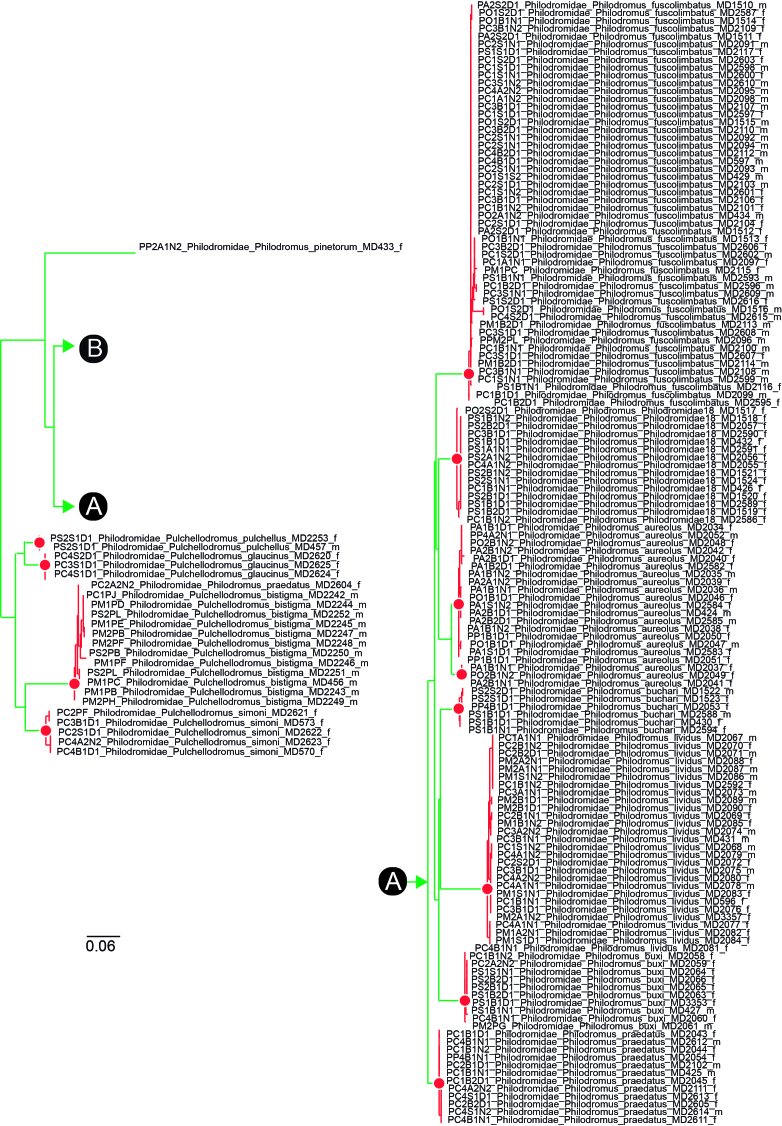


**Figure 20b.**
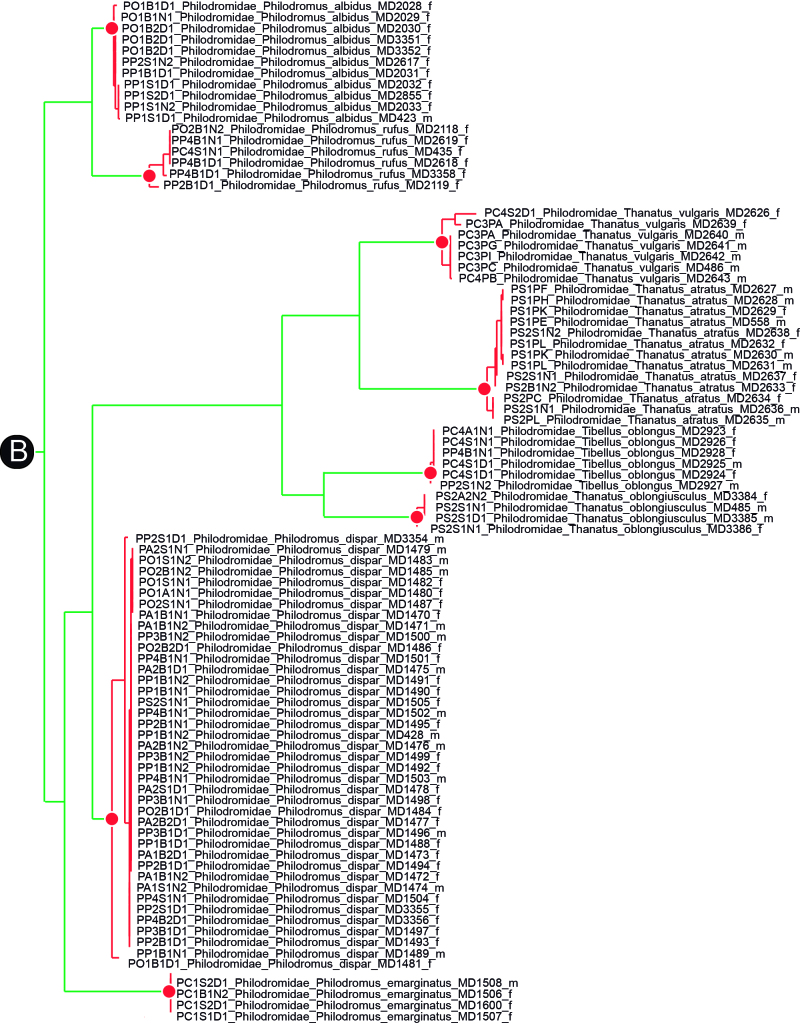


**Table 1.** Information on the sampling sites. Site codes are derived from abbreviated park names. Geographical coordinates are given in the format of decimal degrees (DD).

**Site code**	**Region**	**Province**	**Locality**	**Coordinates (Lat. / Lon.)**	**Altitude (m)**	**Collection dates**	**Habitat**
P1	Castilla y Leon	Leon	Monte Robledo	43.14450 / -4.92675	1071.6	7.VI.2013–21.VI.2013	*Quercus petraea*
P2	Castilla y Leon	Leon	Joyoguelas	43.17771 / -4.90579	764.0	7.VI.2013–22.VI.2013	*Quercus faginea*
P3	Castilla y Leon	Leon	Las Arroyas	43.14351 / -4.94878	1097.1	8.VI.2013–23.VI.2013	*Quercus petraea*
P4	Castilla y Leon	Leon	El Canto	43.17227 / -4.90857	943.5	9.VI.2013–24.VI.2013	*Quercus faginea*
O1	Aragon	Huesca	O Furno	42.60677 / 0.13135	1396.7	12.VI.2013–26.VI.2013	*Quercus subpyrenaica*
O2	Aragon	Huesca	Rebilla	42.59427 / 0.15290	1158.1	13.VI.2013–27.VI.2013	*Quercus subpyrenaica*
A1	Catalonia	Lleida	Sola de Boi	42.54958 / -0.87254	1759.8	15.VI.2013–29.VI.2013	*Quercus pubescens*
A2	Catalonia	Lleida	Sola de Boi	42.54913 / 0.87137	1738.7	16.VI.2013–30.VI.2013	*Quercus pubescens*
M1	Extremadura	Cáceres	Peña Falcón	39.83296 / -6.06410	320.6	23.V.2014–6.VI.2014	*Quercus faginea*
M2	Extremadura	Cáceres	Fuente del Frances	39.82800 / -6.03249	320.7	24.V.2014–7.VI.2014	*Quercus faginea*
C1	Castilla-La Mancha	Ciudad Real	Valle Brezoso	39.35663 / -4.35912	756.6	27.V.2014–9.VI.2014	*Quercus pyrenaica*
C2	Castilla-La Mancha	Ciudad Real	Valle Brezoso	39.35159 / -4.35890	739.3	28.V.2014–10.VI.2014	*Quercus pyrenaica*
C3	Castilla-La Mancha	Ciudad Real	La Quesera	39.36177 / -4.41733	767.6	29.V.2014–11.VI.2014	*Quercus faginea*
C4	Castilla-La Mancha	Ciudad Real	La Quesera	39.36337 / -4.41704	772.3	30.V.2014–12.VI.2014	*Quercus faginea*
S1	Andalucia	Granada	Soportujar	36.96151 / -3.41881	1786.6	31.V.2014–14.VI.2014	*Quercus pyrenaica*
S2	Andalucia	Granada	Camarate	37.18377 / -3.26282	1714.0	1.VI.2014–15.VI.2014	*Quercus pyrenaica*

**Table 2.** Primers used for amplification.

**Location**	**Nickname**	**Sequence**	**Reference**
C1-J-1490	LCOI1490	GGTCAACAAATCATAAAGATATTGG	Folmer et al. 1994
C1-N-2198	HCOI2198	TAAACTTCAGGGTGACCAAAAAATCA	Folmer et al. 1994
C1-N-2191	Nancy	CCCGGTAAAATTAAAATATAAACTTC	Simon et al. 1994
C1-J-1751	Ron	GGATCACCTGATATAGCATTCCC	Simon et al. 1994
C1-J-1834	mlCOIintF	GGWACWGGWTGAACWGTWTAYCCYCC	Leray et al. 2013
C1-N-2198	jgHCOI2198	TAIACYTCIGGRTGICCRAARAAYCA	Leray et al. 2013
